# Supplementary material for: An efficient approach to angular tricyclic molecular architecture via Nazarov-like cyclization and double ring-expansion cascade
Source: Nat Commun. 2022 Apr 28;13:2335. doi: 10.1038/s41467-022-29947-5 (PMC9050659; doi:10.1038/s41467-022-29947-5)
Supplement: Supplementary file 1 — Supplementary Information [file 41467_2022_29947_MOESM1_ESM.pdf]

Supplementary Information

An Efficient Approach to Angular Tricyclic Molecular Architecture via Nazarov-like Cyclization and Double Ring-Expansion Cascade

Yun-Peng Wang,[1†]Kun Fang,[2†] Yong-Qiang Tu,[*1,2] Jun-Jie Yin,[1#] Qi Zhao,[1] and Tian Ke[1]

[1]School of Chemistry and Chemical Engineering, Frontiers Science Center for Transformative Molecules, Shanghai Jiao Tong University, Shanghai, 200240, China.
[2]State Key Laboratory of Applied Organic Chemistry and College of Chemistry and Chemical Engineering, Lanzhou University, Lanzhou, 730000, China.
[*]Corresponding author; E-mail: tuyq@lzu.edu.cn.
[†]These authors contributed equally.
[#]A training graduate student from Harbin Institute of Technology, Shenzhen, 518055, China.

Table of Contents

1. General information 3

2. General procedures for synthesis of substrates **2** or **4**.. 4

3. Preparation of starting materials (**2a-2hh** and **4a-4p**) 5

4. General procedure E for the Nazarov cyclization and two ring expansions reaction............... 79

5. Optimization of diastereoisomeric ratio of **3c** (**β:α**).. 102

6. Plausible mechanism and rationalization for stereo- and regio-selectivity 103

7. Synthetic application.. 106

8. X-ray crystallographic data of **1ff**, **3m**, **4n$_2$**, **111** and **114-1**... 120

9. DFT calculations 130

10. Copies of NMR spectra 160

11. Supplementary References 530

1. General information

Unless otherwise noted, all reactions were performed using oven-dried glassware equipped with a magnetic stir bar under an atmosphere of argon.

All reagents were purchased from commercial suppliers and used without further purification. In addition to commercially available solvents, extra dry solvents were obtained by standard operating method: toluene, tetrahydrofuran (THF), diethyl ether (Et_2O) and benzene were distilled from sodium; Dichloromethane (DCM) were distilled from calcium hydride.

Thin-layer chromatography (TLC) was performed with EMD silica gel 60 F_{254} plates eluting with solvents indicated, visualized by a 254 nm UV lamp and stained with phosphomolybdic acid (PMA). **^{1}H NMR**, **^{13}C NMR**, **^{19}F NMR** and **^{31}P NMR** spectra were obtained on Mercuryplus 400, Bruker AM-400, or Bruker AM-500. Chemical shifts (*δ*) were quoted in ppm relative to tetramethylsilane or residual un-deuterated solvent as internal standard (C_6D_6: 7.16 ppm for ^{1}H NMR, 128.06 ppm for ^{13}C NMR; $CDCl_3$: 7.26 ppm for ^{1}H NMR, 77.00 ppm for ^{13}C NMR), multiplicities are as indicated: s = singlet, d = doublet, t = triplet, q = quartet, m = multiplet, br = broad. The **IR** spectra were recorded on a Fourier transform infrared spectrometer. High-resolution mass spectral analysis **(HRMS)** data were measured on an APEXII 47e FT-ICR spectrometer by means of ESI technique. Crystallographic data were obtained from a Bruker D8 VENTURE diffractometer.

2. General procedures for synthesis of substrates 2 or 4.

Method A

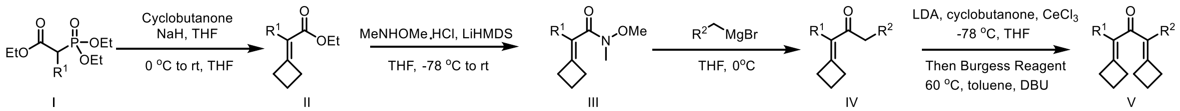

The method is suitable for **2a**, **2c**, **2e**, **2h**, **2i**, **2k**, **2l**, **2p**

Method B

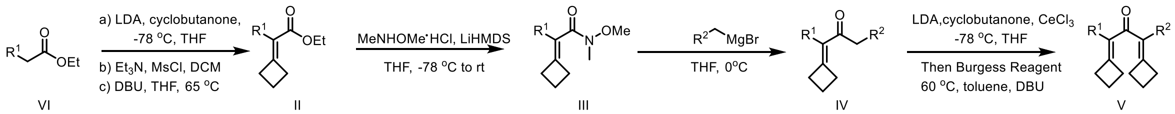

The method is suitable for **2b**, **2d**, **2j, 2n**

Method C

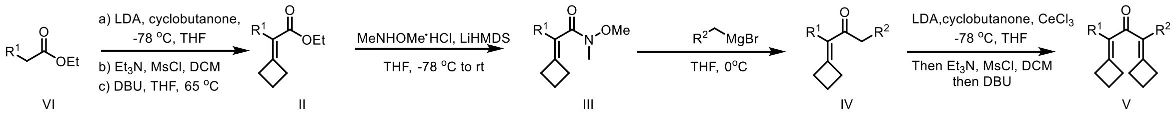

The method is suitable for **2f**, **2g**

Method D

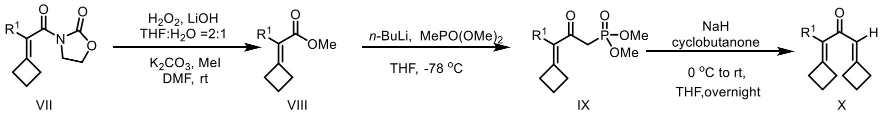

The method is suitable for **2t, 2u**, **2v**

Method E

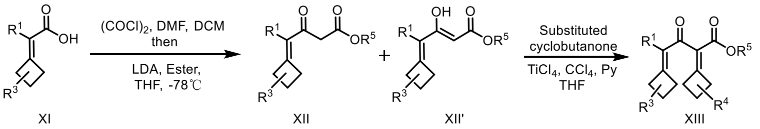

The method is suitable for **2w** to **2hh**, **4a**, **4c** to **4j, 4l** to **4n**

Method F

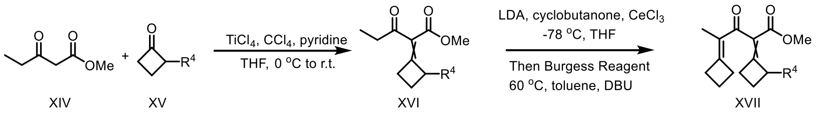

The method is suitable for **4b**, **4K**

Supplementary Figure 1. General procedures for synthesis of substrates **2** or **4**.

Other substrates (**2o**, **2m**, **2q**, **2r**, **4o**, **4p**) not mentioned above can be found below.

3. Preparation of starting materials (2a-2hh and 4a-4p)

3.1 Preparation of starting material 2a

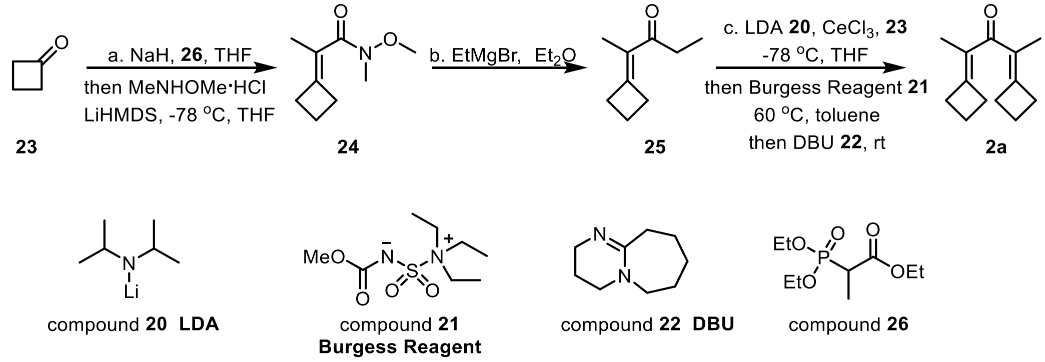

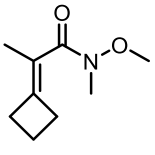

24

Compound **24.** To a stirred solution of NaH (3.5 g, 60% in oil, 87.0 mmol, 1.3 equiv) in THF (130 mL) at 0 ºC was slowly added compound **26** (5.0 mL, 87.0 mmol, 1.3 equiv). After the mixture was stirred at rt for 1 h, cyclobutanone **23** (18.7 mL, 67.0 mmol, 1.0 equiv) was added to the flask. After stirred at the same temperature for 4 h, the reaction was quenched carefully with saturated NH_4Cl aqueous solution and extracted with Et_2O (4 x 80 mL). The combined organic layer was washed with brine, dried over Na_2SO_4 and concentrated under vacuum. The crude product was used for next step without purification. The above crude product was dissolved in THF (120 mL) at -78 ºC, and MeNHOMe·HCl (13.1 g, 134 mmol, 2.0 equiv) was added. After stirred at -78 ºC for 0.5 h, LiHMDS (268 mL, 1.0 M in THF, 268 mmol, 4.0 equiv) was added to the flask. After the temperature of the mixture was warmed to rt and stirring was continued for 0.5 h, the reaction was quenched with saturated NH_4Cl aqueous solution and extracted with EtOAc (3 x 150 mL). The combined organic layer was washed with brine, dried over Na_2SO_4, concentrated and purified by flash column chromatography (EtOAc: petroleum ether = 1:25 to 1:6) to give weinreb amide product **24** (9.1 g, 53.6 mmol, 80% yield for 2 steps) as a colorless oil.

^{1}H NMR (400 MHz, $CDCl_3$): δ 3.63 (s, 3H), 3.19 (s, 3H), 2.74 – 2.65 (m, 4H), 1.99 –1.91 (m, 2H), 1.71 – 1.70 (m, 3H).

^{13}C NMR (100 MHz, $CDCl_3$): δ 170.4, 145.1, 121.9, 61.1, 33.6, 30.6, 29.8, 16.3, 14.3.

HRMS (ESI) calcd for $[M+Na]^+$ $C_9H_{15}NNaO_2$, m/z : 192.0995, found : 192.0998.

IR ν [cm^{-1}] 2954, 2920, 2934, 1640, 1412, 1372, 988.

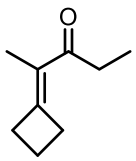

25

Compound **25.** To a stirred solution of weinreb amide **24** (1.7 g, 10.0 mmol, 1.0 equiv) in Et_2O (60 mL) at 0 ºC was slowly added EtMgBr (20 mL, 1.0 M in THF, 20.0 mmol, 2.0 equiv). After stirred at the same temperature for 4 h, the reaction was quenched carefully with saturated NH_4Cl aqueous solution and extracted with Et_2O (4 x 30 mL). The combined organic layer was washed with brine, dried over Na_2SO_4 and concentrated under vacuum. The residue was purified by flash column chromatography (Et_2O: petroleum ether = 1:100 to 1:25) to give enone product **25** (870 mg, 6.3 mmol, 63% yield) as a colorless oil.

^{1}H NMR (400 MHz, $CDCl_3$): δ 3,08 (s, 2H), 2.83 (s, 2H), 2.45 (q, J = 6.4 Hz, 2H), 2.08 – 2,01 (m, 2H), 1.65 (s, 3H), 1.04 (t, J = 7.2 Hz, 3H).

^{13}C NMR (100 MHz, $CDCl_3$): δ 201.5, 157.5, 129.3, 34.4, 34.2, 31.6, 16.4, 12.8, 8.0.

HRMS (ESI) calcd for $[M+Na]^+$ $C_9H_{14}NaO$, m/z : 161.0937, found : 161.0938.

IR ν [cm^{-1}] 2924, 1704, 1574,1116, 1031.

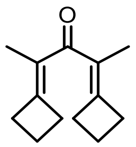

2a

Compound **2a.** To a stirred solution of di*iso*propylamine (2.3 mL, 16.0 mmol) in THF (11.5 mL) at 0 ºC was slowly added *n*-BuLi (6.4 mL, 2.5 M in hexane, 16.0 mmol) for 0.5 h. Compound enone **25** (138 mg, 1.0 mmol, 1.0 equiv) was dissolved in THF (2 mL) at -78 ℃, and the above freshly prepared LDA solution (3.7 mL, 3.0 mmol, 3.0 equiv) was added. After the mixture was stirred at the same temperature for 1 h, $CeCl_3$ (736 mg, 3.0 mmol, 3.0 equiv) in THF (2 mL) was added, and stirred for another 5 h. And then cyclobutanone **23** (0.22 mL, 3.0 mmol, 3.0 equiv) was added and the resultant solution was stirred at –78 ºC for 0.5 h. The reaction was quenched carefully with saturated NH_4Cl aqueous solution and extracted with EtOAc (3 x 50 mL). The combined organic layer was washed with brine, dried over Na_2SO_4 and concentrated under vacuum. The crude product was purified by a short flash column chromatography (EtOAc: petroleum ether = 1:20). The above product was dissolved in toluene (3 mL) at rt, and burgess reagent (714 mg, 3.0 mmol, 3.0 equiv) was added. After stirred at 60 ºC for 0.5 h, starting materials transformed completely (determined by TLC). Then, the mixture was cooled to room temperature and DBU (0.45 mL, 3.0 mmol, 3.0 equiv) was added to the mixture and stirred at that temperature for 3 h.

Upon completion, the reaction was quenched carefully with water and extracted with EtOAc (3 x 10 mL). The combined organic layer was washed with brine, dried over Na_2SO_4 and concentrated under vacuum. The residue was purified by flash column chromatography (EtOAc: petroleum ether = 1:60) to give dienone product **2a** (142.5 mg, 0.8 mmol, 75% yield for 2 steps) as a white solid.

Method for activating $CeCl_3$: anhydrous $CeCl_3$ was heated to 140 ºC in vacuo for 2 hours. After cooling the system to room temperature, THF was added and the mixture was stirred for 1.0 h.

^{1}H NMR (400 MHz, $CDCl_3$): δ 2.76 (dd, J = 8.4, 7.6 Hz, 4H), 2.71 (dd, J = 8.8, 7.6 Hz, 4H), 1.95 (p, J = 8.0 Hz, 4H), 1.65 (s, 6H).

^{13}C NMR (100 MHz, $CDCl_3$): δ 199.1, 151.1, 129.3, 31.9, 31.0, 16.5, 12.9.

HRMS (ESI) calcd for $[M+Na]^+$ $C_{13}H_{18}NaO$, m/z : 213.1250, found : 213.1250.

IR ν [cm^{-1}] 2953, 2917, 1687, 1630, 1337, 1005.

3.2 Preparation of starting material 2b

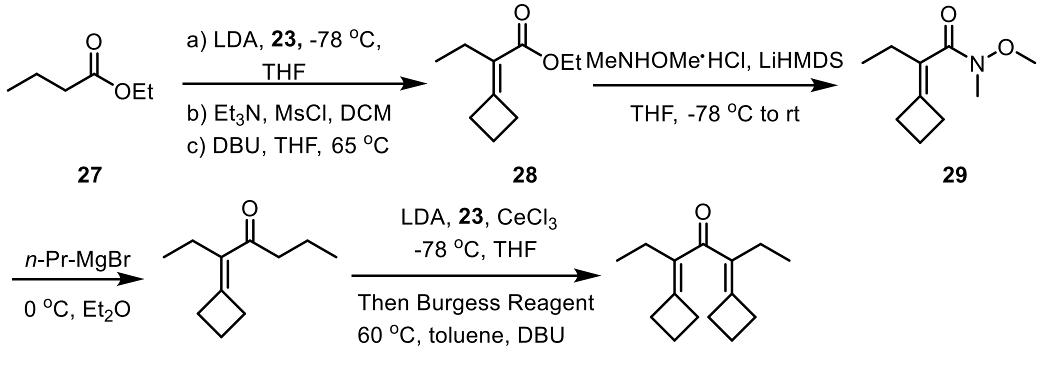

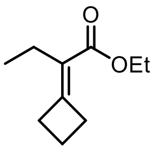

28

Compound **28.** To a stirred solution of di*iso*propylamine (6.7 mL, 45.0 mmol, 1.5 equiv) in THF (45 mL) at 0 ºC was slowly added *n*-BuLi (18 mL, 2.5 M in hexane, 45.0 mmol, 1.5 equiv) for 0.5 h. Compound ethyl butyrate **27** (4.0 mL, 30 mmol, 1.0 equiv) was dissolved in THF (25 mL) and added to the above freshly prepared LDA solution. After the mixture was stirred at the same temperature for 1 h, cyclobutanone **23** (2.7 mL, 36 mmol, 1.2 equiv) was added and the resultant solution was stirred at –78 °C for another 0.5 h. The reaction was quenched carefully with saturated NH_4Cl aqueous solution and extracted with EtOAc (3 x 50 mL). The combined organic layer was washed with brine, dried over Na_2SO_4 and concentrated under vacuum. The crude product was dissolved in DCM (100 mL) at 0 ºC, and Et_3N (50 mL, 360 mmol, 12.0 equiv) and MsCl (11.6

mL, 150 mmol, 5.0 equiv) were added. After stirred at rt for 4 h, starting materials transformed completely (determined by TLC), the reaction was quenched carefully with water and extracted with EtOAc (3 x 10 mL). The combined organic layer was washed with brine, dried over Na_2SO_4 and concentrated under vacuum and redissolved in THF (60 mL) at rt, and DBU (13 mL, 90 mmol, 3.0 equiv) was added. After stirred at 65 °C for 4 h, the solution was concentrated under vacuum and the residue was purified by flash column chromatography (EtOAc: petroleum ether = 1:50) to give product ene ester **28** (2.5 g, 14.9 mmol, 50% yield for 3 steps) as yellow oil.

^{1}H NMR (500 MHz, $CDCl_3$): δ 4.05 (q, J = 7.0 Hz, 2H), 2.97 (ddd, J = 8.0, 8.0, 0.5 Hz, 2H), 2.73 – 2.67 (m, 2H), 2.03 (q, J = 7.5 Hz, 2H), 1.95 – 1.88 (m, 2H), 1.17 (t, J = 7.0 Hz, 3H), 0.89 (t, J = 7.5 Hz, 3H).

^{13}C NMR (125 MHz, $CDCl_3$): δ 167.0, 158.7, 125.5, 59.3, 33.6, 30.4, 20.9, 16.4, 14.1, 13.3.

HRMS (ESI) calcd for $[M+Na]^+$ $C_{10}H_{16}NaO_2$, m/z : 191.1043, found : 191.1046.

IR ν [cm^{-1}] 2963, 2931, 2874, 1706, 1671, 1305, 789.

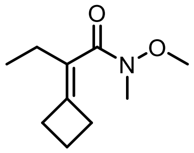

29

Compound **29.** The ene ester **28** (2.0 g, 12 mmol, 1.0 equiv) was dissolved in THF (80 mL) at -78 °C, and MeNHOMe·HCl (2.34 g, 24 mmol, 2.0 equiv) was added. After the mixture was stirred at -78 °C for 0.5 h, LiHMDS (48 mL, 1.0 M in THF, 48 mmol, 4.0 equiv) was added to the flask. After the temperature of the mixture was warmed to rt and stirring was continued for 0.5 h, the mixture was quenched with saturated NH_4Cl aqueous solution and extracted with EtOAc (3 x 50 mL). The combined organic layer was washed with brine, dried over Na_2SO_4 and concentrated under vacuum. The residue was purified by flash column chromatography (EtOAc: petroleum ether = 1:6) to give weinreb amide product **29** (1.1 g, 6.0 mmol, 50% yield) as colorless oil.

^{1}H NMR (500 MHz, $CDCl_3$): δ 3.57 (s, 3H), 3.14 (s, 3H), 2.62 (dd, J = 8.0, 8.0 Hz, 4H), 2.09 (q, J = 7.5 Hz, 2H), 1.90 – 1.84 (m, 2H), 0.88 (t, J = 7.5 Hz, 3H).

^{13}C NMR (125 MHz, $CDCl_3$): δ 169.4, 143.0, 128.2, 60.8, 33.7, 30.4, 29.5, 22.2, 16.3, 12.6.

HRMS (ESI) calcd for $[M+Na]^+$ $C_{10}H_{17}NNaO_2$, m/z : 206.1151, found : 206.1152.

IR ν [cm^{-1}] 2954, 2868, 1641, 1375, 988, 747.

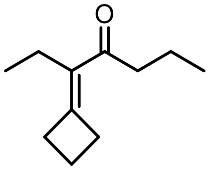

30

Compound **30.** To a stirred solution of weinreb amide **29** (200 mg, 1.1 mmol, 1.0 equiv) in Et_2O (10 mL) at 0 ºC was slowly added *n*-PrMgBr (1.7 mL, 1.7 mmol, 1.5 equiv). After stirred at the same temperature for 5 h, the reaction was quenched carefully with saturated NH_4Cl aqueous solution and extracted with Et_2O (3 x 20 mL). The combined organic layer was washed with brine, dried over Na_2SO_4 and concentrated under vacuum. The residue was purified by flash column chromatography (Et_2O: petroleum ether = 1:20) to give enone product **30** (130 mg, 0.78 mmol, 70% yield) as a colorless oil.

^{1}H NMR (500 MHz, $CDCl_3$): δ 3.10 – 3.06 (m, 2H), 2.86 – 2.82 (m, 2H), 2.39 (t, J = 7.5 Hz, 2H), 2.11 (q, J = 7.5 Hz, 2H), 2.07 – 2.01 (m, 2H), 1.63 – 1.55 (m, 2H), 0.91 – 0.88 (m, 6H).

^{13}C NMR (125 MHz, $CDCl_3$): δ 200.8, 156.7, 136.1, 43.5, 34.2, 31.3, 20.7, 17.5, 16.4, 13.9, 13.5.

HRMS (ESI) calcd for $[M+Na]^+$ $C_{11}H_{18}NaO$, m/z : 189.1250 found : 189.1250.

IR ν [cm^{-1}] 2961, 2932, 2873, 1660, 1459, 1366, 806.

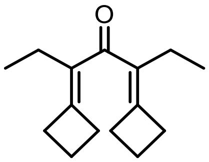

2b

Compound **2b.** To a stirred solution of di*iso*propylamine (2.3 mL, 16.0 mmol) in THF (11.5 mL) at 0 ºC was slowly added *n*-BuLi (6.4 mL, 2.5 M in hexane, 16.0 mmol) for 0.5h. Compound enone **30** (200 mg, 1.26 mmol, 1.0 equiv) was dissolved in THF (2 mL) at -78 ℃, and the above freshly prepared LDA solution (4.7 mL, 3.8 mmol, 3.0 equiv) was added. After the mixture was stirred at the same temperature for 1 h, $CeCl_3$ (932 mg, 3.8 mmol, 3.0 equiv) in THF (2 mL) was added, and stirred for another 5 h. And then cyclobutanone **23** (0.28 mL, 3.8 mmol, 3.0 equiv) was added and the resultant solution was stirred at –78 ºC for 0.5 h. The reaction was quenched carefully with saturated NH_4Cl aqueous solution and extracted with EtOAc (3 x 50 mL). The combined organic layer was washed with brine, dried over Na_2SO_4 and concentrated under vacuum. The crude product was purified by a short flash column chromatography (EtOAc: petroleum ether = 1:20). The above product was dissolved in toluene (9 mL) at rt, and burgess reagent (904 mg, 3.8 mmol, 3.0 equiv) was added. After stirred at 60 ºC for 0.5 h, starting materials transformed completely (determined by TLC). Then, the mixture was cooled to room temperature and DBU (0.57 mL, 3.8 mmol, 3.0 equiv) was added to the mixture and stirred at that temperature for 9 h. Upon completion,

the reaction was quenched carefully with water and extracted with EtOAc (3 x 10 mL). The combined organic layer was washed with brine, dried over Na_2SO_4 and concentrated under vacuum. The residue was purified by flash column chromatography (EtOAc: petroleum ether = 1: 60) to give dienone product **2b** (132 mg, 0.6 mmol, 48% yield for two steps) as a colorless oil.
Method for activating $CeCl_3$: anhydrous $CeCl_3$ was heated to 140 ºC in vacuo for 2 hours. After cooling the system to room temperature, THF was added and the mixture was stirred for 1.0 h.
^{1}H NMR (500 MHz, $CDCl_3$): δ 2.83 (dd, J = 7.0, 7.0 Hz, 4H), 2.77 (dd, J = 7.5, 7.0 Hz, 4H), 2.19 (q, J = 7.5 Hz, 4H), 2.00 – 1.93 (m, 4H), 0.94 (t, J = 7.5 Hz, 6H).
^{13}C NMR (125 MHz, $CDCl_3$): δ 198.3, 150.7, 136.1, 32.3, 30.9, 21.5, 16.7, 13.4.
HRMS (ESI) calcd for $[M+Na]^+$ $C_{15}H_{22}NaO$, m/z : 241.1563, found : 241.1563.
IR ν [cm^{-1}] 2961, 2932, 2873, 1629, 1459, 1334, 1103, 786.

3.3 Preparation of starting material 2c

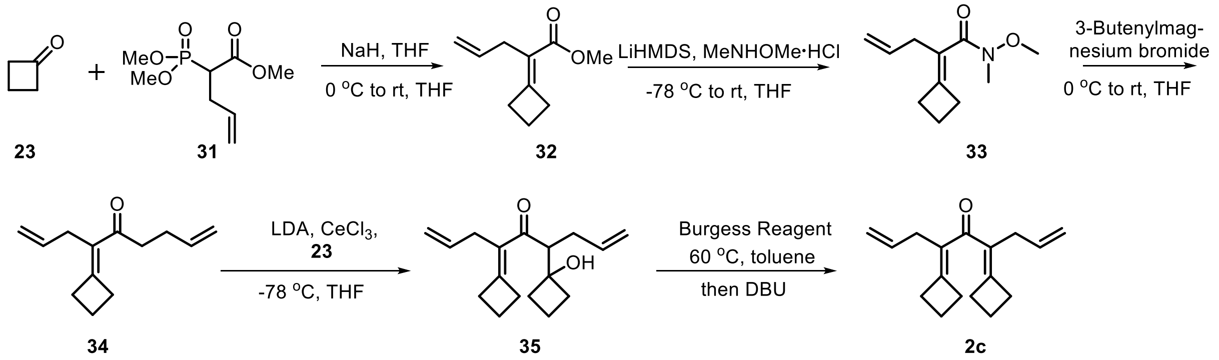

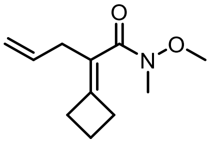

33

Compound **33.** To a stirred solution of NaH (60% in oil, 0.74 g, 18.4 mmol, 1.3 equiv) in THF (60 mL) at 0 ºC was slowly added phospholipids **31** (4.6 g, 18.4 mmol, 1.3 equiv). After the mixture was stirred at rt for 1 h, cyclobutanone **23** (1.05 mL, 14.1 mmol, 1.0 equiv) was added. After stirred at the same temperature for 12 h, the reaction was quenched carefully with saturated NH_4Cl aqueous solution and extracted with EtOAc (4 x 100 mL). The combined organic layer was washed with brine, dried over Na_2SO_4 and concentrated under vacuum, and the residue was subjected to a short plug of silica gel using (EtOAc: petroleum ether = 0 to 1:30) as eluent to give the product **32**. The above product **32** (1.7 g, 10 mmol, 1.0 equiv) was dissolved in THF (20 mL) at -78 ºC, MeNHOMe·HCl (2.0 g, 40 mmol, 2.0 equiv) was added. After the mixture was stirred at -78 ºC for 0.5 h, LiHMDS (40 mL, 1.0 M in THF, 40 mmol, 4.0 equiv) was added to the flask. After the temperature of the mixture was warmed to rt and stirring was continued for 0.5 h, the mixture was

quenched with saturated NH_4Cl aqueous solution and extracted with EtOAc (3 x 70 mL). The combined organic layer was washed with brine, dried over Na_2SO_4 and concentrated under vacuum. The residue was purified by flash column chromatography (EtOAc: petroleum ether = 1:25 to 2:3) to give product weinreb amide **33** (1.0 g, 5.1 mmol, 37% yield for 2 steps) as a colorless oil.

^{1}H NMR (400 MHz, $CDCl_3$) δ 5.61 (ddt, *J* = 17.0, 10.0, 7.0 Hz, 1H), 4.91 (ddd, *J* = 17.0, 3.5, 1.5 Hz, 1H), 4.86 – 4.79 (m, 1H), 3.50 (s, 3H), 3.08 (s, 3H), 2.83 – 2.71 (m, 2H), 2.67 – 2.53 (m, 4H), 1.90 – 1.78 (m, 2H).

^{13}C NMR (100 MHz, $CDCl_3$) *δ* 168.8, 145.2, 134.7, 124.4, 115.6, 60.7, 33.3, 30.5, 29.6, 16.2.

HRMS (ESI) calcd for $[M+Na]^+$ $C_{11}H_{17}NNaO_2$, m/z: 218.1151 , found: 218.1151.

IR *v* [cm^{-1}] 3077, 2934, 1638, 1412, 1375, 1179, 975, 912.

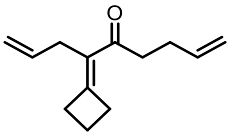

34

Compound **34.** To a stirred solution of weinreb amide **33** (506 mg, 2.6 mmol, 1.0 equiv) in THF (10 mL) at 0 ºC was slowly added 3-butenylmagnesium bromide (3.9 mL, 1.0 M in THF, 3.9 mmol, 1.5 equiv). After the temperature of the mixture was warmed to rt and stirring was continued for 4 h, the reaction was quenched carefully with saturated NH_4Cl aqueous solution and extracted with EtOAc (4 x 50 mL). The combined organic layer was washed with brine, dried over Na_2SO_4 and concentrated under vacuum. The residue was purified by flash column chromatography (EtOAc: petroleum ether = 1:15) to give enone product **34** (353 mg, 1.87 mmol, 72% yield) as a yellow oil.

^{1}H NMR (500 MHz, $CDCl_3$) δ 5.80 (ddt, *J* = 17.0, 10.0, 6.5 Hz, 1H), 5.71 (ddt, *J* = 16.5, 10.0, 6.5 Hz, 1H), 5.01 – 4.87 (m, 4H), 3.16 – 3.05 (m, 2H), 2.85 (t, *J* = 7.5 Hz, 4H), 2.51 (t, *J* = 7.5 Hz, 2H), 2.33 – 2.25 (m, 2H), 2.09 – 1.99 (m, 2H).

^{13}C NMR (125 MHz, $CDCl_3$) *δ* 199.0, 159.5, 137.7, 135.8, 131.7, 114.6, 114.5, 40.5, 34.3, 31.5, 31.4, 27.9, 16.4.

HRMS (ESI) calcd for $[M+Na]^+$ $C_{13}H_{18}NaO$, m/z: 213.1250 , found: 213.1250.

IR *v* [cm^{-1}] 3077, 2913, 1658, 1639, 1404, 1286, 994, 908.

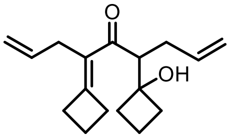

35

Compound **35.** To a stirred solution of di*iso*propylamine (1.4 mL, 10.0 mmol, 4.0 equiv) in THF (4.6 mL) at 0 ºC was slowly added *n*-BuLi (4.0 mL, 2.5 M in hexane, 1.3 mmol, 4.0 equiv) for 0.5

h. Compound enone **34** (340 mg, 1.8 mmol, 1.0 equiv) was dissolved in THF (4 mL) at -78 °C, and the above freshly prepared LDA solution (5.4 mL, 5.4 mmol, 3.0 equiv) was added. After the mixture was stirred at the same temperature for 1 h, $CeCl_3$ (1.32 g, 5.4 mmol, 3.0 equiv) in anhydrous THF (4 mL) was added dropwise via a syringe. After the mixture was stirred at the same temperature for another 1 h, cyclobutanone **23** (0.4 mL, 5.4 mmol, 3.0 equiv) was added and the resultant solution was stirred at -78 °C for 0.5 h. The reaction was quenched carefully with saturated NH_4Cl aqueous solution and extracted with EtOAc (3 x 50 mL). The combined organic layer was washed with brine, dried over Na_2SO_4 and concentrated under vacuum. The residue was purified by flash column chromatography (EtOAc: petroleum ether = 1:12 to 1:6) to give adol product **35** (267mg, 1.0 mmol, 57% yield) as a yellow oil and starting material (114 mg, 0.6 mmol). Method for activating $CeCl_3$: anhydrous $CeCl_3$ was heated to 140 ºC in vacuo for 2 hours. After cooling the system to room temperature, THF was added and the mixture was stirred for 1.0 h.

^{1}H NMR (400 MHz, $CDCl_3$) δ 5.78 – 5.63 (m, 2H), 5.03 – 4.91 (m, 4H), 4.23 (s, 1H), 3.28 – 3.16 (m, 3H), 2.92 – 2.81 (m, 4H), 2.42 – 2.23 (m, 2H), 2.12 – 1.95 (m, 5H), 1.86 – 1.75 (m, 2H), 1.60 – 1.51 (m, 1H).

^{13}C NMR (100 MHz, $CDCl_3$) δ 206.3, 163.1, 135.8, 135.4, 132.7, 116.6, 115.0, 76.2, 52.5, 35.7, 34.3, 34.2, 32.9, 32.2, 31.4, 16.4, 12.8.

HRMS (ESI) calcd for $[M+Na]^+$ $C_{17}H_{24}NaO_2$, m/z: 283.1669 , found: 283.1670.

IR v [cm^{-1}] 3077, 2980, 2934, 1628, 1385, 1272, 912.

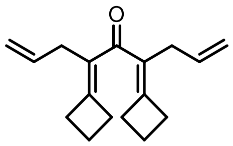

2c

Compound **2c.** The above adol product **35** (260 mg, 1.0 mmol, 1.0 equiv) was dissolved in toluene (4 mL) at rt, and burgess reagent (714 mg, 3.0 mmol, 3.0 equiv) was added. After stirred at 60 ºC for 1 h, starting materials transformed completely (determined by TLC). Then, the mixture was cooled to room temperature and DBU (0.45 mL, 3.0 mmol, 3.0 equiv) was added to the mixture and stirred at that temperature for 4 h. Upon completion, the reaction was quenched carefully with water and extracted with EtOAc (3 x 50 mL). The combined organic layer was washed with brine, dried over Na_2SO_4 and concentrated under vacuum. The residue was purified by flash column chromatography (EtOAc: petroleum ether = 1:30 to 1:12) to give dienone product **2c** (100 mg, 0.43 mmol, 43% yield) as a yellow oil.

^{1}H NMR (400 MHz, $CDCl_3$) δ 5.76 – 5.66 (m, 2H), 4.93 (dd, J = 28.0, 13.6 Hz, 4H), 2.90 (d, J = 6.4 Hz, 4H), 2.83 (dd, J = 8.0, 7.6 Hz, 4H), 2.76 (dd, J = 7.6, 7.6 Hz, 4H), 2.08 – 1.84 (m, 4H).

^{13}C NMR (100 MHz, $CDCl_3$) δ 197.0, 152.8, 135.7, 132.0, 115.2, 32.8, 32.5, 31.0, 16.6.

HRMS (ESI) calcd for $[M+Na]^+$ $C_{17}H_{22}NaO$, m/z: 265.1563 , found: 265.1563.
IR *v* [cm^{-1}] 3077, 2978, 2955, 2914, 1631, 1427, 1332, 994, 910.

3.4 Preparation of starting material 2d

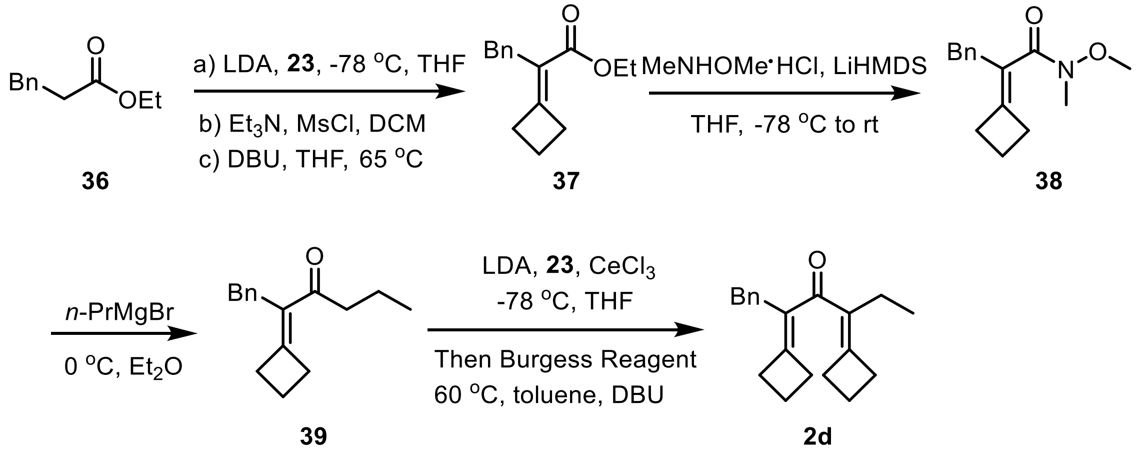

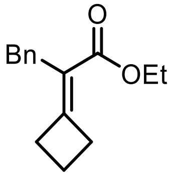

37

Compound **37.** To a stirred solution of di*iso*propylamine (6.7 mL, 45.0 mmol, 1.5 equiv) in THF (45 mL) at 0 ºC was slowly added *n*-BuLi (18 mL, 2.5 M in hexane, 45.0 mmol, 1.5 equiv) for 0.5 h. Compound ethyl 3-phenylpropionate **36** (5.3 mL, 30 mmol, 1.0 equiv) was dissolved in THF (25 mL) and added to the above freshly prepared LDA solution. After the mixture was stirred at the same temperature for 1 h, cyclobutanone **23** (2.7 mL, 36 mmol, 1.2 equiv) was added and the resultant solution was stirred at –78 °C for another 0.5 h. The reaction was quenched carefully with saturated NH_4Cl aqueous solution and extracted with EtOAc (3 x 50 mL). The combined organic layer was washed with brine, dried over Na_2SO_4 and concentrated under vacuum. The crude product was dissolved in DCM (100 mL) at 0 ºC, and Et_3N (50 mL, 360 mmol, 12.0 equiv) and MsCl (11.6 mL, 150 mmol, 5.0 equiv) were added. After stirred at rt for 7 h, starting materials transformed completely (determined by TLC), and the reaction was quenched carefully with water and extracted with EtOAc (3 x 100 mL). The combined organic layer was washed with brine, dried over Na_2SO_4 and concentrated under vacuum and redissolved in THF (60 mL) at rt, and DBU (13 mL, 90 mmol, 3.0 equiv) was added. After stirred at 65 ºC for 4 h, the solution was concentrated under vacuum and the residue was purified by flash column chromatography (EtOAc: petroleum ether = 1:50) to give ene ester product **37** (4.3 g, 18.7 mmol, 62% yield for 3 steps) as a yellow oil.
^{1}**H NMR** (500 MHz, $CDCl_3$): δ 7.27 – 7.15 (m, 5H), 4.12 (q, J = 7.0 Hz, 2H), 3.49 (s, 2H), 3.17 –

3.13 (m, 2H), 2.91 – 2.88 (m, 2H), 2.10 – 2.03 (m, 2H), 1.22 (t, J = 7.5 Hz, 3H).
^{13}C NMR (125 MHz, $CDCl_3$): δ 166.7, 160.8, 140.1, 128.1, 127.9, 125.5, 123.1, 59.5, 33.7, 33.3, 31.0, 16.4, 14.0.
HRMS (ESI) calcd for $[M+Na]^+$ $C_{15}H_{18}NaO_2$, m/z : 253.1199, found : 253.1200.
IR ν [cm^{-1}] 3084, 3062, 3028, 2982, 2956, 1704, 1494, 1453, 1305, 1185, 699.

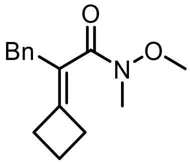

38

Compound **38.** The ene ester **37** (3.0 g, 13 mmol, 1.0 equiv) was dissolved in THF (80 mL) at -78 ºC, and MeNHOMe·HCl (2.54 g, 26 mmol, 2.0 equiv) was added. After the mixture was stirred at -78 °C for 0.5 h, LiHMDS (52 mL, 1.0 M in THF, 52 mmol, 4.0 equiv) was added to the flask. After the temperature of the mixture was warmed to rt and stirring was continued for 0.5 h, the mixture was quenched with saturated NH_4Cl aqueous solution and extracted with EtOAc (3 x 50 mL). The combined organic layer was washed with brine, dried over Na_2SO_4 and concentrated under vacuum. The residue was purified by flash column chromatography (EtOAc: petroleum ether = 1:6) to give weinreb amide product **38** (1.33 g, 5.4 mmol, 42% yield) as colorless oil.
^{1}H NMR (500 MHz, $CDCl_3$): δ 7.22 – 7.10 (m, 5H), 3.48 (s, 2H), 3.36 (s, 3H), 2.95 (s, 3H), 2.79–2.76 (m, 2H), 2.72 – 2.69 (m, 2H), 2.00 – 1.93 (m, 2H).
^{13}C NMR (125 MHz, $CDCl_3$): δ 168.5, 144.5, 139.2, 128.5, 128.2, 126.1, 126.0, 60.6, 35.3, 33.6, 30.6, 30.0, 16.2.
HRMS (ESI) calcd for $[M+Na]^+$ $C_{15}H_{19}NNaO_2$, m/z : 268.1308, found : 268.1313.
IR ν [cm^{-1}] 3061, 3027, 2933, 1648, 1494, 1454, 1376, 700.

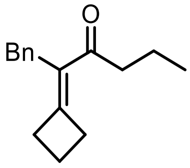

39

Compound **39.** To a stirred solution of weinreb amide **37** (106 mg, 0.43 mmol, 1.0 equiv) in Et_2O (5 mL) at 0 ºC was slowly added *n*-PrMgBr (0.65 mL, 0.65 mmol, 1.5 equiv). After stirred for 8 h, and the reaction was quenched carefully with saturated NH_4Cl aqueous solution and extracted with Et_2O (3 x 10 mL). The combined organic layer was washed with brine, dried over Na_2SO_4 and concentrated under vacuum. The residue was purified by flash column chromatography (Et_2O: petroleum ether = 1:20) to give enone product **39** (42 mg, 0.18 mmol, 43% yield) as colorless oil.

^{1}H NMR (500 MHz, $CDCl_3$): δ 7.26 – 7.14 (m, 5H), 3.52 (s, 2H), 3.20 – 3.17 (m, 2H), 2.94 (dd, J = 8.0, 7.0 Hz, 2H), 2.41 (t, J = 7.5 Hz, 2H), 2.13 – 2.07 (m, 2H), 1.61 – 1.54 (m, 2H), 0.87 (t, J = 7.5 Hz, 3H).
^{13}C NMR (125 MHz, $CDCl_3$): δ 200.5, 159.1, 140.4, 133.4, 128.3, 128.2, 125.7, 43.6, 34.3, 33.1, 31.9, 17.4, 16.5, 13.8.
HRMS (ESI) calcd for $[M+Na]^+$ $C_{16}H_{20}NaO$, m/z : 251.1406, found : 251.1407.
IR ν [cm^{-1}] 3083, 3061, 3017, 2959, 2931, 2873, 1691, 1659, 1494, 1453, 700.

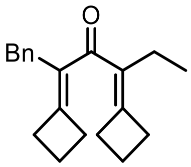

2d

Compound **2d.** To a stirred solution of di*iso*propylamine (1.1 mL, 8.0 mmol) in THF (5.6 mL) at 0 ºC was slowly added *n*-BuLi (3.2 mL, 2.5 M in hexane, 8.0 mmol) for 0.5 h. Compound enone **39** (114 mg, 0.5 mmol, 1.0 equiv) was dissolved in THF (5 mL) at -78 ℃, and the above freshly prepared LDA solution (1.9 mL, 1.5 mmol, 3.0 equiv) was added. After the mixture was stirred at the same temperature for 1 h, $CeCl_3$ (370 mg, 1.5 mmol, 3.0 equiv) in THF (5 mL) was added, and stirred for another 5 h. And then cyclobutanone **23** (0.11 mL, 1.5 mmol, 3.0 equiv) was added and the resultant solution was stirred at -78 ºC for 2 h. The reaction was quenched carefully with saturated NH_4Cl aqueous solution and extracted with EtOAc (3 x 10 mL). The combined organic layer was washed with brine, dried over Na_2SO_4 and concentrated under vacuum. The crude product was purified by a short flash column chromatography (EtOAc: petroleum ether = 1:20). The above product (77 mg, 0.26 mmol, 1.0 equiv) was dissolved in toluene (5 mL) at rt, and burgess reagent (186 mg, 0.78 mmol, 3.0 equiv) was added. After stirred at 60 ºC for 0.5 h, starting materials transformed completely (determined by TLC). Then, the mixture was cooled to room temperature and DBU (0.12 mL, 0.78 mmol, 3.0 equiv) was added to the mixture and stirred at same temperature for 6 h. Upon completion, the reaction was quenched carefully with water and extracted with EtOAc (3 x 10 mL). The combined organic layer was washed with brine, dried over Na_2SO_4 and concentrated under vacuum. The residue was purified by flash column chromatography (EtOAc: petroleum ether = 1:60) to give dienone product **2d** (39 mg, 0.14 mmol, 28% yield for two steps) as a colorless oil.
Method for activating $CeCl_3$: anhydrous $CeCl_3$ was heated to 140 ºC in vacuo for 2 hours. After cooling the system to room temperature, THF was added and the mixture was stirred for 1.0 h.

^{1}H NMR (500 MHz, $CDCl_3$) δ 7.24 – 7.13 (m, 5H), 3.53 (s, 2H), 2.88 (q, *J* = 7.5 Hz, 4H), 2.69 (dd, *J* = 8.0, 7.5 Hz, 2H), 2.47 (dd, *J* = 8.0, 7.5 Hz, 2H), 2.15 (q, *J* = 7.5 Hz, 2H), 2.04 – 1.96 (m, 2H), 1.88 – 1.79 (m, 2H), 0.86 (t, *J* = 7.5 Hz, 3H).
^{13}C NMR (125 MHz, $CDCl_3$): *δ* 197.6, 153.1, 150.3, 140.3, 135.8, 133.9, 128.9, 128.2, 125.8, 34.0, 32.6, 31.8, 31.5, 30.8, 21.7, 16.7, 16.6, 13.3.
HRMS (ESI) calcd for $[M+Na]^+$ $C_{20}H_{24}NaO$, m/z : 303.1719, found : 303.1719.
IR *v* [cm^{-1}] 3084, 3061, 3027, 2960, 2928, 2872, 1659, 1627, 1494, 1331, 700.

3.5 Preparation of starting material 2e

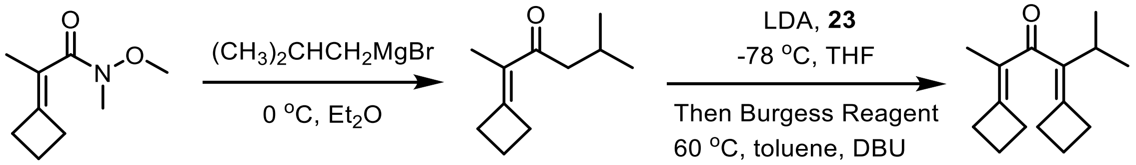

24 **40** **2e**

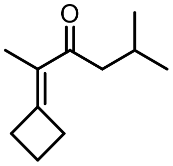

40

Compound **40.** To a stirred solution of weinreb amide **24** (900 mg, 5.3 mmol, 1.0 equiv) in Et_2O (35 mL) at 0 ºC was slowly added $(CH_3)_2CHCH_2MgBr$ (8.0 mL, 8.0 mmol, 1.5 equiv). After stirred for 3 h, the reaction was quenched carefully with saturated NH_4Cl aqueous solution and extracted with Et_2O (3 x 10 mL). The combined organic layer was washed with brine, dried over Na_2SO_4 and concentrated under vacuum. The residue was purified by flash column chromatography (Et_2O: petroleum ether = 1:20) to give enone product **40** (400 mg, 2.4 mmol, 45% yield) as a colorless oil.
^{1}H NMR (500 MHz, $CDCl_3$) δ 3.11 – 3.08 (m, 2H), 2.87 – 2.78 (m, 2H), 2.32 (d, *J* = 7.0 Hz, 2H), 2.14 (dp, *J* = 13.5, 6.5 Hz, 1H), 2.05 (p, *J* = 8.0 Hz, 2H), 1.66 – 1.60 (m, 3H), 0.91 (s, 3H), 0.90 (s, 3H).
^{13}C NMR (125 MHz, $CDCl_3$): *δ* 200.9, 157.1, 129.6, 50.3, 34.1, 31.7, 24.6, 22.8, 16.4, 12.9.
HRMS (ESI) calcd for $[M+Na]^+$ $C_{11}H_{18}NaO$, m/z: 189.1250, found: 189.1248.
IR *v* [cm^{-1}] 2956, 2870, 1658, 1467, 1365, 1083, 806.

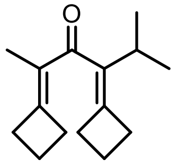

2e

Compound **2e.** To a stirred solution of di*iso*propylamine (1.1 mL, 8.0 mmol) in THF (5.7 mL) at 0 ºC was slowly added *n*-BuLi (3.2 mL, 2.5 M in hexane, 8.0 mmol) for 0.5 h. Compound enone **40** (260 mg, 1.56 mmol, 1.0 equiv) was dissolved in THF (10 mL) at -78 °C, and the above freshly prepared LDA solution (5.9 mL, 4.7 mmol, 3.0 equiv) was added. After the mixture was stirred at the same temperature for 1 h, $CeCl_3$ (1.15 g, 4.7 mmol, 3.0 equiv) in THF (10 mL) was added and stirred for another 5 h. And then cyclobutanone **23** (0.35 mL, 4.7 mmol, 3.0 equiv) was added and the resultant solution was stirred at -78 ºC for 2 h. The reaction was quenched carefully with saturated NH_4Cl aqueous solution and extracted with EtOAc (3 x 30 mL). The combined organic layer was washed with brine, dried over Na_2SO_4 and concentrated under vacuum. The crude product was purified by a short flash column chromatography (EtOAc: petroleum ether = 1:20). The above product (195 mg, 0.83 mmol, 1.0 equiv) was dissolved in toluene (10 mL) at rt, and burgess reagent (593 mg, 2.5 mmol, 3.0 equiv) was added. After stirred at 60 ºC for 0.5 h, starting materials transformed completely (determined by TLC). Then, the mixture was cooled to room temperature and DBU (0.37 mL, 2.5 mmol, 3.0 equiv) was added to the mixture and stirred at that temperature for 12 h. Upon completion, the reaction was quenched carefully with water and extracted with EtOAc (3 x 20 mL). The combined organic layer was washed with brine, dried over Na_2SO_4 and concentrated under vacuum. The residue was purified by flash column chromatography (EtOAc: petroleum ether = 1:60) to give dienone **2e** product (98 mg, 0.45 mmol, 29% yield for two steps) as a colorless oil.

Method for activating $CeCl_3$: anhydrous $CeCl_3$ was heated to 140 ºC in vacuo for 2 hours. After cooling the system to room temperature, THF was added and the mixture was stirred for 1.0 h.

^{1}H NMR (500 MHz, $CDCl_3$): δ 2.91 – 2.88 (m, 2H), 2.82 – 2.78 (m, 2H), 2.77 – 2.73 (m, 2H), 2.71 – 2.68 (m, 2H), 2.57 – 2.52 (m, 1H), 1.96 (p, *J* = 12.5, 8.0 Hz, 2H), 1.96 (p, *J* = 12.5, 8.0 Hz, 2H), 1.65 – 1.64 (m, 3H), 1.11 (s, 3H), 1.09 (s, 3H).

^{13}C NMR (125 MHz, $CDCl_3$): δ 199.1, 153.9, 146.5, 139.0, 130.0, 32.7, 32.0, 31.4, 31.0, 30.0, 21.5, 16.9, 16.6, 12.6.

HRMS (ESI) calcd for $[M+Na]^+$ $C_{15}H_{22}NaO$, m/z: 241.1563, found: 241.1562.

IR ν [cm^{-1}] 2957, 2872, 1631, 1459, 1307, 1098, 934.

3.6 Preparation of starting material 2f

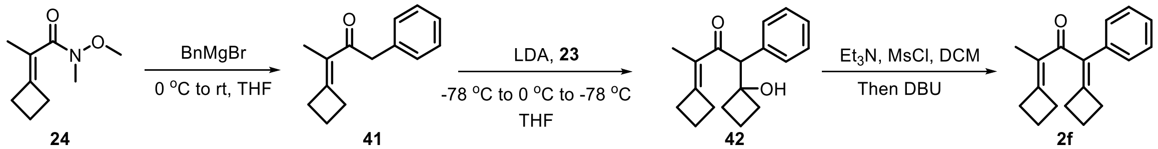

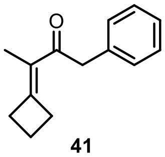

41

Compound **41.** To a stirred solution of weinreb amide **24** (676 mg, 4.0 mmol, 1.0 equiv) in THF (10 mL) at 0 ºC was slowly added BnMgBr (6.0 mL, 1.0 M in THF, 6.0 mmol, 1.5 equiv). The temperature of the mixture was warmed to rt and stirring was continued for 4 h, the reaction was quenched carefully with saturated NH_4Cl aqueous solution and extracted with EtOAc (4 x 50 mL). The combined organic layer was washed with brine, dried over Na_2SO_4 and concentrated under vacuum. The residue was purified by flash column chromatography (EtOAc: petroleum ether = 1:20) to give enone product **41** (392 mg, 1.96 mmol, 49% yield) as a yellow oil.

^{1}H NMR (500 MHz, $CDCl_3$) δ 7.31 (dd, *J* = 10.2, 4.6 Hz, 2H), 7.24 (dd, *J* = 8.5, 6.5 Hz, 1H), 7.17 – 7.13 (m, 2H), 3.81 (s, 2H), 3.22 – 3.10 (m, 2H), 2.89 – 2.82 (m, 2H), 2.07 (p, *J* = 8.0 Hz, 2H), 1.74 – 1.67 (m, 3H).

^{13}C NMR (125 MHz, $CDCl_3$) *δ* 198.2, 159.0, 134.9, 129.5, 129.0, 128.4, 126.5, 47.6, 34.3, 31.8, 16.5, 13.1.

HRMS (ESI) calcd for $[M+Na]^+$ $C_{14}H_{16}NaO$, m/z: 223.1093 , found: 223.1094.

IR *v* [cm^{-1}] 3062, 3029, 2956, 2922, 1716, 1662, 1495, 1267, 1088, 1021, 704.

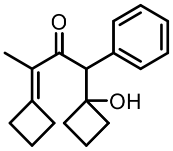

42

Compound **42.** To a stirred solution of di*iso*propylamine (1.4 mL, 10.0 mmol) in THF (4.6 mL) at 0 ºC was slowly added *n*-BuLi (4.0 mL, 2.5 M in hexane, 1.3 mmol) for 0.5 h. Enone **41** (348 mg, 1.74 mmol, 1.0 equiv) was dissolved in THF (6 mL) at -78 °C, and the above freshly prepared LDA solution (2.6 mL, 2.6 mmol, 1.5 equiv) was added. The resulting suspension was stirred at -78 °C for 5 min and then at 0 °C for 1 h. The resultant solution was then cooled to -78 °C. Cyclobutanone **23** (0.2 mL, 2.6 mmol, 1.5 equiv) was added dropwise and the resultant solution was stirred at -78 °C for 6 h. The reaction was quenched carefully with saturated NH_4Cl aqueous solution and extracted with EtOAc (3 x 50 mL). The combined organic layer was washed with brine, dried over Na_2SO_4 and concentrated under vacuum. The residue was purified by flash

column chromatography (EtOAc: petroleum ether = 1:25 to 1:17) to give adol product **42** (186 mg, 0.69 mmol, 40% yield) as a yellow oil and starting material **41** (158 mg, 0.79 mmol).
^{1}H NMR (500 MHz, $CDCl_3$) δ 7.38 – 7.24 (m, 5H), 4.72 (s, 1H), 4.25 (s, 1H), 3.26 – 3.17 (m, 1H), 3.17 – 3.06 (m, 1H), 2.80 (td, *J* = 8.0, 1.0 Hz, 2H), 2.29 – 2.20 (m, 1H), 2.12 – 1.96 (m, 4H), 1.93 – 1.81 (m, 2H), 1.68 – 1.62 (m, 4H).
^{13}C NMR (125 MHz, $CDCl_3$) *δ* 203.4, 161.7, 134.1, 129.9, 128.9, 128.3, 127.3, 59.8, 36.5, 34.3, 32.5, 32.1, 16.5, 13.1, 12.8.
HRMS (ESI) calcd for $[M+Na]^+$ $C_{18}H_{22}NaO_2$, m/z: 293.1512 , found: 293.1512.
IR *ν* [cm^{-1}] 3061, 3027, 2983, 2949, 1632, 1452, 1273, 1100, 1025, 702.

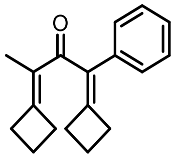

2f

Compound **2f.** To a solution of **42** (186 mg, 0.69 mmol, 1.0 equiv) in DCM (3 mL) at 0 ºC, triethylamine (1.0 mL, 6.9 mmol, 10.0 equiv) and methanesulfonyl chloride (0.21 mL, 2.72 mmol, 4.0 equiv) were added carefully. After stirring for 1 at rt, the resulting mixture was quenched with distilled water. The aqueous layer was extracted with EtOAc (3 x 50 mL). The combined organic layer was washed with brine, dried over Na_2SO_4. After filtration and concentration under reduced pressure, the resulting crude residue was directly dissolved in DCM (3 mL), and DBU (0.31 mL, 2.07 mmol, 3.0 equiv) was added at 23 °C and stirred at that temperature for 4 h. The reaction mixture was quenched with water, and the aqueous layer was extracted with EtOAc (3 x 50 mL). The combined organic layer was washed with brine, dried over Na_2SO_4. After the filtration and concentration under reduced pressure, the resulting crude residue was purified by flash column chromatography on silica gel (EtOAc/hexane = 1/30 to 1/25) to provide dienone **2f** as a yellow oil (114 mg, 0.46 mmol, 66%).
^{1}H NMR (500 MHz, $CDCl_3$) *δ* 7.31 – 7.28 (m, 2H), 7.21 – 7.18 (m, 3H), 3.01 – 2.97 (m, 2H), 2.90 – 2.86 (m, 2H), 2.84 – 2.77 (m, 4H), 2.09 (p, *J* = 8.0 Hz, 2H), 1.93 (p, *J* = 8.0 Hz, 2H), 1.70 – 1.69 (m, 3H).
^{13}C NMR (125 MHz, $CDCl_3$) *δ* 197.4, 160.0, 145.1, 135.5, 135.2, 129.7, 128.2, 127.0, 126.6, 33.3, 33.0, 31.8, 31.6, 17.8, 16.4, 12.3.
HRMS (ESI) calcd for $[M+Na]^+$ $C_{18}H_{20}NaO$, m/z: 275.1406 , found: 275.1406.
IR *ν* [cm^{-1}] 3054, 2954, 2916, 1634, 1493, 1324, 1048, 773, 697.

3.7 Preparation of starting material 2g

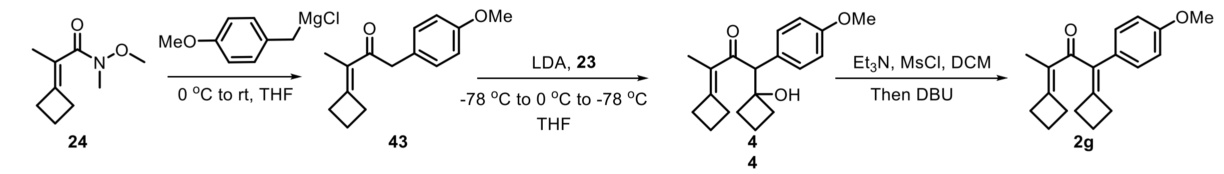

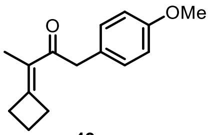

43

Compound **43.** To a stirred solution of weinreb amide **24** (1.23 g, 7.3 mmol, 1.0 equiv) in THF (20 mL) at 0 ºC was slowly added 4-Methoxybenzylmagnesium chloride (55 mL, 0.2 M in THF, 11.0 mmol, 1.5 equiv). The temperature of the mixture was warmed to rt and stirring was continued for 4 h, the reaction was quenched carefully with saturated NH_4Cl aqueous solution and extracted with EtOAc (4 x 80 mL). The combined organic layer was washed with brine, dried over Na_2SO_4 and concentrated under vacuum. The residue was purified by flash column chromatography (EtOAc: petroleum ether = 1:20) to give enone product **43** (731 mg, 3.2 mmol, 43% yield) as a yellow oil.

^{1}H NMR (400 MHz, $CDCl_3$) δ 7.07 (d, J = 8.5 Hz, 2H), 6.85 (d, J = 8.5 Hz, 2H), 3.78 (s, 3H), 3.74 (s, 2H), 3.20 – 3.08 (m, 2H), 2.90 – 2.77 (m, 2H), 2.06 (p, J = 8.0 Hz, 2H), 1.71 – 1.70 (m, 3H).

^{13}C NMR (100 MHz, $CDCl_3$) δ 198.5, 158.7, 158.3, 130.4, 128.9, 126.9, 113.8, 55.2, 46.7, 34.3, 31.8, 16.4, 13.0.

HRMS (ESI) calcd for $[M+Na]^+$ $C_{15}H_{18}NaO_2$, m/z: 253.1199 , found: 253.1199.

IR v [cm^{-1}] 2956, 2837, 1702, 1655, 1612, 1512, 1248, 1034, 820.

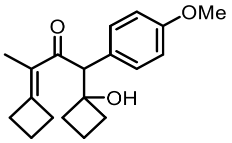

44

Compound **44.** To a stirred solution of di*iso*propylamine (1.4 mL, 10.0 mmol) in THF (4.6 mL) at 0 ºC was slowly added *n*-BuLi (4.0 mL, 2.5 M in hexane, 10.0 mmol) for 0.5 h. Enone **43** (462 mg, 2.0 mmol, 1.0 equiv) was dissolved in THF (6 mL) at -78 °C, and the above freshly prepared LDA solution (3.0 mL, 3.0 mmol, 1.5 equiv) was added. The resulting suspension was stirred at -78 °C for 5 min and then at 0 °C for 1 h. The resultant solution was then cooled to -78 °C. Cyclobutanone **23** (0.23 mL, 3.0 mmol, 1.5 equiv) was added dropwise and the resultant solution was stirred at -78 °C for 6 h. The reaction was quenched carefully with saturated NH_4Cl aqueous solution and extracted with EtOAc (3 x 50 mL). The combined organic layer was washed with brine, dried over Na_2SO_4 and concentrated under vacuum. The residue was purified by flash

column chromatography (EtOAc: petroleum ether = 1:25 to 1:17) to give adol product **44** (439 mg, 1.46 mmol, 73% yield) as a yellow oil and starting material **43** (112 mg, 0.48 mmol).

^{1}H NMR (400 MHz, $CDCl_3$) δ 7.14 (d, J = 8.5 Hz, 2H), 6.85 (d, J = 8.5 Hz, 2H), 4.67 (s, 1H), 4.16 (s, 1H), 3.77 (s, 3H), 3.24 – 3.02 (m, 2H), 2.76 (dd, J = 12.0, 4.7 Hz, 2H), 2.26 – 2.11 (m, 1H), 2.11 – 1.92 (m, 4H), 1.89 – 1.79 (m, 2H), 1.63 – 1.60 (m, 3H), 1.59 – 1.54 (m, 1H).

^{13}C NMR (100 MHz, $CDCl_3$) δ 203.6, 161.4, 158.8, 130.8, 128.8, 126.1, 113.7, 58.8, 55.1, 36.3, 34.3, 32.4, 32.0, 16.5, 13.1, 12.7.

HRMS (ESI) calcd for $[M+Na]^+$ $C_{19}H_{24}NaO_3$, m/z: 323.1618 , found: 323.1613.

IR v [cm^{-1}] 2953, 2836, 1631, 1611, 1511, 1252, 1033, 790.

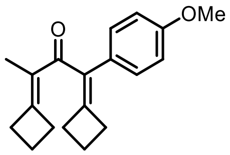

2g

Compound **2g.** To a solution of **44** (416 mg, 1.39 mmol, 1.0 equiv) in DCM (5 mL) at 0 ºC, triethylamine (2.0 mL, 13.8 mmol, 10.0 equiv) and methanesulfonyl chloride (0.43 mL, 5.58 mmol, 4.0 equiv) were added carefully. After stirring for 1 at rt, the resulting mixture was quenched with distilled water. The aqueous layer was extracted with EtOAc (3 x 70 mL). The combined organic layer was dried over Na_2SO_4. After filtration and concentration under reduced pressure, the resulting crude residue was directly dissolved in DCM (5 mL), and DBU (0.62 mL, 4.14 mmol, 3.0 equiv) was added at 23 °C and stirred for 4 h at that temperature. The reaction mixture was quenched with water, and the aqueous layer was extracted with EtOAc (3 x 70 mL). The combined organic layer was washed with brine, dried over Na_2SO_4. After the filtration and concentration under reduced pressure, the resulting crude residue was purified by flash column chromatography on silica gel (EtOAc/hexane = 1/30 to 1/25) to provide dienone **2g** as a yellow oil (287 mg, 1.01 mmol, 73%).

^{1}H NMR (400 MHz, $CDCl_3$) δ 7.11 (d, J = 8.5 Hz, 2H), 6.83 (d, J = 8.5 Hz, 2H), 3.78 (s, 3H), 2.98 – 2.93 (m, 2H), 2.91 – 2.82 (m, 2H), 2.81 – 2.73 (m, 4H), 2.11 – 2.02 (m, 2H), 1.92 (p, J = 8.0 Hz, 2H), 1.69 – 1.65 (m, 3H).

^{13}C NMR (100 MHz, $CDCl_3$) δ 197.6, 159.9, 158.2, 142.9, 134.6, 129.6, 128.2, 113.6, 55.1, 33.3, 32.8, 31.7, 31.4, 17.8, 16.3, 12.3.

HRMS (ESI) calcd for $[M+Na]^+$ $C_{19}H_{22}NaO_2$, m/z: 305.1512 , found: 305.1511.

IR v [cm^{-1}] 2955, 2837, 1633, 1607, 1511, 1250, 1180, 1034, 833.

3.8 Preparation of starting material 2h

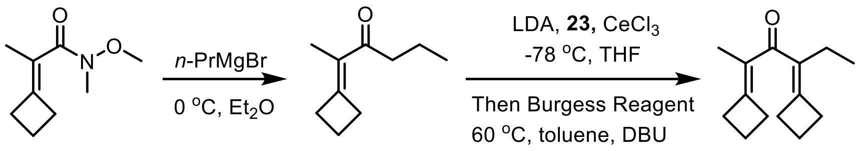

24 **45** **2h**

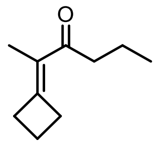

45

Compound **45.** To a stirred solution of weinreb amide **24** (338 mg, 2.0 mmol, 1.0 equiv) in Et_2O (15 mL) at 0 ºC was slowly added *n*-PrMgBr (2.4 mL, 1.0 M in THF, 2.4 mmol, 1.2 equiv). After the mixture was stirred for 4 h, the reaction was quenched carefully with saturated NH_4Cl aqueous solution and extracted with Et_2O (3 x 20 mL). The combined organic layer was washed with brine, dried over Na_2SO_4 and concentrated under vacuum. The residue was purified by flash column chromatography (Et_2O: petroleum ether = 1:10) to give enone product **45** (255 mg, 1.67 mmol, 84% yield) as a colorless oil.

^{1}H NMR (500 MHz, $CDCl_3$): δ 3.01 – 2.98 (m, 2H), 2.74 – 2.71 (m, 2H), 2.31 (t, J = 7.5 Hz, 2H), 1.97 – 1.91 (m, 2H), 1.54 – 1.53 (m, 3H), 1.49 (q, J = 7.0 Hz, 2H), 0.80 (t, J = 7.5 Hz, 3H).

^{13}C NMR (125 MHz, $CDCl_3$): δ 200.5, 157.0, 129.2, 43.0, 33.9, 31.4, 17.1, 16.2, 13.7, 12.4.

HRMS (ESI) calcd for $[M+Na]^+$ $C_{10}H_{16}NaO$, m/z: 175.1093, found: 175.1093.

IR v [cm^{-1}] 2960, 2874, 1659, 1456, 1286, 899.

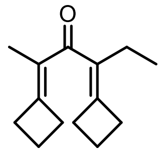

2h

Compound **2h.** To a stirred solution of di*iso*propylamine (1.7 mL, 12.0 mmol) in dry THF (8.5 mL) at 0 ºC was slowly added *n*-BuLi (4.8 mL, 2.5 M in hexane, 12.0 mmol) for 0.5 h. Compound enone **45** (107 mg, 0.7 mmol, 1.0 equiv) was dissolved in THF (6 mL) at -78 °C, and the above freshly prepared LDA solution (2.6 mL, 2.1 mmol, 3.0 equiv) was added. After the mixture was stirred at the same temperature for 1 h, $CeCl_3$ (518 mg, 2.1 mmol, 3.0 equiv) in THF (5 mL) was added, and the reaction stirred another 4 h. And then cyclobutanone **23** (0.16 mL, 2.1 mmol, 3.0 equiv) was added and the resultant solution was stirred at –78 °C for 0.5 h. The reaction was quenched carefully with saturated NH_4Cl aqueous solution and extracted with EtOAc (3 x 50 mL). The combined organic layer was washed with brine, dried over Na_2SO_4 and concentrated under vacuum. The crude product was purified by a short flash column chromatography (EtOAc:

petroleum ether = 1:20). The above product (61 mg, 0.27 mmol, 1.0 equiv) was dissolved in toluene (5 mL) at rt, and burgess reagent (196 mg, 0.81 mmol, 3.0 equiv) was added. After stirred at 60 ºC for 1 h, starting materials transformed completely (determined by TLC). Then, the mixture was cooled to room temperature and DBU (0.12 mL, 0.81 mmol, 3.0 equiv) was added to the mixture and stirred at that temperature for 4 h. Upon completion, the reaction was quenched carefully with water and extracted with EtOAc (3 x 10 mL). The combined organic layer was washed with brine, dried over Na_2SO_4 and concentrated under vacuum. The residue was purified by flash column chromatography (EtOAc: petroleum ether = 1:50) to give dienone product **2q** (40 mg, 0.20 mmol, 29% yield for two steps) as colorless oil.

Method for activating $CeCl_3$: anhydrous $CeCl_3$ was heated to 140 ºC in vacuo for 2 hours. After cooling the system to room temperature, THF was added and the mixture was stirred for 1.0 h.
^{1}H NMR (500 MHz, $CDCl_3$): δ 2.84 – 2.80 (m, 2H), 2.78 – 2.71 (m, 6H), 2.18 (q, J = 7.5 Hz, 2H), 1.95 (pd, J = 8.0, 2.5 Hz, 4H), 1.68 – 1.63 (m, 3H), 0.93 (t, J = 1.5 Hz, 3H).
^{13}C NMR (125 MHz, $CDCl_3$): δ 198.7, 151.0, 150.5, 135.8, 129.6, 32.2, 32.1, 31.0, 30.8, 21.5, 16.6, 16.5, 13.4, 12.9.
HRMS (ESI) calcd for $[M+Na]^+$ $C_{14}H_{20}NaO$, m/z: 227.1406, found: 227.1406.
IR ν [cm^{-1}] 2959, 1631, 1457, 1334, 910, 717.

3.9 Preparation of starting material 2i

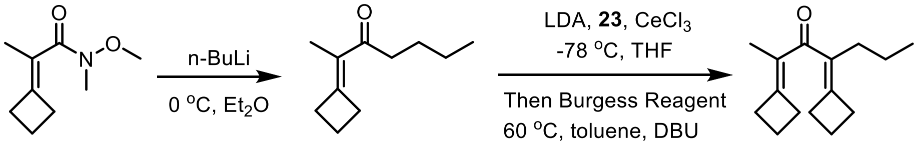

24 **46** **2i**

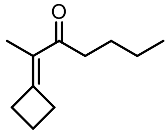

46

Compound **46.** To a stirred solution of weinreb amide **24** (520 mg, 3.1 mmol, 1.0 equiv) in Et_2O (20 mL) at 0 ºC was slowly added *n*-BuLi (1.9 mL, 4.65 mmol, 1.5 equiv). After the mixture was stirred for 3 h, the reaction was quenched carefully with saturated NH_4Cl aqueous solution and extracted with Et_2O (3 x 10 mL). The combined organic layer was washed with brine, dried over Na_2SO_4 and concentrated under vacuum. The residue was purified by flash column chromatography (Et_2O: petroleum ether = 1:20) to give enone product **46** (421 mg, 2.53 mmol, 82% yield) as a colorless oil.

^{1}**H NMR** (500 MHz, $CDCl_3$): δ 3.05 – 3.01 (m, 2H), 2.79 – 2.75 (m, 2H), 2.37 (t, J = 7.5 Hz, 2H), 2.02 – 1.95 (m, 2H), 1.59 – 1.57 (m, 3H), 1.52 – 1.46 (m, 2H), 1.28 – 1.21 (m, 2H), 0.83 (t, J = 7.5 Hz, 3H).
^{13}**C NMR** (125 MHz, $CDCl_3$): δ 200.9, 157.1, 129.3, 40.9, 34.0, 31.5, 26.0, 22.4, 16.3, 13.8, 12.6.
HRMS (ESI) calcd for $[M+Na]^+$ $C_{11}H_{18}NaO$, m/z: 189.1250, found: 189.1250.
IR ν [cm^{-1}] 2957, 2932, 2872, 1659, 1464, 1085, 994.

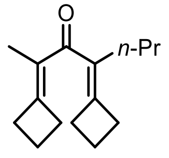

2i

Compound **2i.** To a stirred solution of di*iso*propylamine (1.7 mL, 12.0 mmol) in THF (8.5 mL) at 0 ºC was slowly added *n*-BuLi (4.8 mL, 2.5 M in hexane, 12.0 mmol) for 0.5 h. Compound enone **46** (291 mg, 1.75 mmol, 1.0 equiv) was dissolved in THF (10 mL) at -78 ℃, and the above freshly prepared LDA solution (6.6 mL, 5.25 mmol, 3.0 equiv) was added. After the mixture was stirred at the same temperature for 1 h, $CeCl_3$ (1.3 g, 5.25 mmol, 3.0 equiv) in THF (10 mL) was added. Cyclobutanone **23** (0.39 mL, 5.25 mmol, 3.0 equiv) was added before the mixture was stirred for another 5 h, and the resultant solution was stirred at -78 ºC for 2 h. The reaction was quenched carefully with saturated NH_4Cl aqueous solution and extracted with EtOAc (3 x 30 mL). The combined organic layer was washed with brine, dried over Na_2SO_4 and concentrated under vacuum. The crude product was purified by a short flash column chromatography (EtOAc: petroleum ether = 1:20). The above product (330 mg, 1.4 mmol, 1.0 equiv) was dissolved in toluene (15 mL) at rt, and burgess reagent (1.0 g, 4.2 mmol, 3.0 equiv) was added. After stirred at 60 ºC for 0.5 h, starting materials transformed completely (determined by TLC). Then, the mixture was cooled to room temperature and DBU (0.63 mL, 4.2 mmol, 3.0 equiv) was added to the mixture and stirred at that temperature for 7 h. Upon completion, the reaction was quenched carefully with water and extracted with EtOAc (3 x 20 mL). The combined organic layer was washed with brine, dried over Na_2SO_4 and concentrated under vacuum. The residue was purified by flash column chromatography (EtOAc: petroleum ether = 1:60) to give dienone product **2i** (186 mg, 0.85 mmol, 61% yield for two steps) as a colorless oil.
Method for activating $CeCl_3$: anhydrous $CeCl_3$ was heated to 140 ºC in vacuo for 2 hours. After cooling the system to room temperature, THF was added and the mixture was stirred for 1.0 h.
^{1}**H NMR** (500 MHz, $CDCl_3$): δ 2.84 – 2.71 (m, 8H), 2.13 (t, J = 8.0 Hz, 2H), 2.00 – 1.92 (m, 4H), 1.66 (t, J = 1.5 Hz, 3H), 1.38 – 1.30 (m, 2H), 0.88 (t, J = 7.5 Hz, 3H).
^{13}**C NMR** (125 MHz, $CDCl_3$): δ 198.8, 151.3, 150.7, 134.6, 129.6, 32.2, 32.0, 31.1, 31.1, 30.4,

22.2, 16.7, 16.6, 14.3, 13.1.
HRMS (ESI) calcd for $[M+Na]^+$ $C_{15}H_{22}NaO$, m/z: 241.1563 , found: 241.1563 .
IR *ν* [cm^{-1}] 2957, 2871, 1632, 1455, 1331, 961.

3.10 Preparation of starting material 2j

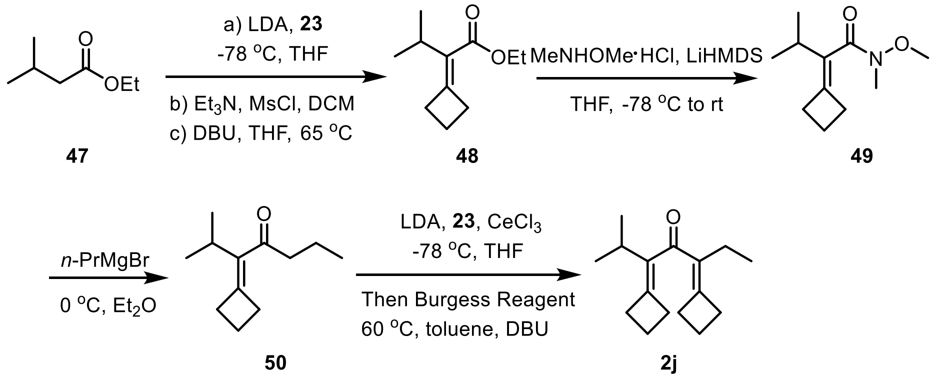

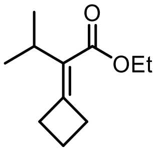

48

Compound **48.** To a stirred solution of di*iso*propylamine (6.7 mL, 45.0 mmol, 1.5 equiv) in THF (45 mL) at 0 °C was slowly added *n*-BuLi (18 mL, 2.5 M in hexane, 45.0 mmol, 1.5 equiv) for 0.5 h. Compound ethyl isovalerate **47** (4.5 mL, 30 mmol, 1.0 equiv) was dissolved in THF (25 mL) and added to the above freshly prepared LDA solution. After the mixture was stirred at the same temperature for 1 h, cyclobutanone **23** (2.7 mL, 36 mmol, 1.2 equiv) was added and the resultant solution was stirred at –78 °C for another 0.5 h. The reaction was quenched carefully with saturated NH_4Cl aqueous solution and extracted with EtOAc (3 x 50 mL). The combined organic layer was washed with brine, dried over Na_2SO_4 and concentrated under vacuum. The crude product (5.7 g, 30 mmol, 1.0 equiv) was dissolved in DCM (100 mL) at 0 °C, and Et_3N (50 mL, 360 mmol, 12.0 equiv) and MsCl (11.6 mL, 150 mmol, 5.0 equiv) were added. After stirred at rt for 7 h, starting materials transformed completely (determined by TLC), the reaction was quenched carefully with water and extracted with EtOAc (3 x 50 mL). The combined organic layer was washed with brine, dried over Na_2SO_4 and concentrated under vacuum. The crude product was redissolved in THF (60 mL) at rt, and DBU (13 mL, 90 mmol, 3.0 equiv) was added. After stirred at 65 °C for 4 h, the solution was concentrated under vacuum and the residue was purified by flash column chromatography (EtOAc: petroleum ether = 1:50) to give ene ester product **48** (2.6 g, 14.3 mmol, 48% yield for 3 steps) as a yellow oil.
^{1}H NMR (500 MHz, $CDCl_3$): *δ* 4.09 (q, *J* = 7.0 Hz, 2H), 3.02 – 2.98 (m, 2H), 2.82 – 2.78 (m, 2H), 2.59 – 2.54 (dt, *J* = 14.0, 7.0 Hz, 1H), 1.98 – 1.91 (m, 2H), 1.22 (t, *J* = 7.0 Hz, 3H), 1.09 (s, 3H),

1.07 (s, 3H).
^{13}C NMR (125 MHz, $CDCl_3$): δ 167.1, 157.5, 129.4, 59.3, 34.1, 31.0, 28.6, 20.7, 16.7, 14.3.
HRMS (ESI) calcd for $[M+Na]^+$ $C_{11}H_{18}NaO_2$, m/z: 205.1199, found: 205.1200.
IR ν [cm^{-1}] 2960, 1706, 1637, 1456, 1302, 1042, 669.

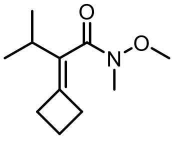

49

Compound **49.** The ene ester **48** (3.0 g, 16.5 mmol, 1.0 equiv) was dissolved in THF (80 mL) at -78 ºC, and MeNHOMe·HCl (3.22 g, 33 mmol, 2.0 equiv) was added. After the mixture was stirred at -78 ℃ for 0.5 h, LiHMDS (66 mL, 1.0 M in THF, 66 mmol, 4.0 equiv) was added to the flask. After the temperature of the mixture was warmed to rt and stirring was continued for 0.5 h, the mixture was quenched with saturated NH_4Cl aqueous solution and extracted with EtOAc (3 x 50 mL). The combined organic layer was washed with brine, dried over Na_2SO_4 and concentrated under vacuum. The residue was purified by flash column chromatography (EtOAc: petroleum ether = 1:6) to give weinreb amide product **49** (1.5 g, 7.6 mmol, 46% yield) as a colorless oil.
^{1}H NMR (500 MHz, $CDCl_3$): δ 3.63 (s, 3H), 3.19 (s, 3H), 2.73 – 2.70 (m, 2H), 2.64 – 2.61 (m, 2H), 2.48 (dt, *J* = 14.0, 7.0 Hz, 1H), 1.96 – 1.89 (m, 2H), 1.05 (s, 3H), 1.04 (s, 3H).
^{13}C NMR (125 MHz, $CDCl_3$): δ 168.8, 140.6, 132.3, 60.7, 34.2, 30.3, 29.8, 21.2, 16.6.
HRMS (ESI) calcd for $[M+Na]^+$ $C_{11}H_{19}NNaO_2$, m/z: 220.1308, found: 220.1310 .
IR ν [cm^{-1}] 2928, 2855, 1648, 1446, 1374, 996, 750.

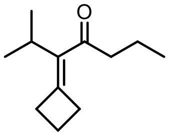

50

Compound **50.** To a stirred solution of weinreb amide **49** (200 mg, 1.0 mmol, 1.0 equiv) in Et_2O (10 mL) at 0 ºC was slowly added *n*-PrMgBr (1.5 mL, 1.5 mmol, 1.5 equiv). The mixture was stirred for 3 h, and the reaction was quenched carefully with saturated NH_4Cl aqueous solution and extracted with Et_2O (3 x 20 mL). The combined organic layer was washed with brine, dried over Na_2SO_4 and concentrated under vacuum. The residue was purified by flash column chromatography (Et_2O: petroleum ether = 1:20) to give enone product **50** (80 mg, 0.39 mmol, 39% yield) as a colorless oil.

^{1}H NMR (500 MHz, $CDCl_3$) δ 3.06 – 3.00 (m, 2H), 2.94 – 2.88 (m, 2H), 2.59 (dt, J = 14.0, 7.0 Hz, 1H), 2.36 (t, J = 7.5 Hz, 2H), 2.01 (dt, J = 11.5, 8.0 Hz, 2H), 1.62 – 1.54 (m, 2H), 1.10 (s, 3H), 1.08 (s, 3H), 0.89 (t, J = 7.5 Hz, 3H).
^{13}C NMR (125 MHz, $CDCl_3$): δ 202.7, 153.7, 139.8, 44.8, 34.2, 31.7, 29.2, 20.9, 17.5, 16.7, 13.9.
HRMS (ESI) calcd for $[M+Na]^+$ $C_{12}H_{20}NaO$, m/z: 203.1406, found: 203.1407.
IR ν [cm^{-1}] 2959, 2925, 1640, 1494, 1081, 669.

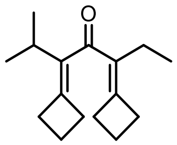

2j

Compound **2j.** To a stirred solution of di*iso*propylamine (1.1 mL, 8.0 mmol) in THF (5.6 mL) at 0 ºC was slowly added *n*-BuLi (3.2 mL, 2.5 M in hexane, 8.0 mmol) for 0.5 h. Compound enone **50** (60 mg, 0.33 mmol, 1.0 equiv) was dissolved in THF (3 mL) at -78 °C, and the above freshly prepared LDA solution (1.3 mL, 1.0 mmol, 3.0 equiv) was added. After the mixture was stirred at the same temperature for 1 h, $CeCl_3$ (246 mg, 1.0 mmol, 3.0 equiv) in THF (3 mL) was added, and stirred for another 5 h. And then cyclobutanone **23** (0.075 mL, 1.0 mmol, 3.0 equiv) was added and the resultant solution was stirred at -78 ºC for 0.5 h. The reaction was quenched carefully with saturated NH_4Cl aqueous solution and extracted with EtOAc (3 x 10 mL). The combined organic layer was washed with brine, dried over Na_2SO_4 and concentrated under vacuum. The crude product was purified by a short flash column chromatography (EtOAc: petroleum ether = 1:20). The above product (17 mg, 0.07 mmol, 1.0 equiv) was dissolved in toluene (4 mL) at rt, and burgess reagent (49 mg, 0.21 mmol, 3.0 equiv) was added. After the mixture was stirred at 60 ºC for 0.5 h, starting materials transformed completely (determined by TLC). Then, the mixture was cooled to room temperature and DBU (0.03 mL, 0.21 mmol, 3.0 equiv) was added to the mixture and stirred at that temperature for 9 h. Upon completion, the reaction was quenched carefully with water and extracted with EtOAc (3 x 10 mL). The combined organic layer was washed with brine, dried over Na_2SO_4 and concentrated under vacuum. The residue was purified by flash column chromatography (EtOAc: petroleum ether = 1:60) to give dienone product **2j** (13 mg, 0.056 mmol, 80% yield for two steps) as a colorless oil.
Method for activating $CeCl_3$: anhydrous $CeCl_3$ was heated to 140 ºC in vacuo for 2 hours. After cooling the system to room temperature, THF was added and the mixture was stirred for 1.0 h.
^{1}H NMR (500 MHz, $CDCl_3$): δ 2.92 – 2.89 (m, 2H), 2.82 – 2.78 (m, 4H), 2.74 (dd, J = 9.0, 7.0 Hz, 2H), 2.54 (dt, J = 14.0, 7.0 Hz, 1H), 2.17 (q, J = 8.0 Hz, 2H), 2.00 – 1.92 (m, 4H), 1.10 (d, J = 7.0 Hz, 6H), 0.93 (t, J = 7.5 Hz, 3H).
^{13}C NMR (125 MHz, $CDCl_3$): δ 198.5, 153.9, 146.5, 139.1, 136.6, 32.9, 32.1, 31.2, 31.1, 30.0,

21.5, 21.0, 16.9, 16.6, 13.6.
HRMS (ESI) calcd for $[M+Na]^+$ $C_{16}H_{24}NaO$, m/z: 255.1719, found: 255.1719.
IR *v* [cm^{-1}] 2957, 2929, 2870, 1631, 1331, 961.

3.11 Preparation of starting material 2k

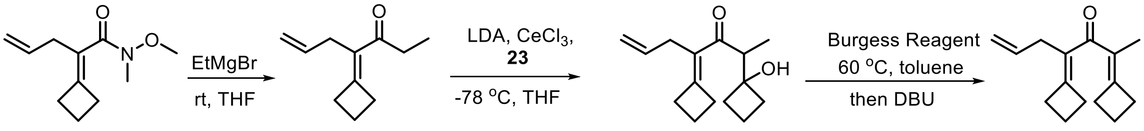

33 51 52 2k

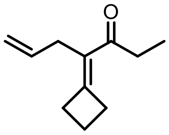
51

Compound **51.** To a stirred solution of weinreb amide **33** (467 mg, 2.4 mmol, 1.0 equiv) in THF (10 mL) at 0 ºC was slowly added EtMgBr (1.8 mL, 2.0 M in THF, 3.6 mmol, 1.5 equiv). The temperature of the mixture was warmed to rt and stirring was continued for 4 h before the reaction was quenched carefully with saturated NH_4Cl aqueous solution and extracted with EtOAc (4 x 50 mL). The combined organic layer was washed with brine, dried over Na_2SO_4 and concentrated under vacuum. The residue was purified by flash column chromatography (EtOAc: petroleum ether = 1:15) to give enone **51** (269 mg, 1.6 mmol, 67% yield) as a yellow oil.
^{1}H NMR (500 MHz, $CDCl_3$) δ 5.71 (ddt, J = 16.5, 10.0, 6.5 Hz, 1H), 4.94 – 4.86 (m, 2H), 3.11 – 3.08 (m, 2H), 2.85 – 2.82 (m, 4H), 2.42 (q, J = 7.0 Hz, 2H), 2.06 – 2.00 (m, 2H), 1.01 (t, J = 7.0 Hz, 3H).
^{13}C NMR (125 MHz, $CDCl_3$) δ 200.4, 159.1, 135.9, 131.6, 114.5, 34.5, 34.3, 31.5, 31.3, 16.4, 7.8.
HRMS (ESI) calcd for $[M+Na]^+$ $C_{11}H_{16}NaO$, m/z: 187.1093 , found: 187.1093.
IR *v* [cm^{-1}] 2922, 1633, 1384, 1113, 669, 653.

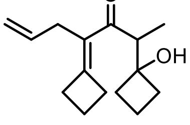

52

Compound **52.** To a stirred solution of di*iso*propylamine (1.4 mL, 10.0 mmol) in THF (4.6 mL) at 0 ºC was slowly added *n*-BuLi (4.0 mL, 2.5 M in hexane, 10.0 mmol) for 0.5 h. Compound enone **51** (246 mg, 1.5 mmol, 1.0 equiv) was dissolved in THF (4 mL) at -78 ℃, and the above freshly prepared LDA solution (4.5 mL, 4.5 mmol, 3.0 equiv) was added. After the mixture was stirred at the same temperature for 1 h, $CeCl_3$ (1.1 g, 4.5 mmol, 3.0 equiv) in anhydrous THF (4 mL) was added dropwise via a syringe. After the mixture was stirred at the same temperature for another 1

h, cyclobutanone **23** (0.34 mL, 4.5 mmol, 3.0 equiv) was added and the resultant solution was stirred at –78 °C for 0.5 h. The reaction was quenched carefully with saturated NH_4Cl aqueous solution and extracted with EtOAc (3 x 50 mL). The combined organic layer was washed with brine, dried over Na_2SO_4 and concentrated under vacuum. The residue was purified by flash column chromatography (EtOAc: petroleum ether = 1:12 to 1:6) to give adol product **52** (214 mg, 0.9 mmol, 60% yield) as a yellow oil and starting material **51** (68 mg, 0.4 mmol).

Method for activating $CeCl_3$: anhydrous $CeCl_3$ was heated to 140 ºC in vacuo for 2 hours. After cooling the system to room temperature, THF was added and the mixture was stirred for 1.0 h.

^{1}H NMR (400 MHz, $CDCl_3$) δ 5.71 (ddt, J = 16.4, 10.0, 6.0 Hz, 1H), 4.96 – 4.91 (m, 2H), 4.38 (s, 1H), 3.18 (s, 2H), 3.10 (dd, J = 14.4, 7.2 Hz, 1H), 2.90 – 2.86 (m, 4H), 2.12 – 1.96 (m, 4H), 1.94 – 1.87 (m, 1H), 1.82 – 1.71 (m, 2H), 1.54 – 1.42 (m, 1H), 1.07 (d, J = 7.2 Hz, 3H).

^{13}C NMR (100 MHz, $CDCl_3$) δ 207.1, 162.3, 135.4, 131.3, 114.9, 76.0, 47.3, 35.8, 33.9, 33.2, 32.0, 31.3, 16.4, 12.4, 11.6.

HRMS (ESI) calcd for $[M+Na]^+$ $C_{15}H_{22}NaO_2$, m/z: 257.1512 , found: 257.1513.

IR v [cm^{-1}] 3078, 2980, 2935, 1634, 1456, 1384, 1270, 911.

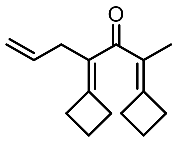

2k

Compound **2k.** The above adol product **52** (200 mg, 0.85 mmol, 1.0 equiv) was dissolved in toluene (4 mL) at rt, and burgess reagent (610 mg, 2.55 mmol, 3.0 equiv) was added. After the mixture was stirred at 60 ºC for 1 h, starting materials transformed completely (determined by TLC). Then, the mixture was cooled to room temperature and DBU (0.4mL, 2.55 mmol, 3.0 equiv) was added to the mixture and stirred at that temperature for 4 h. Upon completion, the reaction was quenched carefully with water and extracted with EtOAc (3 x 50 mL). The combined organic layer was washed with brine, dried over Na_2SO_4 and concentrated under vacuum. The residue was purified by flash column chromatography (EtOAc: petroleum ether = 1:30 to 1:12) to give dienone product **2k** (87 mg, 0.41 mmol, 47% yield) as a yellow oil.

^{1}H NMR (500 MHz, $CDCl_3$) δ 5.73 (ddt, J = 16.9, 10.0, 6.8 Hz, 1H), 5.01 – 4.91 (m, 2H), 2.92 (d, J = 7.0 Hz, 2H), 2.83 – 2.77 (m, 6H), 2.74 – 2.70 (m, 2H), 2.01 – 1.92 (m, 4H), 1.67 – 1.66 (m, 3H).

^{13}C NMR (125 MHz, $CDCl_3$) δ 198.1, 153.1, 150.7, 135.8, 131.9, 129.4, 115.2, 32.7, 32.4, 32.1, 31.1, 31.0, 16.7, 16.6, 13.0.

HRMS (ESI) calcd for $[M+Na]^+$ $C_{15}H_{20}NaO$, m/z: 239.1406 , found: 239.1405.

IR v [cm^{-1}] 3077, 2954, 1718, 1631, 1333, 1239, 911.

3.12 Preparation of starting material 2l

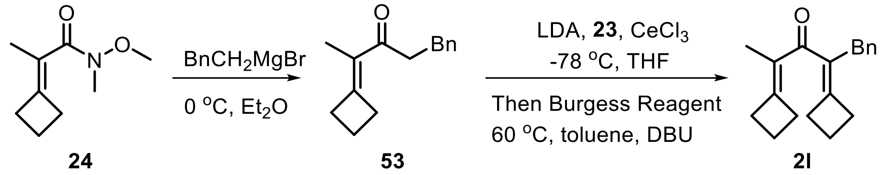

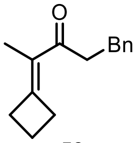

53

Compound **53.** To a stirred solution of weinreb amide **24** (338 mg, 2.0 mmol, 1.0 equiv) in Et_2O (15 mL) at 0 ºC was slowly added $BnCH_2MgBr$ (4.0 mL, 0.77 M in THF, 3.0 mmol, 1.5 equiv). The mixture was continued reaction for 2 h before the reaction was quenched carefully with saturated NH_4Cl aqueous solution and extracted with Et_2O (3 x 20 mL). The combined organic layer was washed with brine, dried over Na_2SO_4 and concentrated under vacuum. The residue was purified by flash column chromatography (Et_2O: petroleum ether = 1:15) to give enone product **53** (296 mg, 1.38 mmol, 69% yield) as a colorless oil.

^{1}H NMR (500 MHz, $CDCl_3$): δ 7.29 (t, J = 7.5 Hz, 2H), 7.23 – 7.18 (m, 3H), 3.12 – 3.09 (m, 2H), 2.95 (t, J = 7.5 Hz, 2H), 2.87 – 2.83 (m, 2H), 2.80 (t, J = 7.5 Hz, 2H), 2.09 – 2.03 (m, 2H), 1.72 – 1.66 (m, 3H).

^{13}C NMR (125 MHz, $CDCl_3$): δ 199.5, 157.9, 141.6, 129.1, 128.2, 128.2, 125.7, 42.9, 34.1, 31.6, 29.9, 16.3, 12.6.

HRMS (ESI) calcd for $[M+Na]^+$ $C_{15}H_{18}NaO$, m/z: 237.1250, found: 237.1262.

IR ν [cm^{-1}] 3062, 3026, 2921, 1657, 1452, 1363, 995.

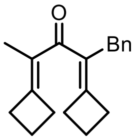

2l

Compound **2l.** To a stirred solution of di*iso*propylamine (1.7 mL, 12.0 mmol) in THF (8.5 mL) at 0 ºC was slowly added *n*-BuLi (4.8 mL, 2.5 M in hexane, 12.0 mmol) for 0.5h. Compound enone **53** (107 mg, 0.5 mmol, 1.0 equiv) was dissolved in THF (6 mL) at -78 °C, and the above freshly prepared LDA solution (1.9 mL, 1.5 mmol, 3.0 equiv) was added. After the mixture was stirred at the same temperature for 1 h, $CeCl_3$ (370 mg, 1.5 mmol, 3.0 equiv) in THF (5 mL) was added, and stirred for another 4 h. And then cyclobutanone **23** (0.11 mL, 1.5 mmol, 3.0 equiv) was added and the resultant solution was stirred at –78 °C for 0.5 h. The reaction was quenched carefully with

saturated NH_4Cl aqueous solution and extracted with EtOAc (3 x 50 mL). The combined organic layer was washed with brine, dried over Na_2SO_4 and concentrated under vacuum. The crude product was purified by a short flash column chromatography (EtOAc: petroleum ether = 1:20). The above product (94 mg, 0.33 mmol, 1.0 equiv) was dissolved in toluene (6 mL) at rt, and burgess reagent (236 mg, 1.0 mmol, 3.0 equiv) was added. After stirred at 60 ^oC for 0.5 h, starting materials transformed completely (determined by TLC). Then, the mixture was cooled to room temperature and DBU (0.15 mL, 1.0 mmol, 3.0 equiv) was added to the mixture and stirred at that temperature for 9 h. Upon completion, the reaction was quenched carefully with water and extracted with EtOAc (3 x 10 mL). The combined organic layer was washed with brine, dried over Na_2SO_4 and concentrated under vacuum. The residue was purified by flash column chromatography (EtOAc: petroleum ether = 1:50) to give dienone product **2l** (42 mg, 0.16 mmol, 32% yield for two steps) as a colorless oil.

Method for activating $CeCl_3$: anhydrous $CeCl_3$ was heated to 140 ºC in vacuo for 2 hours. After cooling the system to room temperature, THF was added and the mixture was stirred for 1.0 h.

^{1}H NMR (500 MHz, $CDCl_3$): δ 7.23 – 7.20 (m, 2H), 7.15 – 7.12 (m, 3H), 3.52 (s, 2H), 2.84 (q, *J* = 8.5 Hz, 4H), 2.65 (dd, *J* = 7.5 Hz, 2H), 2.45 (dd, *J* = 7.5, 7.5 Hz, 2H), 1.99 (p, *J* = 7.5 Hz, 2H), 1.84 (p, *J* = 7.5 Hz, 2H), 1.62 (s, 3H).

^{13}C NMR (125 MHz, $CDCl_3$): δ 198.1, 153.0, 150.6, 140.3, 133.6, 129.3, 128.8, 128.2, 125.8, 34.0, 32.5, 31.6, 31.4, 31.0, 16.6, 16.5, 13.0.

HRMS (ESI) calcd for $[M+Na]^+$ $C_{19}H_{22}NaO$, m/z: 289.1563, found: 289.1564.

IR ν [cm^{-1}] 3061, 3027, 2953, 2917, 1630, 1494, 1329, 978.

3.13 Preparation of starting material 2n

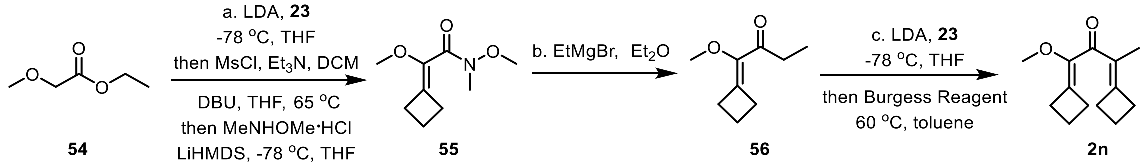

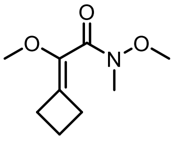

55

Compound **55.** To a stirred solution of di*iso*propylamine (4.5 mL, 32 mmol, 1.6 equiv) in THF (30 mL) at -78 ºC was slowly added *n*-BuLi (12.0 mL, 2.5 M in hexane, 30 mmol, 1.5 equiv). After the mixture was stirred at -78 ºC for 0.5 h, the solution of compound **54** (2.4 g, 20.0 mmol, 1.0 equiv) in THF (20 mL) was added to the flask. The mixture was stirred at the same temperature

for 1 h before cyclobutanone **23** (1.8 mL, 24 mmol, 1.2 equiv) was added to the reaction. After stirred at -78 ºC for another 0.5 h, the reaction was quenched with saturated NH_4Cl aqueous solution and extracted with EtOAc (3 x 30 mL). The combined organic layer was washed with brine, dried over Na_2SO_4 and concentrated under vacuum. The crude product was used for next step without purification. The above crude product was dissolved in DCM (90 mL) at 0 ºC, and Et_3N (33 mL, 60.0 mmol, 3.0 equiv), MsCl (7.7 mL, 100 mmol, 5.0 equiv) were added to the flask successively. After stirred at rt for 4 h, the reaction was quenched with saturated $NaHCO_3$ aqueous solution and extracted with DCM (3 x 60 mL). The combined organic layer was washed with brine, dried over Na_2SO_4 and concentrated under vacuum. The crude product was used for next step without purification. The above crude product was dissolved in THF (90 mL) at rt, and DBU (9.0 mL, 60.0 mmol, 3.0 equiv) was added to the mixture. After stirred at 65 ºC for 2 h, the reaction was quenched with H_2O (30 mL) and extracted with Et_2O (3 x 60 mL). The combined organic layer was washed with brine, dried over Na_2SO_4 and concentrated under vacuum. The crude product was purified by a short flash column chromatography (EtOAc: petroleum ether = 1:20). The above crude product was dissolved in THF (90 mL) at -78 ºC, and MeNHOMe·HCl (3.9 g, 40 mmol, 2.0 equiv) was added. After the mixture was stirred at -78 ºC for 0.5 h, LiHMDS (80mL, 1.0 M in THF, 80 mmol, 4.0 equiv) was added to the flask. The mixture was stirred at -78 ºC for 1 h before the mixture was quenched with saturated NH_4Cl aqueous solution and extracted with EtOAc (3 x 90 mL). The combined organic layer was washed with brine, dried over Na_2SO_4 and concentrated under vacuum. The residue was purified by flash column chromatography (EtOAc: petroleum ether = 1:4) to give weinreb amide product **55** (2.8 g, 15.3 mmol, 76% yield for 4 steps) as a colorless oil.

^{1}H NMR (400 MHz, $CDCl_3$): δ 3.59 (s, 3H), 3.49 (s, 3H), 3.10 (s, 3H), 2.79 – 2.75 (m, 2H), 2.72 (ddd, J = 8.0, 4.8, 1.6 Hz, 2H), 1.90 (p, J = 8.0, 2H).

^{13}C NMR (100 MHz, $CDCl_3$): δ 165.1, 140.7, 130.0, 60.9, 58.1, 33.7, 28.9, 28.4, 17.4.

HRMS (ESI) calcd for $[M+Na]^+$ $C_9H_{15}NNaO_3$, m/z: 208.0944, found: 208.0947.

IR v [cm⁻1] 2936, 1652, 1382, 1259, 1102, 968.

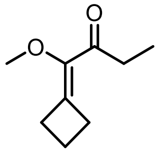

56

Compound **56.** To a stirred solution of weinreb amide **55** (1.0 g, 5.4 mmol, 1.0 equiv) in Et_2O (20 mL) at 0 ºC was slowly added EtMgBr (2.38 mL, 3.4 M in 2-Me-THF, 8.1 mmol, 1.5 equiv). After the mixture was stirred at the same temperature for 4 h, the reaction was quenched carefully with

saturated NH_4Cl aqueous solution and extracted with Et_2O (4 x 15 mL). The combined organic layer was washed with brine, dried over Na_2SO_4 and concentrated under vacuum. The residue was purified by flash column chromatography (Et_2O: petroleum ether = 1:10) to give enone product **56** (756 mg, 4.9 mmol, 91% yield) as a colorless oil.

^{1}H NMR (500 MHz, $CDCl_3$): δ 3.63 (s, 3H), 3.12 – 3.08 (m, 2H), 2.99 – 2.95 (m, 2H), 2.51 (q, J = 7.5 Hz, 2H), 2.09 (p, J = 7.5 Hz, 2H), 1.04 (t, J = 7.5 Hz, 3H).

^{13}C NMR (125 MHz, $CDCl_3$): δ 199.4, 146.3, 141.3, 59.1, 32.7, 32.3, 29.8, 18.2, 7.5.

HRMS (ESI) calcd for $[M+Na]^+$ $C_9H_{14}NaO_2$, m/z: 177.0886, found: 177.0886.

IR ν [cm^{-1}] 2967, 1705, 1647, 1508, 1377.

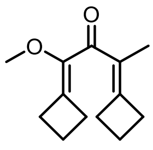

2n

Compound **2n.** To a stirred solution of di*iso*propylamine (1.2 mL, 8.5 mmol, 1.5 equiv) in THF (20 mL) at -78 ºC was slowly added *n*-BuLi (3.2 mL, 2.5 M in hexane, 7.9 mmol, 1.4 equiv). After the mixture was stirred at -78 ºC for 0.5 h, the solution of enone **56** (875 mg, 5.7 mmol, 1.0 equiv) in dry THF (10 mL) was added. After stirred at the same temperature for 1 h, cyclobutanone **23** (0.5 mL, 6.8 mmol, 1.2 equiv) was added to the flask. The reaction was quenched carefully with saturated NH_4Cl aqueous solution before the mixture was stirred for 0.5 h and extracted with EtOAc (3 x 10 mL). The combined organic layer was washed with brine, dried over Na_2SO_4 and concentrated under vacuum. The crude product was purified by a short flash column chromatography (EtOAc: petroleum ether = 1:20). The above crude product was dissolved in toluene (40 mL) at rt, and burgess reagent (4.1 g, 17.0 mmol, 3.0 equiv) was added. After stirred at 60 ºC for 0.5 h, the mixture was cooled to room temperature and the reaction mixture was quenched by water and extracted with EtOAc for three times. The organic phase was concentrated, dried over Na_2SO_4, and evaporated under reduced pressure to give crude product. The residue was purified by flash column chromatography (Et_2O: petroleum ether = 1:40 to 1:20) to give dienone product **2n** (819 mg, 4.0 mmol, 70% yield for 2 steps) as a colorless oil.

^{1}H NMR (400 MHz, $CDCl_3$): δ 3.57 (s, 3H), 2.94 – 2.85 (m, 6H), 2.78 – 2.73 (m, 2H), 2.06 – 1.95 (m, 4H), 1.70 – 1.68 (m, 3H).

^{13}C NMR (100 MHz, $CDCl_3$): δ 193.0, 154.1, 147.0, 135.9, 128.2, 58.6, 32.2, 31.2, 30.5, 29.3, 17.8, 16.6, 12.9.

HRMS (ESI) calcd for $[M+Na]^+$ $C_{13}H_{18}NaO_2$, m/z: 229.1199, found: 229.1197.

IR ν [cm^{-1}] 2953, 1737, 1714, 1439, 1368, 1251, 1119, 1037.

3.14 Preparation of starting material 2o

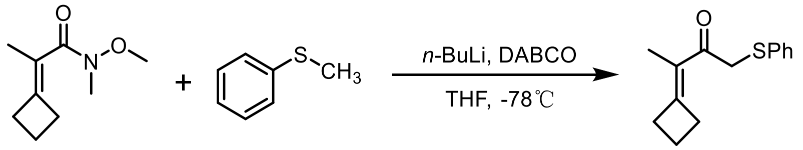

24 **57** **58**

Compound **58.** The thioanisole **57** (0.17 mL,1.5 mmol, 1.5 equiv) was added to a solution of DABCO (168 mg, 1.5 mmol, 1.5 equiv) at -78°C in THF (2 mL). Then *n*-BuLi (0.60 mL, 2.5 M in hexane,1.5 mmol, 1.5 equiv) was added dropwise and the mixture was stirred for 1 h at room temperature. After the mixture was cooled at -78°C, a solution of the weinreb amide **24** (169 mg ,1.0 mmol, 1.0 equiv) in THF (2 mL) was added and the mixture was stirred at -78 °C for 0.5 h. After quenched with aqueous NH_4Cl, the reaction mixture was allowed to reach rt. Extraction with Et_2O (2 × 30 mL), washing with brine (30 mL) and drying the organic phase over Na_2SO_4 afforded a crude product (previous concentration in vacuo) which was purified through flash column chromatography (EtOAc: petroleum ether = 1:15) to give the corresponding enone product **58** (215mg, 0.93mmol, 93%) as a yellow solid.

^{1}H NMR (400 MHz, $CDCl_3$) δ 7.36 – 7.18 (m, 5H), 3.86 (s, 2H), 3.09 (dd, J = 7.2, 7.2 Hz, 2H), 2.86 (dd, J = 6.8, 6.4 Hz, 2H), 2.11 – 2.01 (m, 2H), 1.70 (s, 3H).

^{13}C NMR (100 MHz, $CDCl_3$) δ 194.2, 159.9, 135.4, 129.7, 128.8, 128.2, 126.4, 43.4, 34.1, 31.8, 16.3, 13.0.

HRMS (ESI) calcd for $[M+Na]^+$ $C_{14}H_{16}NaOS$, m/z: 255.0814, found: 255.0817.

IR ν [cm^{-1}] 2956, 1647, 1437, 1398, 1372, 1270, 1217, 1088, 895, 736.

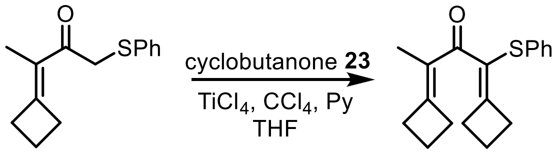

58 **2o**

Compound **2o.** To a stirred solution of in THF (3 mL) at 0°C, $TiCl_4$ (0.14 mL,1.30 mmol, 2.0 equiv) and CCl_4 (0.13 mL, 1.30 mmol, 2.0 equiv) were added. The yellow solution was left to stir for 0.5 hour and then enone **58** (150 mg, 0.65 mmol, 1.0 equiv) and cyclobutanone **23** (75 µL, 0.98 mmol, 1.5 equiv) was added dropwise to the solution at 0°C. The solution was left to stir for 1 hour at 0°C and then pyridine (0.22 mL, 2.6 mmol, 4.0 equiv) was added dropwise. The reaction mixture was allowed to warm to room temperature and stirred for 5 days. The reaction was then quenched with water (10 mL), the organic layer was separated and the aqueous layer was extracted with ether (3 × 30 mL). The combined organic layers were washed with saturated $NaHCO_3$ (10 mL) and brine (10 mL), dried over Na_2SO_4, filtered, concentrated under reduced pressure and purified by flash

chromatography on silica gel (EtOAc: petroleum ether = 1:15) to give the corresponding product **2o** (45 mg, 0.16 mmol, 25%) as a yellow solid and starting material enone **58** (100 mg, 0.43 mmol).
^{1}H NMR (500 MHz, $CDCl_3$) δ 7.29 – 7.27 (m, 2H), 7.24 – 7.21 (m, 2H), 7.15 – 7.12 (m, 1H), 2.95 – 2.91 (m, 2H), 2.88 – 2.80 (m, 4H), 2.72 (dd, J = 8.0, 7.5 Hz, 2H), 2.05 (p, J = 8.0 Hz, 2H), 1.93 (p, J = 8.0 Hz, 2H), 1.60 – 1.59 (m, 3H).
^{13}C NMR (125 MHz, $CDCl_3$) δ 194.3, 160.3, 155.4, 135.6, 129.2, 128.7, 128.2, 126.1, 125.4, 32.5, 32.5, 32.3, 31.2, 16.6, 16.5, 13.0.
HRMS (ESI) calcd for $[M+Na]^+$ $C_{18}H_{20}OS$, m/z: 307.1127, found: 307.1136.
IR ν [cm^{-1}] 2983, 2955, 2917, 1636, 1583, 1478, 1304, 1239, 740, 691.

3.15 Preparation of starting material 2p

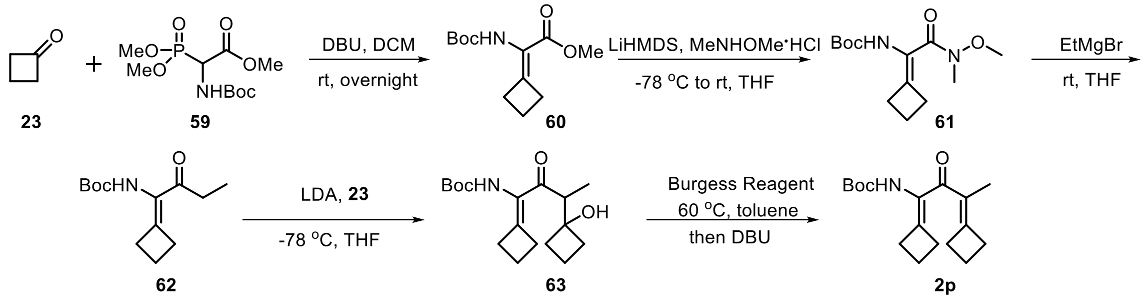

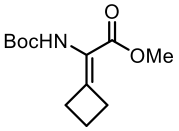

60

Compound **60.** A well-stirred solution of N-Boc-α-phosphonoglycine trimethyl ester **59** (13.2 g, 44.4 mmol, 1.1 equiv.) and DBU (6.1 mL, 42 mmol, 1.05 equiv.) in 125 mL DCM was treated with the cyclobutanone **23** (3.0 mL, 40 mmol, 1.0 equiv.). After stirred overnight at room temperature, the reaction mixture was was quenched with saturated NH_4Cl aqueous solution and extracted with EtOAc (3 x 100 mL). The combined organic phases were washed with brine, dried over anhydrous Na_2SO_4 and concentrated under reduced pressure. The residue was purified by flash column chromatography using EtOAc / petroleum ether (1/40 to 1/20) eluent to afford the desired ene ester product **60** (3.6 g, 14.9 mmol, 37% yield) as a white soild.
^{1}H NMR (500 MHz, $CDCl_3$) δ 5.88 (s, 1H), 3.72 – 3.71 (m, 3H), 3.10 (dd, J = 8.0, 7.5 Hz, 2H), 2.84 (dd, J = 7.5, 7.5 Hz, 2H), 2.03 (pd, J = 8.0, 1.5 Hz, 2H), 1.43 – 1.42 (m, 9H).
^{13}C NMR (125 MHz, $CDCl_3$) δ 165.2, 156.4, 153.0, 118.0, 80.0, 51.6, 32.5, 31.4, 28.2, 17.0.
HRMS (ESI) calcd for $[M+Na]^+$ $C_{12}H_{19}NNaO_4$, m/z: 264.1206, found: 264.1206.
IR ν [cm^{-1}] 2979, 1709, 1494, 1367, 1321, 1245, 1163, 670.

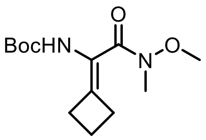

61

Compound **61.** The ene ester **60** (900 mg, 3.7 mmol, 1.0 equiv) was dissolved in THF (15 mL) at -78 ℃, and MeNHOMe·HCl (728 mg, 7.5 mmol, 2.0 equiv) was added. After the mixture was stirred at -78 ℃ for 0.5 h, LiHMDS (19 mL, 1.0 M in THF, 19 mmol, 5.0 equiv) was added to the flask. The temperature of the mixture was warmed to rt and stirring was continued for 0.5 h before the mixture was quenched with saturated NH_4Cl aqueous solution and extracted with EtOAc (3 x 70 mL). The combined organic layer was washed with brine, dried over Na_2SO_4 and concentrated under vacuum. The residue was purified by flash column chromatography (EtOAc: petroleum ether = 1: 1) to give weinreb amide product **61** (833 mg, 3.1 mmol, 83% yield) as a white soild.
^{1}H NMR (400 MHz, $CDCl_3$) δ 6.04 (s, 1H), 3.65 (s, 3H), 3.21 (s, 3H), 2.76 (dd, *J* = 6.8, 6.4 Hz, 2H), 2.67 (dd, *J* = 7.2, 6.8 Hz, 2H), 1.97 – 1.93 (m, 2H), 1.37 (s, 9H).
^{13}C NMR (101 MHz, $CDCl_3$) δ 165.4, 152.7, 140.4, 120.6, 79.8, 61.0, 34.1, 29.6, 29.3, 28.1, 16.7.
HRMS (ESI) calcd for $[M+Na]^+$ $C_{13}H_{22}N_2NaO_4$, m/z: 293.1472, found: 293.1472.
IR ν [cm^{-1}] 2978, 2933, 2820, 1712, 1645, 1501, 1366, 1250, 1168, 778.

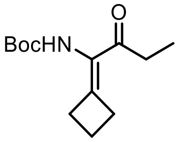

62

Compound **62.** To a stirred solution of weinreb amide **61** (820 mg, 3.0 mmol, 1.0 equiv) in THF (12 mL) at 0 ºC was slowly added EtMgBr (4.5 mL, 2.0 M in THF, 9.0 mmol, 3.0 equiv). After the temperature of the mixture was warmed to rt and stirring was continued for 6 h, the reaction was quenched carefully with saturated NH_4Cl aqueous solution and extracted with EtOAc (4 x 50 mL). The combined organic layer was washed with brine, dried over Na_2SO_4 and concentrated under vacuum. The residue was purified by flash column chromatography (EtOAc: petroleum ether = 1:8) to give enone product **62** (575 mg, 2.4 mmol, 79% yield) as a white soild.
^{1}H NMR (500 MHz, $CDCl_3$) δ 6.21 (s, 1H), 3.15 (dd, *J* = 7.5, 7.5 Hz, 2H), 2.91 (dd, *J* = 8.0, 7.5 Hz, 2H), 2.48 (q, *J* = 7.5 Hz, 2H), 2.08 (p, *J* = 8.0 Hz, 2H), 1.42 (s, 9H), 1.06 (t, *J* = 7.0 Hz, 3H).
^{13}C NMR (125 MHz, $CDCl_3$) δ 197.5, 153.8, 153.0, 127.5, 79.9, 33.5, 33.1, 31.9, 28.2, 17.0, 7.7.
HRMS (ESI) calcd for $[M+Na]^+$ $C_{13}H_{21}NNaO_3$, m/z: 262.1414, found: 262.1410.
IR ν [cm^{-}1] 2978, 2935, 1693, 1647, 1489, 1366, 1250, 1166, 670.

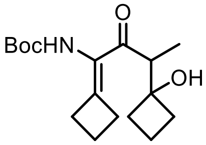

63

Compound **63.** To a stirred solution of di*iso*propylamine (1.4 mL, 10.0 mmol in THF (4.6 mL) at 0 ºC was slowly added *n*-BuLi (4.0 mL, 2.5 M in hexane, 10.0 mmol) for 0.5 h. Compound enone **62** (575 mg, 2.4 mmol, 1.0 equiv) was dissolved in THF (10 mL) at -78 ℃, and the above freshly prepared LDA solution (9.6 mL, 9.6 mmol, 4.0 equiv) was added. After the mixture was stirred at the same temperature for 1 h, cyclobutanone **23** (0.54 mL, 7.2 mmol, 3.0 equiv) was added and the resultant solution was stirred at –78 °C for 0.5 h. The reaction was quenched carefully with saturated NH_4Cl aqueous solution and extracted with EtOAc (3 x 50 mL). The combined organic layer was washed with brine, dried over Na_2SO_4 and concentrated under vacuum. The residue was purified by flash column chromatography (EtOAc: petroleum ether = 1:12 to 1:6) to give adol product **63** (360 mg, 1.2 mmol, 48% yield) as a white soild and starting material **62** (171 mg, 0.7 mmol).

^{1}H NMR (400 MHz, $CDCl_3$) δ 6.04 (s, 1H), 4.05 (s, 1H), 3.20 – 3.11 (m, 3H), 2.89 (t, *J* = 6.8 Hz, 2H), 2.14 – 1.90 (m, 6H), 1.83 – 1.75 (m, 1H), 1.58 – 1.49 (m, 1H), 1.43 (s, 9H), 1.11 (d, *J* = 7.2 Hz, 3H).

^{13}C NMR (100 MHz, $CDCl_3$) δ 203.8, 157.5, 153.4, 127.0, 80.3, 76.1, 47.0, 35.6, 33.1, 32.7, 31.7, 28.1, 16.9, 12.5, 11.4.

HRMS (ESI) calcd for $[M+Na]^+$ $C_{17}H_{27}NNaO_4$, m/z: 332.1832, found: 332.1827.

IR *v* [cm^{-1}] 3437, 2979, 2937, 1705, 1631, 1491, 1368, 1250, 1167, 954.

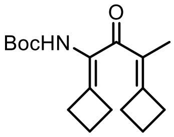

2p

Compound **2p.** The above adol product **63** (340 mg, 1.1 mmol, 1.0 equiv) was dissolved in toluene (5 mL) at rt, and burgess reagent (786 mg, 3.3 mmol, 3.0 equiv) was added. After stirred at 60 ºC for 1 h, starting materials transformed completely (determined by TLC). Then, the mixture was cooled to room temperature and DBU (0.5 mL, 3.3 mmol, 3.0 equiv) was added to the mixture and stirred at that temperature for 4 h. Upon completion, the reaction was quenched carefully with water and extracted with EtOAc (3 x 50 mL). The combined organic layer was washed with brine, dried over Na_2SO_4 and concentrated under vacuum. The residue was purified by flash column chromatography (EtOAc: petroleum ether = 1:30 to 1:12) to give dienone product **2p** (125 mg, 0.44 mmol, 40% yield) as a white soild.

^{1}H NMR (500 MHz, $CDCl_3$) δ 6.18 (s, 1H), 2.86 (dd, J = 7.0, 7.0 Hz, 2H), 2.81 (dd, J = 6.5, 6.5 Hz, 2H), 2.72 (dd, J = 8.0, 7.5 Hz, 2H), 2.68 (dd, J = 8.0, 7.5 Hz, 2H), 1.99 – 1.89 (m, 4H), 1.64 – 1.64 (m, 3H), 1.37 (s, 9H).

^{13}C NMR (125 MHz, $CDCl_3$) δ 192.4, 152.7, 145.9, 128.0, 127.5, 79.9, 32.3, 31.1, 28.1, 16.9, 16.5, 12.8.

HRMS (ESI) calcd for $[M+Na]^+$ $C_{17}H_{25}NNaO_3$, m/z: 314.1727 , found: 314.1723.

IR v [cm^{-1}] 2976, 1703, 1637, 1485, 1366, 1247, 1165, 776.

3.16 Preparation of starting materials 2m and 2q

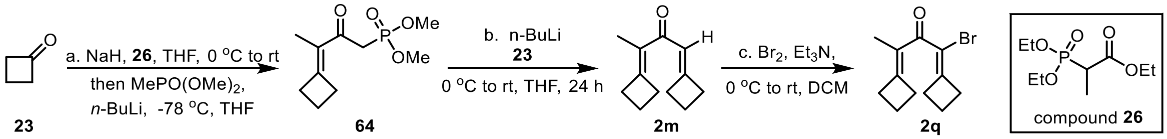

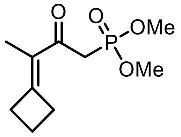

64

Compound **64.** To a stirred solution of NaH (416 mg, 60% in oil, 10.4 mmol, 1.3 equiv) in THF (130 mL) at 0 ºC was slowly added compound **26** (2.2 mL, 10.4 mmol, 1.3 equiv). After the mixture was stirred at rt for 1 h, cyclobutanone **23** (0.6 mL, 8.0 mmol, 1.0 equiv) was added to the flask. The mixture was stirred at the same temperature for 4 h before the reaction was quenched carefully with saturated NH_4Cl aqueous solution and extracted with Et_2O (2 x 80 mL). The combined organic layer was washed with brine, dried over Na_2SO_4 and concentrated under vacuum. The crude product ene ester was used for next step without purification. To a stirred solution of $MePO(OMe)_2$ (1.7 mL, 16 mmol, 2.0 equiv) in THF (60 mL) at -78 ºC was slowly added *n*-BuLi (7.1 mL, 2.5 M in hexane, 17.6 mmol, 2.2 equiv) to the mixture. The reaction was stirred at -78 ºC for 1 h. The crude product ene ester was dissolved in THF (10 mL), and added to the above solution at -78 ºC. After stirred at -78 ºC for 0.5 h, the mixture was quenched with saturated NH_4Cl aqueous solution and extracted with EtOAc (3 x 30 mL). The combined organic layer was washed with brine, dried over Na_2SO_4 and concentrated under vacuum. The residue was purified by flash column chromatography (EtOAc: petroleum ether = 1:10 to 100:0) to give phospholipids product **64** (1.5 g, 6.5 mmol, 81% yield for 2 steps) as a colorless oil.

^{1}H NMR (500 MHz, $CDCl_3$): δ 3.76 (s, 3H), 3.73 (s, 3H), 3.18 – 3.14 (m, 4H), 2.85 – 2.81 (m, 2H), 2.07 – 2.01 (m, 2H), 1.66 (s, 3H).

^{13}C NMR (125 MHz, $CDCl_3$): δ 191.6 (d, J = 7.5 Hz), 161.5, 129.2 (d, J = 3.8 Hz), 52.9, 52.8,

38.8 (d, J = 132.5 Hz), 34.0, 32.0, 16.2, 12.9.
^{31}P NMR (202 MHz, $CDCl_3$) δ 24.10.
HRMS (ESI) calcd for $[M+Na]^+$ $C_{10}H_{17}NaO_4P$, m/z: 255.0757, found: 255.0758.
IR ν [cm^{-1}] 2958, 2922, 2854, 1648, 1316, 1251,1012, 870, 839, 801.

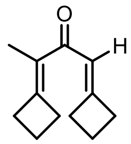

2m

Compound **2m.** To a stirred solution of **64** (770 mg, 3.3 mmol, 1.0 equiv) in THF (3.0 mL) at 0 ºC was slowly added NaH (146 mg, 60% in oil, 3.7 mmol, 1.1 equiv). After the mixture was stirred for 0.5 h, cyclobutanone **23** (323 µL, 4.3 mmol, 1.3 equiv) was added to the flask. After stirred at rt for 12 h, the reaction was quenched with saturated NH_4Cl aqueous solution and extracted with Et_2O (3 x 20 mL). The combined organic layer was washed with brine, dried over Na_2SO_4 and concentrated under vacuum. The residue was purified by flash column chromatography (EtOAc : petroleum ether = 1:20) to give dienone product **2m** (141 mg, 0.8 mmol, 24% yield) as a colorless oil.
^{1}H NMR (500 MHz, $CDCl_3$): δ 6.17 – 6.16 (m, 1H), 3.18 – 3.15 (m, 2H), 3.09 – 3.06 (m, 2H), 2.85 – 2.80 (m, 4H), 2.14 – 2.08 (m, 2H), 2.06 – 1.99 (m, 2H), 1.68 (s, 3H).
^{13}C NMR (125 MHz, $CDCl_3$): δ 191.8, 165.9, 155.9, 130.0, 118.9, 34.7, 33. 9, 32.9, 31.7, 18.5, 16.6, 12.9.
HRMS (ESI) calcd for $[M+Na]^+$ $C_{12}H_{16}NaO$, m/z: 199.1093, found: 199.1093.
IR ν [cm^{-1}] 3451, 2955, 2854, 1670, 1656, 1615,1355, 1280, 1119.

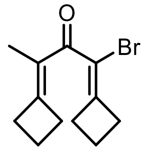

2q

Compound **2q.** To a stirred solution of compound **2m** (176 mg, 1.0 mmol, 1.0 equiv) in DCM (10 mL) at 0 ºC was slowly added Br_2 (50 µL, 1.0 mmol, 1.0 equiv). After the mixture was stirred for 0.5 h, Et_3N (170 µL, 1.2 mmol, 1.2 equiv) was added. The solvent was removed by decompression after 24 hours, and then the crude product was purified by flash column chromatography (EtOAc: petroleum ether = 1:40) to give dienone product **2q** (62 mg, 0.24 mmol, 24% yield) as a white solid.
^{1}H NMR (500 MHz, $CDCl_3$): δ 2.88 – 2.77 (m, 8H), 2.04 – 1.99 (m, 4H), 1.71 (s, 3H).
^{13}C NMR (125 MHz, $CDCl_3$): δ 190.5, 155.5, 154.6, 127.6, 113.7, 33.8, 32.6, 32.2, 31.4, 16.5,

15.8, 13.1.

HRMS (ESI) calcd for $[M+Na]^+$ $C_{12}H_{15}BrNaO$, m/z: 277.0198, found: 1277.0193.

IR *v* [cm^{-1}] 2956, 2926, 2854, 1648, 1612, 1445, 1310, 1098.

3.17 Preparation of starting material 2r

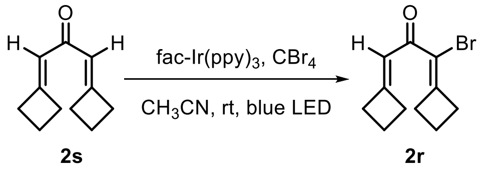

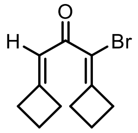

2r

Compound **2r.** To a stirred solution of compound **2s** (16.2 mg, 0.1 mmol, 1.0 equiv) in CH_3CN (1 mL) at rt was added CBr_4 (199.0 mg, 0.6 mmol, 6.0 equiv), $Ir(ppy)_3$ (3.3 mg, 0.005 mmol, 0.05 equiv). The reaction mixture was stirred at room temperature and irradiated by blue LEDs (10 W, 425 nm). After 24 h the reaction was completed (monitored by TLC), the solvent was concentrated in vacuo. The residue was purified by flash column chromatography on silica gel (EtOAc: petroleum ether = 1:40) to give bromo-dienone product **2r** (9.6 mg, 0.04 mmol, 40% yield) as a yellow oil.

^{1}H NMR (500 MHz, $CDCl_3$): δ 6.54 – 6.52 (m, 1H), 3.20 – 3.17 (m, 2H), 3.12 – 3.08 (m, 2H), 2.90 – 2.87 (m, 2H), 2.84 – 2.81 (m, 2H), 2.14 (p, J = 7.5 Hz, 2H), 2.08 (p, J = 8.0 Hz, 2H).

^{13}C NMR (125 MHz, $CDCl_3$): δ 184.4, 170.6, 161.2, 116.8, 116.0, 34.9, 34.9, 34.4, 33.2, 18.4, 16.2.

HRMS (ESI) calcd for $[M+Na]^+$ $C_{11}H_{13}BrNaO$, m/z: 263.0042, found: 263.0042.

IR *v* [cm^{-1}] 2986, 2938, 1716, 1650, 1604, 1357, 1295, 1247, 1182.

3.18 Preparation of starting material 2s

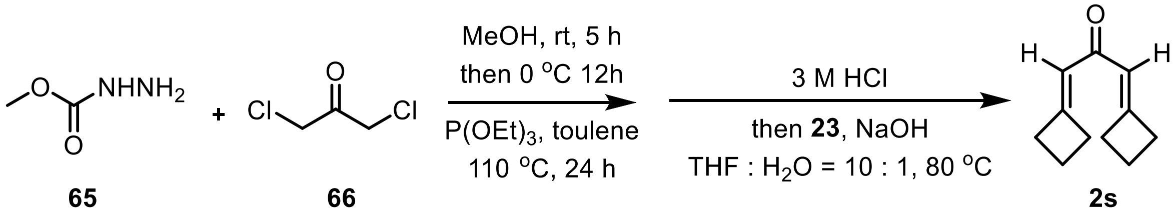

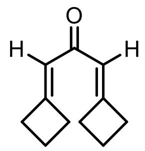

2s

Compound **2s.** To a stirred solution of methyl carbazate **65** (4.9 g, 54 mmol, 1.0 equiv) in MeOH (100 mL) at rt was slowly added 1,3-dichloroacetone **66** (7.0 g, 54 mmol, 1.0 equiv). After stirred at rt for 5 h, the solution was leaved in the fridge overnight, and concentrated under vacuum, filtered and washed with petroleum ether. The crude product was dissolved in toluene (100 mL), and $P(OEt)_3$ (14.2 mL, 119 mmol, 2.2 equiv) was added to the flask dropwise. After refluxed at 110 ºC for 24 h, the reaction mixture was cooled to rt, and extracted with EtOAc (3 x 50 mL). The combined organic layer was washed with brine, and concentrated under vacuum. The crude product was used for next step without purification.

The crude product was dissolved in acetone (20 mL) and added 3 M HCl aqueous solution (36 mL, 108 mmol, 2.0 equiv). After stirred at rt for 5 h, the mixture was extracted with $CHCl_3$ (3 x 30 mL). The combined organic layer was washed with brine, dried over Na_2SO_4 and concentrated under vacuum. The crude product was used for next step without purification. The crude product was dissolved in THF (100 mL) and H_2O (10 mL), NaOH (2.0 g, 50 mmol, 4.0 equiv), and cyclobutanone **23** (1.9 mL, 25 mmol, 2.0 equiv) were added to the mixture. After refluxed at 80 ºC for 24 h, the reaction mixture was cooled to rt, and extracted with EtOAc (3 x 50 mL), then dried over Na_2SO_4 and concentrated under vacuum, The residue was purified by flash column chromatography (EtOAc: petroleum ether = 1:30) to give dienone product **2s** (320 mg, 2.0 mmol, 4% yield for 4 steps) as a white solid.

^{1}H NMR (500 MHz, $CDCl_3$): δ 5.93-5.92 (m, 2H), 3.14 (dd, J = 7.5, 7.5 Hz, 4H), 2.81 (dd, J = 7.5, 7.5 Hz, 4H), 2.11 – 2.05 (m, 4H).

^{13}C NMR (125 MHz, $CDCl_3$): δ 189.8, 166.1, 121.3, 34.5, 32.8, 18.2.

HRMS (ESI) calcd for $[M+Na]^+$ $C_{11}H_{14}NaO$, m/z: 185.0937, found: 185.0937.

IR ν [cm^{-1}] 2985, 2917, 1681, 1594, 1360.

3.19 Preparation of starting material 2t

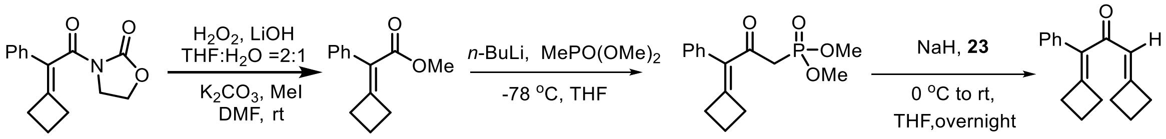

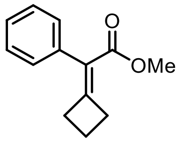

68

Compound **68.** Alkylidenecyclobutanes[1] **67** (2.6 g, 10 mmol, 1.0 equiv) was dissolved in THF (40 mL) and H_2O (20 mL) at 0 °C and then 30 wt. % H_2O_2 in H_2O (3 mL, 30 mmol, 3.0 equiv) was added, followed by LiOH (720 mg, 30 mmol, 3.0 equiv). After 1 h, peroxides were quenched with saturated $Na_2S_2O_3$ solution. 2-Oxazolidinone was removed from the aqueous layer by continuous extraction with DCM overnight. The aqueous layer was then acidified to pH = 1 with 1M HCl and extracted with EtOAc (5 x 50 mL). The combined organic layers were washed with brine (50 mL), dried over Na_2SO_4, filtered and concentrated under reduced pressure as a white powder that was used directly in the next step.

The crude product acid was dissolved in DMF (25 mL) at rt and then K_2CO_3 (4.1 g, 30 mmol, 3.0 equiv) and MeI (1.9 mL, 30 mmol, 3.0 equiv) were added successively. After stirring for 2 h at rt, the reaction mixture was quenched with water. The aqueous layer was extracted with EtOAc (3 x 70 mL). The combined organic layer was washed with water and brine, dried over Na_2SO_4 and concentrated under vacuum. the resulting crude residue was purified by flash column chromatography on silica gel (EtOAc/hexane = 1/30 to 1/20) to provide ene ester **68** (1.5 g, 7.4 mmol, 74%) as a yellow oil.

^{1}H NMR (400 MHz, $CDCl_3$) δ 7.31 – 7.19 (m, 5H), 3.67 (s, 3H), 3.23 (dd, *J* = 7.6, 7.2 Hz, 2H), 2.74 (dd, *J* = 7.6, 7.2 Hz, 2H), 2.01 (p, *J* = 7.6 Hz, 2H).

^{13}C NMR (100 MHz, $CDCl_3$) δ 166.8, 162.8, 135.4, 129.0, 127.6, 126.7, 125.4, 51.0, 34.1, 32.2, 16.9.

HRMS (ESI) calcd for $[M+Na]^+$ $C_{13}H_{14}NaO_2$, m/z: 225.0886 , found: 225.0888.

IR *v* [cm^{-1}] 2951, 1713, 1657, 1434, 1235, 1212, 1037, 791, 698.

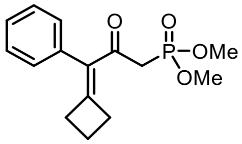

69

Compound **69.** To a stirred solution of $MePO(OMe)_2$ (1.7 mL, 16 mmol, 2.0 equiv) in THF (60 mL) at -78 ºC was slowly added *n*-BuLi (7.1 mL, 2.5 M in hexane, 17.6 mmol, 2.2 equiv). The reaction was stirred at -78 ºC for 1 h. A solution of ene ester **68** (1.6 g 8 mmol, 1.0 equiv) in THF (15 mL) was then added, using another aliquot of THF (5 mL) to complete the transfer. After slowly warmed to 23 °C and stirred for 1 h, the mixture was quenched with saturated NH_4Cl aqueous solution and extracted with EtOAc (3 x 80 mL). The combined organic layer was washed

with brine, dried over Na_2SO_4 and concentrated under vacuum. The residue was purified by flash column chromatography (EtOAc: petroleum ether = 1:12 to 100:0) to give starting material (0.4 g, 2.0 mmol) and phospholipids product **69** (0.9 g, 3.8 mmol, 40%, 55% brsm) as a yellow oil.

^{1}H NMR (500 MHz, $CDCl_3$) δ 7.37 (t, J = 7.5 Hz, 2H), 7.31 – 7.28 (m, 1H), 7.19 – 7.17 (m, 2H), 3.74 (s, 3H), 3.72 (s, 3H), 3.31 – 3.28 (m, 2H), 3.10 (d, J = 21.5 Hz, 2H), 2.72 – 2.68 (m, 2H), 2.13 – 2.04 (m, 2H).

^{13}C NMR (125 MHz, $CDCl_3$) δ 191.6 (d, J = 7.5 Hz), 164.6, 136.2, 134.2 (d, J = 5.0 Hz), 129.3, 128.5, 127.4, 52.8, 52.7, 38.8 (d, J = 132.5 Hz), 34.6, 32.5, 17.2.

^{31}P NMR (202 MHz, $CDCl_3$) δ 24.11.

HRMS (ESI) calcd for $[M+Na]^+$ $C_{15}H_{19}NaO_4P$, m/z: 317.0913 , found: 317.0913.

IR v [cm^{-1}] 2955, 2852, 1683, 1621, 1445, 1258, 1031, 801, 704.

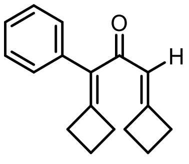

2t

Compound **2t.** To a stirred solution of **69** (294 mg, 1.0 mmol, 1.0 equiv) in THF (4.0 mL) at 0 ºC was slowly added NaH (44 mg, 60% in oil, 1.1 mmol, 1.1 equiv). After the mixture was stirred for 0.5 h, cyclobutanone **23** (97 µL, 1.3 mmol, 1.3 equiv) was added. The resulting mixture was warmed to rt and stirred for 12 h before it was quenched with saturated NH_4Cl aqueous solution and extracted with Et_2O (3 x 20 mL). The combined organic layer was washed with brine, dried over Na_2SO_4 and concentrated under vacuum. The residue was purified by flash column chromatography (EtOAc: petroleum ether = 1:20) to give dienone product **2t** (64 mg, 0.27 mmol, 27%) as a colorless oil.

^{1}H NMR (500 MHz, $CDCl_3$) δ 7.37 – 7.14 (m, 5H), 5.90 (s, 1H), 3.23 (dd, J = 7.5, 7.5 Hz, 2H), 3.18 (t, J = 8.0, 7.0 Hz, 2H), 2.75 (dd, J = 7.5, 7.0 Hz, 4H), 2.10 – 2.06 (m, 4H).

^{13}C NMR (125 MHz, $CDCl_3$) δ 191.3, 167.8, 158.6, 136.9, 135.1, 129.1, 128.3, 126.8, 119.7, 34.8, 34.1, 33.0, 32.5, 18.4, 17.6.

HRMS (ESI) calcd for $[M+Na]^+$ $C_{17}H_{18}NaO$, m/z : 261.1250, found : 261.1248.

IR v [cm^{-1}] 2983, 2955, 1712, 1682, 1627, 1599, 1349, 1163, 762, 699.

3.20 Preparation of starting material 2u

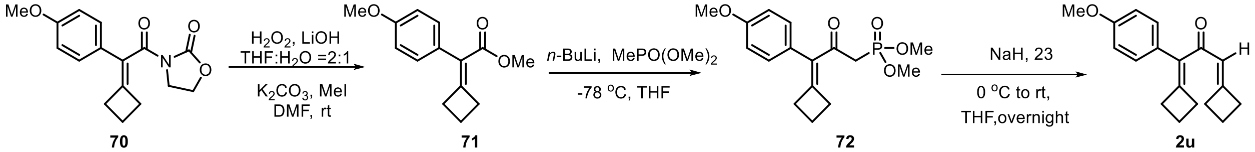

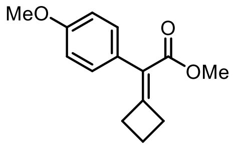

71

Compound **71.** Alkylidenecyclobutanes[1] **70** (2.9 g, 10 mmol, 1.0 equiv) was dissolved in THF (40 mL) and H_2O (20 mL) at 0 °C and then 30 wt. % H_2O_2 in H_2O (3 mL, 30 mmol, 3.0 equiv) was added, followed by LiOH (720 mg, 30 mmol, 3.0 equiv). After 1 h, peroxides were quenched with saturated $Na_2S_2O_3$ solution. 2-Oxazolidinone was removed from the aqueous layer by continuous extraction with DCM overnight. The aqueous layer was then acidified to pH = 1 with 1M HCl and extracted with EtOAc (5 x 50 mL). The combined organic layers were washed with brine (50 mL), dried over Na_2SO_4, filtered and concentrated under reduced pressure as a white powder that was used directly in the next step.

The crude product acid was dissolved in DMF (25 mL) at rt and then K_2CO_3 (4.1 g, 30 mmol, 3.0 equiv) and MeI (1.9 mL, 30 mmol, 3.0 equiv) were added successively. After stirring for 2 h at rt, the reaction mixture was quenched with water. The aqueous layer was extracted with EtOAc (3 x 70 mL). The combined organic layer was washed with water and brine, dried over Na_2SO_4 and concentrated under vacuum. The resulting crude residue was purified by flash column chromatography on silica gel (EtOAc : hexane = 1 : 30 to 1 : 20) to provide ene ester **71** (1.9 g, 8.1 mmol, 81%) as a yellow oil.

^{1}H NMR (400 MHz, $CDCl_3$) δ 7.16 (d, J = 8.4 Hz, 2H), 6.87 (d, J = 8.8 Hz, 2H), 3.79 (s, 3H), 3.72 (s, 3H), 3.24 (dd, J = 7.6, 7.6 Hz, 2H), 2.78 (dd, J = 7.6, 7.6 Hz, 2H), 2.09 – 2.01 (m, 2H).

^{13}C NMR (100 MHz, $CDCl_3$) δ 167.2, 162.2, 158.3, 130.2, 127.8, 125.0, 113.2, 55.0, 51.1, 34.2, 32.3, 17.0.

HRMS (ESI) calcd for $[M+Na]^+$ $C_{14}H_{16}NaO_3$, m/z: 255.0992 , found: 255.1006.

IR ν [cm^{-1}] 2988, 2952, 2837, 1712, 1610, 1512, 1248, 1212, 1035, 833.

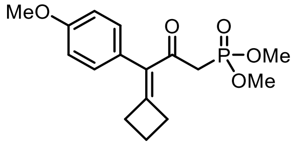

72

Compound **72.** To a stirred solution of $MePO(OMe)_2$ (1.7 mL, 16 mmol, 2.0 equiv) in THF (60 mL) at -78 ºC was slowly added *n*-BuLi (7.1 mL, 2.5 M in hexane, 17.6 mmol, 2.2 equiv). The reaction was stirred at -78 ºC for 1 h. A solution of ene ester **71** (1.9 g 8 mmol, 1.0 equiv) in THF (15 mL) was then added, using another aliquot of THF (5 mL) to complete the transfer. After slowly warmed to 23 °C and stirred for 1 h, the mixture was quenched with saturated NH_4Cl aqueous solution and extracted with EtOAc (3 x 80 mL). The combined organic layer was washed

with brine, dried over Na_2SO_4 and concentrated under vacuum. The residue was purified by flash column chromatography (EtOAc : petroleum ether = 1:12 to 100:0) to give starting material (650 mg, 2.8 mmol) and phospholipids product **72** (710 mg, 2.2 mmol, 27%, 42% brsm) as a yellow oil.

^{1}H NMR (500 MHz, $CDCl_3$) δ 7.08 (d, J = 8.7 Hz, 2H), 6.89 (d, J = 8.7 Hz, 2H), 3.80 (s, 3H), 3.74 (s, 3H), 3.71 (s, 3H), 3.28 – 3.24 (m, 2H), 3.08 (d, J = 21.5 Hz, 2H), 2.70 – 2.67 (m, 2H), 2.07 (p, J = 8.0 Hz, 2H).

^{13}C NMR (125 MHz, $CDCl_3$) δ 192.0 (d, J = 6.3 Hz), 164.2, 158.8, 134.0 (d, J = 5.0 Hz), 130.5, 128.4, 114.0, 55.2, 52.8, 52.8, 38.8 (d, J = 132.5 Hz), 34.6, 32.5, 17.2.

^{31}P NMR (202 MHz, $CDCl_3$) δ 24.25.

HRMS (ESI) calcd for $[M+Na]^+$ $C_{16}H_{21}NaO_5P$, m/z: 347.1019 , found: 347.1020.

IR v [cm^{-1}] 2956, 2851, 1681, 1609, 1511, 1248, 1031, 832.

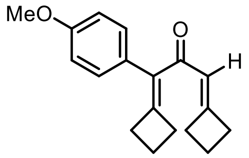

2u

Compound **2u.** To a stirred solution of **72** (324 mg, 1.0 mmol, 1.0 equiv) in THF (4.0 mL) at 0 ºC was slowly added NaH (44 mg, 60% in oil, 1.1 mmol, 1.1 equiv). After the mixture was stirred for 0.5 h, cyclobutanone **23** (97 µL, 1.3 mmol, 1.3 equiv) was added. The resulting mixture was warmed to rt and stirred for 12 h before it was quenched with saturated NH_4Cl aqueous solution and extracted with Et_2O (3 x 20 mL). The combined organic layer was washed with brine, dried over Na_2SO_4 and concentrated under vacuum. The residue was purified by flash column chromatography (EtOAc : petroleum ether = 1: 20) to give dienone product **2u** (70 mg, 0.26 mmol, 26%) as a colorless oil.

^{1}H NMR (400 MHz, $CDCl_3$) δ 7.08 (d, J = 8.8 Hz, 2H), 6.86 (d, J = 8.8 Hz, 2H), 5.92 – 5.90 (m, 1H), 3.78 (s, 3H), 3.21 – 3.14 (m, 4H), 2.75 – 2.71 (m, 4H), 2.10 – 2.00 (m, 4H).

^{13}C NMR (100 MHz, $CDCl_3$) δ 191.6, 167.3, 158.3, 157.3, 134.5, 130.0, 129.1, 119.7, 113.6, 55.1, 34.6, 33.9, 32.8, 32.4, 18.3, 17.5.

HRMS (ESI) calcd for $[M+Na]^+$ $C_{18}H_{20}NaO_2$, m/z: 291.1356 , found: 291.1355.

IR v [cm^{-1}] 2955, 2837, 1643, 1609, 1511, 1247, 1165, 1034, 832.

3.21 Preparation of starting material 2v

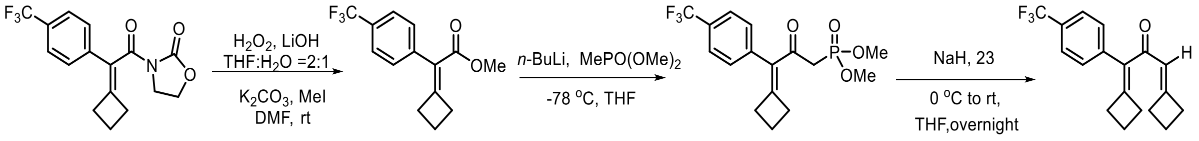

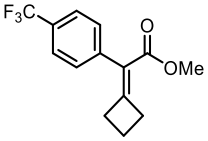

74

Compound **74.** Alkylidenecyclobutanes[1] **73** (3.3 g, 10 mmol, 1.0 equiv) was dissolved in THF (40 mL) and H_2O (20 mL) at 0 °C and then 30 wt. % H_2O_2 in H_2O (3 mL, 30 mmol, 3.0 equiv) was added, followed by LiOH (720 mg, 30 mmol, 3.0 equiv). After 1 h, peroxides were quenched with saturated $Na_2S_2O_3$ solution. 2-Oxazolidinone was removed from the aqueous layer by continuous extraction with DCM overnight. The aqueous layer was then acidified to pH = 1with 1M HCl and extracted with EtOAc (5 x 50 mL). The combined organic layers were washed with brine (50 mL), dried over Na_2SO_4, filtered and concentrated under reduced pressure as a white powder that was used directly in the next step.

The crude product acid was dissolved in DMF (25 mL) at rt and then K_2CO_3 (4.1 g, 30 mmol, 3.0 equiv) and MeI (1.9 mL, 30 mmol, 3.0 equiv) were added successively. After stirring for 2 h at rt, the reaction mixture was quenched with water. The aqueous layer was extracted with EtOAc (3 x 70 mL). The combined organic layer was washed with water and brine, dried over Na_2SO_4 and concentrated under vacuum. The resulting crude residue was purified by flash column chromatography on silica gel (EtOAc : hexane = 1 : 30 to 1 : 20) to provide ene ester **74** (1.3 g, 4.7 mmol, 47%) as a yellow oil.

^{1}H NMR (500 MHz, $CDCl_3$) δ 7.58 (d, J = 8.0 Hz, 2H), 7.34 (d, J = 8.0 Hz, 2H), 3.73 (s, 3H), 3.30 – 3.26 (m, 2H), 2.79 – 2.75 (m, 2H), 2.08 (p, J = 8.0 Hz, 2H).

^{13}C NMR (125 MHz, $CDCl_3$) δ 166.5, 164.9, 139.4, 129.6, 129.0 (q, J = 32.5 Hz), 124.9 (q, J = 3.8 Hz), 124.7, 124.2 (q, J = 270.0 Hz), 51.4, 34.5, 32.5, 17.1.

^{19}F NMR (471 MHz, $CDCl_3$) δ -62.55.

HRMS (ESI) calcd for $[M+Na]^+$ $C_{14}H_{13}F_3NaO_2$, m/z: 293.0760 , found: 293.0758.

IR ν [cm^{-1}] 2954, 1715, 1616, 1326, 1214, 1123, 1068, 844.

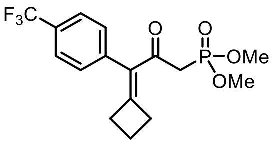

75

Compound **75.** To a stirred solution of $MePO(OMe)_2$ (1.7 mL, 16 mmol, 2.0 equiv) in THF (60 mL) at -78 ºC was slowly added *n*-BuLi (7.1 mL, 2.5 M in hexane, 17.6 mmol, 2.2 equiv). The reaction was stirred at -78 ºC for 1 h. A solution of ene ester **74** (2.2 g 8 mmol, 1.0 equiv) in THF (15 mL) was then added, using another aliquot of THF (5 mL) to complete the transfer. After slowly warmed to 23 °C and stirred for 1 h, the mixture was quenched with saturated NH_4Cl aqueous solution and extracted with EtOAc (3 x 80 mL). The combined organic layer was washed with brine, dried over Na_2SO_4 and concentrated under vacuum. The residue was purified by flash column chromatography (EtOAc: petroleum ether = 1:12 to 100:0) to give starting material (510 mg, 1.9 mmol) and phospholipids product **75** (800 mg, 2.2 mmol, 27%, 36% brsm) as a yellow oil.
^{1}H NMR (500 MHz, $CDCl_3$) δ 7.62 (d, *J* = 8.0 Hz, 2H), 7.31 (d, *J* = 8.0 Hz, 2H), 3.75 (s, 3H), 3.73 (s, 3H), 3.34 – 3.30 (m, 2H), 3.11 (d, *J* = 22.0 Hz, 2H), 2.71 – 2.67 (m, 2H), 2.11 (p, *J* = 8.0 Hz, 2H).
^{13}C NMR (125 MHz, $CDCl_3$) δ 190.7 (d, *J* = 7.5 Hz), 165.8, 139.9, 133.9 (d, *J* = 5.0 Hz), 129.8, 129.6 (q, *J* = 32.5 Hz),125.5 (q, *J* = 3.8 Hz), 124.0 (q, *J* = 270.0 Hz), 52.9, 52.9, 39.1 (d, *J* = 132.5 Hz), 34.7, 32.7, 17.1.
^{19}F NMR (471 MHz, $CDCl_3$) δ -62.63.
^{31}P NMR (202 MHz, $CDCl_3$) δ 23.37.
HRMS (ESI) calcd for $[M+Na]^+$ $C_{16}H_{18}F_3NaO_4P$, m/z: 385.0787 , found: 385.0786.
IR *v* [cm^{-1}] 2958, 2855, 1664, 1614, 1327, 1258, 1066, 1033, 835.

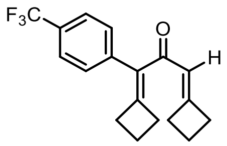

2v

Compound **2v.** To a stirred solution of **75** (324 mg, 1.0 mmol, 1.0 equiv) in THF (4.0 mL) at 0 ºC was slowly added NaH (44 mg, 60% in oil, 1.1 mmol, 1.1 equiv). After the mixture was stirred for 0.5 h, cyclobutanone **23** (97 µL, 1.3 mmol, 1.3 equiv) was added. The resulting mixture was warmed to rt and stirred for 12 h before it was quenched with saturated NH_4Cl aqueous solution and extracted with Et_2O (3 x 20 mL). The combined organic layer was washed with brine, dried over Na_2SO_4 and concentrated under vacuum. The residue was purified by flash column chromatography (EtOAc : petroleum ether = 1:20) to give dienone product **2v** (73 mg, 0.24 mmol, 24%) as a colorless oil.
^{1}H NMR (500 MHz, $CDCl_3$) δ 7.59 (d, *J* = 8.0 Hz, 2H), 7.29 (d, *J* = 8.0 Hz, 2H), 5.91 – 5.90 (m, 1H), 3.25 – 3.22 (m, 2H), 3.19 – 3.15 (m, 2H), 2.80 – 2.73 (m, 4H), 2.09 (p, *J* = 8.0, Hz, 2H), 2.09 (p, *J* = 8.0, Hz, 2H).

^{13}C NMR (125 MHz, $CDCl_3$) δ 190.3, 168.6, 159.7, 140.6, 134.3, 129.4, 128.9 (q, J = 32.5 Hz), 125.2 (q, J = 3.8 Hz), 124.2 (q, J = 270.0 Hz), 119.4, 34.8, 34.1, 33.0, 32.5, 18.4, 17.5.
^{19}F NMR (471 MHz, $CDCl_3$) δ -62.51.
HRMS (ESI) calcd for $[M+Na]^+$ $C_{18}H_{17}F_3NaO$, m/z: 329.1124 , found: 329.1125.
IR v [cm^{-1}] 2957, 1685, 1626, 1326, 1166, 1126, 1067, 844.

3.22 Preparation of starting material 2ee

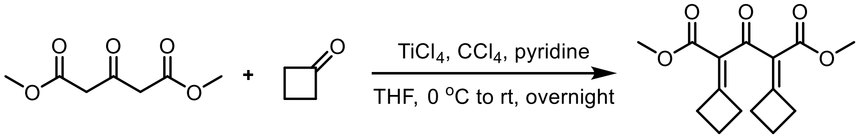

76 23 2ee

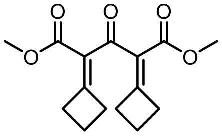

2ee

Compound **2ee.** To a stirred solution of CCl_4 (1.9 mL, 20 mmol, 4.0 equiv) in THF (15 mL) at 0 ºC was slowly added $TiCl_4$ (2.2 mL, 20 mmol, 4.0 equiv). After the mixture was stirred at the same temperature for 0.5 h, Compound **76** (0.7 mL, 5 mmol, 1.0 equiv) and cyclobutanone **23** (1.5 mL, 20 mmol, 4.0 equiv) was added to the solution. The solution was left to stir for 0.5 hour at 0°C and then pyridine (3.2 mL, 40 mmol, 8.0 equiv) was added dropwise. After the mixture was stirred at room temperature overnight, the reaction was quenched carefully with saturated $NaHCO_3$ aqueous solution and extracted with EtOAc (4 x 25 mL). The combined organic layer was washed with saturated $CuSO_4$ aqueous solution, brine, dried over Na_2SO_4 and concentrated under vacuum. The residue was purified by flash column chromatography (EtOAc: petroleum ether = 1:30 to 1:20) to give ene ester product **2ee** (1.25 g, 4.5 mmol, 90% yield) as a white solid.

^{1}H NMR (400 MHz, $CDCl_3$) δ 3.72 – 3.61 (s, 6H), 3.19 (dd, J = 8.0, 7.6 Hz, 2H), 3.12 (dd, J = 8.0, 6.4 Hz, 2H), 2.15 – 2.07 (m, 4H).
^{13}C NMR (100 MHz, $CDCl_3$): δ 188.6, 176.4, 165.4, 126.7, 51.2, 34.9, 34.2, 17.7.
HRMS (ESI) calcd for $[M+Na]^+$ $C_{15}H_{18}NaO_5$, m/z : 301.1046, found : 301.1049.
IR v [cm^{-1}] 2990, 2954, 1718, 1673, 1640,1435,1317,1245,1048.

3.23 Preparation of starting material 4o

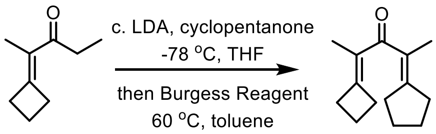

25 **4o**

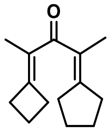

4o

Compound **4o.** To a stirred solution of enone **25** (181 mg, 1.3 mmol, 1.0 equiv) in THF (10 mL) at -78 ºC was slowly added LDA (4.9 mL, 0.8 M in THF, 3.9 mmol, 3.0 equiv). After the mixture was stirred at the same temperature for 1 h, cyclopentanone **23** (348 µl, 3.9 mmol, 3.0 equiv) was added to the flask. After the mixture was stirred for 20 minutes, the reaction was quenched carefully with saturated NH_4Cl aqueous solution and extracted with EtOAc (3 x 10 mL). The combined organic layer was washed with brine, dried over Na_2SO_4 and concentrated under vacuum. The crude product was purified by a short flash column chromatography (EtOAc: petroleum ether = 1:20). The above crude product was dissolved in toluene (10 mL) at rt, and burgess reagent (937 mg, 3.9 mmol, 3.0 equiv) was added to the mixture. After stirred at 60 ºC for 0.5 h, the mixture was cooled to room temperature and the reaction mixture was quenched by water and extracted with EtOAc (3 x 15 mL). The organic phase was dried over Na_2SO_4, and evaporated under reduced pressure to give crude product. The residue was purified by flash column chromatography (EtOAc: petroleum ether = 1:50) to give dienone product **4o** (39 mg, 0.2 mmol, 15% yield for 2 steps) as a colorless oil.

^{1}H NMR (500 MHz, $CDCl_3$): δ 2.81 – 2.78 (m, 4H), 2.22 – 2.16 (m, 4H), 1.96 (p, J = 8.0 Hz, 2H), 1.75 – 1.74 (m, 4H), 1.67 – 1.66 (m, 4H), 1.65 – 1.60 (m, 4H).

^{13}C NMR (125 MHz, $CDCl_3$): δ 201.0, 157.1, 144.7, 128.9, 128.8, 32.5, 31.7, 31.5, 31.2, 27.1, 25.7, 16.4, 16.3, 12.3.

HRMS (ESI) calcd for $[M+Na]^+$ $C_{14}H_{20}NaO$, m/z : 227.1406, found : 227.1407.

IR ν [cm^{-1}] 2954, 2867, 1634, 1450, 1431, 1332, 1302, 1076, 987.

3.24 Preparation of starting material 4p

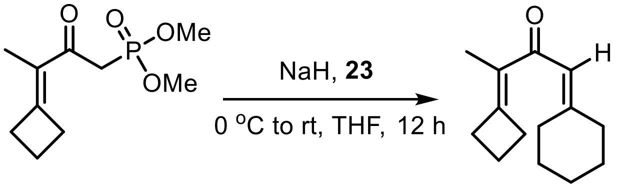

64 **4p**

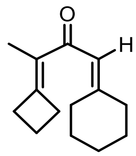

4p

Compound **4p.** To a stirred solution of **64** (348 mg, 1.5 mmol, 1.0 equiv) in THF (10.0 mL) at 0 ºC was slowly added NaH (60% in oil, 99 mg, 1.65 mmol, 1.1 equiv). After the mixture was stirred for 0.5 h, cyclohexanone **23** (0.2 mL, 2.0 mmol, 1.3 equiv) was added. The mixture was allowed to warm to rt and stirred for 10 h, the reaction was quenched with saturated NH_4Cl aqueous solution and extracted with Et_2O (3 x 10 mL). The combined organic layer was washed with brine, dried over Na_2SO_4 and concentrated under vacuum. The residue was purified by flash column chromatography (EtOAc: petroleum ether = 1:50) to give dienone product **4p** (35 mg, 0.2 mmol, 11% yield) as a colorless oil.

^{1}H NMR (400 MHz, $CDCl_3$): δ 6.01 (s, 1H), 3.02-3.06 (m, 2H), 2.84 – 2.79 (m, 2H), 2.61-2.59 (m, 2H), 2.18 – 2.15 (m, 2H), 2.05 – 1.97 (m, 2H), 1.67 (t, J = 1.6 Hz, 3H), 1.65 – 1.56 (m, 6H).

^{13}C NMR (100 MHz, $CDCl_3$): δ 193.7, 157.3, 156.8, 130.7, 122.4, 38.0, 34.2, 31.7, 30.3, 28.7, 27.9, 26.3, 16.5, 12.7.

HRMS (ESI) calcd for $[M+Na]^+$ $C_{14}H_{20}NaO$, m/z : 227.1406, found : 227.1408.

IR ν [cm^{-1}] 2928, 2855, 1770, 1650, 1615, 1447, 1288, 1228, 1109, 1093, 983.

3.25 General procedure A for synthesis corresponding ketene 77 to 88 and enol forms pruducts 77' to 88'

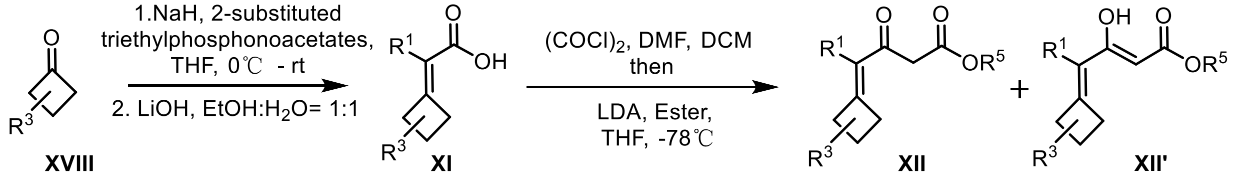

2-substituted triethylphosphonoacetate (14.0 mmol, 1.4 equiv) was added to a stirred suspension of sodium hydride (520 mg, 60% dispersion in oil, 13.0 mmol, 1.3 equiv) in THF (25 mL) at 0 °C. The mixture was allowed to warm to ambient temperature and stirred for 1 h. Then substituted cyclobutanone **I** [3] [4] [5] [6] (10 mmol, 1.0 equiv) was added as a solution in THF (25 mL). After the mixture was stirred for 12 h, saturated aqueous NH_4Cl solution (50 mL) was added and the mixture was extracted with EtOAc (2 x 50 mL). The organic layers were washed with brine (50 mL) then dried over Na_2SO_4, filtered and concentrated under reduced pressure as an oil that was used directly in the next step.

The corresponding ester and powdered LiOH (840 mg, 35.0 mmol, 3.5 equiv) were dissolved in EtOH (10 mL) with H_2O (10 mL) and refluxed for 2 h. The mixture was then cooled to 0°C and acidified to pH = 1 with 1 M hydrochloric acid. The mixture was extracted with EtOAc (2 x 50

mL). The combined organic layers were washed with brine (50 mL), dried over Na_2SO_4, filtered and concentrated under reduced pressure as a white powder **II** that was used directly in the next step.

Oxalyl chloride (1.27 mL, 15.0 mmol, 1.5 equiv) was added to a stirred solution of cinnamic acid **II** in DCM (20 mL) at 0 °C. Dimethylformamide (few drops) was then added carefully. After evolution of gas for a short period of time, the reaction was left to stir at rt for 3 hours. Solvent and excess oxalyl chloride were removed under reduced pressure to give the cinnamic acid chloride which was used for next step without further purification.

To a stirred solution of di*iso*propylamine (2.8 mL, 20.0 mmol, 2.0 equiv) in THF (25 mL) at -78°C, *n*-BuLi (8.0 mL, 2.5 M in hexane, 2.0 equiv) was added dropwise. The solution was left to stir for 30 minutes and then ester (12.0 mmol, 1.2 equiv) was added dropwise. The solution was left to stir at -78°C for another 30 minutes. A solution of the substituted cinnamic acid chloride (made in above step) in THF (25 mL) was then slowly added into the enolate solution. The reaction mixture was left to stir at -78°C for 30 minutes, then the solution was quenched with saturated NH_4Cl solution and then extracted with EtOAc (2 x 100 mL). The combined organic layers were washed with brine (50 mL), dried over Na_2SO_4, filtered and concentrated under reduced pressure. The residue was purified by flash chromatography on silica gel (EtOAc: petroleum ether = 1:25) to give the corresponding product ketene **III** and enol forms pruducts **III'**.

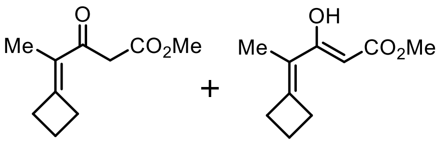

77 **77'**

Compound **77** and **77'** was synthesized following general procedure A, yellow oil, yield 79%.

^{1}H NMR (400 MHz, $CDCl_3$) δ 12.06 (s, 0.09H), 4.97 (s, 0.09H), 3.68 (s, 2.61H), 3.67 (s, 0.26H), 3.50 (s, 1.81H), 3.07 – 3.02 (m, 2H), 2.85 – 2.73 (m, 2H), 2.08 – 1.93 (m, 2H), 1.64 – 1.55 (m, 3H).

^{13}C NMR (100 MHz, $CDCl_3$) δ 192.4, 173.6, 168.2, 160.7, 153.7, 128.9, 120.3, 86.2, 52.0, 50.9, 47.8, 33.8, 31.8, 31.3, 16.8, 16.2, 12.6, 12.5.

HRMS (ESI) calcd for $[M+Na]^+$ $C_{10}H_{14}NaO_3$, m/z : 205.0835, found : 205.0833.

IR ν [cm^{-1}] 2954, 2924, 1744, 1654, 1598, 1440,1333, 1233, 1013, 799.

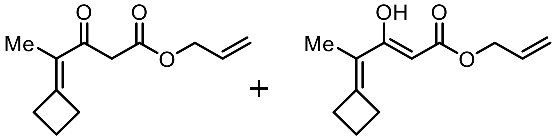

78 **78'**

Compound **78** and **78'** was synthesized following general procedure A, yellow oil, yield 69%.
^{1}H NMR (400 MHz, $CDCl_3$) δ 12.06 (s, 0.27H), 5.93 – 5.83 (m, 1H), 5.32 – 5.26 (m, 1H), 5.19 (dd, J = 10.4, 1.2 Hz, 1H), 5.00 (s, 0.28H), 4.60 – 4.58 (m, 2H), 3.52 (s, 1.52H), 3.07 – 3.03 (m, 2H), 2.85 – 2.73 (m, 2H), 2.08 – 1.93 (m, 2H), 1.64 – 1.63 (m, 2.30H), 1.57 – 1.56 (m, 0.85H).
^{13}C NMR (100 MHz, $CDCl_3$) δ 192.3, 173.8, 172.8, 167.4, 160.6, 153.9, 132.2, 131.7, 128.9, 120.3, 118.3, 117.9, 86.2, 65.5, 64.3, 47.9, 33.8, 33.8, 31.8, 31.3, 16.8, 16.1, 12.6, 12.5.
HRMS (ESI) calcd for $[M+Na]^+$ $C_{12}H_{16}NaO_3$, m/z : 231.0992, found : 231.0996.
IR ν [cm^{-1}] 3089, 2985, 2955, 1744, 1655, 1408,1333, 1196, 991, 931.

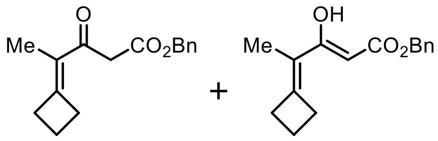

79 **79'**

Compound **79** and **79'** was synthesized following general procedure A, yellow oil, yield 78%.
^{1}H NMR (500 MHz, $CDCl_3$) δ 12.12 (s, 0.11H), 7.37 – 7.29 (m, 5H), 5.18 (s, 2H), 5.08 (s, 0.11H), 3.58 (s, 1.85H), 3.11 – 3.03 (m, 2H), 2.87 – 2.78 (m, 2H), 2.08 – 2.00 (m, 2H), 1.68 – 1.67 (m, 2.90H), 1.61 – 1.60 (m, 0.36H).
^{13}C NMR (125 MHz, $CDCl_3$) δ 192.4, 174.0, 173.1, 167.7, 160.6, 154.1, 136.0, 135.5, 129.1, 128.5, 128.5, 128.3, 128.2, 128.1, 128.1, 120.4, 86.5, 66.8, 65.6, 48.1, 33.9, 33.8, 31.9, 31.5, 16.9, 16.2, 12.8, 12.7.
HRMS (ESI) calcd for $[M+Na]^+$ $C_{16}H_{18}NaO_3$, m/z : 281.1148, found : 281.1150.
IR ν [cm^{-1}] 2956, 2924, 1743, 1657, 1651, 1334,1281, 1192, 743, 699.

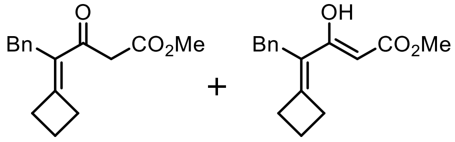

80 **80'**

Compound **80** and **80'** was synthesized following general procedure A, yellow oil, yield 77%.
^{1}H NMR (500 MHz, $CDCl_3$) δ 12.16 (s,0.10H), 7.27 – 7.24 (m, 2H), 7.18 – 7.15 (m, 3H), 4.96 (s, 0.10H), 3.68 (s, 2.65H), 3.67 (s, 0.31H), 3.52 (s, 1.82H), 3.50 (s, 1.76H), 3.44 (s, 0.20H), 3.20-3.17 (m, 2H), 2.98 – 2.87 (m Hz, 2H), 2.15 – 2.08 (m, 2H).
^{13}C NMR (125 MHz, $CDCl_3$) δ 192.1, 173.6, 172.6, 168.2, 162.9, 155.6, 139.6, 132.7, 128.4, 128.4, 128.3, 127.9, 126.0, 124.2, 87.8, 52.2, 51.0, 48.1, 34.2, 34.0, 33.0, 32.9, 32.2, 31.6, 17.0, 16.4.
HRMS (ESI) calcd for $[M+Na]^+$ $C_{16}H_{18}NaO_3$, m/z : 281.1148, found : 281.1151.
IR ν [cm^{-1}] 3061, 3027, 2953, 2918, 1747, 1662,1437, 1332, 1287, 1254, 1149, 1019, 992, 703.

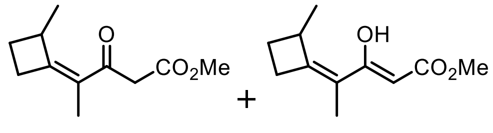

81 **81'**

Compound **81** and **81'** was synthesized following general procedure A, yellow oil, yield 23% and Compound **82** and **82'** was synthesized following general procedure A, yellow oil, yield 57% in one pot.

^{1}H NMR (400 MHz, $CDCl_3$) δ 12.09 (s, 0.38H), 5.04 (s, 0.39H), 3.73 – 3.72 (m, 3H), 3.58 (d, J = 4.0 Hz, 1.25H), 3.42 – 3.21 (m, 1H), 2.95 – 2.80 (m, 1H), 2.70 – 2.59 (m, 1H), 2.29 – 2.16 (m, 1H), 1.73 – 1.50 (m, 4H), 1.30 – 1.27 (m, 3H).

^{13}C NMR (100 MHz, $CDCl_3$) δ 192.8, 173.7, 173.4, 168.4, 165.1, 158.4, 126.9, 120.2, 86.5, 52.2, 51.0, 47.5, 40.5, 40.4, 29.1, 28.6, 24.4, 24.4, 19.8, 19.4, 13.6, 13.3.

HRMS (ESI) calcd for $[M+Na]^+$ $C_{11}H_{16}NaO_3$, m/z : 219.0992, found : 219.0994.

IR ν [cm^{-1}] 2953, 2928, 1745, 1664, 1652, 1600,1438, 1234, 1004.

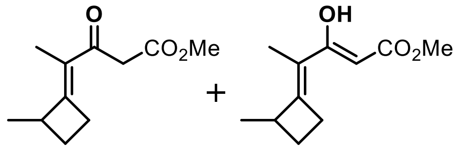

82 **82'**

Compound **81** and **81'** was synthesized following general procedure A, yellow oil, yield 23% and Compound **82** and **82'** was synthesized following general procedure A, yellow oil, yield 57% in one pot.

^{1}H NMR (400 MHz, $CDCl_3$) δ 12.10 (s, 0.37H), 5.00 (s, 0.38H), 3.71 – 3.70 (m, 3H), 3.52 (s, 1.17H), 3.19 – 3.05 (m, 2H), 3.00 – 2.88 (m, 1H), 2.31 – 2.16 (m, 1H), 1.71 – 1.50 (m, 4H), 1.26 – 1.22 (m, 3H).

^{13}C NMR (100 MHz, $CDCl_3$) δ 193.1, 174.1, 173.7, 168.4, 164.7, 158.0, 129.0, 120.6, 86.4, 52.1, 51.0, 47.9, 39.1, 38.7, 31.1, 31.0, 25.2, 24.5, 19.3, 18.8, 12.6, 12.5.

HRMS (ESI) calcd for $[M+Na]^+$ $C_{11}H_{16}NaO_3$, m/z : 219.0992, found : 219.0994.

IR ν [cm^{-1}] 2952, 2929, 1749, 1666, 1638, 1599, 1442, 1241, 1022.

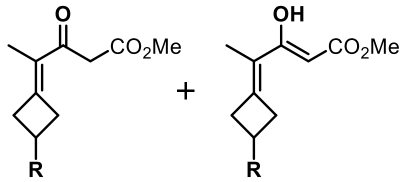

83 **83'**

R = SiPhMe$_2$

Compound **83** and **83'** was synthesized following general procedure A, yellow oil, yield 82%.

^{1}H NMR (500 MHz, $CDCl_3$) δ 12.13 (s, 0.11H), 7.51 – 7.34 (m, 5H), 5.01 (s, 0.09H), 3.73 (s, 3H), 3.51 (s, 1.72H), 3.29 – 3.23 (m, 1H), 3.06 – 2.94 (m, 2H), 2.76 – 2.71 (m, 1H), 2.04 – 1.97 (m, 1H), 1.64 (s, 2.53H), 1.57 (s, 0.40H), 0.33 – 0.16 (m, 6H).

^{13}C NMR (125 MHz, $CDCl_3$) δ 192.4, 173.6, 173.5, 168.3, 160.1, 152.9, 138.0, 137.3, 133.6, 133.6, 129.2, 129.0, 128.6, 127.8, 127.7, 120.4, 86.4, 52.1, 51.0, 47.9, 35.1, 35.0, 33.0, 32.5, 15.6, 15.1, 12.6, 12.5, -5.3, -5.4, -5.5, -5.6.

HRMS (ESI) calcd for $[M+Na]^+$ $C_{18}H_{24}NaO_3Si$, m/z : 339.1387, found : 339.1388.

IR ν [cm^{-1}] 3069, 2953, 1747, 1653, 1438, 1428,1329, 1250, 1114, 831, 811, 772, 736, 710.

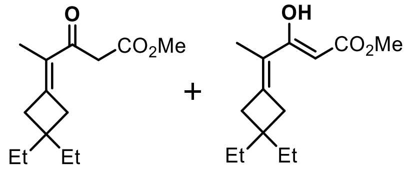

84 **84'**

Compound **84** and **84'** was synthesized following general procedure A, yellow oil, yield 66%.

^{1}H NMR (400 MHz, $CDCl_3$) δ 12.11 (s, 0.11H), 5.01 (s, 0.11H), 3.72 – 3.71 (m, 3H), 3.54 (s, 1.72H), 2.70 – 2.68 (m, 2H), 2.48 – 2.37 (m, 2H), 1.73 – 1.61 (m, 3H), 1.50 – 1.44 (m, 4H), 0.83 – 0.76 (m, 6H).

^{13}C NMR (100 MHz, $CDCl_3$) δ 192.6, 173.7, 168.4, 156.7, 149.6, 130.5, 122.0, 86.3, 52.1, 51.0, 47.9, 43.6, 41.6, 41.1, 38.2, 37.8, 29.7, 13.1, 13.0, 8.3, 8.2.

HRMS (ESI) calcd for $[M+Na]^+$ $C_{14}H_{22}NaO_3$, m/z : 261.1461, found : 261.1464.

IR ν [cm^{-1}] 2961, 2935, 2935, 2905, 1747, 1656,1600, 1456, 1401, 1330, 1282, 1208, 1022, 1006, 799.

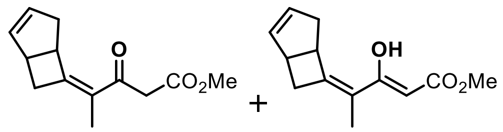

85 **85'**

Compound **85** and **85'**was synthesized following general procedure A, yellow oil, yield 23% and Compound **86** and **86'** was synthesized following general procedure A, yellow oil, yield 57% in

one pot.
^{1}H NMR (400 MHz, $CDCl_3$) δ 12.09 (s, 0.32H), 5.84 – 5.65 (m, 2H), 5.06 (s, 0.34H), 3.91 – 3.83 (m, 1H), 3.74 (s, 1.85H), 3.73 (s, 1H), 3.66 – 3.57 (m, 1.36H), 3.38 – 3.27 (m, 1H), 3.05 – 2.94 (m, 1H), 2.87 – 2.67 (m, 1H), 2.62 – 2.41 (m, 2H), 1.69 – 1.57 (m, 3H).
^{13}C NMR (100 MHz, $CDCl_3$) δ 192.5, 173.7, 173.0, 168.3, 164.5, 157.3, 133.1, 133.0, 131.8, 131.4, 129.5, 122.6, 86.5, 52.2, 51.1, 47.6, 46.9, 46.8, 41.3, 39.7, 39.7, 39.5, 38.9, 13.4, 13.2.
HRMS (ESI) calcd for $[M+Na]^+$ $C_{13}H_{16}NaO_3$, m/z : 243.0992, found : 243.0992 .
IR ν [cm^{-1}] 2955, 1738, 1712, 1653, 1438, 1328, 1206, 1152, 1003, 844.

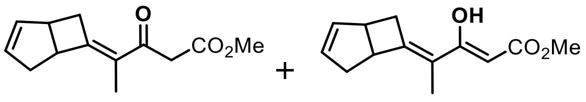

86 **86'**

Compound **85** and **85'**was synthesized following general procedure A, yellow oil, yield 23% and Compound **86** and **86'** was synthesized following general procedure A, yellow oil, yield 57% in one pot.
^{1}H NMR (400 MHz, $CDCl_3$) δ 12.04 (s, 0.19H), 5.80 – 5.73 (m, 2H), 5.01 (s, 0.19H), 3.70 (s, 2.22H), 3.70 (s, 0.63H), 3.68 – 3.54 (m, 1H), 3.49 (s, 1.62H), 3.41 – 3.28 (m, 1H), 3.27 – 3.20 (m, 1H), 2.79 – 2.62 (m, 2H), 2.52 – 2.44 (m, 1H), 1.76 – 1.66 (m, 3H).
^{13}C NMR (100 MHz, $CDCl_3$) δ 192.9, 173.7, 173.6, 168.3, 163.6, 156.9, 133.6, 133.1, 131.4, 131.3, 130.8, 123.0, 86.4, 52.1, 51.0, 47.9, 45.3, 44.8, 42.3, 41.6, 41.3, 41.2, 38.1, 37.8, 13.5.
HRMS (ESI) calcd for $[M+Na]^+$ $C_{13}H_{16}NaO_3$, m/z : 243.0992, found : 243.0992 .
IR ν [cm^{-1}] 2954, 2925, 1742, 1714, 1664, 1559, 1439, 1324, 1276, 1203, 1004, 716.

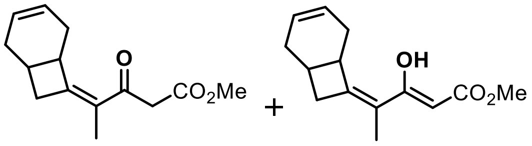

87 **87'**

Compound **87** and **87'** was synthesized following general procedure A, yellow oil, yield 31% and Compound **88** and **88'** was synthesized following general procedure A, yellow oil, yield 58% in one pot.
^{1}H NMR (400 MHz, $CDCl_3$) δ 12.12 (s, 0.43H), 5.92 – 5.84 (m, 2H), 5.04 (s, 0.41H), 3.73 (s, 1.76H), 3.72 (s, 1.29H), 3.58 (d, J = 2.0 Hz, 1H), 3.56 – 3.49 (m, 1H), 2.82 – 2.72 (m, 1H), 2.71 – 2.60 (m,1H), 2.44 – 2.19 (m, 3H), 2.19 – 2.04 (m, 1H), 2.05 – 1.93 (m, 1H), 1.71 – 1.69 (m, 1.85H), 1.57 – 1.56 (m, 1.21H).

^{13}C NMR (100 MHz, $CDCl_3$) δ 192.7, 173.8, 173.3, 168.4, 163.9, 156.6, 129.0, 128.2, 126.6, 126.5, 120.4, 86.3, 52.2, 51.0, 47.5, 42.8, 42.6, 36.1, 35.9, 27.4, 27.3, 27.3, 27.1, 26.0, 25.4, 13.7, 13.3.
HRMS (ESI) calcd for $[M+Na]^+$ $C_{14}H_{18}NaO_3$, m/z : 257.1148, found : 257.1148 .
IR *v* [cm^{-1}] 2952, 2934, 1743, 1652, 1439, 1332, 1208, 1152, 1004.

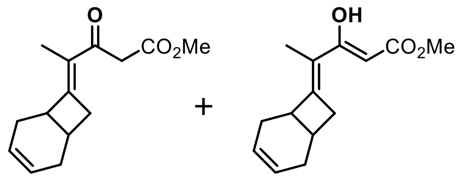

88 **88'**

Compound **87** and **87'** was synthesized following general procedure A, yellow oil, yield 31% and Compound **88** and **88'** was synthesized following general procedure A, yellow oil, yield 58% in one pot.
^{1}H NMR (400 MHz, $CDCl_3$) δ 12.09 (s, 0.09H), 5.95 – 5.88 (m, 2H), 5.01 (s, 0.10H), 3.72 (s, 2.62H), 3.71 (s, 0.36H), 3.51 (s, 1.73H), 3.36 – 3.27 (m, 1H), 3.11 – 3.04 (m, 1H), 2.75 – 2.68 (m, 1H), 2.66 – 2.58 (m, 1H), 2.33 – 2.11 (m, 3H), 2.07 – 1.96 (m, 1H), 1.73 – 1.66 (m, 3H).
^{13}C NMR (100 MHz, $CDCl_3$) δ 193.1, 168.4, 162.6, 129.4, 127.8, 127.1, 86.4, 52.1, 51.0, 48.0, 41.6, 41.1, 38.4, 38.3, 27.9, 27.3, 27.0, 25.5, 24.9, 12.9, 12.8.
HRMS (ESI) calcd for $[M+Na]^+$ $C_{14}H_{18}NaO_3$, m/z : 257.1148, found : 257.1149 .
IR *v* [cm^{-1}] 2947, 1746, 1663, 1637, 1596, 1439, 1402, 1321, 1259, 1203, 1132, 1022.

3.26 General procedure B for synthesis corresponding ketene 89 to 92 and enol forms pruducts 89' to 92'

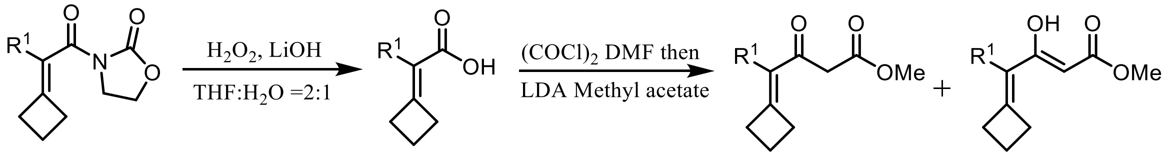

VII **XI** **XII** **XII'**

Alkylidenecyclobutanes[1] **IV** (10.0 mmol, 1.0 equiv) was dissolved in 60 mL of 2:1 THF/H_2O and cooled to 0 °C. 30 wt. % H_2O_2 in H_2O (3 mL, 30.0 mmol, 3.0 equiv) was added to the flask, followed by LiOH (720 mg, 30.0 mmol, 3.0 equiv). After 1 h, peroxides were quenched with saturated $Na_2S_2O_3$ solution. 2-Oxazolidinone was removed from the aqueous layer by continuous extraction with DCM overnight. The aqueous layer was then acidified to pH = 1with 1M HCl and extracted with EtOAc (5 x 50 mL). The combined organic layers were washed with brine (50 mL), dried over Na_2SO_4, filtered and concentrated under reduced pressure as a white powder **II** that was used directly in the next step.

Oxalyl chloride (1.3 ml, 15.0 mmol, 1.5 equiv) was added to a stirred solution of substituted cinnamic acid **II** in DCM (20 mL) at 0 °C. Dimethylformamide (few drops) was then added carefully. After evolution of gas for a short period of time, the reaction was left to stir at rt for 3 hours. Solvent and excess oxalyl chloride were removed under reduced pressure to give the acid chloride which was used for next step without further purification.
To a stirred solution of di*iso*propylamine (2.8 mL, 20.0 mmol, 2.0 equiv) in THF (25 mL) at -78°C, *n*-BuLi (8.0 mL, 2.5 M in hexane, 20 mmol, 2.0 equiv) was added dropwise. The solution was left to stir for 30 minutes and then methyl acetate (1.0 mL, 12.0 mmol, 1.2 equiv) was added dropwise. The solution was left to stir at -78°C for another 30 minutes. A solution of the substituted cinnamic acid chloride (made in above step) in THF (25 mL) was then slowly added into the enolate solution. The reaction mixture was left to stir at -78°C for 30 minutes. Then the solution was quenched with saturated NH_4Cl solution and then extracted with EtOAc (2 × 100 mL). The combined organic layers were washed with brine (50 mL), dried over Na_2SO_4, filtered and concentrated under reduced pressure. The residue was purified by flash chromatography on silica gel (EtOAc: petroleum ether = 1:25) to give the corresponding product ketene **III** and enol forms pruducts **III'**..

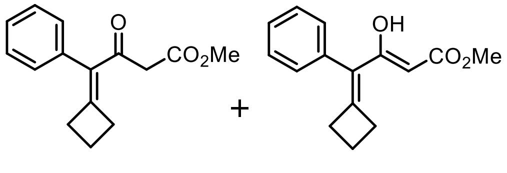

89 **89'**

Compound **89** and **89'** was synthesized following general procedure B, yellow oil, yield 55%.
^{1}H NMR (400 MHz, $CDCl_3$) δ 12.19 (s, 0.49H), 7.38 – 7.13 (m, 5H), 4.69 (s, 0.48H), 3.67 (s, 3H), 3.42 (s, 0.94H), 3.30 – 3.25 (m, 2H), 2.71 – 2.61 (m, 2H), 2.13 – 2.00 (m, 2H).
^{13}C NMR (100 MHz, $CDCl_3$) δ 192.5, 173.6, 173.4, 168.2, 164.5, 156.2, 136.2, 135.9, 133.7, 129.6, 129.3, 128.5, 128.2, 127.5, 127.1, 89.3, 52.0, 51.0, 48.2, 34.6, 34.2, 32.4, 32.2, 17.4, 17.3.
HRMS (ESI) calcd for $[M+Na]^+$ $C_{15}H_{16}NaO_3$, m/z : 267.0992, found : 267.0997.
IR ν [cm^{-1}] 2953, 1745, 1687, 1660, 1634, 1597, 1442, 1320, 1213, 1153, 805, 754, 701.

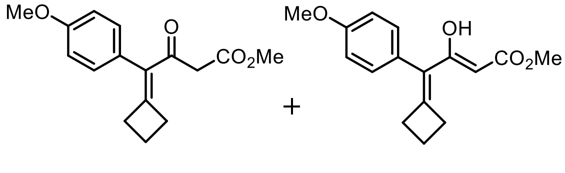

90 **90'**

Compound **90** and **90'** was synthesized following general procedure B, yellow oil, yield 36%.

^{1}H NMR (400 MHz, $CDCl_3$) δ 12.15 (s, 0.20H), 7.12 – 7.05 (m, 2H), 6.90 – 6.88 (m, 2H), 4.70 (s, 0.18H), 3.81 (s, 3H), 3.67 (s, 3H), 3.41 (s, 1.66H), 3.27 – 3.23 (m, 2H), 2.70 – 2.66 (m, 2H), 2.12 – 2.01 (m, 2H).
^{13}C NMR (100 MHz, $CDCl_3$) δ 192.9, 173.7, 173.6, 168.3, 164.2, 158.9, 158.6, 155.9, 133.3, 130.7, 130.4, 128.5, 128.1, 127.8, 114.0, 113.7, 89.2, 55.2, 55.2, 52.1, 51.0, 48.2, 34.6, 34.1, 32.4, 32.3, 17.4, 17.3.
HRMS (ESI) calcd for $[M+Na]^+$ $C_{16}H_{18}NaO_4$, m/z : 297.1097, found : 297.1094 .
IR ν [cm^{-1}] 2935, 1743, 1686, 1662, 1607, 1510, 1441, 1241, 1214, 1175, 1031, 833, 805.

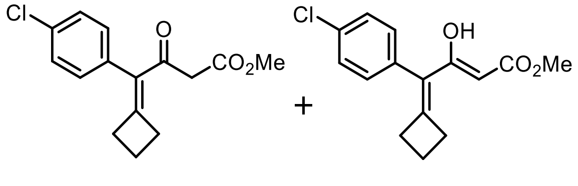

91 **91'**

Compound **91** and **91'** was synthesized following general procedure B, yellow oil, yield 66%.
^{1}H NMR (400 MHz, $CDCl_3$) δ 12.16 (s, 0.23H), 7.35 – 7.29 (m, 2H), 7.10 – 7.06 (m, 2H), 4.65 (s, 0.21H), 3.67 (s, 3H), 3.43 (s, 1.57H), 3.27 – 3.23 (m,2H), 2.70 – 2.61 (m, 2H), 2.13 – 2.02 (m, 2H).
^{13}C NMR (100 MHz, $CDCl_3$) δ 191.9, 173.4, 172.9, 168.0, 165.1, 156.8, 134.7, 134.3, 133.5, 133.1, 133.0, 131.0, 130.7, 128.7, 128.5, 127.2, 89.4, 52.1, 51.1, 48.1, 34.6, 34.2, 32.5, 32.2, 17.4, 17.2.
HRMS (ESI) calcd for $[M+Na]^+$ $C_{15}H_{15}ClNaO_3$, m/z :301.0602, found : 301.0600.
IR ν [cm^{-1}] 2989, 2953, 1747, 1662, 1636, 1598, 1490, 1442, 1399, 1217, 1155, 1091, 1017, 831, 806, 723.

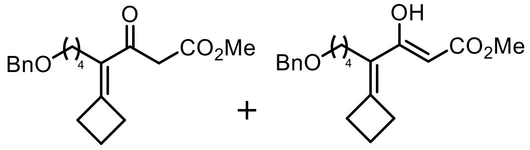

92 **92'**

Compound **92** and **92'** was synthesized following general procedure B, yellow oil, yield 44%.
^{1}H NMR (500 MHz, $CDCl_3$) δ 12.17 (s, 0.40H), 7.34 – 7.32 (m, 4H), 7.29 – 7.26 (m, 1H), 5.03 (s, 0.40H), 4.49 (s, 0.93H), 4.49 (s, 1.18H), 3.72 (s, 3H), 3.52 (s, 1.13H), 3.47 – 3.44 (m, 2H), 3.10 – 3.03 (m, 2H), 2.90 – 2.87 (m, 1H), 2.80 – 2.77 (m, 1H), 2.16 – 2.13 (m, 1H), 2.10 – 2.06 (m, 2H), 2.04 – 1.98 (m, 1H), 1.64 – 1.56 (m, 2H), 1.50 – 1.39 (m, 2H).
^{13}C NMR (125 MHz, $CDCl_3$) δ 192.2, 173.7, 173.0, 168.3, 160.8, 153.4, 138.6, 138.5, 134.2, 128.3, 127.6, 127.4, 125.7, 87.0, 72.9, 72.9, 70.2, 70.1, 52.1, 51.0, 48.2, 33.9, 33.8, 31.8, 31.3, 29.6, 29.5, 27.1, 25.3, 25.3, 17.0, 16.2.

HRMS (ESI) calcd for $[M+Na]^+$ $C_{20}H_{26}NaO_4$, m/z : 353.1723, found : 353.1731.
IR *ν* [cm^{-1}] 2948, 2859, 1744, 1661, 1652, 1599, 1417, 1206, 1100, 1027, 735, 697.

3.27 Preparation of corresponding ketene 94 and enol forms 94'

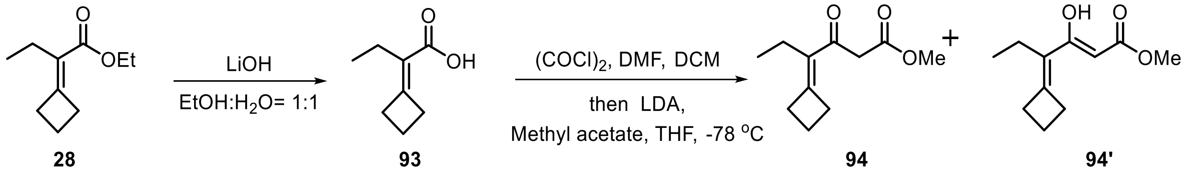

Compound **94** and **94'.** The corresponding ester **28** (5.0 g, 30.0 mmol, 1.0 equiv) and powdered LiOH (2.5 g, 105.0 mmol, 3.5 equiv) were dissolved in EtOH (30 mL) with H_2O (30 mL) and refluxed for 8 h. The mixture was then cooled to 0°C and acidified to pH = 1 with 1 M hydrochloric acid. The mixture was extracted with EtOAc (2 x 100 mL). The combined organic layers were washed with brine (50 mL), dried over Na_2SO_4, filtered and concentrated under reduced pressure as a white powder **93** that was used directly in the next step.
Oxalyl chloride (3.8 mL, 45.0 mmol, 1.5 equiv) was added to a stirred solution of substituted cinnamic acid **93** in DCM (60 mL) at 0 °C. Dimethylformamide (few drops) was then added carefully. After evolution of gas for a short period of time, the reaction was left to stir at rt for 3 hours. Solvent and excess oxalyl chloride were removed under reduced pressure to give the acid chloride which was used for next step without further purification.
To a stirred solution of di*iso*propylamine (8.4 mL, 60.0 mmol, 2.0 equiv) in THF (75 mL) at -78°C, *n*-BuLi (24.0 mL, 2.5 M in hexane, 60.0 mmol, 2.0 equiv) was added dropwise. The solution was left to stir for 30 minutes and then methyl acetate (3.0 mL, 36.0 mmol, 1.2 equiv) was added dropwise. The solution was left to stir at -78°C for another 30 minutes. A solution of the cinnamic acid chloride (made in above step) in THF (75 mL) was then slowly added into the enolate solution. The reaction mixture was left to stir at -78°C for 30 minutes, then the solution was quenched with saturated NH_4Cl solution and then extracted with EtOAc (2 x 150 mL). The combined organic layers were washed with brine (100 mL), dried over Na_2SO_4, filtered and concentrated under reduced pressure. The residue was purified by flash chromatography on silica gel (EtOAc: petroleum ether = 1:25) to give the corresponding product **94** and **94'** (3.6 g, 18.3 mmol, 61% yield) as a yellow oil.
^{1}H NMR (400 MHz, $CDCl_3$) δ 12.11 (s, 0.36H), 4.98 (s, 0.35H), 3.67 (s, 3H), 3.47 (s, 1.43H), 3.06 – 2.98 (m, 2H), 2.87 – 2.73 (m, 2H), 2.11 – 1.93 (m, 4H), 0.94 – 0.86 (m, 3H).
^{13}C NMR (101 MHz, $CDCl_3$) δ 192.0, 173.6, 172.8, 168.2, 160.2, 152.8, 135.6, 127.2, 86.6, 52.0, 50.9, 48.0, 33.8, 33.7, 31.4, 30.9, 20.5, 16.9, 16.2, 13.3, 13.1.
HRMS (ESI) calcd for $[M+Na]^+$ $C_{11}H_{16}NaO_3$, m/z: 219.0992, found: 219.0991.
IR *ν* [cm^{-1}] 2962, 2875, 1747, 1664, 1439, 1205, 1018, 804.

3.28 Preparation of corresponding ketene 96 and enol forms 96'

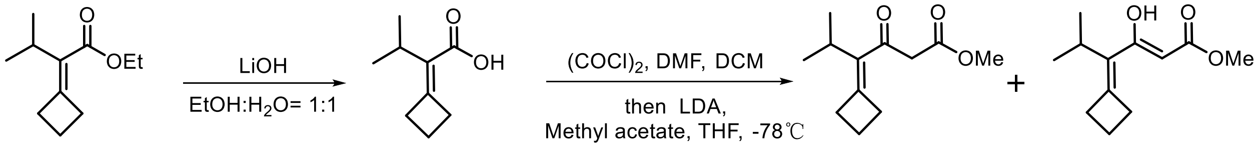

70 95 96 96'

Compound **96** and **96’**. The corresponding ester **70** (5.5 g, 30.0 mmol, 1.0 equiv) and powdered LiOH (2.5 g, 105.0 mmol, 3.5 equiv) were dissolved in EtOH (30 mL) with H_2O (30 mL) and refluxed for 24 h. The mixture was then cooled to 0°C and acidified to pH = 1 with 1 M hydrochloric acid. The mixture was extracted with EtOAc (2 x 100 mL). The combined organic layers were washed with brine (50 mL), dried over Na_2SO_4, filtered and concentrated under reduced pressure as a white powder **95** that was used directly in the next step.

Oxalyl chloride (3.8 mL, 45.0 mmol, 1.5 equiv) was added to a stirred solution of cinnamic acid **95** in DCM (60 mL) at 0 ℃. Dimethylformamide (few drops) was then added carefully. After evolution of gas for a short period of time, the reaction was left to stir at rt for 3 hours. Solvent and excess oxalyl chloride were removed under reduced pressure to give the acid chloride which was used for next step without further purification.

To a stirred solution of di*iso*propylamine (8.4 mL, 60.0 mmol, 2.0 equiv) in THF (75 mL) at -78°C, *n*-BuLi (24.0 mL, 2.5 M in hexane, 60.0 mmol, 2.0 equiv) was added dropwise. The solution was left to stir for 30 minutes and then methyl acetate (3.0 mL, 36.0 mmol, 1.2 equiv) was added dropwise. The solution was left to stir at -78 °C for another 30 minutes. A solution of the substituted cinnamic acid chloride (made in above step) in THF (75 mL) was then slowly added into the enolate solution. The reaction mixture was left to stir at -78 °C for 30 minutes, then the solution was quenched with saturated NH_4Cl solution and then extracted with EtOAc (2 x 150 mL). The combined organic layers were washed with brine (100 mL), dried over Na_2SO_4, filtered and concentrated under reduced pressure. The residue was purified by flash chromatography on silica gel (EtOAc: petroleum ether = 1:25) to give the corresponding product **96** and **96'** (5.0 g, 24.0 mmol, 80% yield) as a yellow oil.

^{1}H NMR (400 MHz, $CDCl_3$) δ 12.11 (s, 0.21H), 4.86 (s, 0.23H), 3.65 (s, 3H), 3.43 (s, 1.68H), 3.02 – 2.74 (m, 4H), 2.59 – 2.48 (m, 1H), 2.04 – 1.88 (m, 2H), 1.04 (d, *J* = 7.0 Hz, 6H).

^{13}C NMR (101 MHz, $CDCl_3$) δ 193.0, 173.8, 173.3, 168.2, 158.2, 148.0, 139.1, 132.0, 89.1, 51.9, 50.8, 49.2, 34.1, 33.1, 31.9, 30.9, 29.0, 20.9, 20.4, 16.9, 16.3.

HRMS (ESI) calcd for $[M+Na]^+$ $C_{12}H_{18}NaO_3$, m/z: 233.1148 , found: 233.1148.

IR *v* [cm^{-1}] 2958, 2876, 1747, 1664, 1439, 1321, 1023, 810.

3.29 Preparation of corresponding ketene 101 and enol forms 101'

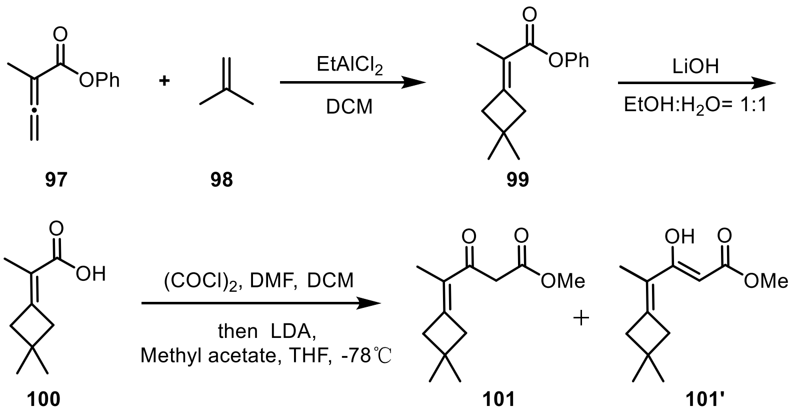

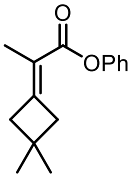

99

Compound **99.** To a flame-dried flask with 32 mL CH_2Cl_2 was added phenyl 2-methylbuta-2,3-dienoate **97** [2] (2.8 g, 16 mmol, 1.0 equiv), and 2-methylprop-1-ene **98** (16 mL, 2M in CH_2Cl_2, 32 mmol, 2.0 equiv). $EtAlCl_2$ (24 mL, 1 M in hexanes, 24 mmol, 1.5 equiv) was added to the flask. The reaction mixture was allowed to stir at room temperature for 6 h before quenched with Et_3N (1 mL) and HCl (10 mL, 1 M in H_2O). The mixture was allowed to stir until two distinct layers were formed, and then the layers were separated. The aqueous phase was extracted with CH_2Cl_2 (3 × 30 mL), and the combined organic layers were dried over $MgSO_4$ and concentrated under reduced pressure. The residue was purified by flash chromatography on silica gel (EtOAc: petroleum ether = 1:100) to give the corresponding ene ester product **99** (1.7 g, 8 mmol, 50%) as a white solid.

^{1}H NMR (400 MHz, $CDCl_3$) δ 7.40 – 7.36 (m, 2H), 7.21 (ddd, J = 7.0, 4.0, 1.2 Hz, 1H), 7.13 – 7.10 (m, 2H), 2.91 – 2.90 (m, 2H), 2.59 – 2.58 (m, 2H), 1.85 – 1.83 (m, 3H), 1.23 (s, 6H).

^{13}C NMR (100 MHz, $CDCl_3$) δ 165.9, 157.4, 150.9, 129.2, 125.3, 121.8, 120.3, 47.1, 44.4, 31.2, 28.9, 13.4.

HRMS (ESI) calcd for $[M+Na]^+$ $C_{15}H_{18}NaO_2$, m/z : 253.1199, found : 253.1198.

IR ν [cm^{-1}] 2950, 2925, 1720, 1675, 1593, 1492, 1191, 1116, 1042, 688.

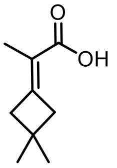

100

Compound **100.** The corresponding ester **99** (2.0 g, 9 mmol, 1.0 equiv) and powdered LiOH (760

mg, 35 mmol, 3.5 equiv) were dissolved in EtOH (10 mL) with H_2O (10 mL) and refluxed for 2 h. The mixture was then cooled to 0°C and acidified to pH = 1 with 1 M hydrochloric acid. The mixture was extracted with EtOAc (2 x 50 mL). The combined organic layers were washed with brine (50 mL), dried over Na_2SO_4, filtered and concentrated under reduced pressure. The residue was purified by flash chromatography on silica gel (EtOAc: petroleum ether = 1:25) to give the corresponding acid product **100** (920 mg, 6.5 mmol, 72%) as a white solid.

^{1}H NMR (400 MHz, $CDCl_3$) δ 2.83 – 2.82 (m, 2H), 2.51 (s, 2H), 1.70 – 1.69 (m, 3H), 1.18 (s, 6H).

^{13}C NMR (100 MHz, $CDCl_3$) δ 173.5, 158.4, 120.4, 47.2, 44.4, 31.2, 28.9, 13.0.

HRMS (ESI) calcd for $[M+Na]^+$ $C_9H_{14}NaO_2$, m/z : 177.0886, found : 177.0887.

IR ν [cm^{-1}] 2948, 2923, 2864, 2577, 1683, 1649,1428, 1321, 1304, 949.

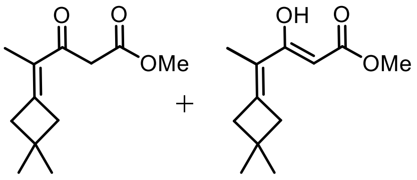

101 **101'**

Compound **101** and **101'**. Oxalyl chloride (0.7 mL, 8.0 mmol, 1.5 equiv) was added to a stirred solution of cinnamic acid **100** (1.1 g, 5.4 mmol, 1.0 equiv) in DCM (15 mL) at 0 ℃. Dimethylformamide (few drops) was then added carefully to the flask. After evolution of gas for a short period of time, the reaction was left to stir at rt for 3 hours. Solvent and excess oxalyl chloride were removed under reduced pressure to give the acid chloride which was used for next step without further purification.

To a stirred solution of di*iso*propylamine (1.5 mL, 10.8mmol, 2.0 equiv) in THF (20 mL) at -78 °C, *n*-BuLi (4.3mL, 10.8mmol, 2.5 M in hexane, 2.0 equiv) was added dropwise. The solution was left to stir for 30 minutes and then methyl acetate (0.5 mL, 6.5 mmol, 1.2 equiv) was added dropwise. The solution was left to stir at -78 °C for another 30 minutes. A solution of the cinnamic acid chloride (made in above step) in THF (10 mL) was then slowly added into the enolate solution. The reaction mixture was left to stir at -78 °C for 30 minutes, then the solution was quenched with saturated NH_4Cl solution and then extracted with EtOAc (2 x 100 mL). The combined organic layers were washed with brine (50 mL), dried over Na_2SO_4, filtered and concentrated under reduced pressure. The residue was purified by flash chromatography on silica gel (EtOAc: petroleum ether = 1:25) to give the corresponding product **101** and **101'** (673mg, 3.5mmol, 64%) as a yellow oil.

^{1}H NMR (400 MHz, $CDCl_3$) δ 12.09 (s, 0.24H), 5.01 (s, 0.24H), 3.72 (s, 2.23H), 3.71 (s, 0.73H), 3.53 (s, 1.47H), 2.79 – 2.75 (m, 2H), 2.58-2.49 (m, 2H), 1.68 – 1.61(m, 3H), 1.19 (s, 4.46H), 1.16 (s, 1.43H).

^{13}**C NMR** (100 MHz, $CDCl_3$) δ 192.6, 173.7, 173.7, 168.4, 155.7, 148.6, 130.7, 122.1, 86.3, 52.1, 51.0, 47.9, 47.1, 47.0, 45.1, 44.6, 31.5, 31.2, 28.9, 28.9, 13.1, 13.0.

HRMS (ESI) calcd for $[M+Na]^+$ $C_{12}H_{18}NaO_3$, m/z : 233.1148, found : 233.1152.

IR ν [cm^{-1}] 2952, 2926, 2902, 1748, 1657,1601,1440, 1403, 1334, 1283.

3.30 General procedure C for synthesis dienone products 2w to 2hh, 4a, 4c to 4j, 4l to 4n

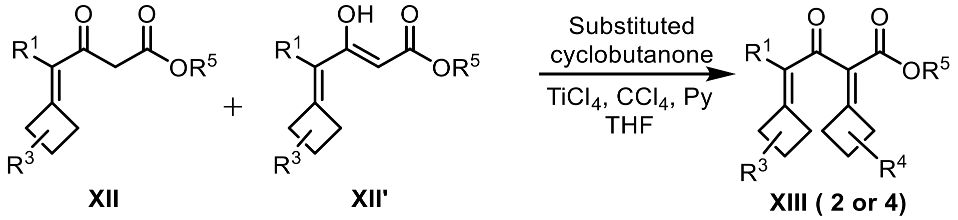

To a stirred solution of CCl_4 (2.0 equiv) in THF (0.2 M) at 0°C, and $TiCl_4$ (2.0 equiv) were added. The yellow solution was left to stir for 0.5 hour and then a mixture of the keto **III** and enol forms **III'** (made in above step) and substituted cyclobutanone [3] [4] [5] [6] (1.5 equiv) was added dropwise to the solution at 0°C. The solution was left to stir for 1 hour at 0°C and then pyridine (4.0 equiv) was added dropwise. The reaction mixture was allowed to warm to room temperature and stir for 5 days. The reaction was then quenched with water, the organic layer was separated and the aqueous layer was extracted with EtOAc for three times. The combined organic layers were washed with saturated $NaHCO_3$ and saturated brine, dried over Na_2SO_4, filtered, concentrated under reduced pressure and purified by flash chromatography on silica gel (EtOAc: petroleum ether = 1:40 to 1:25) to give the corresponding product dienone **2** or **4**.

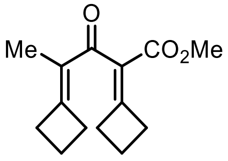

2w

Compound **2w** was synthesized following general procedure C, yellow solid, yield 74% (89% brsm).

^{1}**H NMR** (400 MHz, $CDCl_3$) δ 3.67 (s, 3H), 3.16 – 3.12 (m, 2H), 2.86 – 2.79 (m, 4H), 2.79 – 2.74 (m, 2H), 2.12 – 2.03 (m, 2H), 2.03 – 1.95 (m, 2H), 1.66 – 1.65 (m, 3H).

^{13}**C NMR** (100 MHz, $CDCl_3$) δ 192.8, 166.2, 164.5, 160.9, 129.7, 128.5, 51.4, 34.2, 33.2, 31.8, 31.4, 17.4, 16.3, 12.1.

HRMS (ESI) calcd for $[M+Na]^+$ $C_{14}H_{18}NaO_3$, m/z : 257.1148, found : 257.1152 .

IR ν [cm^{-1}] 2986, 2955, 1716, 1640, 1263, 1243, 1040.

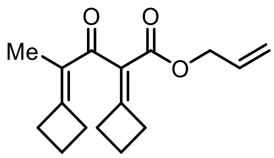

2x

Compound **2x** was synthesized following general procedure C, yellow oil, yield 46% (94% brsm).
^{1}H NMR (500 MHz, $CDCl_3$) δ 5.86 (ttd, J = 10.5, 5.5, 1.0 Hz, 1H), 5.27 – 5.23 (m, 1H), 5.28 – 5.16 (m, 1H), 4.59 – 4.58 (m, 2H), 3.14 (dd, J = 8.0, 8.0 Hz, 2H), 2.86 – 2.76 (m, 6H), 2.07 (p, J = 8.0 Hz, 2H), 1.98 (p, J = 8.0 Hz, 2H), 1.65 (s, 3H).
^{13}C NMR (125 MHz, $CDCl_3$) δ 192.8, 166.7, 163.7, 160.4, 132.0, 129.7, 128.6, 117.7, 64.7, 34.3, 33.1, 31.7, 31.5, 17.4, 16.3, 12.1.
HRMS (ESI) calcd for $[M+Na]^+$ $C_{16}H_{20}NaO_3$, m/z : 283.1305, found : 283.1304.
IR ν [cm^{-1}] 2958, 2923, 1716, 1642, 1259, 1239, 1185, 1028.

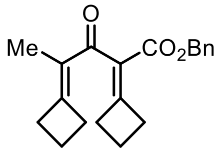

2y

Compound **2y** was synthesized following general procedure C, yellow oil, yield 50% (77% brsm).
^{1}H NMR (500 MHz, $CDCl_3$) δ 7.36 – 7.28 (m, 5H), 5.16 (s, 2H), 3.18 – 3.14 (m, 2H), 2.87 – 2.79 (m, 2H), 2.79 – 2.73 (m, 4H), 2.09 (p, J = 8.0 Hz, 2H), 1.90 (p, J = 8.0 Hz, 2H), 1.67 – 1.66 (m, 3H).
^{13}C NMR (125 MHz, $CDCl_3$) δ 192.6, 167.0, 163.6, 160.1, 135.9, 129.7, 128.6, 128.3, 127.9, 127.9, 65.7, 34.2, 32.9, 31.5, 31.5, 17.3, 16.2, 12.1.
HRMS (ESI) calcd for $[M+Na]^+$ $C_{20}H_{22}NaO_3$, m/z : 333.1461, found : 333.1461.
IR ν [cm^{-1}] 2956, 2922, 1713, 1640, 1259, 1239, 1024, 740, 698.

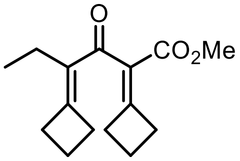

2z

Compound **2z** was synthesized following general procedure C, yellow oil, yield 50% (81% brsm).
^{1}H NMR (500 MHz, $CDCl_3$) δ 3.63 (s, 3H), 3.12 – 3.06 (m, 2H), 2.80 (q, J = 7.5 Hz, 4H), 2.76 – 2.71 (m, 2H), 2.11 (q, J = 7.5 Hz, 2H), 2.07 – 1.99 (m, 2H), 1.95 (p, J = 8.0 Hz, 2H), 0.87 (t, J = 7.5 Hz, 3H).

^{13}C NMR (125 MHz, $CDCl_3$) δ 192.3, 166.0, 164.4, 160.0, 136.1, 128.6, 51.2, 34.1, 33.1, 31.3, 31.3, 20.1, 17.3, 16.3, 13.1.
HRMS (ESI) calcd for $[M+Na]^+$ $C_{15}H_{20}NaO_3$, m/z: 271.1305 , found: 271.1309.
IR *ν* [cm^{-1}] 2960, 2875, 1717, 1642, 1436, 1242, 1039, 797.

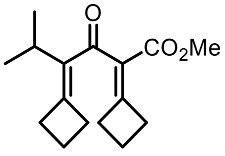

2aa

Compound **2aa** was synthesized following general procedure C, yellow oil, yield 70% (80% brsm).
^{1}H NMR (500 MHz, $CDCl_3$) δ 3.63 (s, 3H), 3.09 (dd, *J* = 8.0, 8.0 Hz, 2H), 2.86 (dd, *J* = 8.0, 8.0 Hz, 2H), 2.82 – 2.72 (m, 4H), 2.64 – 2.55 (m, 1H), 2.03 (p, *J* = 8.0 Hz, 2H), 1.92 (p, *J* = 8.0 Hz, 2H), 1.07 (s, 3H), 1.06 (s, 3H).
^{13}C NMR (125 MHz, $CDCl_3$) δ 193.0, 166.8, 164.7, 157.4, 139.2, 129.1, 51.2, 34.1, 33.4, 31.7, 31.6, 29.0, 20.6, 17.3, 16.5.
HRMS (ESI) calcd for $[M+Na]^+$ $C_{16}H_{22}NaO_3$, m/z: 285.1461 , found: 285.1471.
IR *ν* [cm^{-1}] 2958, 2876, 1747, 1664, 1439, 1321, 1104, 1023, 810.

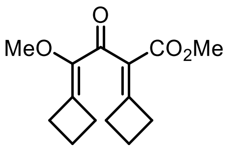

2bb

Compound **2bb** was synthesized following general procedure A and general procedure C, yellow oil, yield 24%.
^{1}H NMR (500 MHz, $CDCl_3$) δ 3.72 (s, 3H), 3.59 (s, 3H), 3.17 – 3.14 (m, 2H), 3.04 – 2.94 (m, 6H), 2.15 – 2.06 (m, 4H).
^{13}C NMR (125 MHz, $CDCl_3$) δ 188.6, 169.6, 165.0, 146.3, 143.7, 127.0, 59.2, 51.4, 33.9, 32.2, 31.8, 29.9, 18.0, 17.5.
HRMS (ESI) calcd for $[M+Na]^+$ $C_{14}H_{18}NaO_4$, m/z : 273.1097, found : 273.1097.
IR *ν* [cm^{-1}] 2954, 1721, 1662, 1435, 1284, 1265, 1243, 1104, 1041.
Note that: Compound ketone **III-2ee** and enol forms **III-2ee’** was synthesized following general procedure A and was unstable. So compound ketone **III-2ee** and enol forms **III-2ee’** was purified by a short silica gel (EtOAc: petroleum ether = 1:15) and was taken with condensation reaction using general procedure C to give compound **2ee**.

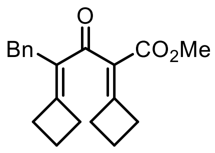

2cc

Compound **2cc** was synthesized following general procedure C, yellow oil, yield 87%.
1H NMR (500 MHz, $CDCl_3$) δ 7.23 (dd, J = 10.0, 5.0 Hz, 2H), 7.15 (dd, J = 14.0, 7.0 Hz, 3H), 3.65 (s, 3H), 3.54 (s, 2H), 3.15 – 3.09 (m, 2H), 2.93 (dd, J = 8.0, 8.0 Hz, 4H), 2.59 – 2.55 (m, 2H), 2.08 – 1.98 (m, 4H).
^{13}C NMR (125 MHz, $CDCl_3$) *δ* 192.2, 167.1, 164.4, 162.2, 140.1, 133.7, 128.6, 128.4, 128.1, 125.8, 51.4, 34.3, 33.5, 32.7, 32.1, 31.4, 17.5, 16.6.
HRMS (ESI) calcd for $[M+Na]^+$ $C_{20}H_{22}NaO_3$, m/z : 333.1461, found : 333.1461.
IR *ν* [cm^{-1}] 2955, 1744, 1716, 1641, 1261, 1242, 1037, 763, 750, 700.

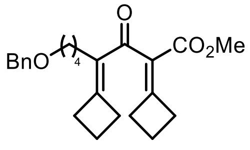

2dd

Compound **2dd** was synthesized following general procedure C, yellow oil, yield 54% (96% brsm).
^{1}H NMR (400 MHz, $CDCl_3$) *δ* 7.35 – 7.24 (m, 5H), 4.49 (s, 2H), 3.68 (s, 3H), 3.45 (dd, *J* = 6.4 Hz, 2H), 3.19 – 3.14 (m, 2H), 2.85 (t, *J* = 8.0 Hz, 4H), 2.82 – 2.78 (m, 2H), 2.21 – 2.17 (m, 2H), 2.09 (p, *J* = 8.0 Hz, 2H), 2.00 (p, *J* = 8.0 Hz, 2H), 1.64 – 1.57 (m, 2H), 1.46 – 1.38 (m, 2H).
^{13}C NMR (100 MHz, $CDCl_3$) *δ* 192.4, 166.4, 164.5, 161.0, 138.6, 134.7, 128.8, 128.3, 127.6, 127.4, 72.9, 70.3, 51.4, 34.3, 33.3, 31.8, 31.5, 29.6, 26.6, 25.2, 17.5, 16.4.
HRMS (ESI) calcd for $[M+Na]^+$ $C_{24}H_{30}NaO_4$, m/z : 405.2036, found : 405.2046.
IR *ν* [cm^{-1}] 2951, 2864, 1716, 1639, 1262, 1242, 1106, 742, 715, 699.

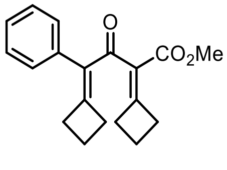

2ff

Compound **2ff** was synthesized following general procedure C, yellow oil, yield 72% (90% brsm).
^{1}H NMR (400 MHz, $CDCl_3$) *δ* 7.33 – 7.28 (m, 2H), 7.26 – 7.21 (m, 1H), 7.16 – 7.13 (m, 2H), 3.59 (s, 3H), 3.15 – 3.09 (m, 4H), 3.00 – 2.94 (m, 2H), 2.85 – 2.79 (m, 2H), 2.13 – 2.03 (m, 4H).
^{13}C NMR (100 MHz, $CDCl_3$) *δ* 192.0, 169.4, 164.7, 160.7, 135.8, 134.9, 129.1, 128.4, 127.9, 127.0, 51.3, 34.1, 33.7, 32.7, 32.4, 17.5, 17.2.

HRMS (ESI) calcd for $[M+Na]^+$ $C_{19}H_{20}NaO_3$, m/z : 319.1305, found : 319.1302.
IR *ν* [cm^{-1}] 2987, 2954, 2917, 1719, 1649, 1269, 1243, 1208, 1041, 745, 701.

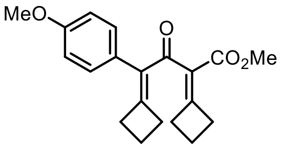

2gg

Compound **2gg** was synthesized following general procedure C, yellow oil, yield 65% (96% brsm).
^{1}H NMR (400 MHz, $CDCl_3$) δ 7.08 (d, J = 8.8 Hz, 2H), 6.84 (d, J = 8.8 Hz, 2H), 3.79 (s, 3H), 3.60 (s, 3H), 3.13 – 3.07 (m, 4H), 3.00 – 2.93 (m, 2H), 2.84 – 2.78 (m, 2H), 2.13 – 2.00 (m, 4H).
^{13}C NMR (100 MHz, $CDCl_3$) δ 192.3, 169.2, 164.7, 159.9, 158.5, 134.4, 130.2, 128.5, 128.1, 113.4, 55.1, 51.3, 34.1, 33.6, 32.7, 32.4, 17.5, 17.2.
HRMS (ESI) calcd for $[M+Na]^+$ $C_{20}H_{22}NaO_4$, m/z : 349.1410, found : 349.1411.
IR *ν* [cm^{-1}] 2954, 1715, 1645, 1607, 1510, 1242, 1175, 1033, 833, 805, 794.

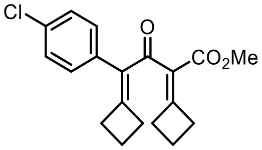

2hh

Compound **2hh** was synthesized following general procedure C, yellow oil, yield 69% (87% brsm)
^{1}H NMR (400 MHz, $CDCl_3$) δ 7.29 (d, J = 8.8 Hz, 2H), 7.11 (d, J = 8.8 Hz, 2H), 3.60 (s, 3H), 3.12 – 3.05 (m, 4H), 2.96 – 2.91 (m, 2H), 2.81 – 2.72 (m, 2H), 2.13 – 2.00 (m, 4H).
^{13}C NMR (100 MHz, $CDCl_3$) δ 191.6, 169.7, 164.6, 161.2, 134.2, 133.9, 132.8, 130.4, 128.2, 128.1, 51.3, 34.1, 33.6, 32.6, 32.3, 17.4, 17.1.
HRMS (ESI) calcd for $[M+Na]^+$ $C_{19}H_{19}ClNaO_3$, m/z : 353.0915, found : 353.0932.
IR *ν* [cm^{-1}] 2954, 1715, 1643, 1490, 1435, 1268, 1241, 1207, 1089, 1040, 1015, 828, 806, 790.

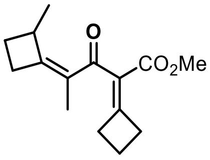

$4a_1$

Compound **$4a_1$** was synthesized following general procedure C, yellow oil, yield 7.5% (11% brsm) from the mixture of **81, 81'** and **82, 82'**. And compound **$4a_2$** was synthesized following general procedure C, yellow oil, yield 22%, (32% brsm) from the mixture of **81**, **81'** and **82**, **82'**. in one

pot.
^{1}H NMR (400 MHz, $CDCl_3$) δ 3.70 (s, 3H), 3.26 – 3.12 (m, 3H), 3.01 – 2.73 (m, 3H), 2.69 – 2.61 (m, 1H), 2.26 – 2.16 (m, 1H), 2.15 – 2.04 (m, 2H), 1.67 (d, J = 1.4 Hz, 3H), 1.57 – 1.52 (m, 1H), 1.19 (d, J = 7.2 Hz, 3H).
^{13}C NMR (100 MHz, $CDCl_3$) δ 193.3, 168.6, 164.9, 162.7, 128.7, 128.1, 51.4, 39.9, 34.2, 32.1, 28.9, 24.5, 19.8, 17.5, 13.3.
HRMS (ESI) calcd for $[M+Na]^+$ $C_{15}H_{20}NaO_3$, m/z : 271.1305, found : 271.1307.
IR ν [cm^{-1}] 2956, 1715, 1642, 1437, 1262, 1243, 1199, 1039.

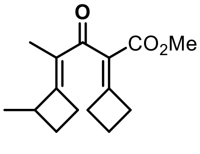

4a$_2$

Compound **4a$_1$** was synthesized following general procedure C, yellow oil, yield 7.5% (11% brsm) from the mixture of **81**, **81'** and **82**, **82’**. And compound **4a$_2$** was synthesized following general procedure C, yellow oil, yield 22% (32% brsm) from the mixture of **81**, **81'** and **82**, **82’** in one pot.
^{1}H NMR (400 MHz, $CDCl_3$) δ 3.70 (s, 3H), 3.19 – 3.14 (m, 3H), 2.94 – 2.83 (m, 1H), 2.82 – 2.67 (m, 3H), 2.26 – 2.16 (m, 1H), 2.16 – 2.06 (m, 2H), 1.72 (dd, J = 3.0, 2.0 Hz, 3H), 1.59 – 1.52 (m, 1H), 1.26 (d, J = 7.2 Hz, 3H).
^{13}C NMR (100 MHz, $CDCl_3$) δ 193.6, 166.5, 164.6, 164.4, 129.7, 128.5, 51.4, 38.9, 34.2, 31.6, 30.4, 24.6, 18.8, 17.5, 12.0.
HRMS (ESI) calcd for $[M+Na]^+$ $C_{15}H_{20}NaO_3$, m/z : 271.1305, found : 271.1304.
IR ν [cm^{-1}] 2955, 1715, 1640, 1436, 1261, 1241, 1195, 1037.

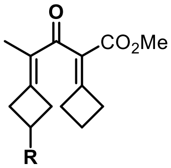

4c
R = SiPhMe$_2$

Compound **4c** was synthesized following general procedure C, yellow oil, yield 73% (93% brsm).
^{1}H NMR (500 MHz, $CDCl_3$) δ 7.49 – 7.47 (m, 2H), 7.39 – 7.34 (m, 3H), 3.68 (s, 3H), 3.17 – 3.13 (m, 2H), 3.06 – 2.99 (m, 2H), 2.78 – 2.62 (m, 4H), 2.12 – 2.03 (m, 2H), 1.96 – 1.89 (m, 1H), 1.64 (s, 3H), 0.27 (s, 3H), 0.26 (s, 3H).
^{13}C NMR (125 MHz, $CDCl_3$) δ 192.8, 166.5, 164.4, 160.1, 137.5, 133.7, 129.7, 129.2, 128.4, 127.8, 51.4, 34.3, 34.2, 33.0, 31.5, 17.5, 15.0, 12.0, -5.2, -5.4.
HRMS (ESI) calcd for $[M+Na]^+$ $C_{22}H_{28}NaO_3Si$, m/z : 391.1700, found : 391.1704.

IR v [cm^{-1}] 3069, 2953, 1747, 1716, 1653, 1488, 1428, 1329, 1250, 1114, 831, 811, 736, 701.

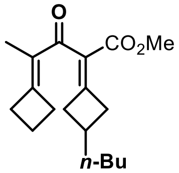

4d

Compound **4d** was synthesized following general procedure C, yellow oil, yield 80%.

^{1}H NMR (400 MHz, $CDCl_3$) δ 3.70 (s, 3H), 3.33 – 3.24 (m, 1H), 2.87 – 2.82 (m, 4H), 2.75 – 2.66 (m, 1H), 2.41 – 2.32 (m, 2H), 2.01 (p, J = 8.0 Hz, 2H), 1.68 – 1.67 (m, 3H), 1.51 – 1.19 (m, 7H), 0.88 (t, J = 7.2 Hz, 3H).

^{13}C NMR (100 MHz, $CDCl_3$) δ 193.1, 164.6, 163.9, 160.8, 129.7, 128.9, 51.4, 40.0, 37.3, 36.3, 33.3, 31.9, 31.1, 29.5, 22.5, 16.4, 14.1, 12.2.

HRMS (ESI) calcd for $[M+Na]^+$ $C_{18}H_{26}NaO_3$, m/z : 313.1774, found : 313.1772.

IR v [cm^{-1}] 2955, 2924, 2873, 2858, 1717, 1640, 1435, 1326, 1256, 1242, 1196.

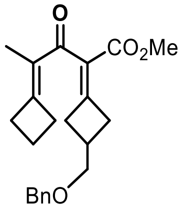

4e

Compound **4e** was synthesized following general procedure C, yellow oil, yield 77%.

^{1}H NMR (400 MHz, $CDCl_3$) δ 7.37 – 7.27 (m, 5H), 4.52 (s, 2H), 3.70 (s, 3H), 3.55 – 3.47 (m, 2H), 3.31 – 3.22 (m, 1H), 2.98 – 2.91 (m, 1H), 2.90 – 2.75 (m, 5H), 2.75 – 2.69 (m, 1H), 2.63 – 2.56 (m, 1H), 2.03 – 1.88 (m, 2H), 1.68 – 1.67 (m, 3H).

^{13}C NMR (100 MHz, $CDCl_3$) δ 192.8, 164.5, 163.0, 161.2, 138.2, 129.7, 129.4, 128.4, 127.6, 127.6, 73.3, 73.2, 51.5, 36.9, 34.3, 33.3, 31.9, 30.7, 16.4, 12.2.

HRMS (ESI) calcd for $[M+Na]^+$ $C_{22}H_{26}NaO_4$, m/z : 377.1723, found : 377.1728.

IR v [cm^{-1}] 2952, 2924, 2859, 1716, 1639, 1453, 1436, 1329, 1258, 1243, 1110, 740, 715, 699.

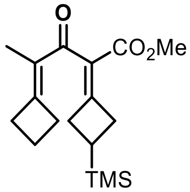

4f

Compound **4f** was synthesized following general procedure C, yellow oil, yield 22%.

^{1}H NMR (400 MHz, $CDCl_3$) δ 3.69 (s, 3H), 3.26 (dddd, J = 18.8, 10.4, 4.2, 1.2 Hz, 1H), 3.03 – 2.94 (m, 1H), 2.93 – 2.83 (m, 5H), 2.60 (ddd, J = 18.0, 7.6, 4.2 Hz, 1H), 2.01 (p, J = 8.0 Hz, 2H), 1.82 – 1.73 (m, 1H), 1.68 – 1.72 (m, 3H), 0.00 (m, 9H).
^{13}C NMR (100 MHz, $CDCl_3$) δ 192.9, 166.5, 164.6, 160.7, 129.7, 128.3, 51.4, 35.1, 33.2, 32.3, 31.9, 17.1, 16.4, 12.2, -3.9.
HRMS (ESI) calcd for $[M+Na]^+$ $C_{17}H_{26}NaO_3Si$, m/z : 329.1543, found : 329.1547.
IR ν [cm^{-1}] 2952, 1718, 1643, 1435, 1325, 1259, 1042, 837, 744.

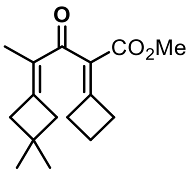

4g

Compound **4g** was synthesized following general procedure C, yellow oil, yield 40% (86% brsm).
^{1}H NMR (500 MHz, $CDCl_3$) δ 3.68 (s, 3H), 3.16 (ss, J = 8.4, 8.4 Hz, 2H), 2.77 (dd, J = 8.4, 8.0 Hz, 2H), 2.54 (s, 4H), 2.09 (p, J = 8.0 Hz, 2H), 1.68 – 1.67 (m, 3H), 1.15 (s, 6H).
^{13}C NMR (125 MHz, $CDCl_3$) δ 193.0, 166.3, 164.5, 155.7, 131.3, 128.6, 51.4, 46.4, 44.9, 34.2, 31.5, 31.1, 28.8, 17.5, 12.5.
HRMS (ESI) calcd for $[M+Na]^+$ $C_{16}H_{22}NaO_3$, m/z : 285.1461, found : 285.1461.
IR ν [cm^{-1}] 2952, 2925, 1717, 1643, 1435, 1324, 1260, 1241, 1194, 1040.

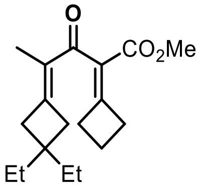

4h

Compound **4h** was synthesized following general procedure C, yellow oil, yield 62% (91% brsm).
^{1}H NMR (400 MHz, $CDCl_3$) δ 3.69 (s, 3H), 3.17 (dd, J = 8.0, 7.6 Hz, 2H), 2.77 (dd, J = 8.0, 7.6 Hz, 2H), 2.47 – 2.46 (m, 4H), 2.10 (p, J = 8.0 Hz, 2H), 1.69 (s, 3H), 1.45 (q, J = 7.6 Hz, 4H), 0.79 (t, J = 7.6 Hz, 6H).
^{13}C NMR (100 MHz, $CDCl_3$) δ 193.0, 166.3, 164.5, 156.8, 131.1, 128.6, 51.4, 42.8, 41.3, 37.8, 34.3, 31.5, 29.8, 17.5, 12.5, 8.3.
HRMS (ESI) calcd for $[M+Na]^+$ $C_{18}H_{26}NaO_3$, m/z : 313.1774, found : 313.1774.
IR ν [cm^{-1}] 2962, 2938, 1717, 1642, 1458, 1436, 1261, 1242, 1198, 1039.

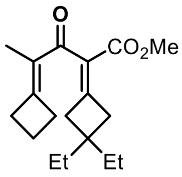

4i

Compound **4i** was synthesized following general procedure C, yellow oil, yield 42% (87% brsm).

^{1}H NMR (400 MHz, $CDCl_3$) δ 3.70 (s, 3H), 2.88 – 2.81 (m, 6H), 2.42 (dd, J = 3.2, 2.0 Hz, 2H), 2.01 (p, J = 8.0 Hz, 2H), 1.69 – 1.68 (m, 3H), 1.48 (q, J = 7.4 Hz, 4H), 0.79 (t, J = 7.4 Hz, 6H).

^{13}C NMR (100 MHz, $CDCl_3$) δ 193.2, 164.6, 162.9, 160.5, 130.2, 129.7, 51.4, 43.9, 41.1, 39.4, 33.3, 31.9, 29.8, 16.4, 12.3, 8.3.

HRMS (ESI) calcd for $[M+Na]^+$ $C_{18}H_{26}NaO_3$, m/z : 313.1774, found : 313.1773.

IR ν [cm^{-1}] 2961, 2917, 2877, 2857, 1716, 1664, 1642, 1434, 1322, 1258, 1241, 1201.

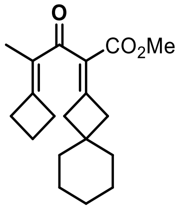

4j

Compound **4j** was synthesized following general procedure C, yellow oil, yield 55% (82% brsm).

^{1}H NMR (400 MHz, $CDCl_3$) δ 3.70 (s, 3H), 2.87 – 2.83 (m, 6H), 2.45 – 2.43 (m, 2H), 2.05 – 1.97 (m, 2H), 1.68 – 1.67 (m, 3H), 1.54 – 1.28 (m, 10H).

^{13}C NMR (100 MHz, $CDCl_3$) δ 193.1, 164.6, 162.8, 160.6, 130.2, 129.7, 51.4, 45.2, 42.5, 37.8, 36.8, 33.3, 31.8, 25.6, 23.1, 16.4, 12.2.

HRMS (ESI) calcd for $[M+Na]^+$ $C_{19}H_{26}NaO_3$, m/z : 325.1774, found : 325.1775.

IR ν [cm^{-1}] 2922, 2852, 1714, 1662, 1641, 1446, 1436, 1324, 1254, 1241, 1199, 1021.

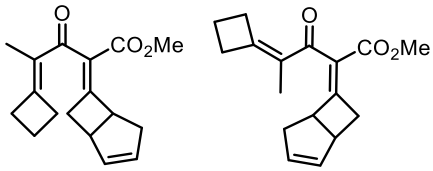

4l

Compound **4l** was synthesized following general procedure C, yellow oil, yield 22% (56% brsm).

^{1}H NMR (400 MHz, $CDCl_3$) δ 5.82 – 5.69 (m, 2H), 3.96 – 3.88 (m, 0.6H), 3.73 – 3.67 (m, 3H), 3.66 – 3.62 (m, 0.29H), 3.45 – 3.34 (m, 1H), 3.32 – 3.24 (m, 0.34H), 3.05 – 2.96 (m, 0.72H), 2.96 – 2.75 (m, 5H), 2.71 – 2.47 (m, 1H), 2.44 – 2.33 (m,1H), 2.05 – 1.94 (m, 2H), 1.73 – 1.63 (m, 3H).

^{13}C NMR (100 MHz, $CDCl_3$) δ 193.0, 193.0, 170.1, 169.1, 164.7, 163.9, 161.2, 161.1, 133.4, 133.0, 131.9, 131.3, 131.2, 130.8, 129.9, 129.7, 51.5, 51.4, 47.5, 45.5, 43.2, 42.3, 41.6, 38.8, 38.4, 37.7, 33.3, 33.3, 31.9, 31.8, 16.4, 16.3, 12.3, 12.1.
HRMS (ESI) calcd for $[M+Na]^+$ $C_{17}H_{20}NaO_3$, m/z : 295.1305, found : 295.1308.
IR ν [cm^{-1}] 2953, 2943, 2919, 1705, 1659, 1632, 1436, 1336, 1285, 1258, 1153, 1003.

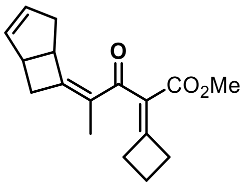

4m$_1$

Compound **4m$_1$** was synthesized following general procedure C, yellow oil, yield 12% (21% brsm) and compound **4m$_2$** was synthesized following general procedure C, yellow oil, yield 32%, (56% brsm) from **85** and **85'** in one pot.
Compound **4m$_1$** was synthesized following general procedure C, yellow oil, yield 11% (20% brsm) and compound **4m$_2$** was synthesized following general procedure C, yellow oil, yield 27% (49% brsm) from **86** and **86'** in one pot.
^{1}H NMR (400 MHz, $CDCl_3$) δ 5.80 – 5.75 (m, 1H), 5.75 – 5.69 (m, 1H), 3.71 (s, 3H), 3.68 – 3.60 (m, 1H), 3.34 – 3.26 (m, 1H), 3.26 – 3.09 (m, 2H), 3.06 – 2.92 (m, 2H), 2.82 – 2.63 (m, 2H), 2.54 – 2.44 (m, 1H), 2.39 – 2.32 (m, 1H), 2.17 – 2.05 (m, 2H), 1.65 (d, J = 1.6 Hz, 3H).
^{13}C NMR (100 MHz, $CDCl_3$) δ 192.9, 168.5, 164.8, 162.5, 133.2, 131.5, 131.1, 128.2, 51.4, 46.3, 41.5, 39.8, 39.2, 34.2, 32.1, 17.5, 13.0.
HRMS (ESI) calcd for $[M+Na]^+$ $C_{17}H_{20}NaO_3$, m/z : 295.1305, found : 295.1305.
IR ν [cm^{-1}] 2951, 2927, 1716, 1659, 1639, 1436, 1321, 1261, 1242, 1040.

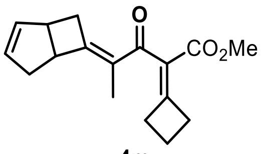

4m$_2$

Compound **4m$_1$** was synthesized following general procedure C, yellow oil, yield 12% (21% brsm) and compound **4m$_2$** was synthesized following general procedure C, yellow oil, yield 32% (56% brsm) from **85** and **85'** in one pot.
Compound **4m$_1$** was synthesized following general procedure C, yellow oil, yield 11% (20% brsm) and compound **4m$_2$** was synthesized following general procedure C, yellow oil, yield 27% (49% brsm) from **86** and **86'** in one pot.

^{1}H NMR (400 MHz, $CDCl_3$) δ 5.77 (s, 2H), 3.66 (s, 3H), 3.65 – 3.58 (m, 1H), 3.37 – 3.26 (m, 1H), 3.18 – 3.09 (m, 2H), 3.07 – 2.94 (m, 1H), 2.79 – 2.63 (m, 3H), 2.57 – 2.45 (m, 2H), 2.15 – 2.00 (m, 2H), 1.75 (dd, J = 3.2, 1.6 Hz, 3H).
^{13}C NMR (100 MHz, $CDCl_3$) δ 193.4, 166.7, 164.5, 163.2, 133.2, 132.1, 131.3, 128.4, 51.4, 45.2, 41.7, 40.5, 37.8, 34.2, 31.5, 17.4, 13.0.
HRMS (ESI) calcd for $[M+Na]^+$ $C_{17}H_{20}NaO_3$, m/z : 295.1305, found : 295.1311.
IR ν [cm^{-1}] 2951, 2921, 1714, 1657, 1640, 1435, 1324, 1259, 1241, 1038.

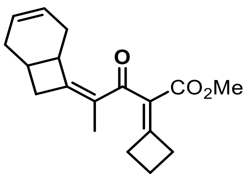

4n$_1$

Compound **4n$_1$** was synthesized following general procedure C, yellow oil, yield 19% (31% brsm) and compound **4n$_2$** was synthesized following general procedure C, yellow oil, yield 35% (58% brsm) from **87** and **87'** in one pot.
Compound **4n$_1$** was synthesized following general procedure C, yellow oil, yield 8% (12% brsm) and compound **4n$_2$** was synthesized following general procedure C, yellow oil, yield 19% (30% brsm) from **88** and **88'** in one pot.
^{1}H NMR (400 MHz, $CDCl_3$) δ 5.92 – 5.75 (m, 2H), 3.70 (s, 3H), 3.41 – 3.28 (m, 1H), 3.26 – 3.06 (m, 2H), 3.02 – 2.85 (m, 1H), 2.84 – 2.70 (m, 2H), 2.68 – 2.55 (m, 1H), 2.48 – 2.30 (m, 1H), 2.20 – 2.00 (m, 5H), 2.00 – 1.89 (m, 1H), 1.66 – 1.64 (m, 3H).
^{13}C NMR (100 MHz, $CDCl_3$) δ 193.3, 168.3, 164.9, 161.3, 128.6, 128.1, 128.0, 126.5, 51.4, 41.8, 36.1, 34.2, 32.0, 27.2, 27.1, 25.8, 17.5, 13.4.
HRMS (ESI) calcd for $[M+Na]^+$ $C_{18}H_{22}NaO_3$, m/z : 309.1461, found : 309.1459.
IR ν [cm^{-1}] 2951, 2921, 1715, 1640, 1436, 1318, 1261, 1243, 1038.

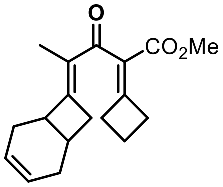

4n$_2$

Compound **4n$_1$** was synthesized following general procedure C, yellow oil, yield 19% (31% brsm) and compound **4n$_2$** was synthesized following general procedure C, yellow oil, yield 35% (58% brsm) from **87** and **87'** in one pot.
Compound **4n$_1$** was synthesized following general procedure C, yellow oil, yield 8% (12% brsm)

and compound **4n₂** was synthesized following general procedure C, yellow oil, yield 19% (30% brsm) from **88** and **88'** in one pot.

^{1}H NMR (400 MHz, $CDCl_3$) δ 6.01 – 5.77 (m, 2H), 3.67 (s, 3H), 3.38 – 3.25 (m, 1H), 3.25 – 3.05 (m, 2H), 2.89 – 2.59 (m, 4H), 2.44 – 2.31 (m, 1H), 2.32 – 2.16 (m, 2H), 2.17 – 1.93 (m, 4H), 1.77 – 1.64 (m, 3H).

^{13}C NMR (100 MHz, $CDCl_3$) δ 193.5, 166.6, 164.5, 162.7, 129.9, 128.4, 127.9, 127.1, 51.4, 41.4, 37.7, 34.2, 31.5, 27.3, 27.1, 25.0, 17.5, 12.2.

HRMS (ESI) calcd for $[M+Na]^+$ $C_{18}H_{22}NaO_3$, m/z : 309.1461, found : 309.1462.

IR ν [cm^{-1}] 2949, 1717, 1640, 1434, 1325, 1258, 1241, 1041.

Mp 55.7 – 56.9 °C.

3.31 General procedure D for synthesis starting material 4b.

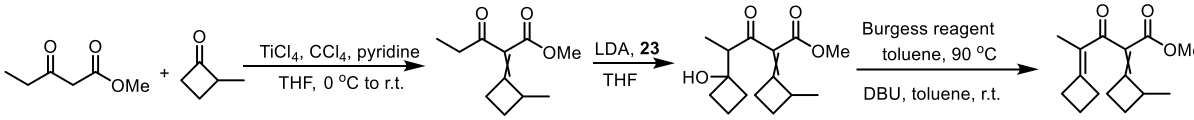

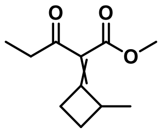

104

Compound **104**. To a solution of CCl_4 (1.9 mL, 20.0 mmol, 2.0 equiv) in 40 mL THF was added $TiCl_4$ (2.2 mL, 20.0 mmol, 2.0 equiv) dropwise at 0 ºC. The mixture was stirred at the same temperature for 30 minutes before the solution of methyl 3-oxopentanoate **102** [6] (1.3 mL, 10.0 mmol, 1.0 equiv) was added to the flask. Then 2-methylcyclobutan-1-one **103** (1.3 mL, 15.0 mmol, 1.5 equiv) was added to the mixture 5 minutes later and stirred for another 30 minutes at 0 ºC. In the end, pyridine (3.2 mL, 40.0 mmol, 4.0 equiv) was added to the reaction flask at 0 ºC and the reaction mixture was stirred at room temperature overnight. The reaction was determined by TLC until the methyl 3-oxopentanoate transformed completely. The mixture was quenched by cold water at 0 ºC. Then, the solution was extracted with EtOAc (3 x 80 mL). Then saturated Na_2CO_3 solution was added to the concentrated organic layers for three times, subsequently the organic phase was washed by saturated $CuSO_4$ solution and brine for three times respectively. The organic phase was concentrated, dried over Na_2SO_4, and concentrated under reduced pressure and purified by flash chromatography on silica gel (EtOAc: petroleum ether = 1:30) to give the corresponding ene ester product **104** (1.5g, 7.4mmol, 74%) as a yellow oil.

^{1}H NMR (400 MHz, $CDCl_3$) δ 3.73 (s, 1.64H), 3.71 (s, 1.40H), 3.43 – 3.32 (m, 1H), 3.24 – 3.09 (m, 1H), 3.01 – 2.88 (m, 1H), 2.77 – 2.48 (m, 2H), 2.32 – 2.20 (m, 1H), 1.69 – 1.59 (m, 1H), 1.22 (d, J = 7.2 Hz, 1.71H), 1.16 (d, J = 7.2 Hz, 1.47H), 1.06 – 1.01 (m, 3H).
^{13}C NMR (100 MHz, $CDCl_3$) δ 201.7, 200.2, 176.8, 176.7, 165.9, 165.6, 127.3, 127.1, 51.5, 51.4, 40.9, 40.4, 35.9, 35.3, 31.5, 31.4, 25.3, 25.2, 18.6, 18.6, 8.0, 7.8.
HRMS (ESI) calcd for $[M+Na]^+$ $C_{11}H_{16}NaO_3$, m/z : 219.0992, found : 219.0994.
IR ν [cm^{-1}] 2977, 2953, 1710, 1692, 1629, 1435, 1238, 1121, 1036.

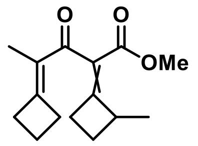

$4b_1$ and $4b_2$

Compound **$4b_1$** and **$4b_2$**. LDA was prepared in situ by adding *n*-BuLi (4.8 mL, 12.0 mmol, 2.5 M in hexane, 6.0 equiv) to a solution of di*iso*propylamine (1.7 mL, 12.0 mmol, 6.0 equiv) in THF (12 mL) and stirred for 30 minutes at 0 ºC. Then the flask was colded to -78 ºC and stirred for 10 minutes, subsequently, starting materials **104** (0.4 g, 2.0 mmol, 1.0 equiv) was dissolved in THF (4 mL) and added dropwise to LDA solution. The mixture was stirred at -78 ºC for 5 minutes and moved to 0 ºC stirring for another 1 hour. Then the reaction flask was colded to -78 ºC again and stirred for 10 minutes before cyclobutanone **23** (0.5 mL, 6.0 mmol, 3.0 equiv) was added to the mixture. The reaction mixture was stirred at -78 ºC for 30 minutes and determined by TLC until the majority of starting materials **104** transformed. Then the reaction mixture was quenched by saturated NH_4Cl solution and extracted with EtOAc (3 x 50 mL). The organic phase was concentrated, dried over Na_2SO_4, and evaporated under reduced pressure. The crude product was purified by a short flash column chromatography (EtOAc: petroleum ether = 1:10) to give crude product **105**.
To a solution of compound **105** in toluene (10 mL) was added burgess reagent (1.4 g, 6.0 mmol, 3.0 equiv) and stirred at 90 ºC for an hour until starting materials **105** transformed completely (determined by TLC). Then, the mixture was cooled to room temperature and DBU (0.9 mL, 6.0 mmol, 3.0 equiv) was added to the reaction mixture. Ten minutes later, the reaction was determined by TLC until complete isomerization of intermediate product. Then the transformation was quenched by water and extracted with EtOAc (3 x 20 mL). The organic phase was concentrated, dried over Na_2SO_4, and evaporated under reduced pressure to give crude product, which was purified by column chromatography (EtOAc: petroleum ether = 1:50) to give **$4b_1$** (low polarity) and **$4b_2$** (high polarity) (159mg, 0.64mmol, 32% for three steps) as yellow oils.
Note that: The E/Z of **$4b_1$** and **$4b_2$** were unidentified.

Compound **4b₁** (low polarity)

^{1}H NMR (400 MHz, $CDCl_3$) δ 3.70 (s, 3H), 3.55 – 3.38 (m, 1H), 3.01 – 2.77 (m, 5H), 2.68 – 2.53 (m, 1H), 2.35 – 2.23 (m, 1H), 2.02 (p, J = 8.0 Hz, 2H), 1.71 – 1.67 (m, 3H), 1.67 – 1.61 (m, 1H), 1.34 (d, J = 7.2 Hz, 3H).

^{13}C NMR (100 MHz, $CDCl_3$) δ 193.1, 169.5, 164.0, 160.7, 129.8, 128.7, 51.4, 41.0, 33.5, 31.9, 28.6, 25.0, 18.4, 16.4, 12.2.

HRMS (ESI) calcd for $[M+Na]^+$ $C_{15}H_{20}NaO_3$, m/z : 271.1305, found : 271.1309.

IR ν [cm^{-1}]2954, 1715, 1640, 1436, 1257, 1240, 1065.

Compound **4b₂** (high polarity)

^{1}H NMR (400 MHz, $CDCl_3$) δ 3.69 (s, 3H), 3.22-3.10 (m, 2H), 3.06-2.94 (m, 1H), 2.94-2.78 (m, 4H), 2.31-2.20 (m, 1H), 2.06-1.95 (m, 2H), 1.69 (m, 3H), 1.67-1.65 (m, 1H), 1.07 (d, J = 7.2 Hz, 3H).

^{13}C NMR (100 MHz, $CDCl_3$) δ 193.0, 170.2, 164.8, 161.4, 130.1, 128.4, 51.5, 39.2, 33.5, 31.9, 31.2, 25.5, 18.4, 16.4, 12.3.

HRMS (ESI) calcd for $[M+Na]^+$ $C_{15}H_{20}NaO_3$, m/z : 271.1305, found : 271.1305.

IR ν [cm^{-1}]2956, 1716, 1590, 1439, 1258, 1242.

3.32 General procedure D for synthesis starting material 4k.

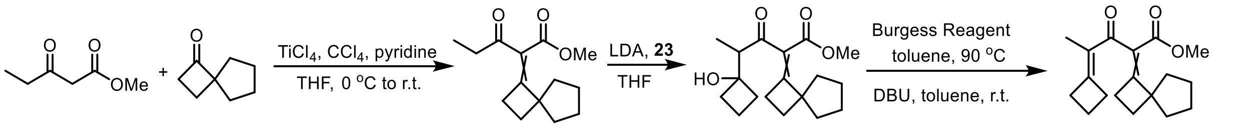

102 106 107 108 $4k_1$ and $4k_2$

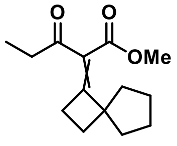

107

Compound **107.** To a solution of CCl_4 (1.9 mL, 20.0 mmol, 2.0 equiv) in THF (40 mL) was added $TiCl_4$ (2.2 mL, 20.0 mmol, 2.0 equiv) dropwise at 0 ºC. The mixture was stirred at the same temperature for 30 minutes before the solution of methyl 3-oxopentanoate **102** (1.3mL, 10.0 mmol, 1.0 equiv) was added to the flask. Then spiro [3.4] octan-1-one **106** [7] (1.9 g, 15.0 mmol, 1.5 equiv) was added to the mixture and stirred for another 30 minutes at 0 ºC. Then pyridine (3.2 mL, 40.0 mmol, 4.0 equiv) was added to the reaction flask at 0 ºC and the reaction mixture was stirred at room temperature overnight. The reaction was determined by TLC until the methyl 3-oxopentanoate transformed completely. The mixture was quenched by cold water at 0 ºC. Then, the solution was extracted with EtOAc (3 x 80 mL). After that, saturated Na_2CO_3 solution was added to the concentrated organic layers for three times, subsequently the organic phase was

washed by saturated $CuSO_4$ solution and saturated brine for three times respectively. The organic phase was concentrated, dried over Na_2SO_4, and concentrated under reduced pressure and purified by flash chromatography on silica gel (EtOAc: petroleum ether = 1:30) to give the mixture of **107-1** (low polarity) and **107-2** (high polarity) (0.9 g, 3.9 mmol, 39%) as yellow oils.
Note that: The E/Z of **107-1** and **107-2** were unidentified.

Compound **107-1.** (low polarity)
^{1}H NMR (500 MHz, $CDCl_3$) δ 3.74 (s, 3H), 3.04 (t, J = 8.0 Hz, 2H), 2.67 (q, J = 7.5 Hz, 2H), 1.92 – 1.83 (m, 4H), 1.73 – 1.64 (m, 4H), 1.61 – 1.56 (m, 2H), 1.09 (t, J = 7.5 Hz, 3H).
^{13}C NMR (125 MHz, $CDCl_3$) δ 203.5, 176.3, 165.5, 127.6, 56.8, 51.4, 38.7, 36.4, 34.1, 29.8, 24.2, 8.0.
HRMS (ESI) calcd for $[M+Na]^+$ $C_{14}H_{20}NaO_3$, m/z : 259.1305, found : 259.1304.
IR v [cm^{-1}]2951, 2870, 1724, 1701, 1434, 1302, 1254, 1231, 1092.

Compound **107-2.** (high polarity)
^{1}H NMR (500 MHz, $CDCl_3$) δ 3.74 (s, 3H), 3.01 (t, J = 8.0 Hz, 2H), 2.55 (q, J = 7.5 Hz, 2H), 2.00 – 1.94 (m, 2H), 1.92 (t, J = 8.5 Hz, 2H), 1.74 – 1.68 (m, 2H), 1.67 – 1.61 (m, 2H), 1.61 – 1.53 (m, 2H), 1.04 (t, J = 7.0 Hz, 3H).
^{13}C NMR (125 MHz, $CDCl_3$) δ 199.4, 175.6, 166.7, 127.5, 56.9, 51.7, 38.4, 35.0, 34.9, 29.9, 24.7, 7.8.
HRMS (ESI) calcd for $[M+Na]^+$ $C_{14}H_{20}NaO_3$, m/z : 259.1305, found : 259.1303.
IR v [cm^{-1}]2949, 2871, 1726, 1696, 1435, 1291, 1240, 1199, 1036.

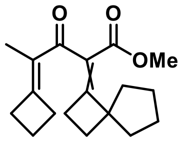

$4k_1$ and $4k_2$

Compound **$4k_1$ and $4k_2$.** LDA was prepared in situ by adding *n*-BuLi (4.8 mL, 2.5 M in hexane, 12.0 mmol, 6.0 equiv) to a solution of di*iso*propylamine (1.7 mL, 12.0 mmol, 6.0 equiv) in 40mL THF and stirred for 30 minutes at 0 ºC. Then the flask was moved into -78 ºC and stirred for 10 minutes subsequently. The mixture of the starting materials **107-1** and **107-2** (470 mg, 2.0 mmol, 1.0 equiv) was dissolved in THF (5 mL) and added dropwise to LDA solution. The mixture was stirred at -78 ºC for 5 minutes and moved to 0 ºC for another 1 hour. Then the reaction flask was moved into -78 ºC and stirred for 10 minutes before cyclobutanone **23** (0.5ml, 6.0 mmol, 3.0 equiv) was added to the mixture. The reaction mixture was stirred at -78 ºC for 30 minutes and determined by TLC until the majority of starting materials transformed. Then the reaction was quenched by

saturated NH_4Cl solution and extracted with EtOAc (3 x 60 mL). The organic phase was concentrated, dried over Na_2SO_4, and evaporated under reduced pressure.The crude product was purified by a short flash column chromatography (EtOAc: petroleum ether = 1:10) to give crude product **108**.

To a solution of compound **108** in toluene (10 mL) was added burgess reagent (1.4 g, 6.0 mmol, 3.0 equiv) and stirred at 80 ºC for an hour until starting materials **108** transformed completely (determined by TLC). Then, the mixture was cooled to room temperature and DBU (0.9 mL, 6.0 mmol, 3.0 equiv) was added to the reaction mixture. Ten minutes later, the reaction was determined by TLC until complete isomerization of intermediate product. Then the transformation was quenched by water and extracted with EtOAc (3 x 20 mL). The organic phase was concentrated, dried over Na_2SO_4, and evaporated under reduced pressure to give crude product, which was purified by column chromatography (EtOAc: petroleum ether = 1:50) to give **4k$_1$** (low polarity) and **4k$_2$** (high polarity) as yellow oils in 58% yield for three steps.

Note that: The E/Z of **4k$_1$** and **4k$_2$** were unidentified.

Compound **4k$_1$** (low polarity)

^{1}H NMR (500 MHz, $CDCl_3$) δ 3.68 (s, 3H), 2.92 – 2.81 (m, 4H), 2.61 (t, J = 8.5 Hz, 2H), 2.31–2.22 (m, 2H), 2.05 – 1.97 (m, 2H), 1.91 (t, J = 8.0 Hz, 2H), 1.79 – 1.70 (m, 3H), 1.69 – 1.67 (m, 3H), 1.67 – 1.58 (m, 3H).

^{13}C NMR (125 MHz, $CDCl_3$) δ 193.3, 171.4, 163.6, 160.7, 129.8, 128.8, 57.0, 51.5, 37.6, 34.7, 33.5, 31.9, 26.5, 24.8, 16.4, 12.2.

HRMS (ESI) calcd for $[M+Na]^+$ $C_{18}H_{24}NaO_3$, m/z : 311.1618, found : 311.1621.

IR ν [cm^{-1}] 2951, 2868, 1720, 1644, 1434, 1319, 1240, 1213, 1193, 1048.

Compound **4k$_2$** (high polarity)

^{1}H NMR (500 MHz, $CDCl_3$) δ 3.68 (s, 3H), 3.06 (t, J = 8.0 Hz, 2H), 2.94 – 2.80 (m, 4H), 2.05 – 1.98 (m, 2H), 1.89 (t, J = 8.0 Hz, 2H), 1.76 – 1.70 (m, 4H), 1.69 (s, 3H), 1.61 – 1.50 (m, 4H).

^{13}C NMR (125 MHz, $CDCl_3$) δ 193.4, 172.0, 164.9, 161.7, 130.5, 128.1, 56.3, 51.4, 38.4, 34.0, 33.7, 31.9, 30.0, 23.9, 16.4, 12.3.

HRMS (ESI) calcd for $[M+Na]^+$ $C_{18}H_{24}NaO_3$, m/z : 311.1618, found : 311.1615.

IR ν [cm^{-1}] 2951, 2869, 1718,1706, 1643, 1434, 1314, 1241, 1192, 1160, 1084.

4. General procedure E for the Nazarov cyclization and two ring expansions reaction

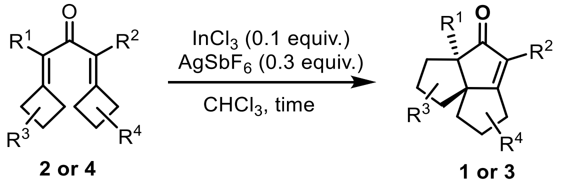

A flame-dried round-bottomed flask was placed in a glovebox and loaded with $InCl_3$ (4.4 mg, 0.02 mmol, 0.1 equiv) and $AgSbF_6$ (20.6 mg, 0.06 mmol, 0.3 equiv). The flask was removed from the glovebox, and freshly distilled chloroform (1 mL) was added to the mixture. Then **2** or **4** (0.2 mmol, 1.0 equiv) and chloroform (1 mL) were added to the mixture above (or a solution of **2** or **4** in chloroform was added to the mixture above when the **2** or **4** was an oil). The reaction mixture was reacted at the corresponding temperature for the corresponding time. The reaction was filtered through a pad of silica gel and washed with EtOAc. The filtrate was concentrated under reduced pressure and purified by flash column chromatography (EtOAc: petroleum ether = 1:5) to give the corresponding product **1** or **3**.

Method for purifying chloroform: $CHCl_3$ (100mL) was shaken (mechanically) with concentrated sulfuric acid (3 x 5 mL), washed thoroughly with water (3 x 20 mL), brine (3 x 20 mL), and dried over $CaCl_2$ (100 g) for overnight before filtering and distilling. The resulting clarified chloroform should be stored in a dark place and it can usually be stored for 2 weeks for the reaction.

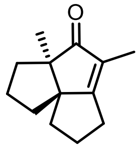

1a

Compound **1a** was synthesized following general procedure E, yellow oil, yield 95%. Reaction time: 0.5 h.

^{1}H NMR (400 MHz, $CDCl_3$) δ 2.58 – 2.38 (m, 2H), 2.09 – 1.98 (m, 3H), 1.77 (dd, *J* = 12.4, 5.6 Hz, 1H), 1.65 (s, 3H), 1.63 – 1.57 (m, 1H), 1.51 – 1.44 (m, 2H), 1.39 – 1.35 (m, 1H), 1.33 – 1.26 (m, 1H), 1.09 – 0.99 (m, 1H), 0.97 (s, 3H).

^{13}C NMR (100 MHz, $CDCl_3$): *δ* 216.0, 184.0, 130.1, 61.9, 56.7, 37.9, 36.9, 33.2, 24.0, 23.8, 22.4, 18.9, 8.6.

HRMS (ESI) calcd for $[M+Na]^+$ $C_{13}H_{18}NaO$, m/z : 213.1250, found : 213.1250.

IR *ν* [cm^{-1}] 2955, 2925, 2853, 1716, 1630, 1152.

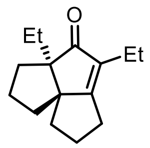

1b

Compound **1b** was synthesized following general procedure E, yellow oil, yield 91%. Reaction time: 0.5 h.

^{1}H NMR (500 MHz, $CDCl_3$): δ 2.57 – 2.46 (m, 2H), 2.22 – 2.14 (m, 1H), 2.11 – 2.05 (m, 1H), 2.04 – 1.95 (m, 3H), 1.77 (dd, J = 12.5, 6.5 Hz, 1H), 1.64 – 1.54 (m, 2H), 1.53 – 1.45 (m, 2H), 1.43 – 1.31 (m, 3H), 1.08 – 1.02 (m, 1H), 0.99 (t, J = 7.5 Hz, 3H), 0.90 (t, J = 7.5 Hz, 3H).

^{13}C NMR (125 MHz, $CDCl_3$): δ 215.4, 182.9, 136.1, 62.1, 61.1, 37.8, 34.0, 33.6, 25.6, 24.2, 23.8, 22.3, 17.1, 12.9, 9.3.

HRMS (ESI) calcd for $[M+Na]^+$ $C_{15}H_{22}NaO$, m/z : 241.1563, found : 241.1563.

IR ν [cm^{-1}] 2959, 2867, 1700, 1663, 1455, 1350, 1034, 843.

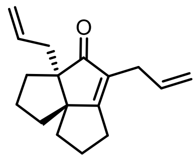

1c

Compound **1c** was synthesized following general procedure E, yellow oil, yield 72%. Reaction time: 5 h.

^{1}H NMR (500 MHz, $CDCl_3$) δ 5.84 – 5.76 (m, 2H), 5.06 – 4.95 (m, 4H), 2.98 – 2.82 (m, 2H), 2.52 – 2.49 (m, 2H), 2.25 (dd, J = 14.0, 7.5 Hz, 1H), 2.08 (dd, J = 14.5, 6.5 Hz, 1H), 2.05 – 1.99 (m, 2H), 1.96 – 1.92 (m, 1H), 1.80 (dd, J = 13.0, 7.0 Hz, 1H), 1.69 – 1.61 (m, 2H), 1.54 – 1.46 (m, 2H), 1.40 – 1.34 (m, 1H), 1.11 – 1.00 (m, 1H).

^{13}C NMR (125 MHz, $CDCl_3$) δ 214.1, 185.1, 134.8, 132.1, 117.5, 115.2, 62.4, 60.1, 37.5, 37.2, 34.6, 33.9, 28.1, 24.2, 23.9, 22.2.

HRMS (ESI) calcd for $[M+Na]^+$ $C_{17}H_{22}NaO$, m/z: 265.1563 , found: 265.1565.

IR ν [cm^{-1}] 3076, 2952, 2866, 1701, 1661, 1441, 995, 910.

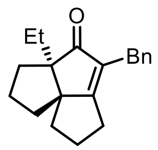

1d

Compound **1d** was synthesized following general procedure E, yellow oil, yield 81%. Reaction time: 0.5 h.

^{1}H NMR (500 MHz, $CDCl_3$): *δ* 7.25 – 7.14 (m, 5H), 3.56 – 3.38 (m, 2H), 2.38 – 2.24 (m, 2H), 2.04 – 1.93 (m, 3H), 1.79 – 1.75 (m, 1H), 1.63 – 1.55 (m, 2H), 1.54 – 1.48 (m, 2H), 1.47 – 1.42 (m, 1H), 1.40 – 1.33 (m, 2H), 1.08 – 0.98 (m, 1H), 0.92 (t, *J* = 7.5 Hz, 3H).
^{13}C NMR (125 MHz, $CDCl_3$): *δ* 215.0, 185.0, 139.6, 133.5, 128.5, 128.3, 125.8, 62.5, 61.1, 37.8, 34.1, 33.6, 30.0, 25.6, 24.2, 24.0, 22.4, 9.3.
HRMS (ESI) calcd for $[M+Na]^+$ $C_{20}H_{24}NaO$, m/z : 303.1719, found : 303.1719.
IR *ν* [cm^{-1}] 3061, 3027, 2953, 2864, 1700, 1660, 1494, 1452, 1349, 1030, 699.

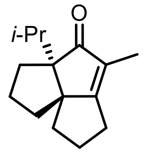

1e

Compound **1e** was synthesized following general procedure E, yellow oil, yield 80%. Reaction time: 0.5 h.
^{1}H NMR (500 MHz, $CDCl_3$): *δ* 2.53 – 2.42 (m, 2H), 2.06 – 1.99 (m, 3H), 1.86 – 1.74 (m, 3H), 1.65 (s, 3H), 1.63 – 1.60 (m, 1H), 1.50 – 1.44 (m, 2H), 1.38 – 1.31 (m, 1H), 1.03 – 0.97 (m, 1H), 0.94 (d, *J* = 7.0 Hz, 3H), 0.83 (d, *J* = 7.0 Hz, 3H).
^{13}C NMR (125 MHz, $CDCl_3$): *δ* 215.7, 183.7, 130.9, 64.9, 62.3, 38.8, 33.7, 33.7, 30.9, 24.3, 23.7, 22.2, 19.0, 18.8, 8.4.
HRMS (ESI) calcd for $[M+Na]^+$ $C_{15}H_{22}NaO$, m/z : 241.1563, found : 241.1563.
IR *ν* [cm^{-1}] 2956, 2874, 1702, 1668, 1467, 1385, 996.

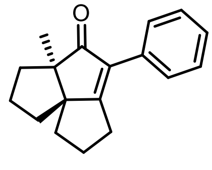

1f

Compound **1f** was synthesized following general procedure E, yellow oil, yield 75%. Reaction time: 0.5 h.
^{1}H NMR (500 MHz, $CDCl_3$) *δ* 7.60 – 7.59 (m, 2H), 7.38 (t, *J* = 7.5 Hz, 2H), 7.30 – 7.27 (m, 1H), 2.92 (ddd, *J* = 19.0, 11.0, 2.5 Hz, 1H), 2.65 (dt, *J* = 19.0, 8.0 Hz, 1H), 2.22 – 2.01 (m, 3H), 1.98 – 1.93 (m, 1H), 1.70 – 1.66 (m, 1H), 1.58 – 1.49 (m, 3H), 1.43 – 1.36 (m, 1H), 1.20 – 1.12 (m, 1H), 1.10 (s, 3H).
^{13}C NMR (125 MHz, $CDCl_3$) *δ* 213.8, 185.6, 132.7, 131.9, 128.3, 128.2, 127.6, 62.2, 57.8, 38.2, 37.6, 32.9, 26.2, 24.3, 22.6, 19.1.
HRMS (ESI) calcd for $[M+Na]^+$ $C_{18}H_{20}NaO$, m/z: 275.1406 , found: 275.1407.

IR v [cm^{-1}] 2956, 2876, 1745, 1712, 1645, 1436, 1348, 1226, 1024, 727.

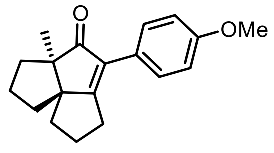

1g

Compound **1g** was synthesized following general procedure E, yellow oil, yield 77%. Reaction time: 0.5 h.

^{1}H NMR (500 MHz, $CDCl_3$) δ 7.58 – 7.55 (m, 2H), 6.93 – 6.90 (m, 2H), 3.82 (s, 3H), 2.92 – 2.86 (m, 1H), 2.67 – 2.60 (m, 1H), 2.20 – 2.15 (m, 1H), 2.14 – 2.07 (m, 1H), 2.05 – 2.01 (m, 1H), 1.94 – 1.91 (m, 1H), 1.69 – 1.66 (m, 1H), 1.56 – 1.46 (m, 3H), 1.41 – 1.35 (m, 1H), 1.17 – 1.12 (m, 1H), 1.09 (s, 3H).

^{13}C NMR (125 MHz, $CDCl_3$) δ 214.1, 183.8, 159.0, 132.1, 129.5, 124.6, 113.6, 62.1, 57.6, 55.3, 38.2, 37.5, 32.9, 26.2, 24.3, 22.6, 19.1.

HRMS (ESI) calcd for $[M+Na]^+$ $C_{19}H_{22}NaO_2$, m/z: 305.1512 , found: 305.1512.

IR v [cm^{-1}] 2954, 2864, 1695, 1607, 1512, 1250, 1178, 1034, 829.

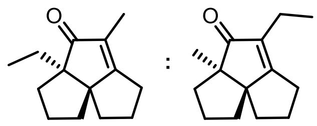

1h **1h'**

6.5 : 1

Compound **1h** and **1h’** was synthesized following general procedure E, yellow oil, yield 89%. Reaction time: 0.5 h.

^{1}H NMR (500 MHz, $CDCl_3$): δ 2.50 – 2.44 (m, 1H), 2.06 – 1.95 (m, 3H), 1.79 – 1.74 (m, 1H), 1.65 (s, 3H), 1.63 – 1.54 (m, 2H), 1.52 – 1.46 (m, 2H), 1.45 – 1.40 (m, 1H), 1.38 – 1.30 (m, 2H), 1.07 – 1.00 (m, 1.7H), 0.96 (s, 0.4H), 0.91 (t, J = 7.5 Hz, 3H).

^{13}C NMR (125 MHz, $CDCl_3$): δ 215.8, 215.5, 183.6, 183.2, 136.0, 130.2, 62.1, 61.9, 61.1, 56.7, 37.9, 37.6, 37.1, 33.9, 33.6, 33.2, 25.6, 24.2, 24.0, 23.9, 23.7, 22.4, 22.3, 18.9, 17.2, 12.9, 9.3, 8.6.

HRMS (ESI) calcd for $[M+Na]^+$ $C_{14}H_{20}NaO$, m/z : 227.1406, found : 227.1408.

IR v [cm^{-1}] 2960, 2919, 2873, 1631, 1458, 1335, 1023, 911.

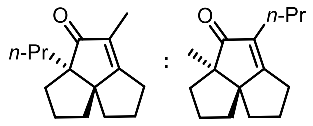

1i **1i'**

5 : 1

Compound **1i** and **1i’** was synthesized following general procedure E, yellow oil, yield 83%. Reaction time: 0.5 h.

^{1}H NMR (500 MHz, $CDCl_3$): *δ* 2.54 – 2.41 (m, 2H), 2.08 – 1.98 (m, 3H), 1.80 – 1.75 (m, 1H), 1.65 (s, 2.5H), 1.62 – 1.44 (m, 4H), 1.43 – 1.29 (m, 3H), 1.28 – 1.15 (m, 2H), 1.06 – 0.98 (m, 1H), 0.97 (s, 0.5H), 0.84 (t, *J* = 7.0 Hz, 3H).

^{13}C NMR (125 MHz, $CDCl_3$): *δ* 215.8, 215.7, 184.0, 183.6, 134.4, 130.1, 62.2, 61.9, 61.0, 56.7, 37.9, 37.6, 37.1, 35.5, 34.5, 33.6, 33.2, 25.7, 24.1, 24.1, 24.0, 23.8, 22.4, 21.4, 18.9, 18.0, 14.8, 13.8, 8.6.

HRMS (ESI) calcd for $[M+Na]^+$ $C_{15}H_{22}NaO$, m/z : 241.1563, found : 241.1563.

IR *ν* [cm^{-1}] 2957, 2869, 1631, 1457, 1329, 1036, 797.

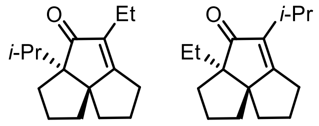

1j **1j'**

1 : 1

Compound **1j** and **1j’** was synthesized following general procedure E, yellow oil, yield 78%. Reaction time: 0.5 h.

^{1}H NMR (500 MHz, $CDCl_3$): *δ* 2.72 – 2.66 (m, 1H), 2.60 – 2.57 (m, 2H), 2.54 – 2.50 (m, 2H), 2.21 – 2.09 (m, 2H), 2.06 – 1.97 (m, 6H), 1.87 – 1.74 (m, 4H), 1.61 – 1.54 (m, 4H), 1.52 – 1.32 (m, 9H), 1.10 (d, *J* = 7.0 Hz, 3H), 1.07 (d, *J* = 7.0 Hz, 3H), 1.00 (t, *J* = 7.5 Hz, 3H), 0.95 (d, *J* = 7.0 Hz, 3H), 0.89 (t, *J* = 7.5 Hz, 3H), 0.83 (d, *J* = 7.0 Hz, 3H).

^{13}C NMR (125 MHz, $CDCl_3$): *δ* 215.4, 215.3, 183.1, 181.6, 139.7, 136.7, 64.8, 62.3, 62.2, 61.0, 39.0, 38.1, 34.2, 34.0, 33.6, 31.0, 25.6, 24.7, 24.6, 24.5, 24.5, 23.8, 22.3, 22.1, 21.3, 20.4, 18.9, 18.9, 17.1, 12.7, 9.3.

HRMS (ESI) calcd for $[M+Na]^+$ $C_{16}H_{24}NaO$, m/z : 255.1719, found : 255.1719.

IR *ν* [cm^{-1}] 2958, 2872, 1698, 1659, 1462, 1367, 1007, 839.

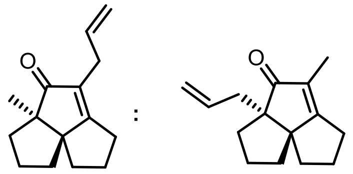

1k **1k'**

1 : 1

Compound **1k** and **1k'** was synthesized following general procedure E, yellow oil, yield 68%. Reaction time: 0.5 h.

^{1}**H NMR** (500 MHz, $CDCl_3$) δ 5.84 – 5.76 (m, 1H), 5.06 – 4.95 (m, 2H), 2.98 – 2.78 (m, 1H), 2.57 – 2.42 (m, 2H), 2.26 – 2.22 (m, 0.5H), 2.10 – 1.97 (m, 3H), 1.93 – 1.90 (m, 0.5H), 1.82 – 1.76 (m, 1H), 1.68 – 1.65 (m, 1.5H), 1.65 – 1.59 (m, 1H), 1.52 – 1.47 (m, 2H), 1.45 – 1.25 (m, 2H), 1.09 – 1.00 (m, 1H), 0.99 (s, 1.5H).

^{13}**C NMR** (125 MHz, $CDCl_3$) δ 215.0, 215.0, 185.2, 183.9, 134.8, 131.8, 130.3, 117.5, 115.1, 62.3, 62.0, 60.0, 56.8, 37.9, 37.3, 37.2, 37.1, 34.6, 33.9, 33.3, 28.1, 24.2, 24.1, 24.1, 23.7, 22.5, 22.2, 18.9, 8.6.

HRMS (ESI) calcd for $[M+Na]^+$ $C_{15}H_{20}NaO$, m/z: 239.1406 , found: 239.1407.

IR ν [cm^{-1}] 2953, 2865, 1703, 1664, 1447, 1336, 995, 670.

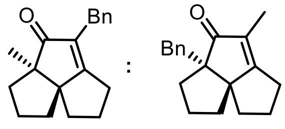

1l **1l'**

1 : 1.2

Compound **1l** and **1l'** was synthesized following general procedure E, yellow oil, yield 88%. Reaction time: 0.5 h.

^{1}**H NMR** (500 MHz, $CDCl_3$): δ 7.26 – 7.13 (m, 10H), 3.58 – 3.55 (m, 1H), 3.41 – 3.38 (m, 1H), 2.73 (s, 2H), 2.56 – 2.25 (m, 4H), 2.12 – 1.88 (m, 6H), 1.80 – 1.71 (m, 4H), 1.70 (s, 3H), 1.62 – 1.58 (m, 1H), 1.52 – 1.28 (m, 8H), 1.07 – 1.02 (m, 1H), 1.01 (s, 3H).

^{13}**C NMR** (125 MHz, $CDCl_3$): δ 215.1, 214.6, 185.3, 183.7, 139.6, 138.2, 133.4, 130.7, 130.4, 128.5, 128.3, 127.6, 126.0, 125.8, 62.5, 62.3, 61.4, 56.7, 38.9, 37.9, 37.2, 37.2, 35.2, 34.3, 33.2, 30.0, 24.2, 24.1, 24.1, 23.6, 22.5, 22.0, 18.9, 8.7.

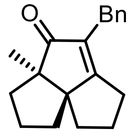

1l

^{1}**H NMR** (500 MHz, $CDCl_3$): δ 7.26 – 7.13 (m, 5H), 3.58 – 3.55 (m, 1H), 3.41 – 3.38 (m, 1H), 2.39 – 2.25 (m, 2H), 2.10 (dd, J = 5.5, 12.0 Hz, 1H), 2.04 – 1.93 (m, 2H), 1.78 (dd, J = 6.0, 12.5 Hz, 1H), 1.63 – 1.58 (m, 2H), 1.52 – 1.29 (m, 5H), 1.07 – 1.02 (m, 1H), 1.00 (s, 3H).

^{13}**C NMR** (125 MHz, $CDCl_3$): δ 215.1, 185.3, 139.6, 133.4, 128.5, 128.3, 125.9, 62.3, 56.8, 38.0, 37.2, 33.2, 30.0, 24.2, 24.1, 22.5, 18.9.

HRMS (ESI) calcd for $[M+Na]^+$ $C_{19}H_{22}NaO$, m/z : 289.1563, found : C19H22NaO.

IR ν [cm^{-1}] 3084, 3061, 3027, 2954, 2864, 1702, 1659, 1494, 1452, 1368, 972.

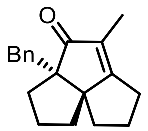

1l'

^{1}H NMR (500 MHz, $CDCl_3$): *δ* 7.26 – 7.15 (m, 5H), 2.73 (s, 2H), 2.56 – 2.43 (m, 2H), 2.06 – 2.00 (m, 2H), 1.92 – 1.87 (m, 1H), 1.78 – 1.71 (m, 3H), 1.70 (s, 3H), 1.54 – 1.43 (m, 2H), 1.34 – 1.25 (m, 1H), 1.05 – 0.95 (m, 1H).

^{13}C NMR (125 MHz, $CDCl_3$): *δ* 214.7, 183.7, 138.2, 130.7, 127.7, 126.0, 62.5, 61.5, 38.9, 37.3, 35.2, 34.3, 24.2, 23.7, 22.0, 8.7.

HRMS (ESI) calcd for $[M+Na]^+$ $C_{19}H_{22}NaO$, m/z : 289.1563, found : C19H22NaO.

IR *ν* [cm^{-1}] 3085, 3061, 3028, 2953, 2866, 1704, 1667, 1452, 1334, 1036, 997.

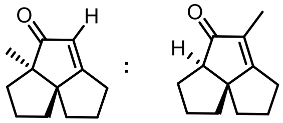

1m **1m'**

1 : 1

Compound **1m** and **1m'** was synthesized following general procedure E, yellow oil, yield 81%. Reaction time: 0.5 h.

^{1}H NMR (500 MHz, $CDCl_3$) *δ* 5.80 (s, 1H), 2.69 – 2.61 (m, 1H), 2.56 – 2.46 (m, 3H), 2.38 (d, *J* = 8.5 Hz, 1H), 2.10 – 2.07 (m, 1H), 2.05 – 1.99 (m, 4H), 1.86 – 1.81 (m, 2H), 1.73 – 1.69 (m, 2H), 1.66 (s, 3H), 1.63 – 1.58 (m, 2H), 1.56 – 1.48 (m, 3H), 1.45 – 1.40 (m, 1H), 1.34 – 1.30 (m, 1H), 1.30 – 1.26 (m, 1H), 1.26 – 1.25 (m, 1H), 1.21 – 1.17 (m, 1H), 1.16 – 1.05 (m, 1H), 1.00 (s, 3H).

^{13}C NMR (125 MHz, $CDCl_3$): *δ* 216.6, 213.8, 191.6, 185.2, 131.7, 123.0, 64.1, 59.2, 57.7, 56.6, 37.8, 37.2, 36.4, 36.0, 33.0, 29.3, 25.2, 24.7, 24.4, 24.1, 23.8, 22.5, 18.9, 8.7.

HRMS (ESI) calcd for $[M+Na]^+$ $C_{12}H_{16}NaO$, m/z : 199.1093 , found : 199.1097.

IR *ν* [cm^{-1}] 2956, 2926, 2834, 1648, 1612, 1310, 1098.

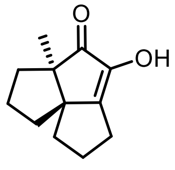

1n

Compound **1n** was synthesized following general procedure E, yellow oil, yield 80%. Reaction time: 1 h.

^{1}H NMR (400 MHz, $CDCl_3$): *δ* 6.17 (bs, 1H), 2.27 – 2.14 (m, 8H), 2.11 (s, 3H), 2.09 – 2.06 (m,

1H), 1.98 – 1.84 (m, 3H).
^{13}C NMR (100 MHz, $CDCl_3$): δ 205.2, 147.0, 145.4, 53.9, 50.4, 27.8, 27.4, 15.8, 15.2, 10.2.
HRMS (ESI) calcd for $[M+Na]^+$ $C_{12}H_{16}NaO_2$, m/z : 215.1043 , found : 215.1044.
IR *v* [cm^{-1}] 3446, 2949, 1747, 1292, 1260, 1106, 1050, 739.

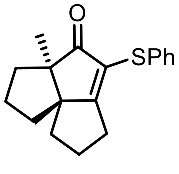

1o

Compound **1o** was synthesized following general procedure E, yellow oil, yield 96%. Reaction time: 0.5 h.
^{1}H NMR (500 MHz, $CDCl_3$) δ 7.29 – 7.27 (m, 4H), 7.23 – 7.18 (m, 1H), 2.51 – 2.43 (m, 1H), 2.41 – 2.34 (m, 1H), 2.21 (dd, *J* = 12.5, 6.0 Hz, 1H), 2.03 – 2.00 (m, 2H), 1.90 (dd, *J* = 12.5, 6.5 Hz, 1H), 1.73 – 1.69 (m, 1H), 1.64 – 1.59 (m, 2H), 1.56 – 1.49 (m, 1H), 1.44 – 1.38 (m, 1H), 1.24 – 1.14 (m, 1H), 1.08 (s, 3H).
^{13}C NMR (125 MHz, $CDCl_3$) δ 211.3, 191.2, 134.1, 129.7, 128.9, 127.3, 126.5, 63.5, 57.5, 38.1, 37.5, 33.5, 25.7, 23.8, 22.8, 18.9.
HRMS (ESI) calcd for $[M+Na]^+$ $C_{18}H_{20}NaOS$, m/z : 307.1127 , found : 307.1123.
IR *v* [cm^{-1}] 2953, 2862, 1710, 1619, 1439, 1233, 738, 689.

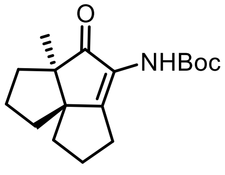

1p

Compound **1p** was synthesized following general procedure E, yellow oil, yield 55%. Reaction time: 6 h.
^{1}H NMR (500 MHz, $CDCl_3$) δ 6.42 (bs, 1H), 2.92 – 2.86 (m, 1H), 2.72 (dt, *J* = 20.0, 8.5 Hz, 1H), 2.07 – 1.98 (m, 3H), 1.91 (dd, *J* = 12.5, 6.0 Hz, 1H), 1.66 – 1.62 (m, 1H), 1.53 – 1.49 (m, 2H), 1.46 (s, 9H), 1.41 – 1.28 (m, 2H), 1.18 – 1.08 (m, 1H), 1.02 (s, 3H).
^{13}C NMR (125 MHz, $CDCl_3$) δ 209.5, 166.5, 152.4, 127.8, 80.4, 61.8, 54.7, 38.0, 37.0, 34.1, 28.2, 27.1, 24.5, 22.4, 19.0.
HRMS (ESI) calcd for $[M+Na]^+$ $C_{17}H_{25}NNaO_3$, m/z: 314.1727 , found: 314.1728.
IR *v* [cm^{-1}] 2955, 2865, 1708, 1661, 1511, 1160, 942.

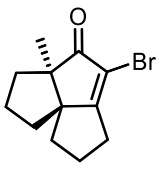

1q

Compound **1q** was synthesized following general procedure E, yellow oil, yield 92%. Reaction time: 1 h.

^{1}H NMR (500 MHz, $CDCl_3$): *δ* 2.66-2.52 (m, 2H), 2.17-2.05 (m, 3H), 1.89-1.85 (m, 1H), 1.71-1.65 (m, 1H), 1.62-1.54 (m, 2H), 1.48-1.42 (m, 1H), 1.40-1.33 (m, 1H), 1.18-1.08 (m, 1H), 1.05 (s, 3H).

^{13}C NMR (125 MHz, $CDCl_3$): *δ* 208.1, 187.5, 114.8, 63.9, 57.1, 38.2, 36.9, 33.5, 25.9, 23.6, 22.5, 19.0.

HRMS (ESI) calcd for $[M+Na]^+$ $C_{12}H_{15}BrNaO$, m/z : 277.0198 , found : 277.0195.

IR *v* [cm^{-1}] 2955, 2864, 1718, 1636, 764, 750.

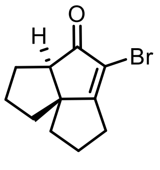

1r

Compound **1r** was synthesized following general procedure E, yellow oil, yield 30%. Reaction time: 15 h.

^{1}H NMR (500 MHz, $CDCl_3$): *δ* 2.67-2.55 (m, 3H), 2.16-2.05 (m, 3H), 1.95-1.92 (m, 1H), 1.83-1.77 (m, 1H), 1.77-1.64 (m, 3H), 1.48-1.41 (m, 1H), 1.38-1.29 (m, 1H).

^{13}C NMR (125 MHz, $CDCl_3$): *δ* 205.7, 188.5, 116.2, 61.3, 56.2, 36.6, 36.1, 29.6, 25.8, 24.9, 23.9.

HRMS (ESI) calcd for $[M+Na]^+$ $C_{11}H_{13}BrNaO$, m/z : 263.0042, found : 263.0042 .

IR *v* [cm^{-1}] 2990, 2940, 1716, 1648, 1296, 668.

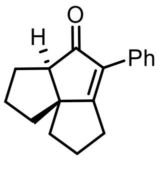

1t

Compound **1t** was synthesized following general procedure E, yellow oil, yield 71%. Reaction time: 4 h.

^{1}H NMR (500 MHz, $CDCl_3$) *δ* 7.61 – 7.59 (m, 2H), 7.40 – 7.37 (m, 2H), 7.31 – 7.28 (m, 1H), 2.93 – 2.86 (m, 1H), 2.70 – 2.59 (m, 1H), 2.60 (d, *J* = 8.6 Hz, 1H), 2.20 – 2.12 (m, 2H), 2.07 – 2.00 (m,

1H), 1.92 (dd, *J* = 12.0, 7.0 Hz, 1H), 1.86 (dd, *J* = 12.5, 6.5 Hz, 1H), 1.81 – 1.73 (m, 1H), 1.70 – 1.65 (m, 1H), 1.64 – 1.57 (m, 1H), 1.52 – 1.46 (m, 1H), 1.38 – 1.29 (m, 1H).

^{13}C NMR (125 MHz, $CDCl_3$) δ 211.6, 186.7, 134.3, 131.7, 128.3, 128.2, 127.6, 59.4, 57.8, 36.6, 36.1, 29.6, 26.2, 24.9, 24.7.

HRMS (ESI) calcd for $[M+Na]^+$ $C_{17}H_{18}NaO$, m/z : 261.1250 , found : 261.1253.

IR *v* [cm^{-1}] 2950, 2863, 1692, 1638, 1445, 1146, 755, 695.

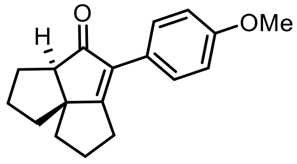

1u

Compound **1u** was synthesized following general procedure E, yellow oil, yield 75%. Reaction time: 4 h.

^{1}H NMR (500 MHz, $CDCl_3$) δ 7.56 (d, *J* = 8.8 Hz, 2H), 6.92 (d, *J* = 8.8 Hz, 2H), 3.82 (s, 3H), 2.87 (ddd, *J* = 19.0, 11.0, 3.0 Hz, 1H), 2.64 (dt, *J* = 18.5, 8.0 Hz, 1H), 2.57 (d, *J* = 8.5 Hz, 1H), 2.19 – 2.11 (m, 2H), 2.06 – 2.00 (m, 1H), 1.91 (dd, *J* = 12.0, 7.0 Hz, 1H), 1.84 (dd, *J* = 12.5, 6.5 Hz, 1H), 1.75 (tdd, *J* = 12.5, 8.5, 6.0 Hz, 1H), 1.68 – 1.64 (m, 1H), 1.62 – 1.55 (m, 1H), 1.50 – 1.44 (m, 1H), 1.36 – 1.24 (m, 1H).

^{13}C NMR (125 MHz, $CDCl_3$) δ 211.9, 185.0, 159.0, 133.8, 129.5, 124.4, 113.6, 59.3, 57.7, 55.2, 36.6, 36.1, 29.5, 26.2, 24.9, 24.8.

HRMS (ESI) calcd for $[M+Na]^+$ $C_{18}H_{20}NaO_2$, m/z: 291.1356 , found: 291.1356.

IR *v* [cm^{-1}] 2951, 2862, 1693, 1607, 1511, 1248, 1180, 830.

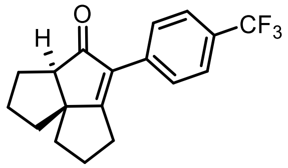

1v

Compound **1v** was synthesized following general procedure E, yellow oil, yield 51%. Reaction time: 48 h.

^{1}H NMR (500 MHz, $CDCl_3$) δ 7.71 (d, *J* = 8.5 Hz, 2H), 7.63 (d, *J* = 8.0 Hz, 2H), 2.93 (ddd, *J* = 19.0, 11.0, 3.0 Hz, 1H), 2.70 – 2.65 (m, 1H), 2.63 (d, *J* = 8.5 Hz, 1H), 2.23 – 2.13 (m, 2H), 2.10 – 2.04 (m, 1H), 1.95 (dd, *J* = 12.0, 7.0 Hz, 1H), 1.88 (dd, *J* = 12.5, 6.5 Hz, 1H), 1.83 – 1.75 (m, 1H), 1.73 – 1.68 (m, 1H), 1.66 – 1.61 (m, 1H), 1.52 (td, *J* = 12.5, 6.5 Hz, 1H), 1.39 – 1.28 (m, 1H).

^{13}C NMR (125 MHz, $CDCl_3$) δ 211.1, 188.8, 135.3, 133.3, 129.4 (q, J = 32.5 Hz), 128.5, 125.1(q, J = 3.8 Hz), 124.1(q, J = 271.3 Hz), 59.8, 57.9, 36.7, 36.0, 29.6, 26.3, 25.0, 24.7.
^{19}F NMR (471 MHz, $CDCl_3$) δ -62.60.
HRMS (ESI) calcd for $[M+Na]^+$ $C_{18}H_{17}F_3NaO$, m/z: 329.1124 , found: 329.1124.
IR ν [cm^{-1}] 2954, 2865, 1700, 1639, 1325, 1123, 1067, 838.

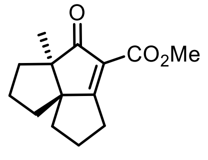

1w

Compound **1w** was synthesized following general procedure E, yellow oil, yield 81%. Reaction time: 12 h.
^{1}H NMR (400 MHz, $CDCl_3$) δ 3.77 (s, 3H), 2.99 – 2.83 (m, 2H), 2.15 (dd, J = 12.4, 6.0 Hz, 1H), 2.09 – 2.01 (m, 2H), 1.91 – 1.85 (m, 1H), 1.68 – 1.65 (m, 1H), 1.58 – 1.47 (m, 3H), 1.34 (tt, J = 13.6, 6.6 Hz, 1H), 1.13 – 1.04 (m, 1H), 0.99 (s, 3H).
^{13}C NMR (100 MHz, $CDCl_3$) δ 209.7, 202.5, 162.9, 124.7, 63.0, 58.8, 51.5, 38.1, 38.0, 33.0, 27.4, 23.7, 22.5, 18.7.
HRMS (ESI) calcd for $[M+Na]^+$ $C_{14}H_{18}NaO_3$, m/z : 257.1148, found : 257.1150 .
IR ν [cm^{-1}] 2953, 2867, 1744, 1708, 1640, 1435, 1347, 1334, 1230, 1003.

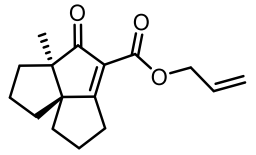

1x

Compound **1x** was synthesized following general procedure E, yellow oil, yield 54%. Reaction time: 12 h.
^{1}H NMR (400 MHz, $CDCl_3$) δ 6.02 – 5.92 (m, 1H), 5.43 – 5.37 (m, 1H), 5.26 – 5.23 (m, 1H), 4.72 – 4.69 (m, 2H), 3.04 – 2.85 (m, 2H), 2.19 (dd, J = 12.4, 6.4 Hz, 1H), 2.12 – 2.04 (m, 2H), 1.93 – 1.88 (m, 1H), 1.74 – 1.67 (m, 1H), 1.61 – 1.49 (m, 3H), 1.41 – 1.34 (m, 1H), 1.21 – 1.08 (m, 1H), 1.03 (s, 3H).
^{13}C NMR (100 MHz, $CDCl_3$) δ 209.7, 202.2, 162.1, 131.9, 124.8, 118.4, 65.1, 63.1, 58.8, 38.2, 38.1, 33.1, 27.6, 23.8, 22.6, 18.7.
HRMS (ESI) calcd for $[M+Na]^+$ $C_{16}H_{20}NaO_3$, m/z : 283.1305, found : 283.1305 .
IR ν [cm^{-1}] 2956, 2896, 2867, 1745, 1715, 1644, 1452, 1370, 1333, 1290, 1225, 1197, 1183, 1015, 994, 934, 809.

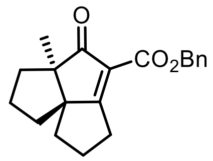

1y

Compound **1y** was synthesized following general procedure E, yellow oil, yield 48%. Reaction time: 12 h.

^{1}H NMR (400 MHz, $CDCl_3$) δ 7.45 – 7.43 (m, 2H), 7.38 – 7.31 (m, 3H), 5.30 – 5.22 (m, 2H), 3.02 – 2.83 (m, 2H), 2.23 – 2.17 (m, 1H), 2.09 – 2.03 (m, 2H), 1.93 – 1.88 (m, 1H), 1.72 – 1.67 (m, 1H), 1.61 – 1.50 (m, 3H), 1.42 – 1.34 (m, 1H), 1.22 – 1.09 (m, 1H), 1.04 (s, 3H).

^{13}C NMR (100 MHz, $CDCl_3$) δ 209.7, 202.1, 162.2, 135.9, 128.5, 128.1, 128.0, 124.7, 66.0, 63.2, 58.8, 38.2, 38.1, 33.1, 27.6, 23.9, 22.6, 18.8.

HRMS (ESI) calcd for $[M+Na]^+$ $C_{20}H_{22}NaO_3$, m/z : 333.1461, found : 333.1463 .

IR ν [cm^{-1}] 2956, 2894, 2866, 1747, 1715, 1641, 1454, 1379, 1340, 1291, 1224, 1195, 1184, 1045, 1014, 1000, 739, 698.

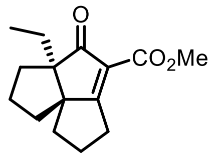

1z

Compound **1z** was synthesized following general procedure E, yellow oil, yield 81%. Reaction time: 12 h.

^{1}H NMR (500 MHz, $CDCl_3$) δ 3.78 (s, 3H), 3.01 – 2.82 (m, 2H), 2.12 – 2.02 (m, 3H), 1.88 (dd, *J* = 13.0, 7.0 Hz, 1H), 1.72 – 1.61 (m, 2H), 1.58 – 1.36 (m, 5H), 1.14 – 1.07 (m, 1H), 0.91 (t, *J* = 7.5 Hz, 3H).

^{13}C NMR (125 MHz, $CDCl_3$) δ 209.5, 202.3, 163.0, 124.9, 63.3, 63.1, 51.5, 38.7, 34.3, 33.4, 27.4, 25.6, 23.9, 22.5, 9.1.

HRMS (ESI) calcd for $[M+Na]^+$ $C_{15}H_{20}NaO_3$, m/z: 271.1305 , found: 271.1305.

IR ν [cm^{-1}] 2952, 2866, 1746, 1713, 1643, 1436, 1348, 1224, 1016, 814.

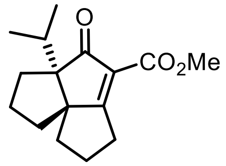

1aa

Compound **1aa** was synthesized following general procedure E, yellow oil, yield 85%. Reaction time: 12 h.

^{1}H NMR (500 MHz, $CDCl_3$) δ 3.79 (s, 3H), 3.01 – 2.86 (m, 2H), 2.15 – 2.00 (m, 3H), 1.93 – 1.80 (m, 3H), 1.72 – 1.68 (m, 1H), 1.56 – 1.47 (m, 3H), 1.13 – 1.02 (m, 1H), 0.97 (d, J = 7.0 Hz, 3H), 0.82 (d, J = 7.0 Hz, 3H).

^{13}C NMR (125 MHz, $CDCl_3$) δ 209.4, 202.7, 162.9, 125.6, 66.9, 63.5, 51.5, 39.9, 34.4, 33.6, 31.2, 27.5, 24.1, 22.3, 19.0, 18.6.

HRMS (ESI) calcd for $[M+Na]^+$ $C_{16}H_{22}NaO_3$, m/z: 285.1461, found: 285.1461.

IR v [cm^{-1}] 2960, 2874, 1659, 1456, 1286, 899.

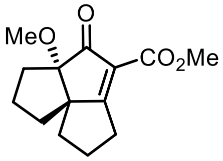

1bb

Compound **1bb** was synthesized following general procedure E, yellow oil, yield 36%. Reaction time: 12 h.

^{1}H NMR (500 MHz, C_6D_6) δ 3.47 (s, 3H), 3.39 (s, 3H), 2.80 – 2.73 (m, 1H), 2.51 – 2.44 (m, 1H), 2.40 (dd, J = 11.5, 5.5 Hz, 1H), 1.85 – 1.78 (m, 1H), 1.55 – 1.48 (m, 2H), 1.39 – 1.25 (m, 4H), 1.16 – 1.10 (m, 1H), 0.97 – 0.85 (m, 1H).

^{13}C NMR (125 MHz, C_6D_6) δ 202.3, 200.6, 162.9, 124.9, 90.1, 62.1, 53.0, 51.0, 37.4, 32.1, 31.5, 27.7, 23.5, 21.1.

HRMS (ESI) calcd for $[M+Na]^+$ $C_{14}H_{18}NaO_4$, m/z : 273.1097, found : 273.1096 .

IR v [cm^{-1}] 2955, 2873, 2834, 1747, 1715, 1644, 1435, 1352, 1296, 1231, 1114, 1023, 1002, 827, 815, 731, 563, 554.

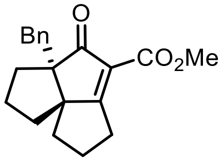

1cc

Compound **1cc** was synthesized following general procedure E, yellow oil, yield 71%. Reaction time: 12 h.

^{1}H NMR (500 MHz, $CDCl_3$) δ 7.27 – 7.14 (m, 5H), 3.83 (s, 3H), 3.03 – 2.89 (m, 2H), 2.76 (s, 2H), 2.11 – 2.00 (m, 3H), 1.91 – 1.81 (m, 2H), 1.79 – 1.75 (m, 1H), 1.59 – 1.45 (m, 3H), 1.15 – 1.04 (m, 1H).

^{13}C NMR (125 MHz, $CDCl_3$) δ 208.4, 201.9, 163.0, 137.3, 130.7, 127.8, 126.2, 125.2, 63.7, 63.4, 51.6, 38.7, 38.5, 35.6, 34.1, 27.4, 24.0, 22.1.
HRMS (ESI) calcd for $[M+Na]^+$ $C_{20}H_{22}NaO_3$, m/z : 333.1461, found : 333.1461 .
IR ν [cm^{-1}] 2956, 2894, 2866, 1744, 1715, 1642, 1455, 1379, 1340, 1291, 1224, 1000, 740, 698.

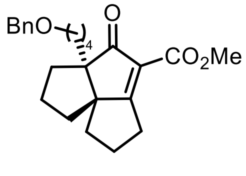

1dd

Compound **1dd** was synthesized following general procedure E, yellow oil, yield 53%. Reaction time: 48 h at 50 ºC.
^{1}H NMR (400 MHz, $CDCl_3$) δ 7.35 – 7.24 (m, 5H), 4.47 (s, 2H), 3.81 (s, 3H), 3.43 (t, *J* = 6.4 Hz, 2H), 3.03 – 2.85 (m, 2H), 2.16 – 2.02 (m, 3H), 1.89 (dd, *J* = 12.8, 6.4 Hz, 1H), 1.73 – 1.66 (m, 2H), 1.63 – 1.38 (m, 8H), 1.36 – 1.25 (m, 2H), 1.18 – 1.04 (m, 1H).
^{13}C NMR (100 MHz, $CDCl_3$) δ 209.4, 202.5, 163.0, 138.5, 128.3, 127.6, 127.4, 124.9, 72.8, 70.0, 63.4, 62.9, 51.6, 38.7, 35.0, 33.5, 32.8, 30.3, 27.5, 23.9, 22.6, 21.1.
HRMS (ESI) calcd for $[M+Na]^+$ $C_{24}H_{30}NaO_4$, m/z : 405.2036, found : 405.2036 .
IR ν [cm^{-1}] 2948, 2865, 1744, 1709, 1644, 1453, 1435, 1348, 1336, 1293, 1225, 1101, 736, 698.

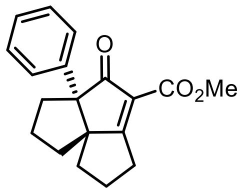

1ff

Compound **1ff** was synthesized following general procedure E, white solid, yield 85%. Reaction time: 12 h.
^{1}H NMR (400 MHz, $CDCl_3$) δ 7.27 – 7.03 (m, 5H), 3.85 (s, 3H), 3.03 – 2.86 (m, 2H), 2.48 (dd, *J* = 12.4, 5.2 Hz, 1H), 2.18 – 2.10 (m, 1H), 2.07 – 1.96 (m, 2H), 1.95 – 1.87 (m, 1H), 1.80 – 1.67 (m, 2H), 1.47 (dd, *J* = 12.4, 6.4 Hz, 1H), 1.37 – 1.23 (m, 1H), 1.02 – 0.94 (m, 1H).
^{13}C NMR (100 MHz, $CDCl_3$) δ 208.0, 202.6, 162.8, 140.1, 128.3, 127.4, 126.6, 126.5, 68.8, 65.7, 51.7, 38.7, 38.1, 34.9, 27.8, 23.8, 22.9.
HRMS (ESI) calcd for $[M+Na]^+$ $C_{19}H_{20}NaO_3$, m/z : 319.1305, found : 319.1301 .
IR ν [cm^{-1}] 2953, 2868, 1744, 1714, 1642, 1435, 1347, 1290, 1220, 1021, 767, 730, 699.
Mp 92.6 – 94.7 ºC.

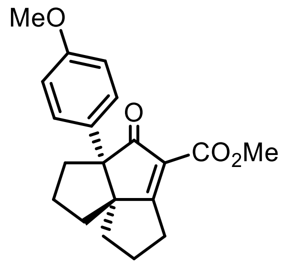

1gg

Compound **1gg** was synthesized following general procedure E, yellow oil, yield 88%. Reaction time: 12 h.

^{1}H NMR (400 MHz, $CDCl_3$) δ 6.98 – 6.96 (m, 2H), 6.80 – 6.77 (m, 2H), 3.84 (s, 3H), 3.74 (s, 3H), 3.02 – 2.86 (m, 2H), 2.44 (dd, J = 12.8, 5.2 Hz, 1H), 2.14 – 2.05 (m, 1H), 2.05 – 1.96 (m, 2H), 1.95 – 1.87 (m, 1H), 1.76 – 1.67 (m, 2H), 1.47 (dd, J = 12.4, 6.4 Hz, 1H), 1.31 – 1.24 (m, 1H), 1.05 – 0.97 (m, 1H).

^{13}C NMR (100 MHz, $CDCl_3$) δ 208.2, 202.7, 162.8, 158.1, 132.1, 128.4, 126.4, 113.6, 68.2, 65.6, 55.1, 51.6, 38.6, 38.1, 34.8, 27.8, 23.8, 22.8.

HRMS (ESI) calcd for $[M+Na]^+$ $C_{20}H_{22}NaO_4$, m/z : 349.1410, found : 349.1409 .

IR ν [cm^{-1}] 2953, 2868, 1744, 1714, 1642, 1514, 1437, 1349, 1291, 1253, 1221, 1186, 1033, 829, 809, 738, 572, 535.

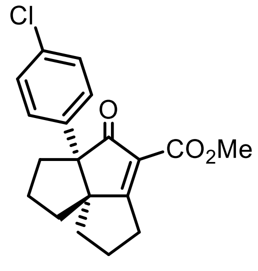

1hh

Compound **1hh** was synthesized following general procedure E, yellow oil, yield 75%. Reaction time: 12 h.

^{1}H NMR (400 MHz, $CDCl_3$) δ 7.25 – 7.21 (m, 2H), 7.02 – 6.98 (m, 2H), 3.86 (s, 3H), 3.02 – 2.93 (m, 2H), 2.48 (dd, J = 12.2, 5.2 Hz, 1H), 2.13 – 1.90 (m, 4H), 1.81 – 1.68 (m, 2H), 1.49 (dd, J = 12.4, 6.4 Hz, 1H), 1.37 – 1.23 (m, 1H), 1.03 – 0.95 (m, 1H).

^{13}C NMR (100 MHz, $CDCl_3$) δ 207.4, 202.7, 162.6, 138.6, 132.5, 128.9, 128.4, 126.5, 68.2, 65.6, 51.8, 38.6, 38.4, 34.8, 27.8, 23.8, 22.8.

HRMS (ESI) calcd for $[M+Na]^+$ $C_{19}H_{19}ClNaO_3$, m/z : 353.0915, found : 353.0907 .

IR ν [cm^{-1}] 2953, 2868, 1745, 1715, 1642, 1494, 1436, 1350, 1297, 1133, 1095, 1019, 820, 784,

737.

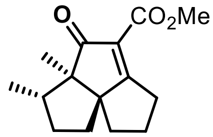

3a

Compound **3a** was synthesized following general procedure E, yellow oil, yield 62%. Reaction time: 12 h.

^{1}H NMR (400 MHz, $CDCl_3$) δ 3.81 (s, 3H), 2.96 – 2.92 (m, 2H), 2.44 – 2.38 (m, 1H), 2.11 – 2.05 (m, 2H), 1.80 – 1.66 (m, 2H), 1.67 – 1.55 (m, 2H), 1.44 – 1.24 (m, 2H), 0.92 – 0.90 (m, 6H).

^{13}C NMR (100 MHz, $CDCl_3$) δ 210.4, 202.8, 163.2, 123.5, 63.1, 62.8, 51.6, 39.3, 35.6, 34.0, 29.6, 27.7, 24.3, 15.6, 15.3.

HRMS (ESI) calcd for $[M+Na]^+$ $C_{15}H_{20}NaO_3$, m/z : 271.1305, found : 271.1305 .

IR ν [cm^{-1}] 2955, 2869, 1745, 1715, 1642, 1435, 1347, 1334, 1294, 1231, 1209, 1024, 1007.

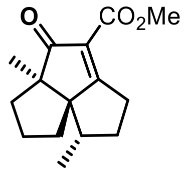

3b

Compound **3b** was synthesized following general procedure E, yellow oil, yield 90%. Reaction time: 12 h.

^{1}H NMR (400 MHz, $CDCl_3$) δ 3.80 (s, 3H), 3.00 (dt, J = 21.0, 8.5 Hz, 1H), 2.90 – 2.82 (m, 1H), 2.37 – 2.24 (m, 2H), 2.07 (ddd, J = 12.0, 6.0, 1.5 Hz, 1H), 1.90 – 1.82 (m, 1H), 1.66 – 1.58 (m, 2H), 1.50 – 1.42 (m, 2H), 1.17 (s, 3H), 1.10 – 0.99 (m, 1H), 0.86 (d, J = 7.0 Hz, 3H).

^{13}C NMR (100 MHz, CDCl3) δ 210.0, 202.6, 162.8, 126.3, 66.6, 58.9, 51.5, 41.5, 40.6, 40.1, 34.5, 25.6, 21.6, 20.6, 17.8.

HRMS (ESI) calcd for $[M+Na]^+$ $C_{15}H_{20}NaO_3$, m/z : 271.1305, found : 271.1306.

IR ν [cm^{-1}] 2953, 2877, 1745, 1716, 1644, 1437, 1352, 1332, 1290, 1260, 1139.

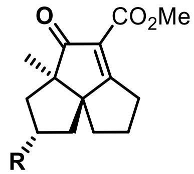

R = $SiPhMe_2$

3cα

Compound **3c** was synthesized following entry 9 in **Supplementary Table 1** , yellow oil, yield

81%, **3cα**: **3cβ** =1： 7. Reaction time: 48 h at -10 ºC.

^{1}H NMR (500 MHz, $CDCl_3$) δ 7.42 – 7.40 (m, 2H), 7.35 – 7.33 (m, 3H), 3.81 (s, 3H), 3.02 – 2.84 (m, 2H), 2.24 – 2.20 (m, 1H), 2.07 – 2.00 (m, 2H), 1.90 – 1.86 (m, 1H), 1.66 – 1.62 (m, 1H), 1.59 – 1.53 (m, 1H), 1.39 (t, J = 13.0 Hz, 1H), 1.21 (dd, J = 14.0, 12.5 Hz, 1H), 0.99 (s, 3H), 0.91 – 0.83 (m, 1H), 0.22 (s, 6H).

^{13}C NMR (125 MHz, $CDCl_3$) δ 210.0, 202.9, 163.0, 137.4, 133.7, 129.0, 127.7, 125.0, 64.4, 60.0, 51.6, 40.6, 40.1, 33.0, 27.5, 23.8, 22.4, 18.4, 4.5, -4.9.

HRMS (ESI) calcd for $[M+Na]^+$ $C_{22}H_{28}NaO_3Si$, m/z : 391.1700, found : 391.1698.

IR ν [cm^{-1}] 2952, 1746, 1710, 1639, 1434, 1428, 1346, 1334, 1293, 1113, 1021, 1012, 837, 810, 731, 669.

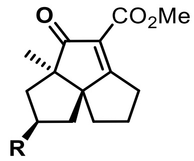

R = SiPhMe$_2$
3cβ

Compound **3c** was synthesized following general procedure E, yellow oil, yield 81%, **3cα**: **3cβ** = 1: 7. Reaction time: 48 h at -10 ºC.

^{1}H NMR (500 MHz, $CDCl_3$) δ 7.44 – 7.30 (m, 5H), 3.79 (s, 3H), 3.02 – 2.94 (m, 1H), 2.86 – 2.80 (m, 1H), 2.08 – 2.01 (m, 2H), 1.97 – 1.89 (m, 1H), 1.77 – 1.74 (m, 1H), 1.71 – 1.60 (m, 3H), 1.59 – 1.51 (m, 1H), 1.16 – 1.10 (m, 1H), 1.08 (s, 3H), 0.21 (s, 3H), 0.21 (s, 3H).

^{13}C NMR (125 MHz, $CDCl_3$) δ 210.1, 201.4, 163.3, 137.5, 133.5, 129.0, 127.7, 120.5, 66.6, 62.6, 51.5, 42.2, 37.7, 30.0, 28.8, 28.2, 23.9, 20.6, -4.8, -5.1.

HRMS (ESI) calcd for $[M+Na]^+$ $C_{22}H_{28}NaO_3Si$, m/z : 391.1700, found : 391.1691.

IR ν [cm^{-1}] 2953, 1746, 1710, 1640, 1439, 1429, 1347, 1352, 1334, 1292, 1111, 1014, 1004, 843, 815, 735, 700.

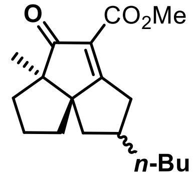

3dα : 3dβ = 1:3

Compound **3d** was synthesized following general procedure E, yellow oil, yield 85%, **3dα**: **3dβ** = 1 : 3. Reaction time: 12 h.

^{1}H NMR (400 MHz, $CDCl_3$) δ 3.79 (s, 3H), 3.35 (dd, J = 17.8, 7.7 Hz, 0.78H), 3.10 (dd, J = 20.7, 9.6 Hz, 0.26H), 2.59 – 2.37 (m, 1H), 2.24 – 2.10 (m, 2H), 2.06 – 1.77 (m, 2H), 1.65 – 1.49 (m,

3H), 1.42 – 1.37 (m, 1H), 1.32 – 1.17 (m, 6H), 1.12 – 1.03 (m, 1H), 1.00 (s, 3H), 0.91 – 0.87 (m, 3H).

^{13}C NMR (100 MHz, $CDCl_3$) δ 209.7, 209.5, 202.3, 200.8, 163.1, 163.0, 124.7, 124.4, 63.3, 61.0, 59.7, 58.9, 51.6, 51.5, 42.1, 40.5, 38.7, 38.6, 38.1, 38.0, 37.8, 37.6, 37.3, 35.9, 34.8, 34.5, 30.6, 30.3, 23.1, 22.7, 22.6, 22.6, 19.3, 18.7, 14.0, 14.0.

HRMS (ESI) calcd for $[M+Na]^+$ $C_{18}H_{26}NaO_3$, m/z : 313.1774, found : 313.1775.

IR *ν* [cm^{-1}] 2953, 2930, 2859, 1748, 1712, 1644, 1435, 1346, 1231, 1199, 1003, 799, 739.

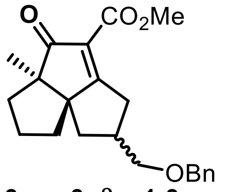

3eα : 3eβ = 1:3

Compound **3e** was synthesized following general procedure E, yellow oil, yield 85%, **3eα**: **3eβ**=1:3. Reaction time: 12 h.

^{1}H NMR (400 MHz, $CDCl_3$) δ 7.38 – 7.27 (m, 5H), 4.55 (s, 1.51H), 4.53 (s, 0.49H), 3.81 (s, 3H), 3.55 – 3.33 (m, 3H), 2.82 – 2.58 (m, 2H), 2.20 – 2.13 (m, 1H), 2.04 – 1.79 (m, 2H), 1.61 – 1.49 (m, 3H), 1.39 – 1.28 (m, 1H), 1.14 – 1.06 (m, 1H), 1.05 – 1.02 (m, 3H).

^{13}C NMR (100 MHz, $CDCl_3$) δ 209.6, 209.4, 200.9, 199.9, 162.9, 162.9, 138.1, 128.4, 127.7, 127.6, 125.0, 124.9, 74.1, 73.7, 73.2, 73.1, 63.2, 61.1, 59.8, 58.9, 51.7, 51.6, 41.6, 38.8, 38.6, 38.1, 37.9, 37.9, 37.2, 34.1, 31.9, 31.2, 23.1, 22.6, 19.3, 18.8.

HRMS (ESI) calcd for $[M+Na]^+$ $C_{22}H_{26}NaO_4$, m/z : 377.1723, found : 377.1725.

IR *ν* [cm^{-1}] 2951, 2864, 1746, 1713, 1644, 1436, 1349, 1239, 1198, 1009, 1004, 739, 699.

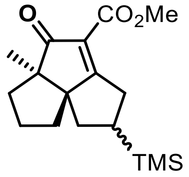

3fα : 3fβ = 1:10

Compound **3f** was synthesized following general procedure E, yellow oil, yield 65%, **3fα**: **3fβ** = 1:10. Reaction time: 12 h.

^{1}H NMR (400 MHz, $CDCl_3$) δ 3.81 (s, 3H), 3.25 (dd, *J* = 16.0, 7.6 Hz, 1H), 2.45 (dd, *J* = 16.0, 12.0 Hz, 1H), 2.17 – 2.06 (m, 2H), 1.91 – 1.85 (m, 1H), 1.58 – 1.41 (m, 3H), 1.35 – 1.22 (m, 2H), 1.09 – 1.03 (m, 4H), 0.05 (s, 9H).

^{13}C NMR (100 MHz, $CDCl_3$) δ 210.2, 200.4, 163.2, 123.5, 60.9, 60.0, 51.7, 42.6, 38.2, 31.4, 29.8, 24.3, 23.2, 19.6, -3.1.

HRMS (ESI) calcd for $[M+Na]^+$ $C_{17}H_{26}NaO_3Si$, m/z : 329.1543, found : 329.1540.
IR *v* [cm^{-1}] 2952, 2867, 1748, 1717, 1643, 1435, 1343, 1326, 1248, 1197, 1004, 837, 741.

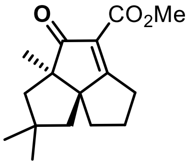

3g

Compound **3g** was synthesized following general procedure E, yellow oil, yield 82%. Reaction time: 12 h.
^{1}H NMR (400 MHz, $CDCl_3$) δ 3.80 (s, 3H), 3.06 – 2.97 (m, 1H), 2.89 – 2.81 (m, 1H), 2.15 – 2.12 (m, 1H), 2.06 – 1.99 (m, 2H), 1.73 (d, J = 13.2 Hz, 2H), 1.57 – 1.47 (m, 3H), 1.02 (s, 3H), 1.00 (s, 3H), 0.80 (s, 3H).
^{13}C NMR (100 MHz, $CDCl_3$) δ 210.3, 203.1, 163.2, 123.9, 64.5, 59.9, 51.6, 51.5, 51.3, 38.9, 34.0, 31.3, 28.6, 27.3, 23.7, 20.5.
HRMS (ESI) calcd for $[M+Na]^+$ $C_{16}H_{22}NaO_3$, m/z : 285.1461, found : 285.1463.
IR *v* [cm^{-1}] 2953, 2930, 2867, 1747, 1709, 1640, 1436, 1345, 1333, 1286, 1218, 1156, 1023, 1004, 809, 747.

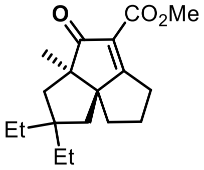

3h

Compound **3h** was synthesized following general procedure E, yellow oil, yield 85%. Reaction time: 12 h.
^{1}H NMR (400 MHz, $CDCl_3$) δ 3.79 (s, 3H), 3.04-2.95 (m, 1H), 2.89 – 2.80 (m, 1H), 2.23 (d, J = 13.6 Hz, 1H), 2.08 – 2.00 (m, 2H), 1.77 – 1.69 (m, 2H), 1.54 – 1.46 (m, 2H), 1.40 – 1.22 (m, 3H), 1.09 (q, J = 7.2 Hz, 2H), 1.02 (s, 3H), 0.73 (t, J = 7.6 Hz, 3H), 0.63 (t, J = 7.6 Hz, 3H).
^{13}C NMR (100 MHz, $CDCl_3$) δ 210.2, 203.4, 163.3, 123.4, 63.7, 59.5, 51.6, 48.0, 47.4, 45.6, 33.9, 31.7, 28.2, 27.5, 23.7, 20.7, 8.8, 8.5.
HRMS (ESI) calcd for $[M+Na]^+$ $C_{18}H_{26}NaO_3$, m/z : 313.1774, found : 313.1775.
IR *v* [cm^{-1}] 2962, 2877, 1748, 1710, 1642, 1459, 1436, 1346, 1333, 1287, 1233, 1211, 1024, 1008, 804, 723.

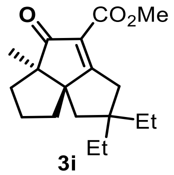

3i

Compound **3i** was synthesized following general procedure E, yellow oil, yield 80%. Reaction time: 12 h.

^{1}H NMR (400 MHz, $CDCl_3$) δ 3.81 (s, 3H), 3.04 (d, J = 17.6 Hz, 1H), 2.61 (d, J = 17.6 Hz, 1H), 2.12 (dd, J = 12.0, 5.6 Hz, 1H), 2.03 (dd, J = 12.8, 6.4 Hz, 1H), 1.69 – 1.59 (m, 4H), 1.55 – 1.49 (m, 2H), 1.35 – 1.24 (m, 3H), 1.12 – 1.04 (m, 1H), 1.03 (s, 3H), 0.89 (t, J = 7.4 Hz, 3H), 0.76 (t, J = 7.4 Hz, 3H).

^{13}C NMR (100 MHz, $CDCl_3$) δ 209.6, 200.5, 163.1, 125.1, 61.5, 59.7, 51.6, 46.3, 42.9, 42.0, 39.5, 37.9, 32.5, 32.0, 23.1, 19.5, 9.1, 8.4.

HRMS (ESI) calcd for $[M+Na]^+$ $C_{18}H_{26}NaO_3$, m/z : 313.1774, found : 313.1773.

IR ν [cm^{-1}] 2958, 2865, 1747, 1712, 1643, 1456, 1435, 1347, 1321, 1239, 1229, 1119, 1004, 799, 741.

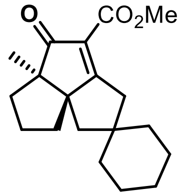

3j

Compound **3j** was synthesized following general procedure E, yellow oil, yield 75%. Reaction time: 12 h.

^{1}H NMR (400 MHz, $CDCl_3$) δ 3.81 (s, 3H), 3.08 (d, J = 18.0 Hz, 1H), 2.65 (d, J = 18.4 Hz, 1H), 2.15 – 2.00 (m, 2H), 1.75 – 1.36 (m, 12H), 1.34 – 1.23 (m, 3H), 1.13 – 1.04 (m, 1H), 1.02 (s, 3H).

^{13}C NMR (100 MHz, $CDCl_3$) δ 209.6, 200.5, 163.1, 125.2, 61.5, 59.7, 51.6, 43.5, 41.7, 40.3, 40.0, 37.8, 25.6, 23.5, 23.2, 23.1, 19.4.

HRMS (ESI) calcd for $[M+Na]^+$ $C_{19}H_{26}NaO_3$, m/z : 325.1774, found : 325.1774.

IR ν [cm^{-1}] 2926, 2856, 1747, 1713, 1643, 1448, 1435, 1340, 1327, 1226, 1200, 1005, 800, 742.

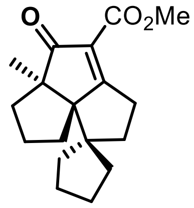

3k

Compound **3k** was synthesized following general procedure E from the mixture of **4k1** and **4k2**, yellow oil, yield 75%. Reaction time: 48 h at 50 ºC.

¹H NMR (500 MHz, $CDCl_3$) δ 3.81 (s, 3H), 3.03 – 2.96 (m, 1H), 2.92 – 2.86 (m, 1H), 2.08 – 2.04 (m, 1H), 2.00 – 1.86 (m, 3H), 1.80 – 1.68 (m, 5H), 1.69 – 1.55 (m, 3H), 1.50 – 1.40 (m, 2H), 1.22 (s, 3H), 1.11 – 1.00 (m, 2H).

¹³C NMR (125 MHz, $CDCl_3$) δ 209.5, 203.5, 162.9, 125.6, 66.9, 57.6, 54.0, 51.5, 42.1, 41.0, 35.1, 34.6, 34.0, 26.8, 25.7, 25.5, 21.0, 19.2.

HRMS (ESI) calcd for $[M+Na]^+$ $C_{18}H_{24}NaO_3$, m/z : 311.1618, found : 311.1622.

IR v [cm^{-1}] 2950, 2869, 1747, 1712, 1642, 1435, 1346, 1334, 1226, 1197, 1009, 803, 731.

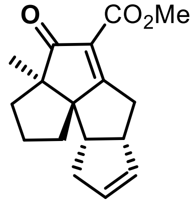

3l

Compound **3l** was synthesized following general procedure E from the mixture of **4l1** and **4l2**, yellow oil, yield 50%. Reaction time: 12 h.

¹H NMR (400 MHz, $CDCl_3$) δ 5.82 – 5.81 (m, 1H), 5.66 – 5.65 (m, 1H), 3.79 (s, 3H), 3.44 – 3.43 (m, 1H), 3.03 (dd, J = 20.0, 10.4 Hz, 1H), 2.80 – 2.68 (m, 2H), 2.33 (dd, J = 15.6, 8.8 Hz, 1H), 2.09 (dd, J = 10.4, 5.6 Hz, 1H), 1.95 – 1.83 (m, 3H), 1.76 – 1.68 (m, 1H), 1.55 – 1.45 (m, 2H), 1.14 (s, 3H).

¹³C NMR (100 MHz, $CDCl_3$) δ 209.2, 200.9, 162.7, 133.3, 131.5, 126.0, 66.5, 57.4, 51.5, 51.4, 49.7, 40.8, 40.3, 34.6, 33.6, 22.2, 19.5.

HRMS (ESI) calcd for $[M+Na]^+$ $C_{17}H_{20}NaO_3$, m/z : 295.1305, found : 295.1304.

IR v [cm^{-1}] 2948, 2867, 1747, 1715, 1641, 1436, 1347, 1328, 1281, 1239, 1223, 1007, 727, 697.

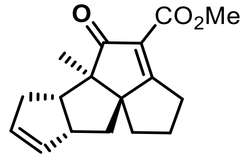

3m

Compound **3m** was synthesized following general procedure E from the mixture of **4m1** and **4m2**, white solid, yield 87%. Reaction time: 12 h.

¹H NMR (400 MHz, $CDCl_3$) δ 5.76 – 5.58 (s, 2H), 3.81 (s, 3H), 3.05 – 2.80 (m, 4H), 2.46 – 2.24 (m, 2H), 2.11 – 1.98 (m, 2H), 1.92 (dd, J = 13.6, 8.4 Hz, 1H), 1.72 – 1.66 (m, 1H), 1.59 – 1.46 (m, 2H), 1.00 (s, 3H).

^{13}C NMR (100 MHz, $CDCl_3$) δ 210.0, 203.2, 163.3, 134.8, 129.9, 122.1, 66.4, 62.7, 51.6, 50.9, 47.9, 44.0, 34.0, 32.8, 27.9, 24.3, 17.6.
HRMS (ESI) calcd for $[M+Na]^+$ $C_{17}H_{20}NaO_3$, m/z : 295.1305, found : 295.1304.
IR v [cm^{-1}] 2930, 2851, 1745, 1713, 1641, 1435, 1346, 1293, 1226, 1008.
Mp 51.5 – 51.9 °C.

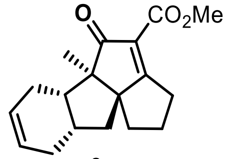

3n

Compound **3n** was synthesized following general procedure E from the mixture of **4n₁** and **4n₂**, yellow oil, yield 71%. Reaction time: 12 h.
^{1}H NMR (400 MHz, $CDCl_3$) δ 5.58 – 5.57 (m, 2H), 3.81 (s, 3H), 2.97 – 2.93 (m, 2H), 2.30 – 2.17 (m, 2H), 2.11 – 2.02 (m, 3H), 1.86 – 1.75 (m, 3H), 1.72 – 1.54 (m, 4H), 0.95 (s, 3H).
^{13}C NMR (100 MHz, $CDCl_3$) δ 210.2, 203.0, 163.1, 124.8, 124.2, 124.2, 63.6, 62.6, 51.6, 41.0, 40.1, 34.1, 31.5, 27.7, 25.3, 24.4, 22.3, 14.5.
HRMS (ESI) calcd for $[M+Na]^+$ $C_{18}H_{22}NaO_3$, m/z : 309.1461, found : 309.1461.
IR v [cm^{-1}] 2952, 2929, 2900, 2880, 1742, 1709, 1640, 1434, 1347, 1333, 1294, 1222, 1005.

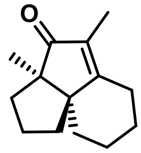

3o

Compound **3o** was synthesized following general procedure E, colorless oil, yield 85%. Reaction time: 5 mins
^{1}H NMR (500 MHz, $CDCl_3$): δ 2.77 – 2.72 (m, 1H), 2.26 – 2.18 (m, 1H), 2.02 – 1.91 (m, 3H), 1.79 – 1.68 (m, 2H), 1.65 (d, J = 1.0Hz, 3H), 1.61 – 1.53 (m, 1H), 1.48 – 1.42 (m, 1H), 1.39 – 1.23 (m, 4H), 0.98 (s, 3H), 0.96 – 0.85 (m, 1H).
^{13}C NMR (125 MHz, $CDCl_3$): δ 214.2, 175.8, 132.3, 55.9, 55.2, 38.1, 36.3, 26.9, 25.8, 23.0, 22.5, 18.5, 7.9.
HRMS (ESI) calcd for $[M+Na]^+$ $C_{14}H_{20}NaO$, m/z : 227.1406, found : 227.1407.
IR v [cm^{-1}] 2933, 2858, 1698, 1650, 1444,1322, 1030, 991.

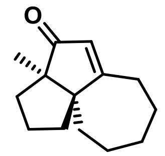

3p

Compound **3p** was synthesized following general procedure E, colorless oil, yield 70%. Reaction time: 12 h at 50 ºC.

^{1}H NMR (500 MHz, $CDCl_3$): δ 5.95 (s, 1 H), 2.80 – 2.76 (m, 1H), 2.32 – 2.27 (m, 1H), 2.02 – 1.90 (m, 3H), 1.85 – 1.77 (m, 2H), 1.66 – 1.62 (m, 1H), 1.52 – 1.44 (m, 2H), 1.43 – 1.35 (m, 3H), 1.33 – 1.25 (m, 2H), 1.21 – 1.17 (m, 1H), 1.04 (s, 3H).

^{13}C NMR (125 MHz, $CDCl_3$): δ 215.2, 187.8, 129.9, 59.6, 57.9, 39.3, 38.3, 34.4, 31.2, 30.6, 29.7, 25.0, 22.4, 18.7.

HRMS (ESI) calcd for $[M+Na]^+$ $C_{14}H_{20}NaO$, m/z : 227.1406, found : 227.1406.

IR ν [cm^{-1}] 2929, 2855, 1650, 1615, 1447, 1385, 1288, 1109, 1093, 983, 851.

5. Optimization of diastereoisomeric ratio of 3c (β:α)

Supplementary Table 1. Optimization of diastereoisomeric ratio of 3c (β:α) [a]

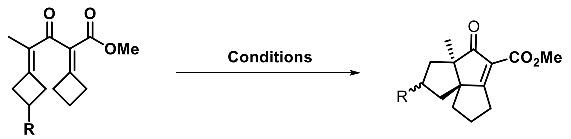

entry	temperature(℃)	solvent	additive[b]	dr (β: α)[c]	yield(%)[d]	time(h)
1	25	DCM	/	4.5:1	80	36
2	25	$CHCl_3$	/	4:1	81	12
3	25	HFIP	/	1.5:1	53	12
4	25	DCM:HFIP=1:1	/	4.7:1	78	36
5	25	$CHCl_3$:HFIP=1:1	/	3.5:1	80	12
6	25	DCM:$CHCl_3$=1:1	/	4:1	75	24
7	60	DCM:HFIP=1:1	/	1:1	32	24
8	0	DCM:HFIP=1:1	NaBArF	5.6:1	80	48
9	-10	DCM:HFIP=1:1	NaBArF	7.0:1	81	48
10	-10	$CHCl_3$:HFIP=1:1	NaBArF	6.5:1	80	48

[a] Reactions were carried out with 0.2 mmol of substrate and $In(SbF_6)_3$ (10mol %) in 2.0 mL solution. [b] with 0.6 equiv additives. [c] The ratio determined by ^{1}H-NMR. [d] Isolated yields.

6. Plausible mechanism and rationalization for stereo- and regio-selectivity

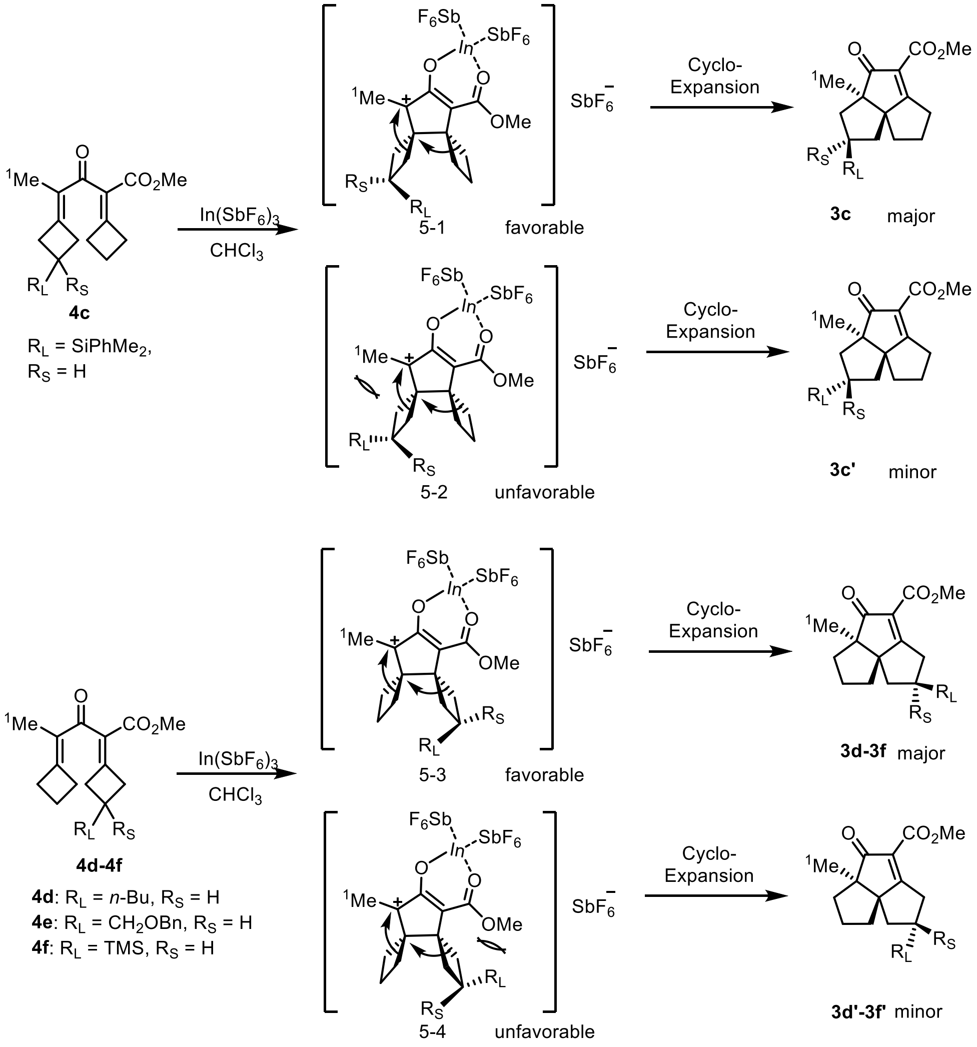

Supplementary Figure 2. Rationalization for stereoselectivity of *para*-mono substituent on cyclobutene.

According to the experimental results and literature reports, the possible origin for producing the diastereo-isomerization during reaction of *para*-mono substituted examples is that when **4c** underwent conrotatory Nazarov cyclization, 5-membered ring′s 2π-oxyallylic cation was formed in two possible intermediates **5-1** and **5-2**. Due to the steric-repulsion between C1-Me and R_L in **5-2**, **5-1** would be more stable than **5-2** and the subsequent two cycle-expansions should occur preferentially in **5-1** to give the dominant **3c**. The relative configuration of **3c** was confirmed by the single crystal X-ray diffraction analysis of its derivate **111**. Similarly, products **3d** to **3f** were also formed dominantly (**Supplementary Figure 2.**). The process is also confirmed with DFT calculations.

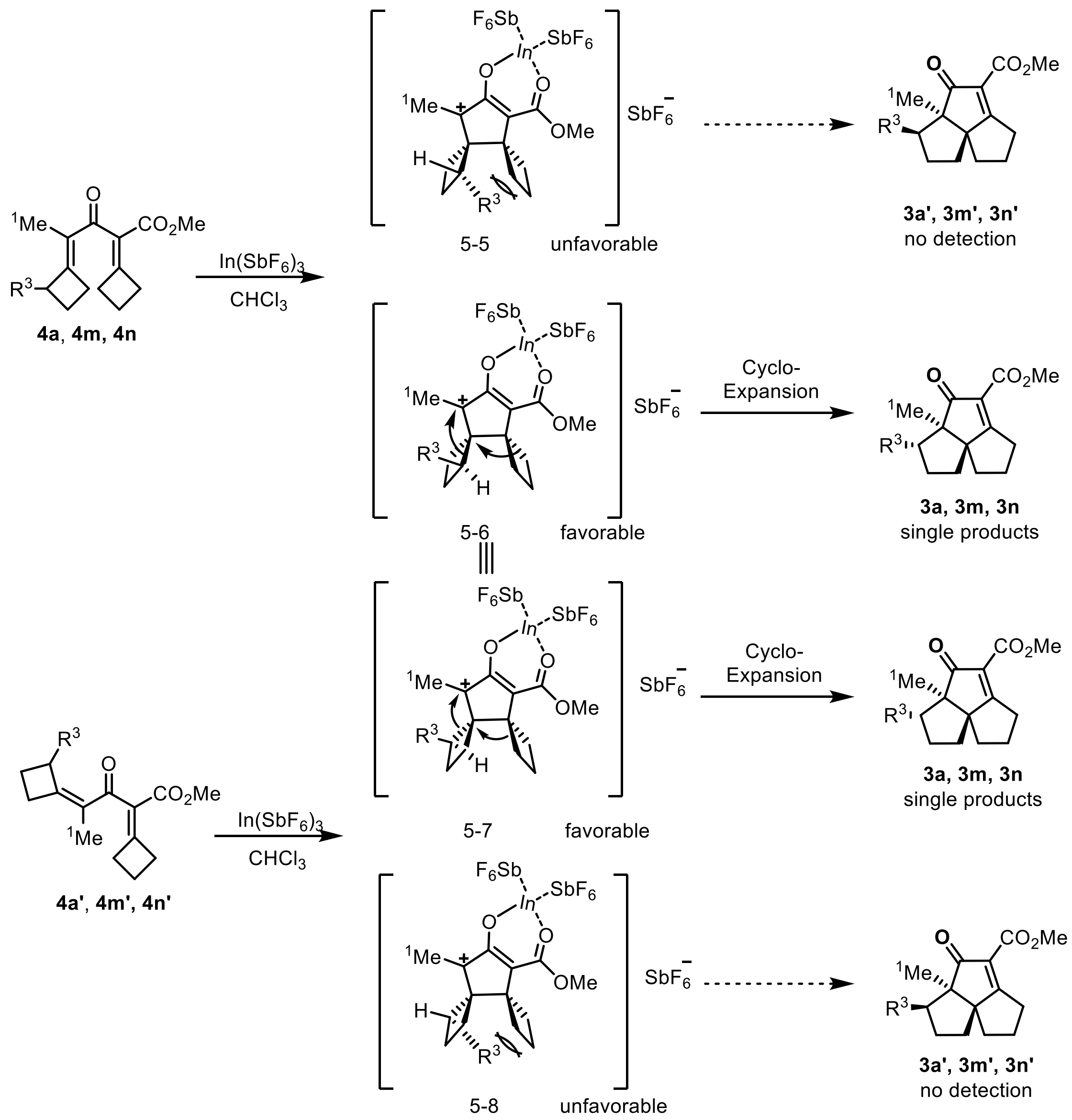

Supplementary Figure 3. Rationalization for regioselectivity of *orth*-mono substituent on first cyclo-expansioned cyclobutene.

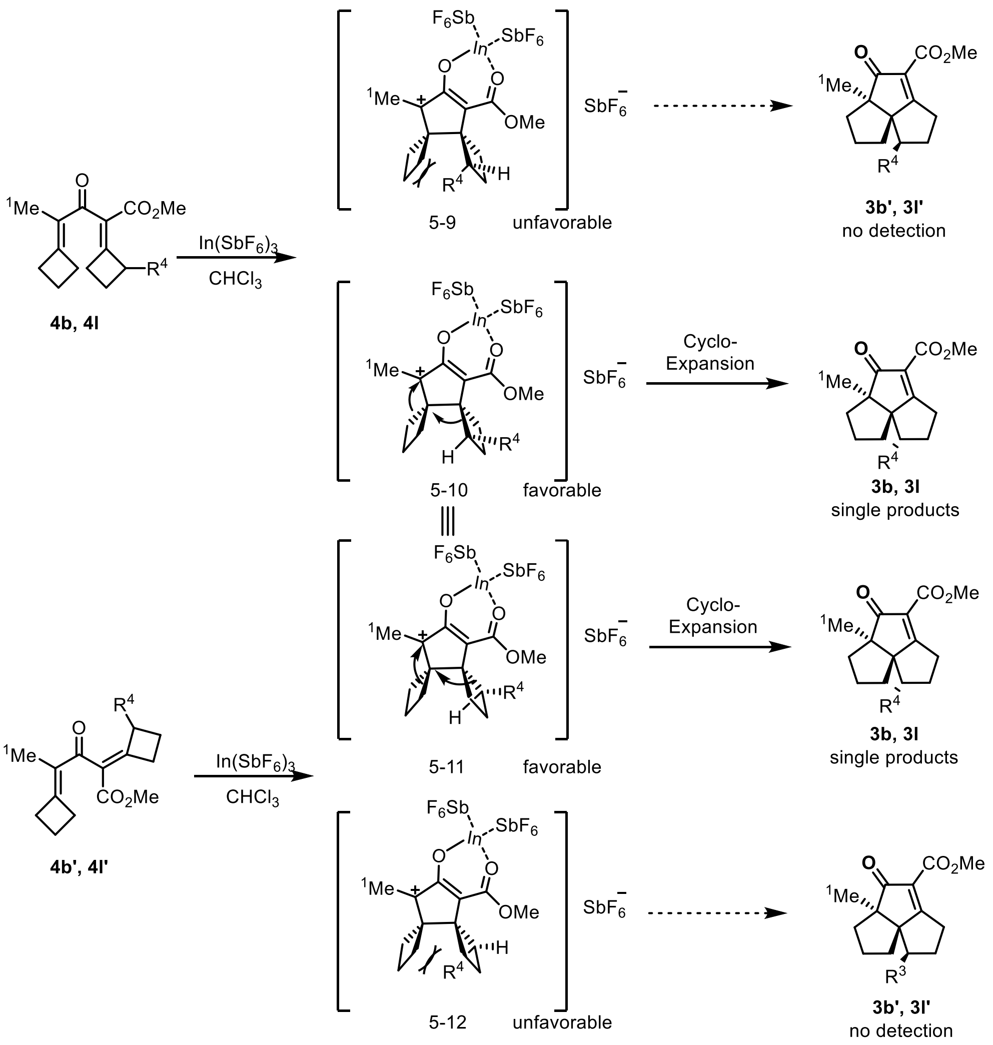

Supplementary Figure 4. Rationalization for regioselectivity control of *orth*-mono substituent on second cyclo-expansioned cyclobutene.

There are two main reasons why a mixture of *E/Z* isomers with *orth*-mono substituent always gave the single product (**Supplementary Figure 3**). First theoretically, there possibly were four 4π-electrocyclized intermediates **5-5** to **5-8**. However due to the stereo-repulsion between R_3 and another cyclobutyl ring, only one (**5-6** or **5-7**) was dominantly formed via E/Z isomerization and cyclization; Second, in states **5-6** or **5-7**, the migration of methine moiety was generally prior to the methylene, resulting in the formation of the single diastereomer **3a**, **3m** and **3n**. The relative configuration of **3m** was confirmed by the single crystal X-ray diffraction analysis. Similarly, products **3b**, **3k** and **3l** were also formed as single isomer in each case (**Supplementary Figure 4**). The relative configuration of **3b** was confirmed by the single crystal X-ray diffraction analysis of its derivate **114-1**. The process is also confirmed with DFT calculations.

7. Synthetic application

7.1 Synthesis of (±)-waihoensene

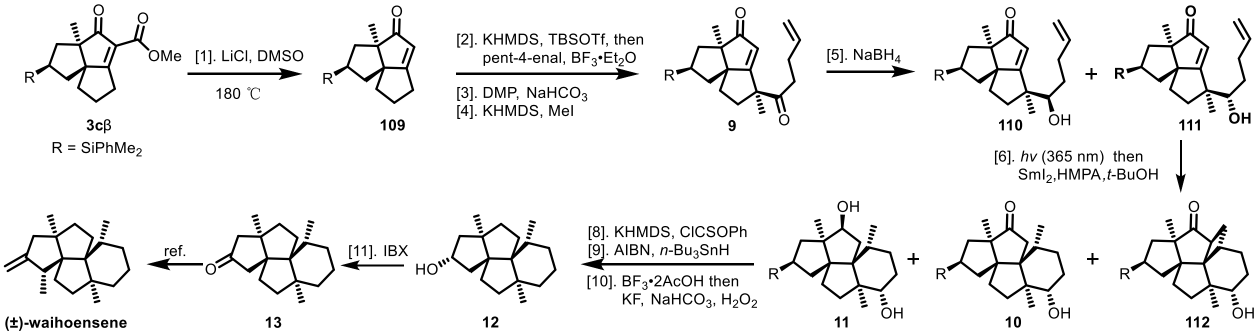

Supplementary Figure 5. An overview of the synthetic pathways on (±) waihoensene.

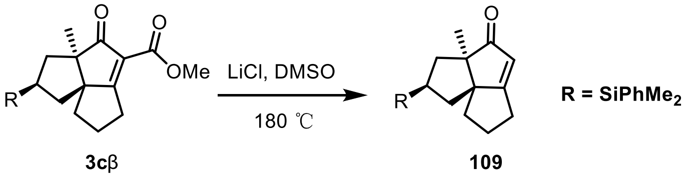

Compound **109**. Ene ester **3cβ** (1.38 g, 3.75 mmol, 1.0 equiv) was dissolved in DMSO (40 mL), and then LiCl (0.47 g, 11.25 mmol, 3.0 equiv) and H_2O (2.00 mL, 112.50 mmol, 30.0 equiv) were added. The mixture was stirred at 180 °C for 1 hour. The reaction mixture was cooled to rt, diluted with EtOAc (150 mL) and H_2O (50 mL), extracted with H_2O (2 × 50 mL). The combined organic layers were washed with brine (50 mL), dried over Na_2SO_4, filtered and concentrated in vacuum. The residue was purified by a flash column chromatography on silica gel (EtOAc: petroleum ether = 1:10) to give enone **109** (1.03g, 3.34 mmol, 89%).

^{1}H NMR (500 MHz, $CDCl_3$) δ 7.51 – 7.28 (m, 5H), 5.50 (s, 1H), 2.63 – 2.44 (m, 2H), 2.01 (m, 2H), 1.84 – 1.75 (m, 1H), 1.73 – 1.55 (m, 4H), 1.50 (dd, J = 21.0, 10.5 Hz, 1H), 1.17 (t, J = 13.0 Hz, 1H), 1.07 (s, 3H), 0.22 (s, 6H).

^{13}C NMR (125 MHz, $CDCl_3$) δ 216.80, 190.5, 137.9, 133.6, 128.9, 127.7, 118.7, 67.6, 61.2, 41.6, 37.7, 30.5, 29.0, 25.8, 24.1, 20.5, -4.7, -5.0.

HRMS (ESI) calcd for $[M+Na]^+$ $C_{20}H_{26}NaOSi$, m/z : 333.1645, found : 333.1646.

IR ν [cm^{-1}] 2954, 1698, 1626, 1427, 1249, 1112, 829, 810, 731, 699.

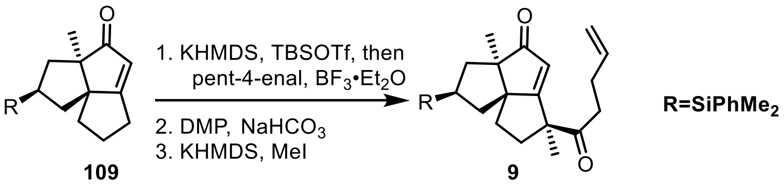

Compound **9**. KHMDS (7.00 mL, 1.0 M in toluene, 7.0 mmol, 1.5 equiv) was added to a solution

of enone **109** (1.46 g, 4.7mmol, 1.0 equiv) in THF (35 mL) at 0 °C. After the reaction mixture was stirred at 0 °C for 0.5 h, TBSOTf (1.60 mL, 7.0 mmol, 1.5 equiv) was added and the mixture was stirred for 10 minutes. The reaction was quenched with saturated aqueous $NaHCO_3$(20 mL) and allowed to warm to room temperature. The mixture was then extracted with PE (2×50 mL). The organic extracts were combined, washed with brine (50 mL), dried over Na_2SO_4, filtered, and concentrated in vacuum to a yellow oil that was used directly in the next step.

$BF_3 \cdot Et_2O$ (0.58 mL, 4.7 mmol, 1.0 equiv) was added to a solution of pent-4-enal (0.56 mL, 5.6 mmol, 1.2 equiv) and silyl ketene acetal in CH_2Cl_2 (35 mL) over a period of 30 min at -78 °C. The reaction mixture was stirred for another 30 min at this temperature. The reaction was quenched with saturated aqueous $NaHCO_3$ and allowed to warm to room temperature. The mixture was then extracted with EtOAc (3×50mL). The organic extracts were combined, washed with brine (50mL), dried over Na_2SO_4, filtered, and concentrated in vacuum to a yellow oil that was used directly in the next step.

$NaHCO_3$ (0.80 g, 9.5 mmol, 2.0 equiv) and DMP (3.00 g, 7 mmol, 1.5 equiv) were successively added to a solution of the above crude product in CH_2Cl_2 (15 mL) at 0 °C. The reaction mixture was stirred at 0 °C for 1 h, and then saturated aqueous $Na_2S_2O_3$ (100 mL) were successively added. The resultant solution was extracted with EtOAc (3 × 150mL), and the combined organic layers were washed with brine (75 mL), dried over Na_2SO_4, filtered, and concentrated. The residue was purified quickly by a flash column chromatography on silica gel (EtOAc: petroleum ether = 1:1) to give desired ketone product as a yellow oil. (Note that: the ketone product was unstable). To a stirred solution of KHMDS (5.60 mL, 1.0 M in toluene, 5.6 mmol, 1.2 equiv) was added a solution of the above ketone product in THF (45 mL) at -78 °C. The mixture was stirred for 30 min. To this solution was added a solution of MeI (2.90 mL, 47 mmol, 10.0 equiv) at -78 °C over 5 min, and the resultant mixture was stirred at rt for 30 min. The reaction mixture was quenched with addition of a saturated solution of NH_4Cl (50 mL), and the mixture was extracted with EtOAc (3 x 100 mL). The combined organic layers were washed with brine (50 mL), dried over Na_2SO_4, filtered, and concentrated. The residue was purified by a flash column chromatography on silica gel (EtOAc: petroleum ether = 1:20) to give enone **9** (0.67 g, 1.64 mmol, 35% for three steps) as a yellow oil.

^{1}H NMR (500 MHz, $CDCl_3$) δ 7.36 (m, 5H), 5.82 – 5.72 (m, 1H), 5.71 (s, 1H), 5.04 – 4.90 (m, 2H), 2.82 – 2.74 (m, 1H), 2.71 – 2.64 (m, 1H), 2.57 – 2.50 (m, 1H), 2.35 – 2.26 (m, 2H), 1.84 – 1.72 (m, 2H), 1.70 – 1.66 (m, 1H), 1.64 – 1.58 (m, 4H), 1.38 (s, 3H), 1.08 (s, 3H), 1.04 – 0.96 (m, 1H), 0.21 (s, 3H), 0.20 (s, 3H).

^{13}C NMR (125 MHz, $CDCl_3$) δ 215.8, 207.7, 190.8, 137.5, 136.7, 133.6, 129.0, 127.7, 120.7, 115.6, 68.7, 62.8, 57.1, 41.9, 37.4, 37.3, 35.5, 29.4, 28.6, 27.8, 25.0, 20.8, -4.9, -4.9.

HRMS (ESI) calcd for $[M+Na]^+$ $C_{26}H_{34}NaO_2Si$, m/z : 429.2220, found : 429.2221.

IR ν [cm^{-1}]3069, 2954, 2864, 1696, 1650, 1615, 1453, 1427, 1250, 1111, 838, 812, 733, 701.

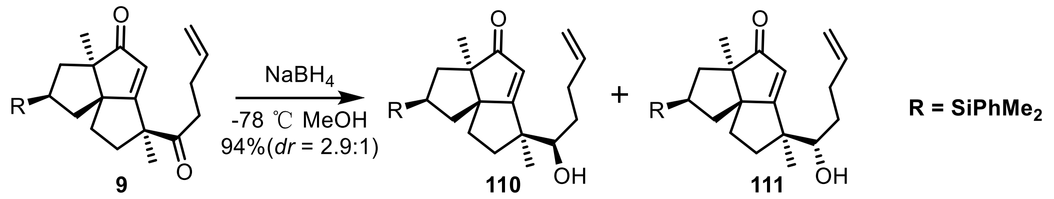

Compound **110** and **111**. To a 50 mL flask equipped with a magnetic stirring bar was added ketone **9** (330 mg, 0.82 mmol, 1.0 equiv) and 14 mL of methanol. The flask was placed at -78°C for 15 min. Then sodium borohydride (49 mg, 1.23 mmol, 1.5 equiv) was added in 3 portions and the mixture was stirred at -78°C for 8 h. After the reaction was completed, the reaction mixture was quenched with addition of a saturated solution of NH_4Cl (15 mL), and the mixture was extracted with EtOAc (5 x 50 mL). The combined organic layers were washed with brine, dried over Na_2SO_4, filtered, and concentrated. The residue was purified by a flash column chromatography on silica gel (EtOAc: petroleum ether = 1:6) to give **110** and **111** as colorless oil and white solid respectively (315mg, 94%, **66**: **67**=1:2.9).

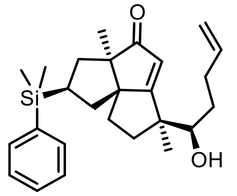

110

^{1}H NMR (500 MHz, $CDCl_3$) δ 7.51 – 7.40 (m, 2H), 7.39 – 7.29 (m, 3H), 5.87 – 5.72 (m, 1H), 5.53 (s, 1H), 5.11 – 4.94 (m, 2H), 3.61 (dd, J = 10.0, 3.0 Hz, 1H), 2.33 – 2.25 (m, 1H), 2.24 – 2.06 (m, 2H), 1.83 – 1.75 (m, 1H), 1.71 – 1.57 (m, 7H), 1.55 – 1.47 (m, 1H), 1.15 – 1.10 (m, 1H), 1.09 (s, 3H), 1.08 (s, 3H), 0.22 (s, 3H), 0.22 (s, 3H).
^{13}C NMR (125 MHz, $CDCl_3$) δ 216.6, 196.0, 138.1, 137.9, 133.7, 129.0, 127.8, 120.1, 115.4, 75.8, 69.1, 63.4, 49.2, 42.8, 37.0, 37.0, 31.5, 30.6, 29.5, 28.9, 21.9, 21.1, -4.7, -4.9.
HRMS (ESI) calcd for $[M+Na]^+$ $C_{26}H_{36}NaO_2Si$, m/z: 431.2377, found: 431.2377.
IR ν [cm^{-1}]3069, 2955, 2866, 1703, 1640, 1620, 1455, 1427, 1251, 1112, 829, 813, 733, 701.

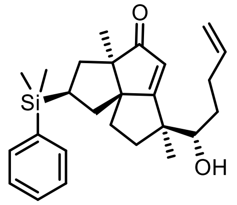

111

^{1}H NMR (500 MHz, $CDCl_3$) δ 7.51 – 7.40 (m, 2H), 7.40 – 7.29 (m, 3H), 5.91 – 5.78 (m, 1H), 5.58 (s, 1H), 5.10 – 4.95 (m, 2H), 3.49 – 3.36 (m, 1H), 2.40 – 2.31 (m, 1H), 2.19 – 2.09 (m, 1H), 1.95 – 1.87 (m, 1H), 1.78 – 1.68 (m, 2H), 1.67 – 1.52 (m, 7H), 1.16 (t, *J* = 12.5 Hz, 1H), 1.11 (s, 3H), 1.08 (s, 3H), 0.23 (s, 3H), 0.22 (s, 3H).
^{13}C NMR (125 MHz, $CDCl_3$) δ 216.6, 195.5, 138.2, 138.0, 133.7, 129.0, 127.8, 120.7, 115.2, 75.3, 68.9, 63.3, 48.7, 42.6, 37.4, 36.9, 31.0, 30.9, 29.7, 29.0, 21.1, 20.7, -4.7, -5.0.
HRMS (ESI) calcd for $[M+Na]^+$ $C_{26}H_{36}NaO_2Si$, m/z: 431.2377, found: 431.2379.
IR *ν* [cm^{-1}] 3069, 2926, 2855, 1715, 1463, 1249, 1114, 832, 813, 733, 700.
Mp 101.5 – 104.7 °C.

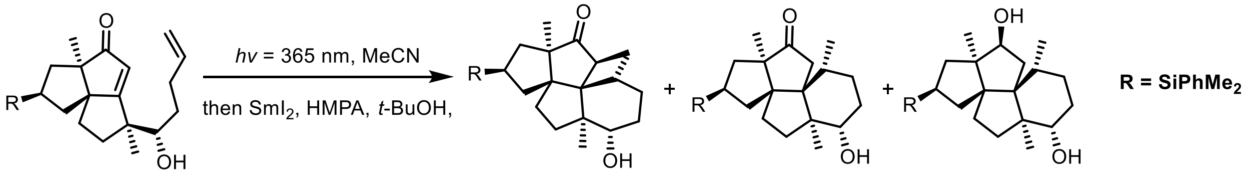

111 **112** **10** **11**

Compound **112**, **10** and **11**. The enone **112** (50.0 mg, 0.12 mmol, 1.0 equiv) was dissolved in 5 mL of MeCN and irradiated by blue LEDs (10W, 365nm) for 2 h at room temperature. The solvent was removed on a rotary evaporator to give the cyclobutane **112**. The residue was dissolved in THF (1 mL). The SmI_2–THF solution (36 mL, 0.1M in THF, 3.60 mmol, 30.0 equiv), *t*-BuOH (45 μL, 0.50 mmol, 4.0 equiv), and HMPA (0.42 mL, 2.40 mmol, 20.0 equiv) were successively added to the solution. After 8 hours, the reaction mixture was quenched with MeOH. 1M HCl was added to dissolve the samarium salts. The aqueous portion was extracted with EtOAc (5 x 50 mL), and the combined organic layers were washed with saturated aqueous $Na_2S_2O_3$, saturated aqueous $NaHCO_3$, and brine. The extracts were dried over Na_2SO_4, filtered, and concentrated under reduced pressure. The residue was purified by a flash column chromatography on silica gel (EtOAc: petroleum ether = 1:6 to 1:3) to give **112** (5.6mg, 11%), **10** (19.9 mg, 40%) and **11** (6.6mg, 13.2%) as yellow oils.

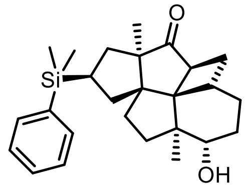

112

^{1}H NMR (500 MHz, $CDCl_3$) δ 7.53 – 7.44 (m, 2H), 7.38 – 7.31 (m, 3H), 3.60 (d, *J* = 6.5 Hz, 1H), 2.82 – 2.70 (m, 1H), 2.30 – 2.14 (m, 1H), 2.02 – 1.97 (m, 1H), 1.89 – 1.73 (m, 3H), 1.73 – 1.55

(m, 6H), 1.54 – 1.47 (m, 2H), 1.42 – 1.38 (m, 1H), 1.28 (dd, J = 12.0, 6.0 Hz, 1H), 1.14 (s, 3H), 1.12 – 1.04 (m, 1H), 1.00 (s, 3H), 0.29 (s, 6H).
^{13}C NMR (125 MHz, $CDCl_3$) δ 227.9, 137.8, 133.8, 129.0, 127.7, 74.2, 66.9, 62.6, 57.9, 49.4, 44.8, 42.7, 39.3, 34.4, 34.3, 33.1, 27.3, 26.8, 25.2, 25.2, 23.6, 21.1, -4.4, -4.5.
HRMS (ESI) calcd for $[M+Na]^+$ $C_{26}H_{36}NaO_2Si$, m/z : 431.2377, found : 431.2370.
IR ν [cm^{-1}] 3069, 2954, 2865, 1686, 1617, 1455, 1251, 1113, 913, 839, 813, 733, 700.

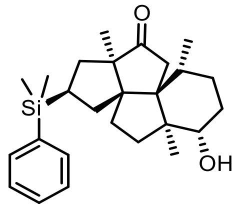

10

^{1}H NMR (500 MHz, $CDCl_3$) δ 7.48 – 7.44 (m, 2H), 7.36 – 7.31 (m, 3H), 3.58 – 3.42 (m, 1H), 2.31 (q, J = 18.4 Hz, 2H), 2.08 (t, J = 13.0 Hz,, 1H), 2.03 – 1.87 (m, 2H), 1.87 – 1.78 (m, 1H), 1.75 – 1.67 (m, 1H), 1.64 – 1.59 (m, 1H), 1.58 (d, J = 4.5 Hz, 1H), 1.54 – 1.47 (m, 2H), 1.47 – 1.35 (m, 4H), 1.18 (s, 3H), 1.08 – 0.99 (m, 1H), 0.94 – 0.87 (m, 3H), 0.65 (d, J = 6.5 Hz, 3H), 0.26 (s, 6H).
^{13}C NMR (125 MHz, $CDCl_3$) δ 224.1, 137.7, 133.8, 129.0, 127.7, 77.1, 67.4, 60.5, 54.8, 50.9, 43.5, 42.6, 40.7, 36.8, 33.3, 32.0, 31.2, 29.2, 26.4, 24.9, 21.5, 21.5, -4.4, -4.5.
HRMS (ESI) calcd for $[M+Na]^+$ $C_{26}H_{38}NaO_2Si$, m/z : 433.2533, found : 433.2530.
IR ν [cm^{-1}] 3068, 3048, 2954, 2930, 2874, 1725, 1461, 1375, 1248, 1177, 1114, 1058, 834, 813, 732, 700.

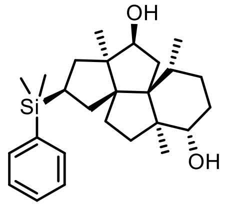

11

^{1}H NMR (500 MHz, $CDCl_3$) δ 7.53 – 7.43 (m, 2H), 7.42 – 7.30 (m, 3H), 3.82 (d, J = 11.5 Hz, 1H), 3.60 (s, 1H), 2.05 – 1.93 (m, 1H), 1.92 – 1.87 (m, 1H), 1.87 – 1.80 (m, 2H), 1.80 – 1.76 (m, 1H), 1.71 – 1.62 (m, 2H), 1.58 (d, J =5.0 Hz, 1H), 1.55 – 1.51 (m, 1H), 1.51 – 1.39 (m, 6H), 1.33 (s, 1H), 1.25 (s, 1H), 1.24 – 1.17 (m, 1H), 1.00 (d, J = 7.0 Hz, 3H), 0.97 (s, 3H), 0.96 (s, 3H), 0.24 (s, 3H), 0.23 (s, 3H).
^{13}C NMR (125 MHz, $CDCl_3$) δ 138.6, 133.8, 128.8, 127.6, 81.4, 76.9, 70.7, 55.8, 53.1, 48.3, 45.0, 42.5, 41.2, 37.5, 36.6, 32.0, 28.0, 27.1, 26.6, 25.1, 19.8, 18.6, -4.5, -4.6.
HRMS (ESI) calcd for $[M+Na]^+$ $C_{26}H_{40}NaO_2Si$, m/z : 435.2690, found : 435.2684.

IR *v* [cm^{-1}] 3068, 3048, 2959, 2934, 2873, 1456, 1378, 1249, 1113, 1034, 832, 813, 734, 700.

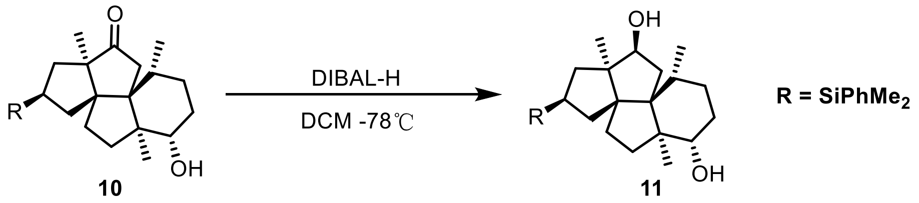

To a stirred solution of ketone **10** (29.1 mg, 0.07 mmol, 1.0 equiv) in CH_2Cl_2 (2 mL) was added DIBAL-H (0.4 mL, 1.0 M in hexane, 0.40 mmol, 6.0 equiv) at −78 °C under argon atmosphere. The reaction mixture was stirred at that temperature for 30 min before it was quenched with saturated aqueous potassium sodium tartrate (10 mL). The resultant mixture was diluted with EtOAc (20 mL) and stirred at 22 °C for 1h. The organic layer was separated, and the aqueous layer was extracted with EtOAc (9 × 30 mL). The combined organic phases were washed with brine (30 mL), dried over anhydrous Na_2SO_4, filtered, and concentrated under vacuum. The residue was subjected to a short plug of silica gel for purification using EtOAc/petroleum ether (1:6) as eluent to give the desired diol **11** (24.6 mg, 85%) as yellow oil.

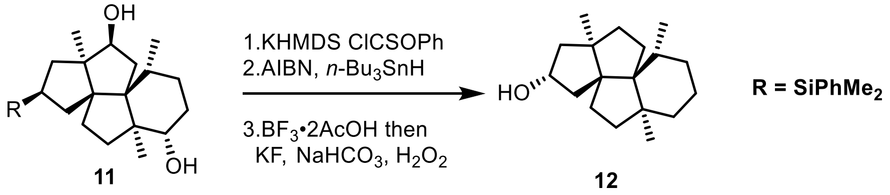

Compound **12**. To a solution of diol **11** (23.5 mg, 0.057 mmol, 1.0 equiv) in THF (3 mL) was added KHMDS (0.23 mL, 1 M in toluene, 0.23 mmol, 4.0 equiv) at -78°C under argon atmosphere. The solution stirred at -78°C for 30 min. This solution was added to ClCSOPh (31 µL, 0.23 mmol, 4.0 equiv) at -78°C, and the resultant mixture was stirred for 0.5 h at the same temperature. The reaction mixture was diluted with EtOAc (2 × 15 mL), washed with H_2O and brine, and dried over Na_2SO_4 and concentrated under vacuum. The residue was subjected to a short plug of silica gel for purification using EtOAc/petroleum ether (1:70) as eluent to give the corresponding xanthate as a yellow foam. The xanthate was dissolved in degassed toluene (2.5 mL). To this solution were sequentially added AIBN (3.7 mg, 0.02 mmol, 0.4 equiv) and *n*-Bu_3SnH (77 µL, 0.29 mmol, 5.0 equiv). The resulting mixture was heated at 110 °C for 1 h before it was cooled to 22 °C. After removal of the solvent, the residue was subjected to a short plug of silica gel using petroleum ether as eluent to give the tetracyclic core product. To a solution of tetracyclic core product in CH_2Cl_2 (2 mL) at 23 °C was added BF_3•2AcOH (38 µL, 0.27 mmol, 4.8 equiv). The resultant mixture was stirred at 23 °C for 2 h before it was quenched by being poured into saturated aqueous $NaHCO_3$ (2 mL) and extracted with EtOAc (3 × 10 mL). The combined organic phases were washed with

saturated aqueous $NaHCO_3$ and brine, dried over Na_2SO_4, filtered, and concentrated. Pressing forward without any further purification, the resultant residue was dissolved in THF/MeOH (2 mL, 1:1). Next, KF (33 mg, 0.57 mmol, 10 equiv), $NaHCO_3$ (48 mg, 0.57 mmol, 10 equiv) and 30% aqueous H_2O_2 (0.06 mL) were added sequentially at 23 °C. The resultant mixture was stirred at 23 °C for 12 h before it was quenched with the addition of saturated aqueous $Na_2S_2O_3$ (2 mL) and extracted with EtOAc (5 × 10 mL). The combined organic phases were washed with brine (10 mL), dried over Na_2SO_4, filtered, and concentrated. The resultant residue was purified by flash column chromatography silica gel (EtOAc/hexane, 1:6) to give alcohol **12** (6.0 mg, 40% yield over the two steps) as a pale white foam.

^{1}H NMR (500 MHz, $CDCl_3$) δ 4.21 – 4.13 (m, 1H), 1.99 – 1.91 (m, 2H), 1.91 – 1.80 (m, 2H), 1.77 – 1.67 (m, 2H), 1.52 – 1.45 (m, 2H), 1.45 – 1.36 (m, 7H), 1.36 – 1.29 (m, 2H), 1.18 – 1.13 (m, 1H), 1.10 (dd, *J* = 10.5, 5.0 Hz, 1H), 1.05 (d, *J* = 7.0 Hz, 3H), 1.00 (s, 6H).
^{13}C NMR (125 MHz, $CDCl_3$) δ 73.9, 64.1, 58.7, 51.7, 51.3, 51.3, 42.8, 41.0, 40.9, 36.0, 35.2, 33.1, 31.4, 30.53, 26.6, 23.7, 18.8, 16.8.
HRMS (ESI) calcd for $[M+Na]^+$ $C_{18}H_{30}NaO$, m/z : 285.2189, found : 285.2191.
IR ν [cm^{-1}] 2934, 2864, 1463, 1063.

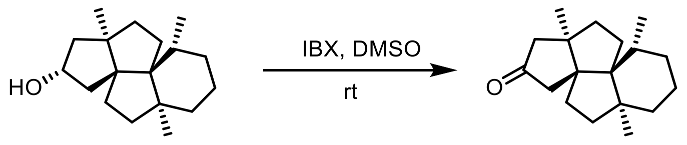

12 **13**

Compound **13**. To a solution of **12** (3.0 mg, 0.01 mmol, 1.0 equiv) in DMSO (0.8 mL) at 23 °C was added IBX (8.4 mg, 0.03 mmol, 3.0 equiv). The resultant mixture was stirred at 23 °C for 6 h. Upon completion, the reaction contents were diluted with EtOAc (3× 10 mL) and washed with water (5 mL) and brine (5 mL), dried (MgSO4), filtered, and concentrated. The resultant residue was purified by flash column chromatography (silica gel, EtOAc/hexane, 1:15) to give ketone **13** (2.7 mg, 90% yield) as a white powder. ^{1}H NMR spectrum and ^{13}C NMR matched that reported by the previous works [8] [9] [10].

^{1}H NMR (500 MHz, C_6D_6) δ 2.47 (d, *J* = 20.0 Hz, 1H), 2.06 (d, *J* = 18.5 Hz, 1H), 1.97 – 1.88 (m, 2H), 1.53 – 1.46 (m, 2H), 1.46 – 1.40 (m, 2H), 1.38 – 1.30 (m, 4H), 1.26 – 1.22 (m, 1H), 1.21 – 1.16 (m, 1H), 1.16 – 1.06 (m, 3H), 1.06 – 1.04 (m, 1H), 0.94 – 0.90 (m, 1H), 0.89 (s, 3H), 0.78 (s, 3H), 0.66 (d, *J* = 7.0 Hz, 3H).

^{13}C NMR (125 MHz, C_6D_6) δ 216.8, 62.9, 58.9, 53.6, 49.1, 49.1, 45.6, 42.1, 40.1, 34.6, 34.2, 32.8, 28.8, 28.2, 24.7, 24.1, 19.3, 17.9.
HRMS (ESI) calcd for $[M+Na]^+$ $C_{18}H_{28}NaO$, m/z : 283.2032, found : 283.2037.
IR ν [cm^{-1}] 2963, 2925, 2867, 1740, 1262, 1020.

7.2 Synthesis of (±)-*epi*-isocomene

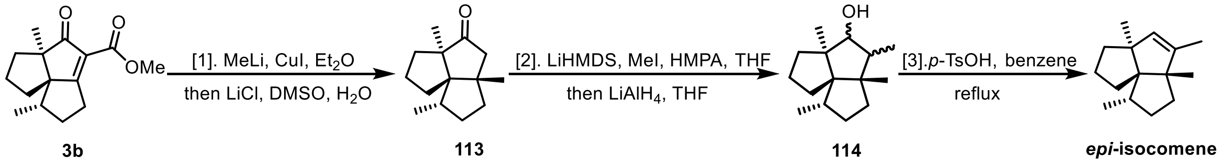

Supplementary Figure 6. An overview of the synthetic pathways on (±)-*epi*-isocomene.

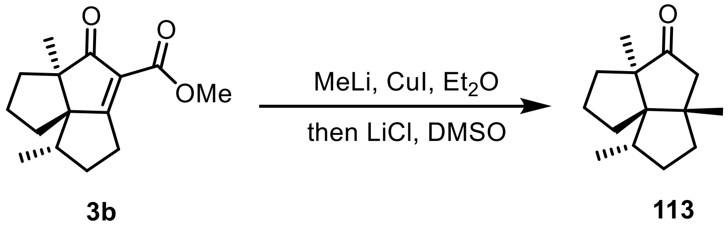

Compound **113**. CuI (380 mg, 2.0 mmol, 2.0 equiv) and 1.5 mL ether was added to the flamed flask. The mixture was cooled to 0 °C for 10 minutes. To the stirred solution was added MeLi (2.5 mL, 4.0 mmol, 1.6 M in ether, 4.0 equiv), and the reaction mixture was kept in 0 °C for 10 minutes before the flask was cooled to -78 °C for another 10 minutes. Then the solution of **3b** (248 mg, 1.0 mmol, 1.0 equiv) in 1.0 mL ether was added to the flask at -78 °C and stirred for 1 h at the same temperature. The reaction was determined by TLC until the starting materials converted completely. Then the reaction was quenched by saturated NH_4Cl, and extracted with EtOAc. The organic layer was washed with brine and dried over Na_2SO_4. The organic phase was concentrated under reduced pressure for next step without further purification.
The previous crude product was dissolved in DMSO at room temperature. Then H_2O (540 mg, 30.0 mmol, 30.0 equiv) and LiCl (126 mg, 3.0 mmol, 3.0 equiv) as added to the flask. The mixture was heated to 180 °C for 1 h. The reaction was determined by TLC until the starting materials converted completely. Then the reaction was quenched by water and extracted with EtOAc for three times. The organic layer was washed with brine for three times and dried over Na_2SO_4. The organic phase was concentrated under reduced pressure and purified by column chromatography (petroleum ether: EtOAc = 200:1) to afford **113** (179 mg) as white solid in 87% yield for two steps.
^{1}H NMR (400 MHz, $CDCl_3$) δ 2.36-2.31 (m, 1H), 2.27-2.16 (m, 1H), 2.14-2.10 (m, 1H), 1.98-1.80 (m, 3H), 1.73-1.60 (m, 3H), 1.58-1.29 (m, 4H), 1.25 (s, 3H), 1.15 (d, J = 7.2 Hz, 3H), 1.11 (s, 3H).

^{13}**C NMR** (100 MHz, $CDCl_3$) *δ* 225.1, 64.3, 60.2, 52.0, 47.2, 42.9, 42.3, 39.5, 34.4, 30.6, 24.6, 24.1, 21.0, 14.6.
HRMS (ESI) calcd for $[M+Na]^+$ $C_{14}H_{22}NaO$, m/z : 229.1563, found : 229.1563.
IR *ν* [cm^{-1}] 2948, 2872, 1734, 1472, 1381, 1084.

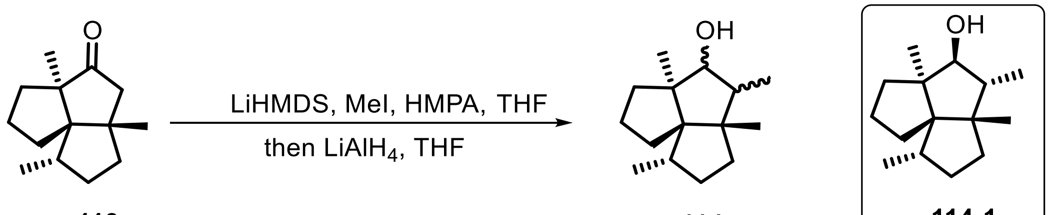

113 114

Compound **114**. Compound **113** (179 mg, 0.87 mmol, 1.0 equiv) was dissolved in 4.0 mL THF at -78 °C and HMPA (227μL, 1.30 mmol, 1.5 equiv) was added to the flask. The mixture was stirred at -78 °C for 5 minutes before LiHMDS (1.3 mL, 1.30 mmol, 1.0 M in THF, 1.5 equiv) was added dropwise to the flask. Then the reaction was stirred for 1 h at the same temperature, then MeI (272μL, 4.35 mmol, 5.0 equiv) was added to the mixture and stirred for another 10 minutes. The reaction flask was moved to room temperature and stirred overnight. The reaction was determined by TLC until the starting materials converted basically. Then the reaction was quenched by saturated NH_4Cl, and extracted with EtOAc. The organic layer was washed with brine and dried over Na_2SO_4. The organic phase was concentrated under reduced pressure for next step without further purification.
$LiAlH_4$ (66 mg, 1.74 mmol, 2.0 equiv) and 3.0 mL THF were added to a flamed flask which was put into 0 °C. To the stirred mixture was added solution of previous crude product in 1.0 mL THF and stirred for 1 h. The reaction was determined by TLC until the starting materials converted completely. Then the reaction was quenched by 10% NaOH and EtOAc was added. The mixture was stirred for at least 3 h. After that, the reaction was extracted with ethyl over Na_2SO_4. The organic phase was concentrated under reduced pressure and purified by column chromatography (petroleum ether : EtOAc = 100:1) to afford **114** (89 mg) as white solid with 4:1 *dr* in 46% yield for two steps. Though there is a mixture of two diastereomers, we utilized the mixture for cultivation of single crystal, fortunately, one of them (**114-1**) was confirmed by single crystal X-ray diffraction analysis.
^{1}**H NMR** (400 MHz, $CDCl_3$) *δ* 3.22 (d, *J* = 10.8 Hz, 0.80H), 3.08 (d, *J* = 12.0 Hz, 0.20H), 1.94-1.62 (m, 4H), 1.61-1.42 (m, 4H), 1.42-1.17 (m, 4H), 1.11 (m, 6H), 0.94 (d, *J* = 6.8 Hz, 2.40H), 0.90 (d, *J* = 6.8 Hz, 0.60H), 0.87 (s, 2.40H), 0.78 (s, 0.60H).

^{13}C NMR (100 MHz, $CDCl_3$) δ 85.2, 79.9, 69.6, 63.7, 54.8, 54.1, 50.5, 49.3, 47.9, 47.1, 46.0, 43.7, 40.0, 39.8, 37.5, 36.2, 35.8, 32.6, 32.1, 31.7, 25.0, 24.9, 24.4, 23.4, 20.7, 16.6, 14.9, 14.2, 12.4, 12.0.
HRMS (ESI) calcd for $[M+Na]^+$ $C_{15}H_{26}NaO$, m/z : 245.1876, found : 245.1875.
IR *v* [cm^{-1}] 3378, 2954, 2870, 1449, 1379, 1049.
Mp for **70-1**: 103.2 – 104.0 °C.

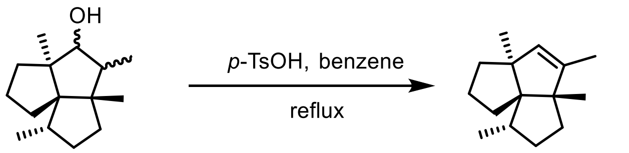

114 **_epi_-isocomene**

Compound **(±)-_epi_-isocomene**. **114** (20.0 mg, 0.09 mmol, 1.0 equiv) was dissolved in 1.0 mL benzene at room temperature and *p*-TsOH (15.5 mg, 0.09 mmol, 1.0 equiv) was added to the flask. The reaction mixture was stirred at 85 °C and the reaction was determined by TLC until the starting materials converted completely. The residue was purified by column chromatography (petroleum ether) to afford (±)-*epi*-isocomene (15.4 mg) as colorless oil in 84% yield.
Note that: The ^{1}H NMR and ^{13}C NMR spectrum of (±)-*epi*-isocomene was compared with the previous reports [11] [12] by Prof. Dr. Hudlický. Herein, we gratefully acknowledged kind help from Prof. Dr. Hudlický, because he provided us the old files of ^{1}H NMR and ^{13}C NMR spectrum for (±)-*epi*-isocomene and made a detailed explanation. It is worth mentioning that the old files have been kept intact for 36 years! His rigorous and scrupulous attitudes towards work and academics are admiring.

^{1}H NMR (400 MHz, $CDCl_3$) δ 4.72 (s, 1H), 1.76-1.59 (m, 4H), 1.57 (d, *J* = 1.6 Hz, 3H), 1.55-1.39 (m, 6H), 1.34-1.28 (m, 1H), 1.20 (s, 3H), 1.11 (d, *J* = 7.6 Hz, 3H), 0.96 (s, 3H).
^{13}C NMR (100 MHz, $CDCl_3$) δ 143.0, 133.8, 63.5, 60.1, 57.3, 44.0, 43.4, 38.0, 35.2, 32.0, 25.5, 23.7, 23.6, 14.5, 13.2.
MS (EI) m/z (%): 41 (15), 70 (15), 91 (26), 105 (28), 119 (47), 147 (66), 162 (100), 176 (13), 189 (13), 204(10).
IR *v* [cm^{-1}] 2939, 2866, 1676, 1444, 1378, 999.

Supplementary Table 2. **^{1}H NMR Spectroscopic (C_6D_6, 25 °C) Comparison of Yang's and Our Synthetic Ketone 13.**

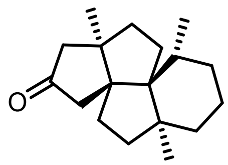

13

No.	Yang's<br>δ ^{1}H [ppm, mult, *J* (Hz)]	Ours<br>δ ^{1}H [ppm, mult, *J* (Hz)]
1	2.46 (d, *J* = 20.1 Hz, 1H)	2.47 (d, *J* = 20.0 Hz, 1H)
2	2.05 (d, *J* = 18.3 Hz, 1H)	2.06 (d, *J* = 18.5 Hz, 1H)
3	1.98 – 1.86 (m, 2H)	1.97 – 1.88 (m, 2H)
4	1.52 – 1.44 (m, 2H)	1.53 – 1.46 (m, 2H)
5	1.44 – 1.39 (m, 2H)	1.46 – 1.40 (m, 2H)
6	1.38 – 1.30 (m, 4H)	1.38 – 1.30 (m, 4H)
7	1.23 (ddd, *J* = 12.2, 6.1, 2.5 Hz, 1H)	1.26 – 1.22 (m, 1H)
8	1.20 – 1.15 (m, 1H)	1.21 – 1.16 (m, 1H)
9	1.14 – 1.06 (m, 3H)	1.16 – 1.06 (m, 3H)
10	1.05 – 1.03 (m, 1H)	1.06 – 1.04 (m, 1H)
11	0.93 – 0.89 (m, 1H)	0.94 – 0.90 (m, 1H)
12	0.89 (s, 3H)	0.89 (s, 3H)
13	0.77 (s, 3H)	0.78 (s, 3H)
14	0.65 (d, *J* = 6.8 Hz, 3H)	0.66 (d, *J* = 7.0 Hz, 3H).

Supplementary Table 3. ^{13}C NMR Spectroscopic (C_6D_6, 25 °C) Comparison of Yang's and Our Synthetic ketone 13.

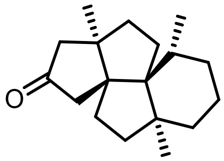

13

No.	Yang's δ ^{13}C (ppm)	Ours δ ^{13}C (ppm)
1	216.9	216.8
2	62.9	62.9
3	58.9	58.9
4	53.6	53.6
5	49.1	49.1
6	49.1	49.1
7	45.6	45.6
8	42.1	42.1
9	40.1	40.1
10	34.6	34.6
11	34.2	34.2
12	32.7	32.8
13	28.8	28.8
14	28.1	28.2
15	24.7	24.7
16	24.1	24.1
17	19.3	19.3
18	17.9	17.9

Supplementary Table 4. **^{1}H NMR Spectroscopic ($CDCl_3$, 25 °C) Comparison of Hudlický′s and Our Synthetic (±)-*Epi*-Isocomene.**

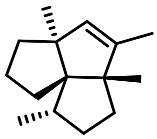

***epi*-isocomene**

<table>
<tr><th>No.</th><th>Hudlický’s work
δ ^{1}H [ppm, mult, J (Hz)]</th><th>Our work ($CDCl_3$, 400 MHz)
δ ^{1}H [ppm, mult, J (Hz)]</th></tr>
<tr><td>1</td><td>4.72 (s, 1H)</td><td>4.72 (s, 1H)</td></tr>
<tr><td>2</td><td rowspan="3">1.2-1.8 (m, 11H)</td><td>1.76-1.59 (m, 4H)</td></tr>
<tr><td>3</td><td>1.55-1.39 (m, 6H)</td></tr>
<tr><td>4</td><td>1.34-1.28 (m, 1H)</td></tr>
<tr><td>5</td><td>1.57 (d, J = 2.0 Hz, 3H)</td><td>1.57 (d, J = 1.6 Hz, 3H)</td></tr>
<tr><td>6</td><td>1.20 (s, 3H)</td><td>1.20 (s, 3H)</td></tr>
<tr><td>7</td><td>1.11 (d, J = 8.0 Hz, 3H)</td><td>1.11 (d, J = 7.6 Hz, 3H)</td></tr>
<tr><td>8</td><td>0.96 (s, 3H)</td><td>0.96 (s, 3H)</td></tr>
</table>

Supplementary Table 5. **^{13}C NMR Spectroscopic ($CDCl_3$, 25 °C) Comparison of Hudlický′s and Our Synthetic (±)-*Epi*-Isocomene.**

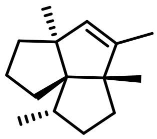

***epi*-isocomene**

No.	Hudlický's work<br>δ ^{13}C (ppm)	Our work ($CDCl_3$, 100 MHz)<br>δ ^{13}C (ppm)
1	143.6	143.0
2	133.8	133.8
3	63.6	63.5
4	60.4	60.1
5	57.3	57.3
6	44.1	44.0
7	43.5	43.4
8	38.0	38.0
9	35.2	35.2
10	32.0	32.0
11	25.5	25.5
12	23.8	23.7
13	23.6	23.6
14	14.5	14.5
15	13.2	13.2

8. X-ray crystallographic data of 1ff, 3m, 4n₂, 111 and 114-1

Supplementary Table 6. Crystal data and structure refinement for CCDC 2074121

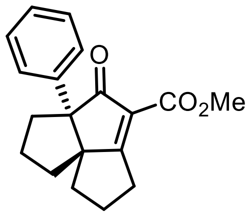

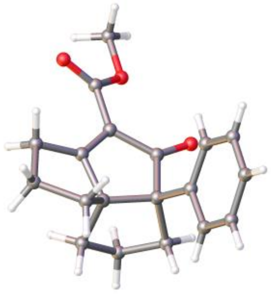

1ff **[X-ray for 1ff]**

Identification code	CCDC 2074121
Empirical formula	C19 H20 O3
Formula weight	296.35
Temperature	296(2) K
Wavelength	1.54178 A
Crystal system, space group	Orthorhombic, P2(1)2(1)2(1)
Unit cell dimensions	a = 7.8259(6) A alpha = 90 deg.<br>b = 11.6413(9) A beta = 90 deg.<br>c = 16.9665(12) A gamma = 90 deg.
Volume	1545.7(2) A^3
Z, Calculated density	4, 1.273 Mg/m^3
Absorption coefficient	0.681 mm^-1
F(000)	632
Crystal size	0.240 x 0.220 x 0.200 mm

Theta range for data collection	4.606 to 68.585 deg.
Limiting indices	-9<=h<=9, -14<=k<=13, -20<=l<=20
Reflections collected / unique	21649 / 2819 [R(int) = 0.0449]
Completeness to theta = 67.679	99.0 %
Refinement method	Full-matrix least-squares on F^2
Data / restraints / parameters	2819 / 0 / 201
Goodness-of-fit on F^2	1.003
Final R indices [I>2sigma(I)]	R1 = 0.0361, wR2 = 0.1168
R indices (all data)	R1 = 0.0376, wR2 = 0.1217
Absolute structure parameter	-0.04(7)
Extinction coefficient	0.017(3)
Largest diff. peak and hole	0.164 and -0.145 e.A^-3

Supplementary Table 7. Crystal data and structure refinement for CCDC 2074122

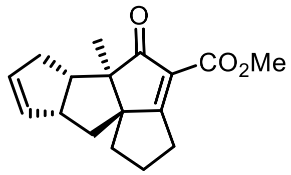

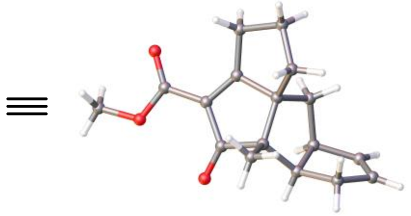

3m **[X-ray for 3m]**

Identification code	CCDC 2074122
Empirical formula	C17 H20 O3
Formula weight	272.33
Temperature	296(2) K
Wavelength	1.54178 A
Crystal system, space group	Triclinic, P-1
Unit cell dimensions	a = 7.2336(8) A alpha = 76.954(4) deg.<br>b = 8.6113(10) A beta = 89.536(4) deg.<br>c = 11.7738(13) A gamma = 85.457(5) deg.
Volume	712.19(14) A^3
Z, Calculated density	2, 1.270 Mg/m^3
Absorption coefficient	0.689 mm^-1
F(000)	292
Crystal size	0.220 x 0.200 x 0.180 mm
Theta range for data collection	3.854 to 65.070 deg.

Limiting indices	-8<=h<=8, -10<=k<=10, -13<=l<=13
Reflections collected / unique	6368 / 2350 [R(int) = 0.0668]
Completeness to theta = 65.070	97.0 %
Refinement method	Full-matrix least-squares on F^2
Data / restraints / parameters	2350 / 0 / 183
Goodness-of-fit on F^2	1.344
Final R indices [I>2sigma(I)]	R1 = 0.0678, wR2 = 0.2110
R indices (all data)	R1 = 0.2813, wR2 = 0.3207
Extinction coefficient	n/a
Largest diff. peak and hole	1.010 and -1.360 e.A^-3

Supplementary Table 8. Crystal data and structure refinement for CCDC 2074127

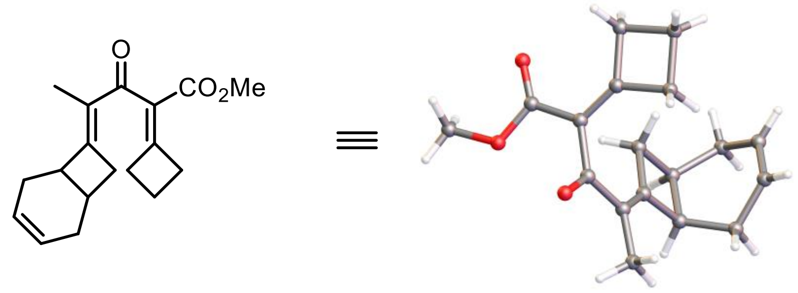

$4n_2$ **[X-ray for $4n_2$]**

Identification code	CCDC 2074127
Empirical formula	C18 H22 O3
Formula weight	286.35
Temperature	173(2) K
Wavelength	1.54178 A
Crystal system, space group	Triclinic, P-1
Unit cell dimensions	a = 9.3532(3) A alpha = 74.723(2) deg.<br>b = 9.4135(3) A beta = 76.922(2) deg.<br>c = 10.4767(3) A gamma = 61.770(2) deg.
Volume	778.37(4) A^3
Z, Calculated density	2, 1.222 Mg/m^3
Absorption coefficient	0.654 mm^-1
F(000)	308
Crystal size	0.160 x 0.150 x 0.140 mm

Theta range for data collection	4.406 to 68.257 deg.
Limiting indices	-11<=h<=11, -11<=k<=11, -12<=l<=12
Reflections collected / unique	8066 / 2836 [R(int) = 0.0340]
Completeness to theta = 67.679	99.5 %
Refinement method	Full-matrix least-squares on F^2
Data / restraints / parameters	2836 / 0 / 192
Goodness-of-fit on F^2	1.045
Final R indices [I>2sigma(I)]	R1 = 0.0427, wR2 = 0.1107
R indices (all data)	R1 = 0.0546, wR2 = 0.1186
Extinction coefficient	n/a
Largest diff. peak and hole	0.195 and -0.154 e.A^-3

Supplementary Table 9. Crystal data and structure refinement for CCDC 2074128

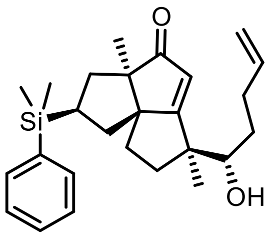

111

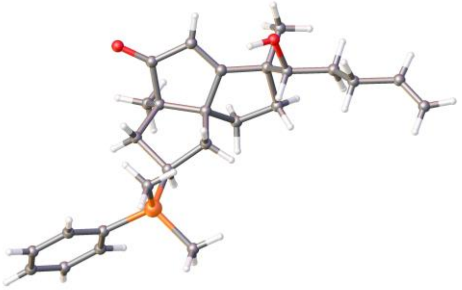

[X-ray for 111]

Identification code	CCDC 2074128
Empirical formula	C26 H36 O2 Si
Formula weight	408.64
Temperature	173(2) K
Wavelength	1.54178 A
Crystal system, space group	Monoclinic, P2(1)/c
Unit cell dimensions	a = 11.9765(3) A alpha = 90 deg.<br>b = 23.8719(6) A beta = 94.0300(10) deg.<br>c = 8.6720(2) A gamma = 90 deg.
Volume	2473.21(10) A^3
Z, Calculated density	4, 1.097 Mg/m^3
Absorption coefficient	0.961 mm^-1
F(000)	888
Crystal size	0.200 x 0.200 x 0.200 mm

Theta range for data collection	3.703 to 68.177 deg.
Limiting indices	-14<=h<=14, -28<=k<=28, -10<=l<=10
Reflections collected / unique	36061 / 4532 [R(int) = 0.0609]
Completeness to theta = 67.679	99.9 %
Refinement method	Full-matrix least-squares on F^2
Data / restraints / parameters	4532 / 1 / 268
Goodness-of-fit on F^2	1.078
Final R indices [I>2sigma(I)]	R1 = 0.0546, wR2 = 0.1534
R indices (all data)	R1 = 0.0667, wR2 = 0.1634
Extinction coefficient	n/a
Largest diff. peak and hole	1.240 and -0.605 e.A^-3

Supplementary Table 10. Crystal data and structure refinement for CCDC 2074827.

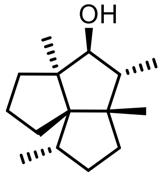

≡

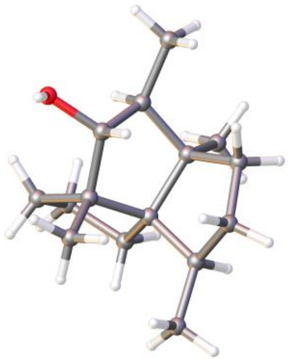

114-1 **[X-ray for 114-1]**

Identification code	CCDC 2074827
Empirical formula	C15 H26 O
Formula weight	222.36
Temperature	173(2) K
Wavelength	1.54178 A
Crystal system, space group	Tetragonal, I4(1)/a
Unit cell dimensions	a = 25.1624(3) A alpha = 90 deg.<br>b = 25.1624(3) A beta = 90 deg.<br>c = 8.3929(2) A gamma = 90 deg.
Volume	5313.94(18) A^3
Z, Calculated density	16, 1.112 Mg/m^3
Absorption coefficient	0.503 mm^-1
F(000)	1984
Crystal size	0.220 x 0.200 x 0.150 mm

Theta range for data collection	3.513 to 68.286 deg.
Limiting indices	-30<=h<=28, -30<=k<=30, -10<=l<=10
Reflections collected / unique	25443 / 2427 [R(int) = 0.0934]
Completeness to theta = 67.679	100.0 %
Absorption correction	Semi-empirical from equivalents
Refinement method	Full-matrix least-squares on F^2
Data / restraints / parameters	2427 / 0 / 153
Goodness-of-fit on F^2	1.030
Final R indices [I>2sigma(I)]	R1 = 0.0401, wR2 = 0.1025
R indices (all data)	R1 = 0.0504, wR2 = 0.1102
Extinction coefficient	n/a
Largest diff. peak and hole	0.220 and -0.160 e.A^-3

9. DFT calculations

The M06-2X density functional [13] [14] was employed for the computational study. The LANL2DZ basis set together with the LANL2DZ pseudopotential [15] [16] was used to describe In and Sb atoms and 6-31G(d) basis set [17] was utilized for other atoms. Vibrational frequency analyses were carried out to characterize the stationary points as local minima or transition states. To verify that each transition state connects to its appropriate reactant and product, the intrinsic reaction coordinate (IRC) calculations [18] were employed. The solvent effect of chloroform in the reaction was evaluated using the SMD solvation model developed by Truhlar and Cramer [19]. This model was used for single point energy calculations based on the gas phase optimized geometries at a larger basis set (SDD [20] [21] for In, Sb atoms and 6-311++G(d,p) [23] [24] for other atoms). For the purpose of discussion, the Gibbs free energy in solvation was used and it was obtained from the addition of solvation single point energy and gas-phase thermal correction to Gibbs free energy. All calculations were carried out by Gaussian 09 program package [24].

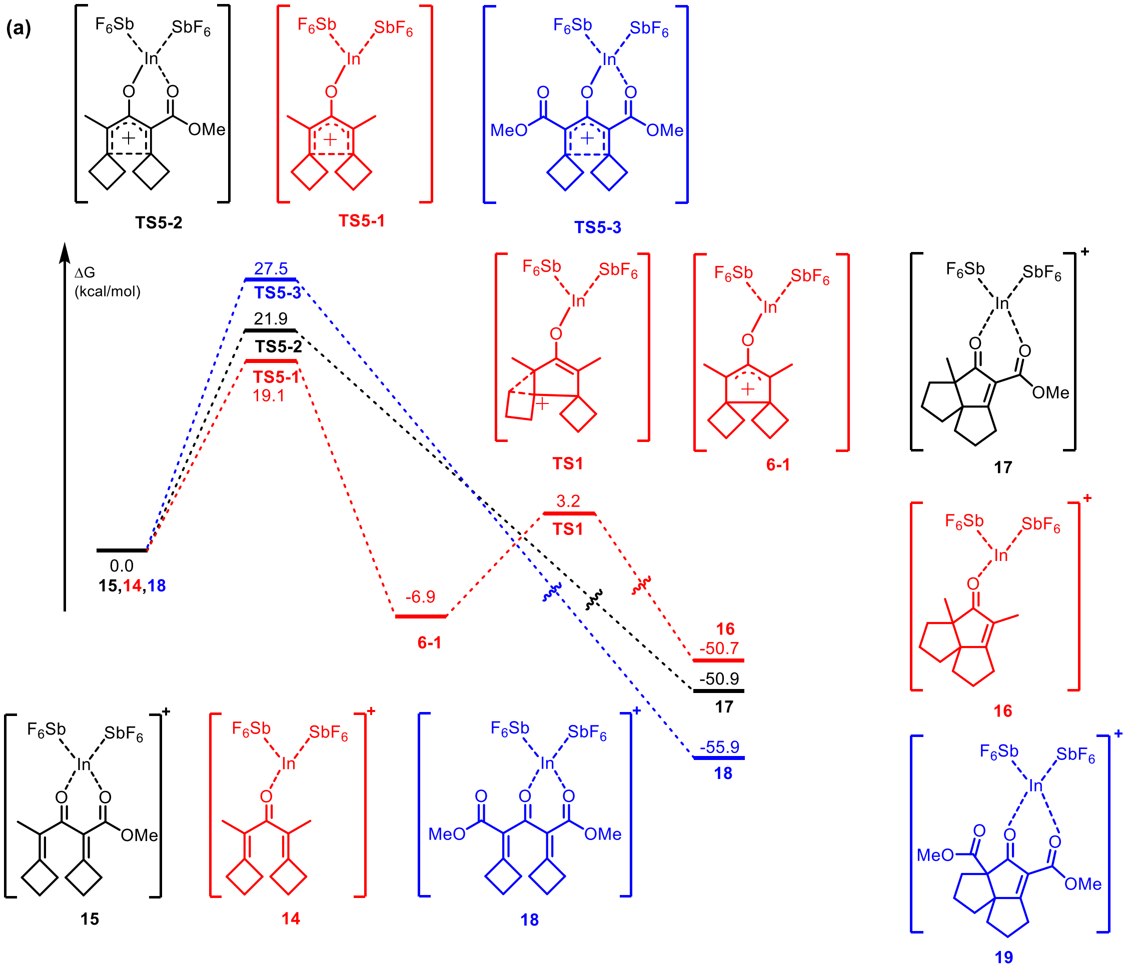

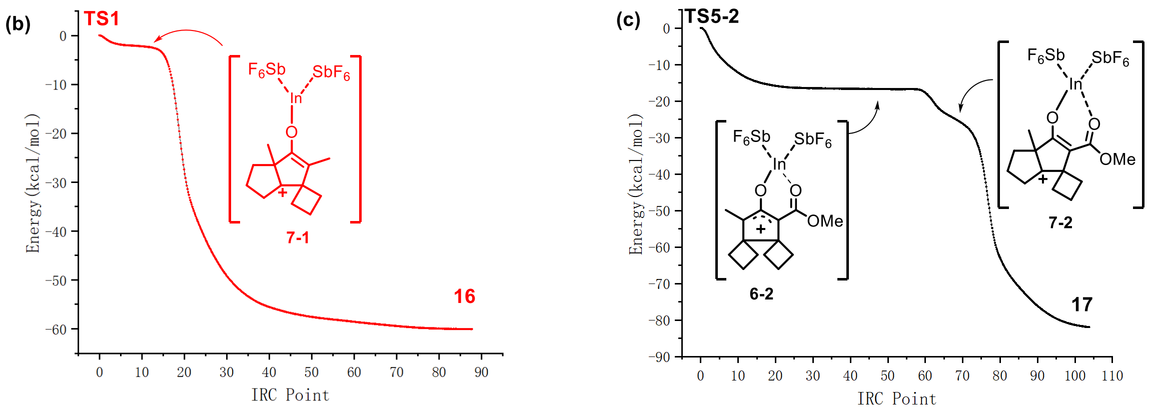

Supplementary Figure 7. (a). Computed Gibbs free energy changes of the reaction pathways in $CHCl_3$. (b). DFT IRC pathway of the formation of **16** for the twice cyclo-expansion process from **TS1**. (c). DFT IRC pathway of the formation of **17** for the completed Nazarov cyclization and twice ring expansion process from **TS5-2**.

We also carried DFT calculation to substrate **2hh**. Reaction needed the highest Gibbs free energy barrier of 27.5 kcal/mol in **TS5-3** to activate the key Nazarov cyclization, which was difficult to access even under stronger conditions. This result was compatible with our experimental fact.

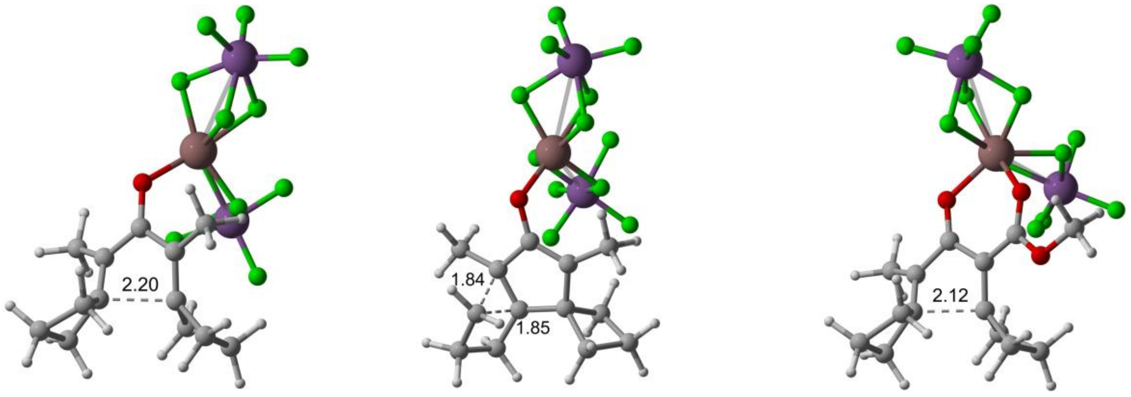

TS5-1 **TS1** **TS5-2**

The unit is Å

Supplementary Figure 8. Comment on the the key distances in the TSs given in **Supplementary Figure 7.**

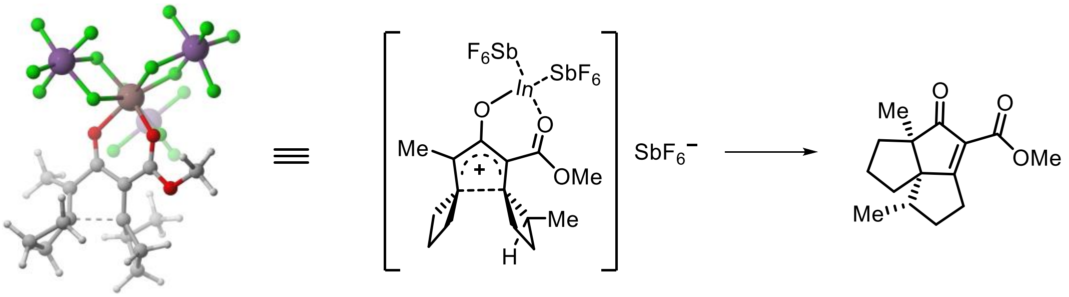

ΔΔG : 0.0 kcal/mol **TS_{3b}** **3b**

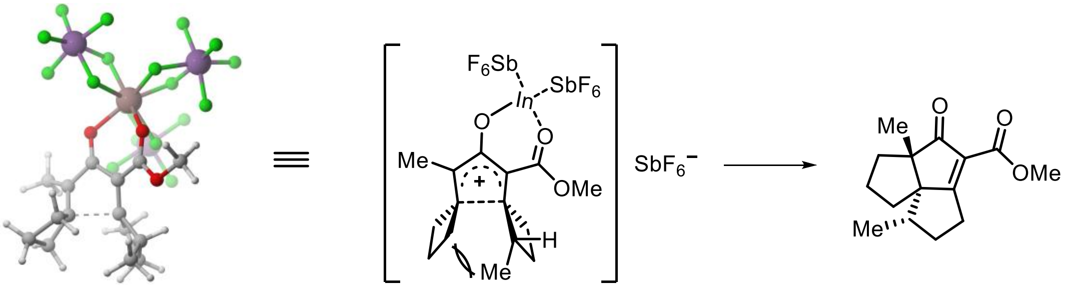

ΔΔG : 3.8 kcal/mol **TS_{3b}'** **3b'**

Supplementary Figure 9. Comment on the diastereoselectivity of **3b** and **3b'** by DFT calculation

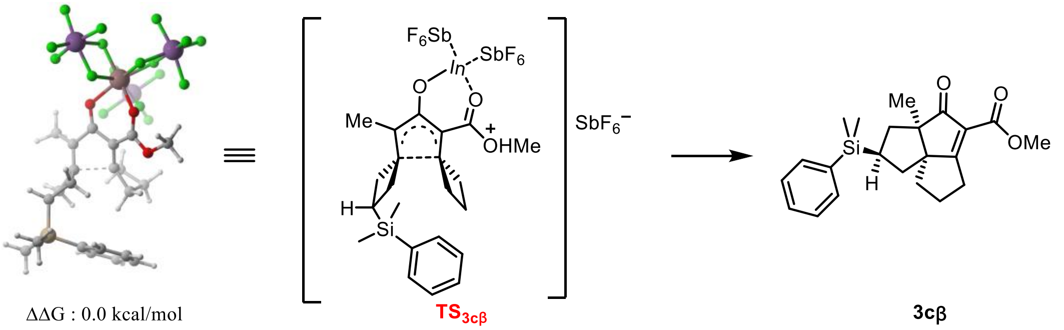

ΔΔG : 0.0 kcal/mol **$TS_{3c\beta}$** **3cβ**

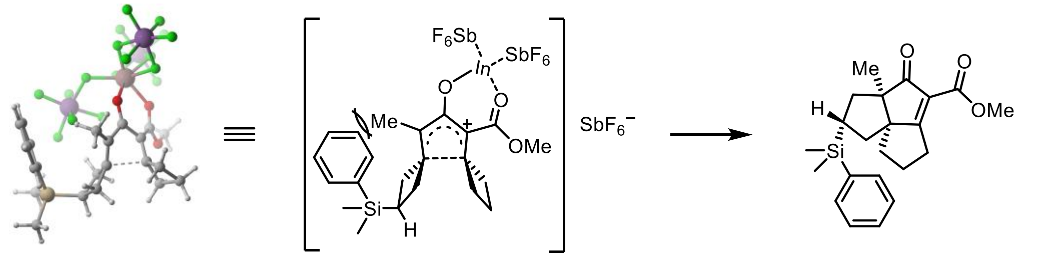

ΔΔG : 1.4 kcal/mol **$TS_{3c\alpha}$** **3cα**

Supplementary Figure 10. Comment on the diastereoselectivity of **3cβ** and **3cα** by DFT calculation

According to the formal DFT calculations, we know that the Nazarov electrocyclization process is the rate determining step during the overall mechanism. Thus, the diastereoselectivity of the electrocyclization reaction followed by cycle-expansion shall be controlled by the first transition state. In the below **Supplementary Figure 9.** shows us the steric hindrance between Me

and cyclobutyl group decides the diastereoselectivity. The $\mathbf{TS_{3b}}$ provides longer spacial distance and smaller hindrance effect, leading to more stable transition state (3.8 kcal/mol lower than $\mathbf{TS_{3b'}}$), which contributes to single product **3b**. Like the case indicated in **Supplementary Figure 10.**, the hindrance effect between $SiPhMe_2$ and catalyst, Me of substrate causes $\mathbf{TS_{3ca}}$ has higher free energy (1.4 kcal/mol) than $\mathbf{TS_{3c\beta}}$, leading to more favorable product **3cβ**. Given that smaller difference between $\mathbf{TS_{3c\beta}}$ and $\mathbf{TS_{3ca}}$ (1.4 kcal/mol), compared with $\mathbf{TS_{3b}}$ and $\mathbf{TS_{3b'}}$ (3.8 kcal/mol), we found that the second electrocyclization reaction gives the amount ratio of 4:1 for **3cβ** and **3ca**.

Coordinates and Energies:

15

Zero-point correction= 0.331513 (Hartree/Particle)
Thermal correction to Energy= 0.369510
Thermal correction to Enthalpy= 0.370454
Thermal correction to Gibbs Free Energy= 0.257984
Sum of electronic and zero-point Energies= -1979.773263
Sum of electronic and thermal Energies= -1979.735266
Sum of electronic and thermal Enthalpies= -1979.734322
Sum of electronic and thermal Free Energies= -1979.846793
SCF Done: E(RM062X) = -1980.78082592

C	-1.73418961	-0.65217390	0.00000000
C	-0.98329561	-1.76949790	-0.67957000
C	-0.80372261	-2.41829490	0.71812900
C	-1.20991861	-1.06717090	1.36207400
H	-0.03184561	-1.41264690	-1.09277500
H	-1.51353661	-2.32997290	-1.45314800
H	0.18842639	-2.79770690	0.96082600
H	-1.54727261	-3.19489490	0.90161200
H	-0.32908961	-0.48346890	1.65315300
H	-1.91375061	-1.07360590	2.19786700
C	-2.69996461	0.16589710	-0.48169900
C	-3.47207961	1.00008210	0.41637500
O	-4.63568561	1.32043610	0.05855300
C	-2.90856961	1.49486910	1.68737200

C	-1.66462161	2.03018610	1.72259000
C	-0.65829561	2.35764310	0.64780700
C	-0.96786361	2.91407510	2.72100200
C	-0.26115161	3.58419110	1.51332200
H	-1.03755961	2.51221610	-0.36436100
H	0.14742639	1.61551710	0.61816000
H	-1.59502161	3.51912710	3.37815100
H	-0.27013861	2.33019710	3.33281600
H	-0.78836961	4.48400310	1.19413800
H	0.80346239	3.79041510	1.61521900
C	-3.79719061	1.67995910	2.84660200
O	-5.02815361	1.86412010	2.77512000
O	-3.20500361	1.63338510	4.00177100
C	-4.01565061	1.87828310	5.17817800
H	-4.77379861	1.09969210	5.26181000
H	-3.31794261	1.84054110	6.01021800
H	-4.48801261	2.85759510	5.09722600
C	-3.15638261	0.12142410	-1.91806600
H	-3.28003761	1.13076010	-2.32034500
H	-2.43157261	-0.41062690	-2.53585300
H	-4.12495961	-0.38083290	-2.00267600
In	-6.11795261	2.32703410	1.06106900
Sb	-8.67773561	0.70696610	1.11124700
F	-9.16004461	-0.18587190	2.61132800
F	-6.82444061	0.22895210	1.32272300
F	-8.93548761	-0.58156690	-0.13442300
F	-7.70222961	1.83770910	-0.17419800
F	-10.17866761	1.68286810	0.84499200
F	-7.89210561	2.16825810	2.15445200
Sb	-5.19615061	5.30932110	0.60881800
F	-4.62974261	6.41427010	1.93329300
F	-6.72765961	6.18392610	0.18636700
F	-4.17364061	5.88119010	-0.77678800
F	-5.86303761	3.76525910	-0.40883600
F	-3.95308361	3.98219610	1.09137300
F	-6.24025561	4.21509810	1.87633600

TS5-2

Zero-point correction= 0.330790 (Hartree/Particle)
Thermal correction to Energy= 0.367792
Thermal correction to Enthalpy= 0.368736
Thermal correction to Gibbs Free Energy= 0.258539
Sum of electronic and zero-point Energies= -1979.737982
Sum of electronic and thermal Energies= -1979.700979
Sum of electronic and thermal Enthalpies= -1979.700035
Sum of electronic and thermal Free Energies= -1979.810232
SCF Done: E(RM062X) = -1980.74650032

C	-1.16106712	0.17786561	0.00000000
C	0.12051588	-0.26029539	-0.66514300
C	-0.22220812	-1.69553739	-0.17734800
C	-1.15950412	-1.08144239	0.89123600
H	0.99368088	0.12771961	-0.12621500
H	0.23891088	-0.06710739	-1.73302200
H	0.60854388	-2.29904239	0.18855900
H	-0.78246512	-2.25005439	-0.93201600
H	-0.63663612	-0.93592439	1.83945800
H	-2.13718012	-1.53234539	1.06925200
C	-2.23190512	0.80348261	-0.63068000
C	-3.28422312	1.25464061	0.25578400
O	-4.47759112	1.44037261	-0.15588100
C	-2.74755912	1.46344761	1.55239300
C	-1.32852512	1.61299561	1.54660000
C	-0.62707512	2.83462461	0.95185400
C	-0.38736112	1.59541861	2.71966100
C	-0.19834712	3.11331561	2.41370900
H	-1.27748512	3.54232961	0.43435700
H	0.23784488	2.57507461	0.33581100
H	-0.74770212	1.25743161	3.68823500
H	0.53569388	1.06853361	2.44999500
H	-0.94132412	3.71903561	2.93622000

H	0.80240788	3.51683861	2.56784200
C	-3.61989312	1.56143261	2.71473600
O	-4.84698612	1.76917061	2.62218500
O	-3.06171512	1.43300961	3.88552700
C	-3.91957412	1.56410161	5.04534600
H	-4.68560212	0.78892661	5.02022900
H	-3.25787612	1.43761661	5.89801200
H	-4.38244112	2.55129161	5.04463400
C	-2.26095012	1.14966661	-2.08375600
H	-3.22925912	1.57428361	-2.35304600
H	-1.48192512	1.89140161	-2.30477800
H	-2.06366012	0.27326261	-2.70849300
In	-5.94287312	2.32092661	0.92573500
Sb	-8.55937312	0.79032961	0.95248800
F	-8.99543912	-0.27756739	2.35012500
F	-6.71549812	0.23704461	0.99859700
F	-8.93097512	-0.32365539	-0.42688300
F	-7.61639712	2.04114661	-0.24594800
F	-10.04081712	1.83024061	0.89368200
F	-7.67216912	2.08418361	2.11463600
Sb	-5.02241412	5.34159161	0.70926000
F	-4.45861512	6.34498061	2.11468300
F	-6.54279212	6.26505361	0.35244300
F	-3.98526612	6.01509561	-0.62083800
F	-5.68782412	3.89769061	-0.43049800
F	-3.76743012	3.99019561	1.09377700
F	-6.06546812	4.15198961	1.88140200

17

Zero-point correction= 0.336948 (Hartree/Particle)
Thermal correction to Energy= 0.372734
Thermal correction to Enthalpy= 0.373678
Thermal correction to Gibbs Free Energy= 0.267758
Sum of electronic and zero-point Energies= -1979.863105
Sum of electronic and thermal Energies= -1979.827319

Sum of electronic and thermal Enthalpies= -1979.826375
Sum of electronic and thermal Free Energies= -1979.932295
SCF Done: E(RM062X) = -1980.87177593

C	-0.16302950	0.01976285	-0.00213417
C	-0.06986950	-1.48704615	-0.39510017
C	-0.22252850	-1.43783715	-1.91696417
C	-1.38937250	-0.46524115	-2.13288817
H	-0.90476850	-2.04687315	0.04473083
H	0.86235150	-1.94719615	-0.05816317
H	-0.39832750	-2.42248215	-2.35556517
H	0.69228850	-1.03400615	-2.36566917
H	-2.34882750	-0.98776115	-2.05640317
H	-1.36450950	0.02171785	-3.11033217
C	-1.23777950	0.56811685	-0.98535717
C	-2.45586450	0.65432585	-0.09735117
O	-3.59421950	0.87365185	-0.55023417
C	-2.07833650	0.53055785	1.30283383
C	-0.74820150	0.22829785	1.35500283
C	1.23208050	0.64856285	0.17013583
C	0.30055850	0.23866085	2.41046583
C	1.62515450	0.22193685	1.60401383
H	1.17600850	1.74044085	0.10319583
H	1.94355350	0.30129585	-0.58406017
H	0.18082550	1.14157885	3.02173483
H	0.18345550	-0.60676515	3.09901283
H	2.37509750	0.88357985	2.03972383
H	2.04532150	-0.78729015	1.59854483
C	-2.98364150	0.78894785	2.41567183
O	-4.17193050	1.13735785	2.27234783
O	-2.46889350	0.62190985	3.59599283
C	-3.32297050	0.87564785	4.73987583
H	-4.18678950	0.21209085	4.69936583
H	-2.70164450	0.66275385	5.60523483
H	-3.64367050	1.91744085	4.72698383
C	-0.97779450	2.00105685	-1.48552917

H	-1.82878450	2.33753085	-2.08487217
H	-0.84268350	2.70651485	-0.66011917
H	-0.08095250	2.00869685	-2.11098517
In	-5.15951750	1.70528785	0.52287783
Sb	-7.70830150	0.08727885	0.34707583
F	-8.20390250	-0.96396415	1.73603583
F	-5.84795550	-0.38858715	0.56259083
F	-7.90372650	-1.07136415	-1.03029917
F	-6.71144050	1.34884185	-0.79236317
F	-9.21856650	1.06220285	0.14150883
F	-6.96764650	1.44438485	1.54904083
Sb	-4.11651150	4.65531185	0.31997283
F	-3.57177950	5.62087385	1.75610883
F	-5.53124350	5.68310985	-0.15709217
F	-2.93273150	5.20581285	-0.94161117
F	-4.80636350	3.23736685	-0.84545717
F	-3.00850950	3.19920685	0.79401483
F	-5.32263050	3.55310885	1.42727683

14

Zero-point correction= 0.315314 (Hartree/Particle)
Thermal correction to Energy= 0.350540
Thermal correction to Enthalpy= 0.351484
Thermal correction to Gibbs Free Energy= 0.245993
Sum of electronic and zero-point Energies= -1791.259656
Sum of electronic and thermal Energies= -1791.224430
Sum of electronic and thermal Enthalpies= -1791.223486
Sum of electronic and thermal Free Energies= -1791.328977
SCF Done: E(RM062X) = -1792.18577643

C	0.19094138	-1.26998222	0.00000000
C	1.27884038	-1.88512522	-0.84142300
C	1.44359238	-2.94975122	0.27647500
C	0.65789738	-2.01403122	1.23202200
H	2.15193838	-1.22346022	-0.89946000

H	1.01354338	-2.21088022	-1.84987400
H	2.46215738	-3.20630222	0.56604100
H	0.88540438	-3.85920722	0.05051700
H	1.34042038	-1.40358522	1.83328800
H	-0.10513662	-2.43661122	1.88982400
C	-0.92167262	-0.59021922	-0.37364900
C	-1.95980762	-0.32408022	0.58673100
O	-3.16267062	-0.26741122	0.11487300
C	-1.75995262	-0.10350222	2.00168800
C	-0.68424362	0.60507878	2.41884100
C	0.42464538	1.38516378	1.75618600
C	-0.47072162	1.31504178	3.72902900
C	0.23980438	2.42002478	2.89779300
H	0.28682338	1.69183578	0.71854800
H	1.38550238	0.86855178	1.86076300
H	-1.35581562	1.57064278	4.31663600
H	0.24330138	0.77864478	4.36512600
H	-0.46101462	3.20313678	2.60466700
H	1.14057938	2.86062678	3.32409200
C	-2.88067362	-0.45386022	2.95746800
C	-1.20918662	-0.23017822	-1.81019100
H	-1.63133162	0.77708178	-1.87208400
H	-0.29500062	-0.25686922	-2.40510900
H	-1.93309762	-0.92148522	-2.25264600
In	-4.67597462	0.87172878	0.59191700
Sb	-7.53057962	0.06449178	0.95727200
F	-8.34484462	0.53345078	2.49939200
F	-5.81675462	-0.22134322	1.91605900
F	-7.81426262	-1.72031622	0.95238100
F	-6.13969762	-0.14609722	-0.43284600
F	-8.73783362	0.60928478	-0.26912500
F	-6.56315262	1.75670578	0.88515100
Sb	-3.25701462	3.74133278	0.48047600
F	-2.39072562	4.57693478	1.84133000
F	-4.90432462	4.43856778	0.79714700
F	-2.82577062	4.75636978	-0.95438200

F	-4.25686762	2.40564378	-0.62697900
F	-1.90594862	2.51133578	0.21914300
F	-3.87133562	2.29098178	1.74540400
H	-2.46764362	-0.80133622	3.90752300
H	-3.51544162	0.40932678	3.20185900
H	-3.51087062	-1.25696122	2.56456600

TS5-1

Zero-point correction= 0.314939 (Hartree/Particle)
Thermal correction to Energy= 0.348989
Thermal correction to Enthalpy= 0.349934
Thermal correction to Gibbs Free Energy= 0.248074
Sum of electronic and zero-point Energies= -1791.230789
Sum of electronic and thermal Energies= -1791.196739
Sum of electronic and thermal Enthalpies= -1791.195795
Sum of electronic and thermal Free Energies= -1791.297654
SCF Done: E(RM062X) = -1792.15735675

C	2.76642974	-1.36767316	0.00000000
C	4.18803874	-1.24958016	-0.48736600
C	4.19045974	-2.80468016	-0.53492400
C	3.02104574	-2.80126716	0.48081500
H	4.85063574	-0.89946916	0.31343000
H	4.37846274	-0.67436516	-1.39532100
H	5.11061074	-3.30181616	-0.22691100
H	3.87337674	-3.17415316	-1.51173500
H	3.40222574	-2.84937016	1.50423400
H	2.19214374	-3.50091916	0.35795100
C	1.64384574	-0.85003016	-0.66383300
C	0.45359874	-0.89012516	0.09307700
O	-0.74561326	-0.82946516	-0.45590400
C	0.69027474	-1.01673116	1.48143500
C	1.96005874	-0.59081016	1.89390600
C	2.56404674	0.81428284	1.77349600
C	2.54393274	-0.74906616	3.27622200

C	2.58238674	0.80622284	3.31978900
H	1.95379874	1.54473884	1.24510100
H	3.58155374	0.81173384	1.37422400
H	1.98095674	-1.30154016	4.02999600
H	3.56642874	-1.14203216	3.22299200
H	1.65786374	1.21884184	3.72860200
H	3.44455974	1.26028584	3.80863600
C	-0.34210326	-1.60221016	2.40092200
C	1.71100774	-0.19031716	-2.00736400
H	0.70314874	0.02022284	-2.36843100
H	2.24526774	0.76383784	-1.92661200
H	2.23261074	-0.81103216	-2.74110800
In	-2.21368226	0.22814584	0.22523800
Sb	-5.06414526	-0.52351216	0.75363400
F	-5.69707126	-0.43891016	2.44392600
F	-3.29009826	-1.11341316	1.39682800
F	-5.48071726	-2.23127616	0.33172600
F	-3.84773726	-0.43383816	-0.81003600
F	-6.34472326	0.37082784	-0.15254900
F	-3.99664126	1.08381484	0.99542600
Sb	-0.74739626	3.05911884	0.54239600
F	0.46729874	3.47520084	1.83070200
F	-2.22377326	3.67553084	1.40254200
F	-0.57240926	4.42791284	-0.62998900
F	-2.03015826	2.05355484	-0.60747300
F	0.43321374	1.94704184	-0.34193600
F	-1.15222026	1.34655484	1.52368100
H	0.12432374	-2.24820716	3.14930700
H	-0.87513026	-0.81541616	2.95237600
H	-1.07046626	-2.20316716	1.84948500

6-1

Zero-point correction= 0.317021 (Hartree/Particle)
Thermal correction to Energy= 0.350941
Thermal correction to Enthalpy= 0.351885

Thermal correction to Gibbs Free Energy= 0.250657
Sum of electronic and zero-point Energies= -1791.269674
Sum of electronic and thermal Energies= -1791.235754
Sum of electronic and thermal Enthalpies= -1791.234810
Sum of electronic and thermal Free Energies= -1791.336038
SCF Done: E(RM062X) = -1792.20145939

C	-0.80372999	-0.63943161	0.00000000
C	0.31051701	0.32613739	-0.47139800
C	0.85445801	-0.75888761	-1.42417400
C	0.07629001	-1.81116261	-0.61198200
H	1.01131201	0.50603339	0.34942300
H	-0.00983099	1.28828939	-0.87925300
H	1.93711201	-0.87790261	-1.46974500
H	0.47087501	-0.65487561	-2.44140800
H	0.68113501	-2.25737861	0.18111500
H	-0.45697999	-2.60241961	-1.14323000
C	-2.08441199	-0.58024061	-0.72799600
C	-3.13952899	-0.94680161	0.11298700
O	-4.41715099	-1.05600661	-0.27224200
C	-2.65182599	-1.13040261	1.40340000
C	-1.22867599	-0.74149761	1.47246800
C	-1.14236799	0.58469339	2.34604500
C	-0.34143099	-1.41182561	2.54744800
C	-0.65675499	-0.26709761	3.53366100
H	-2.06169899	1.16336139	2.45073500
H	-0.36417499	1.23677439	1.94298000
H	-0.59111299	-2.43697461	2.83354900
H	0.70643401	-1.38157761	2.23406200
H	-1.46389899	-0.51953061	4.22490800
H	0.18032501	0.13024439	4.10821400
C	-3.50209799	-1.52700061	2.54923600
C	-2.27385299	-0.21350861	-2.14613800
H	-3.32074099	0.02326639	-2.34547500
H	-1.63377299	0.62681539	-2.42834800
H	-1.99936399	-1.06757161	-2.78192400

In	-5.69777499	0.29500739	0.19727700
Sb	-8.64184399	0.00667939	0.61772300
F	-9.30856799	0.34383339	2.26259100
F	-7.01081199	-0.77612261	1.40260600
F	-9.30715199	-1.64501861	0.31126100
F	-7.36878499	-0.21412461	-0.88194600
F	-9.73337699	1.00906539	-0.41482000
F	-7.34342399	1.46147939	0.77019300
Sb	-3.96058499	2.99257439	0.62236100
F	-3.06453599	3.47895939	2.12580700
F	-5.60019699	3.59288939	1.13380100
F	-3.58665099	4.32842239	-0.54156100
F	-5.00066399	1.94750339	-0.73104800
F	-2.57853999	1.92365939	0.02710300
F	-4.55180899	1.30386939	1.53895200
H	-3.01410799	-2.29634661	3.15675600
H	-3.66418999	-0.65903561	3.20437400
H	-4.47108099	-1.90121461	2.20952900

TS1

Zero-point correction= 0.316861 (Hartree/Particle)
Thermal correction to Energy= 0.350322
Thermal correction to Enthalpy= 0.351266
Thermal correction to Gibbs Free Energy= 0.251031
Sum of electronic and zero-point Energies= -1791.251340
Sum of electronic and thermal Energies= -1791.217879
Sum of electronic and thermal Enthalpies= -1791.216935
Sum of electronic and thermal Free Energies= -1791.317169
SCF Done: E(RM062X) = -1792.18578263

C	-2.08273101	0.10783589	-0.10774452
C	-0.71425501	0.22602189	-0.72934052
C	-0.77614301	-1.16849411	-1.39316952
C	-2.14121001	-1.59952111	-0.82344752
H	0.06714899	0.31418489	0.02686648

H	-0.62940201	1.06643489	-1.42240352
H	0.02108099	-1.84320111	-1.07792152
H	-0.77931501	-1.12686111	-2.48283452
H	-2.12593401	-2.12613511	0.13352648
H	-2.82705401	-2.12602911	-1.49167252
C	-3.24281101	-0.13117811	-0.86416352
C	-4.34818401	-0.32953111	0.10764848
O	-5.59928701	-0.51532911	-0.34425352
C	-3.89051401	-0.23537511	1.37215548
C	-2.43450601	0.13362189	1.34440448
C	-2.13108001	1.53517689	2.01134848
C	-1.50646101	-0.49674511	2.41622848
C	-1.63246201	0.78741689	3.26443848
H	-2.98832701	2.20413089	2.09463848
H	-1.32242301	2.05604189	1.49076348
H	-1.85232601	-1.43789411	2.85134848
H	-0.49001801	-0.63503211	2.03514648
H	-2.40994601	0.70121389	4.02645948
H	-0.72167101	1.16400989	3.73054748
C	-4.69924901	-0.36939511	2.61979948
C	-3.52887601	0.22139689	-2.29116252
H	-3.99867201	1.20960989	-2.28767052
H	-2.62794301	0.27135689	-2.90542252
H	-4.23543701	-0.49030511	-2.72434752
In	-6.91697801	0.81712689	0.07029648
Sb	-9.86536501	0.51148389	0.47837248
F	-10.56064901	0.89744289	2.10123948
F	-8.23070001	-0.21023011	1.31162248
F	-10.50205901	-1.16271811	0.23307248
F	-8.57757401	0.24268589	-0.99890152
F	-10.97022601	1.44583989	-0.60373052
F	-8.60141601	1.99184889	0.57854448
Sb	-5.31247201	3.62114589	0.22510848
F	-4.41321401	4.28494289	1.65902748
F	-6.95053601	4.28084689	0.65794148
F	-4.93494101	4.81880089	-1.08188152

F	-6.32212401	2.42724189	-1.01419652
F	-3.91594401	2.50683189	-0.24750252
F	-5.88801301	2.04584589	1.31330848
H	-4.23757701	-1.06937711	3.32449048
H	-4.80902701	0.59311089	3.13427048
H	-5.69909901	-0.75704311	2.39589048

16

Zero-point correction= 0.320400 (Hartree/Particle)
Thermal correction to Energy= 0.353872
Thermal correction to Enthalpy= 0.354816
Thermal correction to Gibbs Free Energy= 0.252498
Sum of electronic and zero-point Energies= -1791.343435
Sum of electronic and thermal Energies= -1791.309963
Sum of electronic and thermal Enthalpies= -1791.309019
Sum of electronic and thermal Free Energies= -1791.411336
SCF Done: E(RM062X) = -1792.27306819

C	1.00806450	-0.13440860	0.00000000
C	1.18655750	-1.67460360	0.13535900
C	0.83661350	-2.17775460	-1.26651500
C	-0.43511050	-1.39180060	-1.61220000
H	0.47004150	-2.07764360	0.86144900
H	2.19217650	-1.94883860	0.46468500
H	0.69757750	-3.26019760	-1.31153700
H	1.64024550	-1.91641560	-1.96533000
H	-1.31062650	-1.87094060	-1.15953400
H	-0.62151850	-1.32188260	-2.68642400
C	-0.18775550	0.00574640	-0.98349000
C	-1.28762950	0.43959240	-0.04993100
O	-2.48639550	0.50669040	-0.47131600
C	-0.81861950	0.78995040	1.25276100
C	0.52032650	0.52208240	1.24932600
C	2.35761050	0.57702540	-0.20454800
C	1.65548350	0.90550340	2.13814400

C	2.90425250	0.69021340	1.23891500
H	2.21085750	1.57535240	-0.63018400
H	3.02962950	0.02440140	-0.86751500
H	1.53741350	1.94636240	2.46143300
H	1.67433850	0.29728940	3.05071000
H	3.62772550	1.50019740	1.34299200
H	3.41137050	-0.23529560	1.52426000
C	-1.64930950	1.40497940	2.33547100
C	-0.01838250	1.10963540	-2.04055400
H	0.17891450	2.08465240	-1.58599000
H	0.81223950	0.85331440	-2.70409400
H	-0.93180850	1.18634740	-2.63734500
In	-4.16373350	1.43857240	-0.06835000
Sb	-6.71236650	-0.09864560	0.20468000
F	-7.67989850	0.12591740	1.71310400
F	-5.02031350	0.08880440	1.22821100
F	-6.50684850	-1.89464460	0.20200200
F	-5.27170850	0.07137940	-1.13602200
F	-7.97932050	0.10358540	-1.06501900
F	-6.23390750	1.79004340	0.14185600
Sb	-3.22608450	4.46202340	-0.11162200
F	-2.52418450	5.33903840	1.30963700
F	-4.91689850	5.11067940	-0.01664000
F	-2.65490450	5.43172540	-1.52772300
F	-4.02202050	3.02509940	-1.27447700
F	-1.82466450	3.24964140	-0.18211300
F	-3.90260450	3.01255540	1.11945600
H	-1.18277550	1.26317440	3.31227200
H	-1.78227050	2.47847440	2.17377500
H	-2.64065250	0.93594640	2.39447900

18

Zero-point correction= 0.347592 (Hartree/Particle)
Thermal correction to Energy= 0.388205
Thermal correction to Enthalpy= 0.389149

Thermal correction to Gibbs Free Energy= 0.271484
Sum of electronic and zero-point Energies= -2168.244205
Sum of electronic and thermal Energies= -2168.203592
Sum of electronic and thermal Enthalpies= -2168.202647
Sum of electronic and thermal Free Energies= -2168.320313
SCF Done: E(RM062X) = -2169.32812639

C	-1.03463584	-0.17761989	0.00000000
C	-0.06819384	-1.10155589	-0.69954000
C	-0.35484684	-2.14975289	0.40781200
C	-1.03805184	-1.03720989	1.24793500
H	0.95393716	-0.70596289	-0.66168200
H	-0.30326584	-1.35178489	-1.73428300
H	0.50751616	-2.63829689	0.86041100
H	-1.06831784	-2.90535289	0.07667400
H	-0.36337684	-0.63288689	2.01252800
H	-2.00464284	-1.25083089	1.71183300
C	-1.76051784	0.88237611	-0.39347900
C	-2.82784484	1.43480111	0.46040600
O	-3.95509384	1.53926511	-0.05903800
C	-2.55913384	1.81960811	1.85039500
C	-1.31871384	2.23160911	2.22882000
C	-0.03955384	2.51614111	1.47866100
C	-0.79355184	2.88414411	3.47835600
C	0.34506816	3.49214011	2.62108800
H	-0.15503884	2.91085911	0.46596900
H	0.61163016	1.63527911	1.44373300
H	-1.47439984	3.54987011	4.01328100
H	-0.43370084	2.12376011	4.18235000
H	0.14689516	4.53052811	2.35427400
H	1.35506416	3.40089911	3.01814200
C	-3.68416084	1.96623111	2.79686400
O	-4.86827784	2.17124311	2.47449400
O	-3.35506184	1.86436411	4.05041400
C	-4.40406484	2.05093711	5.03340300
H	-5.16379484	1.28103711	4.89980600

H	-3.90680984	1.95192811	5.99436700
H	-4.84462884	3.04068811	4.91227200
In	-5.65422884	2.51441611	0.57170700
Sb	-8.05109084	0.67579911	0.28500300
F	-8.75343484	-0.14623889	1.73886700
F	-6.25110984	0.36876411	0.88152500
F	-7.97953584	-0.71494889	-0.87301600
F	-6.92667584	1.79554611	-0.88367800
F	-9.53445884	1.50251911	-0.34190900
F	-7.58608184	2.26852811	1.33231400
Sb	-4.75634584	5.46195211	0.04734400
F	-4.51723484	6.66708811	1.38399000
F	-6.17823384	6.25619711	-0.75136100
F	-3.46469584	5.98840311	-1.10886300
F	-5.17576284	3.83028811	-0.95583100
F	-3.65509784	4.19676711	0.90631000
F	-6.04508884	4.43119211	1.15373500
C	-1.63890484	1.58252711	-1.70951700
O	-2.11510384	2.67754611	-1.89506800
O	-0.91751884	0.90419111	-2.59595600
C	-0.73152684	1.56668311	-3.85842100
H	-0.24360484	2.53066711	-3.70642000
H	-0.10610884	0.90038411	-4.44799600
H	-1.69818084	1.72191811	-4.33981900

TS5-3

Zero-point correction= 0.346780 (Hartree/Particle)
Thermal correction to Energy= 0.386454
Thermal correction to Enthalpy= 0.387398
Thermal correction to Gibbs Free Energy= 0.271231
Sum of electronic and zero-point Energies= -2168.199061
Sum of electronic and thermal Energies= -2168.159387
Sum of electronic and thermal Enthalpies= -2168.158443
Sum of electronic and thermal Free Energies= -2168.274610
SCF Done: E(RM062X) = -2169.28399442

C	-0.00444050	-0.68383658	0.00000000
C	1.42218150	-0.96261658	-0.41003200
C	1.15925550	-2.42809958	0.03296600
C	0.01114350	-1.92465258	0.93773700
H	2.11737850	-0.48217158	0.28858200
H	1.71555950	-0.75607558	-1.43832900
H	1.98032250	-2.94563758	0.52924500
H	0.77729450	-3.02123858	-0.79983700
H	0.36807950	-1.68484658	1.94165100
H	-0.92633950	-2.48027358	0.98679100
C	-1.05606050	-0.33120558	-0.85114900
C	-2.26413050	0.08647142	-0.18348700
O	-3.40661550	0.06899942	-0.74443200
C	-1.91933550	0.49766942	1.12866700
C	-0.51275250	0.73223342	1.27935400
C	0.14208150	1.99974342	0.69331100
C	0.23479950	0.91746442	2.57512200
C	0.29810050	2.42266242	2.17168200
H	-0.49276650	2.57872742	0.02055900
H	1.11506150	1.79860942	0.23846400
H	-0.22851950	0.60771242	3.50878200
H	1.24140850	0.49234942	2.48896100
H	-0.58157950	2.96586542	2.52340600
H	1.21023850	2.95592242	2.44016200
C	-2.95701950	0.70288242	2.13769800
O	-4.16342850	0.80640942	1.84723100
O	-2.56201650	0.78803242	3.37595300
C	-3.58086450	1.02192442	4.38000200
H	-4.28857750	0.19280942	4.37779900
H	-3.03898550	1.07655942	5.32020900
H	-4.09552850	1.95797942	4.16196000
In	-5.03762050	1.04203542	-0.05959600
Sb	-7.59803050	-0.57884458	-0.12305300
F	-8.16118850	-1.50306758	1.33016400
F	-5.75517650	-1.04817358	0.17796500

F	-7.77624050	-1.85049658	-1.40037100
F	-6.56252450	0.57333042	-1.35050400
F	-9.09233750	0.38405442	-0.46712800
F	-6.88456350	0.86348042	0.97374200
Sb	-4.18942650	4.04467942	-0.58099500
F	-3.94272950	5.26244642	0.74462200
F	-5.66606550	4.79662242	-1.32083500
F	-2.96181450	4.62116242	-1.78624300
F	-4.58435650	2.41242642	-1.57678600
F	-2.96857950	2.85035042	0.21701900
F	-5.37558550	2.96863442	0.57354500
C	-0.94396450	-0.09618258	-2.33160200
O	-1.45693950	0.84956542	-2.86817700
O	-0.22799250	-1.04298958	-2.92963600
C	-0.10245350	-0.89642258	-4.35757500
H	0.39067650	0.04854342	-4.59099500
H	0.49486650	-1.74385158	-4.68481300
H	-1.09246250	-0.91399558	-4.81601300

19

Zero-point correction= 0.352136 (Hartree/Particle)
Thermal correction to Energy= 0.391194
Thermal correction to Enthalpy= 0.392138
Thermal correction to Gibbs Free Energy= 0.278068
Sum of electronic and zero-point Energies= -2168.338695
Sum of electronic and thermal Energies= -2168.299637
Sum of electronic and thermal Enthalpies= -2168.298693
Sum of electronic and thermal Free Energies= -2168.412763
SCF Done: E(RM062X) = -2169.42384314

C	-1.42539959	-0.91474244	0.00000000
C	-1.34927359	-2.45590244	-0.22270300
C	-1.48578959	-2.57895944	-1.74255200
C	-2.64185459	-1.62903044	-2.07337600
H	-2.19296659	-2.95618944	0.26831500

H	-0.42379359	-2.87918044	0.17506300
H	-1.66908459	-3.60494944	-2.06892100
H	-0.57100759	-2.22477944	-2.23073500
H	-3.60608559	-2.10866644	-1.87403400
H	-2.63658459	-1.28721644	-3.10884000
C	-2.42719459	-0.44485544	-1.10074100
C	-3.65810659	-0.08479144	-0.29923100
O	-4.72856359	0.23572756	-0.84067800
C	-3.37592959	-0.15398644	1.12717800
C	-2.07710359	-0.54025844	1.28876000
C	-0.02572159	-0.29784244	0.18679800
C	-1.08884159	-0.50216144	2.40092500
C	0.27876741	-0.60540444	1.67178700
H	-0.06209859	0.78597956	0.02924100
H	0.71081241	-0.71905144	-0.50197400
H	-1.21279759	0.43293356	2.95871800
H	-1.26966759	-1.30973844	3.12083800
H	1.01269241	0.08463356	2.09011900
H	0.68481741	-1.61441544	1.77804800
C	-4.33348659	0.20193456	2.16922300
O	-5.51693859	0.51760056	1.94162400
O	-3.87494759	0.14005856	3.38214900
C	-4.78360259	0.48919256	4.45744800
H	-5.61441459	-0.21654144	4.46931600
H	-4.18761759	0.41383356	5.36265400
H	-5.15011659	1.50450956	4.30555000
In	-6.38625159	1.04217756	0.12049100
Sb	-8.82309659	-0.72318244	-0.23590300
F	-9.32259259	-1.86426144	1.07928400
F	-6.95008359	-1.09806844	0.05488400
F	-8.88088359	-1.83181044	-1.66660300
F	-7.84894959	0.64008956	-1.27621700
F	-10.37721059	0.16937356	-0.48513700
F	-8.23042959	0.62108956	1.05100100
Sb	-5.51701559	4.02698556	0.02330000
F	-5.16207459	4.99617156	1.51589000

F	-6.91062659	5.00624156	-0.59529000
F	-4.22913059	4.65802456	-1.09507100
F	-6.01158759	2.59486156	-1.21773700
F	-4.41896859	2.60335756	0.60255200
F	-6.78764459	2.86623756	0.99045600
C	-1.87645359	0.79270256	-1.81289000
O	-1.25173259	0.74413456	-2.83506200
O	-2.12178759	1.89794856	-1.10502400
C	-1.58131759	3.11305456	-1.65926100
H	-2.04509659	3.31366956	-2.62587900
H	-1.82833559	3.89441956	-0.94559700
H	-0.50177759	3.01309556	-1.78356200

TS_{3b}

Value of imaginary frequency = -270.51 cm^{-1}

Sb	-0.84240000	-2.45313800	-1.88263200
F	0.33538400	-0.92027800	-2.02433900
F	-2.29144600	-1.29561200	-2.03730900
F	-0.81334000	-2.71415000	-3.69048900
F	-1.98980500	-3.80608000	-1.40894400
F	0.63707300	-3.47952700	-1.56574000
F	-0.74549100	-1.81771200	-0.06655200
C	-3.85137600	-0.11825600	1.51414500
C	-4.57072900	-0.37402100	2.82548200
C	-5.70598600	-1.08259600	2.05322100
C	-4.81639500	-1.08750200	0.78565100
H	-4.83282800	0.51034500	3.41436800
H	-3.98484000	-1.03270100	3.47202100
H	-6.60260200	-0.46679100	1.95440800
H	-5.99193900	-2.06974400	2.42194800
C	-2.47567800	0.12444300	1.25381600
C	-2.23617100	0.73624300	-0.00937900
O	-1.17953500	0.79780700	-0.69190800
C	-3.46918400	1.34119500	-0.48141700
C	-4.36486800	1.67349000	0.53387600
C	-4.09710500	2.69179100	1.66463600
C	-5.75741900	2.23361500	0.33627200
C	-5.24467800	3.51968400	1.03747400
H	-3.09069600	3.11245300	1.68949300
H	-4.37166600	2.32145900	2.65433700
H	-6.15183800	2.29861100	-0.67944000
H	-6.48012100	1.71101700	0.97284000

H	-4.86446500	4.24153600	0.31206900
H	-5.92972300	4.01093500	1.72981800
C	-3.65964500	1.48729600	-1.94625800
In	0.63540700	0.03399600	-0.12896400
Sb	3.32561100	-1.25963300	1.22827400
F	4.22265300	-0.66388500	-0.24940500
F	1.83700300	-1.61560200	-0.01750700
F	3.95304600	-2.96567500	1.08765300
F	2.10117700	-1.72521100	2.52693600
F	4.51314300	-0.58749300	2.43745700
F	2.31596500	0.39428300	1.11354700
Sb	1.53974800	3.13971900	-0.83972500
F	2.43888800	3.66234800	-2.34050900
F	3.08438000	2.84800500	0.09672700
F	1.21707000	4.77206600	-0.08175400
F	0.61751100	2.19730900	0.55750000
F	-0.06861000	3.12980500	-1.73580100
F	1.67056800	1.23442900	-1.38630200
H	-3.65933400	0.50176700	-2.42685100
H	-4.55701500	2.04597300	-2.21088200
H	-2.76892800	2.00416400	-2.33016700
C	-1.37015600	-0.13639100	2.16878500
O	-0.17981200	-0.09081800	1.83009000
O	-1.69092900	-0.41825500	3.40971100
C	-0.58043100	-0.68335600	4.29896900
H	-0.03053500	0.24317700	4.47058400
H	-1.03648700	-1.04453600	5.21752000
H	0.08609900	-1.42262800	3.85428500
H	-5.28103200	-0.72539800	-0.13484900
C	-4.10994100	-2.41689900	0.53245100
H	-4.83683700	-3.16400500	0.19998800
H	-3.34558600	-2.31256500	-0.23606100
H	-3.63116100	-2.78872100	1.44607100

TS$_{3b}$'

Value of imaginary frequency = -298.29 cm^{-1}

Sb	-1.13989600	-1.76920200	-2.19372000
F	0.19301700	-0.38312800	-2.15977300
F	-2.41530300	-0.41655700	-2.12066400
F	-1.24272200	-1.80365600	-4.01687900
F	-2.46620100	-2.97925100	-1.78592400
F	0.17061200	-3.03785700	-2.11464200
F	-0.89083900	-1.43840800	-0.30243900
C	-3.86534200	-0.13152300	1.00670200

C	-4.68036700	-0.92036400	2.04099600
C	-5.60982000	-1.40272800	0.90558800
C	-4.57892300	-0.90640900	-0.12644800
H	-4.01785000	-1.73694200	2.35777300
H	-6.53970700	-0.82609300	0.87633800
H	-5.84967600	-2.46652300	0.88658000
C	-2.50431800	0.25861400	1.05415400
C	-2.12358300	1.15173900	0.01042800
O	-0.97336300	1.34868700	-0.48001800
C	-3.31561500	1.80674500	-0.47545600
C	-4.37299900	1.79710800	0.44495900
C	-4.36406000	2.59474100	1.77070500
C	-5.77642300	2.27369100	0.13475800
C	-5.51836700	3.42001600	1.14952800
H	-3.42518500	3.10288400	1.99543200
H	-4.68805400	2.02076900	2.63563300
H	-6.01567800	2.52263600	-0.90147000
H	-6.52333800	1.57252600	0.52192500
H	-5.14433900	4.31656300	0.65070900
H	-6.34450300	3.68262100	1.81183900
C	-3.39750600	2.28573300	-1.88027100
In	0.67018400	0.16774100	-0.07633600
Sb	2.82761900	-2.00759400	1.07939000
F	4.20294700	-1.33174600	0.08693700
F	1.66802100	-1.60409400	-0.45387900
F	3.09909700	-3.72860500	0.54211900
F	1.22243600	-2.43389500	1.89880100
F	3.74422100	-2.02849300	2.65687800
F	2.23208100	-0.17986100	1.33116100
Sb	2.48315800	2.89033000	-0.35110900
F	3.64432700	3.30775100	-1.69608500
F	3.76722000	2.03511900	0.63147800
F	2.55430500	4.43572300	0.61968300
F	1.18827400	2.08547800	0.83720700
F	1.02618200	3.42604300	-1.33319900
F	2.10934300	1.10925700	-1.14386800
H	-3.89749100	1.52358900	-2.49339100
H	-3.95692300	3.22150900	-1.96424900
H	-2.38729300	2.40834800	-2.27752100
C	-1.51447500	-0.17458500	2.03477400
O	-0.29720900	-0.06751900	1.84633600
O	-1.98325800	-0.67220700	3.15526900
C	-0.98528700	-1.14094200	4.09330700
H	-0.43916300	-0.28552900	4.49366900
H	-1.54798200	-1.64673900	4.87415500
H	-0.29428200	-1.81427300	3.58484400

H	-4.94180100	-0.35190800	-0.99341800
H	-3.90699600	-1.69350600	-0.48215100
C	-5.30226000	-0.29332300	3.27774700
H	-4.54507600	0.16098300	3.92385200
H	-6.04890700	0.46196200	3.01164200
H	-5.81613500	-1.06958300	3.85194500

TS$_{3c\beta}$

Value of imaginary frequency = -219.56 cm^{-1}

Sb	0.72511600	-2.22891100	-2.31491500
F	1.77644400	-0.62833900	-2.07399100
F	-0.69701400	-1.11287900	-2.75569100
F	1.18527800	-2.38946300	-4.07538700
F	-0.46802700	-3.62353000	-2.18882000
F	2.13191800	-3.23301400	-1.72509400
F	0.35876700	-1.74372200	-0.48863200
C	-3.01447300	-0.86729900	0.08149800
C	-3.93609000	-1.58107900	1.03290600
C	-3.58667600	-2.86085800	0.21952700
C	-3.21016300	-1.95638200	-0.97895200
H	-4.97540700	-1.30342500	0.81605900
H	-3.74763500	-1.52372900	2.10296500
H	-4.40090800	-3.57138800	0.06980100
H	-2.70582200	-3.36586000	0.62117500
C	-1.75677100	-0.26269900	0.35409700
C	-1.29852800	0.57318400	-0.70545800
O	-0.11891800	0.94290600	-0.97131400
C	-2.43455700	0.95565400	-1.52520000
C	-3.66636200	0.87679300	-0.88533000
C	-4.16177000	1.62114400	0.37390600
C	-5.02041300	1.00329600	-1.53732900
C	-5.36117700	2.11164200	-0.49067700
H	-3.46282500	2.36415300	0.76338100
H	-4.47975400	0.95718400	1.18344700
H	-5.04784300	1.25265200	-2.60119700
H	-5.61394100	0.09329100	-1.36759100
H	-5.14462100	3.09970200	-0.91137500
C	-2.24814600	1.24920300	-2.97480400
In	1.57023100	0.18325300	-0.06695600
Sb	3.83795600	-1.26380500	1.83036500
F	5.11599600	-0.41231600	0.83937800
F	2.82576400	-1.42846100	0.14973200
F	4.58822500	-2.91281200	1.61947500
F	2.30968000	-1.97482900	2.58948300
F	4.54431900	-0.81085200	3.45047700
F	2.80059100	0.36148900	1.68472000

Sb	2.93025300	3.15810700	-0.34771900
F	4.32731300	3.63386500	-1.42155000
F	4.01615300	2.75087600	1.06620700
F	2.46295600	4.80093000	0.30055100
F	1.50873900	2.26127800	0.61085400
F	1.73012700	3.23035900	-1.73796300
F	3.09663000	1.25394900	-0.86753300
H	-2.45760900	0.34720800	-3.56341400
H	-2.90646800	2.05292500	-3.31437600
H	-1.20379100	1.51447200	-3.15352400
C	-0.93997500	-0.44739900	1.54452500
O	0.25845400	-0.13063200	1.59134000
O	-1.52295200	-0.97720600	2.59342900
C	-0.67124500	-1.20084300	3.74061100
H	-0.36314300	-0.23968700	4.15470400
H	-1.29090400	-1.75052000	4.44527100
H	0.21044400	-1.76867600	3.44300400
H	-4.07703600	-1.77548700	-1.62096200
H	-2.32884000	-2.19409800	-1.57379300
Si	-7.08913800	2.04261200	0.29351400
C	-8.37347500	2.28663400	-1.05593700
H	-8.24872500	1.56531500	-1.87081200
H	-9.38756200	2.17264200	-0.66057300
H	-8.29088100	3.28908700	-1.48840700
C	-7.21509400	3.35226000	1.63383400
H	-6.39962900	3.26890700	2.36037900
H	-7.16237300	4.35464800	1.19664900
H	-8.16056600	3.27252000	2.17965900
C	-7.24378500	0.32248900	1.05151500
C	-7.63173400	-0.78794000	0.28367100
C	-6.89742200	0.09598500	2.39264100
C	-7.65218400	-2.07279500	0.82405700
H	-7.93380100	-0.64998000	-0.75305800
C	-6.92131000	-1.18390200	2.94294900
H	-6.60856300	0.93385200	3.02406100
C	-7.29198900	-2.27210700	2.15558100
H	-7.95701400	-2.91577000	0.21118200
H	-6.65390700	-1.33303200	3.98476700
H	-7.31116900	-3.27101800	2.58058900

TS$_{3ca}$

Value of imaginary frequency = -212.74 cm^{-1}

Sb	-1.18450200	-1.58941200	-1.55134200
F	0.36487000	-0.42533600	-1.74948800
F	-2.15569400	-0.08110600	-1.09296800

F	-1.64633100	-1.35176800	-3.31031400
F	-2.63095300	-2.64318400	-1.08950600
F	-0.10742400	-3.02377100	-1.90458400
F	-0.56858400	-1.55878000	0.23923000
C	-1.96059900	0.76664600	3.36585600
C	-2.22900600	2.14389600	3.97180500
C	-1.99964800	1.46361100	5.34299300
C	-2.31961300	0.09871400	4.67042300
H	-3.27039300	2.45849500	3.86472400
H	-1.56219600	2.94371800	3.64241800
H	-2.64831200	1.77036200	6.16430100
H	-0.95386600	1.51706500	5.65091500
C	-0.94303200	0.44368900	2.44205400
C	-1.10352000	1.06418600	1.16475000
O	-0.25277000	1.21465100	0.24606000
C	-2.47400700	1.52680200	1.00975300
C	-3.41506300	0.78550300	1.70344000
C	-3.64417700	-0.74179300	1.68272700
C	-4.88081300	1.06333900	1.92044300
C	-5.17451100	-0.44601000	1.65580100
H	-3.32009600	-1.29781700	2.56614900
H	-3.19618200	-1.19826600	0.79460800
H	-5.07839500	1.35599000	2.96175300
H	-5.33752300	1.80835000	1.26067200
H	-5.70310800	-0.92179700	2.48945200
C	-2.76723900	2.68144000	0.11385900
In	1.45037900	0.11398200	-0.01693200
Sb	3.87752100	-2.09065000	0.03433500
F	4.14966600	-1.42469400	-1.64958100
F	1.96949100	-1.85498700	-0.38178100
F	3.86414200	-3.83089500	-0.50865000
F	3.29013800	-2.52305200	1.72493800
F	5.61606300	-1.93594400	0.55777600
F	3.45071800	-0.24953600	0.50797300
Sb	2.86785700	3.02268300	-0.53972100
F	3.34460000	3.72095800	-2.15690000
F	4.47938900	2.19122500	-0.30523400
F	3.26872800	4.43345200	0.54858700
F	2.21908800	1.93921200	0.92424800
F	1.11387500	3.53115200	-0.74549600
F	2.28416600	1.32406600	-1.39664800
H	-2.95382000	2.27168500	-0.89042000
H	-3.66475200	3.22007100	0.42665600
H	-1.91303800	3.35904600	0.04349600
C	0.11904900	-0.53709500	2.63435300
O	1.15883200	-0.54644800	1.95583500

O	-0.04843200	-1.42221700	3.58347200
C	0.97508400	-2.44294900	3.66980600
H	1.94026900	-1.98361500	3.88158200
H	0.65079100	-3.09475400	4.47713400
H	1.03405600	-2.97145500	2.71840900
H	-3.40283400	-0.07880500	4.69653300
H	-1.78952500	-0.80348300	4.96645800
Si	-6.15049500	-0.79523300	0.05850600
C	-7.94893900	-0.40964900	0.46722200
H	-8.06492800	0.59469600	0.88967600
H	-8.57555800	-0.46738800	-0.42826000
H	-8.34121900	-1.12272500	1.20003400
C	-5.92153200	-2.59191800	-0.42270000
H	-4.86929600	-2.82711200	-0.61062000
H	-6.27951100	-3.25366300	0.37331800
H	-6.48524800	-2.82663600	-1.33138500
C	-5.56001600	0.34894400	-1.30870100
C	-6.03267600	1.66876500	-1.41301000
C	-4.62942100	-0.08720000	-2.25989800
C	-5.57884100	2.52234400	-2.41426900
H	-6.78171100	2.03468400	-0.71196800
C	-4.16110800	0.76285800	-3.26049000
H	-4.24480800	-1.10288700	-2.21264100
C	-4.63584200	2.06898200	-3.33704100
H	-5.96174100	3.53651800	-2.48047400
H	-3.41960200	0.40054900	-3.96506900
H	-4.27555200	2.73401800	-4.11616800

10. Copies of NMR spectra

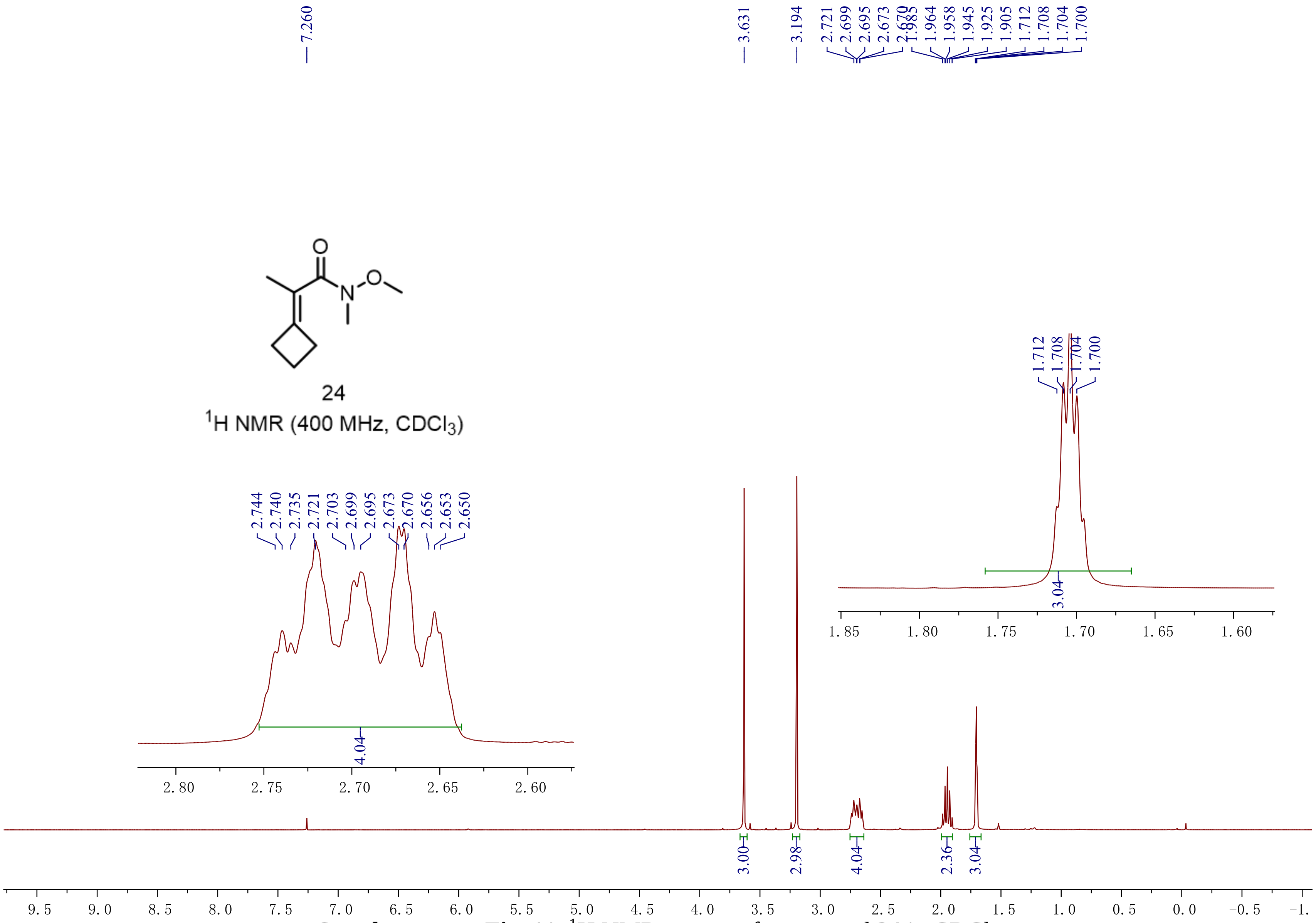

Supplementary Fig. 11. ^{1}H NMR spectra of compound **24** in $CDCl_3$

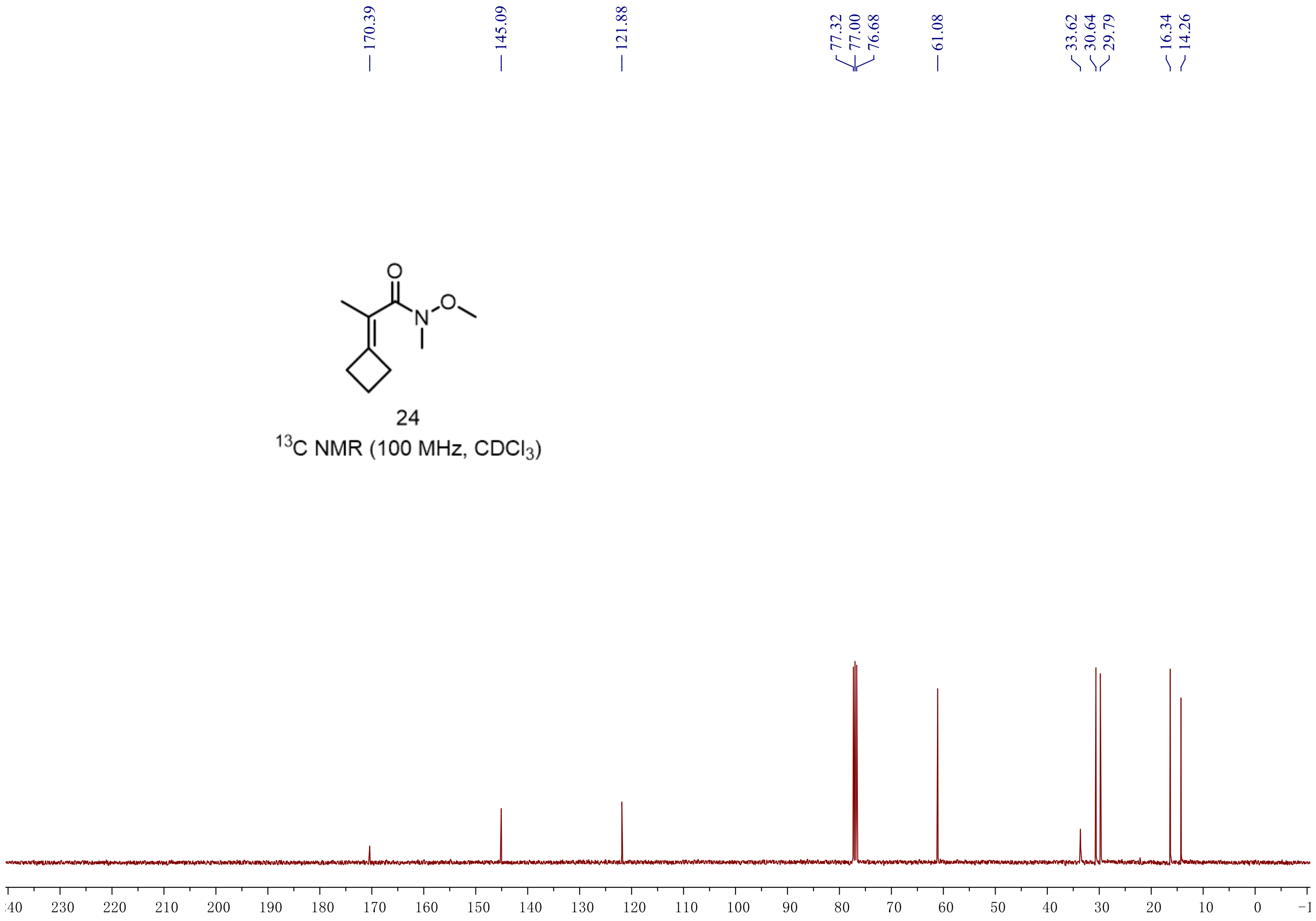

Supplementary Fig. 12. ^{13}C NMR spectra of compound **24** in $CDCl_3$

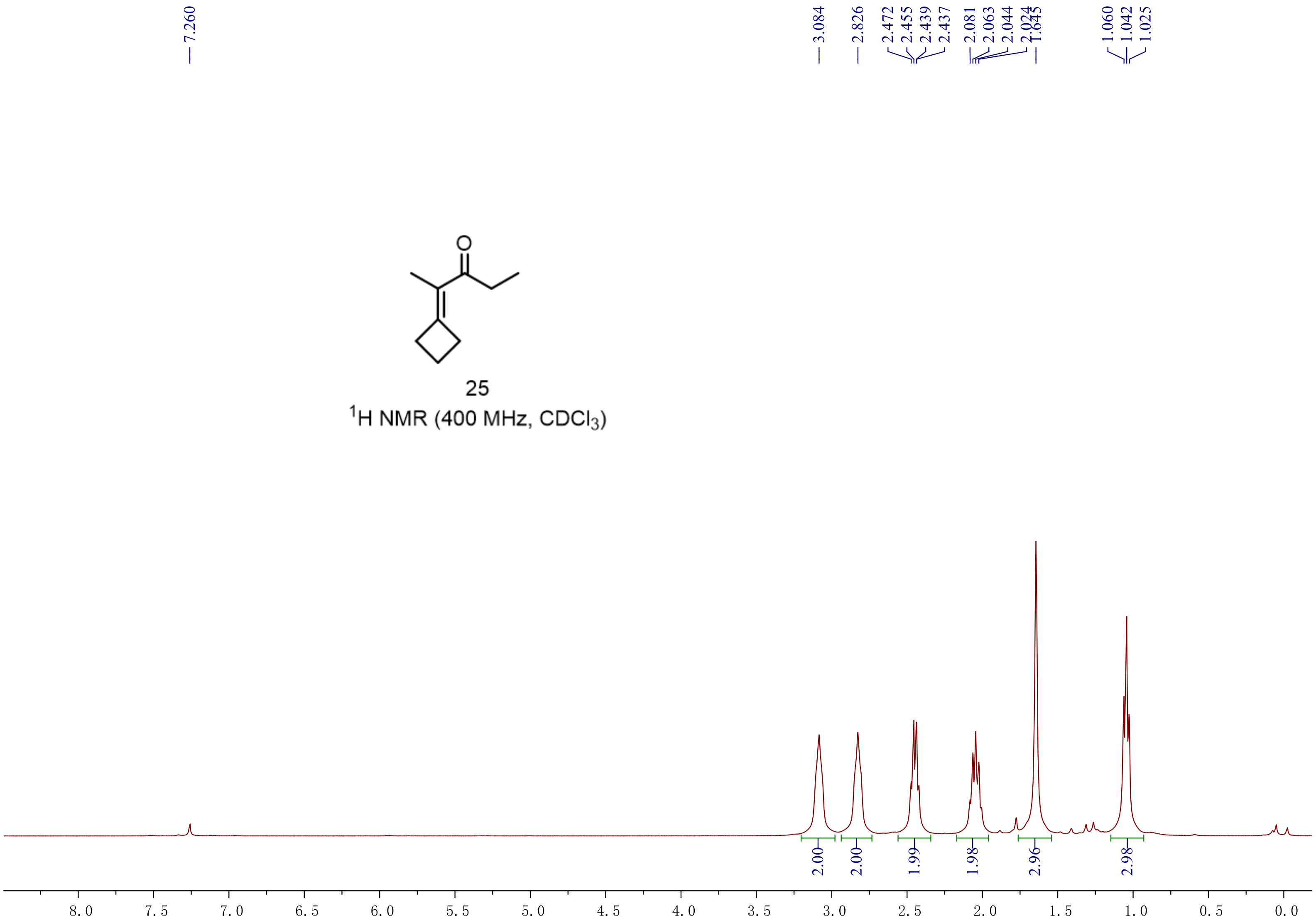

Supplementary Fig. 13. ^{1}H NMR spectra of compound **25** in $CDCl_3$

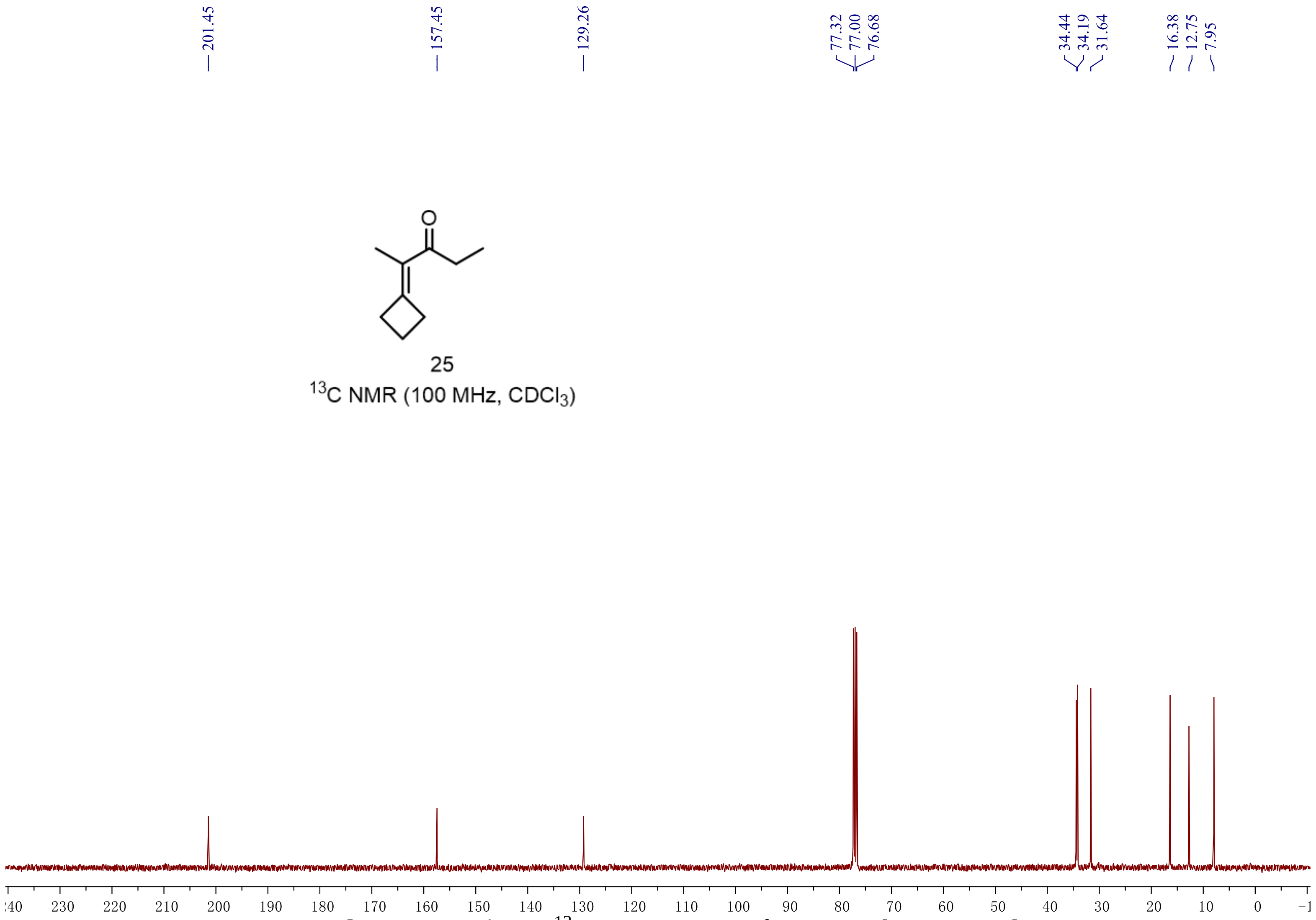

Supplementary Fig. 14. ^{13}C NMR spectra of compound **25** in $CDCl_3$

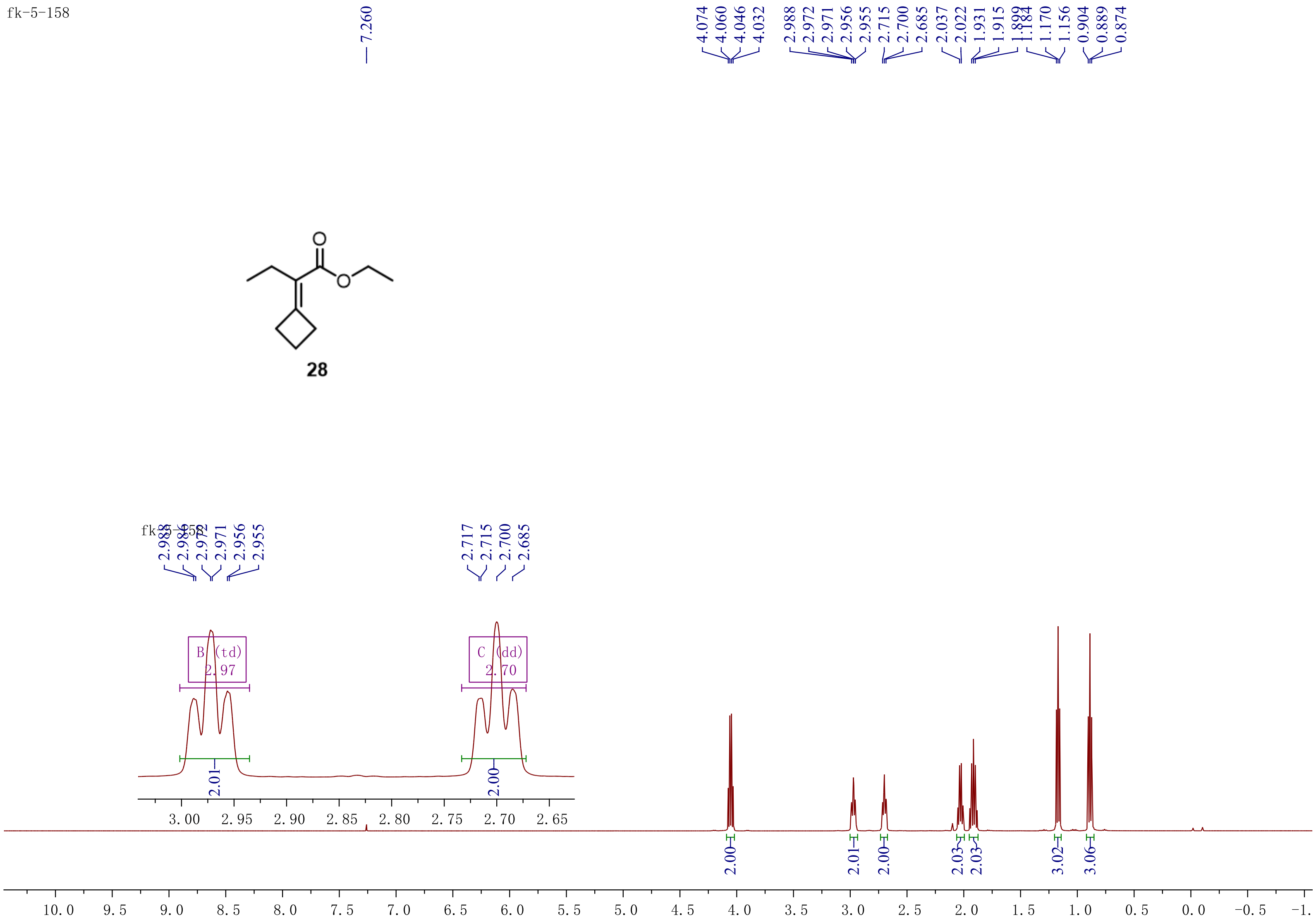

Supplementary Fig. 15. ^{1}H NMR spectra of compound **28** in $CDCl_3$

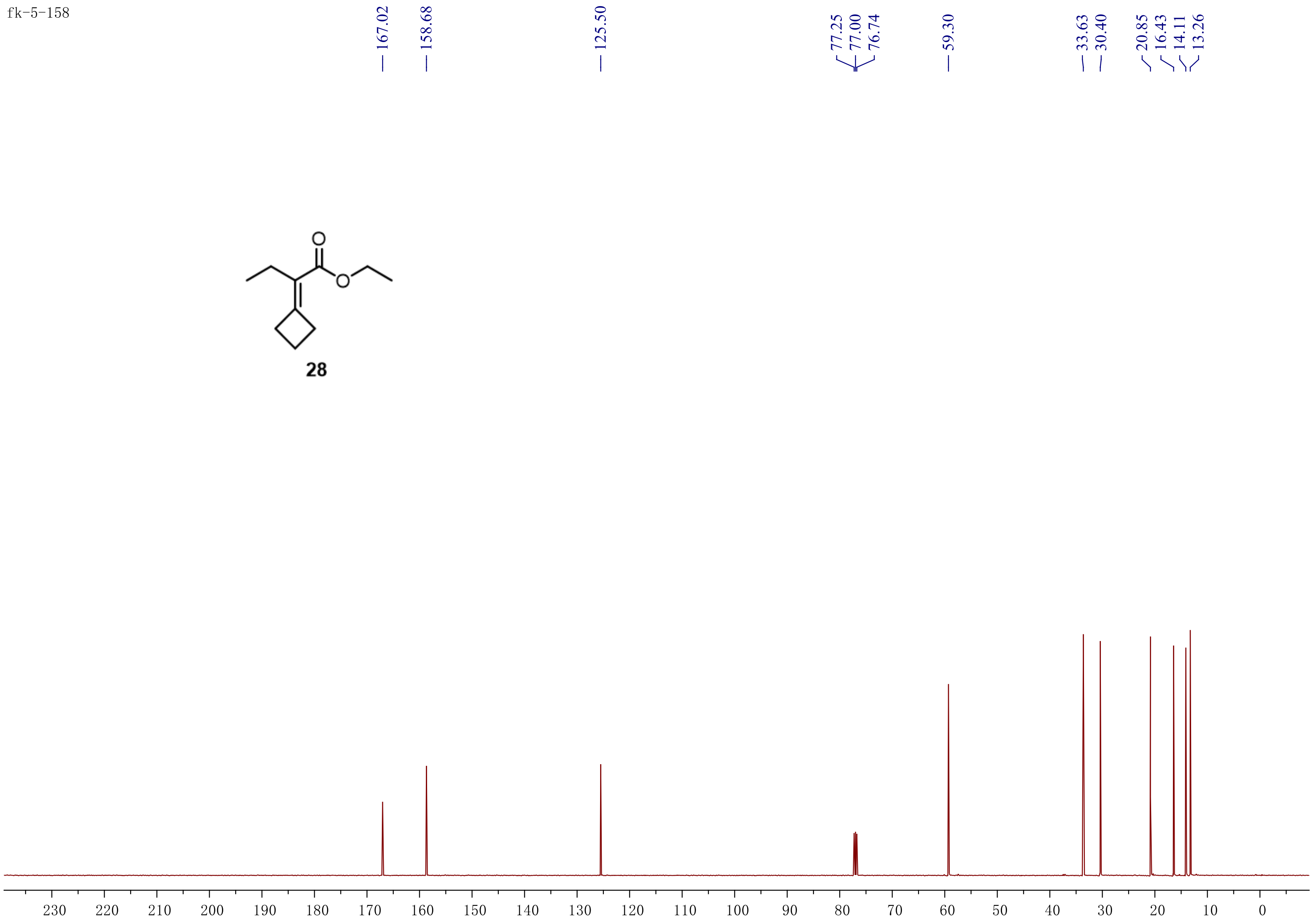

28

Supplementary Fig. 16. ^{13}C NMR spectra of compound **28** in $CDCl_3$

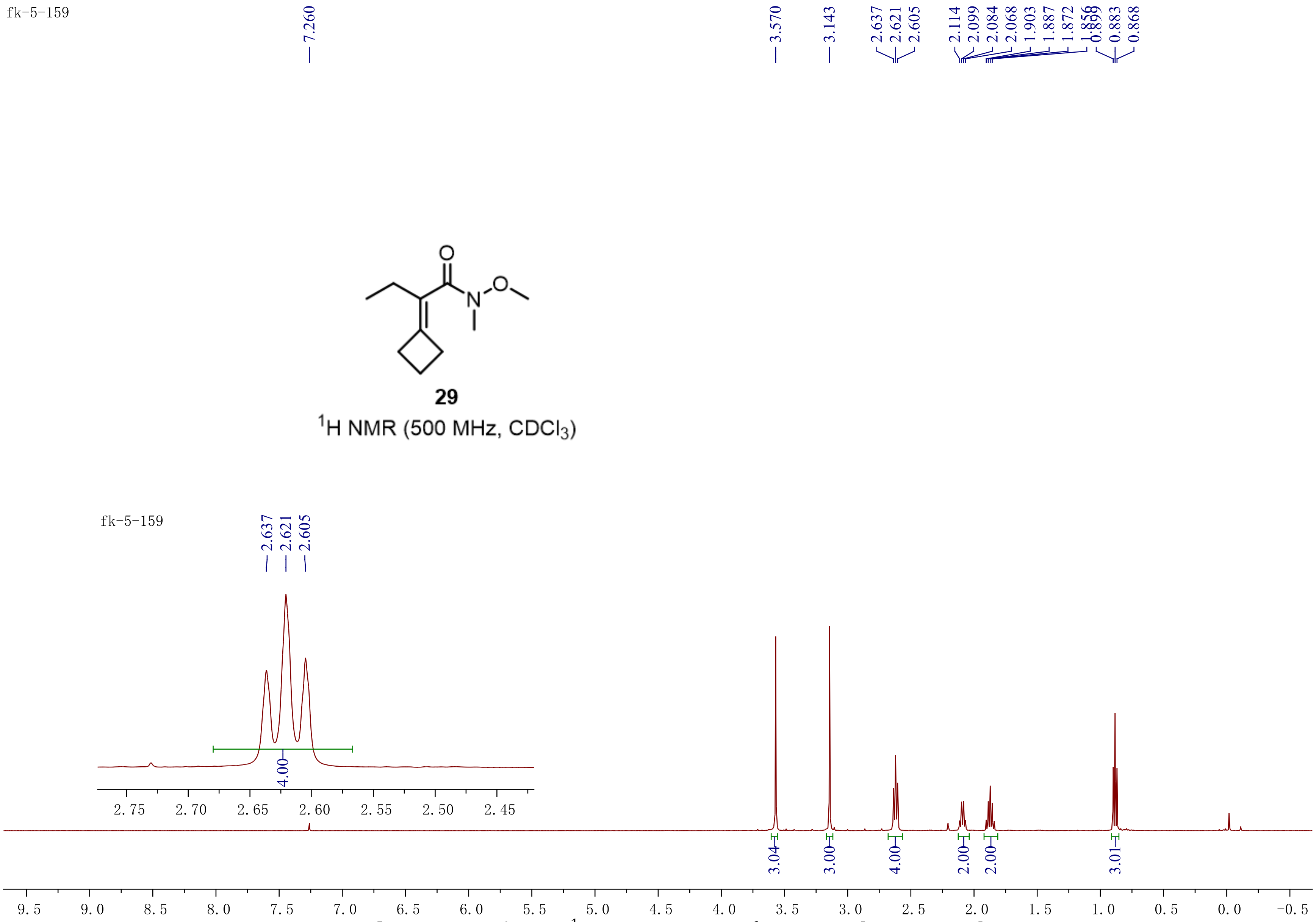

Supplementary Fig. 17. ^{1}H NMR spectra of compound **29** in $CDCl_3$

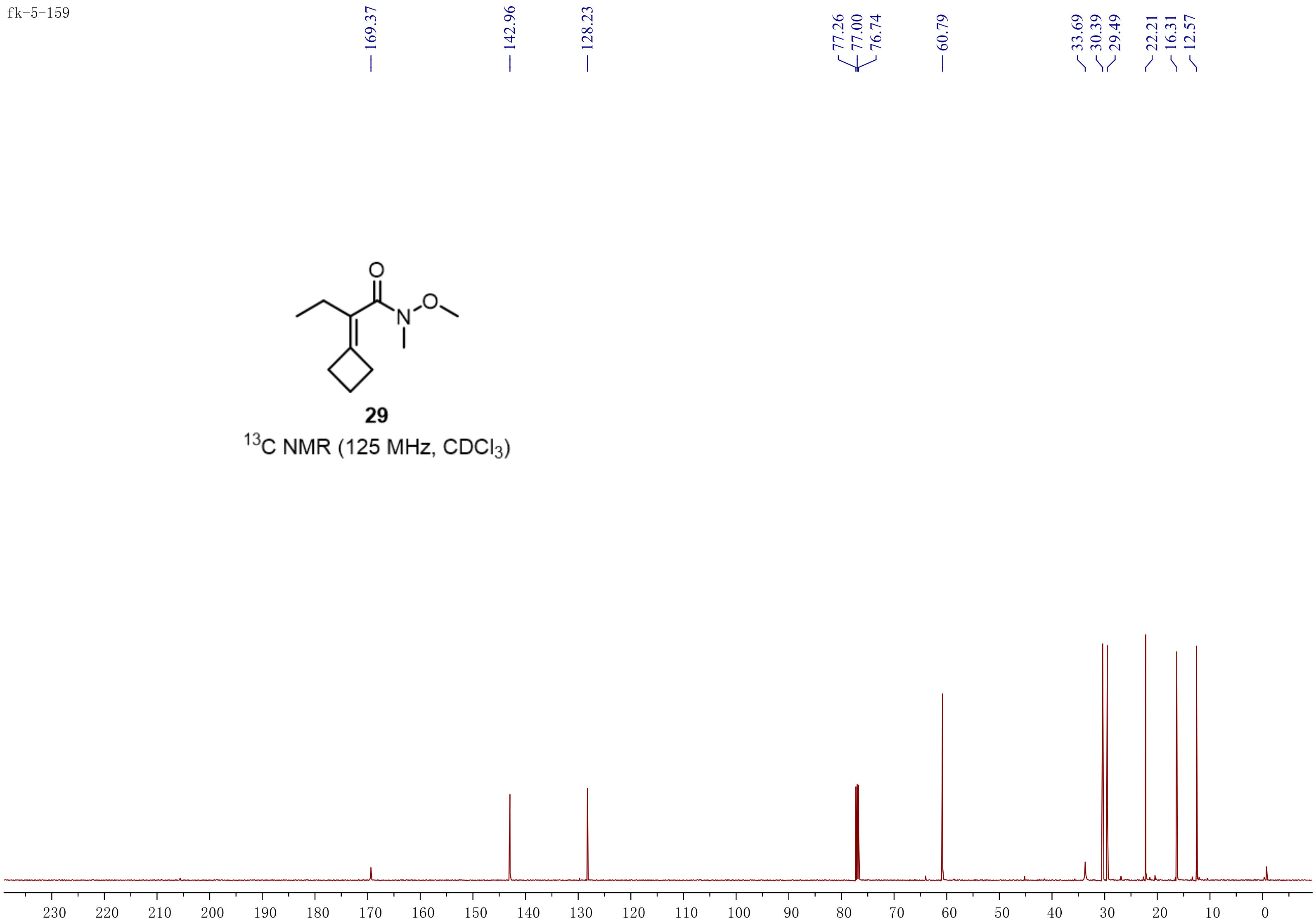

Supplementary Fig. 18.^{13}C NMR spectra of compound **29** in $CDCl_3$

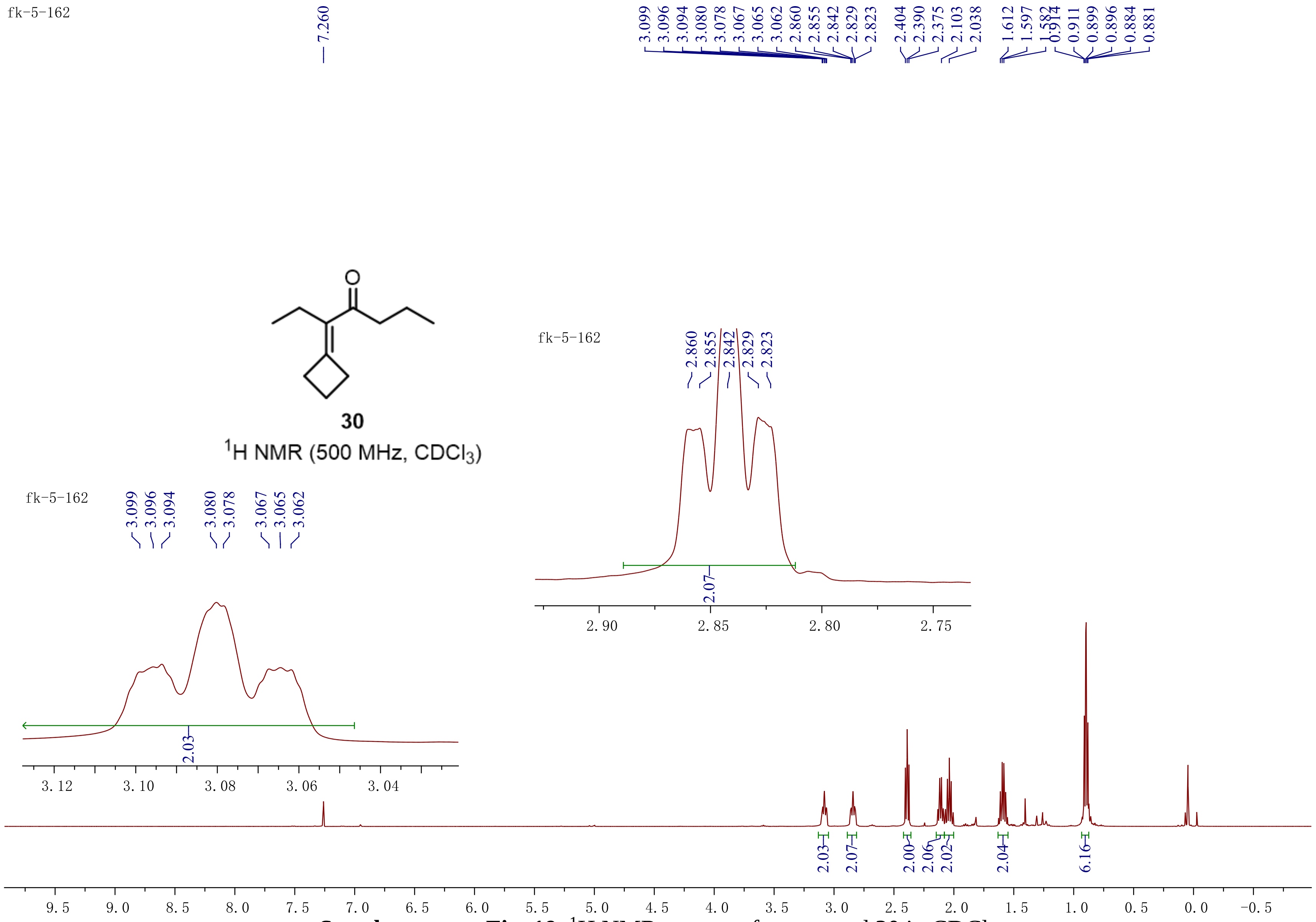

Supplementary Fig. 19. ^{1}H NMR spectra of compound **30** in $CDCl_3$

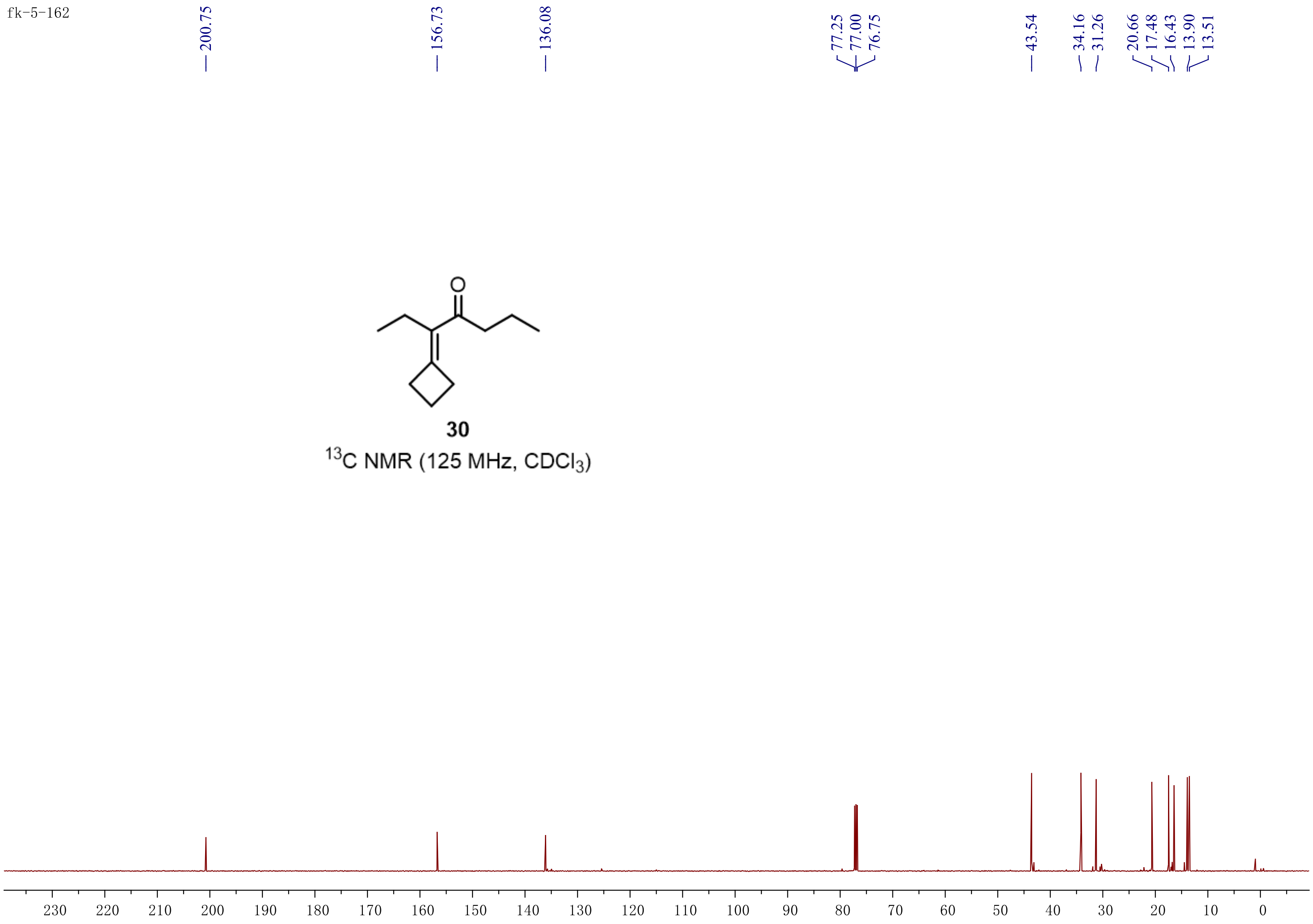

Supplementary Fig. 20.^{13}C NMR spectra of compound **30** in $CDCl_3$

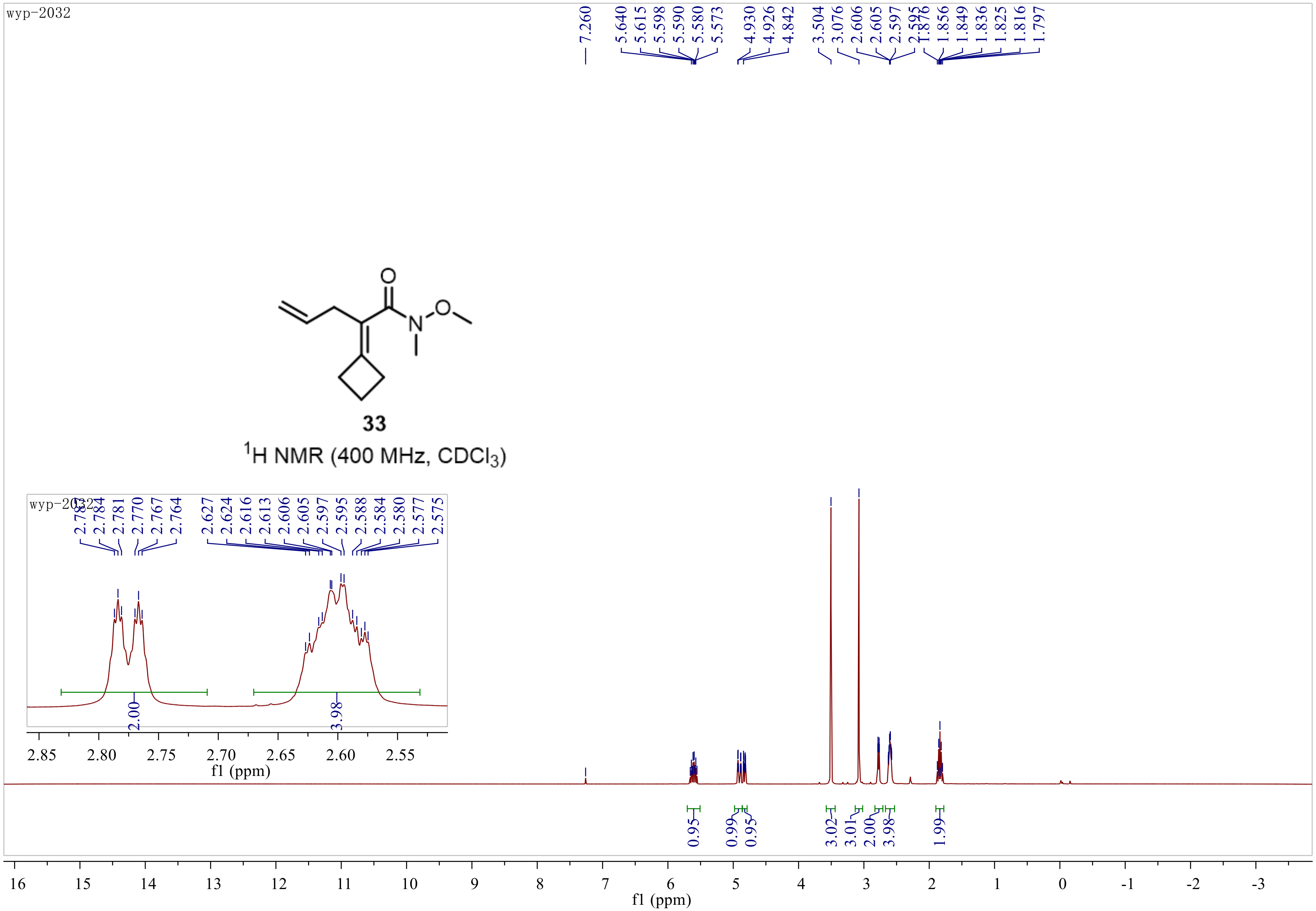

Supplementary Fig. 21. ^{1}H NMR spectra of compound **33** in $CDCl_3$

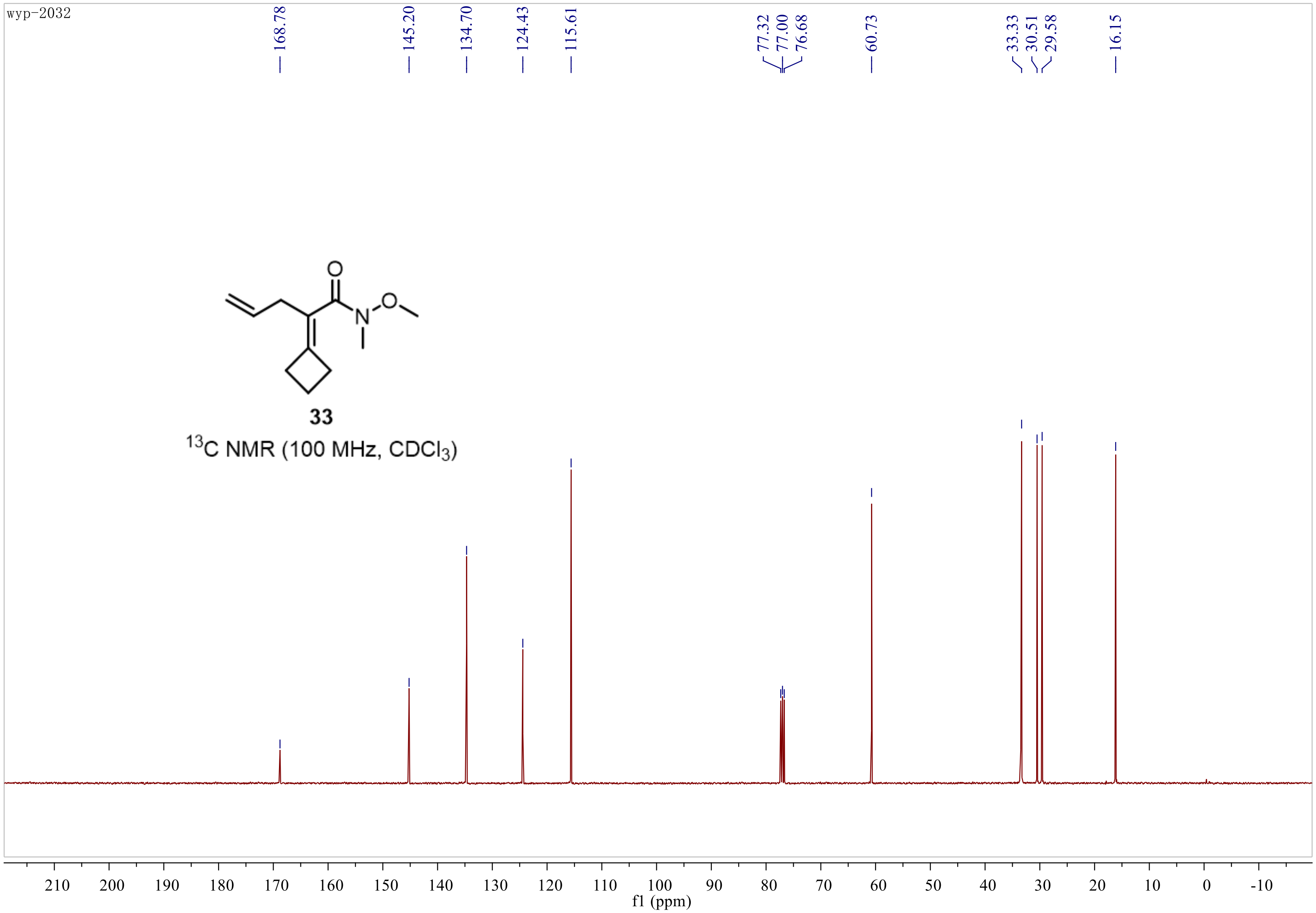

Supplementary Fig. 22. ^{13}C NMR spectra of compound **33** in $CDCl_3$

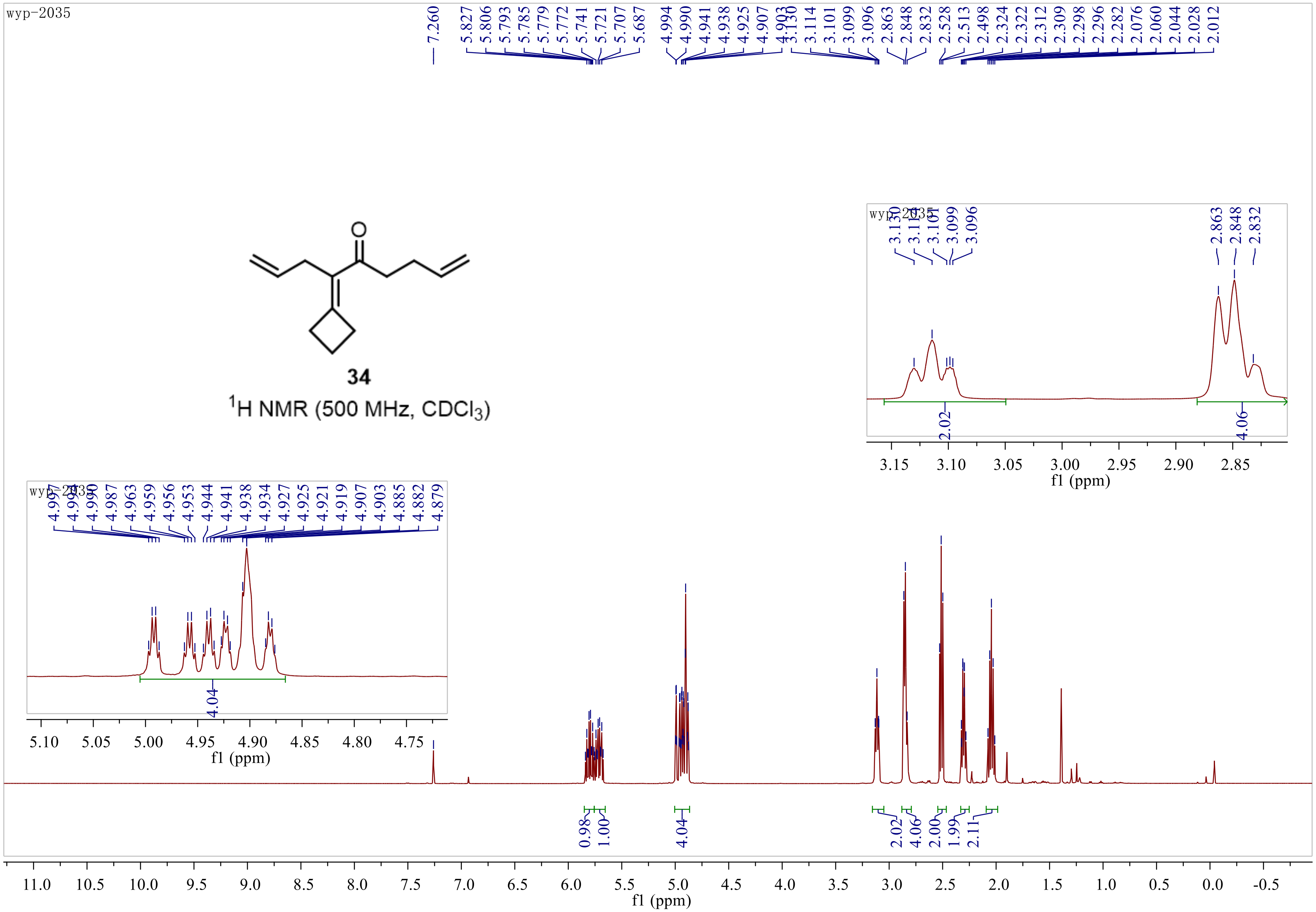

Supplementary Fig. 23. ^{1}H NMR spectra of compound **34** in $CDCl_3$

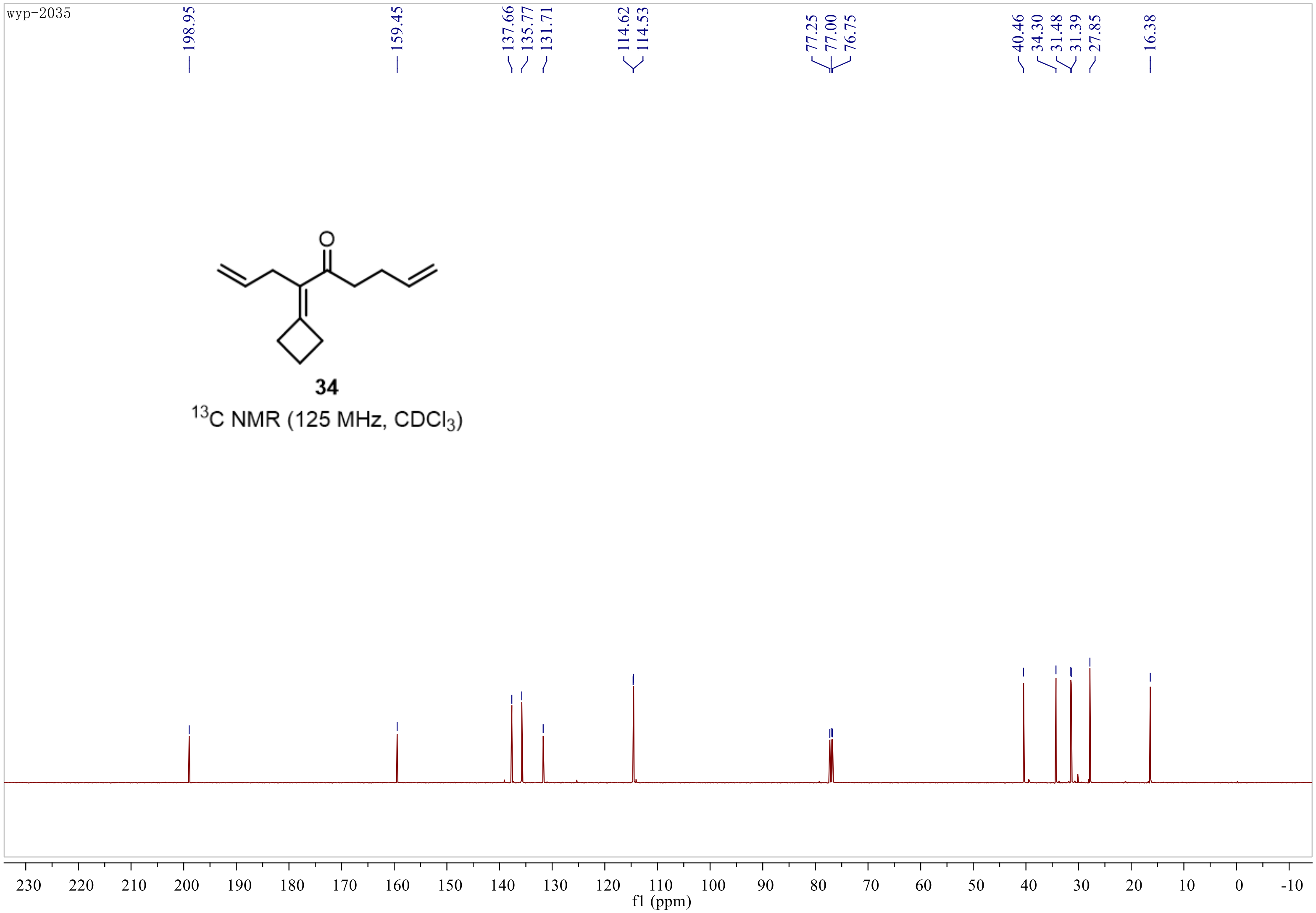

Supplementary Fig. 24. ^{13}C NMR spectra of compound **34** in $CDCl_3$

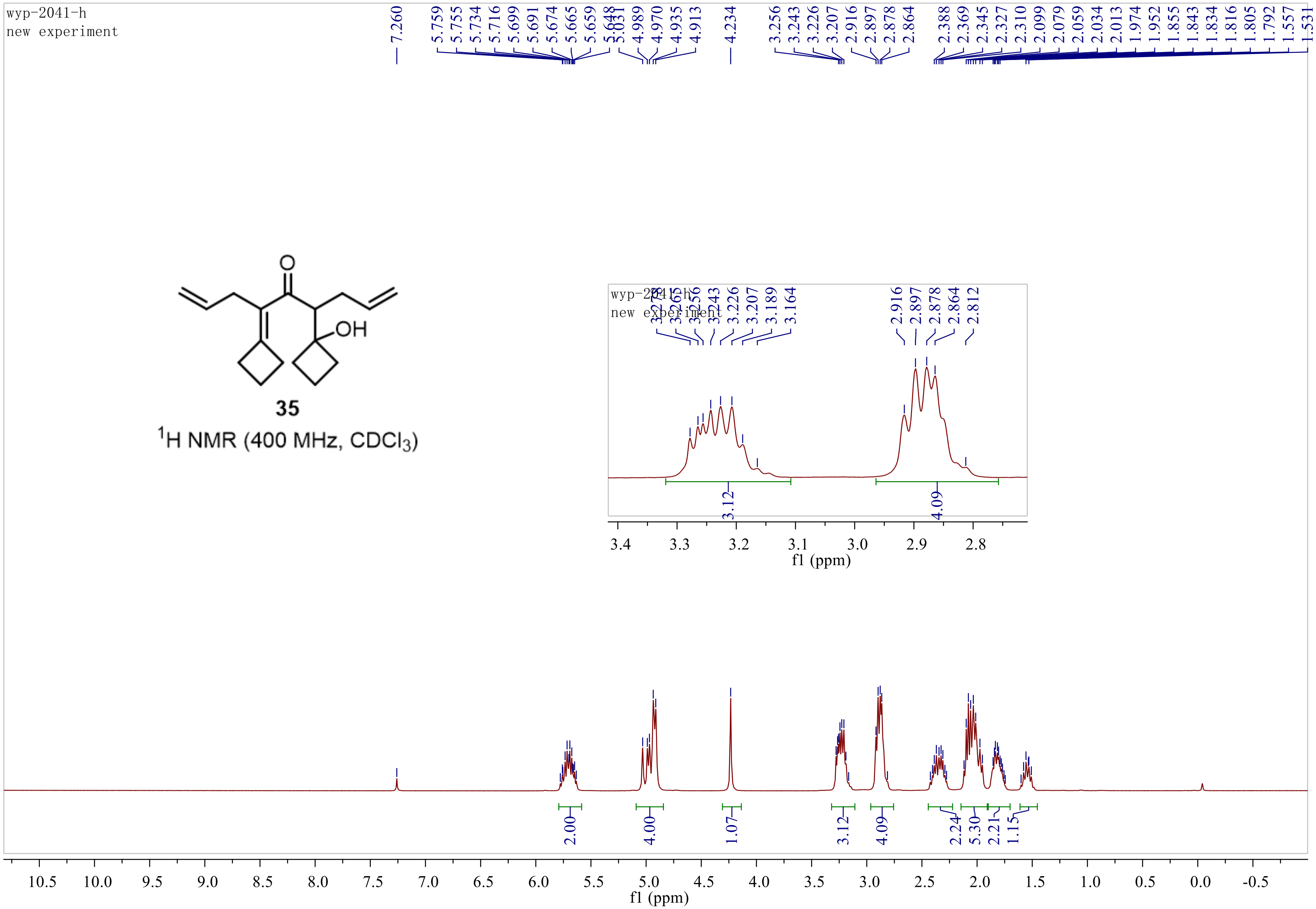

Supplementary Fig. 25. ^{1}H NMR spectra of compound **35** in $CDCl_3$

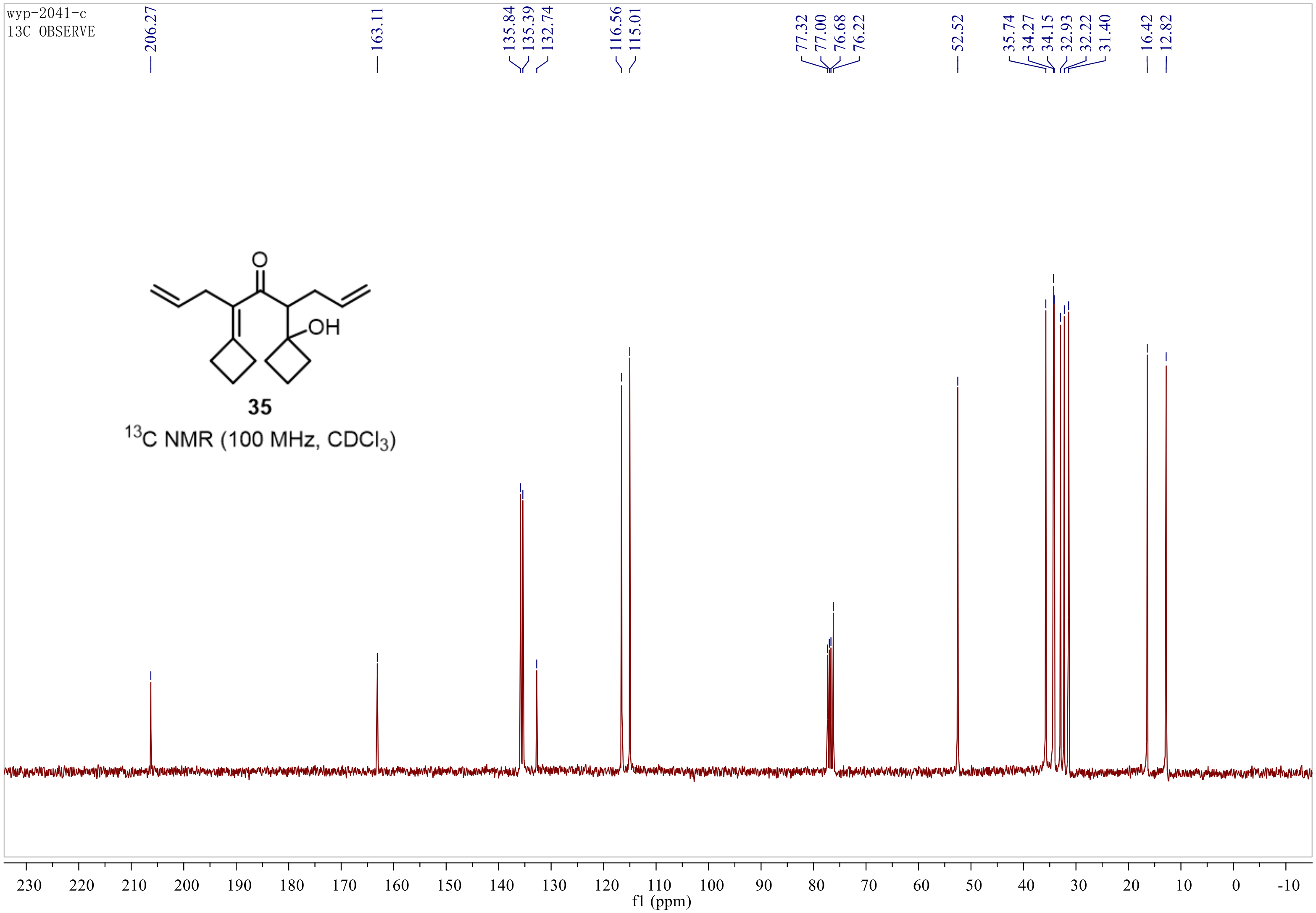

Supplementary Fig. 26.^{13}C NMR spectra of compound **35** in $CDCl_3$

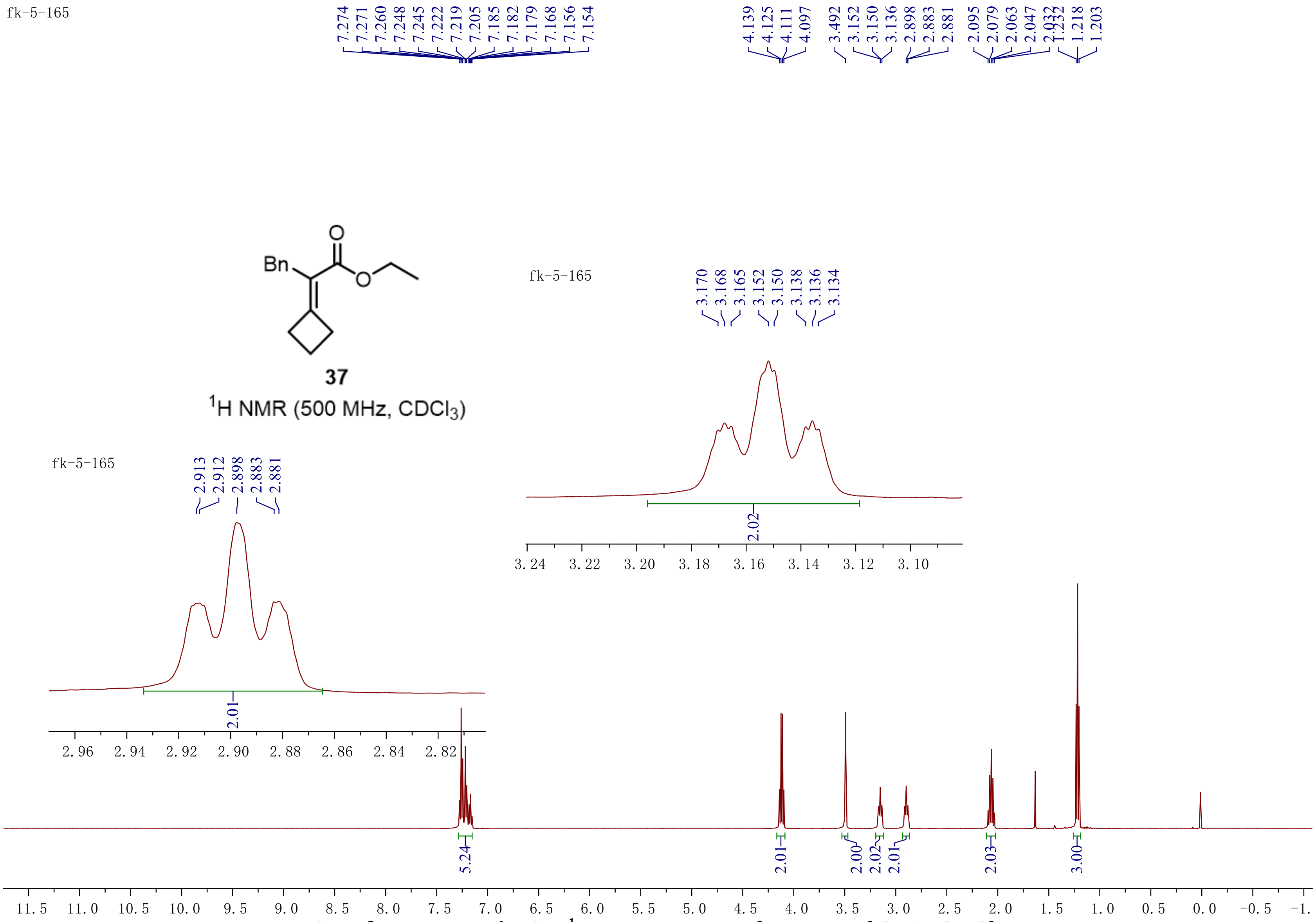

Supplementary Fig. 27. ^{1}H NMR spectra of compound **37** in $CDCl_3$

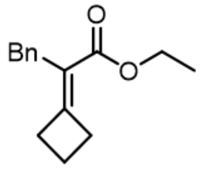

37

^{13}C NMR (125 MHz, $CDCl_3$)

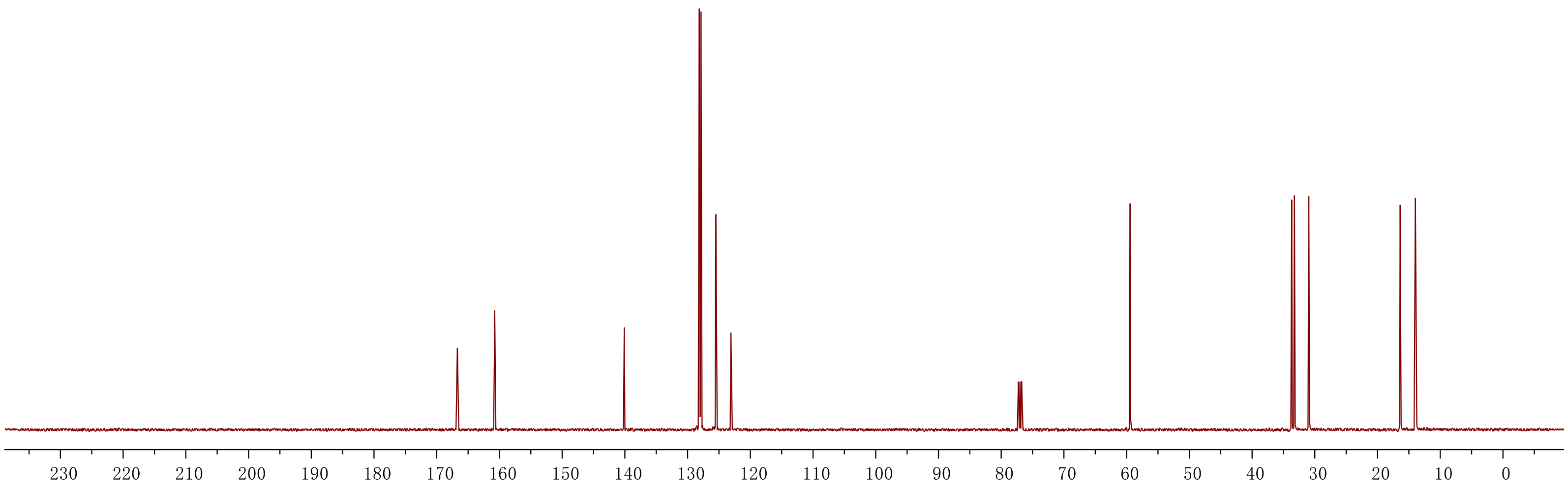

Supplementary Fig. 28. ^{13}C NMR spectra of compound **37** in $CDCl_3$

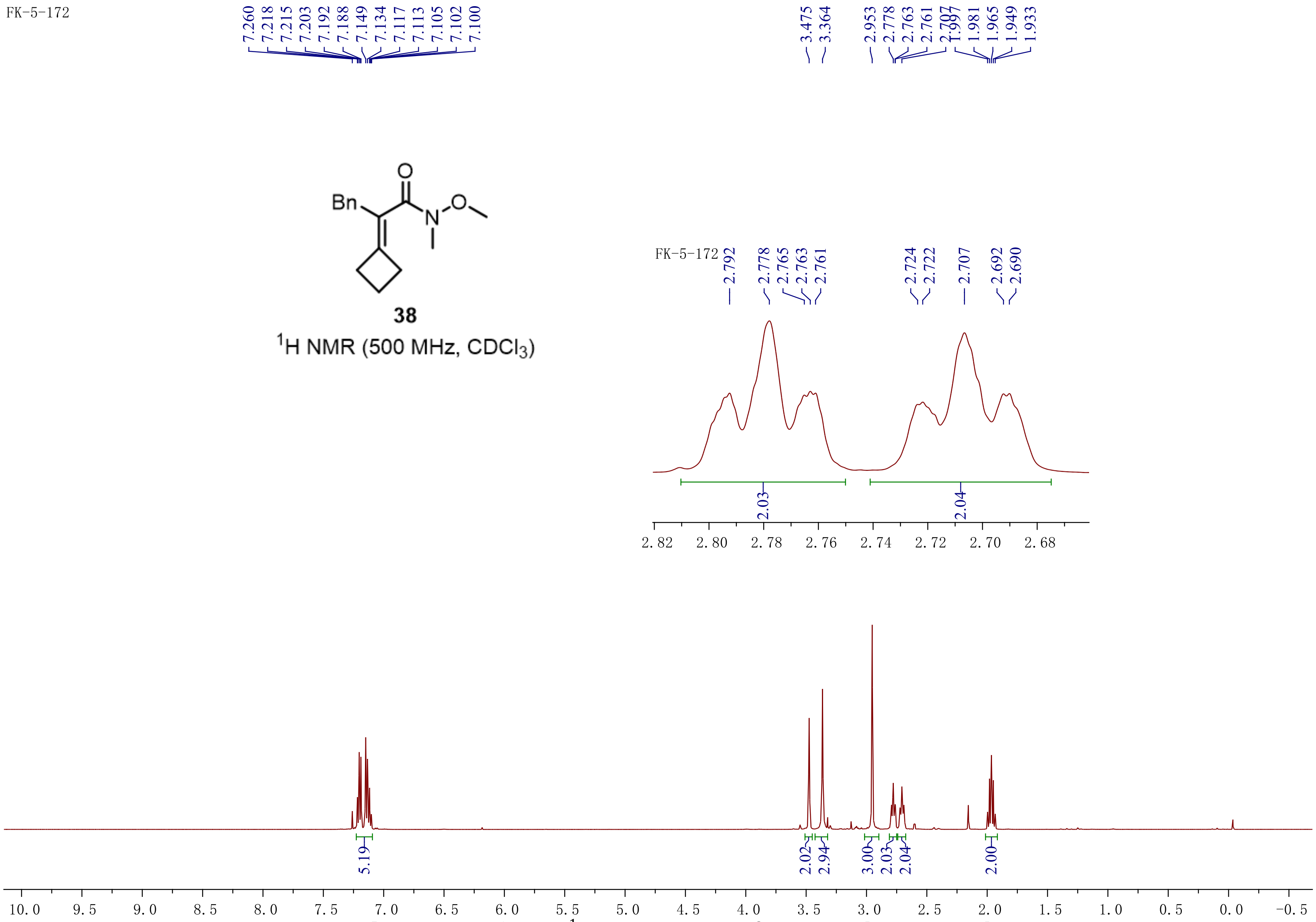

Supplementary Fig. 29. ^{1}H NMR spectra of compound **38** in $CDCl_3$

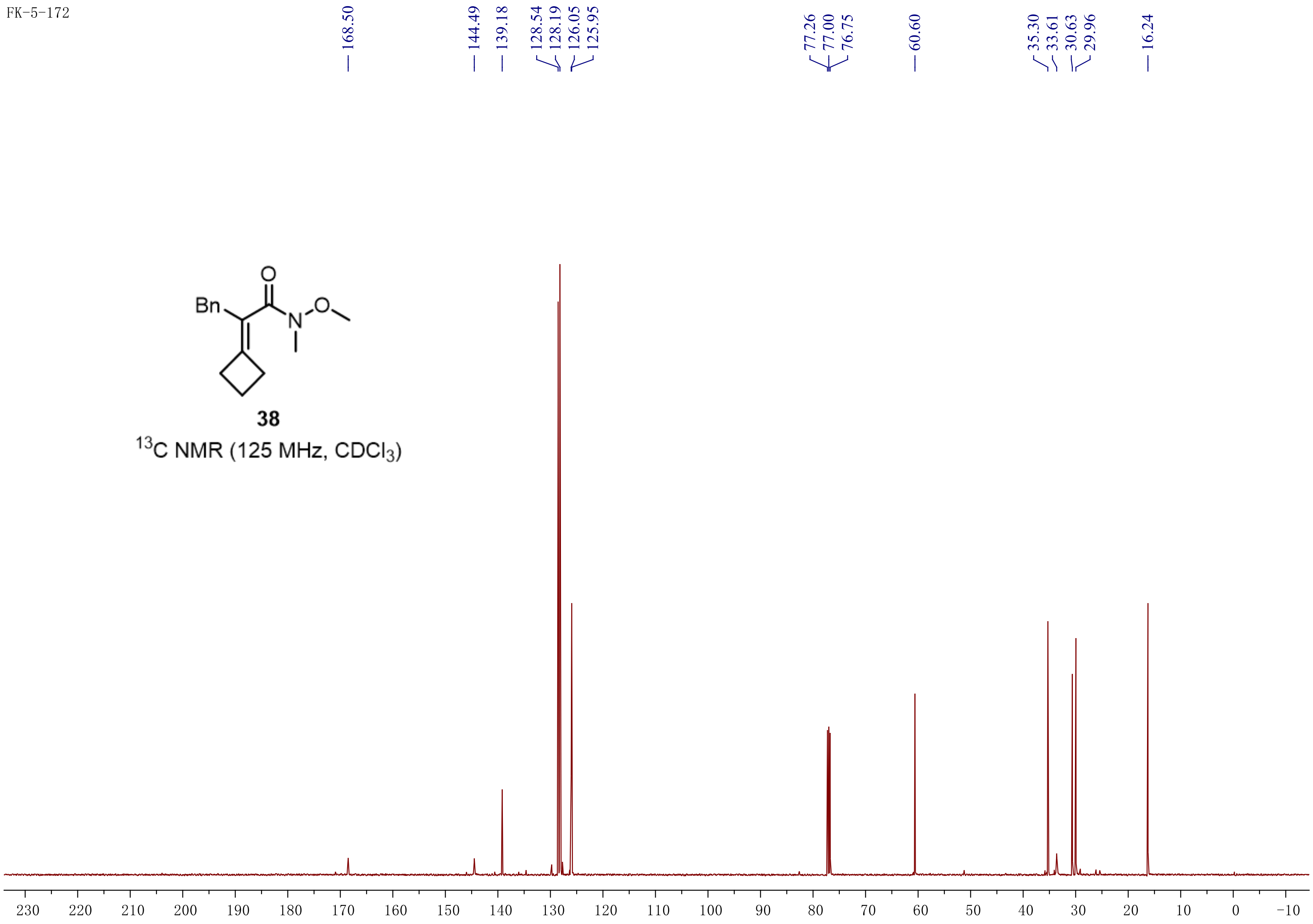

Supplementary Fig. 30. ^{13}C NMR spectra of compound **38** in $CDCl_3$

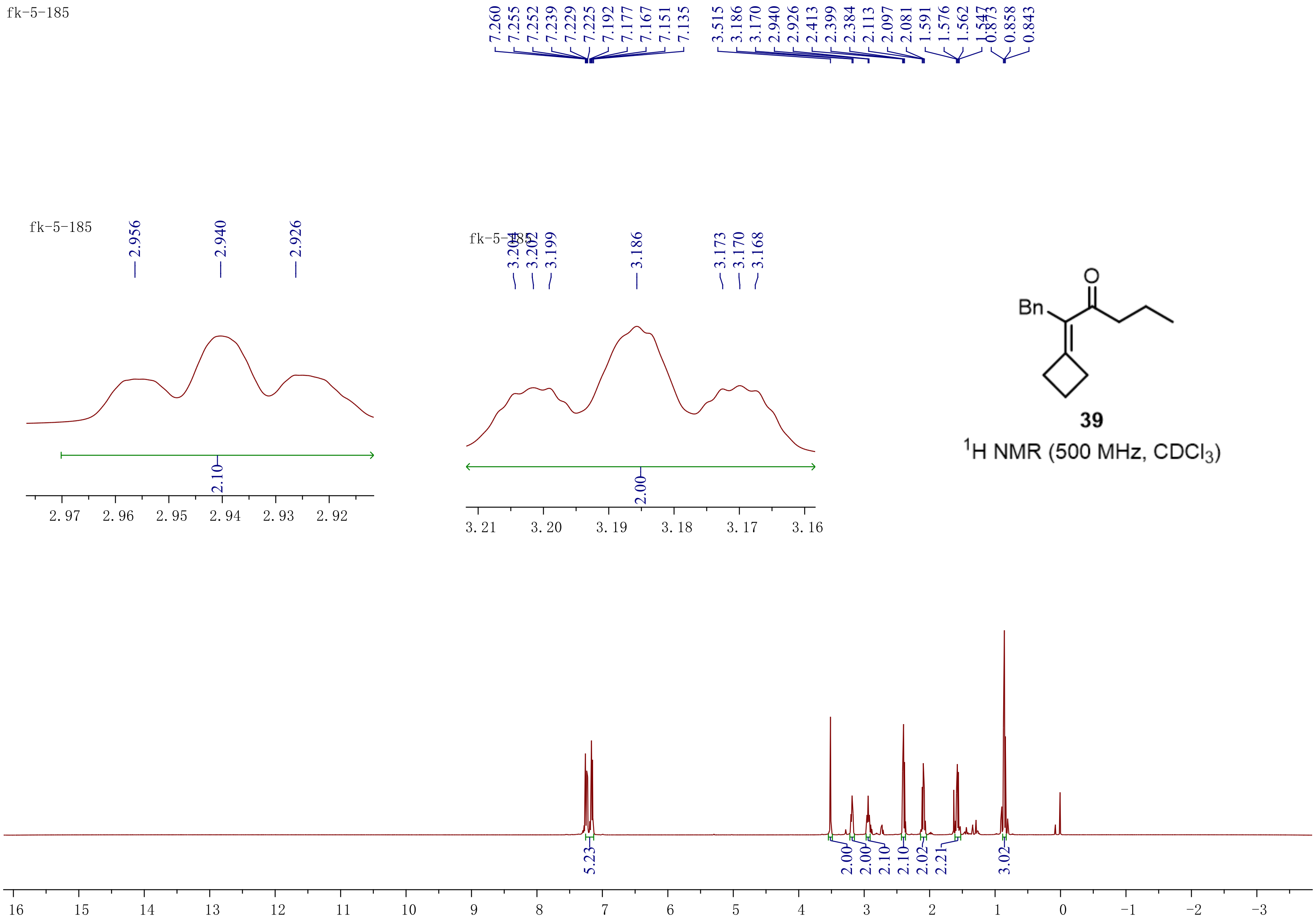

Supplementary Fig. 31. ^{1}H NMR spectra of compound **39** in $CDCl_3$

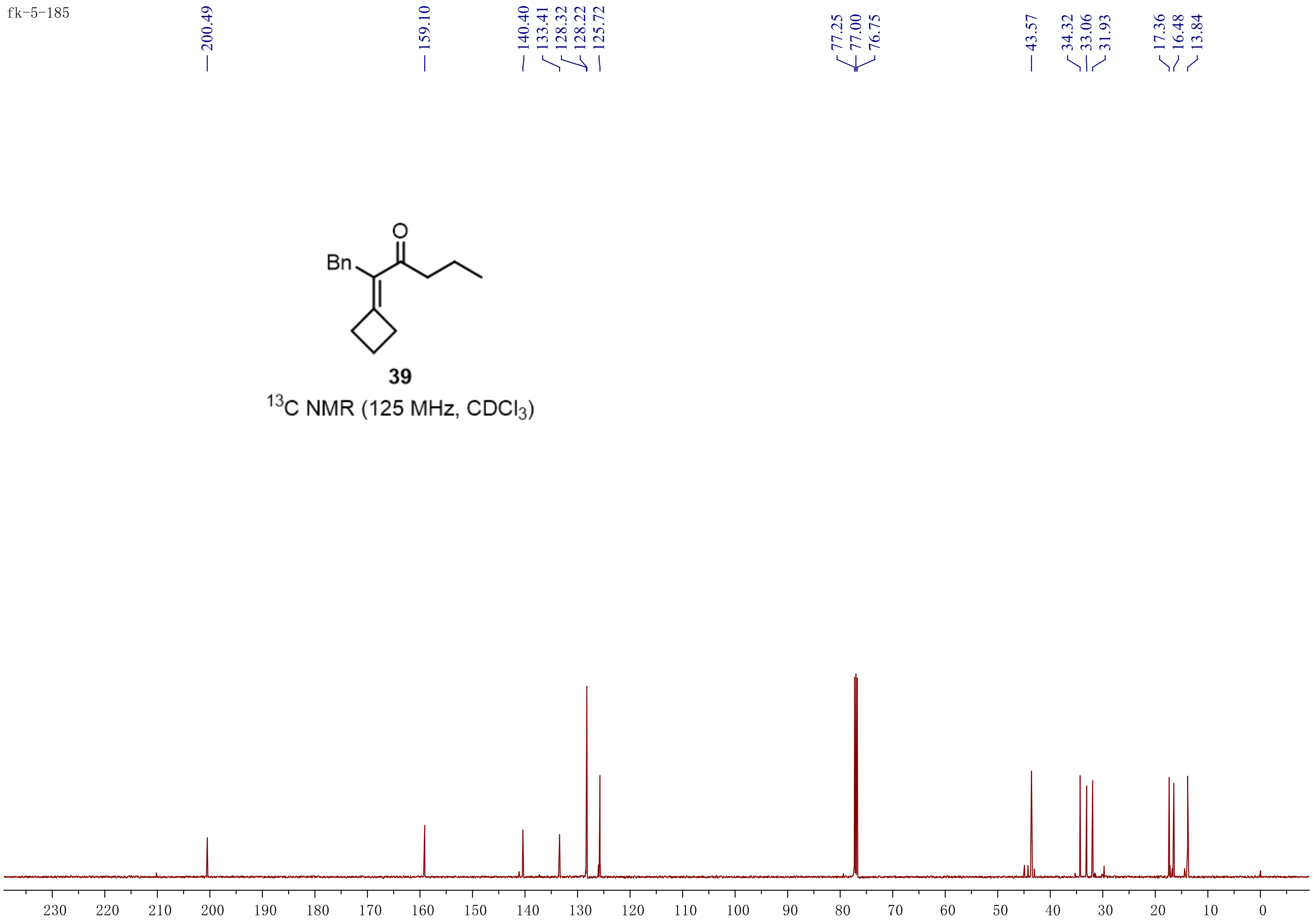

Supplementary Fig. 32. ^{13}C NMR spectra of compound **39** in $CDCl_3$

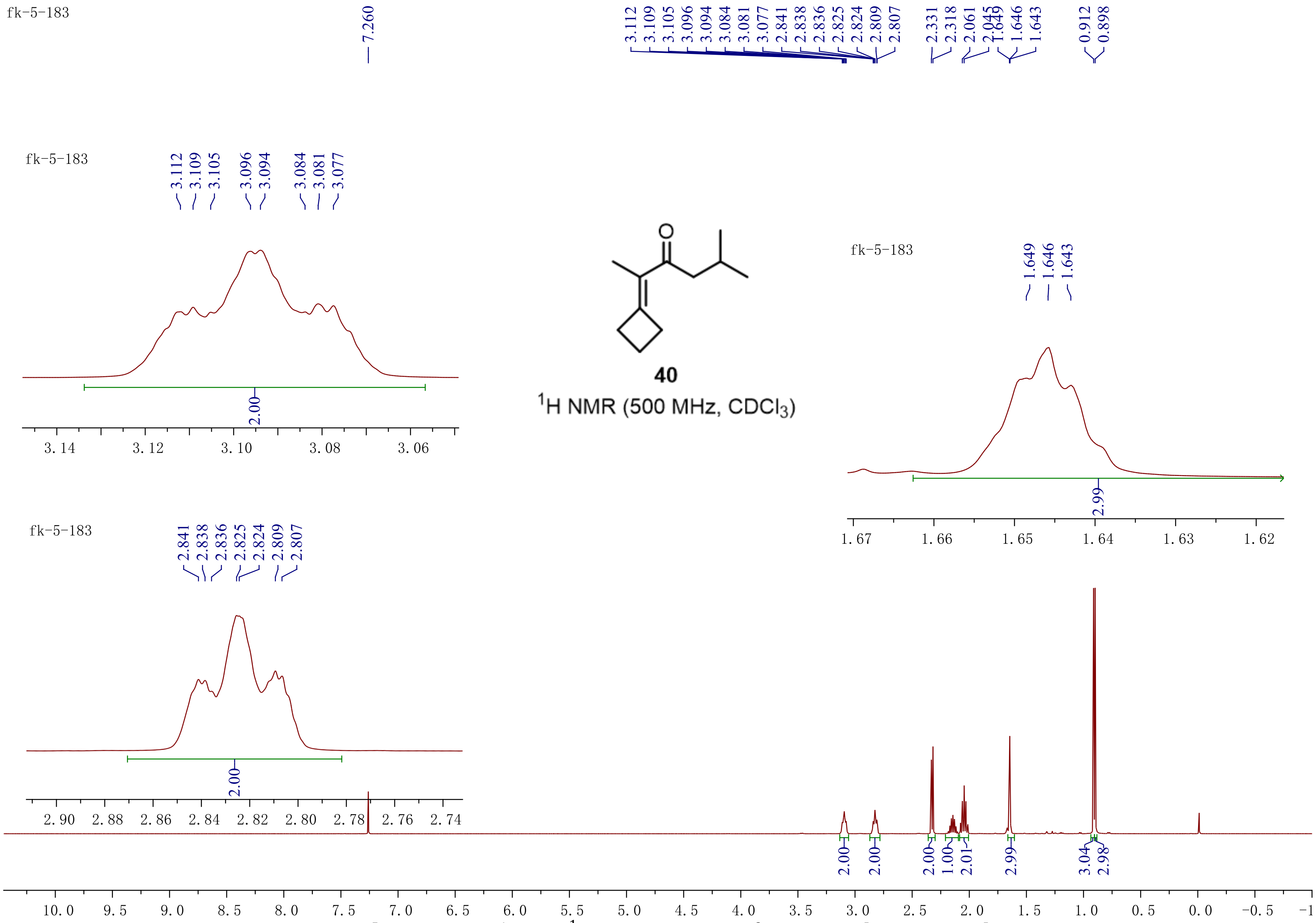

Supplementary Fig. 33. ^{1}H NMR spectra of compound **40** in $CDCl_3$

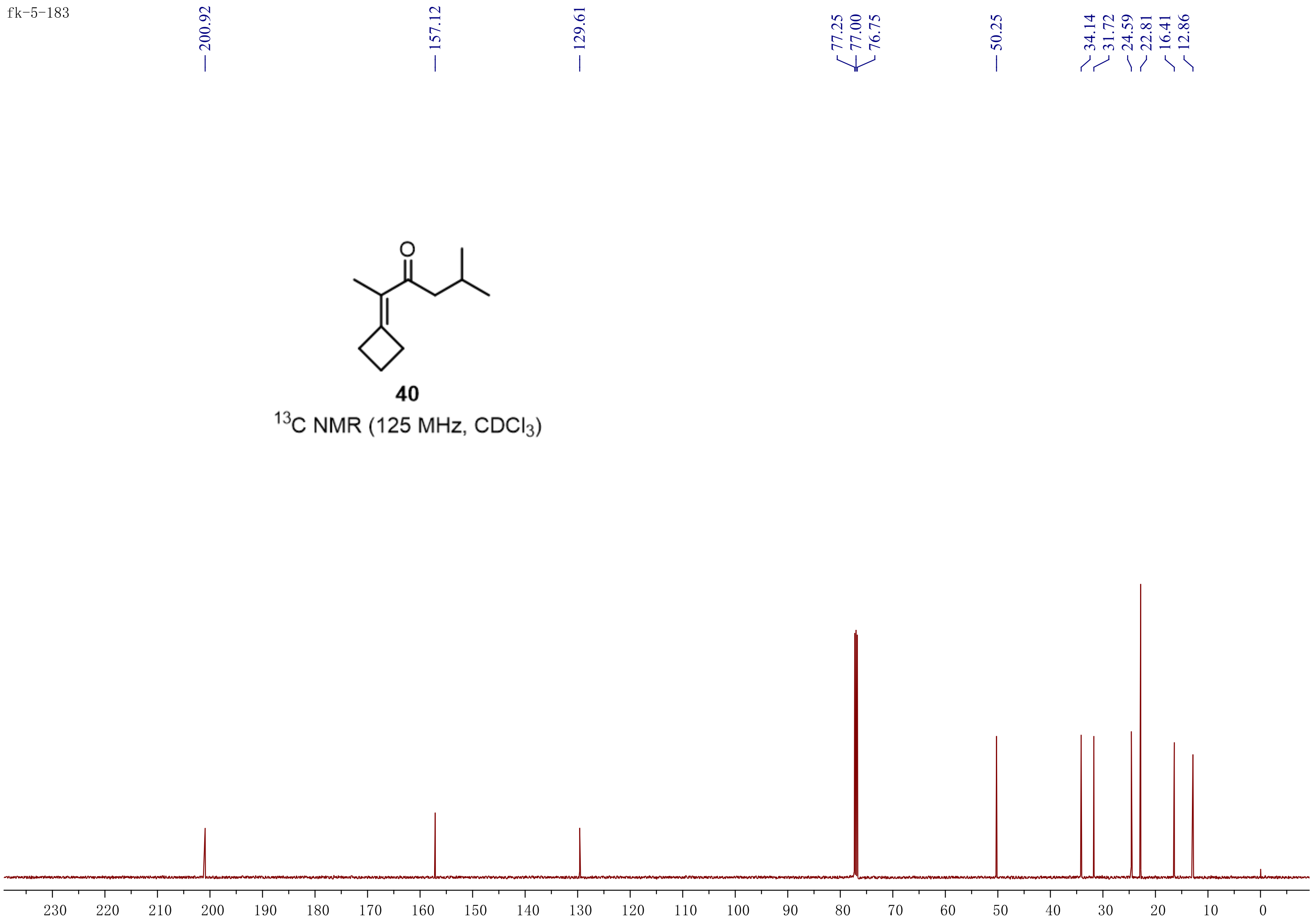

Supplementary Fig. 34. ^{13}C NMR spectra of compound **40** in $CDCl_3$

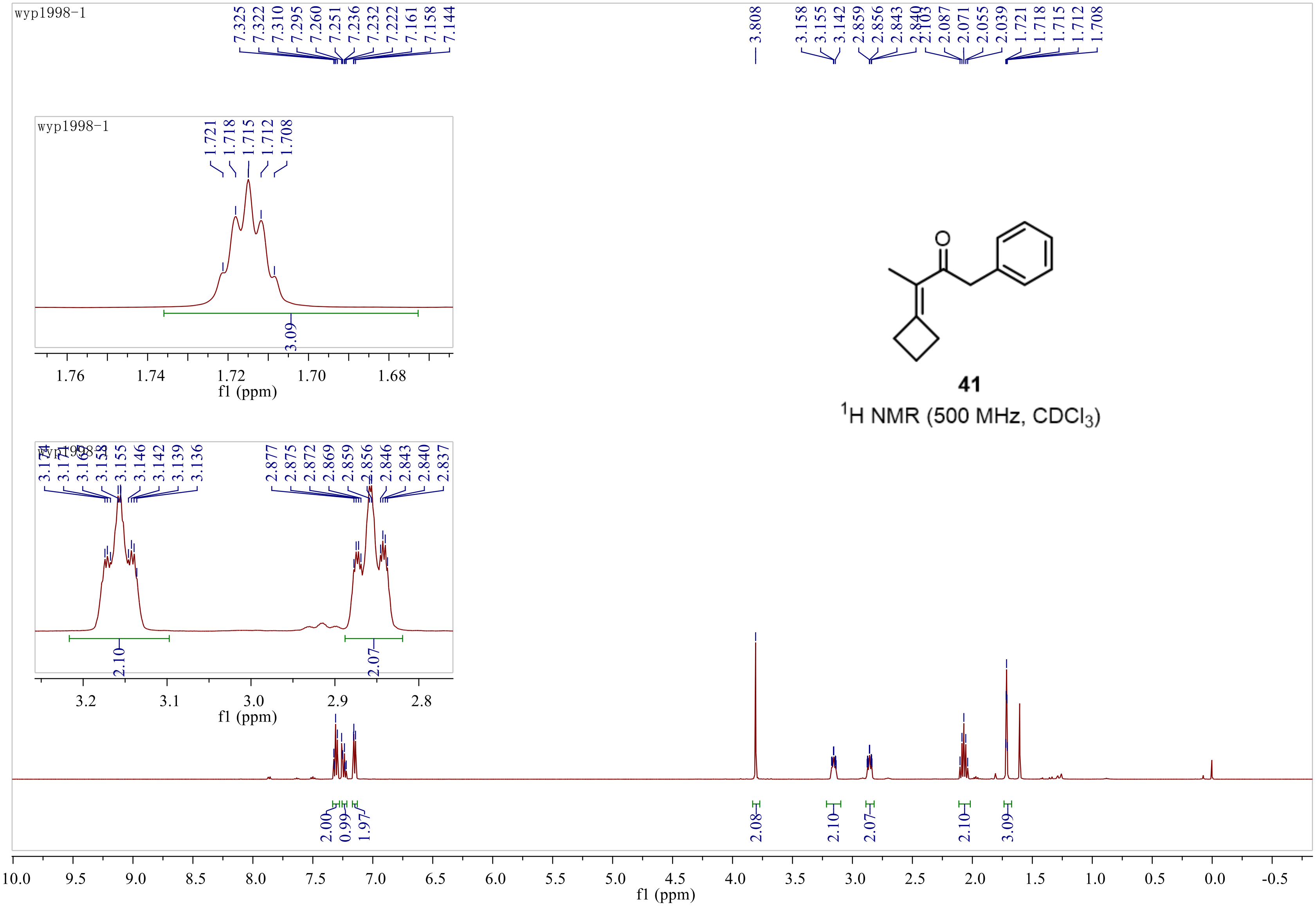

Supplementary Fig. 35. ^{1}H NMR spectra of compound **41** in $CDCl_3$

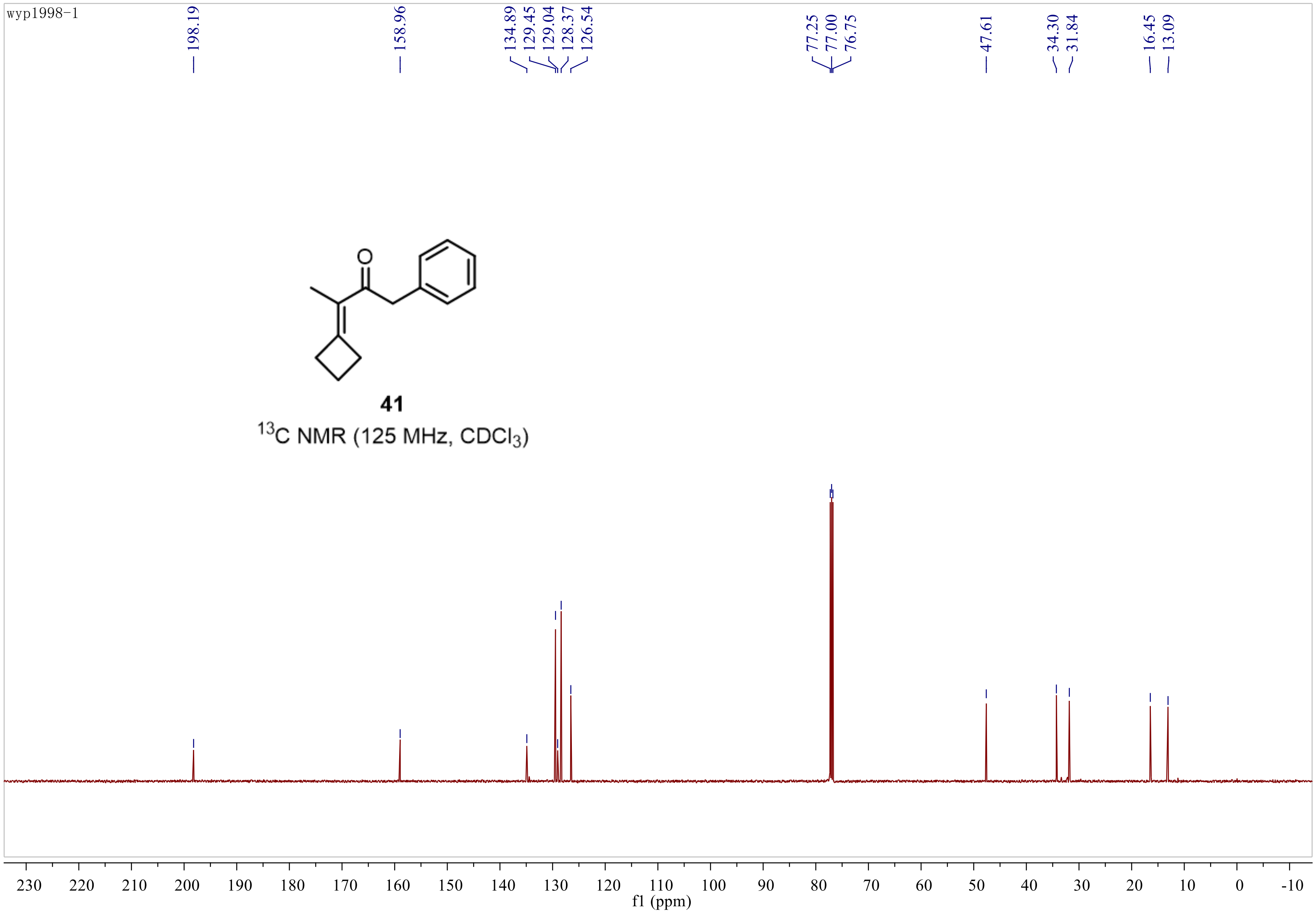

Supplementary Fig. 36. ^{13}C NMR spectra of compound **41** in $CDCl_3$

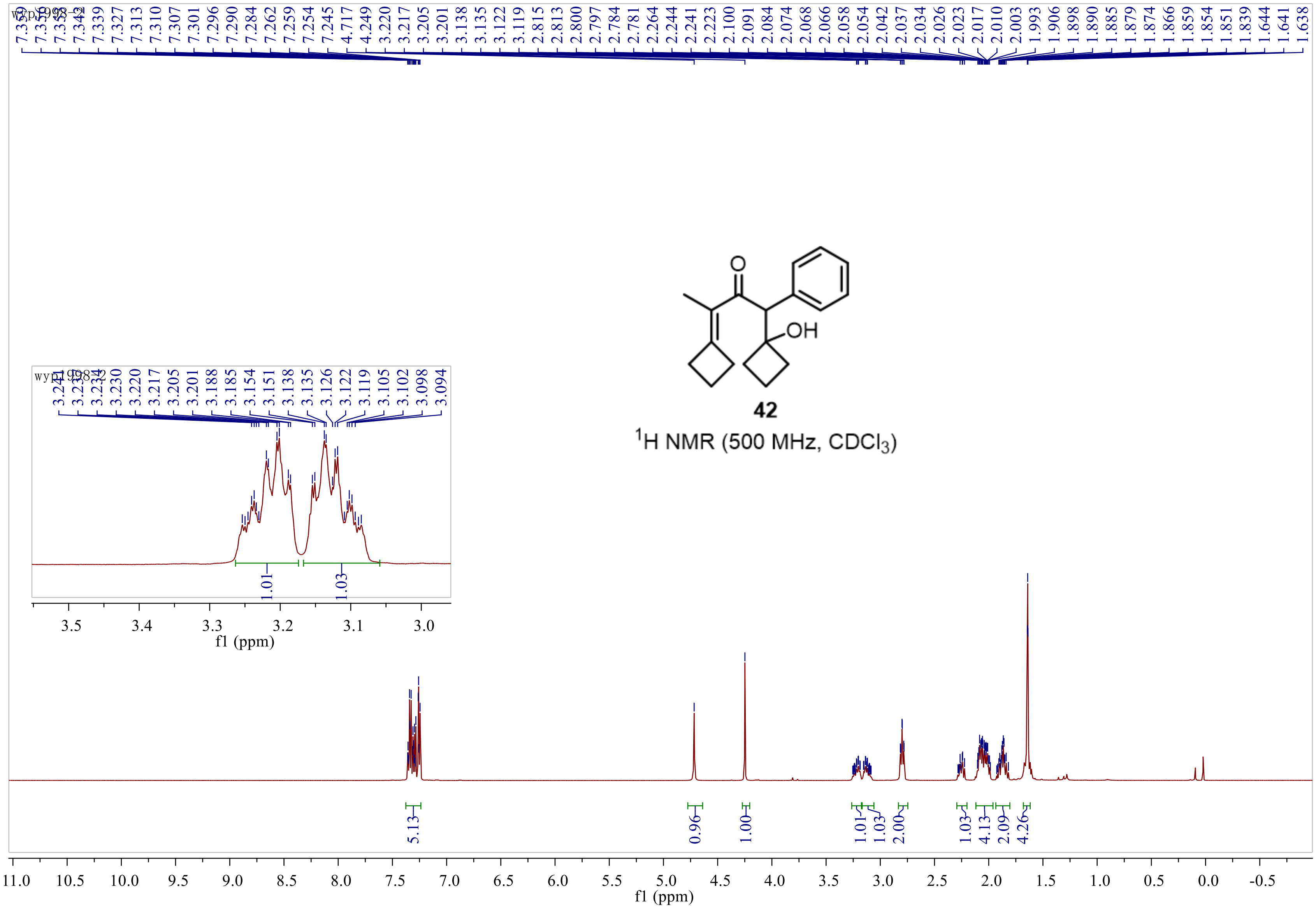

Supplementary Fig. 37. ^{1}H NMR spectra of compound **42** in $CDCl_3$

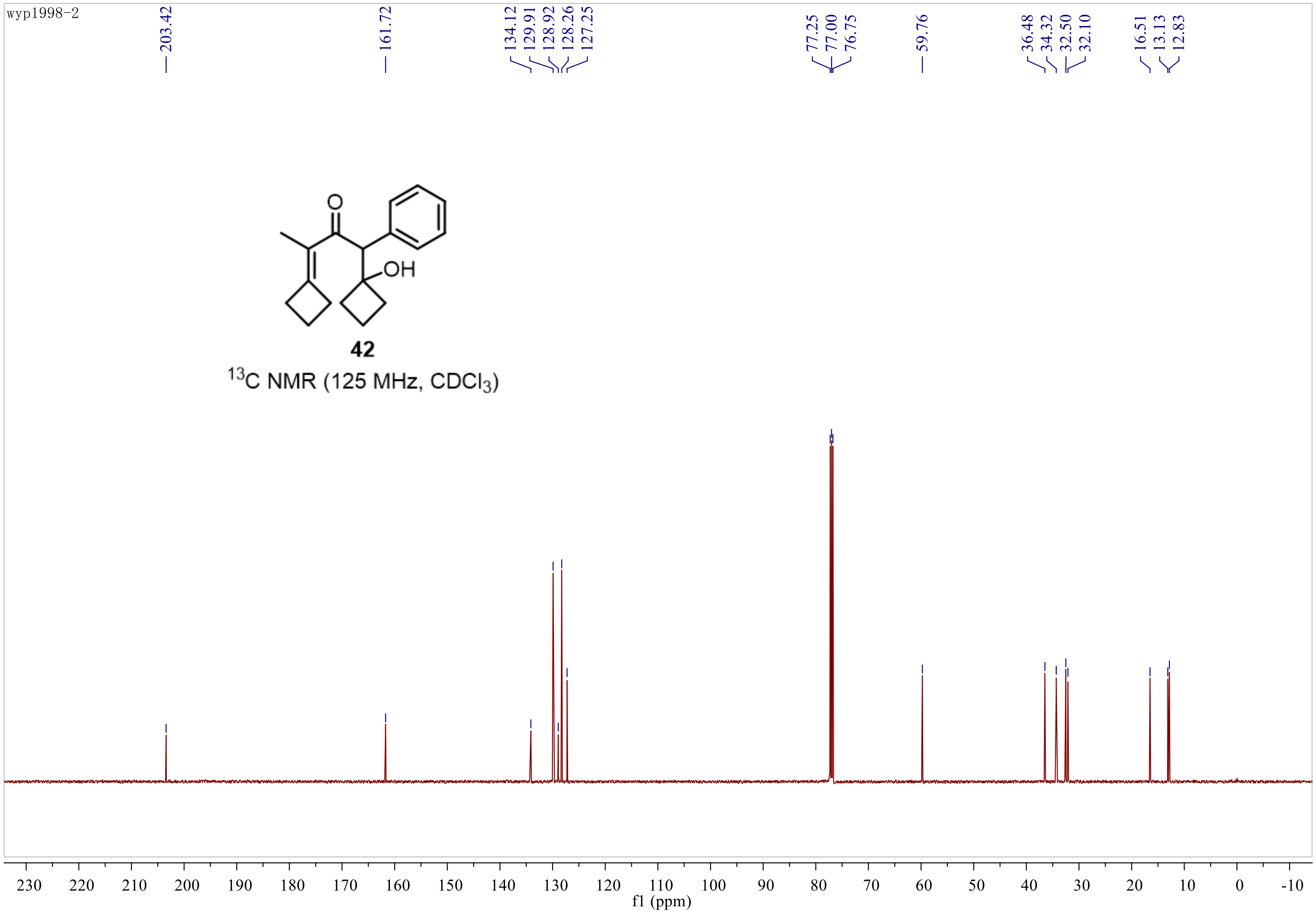

Supplementary Fig. 38. ^{13}C NMR spectra of compound **42** in $CDCl_3$

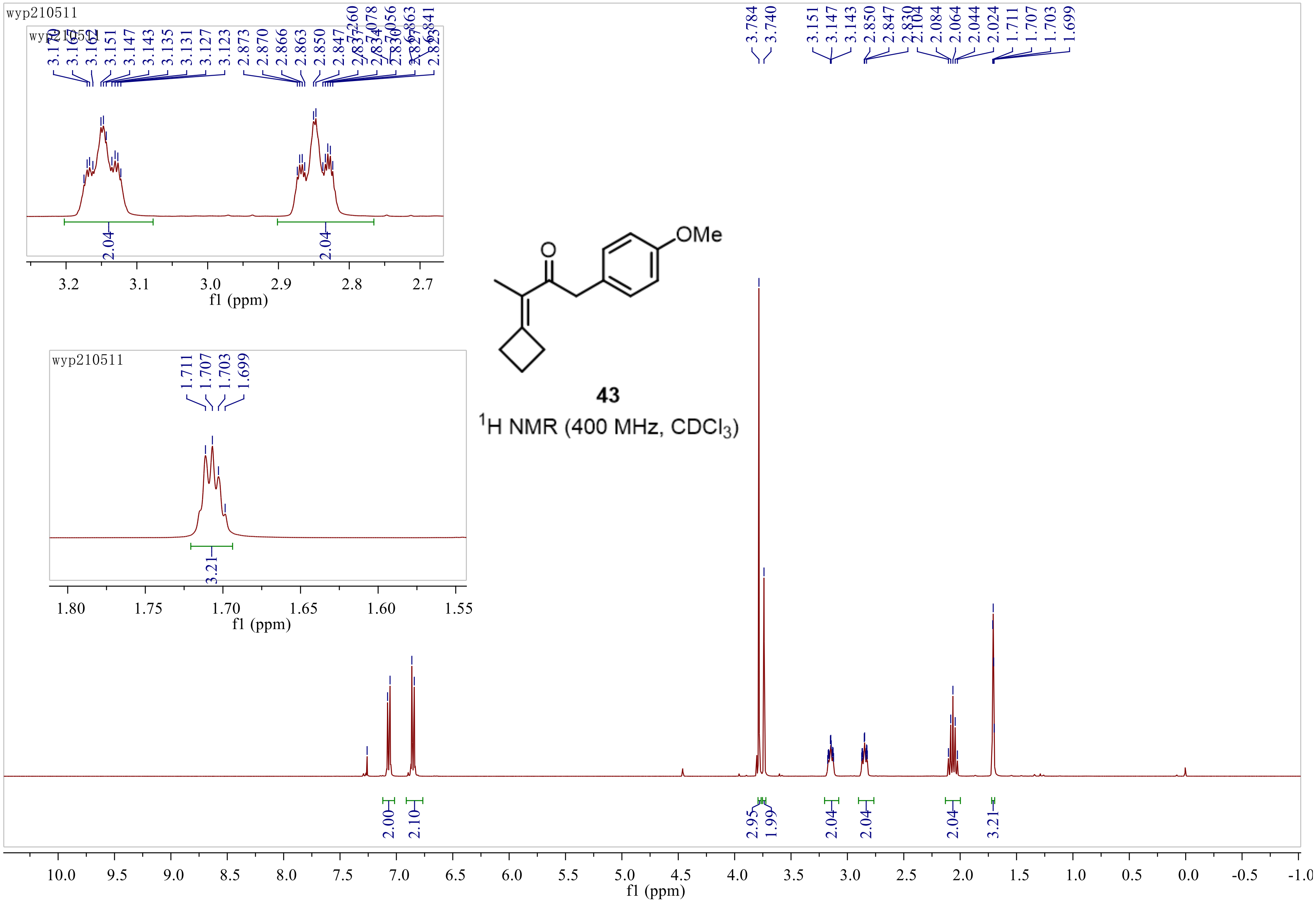

Supplementary Fig. 39. ^{1}H NMR spectra of compound **43** in $CDCl_3$

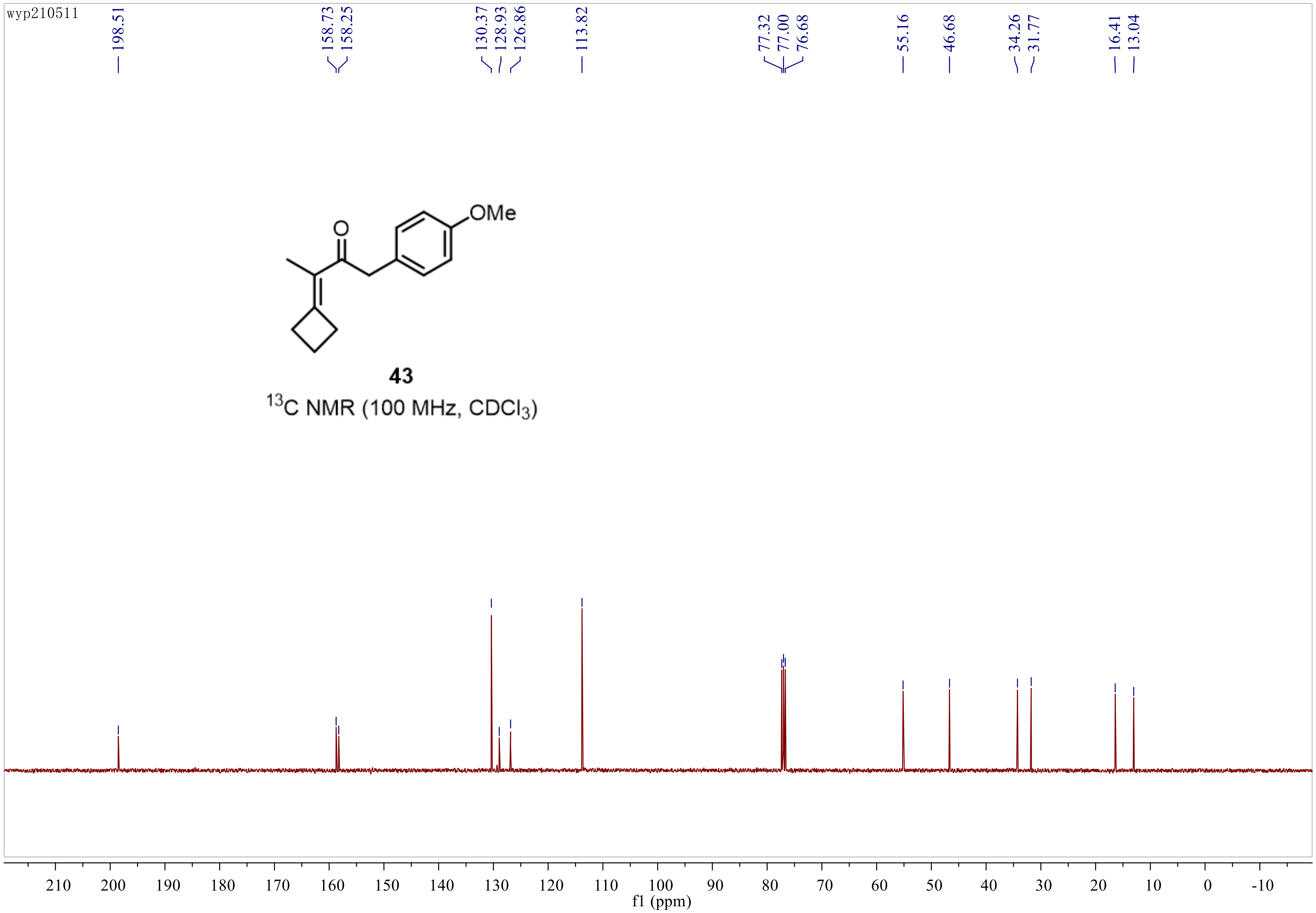

Supplementary Fig. 40. ^{13}C NMR spectra of compound **43** in $CDCl_3$

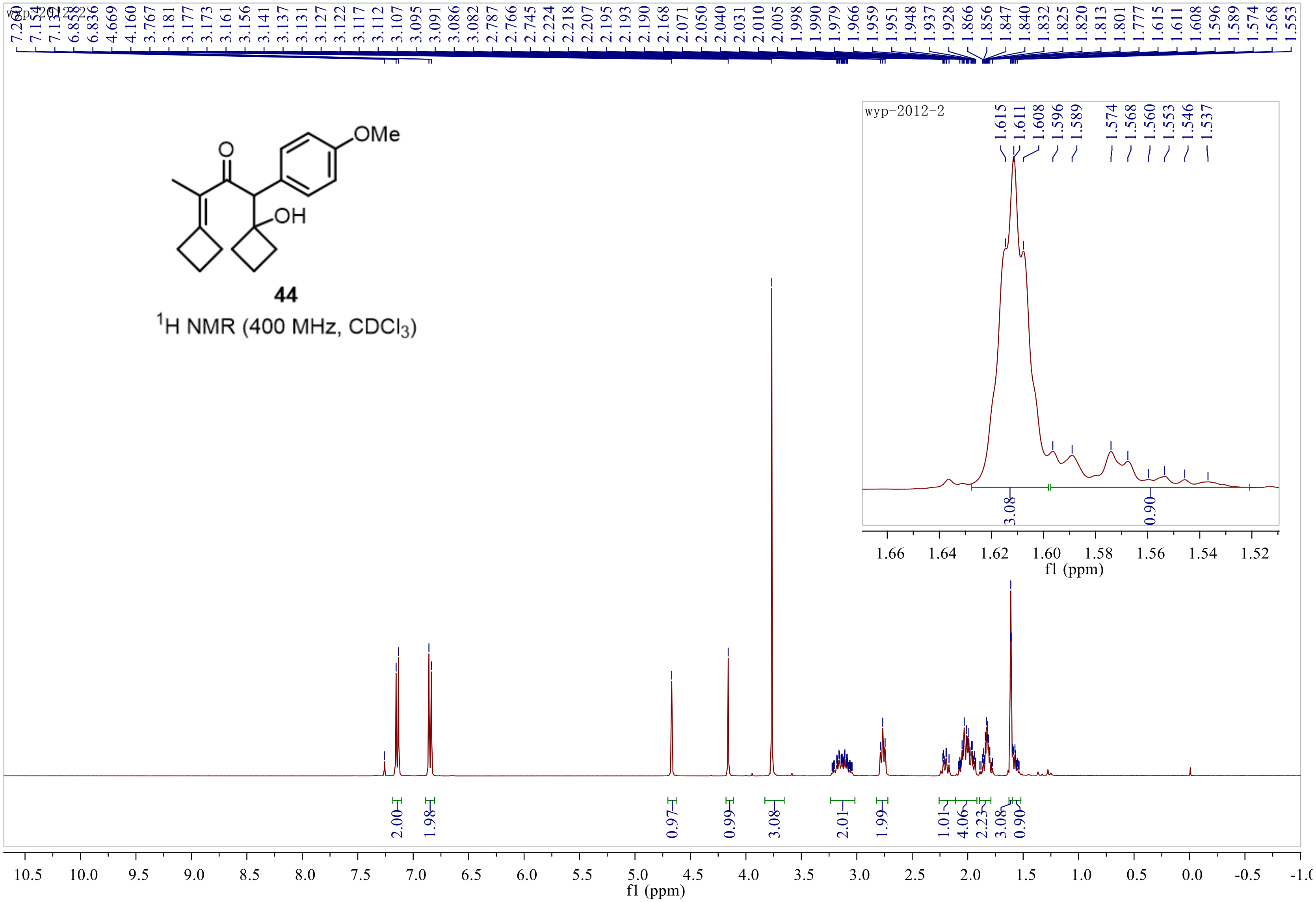

Supplementary Fig. 41. ^{1}H NMR spectra of compound **44** in $CDCl_3$

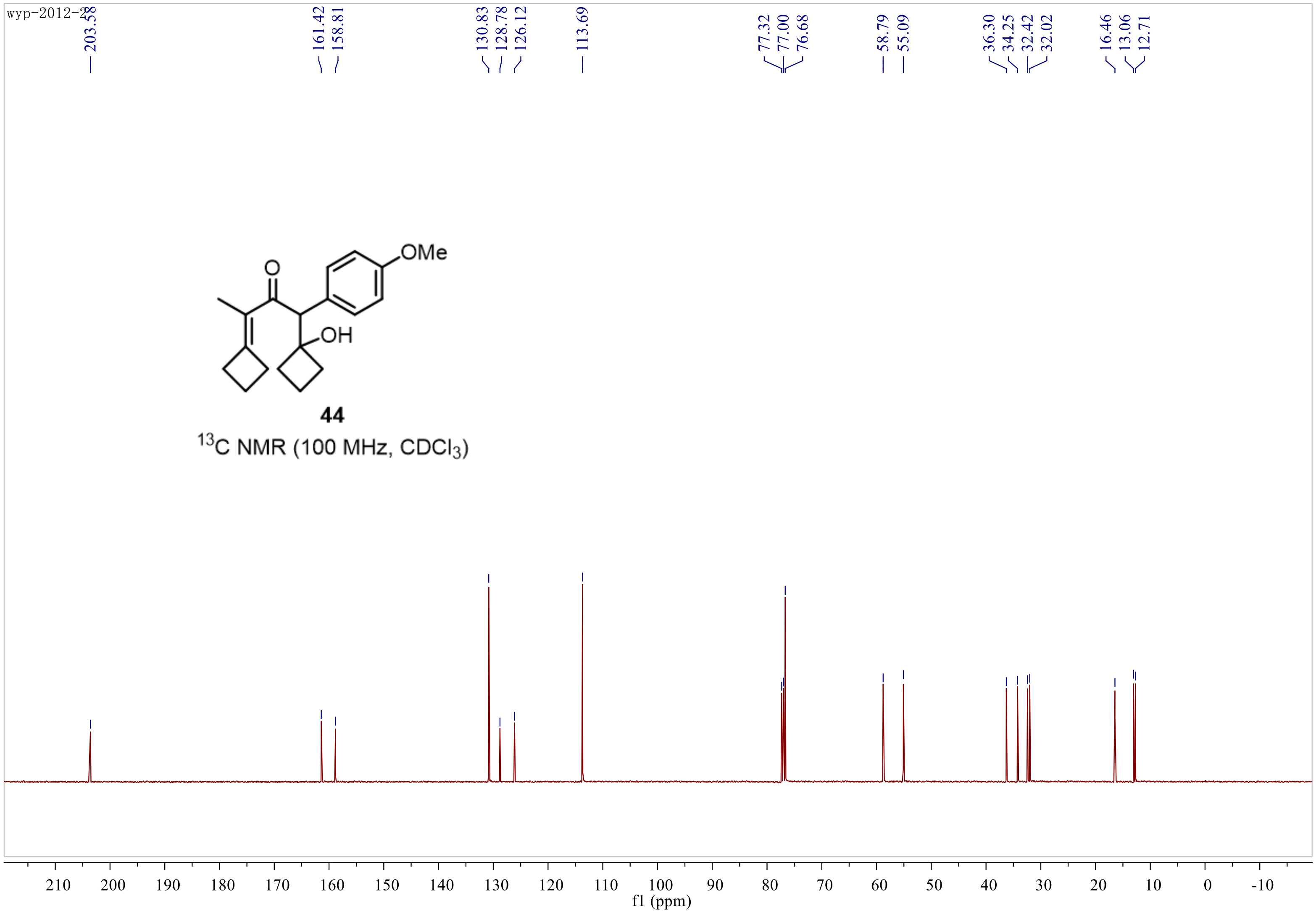

Supplementary Fig. 42. ^{13}C NMR spectra of compound **44** in $CDCl_3$

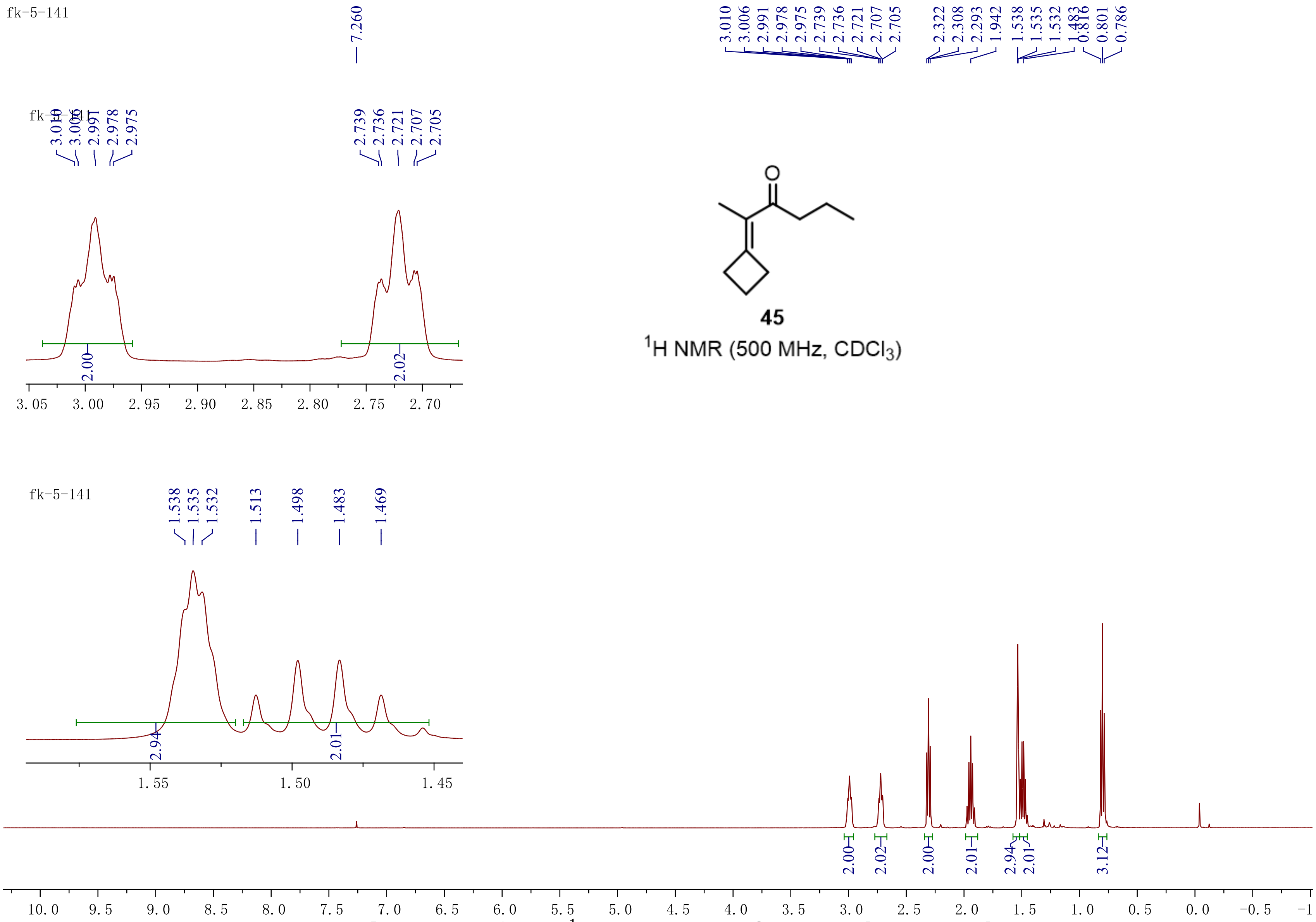

Supplementary Fig. 43. ^{1}H NMR spectra of compound **45** in $CDCl_3$

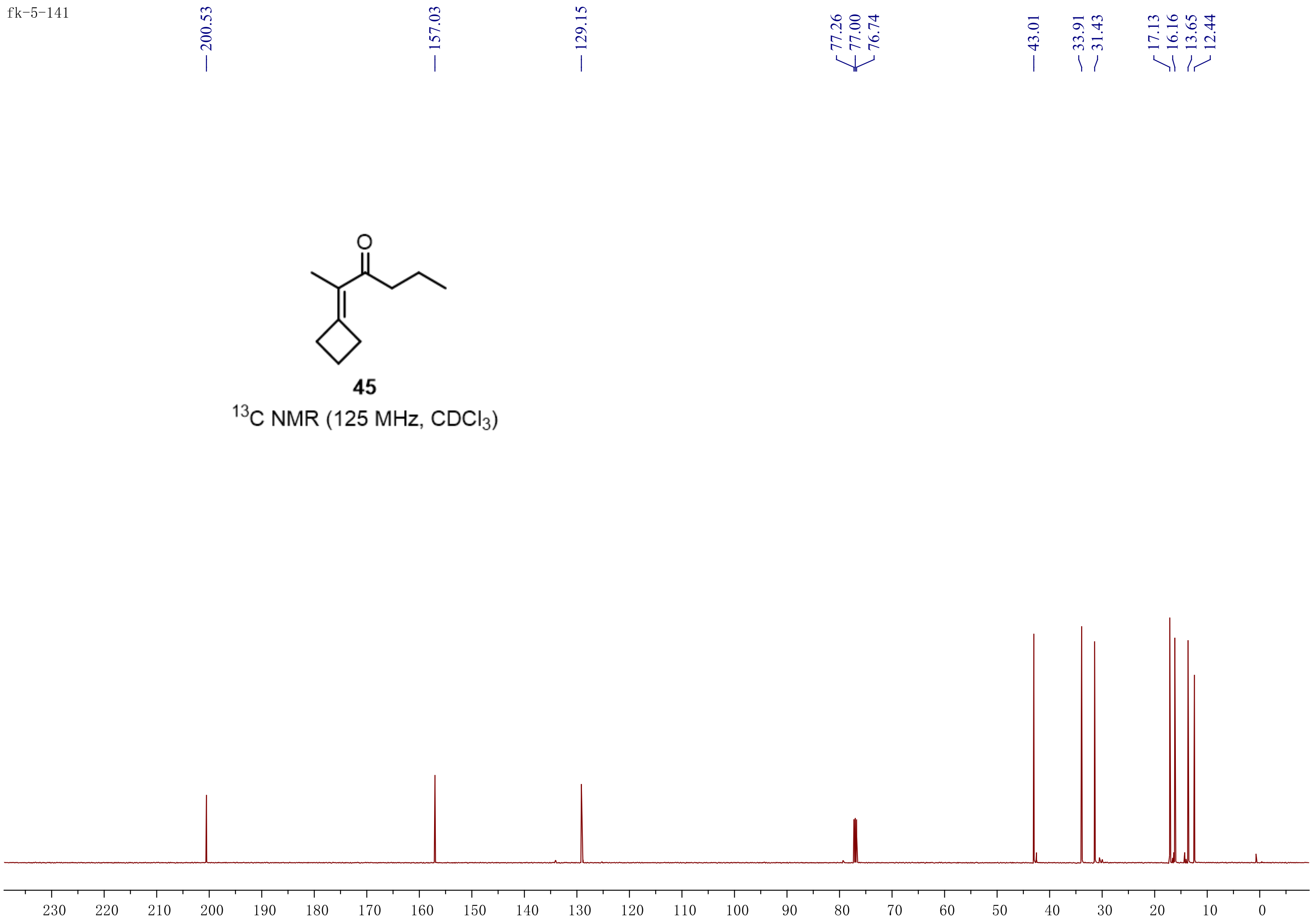

Supplementary Fig. 44. ^{13}C NMR spectra of compound **45** in $CDCl_3$

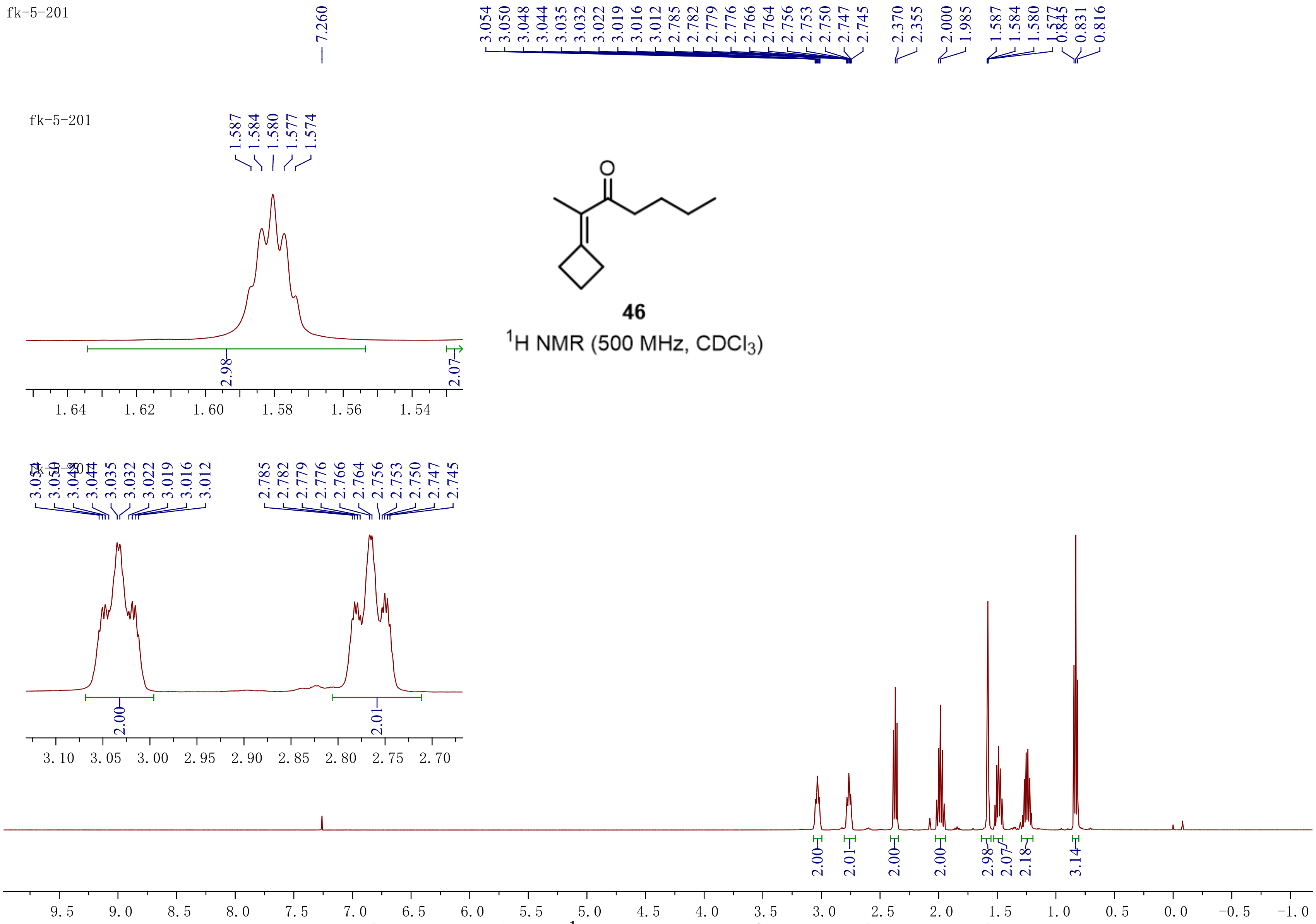

Supplementary Fig. 45. ^{1}H NMR spectra of compound **46** in $CDCl_3$

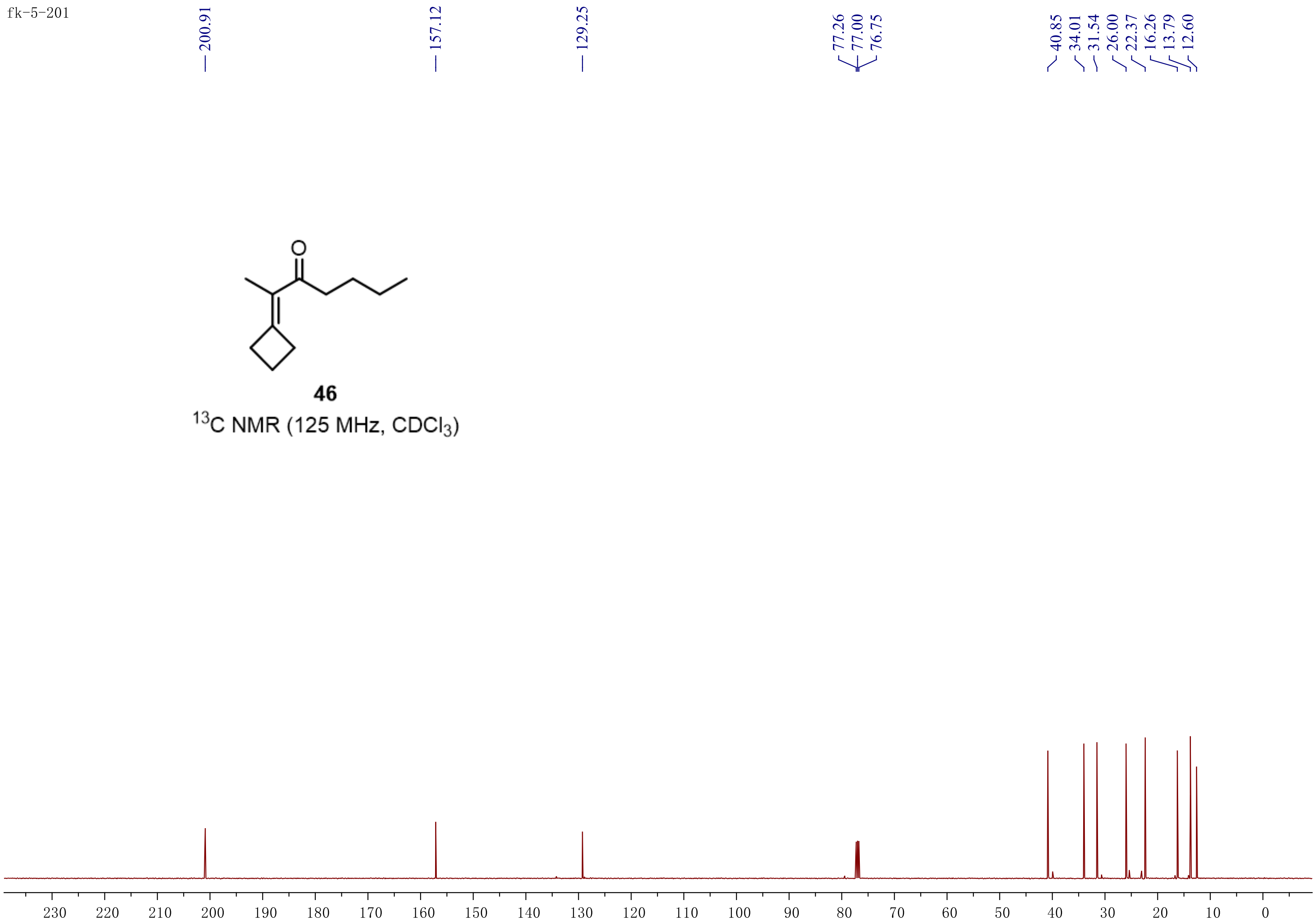

Supplementary Fig. 46. ^{13}C NMR spectra of compound **46** in $CDCl_3$

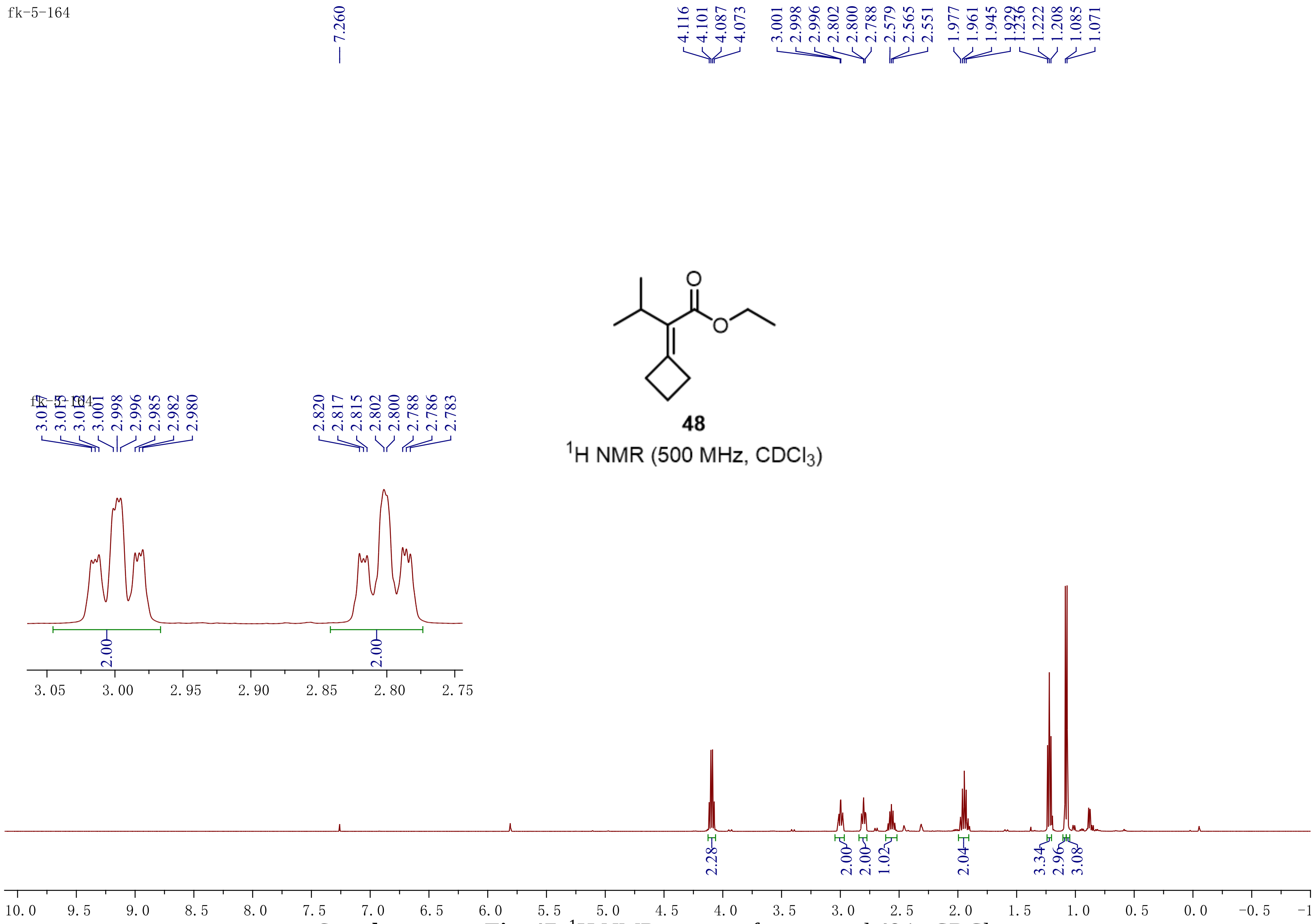

Supplementary Fig. 47. ^{1}H NMR spectra of compound **48** in $CDCl_3$

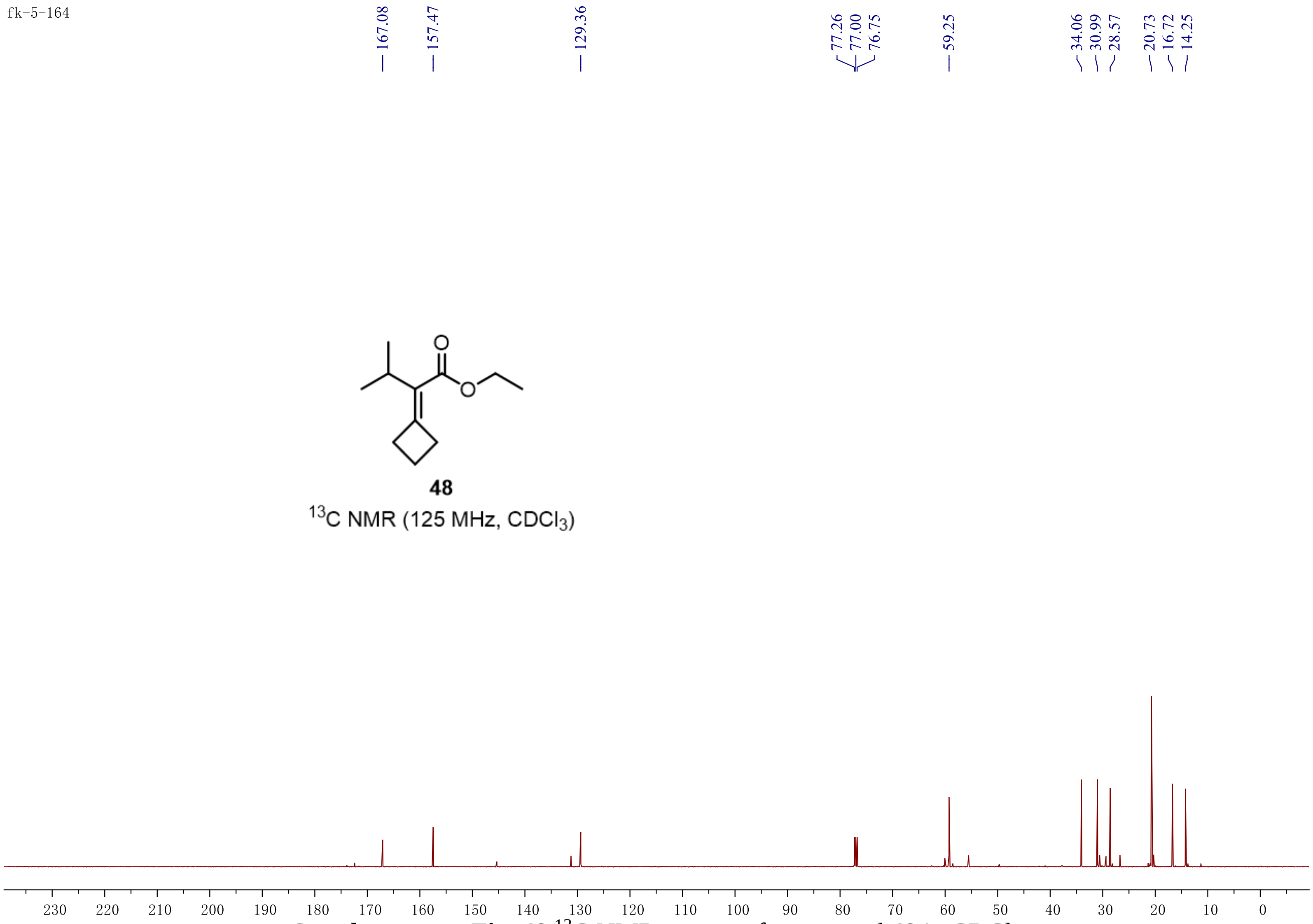

Supplementary Fig. 48. ^{13}C NMR spectra of compound **48** in $CDCl_3$

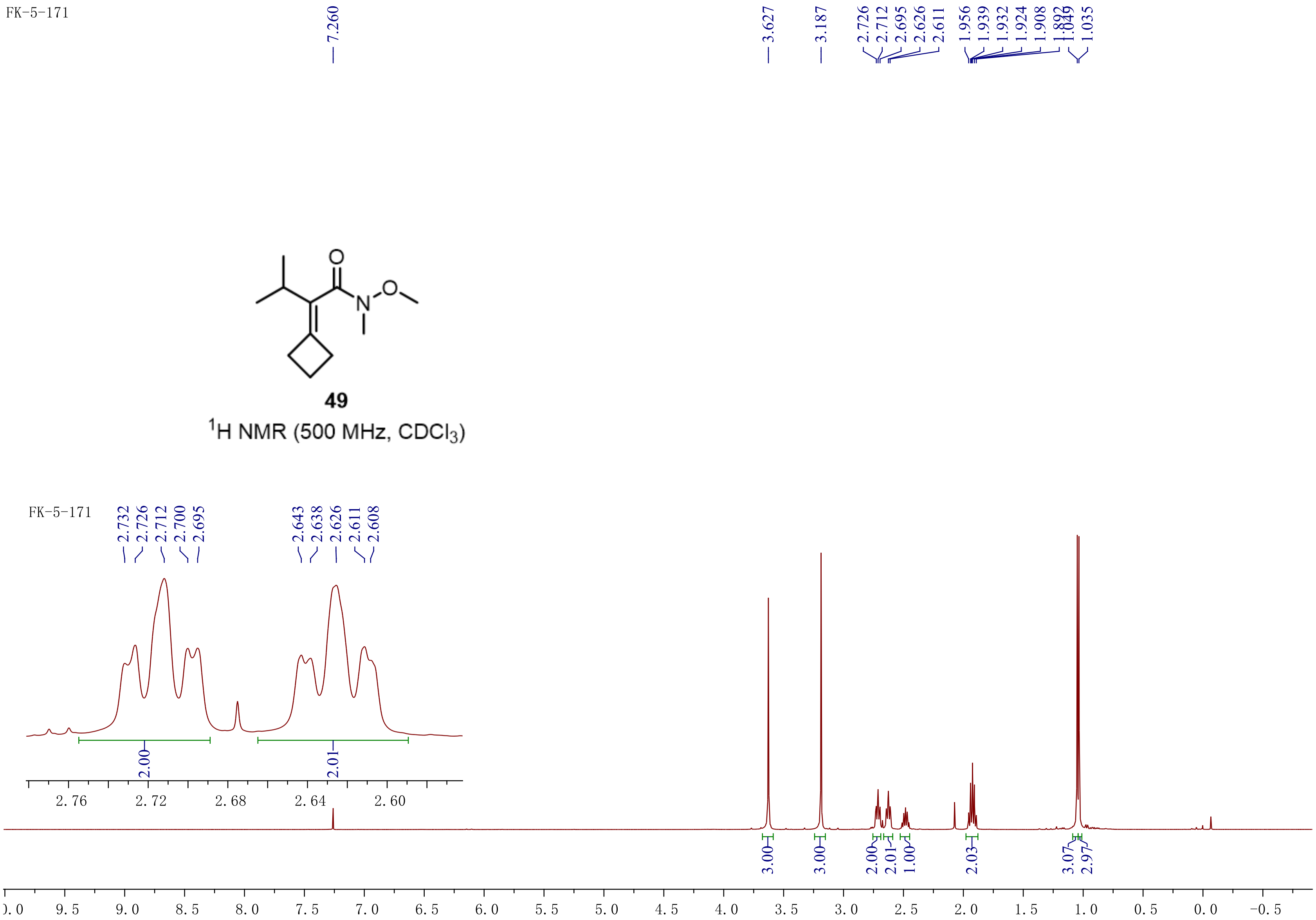

Supplementary Fig. 49. ^{1}H NMR spectra of compound **49** in $CDCl_3$

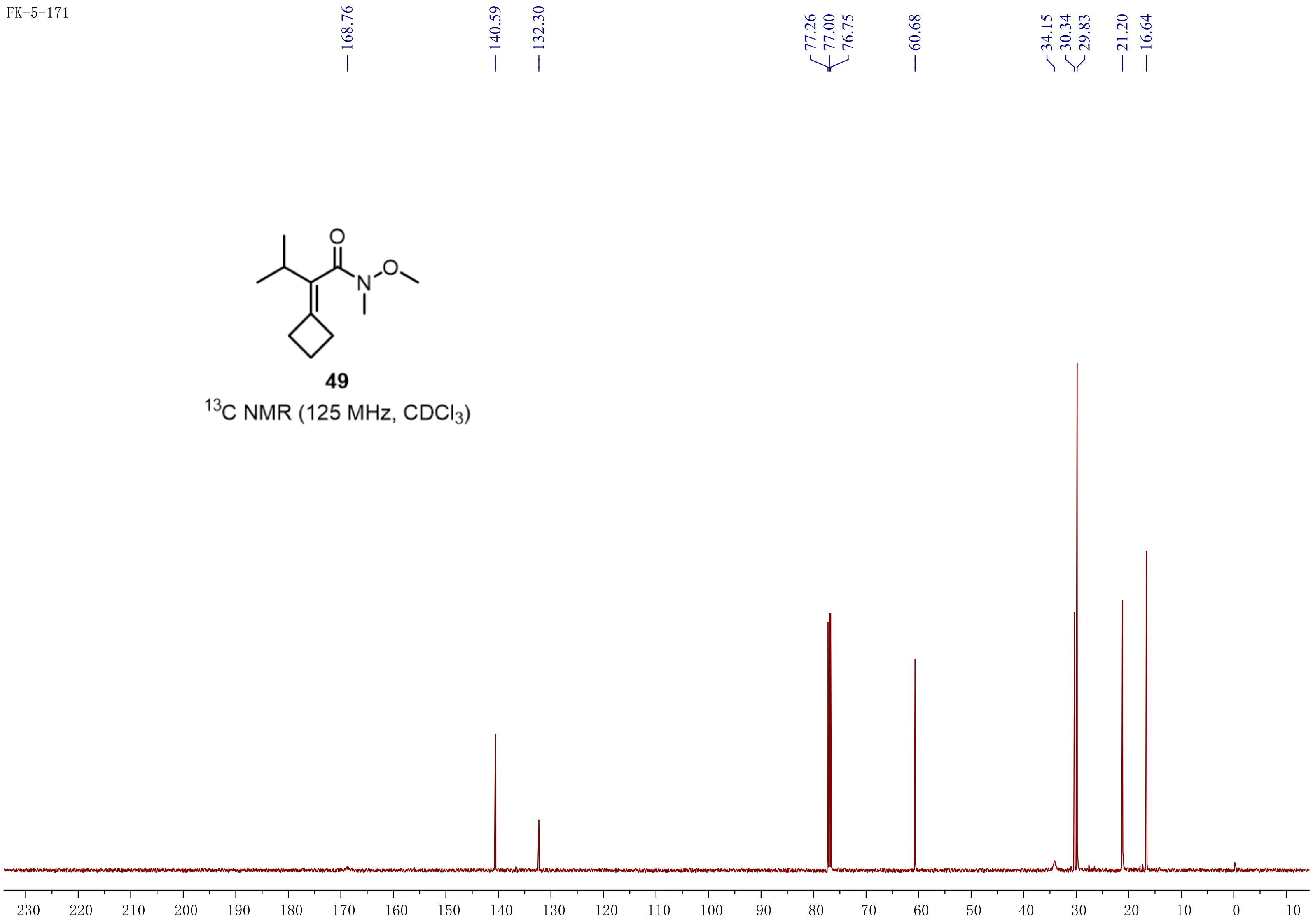

Supplementary Fig. 50. ^{13}C NMR spectra of compound **49** in $CDCl_3$

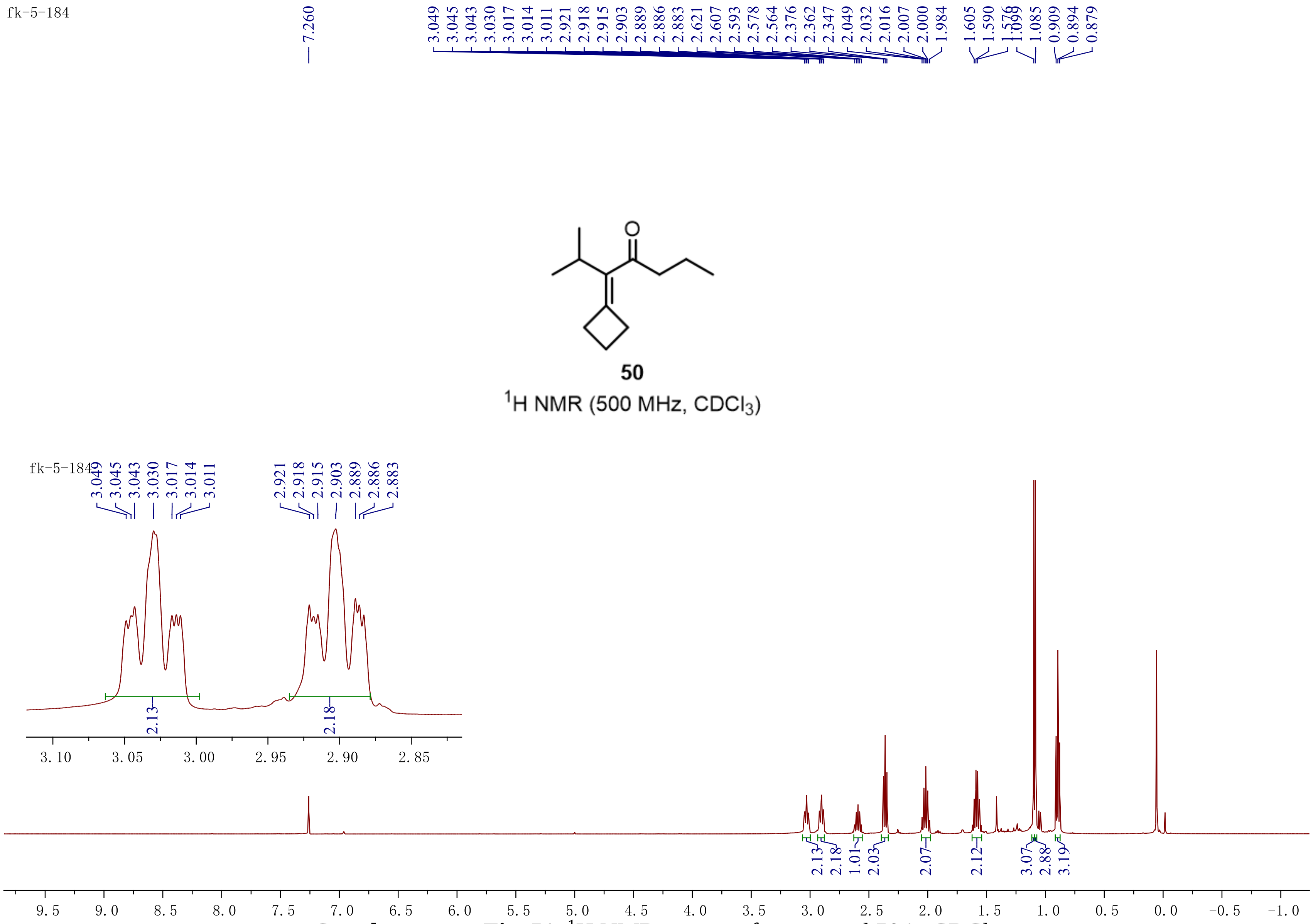

Supplementary Fig. 51. ^{1}H NMR spectra of compound **50** in $CDCl_3$

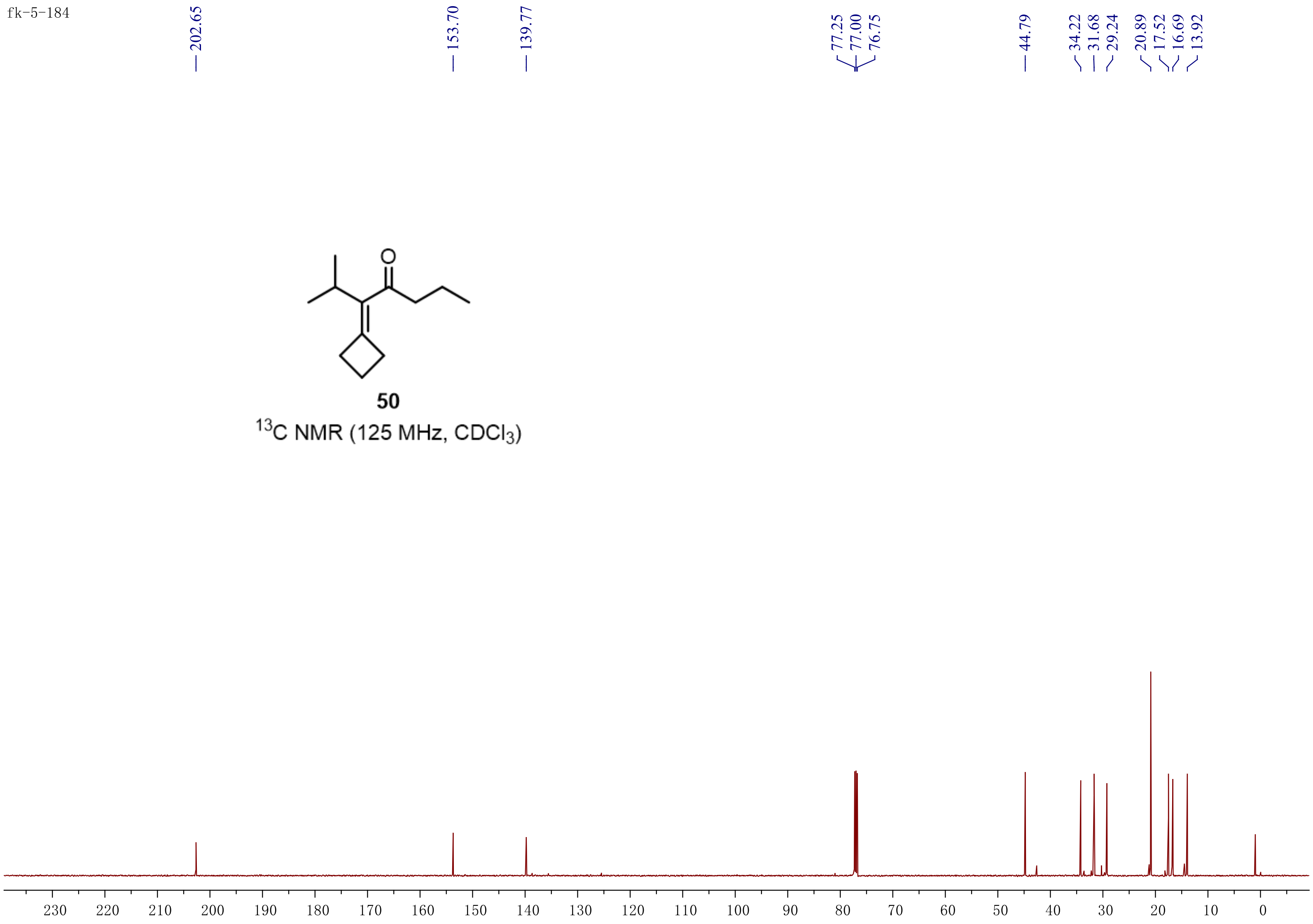

Supplementary Fig. 52. ^{13}C NMR spectra of compound **50** in $CDCl_3$

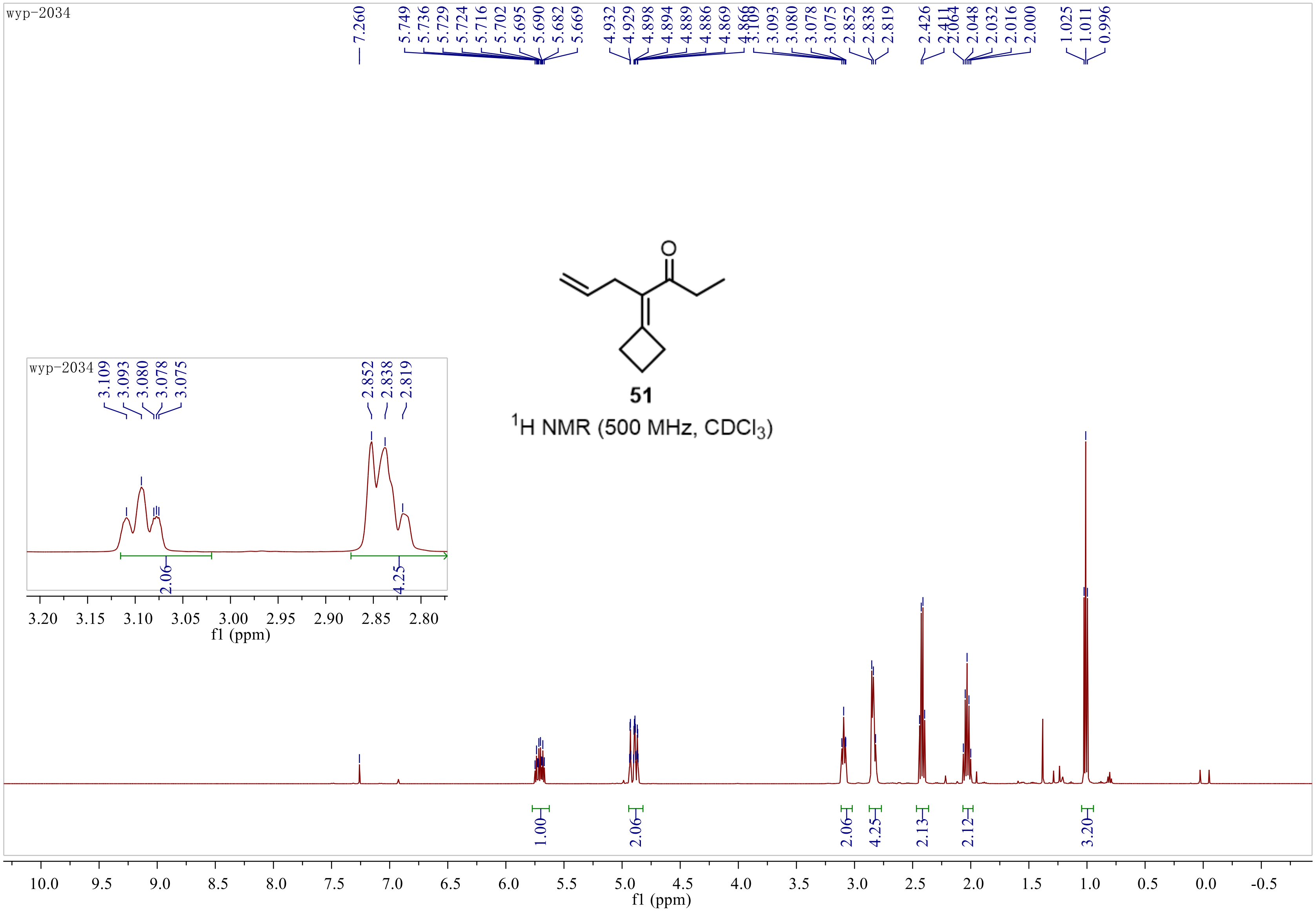

Supplementary Fig. 53. ^{1}H NMR spectra of compound **51** in $CDCl_3$

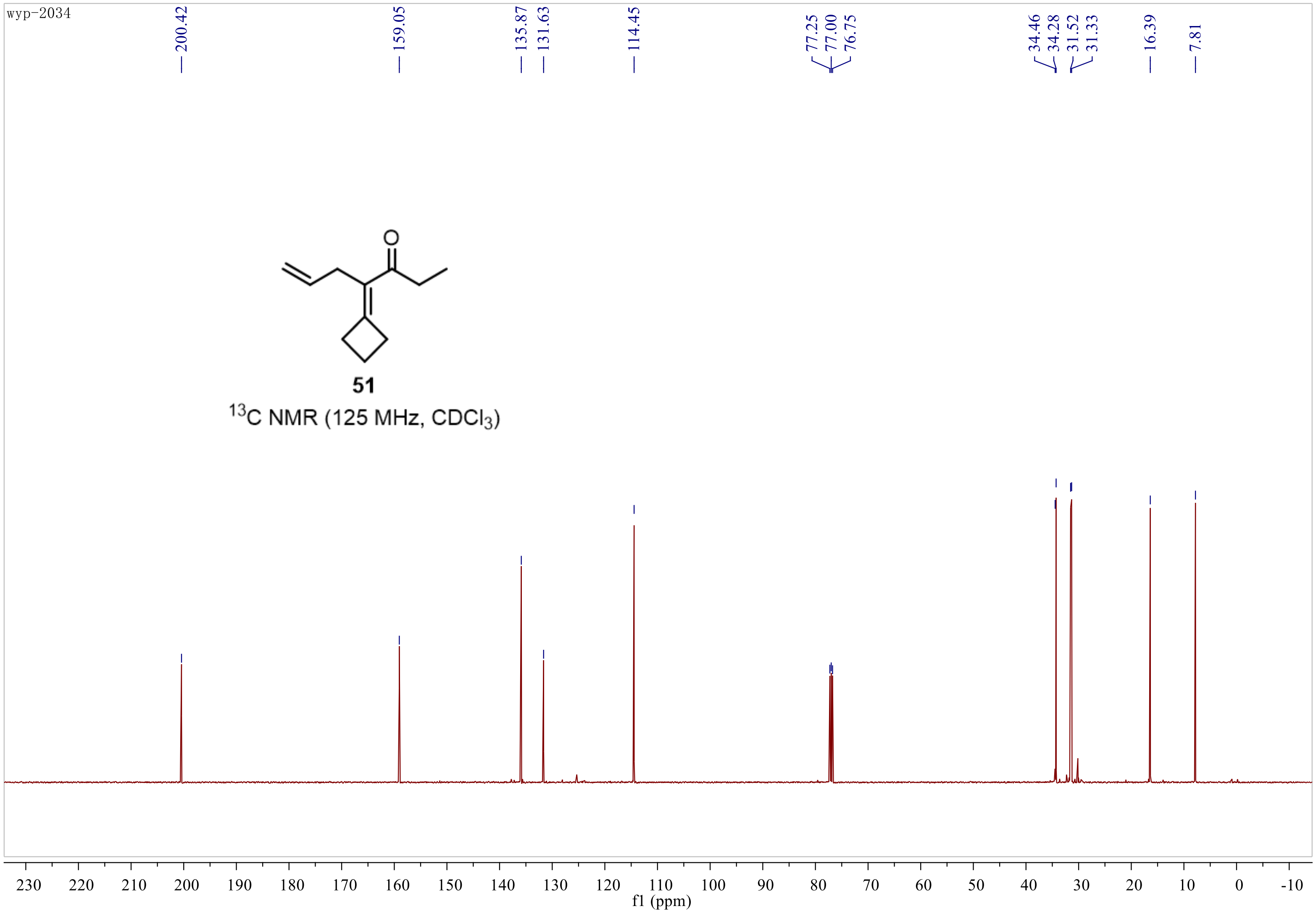

Supplementary Fig. 54.^{13}C NMR spectra of compound **51** in $CDCl_3$

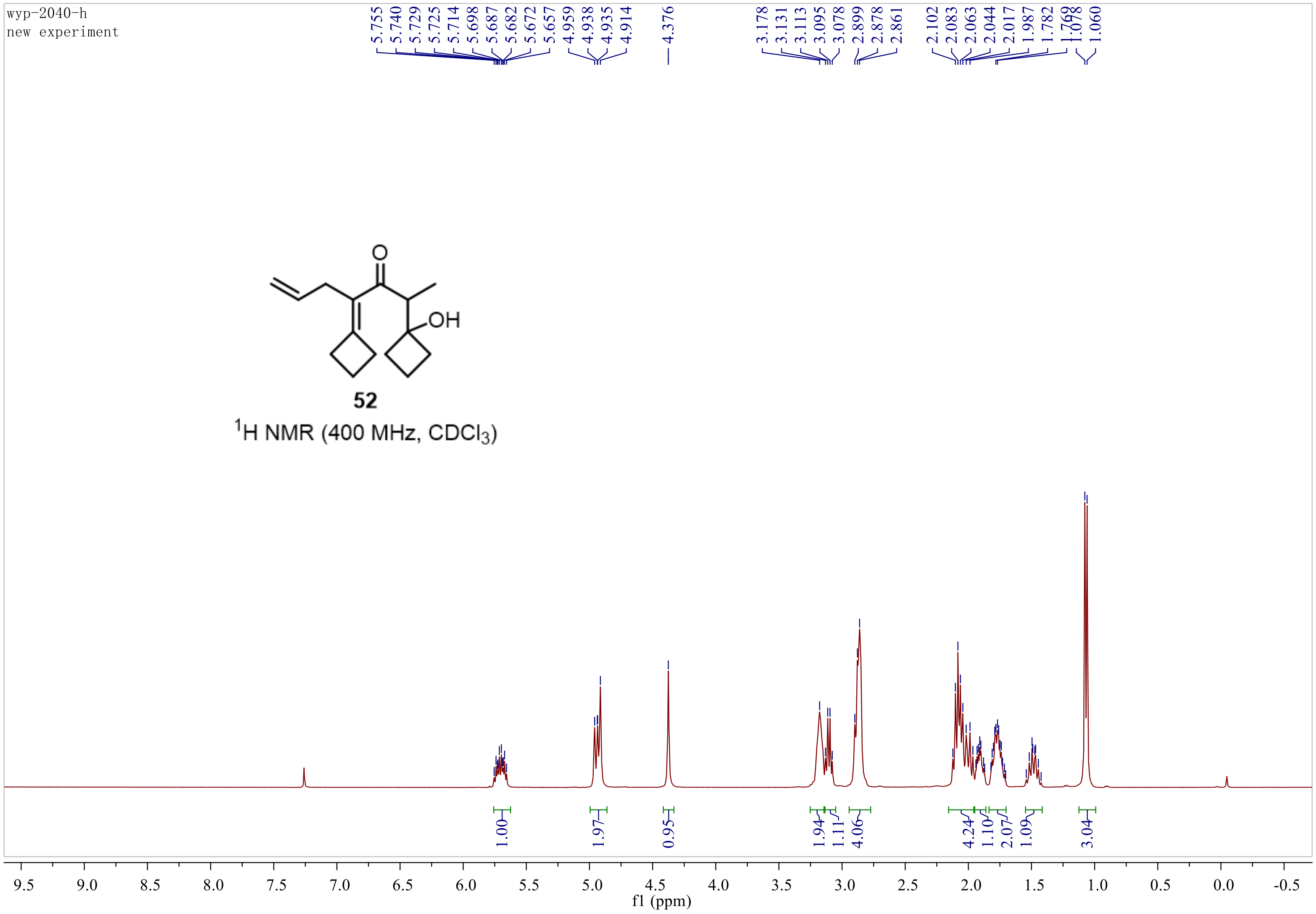

Supplementary Fig. 55. ^{1}H NMR spectra of compound **52** in $CDCl_3$

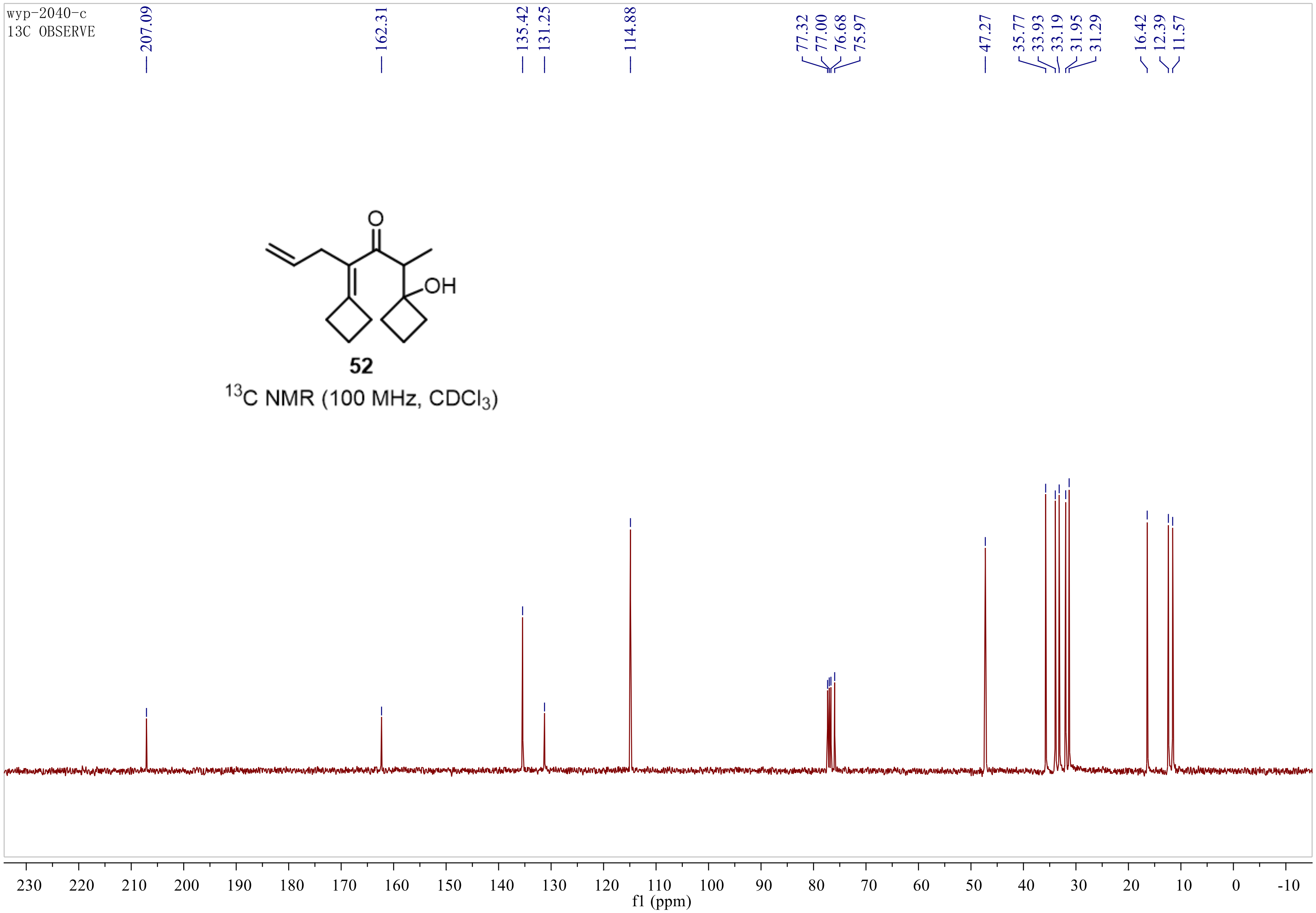

Supplementary Fig. 56. ^{13}C NMR spectra of compound **52** in $CDCl_3$

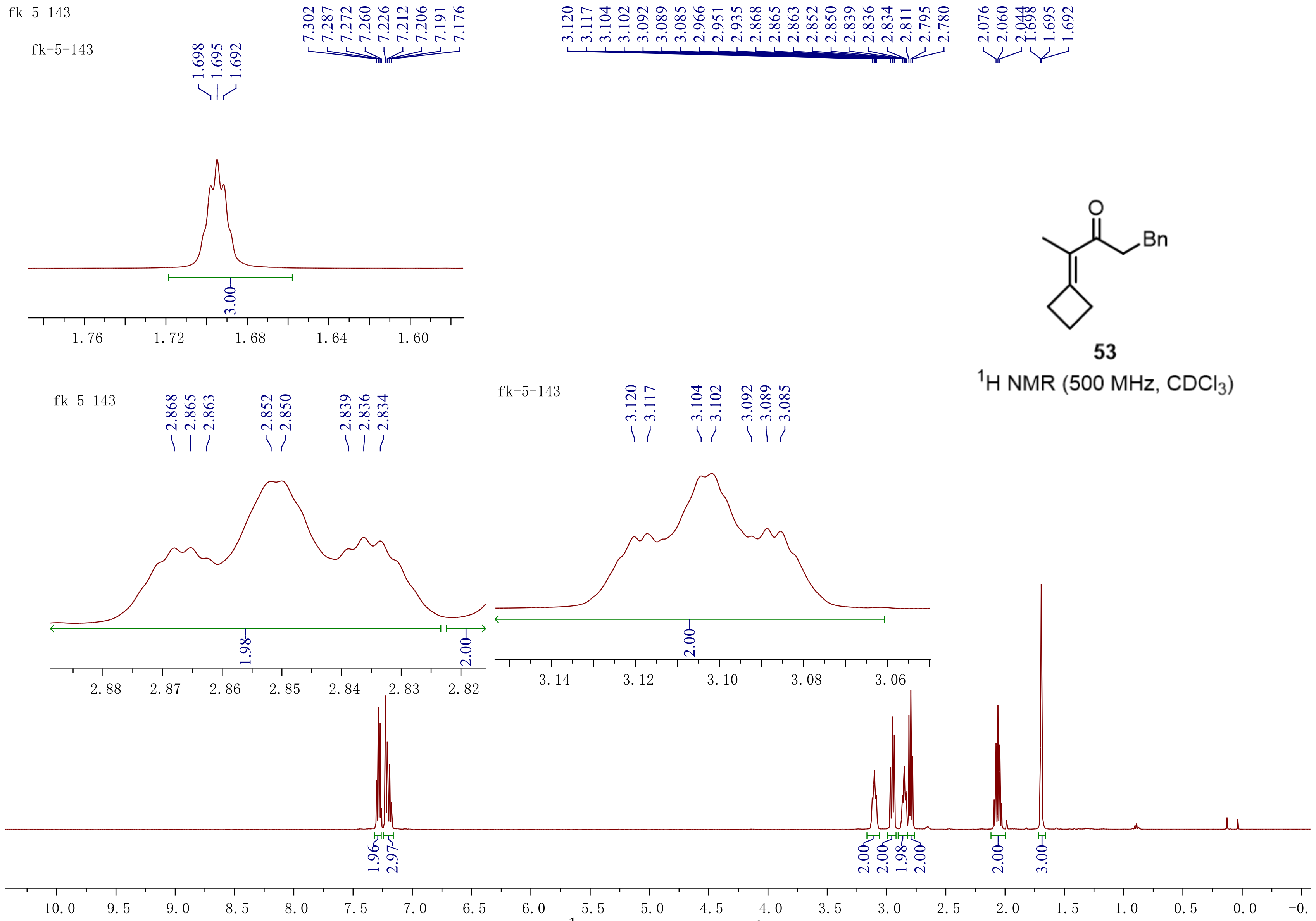

Supplementary Fig. 57. ^{1}H NMR spectra of compound **53** in $CDCl_3$

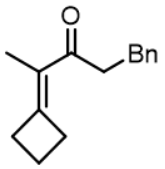

53

^{13}C NMR (125 MHz, $CDCl_3$)

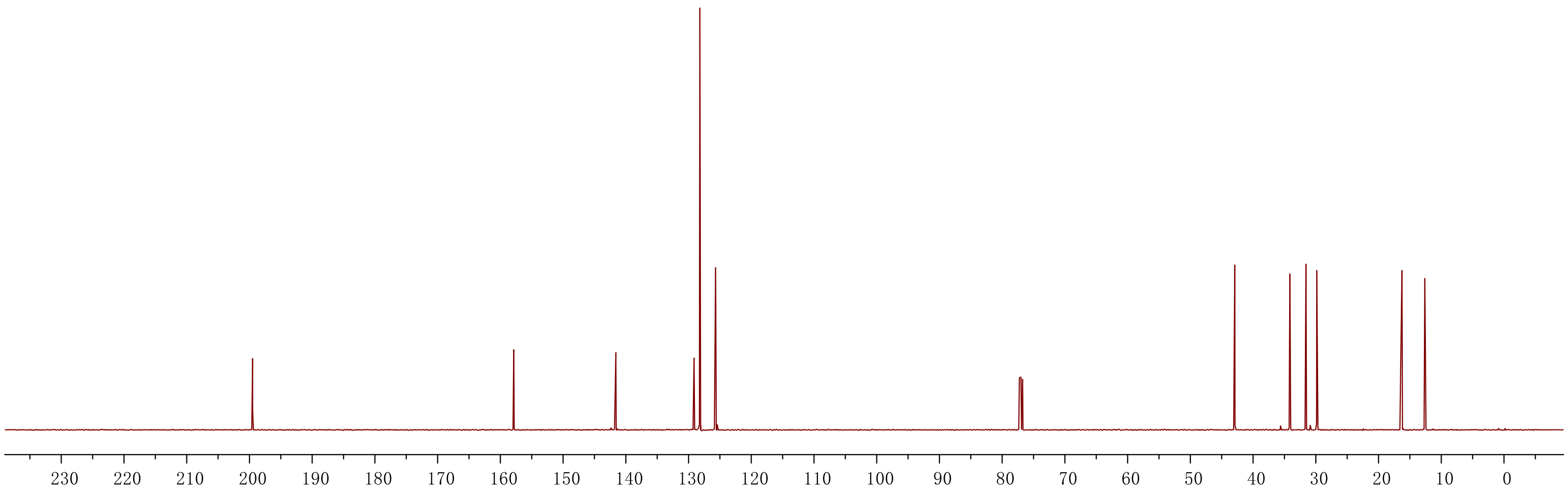

Supplementary Fig. 58. ^{13}C NMR spectra of compound **53** in $CDCl_3$

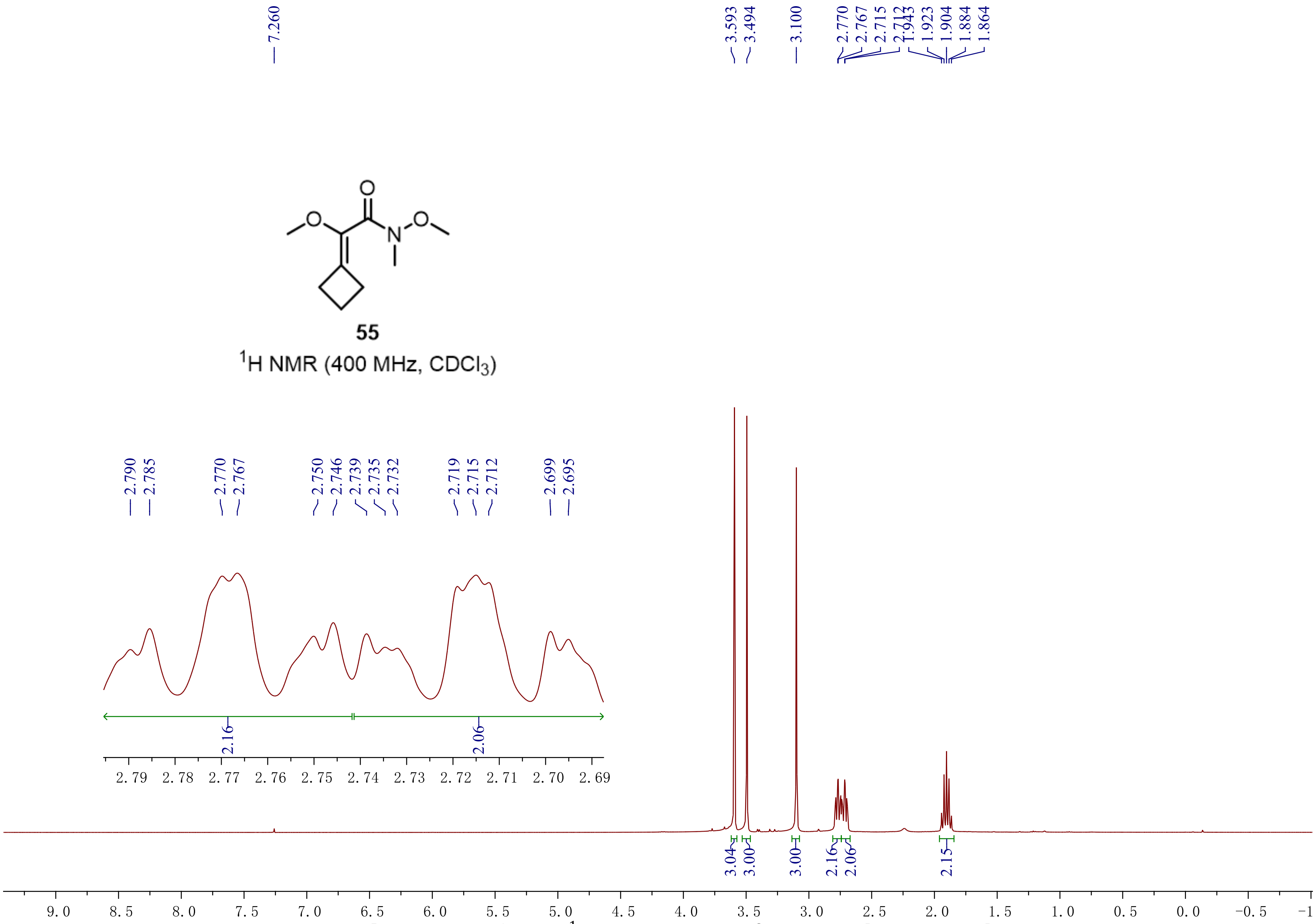

Supplementary Fig. 59. ^{1}H NMR spectra of compound **55** in $CDCl_3$

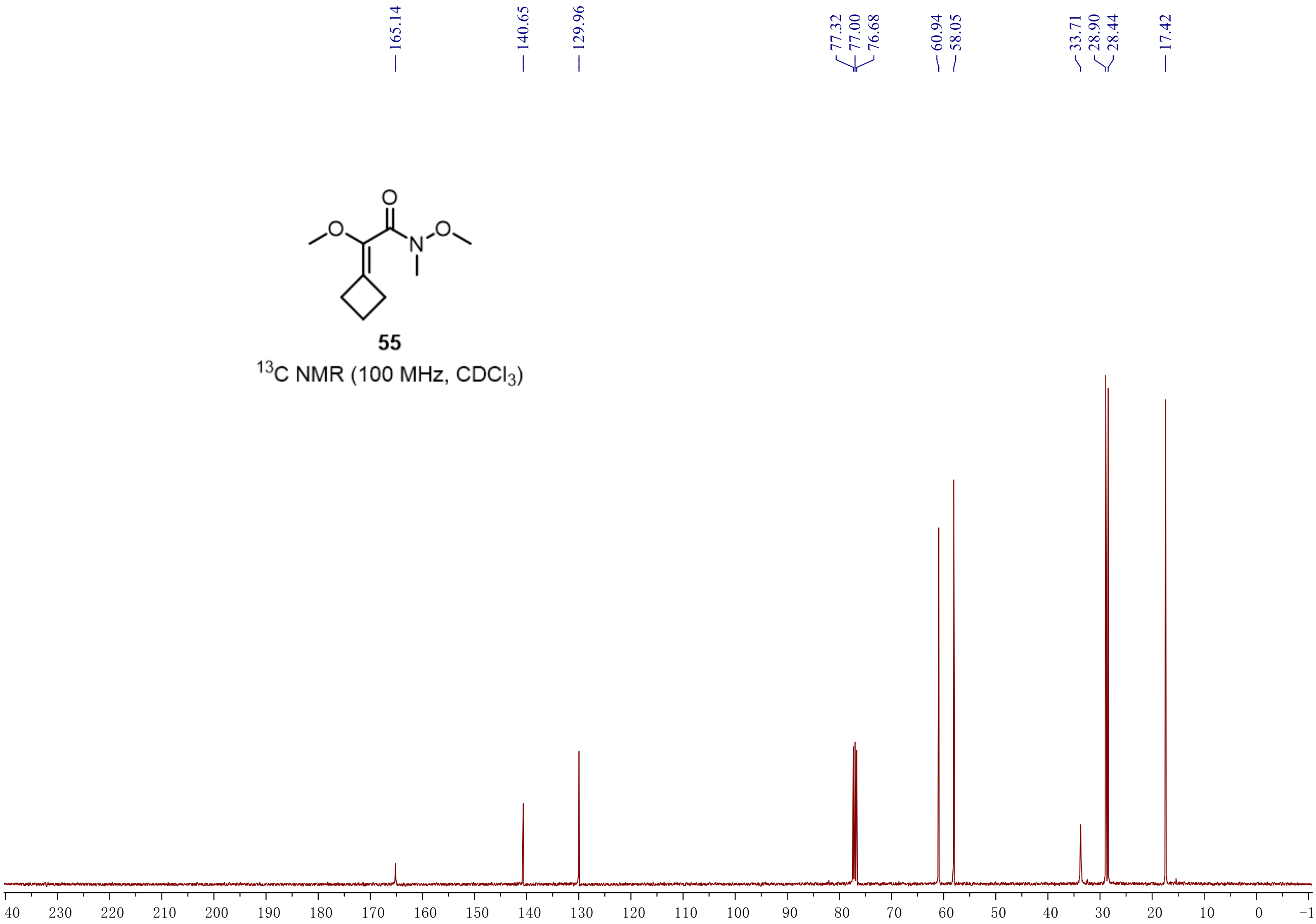

Supplementary Fig. 60. ^{13}C NMR spectra of compound **55** in $CDCl_3$

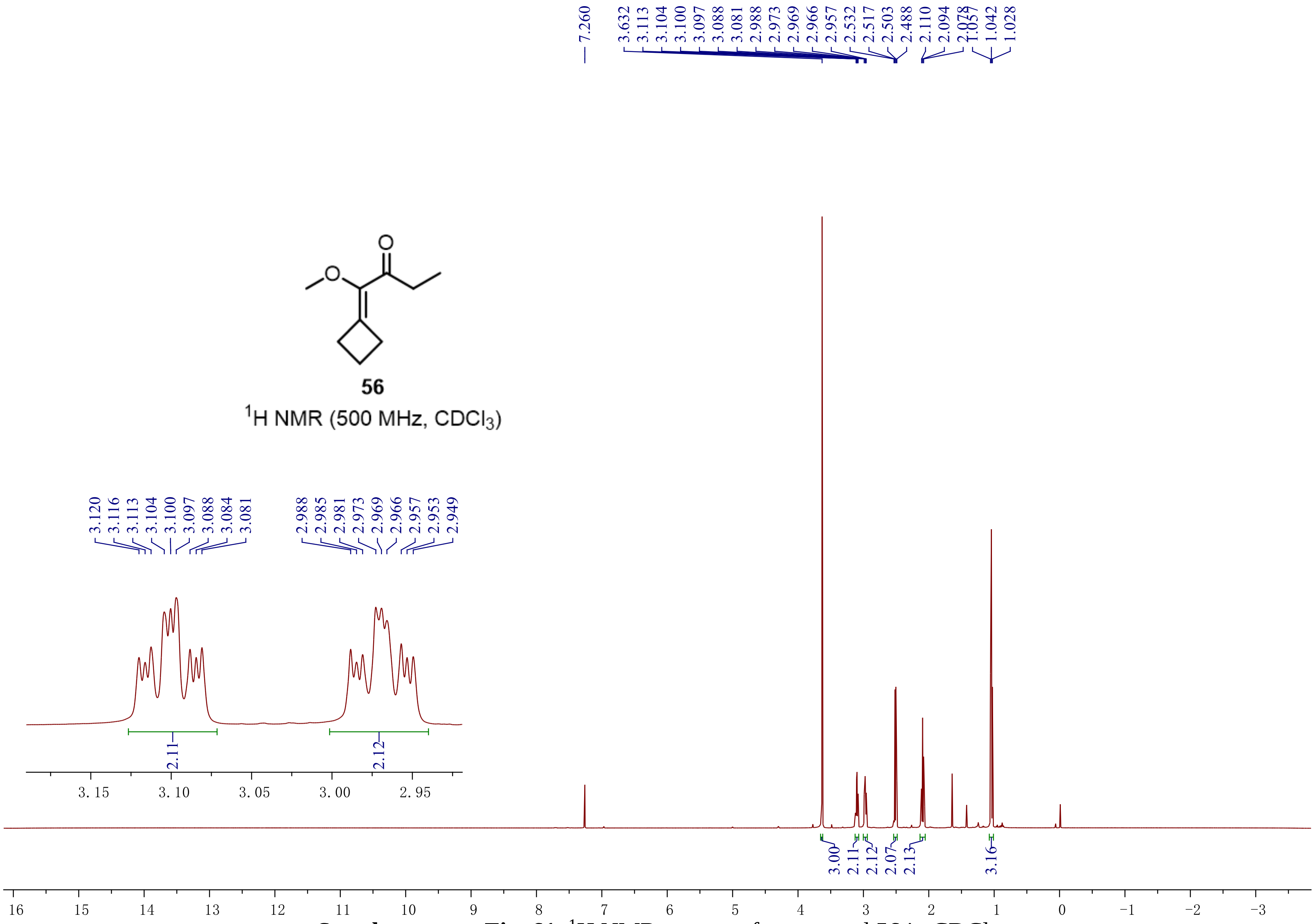

Supplementary Fig. 61. ^{1}H NMR spectra of compound **56** in $CDCl_3$

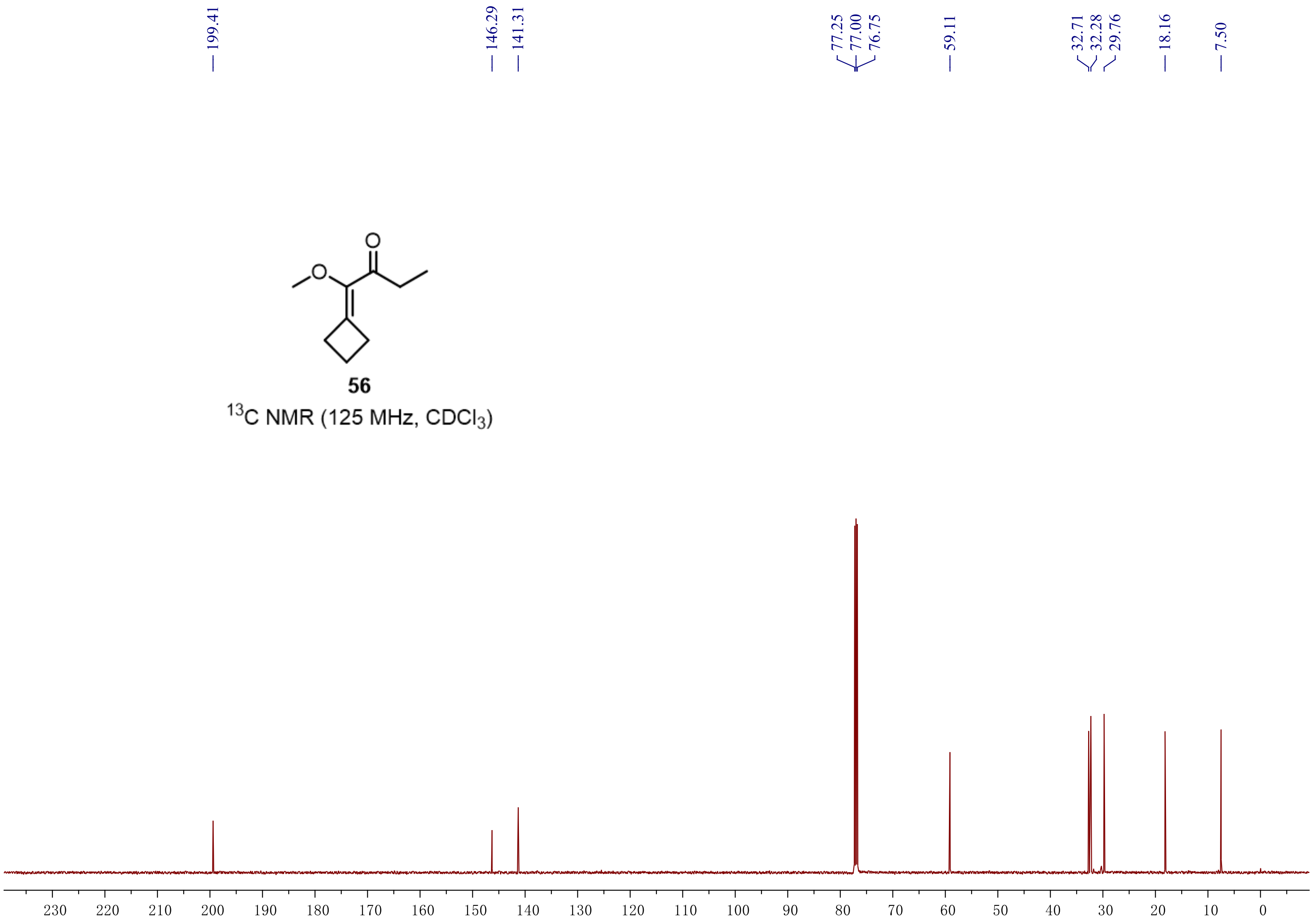

Supplementary Fig. 62. ^{13}C NMR spectra of compound **56** in $CDCl_3$

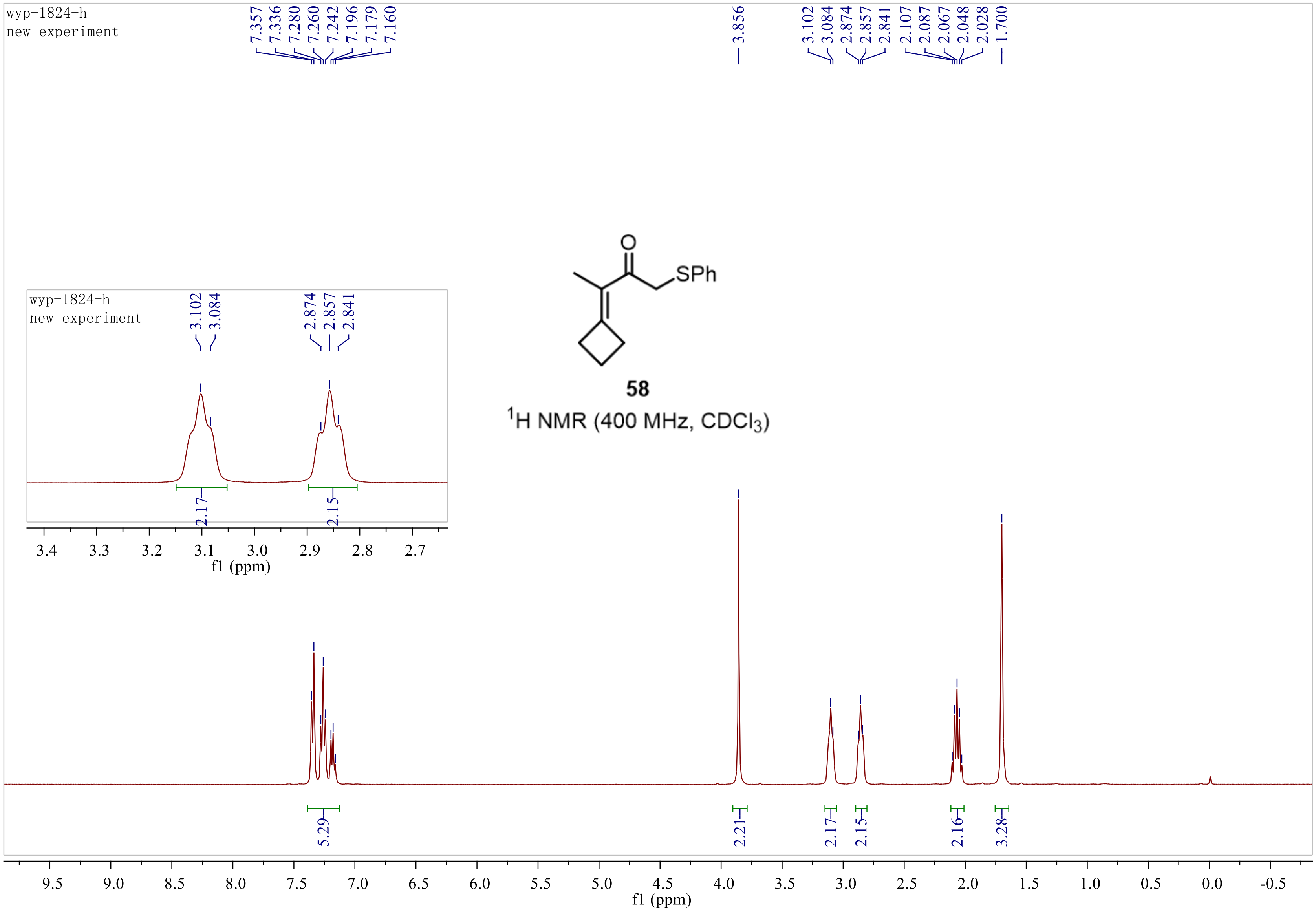

Supplementary Fig. 63. ^{1}H NMR spectra of compound **58** in $CDCl_3$

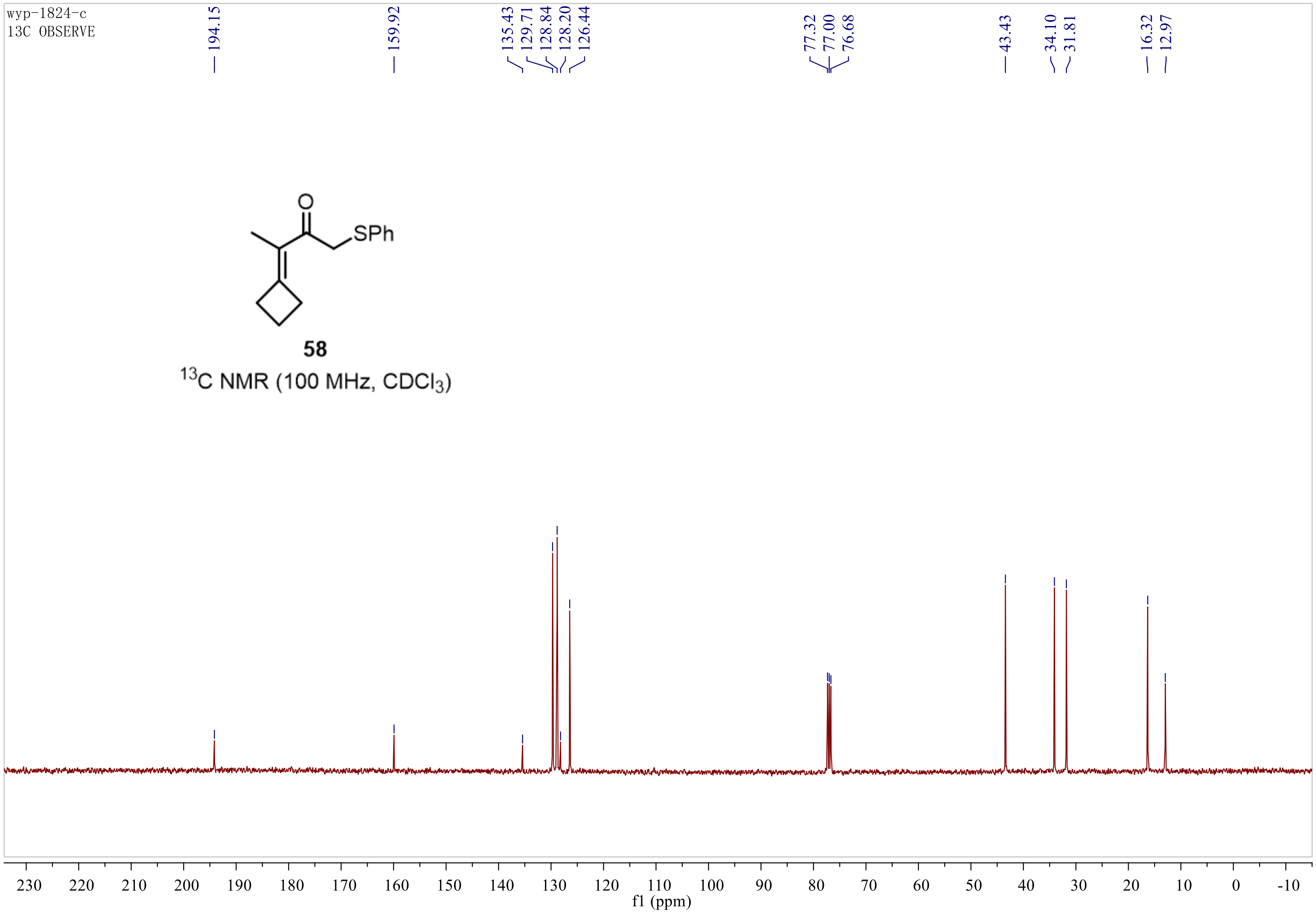

Supplementary Fig. 64. ^{13}C NMR spectra of compound **58** in $CDCl_3$

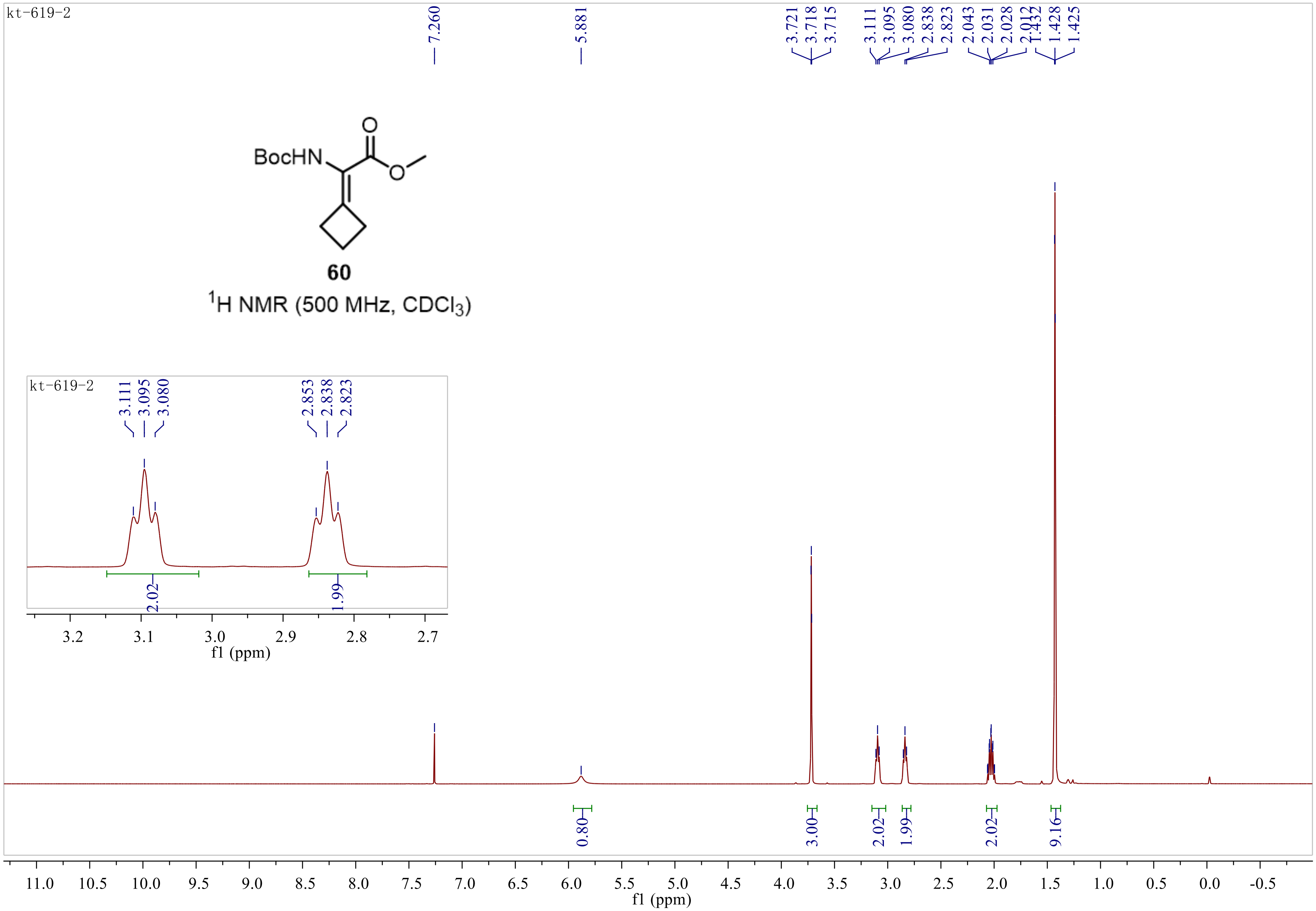

Supplementary Fig. 65. ^{1}H NMR spectra of compound **60** in $CDCl_3$

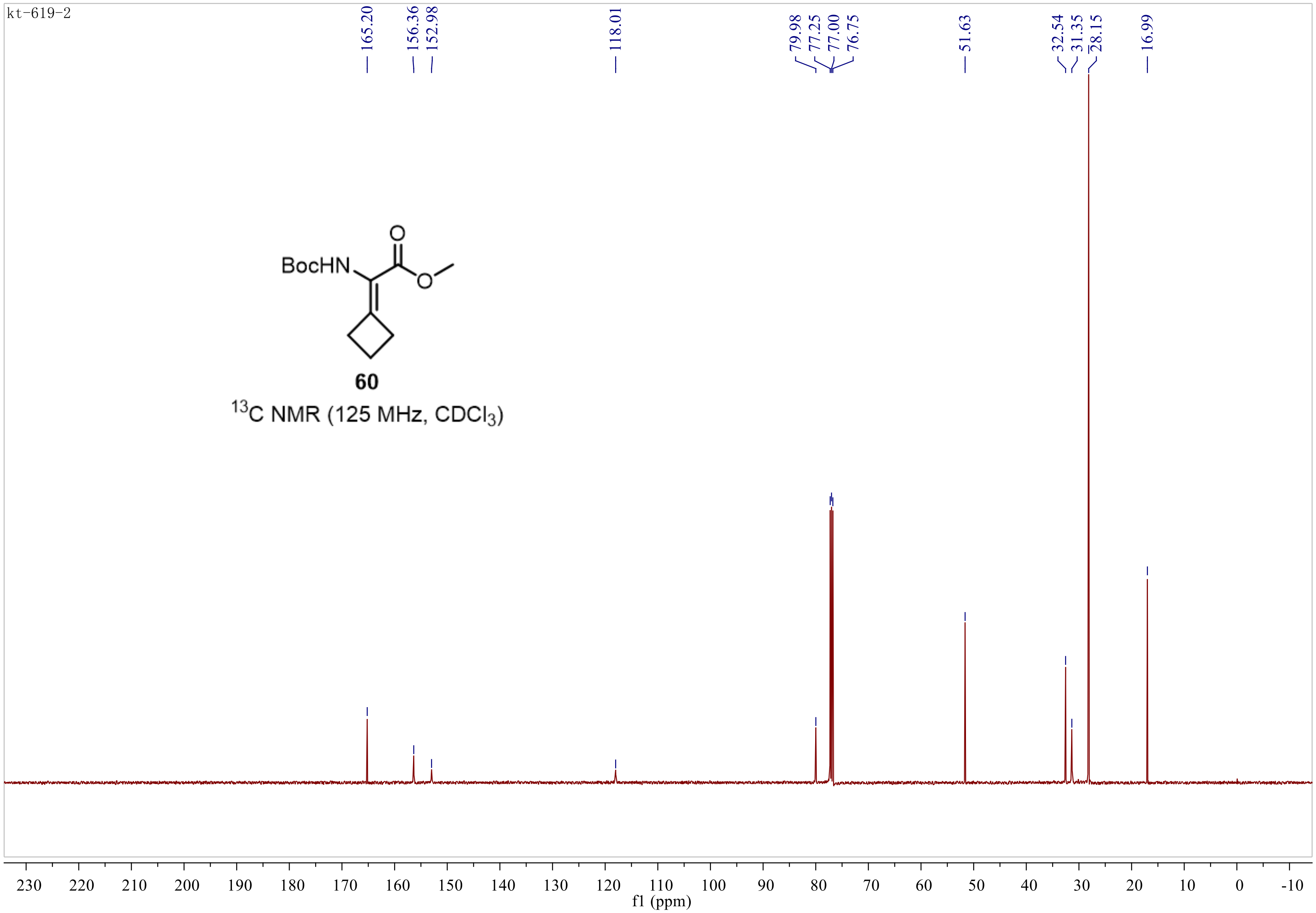

Supplementary Fig. 66. ^{13}C NMR spectra of compound **60** in $CDCl_3$

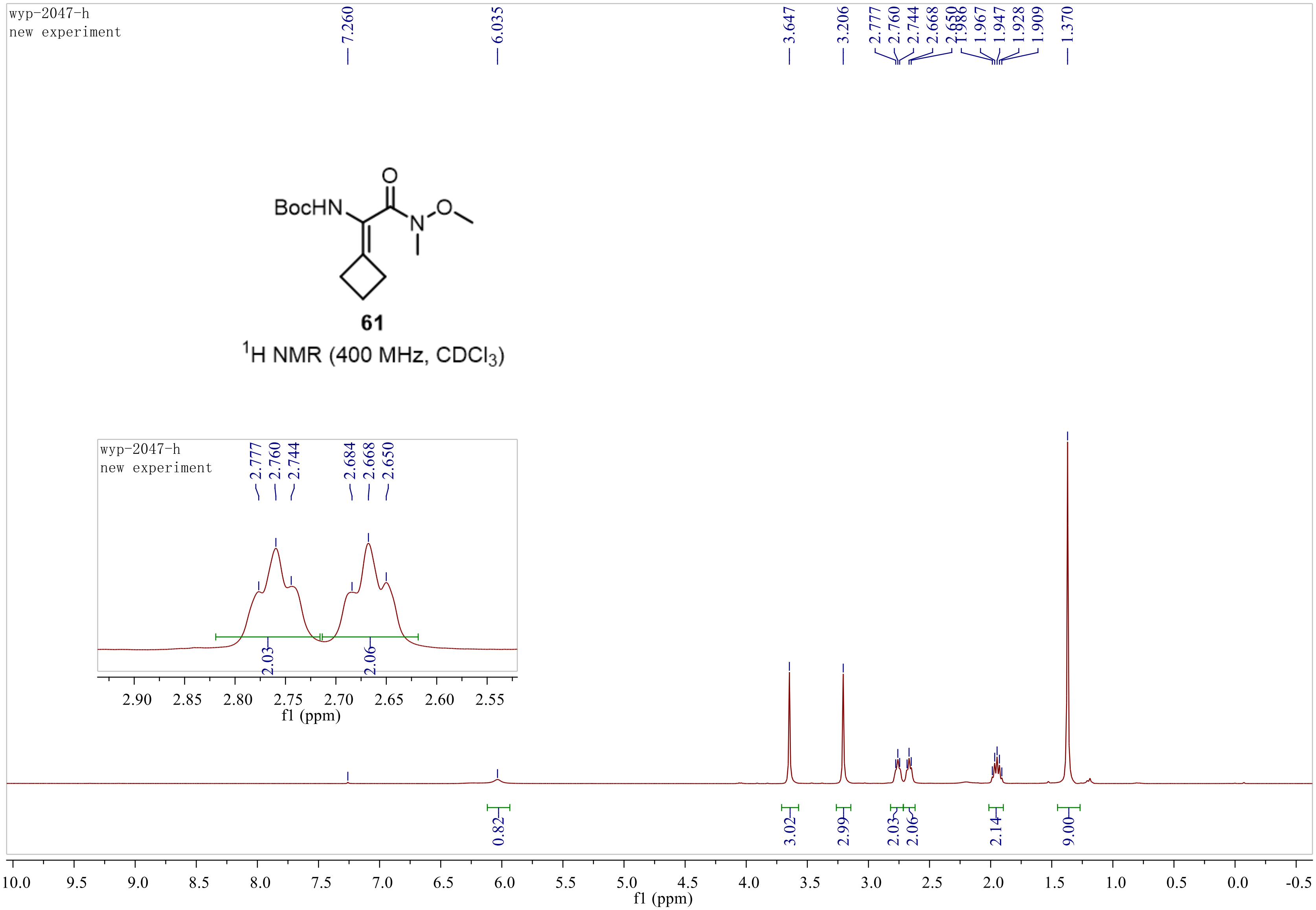

Supplementary Fig. 67. ^{1}H NMR spectra of compound **61** in $CDCl_3$

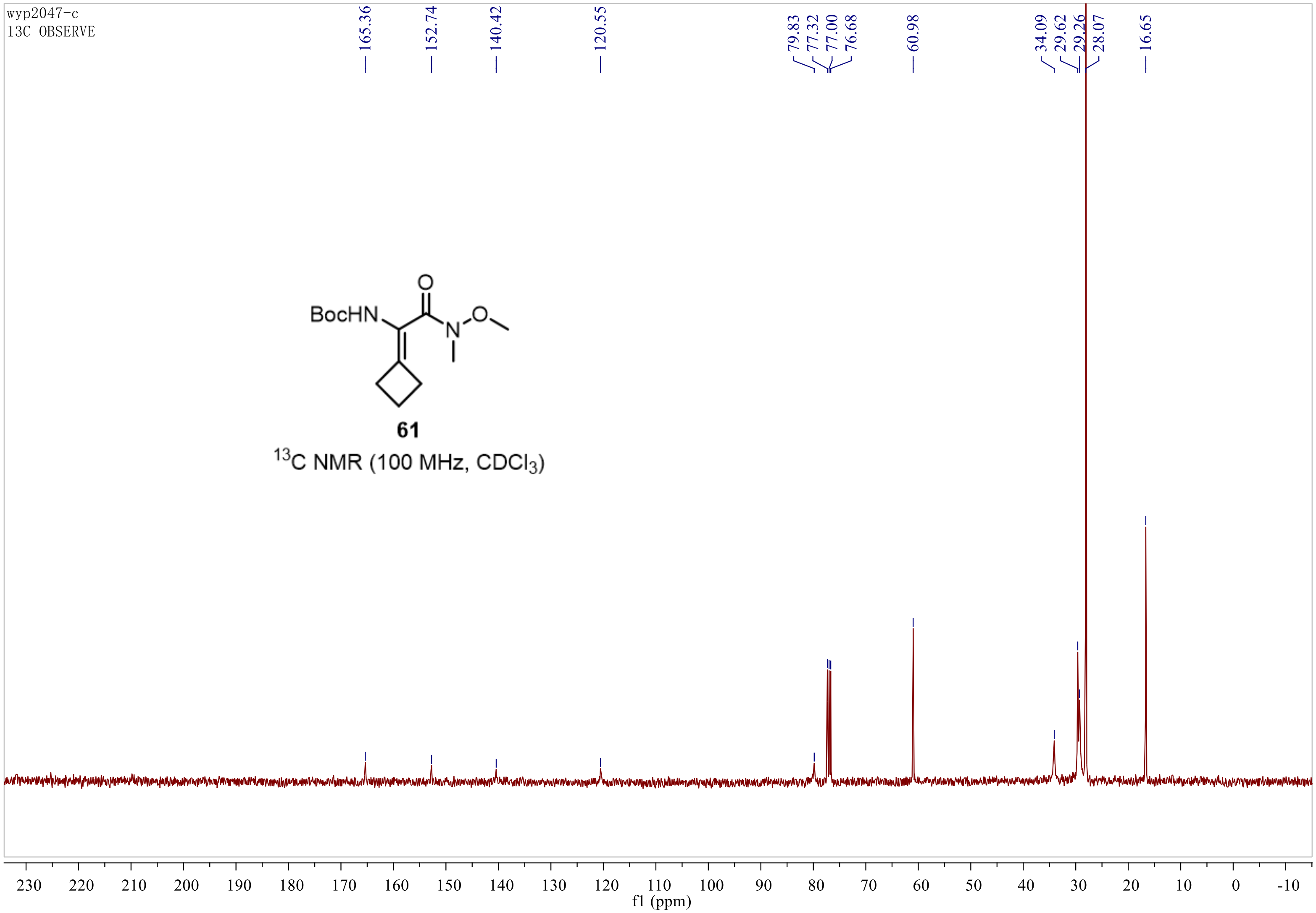

Supplementary Fig. 68. ^{13}C NMR spectra of compound **61** in $CDCl_3$

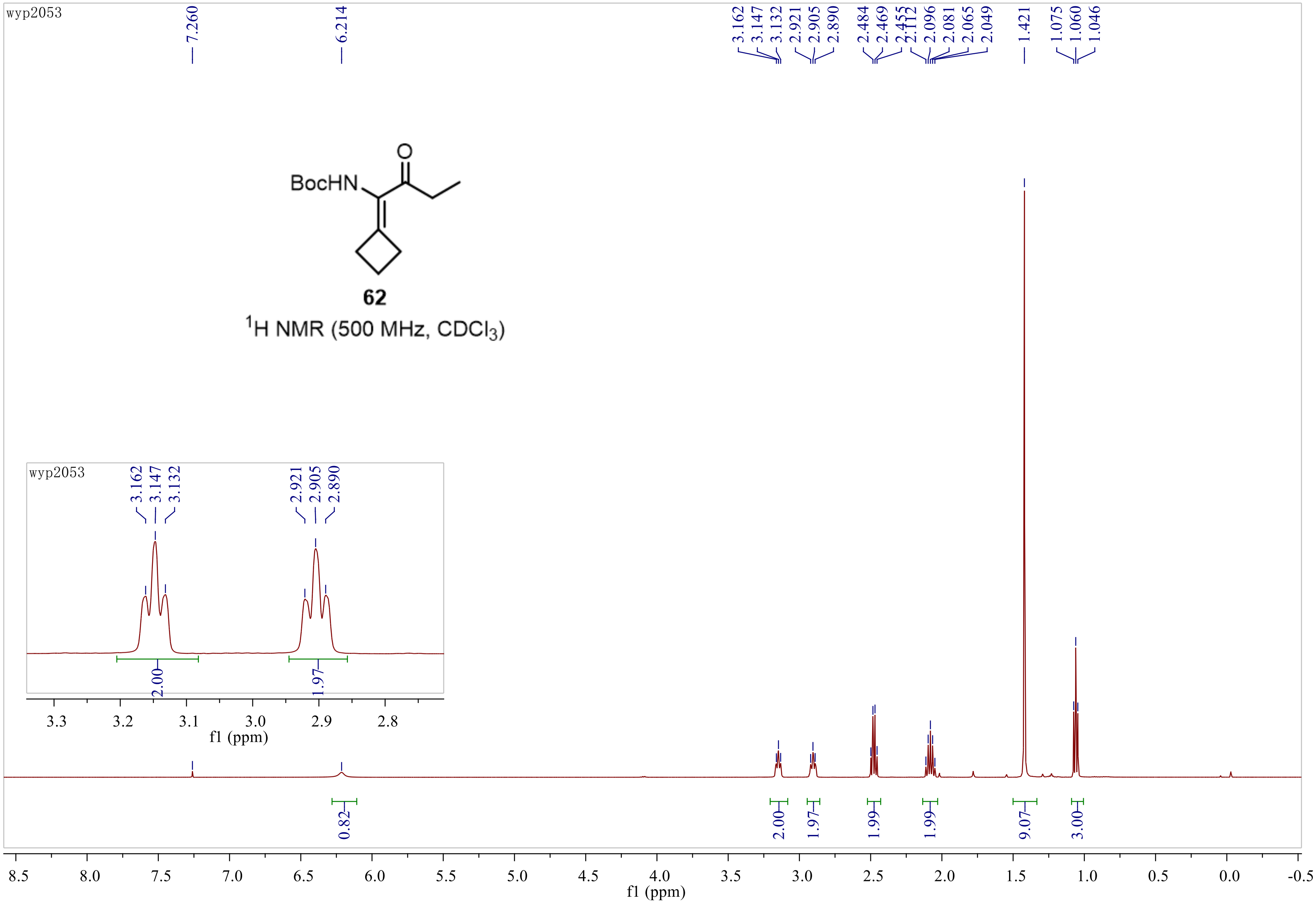

Supplementary Fig. 69. ^{1}H NMR spectra of compound **62** in $CDCl_3$

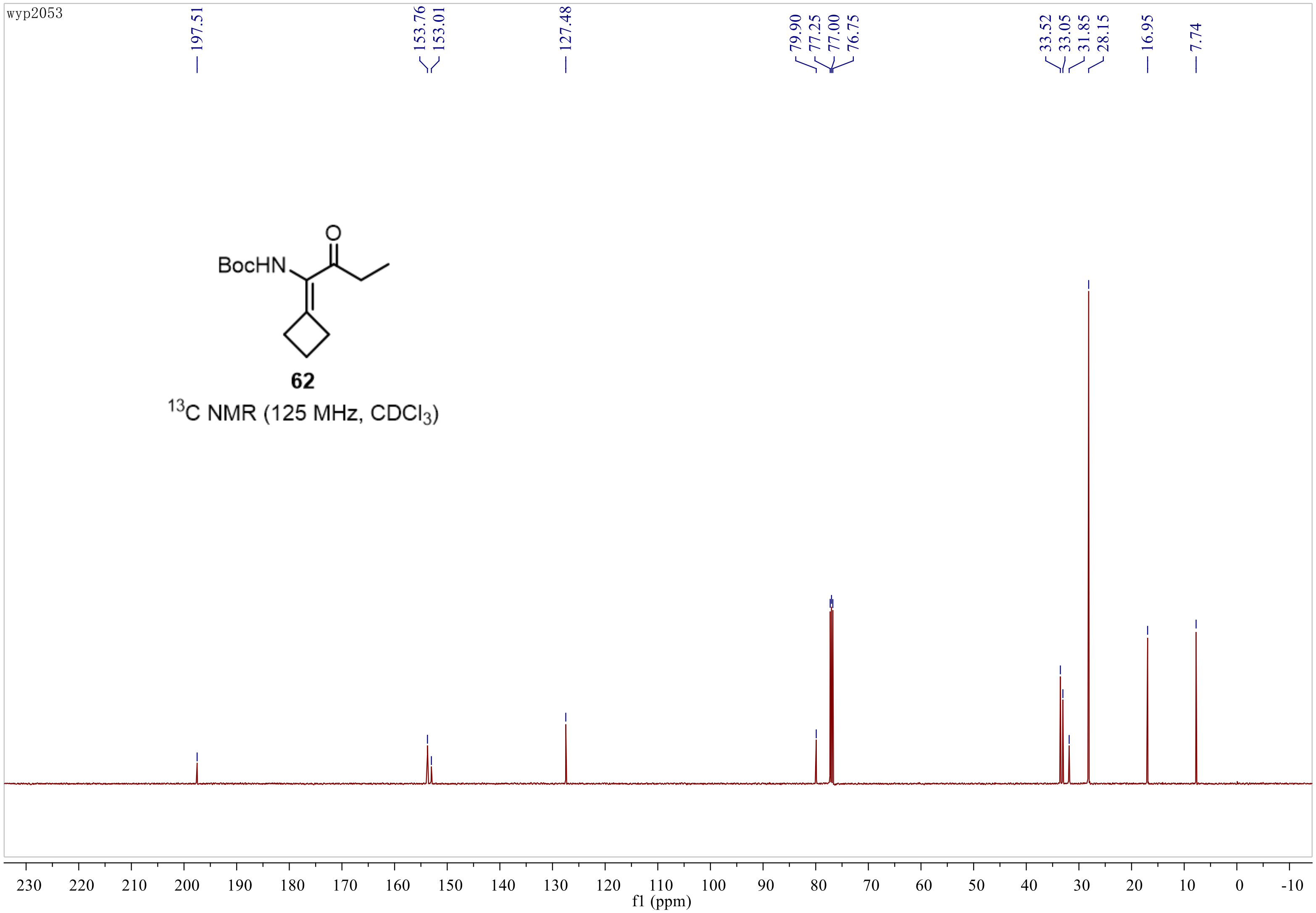

Supplementary Fig. 70. ^{13}C NMR spectra of compound **62** in $CDCl_3$

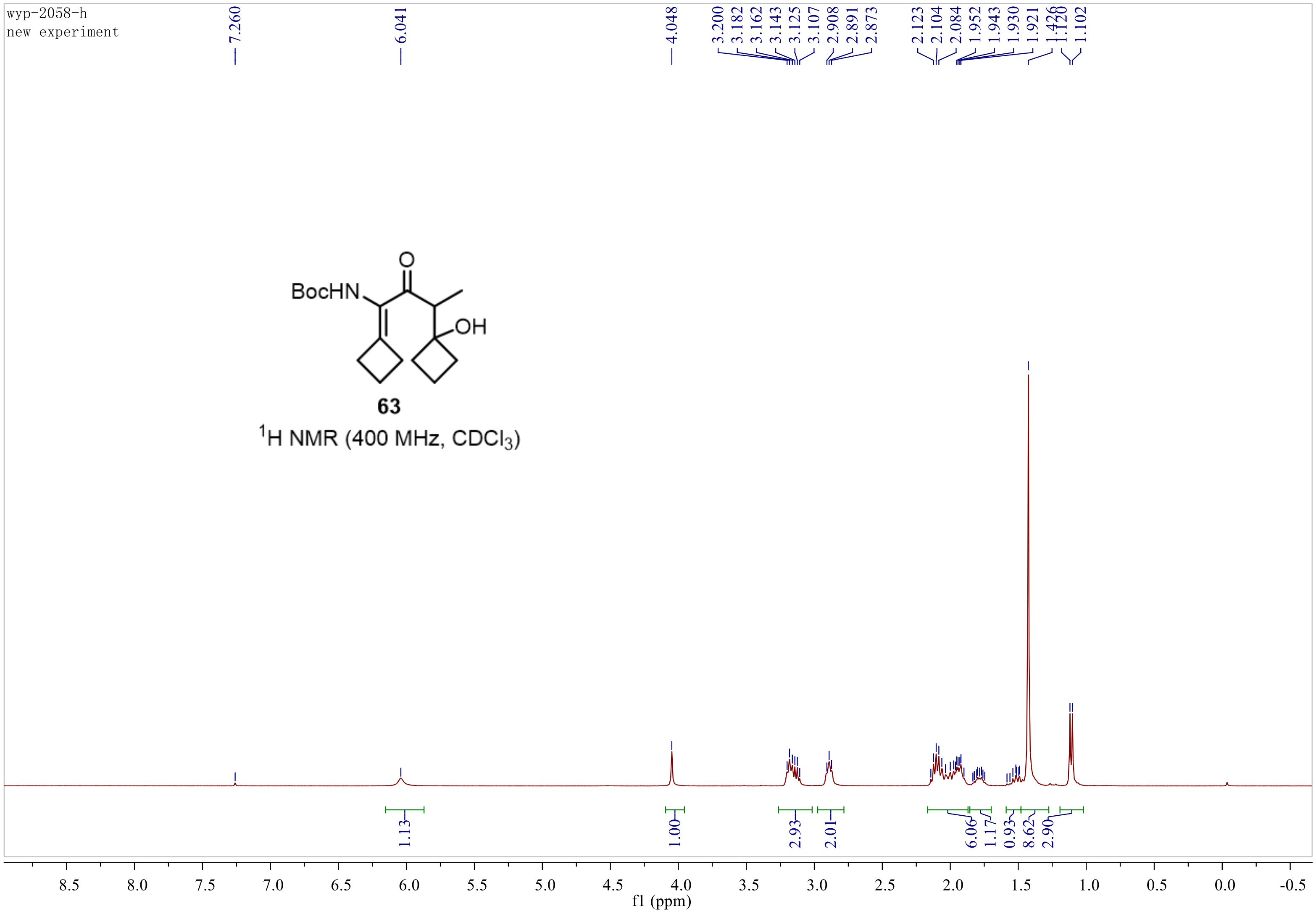

Supplementary Fig. 71. ^{1}H NMR spectra of compound **63** in $CDCl_3$

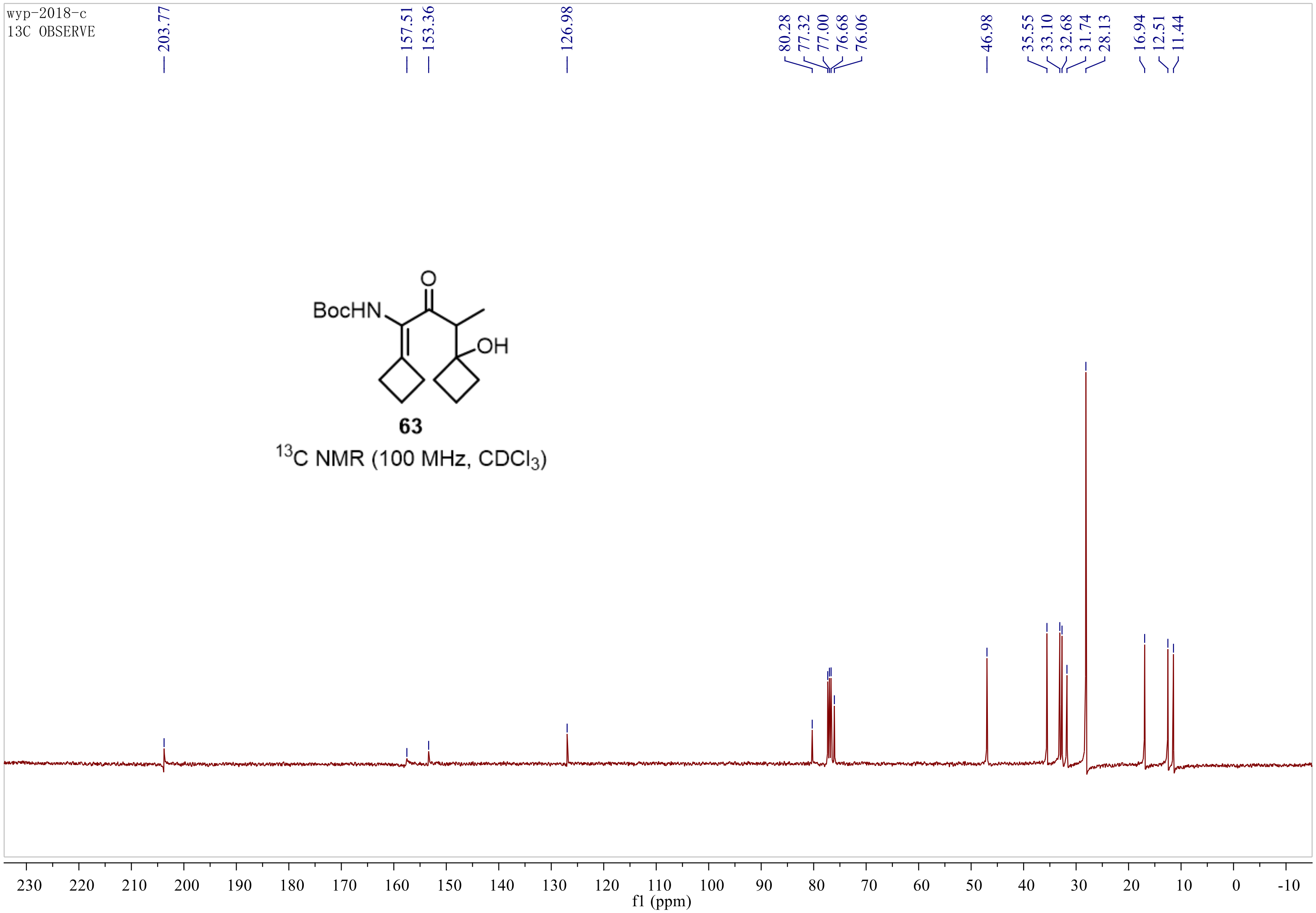

Supplementary Fig. 72. ^{13}C NMR spectra of compound **63** in $CDCl_3$

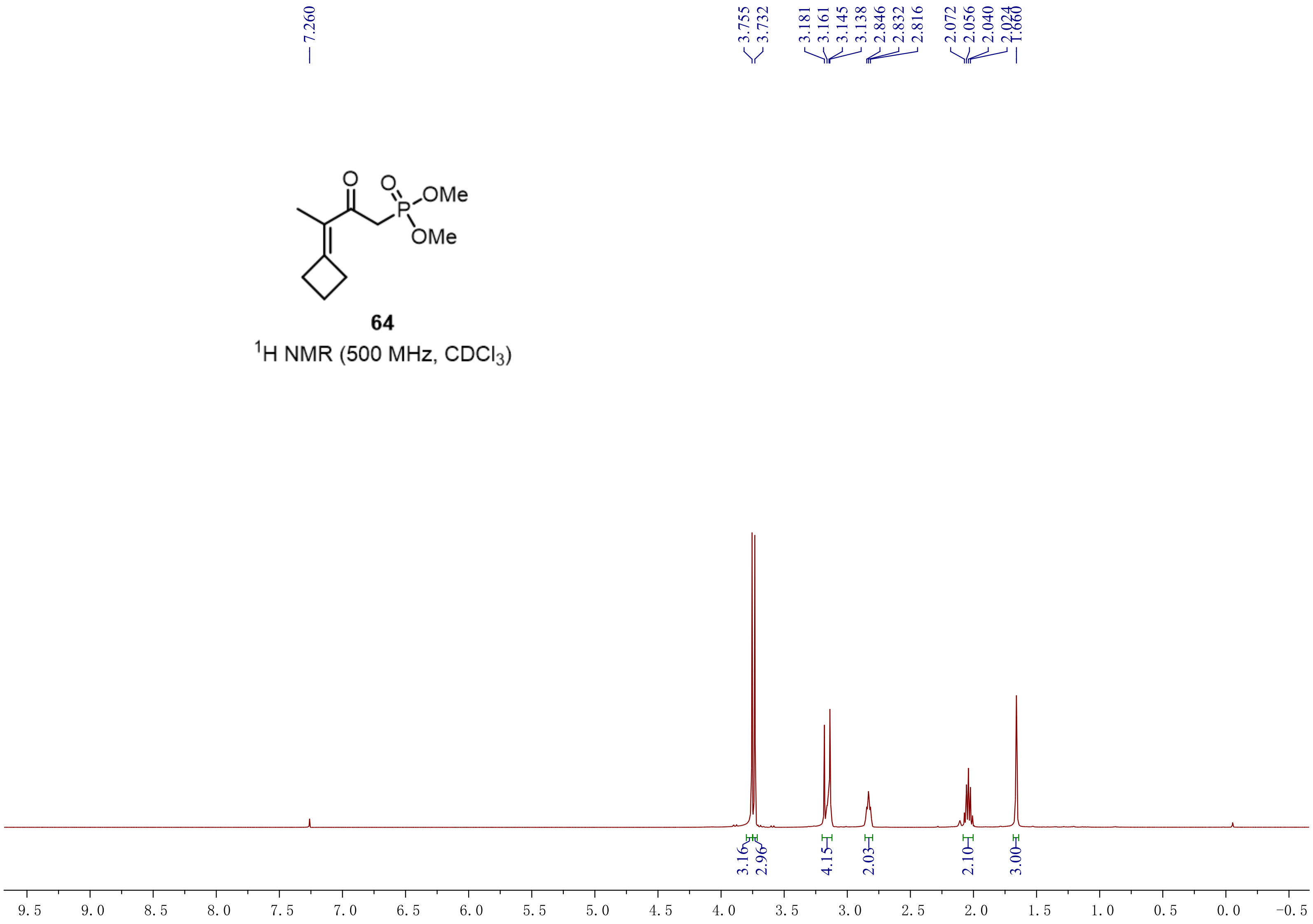

Supplementary Fig. 73. ^{1}H NMR spectra of compound **64** in $CDCl_3$

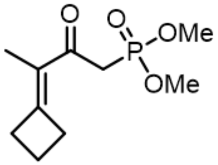

64

^{13}C NMR (125 MHz, $CDCl_3$)

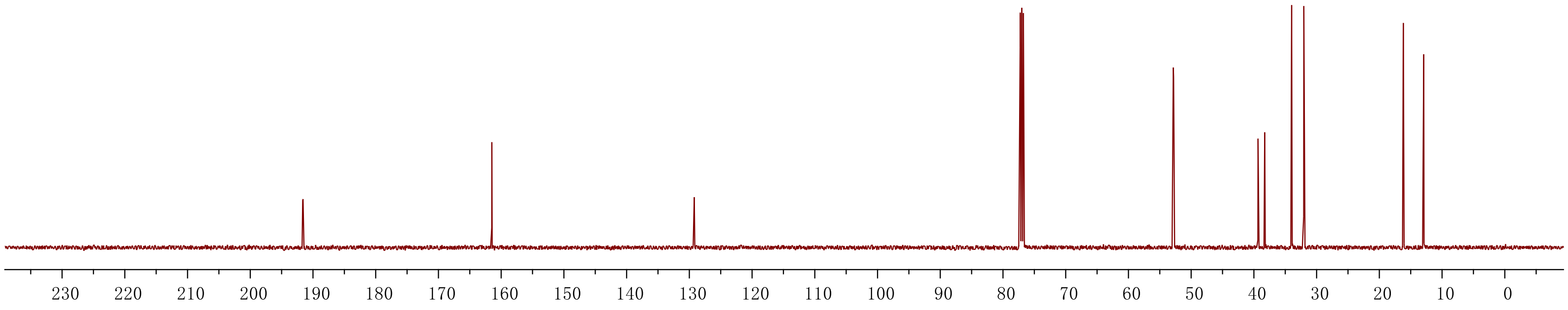

Supplementary Fig. 74. ^{13}C NMR spectra of compound **64** in $CDCl_3$

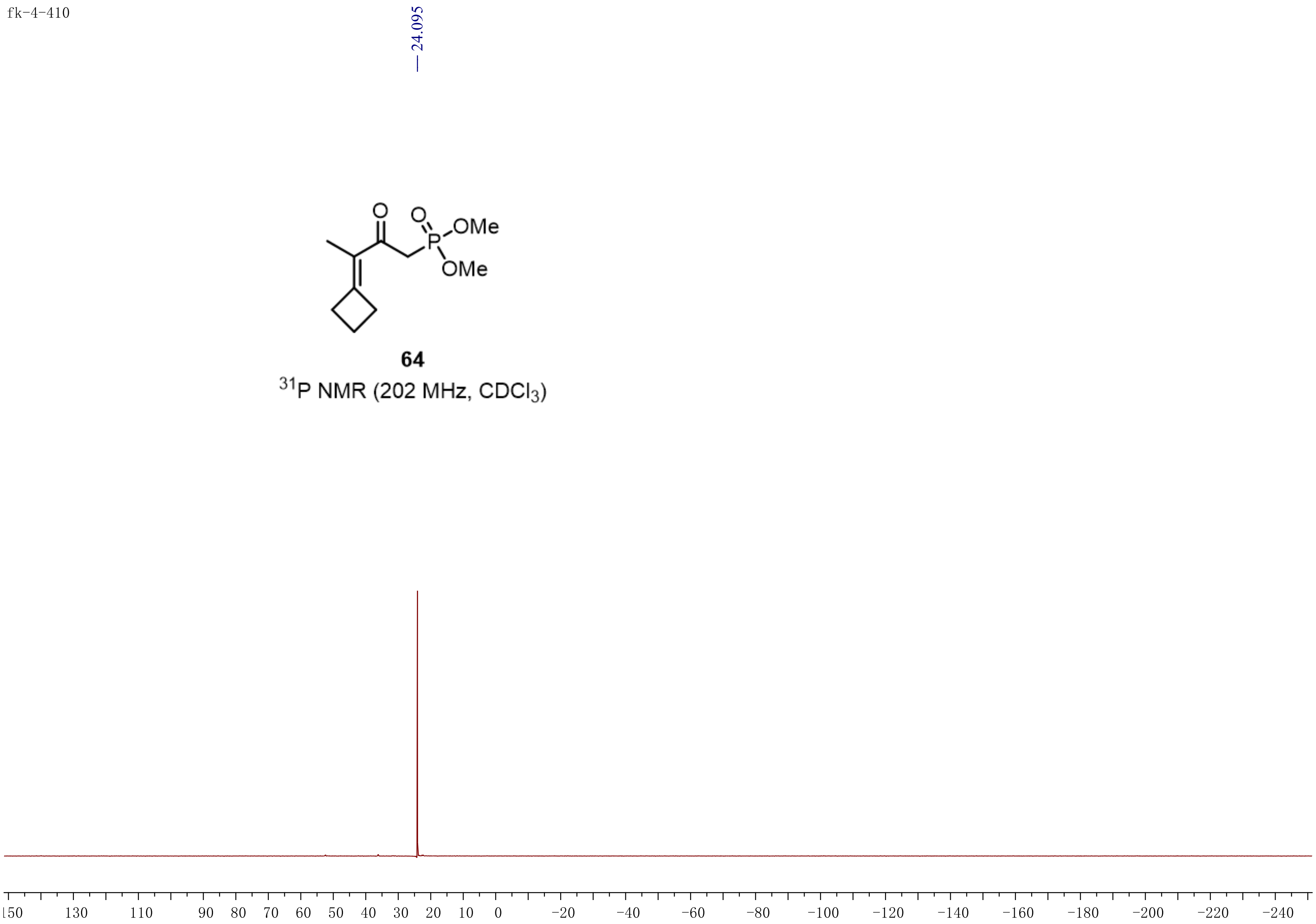

Supplementary Fig. 75. ^{31}P NMR spectra of compound **64** in $CDCl_3$

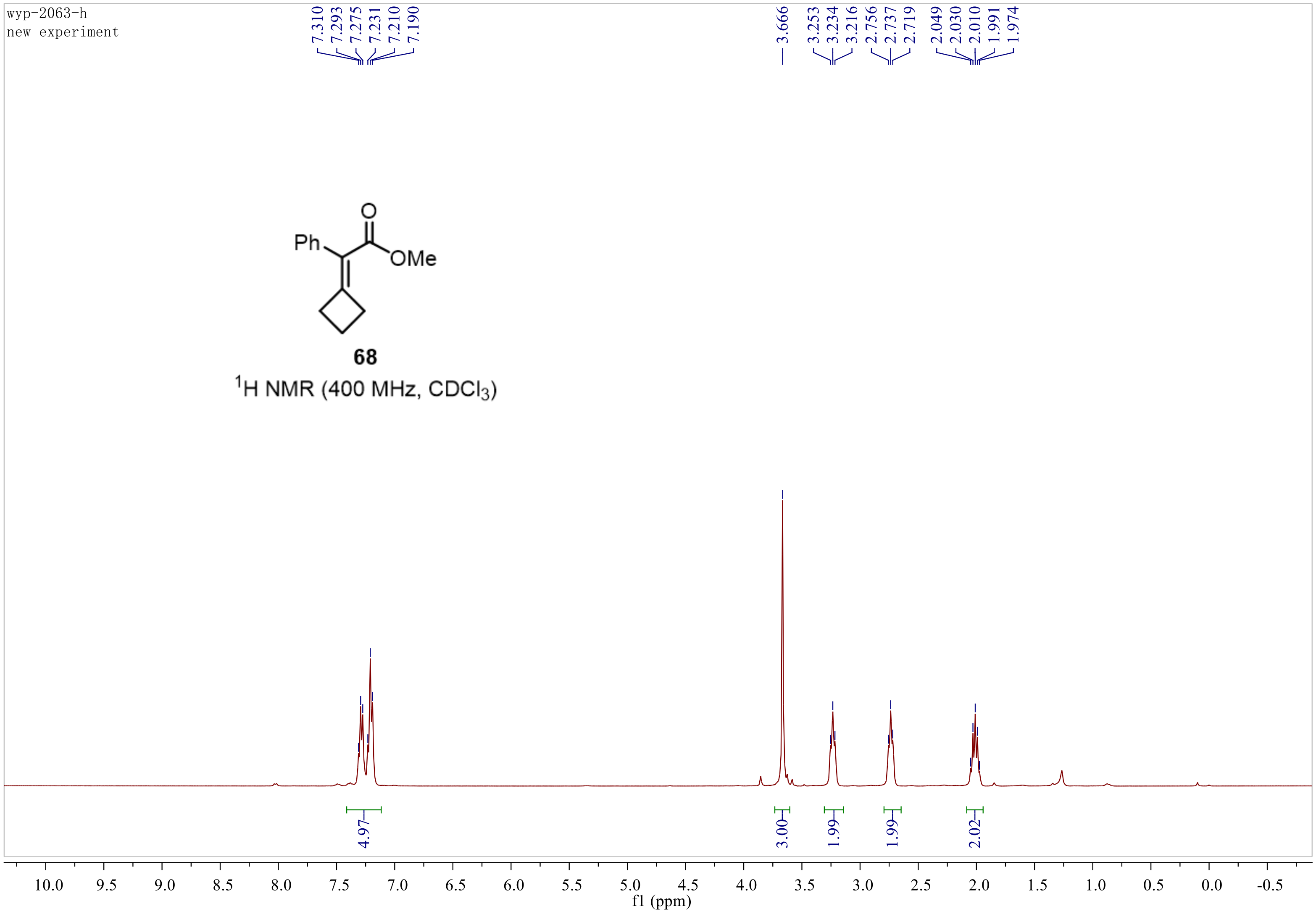

Supplementary Fig. 76. ^{1}H NMR spectra of compound **68** in $CDCl_3$

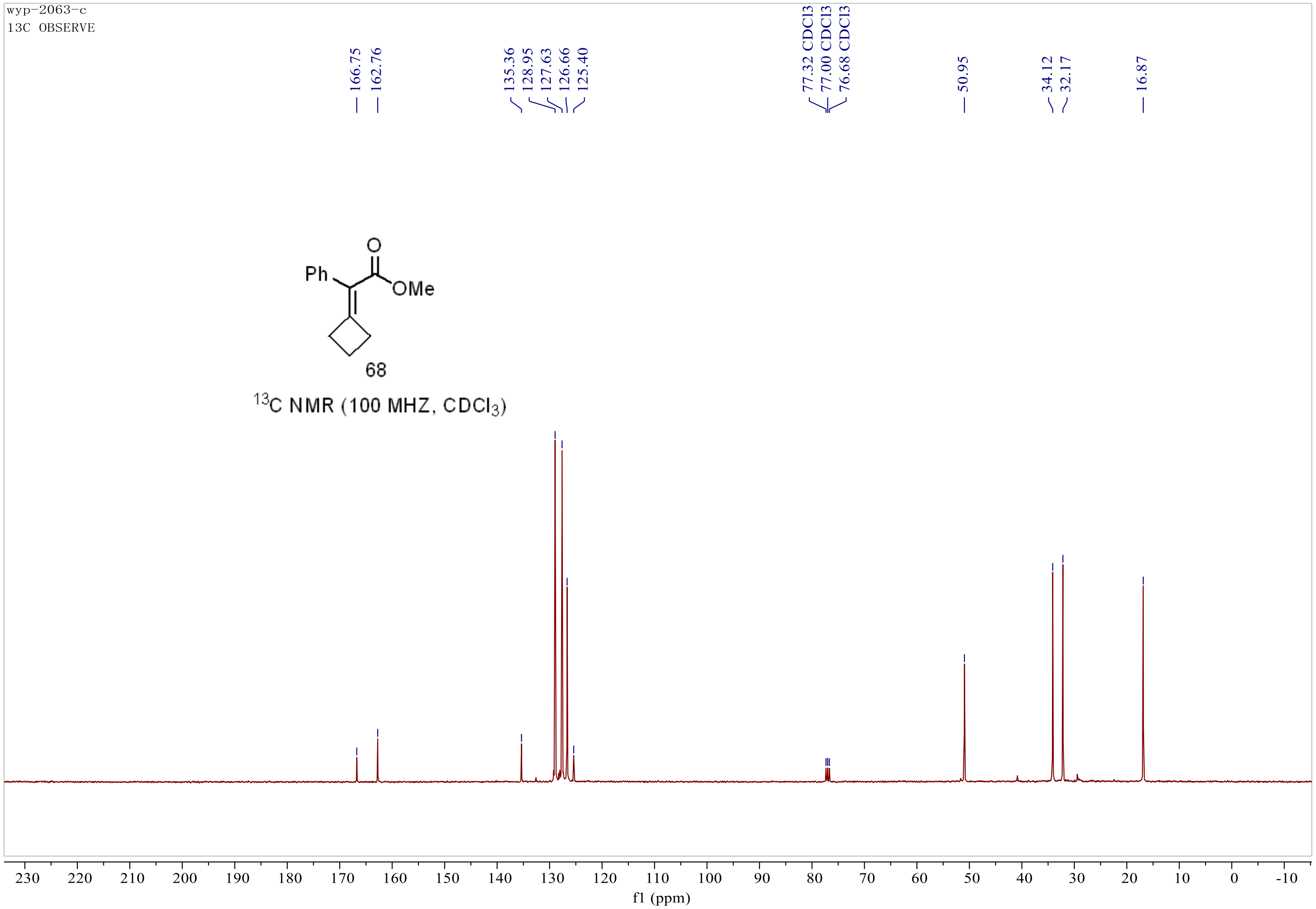

Supplementary Fig. 77. ^{13}C NMR spectra of compound **68** in $CDCl_3$

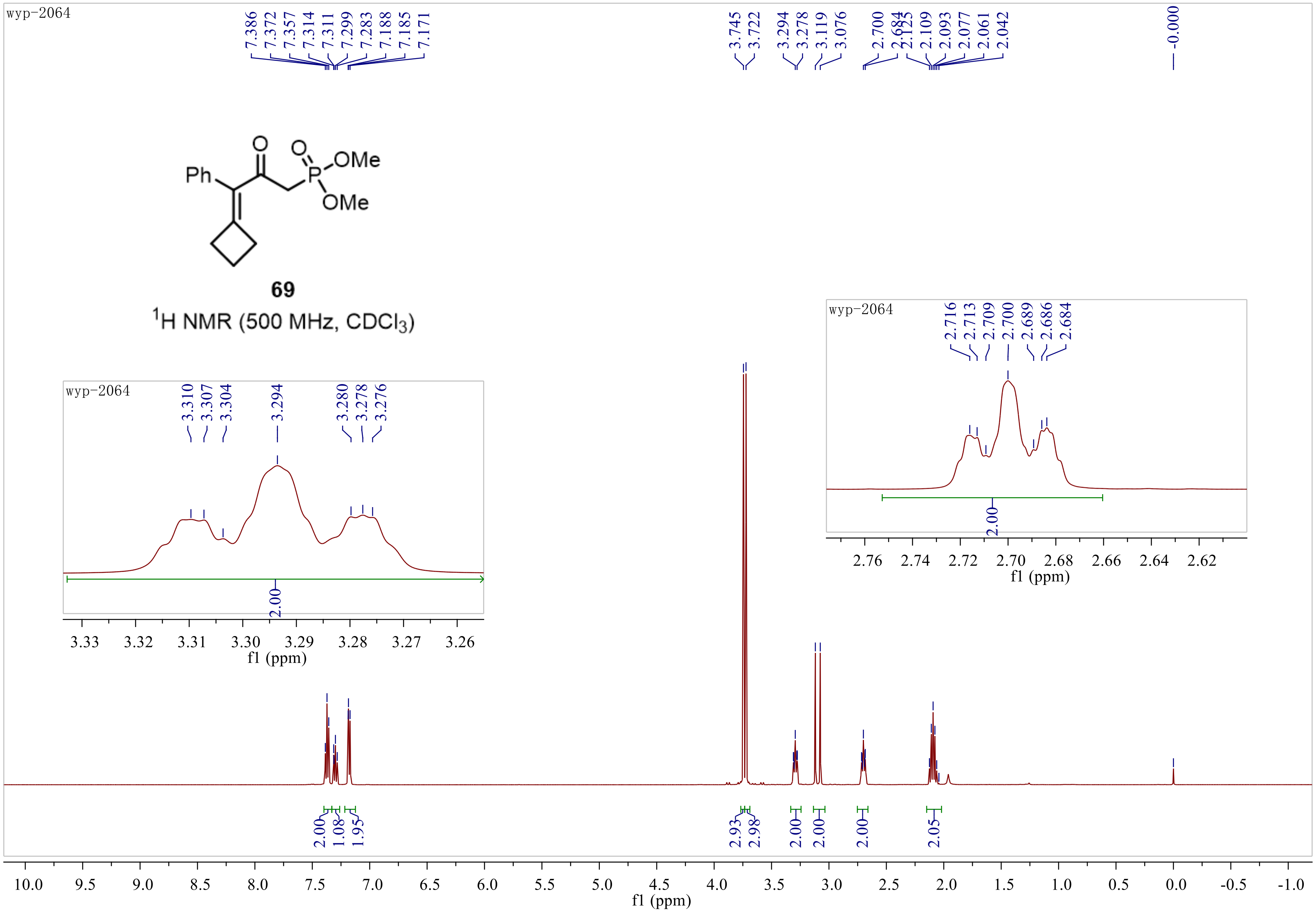

Supplementary Fig. 78. ^{1}H NMR spectra of compound **69** in $CDCl_3$

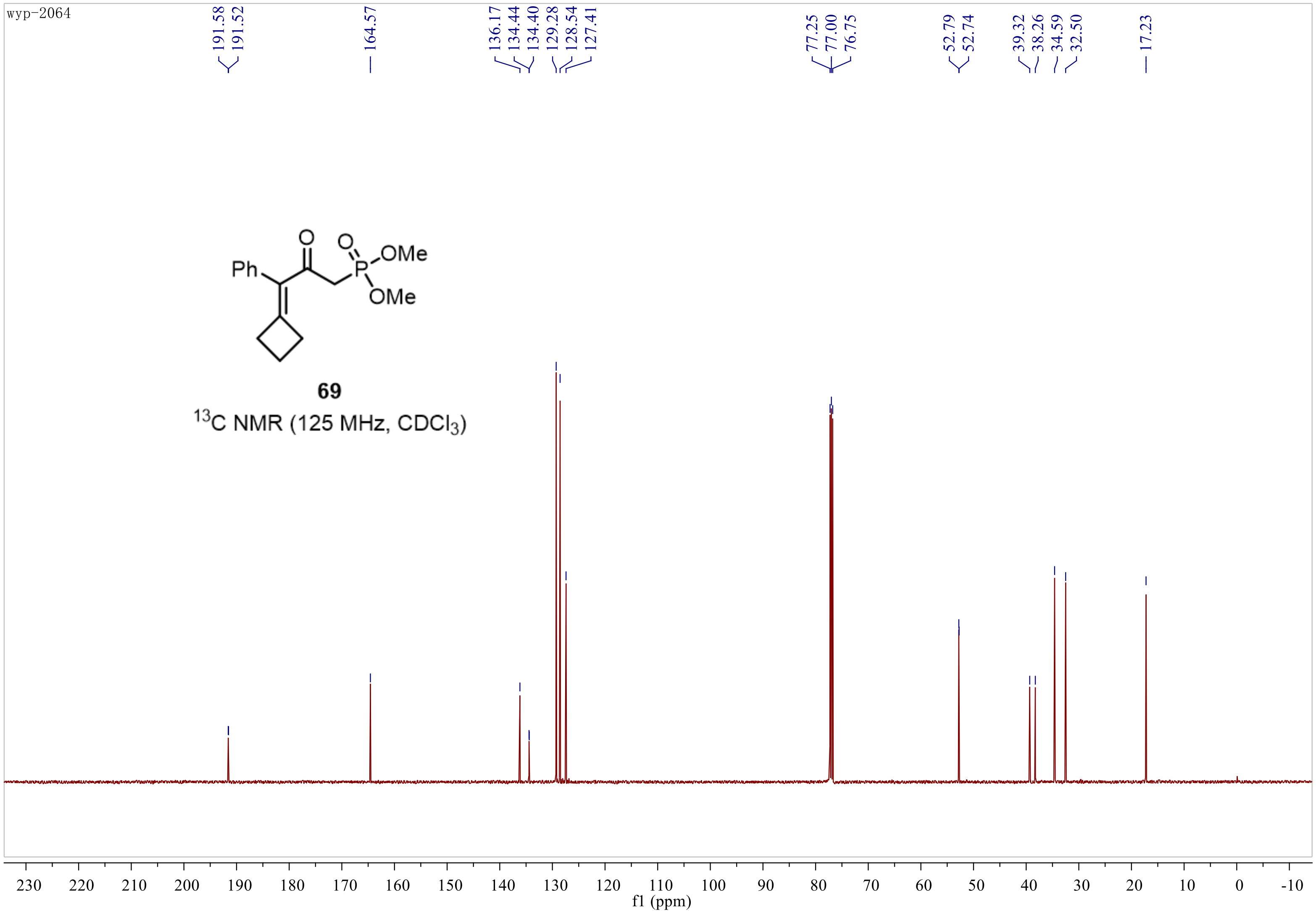

Supplementary Fig. 79.^{13}C NMR spectra of compound **69** in $CDCl_3$

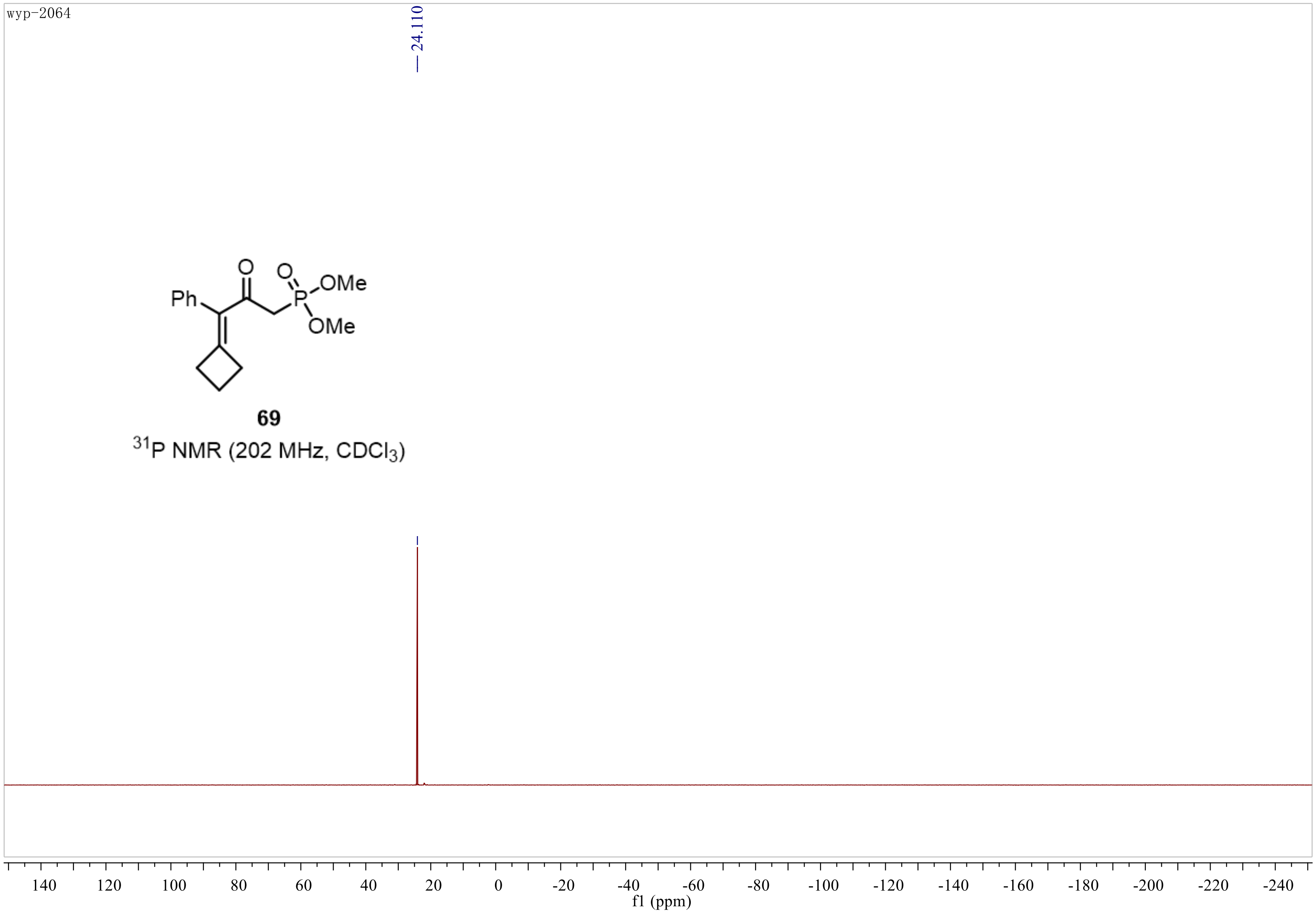

Supplementary Fig. 80. ^{31}P NMR spectra of compound **69** in $CDCl_3$

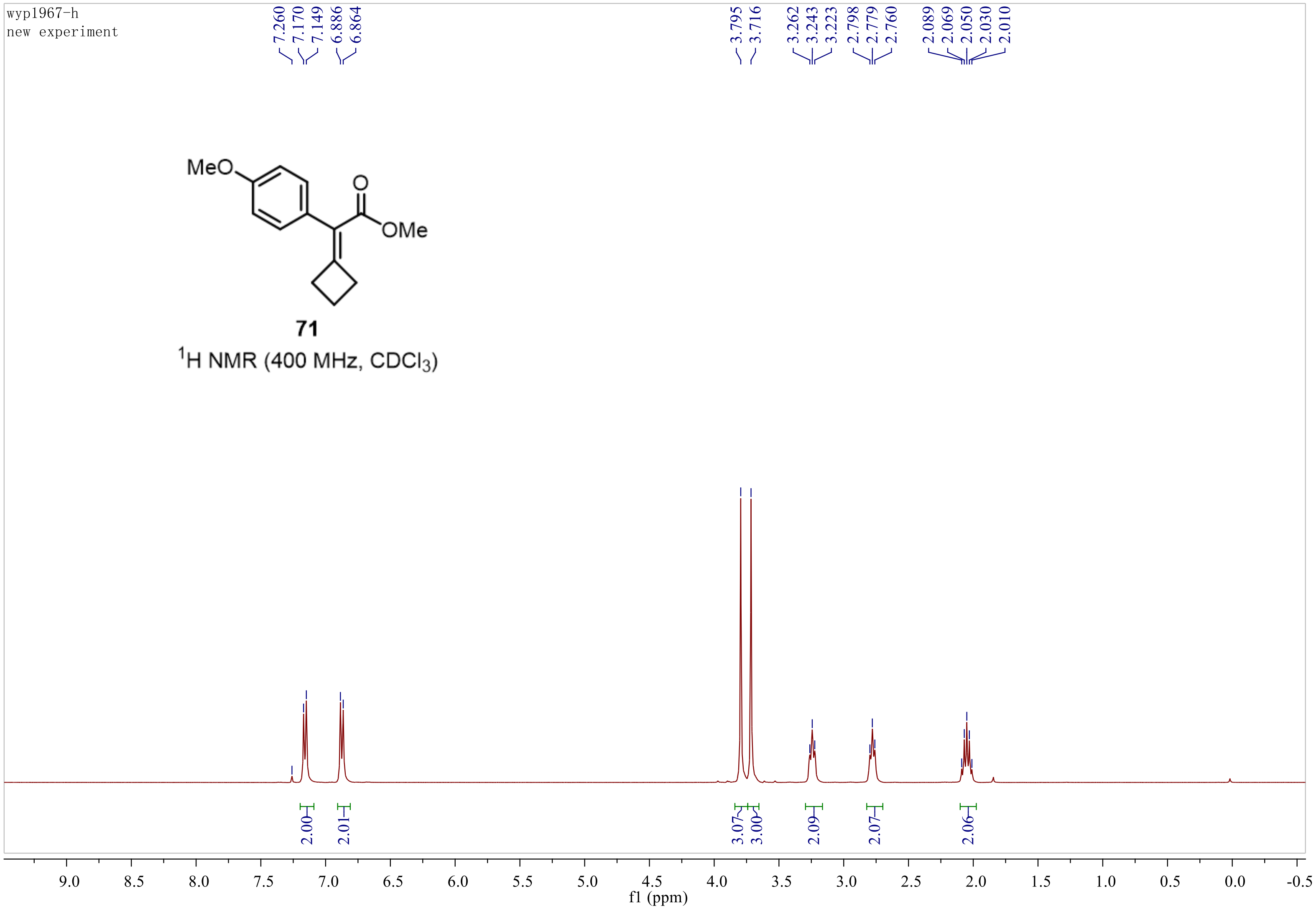

Supplementary Fig. 81. ^{1}H NMR spectra of compound **71** in $CDCl_3$

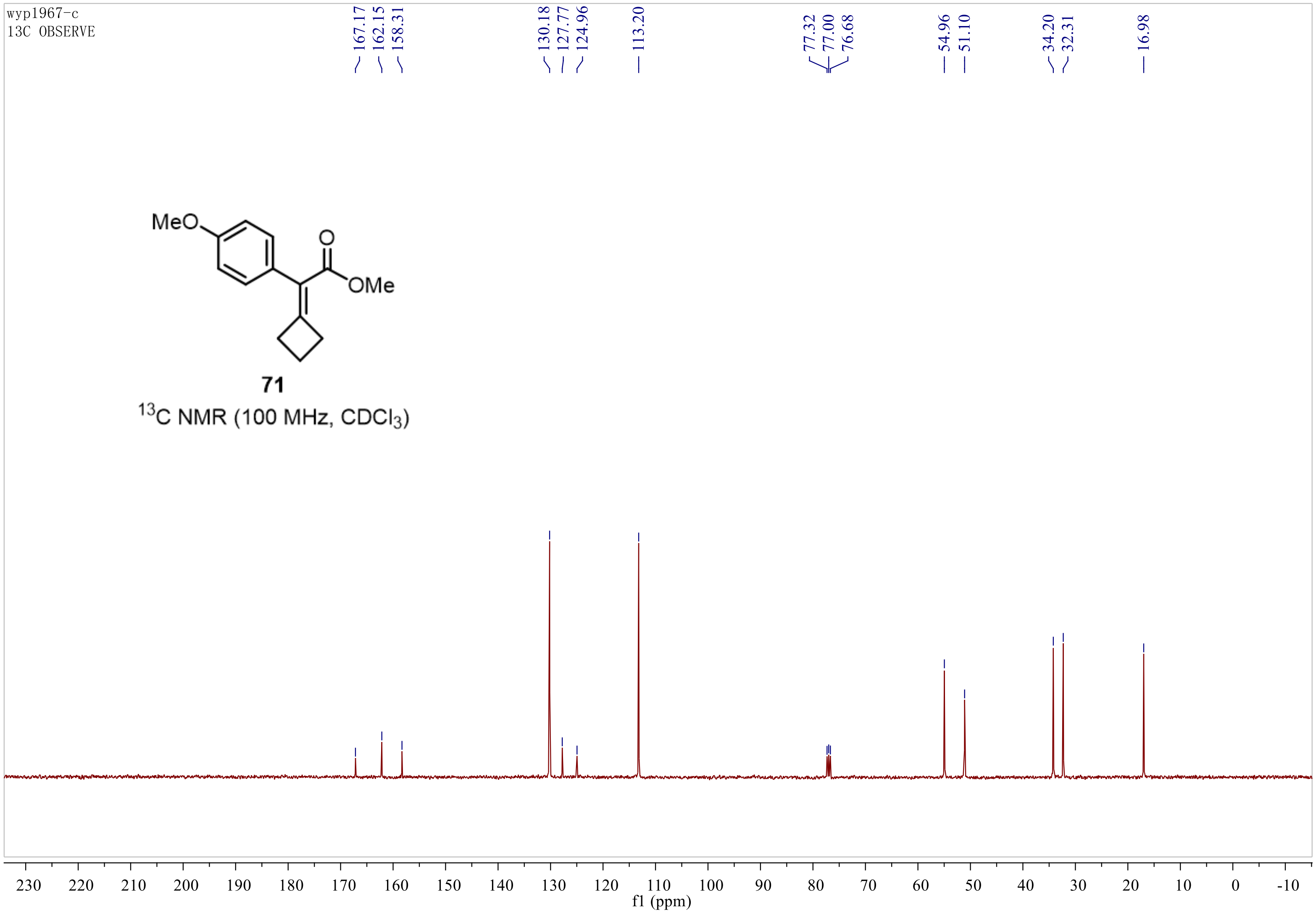

Supplementary Fig. 82. ^{13}C NMR spectra of compound **71** in $CDCl_3$

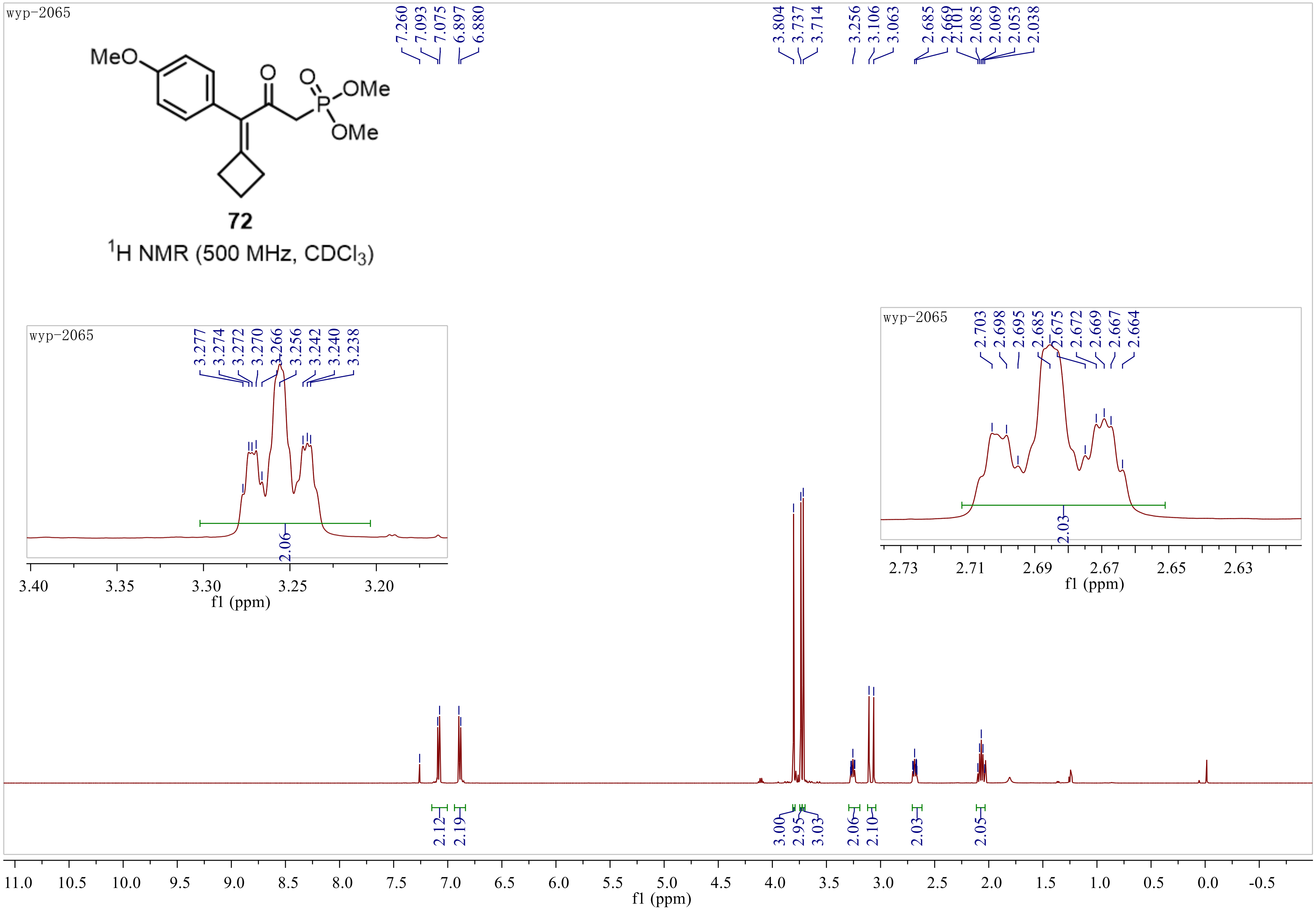

Supplementary Fig. 83. ^{1}H NMR spectra of compound **72** in $CDCl_3$

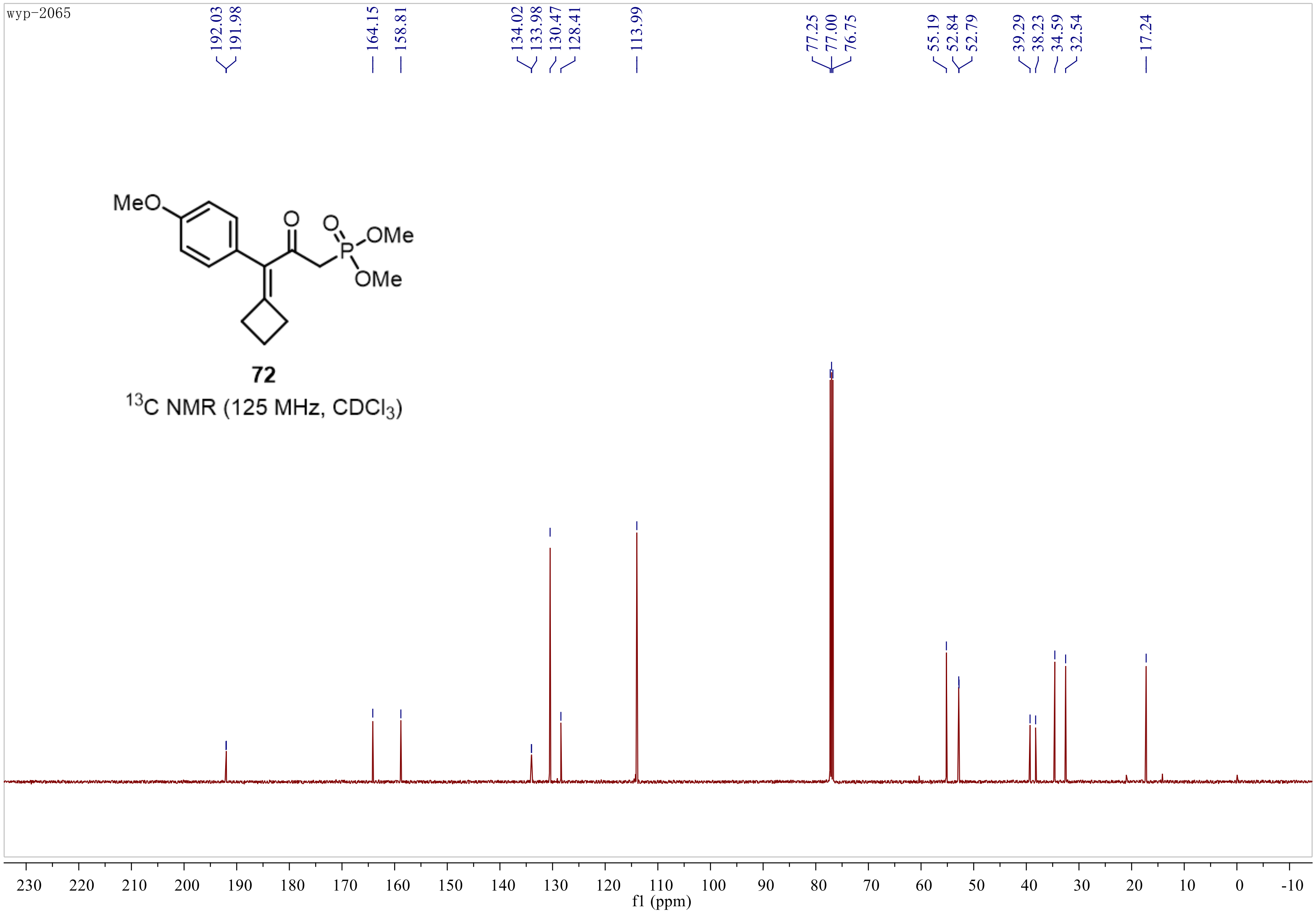

Supplementary Fig. 84. ^{13}C NMR spectra of compound **72** in $CDCl_3$

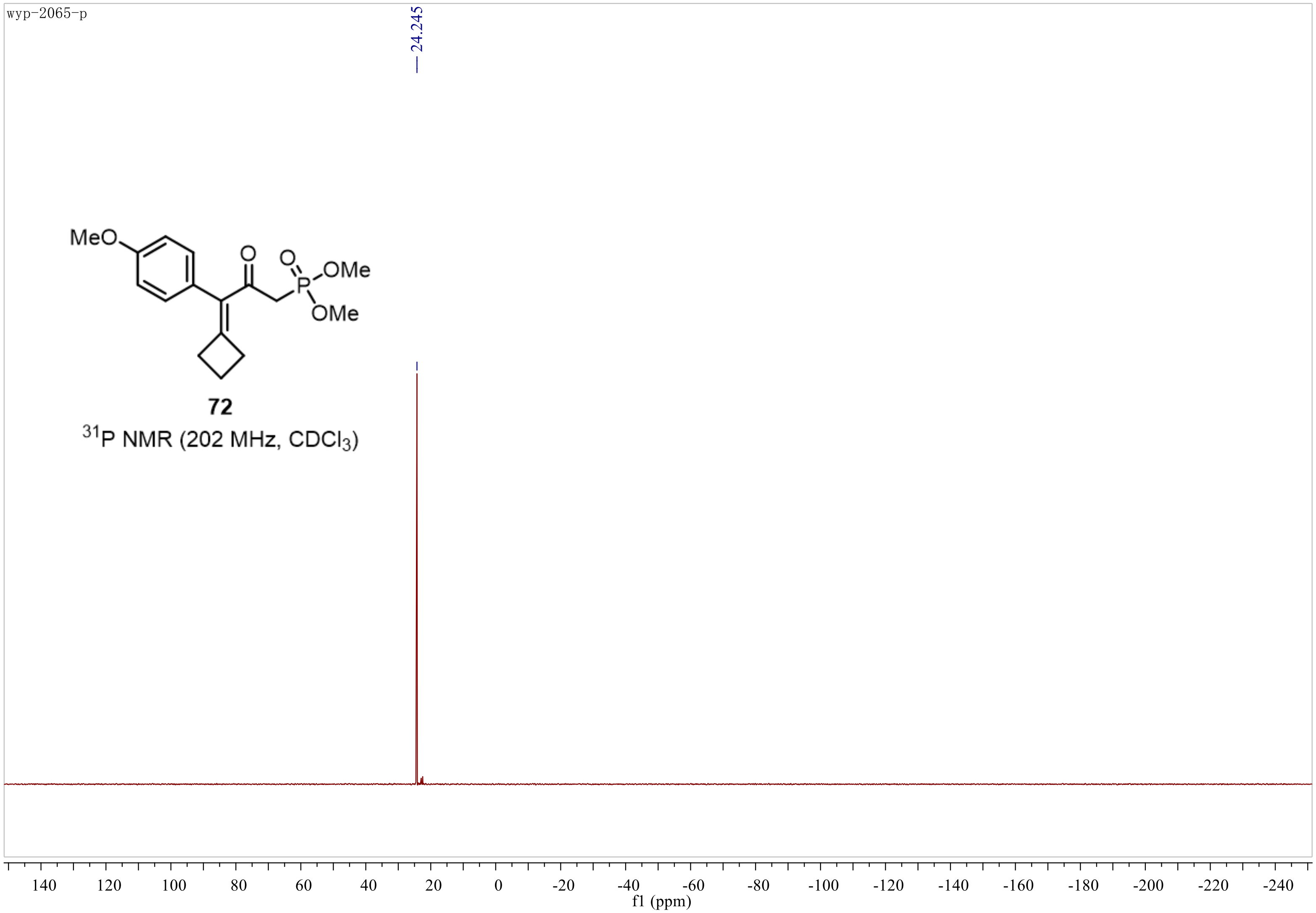

Supplementary Fig. 85. ^{31}P NMR spectra of compound **72** in $CDCl_3$

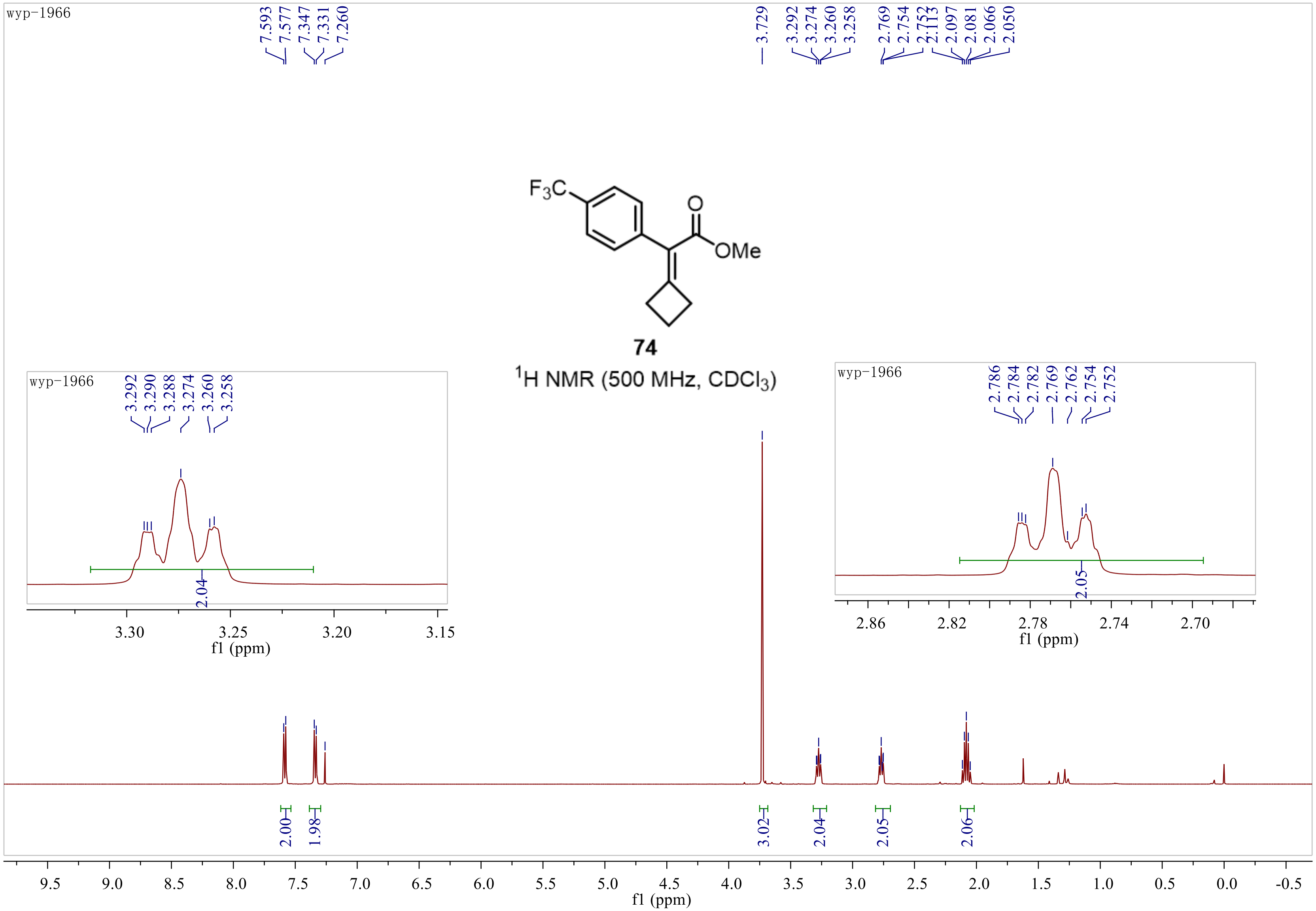

Supplementary Fig. 86. ^{1}H NMR spectra of compound **74** in $CDCl_3$

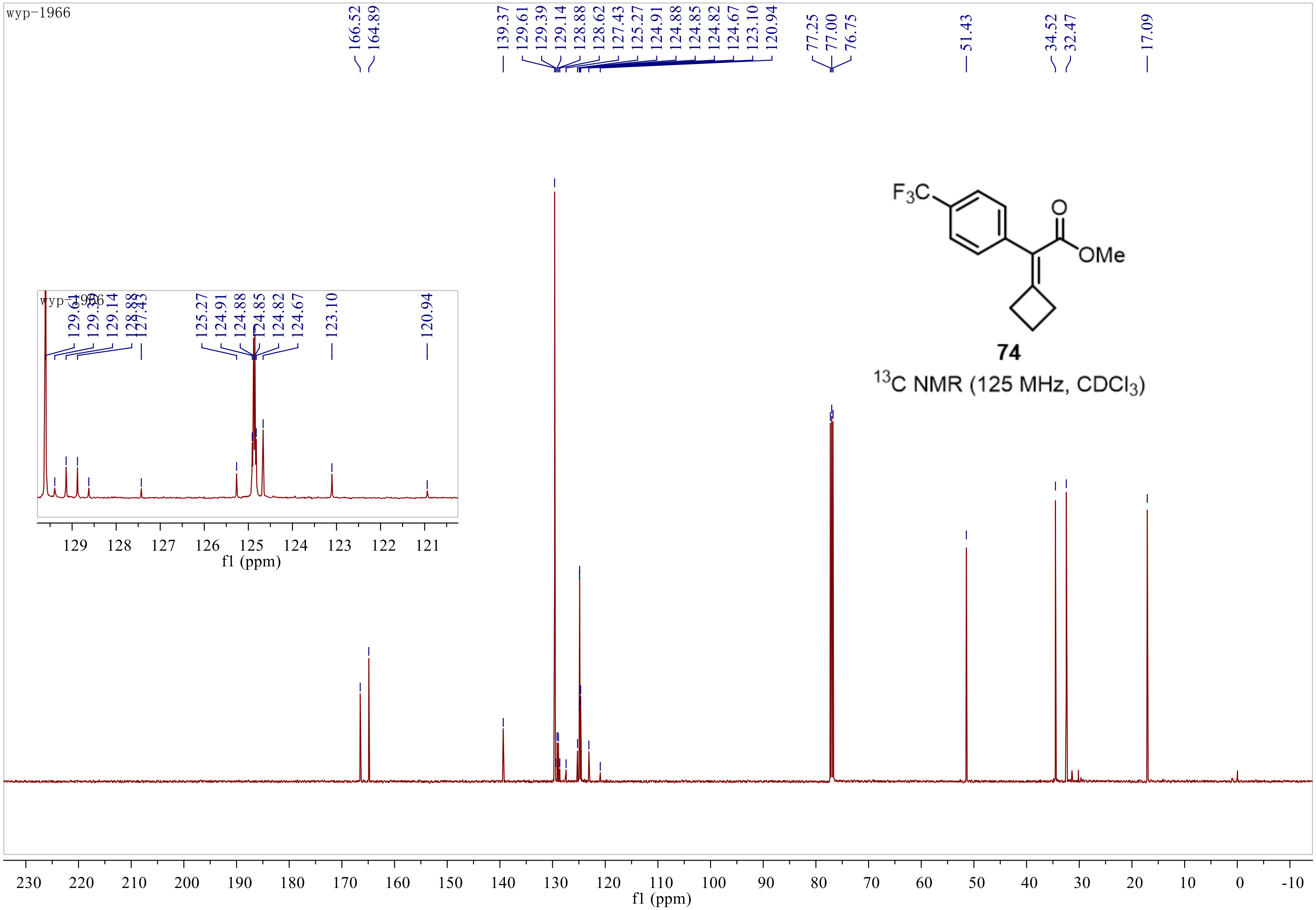

Supplementary Fig. 87. ^{13}C NMR spectra of compound **74** in $CDCl_3$

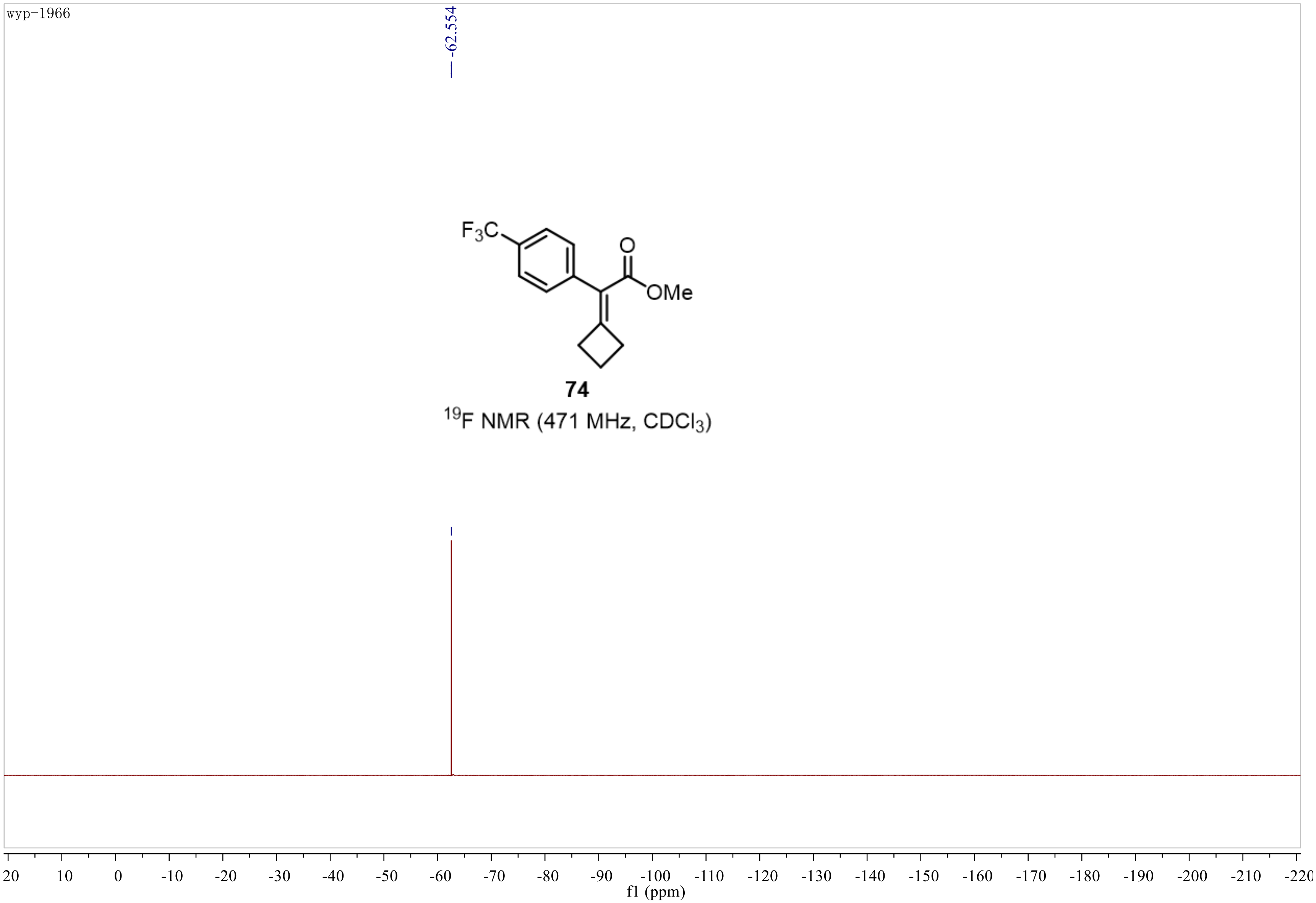

Supplementary Fig. 88.^{19}F NMR spectra of compound **74** in $CDCl_3$

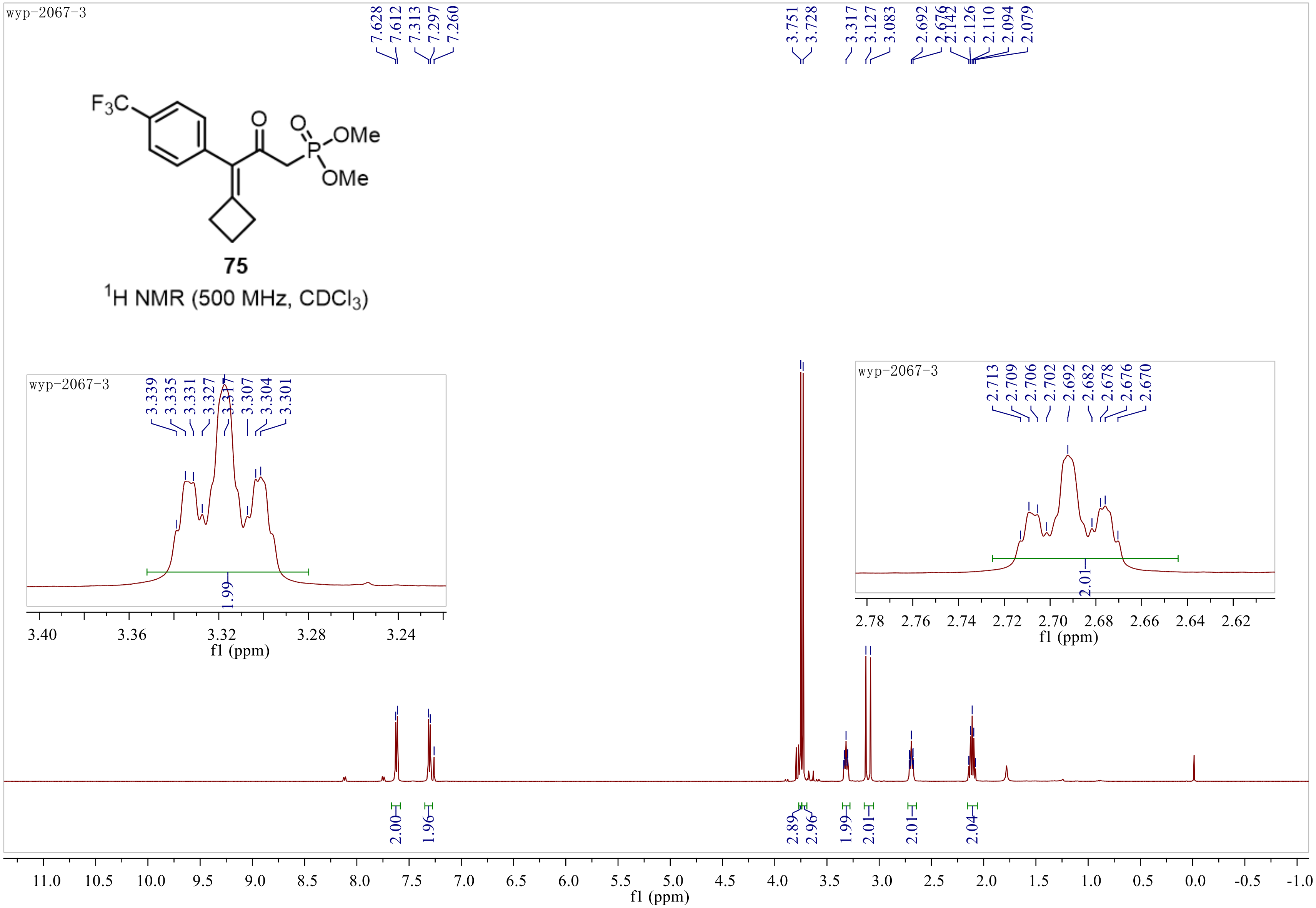

Supplementary Fig. 89. ^{1}H NMR spectra of compound **75** in $CDCl_3$

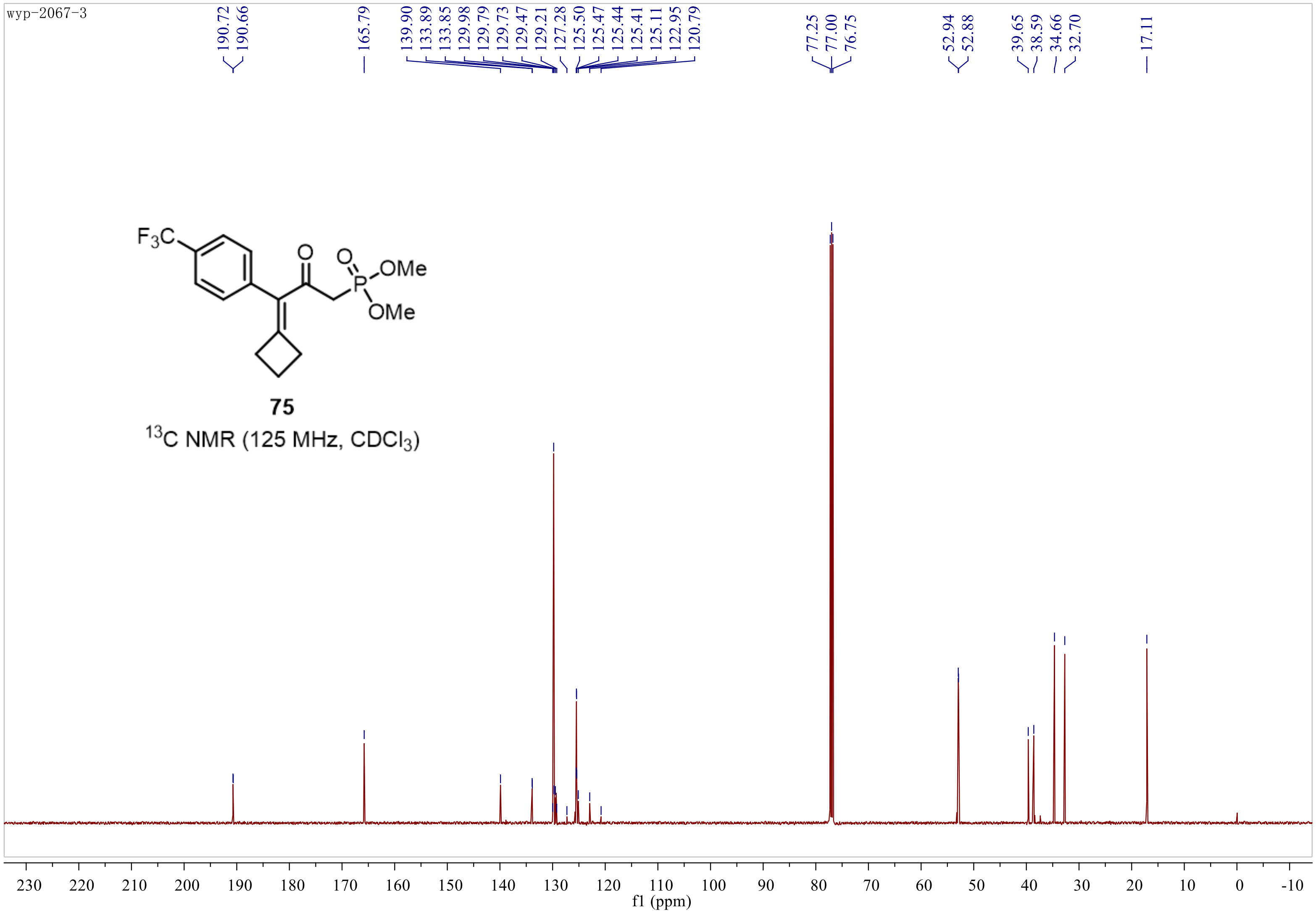

Supplementary Fig. 90. ^{13}C NMR spectra of compound **75** in $CDCl_3$

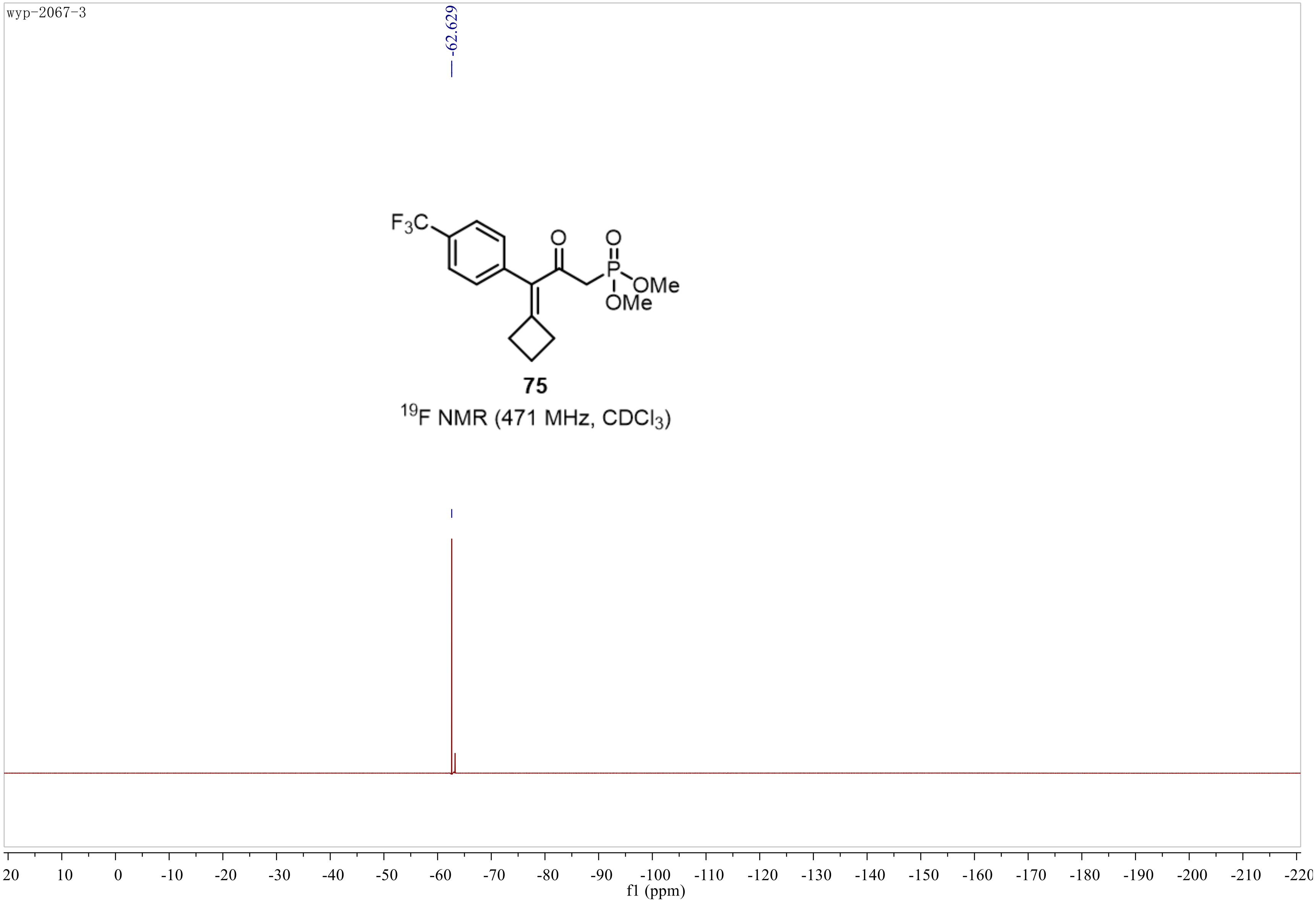

Supplementary Fig. 91. ^{19}F NMR spectra of compound **75** in $CDCl_3$

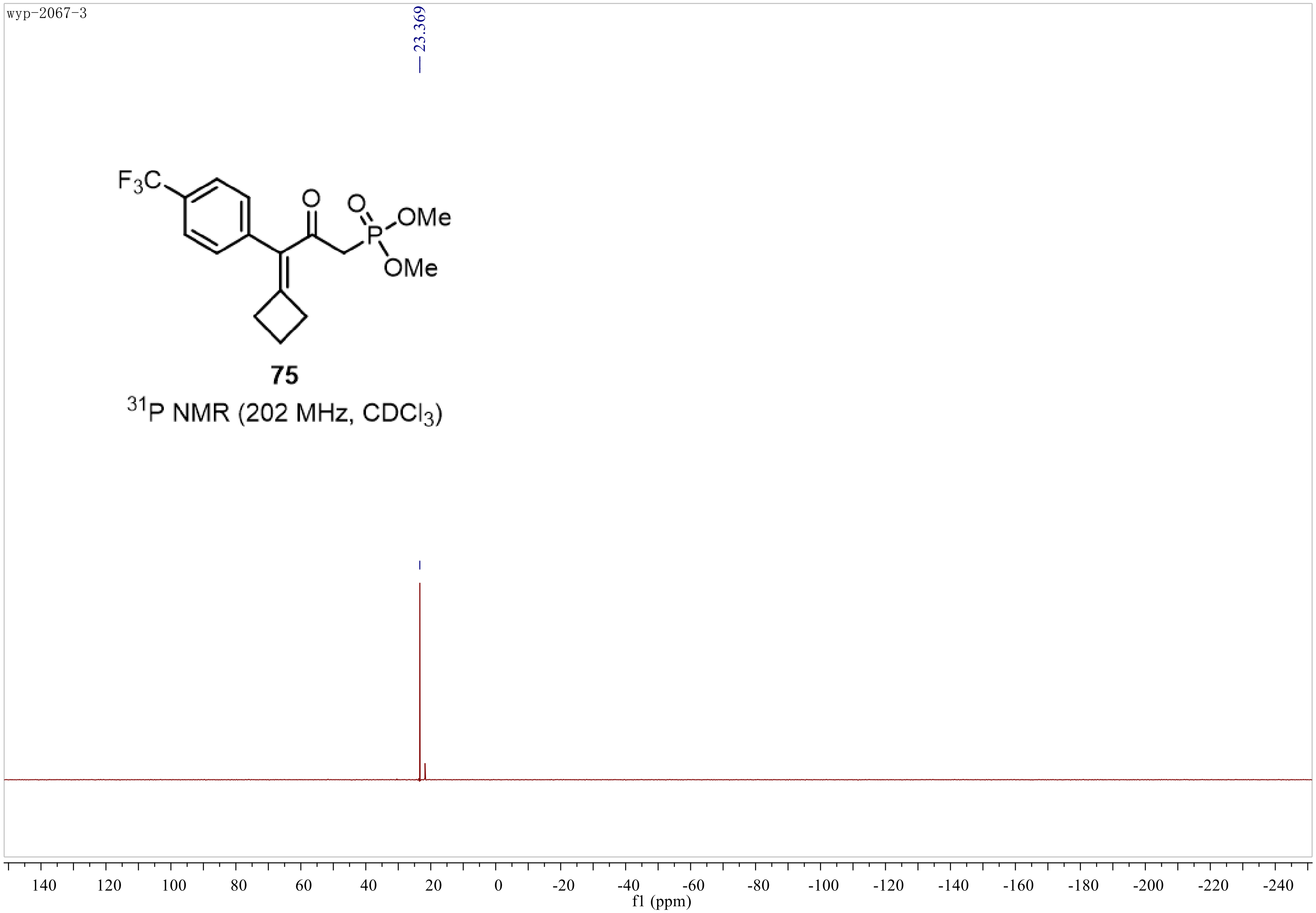

Supplementary Fig. 92. ^{31}P NMR spectra of compound **75** in $CDCl_3$

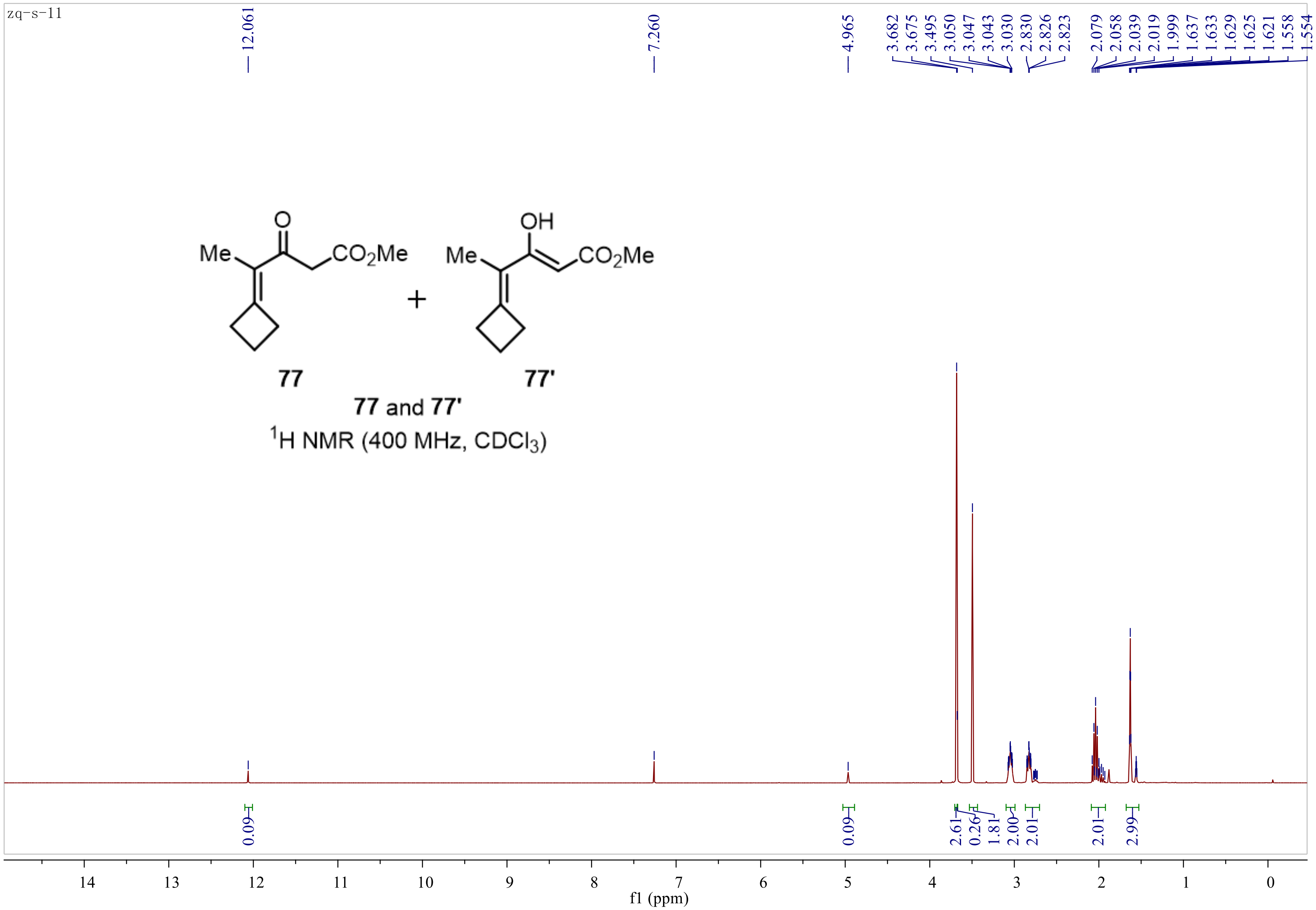

Supplementary Fig. 93. ^{1}H NMR spectra of compound **77** and **77'** in $CDCl_3$

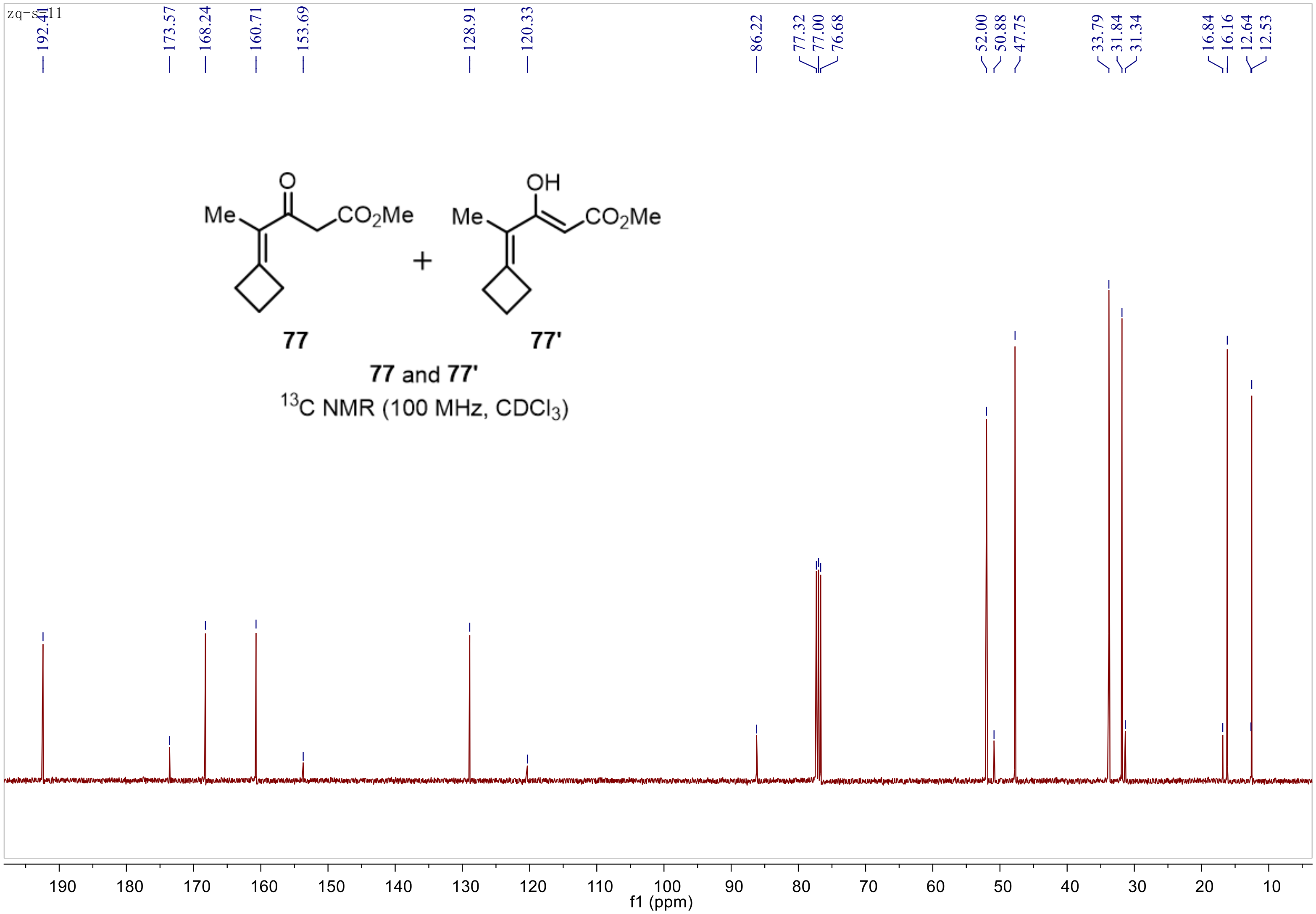

Supplementary Fig. 94. ^{13}C NMR spectra of compound **77** and **77'** in $CDCl_3$

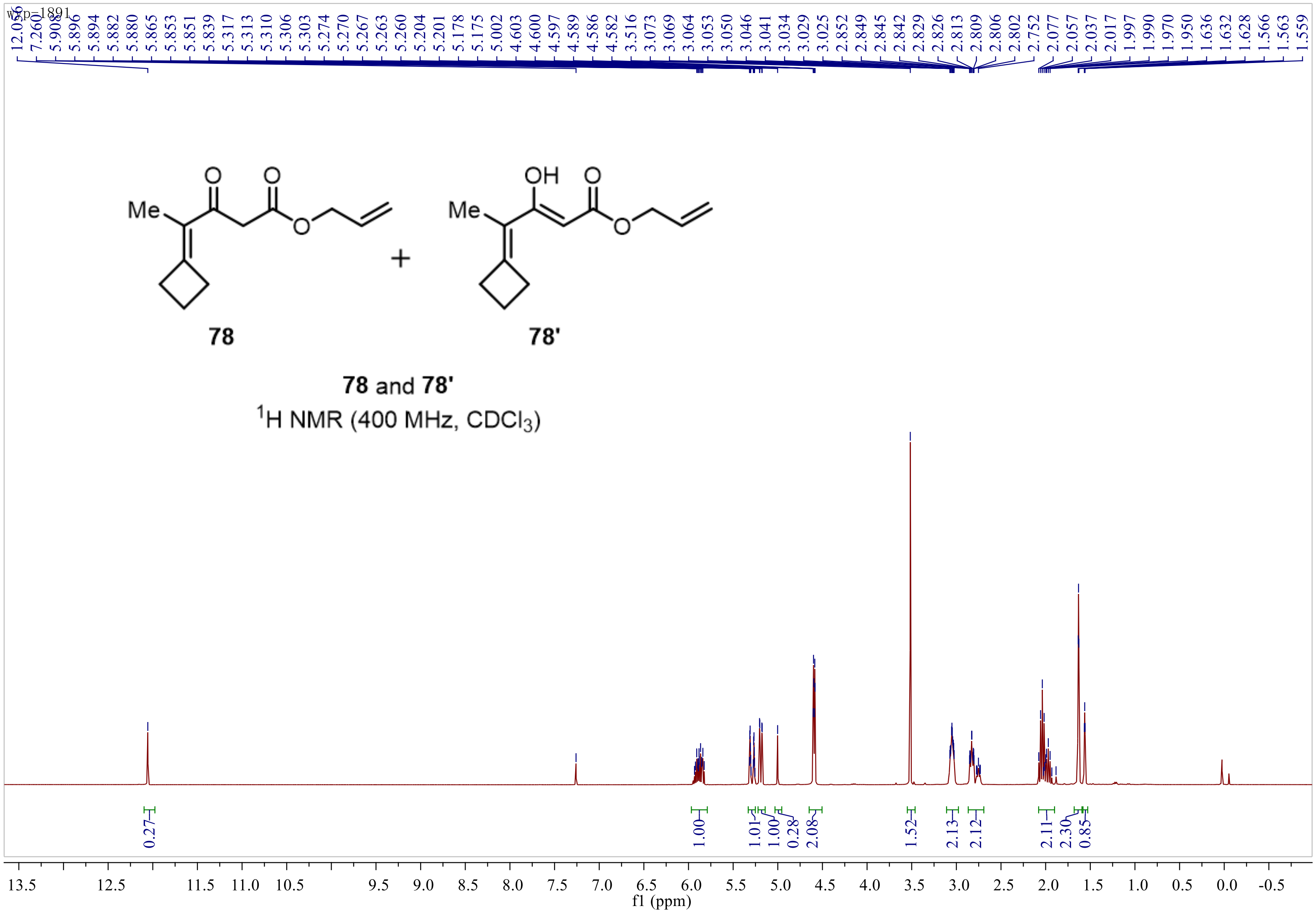

Supplementary Fig. 95. ^{1}H NMR spectra of compound **78** and **78'** in $CDCl_3$

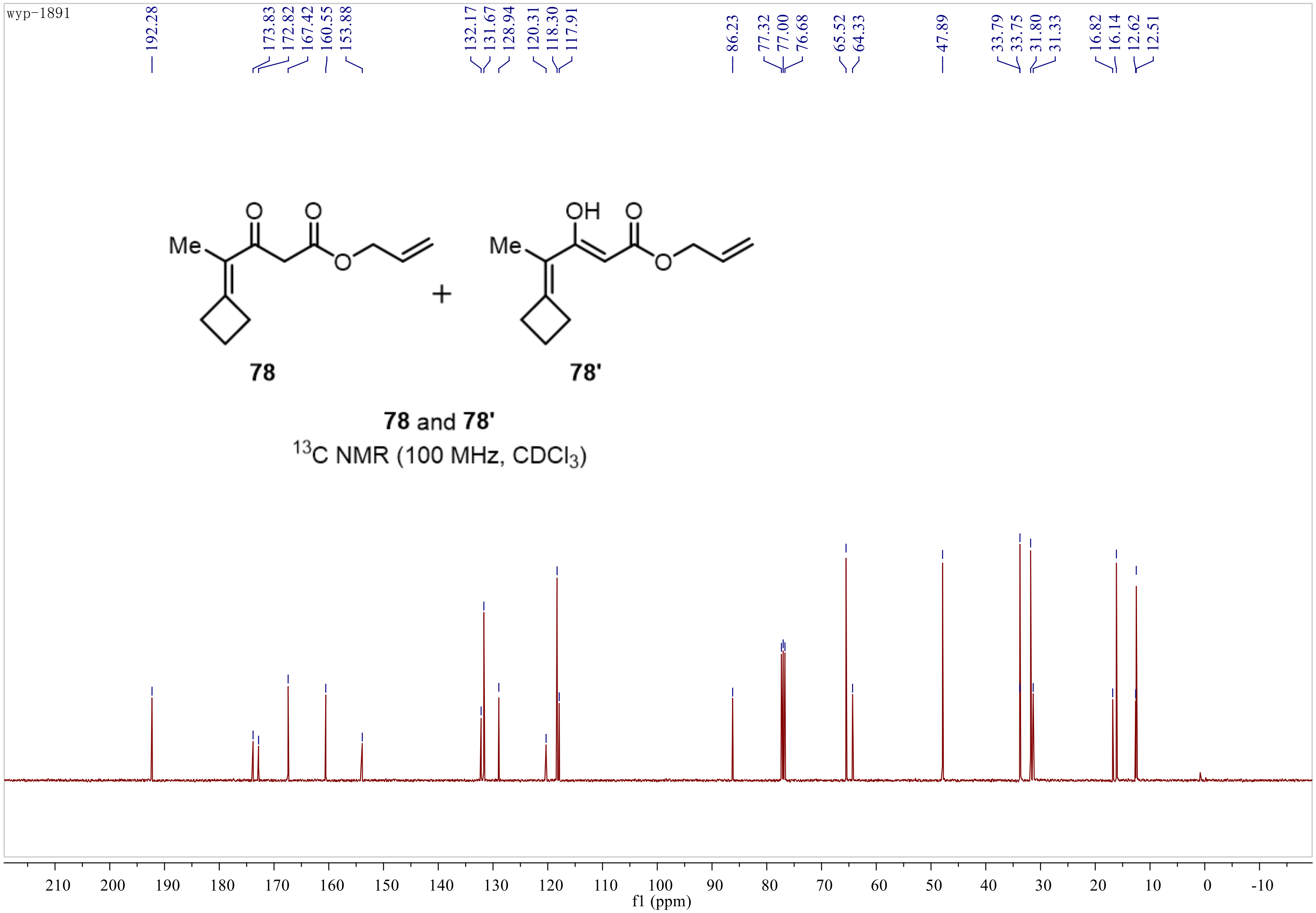

Supplementary Fig. 96. ^{13}C NMR spectra of compound **78** and **78'** in $CDCl_3$

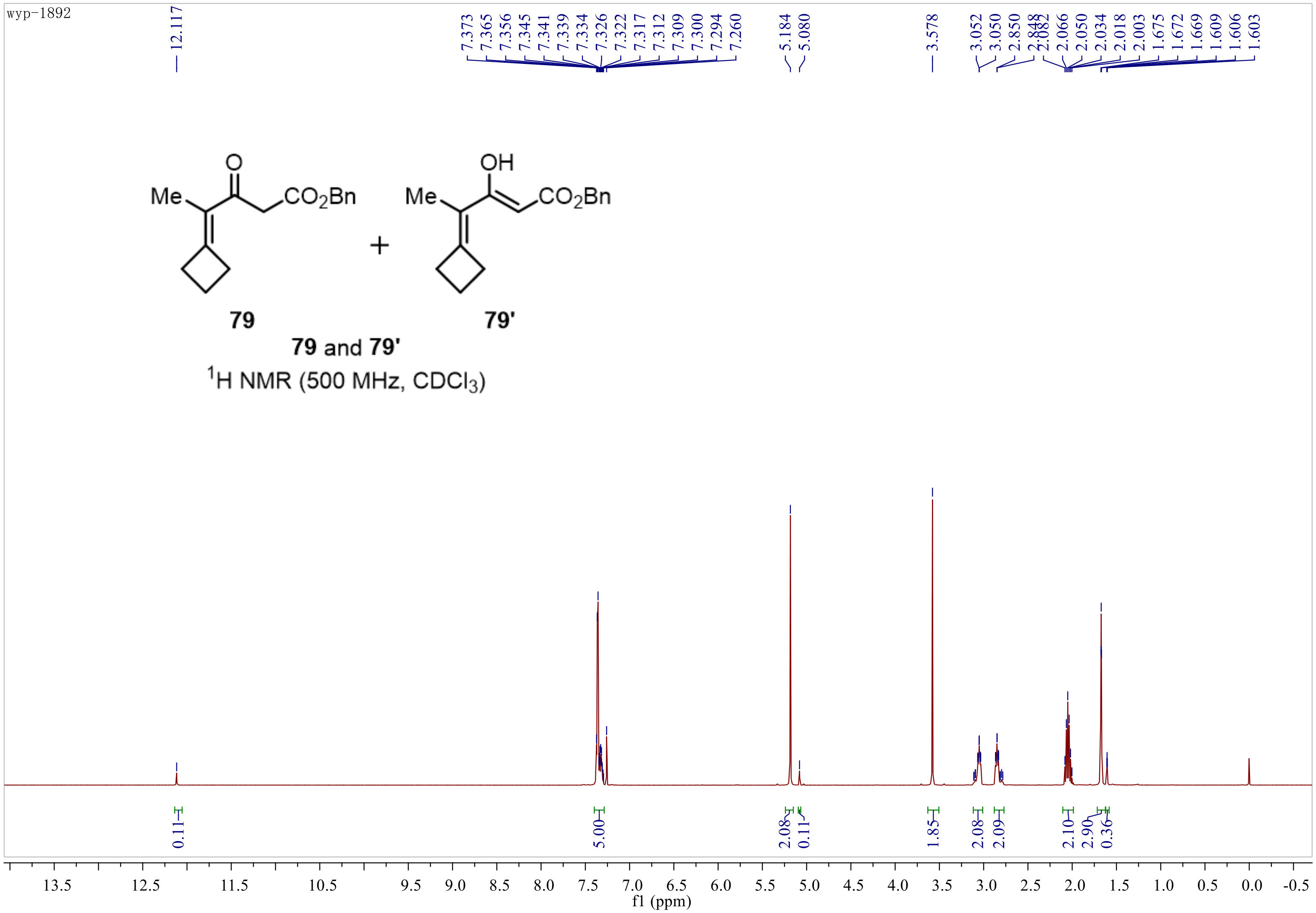

Supplementary Fig. 97. ^{1}H NMR spectra of compound **79** and **79'** in $CDCl_3$

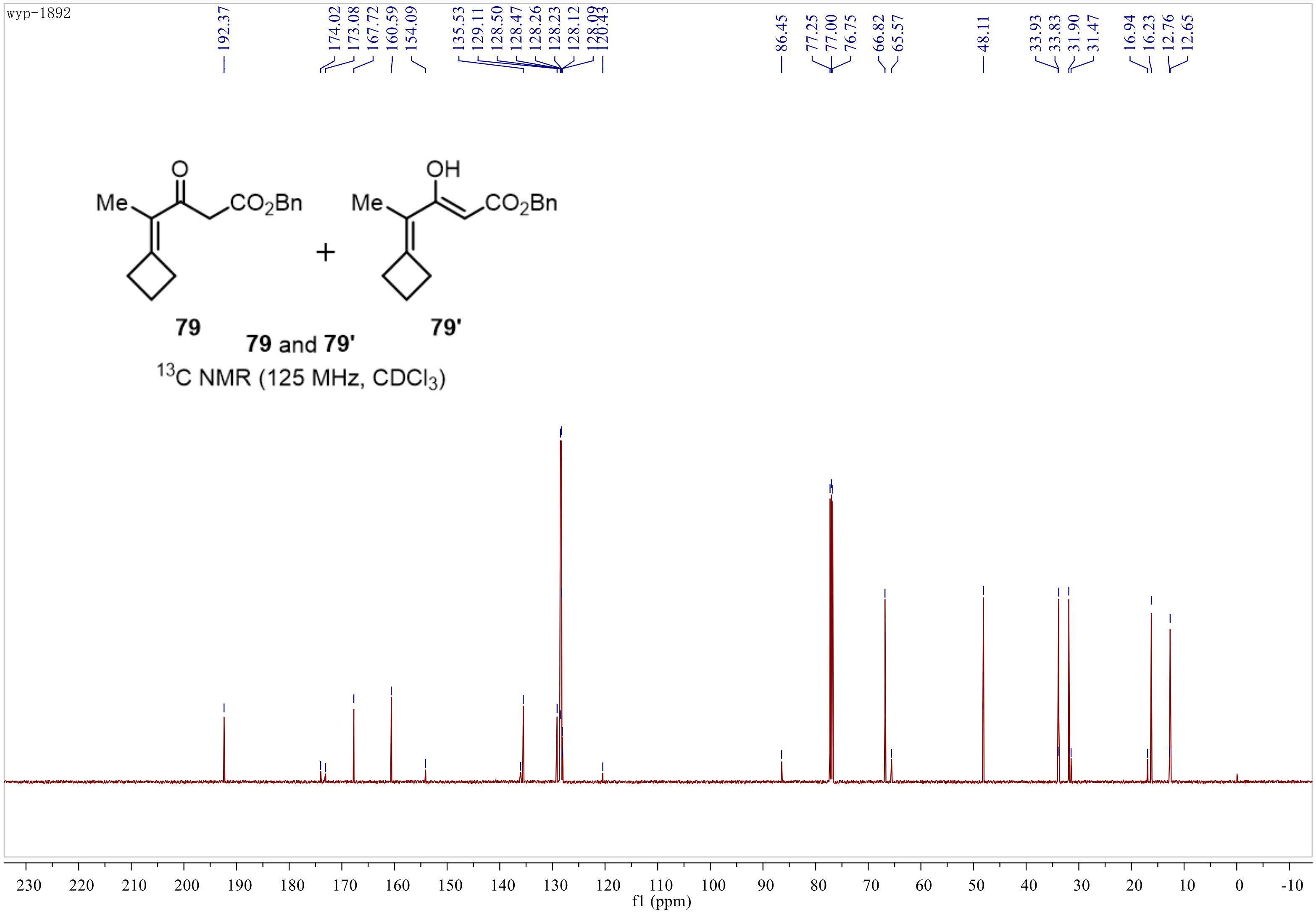

Supplementary Fig. 98. ^{13}C NMR spectra of compound **79** and **79'** in $CDCl_3$

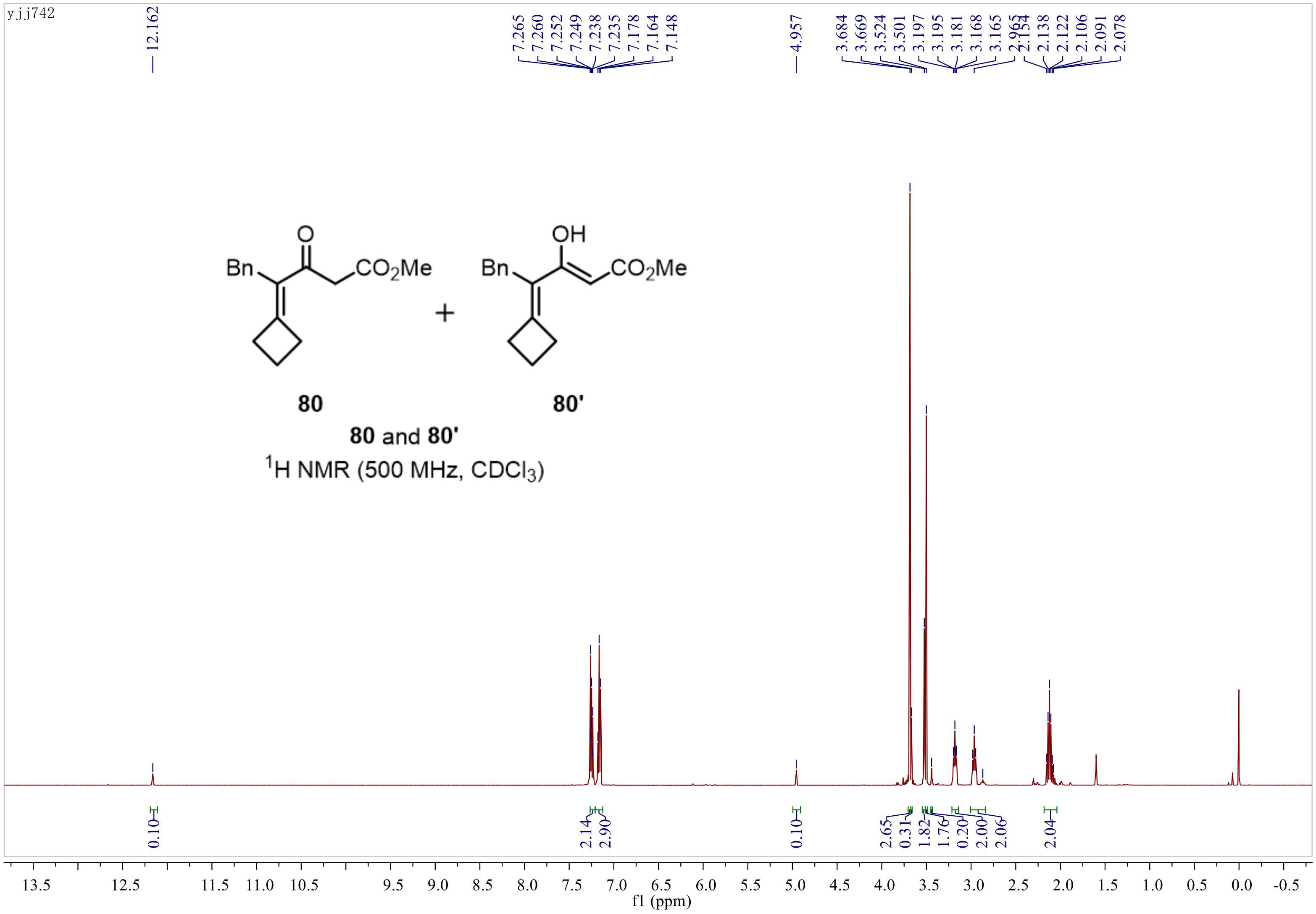

Supplementary Fig. 99. ^{1}H NMR spectra of compound **80** and **80'** in $CDCl_3$

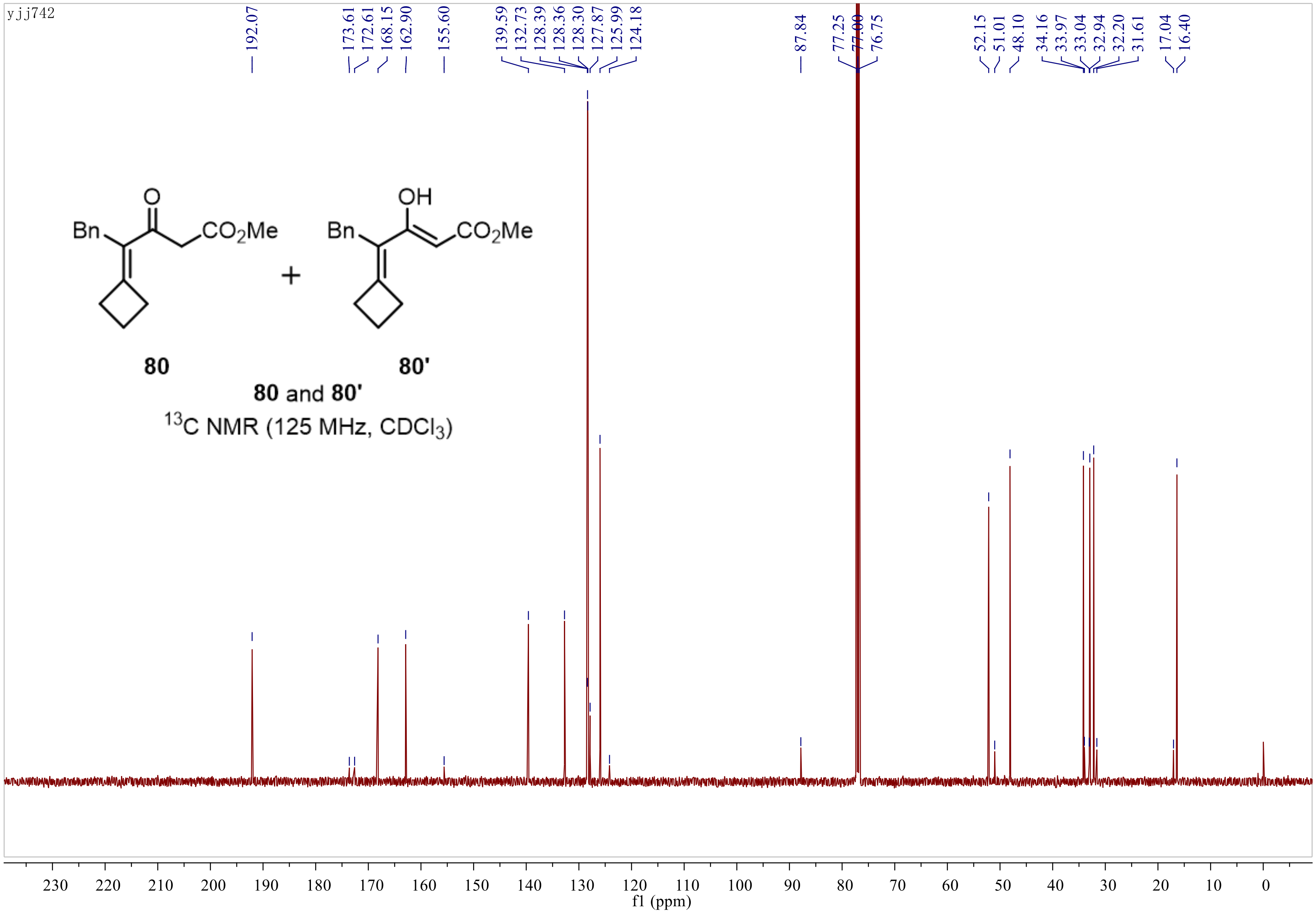

Supplementary Fig. 100. ^{13}C NMR spectra of compound **80** and **80'** in $CDCl_3$

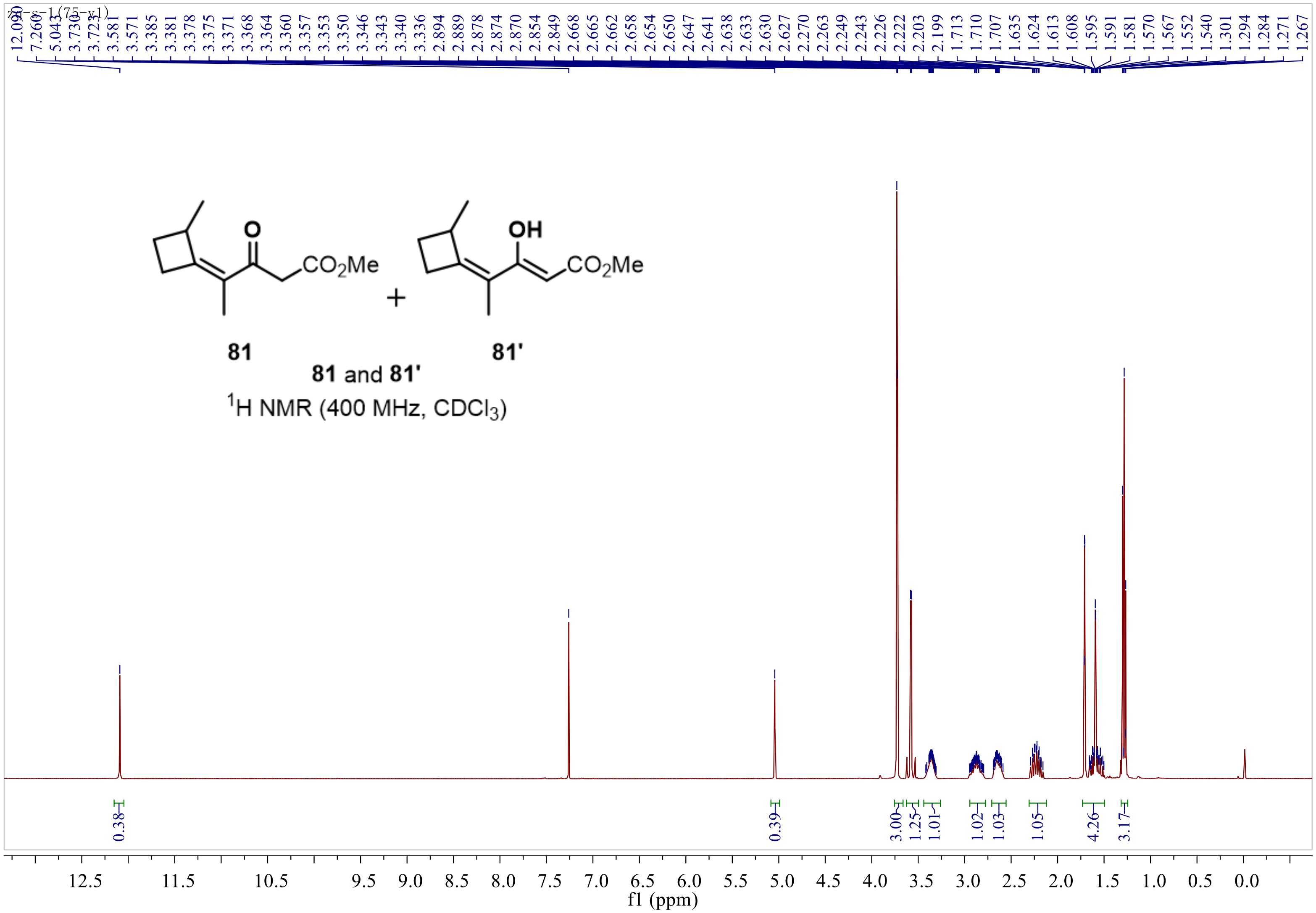

Supplementary Fig. 101. ^{1}H NMR spectra of compound **81** and **81'** in $CDCl_3$

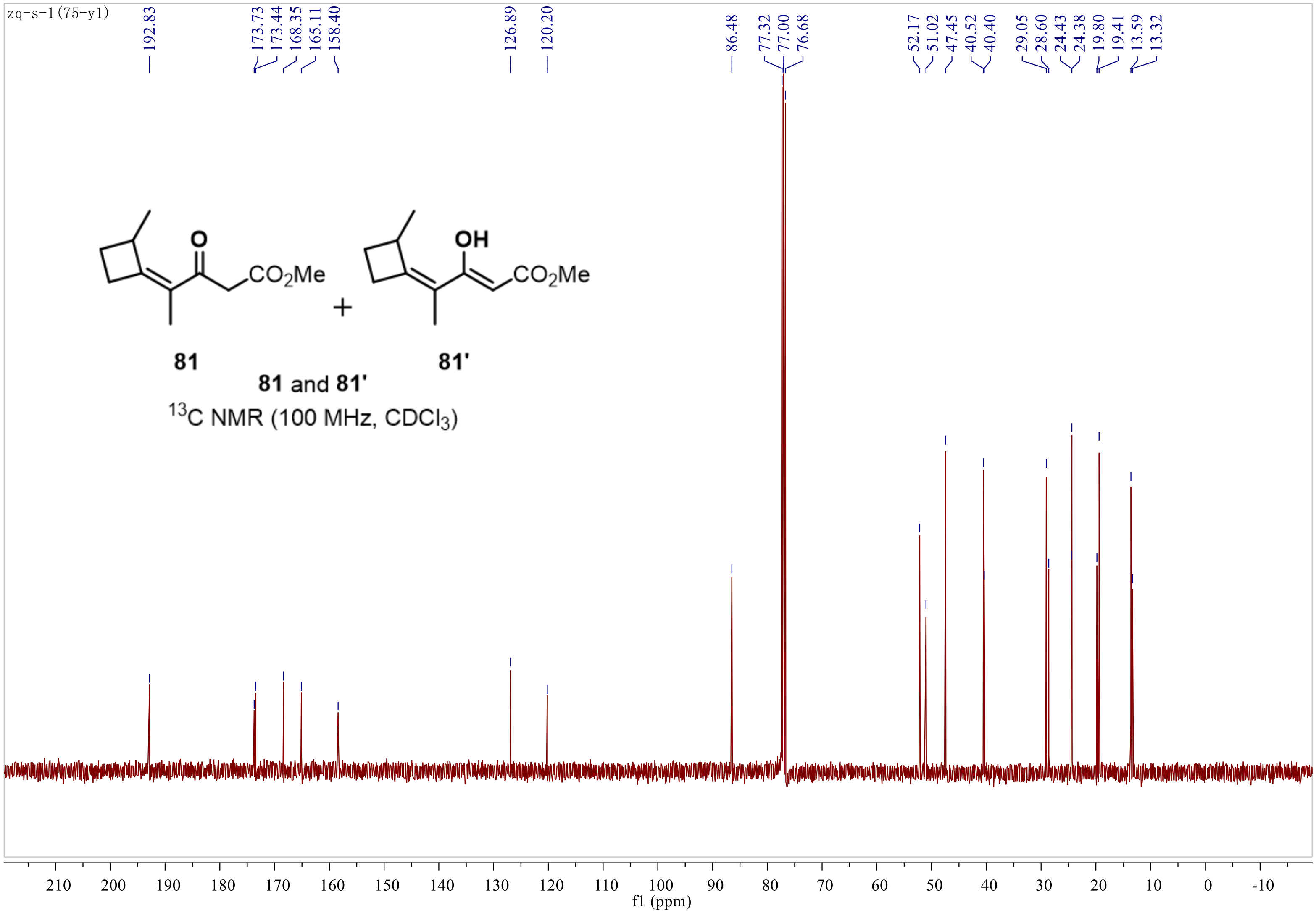

Supplementary Fig. 102. ^{13}C NMR spectra of compound **81** and **81'** in $CDCl_3$

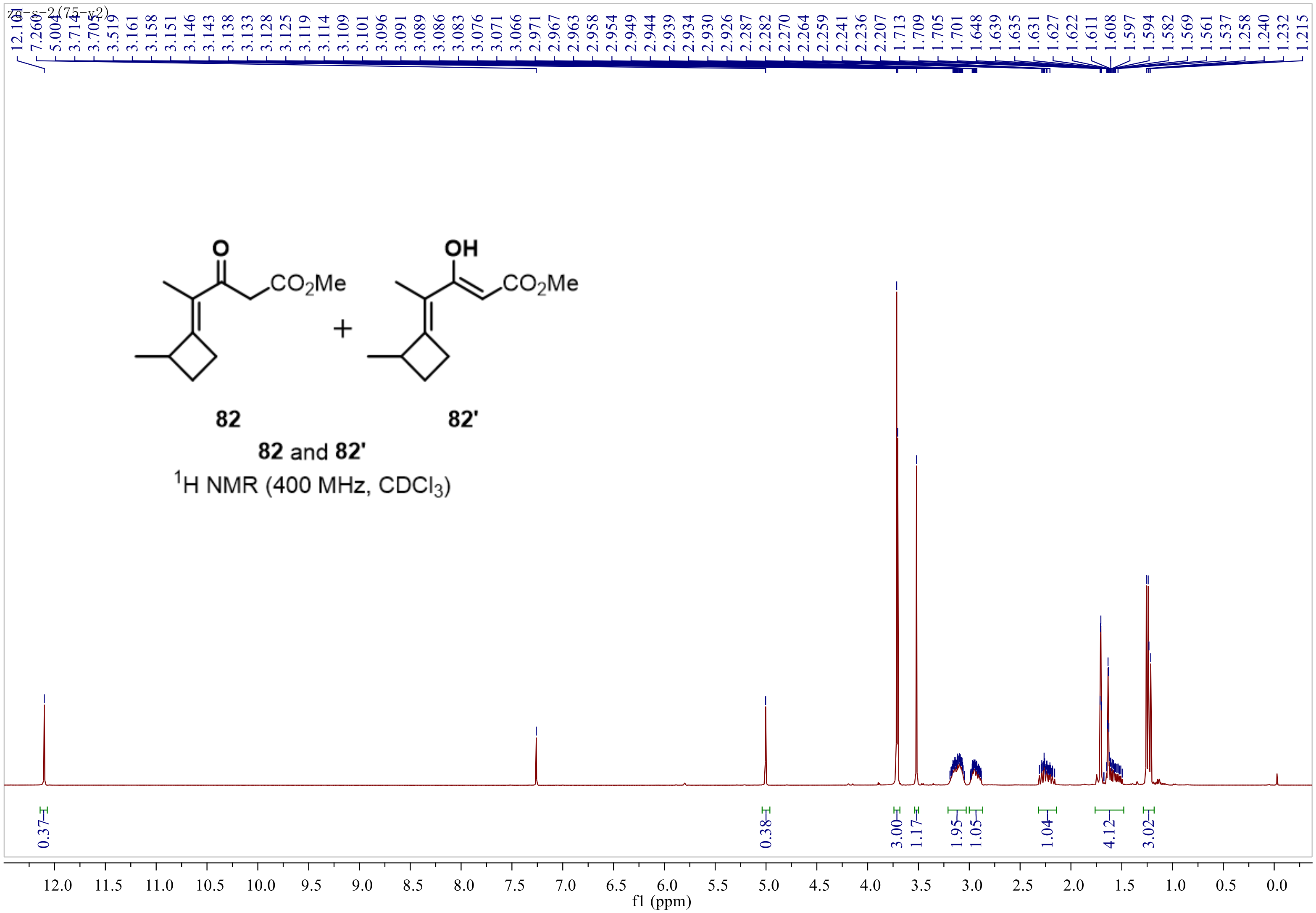

Supplementary Fig. 103. ^{1}H NMR spectra of compound **82** and **82'** in $CDCl_3$

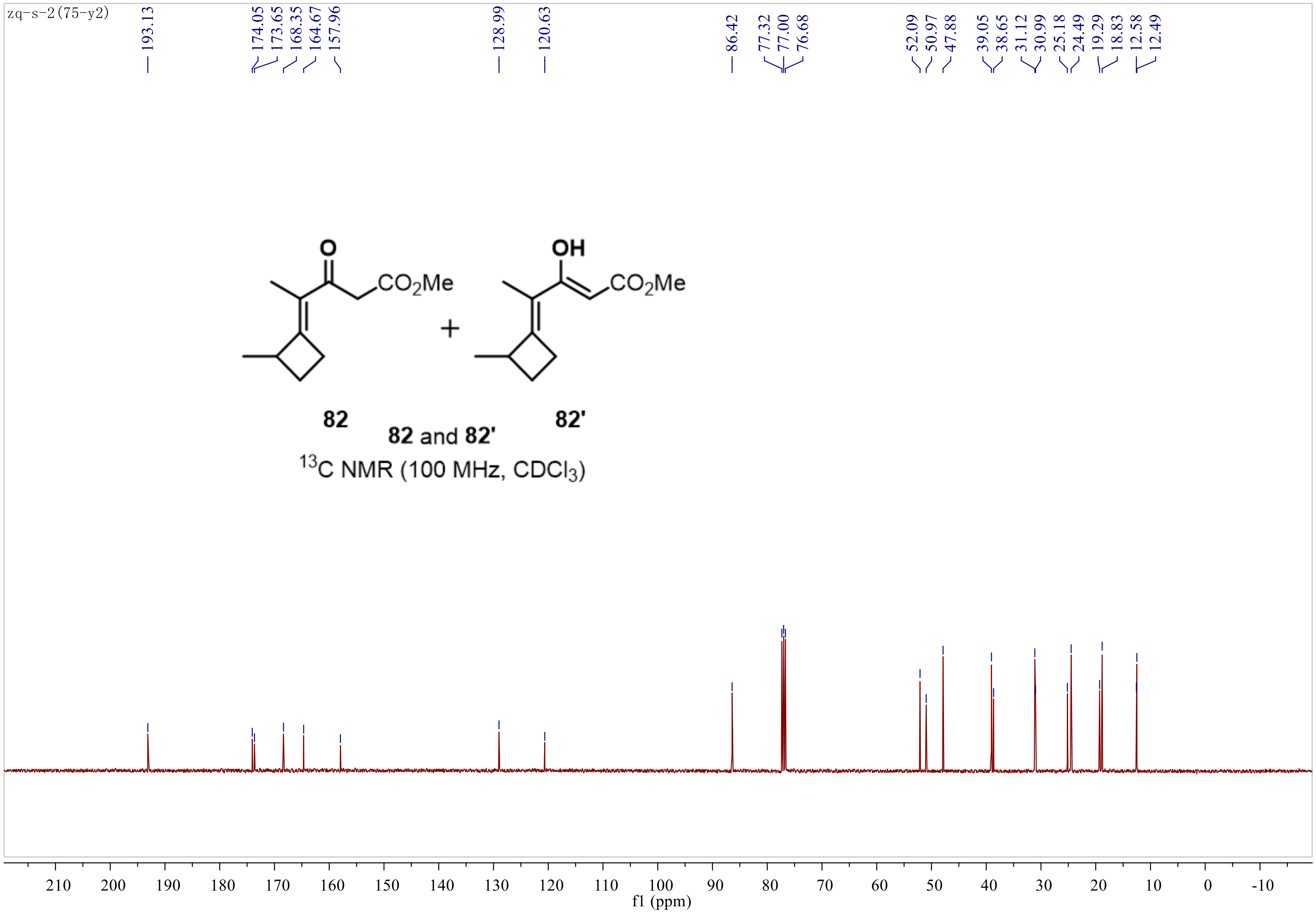

Supplementary Fig. 104. ^{13}C NMR spectra of compound **82** and **82'** in $CDCl_3$

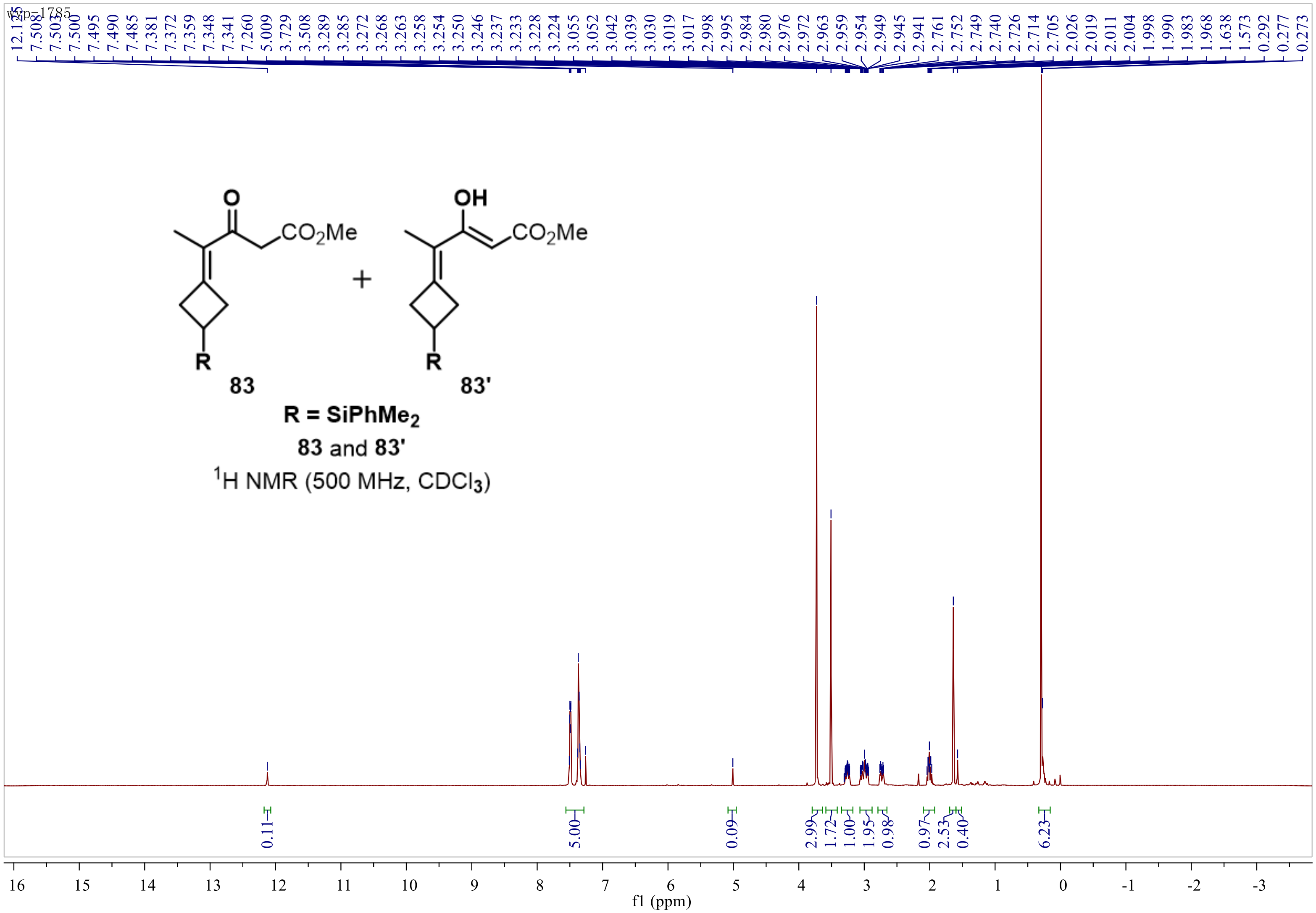

Supplementary Fig. 105. ^{1}H NMR spectra of compound **83** and **83'** in $CDCl_3$

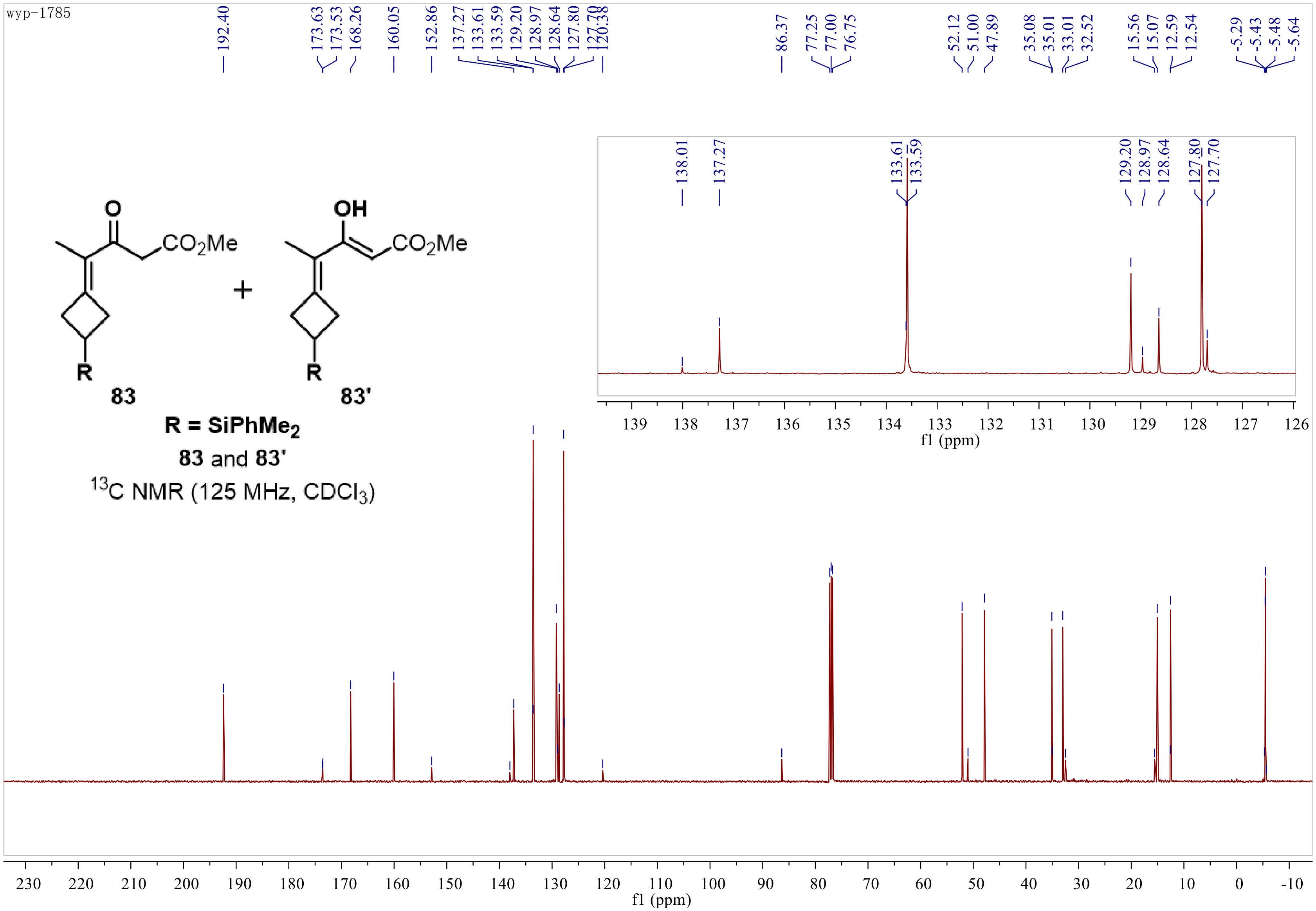

Supplementary Fig. 106. ^{13}C NMR spectra of compound **83** and **83'** in $CDCl_3$

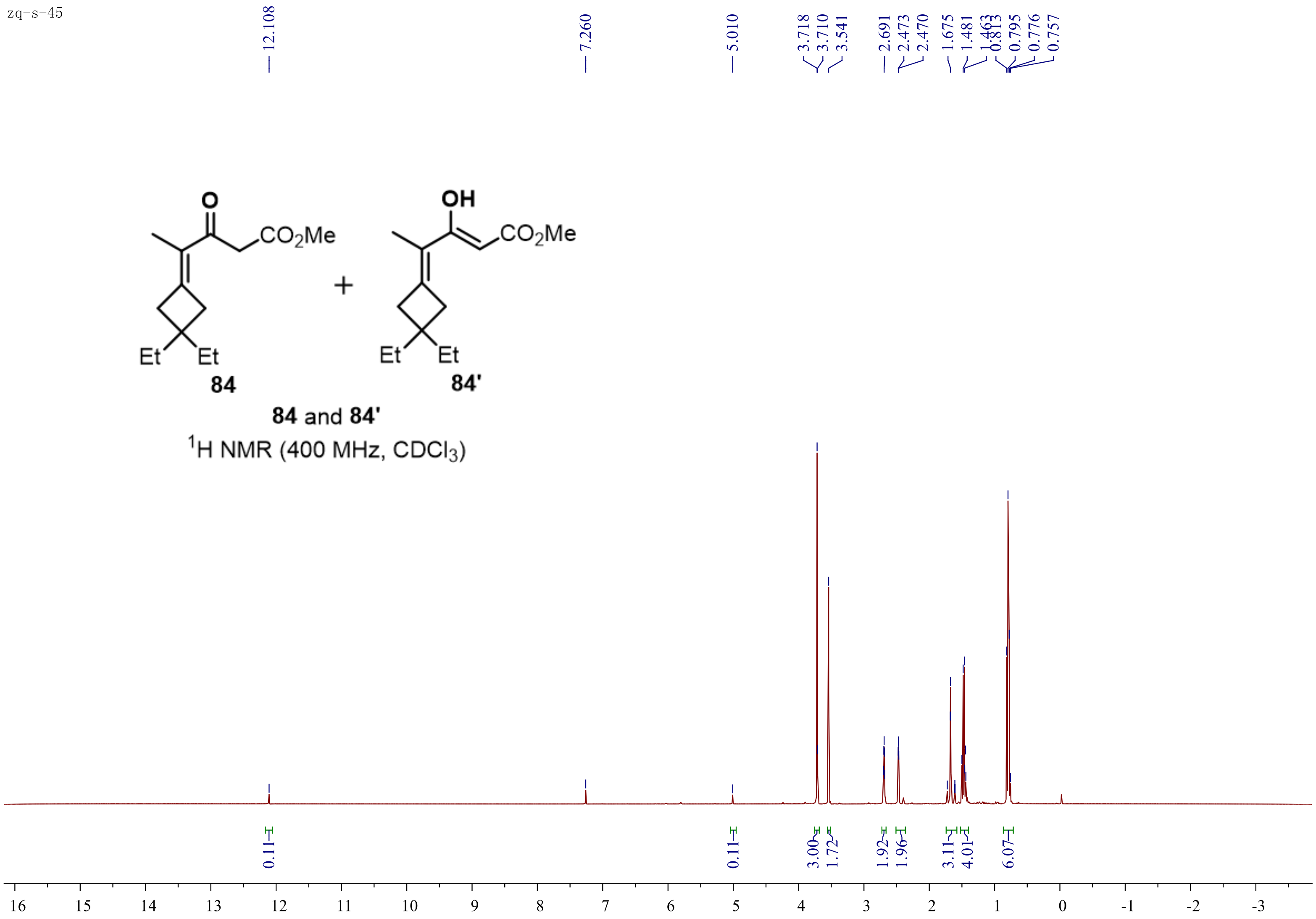

Supplementary Fig. 107. ^{1}H NMR spectra of compound **84** and **84'** in $CDCl_3$

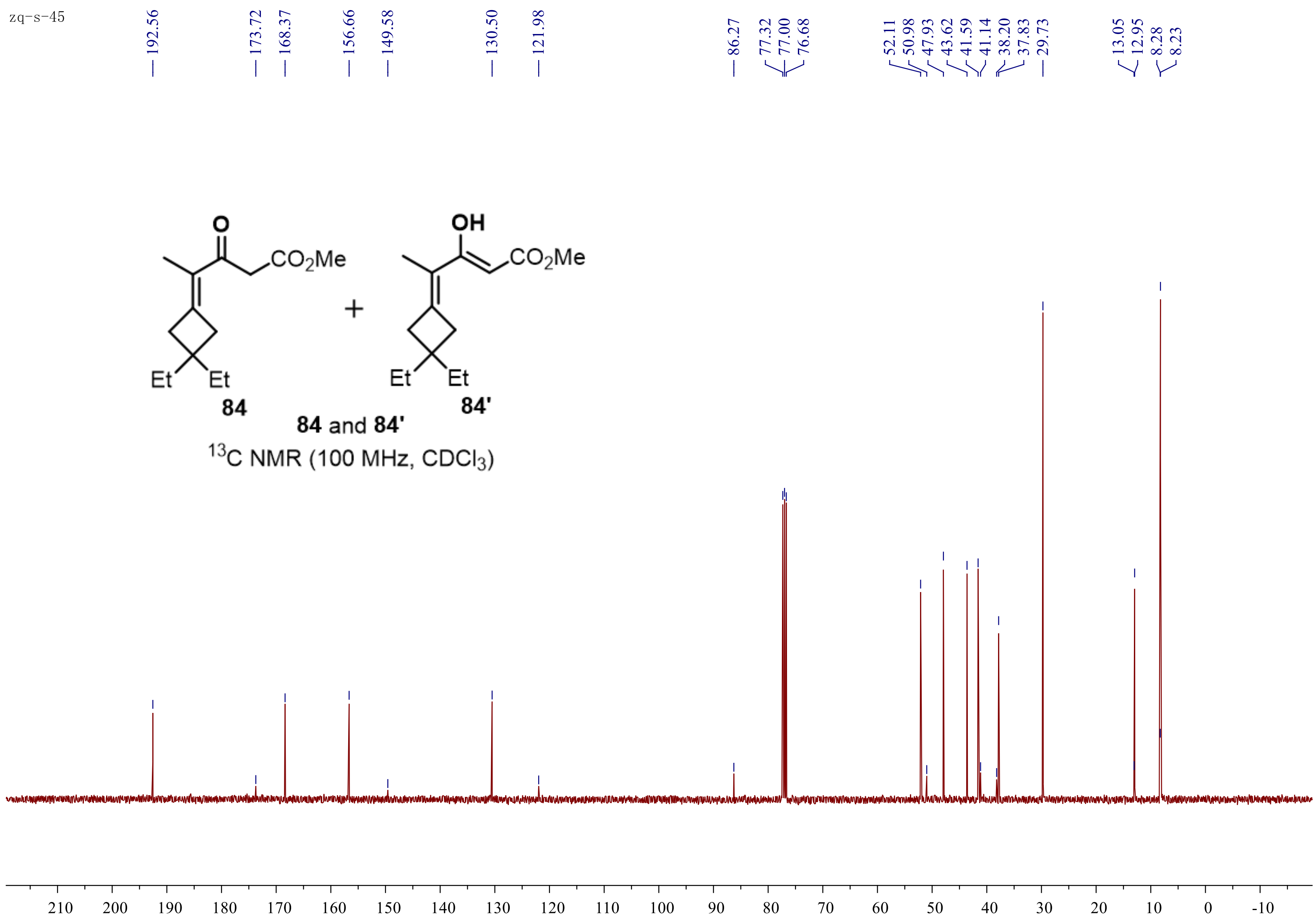

Supplementary Fig. 108. ^{13}C NMR spectra of compound **84** and **84'** in $CDCl_3$

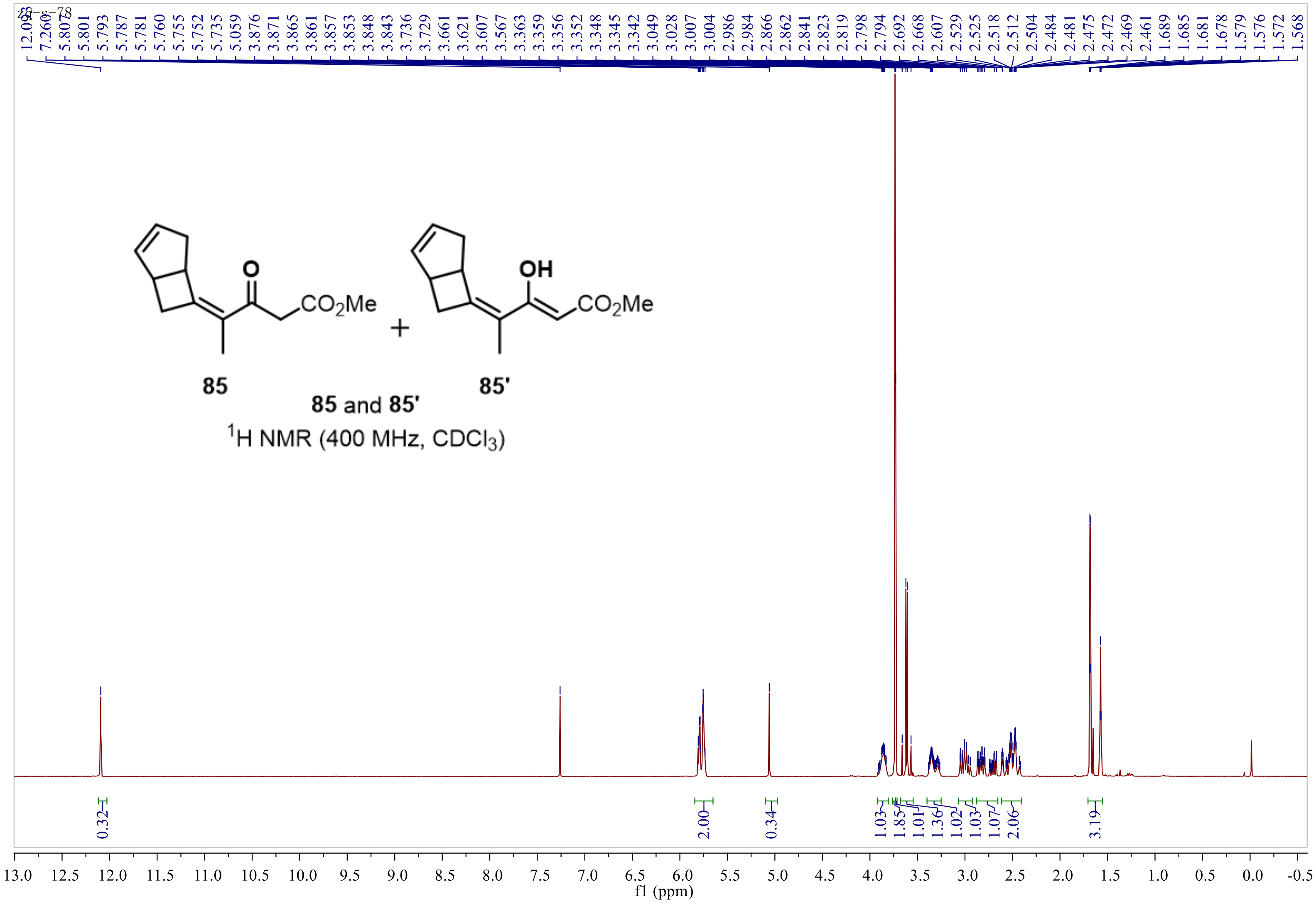

Supplementary Fig. 109. ^{1}H NMR spectra of compound **85** and **85'** in $CDCl_3$

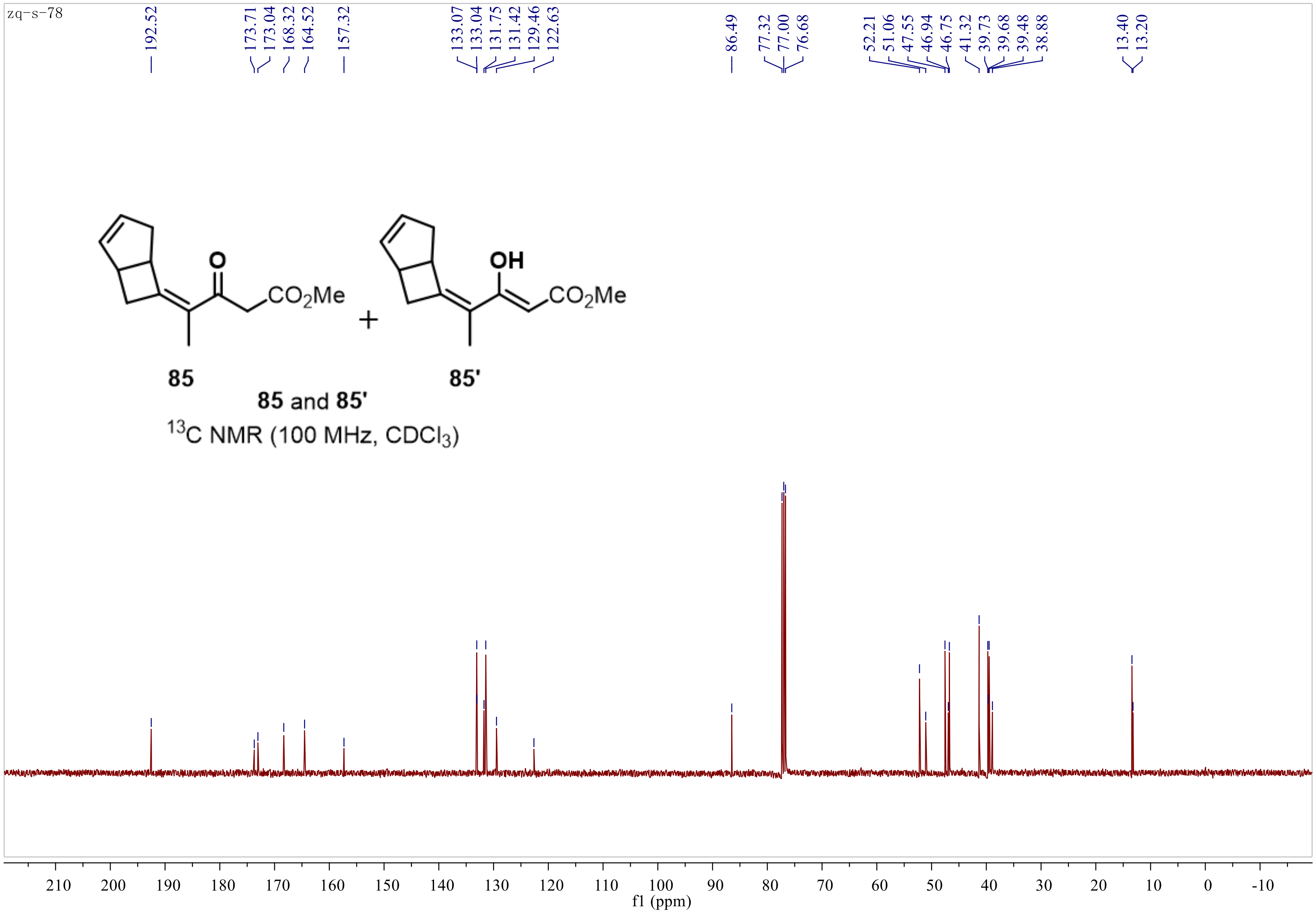

Supplementary Fig. 110. ^{13}C NMR spectra of compound **85** and **85'** in $CDCl_3$

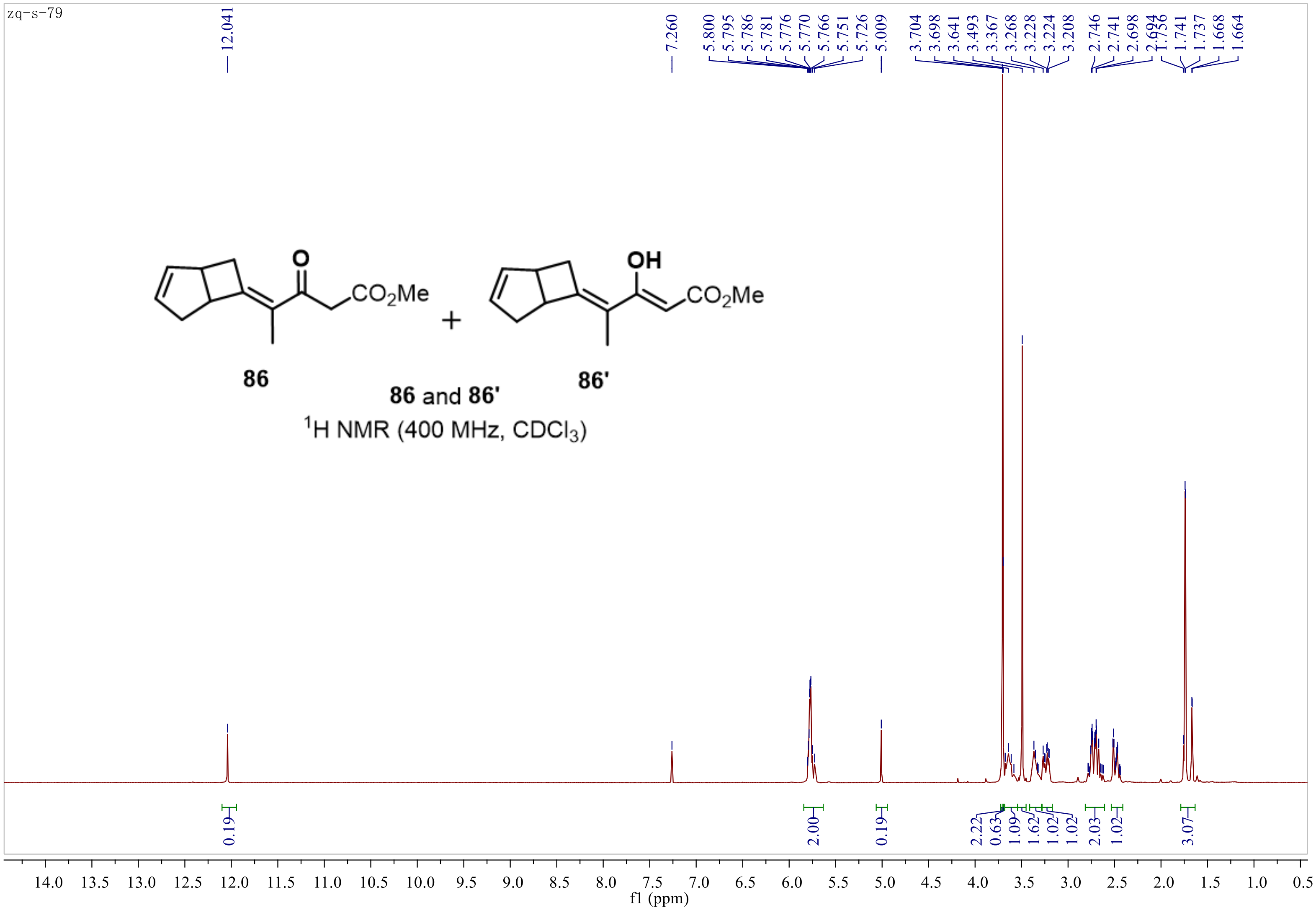

Supplementary Fig. 111. ^{1}H NMR spectra of compound **86** and **86'** in $CDCl_3$

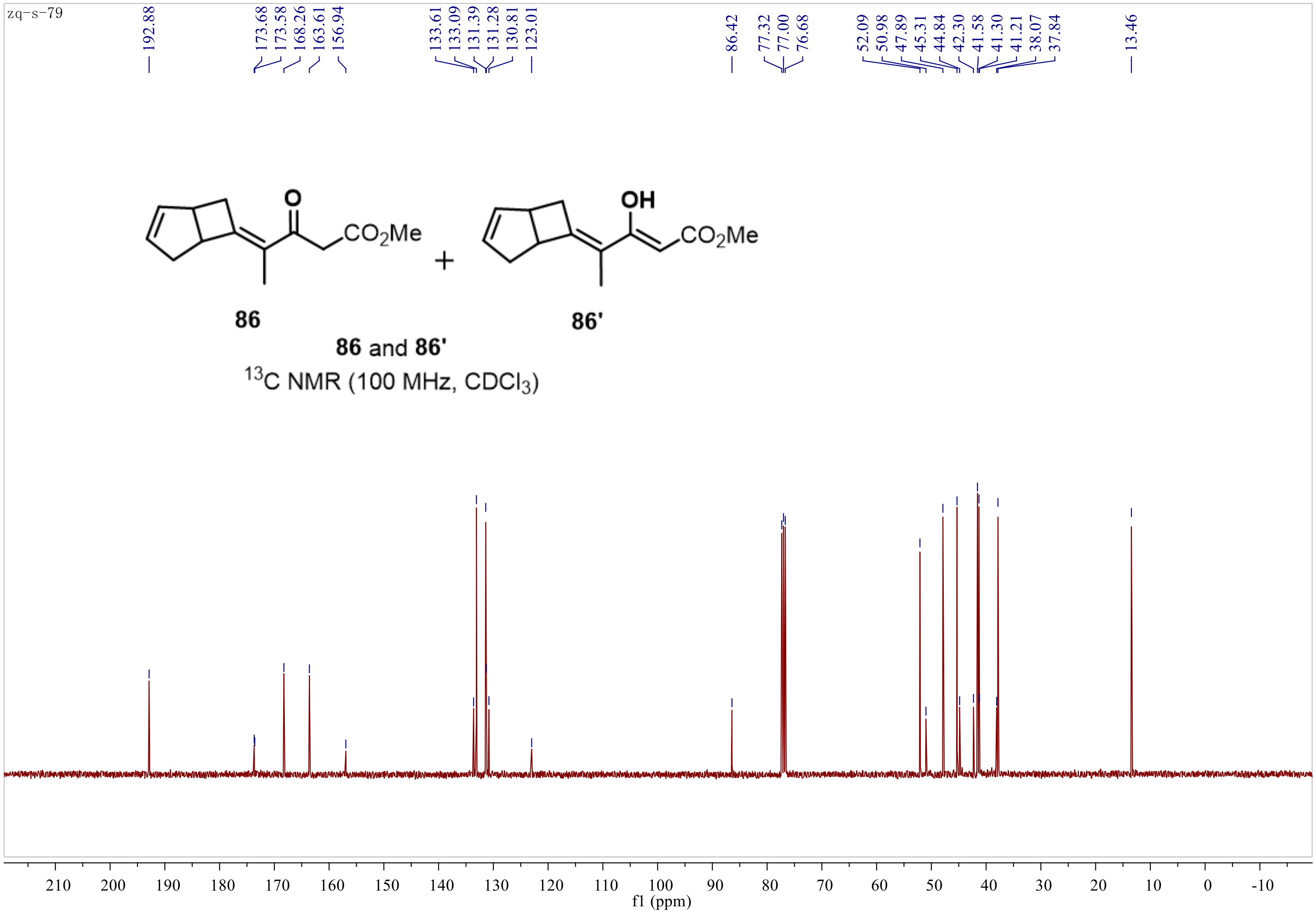

Supplementary Fig. 112. ^{13}C NMR spectra of compound **86** and **86'** in $CDCl_3$

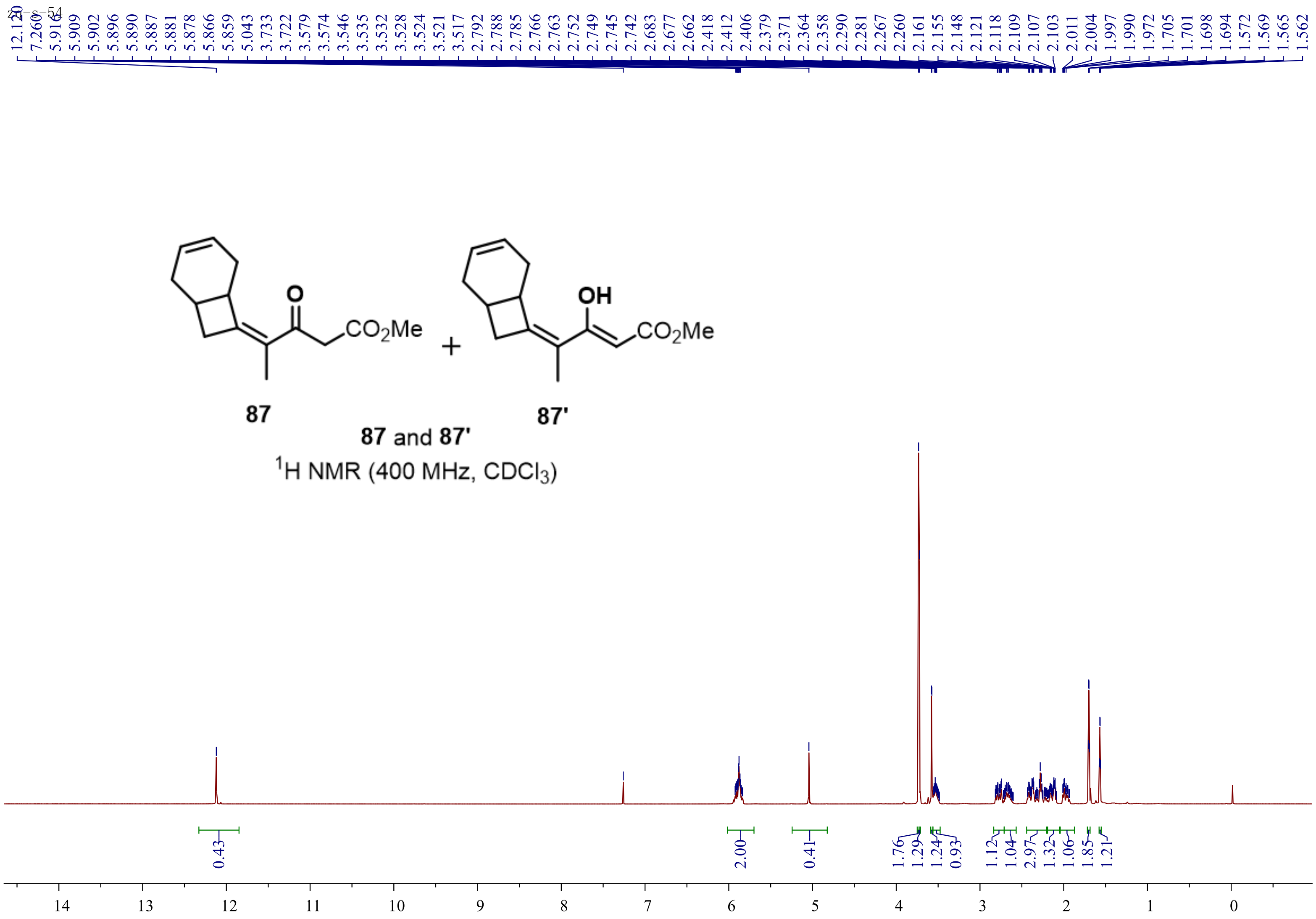

Supplementary Fig. 113. ^{1}H NMR spectra of compound **87** and **87'** in $CDCl_3$

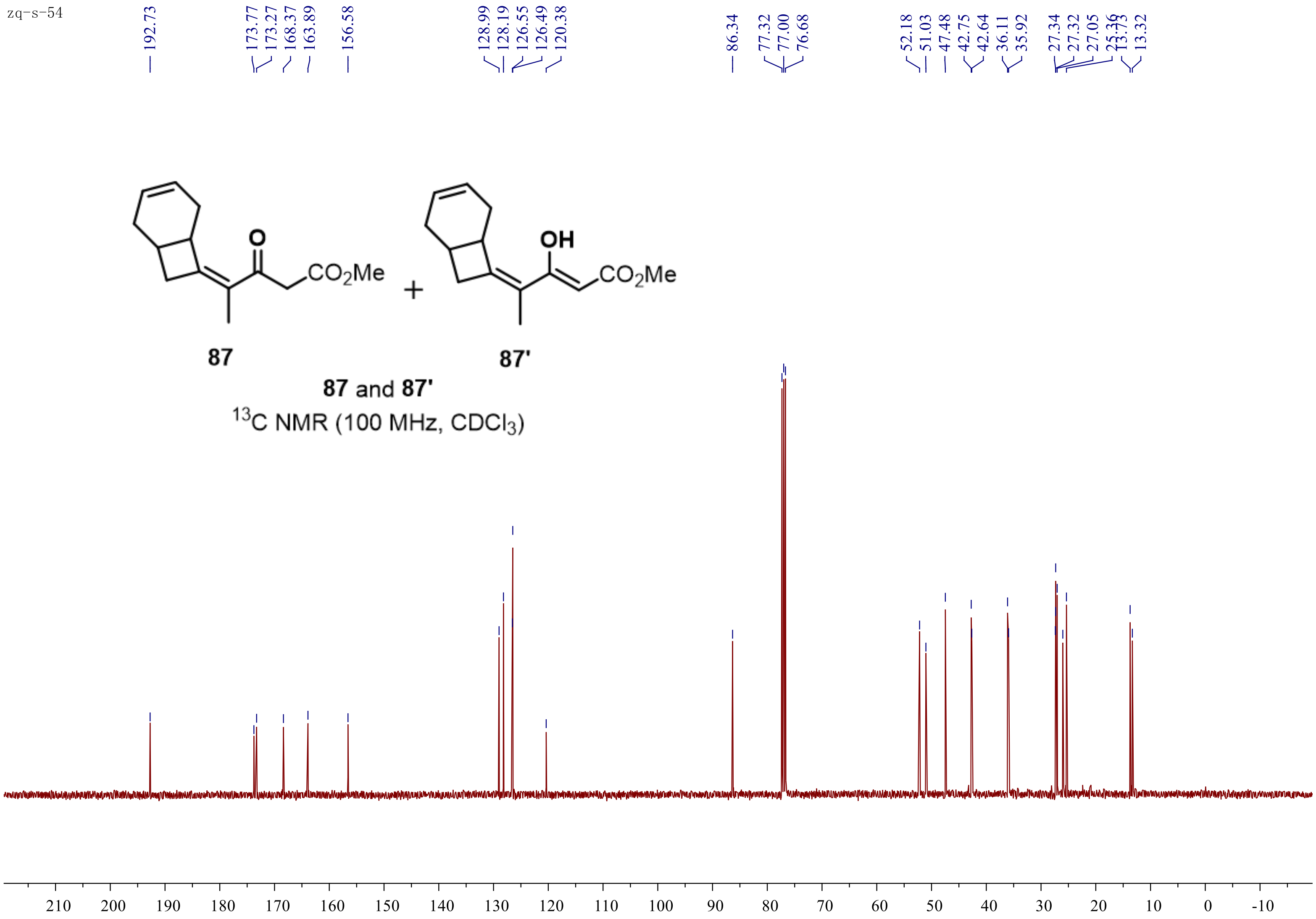

Supplementary Fig. 114. ^{13}C NMR spectra of compound **87** and **87'** in $CDCl_3$

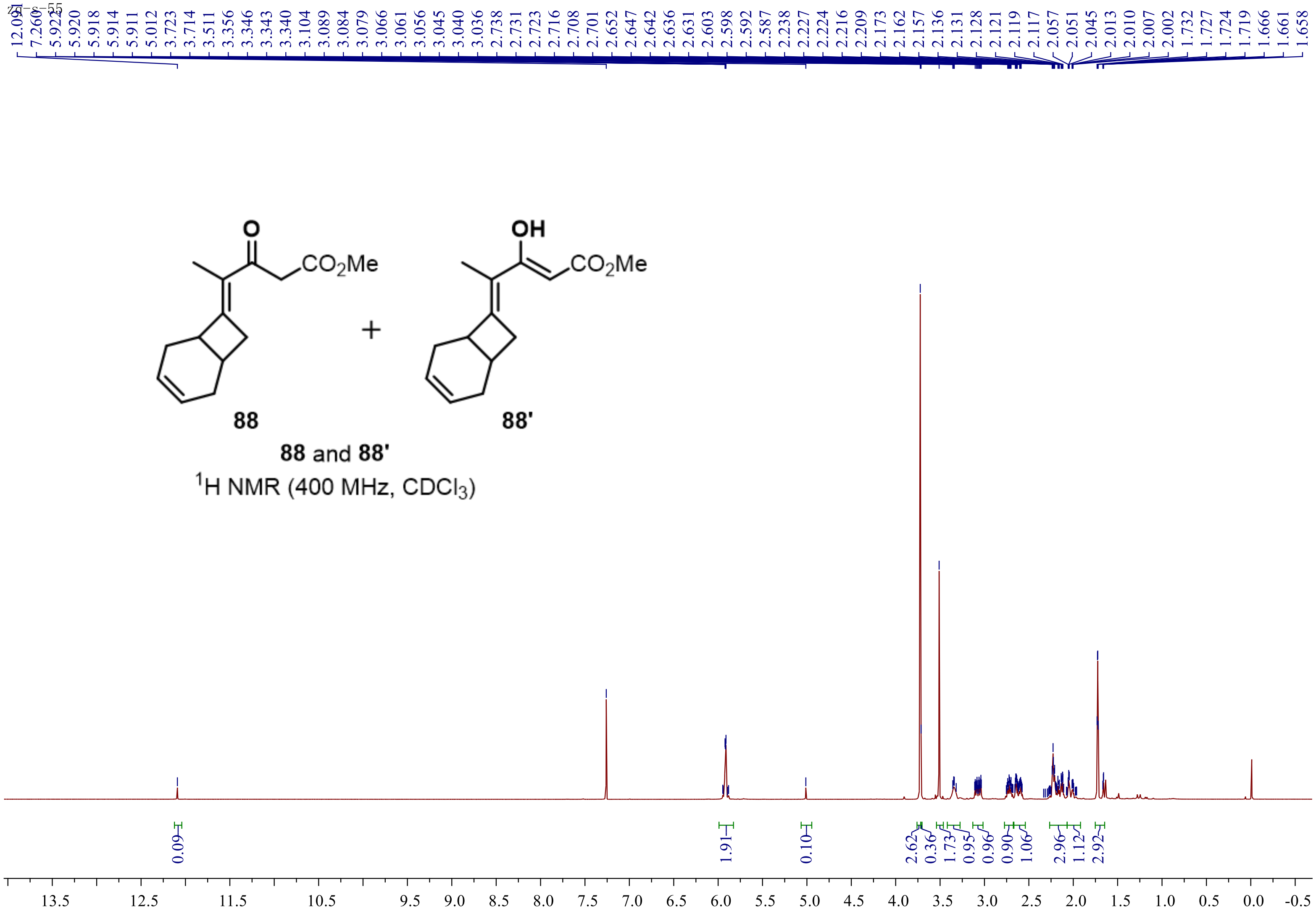

Supplementary Fig. 115. ^{1}H NMR spectra of compound **88** and **88'** in $CDCl_3$

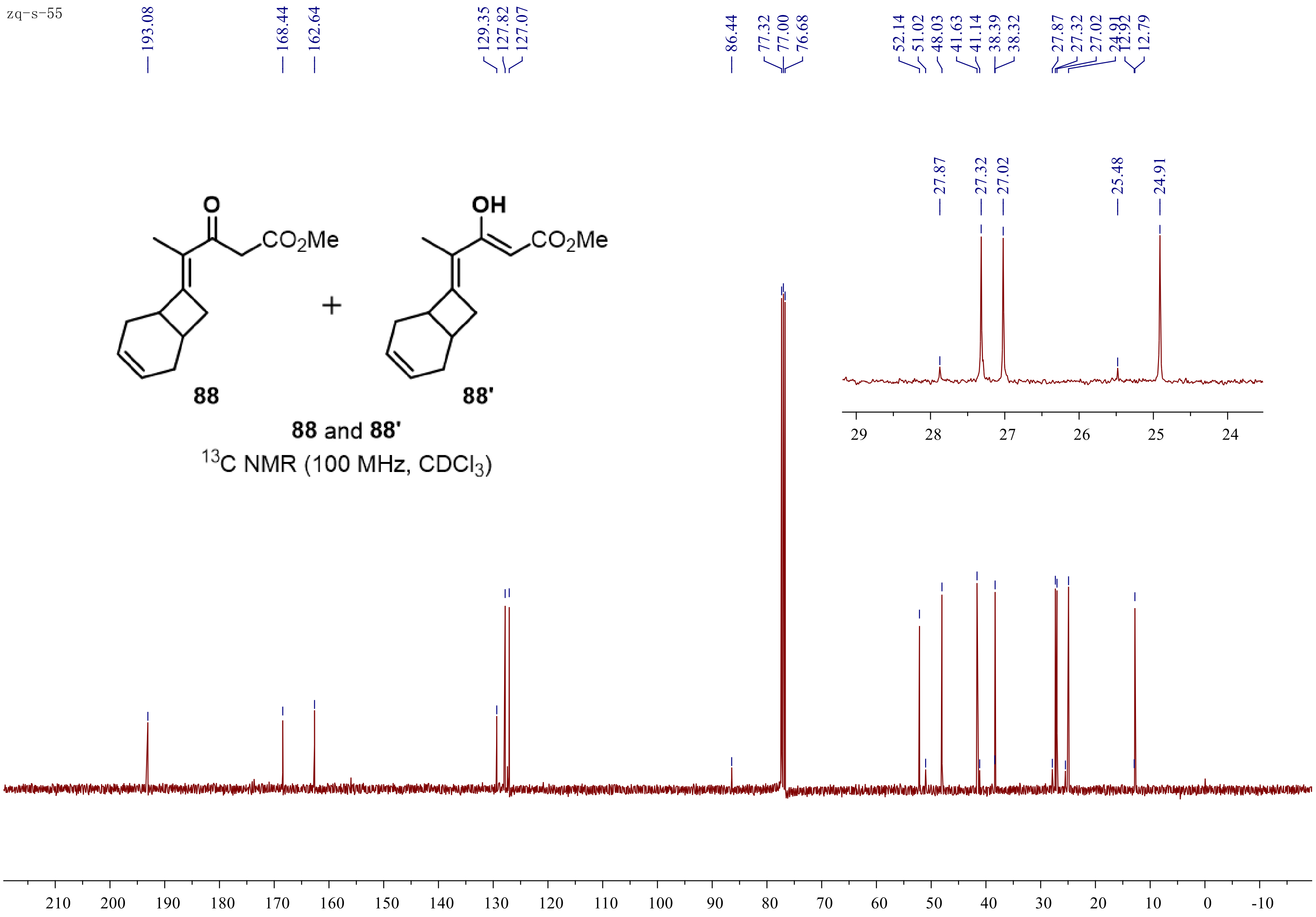

Supplementary Fig. 116. ^{13}C NMR spectra of compound **88** and **88'** in $CDCl_3$

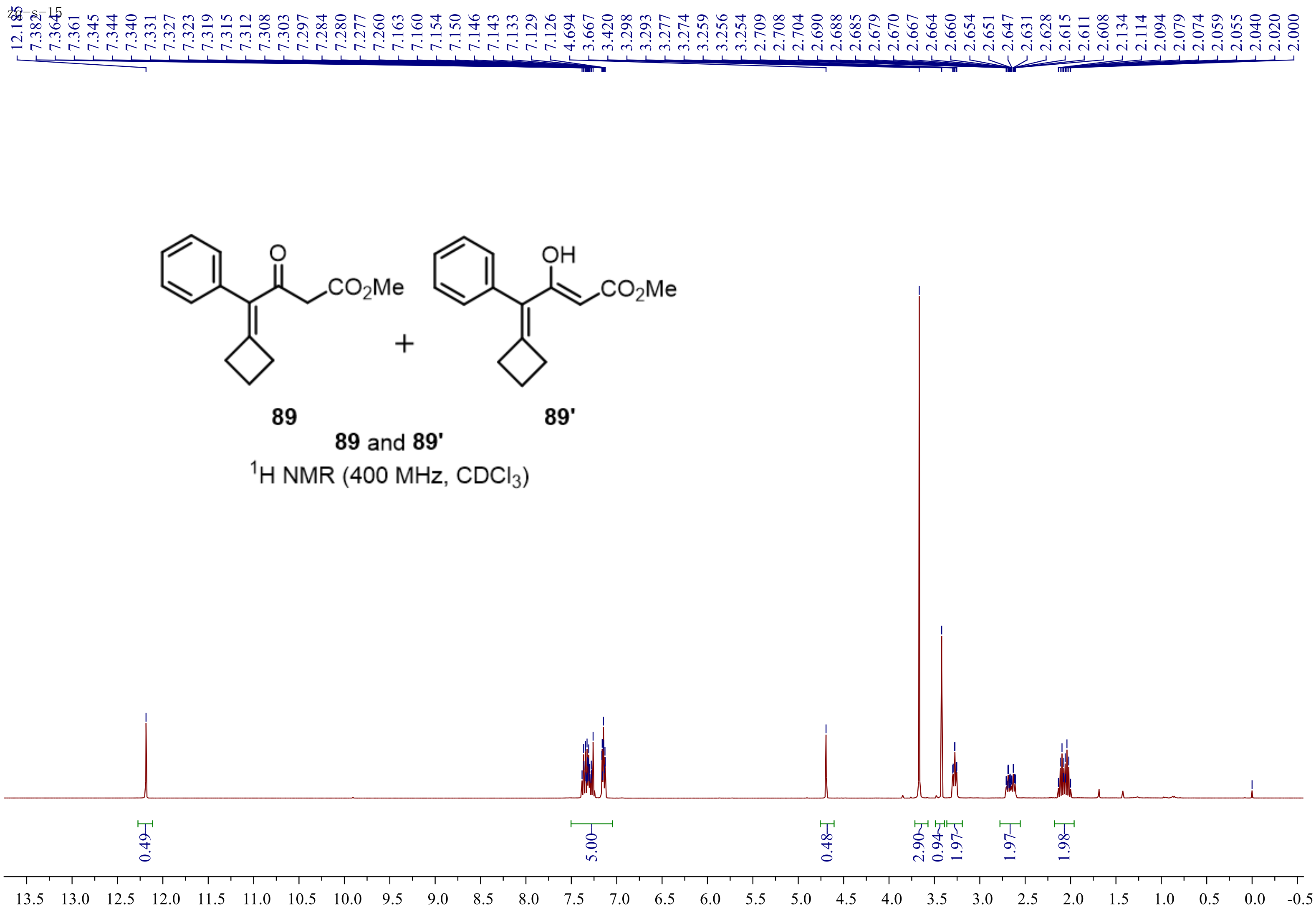

Supplementary Fig. 117. ^{1}H NMR spectra of compound **89** and **89'** in $CDCl_3$

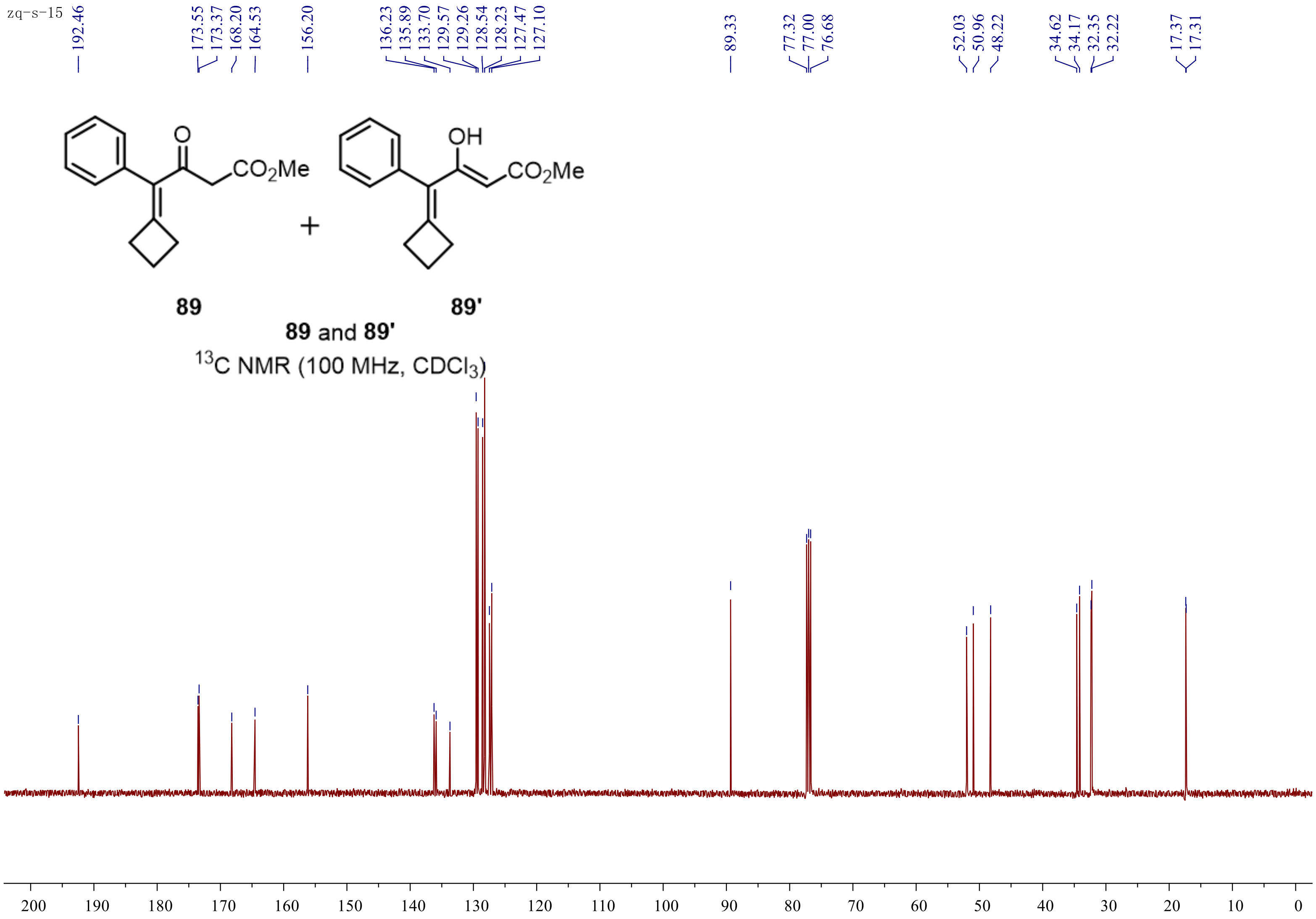

Supplementary Fig. 118. ^{13}C NMR spectra of compound **89** and **89'** in $CDCl_3$

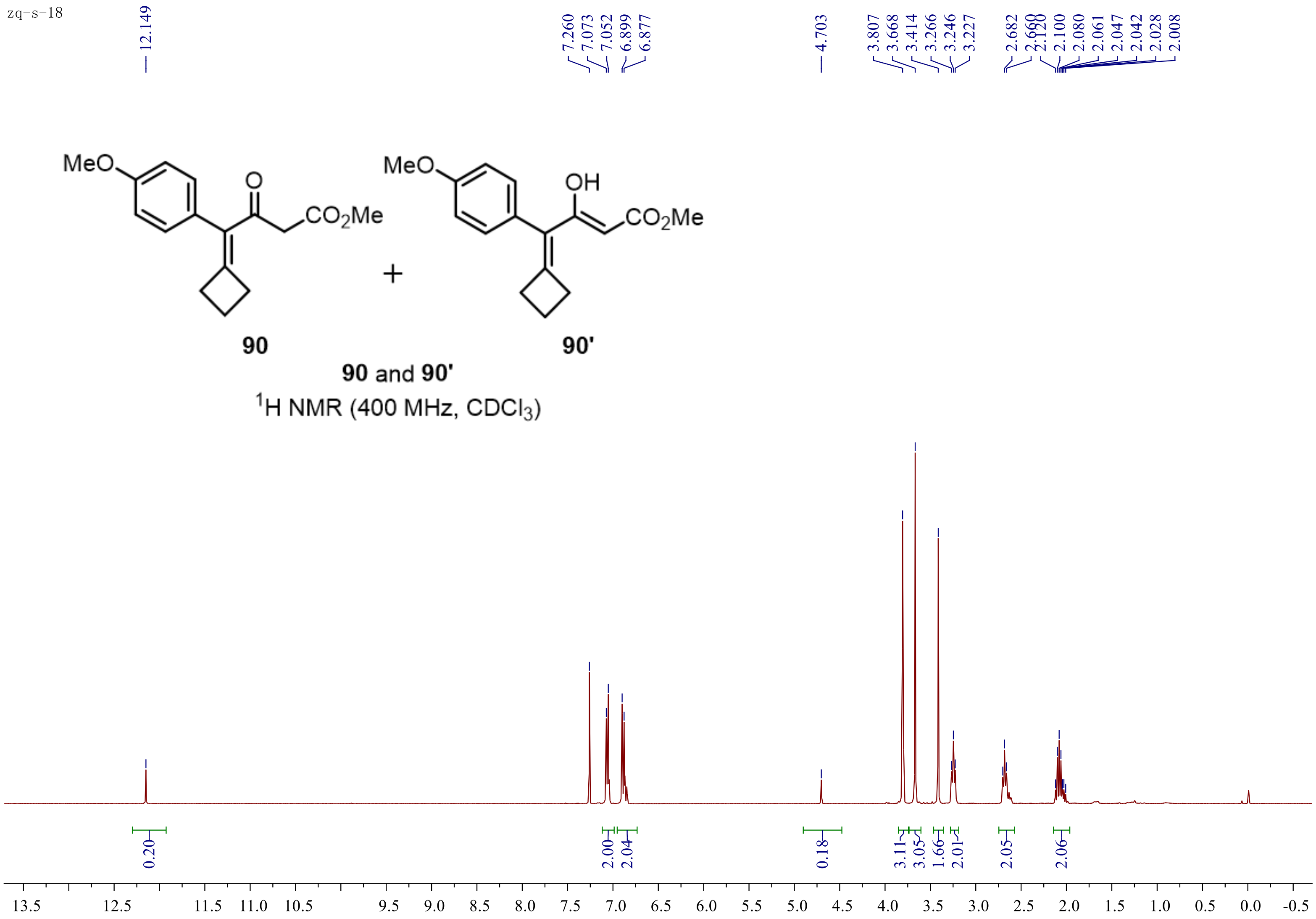

Supplementary Fig. 119. ^{1}H NMR spectra of compound **90** and **90'** in $CDCl_3$

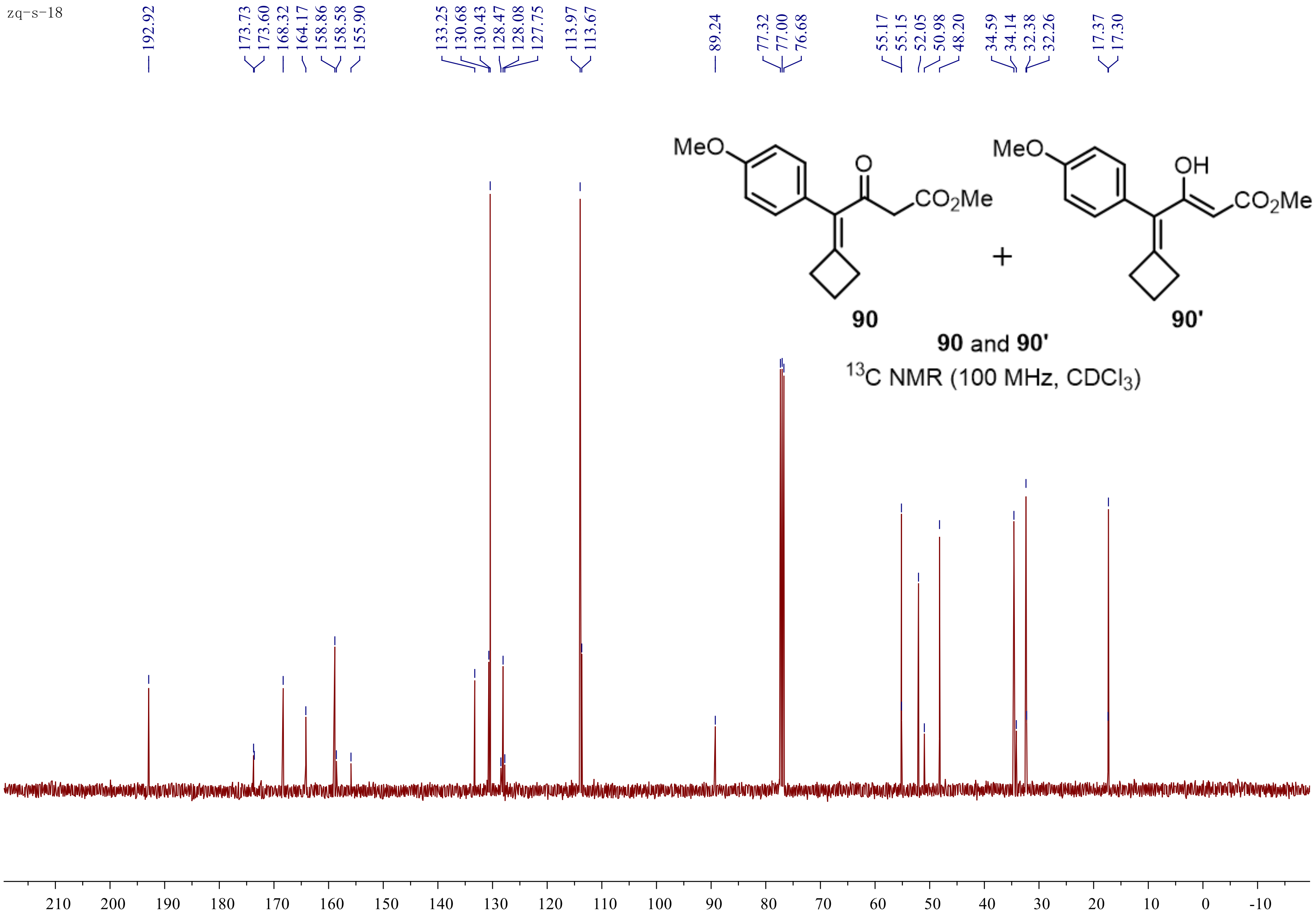

Supplementary Fig. 120. ^{13}C NMR spectra of compound **90** and **90'** in $CDCl_3$

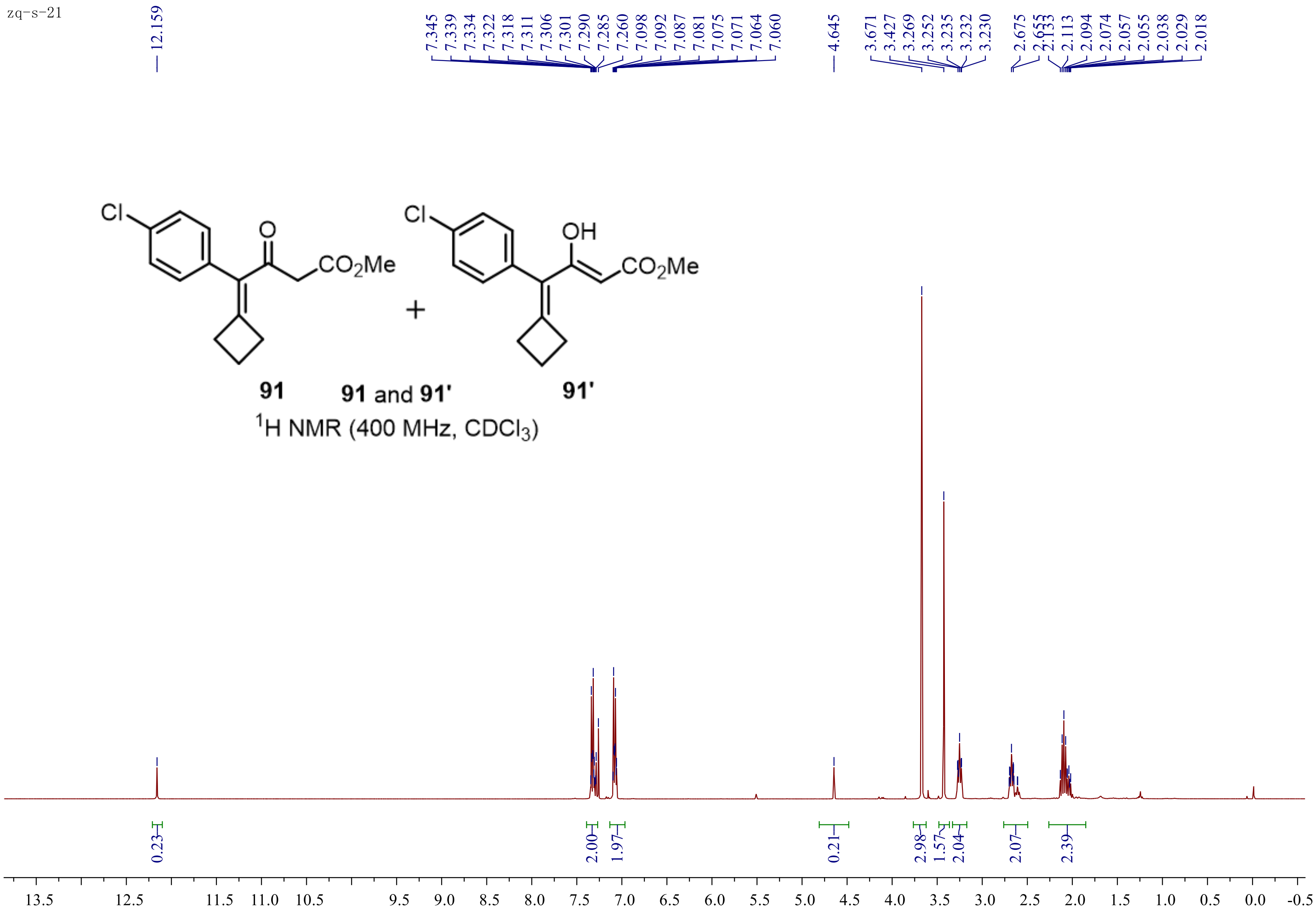

Supplementary Fig. 121. ^{1}H NMR spectra of compound **91** and **91'** in $CDCl_3$

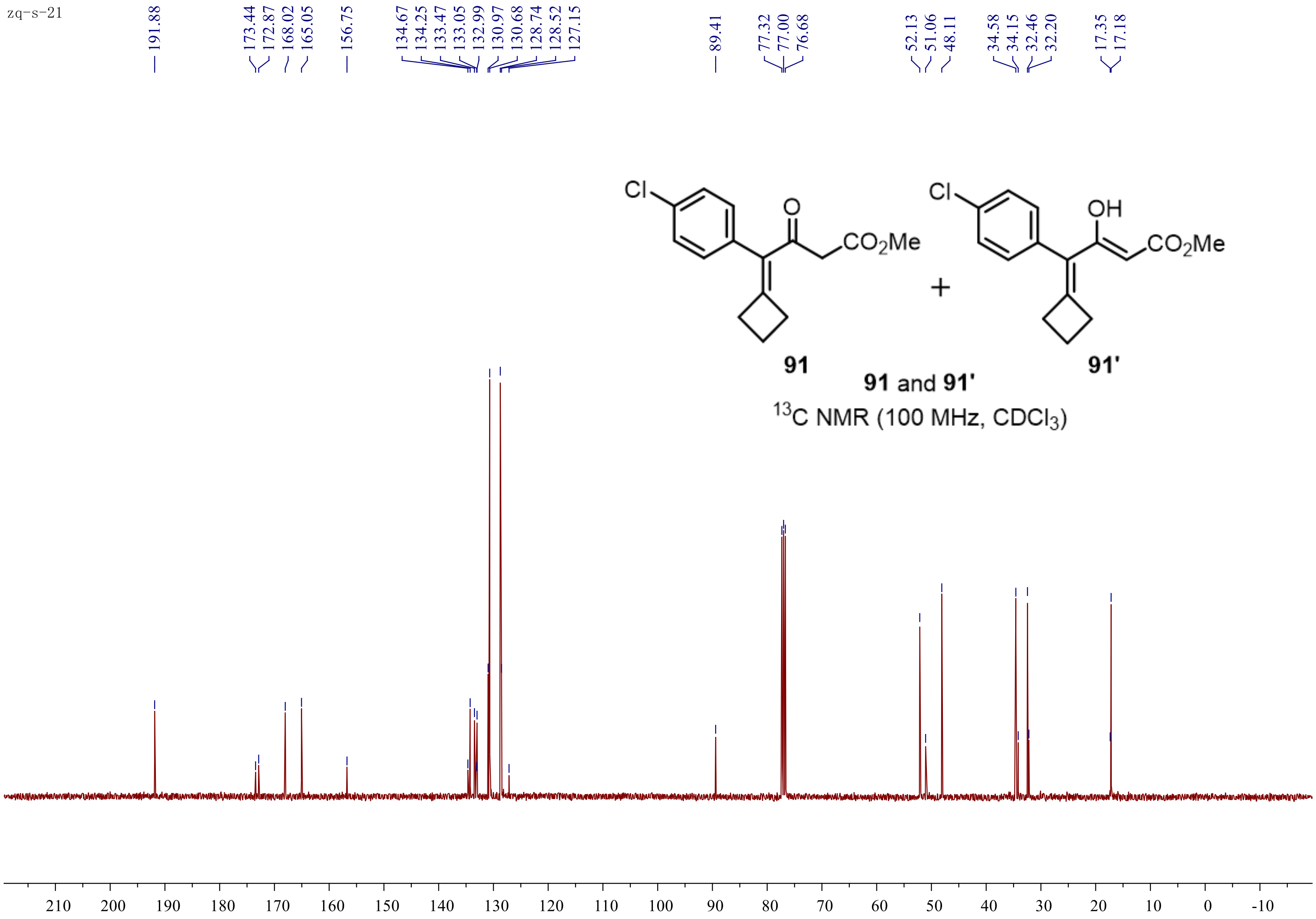

Supplementary Fig. 122. ^{13}C NMR spectra of compound **91** and **91'** in $CDCl_3$

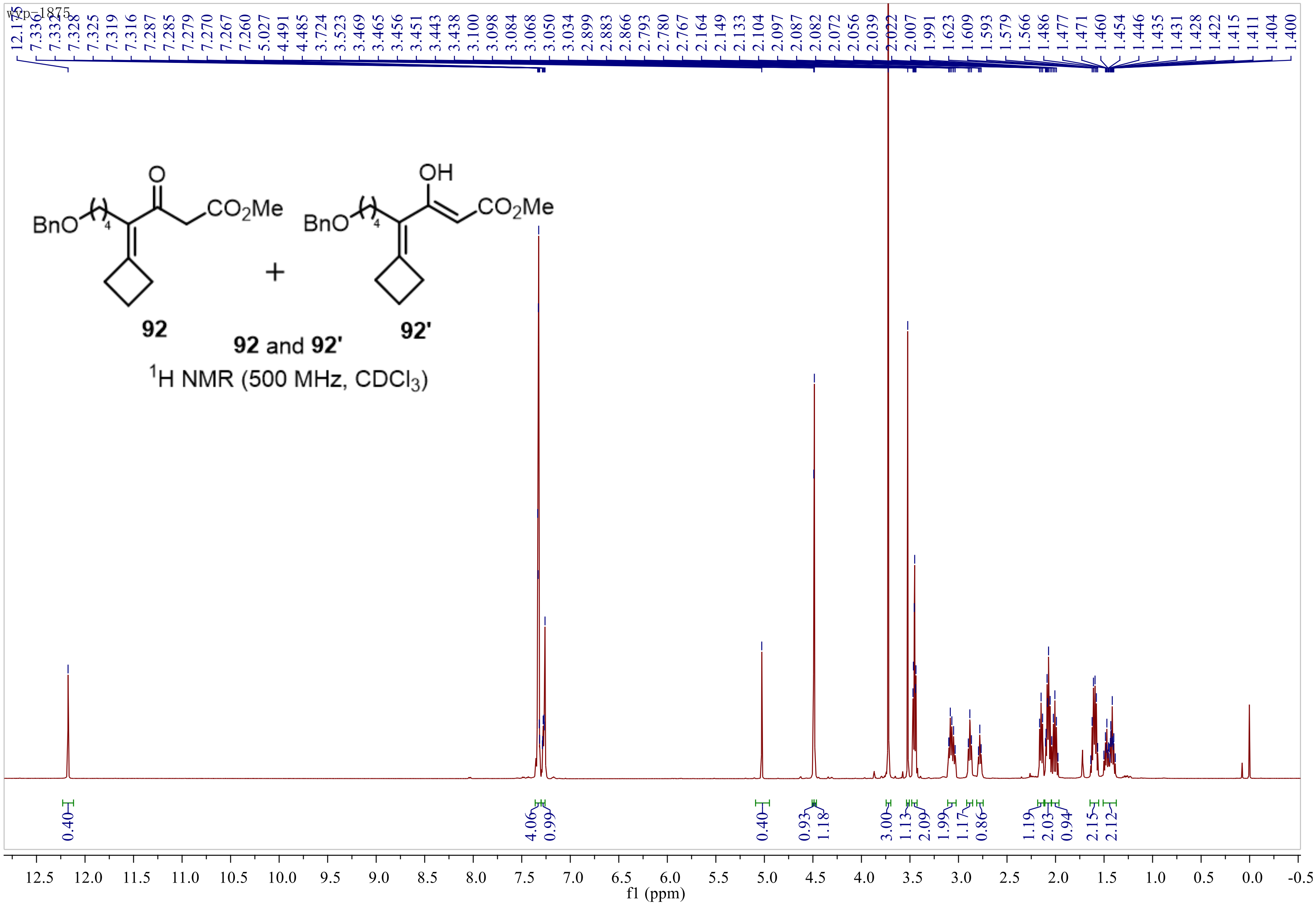

Supplementary Fig. 123. ^{1}H NMR spectra of compound **92** and **92'** in $CDCl_3$

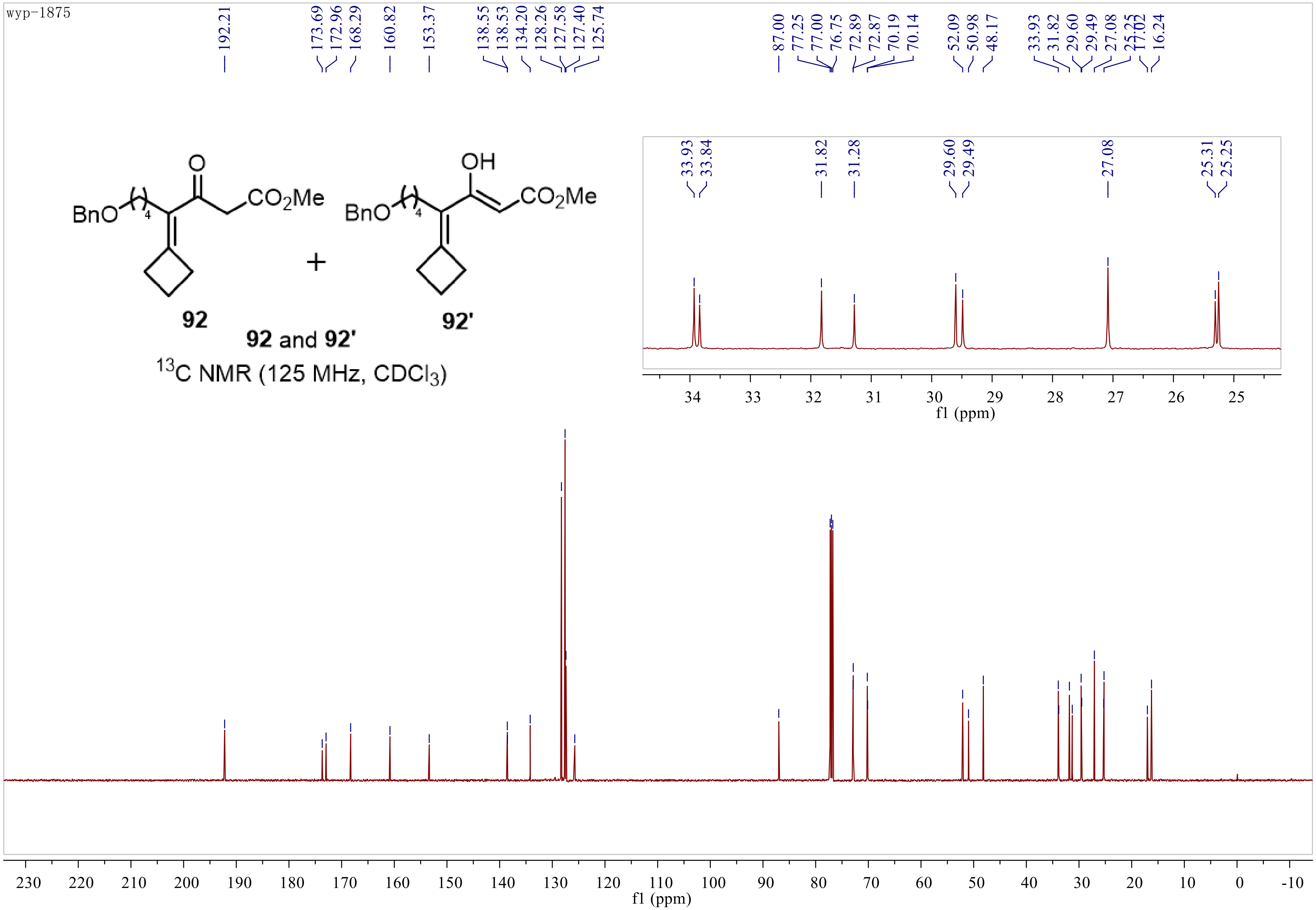

Supplementary Fig. 124. ^{13}C NMR spectra of compound **92** and **92'** in $CDCl_3$

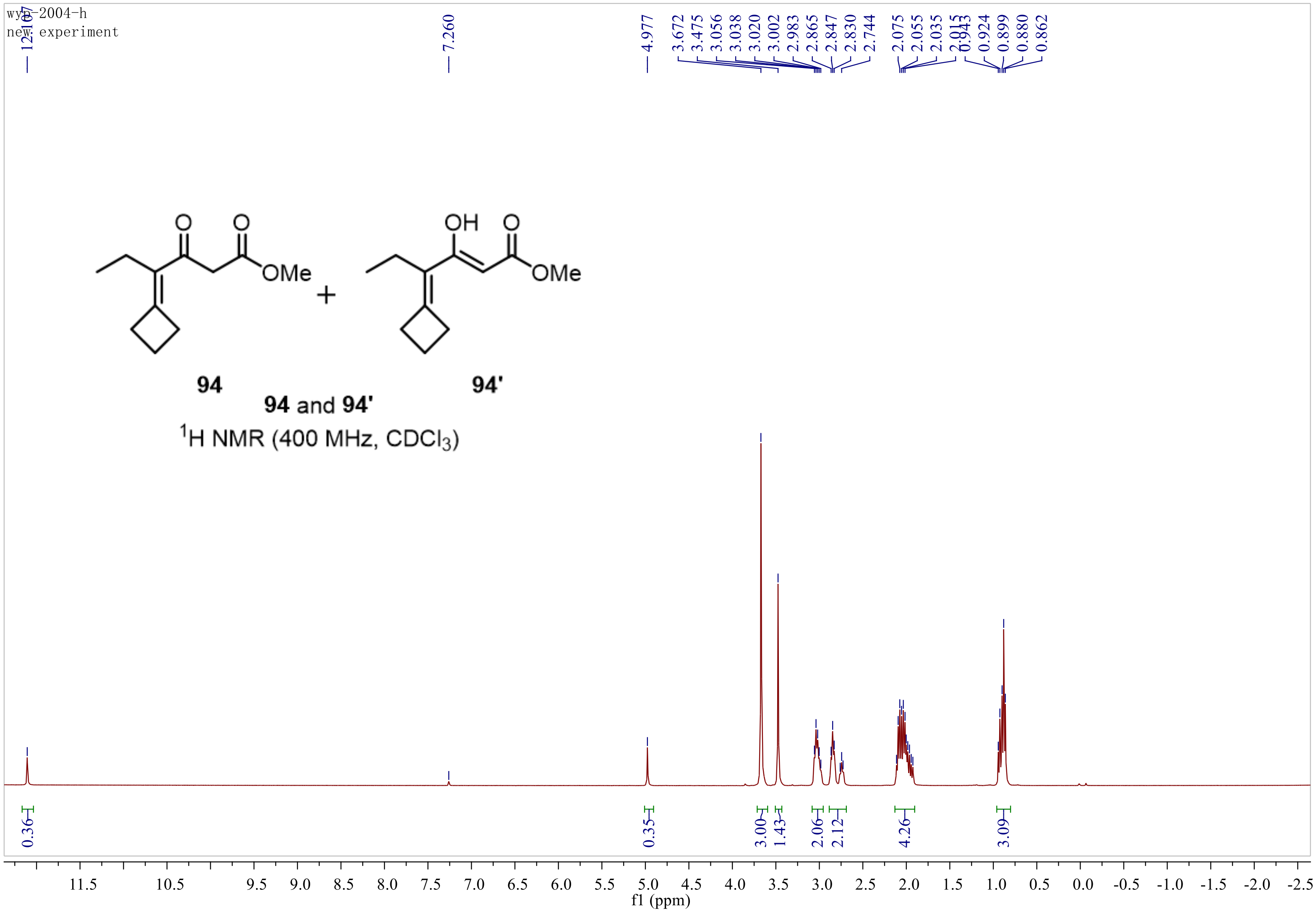

Supplementary Fig. 125. ^{1}H NMR spectra of compound **94** and **94'** in $CDCl_3$

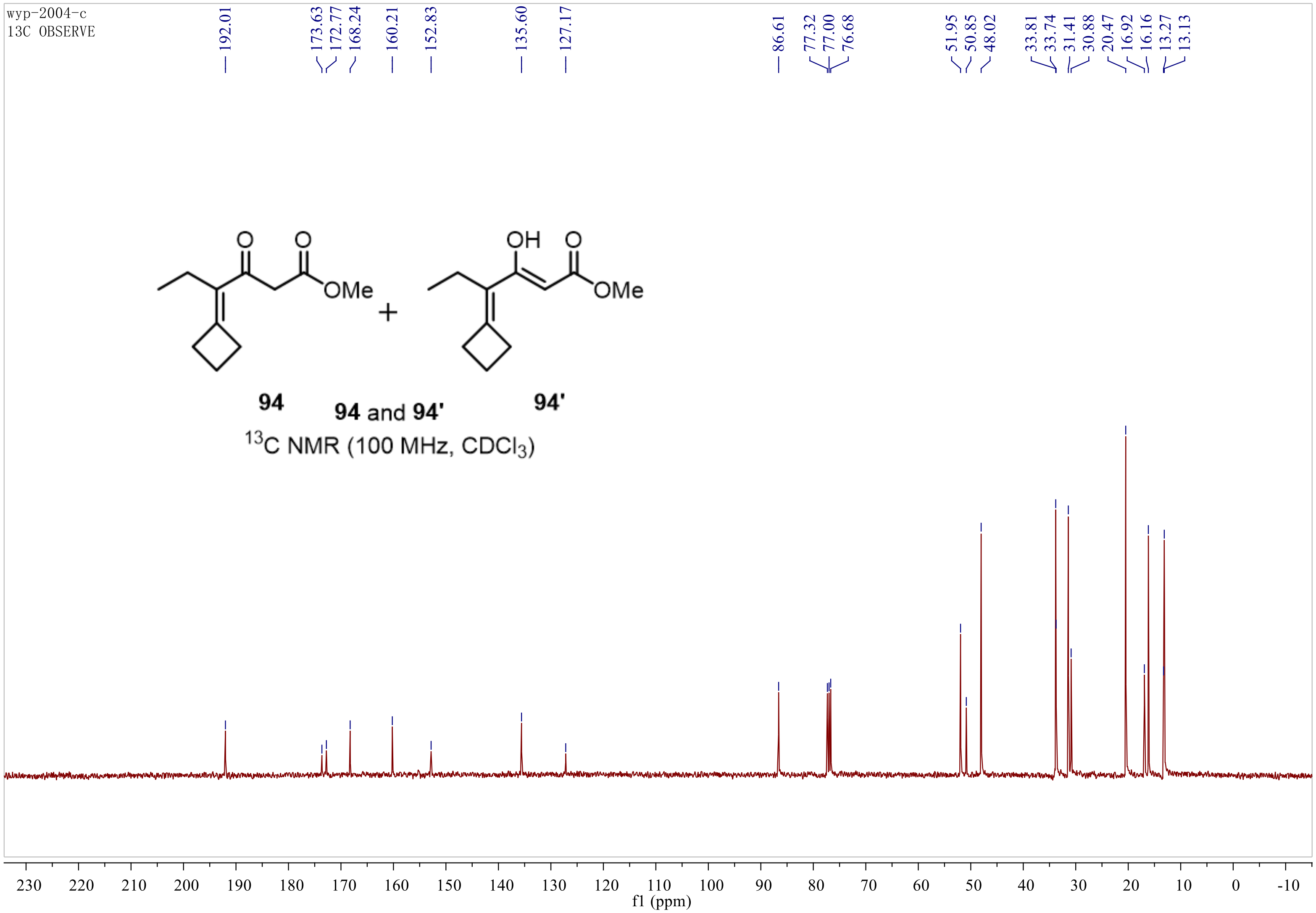

Supplementary Fig. 126. ^{13}C NMR spectra of compound **94** and **94'** in $CDCl_3$

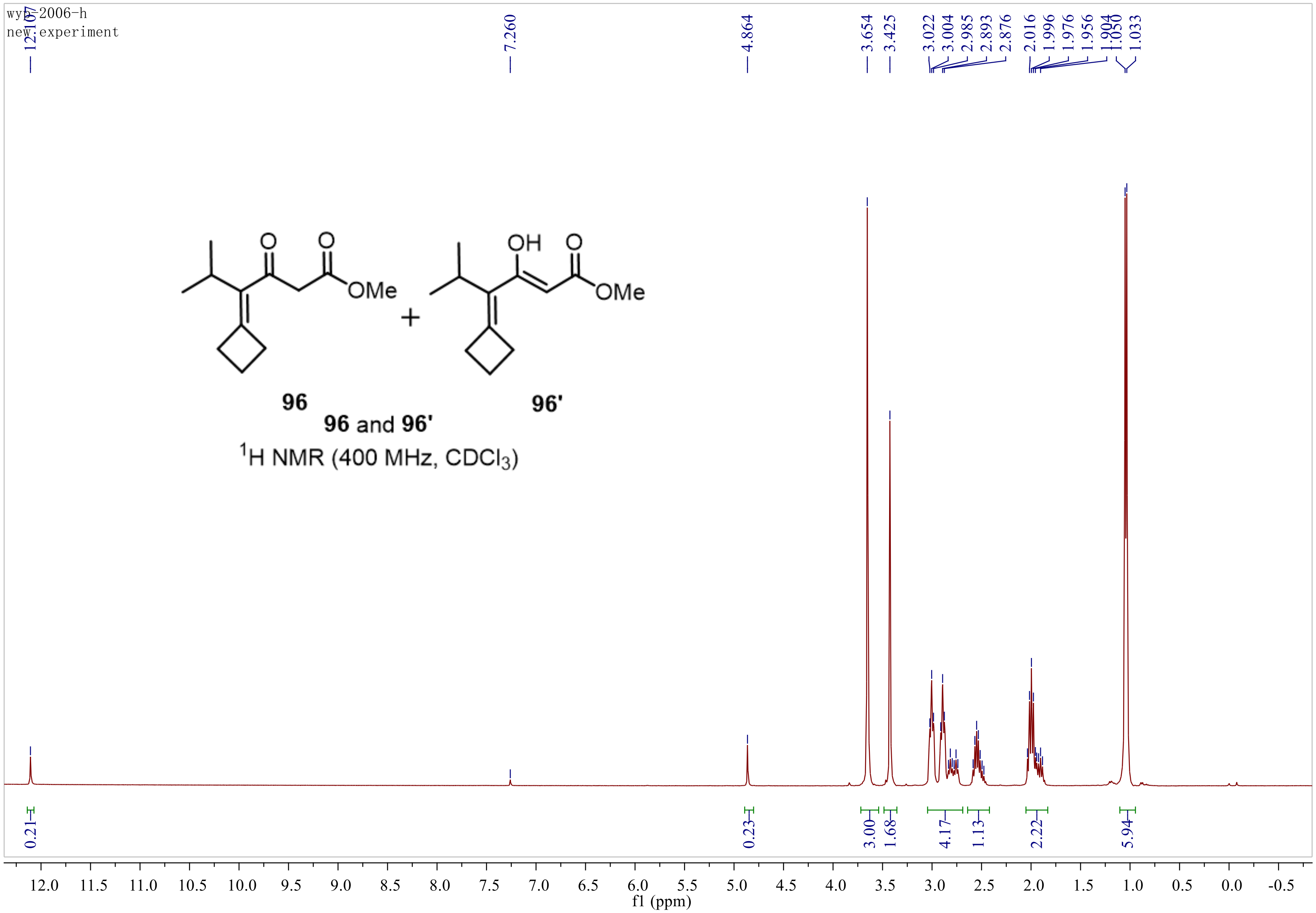

Supplementary Fig. 127. ^{1}H NMR spectra of compound **96** and **96'** in $CDCl_3$

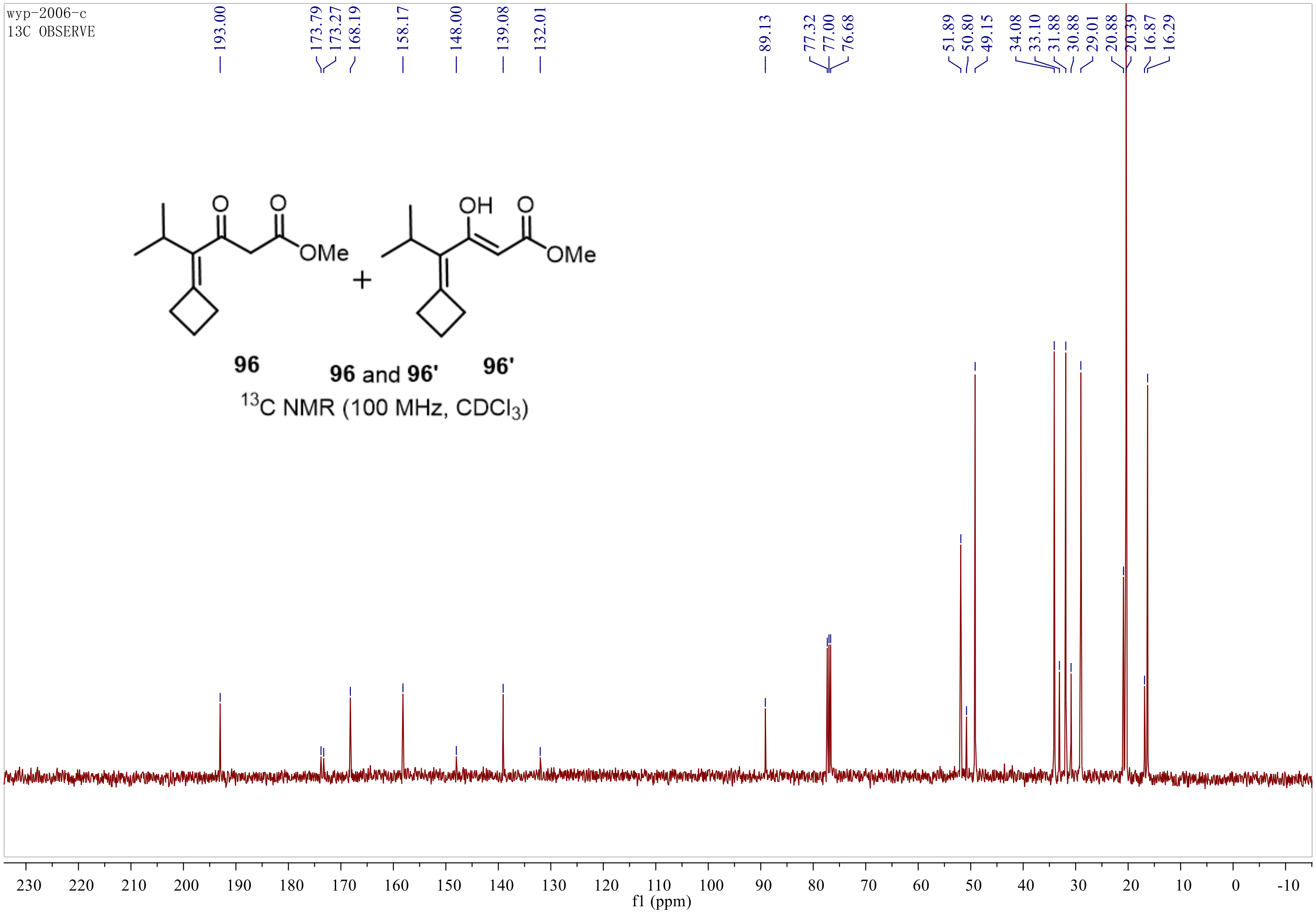

Supplementary Fig. 128. ^{13}C NMR spectra of compound **96** and **96'** in $CDCl_3$

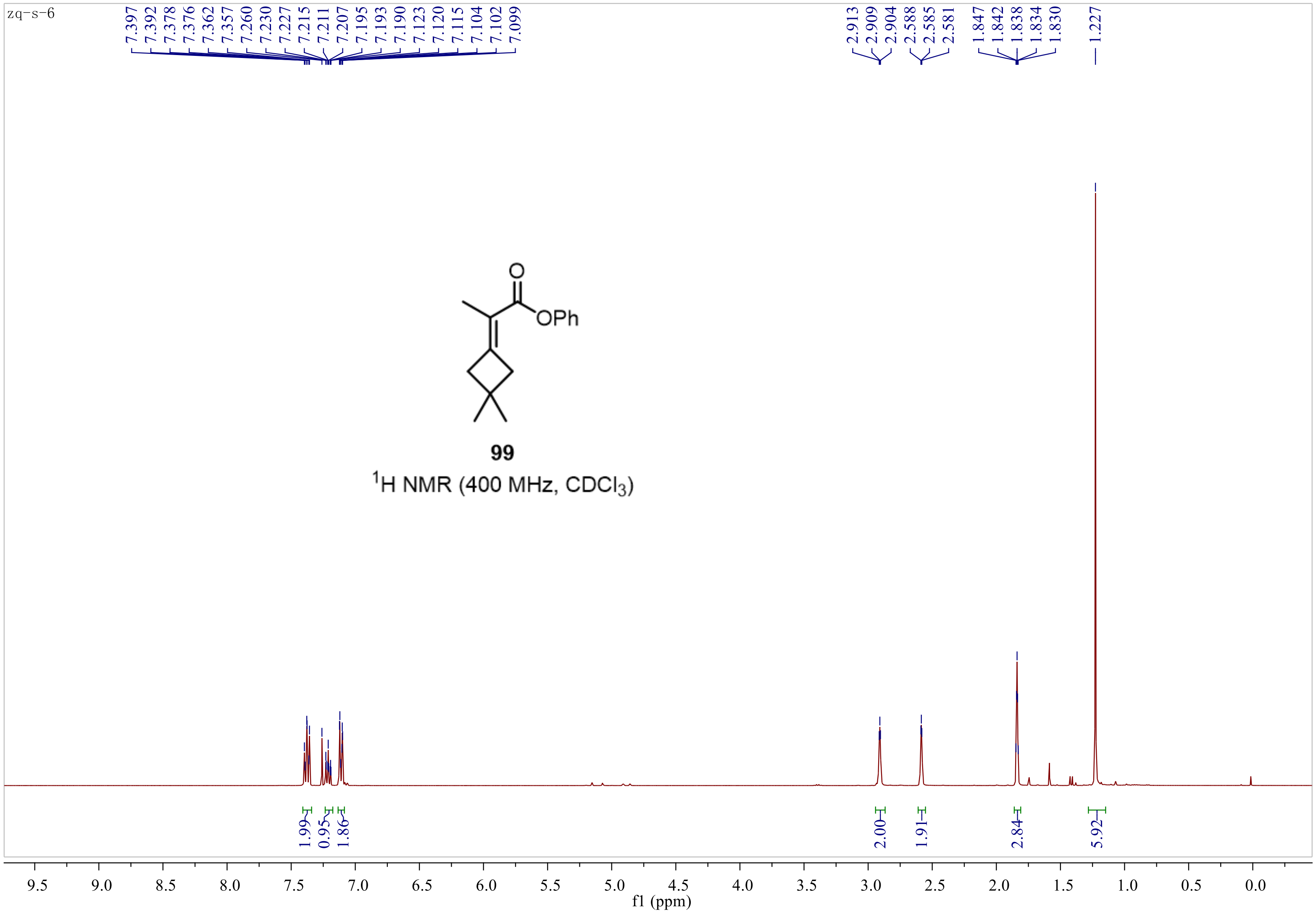

Supplementary Fig. 129. ^{1}H NMR spectra of compound **99** in $CDCl_3$

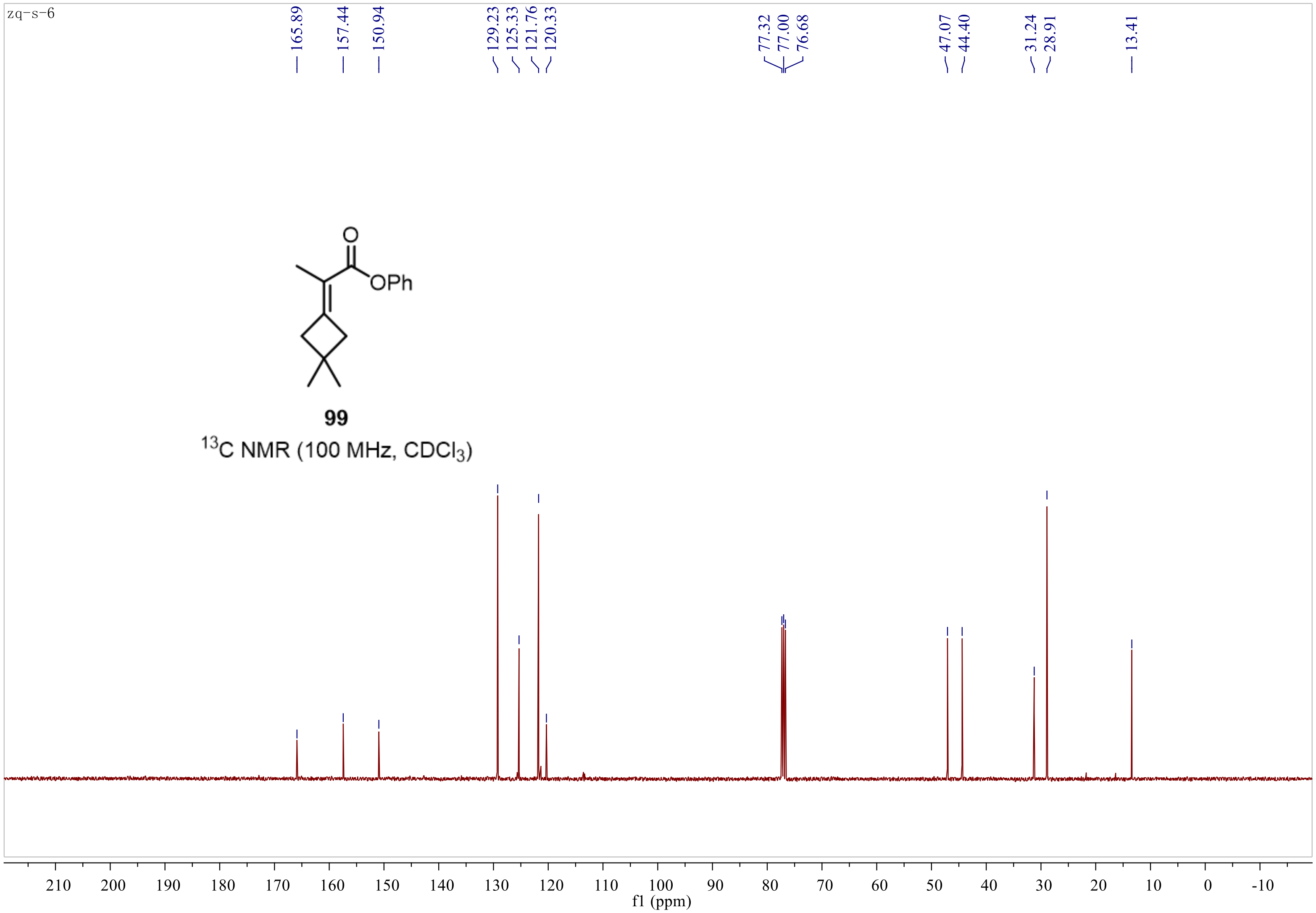

Supplementary Fig. 130. ^{13}C NMR spectra of compound **99** in $CDCl_3$

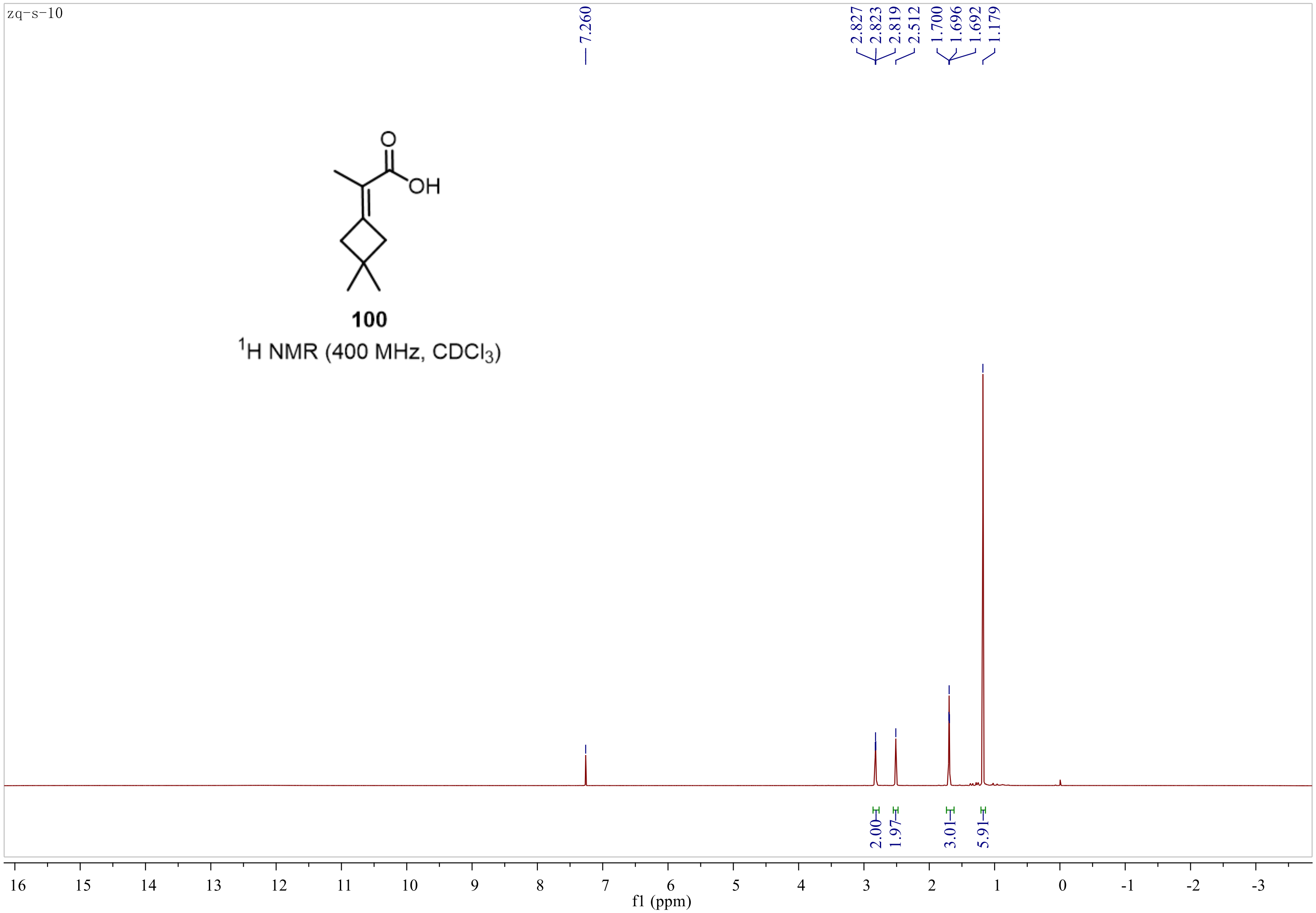

Supplementary Fig. 131. ^{1}H NMR spectra of compound **100** in $CDCl_3$

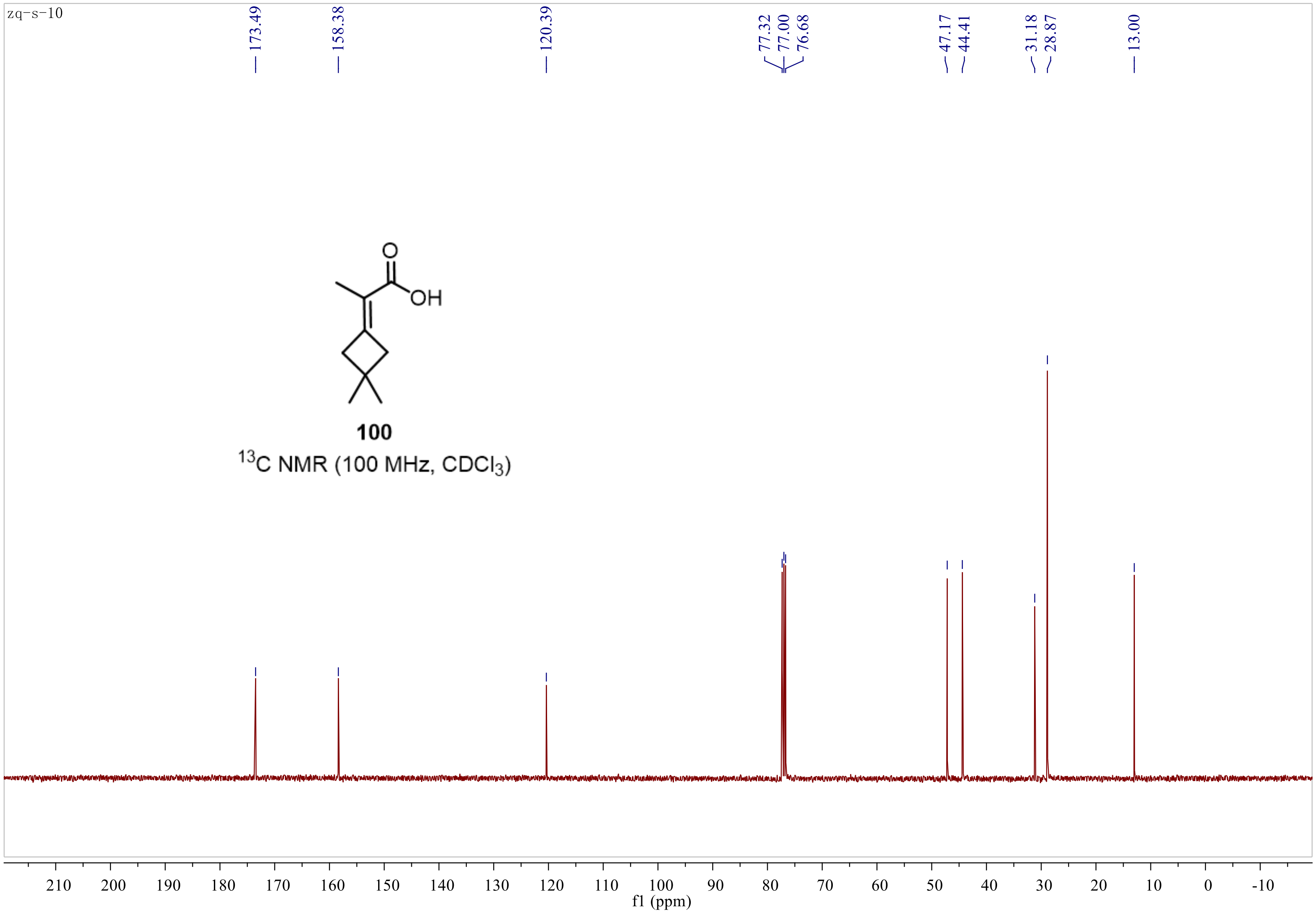

Supplementary Fig. 132. ^{13}C NMR spectra of compound **100** in $CDCl_3$

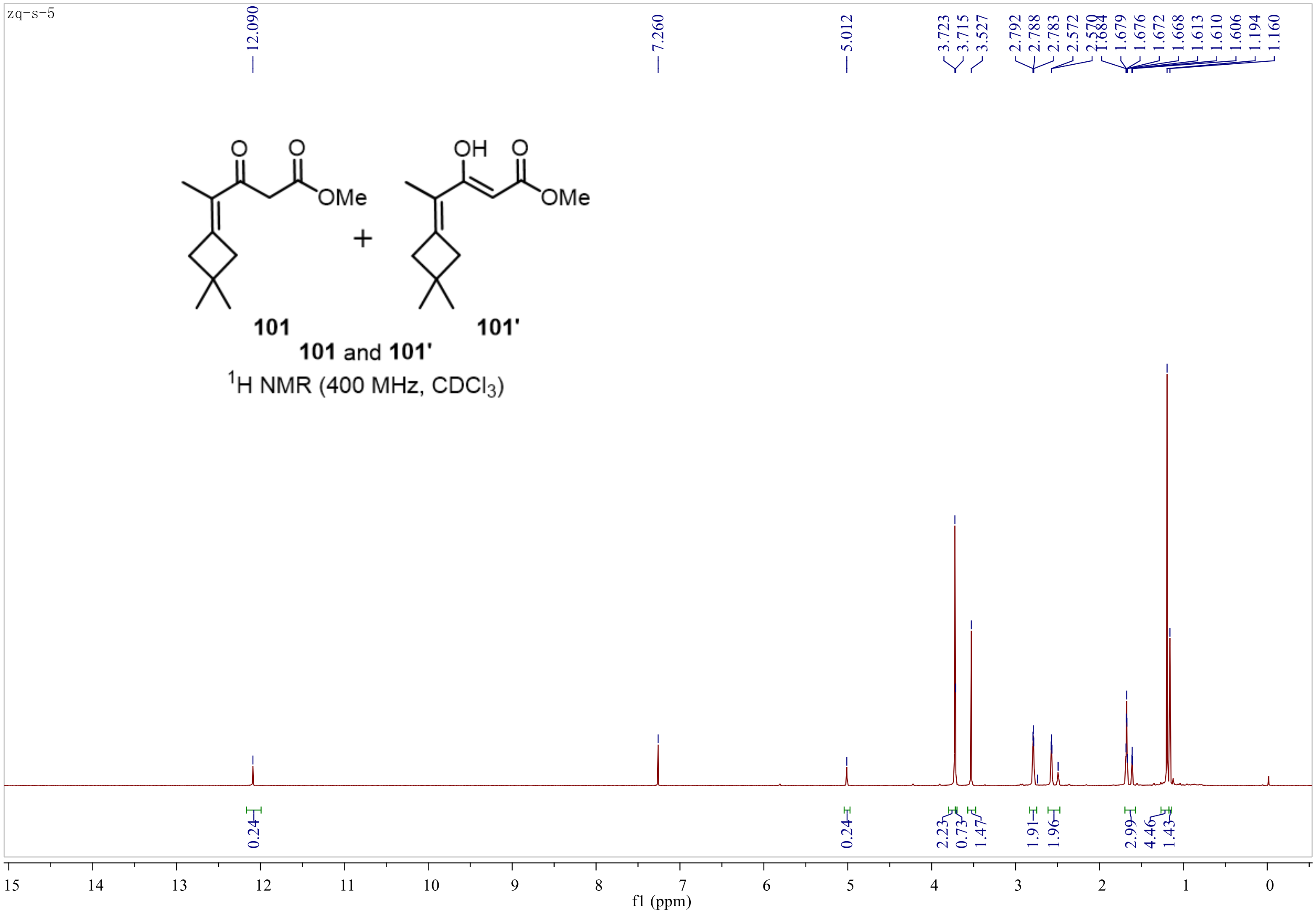

Supplementary Fig. 133. ^{1}H NMR spectra of compound **101** and **101'** in $CDCl_3$

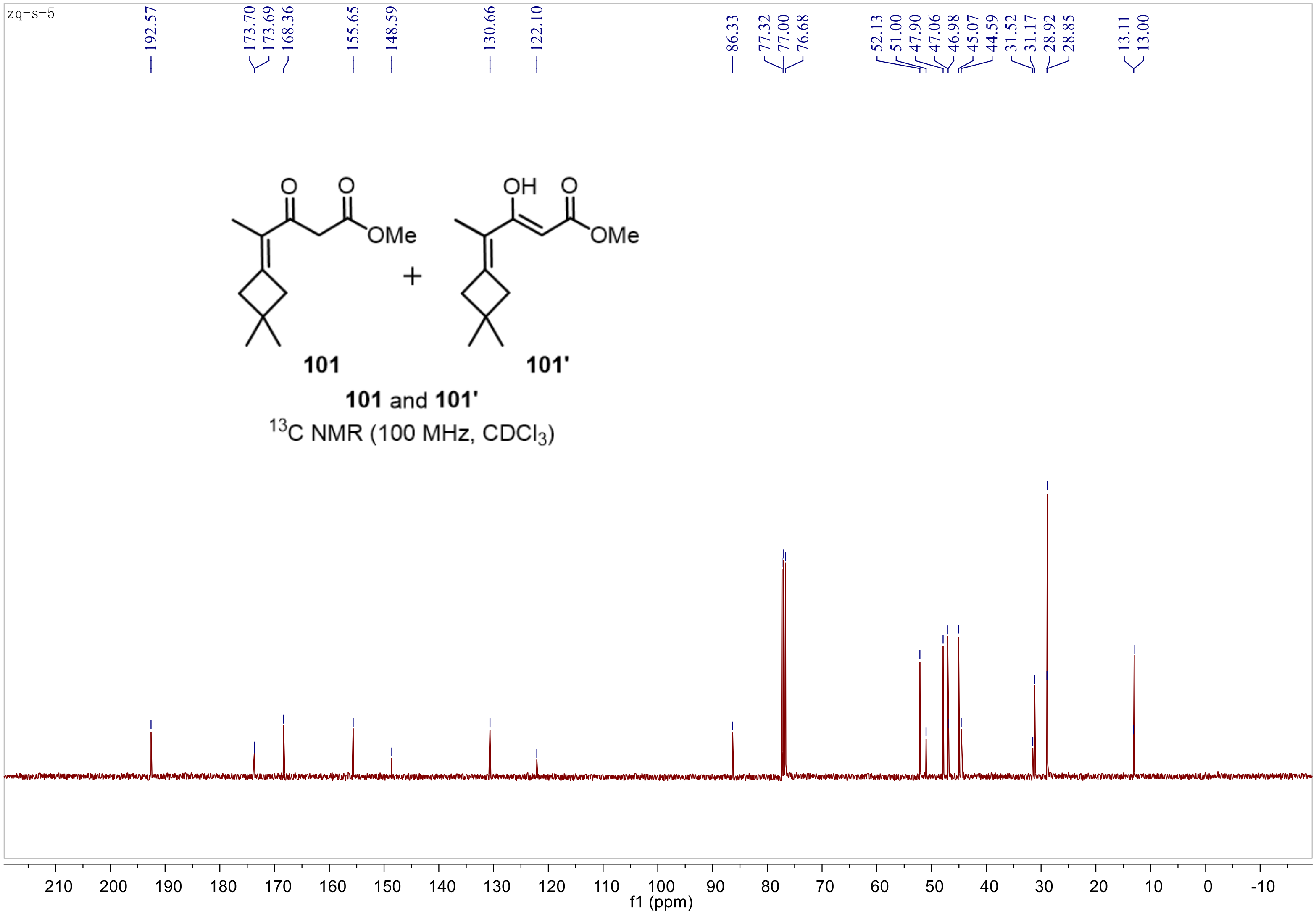

Supplementary Fig. 134. ^{13}C NMR spectra of compound **101** and **101'** in $CDCl_3$

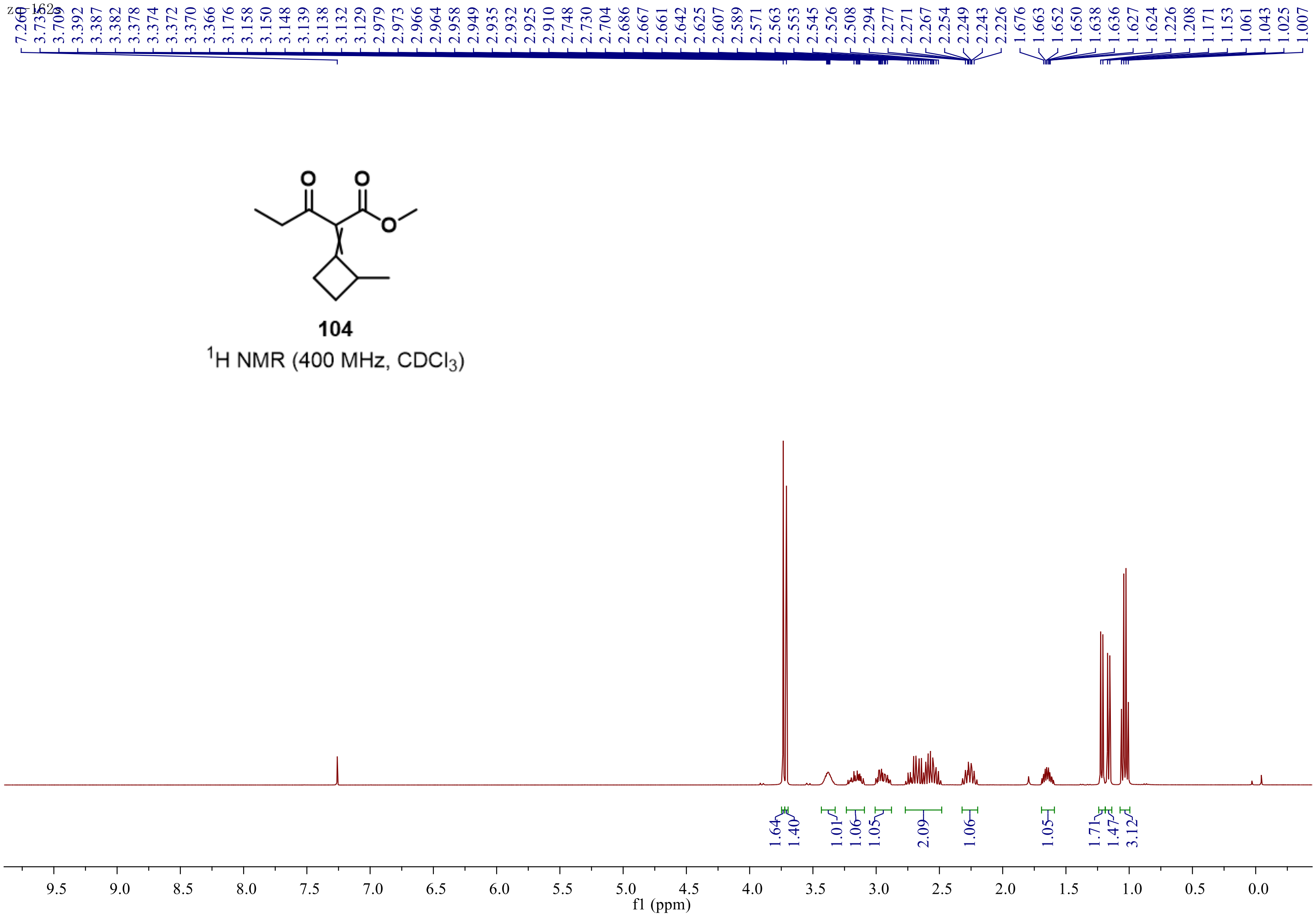

Supplementary Fig. 135. ^{1}H NMR spectra of compound **104** in $CDCl_3$

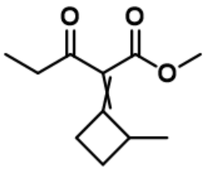

104

^{13}C NMR (100 MHz, $CDCl_3$)

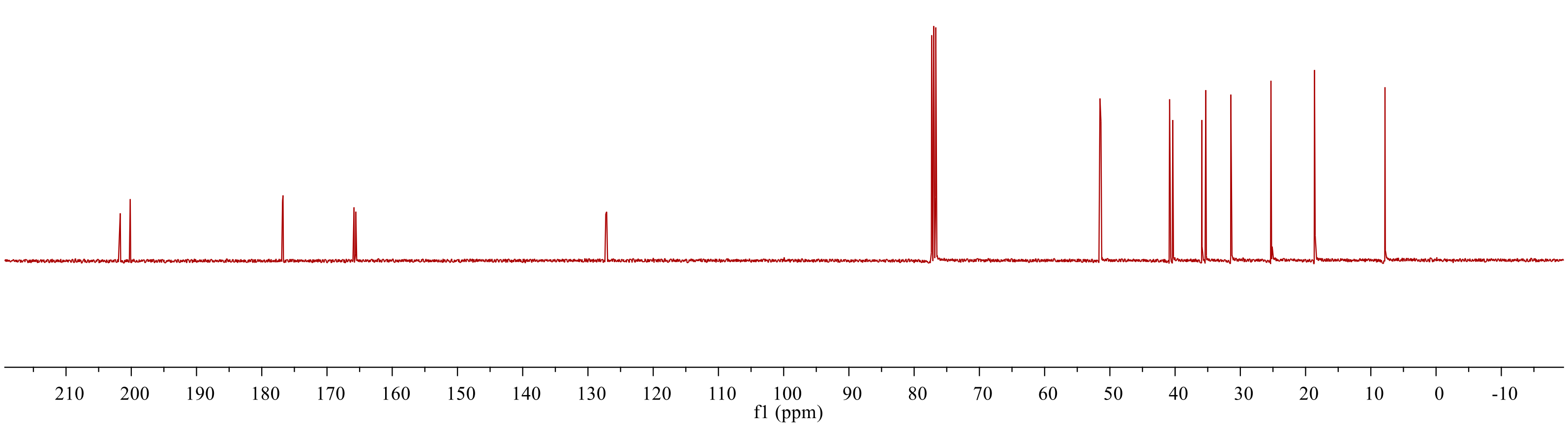

Supplementary Fig. 136. ^{13}C NMR spectra of compound **104** in $CDCl_3$

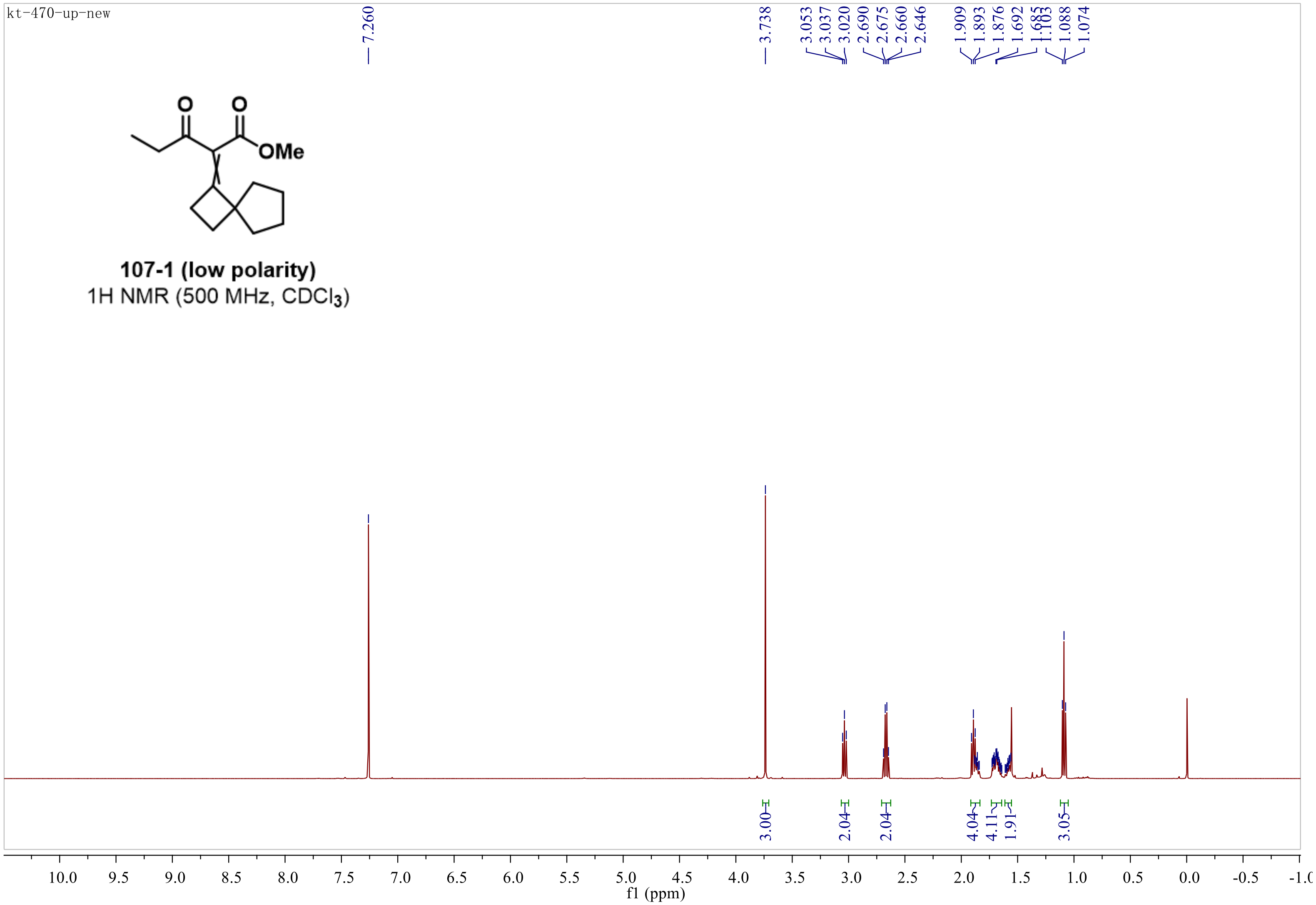

Supplementary Fig. 137. ^{1}H NMR spectra of compound **107-1** in $CDCl_3$

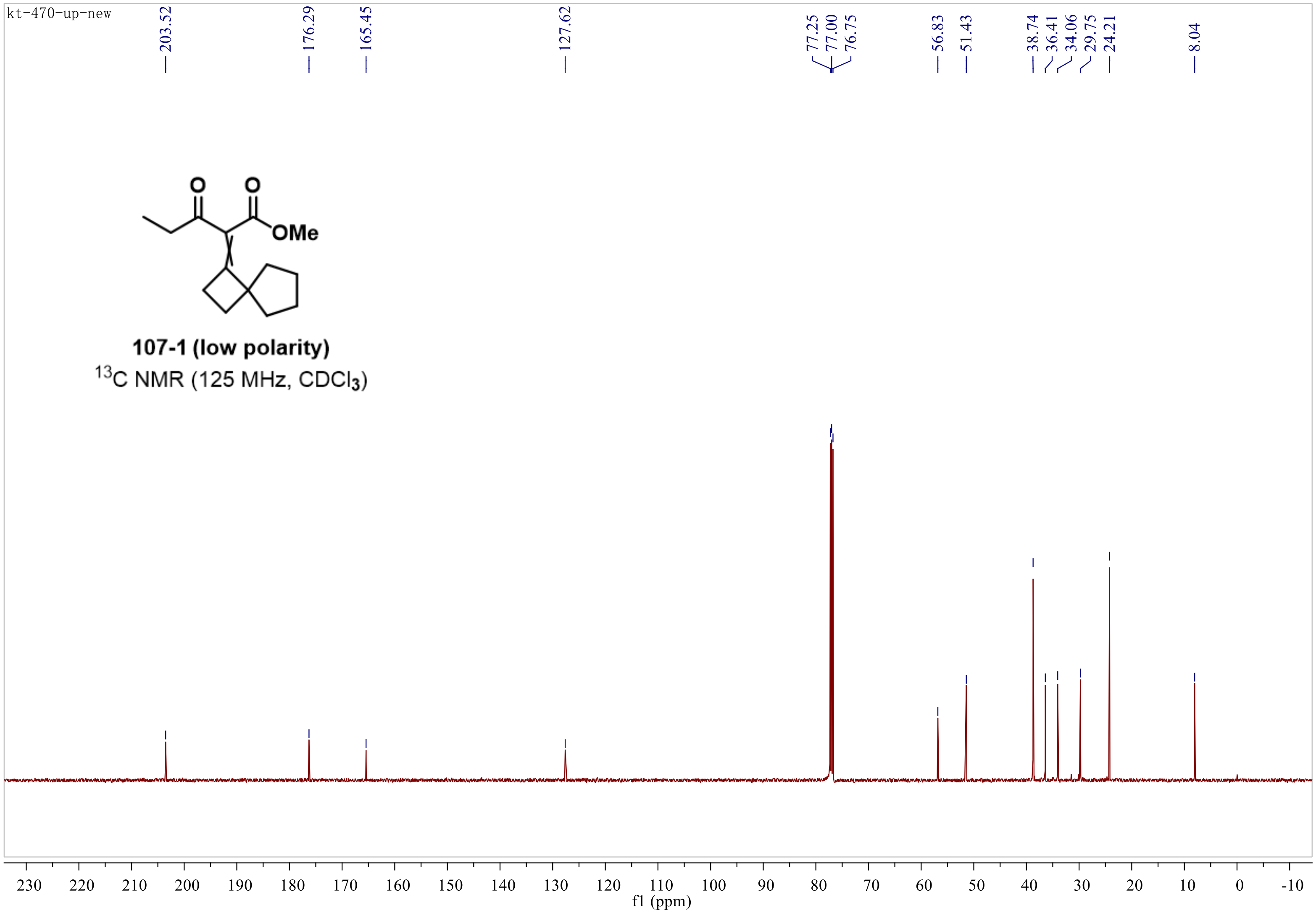

Supplementary Fig. 138. ^{13}C NMR spectra of compound **107-1** in $CDCl_3$

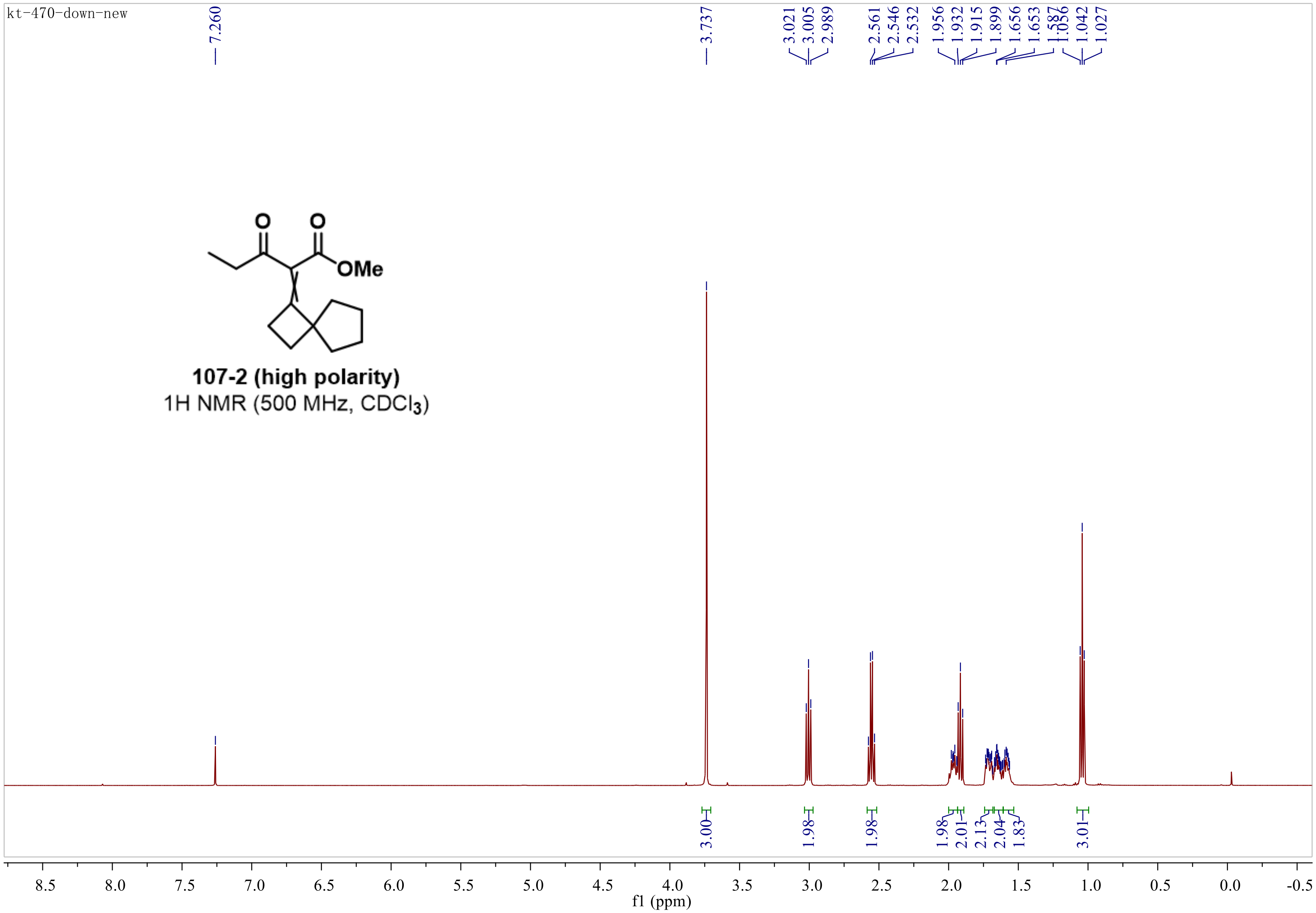

Supplementary Fig. 139. ^{1}H NMR spectra of compound **107-2** in $CDCl_3$

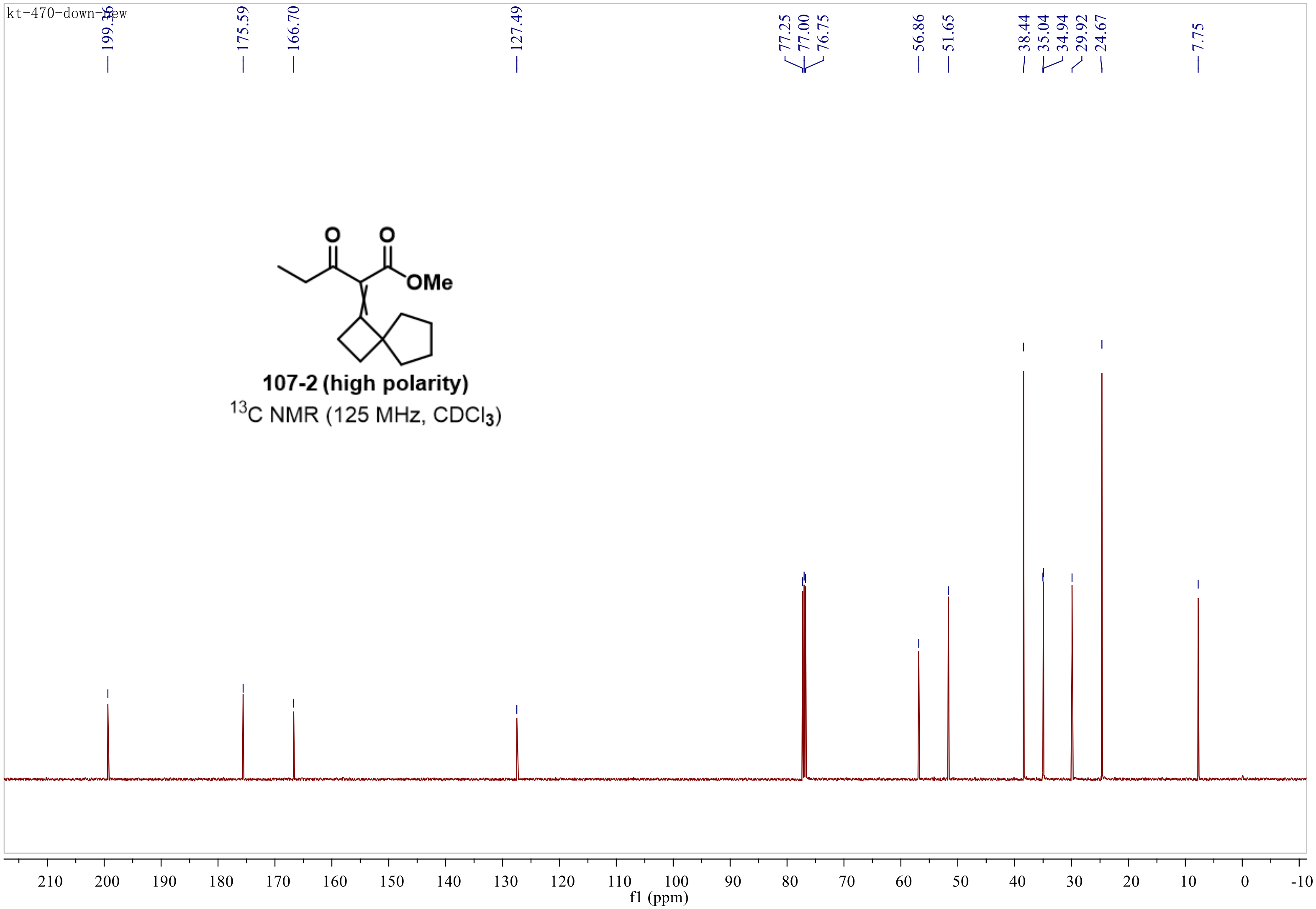

Supplementary Fig. 140. ^{13}C NMR spectra of compound **107-2** in $CDCl_3$

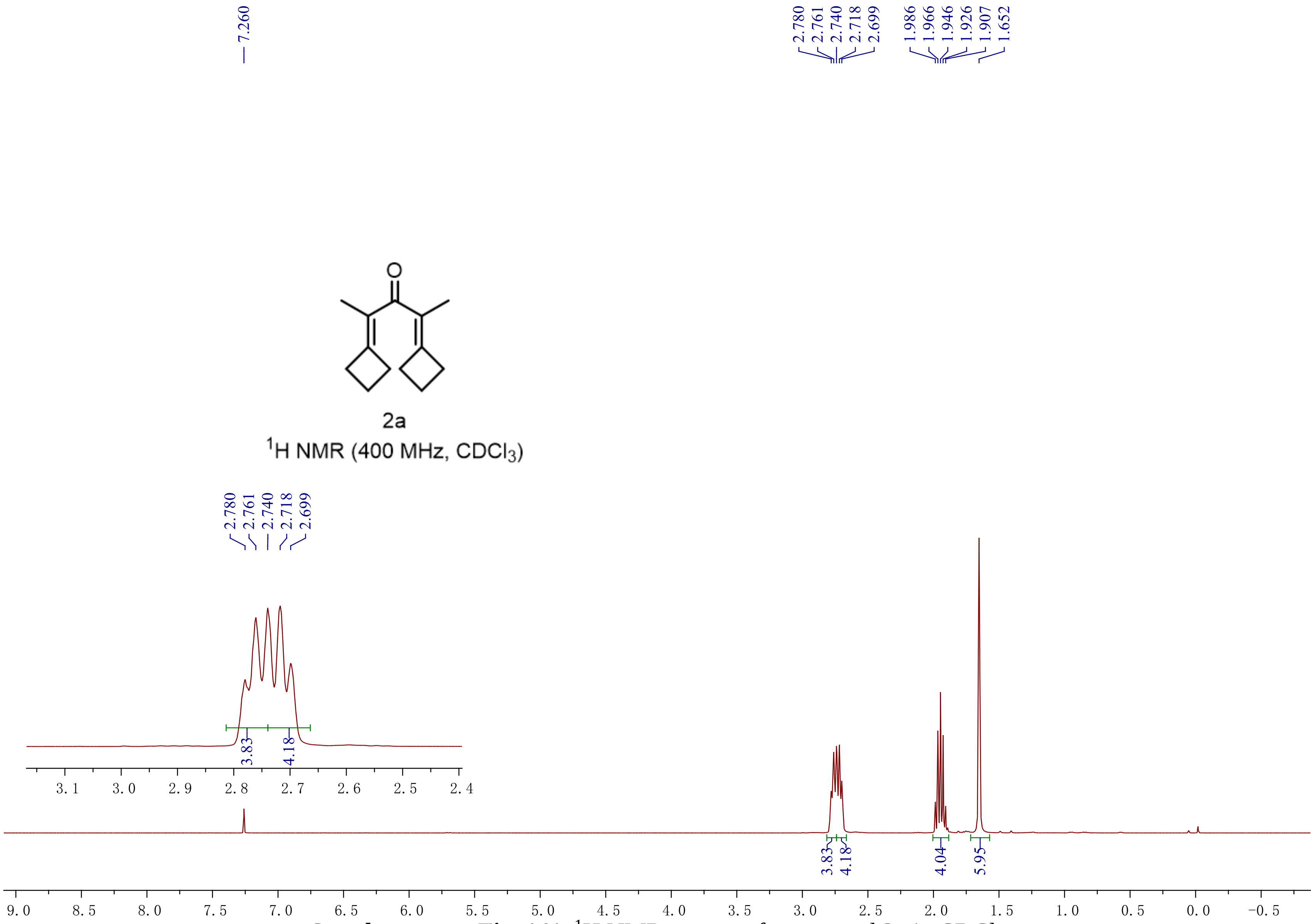

Supplementary Fig. 141. ^{1}H NMR spectra of compound **2a** in $CDCl_3$

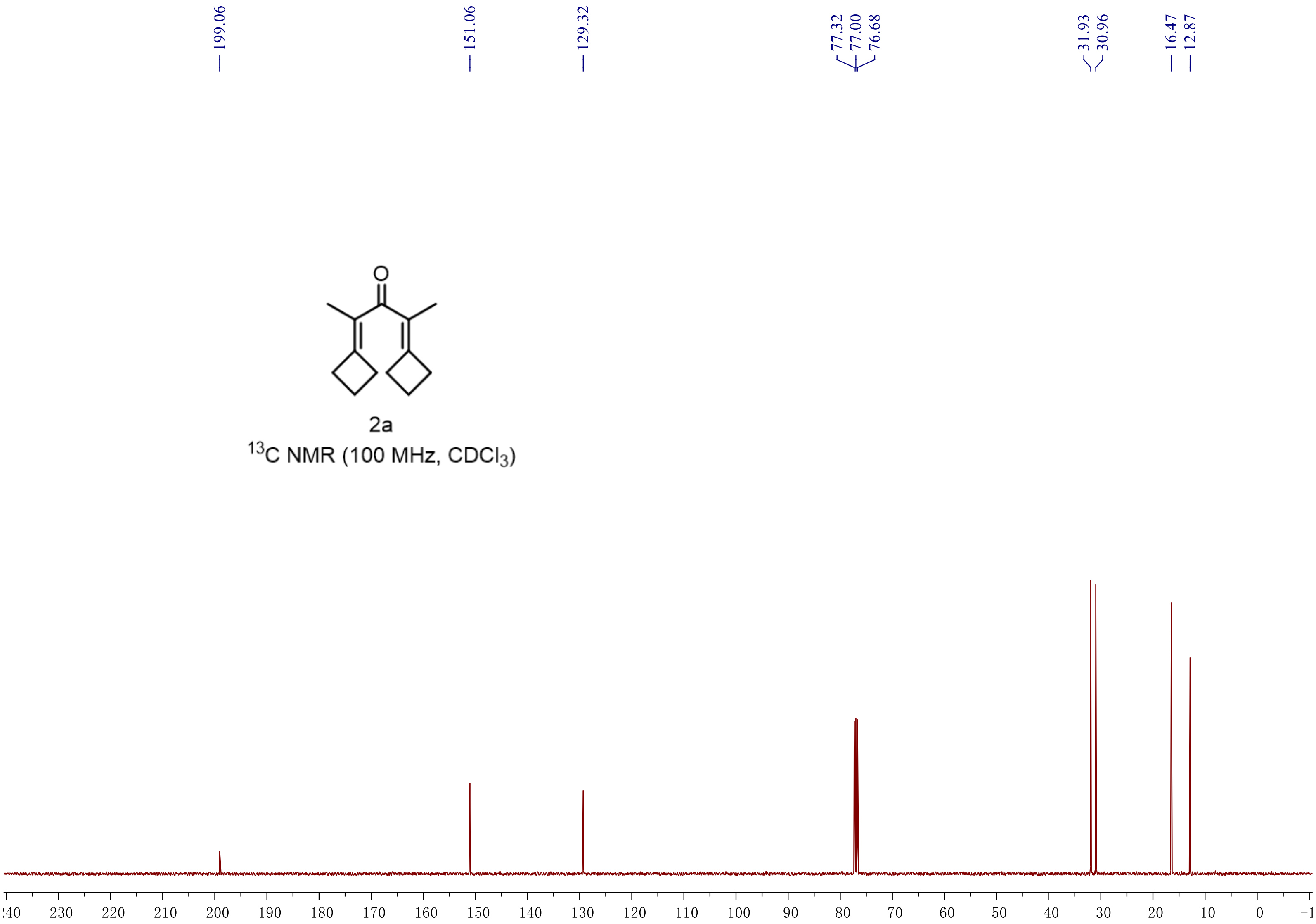

Supplementary Fig. 142. ^{13}C NMR spectra of compound **2a** in $CDCl_3$

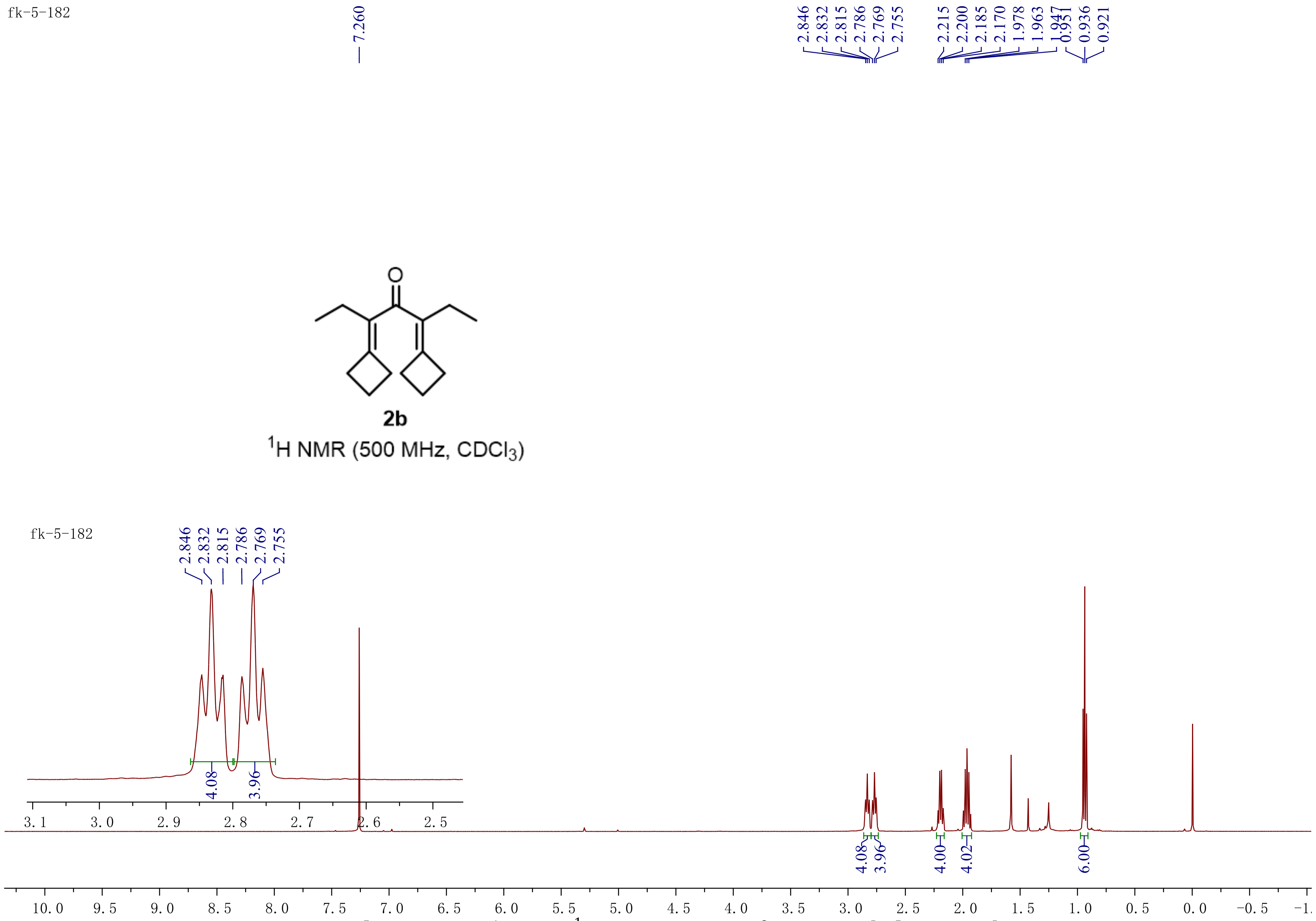

Supplementary Fig. 143. ^{1}H NMR spectra of compound **2b** in $CDCl_3$

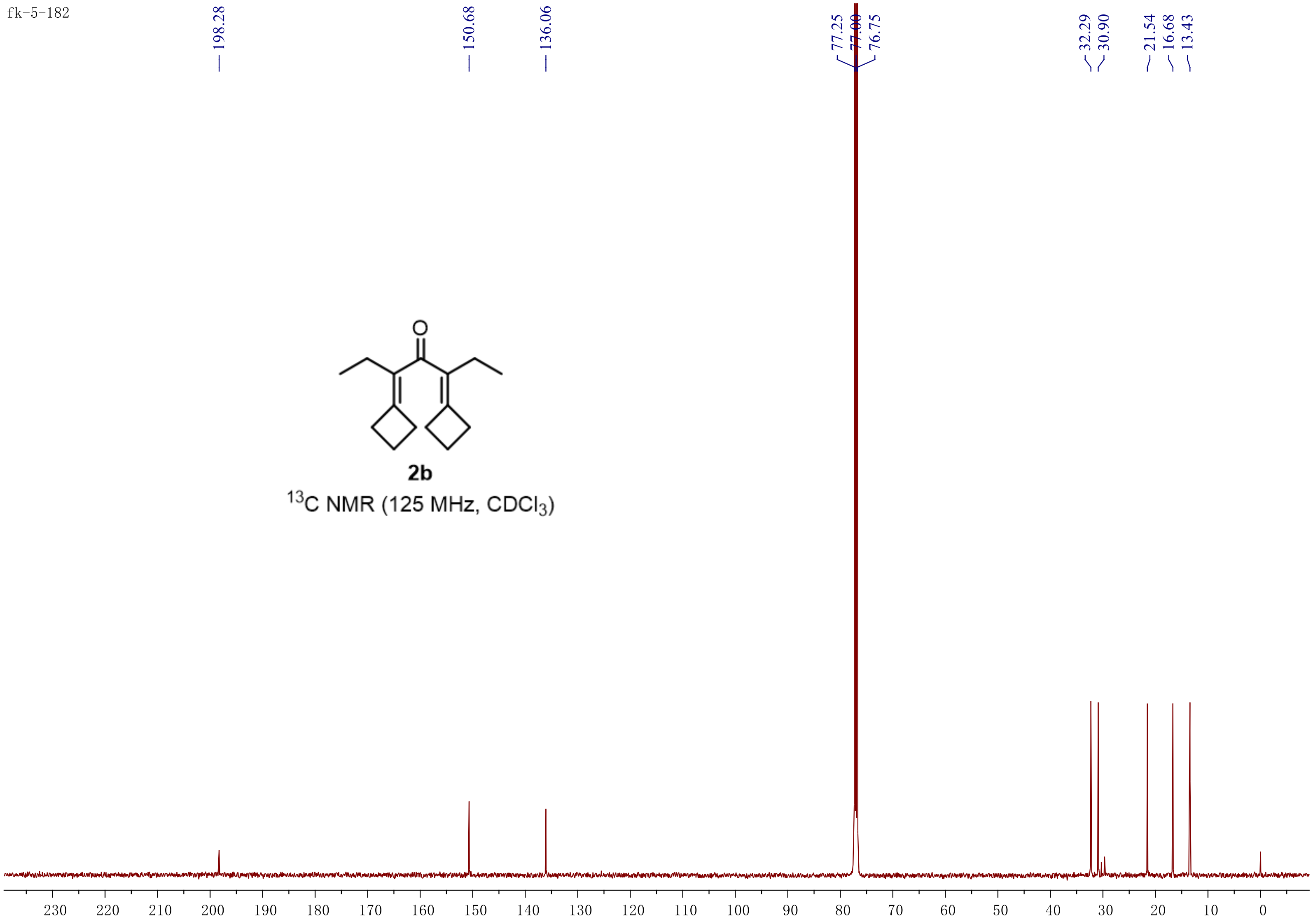

Supplementary Fig. 144. ^{13}C NMR spectra of compound **2b** in $CDCl_3$

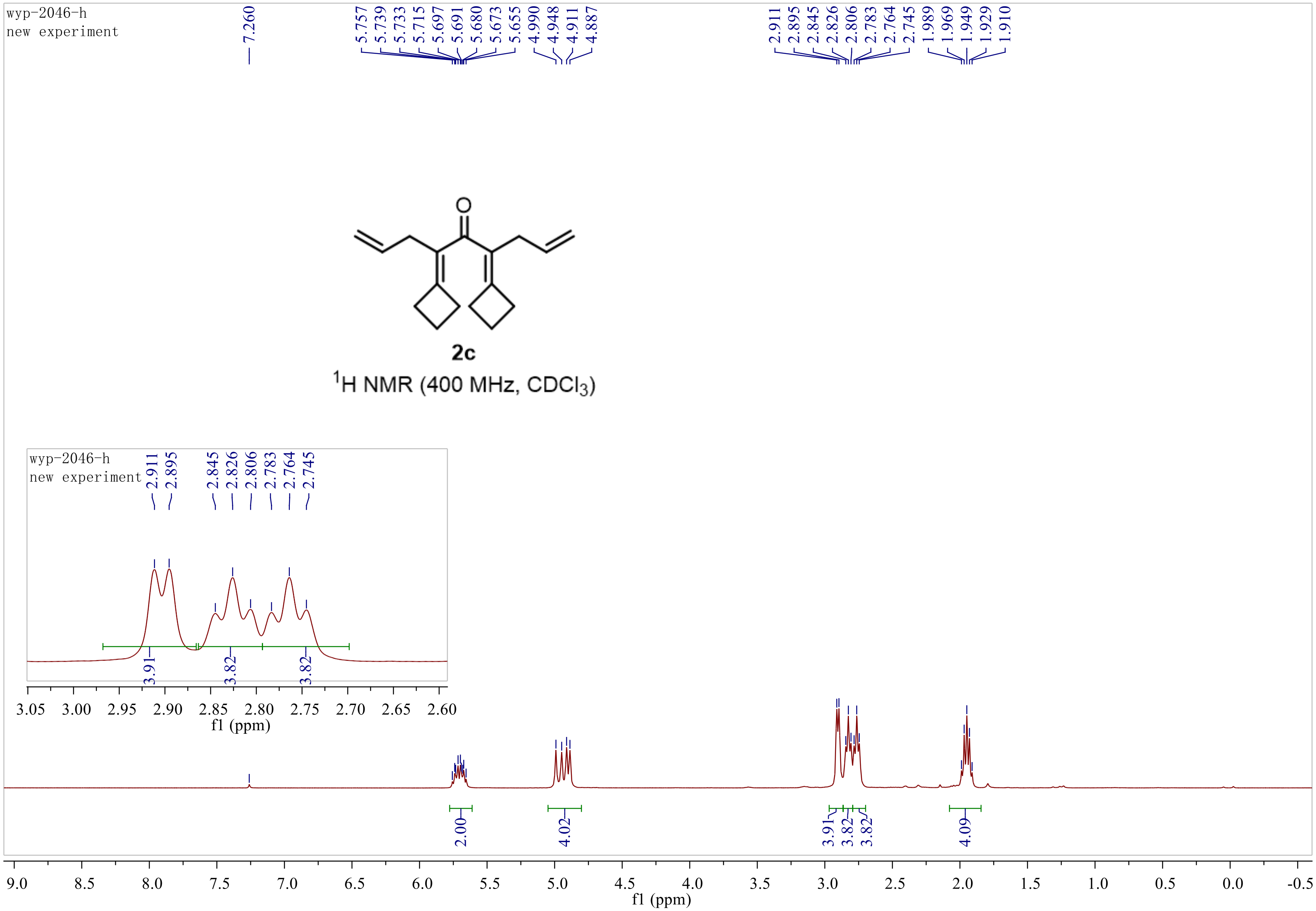

Supplementary Fig. 145. ^{1}H NMR spectra of compound **2c** in $CDCl_3$

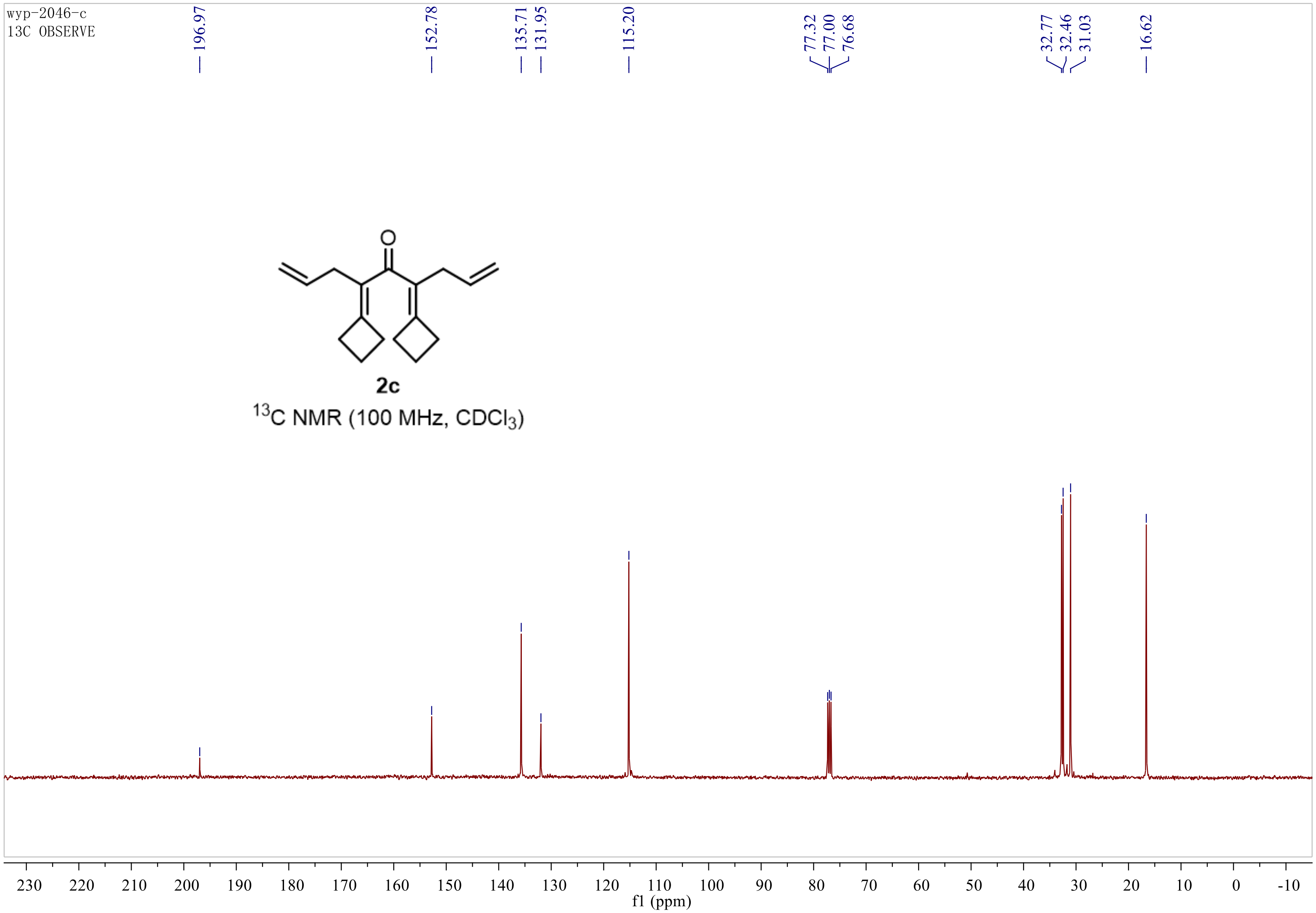

Supplementary Fig. 146. ^{13}C NMR spectra of compound **2c** in $CDCl_3$

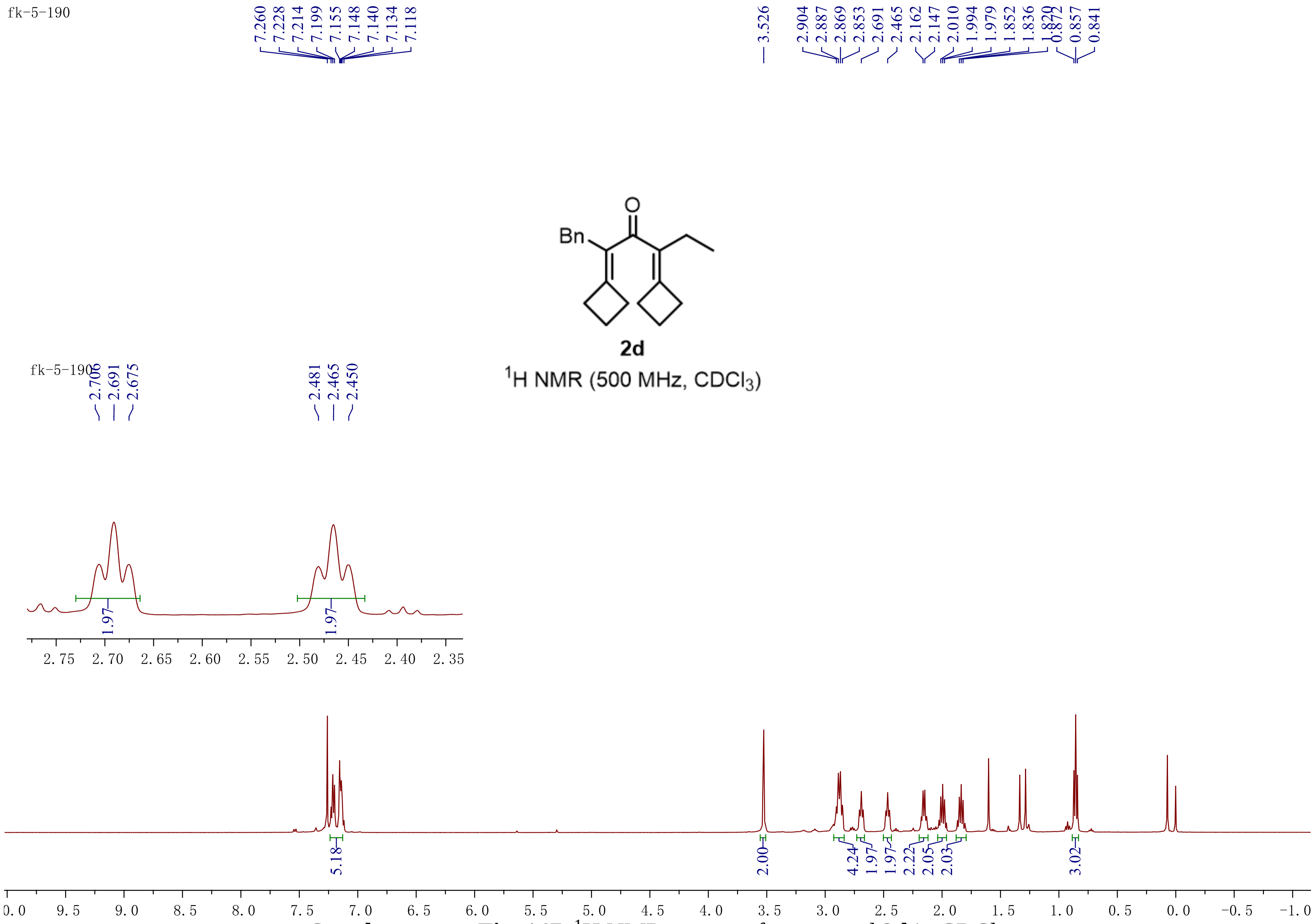

Supplementary Fig. 147. ^{1}H NMR spectra of compound **2d** in $CDCl_3$

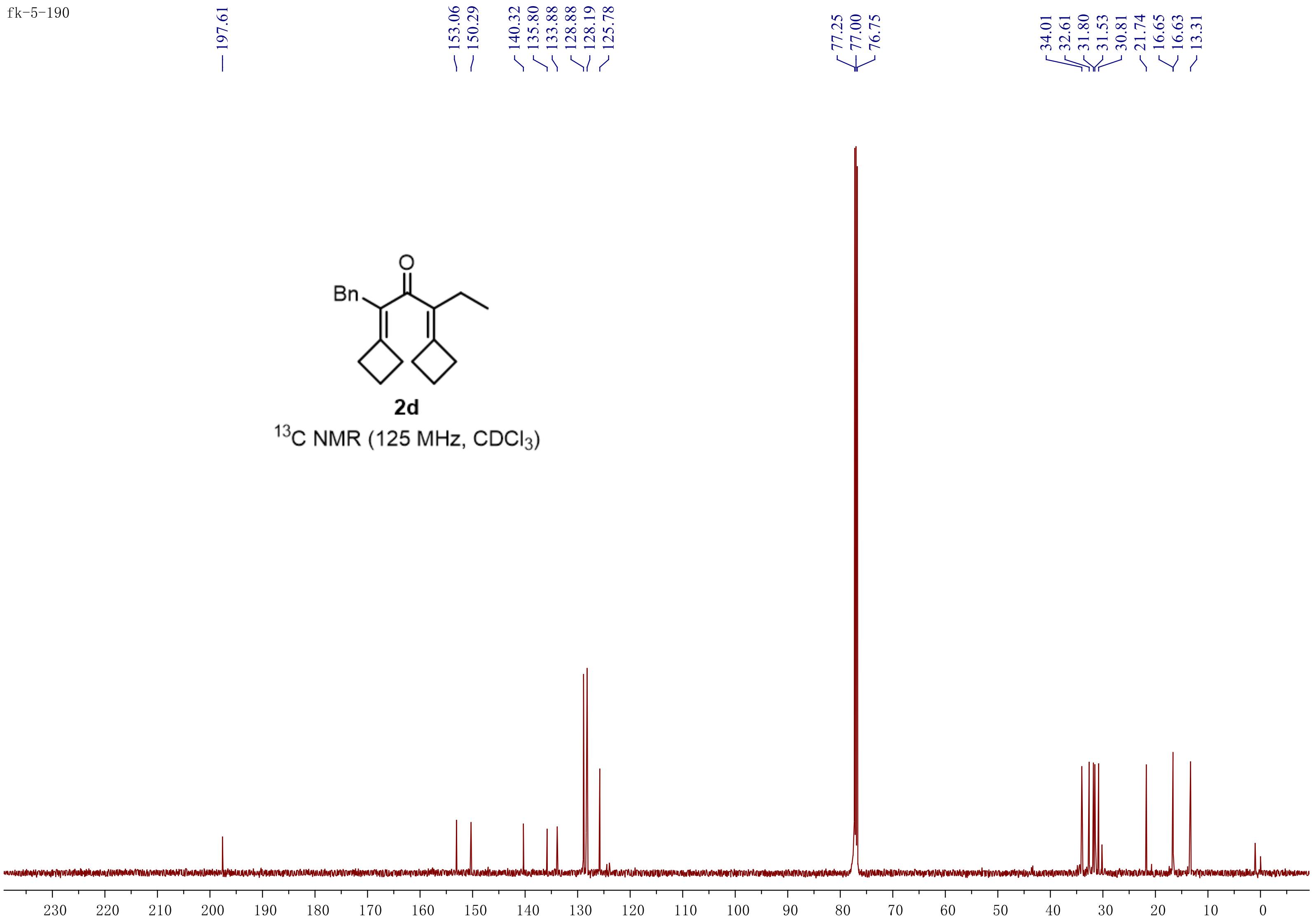

Supplementary Fig. 148. ^{13}C NMR spectra of compound **2d** in $CDCl_3$

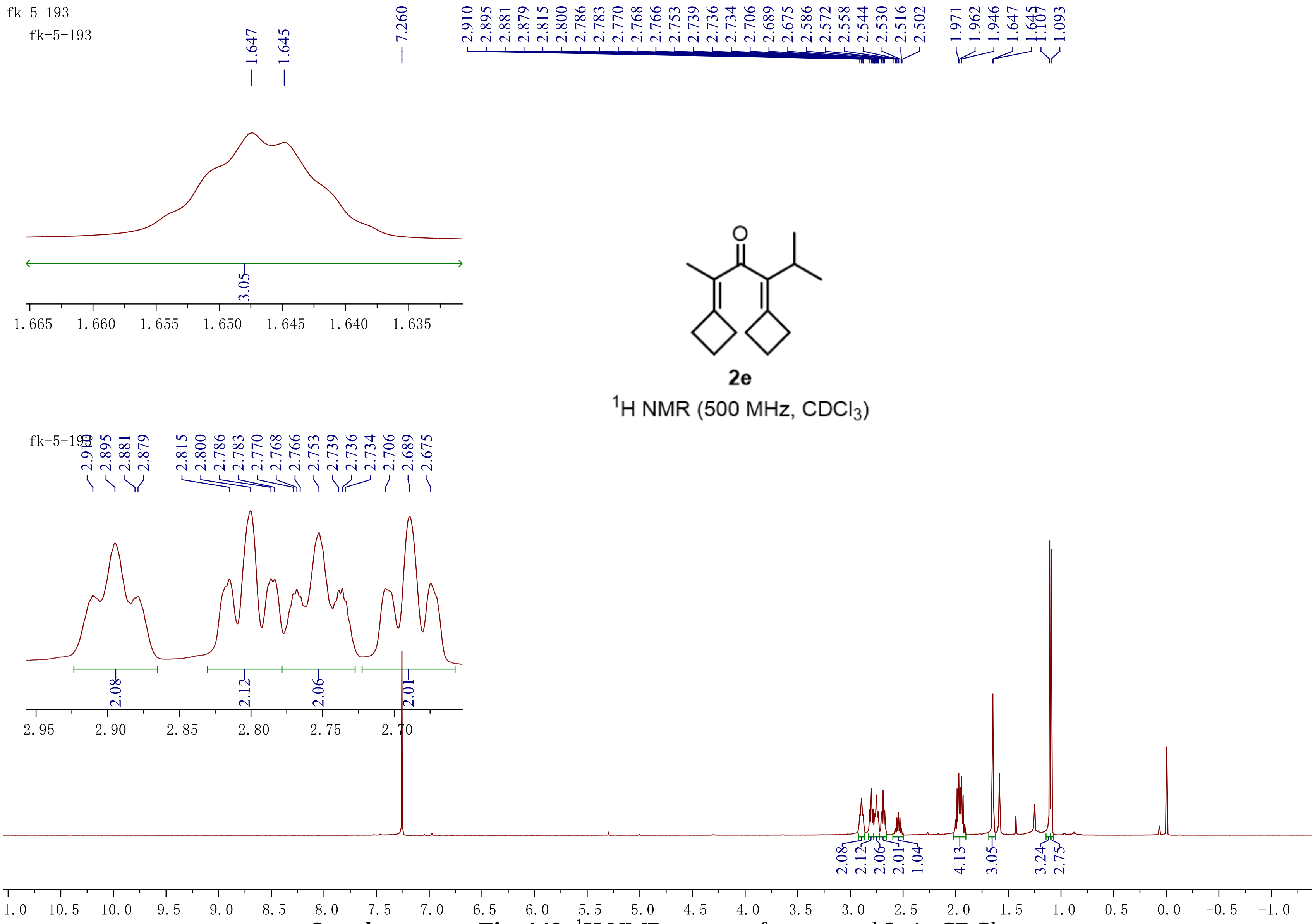

Supplementary Fig. 149. ^{1}H NMR spectra of compound **2e** in $CDCl_3$

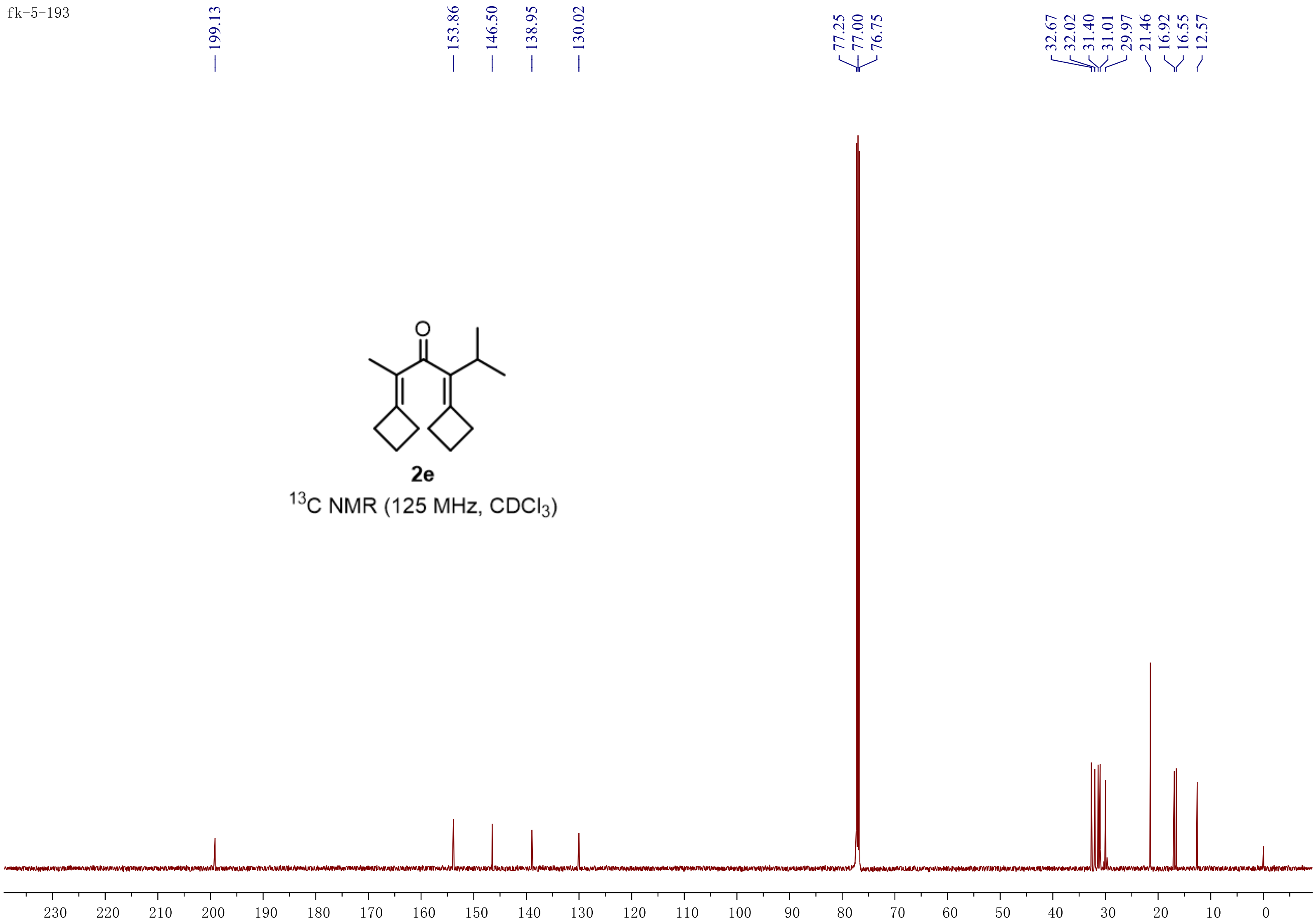

Supplementary Fig. 150. ^{13}C NMR spectra of compound **2e** in $CDCl_3$

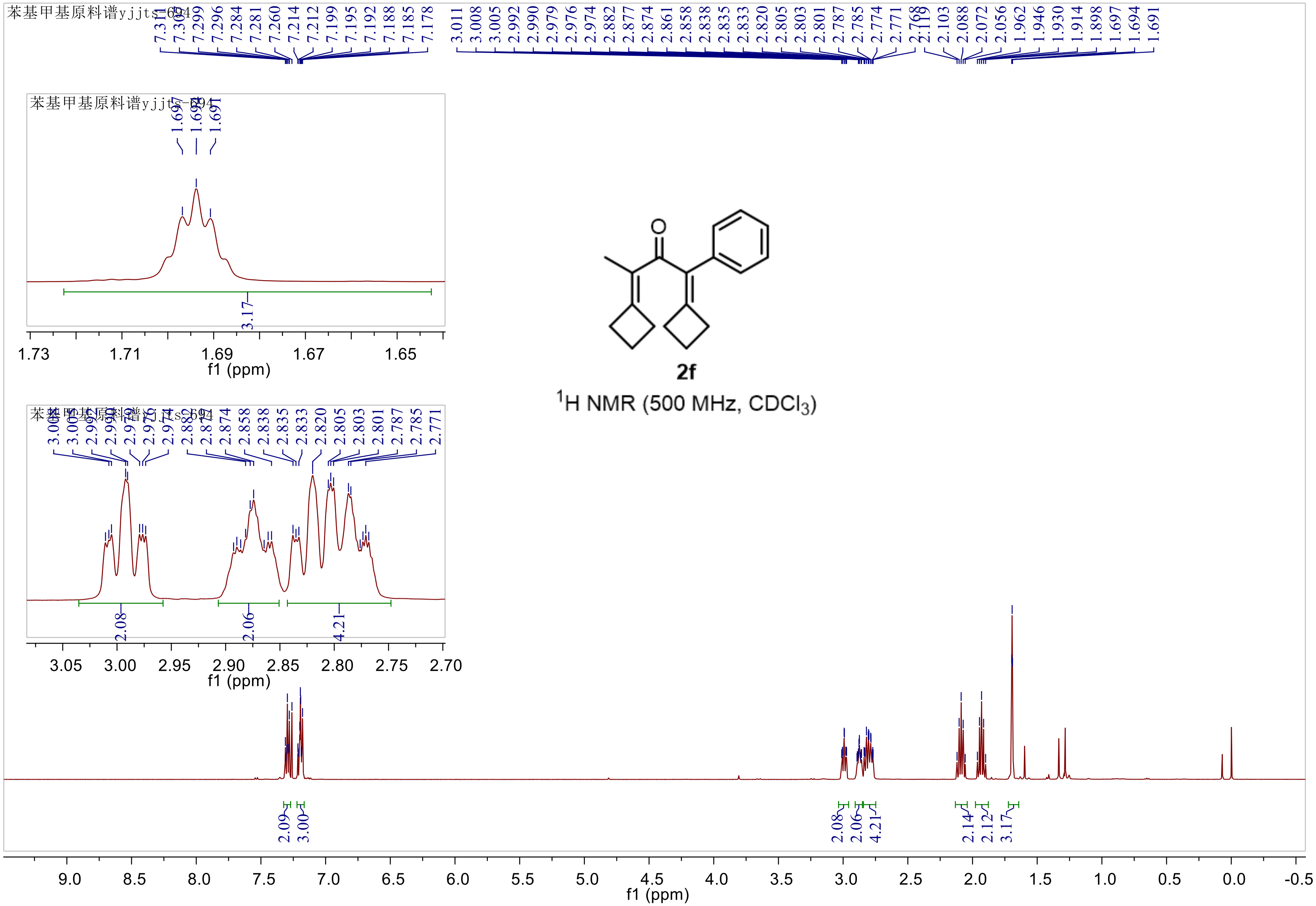

Supplementary Fig. 151. ^{1}H NMR spectra of compound **2f** in $CDCl_3$

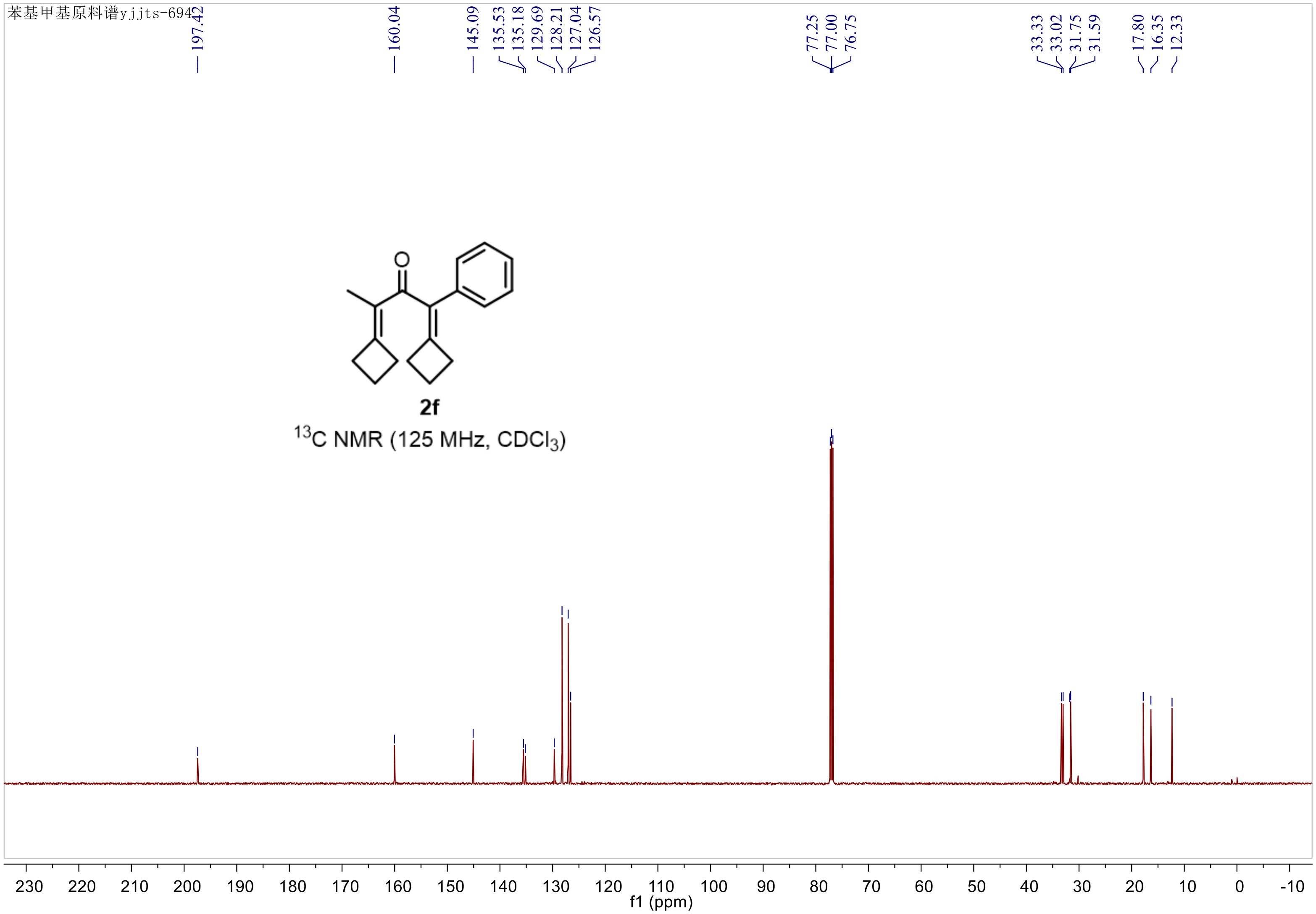

Supplementary Fig. 152. ^{13}C NMR spectra of compound **2f** in $CDCl_3$

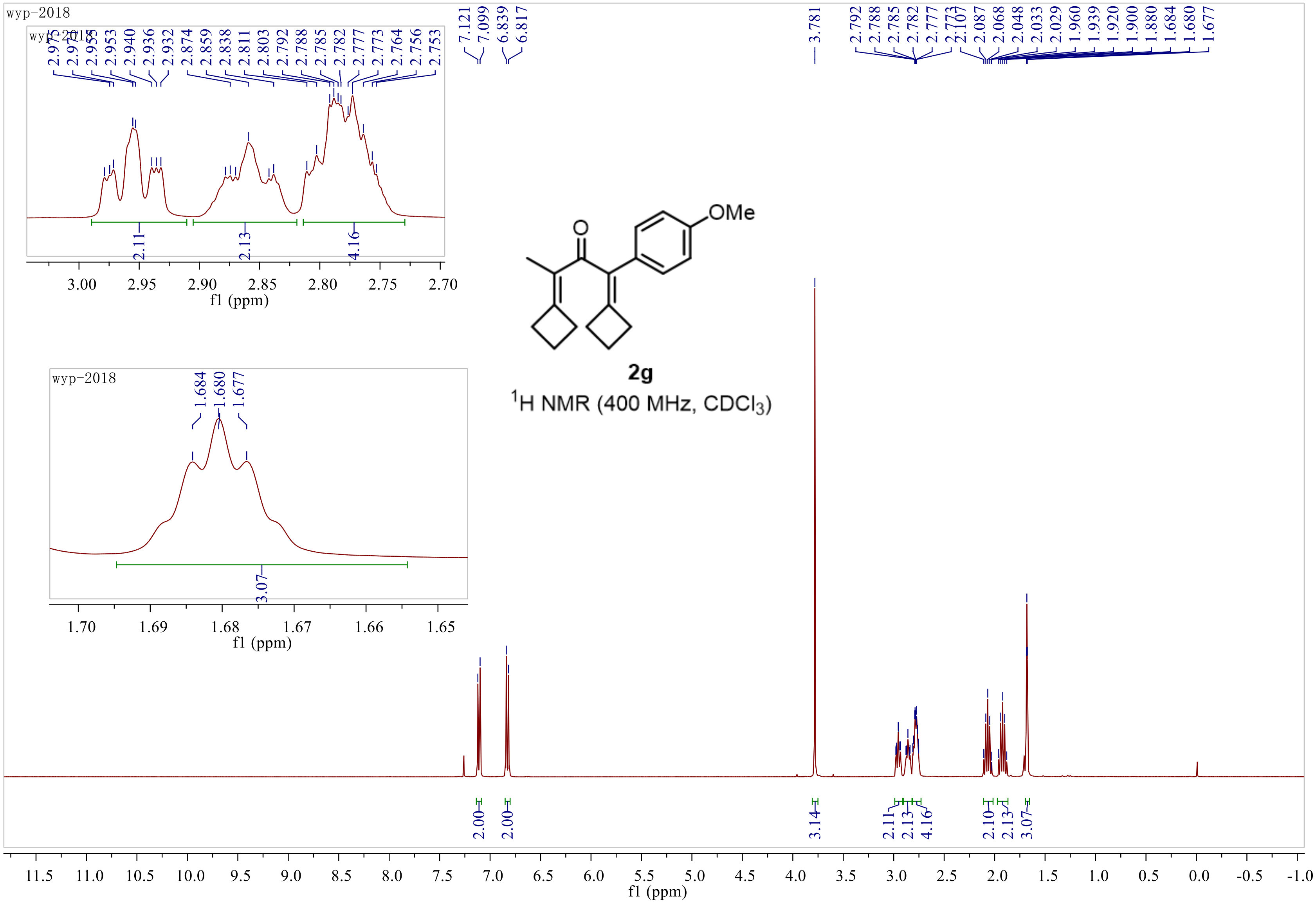

Supplementary Fig. 153. ^{1}H NMR spectra of compound **2g** in $CDCl_3$

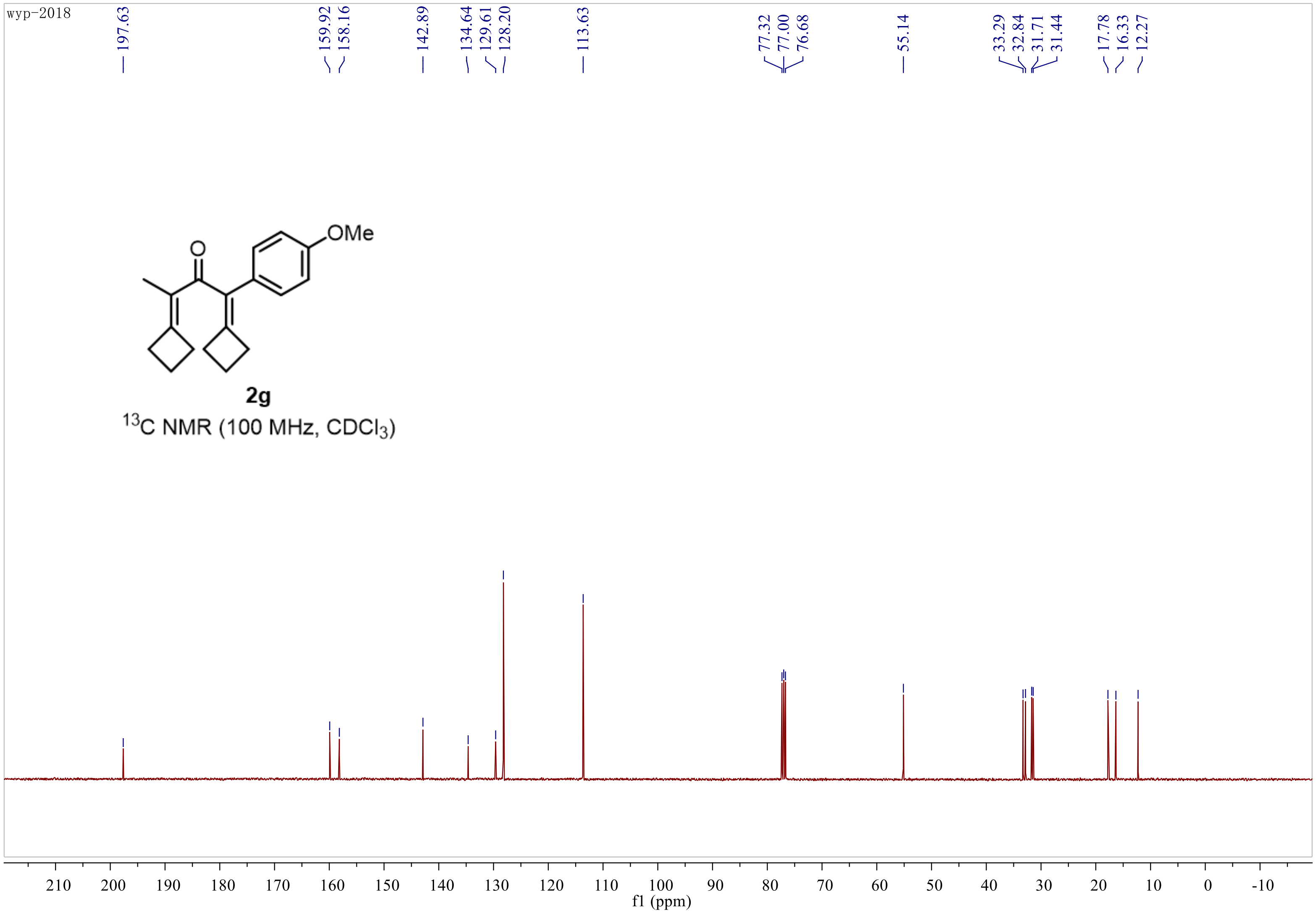

Supplementary Fig. 154. ^{13}C NMR spectra of compound **2g** in $CDCl_3$

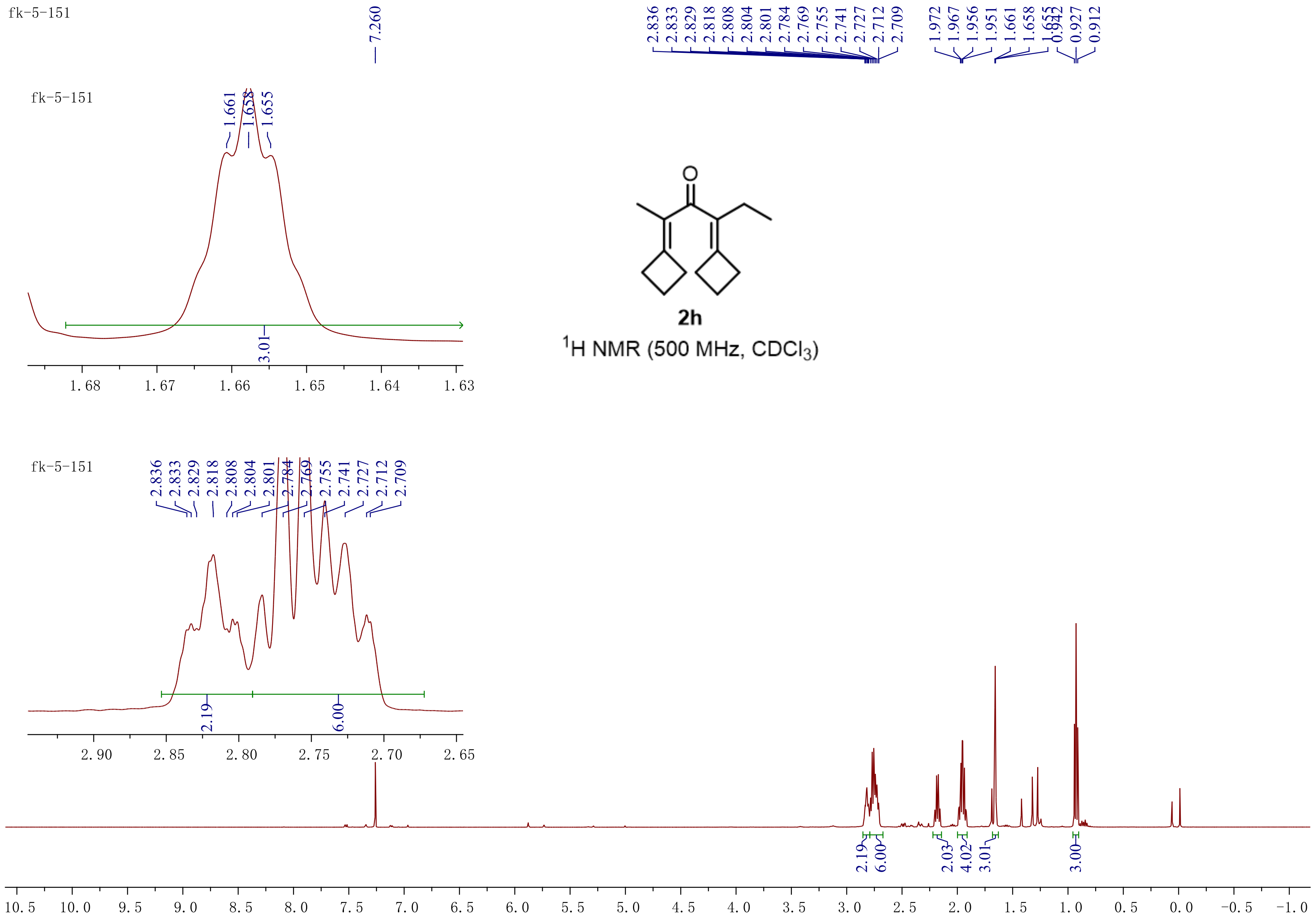

Supplementary Fig. 155. ^{1}H NMR spectra of compound **2h** in $CDCl_3$

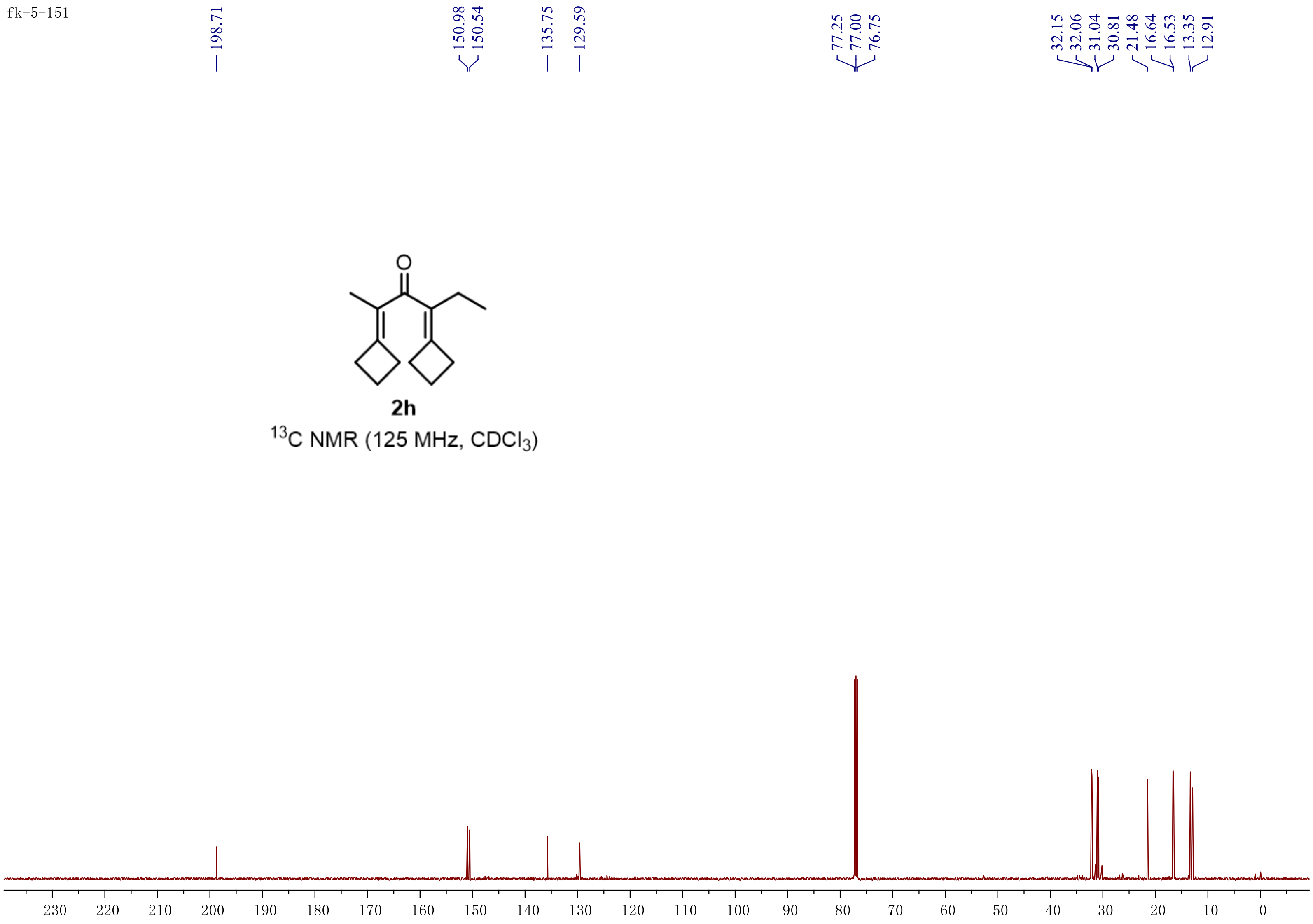

Supplementary Fig. 156. ^{13}C NMR spectra of compound **2h** in $CDCl_3$

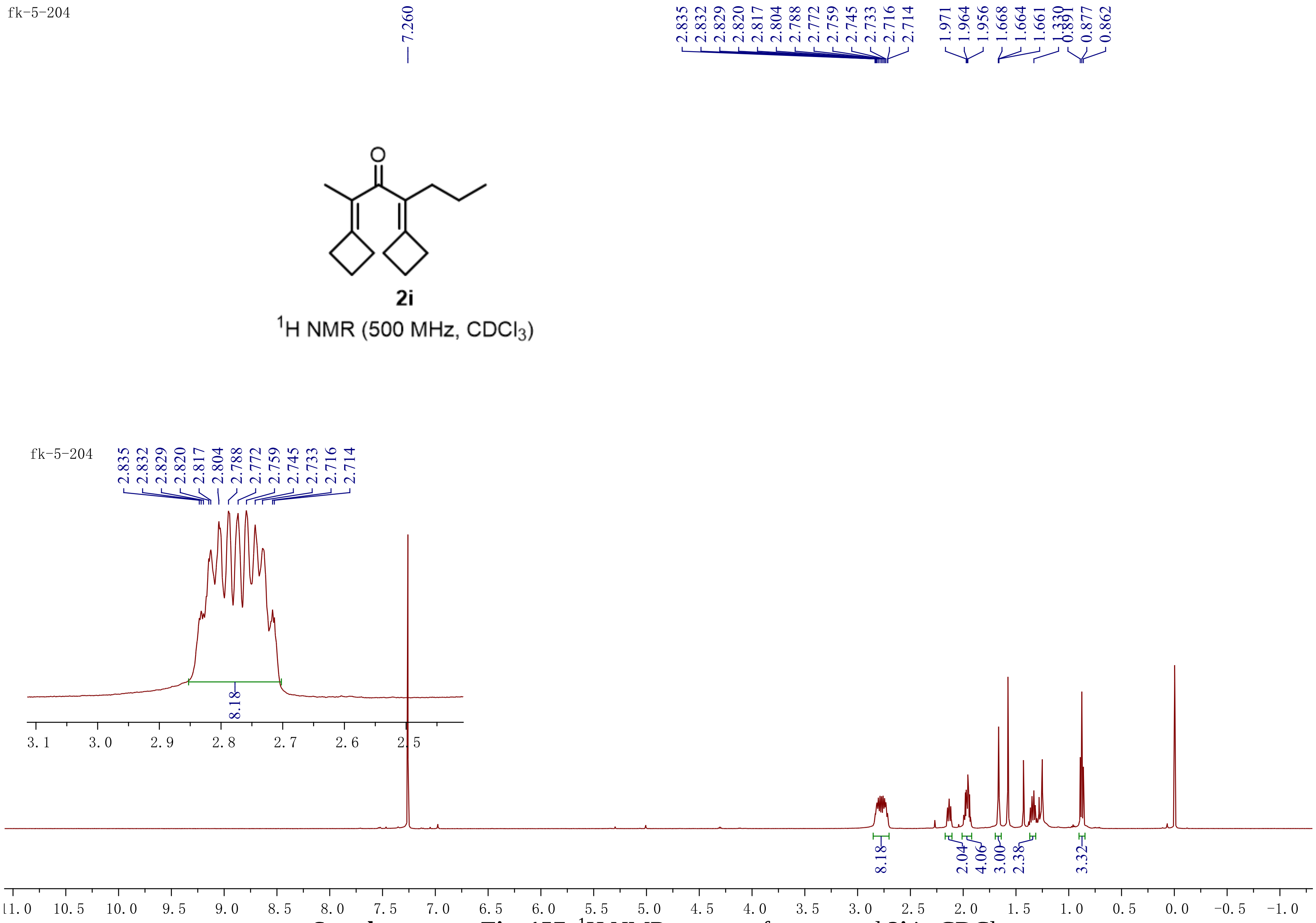

Supplementary Fig. 157. ^{1}H NMR spectra of compound **2i** in $CDCl_3$

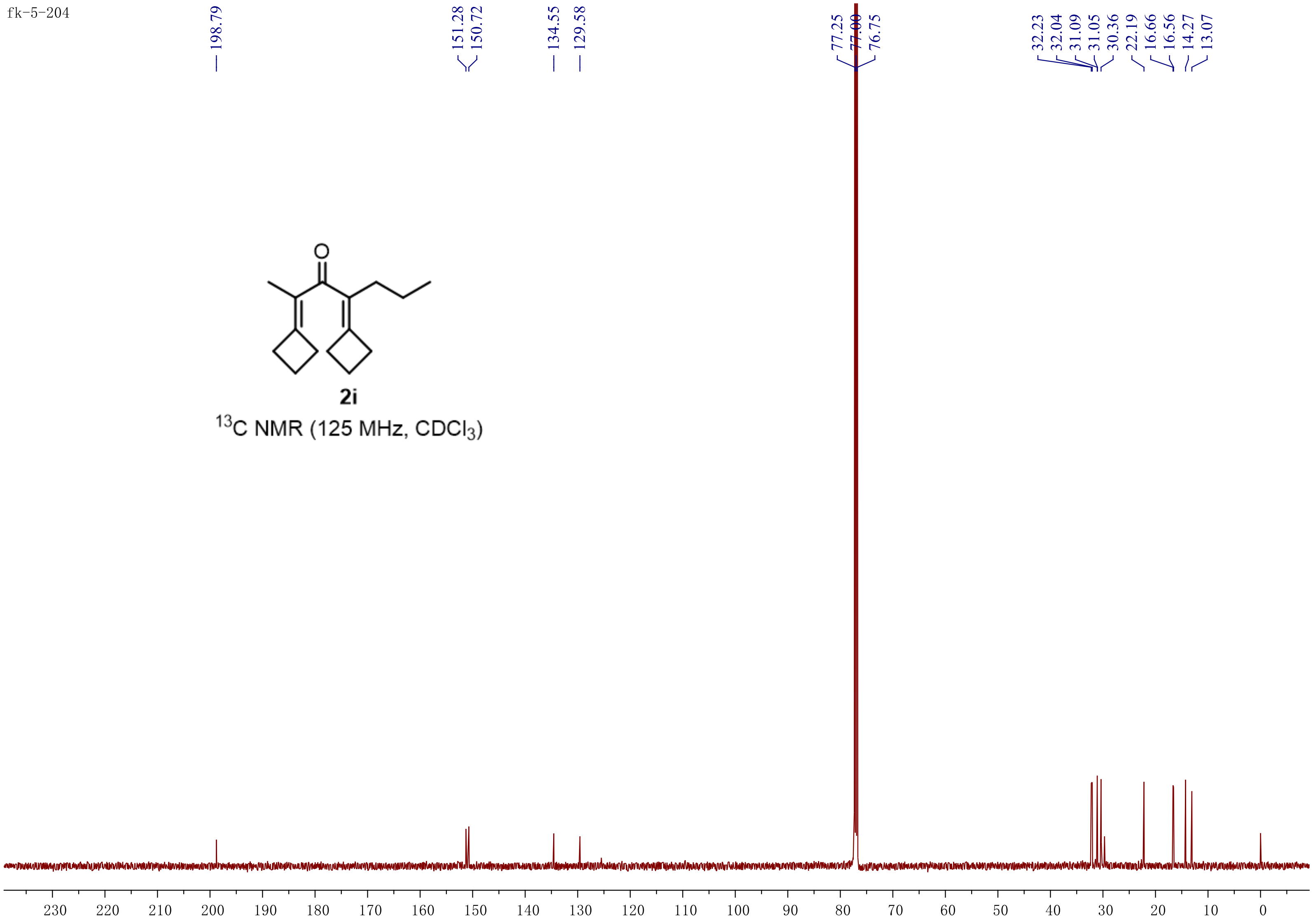

Supplementary Fig. 158. ^{13}C NMR spectra of compound **2i** in $CDCl_3$

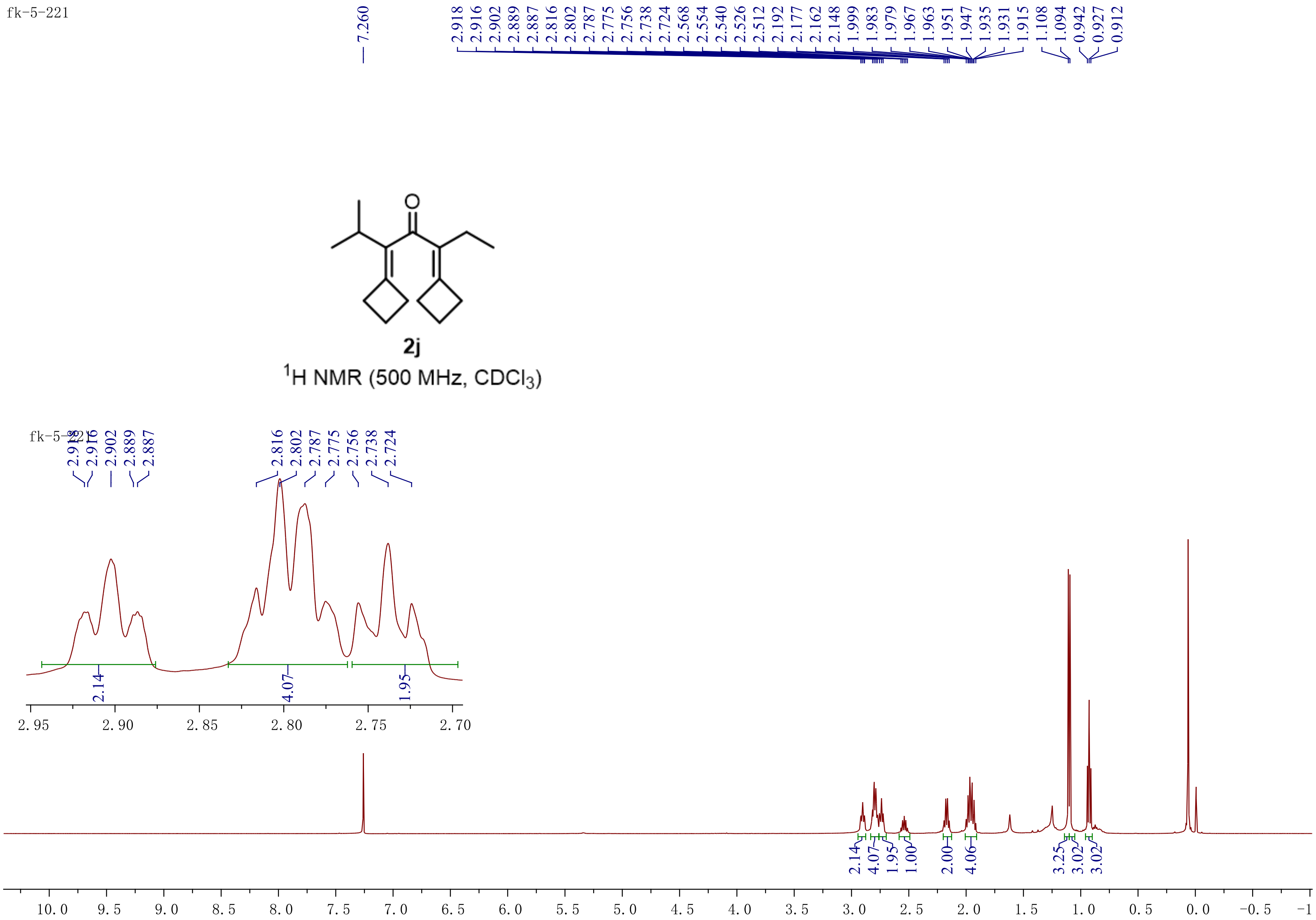

Supplementary Fig. 159. ^{1}H NMR spectra of compound **2j** in $CDCl_3$

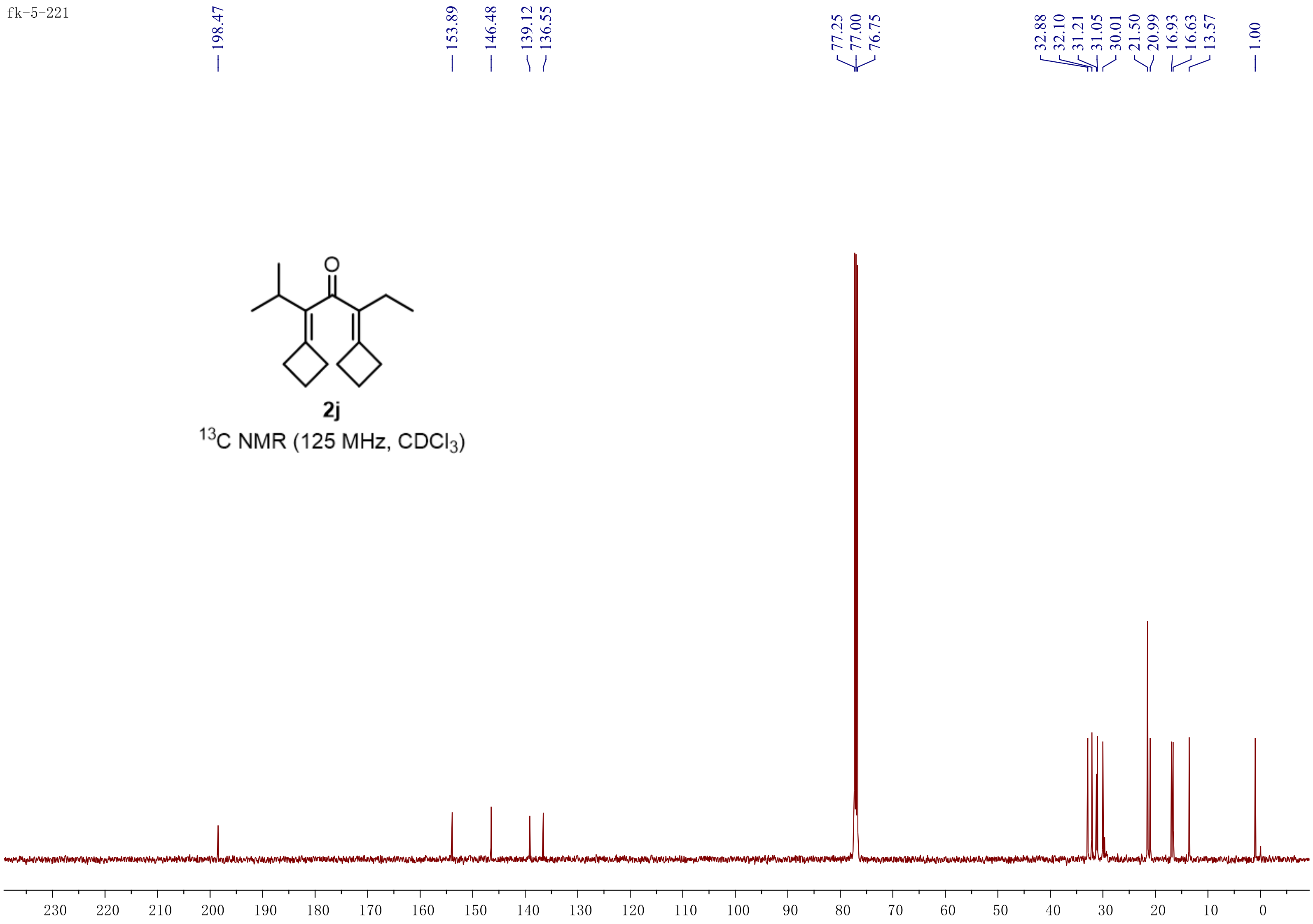

Supplementary Fig. 160. ^{13}C NMR spectra of compound **2j** in $CDCl_3$

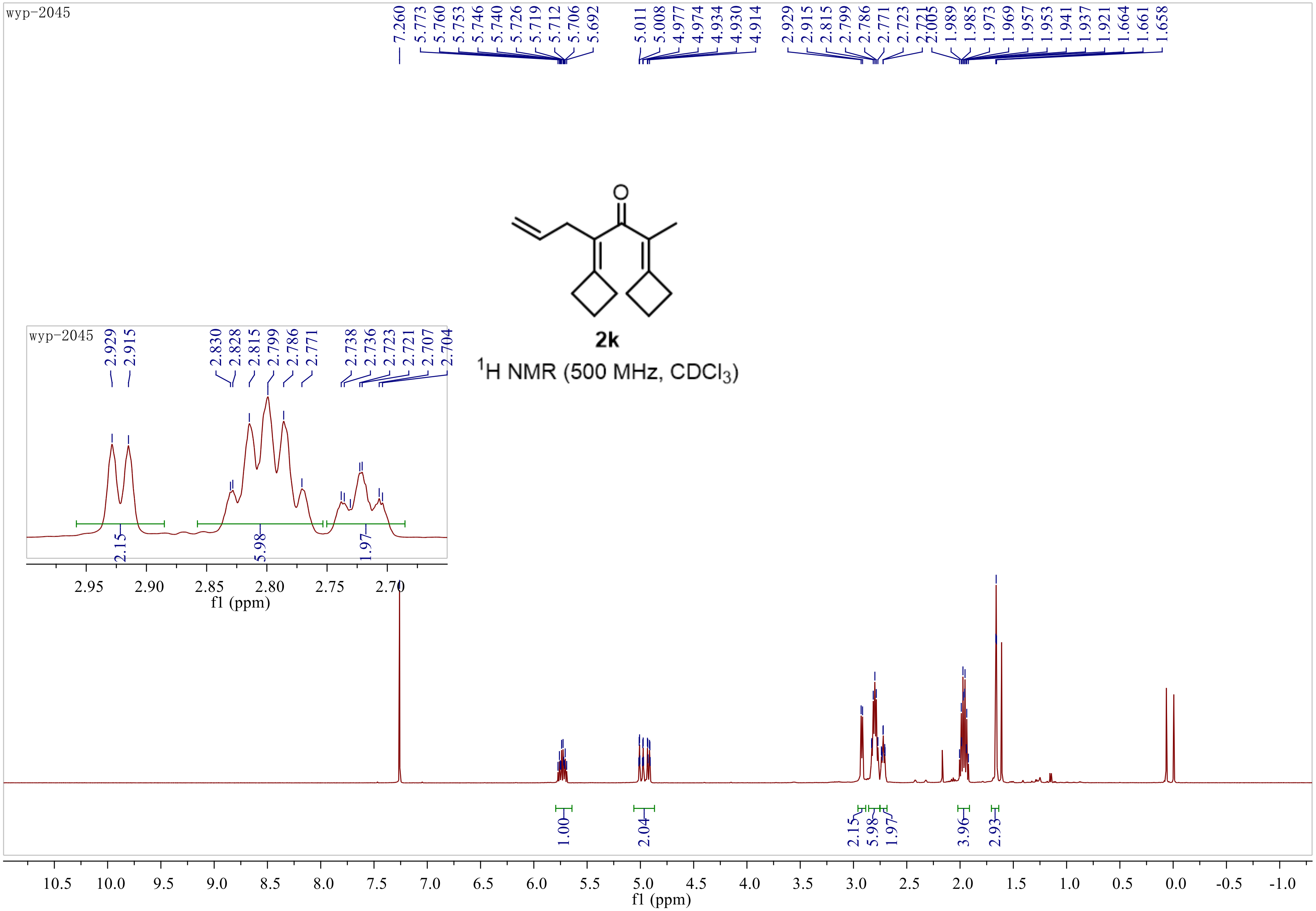

Supplementary Fig. 161. ^{1}H NMR spectra of compound **2k** in $CDCl_3$

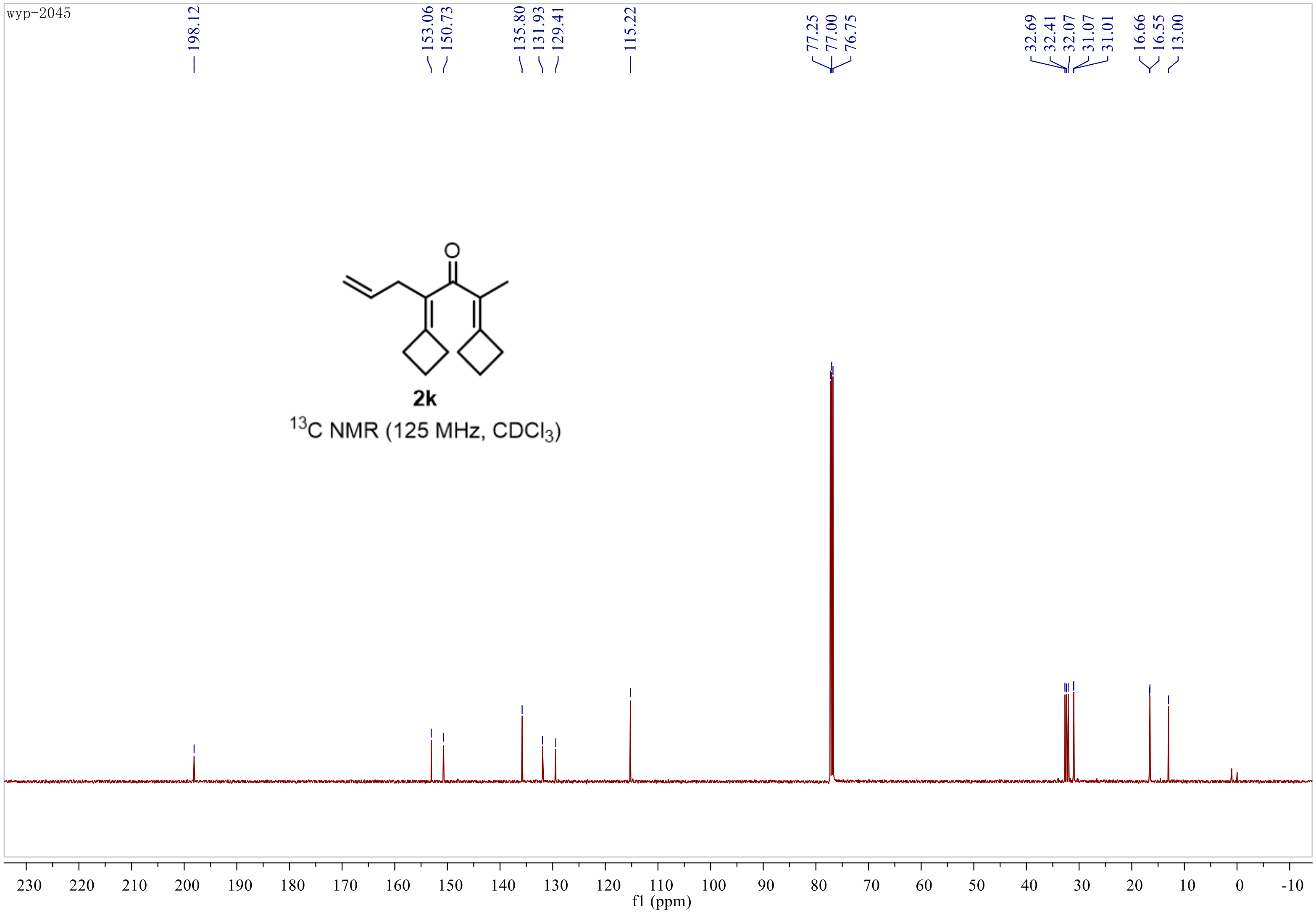

Supplementary Fig. 162. ^{13}C NMR spectra of compound **2k** in $CDCl_3$

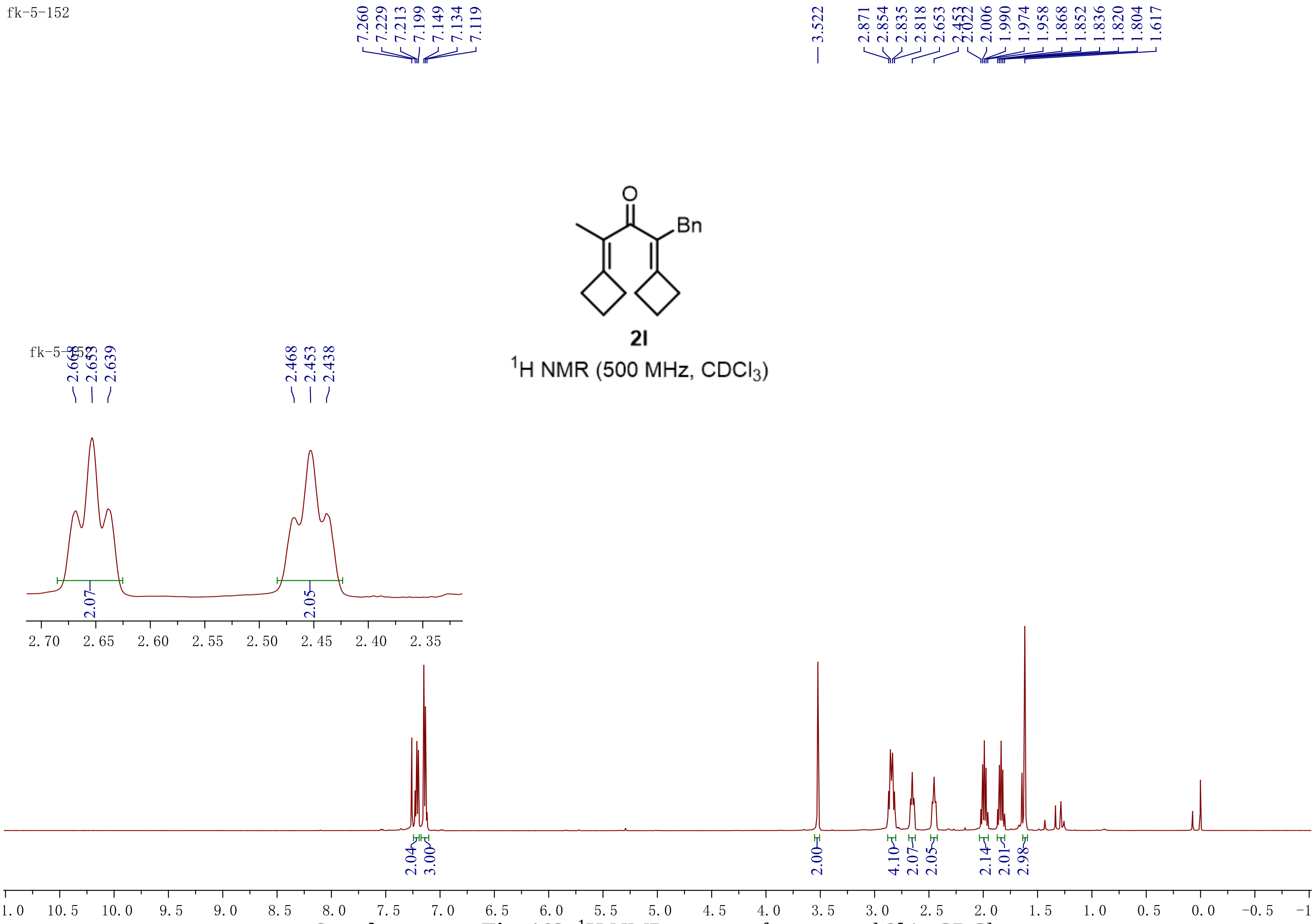

Supplementary Fig. 163. ^{1}H NMR spectra of compound **2l** in $CDCl_3$

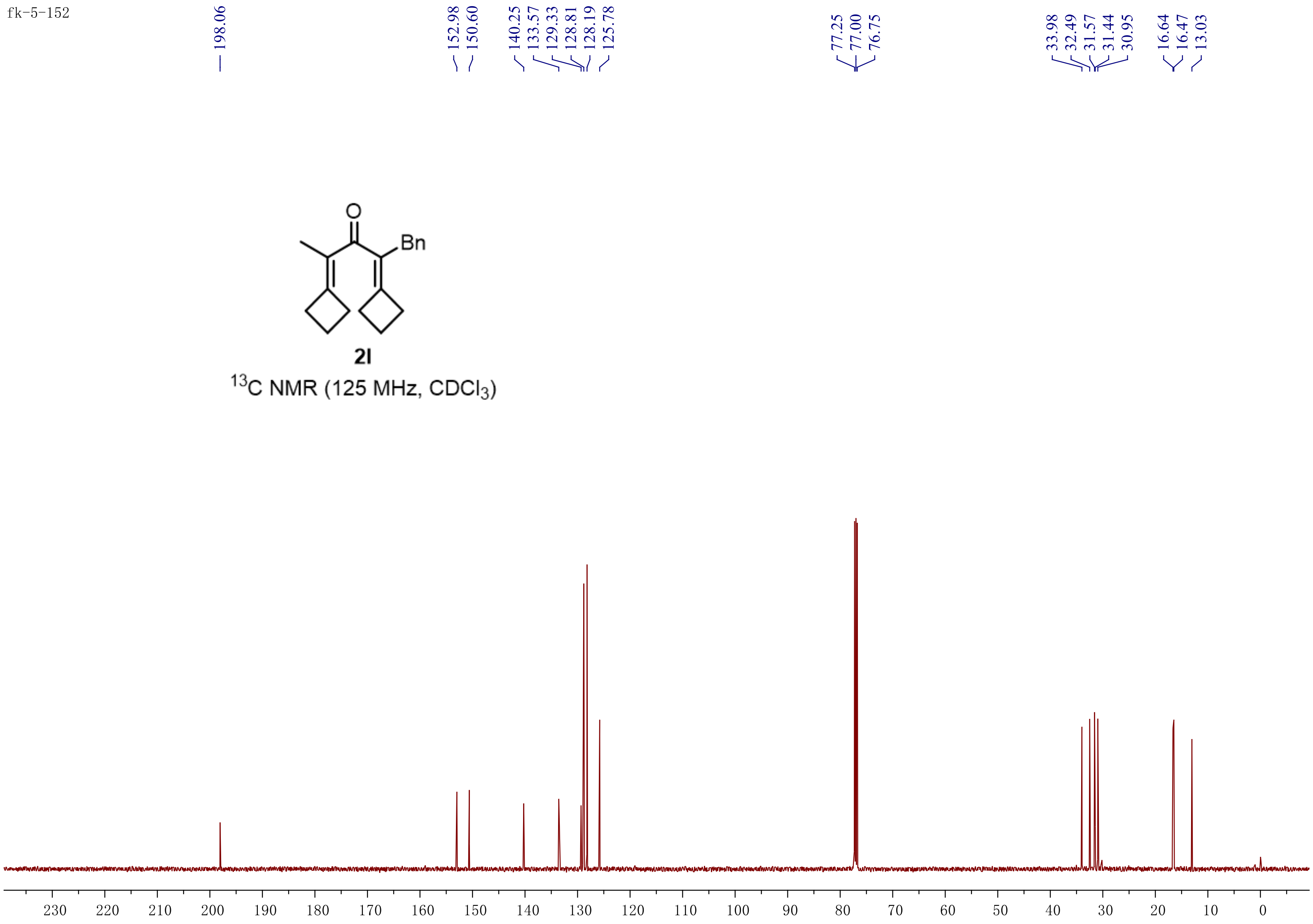

Supplementary Fig. 164. ^{13}C NMR spectra of compound **2l** in $CDCl_3$

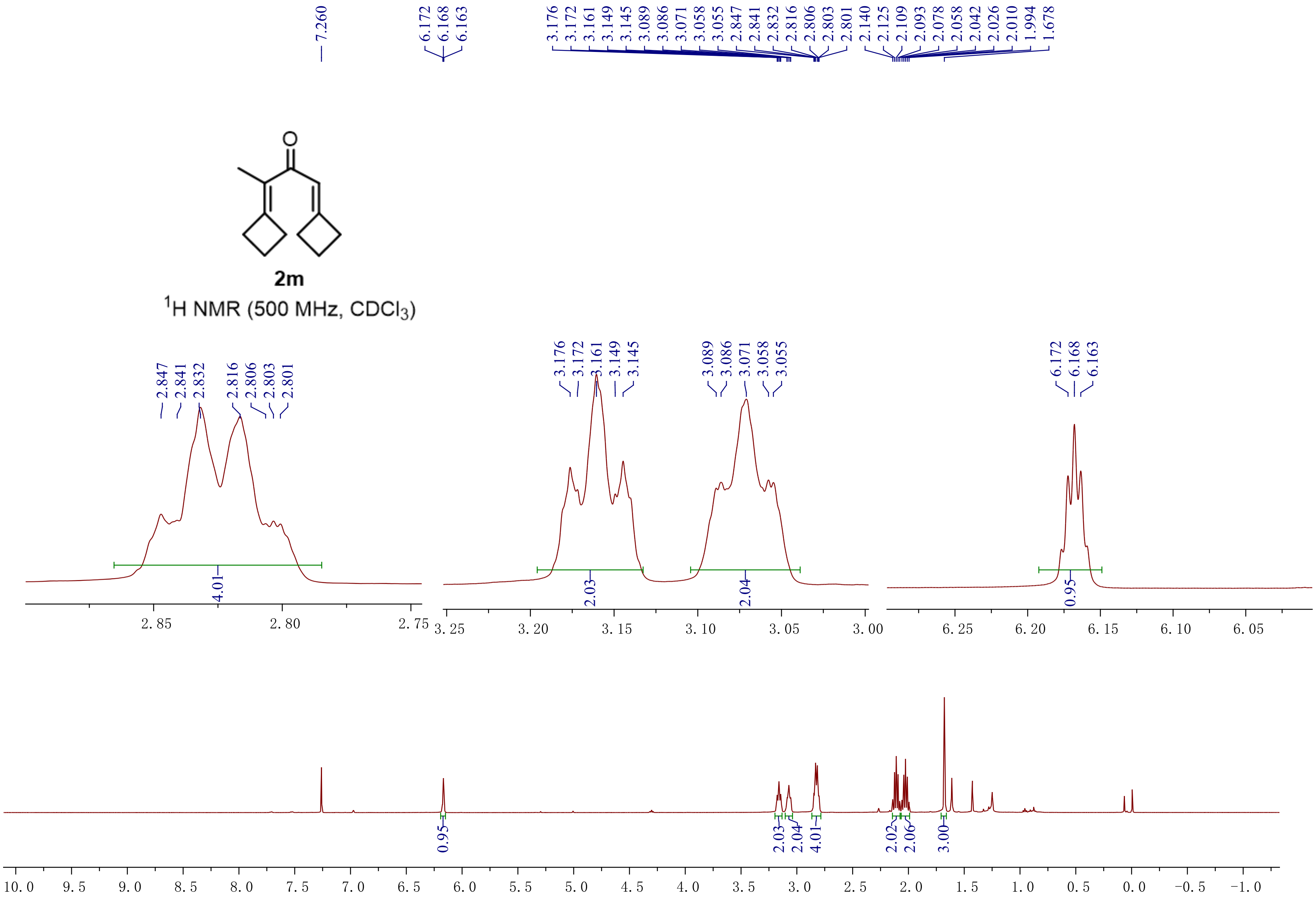

Supplementary Fig. 165. ^{1}H NMR spectra of compound **2m** in $CDCl_3$

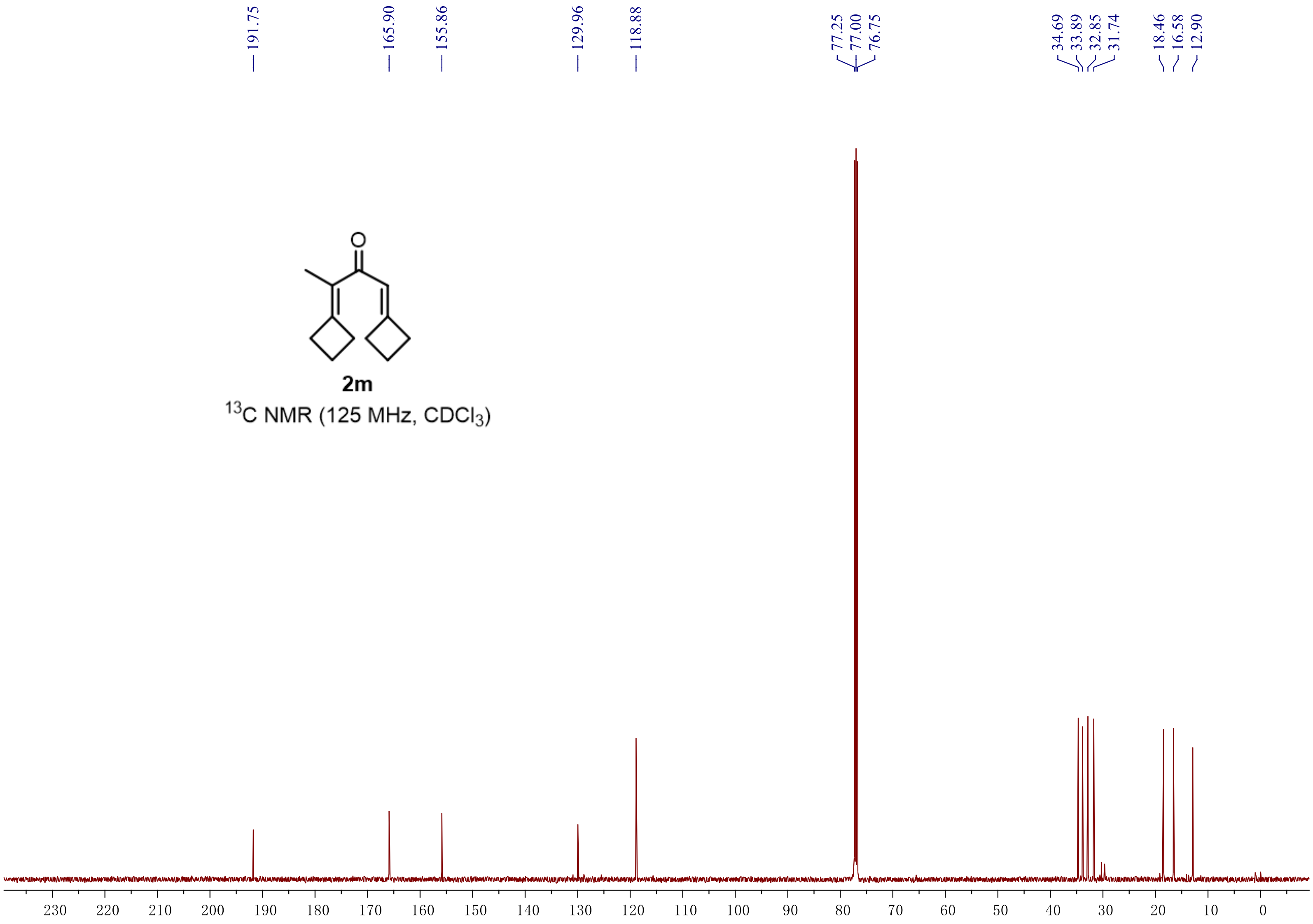

Supplementary Fig. 166. ^{13}C NMR spectra of compound **2m** in $CDCl_3$

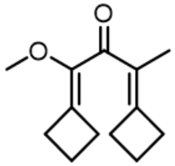

2n

^{1}H NMR (400 MHz, $CDCl_3$)

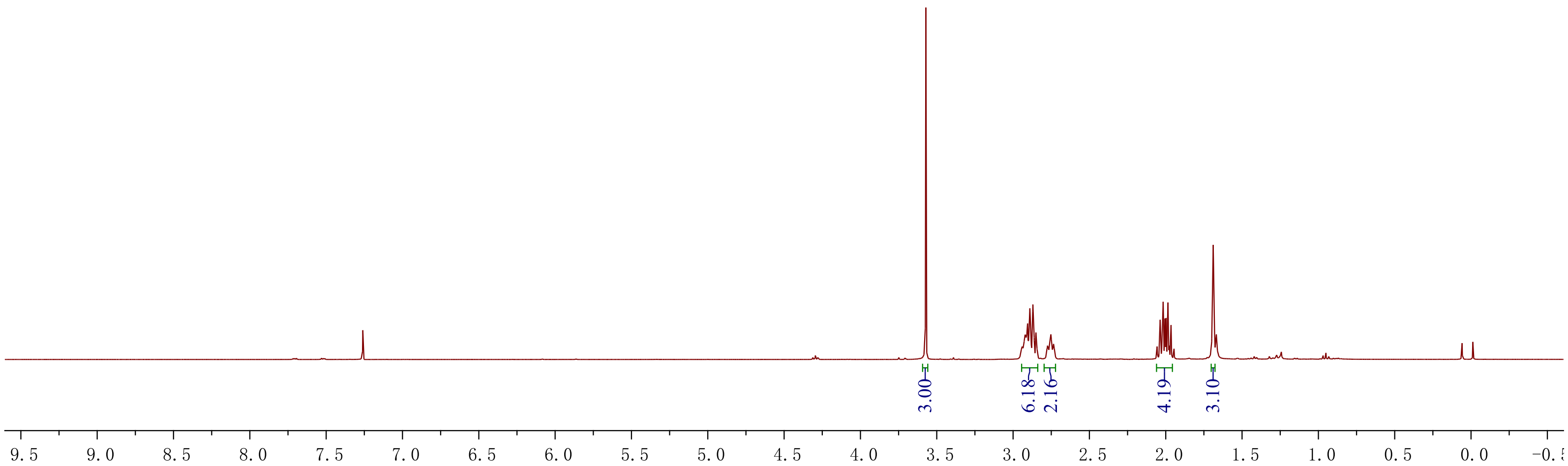

Supplementary Fig. 167. ^{1}H NMR spectra of compound **2n** in $CDCl_3$

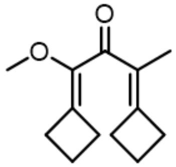

2n

^{13}C NMR (100 MHz, $CDCl_3$)

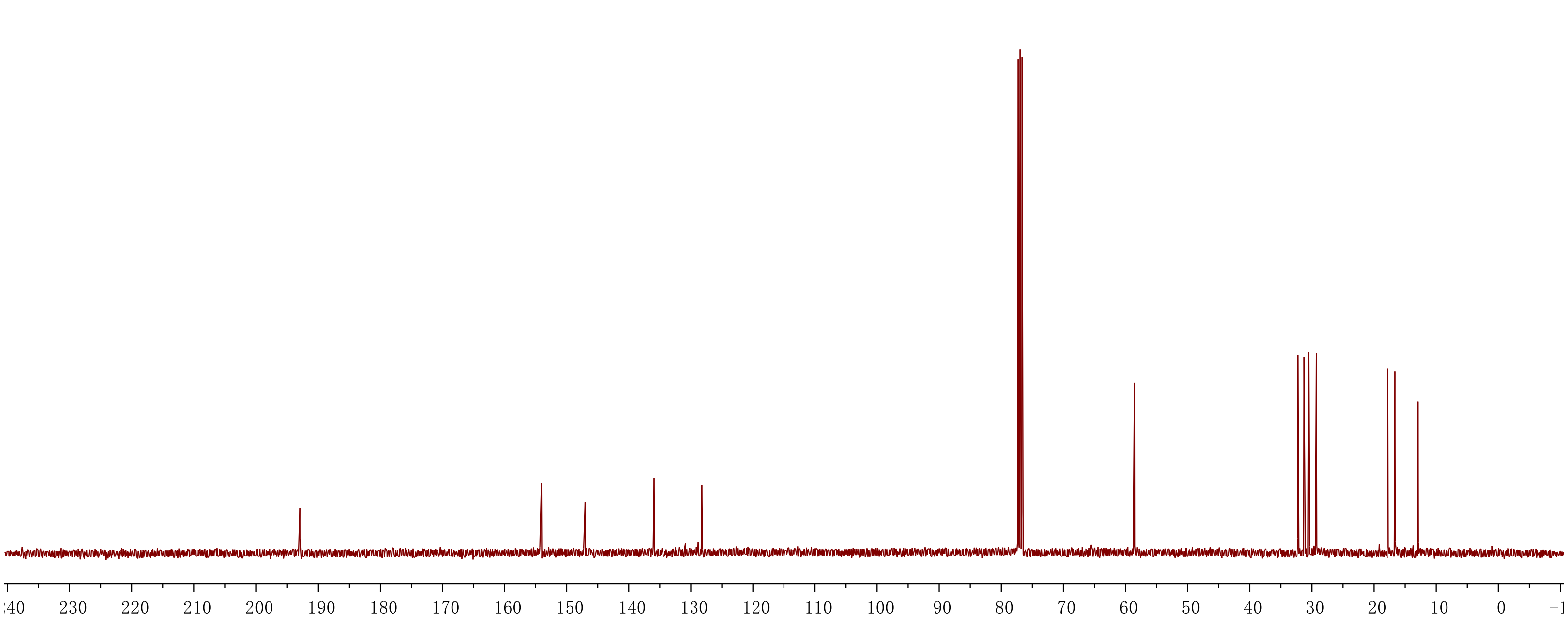

Supplementary Fig. 168. ^{13}C NMR spectra of compound **2n** in $CDCl_3$

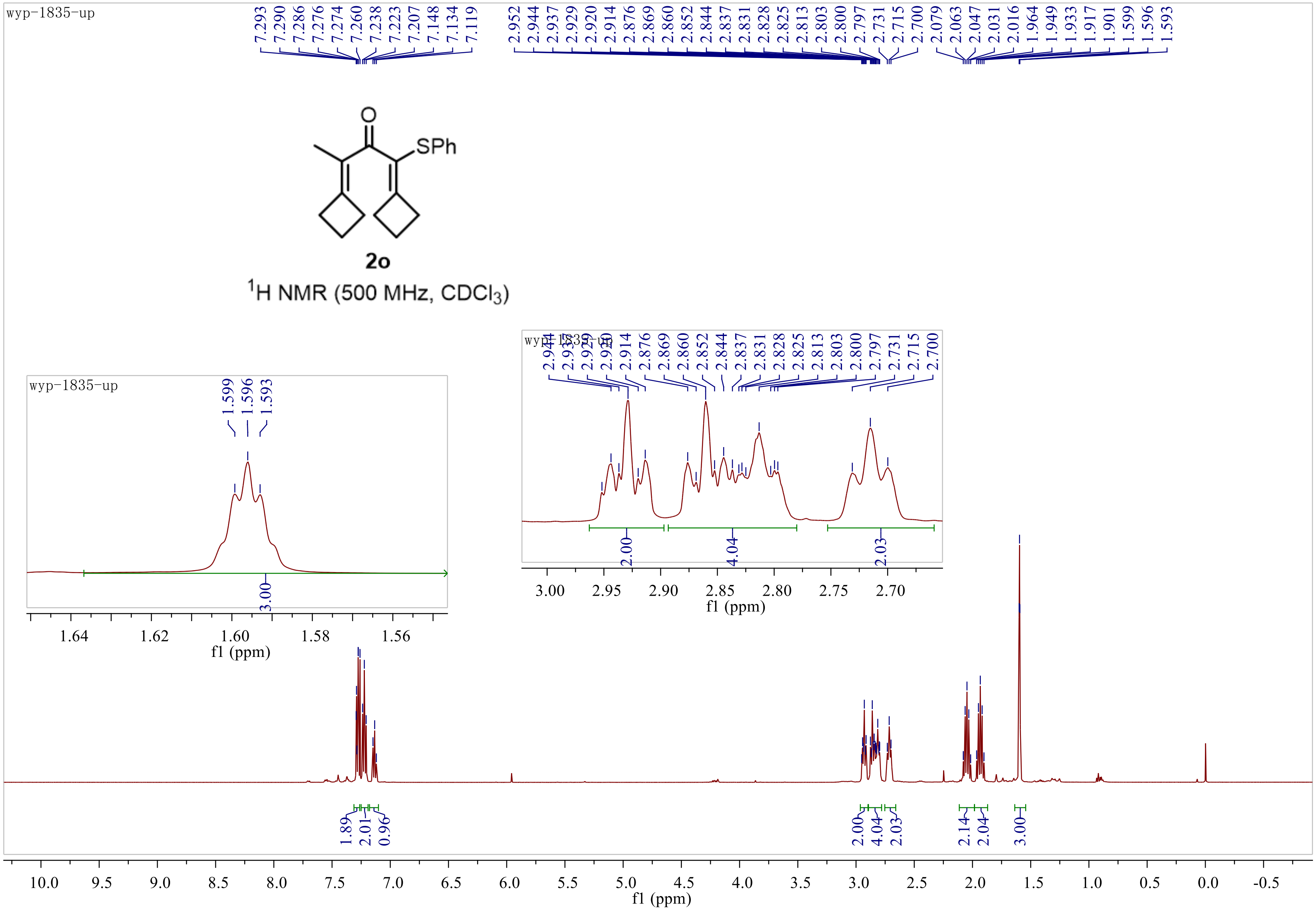

Supplementary Fig. 169. ^{1}H NMR spectra of compound **2o** in $CDCl_3$

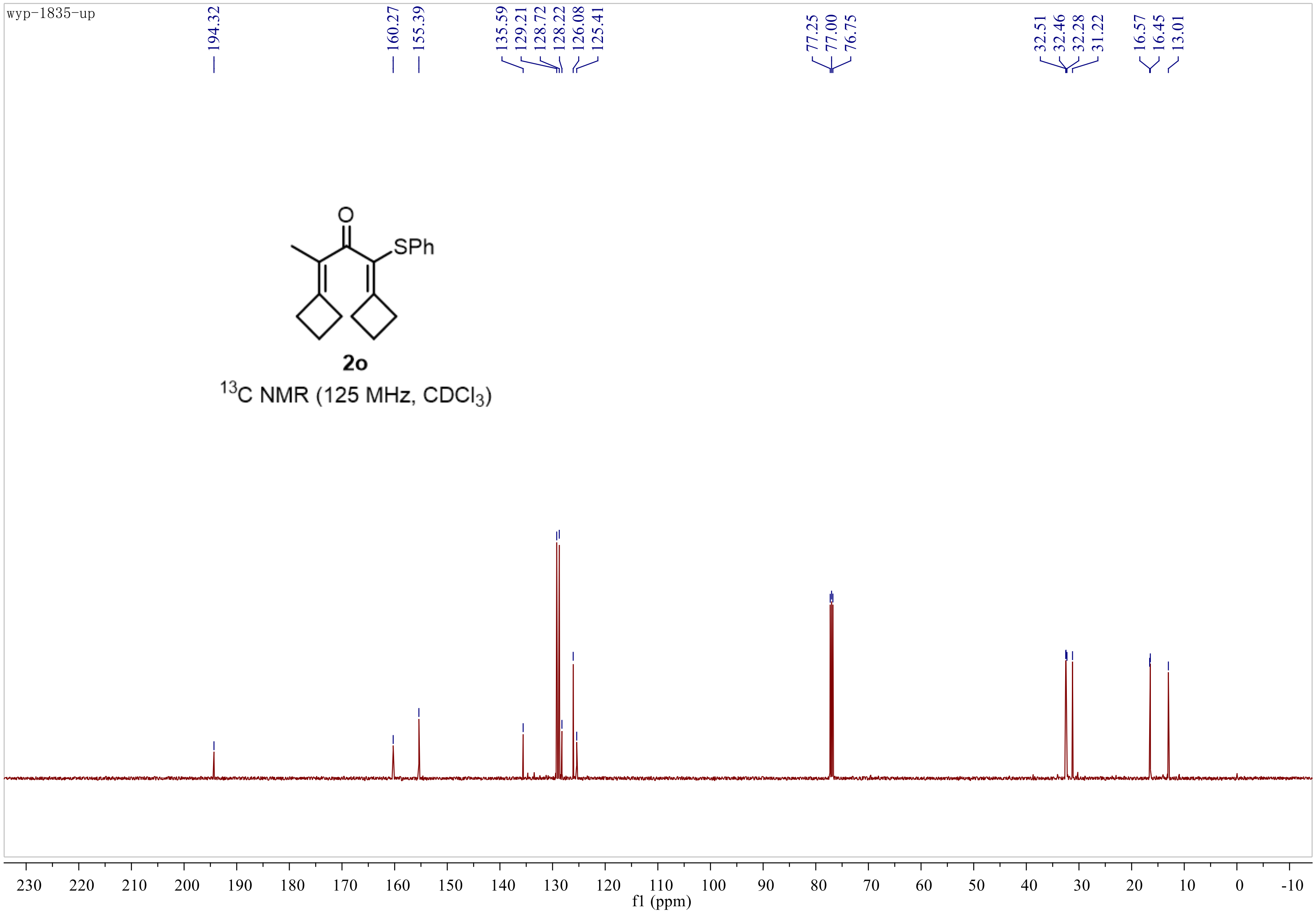

Supplementary Fig. 170. ^{13}C NMR spectra of compound **2o** in $CDCl_3$

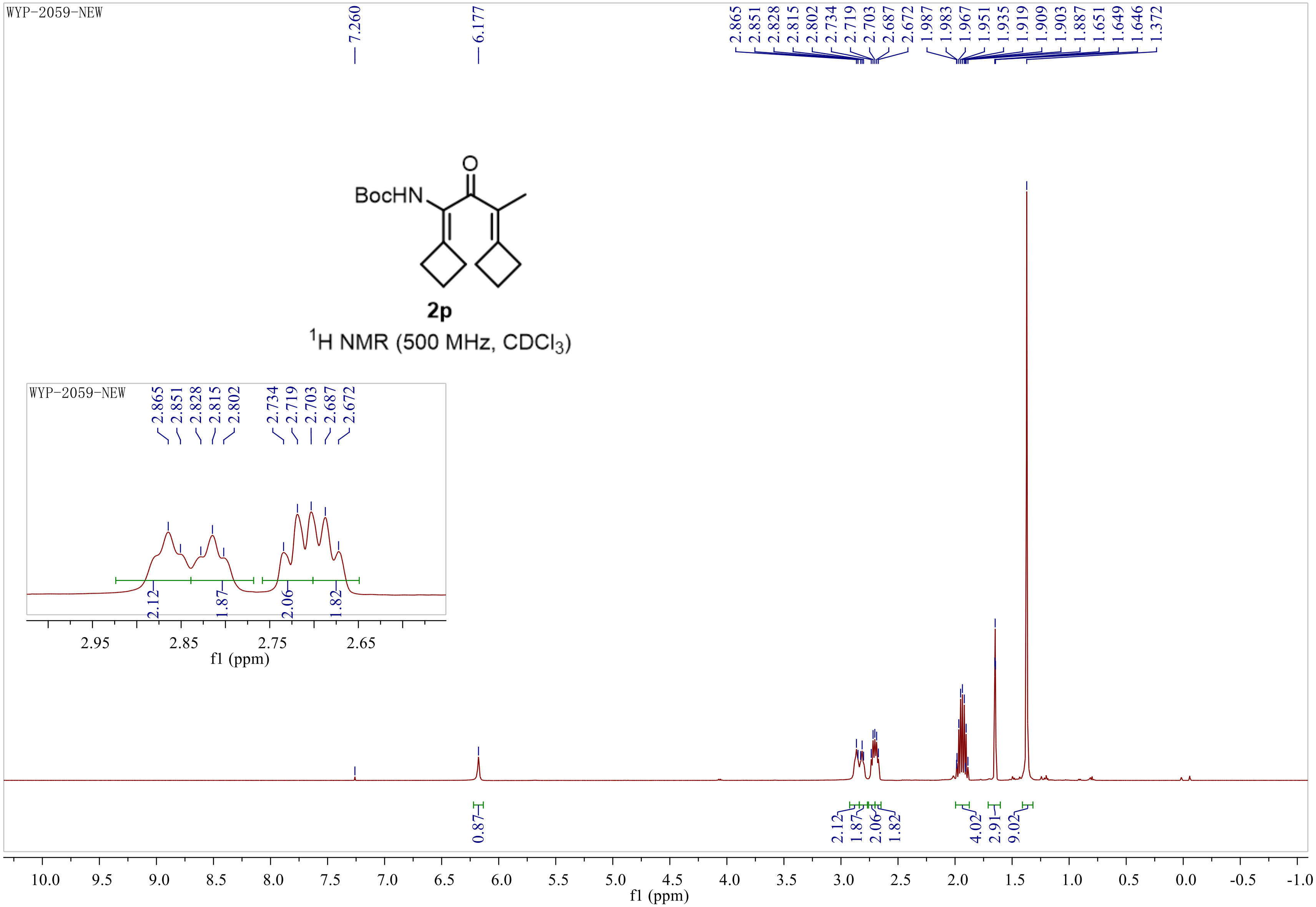

Supplementary Fig. 171. ^{1}H NMR spectra of compound **2p** in $CDCl_3$

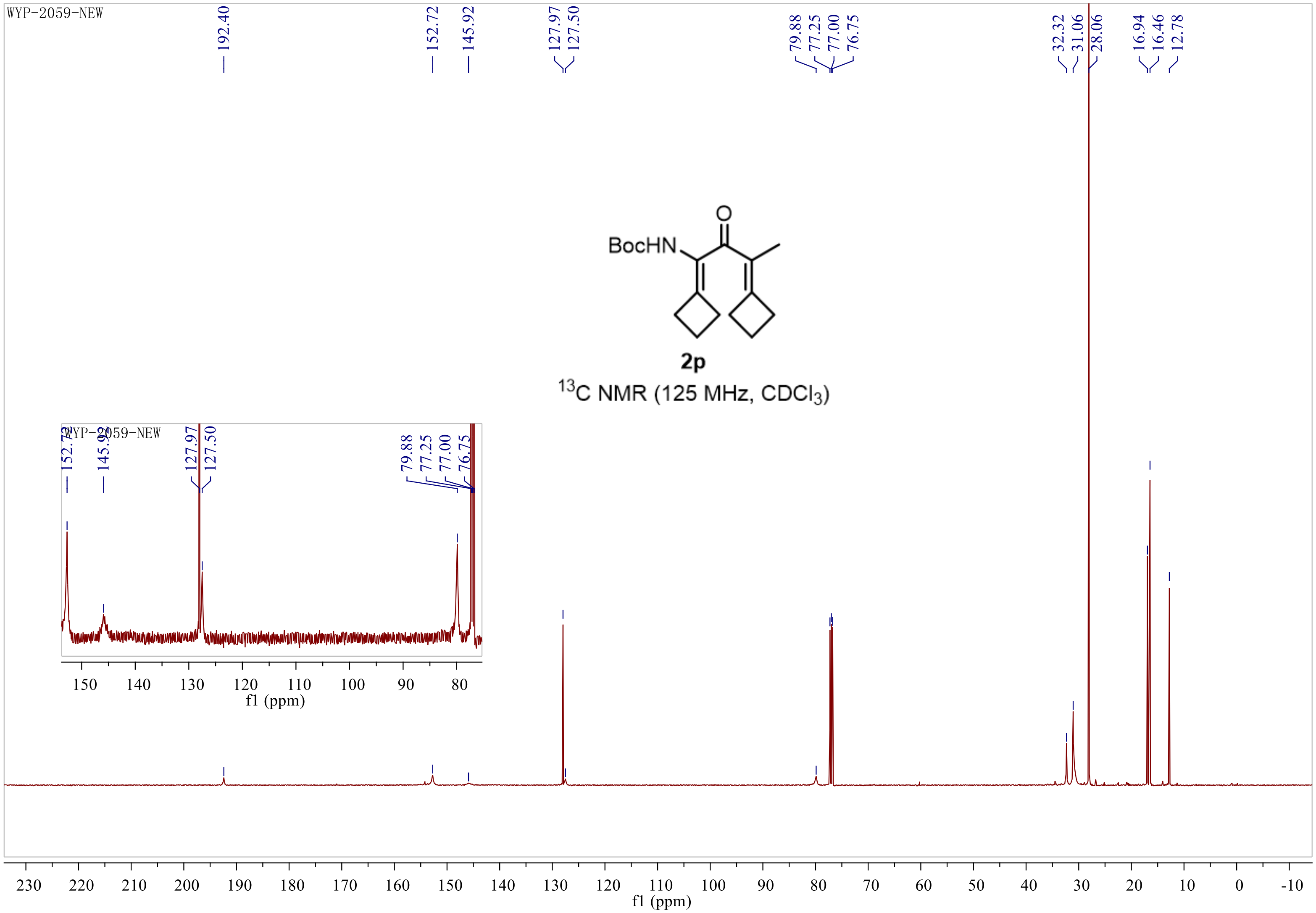

Supplementary Fig. 172. ^{13}C NMR spectra of compound **2p** in $CDCl_3$

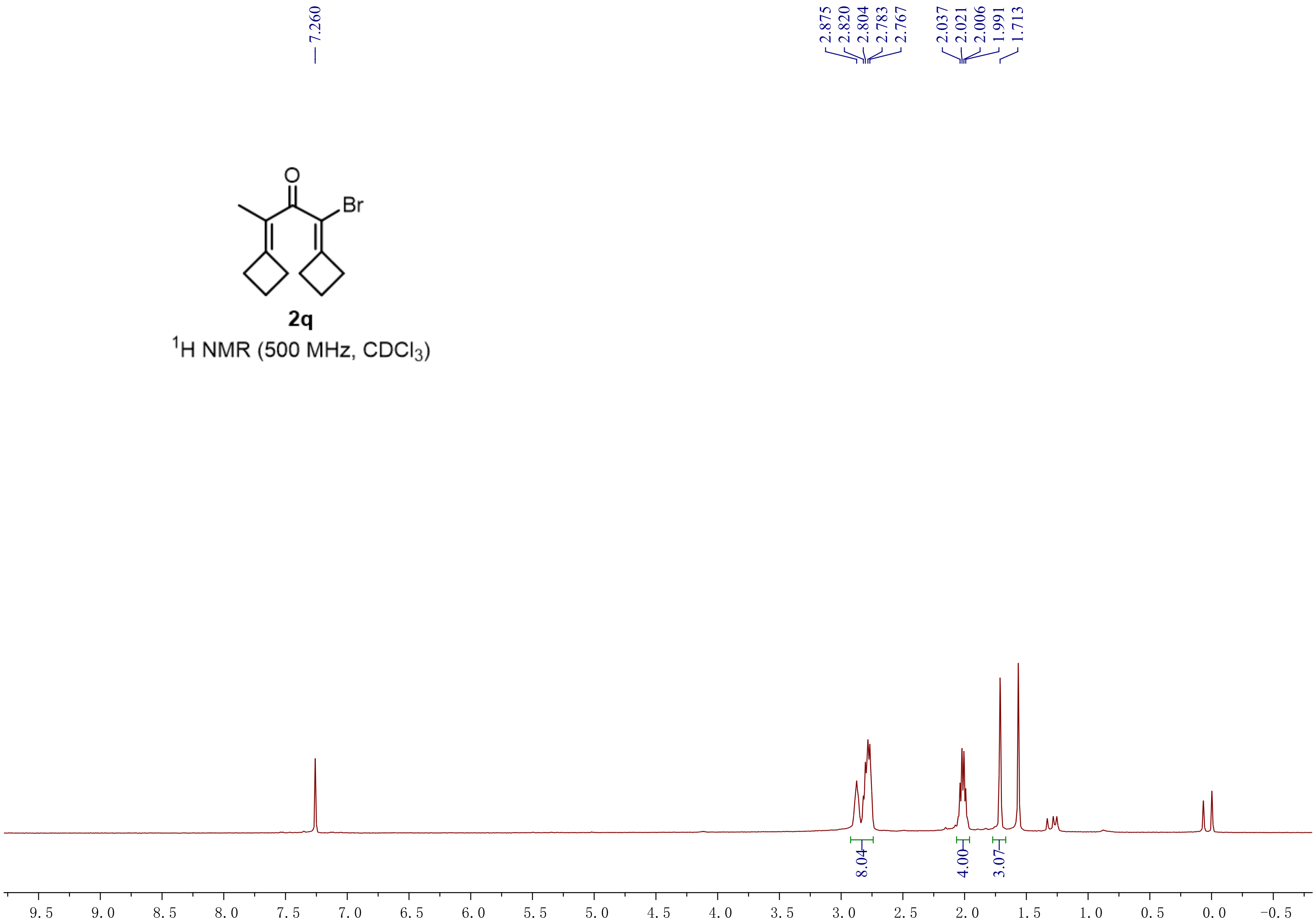

Supplementary Fig. 173. ^{1}H NMR spectra of compound **2q** in $CDCl_3$

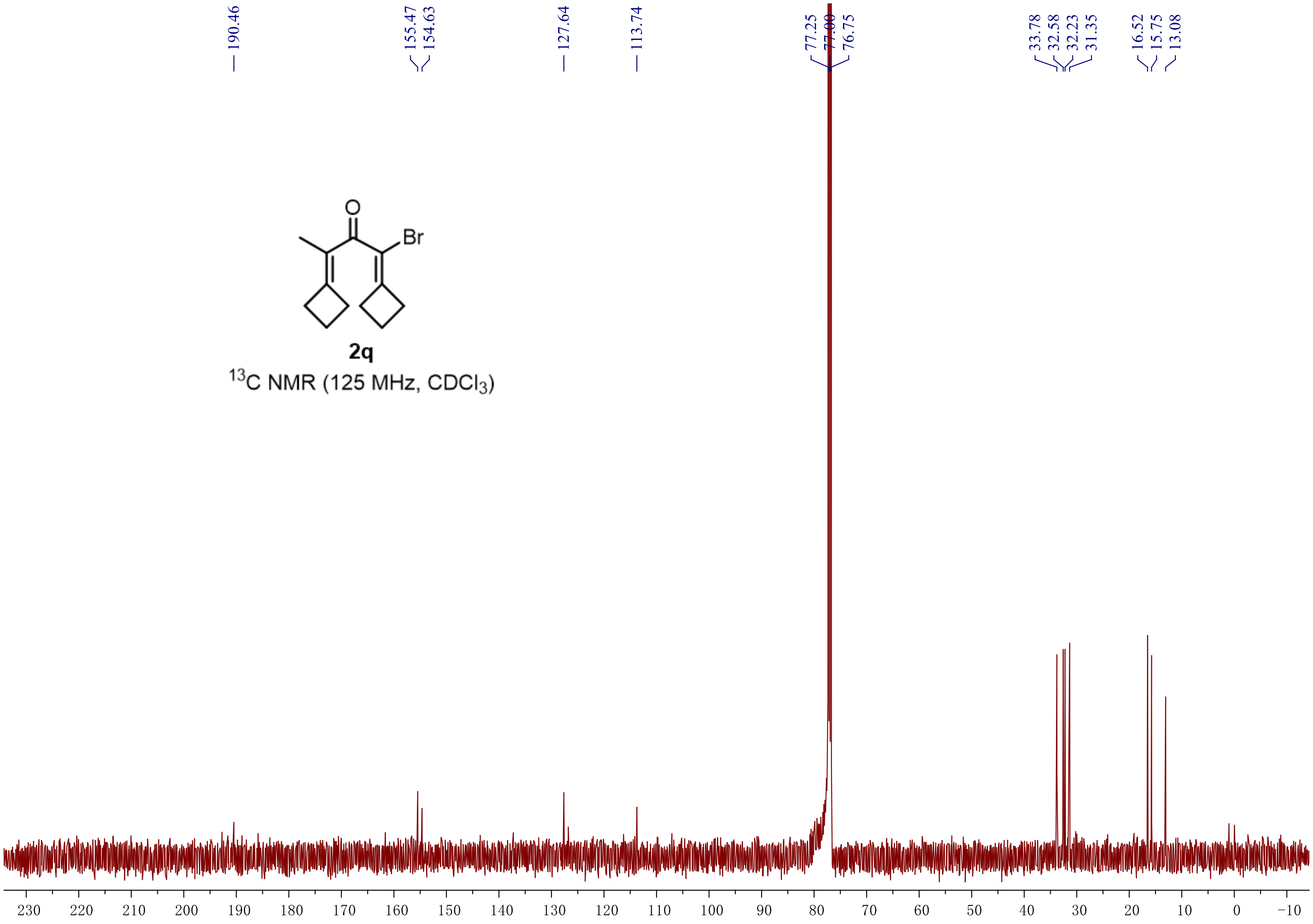

Supplementary Fig. 174. ^{13}C NMR spectra of compound **2q** in $CDCl_3$

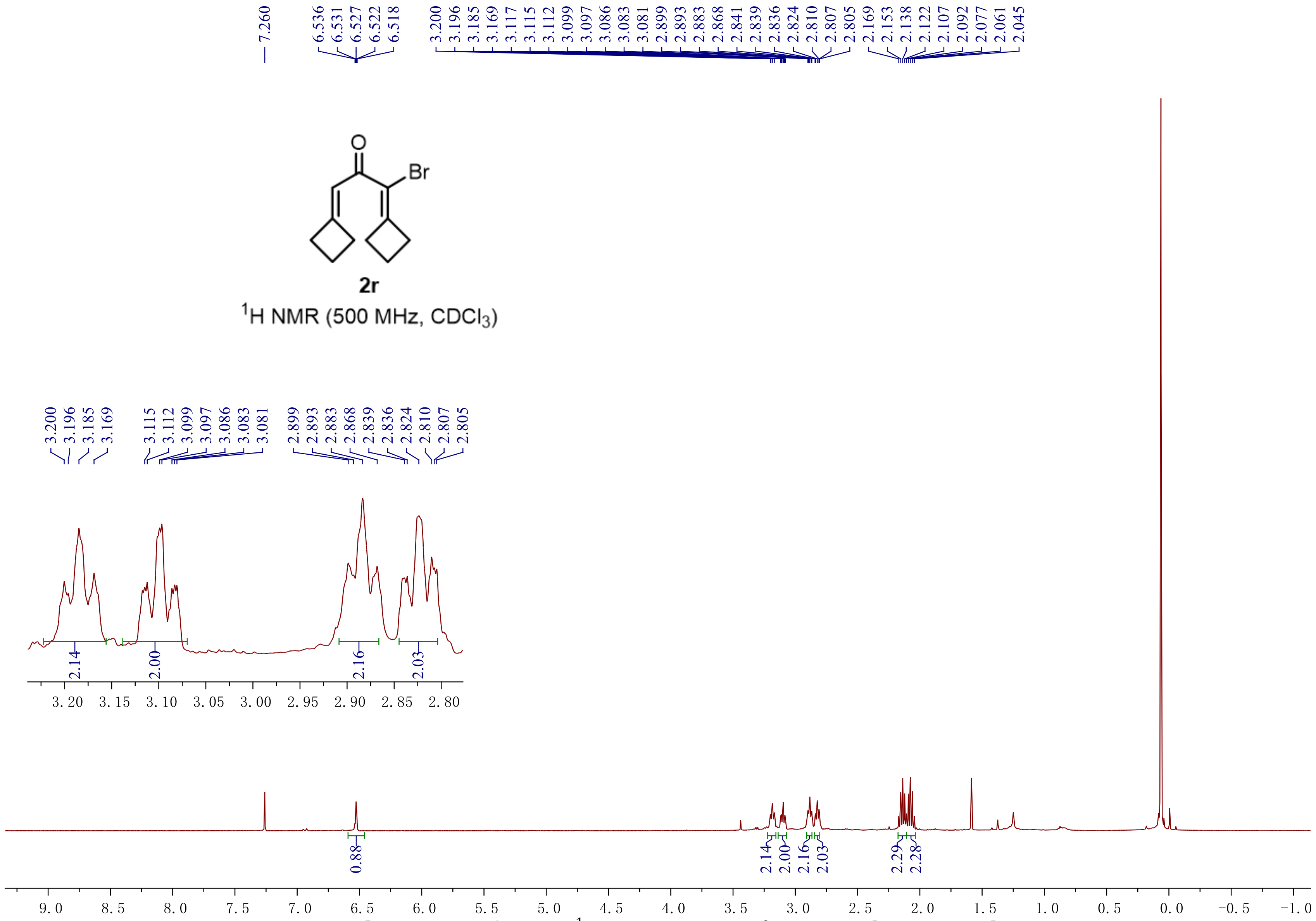

Supplementary Fig. 175. ^{1}H NMR spectra of compound **2r** in $CDCl_3$

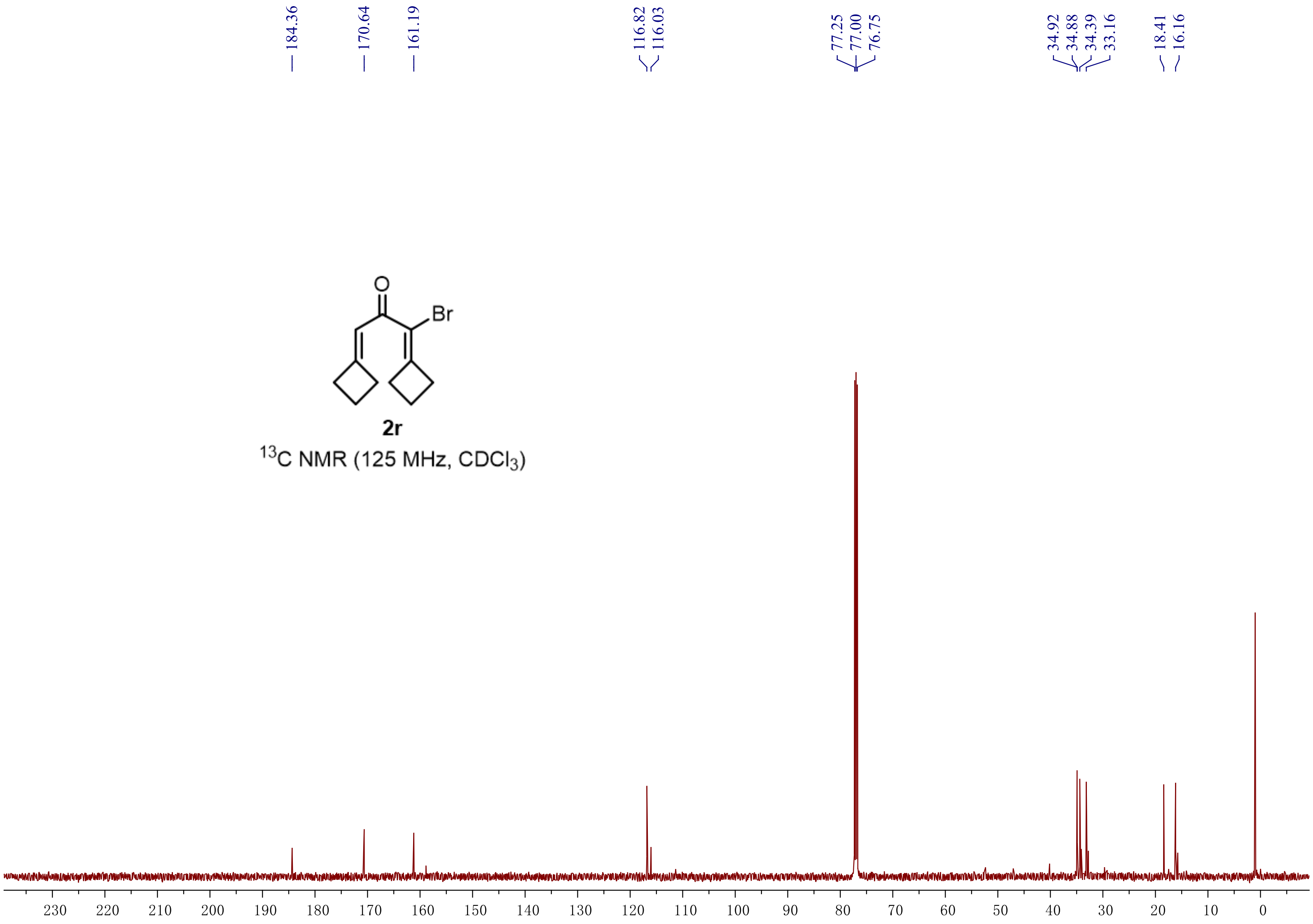

Supplementary Fig. 176. ^{13}C NMR spectra of compound **2r** in $CDCl_3$

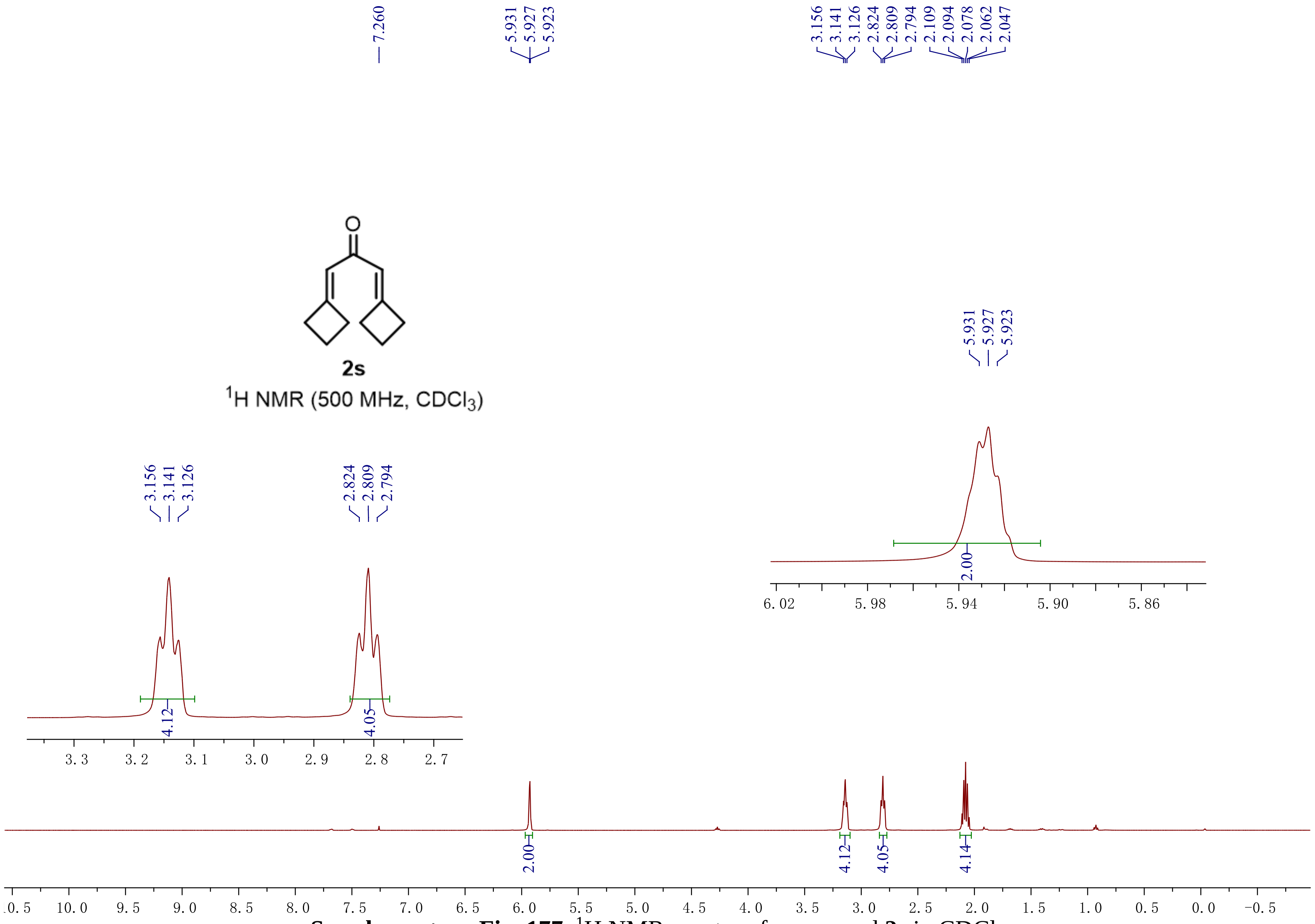

Supplementary Fig. 177. ^{1}H NMR spectra of compound **2s** in $CDCl_3$

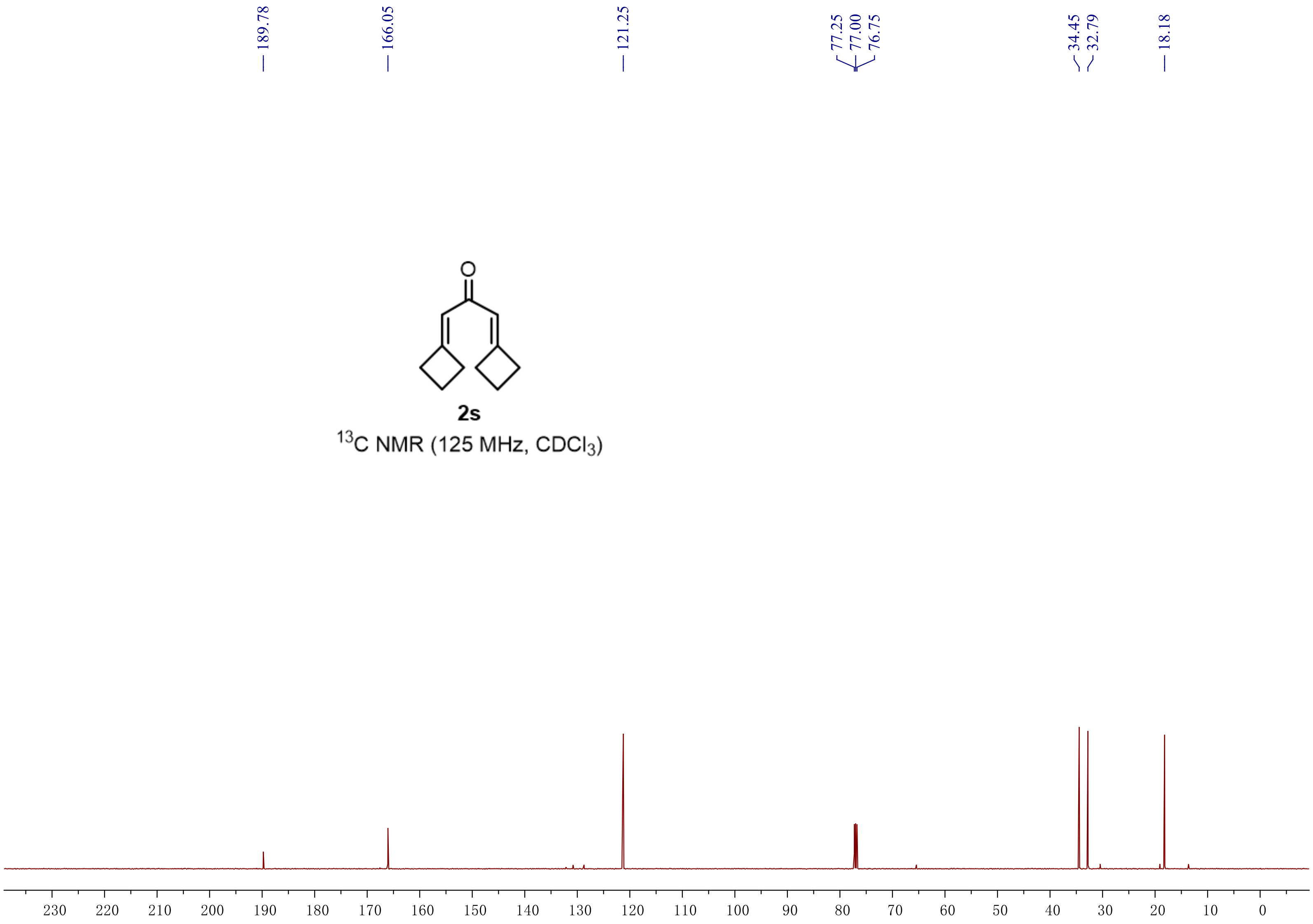

Supplementary Fig. 178. ^{13}C NMR spectra of compound **2s** in $CDCl_3$

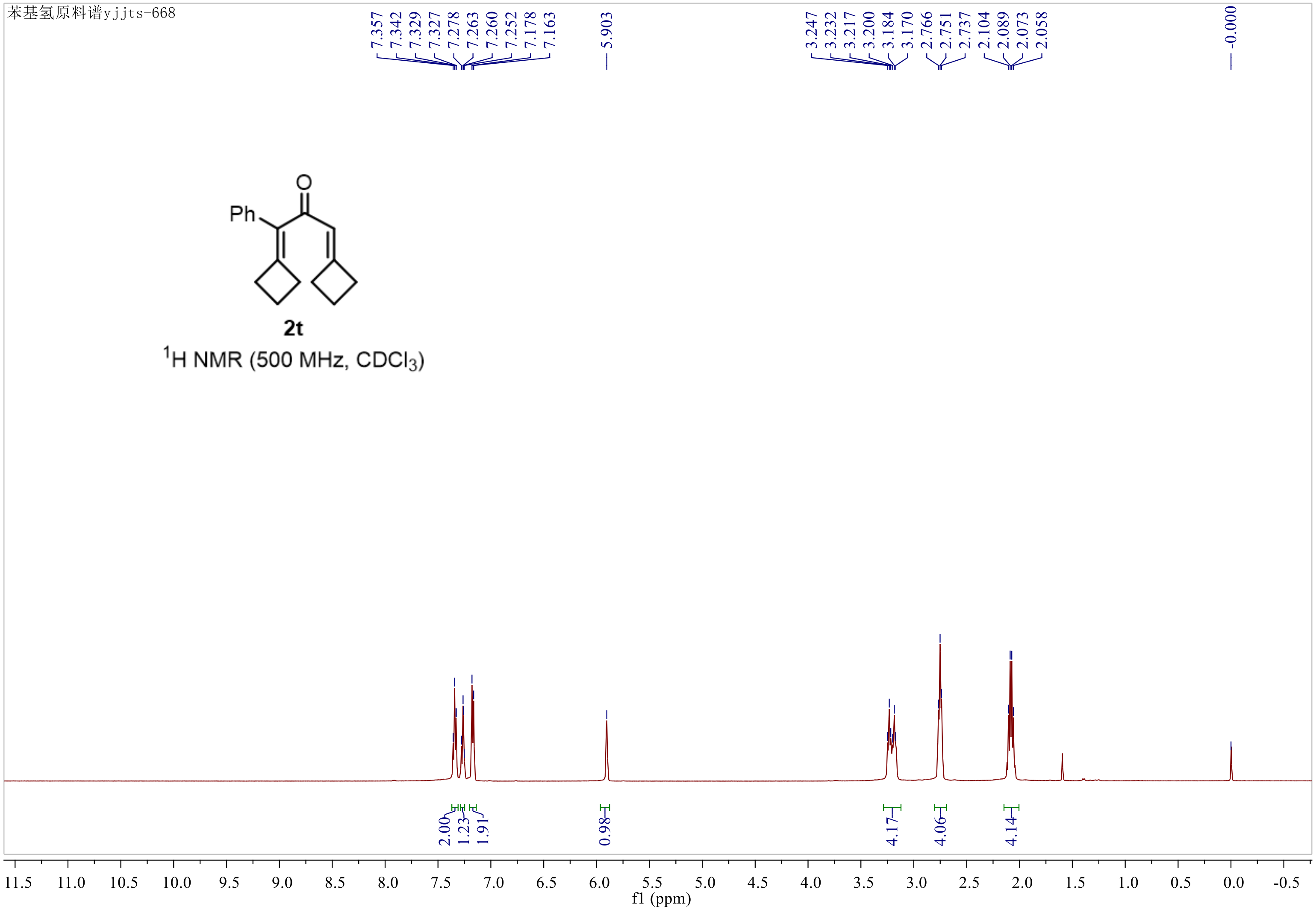

Supplementary Fig. 179. ^{1}H NMR spectra of compound **2t** in $CDCl_3$

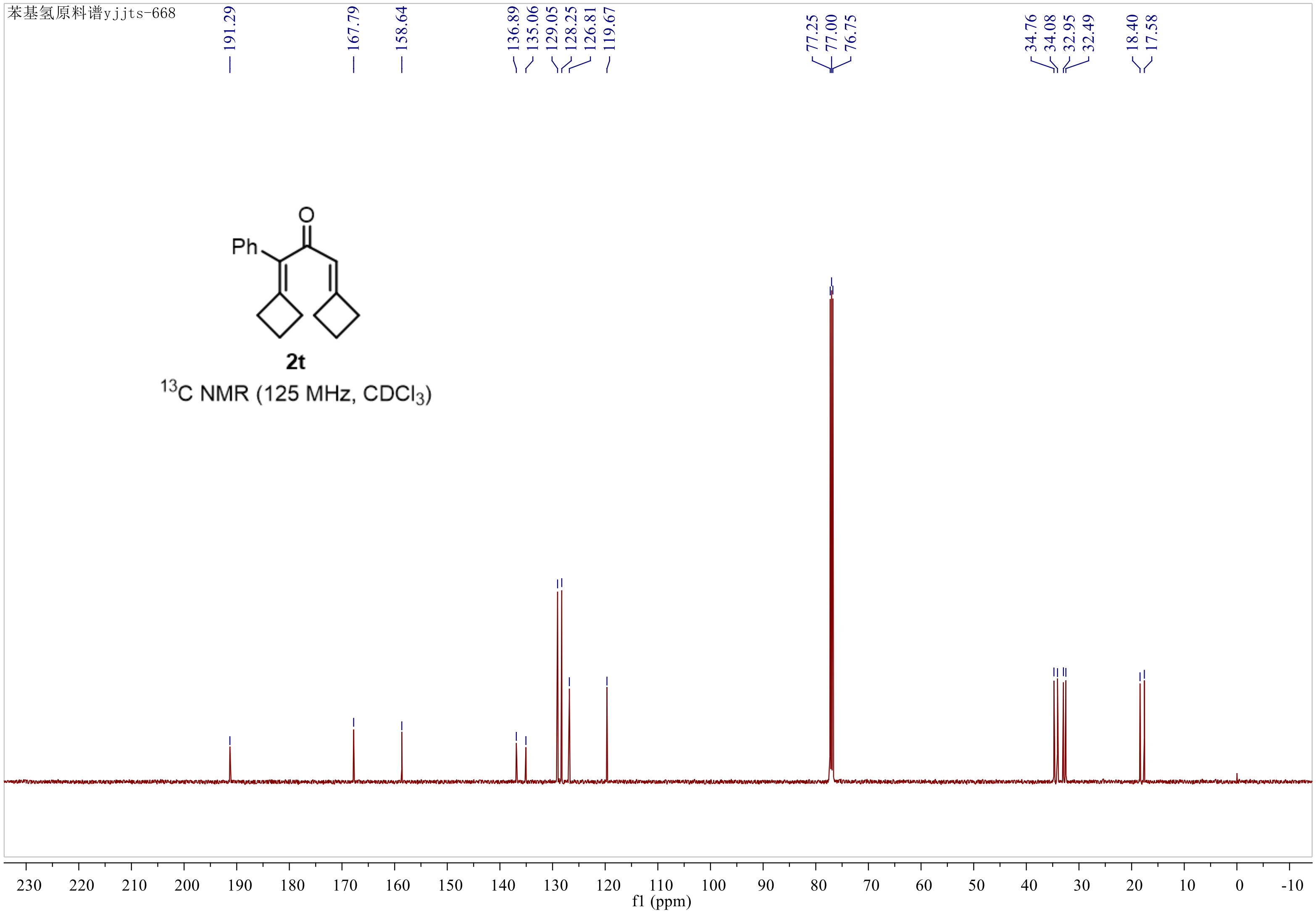

Supplementary Fig. 180. ^{13}C NMR spectra of compound **2t** in $CDCl_3$

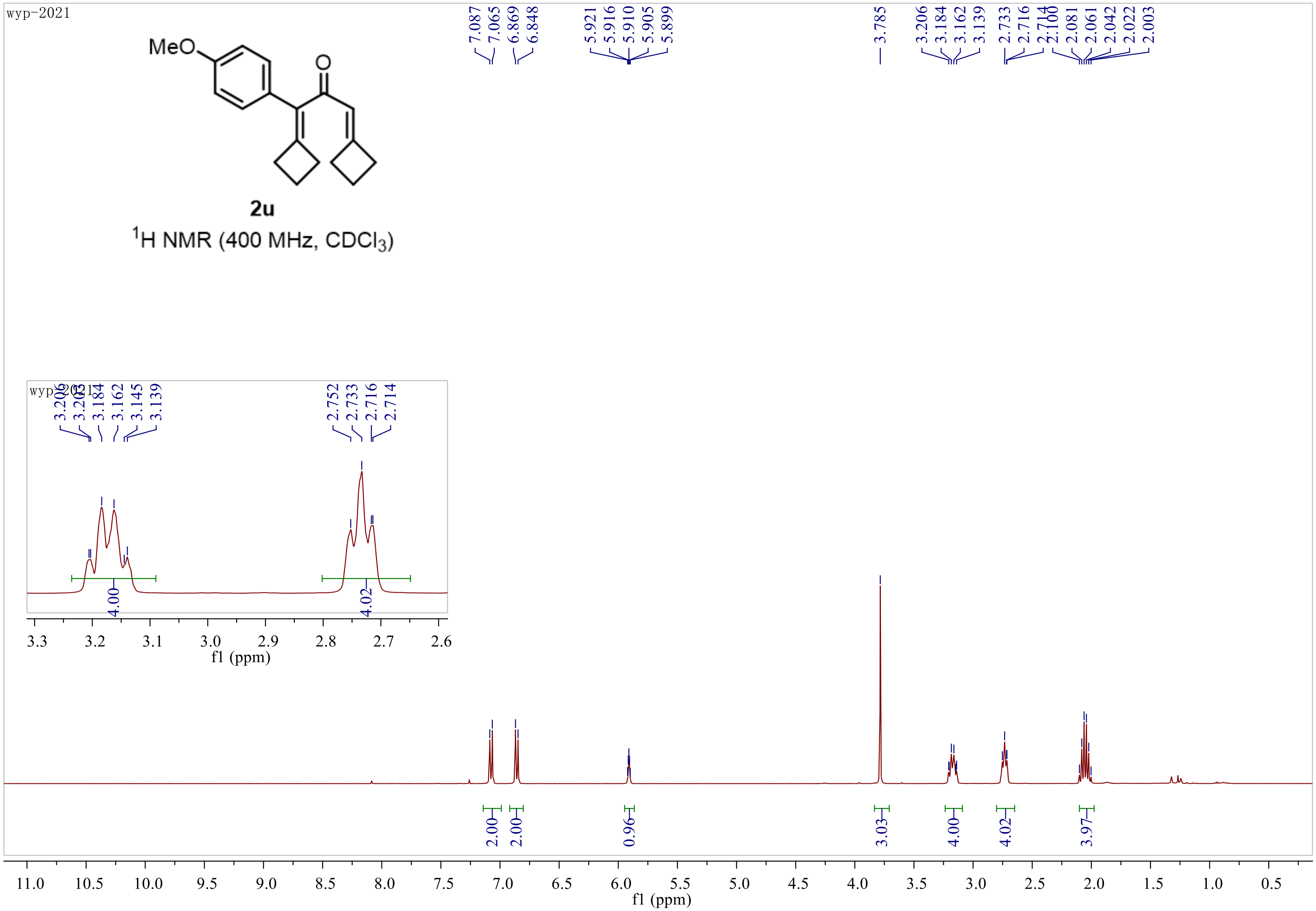

Supplementary Fig. 181. ^{1}H NMR spectra of compound **2u** in $CDCl_3$

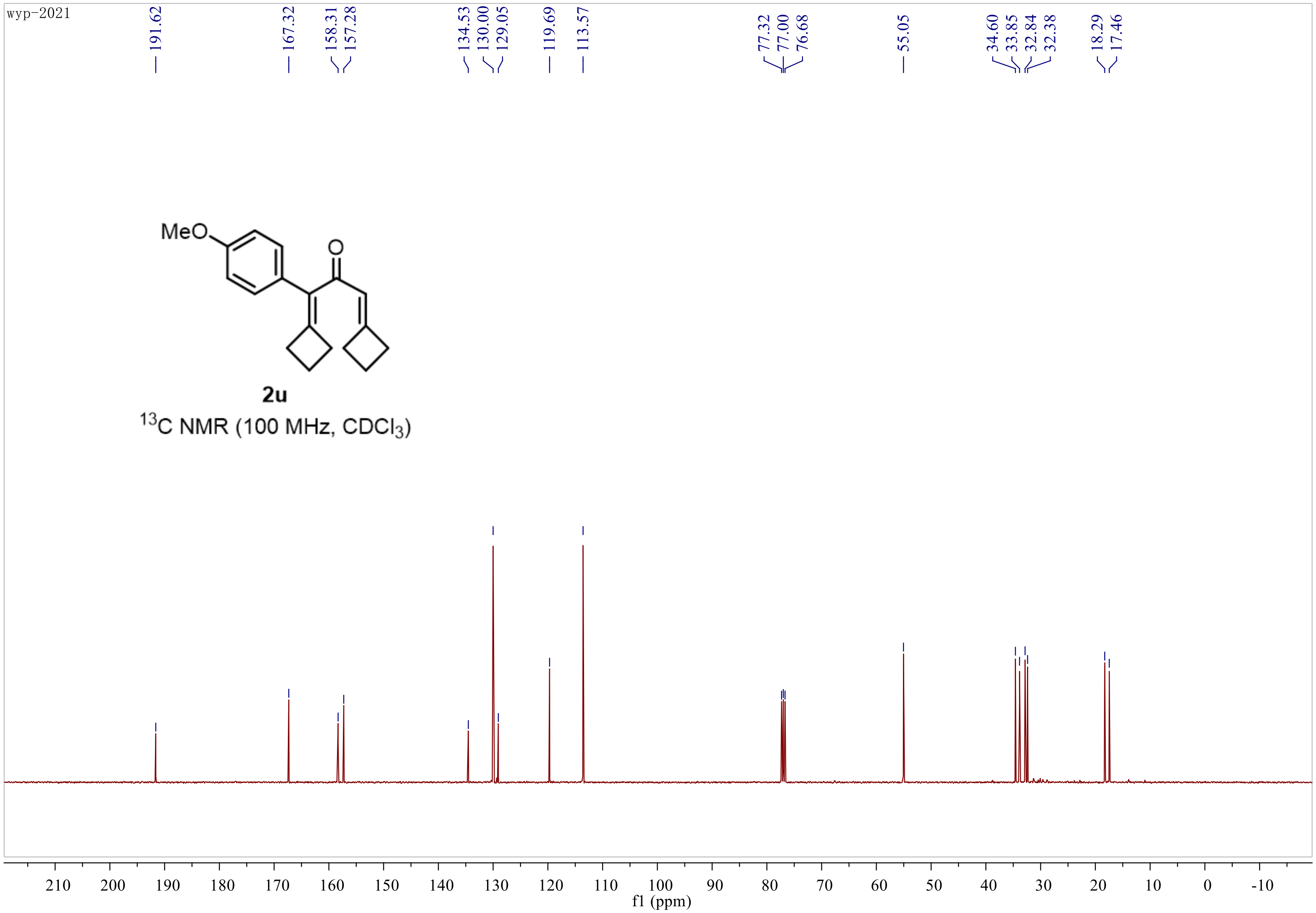

Supplementary Fig. 182. ^{13}C NMR spectra of compound **2u** in $CDCl_3$

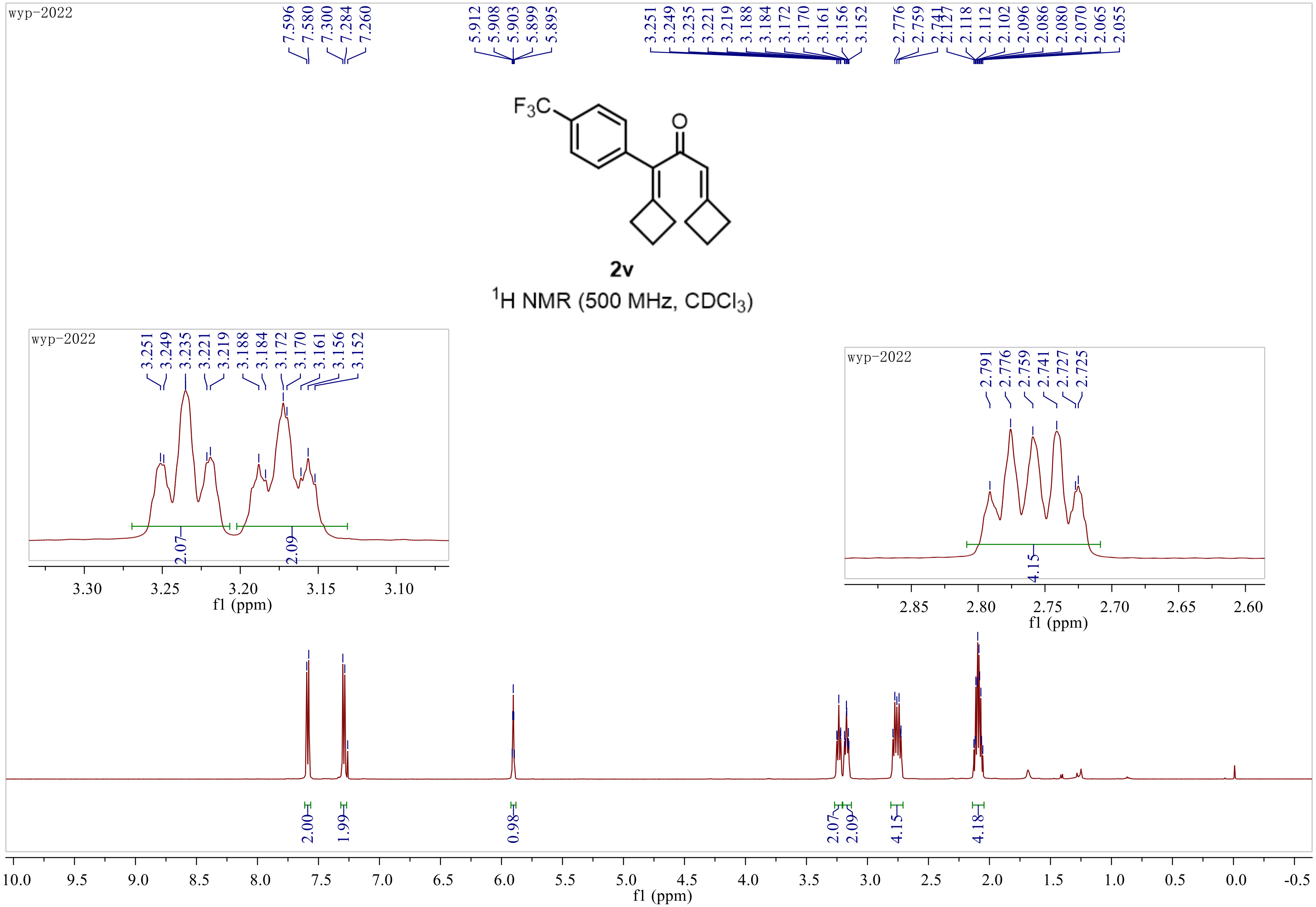

Supplementary Fig. 183. ^{1}H NMR spectra of compound **2v** in $CDCl_3$

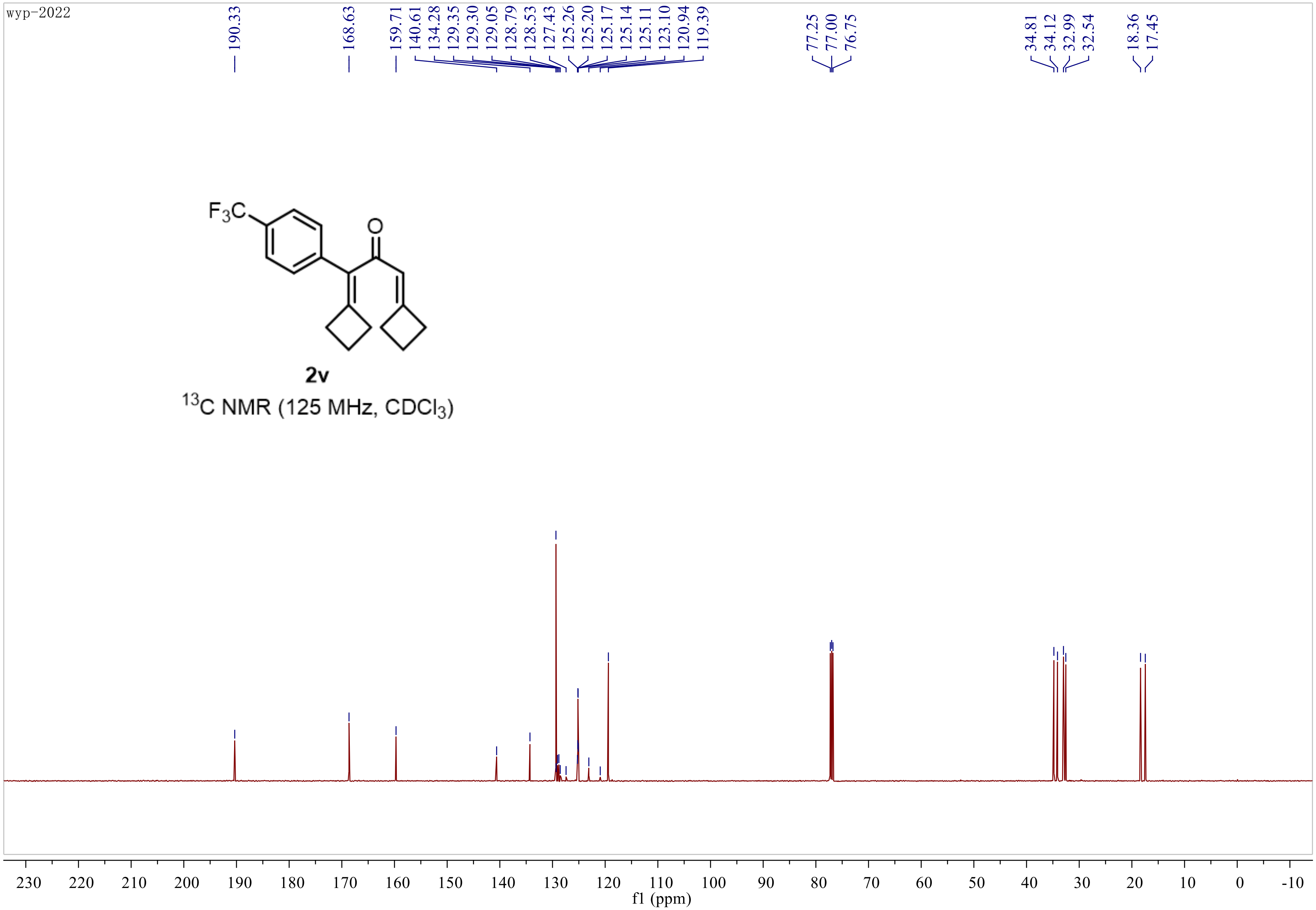

Supplementary Fig. 184. ^{13}C NMR spectra of compound **2v** in $CDCl_3$

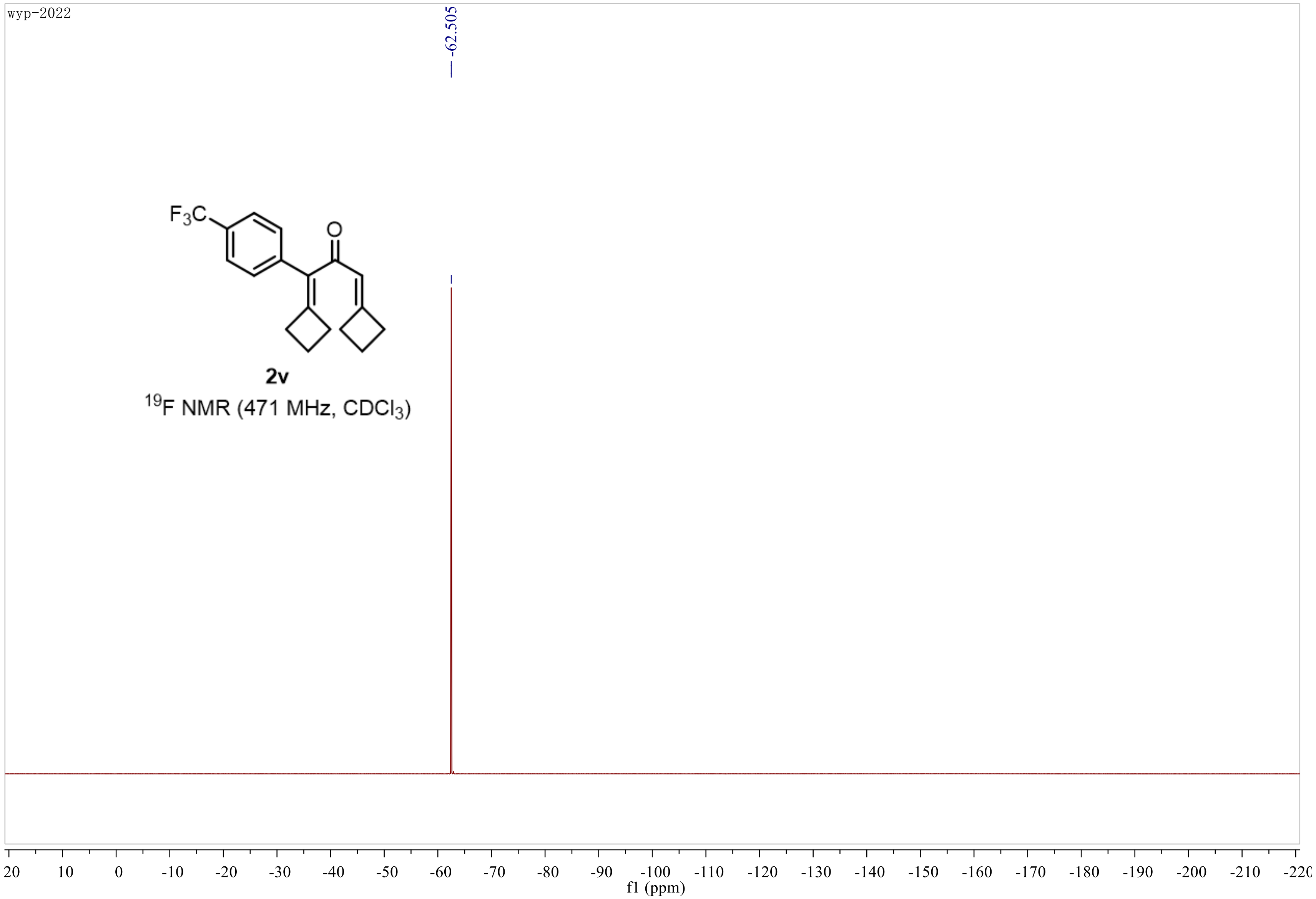

Supplementary Fig. 185. ^{19}F NMR spectra of compound **2v** in $CDCl_3$

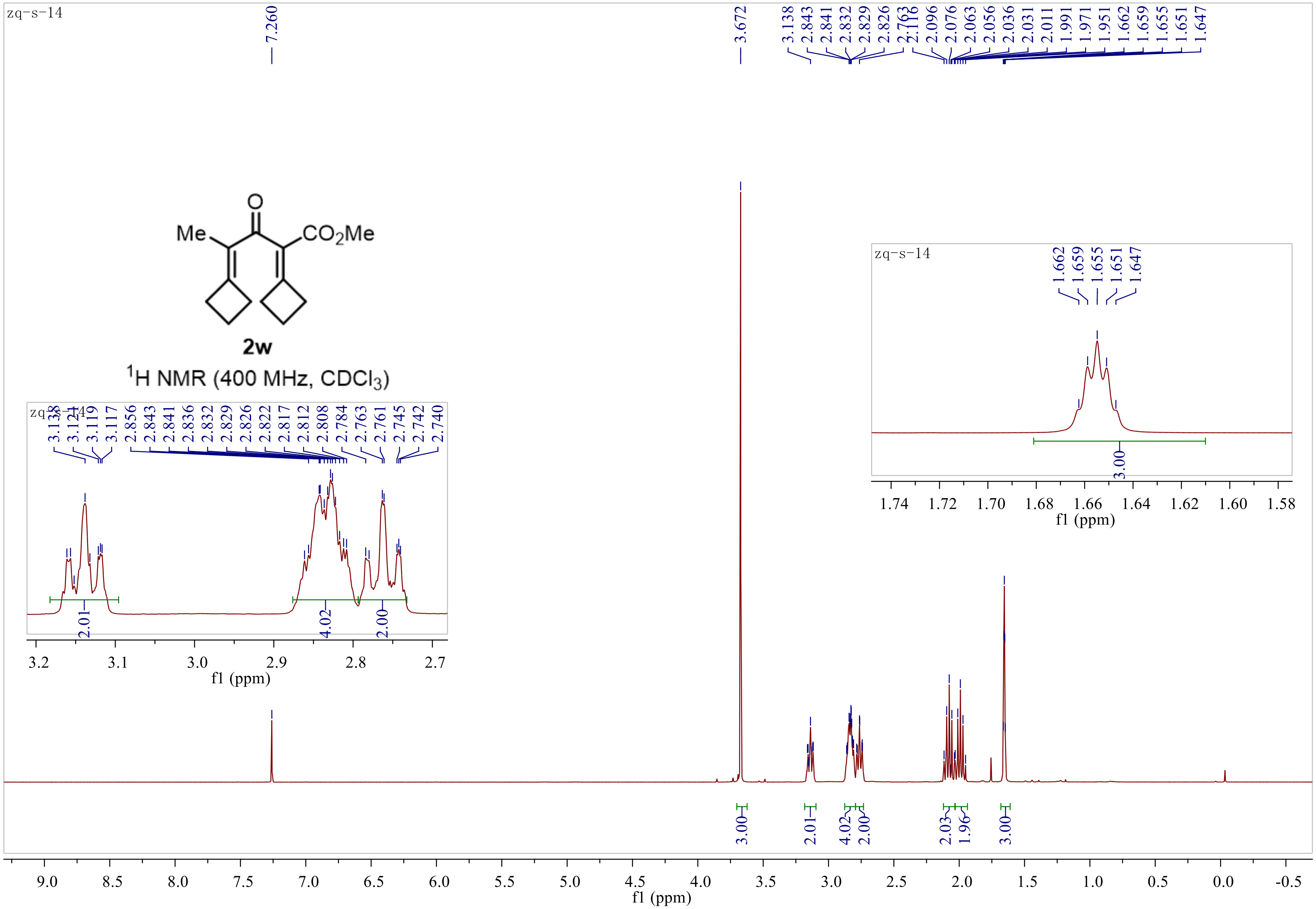

Supplementary Fig. 186. ^{1}H NMR spectra of compound **2w** in $CDCl_3$

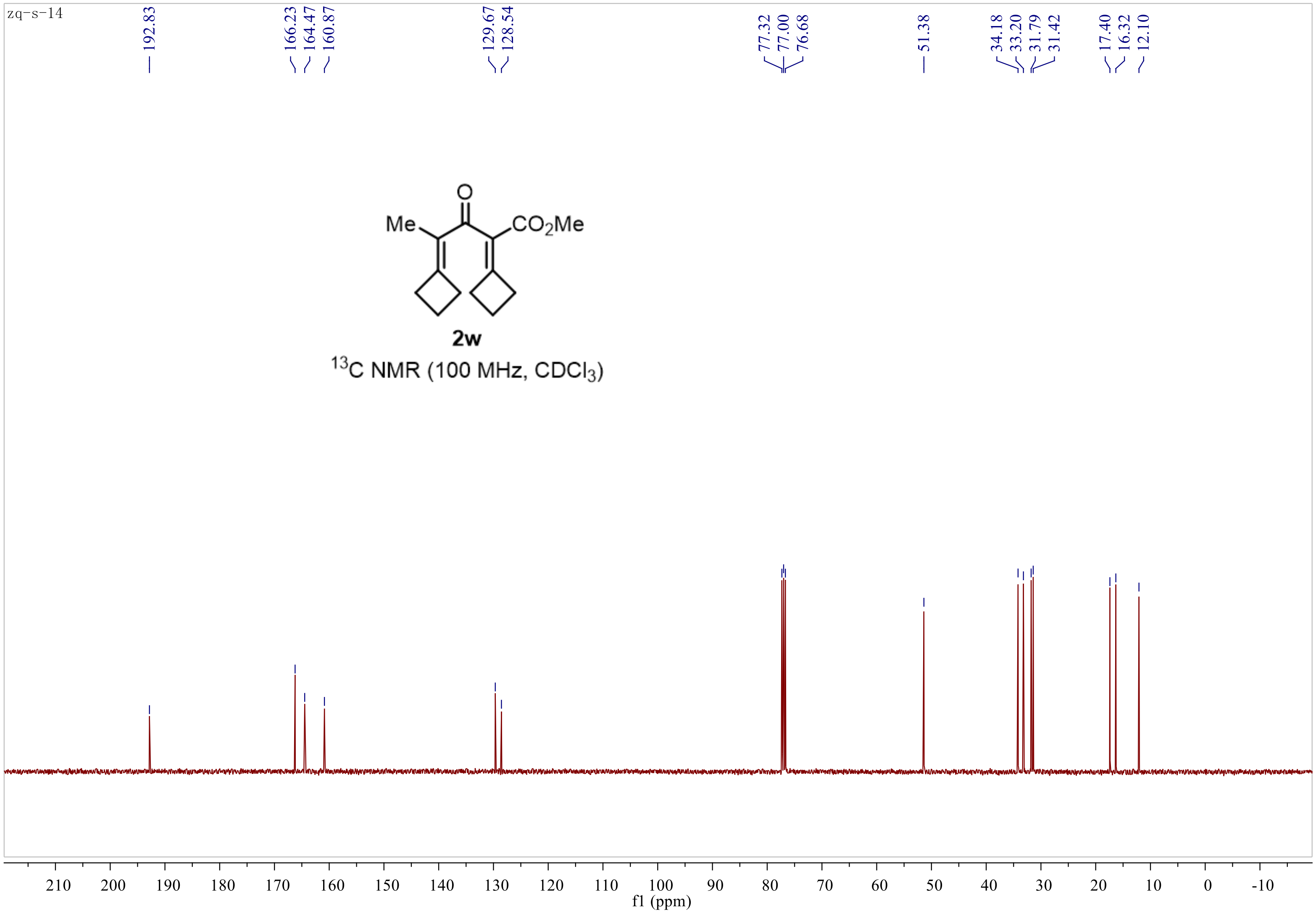

Supplementary Fig. 187. ^{13}C NMR spectra of compound **2w** in $CDCl_3$

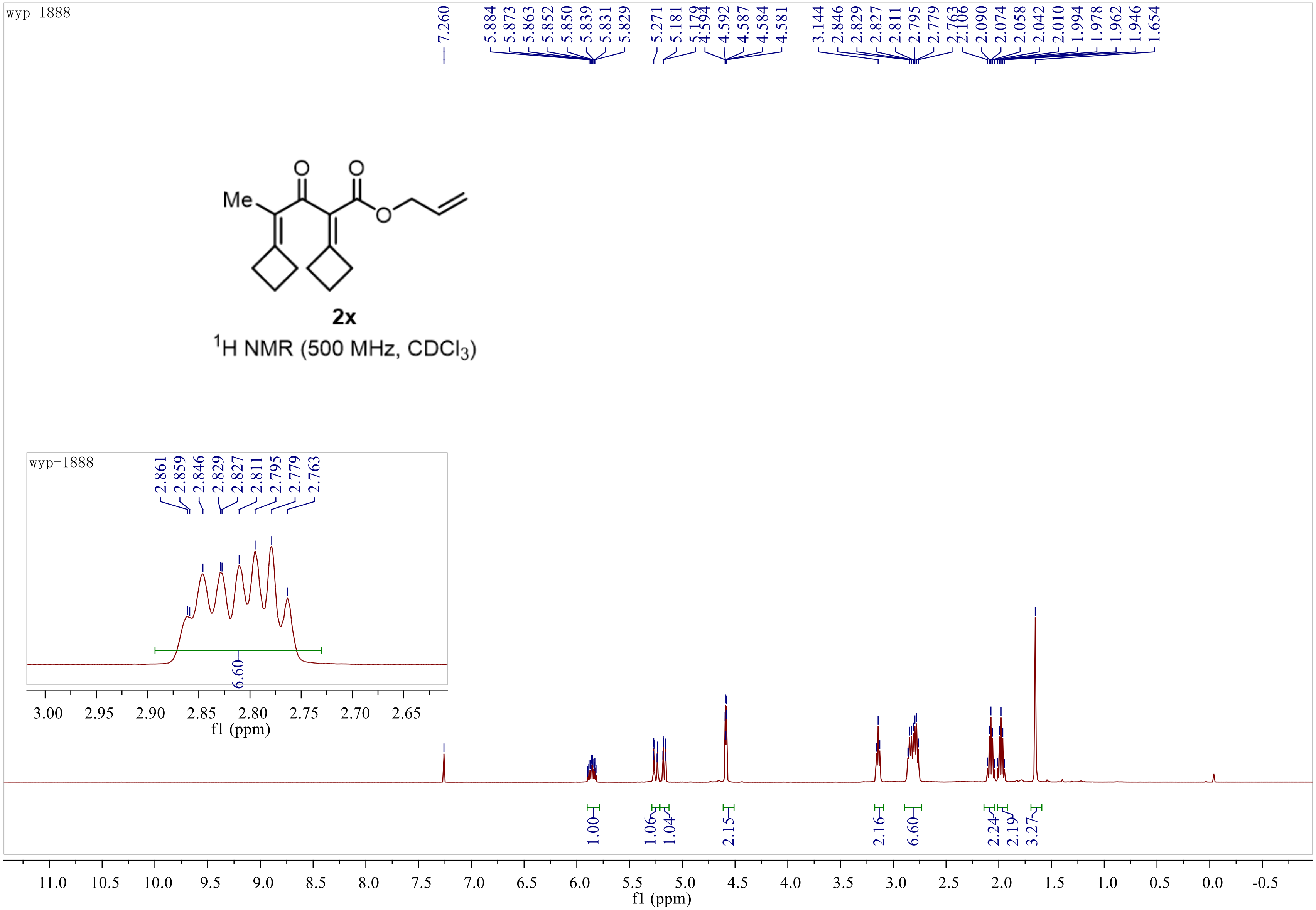

Supplementary Fig. 188. ^{1}H NMR spectra of compound **2x** in $CDCl_3$

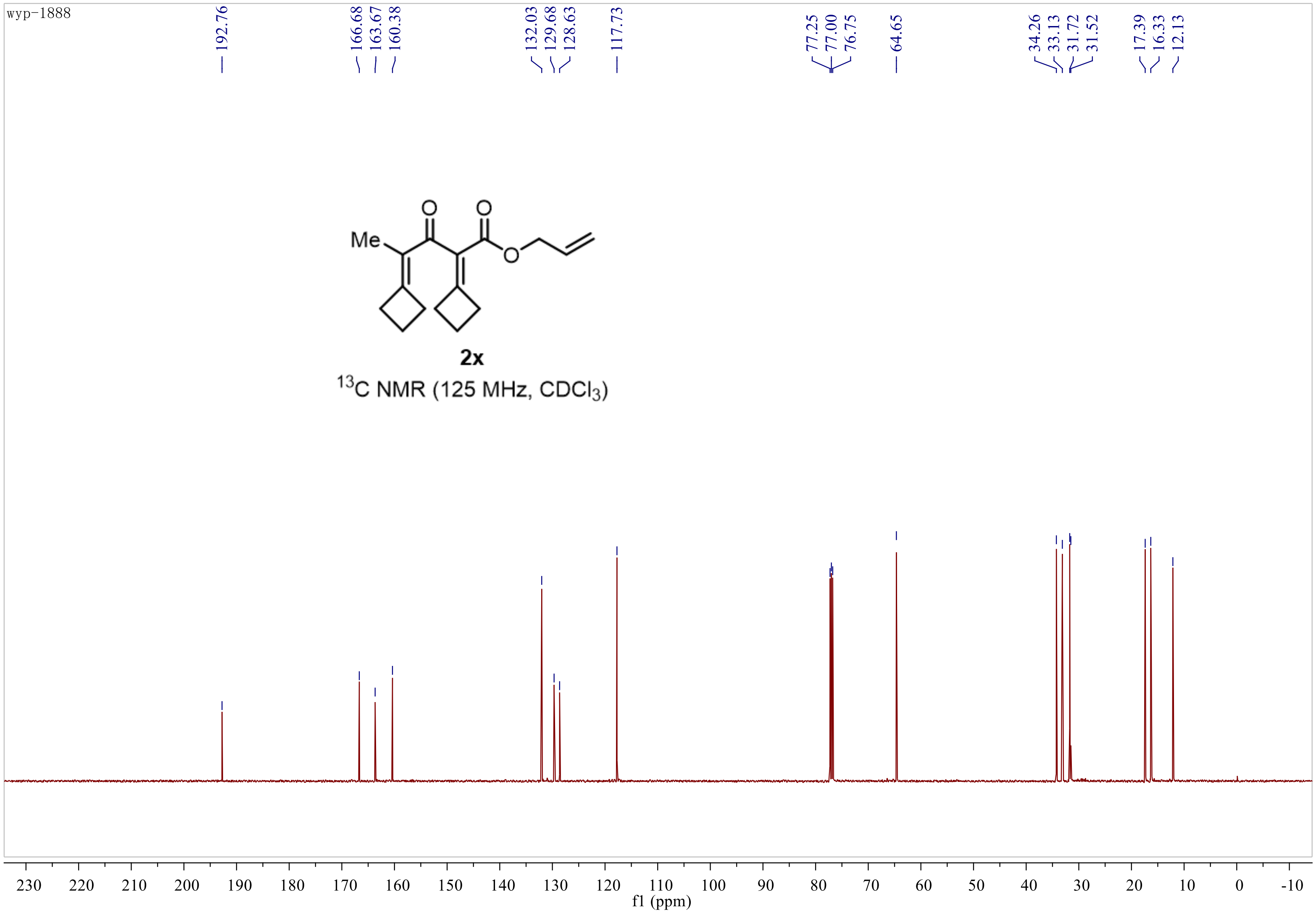

Supplementary Fig. 189. ^{13}C NMR spectra of compound **2x** in $CDCl_3$

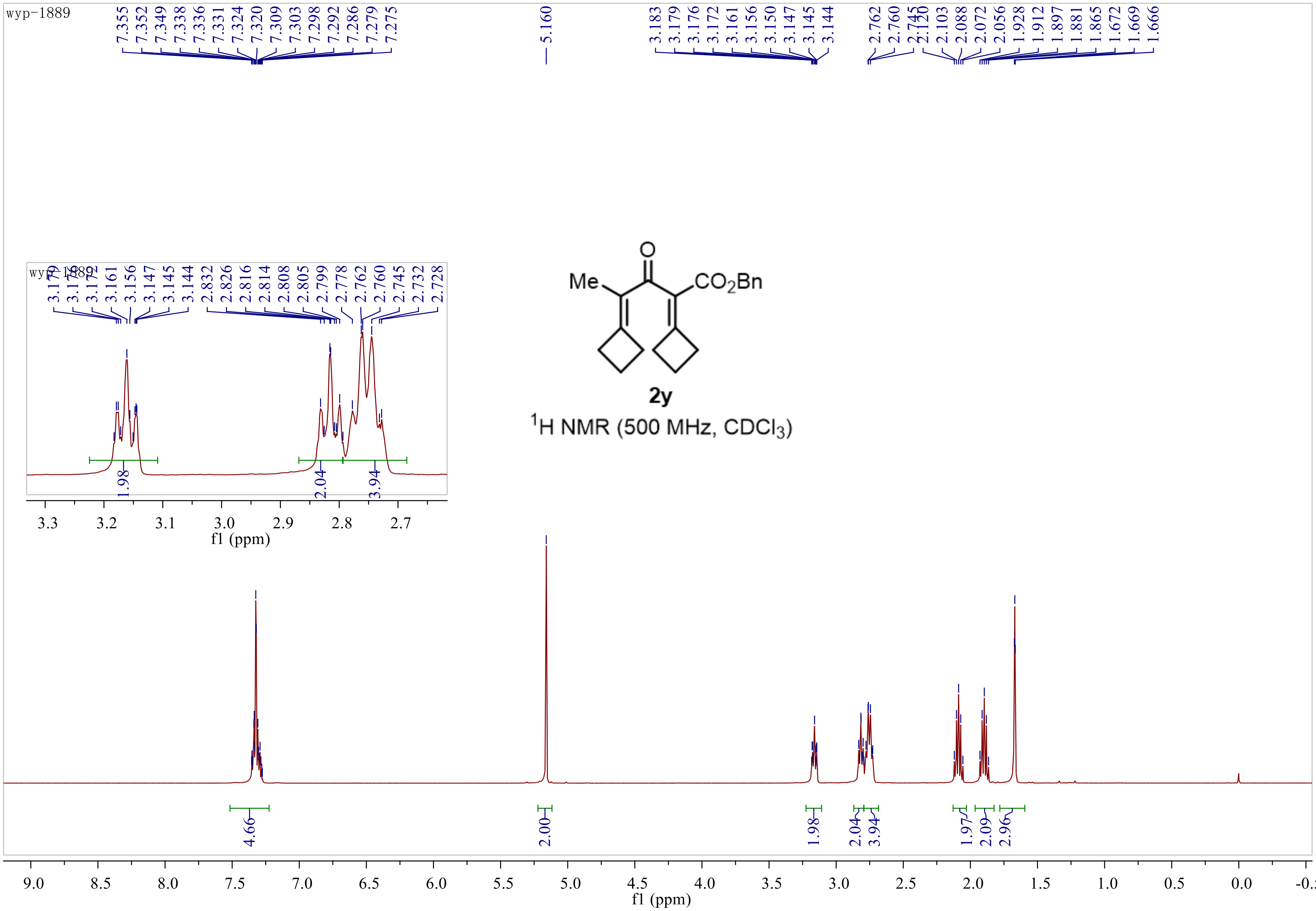

Supplementary Fig. 190. ^{1}H NMR spectra of compound **2y** in $CDCl_3$

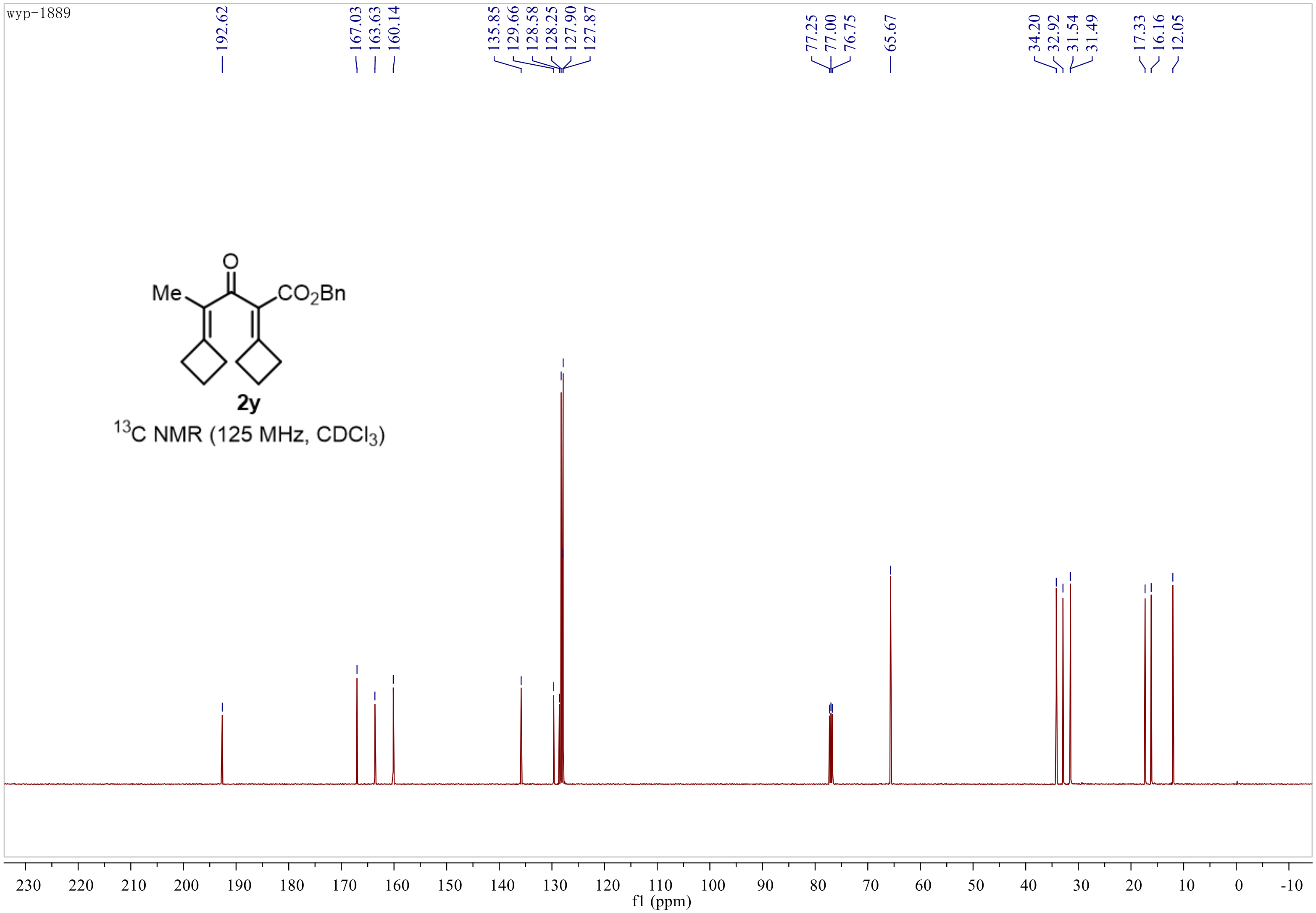

Supplementary Fig. 191. ^{13}C NMR spectra of compound **2y** in $CDCl_3$

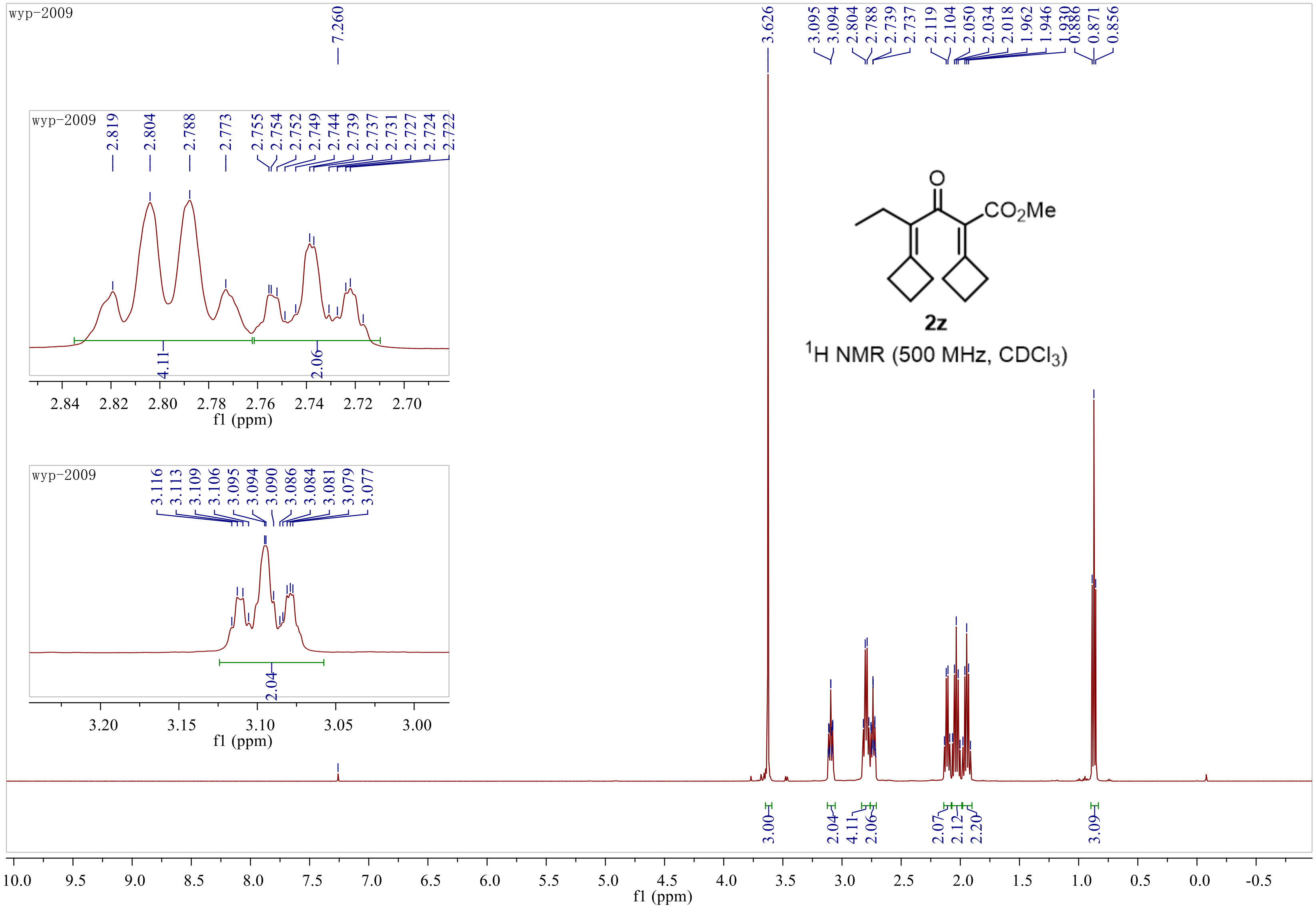

Supplementary Fig. 192. ^{1}H NMR spectra of compound **2z** in $CDCl_3$

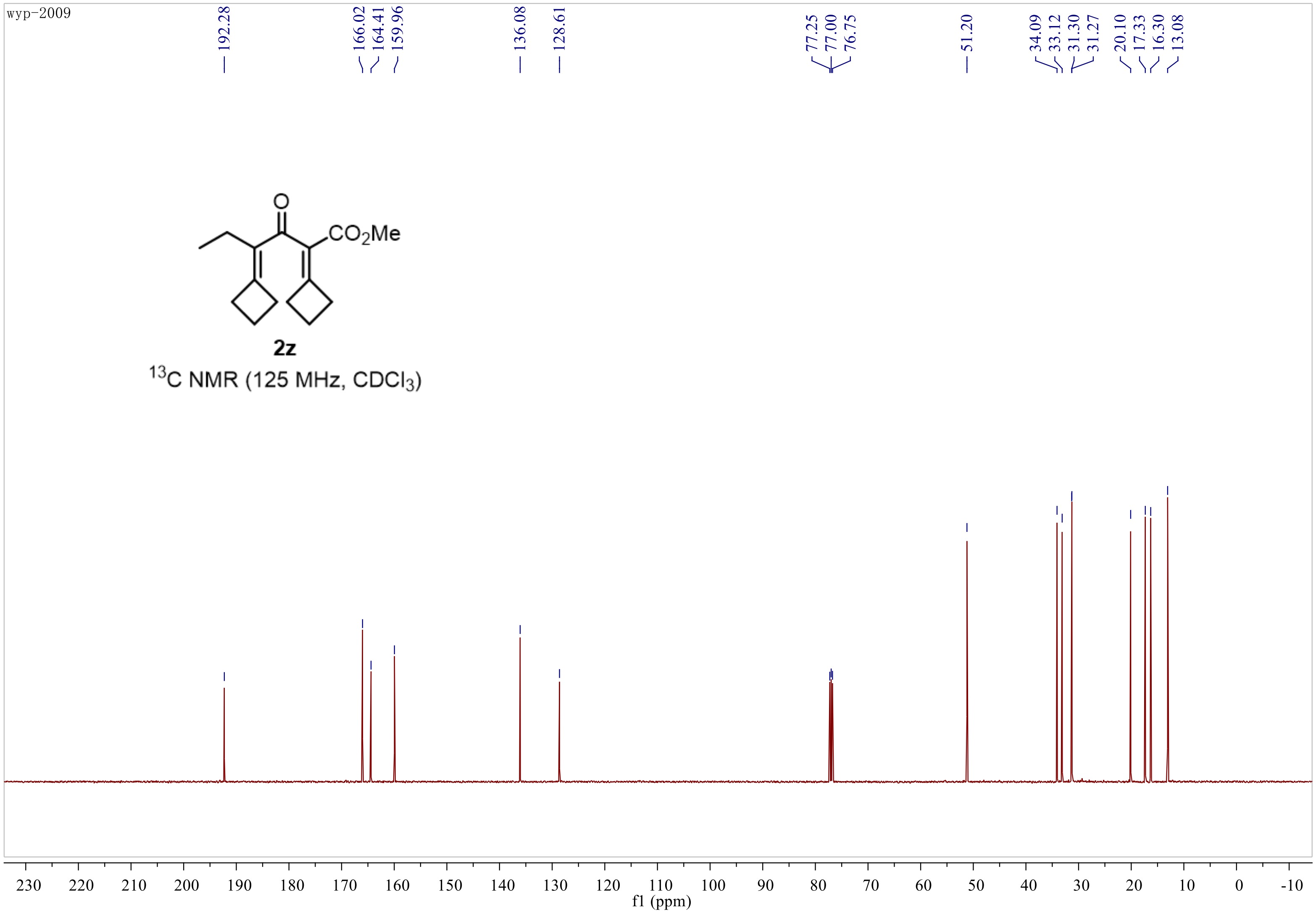

Supplementary Fig. 193. ^{13}C NMR spectra of compound **2y** in $CDCl_3$

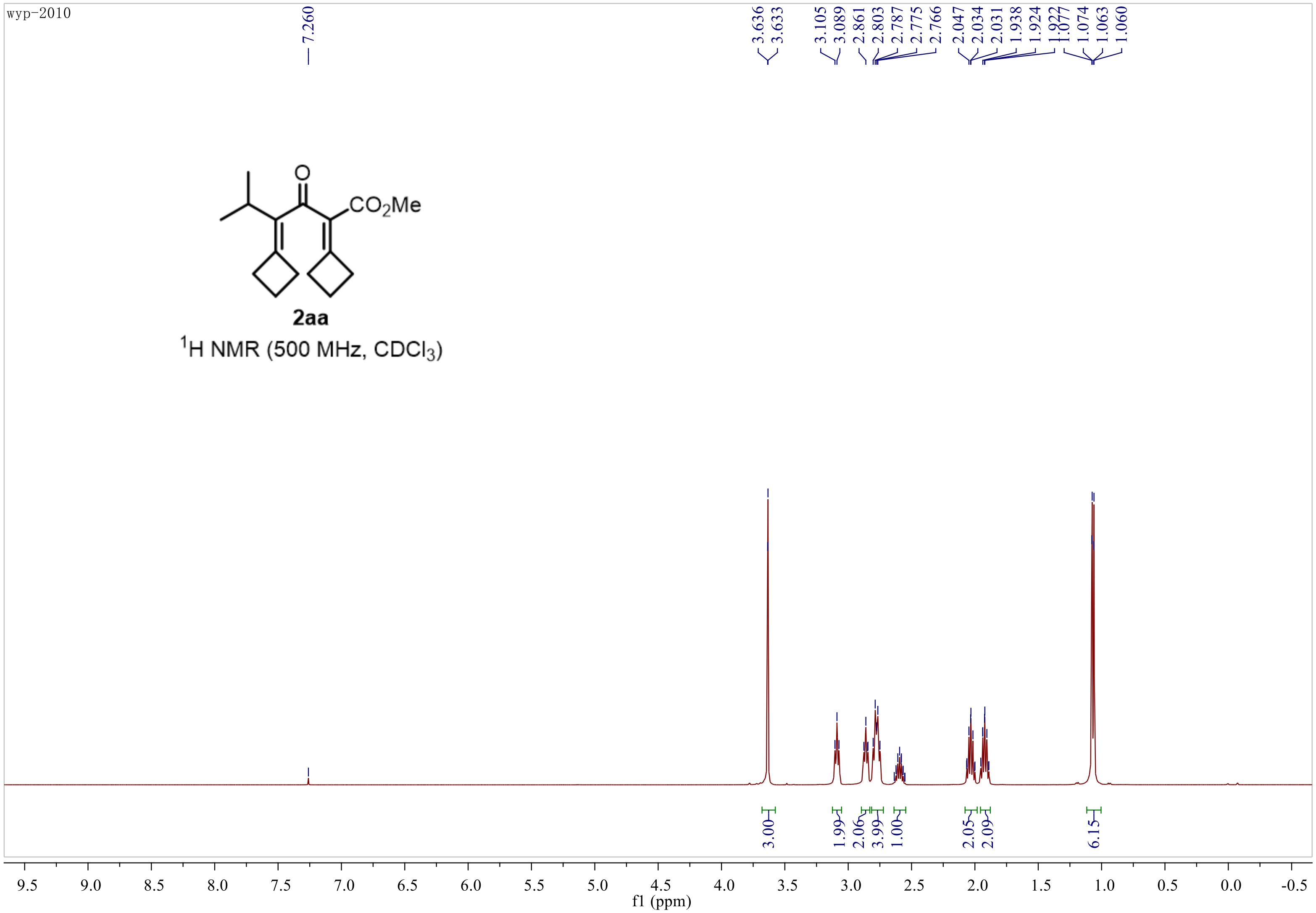

Supplementary Fig. 194. ^{1}H NMR spectra of compound **2aa** in $CDCl_3$

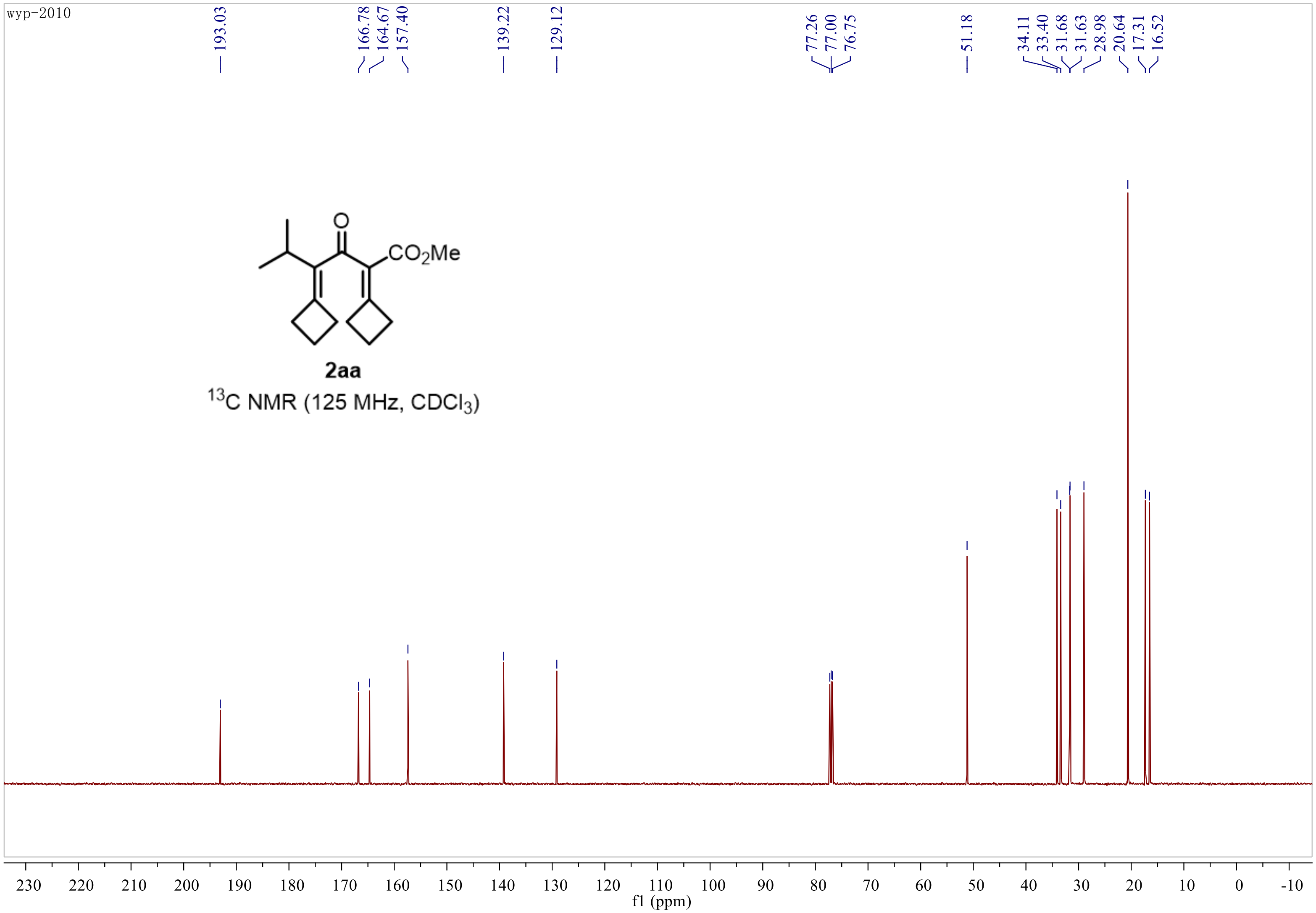

Supplementary Fig. 195. ^{13}C NMR spectra of compound **2aa** in $CDCl_3$

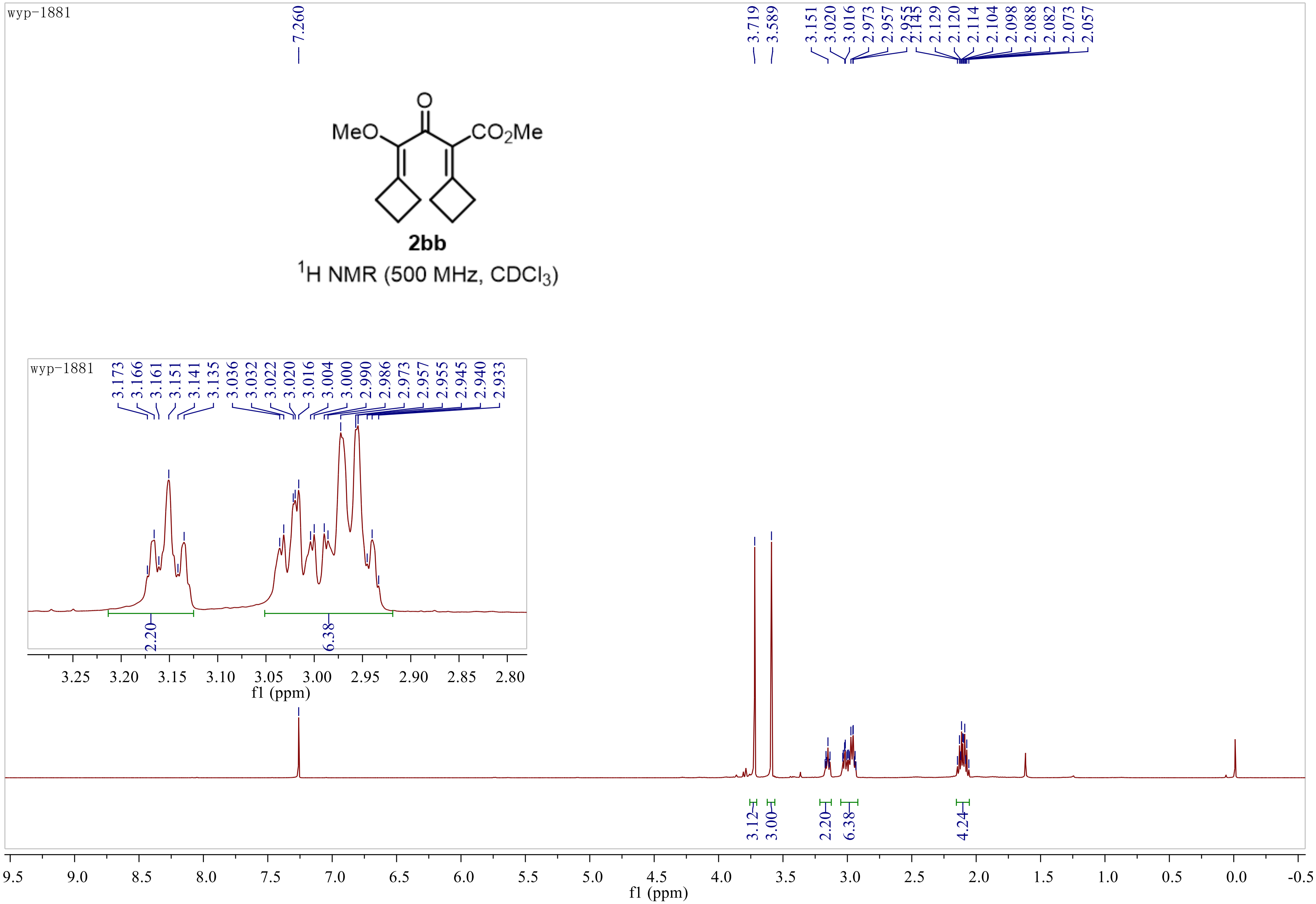

Supplementary Fig. 196. ^{1}H NMR spectra of compound **2bb** in $CDCl_3$

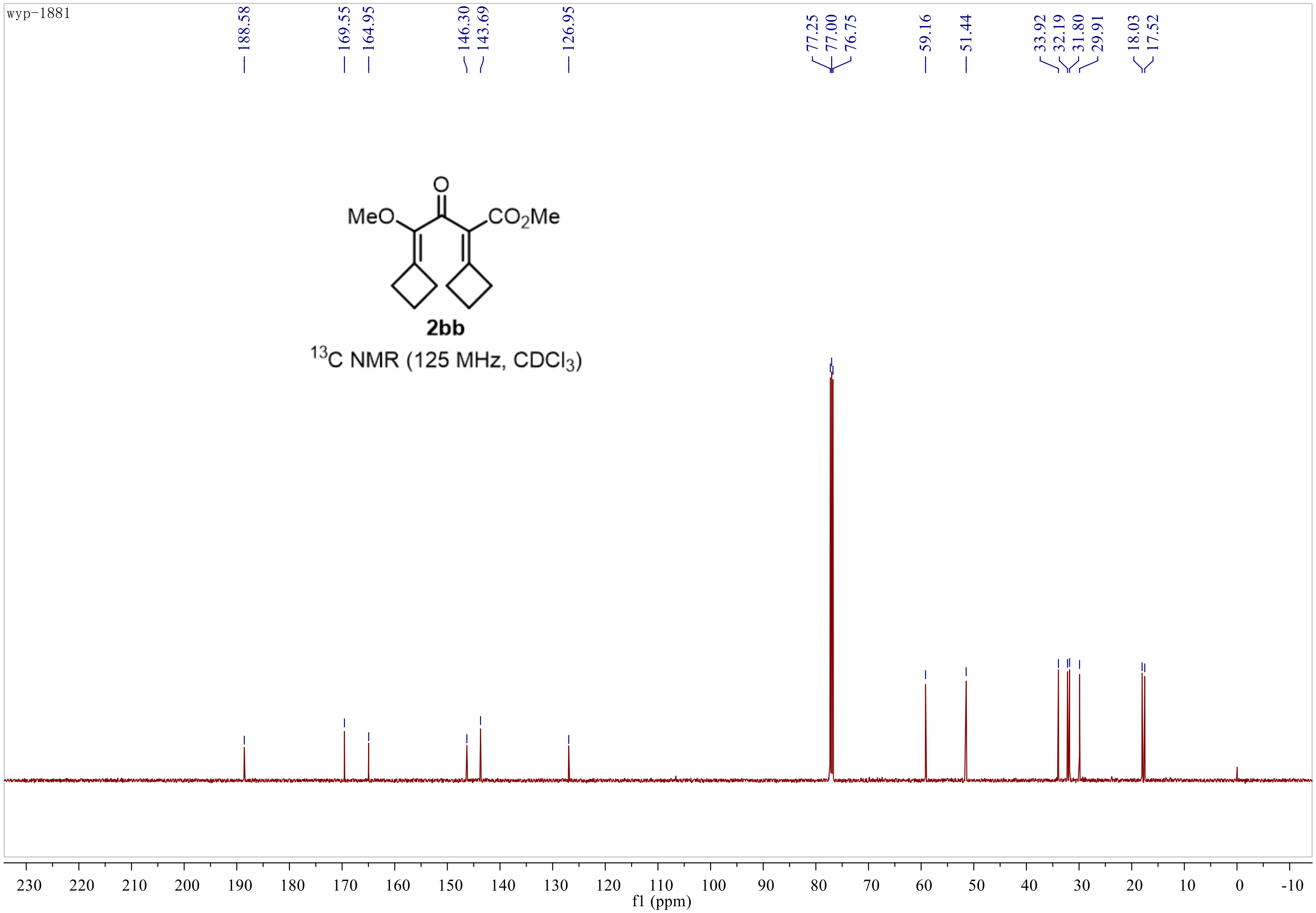

Supplementary Fig. 197. ^{13}C NMR spectra of compound **2bb** in $CDCl_3$

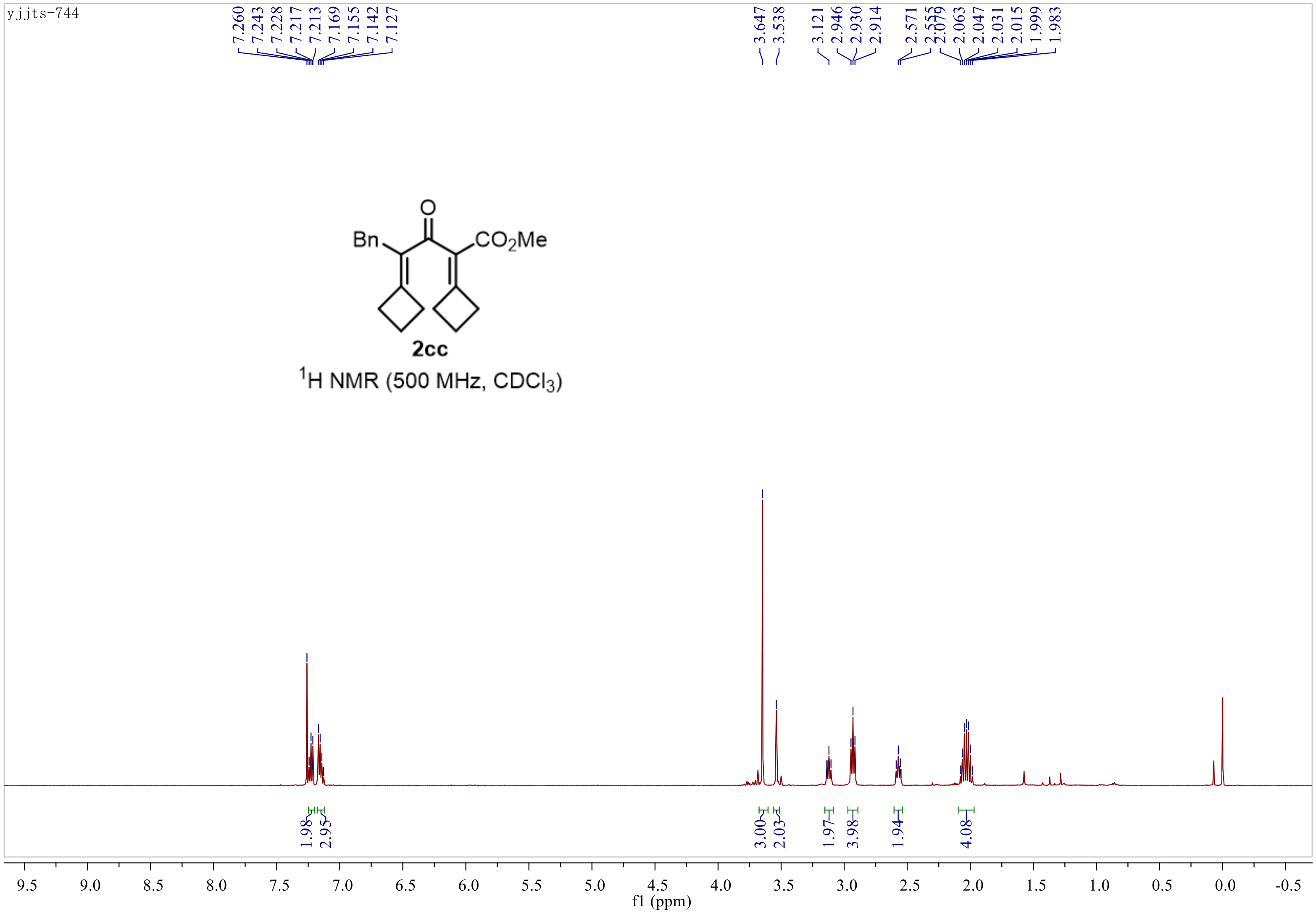

Supplementary Fig. 198. ^{1}H NMR spectra of compound **2cc** in $CDCl_3$

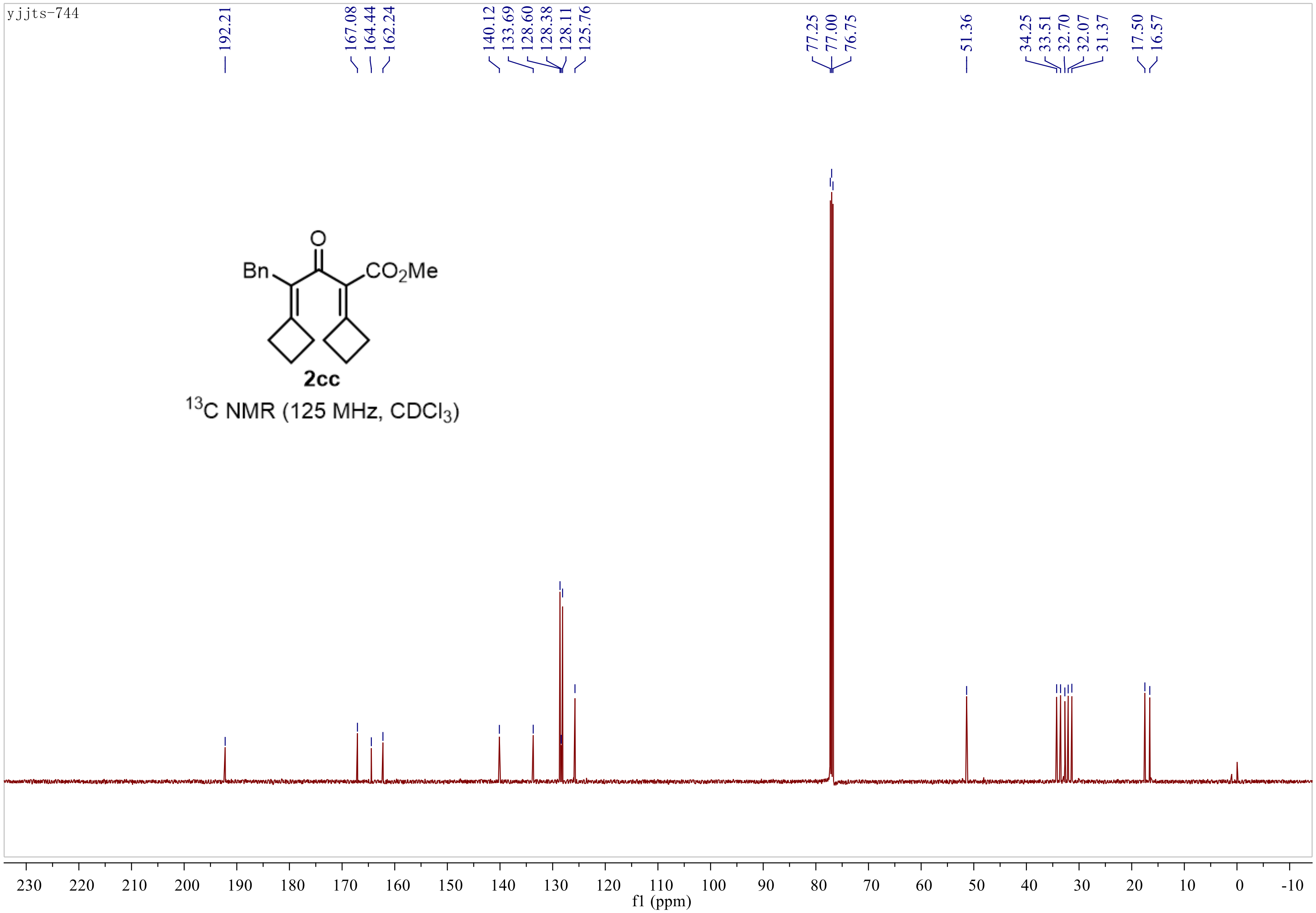

Supplementary Fig. 199. ^{13}C NMR spectra of compound **2cc** in $CDCl_3$

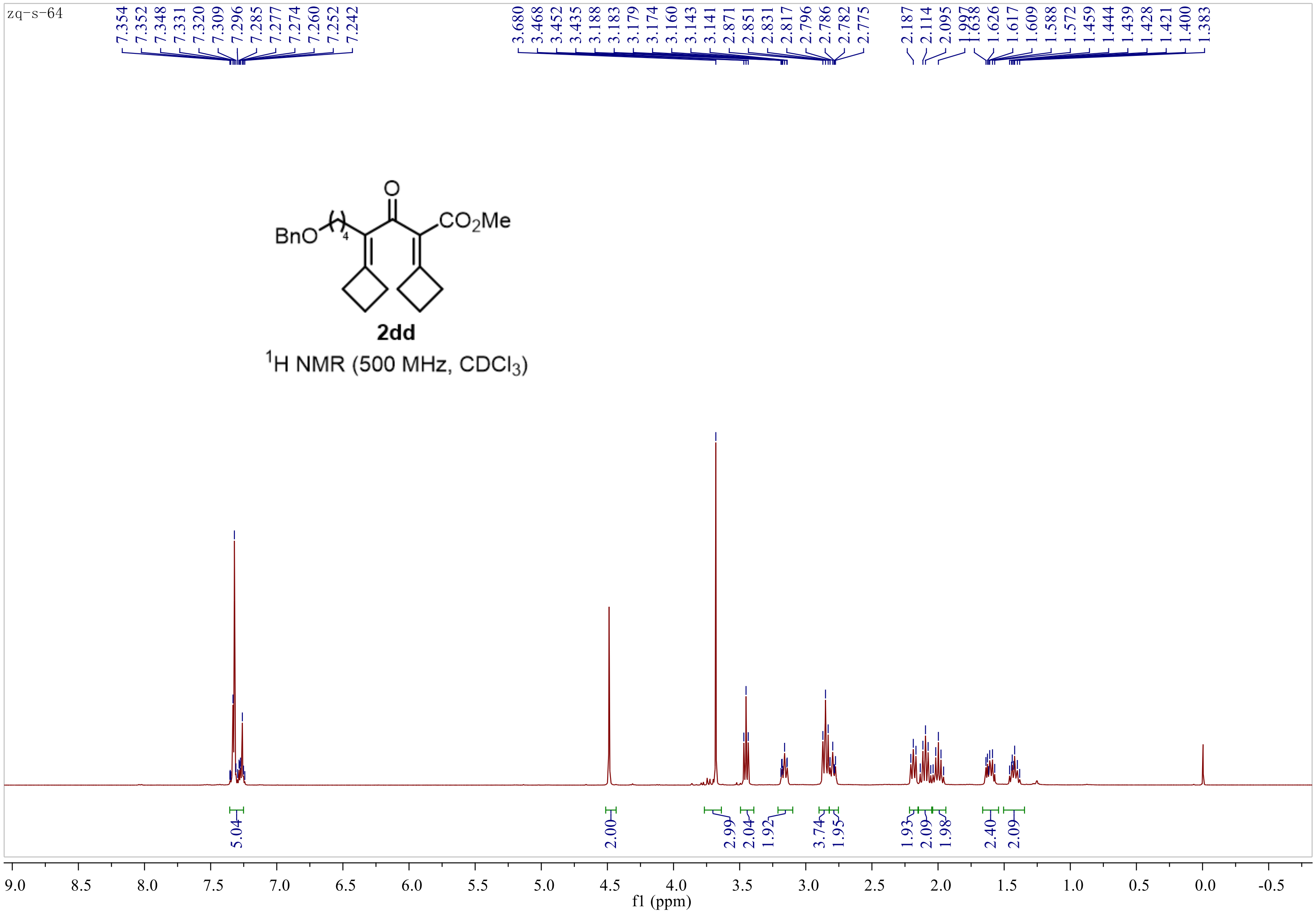

Supplementary Fig. 200. ^{1}H NMR spectra of compound **2dd** in $CDCl_3$

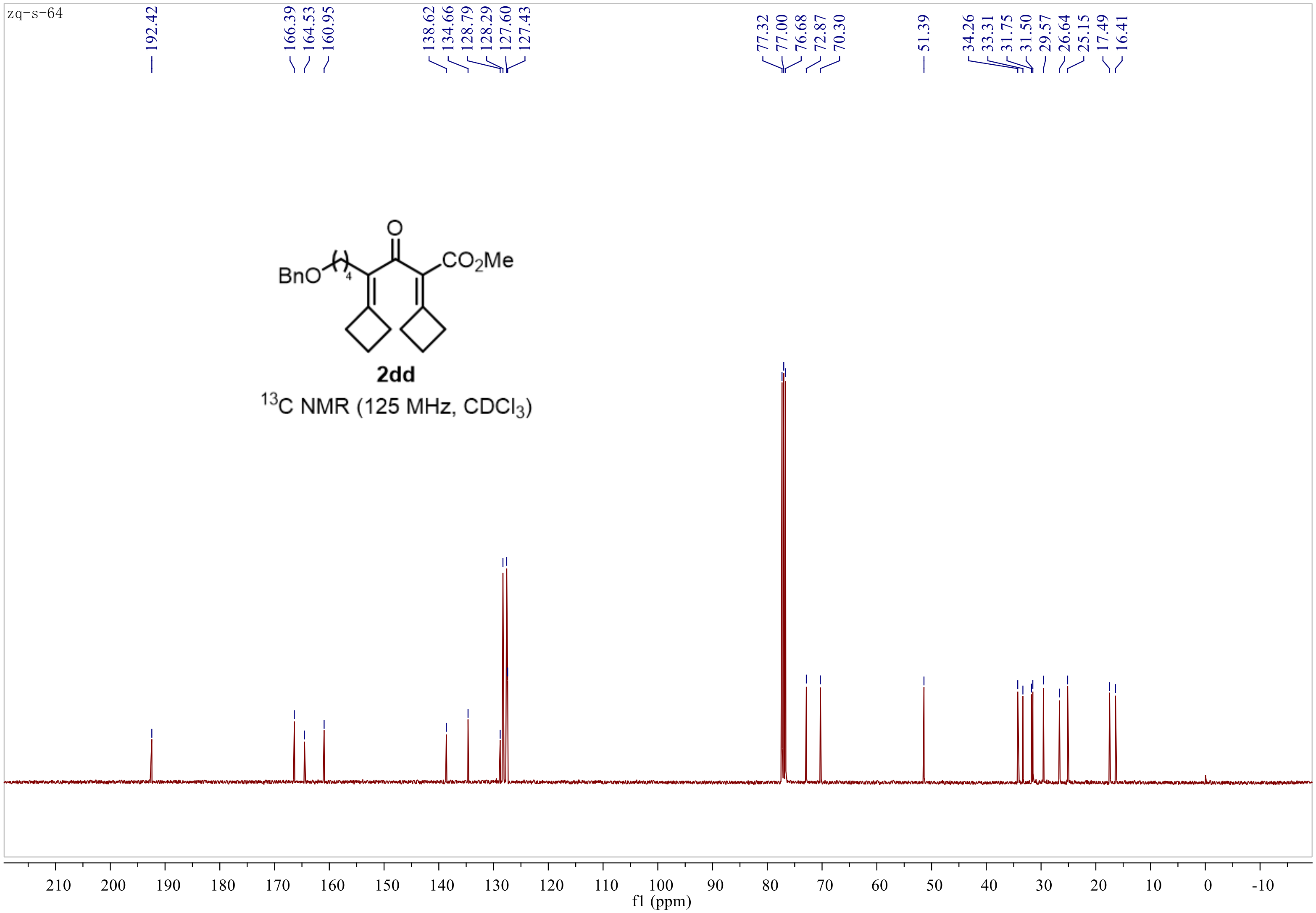

Supplementary Fig. 201. ^{13}C NMR spectra of compound **2dd** in $CDCl_3$

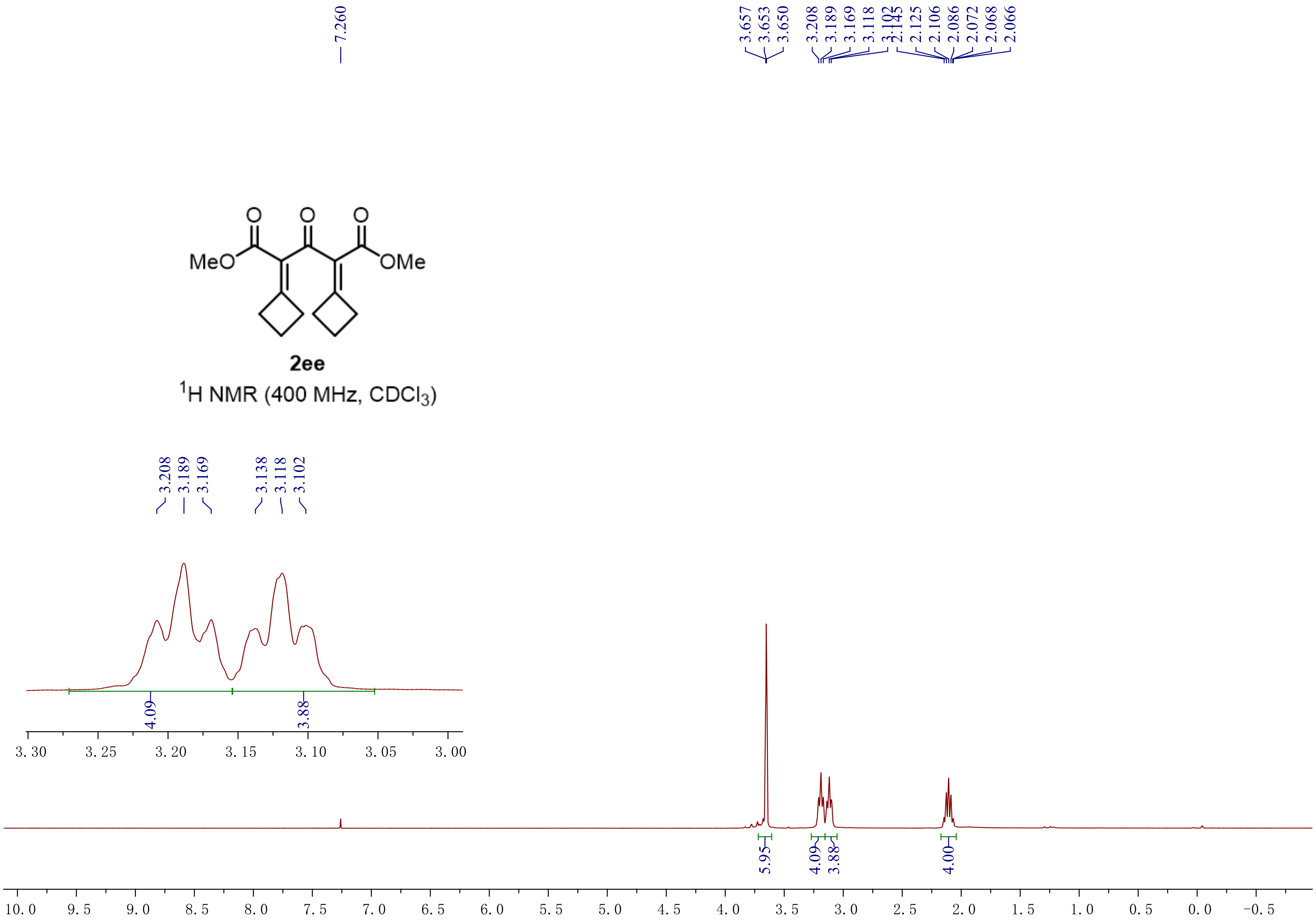

Supplementary Fig. 202. ^{1}H NMR spectra of compound **2ee** in $CDCl_3$

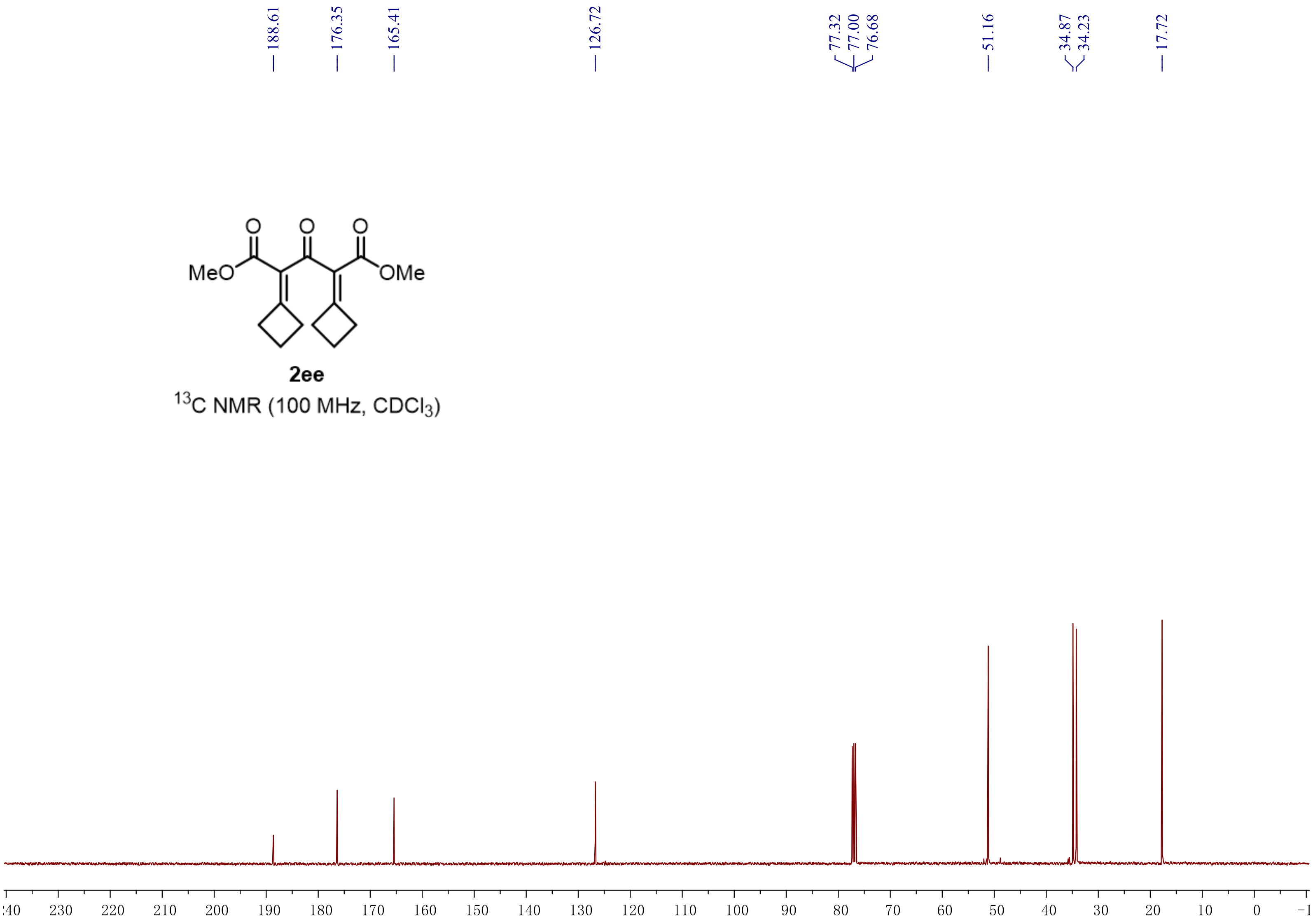

Supplementary Fig. 203. ^{13}C NMR spectra of compound **2ee** in $CDCl_3$

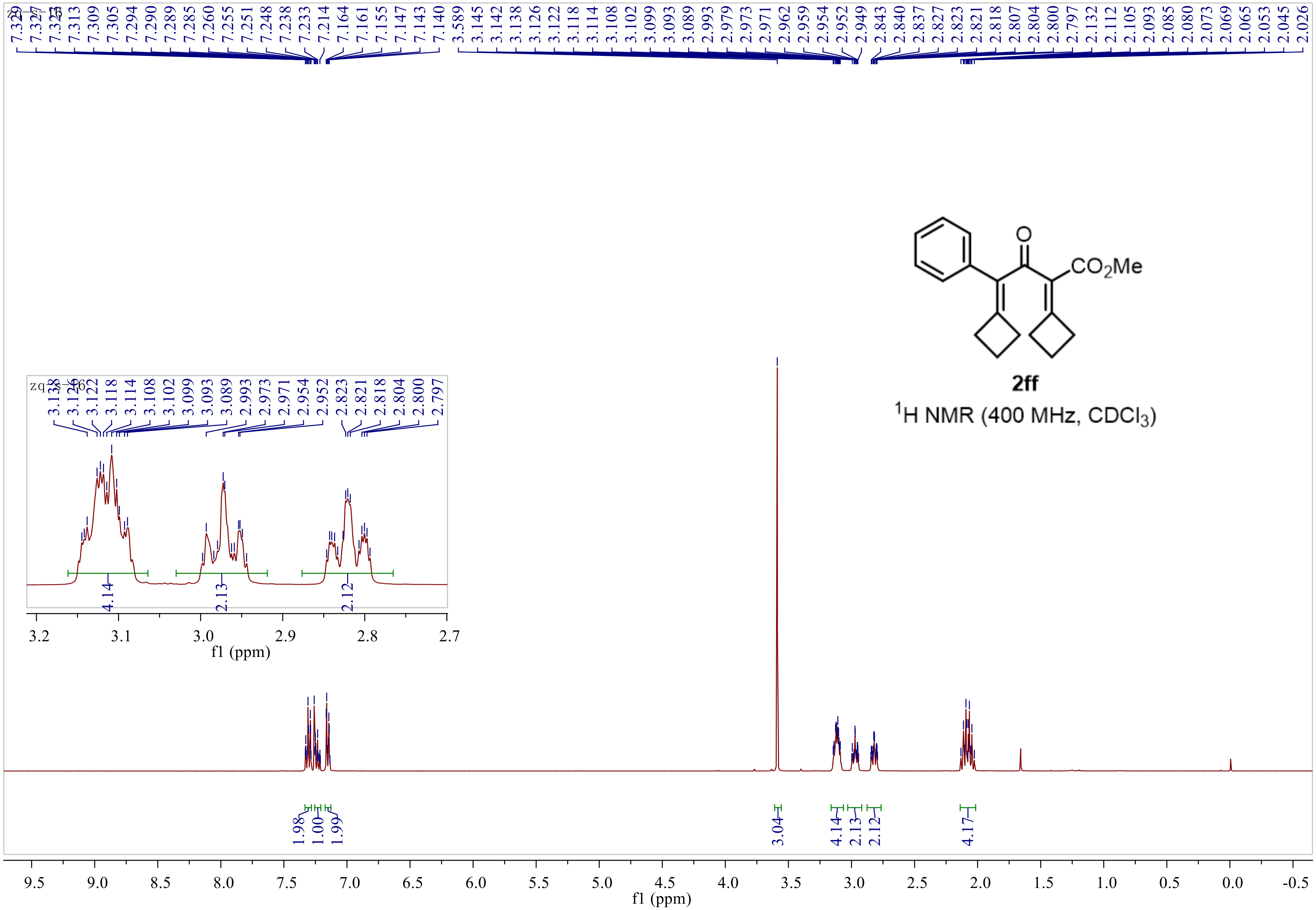

Supplementary Fig. 204. ^{1}H NMR spectra of compound **2ff** in $CDCl_3$

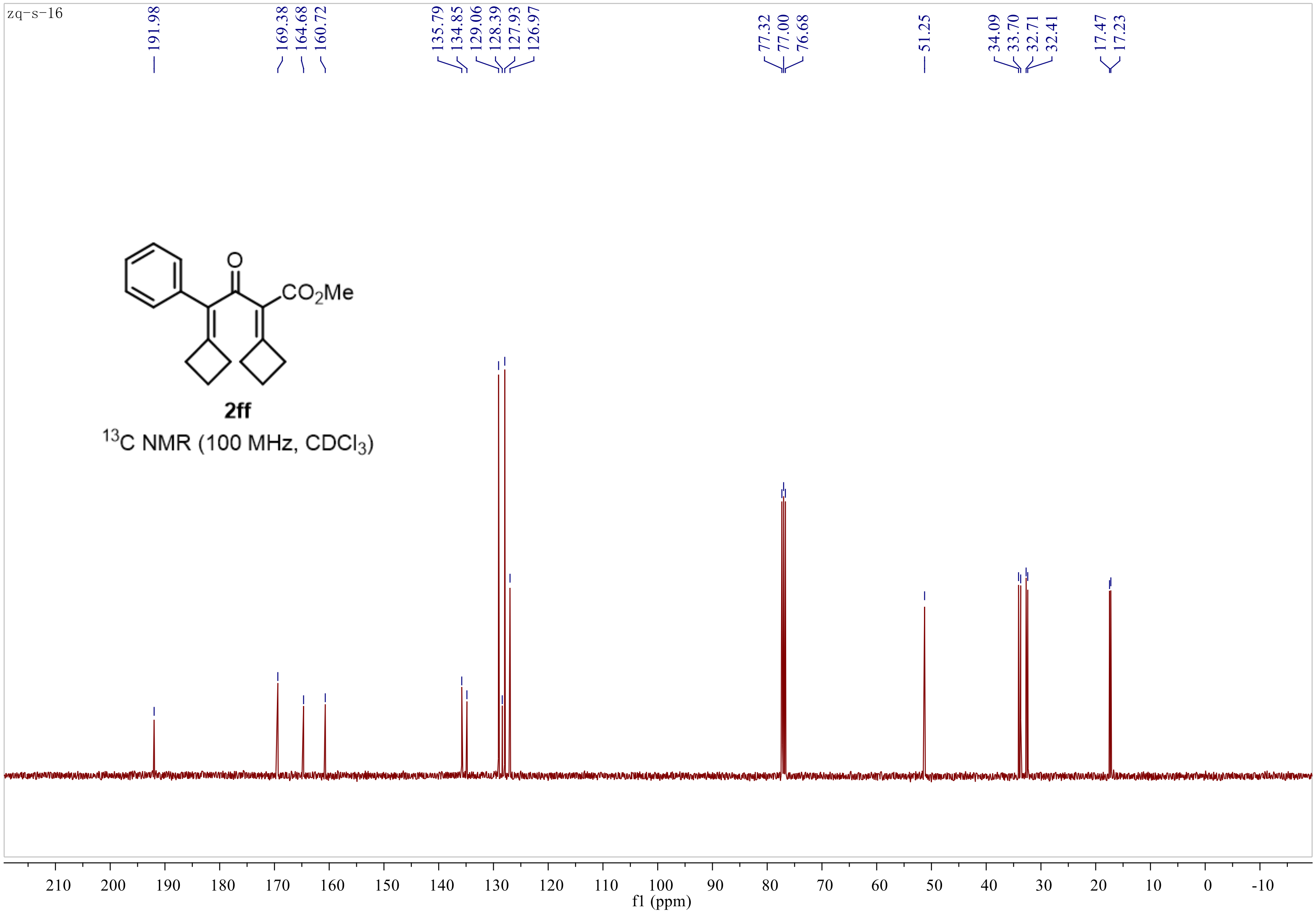

Supplementary Fig. 205. ^{13}C NMR spectra of compound **2ff** in $CDCl_3$

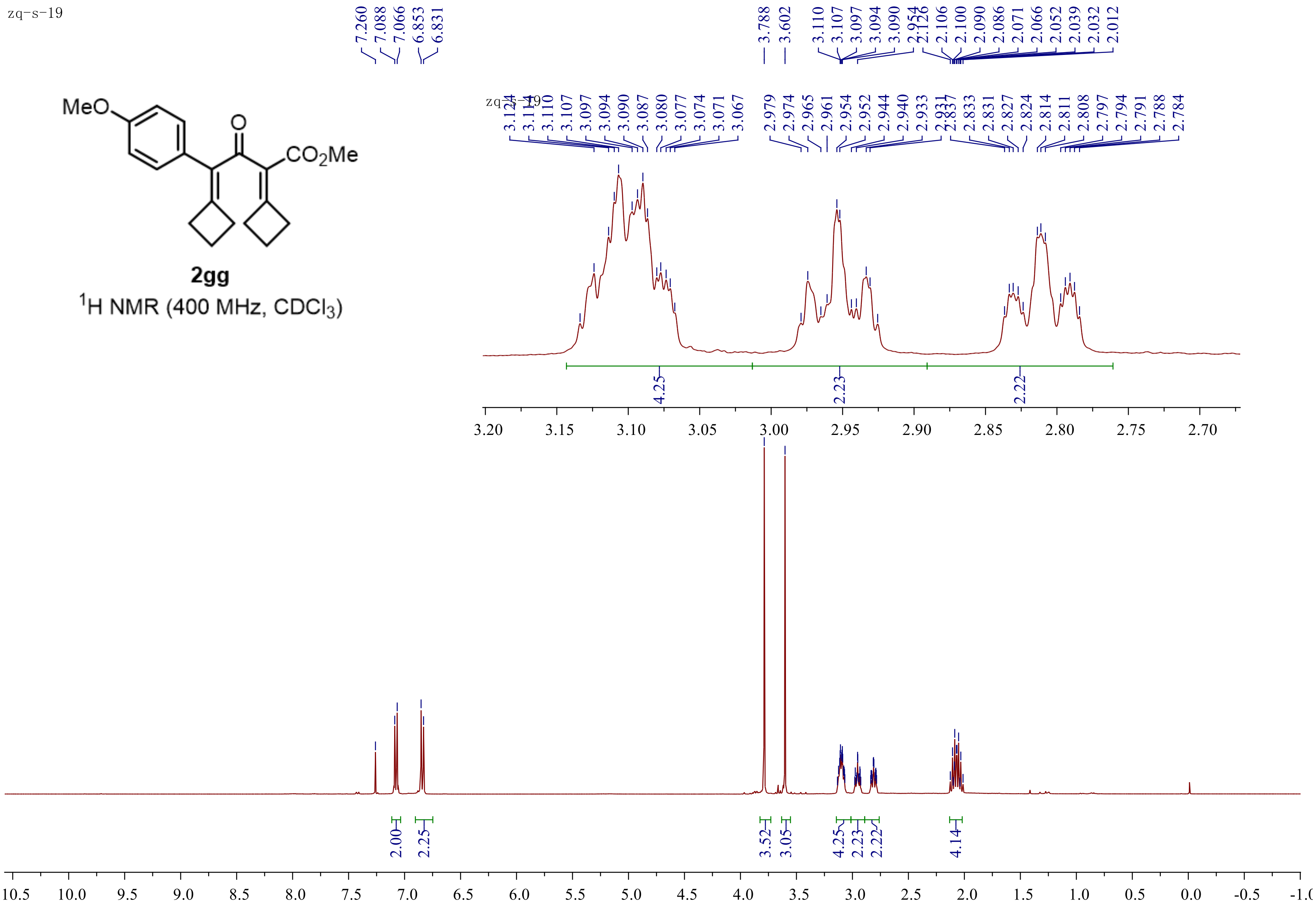

Supplementary Fig. 206. ^1H NMR spectra of compound **2gg** in $CDCl_3$

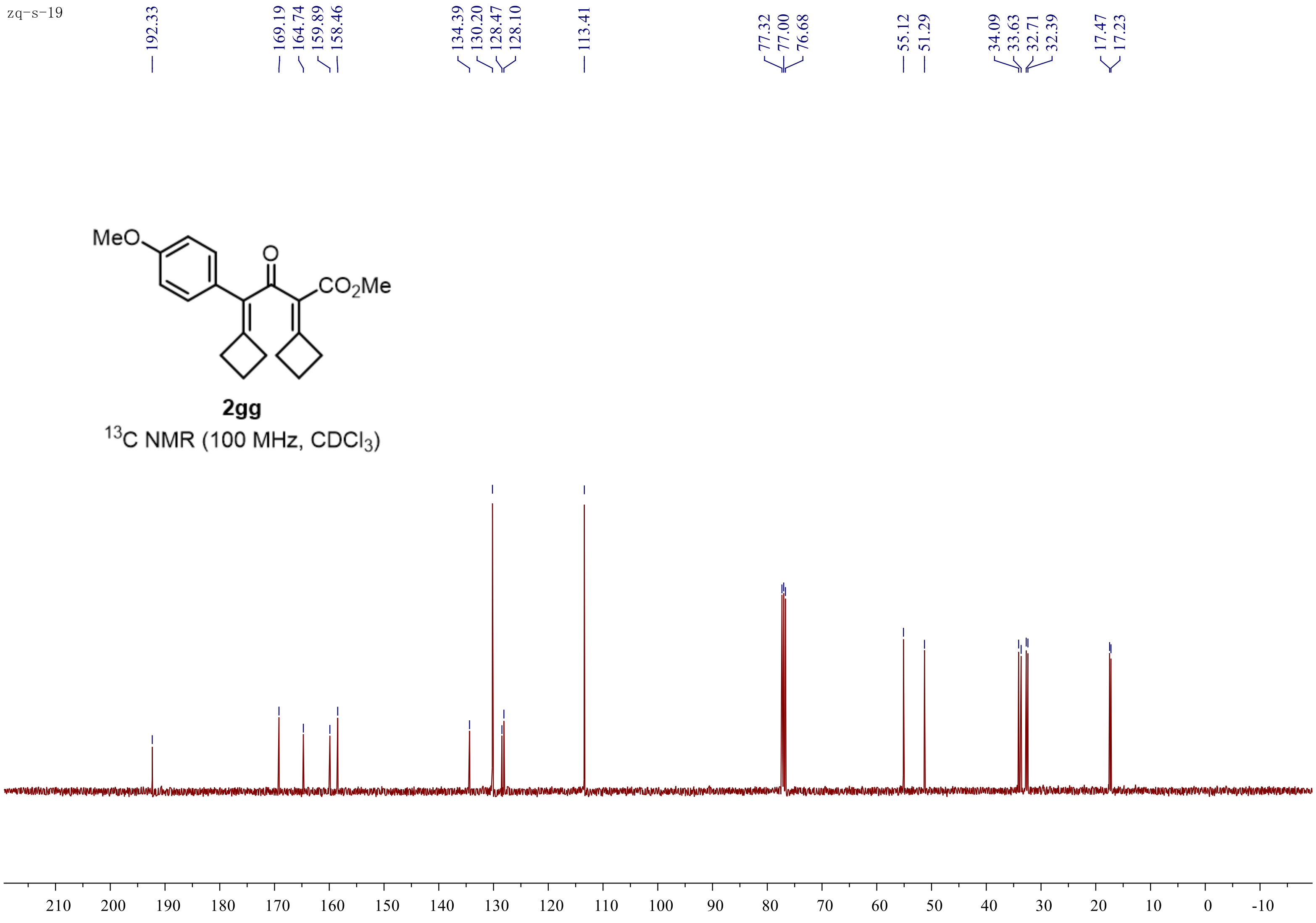

Supplementary Fig. 207. ^{13}C NMR spectra of compound **2gg** in $CDCl_3$

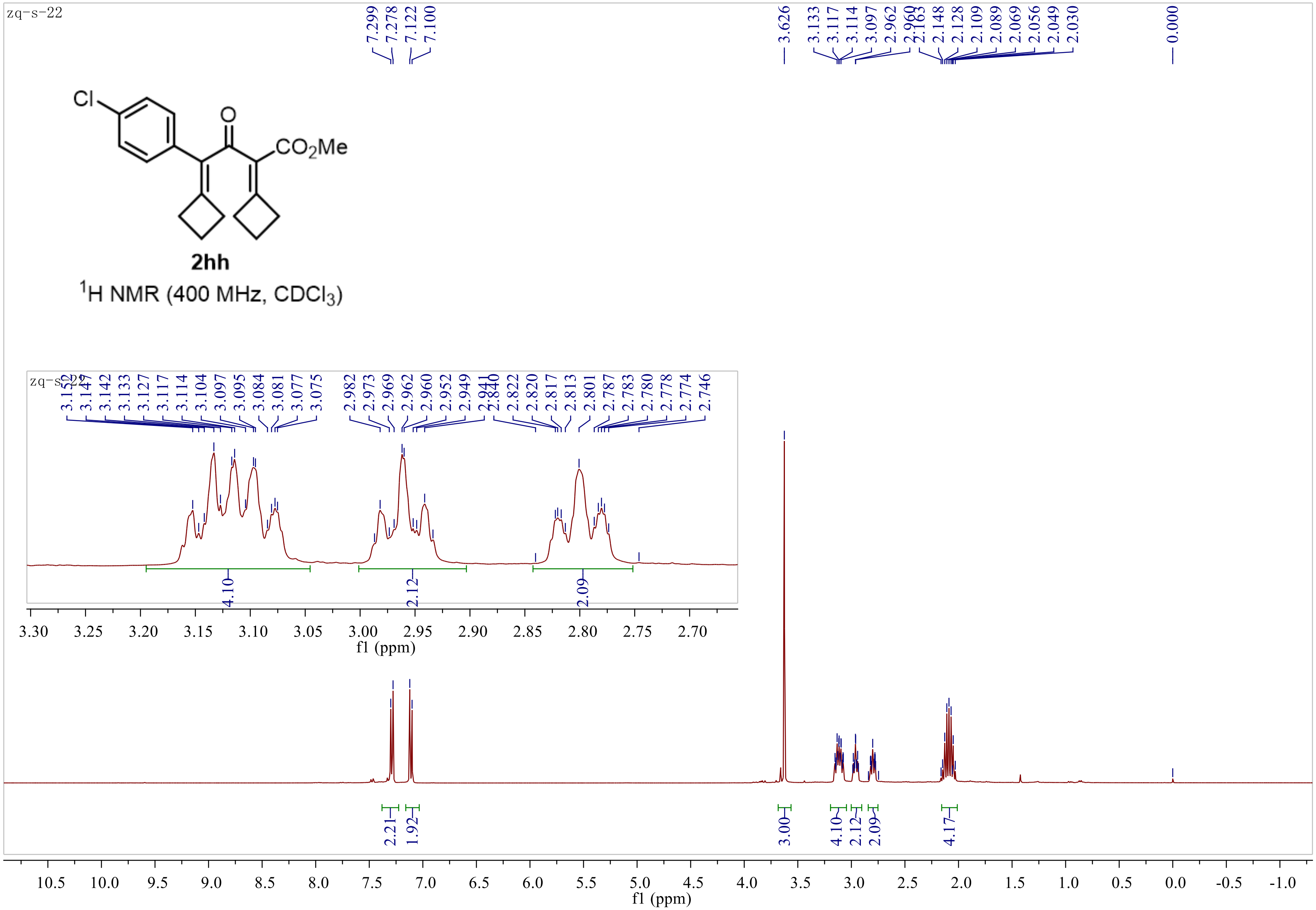

Supplementary Fig. 208. ¹H NMR spectra of compound **2hh** in $CDCl_3$

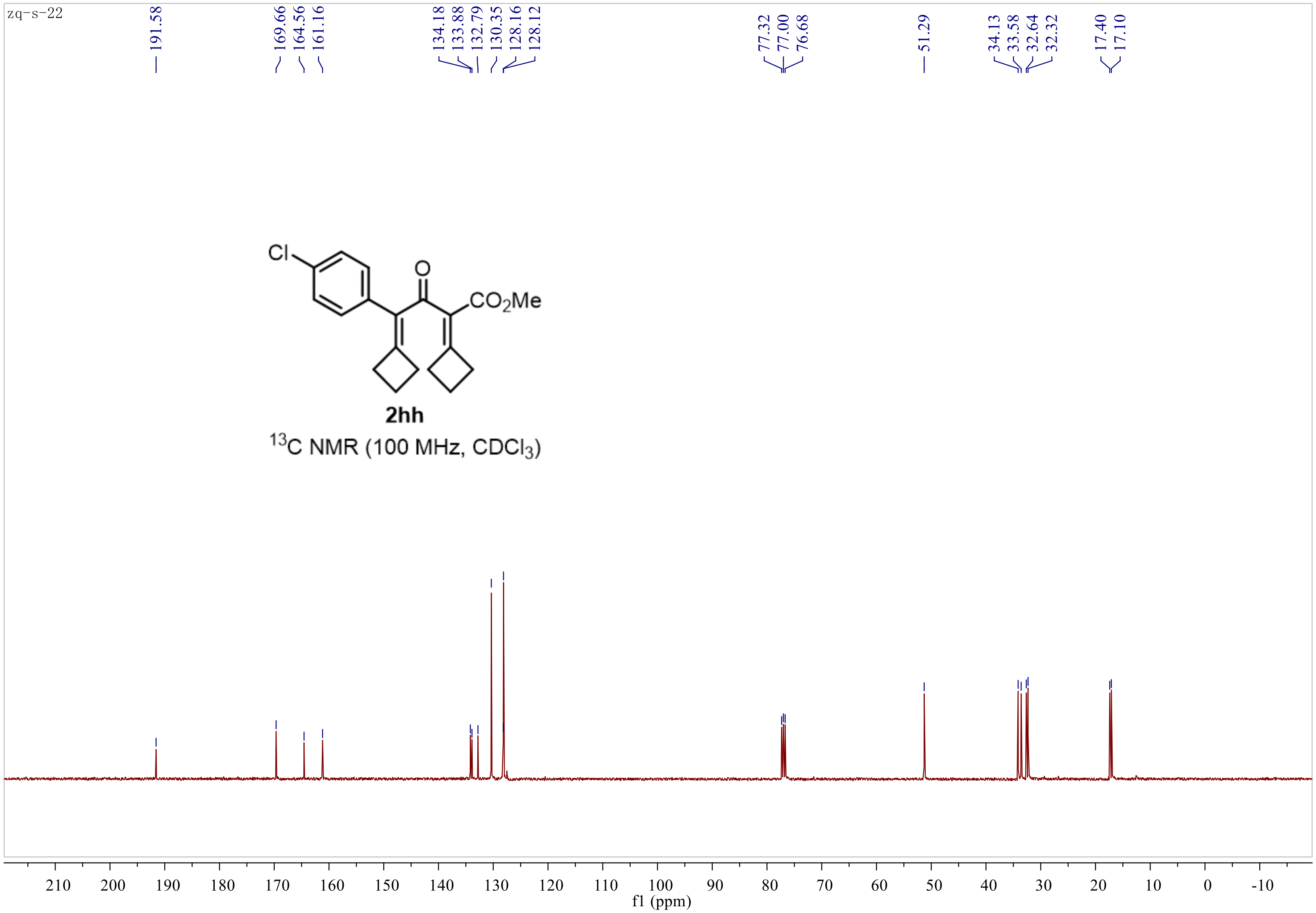

Supplementary Fig. 209. ^{13}C NMR spectra of compound **2hh** in $CDCl_3$

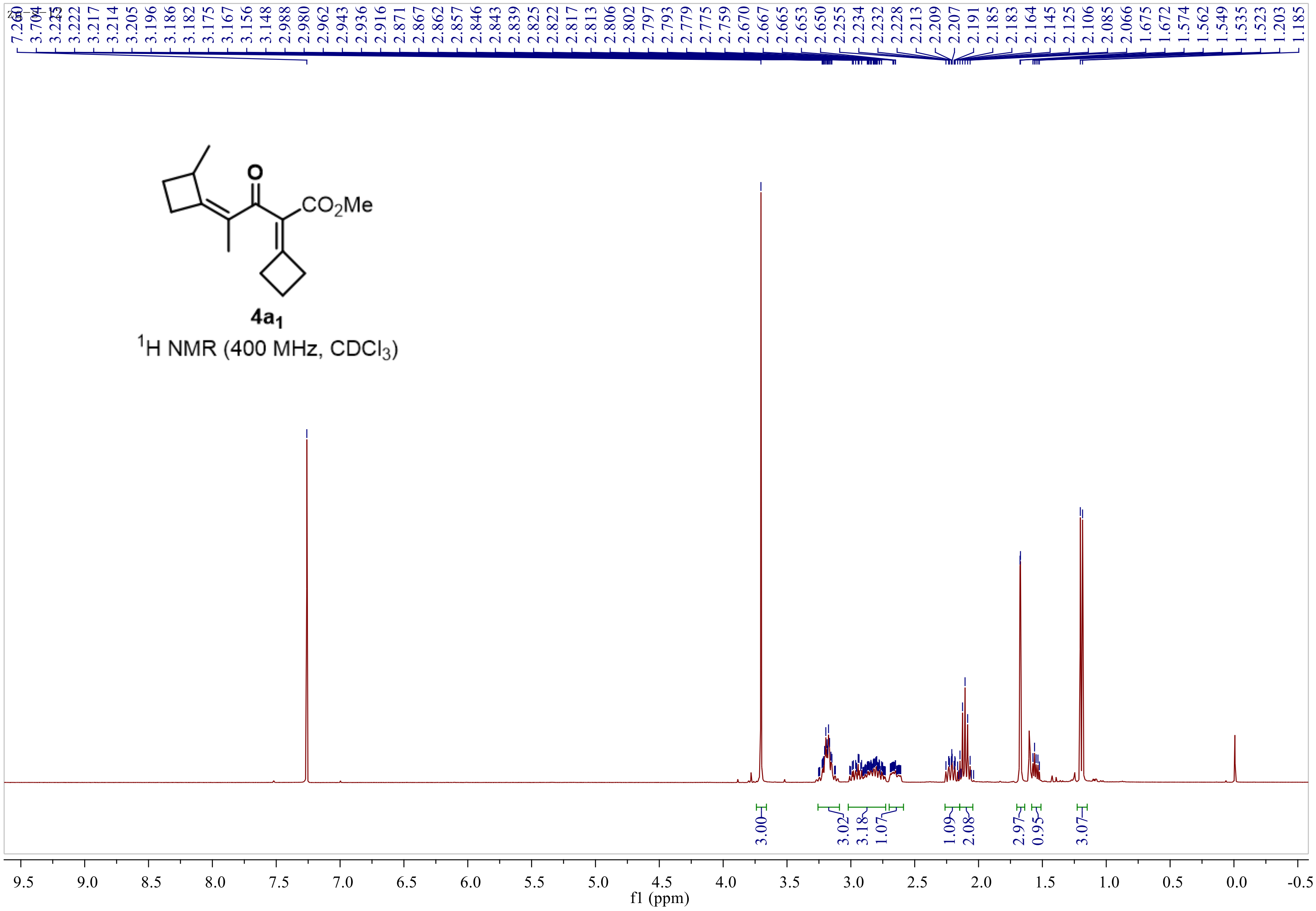

Supplementary Fig. 210. ^{1}H NMR spectra of compound **4a$_1$** in $CDCl_3$

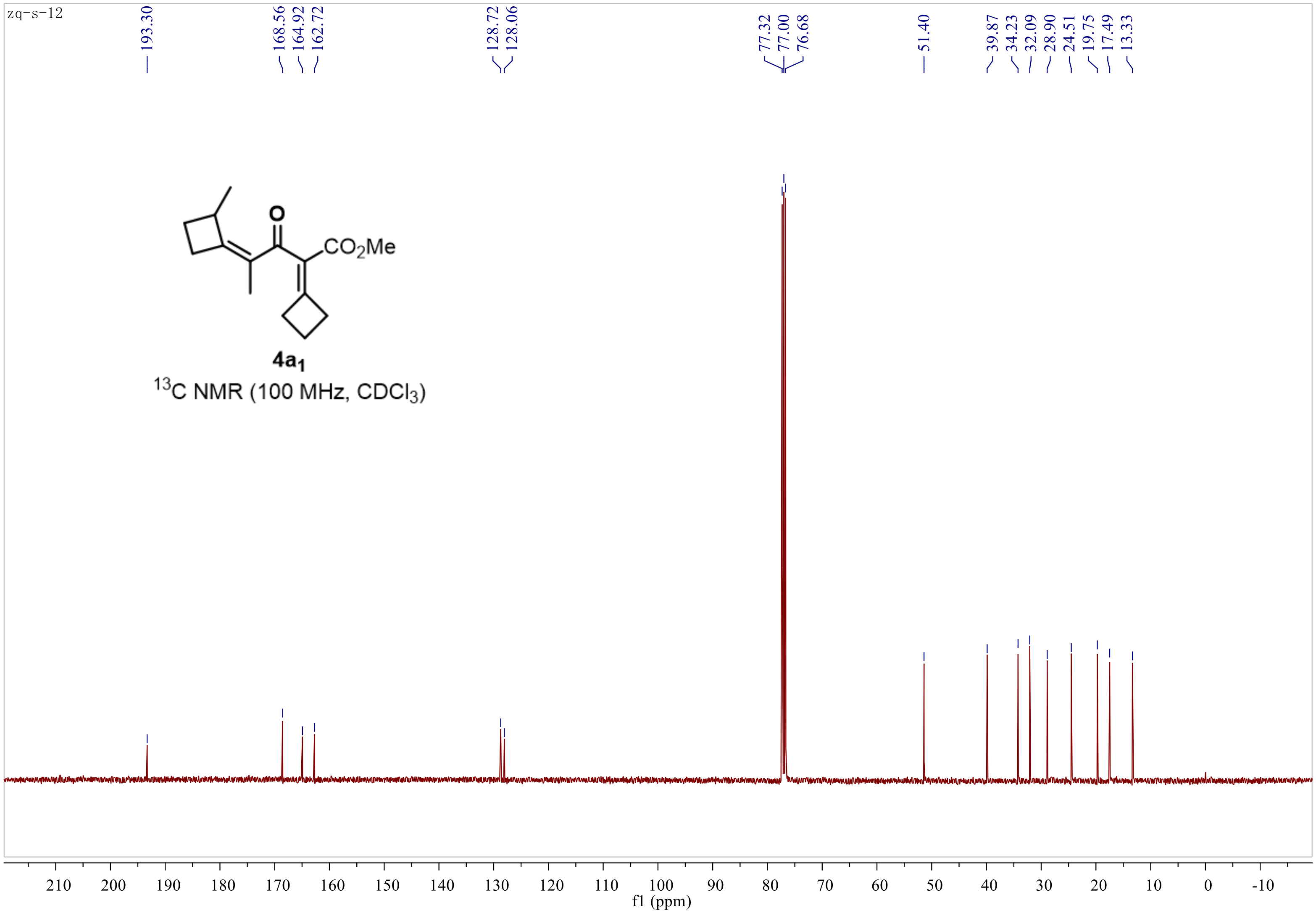

Supplementary Fig. 211. ^{13}C NMR spectra of compound **4a₁** in $CDCl_3$

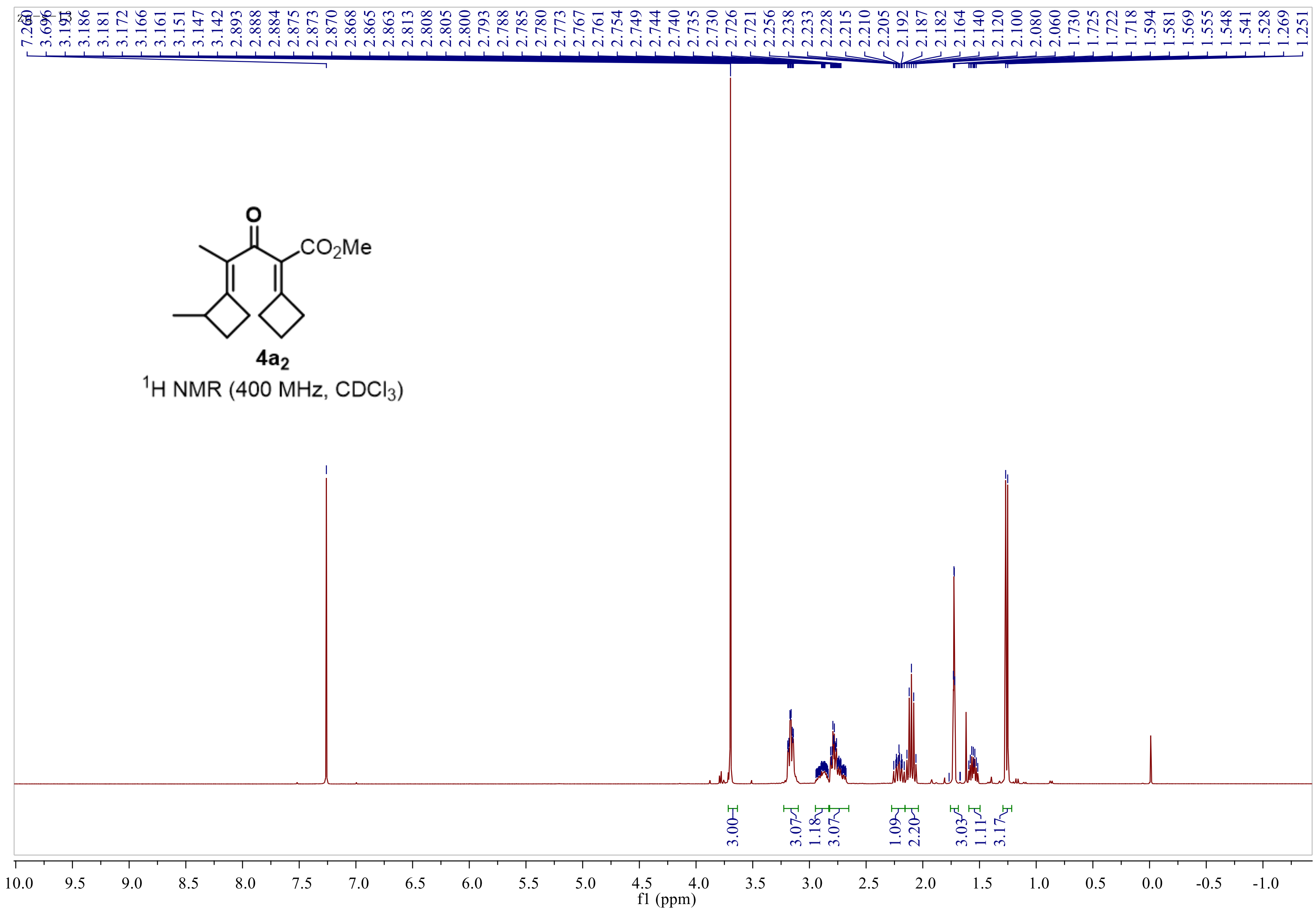

Supplementary Fig. 212. ^{1}H NMR spectra of compound **4a$_2$** in $CDCl_3$

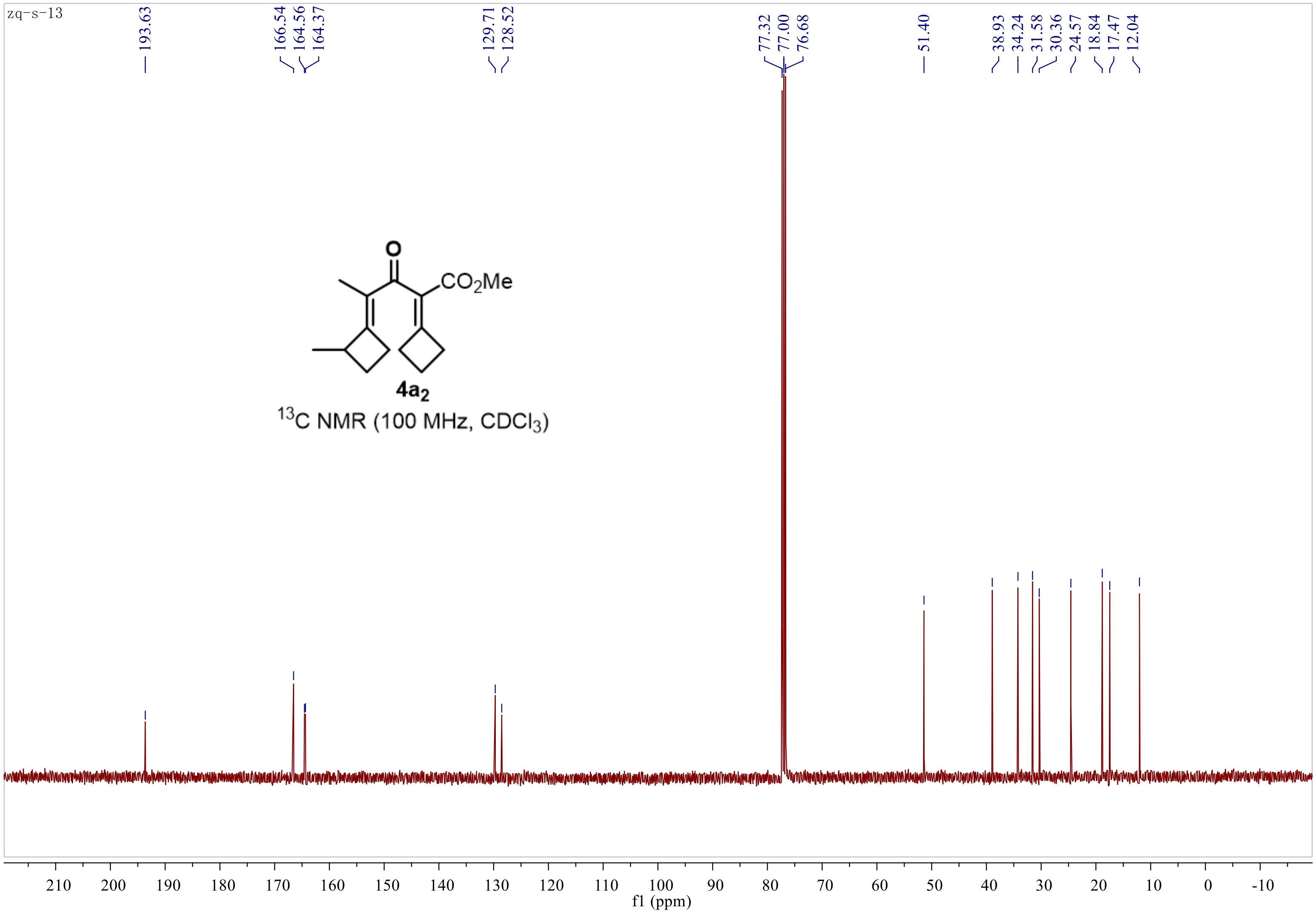

Supplementary Fig. 213. ^{13}C NMR spectra of compound **4a₂** in $CDCl_3$

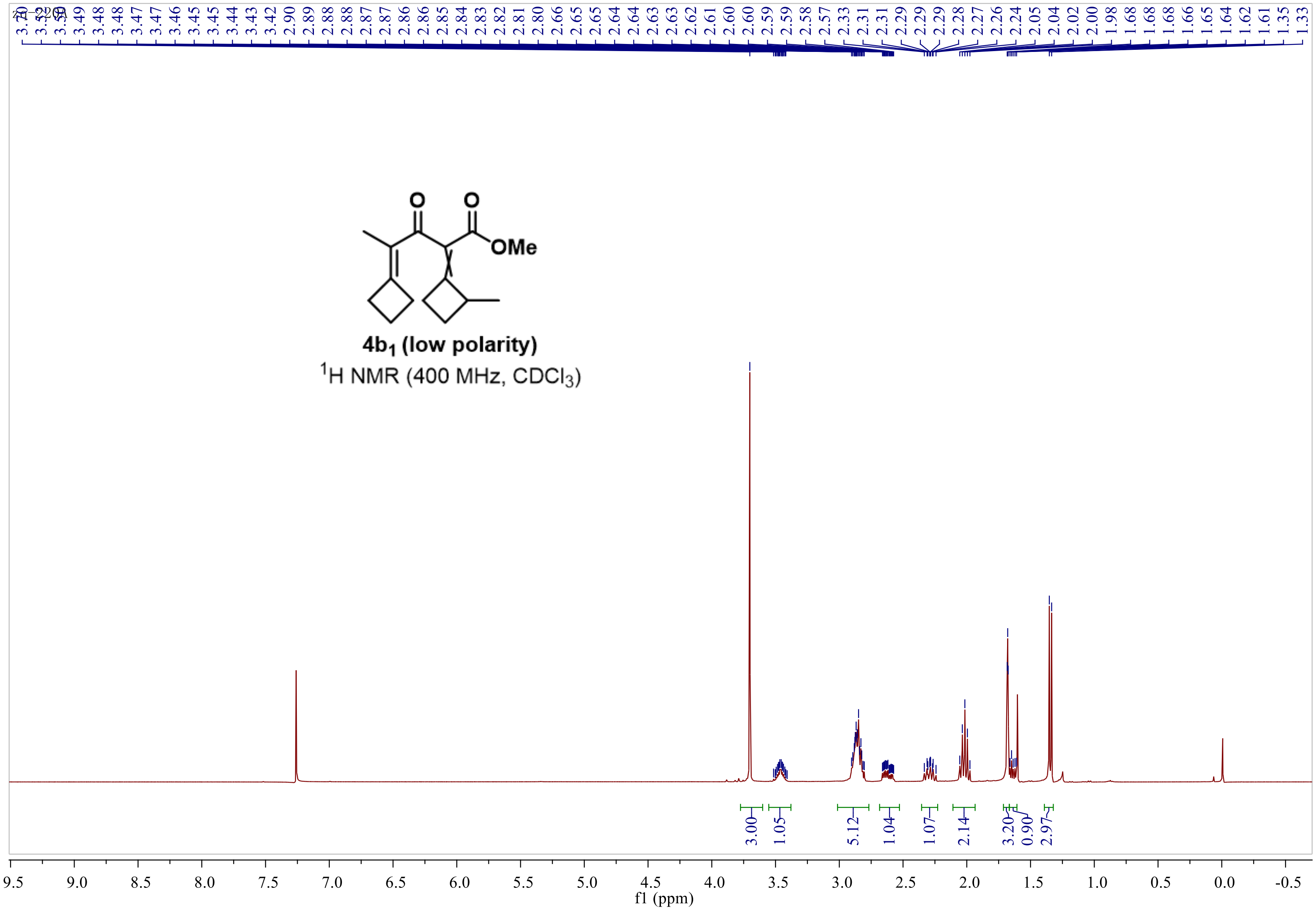

Supplementary Fig. 214. ^{1}H NMR spectra of compound **4b₁** in $CDCl_3$

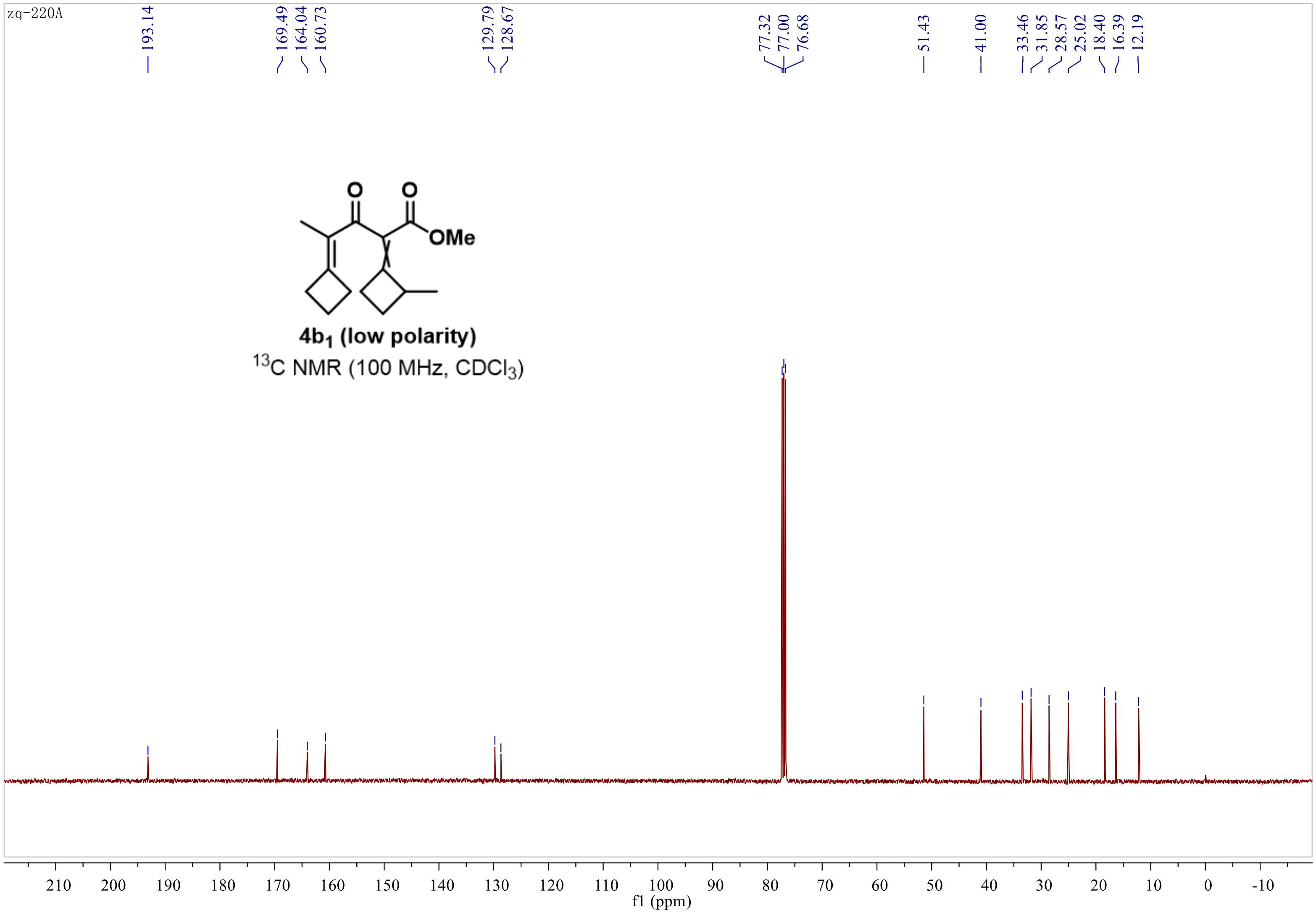

Supplementary Fig. 215. ^{13}C NMR spectra of compound **4b$_1$** in $CDCl_3$

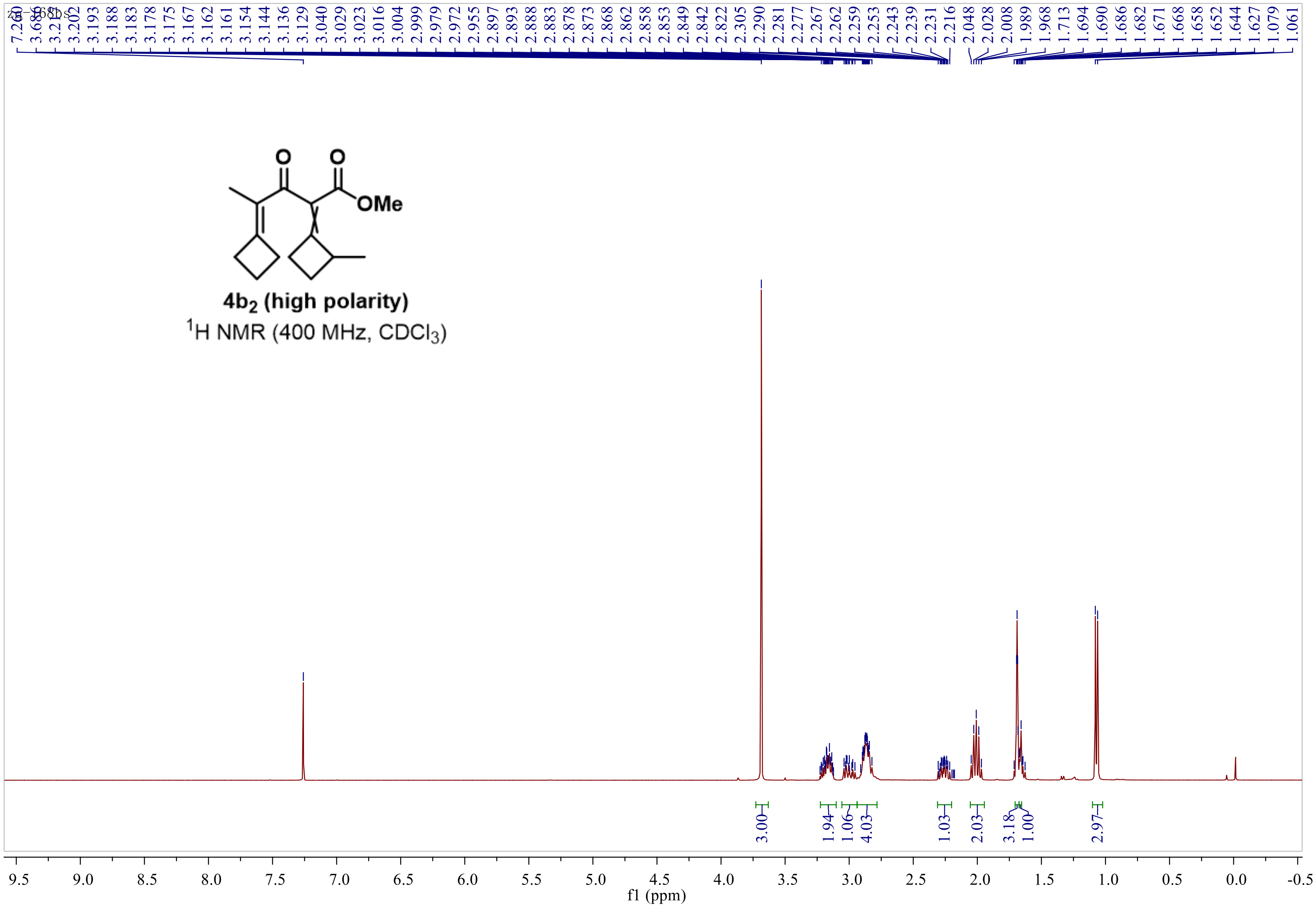

Supplementary Fig. 216. ^{1}H NMR spectra of compound **4b$_2$** in $CDCl_3$

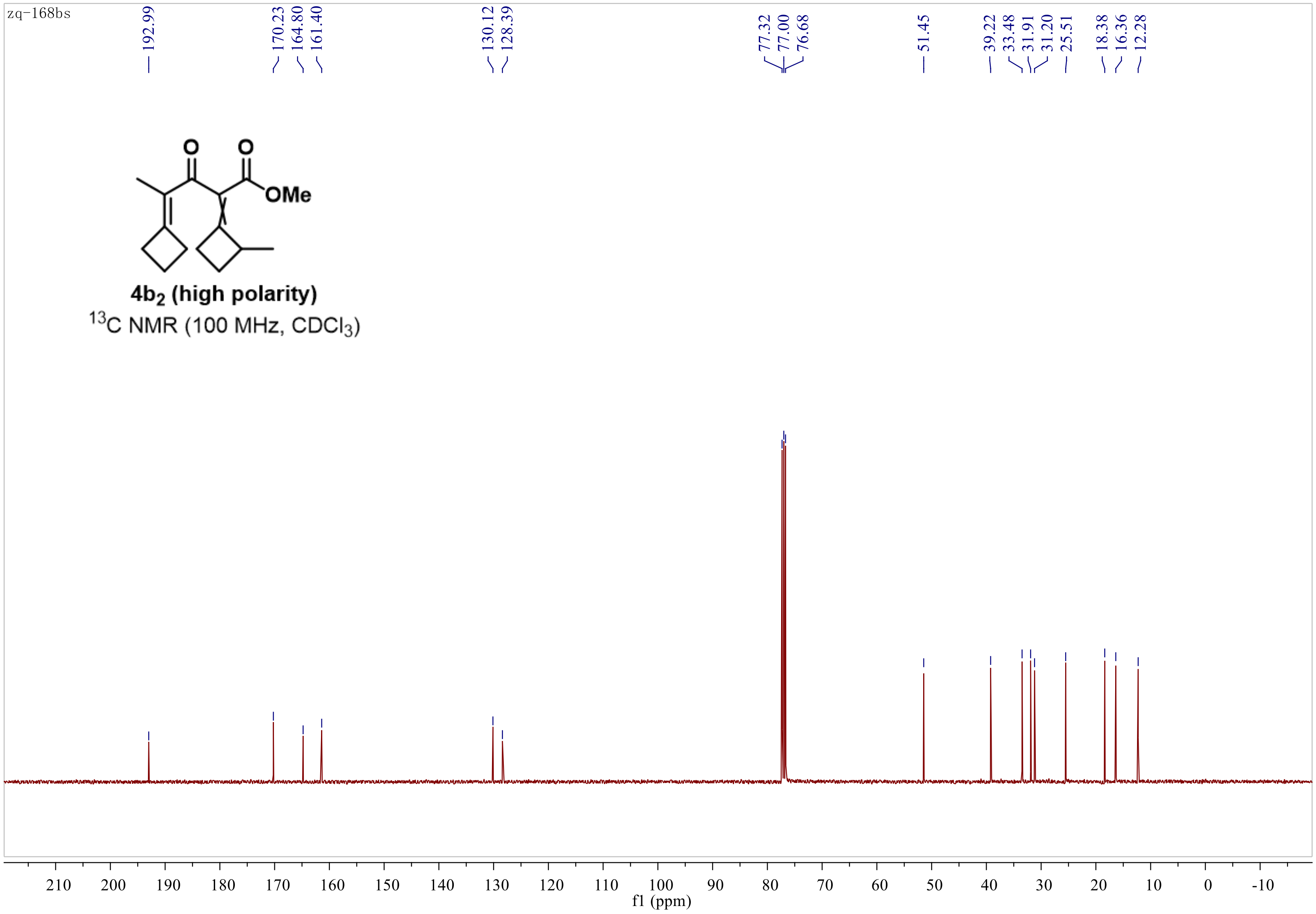

Supplementary Fig. 217. ^{13}C NMR spectra of compound **4b$_2$** in $CDCl_3$

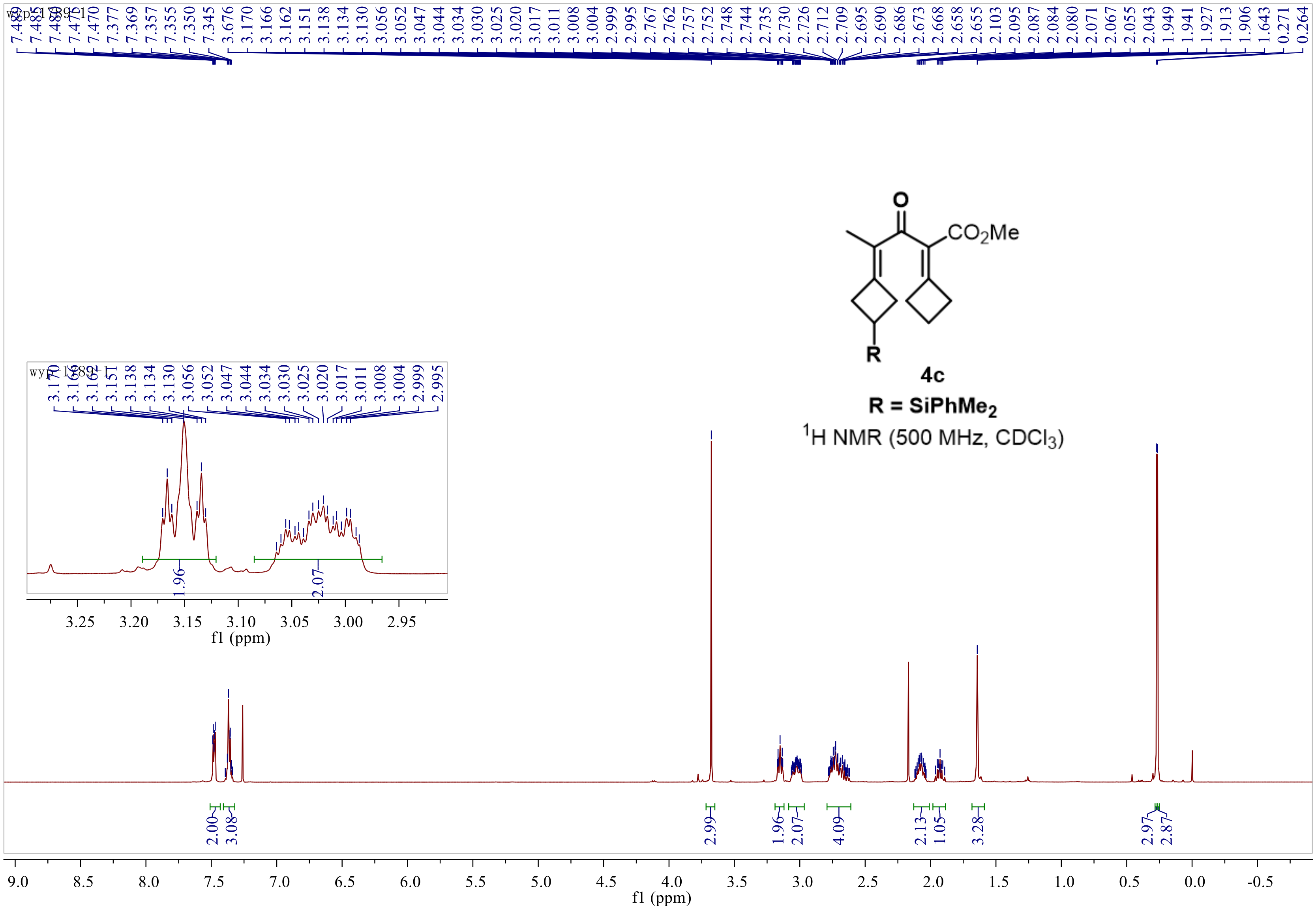

Supplementary Fig. 218. ^{1}H NMR spectra of compound **4c** in $CDCl_3$

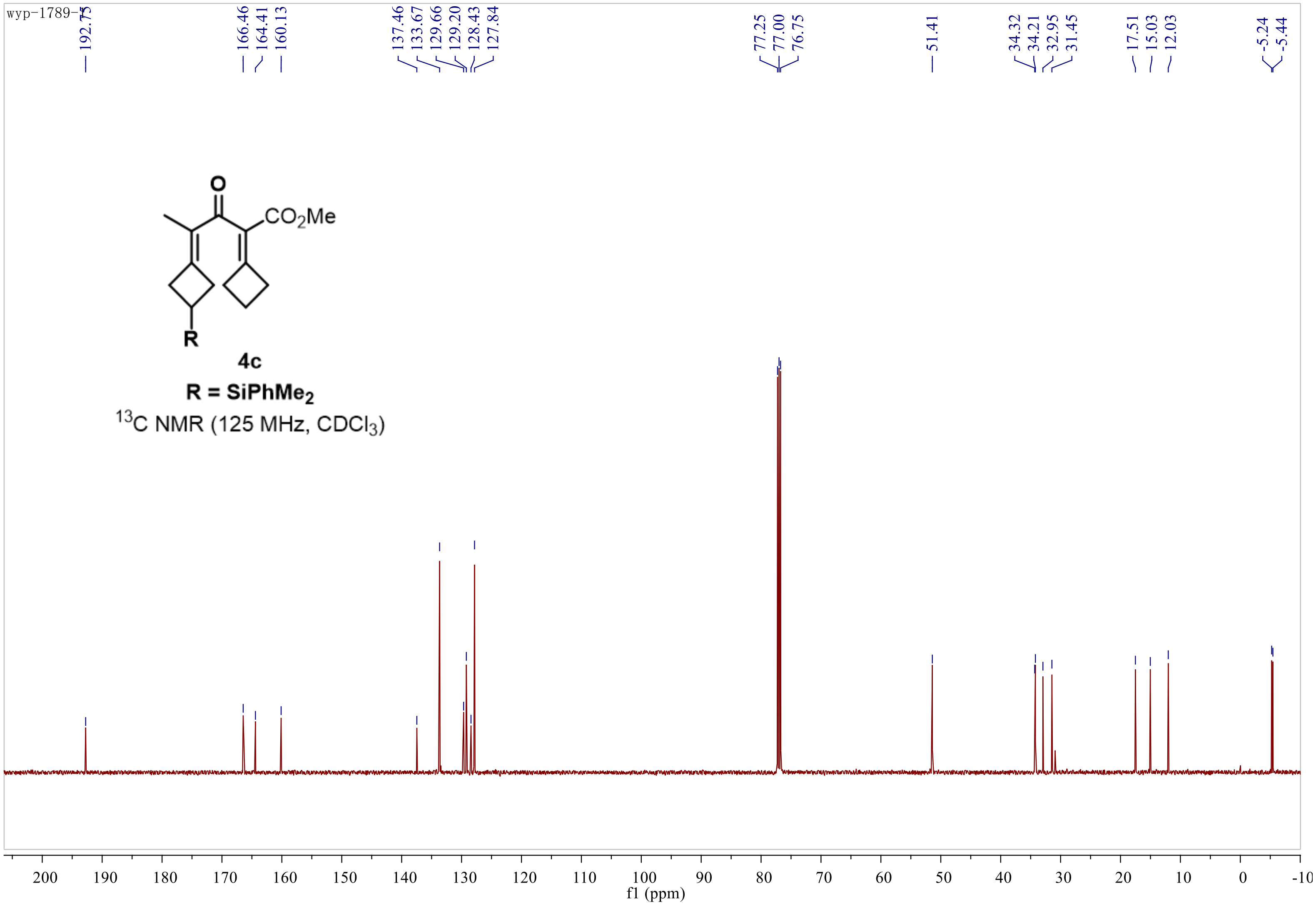

Supplementary Fig. 219. ^{13}C NMR spectra of compound **4c** in $CDCl_3$

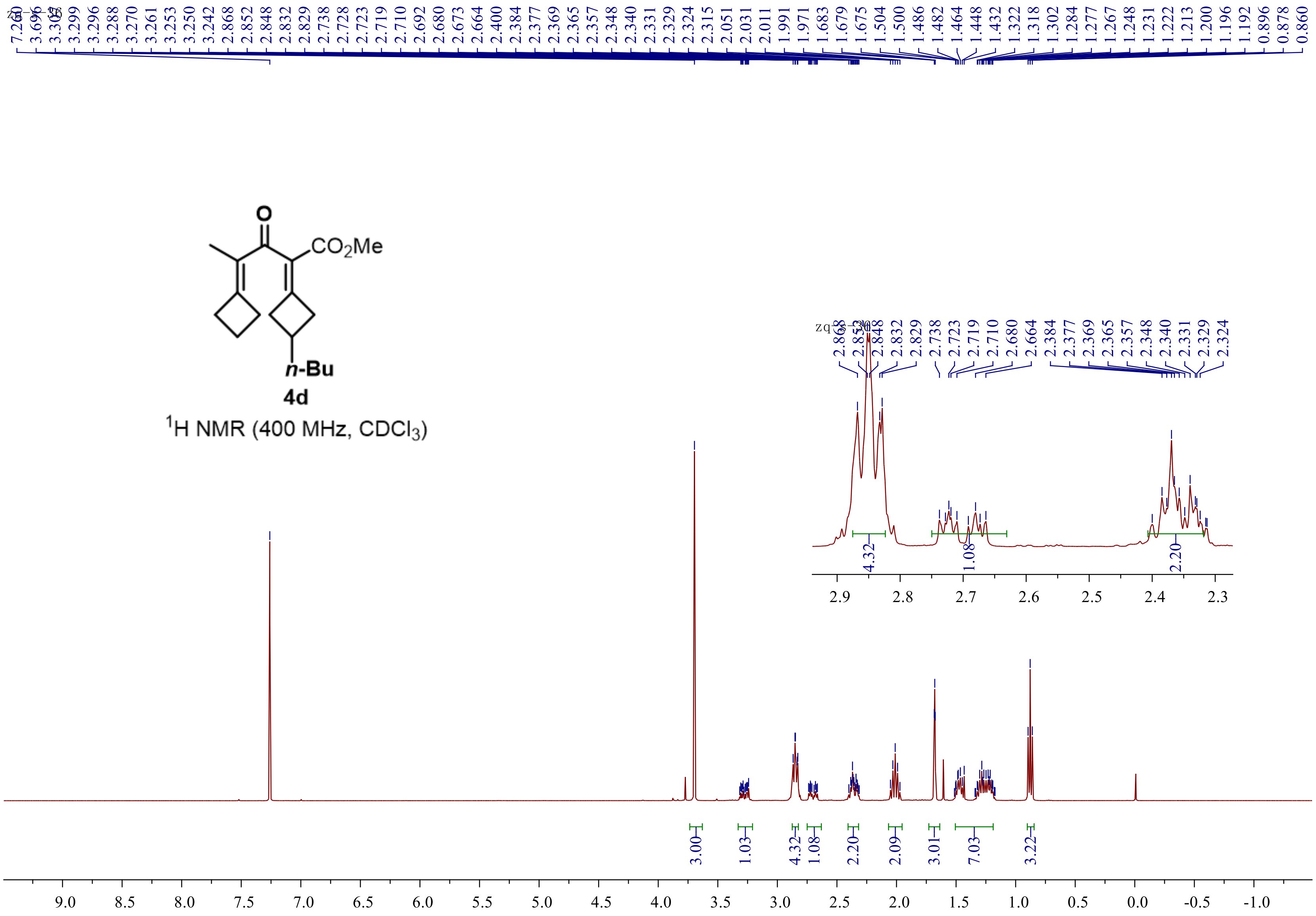

Supplementary Fig. 220. ¹H NMR spectra of compound **4d** in $CDCl_3$

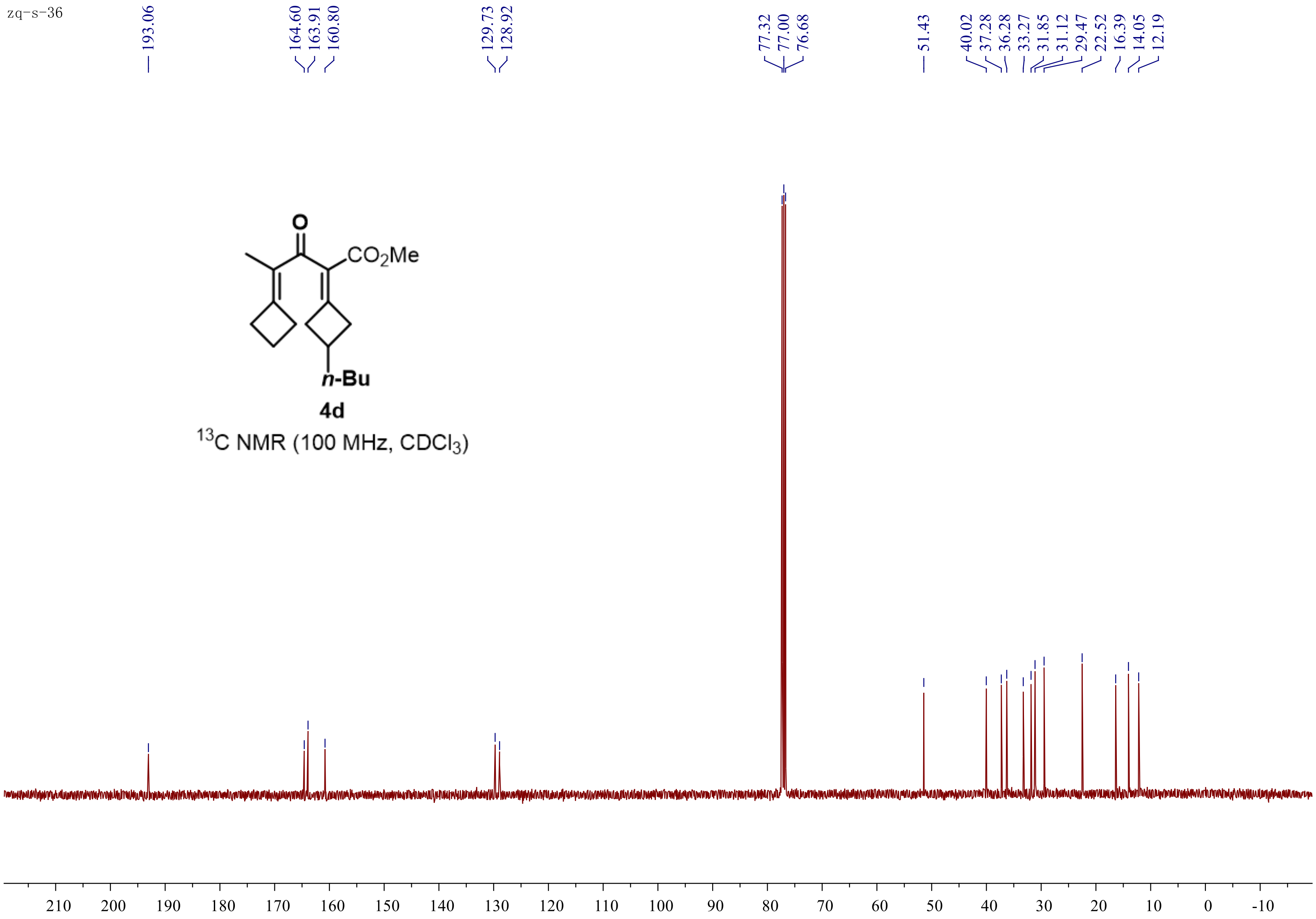

Supplementary Fig. 221. ^{13}C NMR spectra of compound **4d** in $CDCl_3$

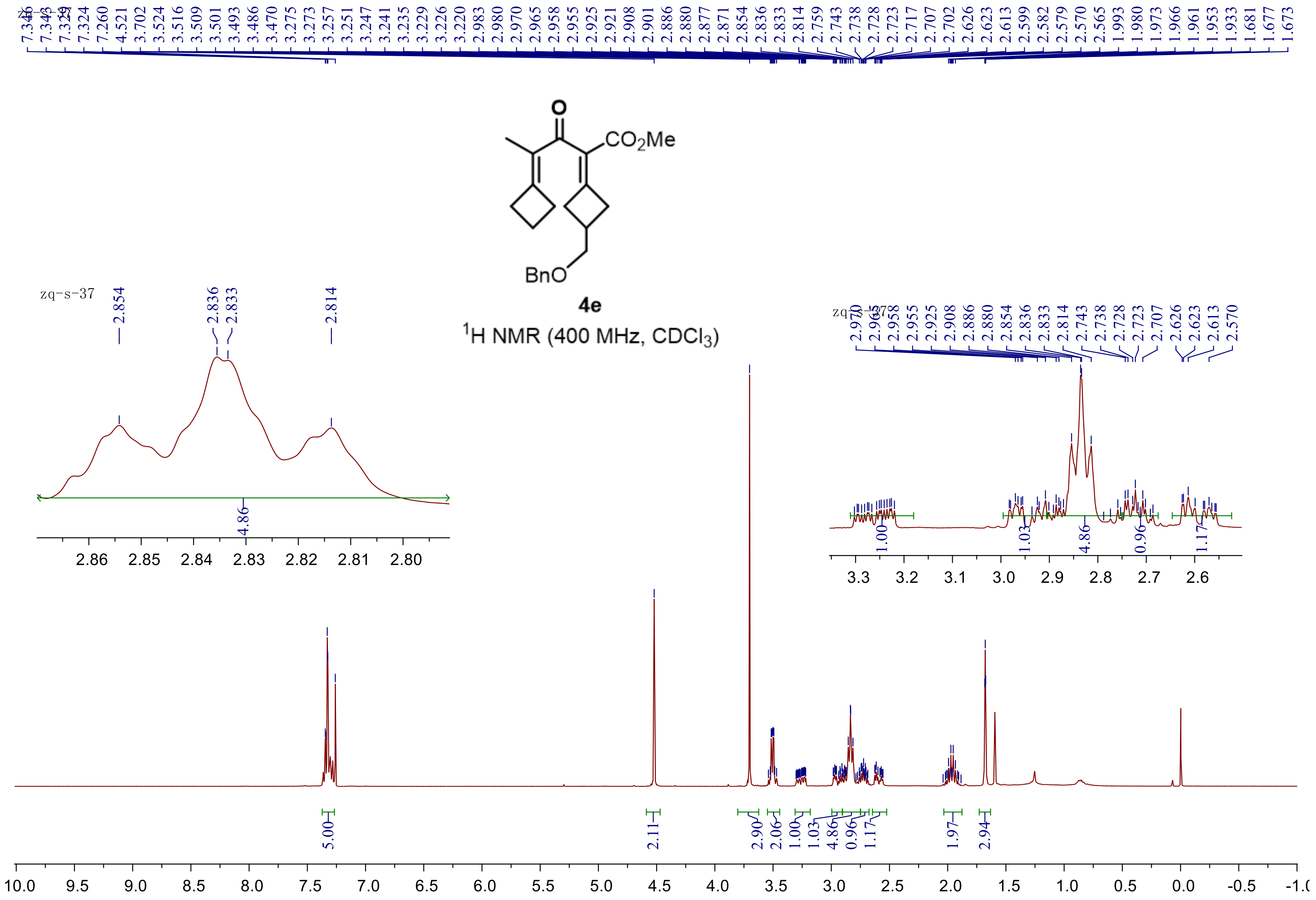

Supplementary Fig. 222. ^{1}H NMR spectra of compound **4e** in $CDCl_3$

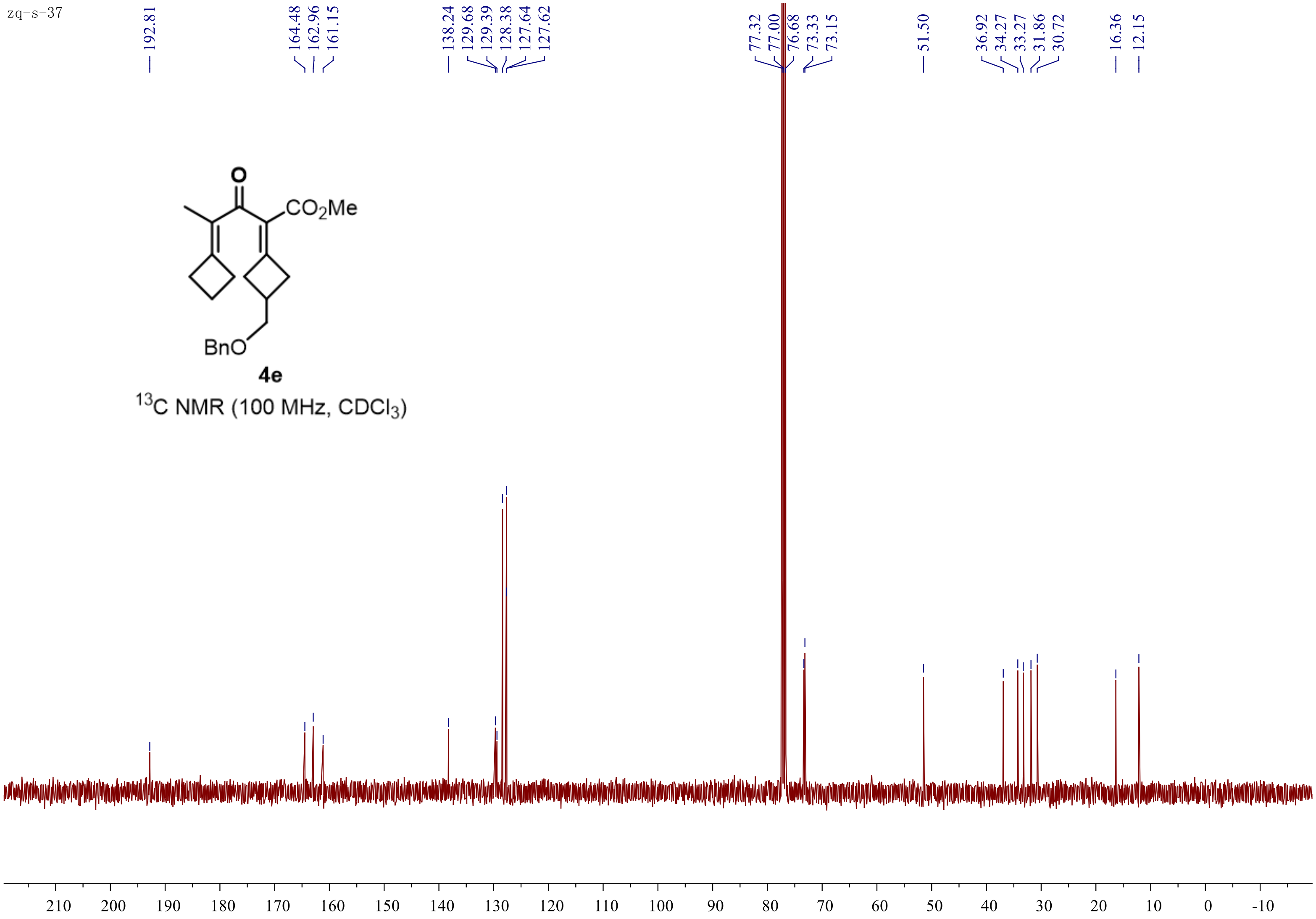

Supplementary Fig. 223. ^{13}C NMR spectra of compound **4e** in $CDCl_3$

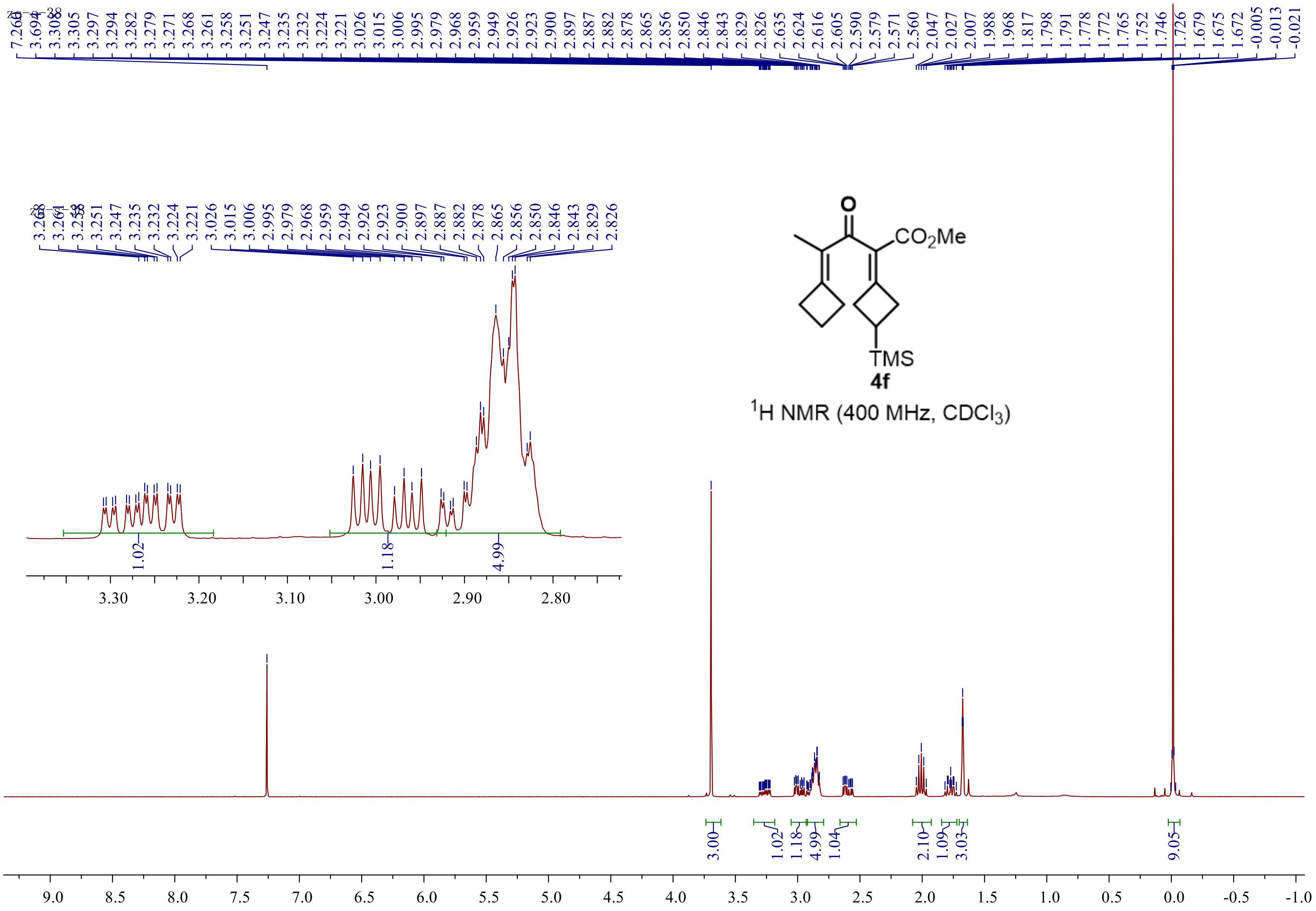

Supplementary Fig. 224. ^{1}H NMR spectra of compound **4f** in $CDCl_3$

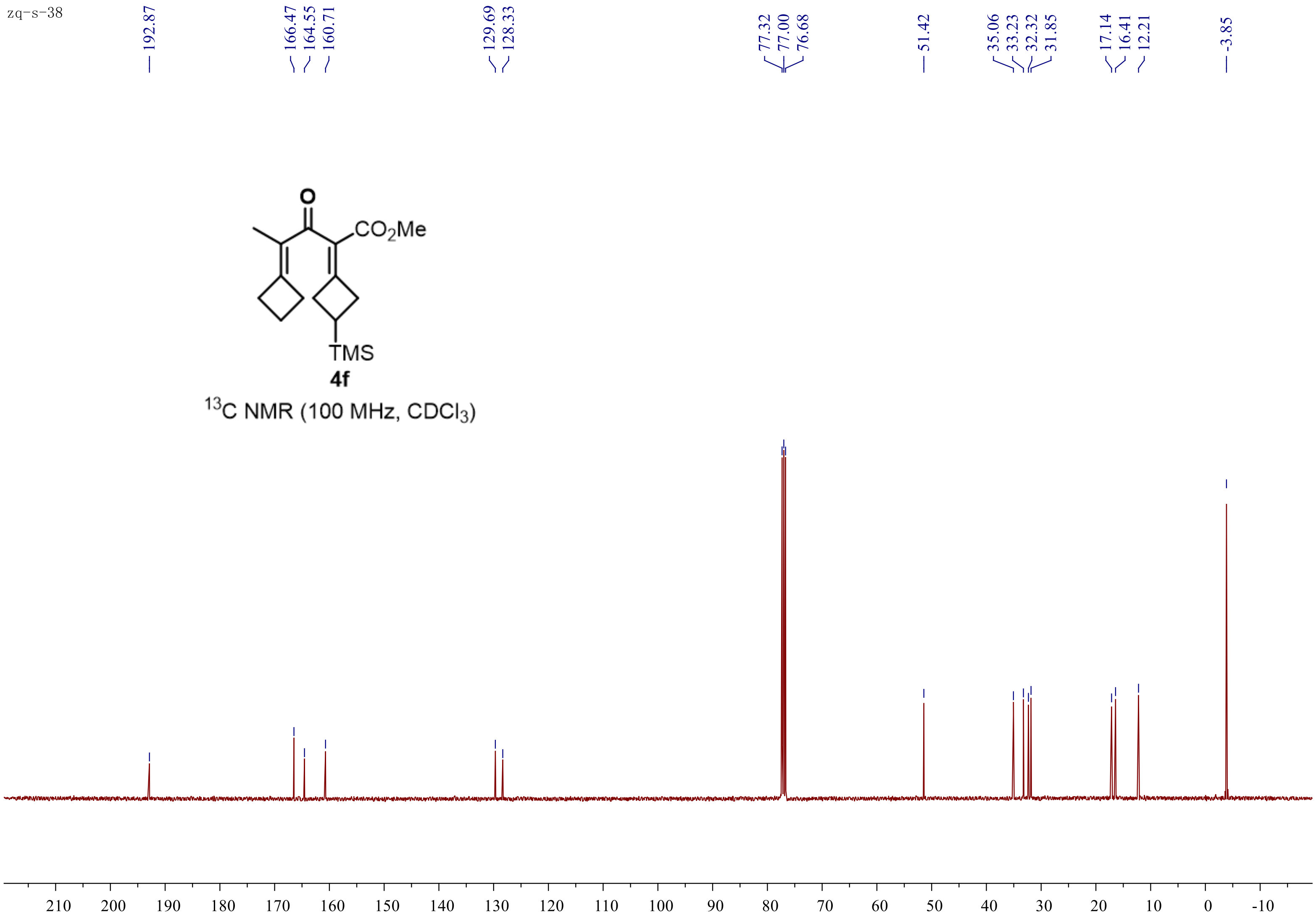

Supplementary Fig. 225. ^{13}C NMR spectra of compound **4f** in $CDCl_3$

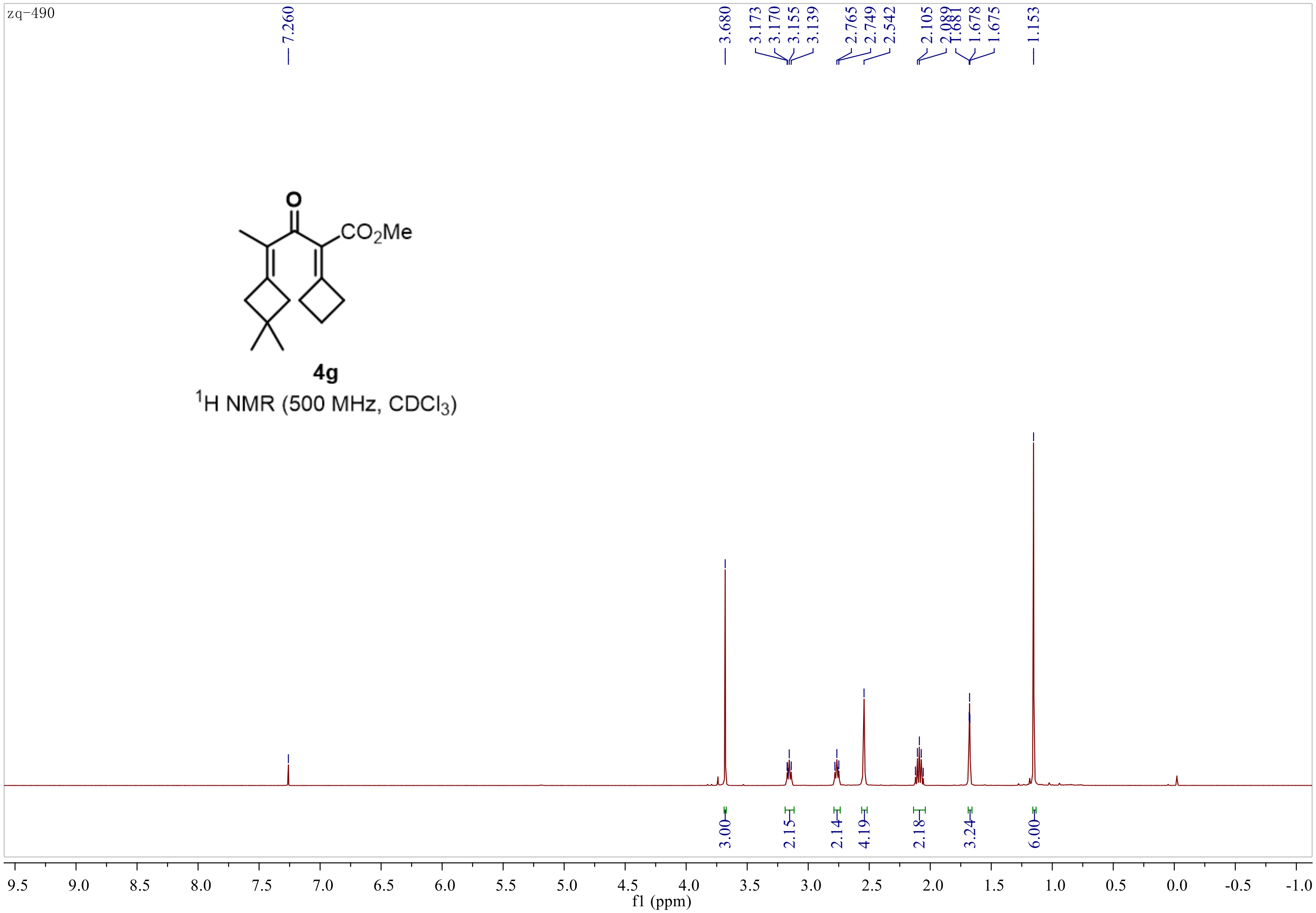

Supplementary Fig. 226. ^{1}H NMR spectra of compound **4g** in $CDCl_3$

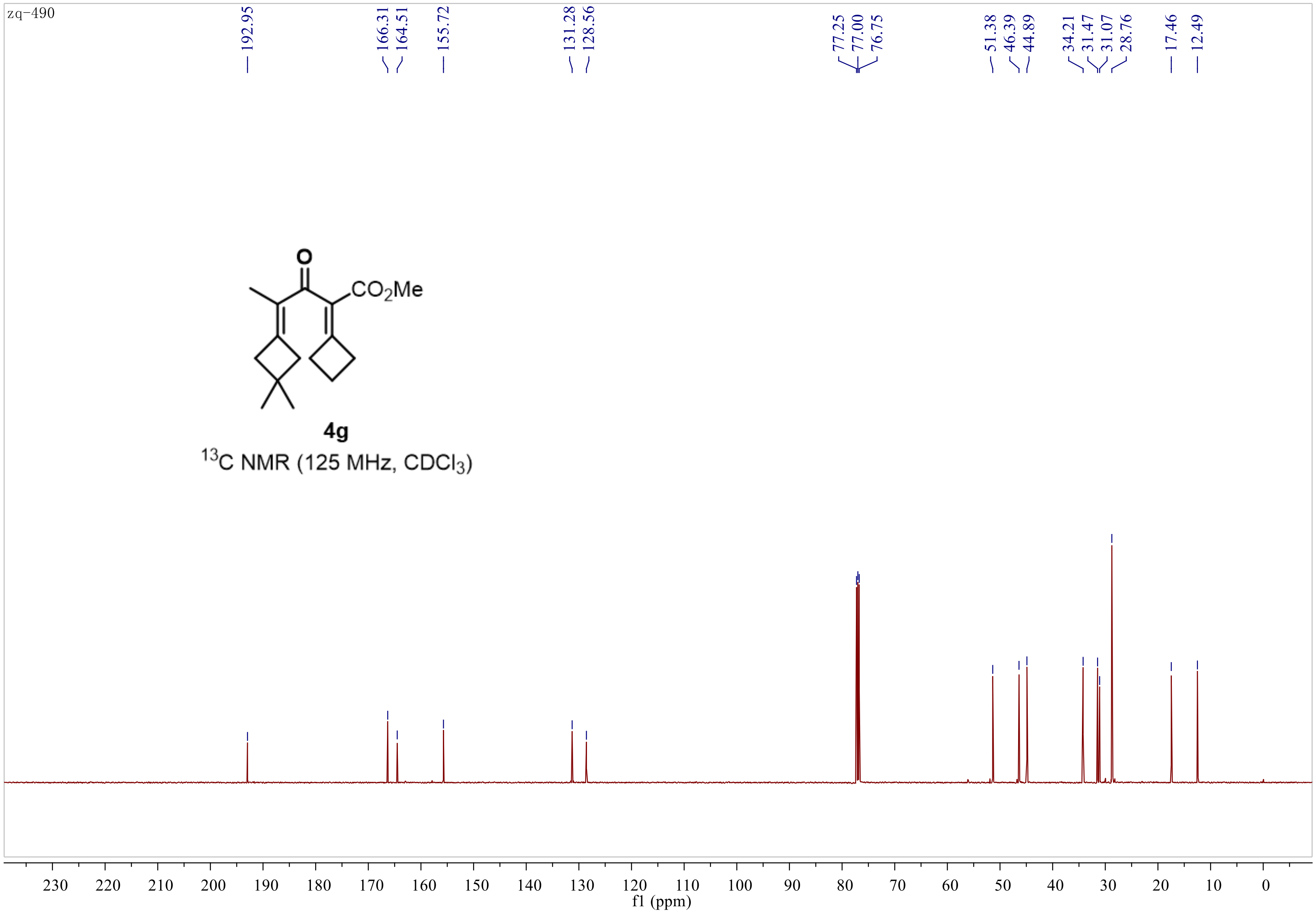

Supplementary Fig. 227. ^{13}C NMR spectra of compound **4g** in $CDCl_3$

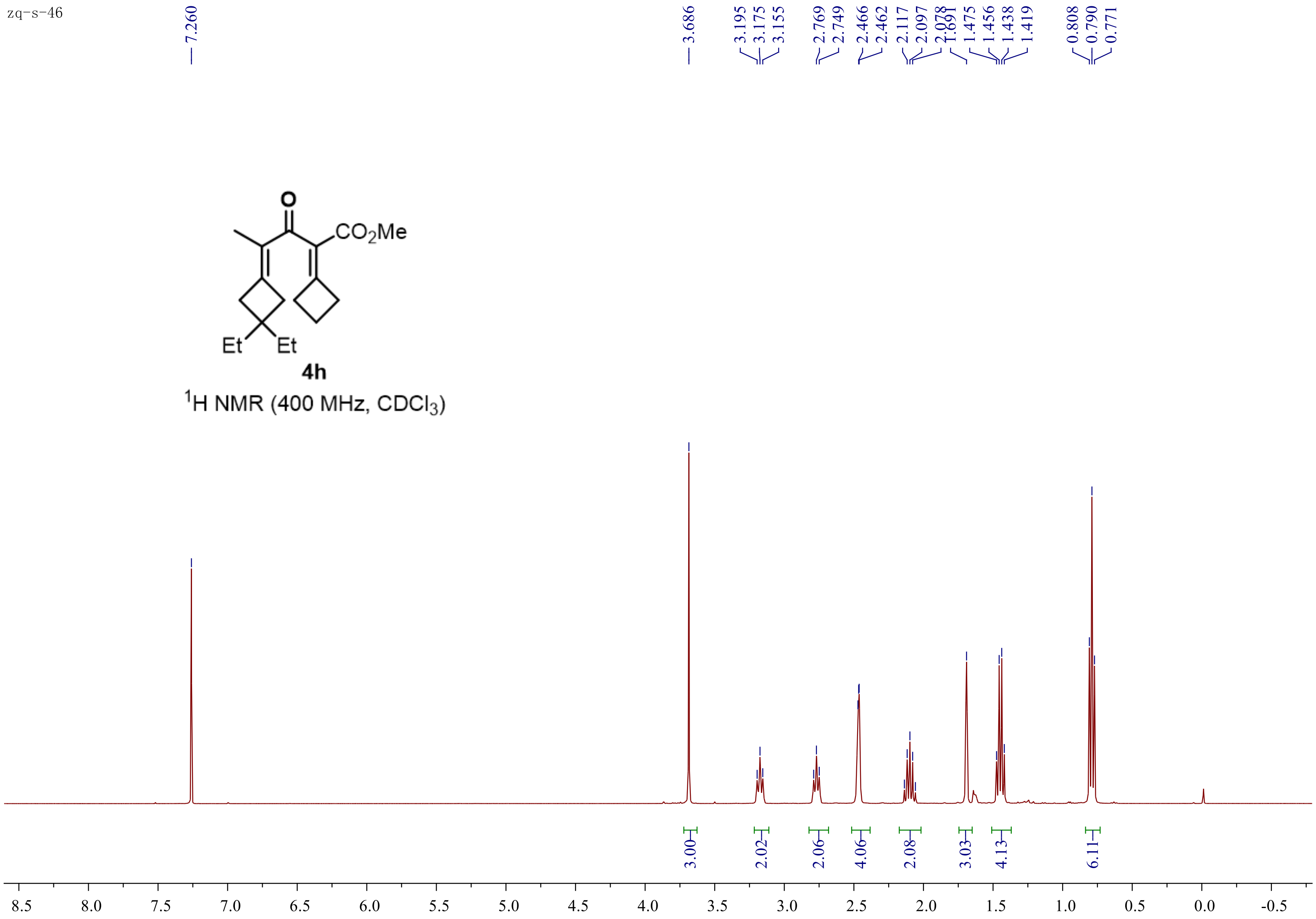

Supplementary Fig. 228. ^{1}H NMR spectra of compound **4h** in $CDCl_3$

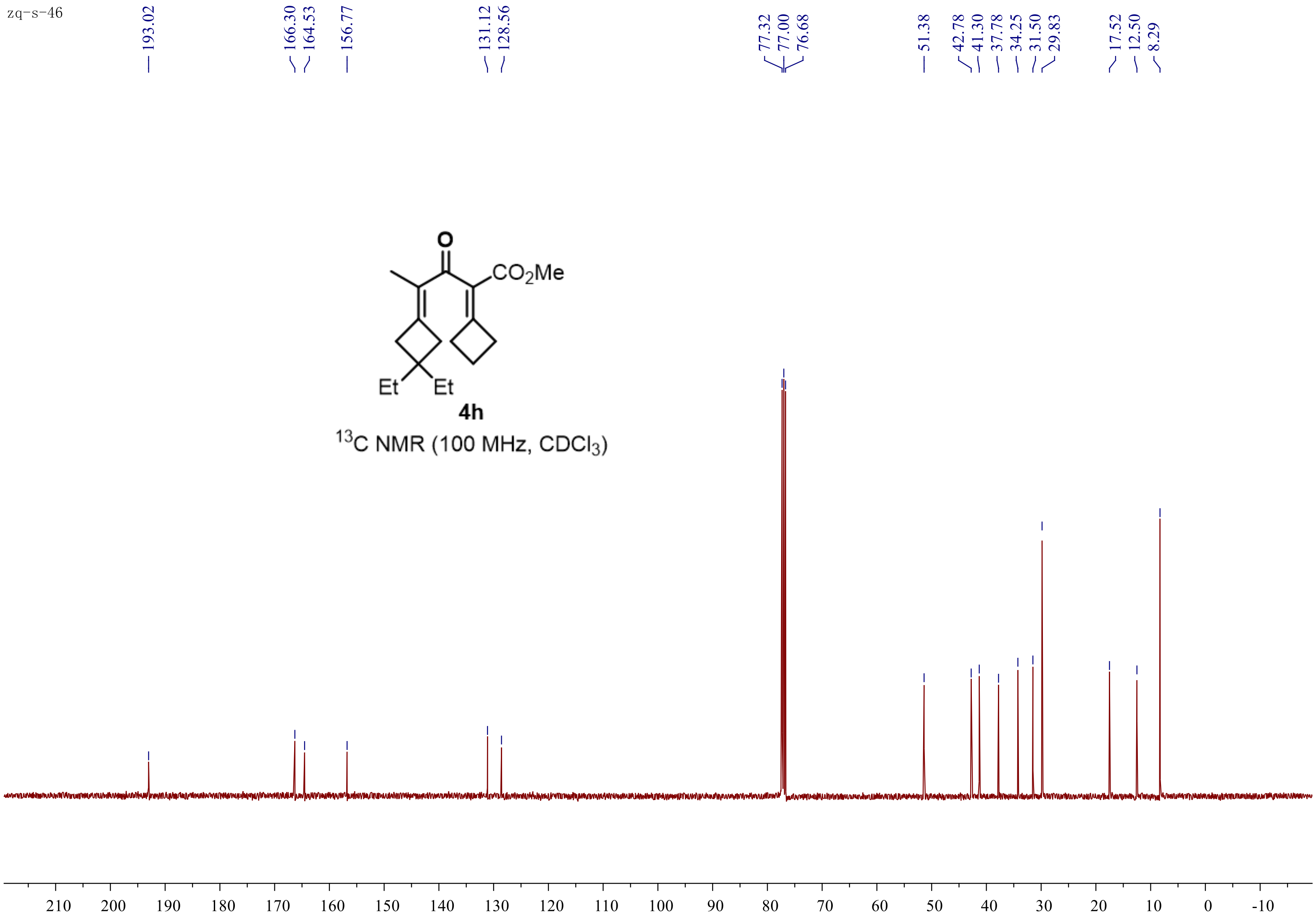

Supplementary Fig. 229. ^{13}C NMR spectra of compound **4h** in $CDCl_3$

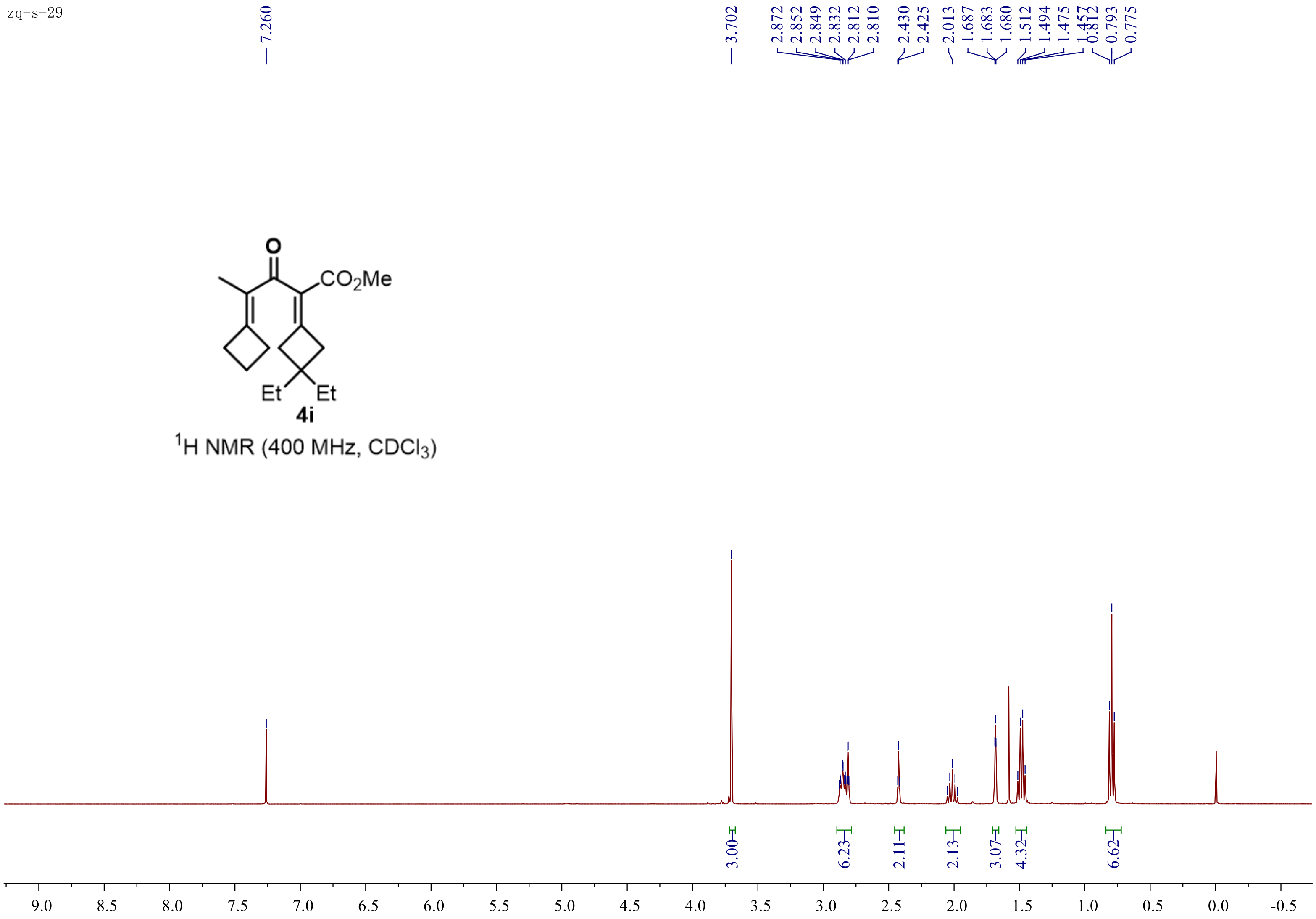

Supplementary Fig. 230. ^{1}H NMR spectra of compound **4i** in $CDCl_3$

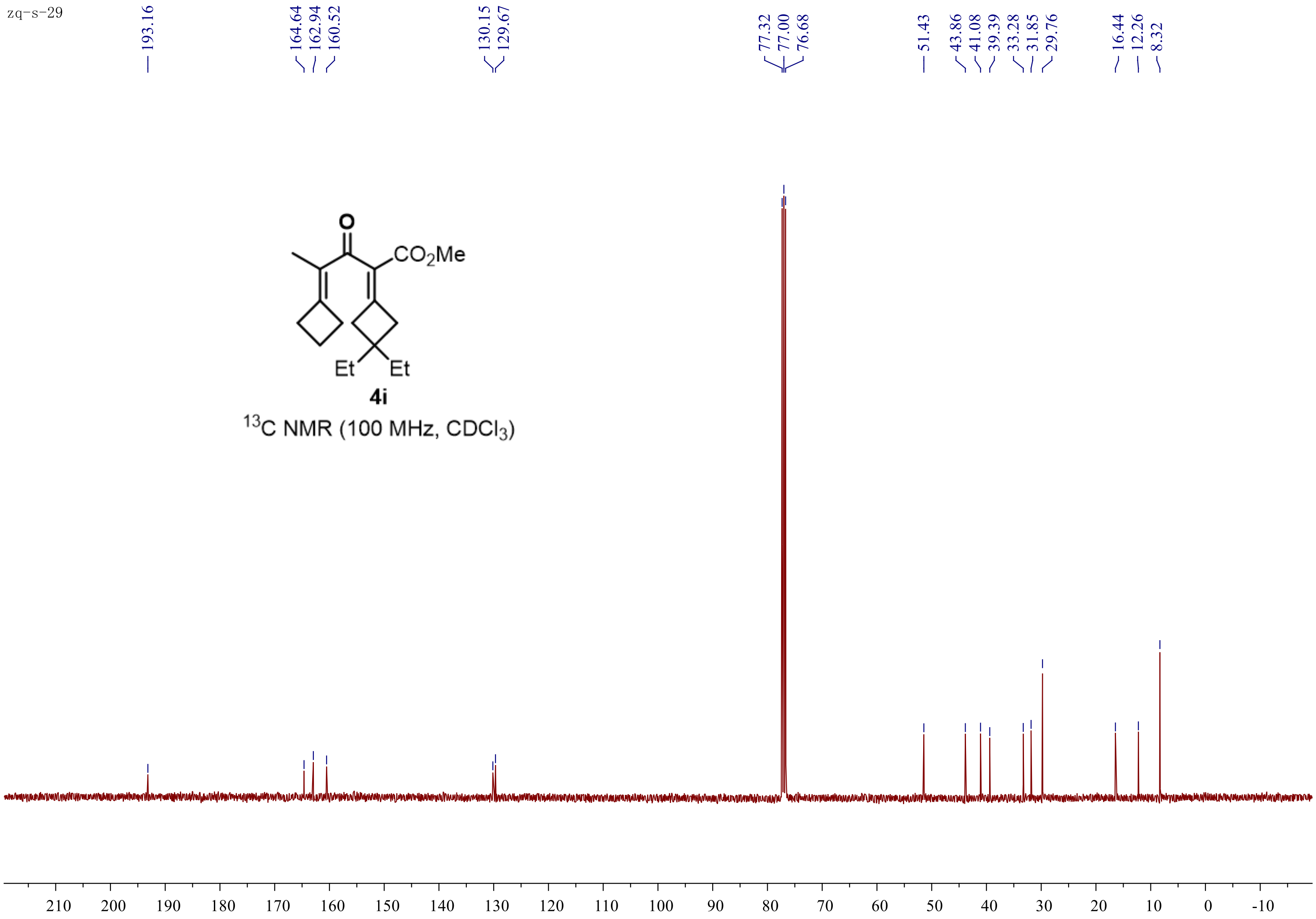

4i

^{13}C NMR (100 MHz, $CDCl_3$)

Supplementary Fig. 231. ^{13}C NMR spectra of compound **4i** in $CDCl_3$

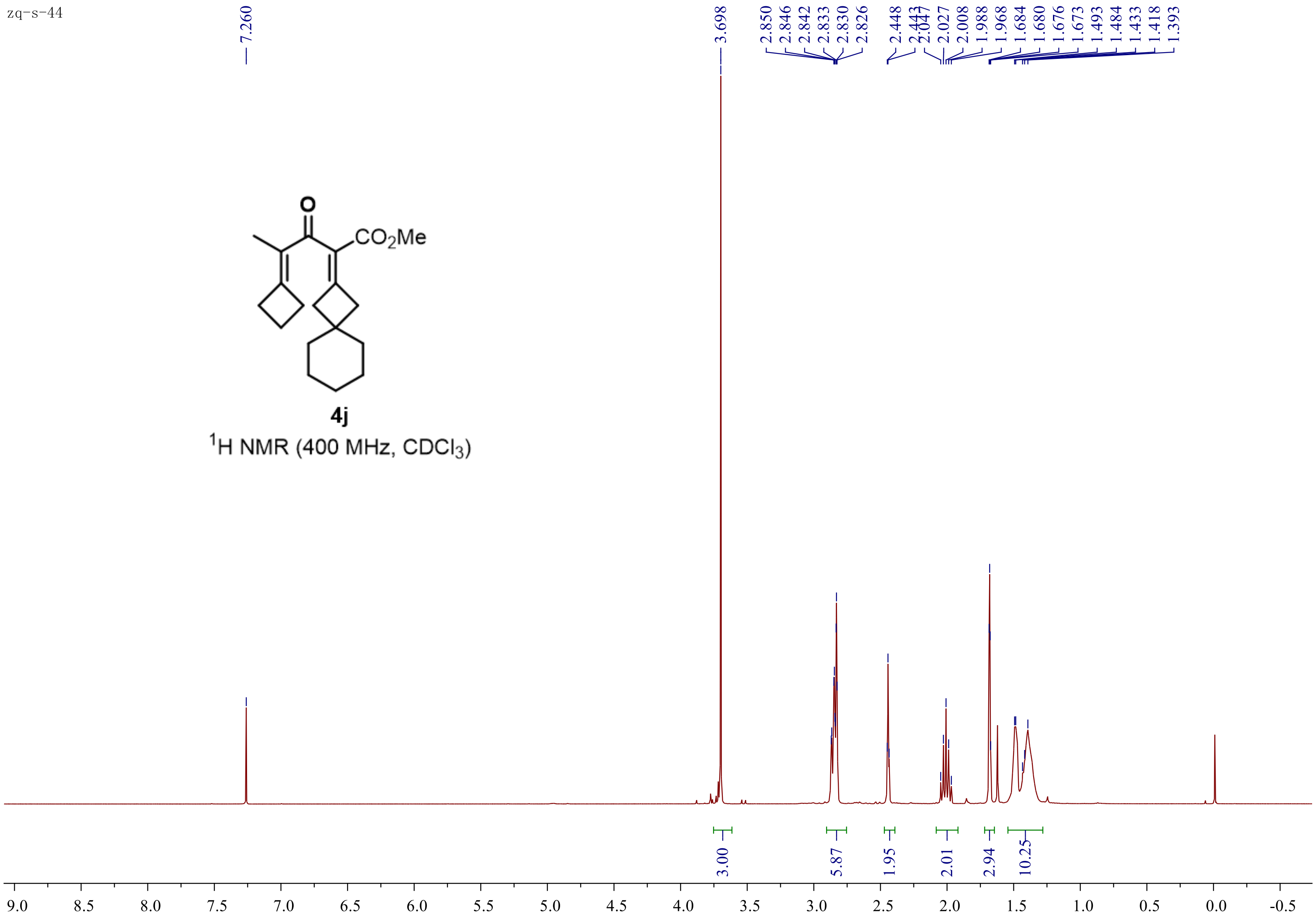

Supplementary Fig. 232. ^{1}H NMR spectra of compound **4j** in $CDCl_3$

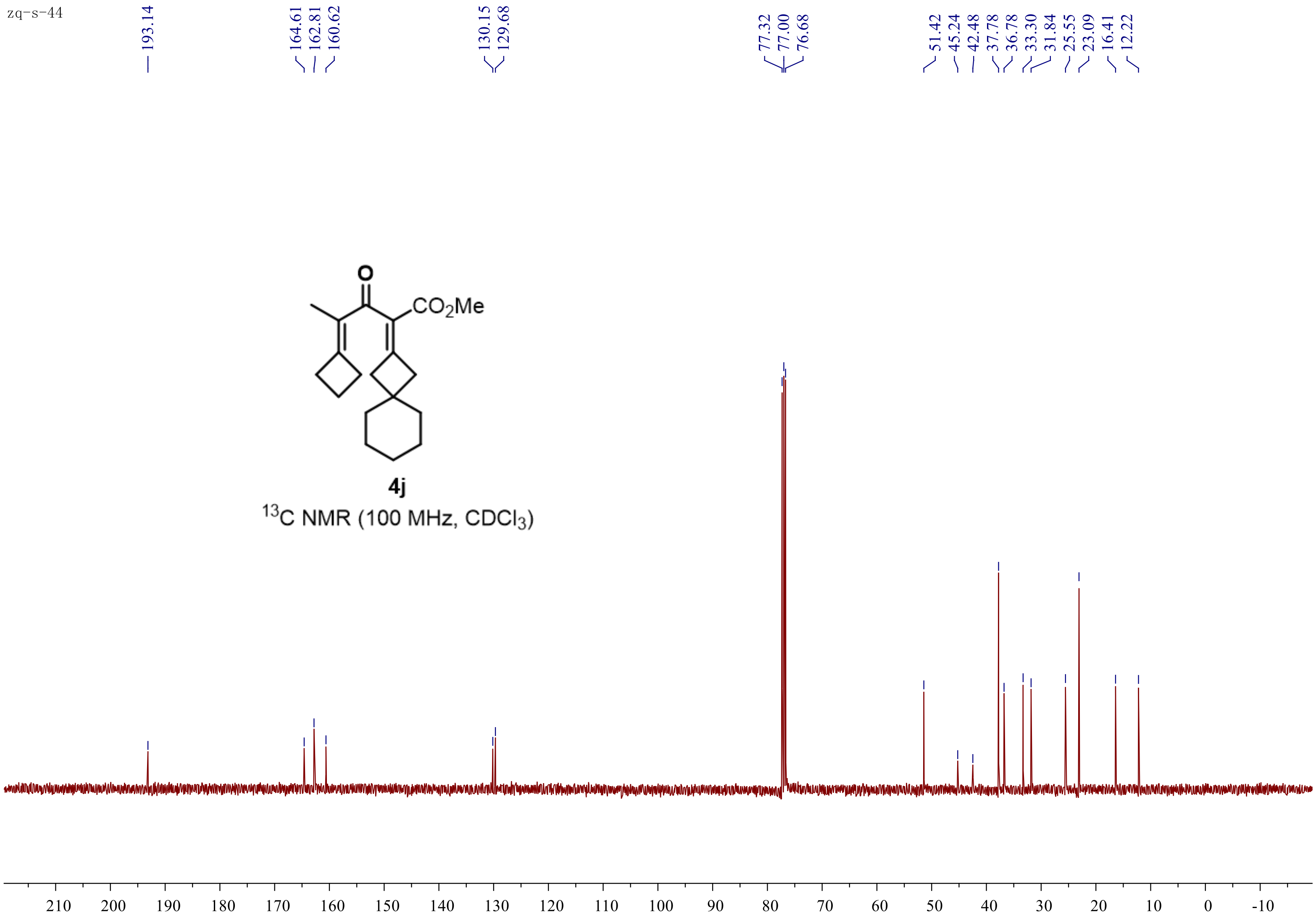

Supplementary Fig. 233. ^{13}C NMR spectra of compound **4j** in $CDCl_3$

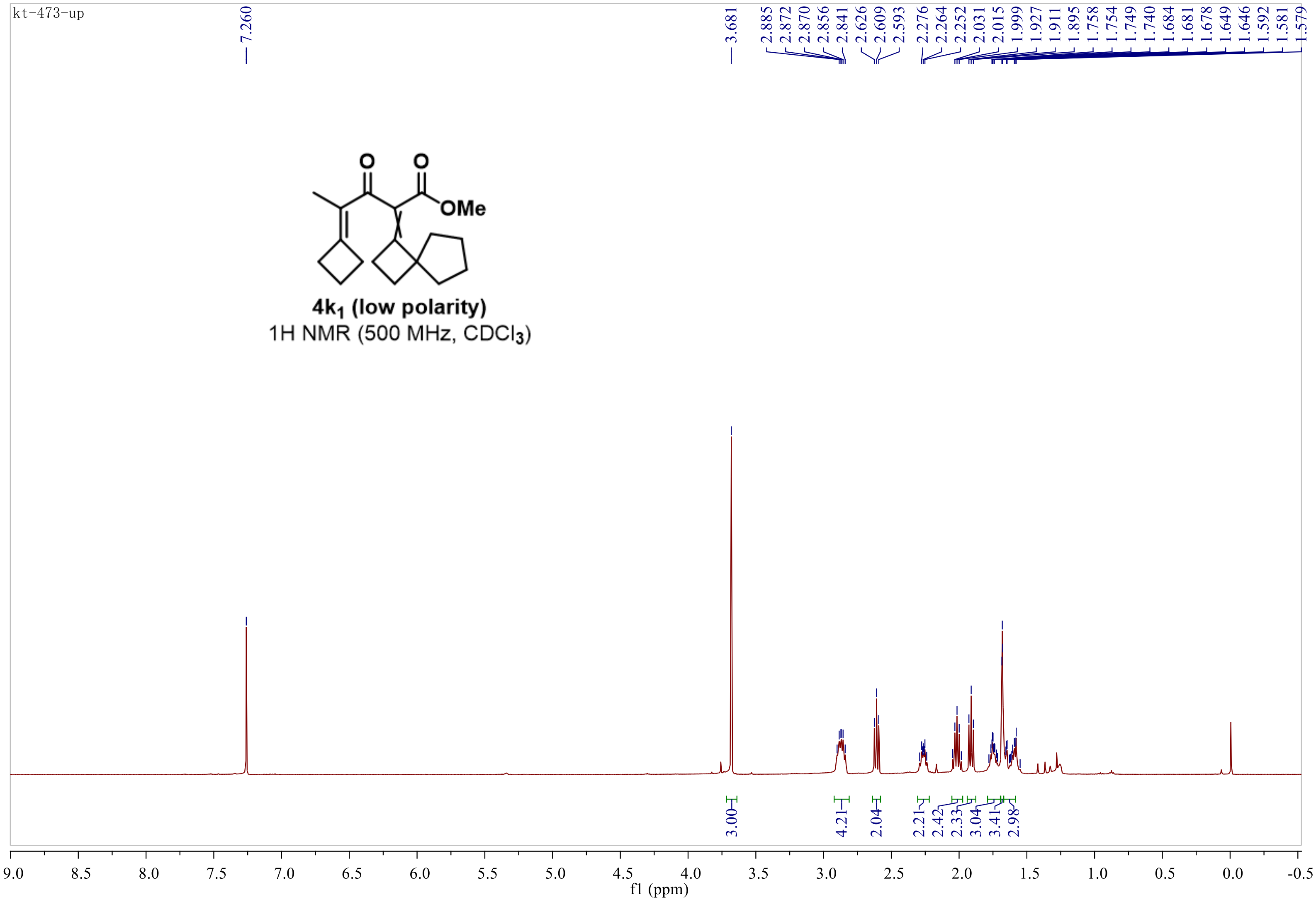

Supplementary Fig. 234. ^{1}H NMR spectra of compound **4k$_1$** in $CDCl_3$

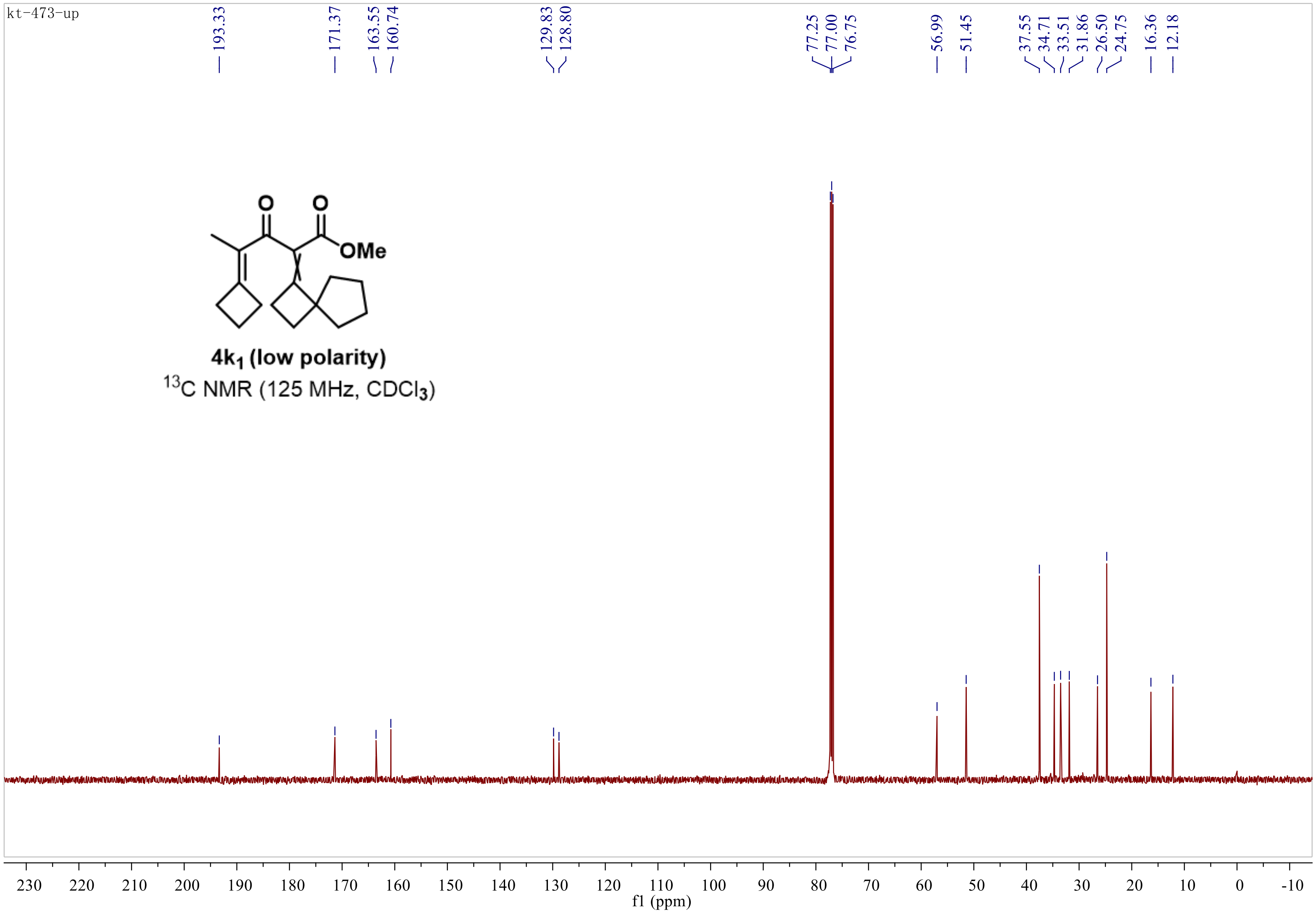

Supplementary Fig. 235. ^{13}C NMR spectra of compound **4k₁** in $CDCl_3$

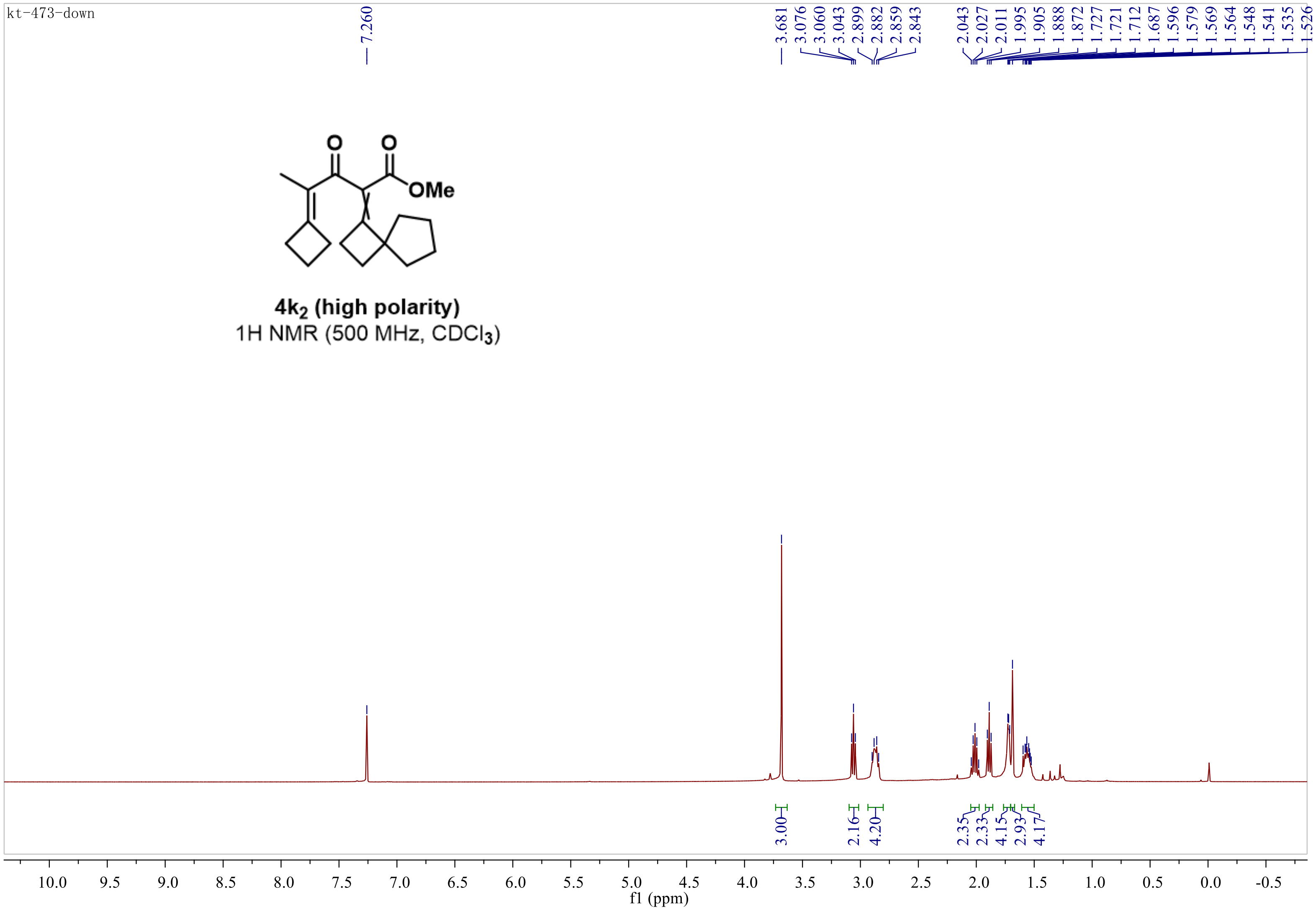

Supplementary Fig. 236. ^{1}H NMR spectra of compound **4k$_2$** in $CDCl_3$

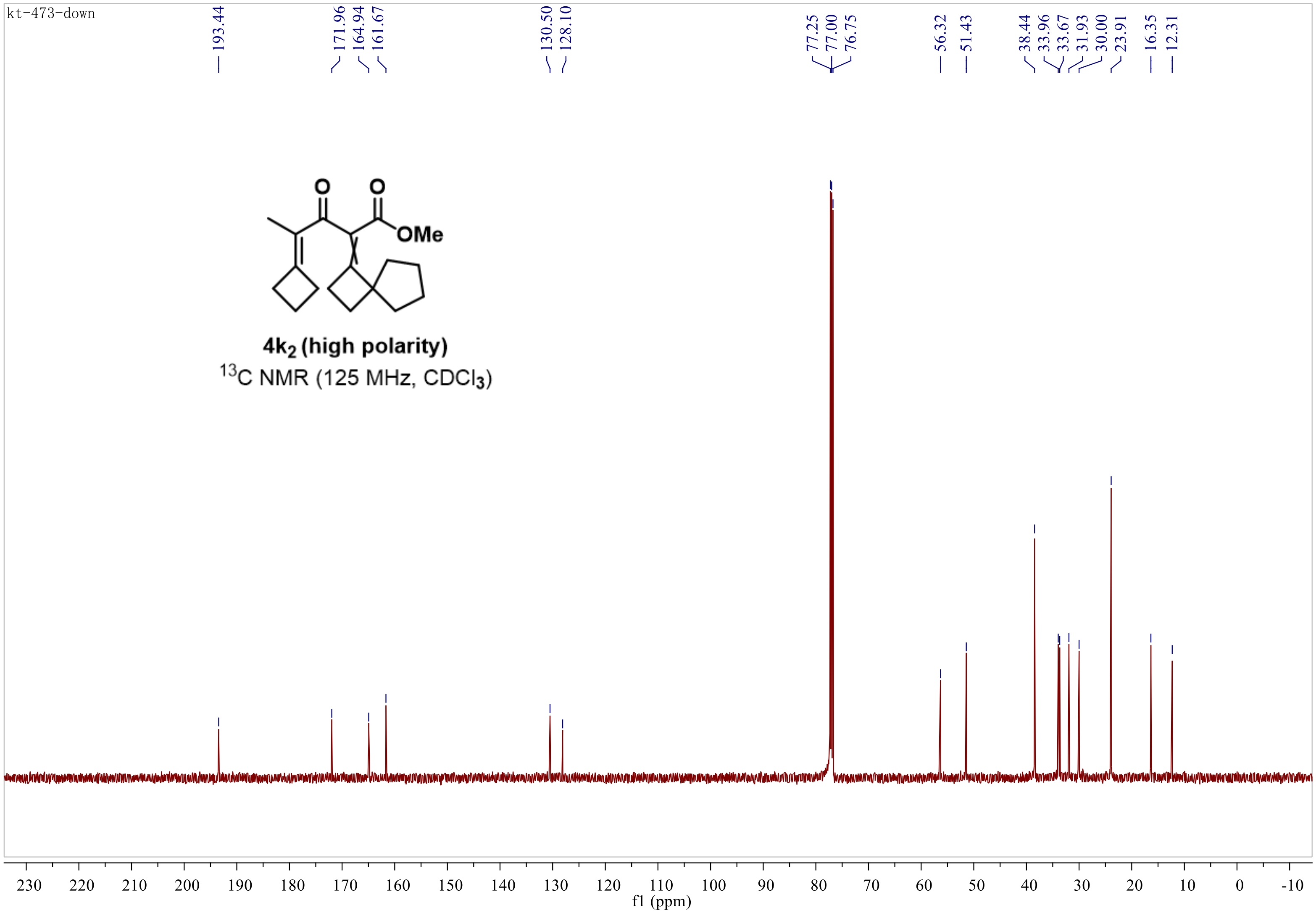

Supplementary Fig. 237. ^{13}C NMR spectra of compound **$4k_2$** in $CDCl_3$

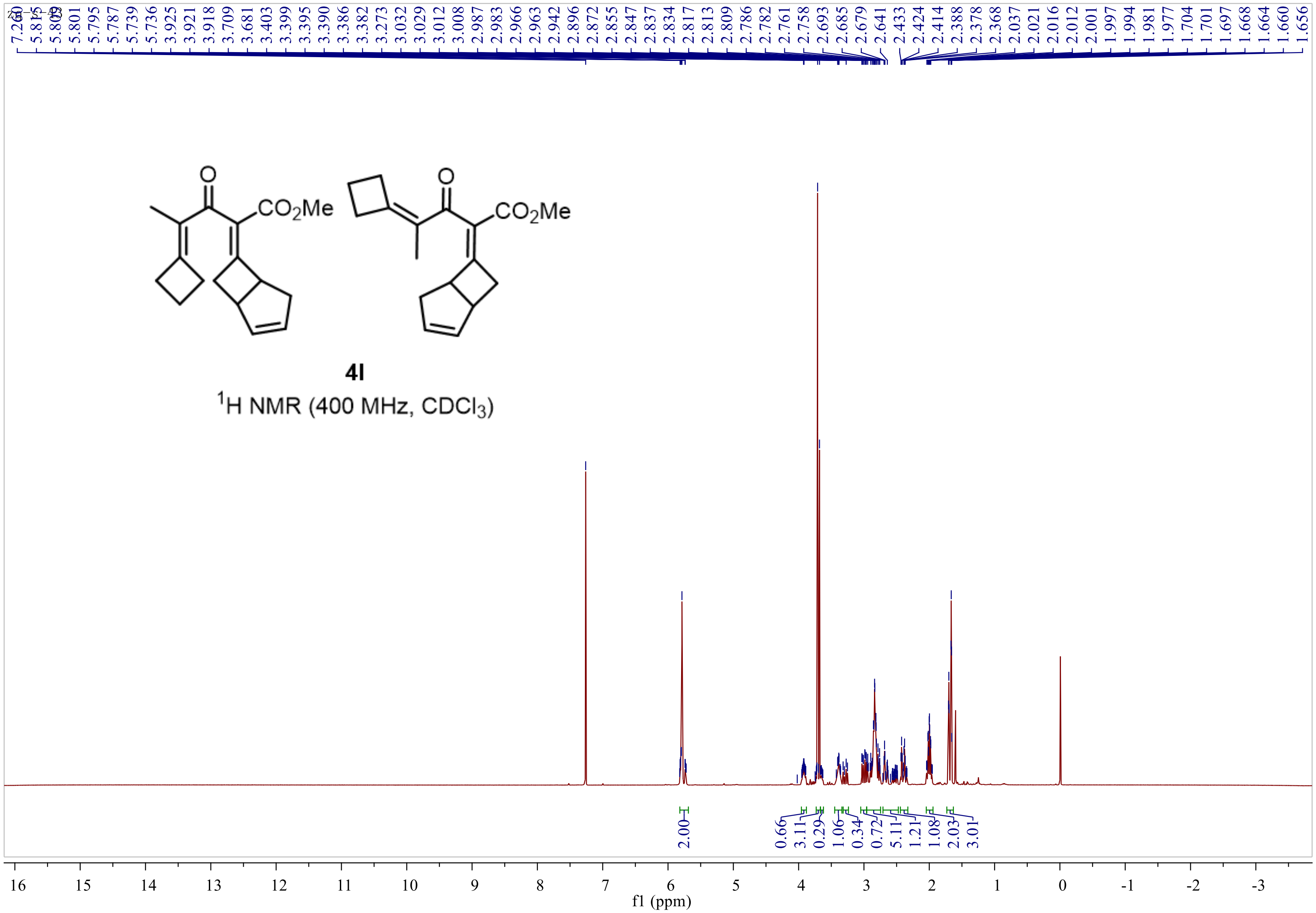

Supplementary Fig. 238. ^{1}H NMR spectra of compound **4l** in $CDCl_3$

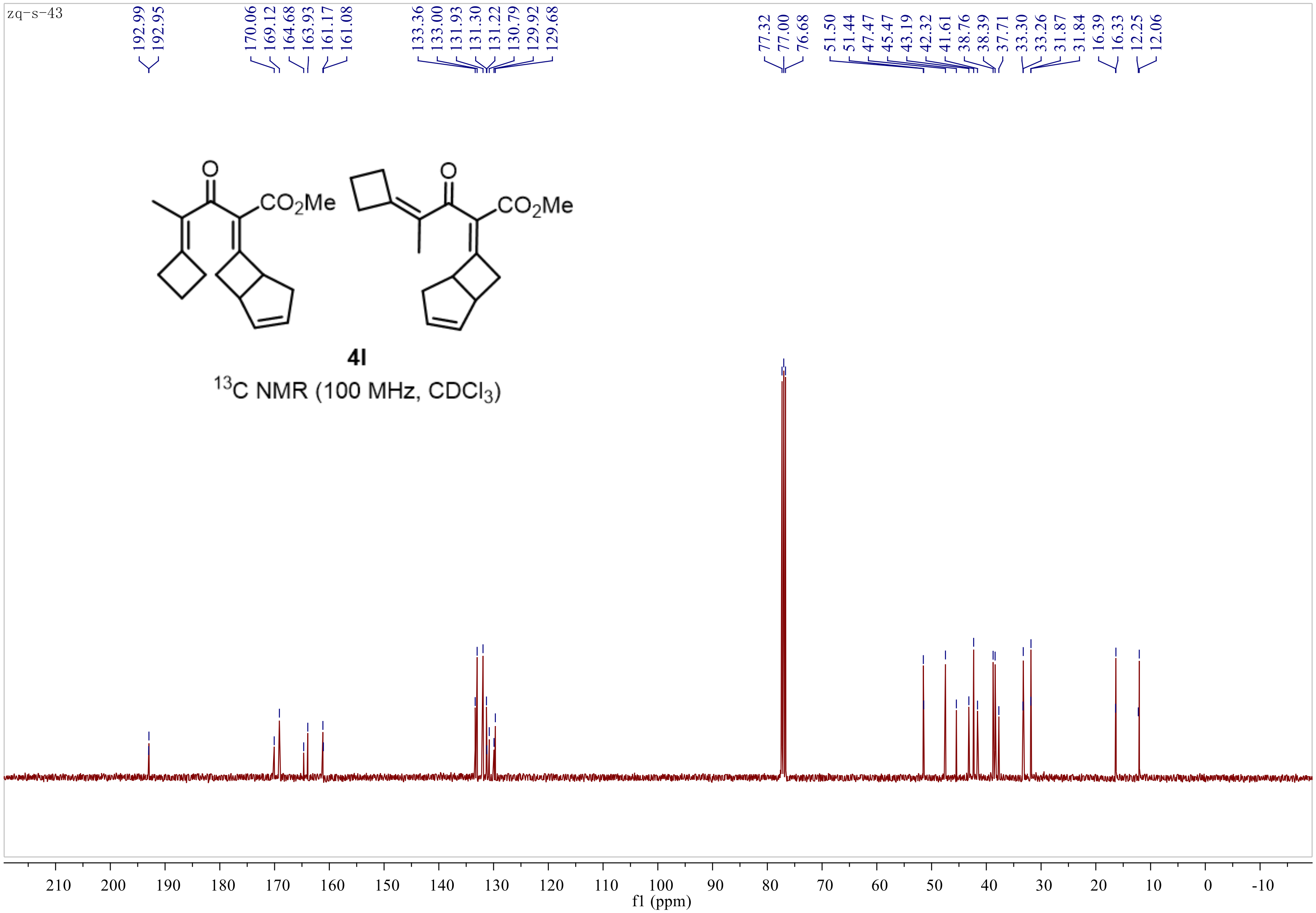

Supplementary Fig. 239. ^{13}C NMR spectra of compound **4l** in $CDCl_3$

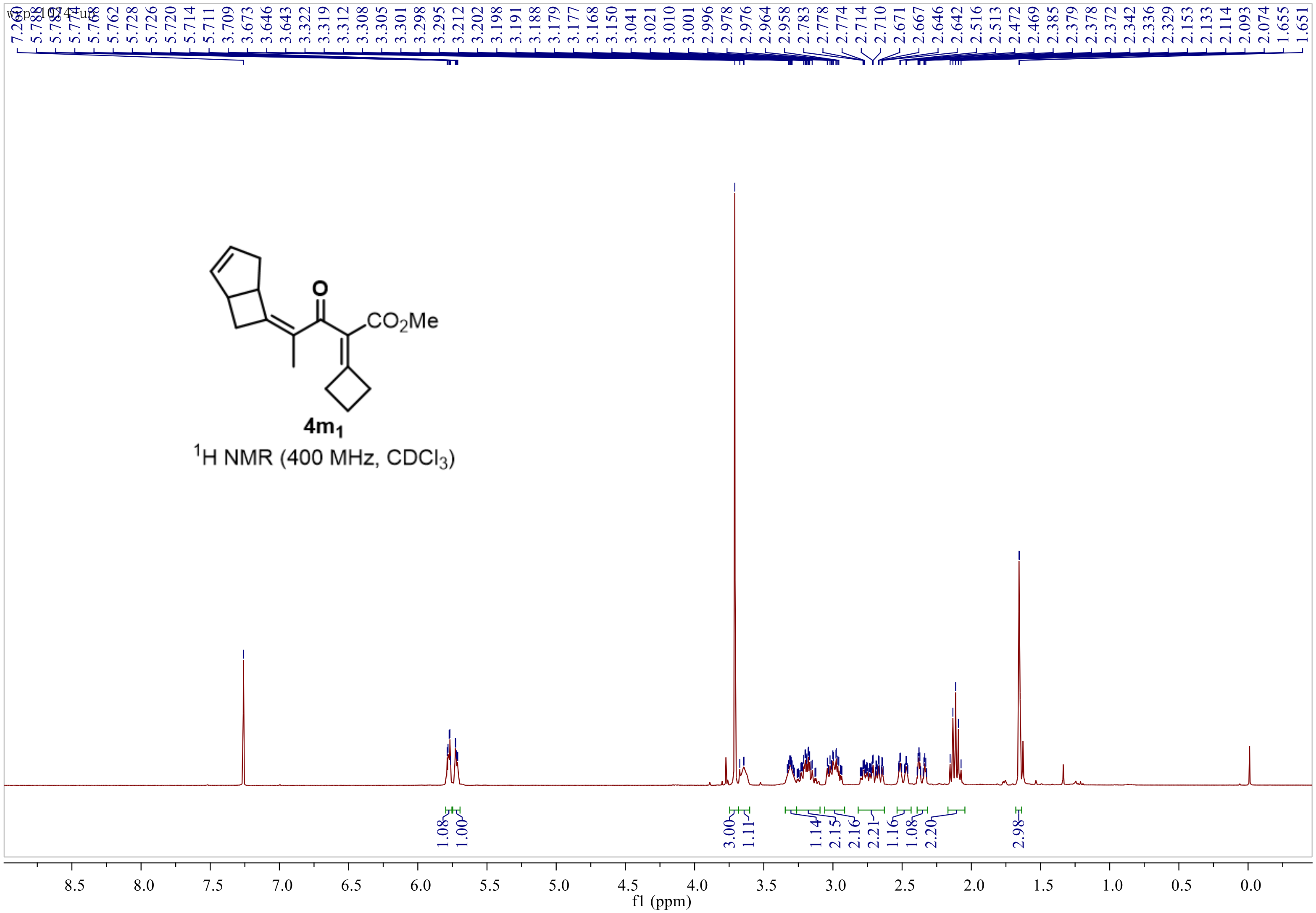

Supplementary Fig. 240. ^{1}H NMR spectra of compound **4m₁** in $CDCl_3$

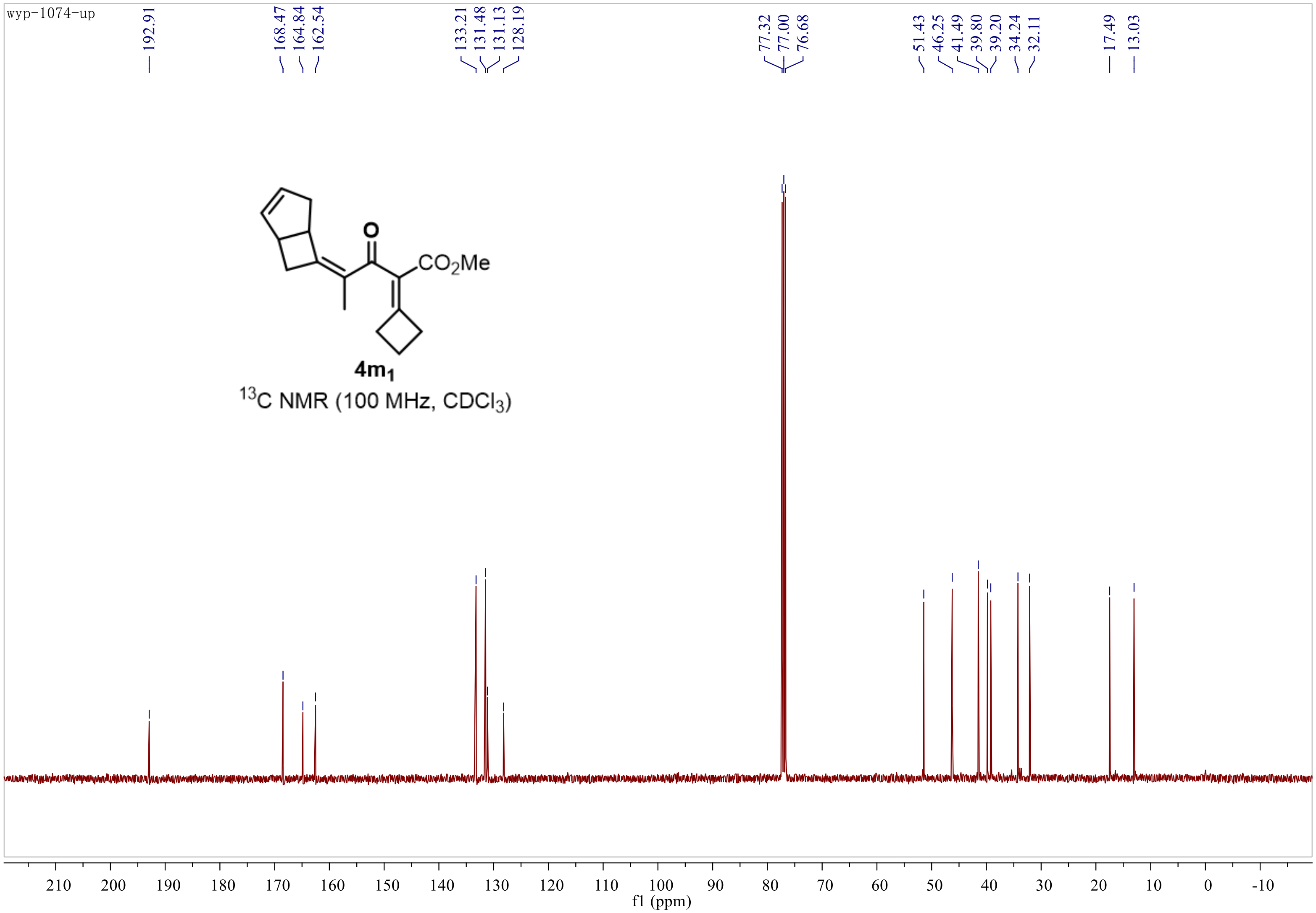

Supplementary Fig. 241. ^{13}C NMR spectra of compound **$4m_1$** in $CDCl_3$

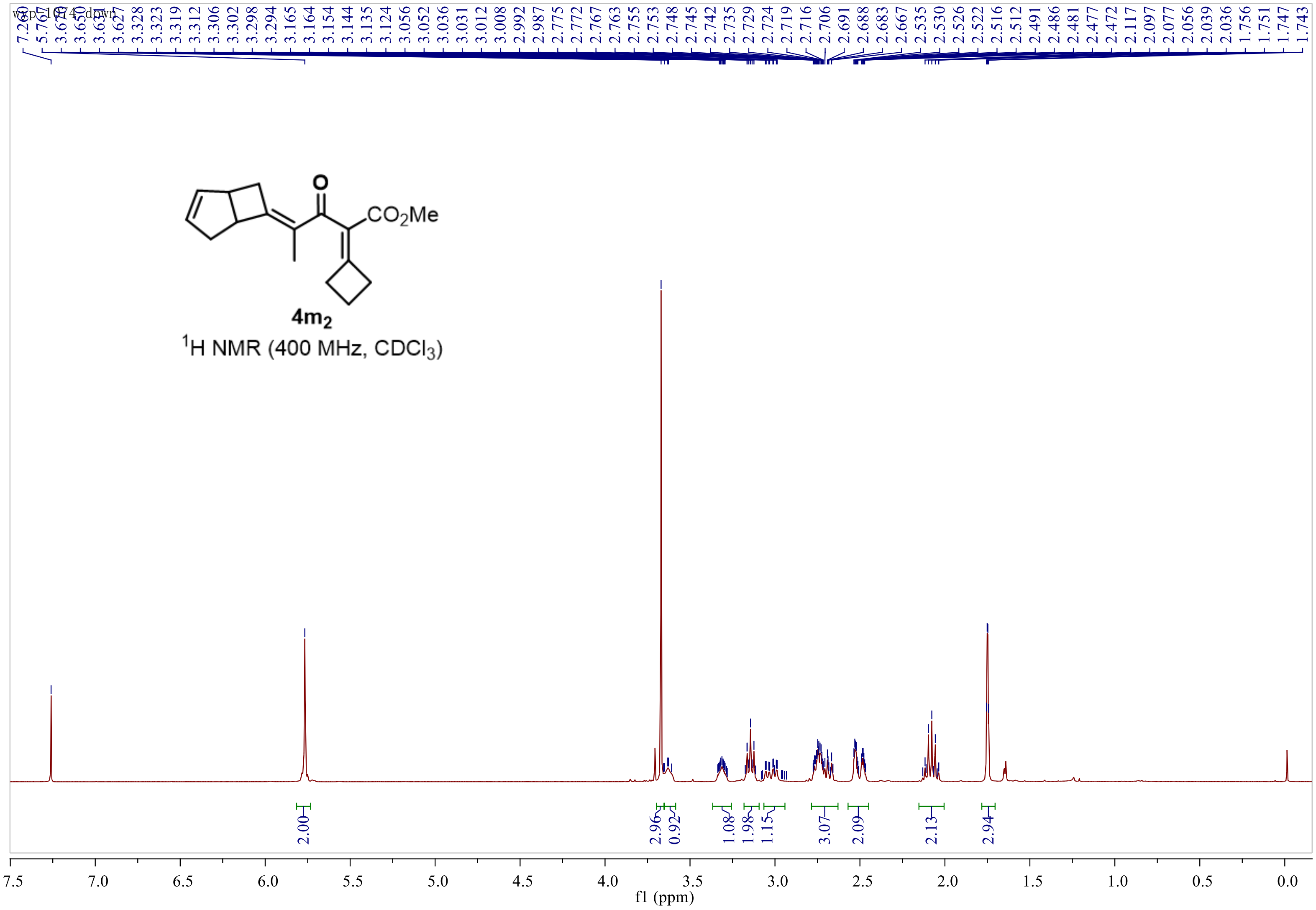

Supplementary Fig. 242. ^{1}H NMR spectra of compound **4m$_2$** in $CDCl_3$

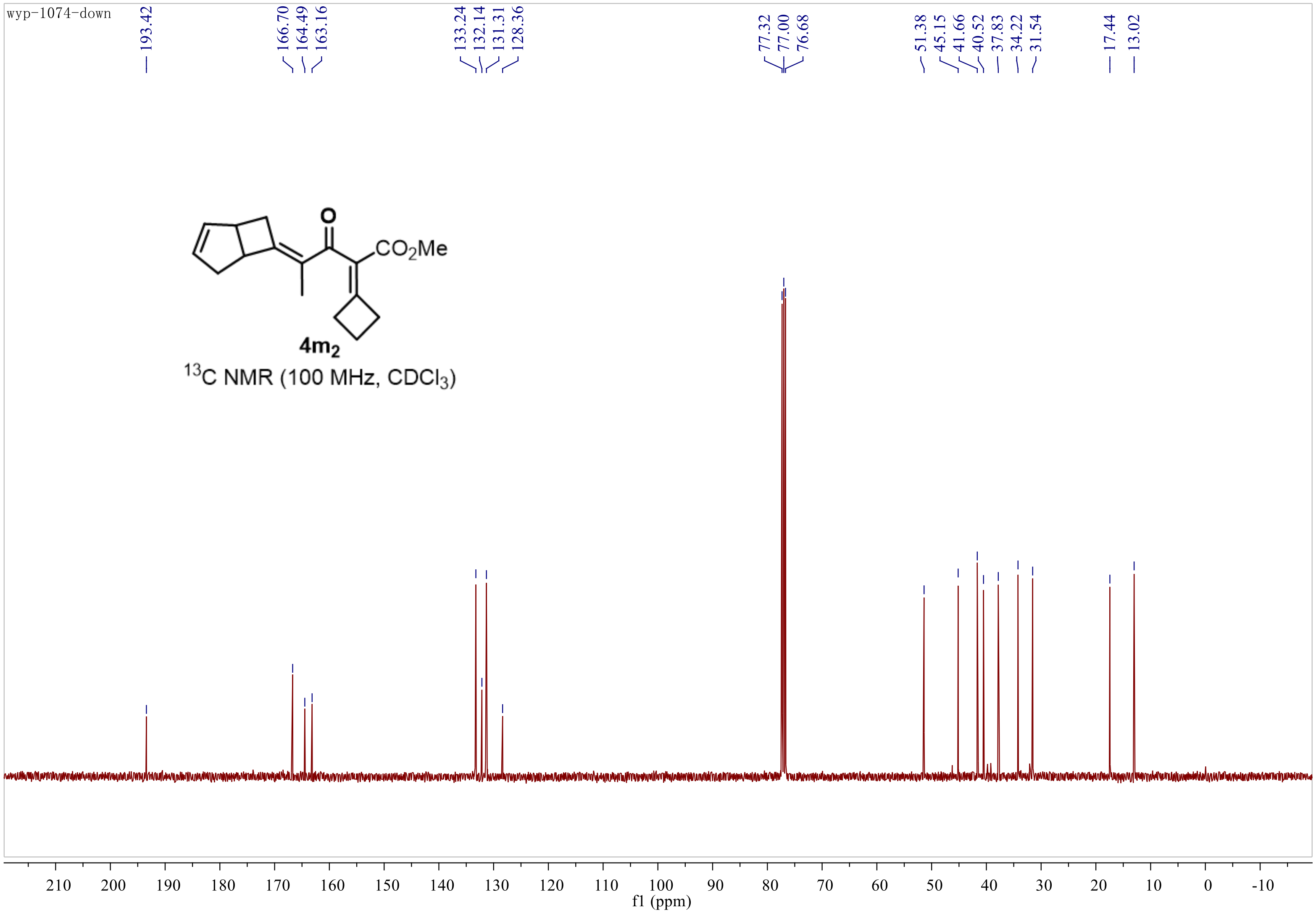

Supplementary Fig. 243. ^{13}C NMR spectra of compound **$4m_2$** in $CDCl_3$

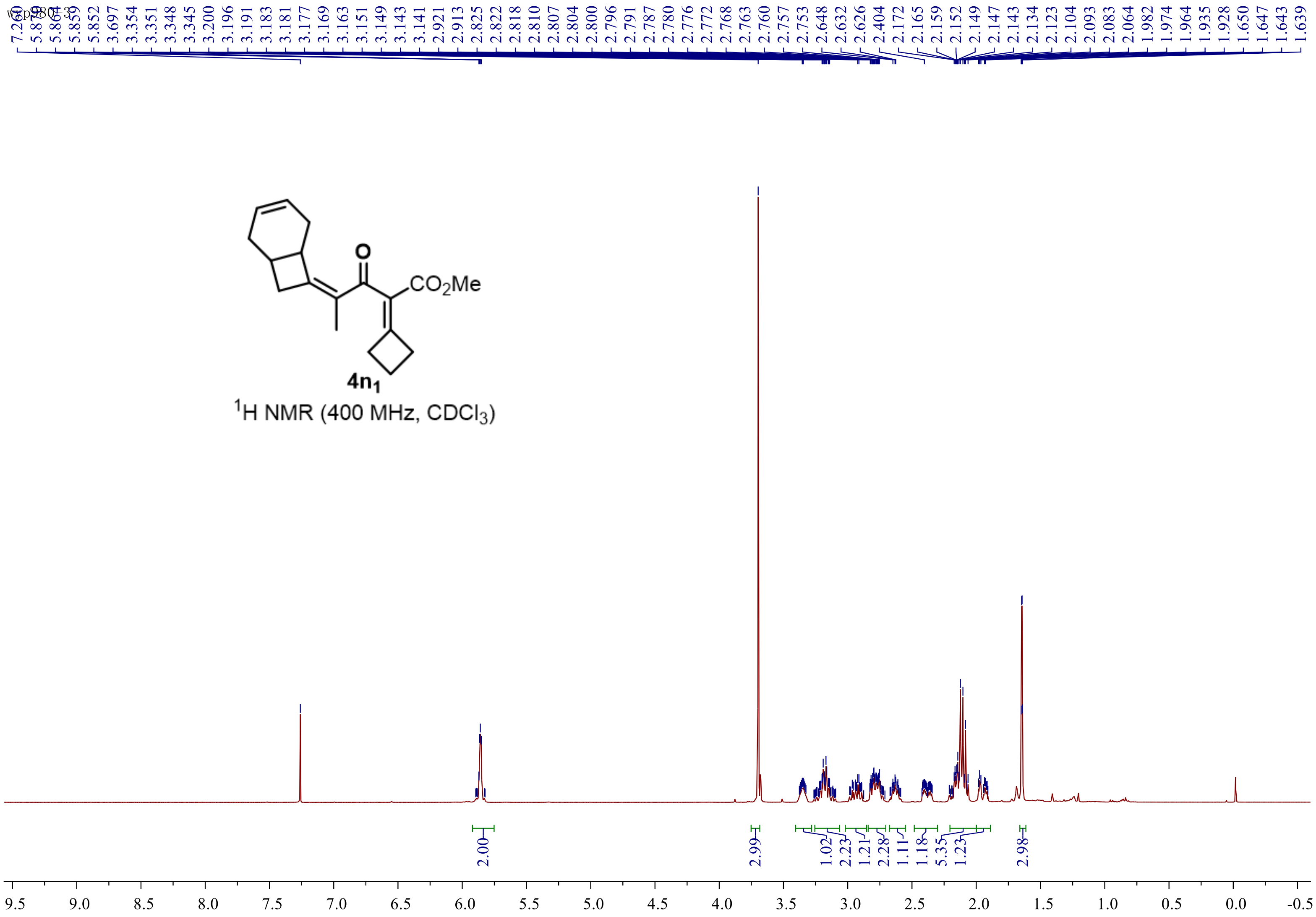

Supplementary Fig. 244. ^{1}H NMR spectra of compound **4n$_1$** in $CDCl_3$

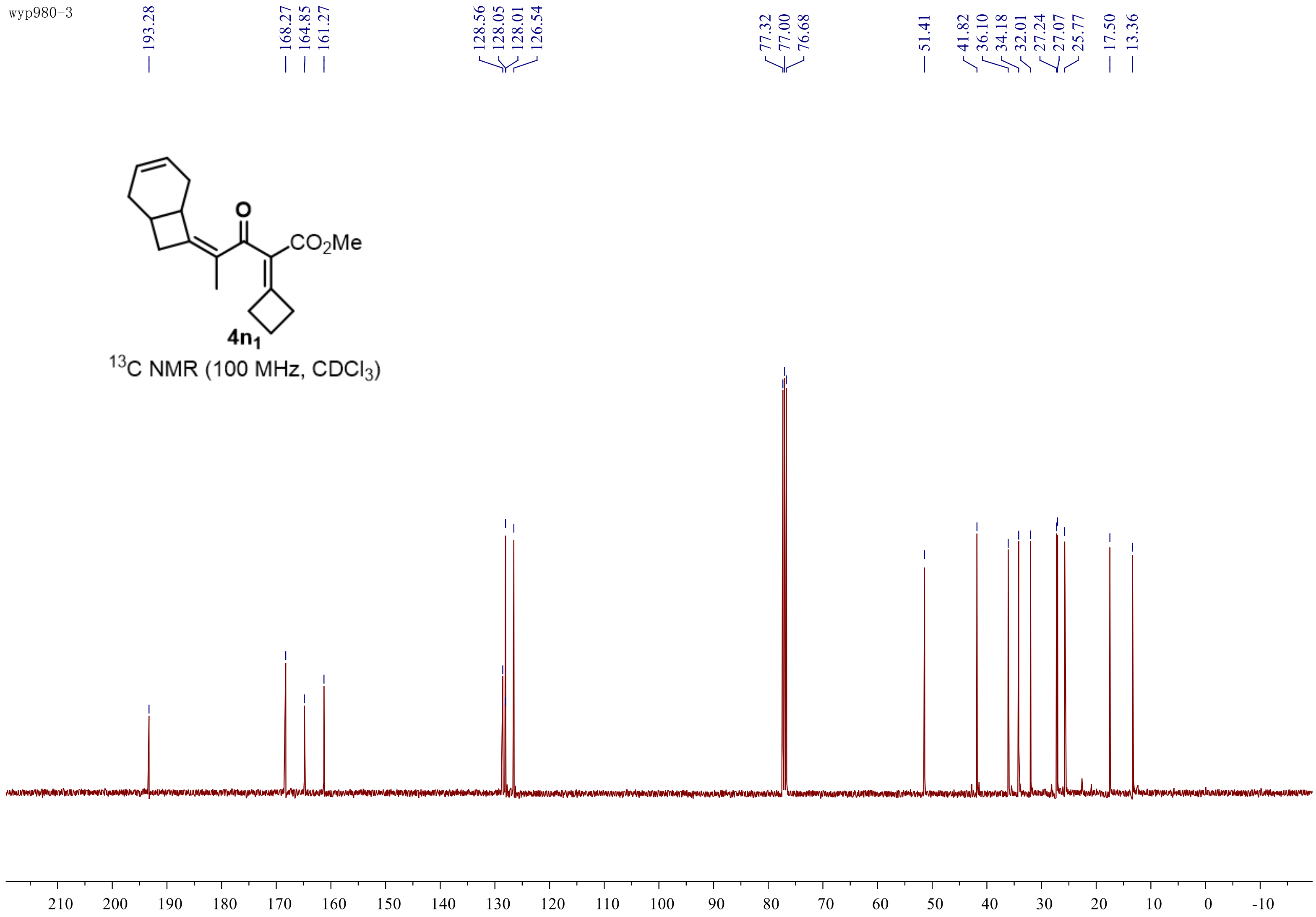

Supplementary Fig. 245. ^{13}C NMR spectra of compound **4n₁** in $CDCl_3$

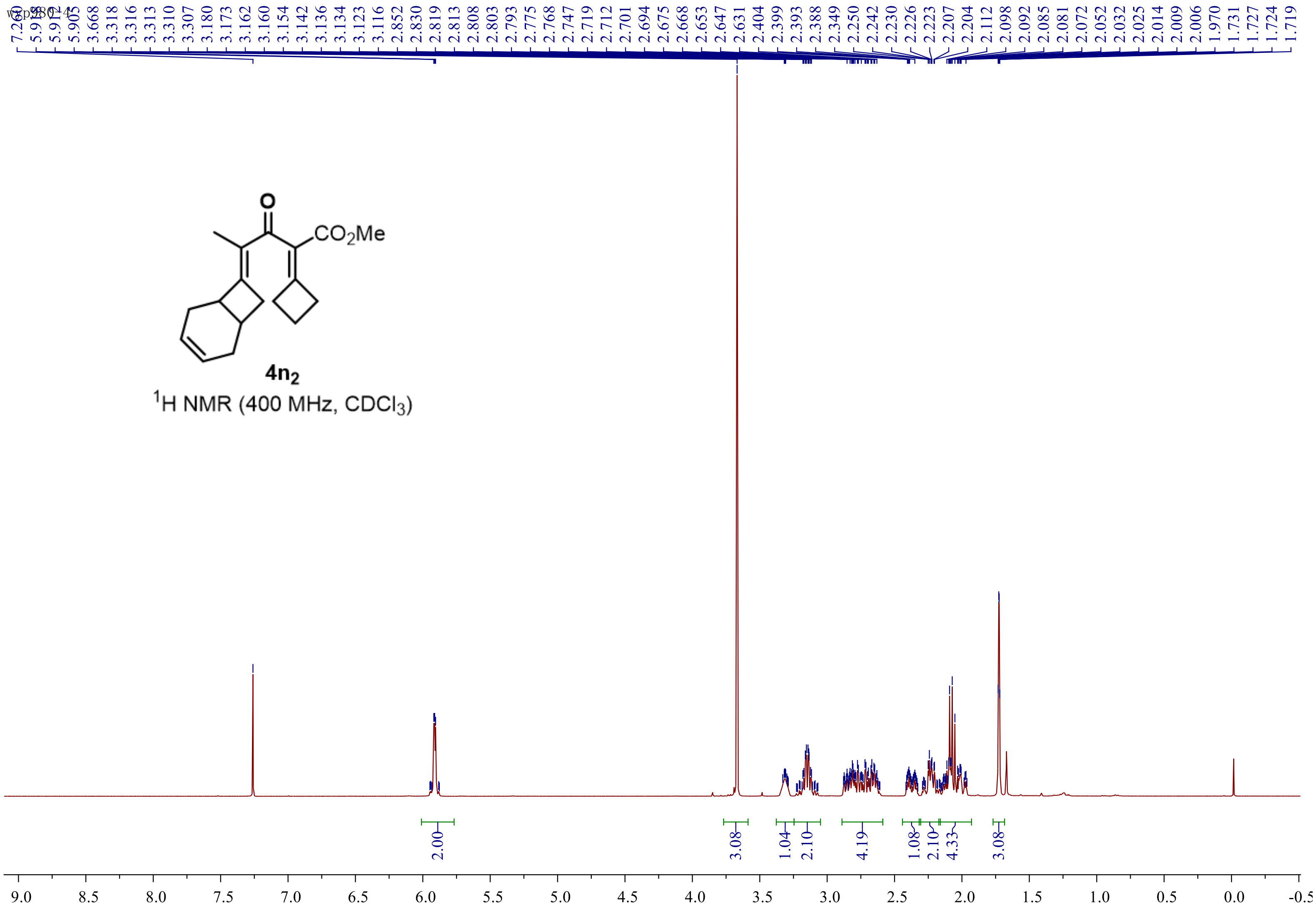

Supplementary Fig. 246. ^{1}H NMR spectra of compound **4n$_2$** in $CDCl_3$

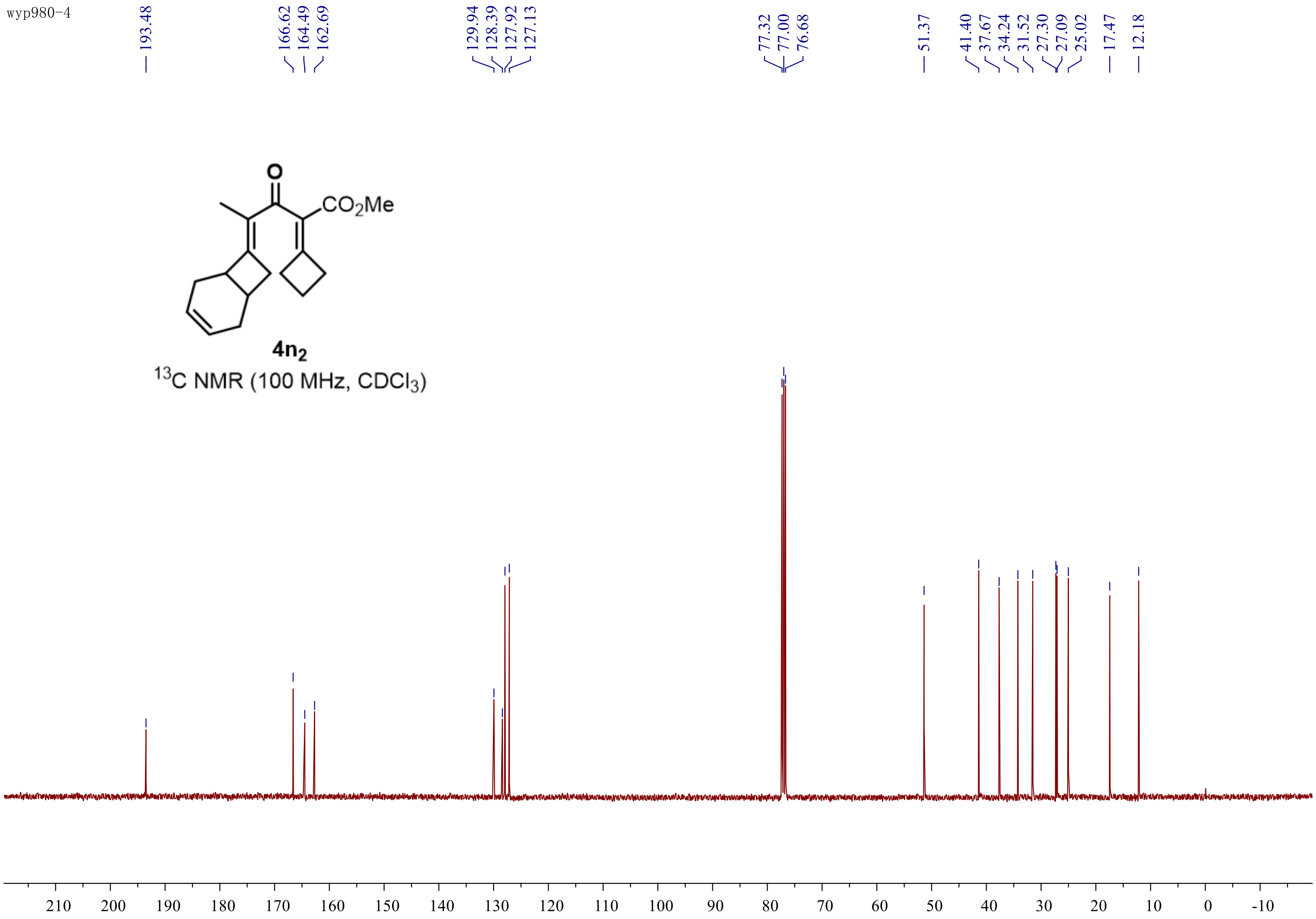

Supplementary Fig. 247. ^{13}C NMR spectra of compound **4n$_2$** in $CDCl_3$

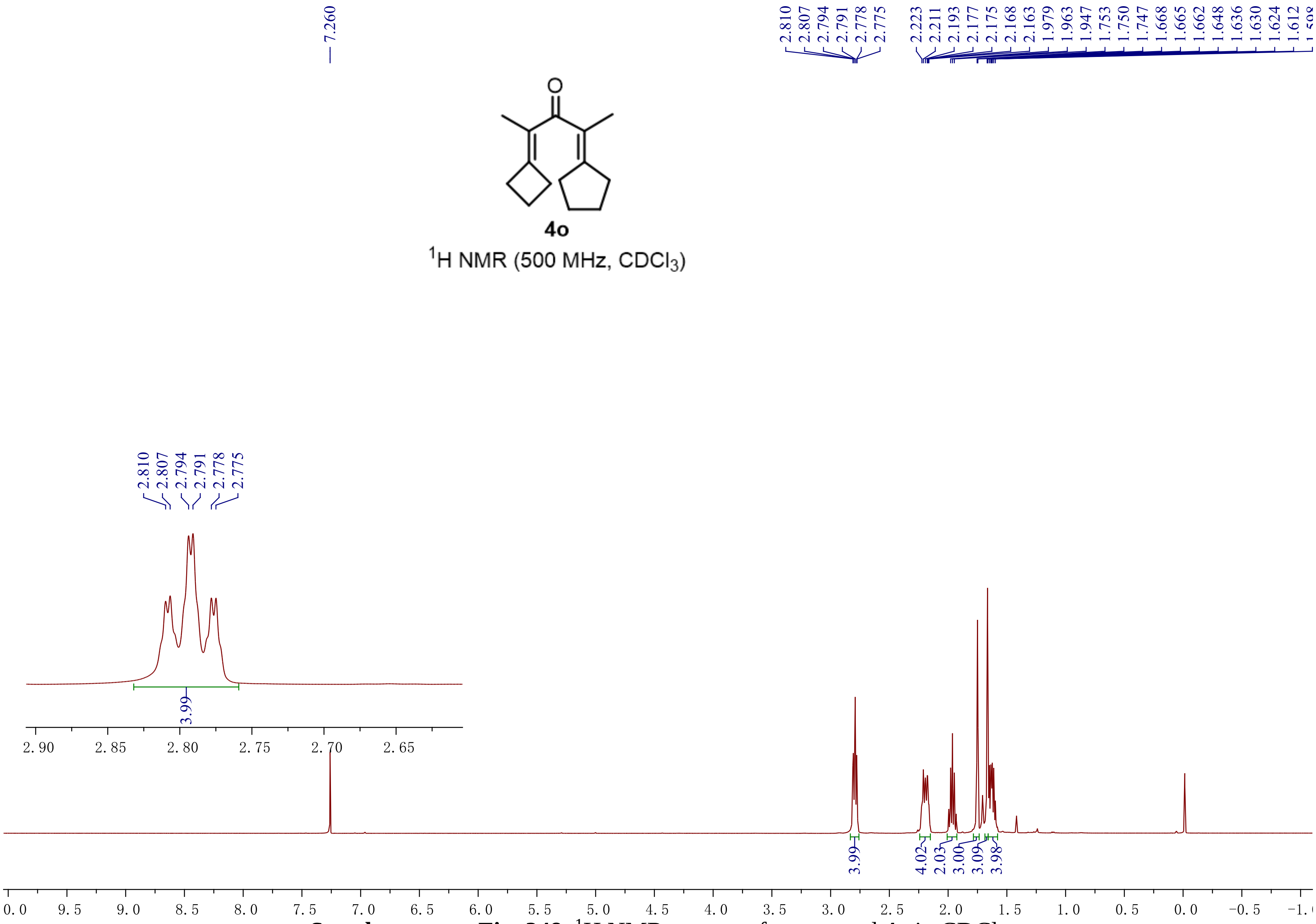

Supplementary Fig. 248. ¹H NMR spectra of compound **4o** in $CDCl_3$

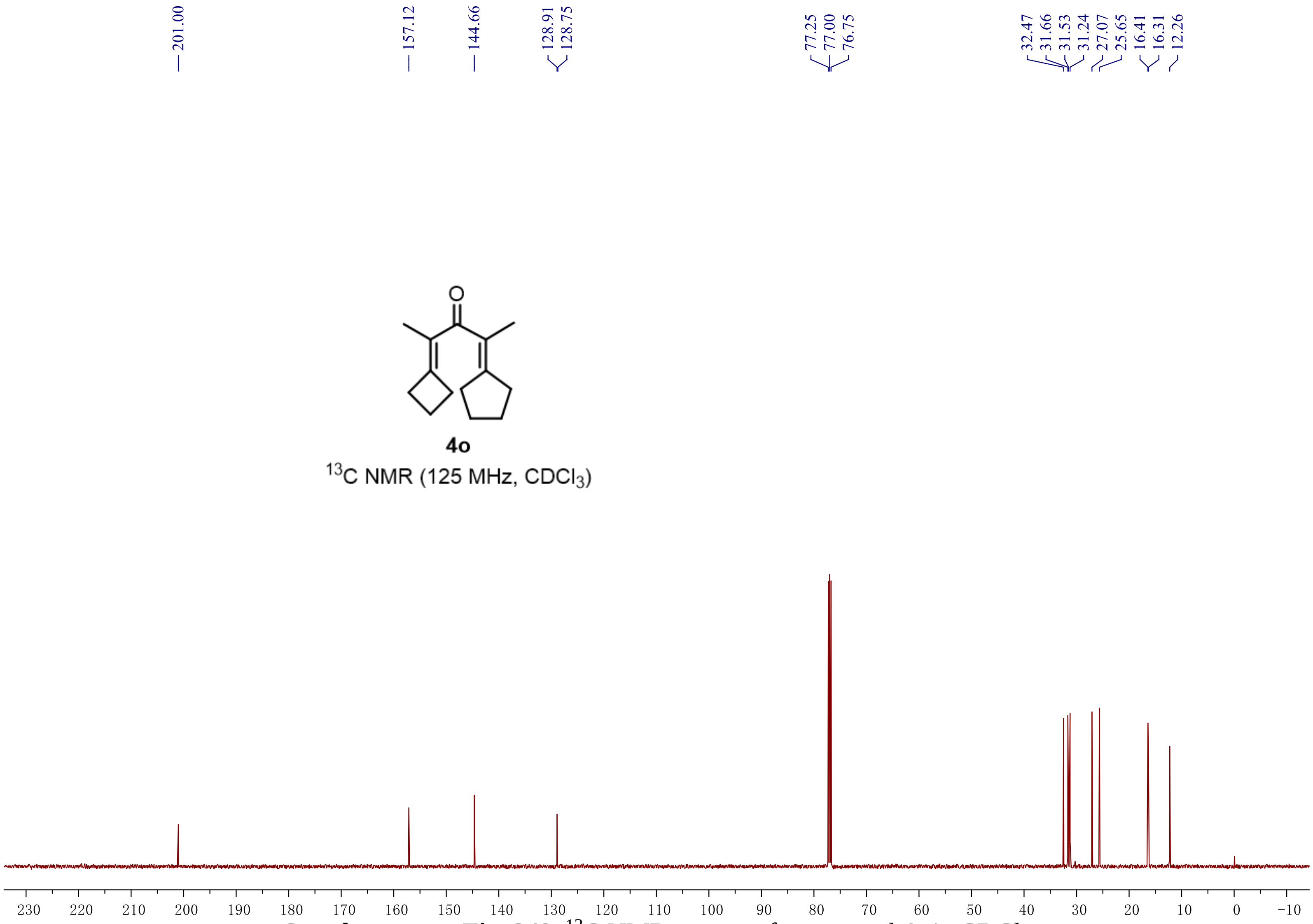

Supplementary Fig. 249. ^{13}C NMR spectra of compound **4o** in $CDCl_3$

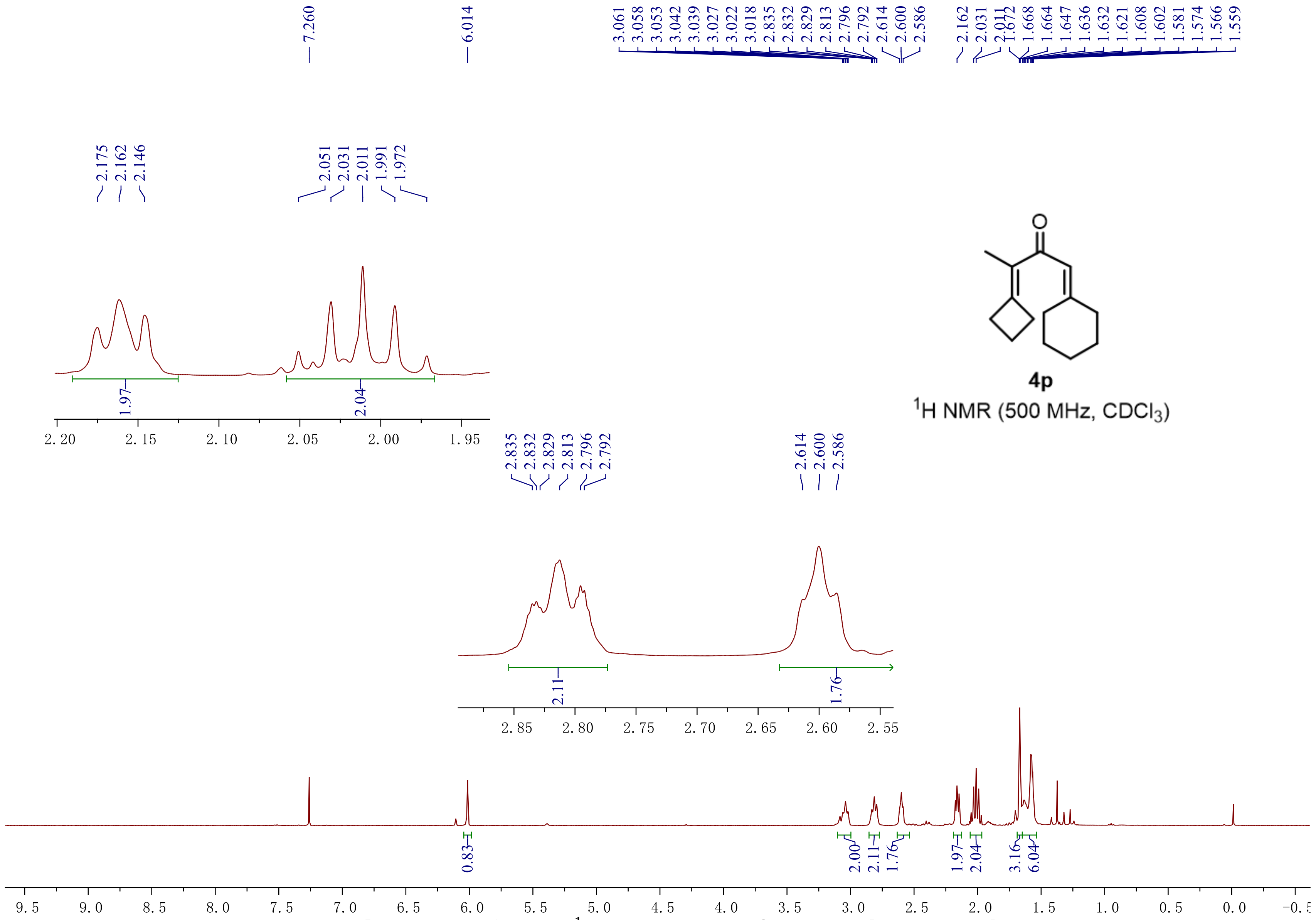

Supplementary Fig. 250. ^{1}H NMR spectra of compound **4p** in $CDCl_3$

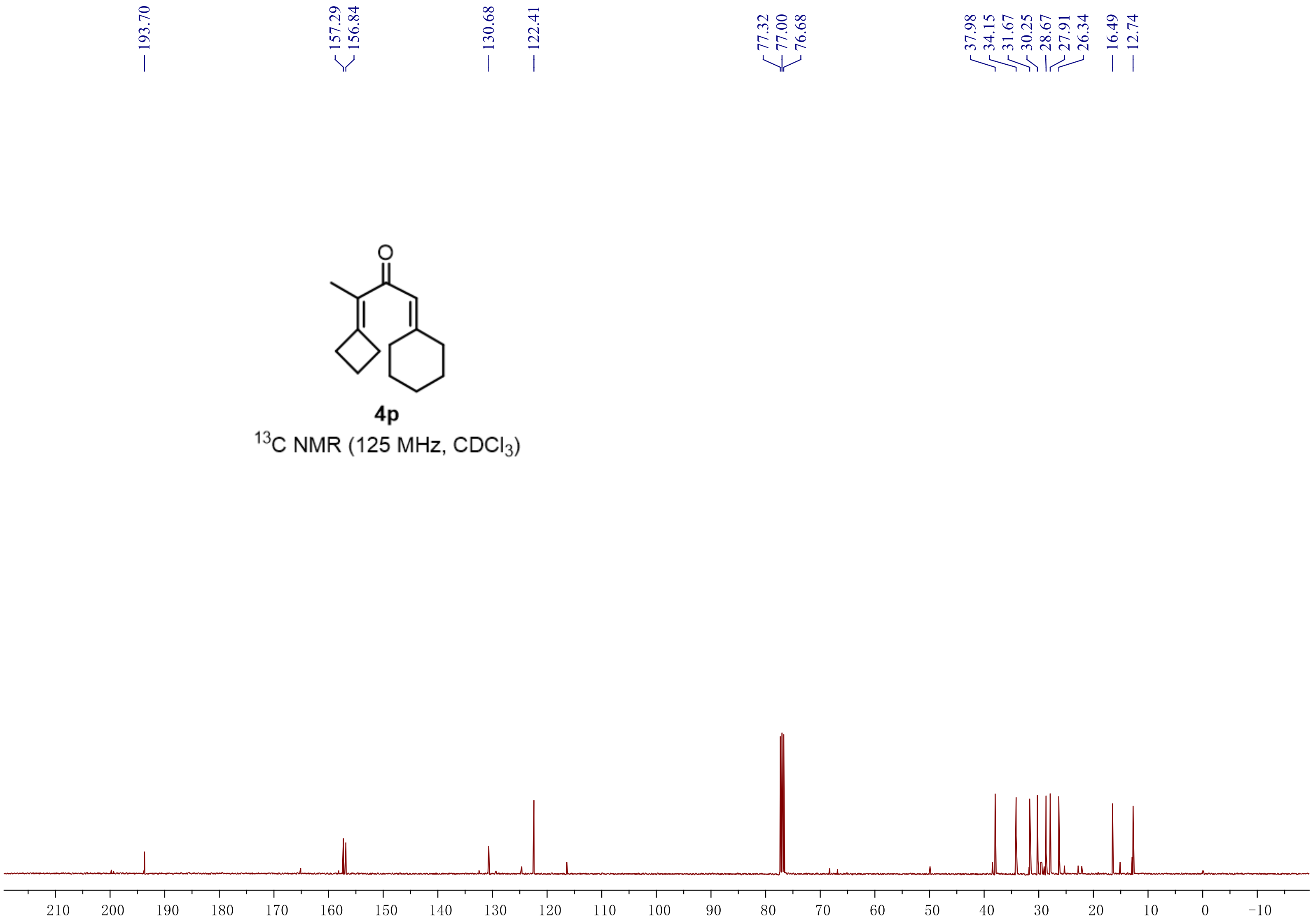

Supplementary Fig. 251. ^{13}C NMR spectra of compound **4p** in $CDCl_3$

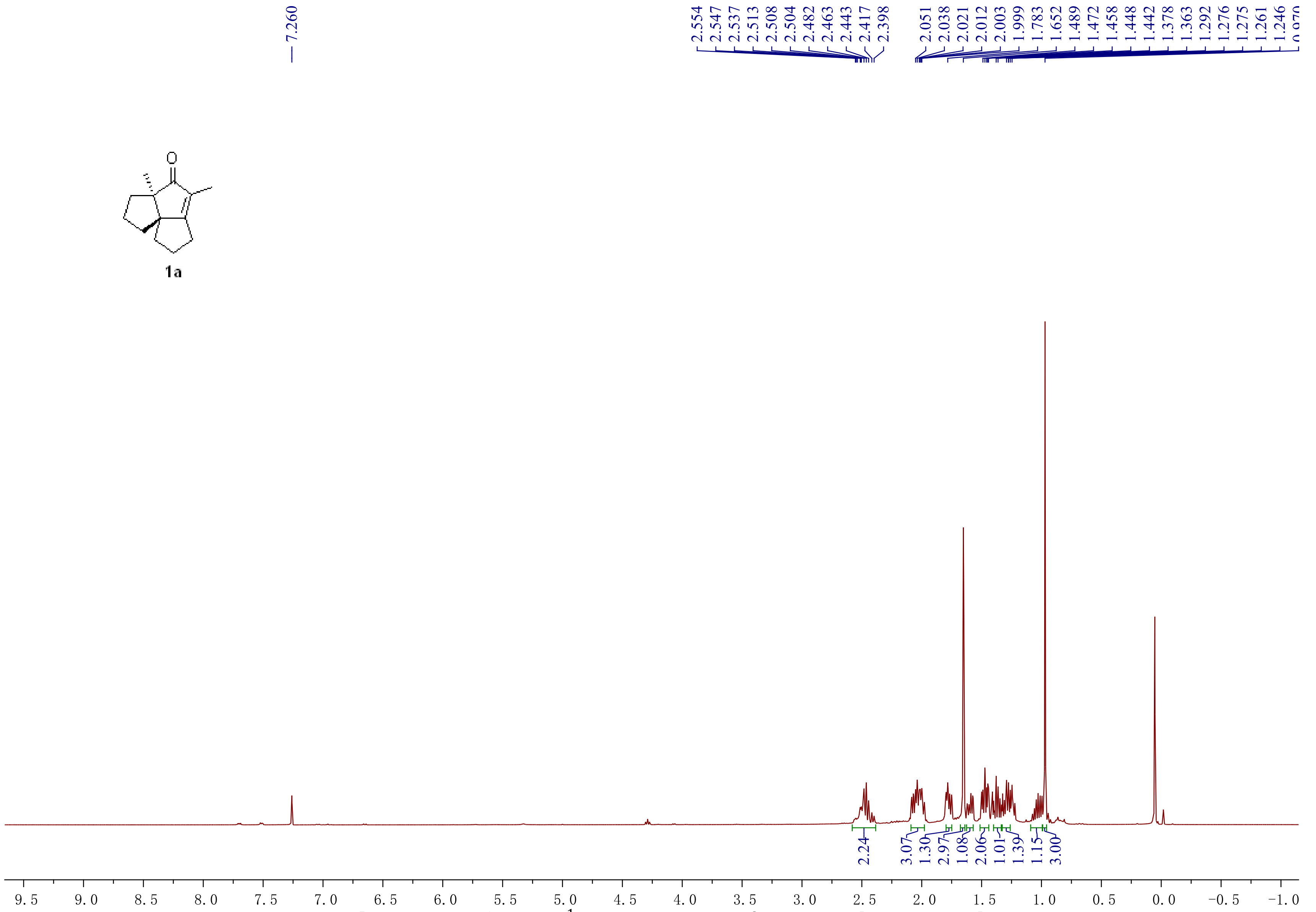

Supplementary Fig. 252. ¹H NMR spectra of compound **1a** in $CDCl_3$

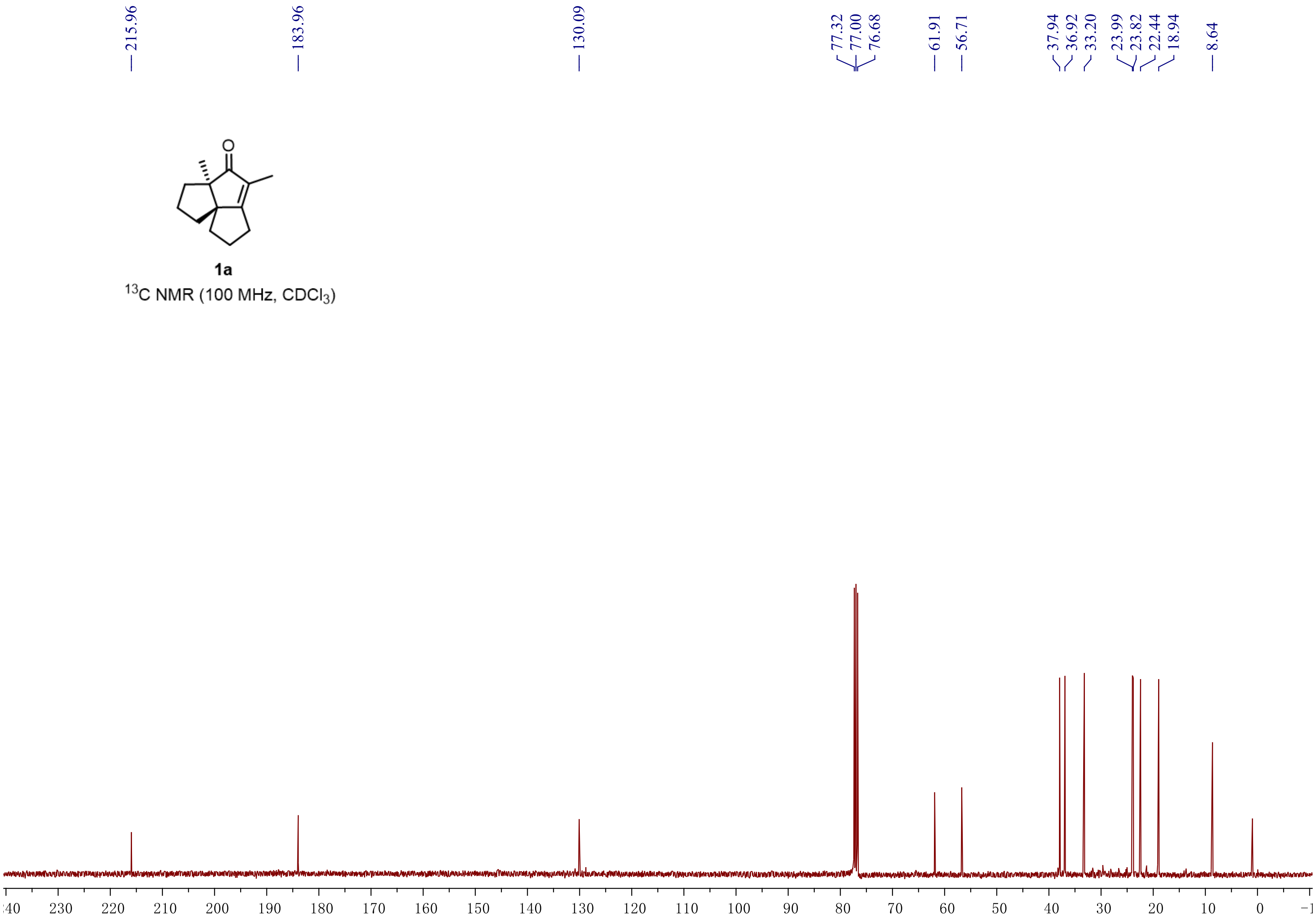

Supplementary Fig. 253. ^{13}C NMR spectra of compound **1a** in $CDCl_3$

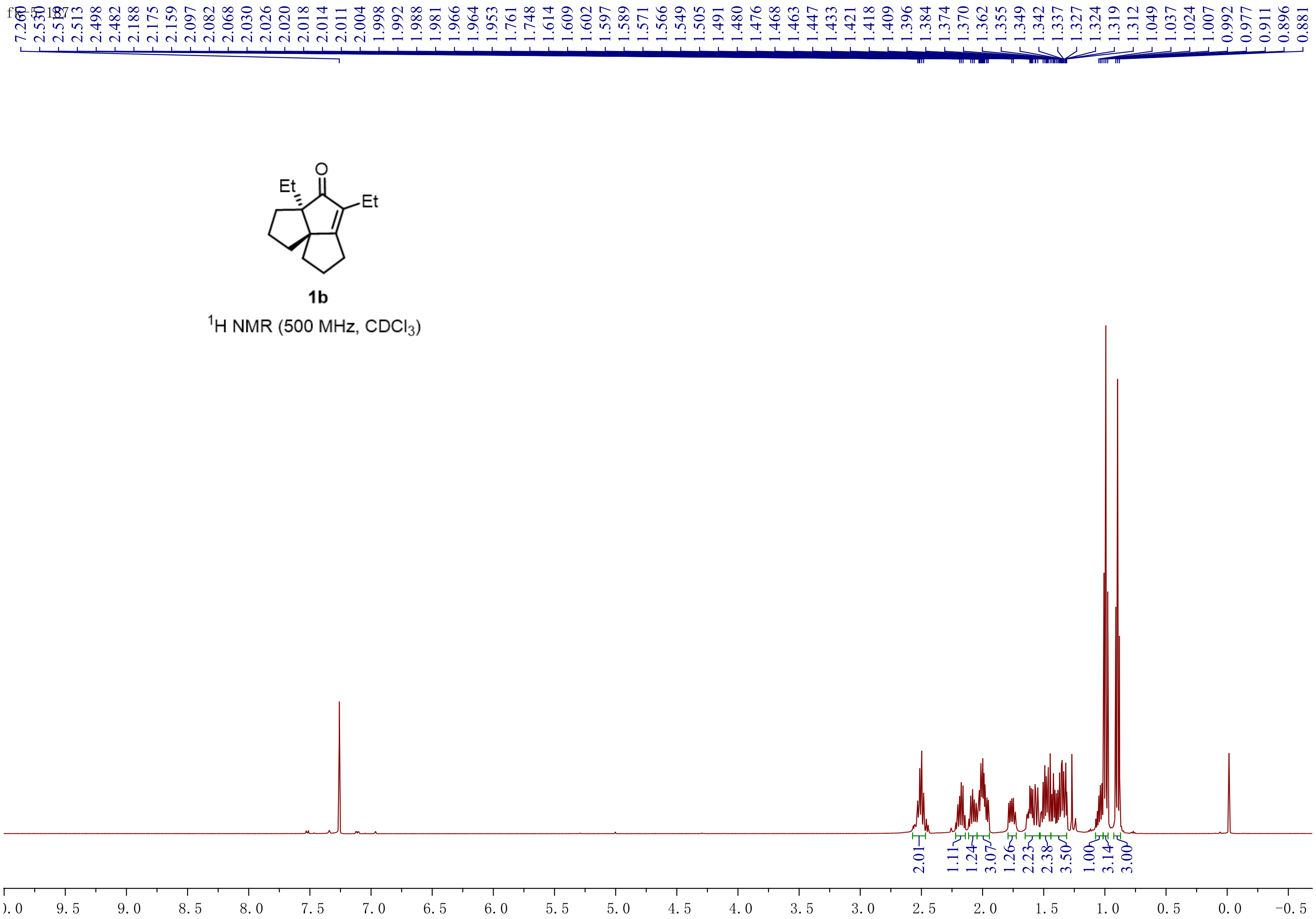

Supplementary Fig. 254. ^{1}H NMR spectra of compound **1b** in $CDCl_3$

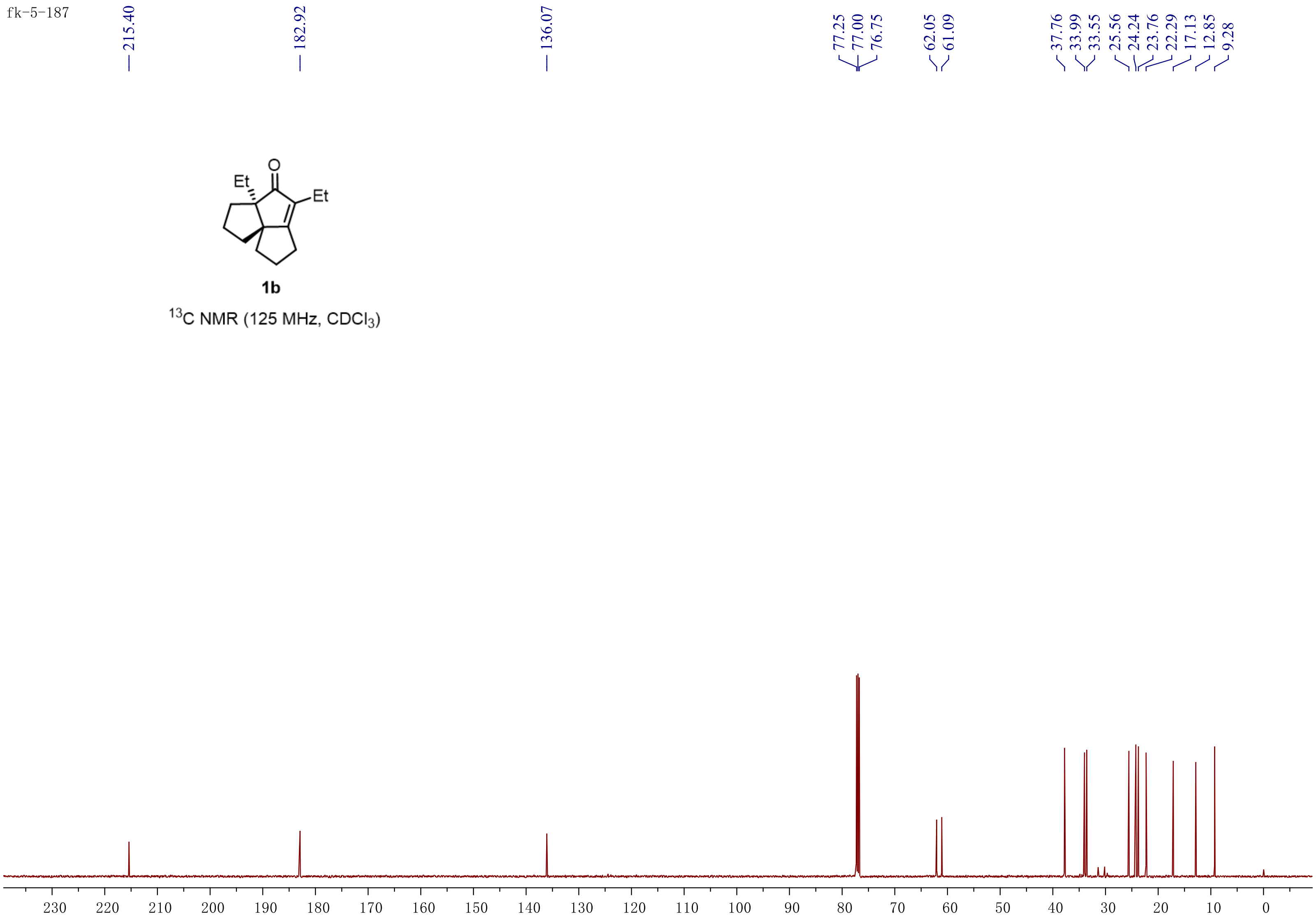

Supplementary Fig. 255. ^{13}C NMR spectra of compound **1b** in $CDCl_3$

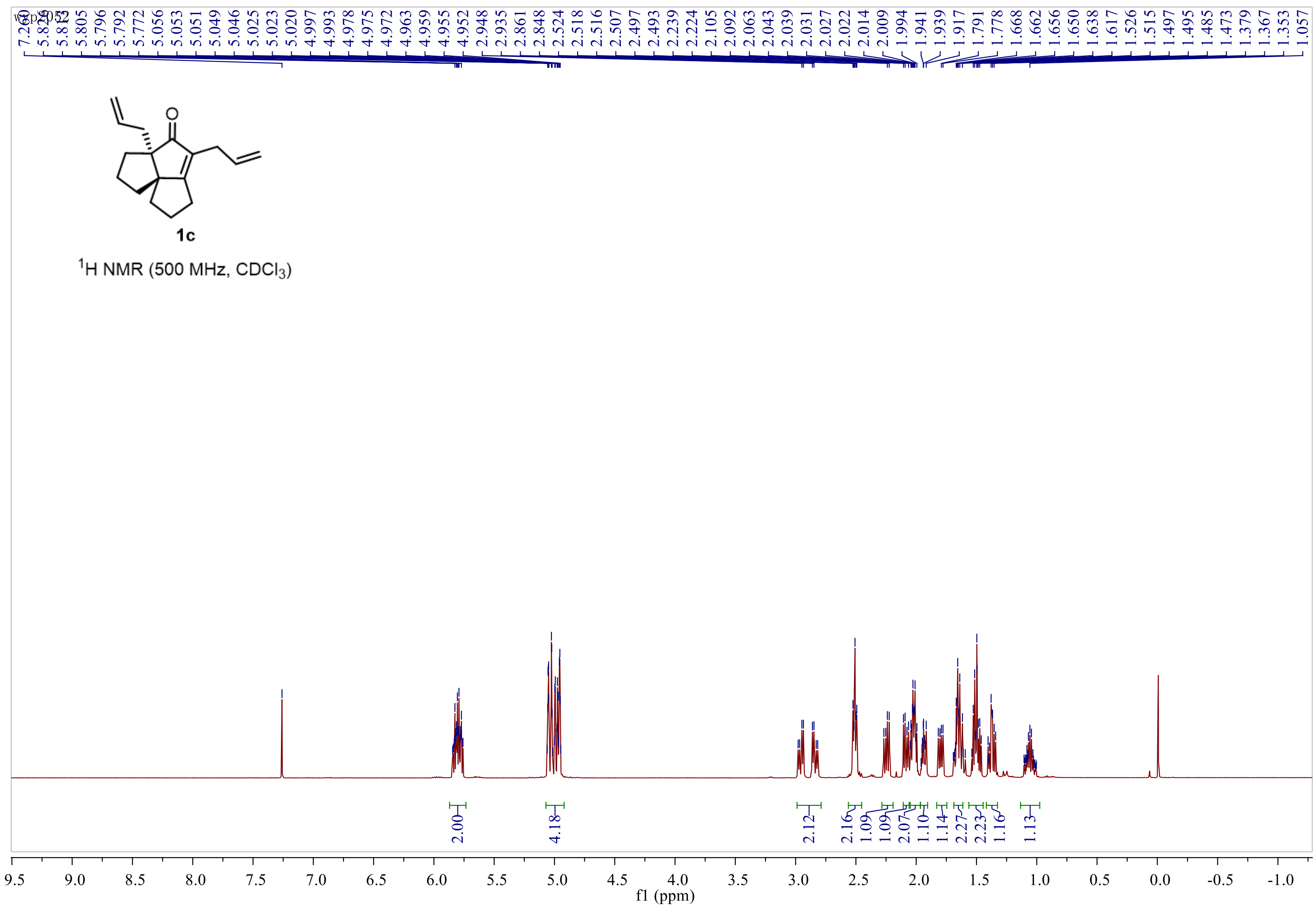

Supplementary Fig. 256. ^{1}H NMR spectra of compound **1c** in $CDCl_3$

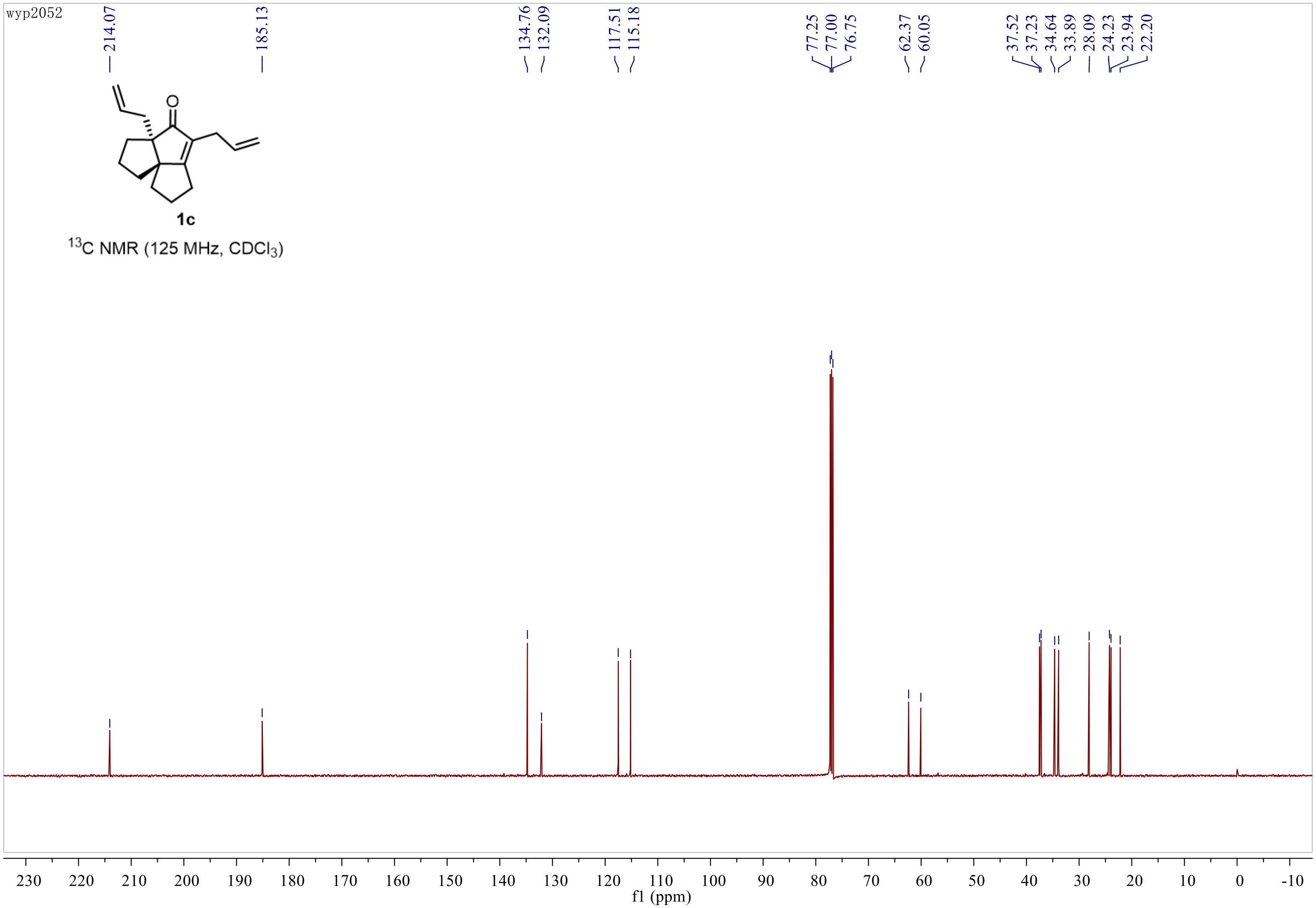

Supplementary Fig. 257. ^{13}C NMR spectra of compound **1c** in $CDCl_3$

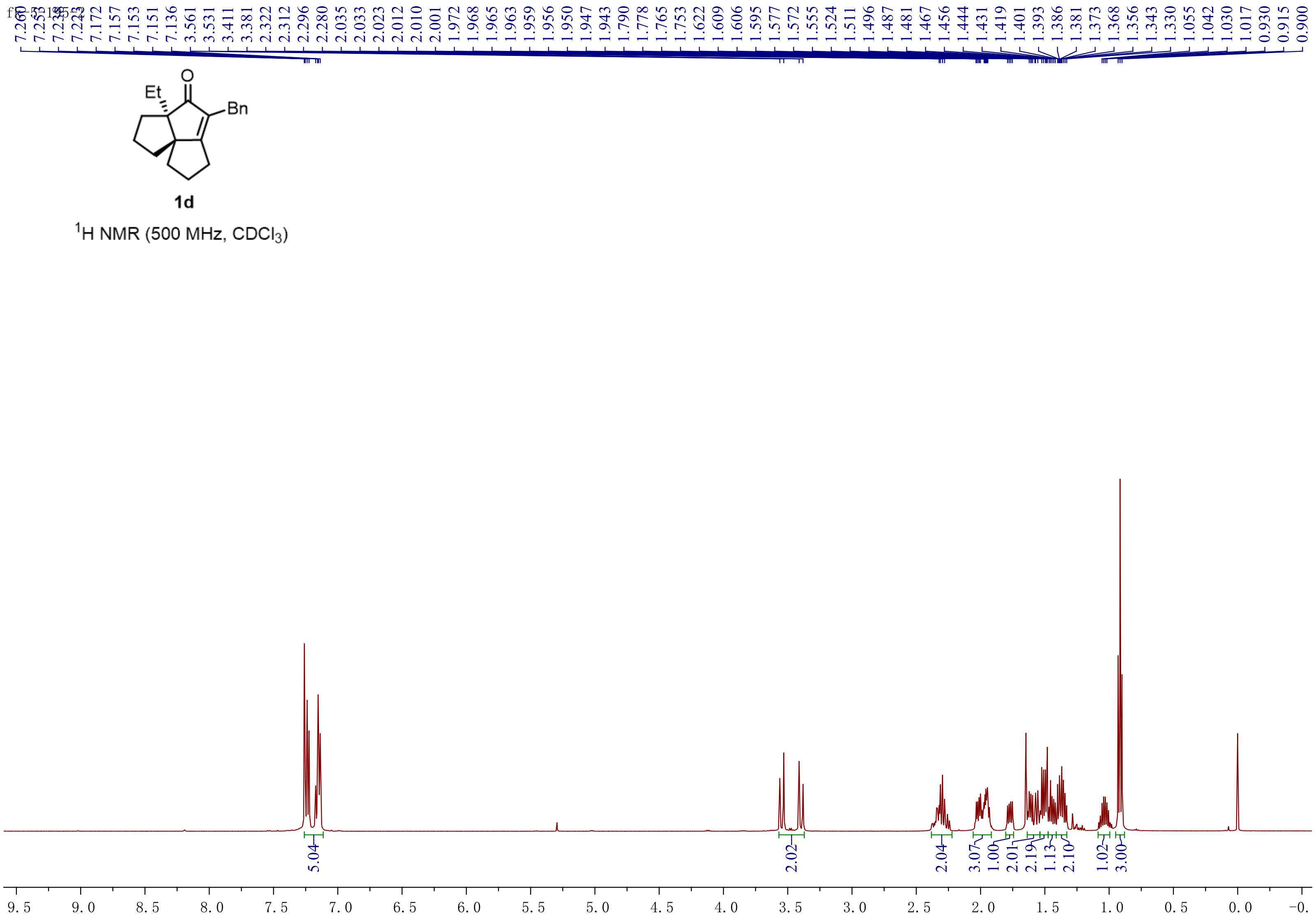

Supplementary Fig. 258. ^{1}H NMR spectra of compound **1d** in $CDCl_3$

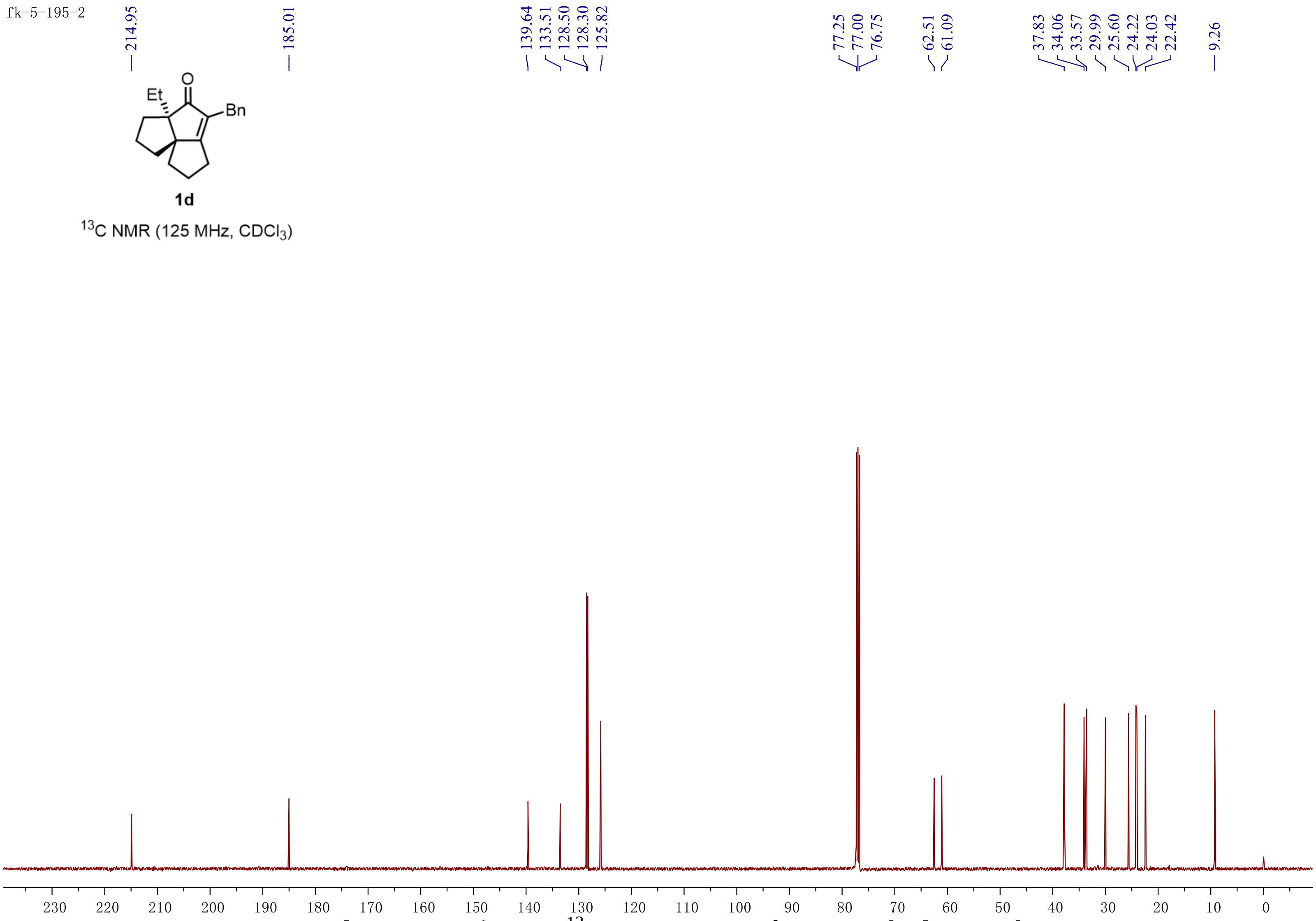

Supplementary Fig. 259. ^{13}C NMR spectra of compound **1d** in $CDCl_3$

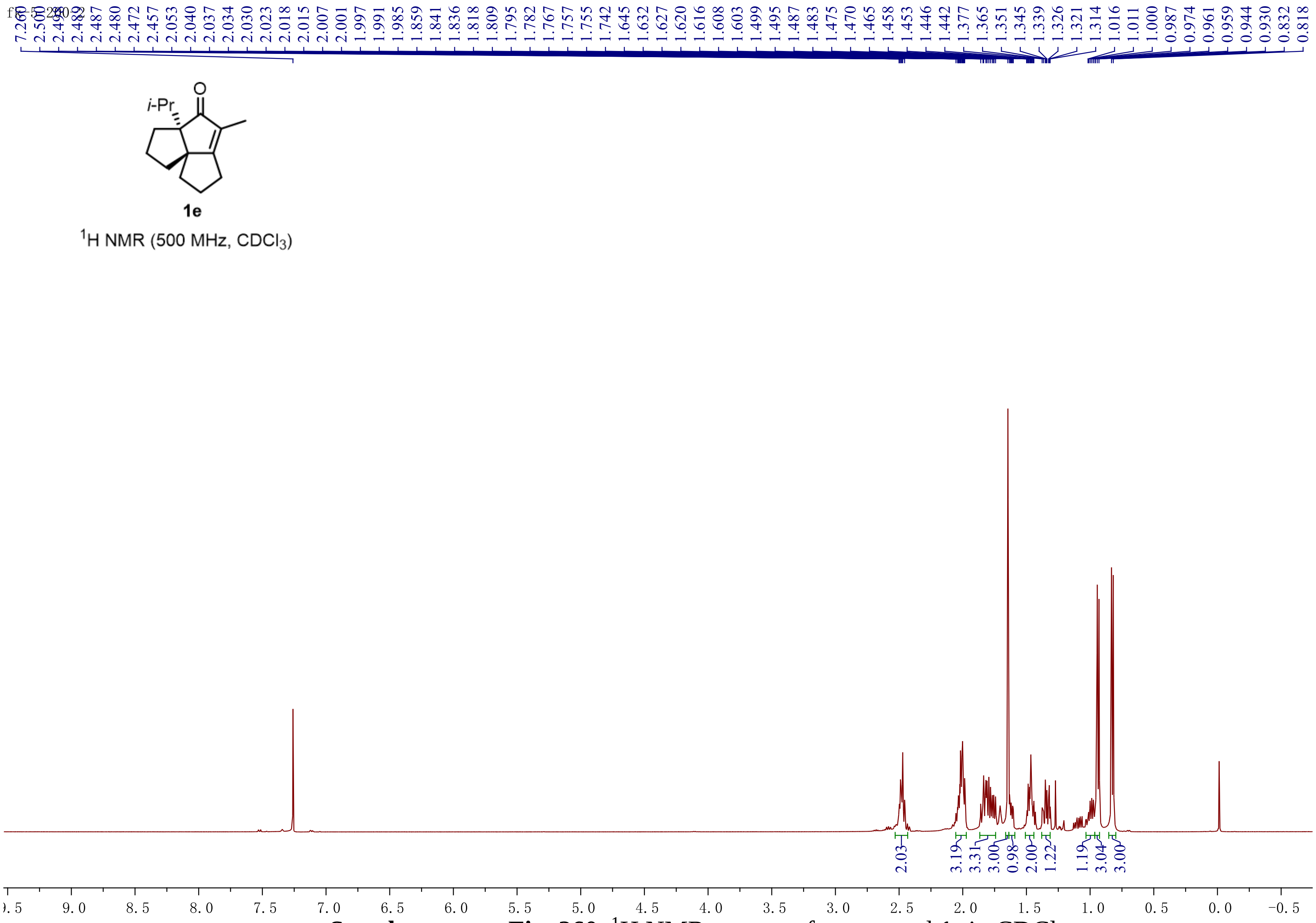

Supplementary Fig. 260. ^{1}H NMR spectra of compound **1e** in $CDCl_3$

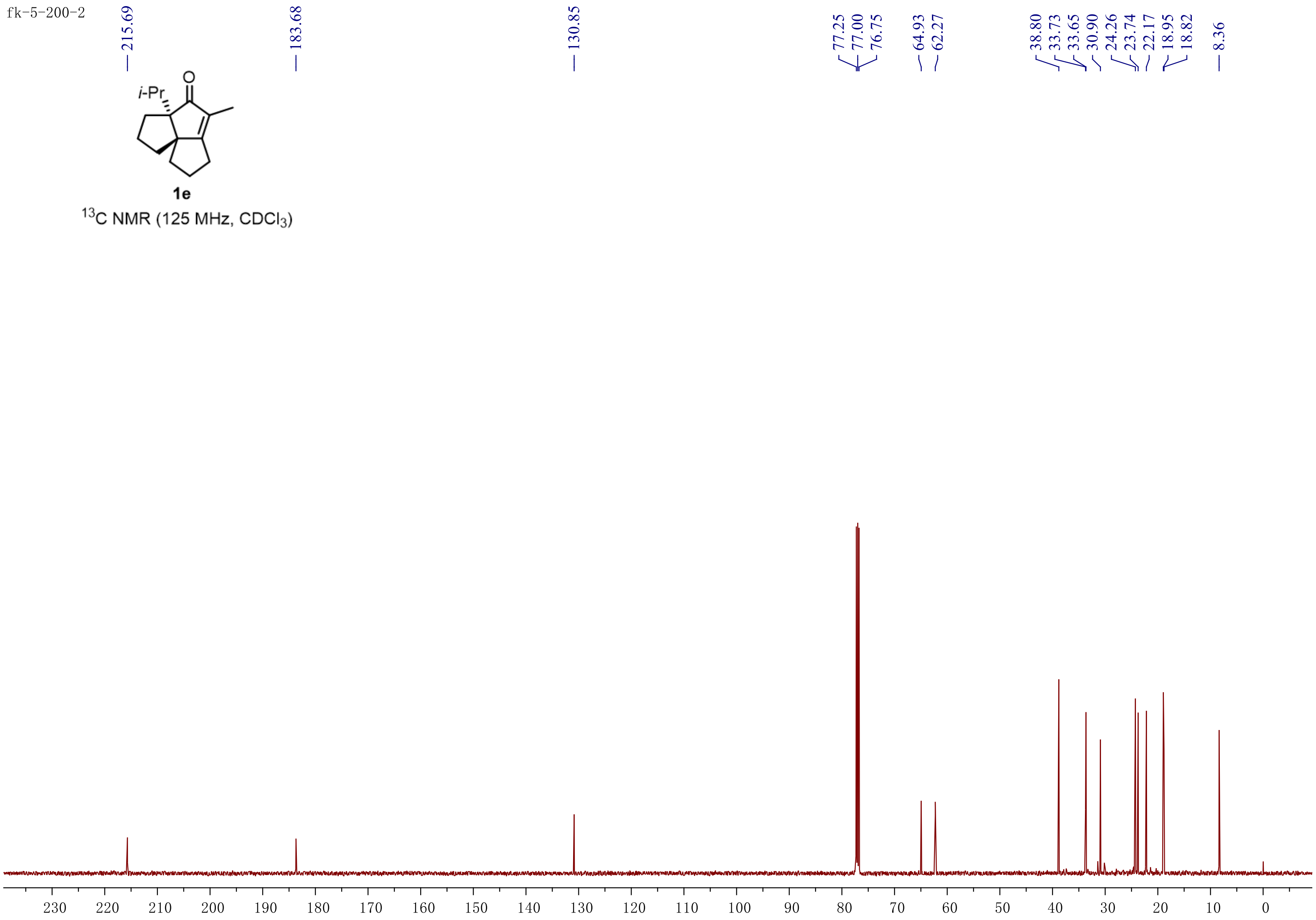

Supplementary Fig. 261. ^{13}C NMR spectra of compound **1e** in $CDCl_3$

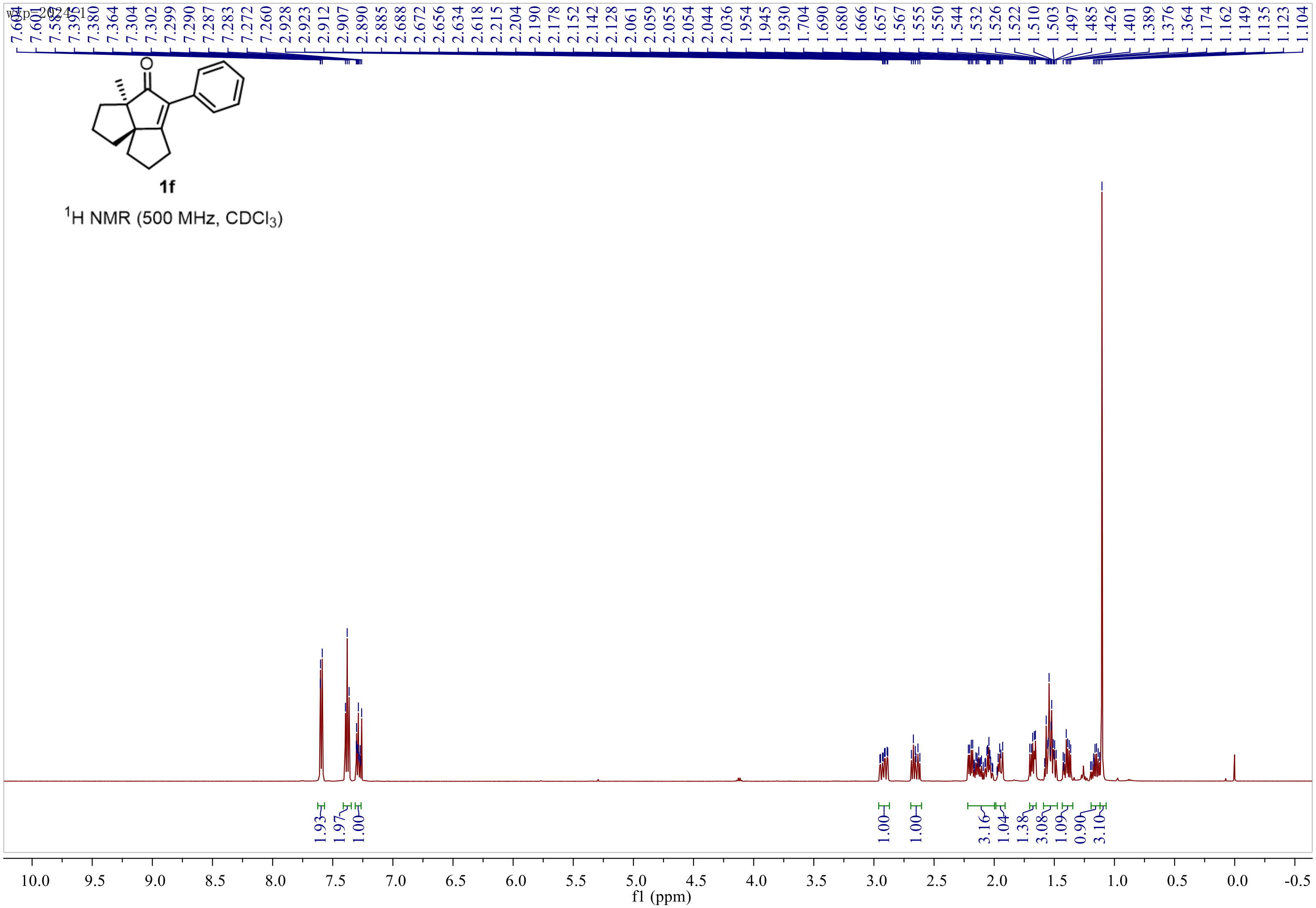

Supplementary Fig. 262. ^{1}H NMR spectra of compound **1f** in $CDCl_3$

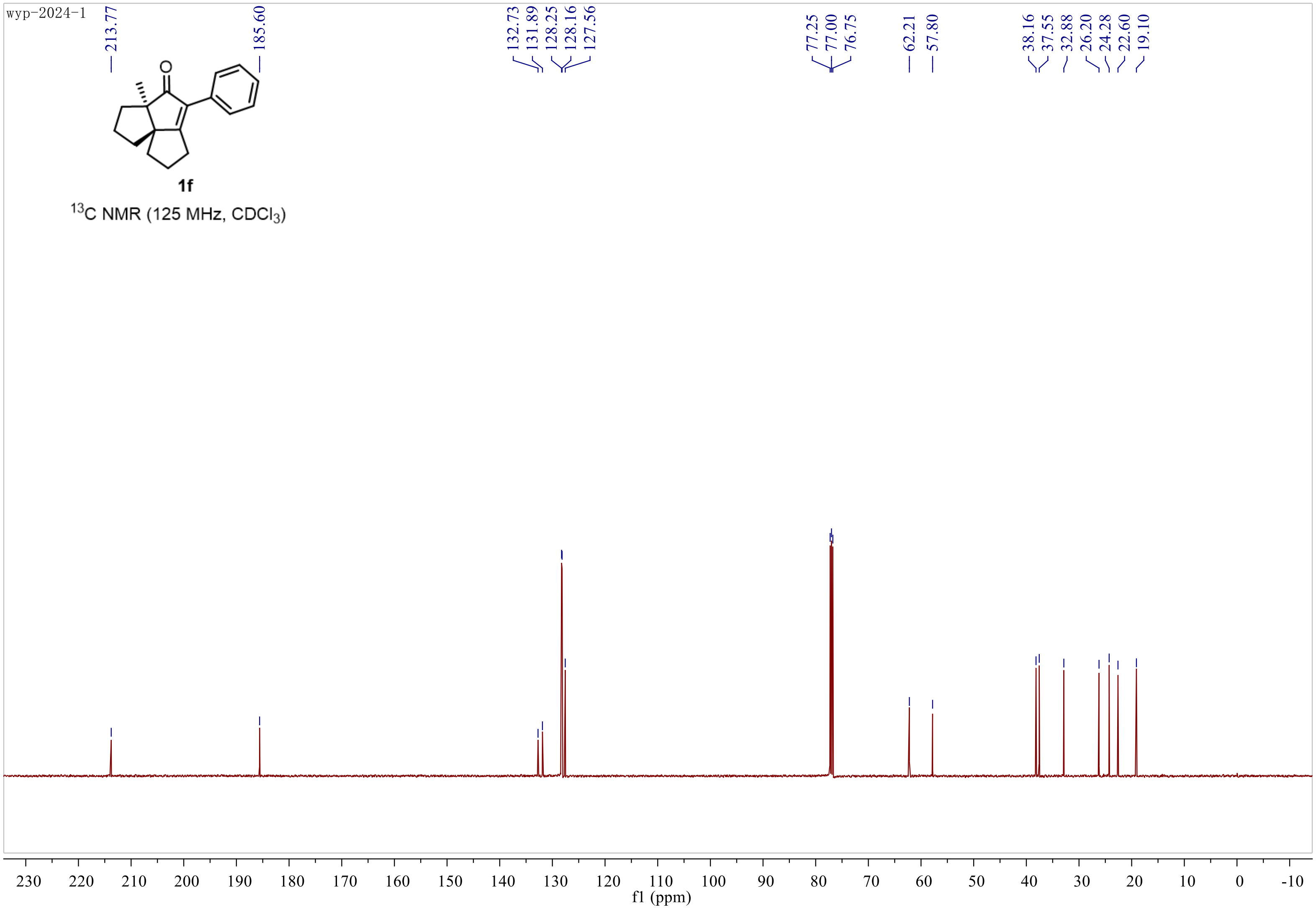

Supplementary Fig. 263. ^{13}C NMR spectra of compound **1f** in $CDCl_3$

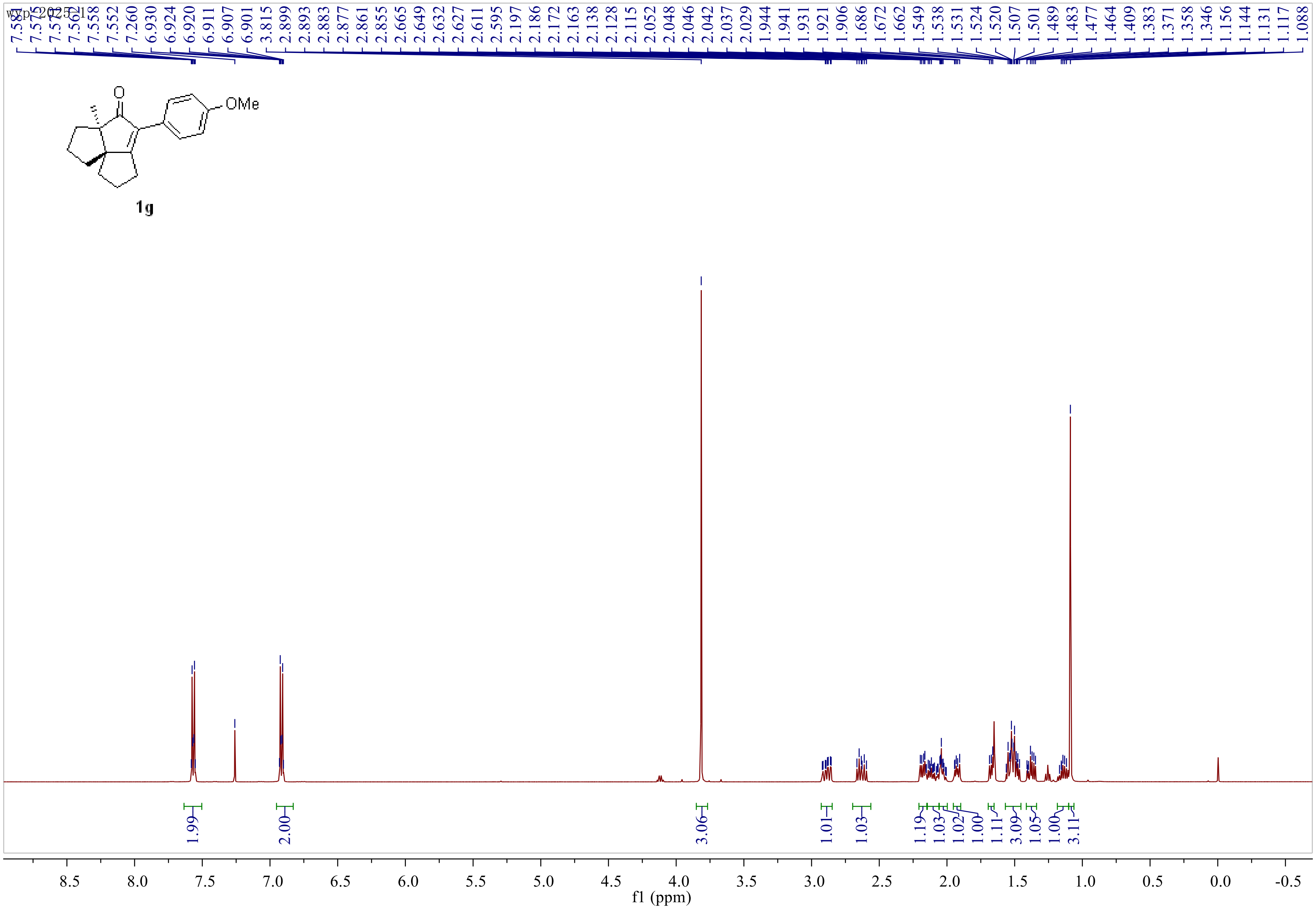

Supplementary Fig. 264. ^{1}H NMR spectra of compound **1g** in $CDCl_3$

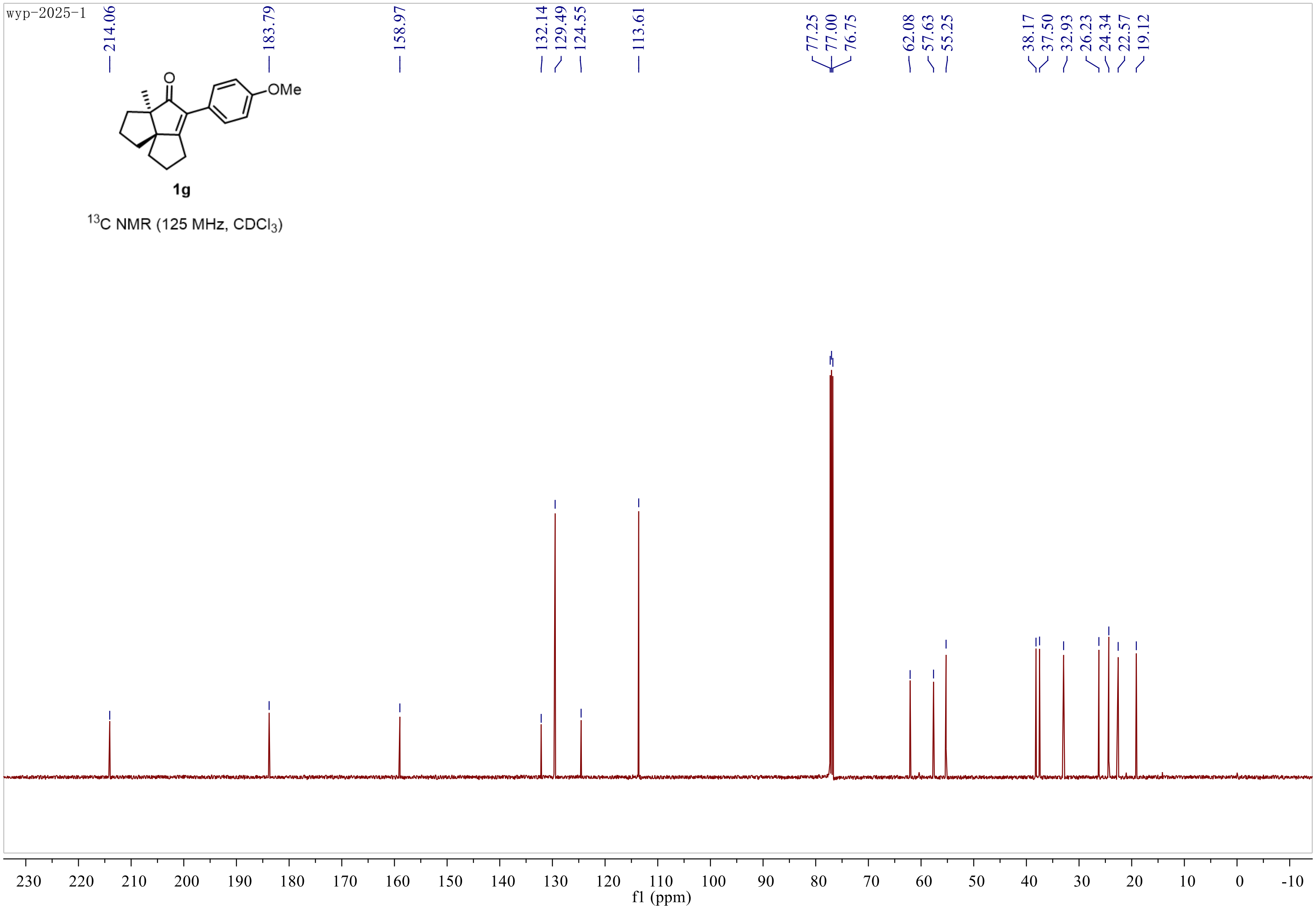

Supplementary Fig. 265. ^{13}C NMR spectra of compound **1g** in $CDCl_3$

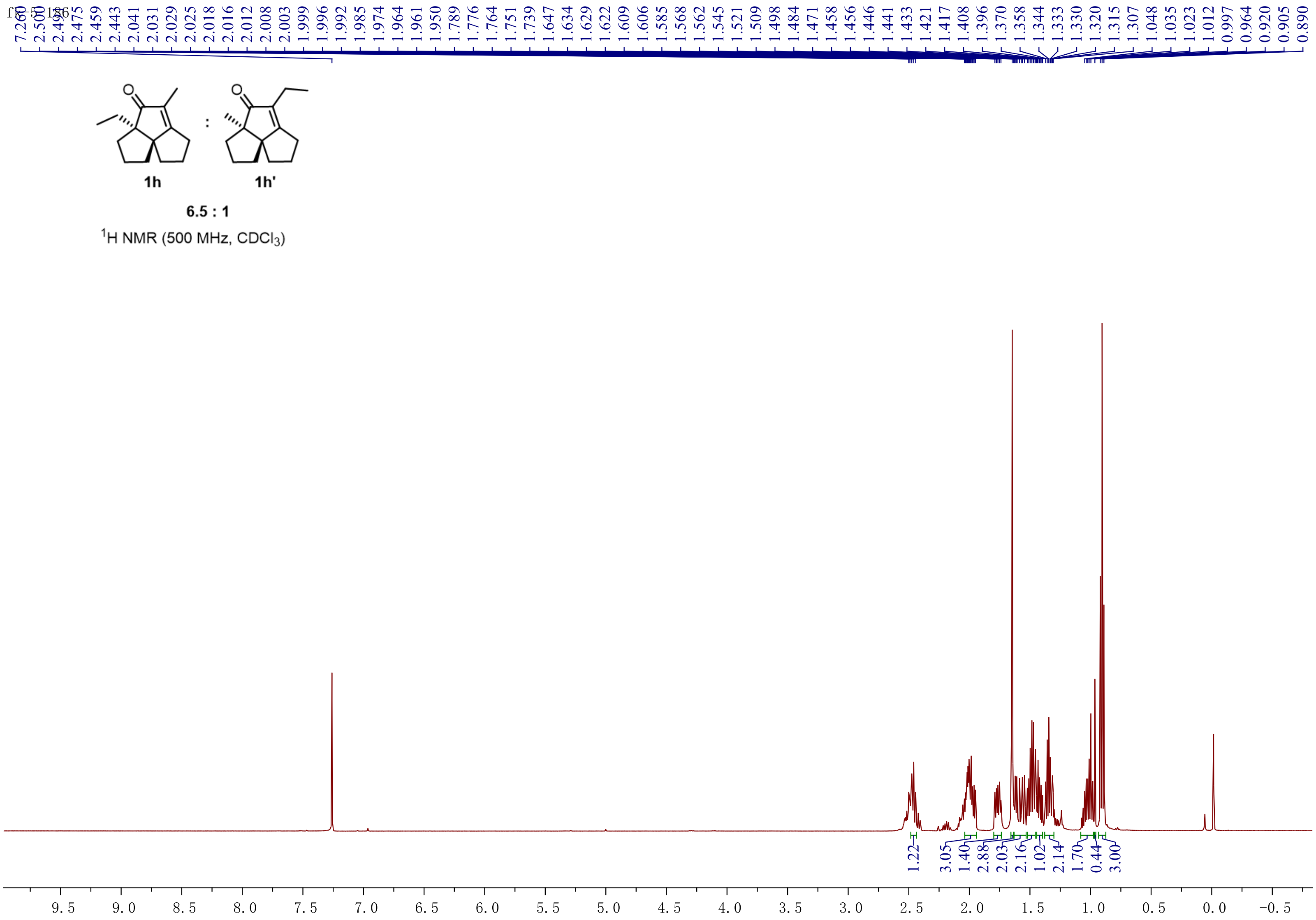

Supplementary Fig. 266. ^{1}H NMR spectra of compound **1h** and **1h'** in $CDCl_3$

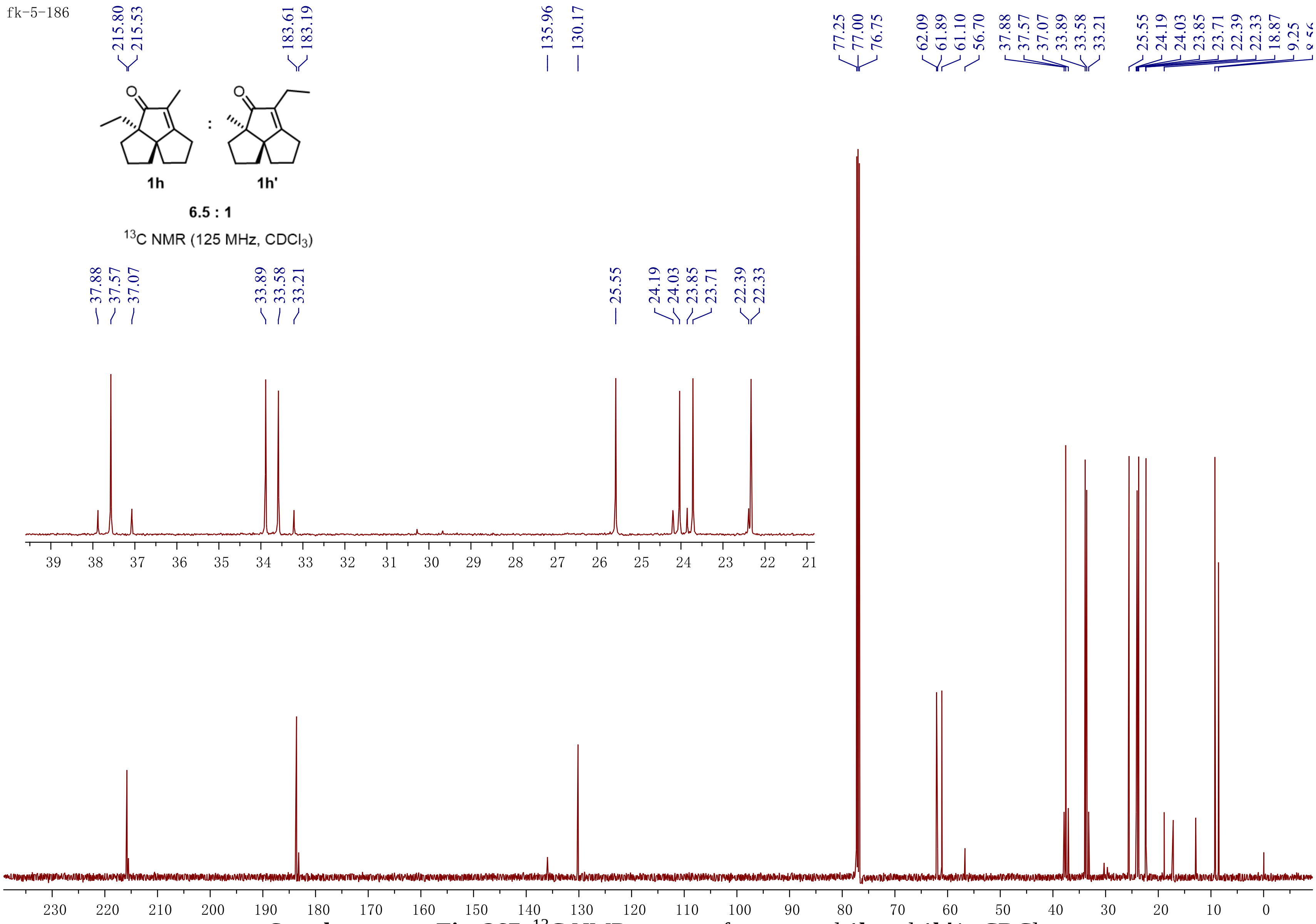

Supplementary Fig. 267. ^{13}C NMR spectra of compound **1h** and **1h'** in $CDCl_3$

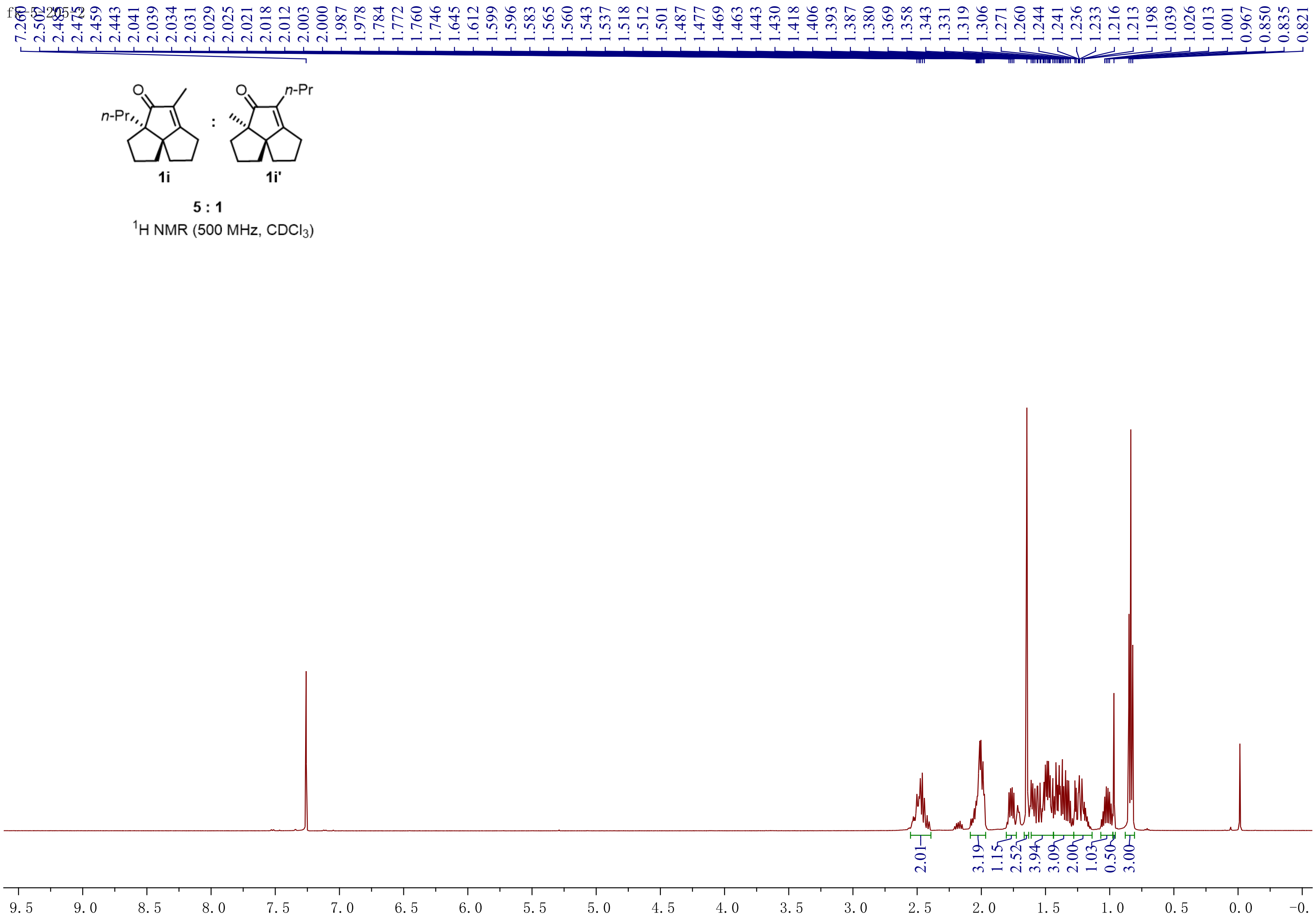

Supplementary Fig. 268. ^{1}H NMR spectra of compound **1i** and **1i'** in $CDCl_3$

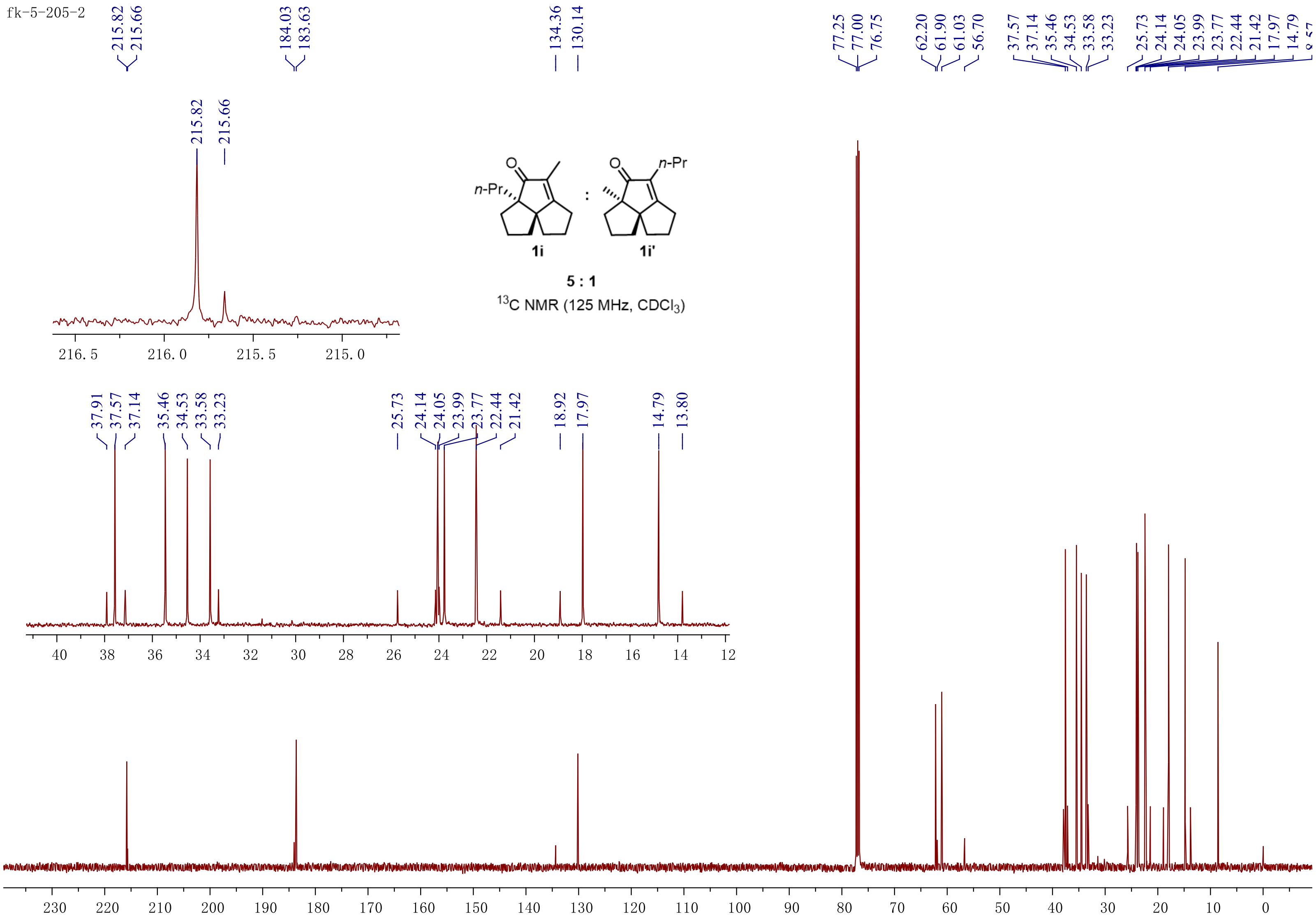

Supplementary Fig. 269. ^{13}C NMR spectra of compound **1i** and **1i'** in $CDCl_3$

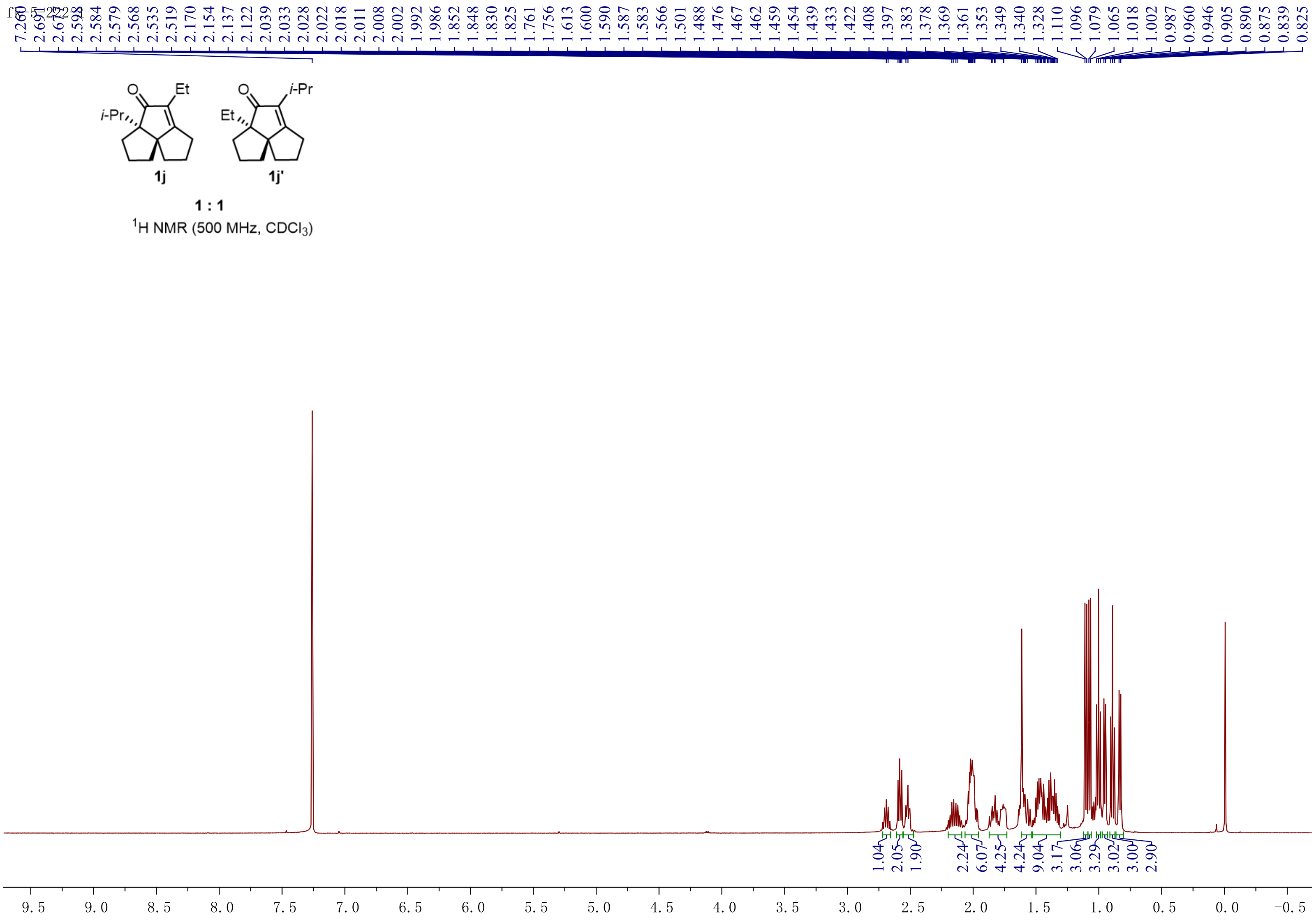

Supplementary Fig. 270. ^{1}H NMR spectra of compound **1j** and **1j'** in $CDCl_3$

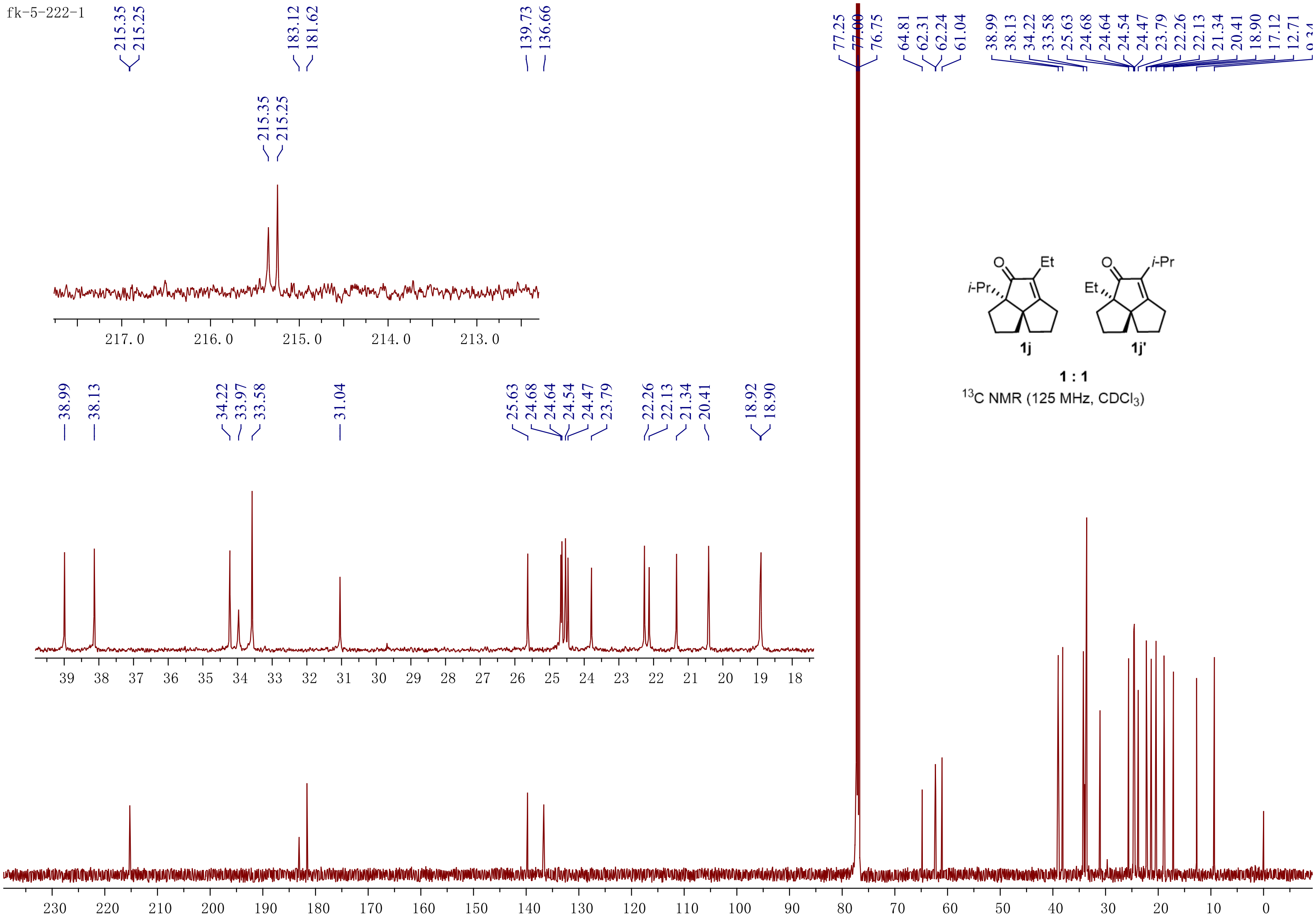

Supplementary Fig. 271. ^{13}C NMR spectra of compound **1j** and **1j'** in $CDCl_3$

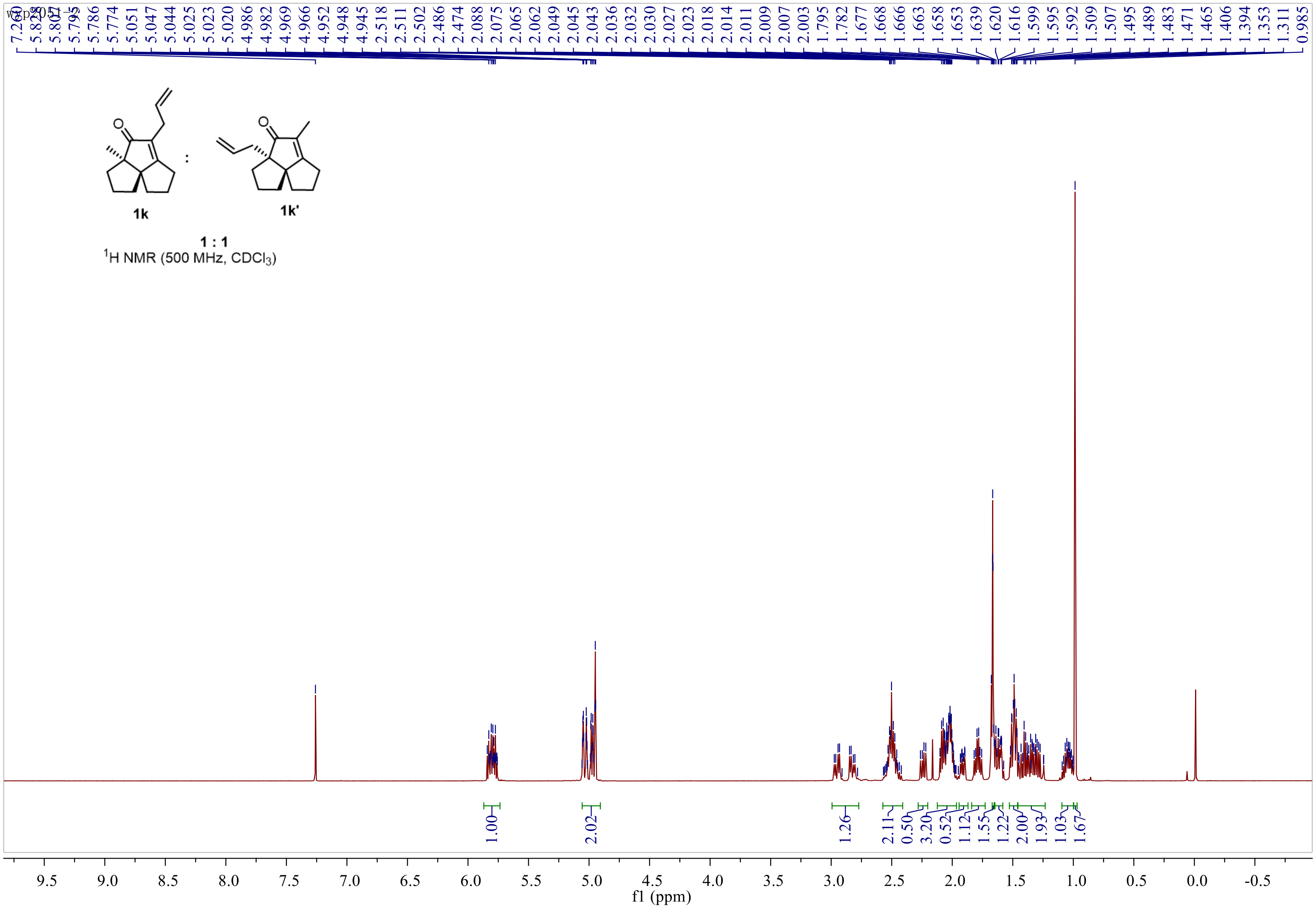

Supplementary Fig. 272. ^{1}H NMR spectra of compound **1k** and **1k'** in $CDCl_3$

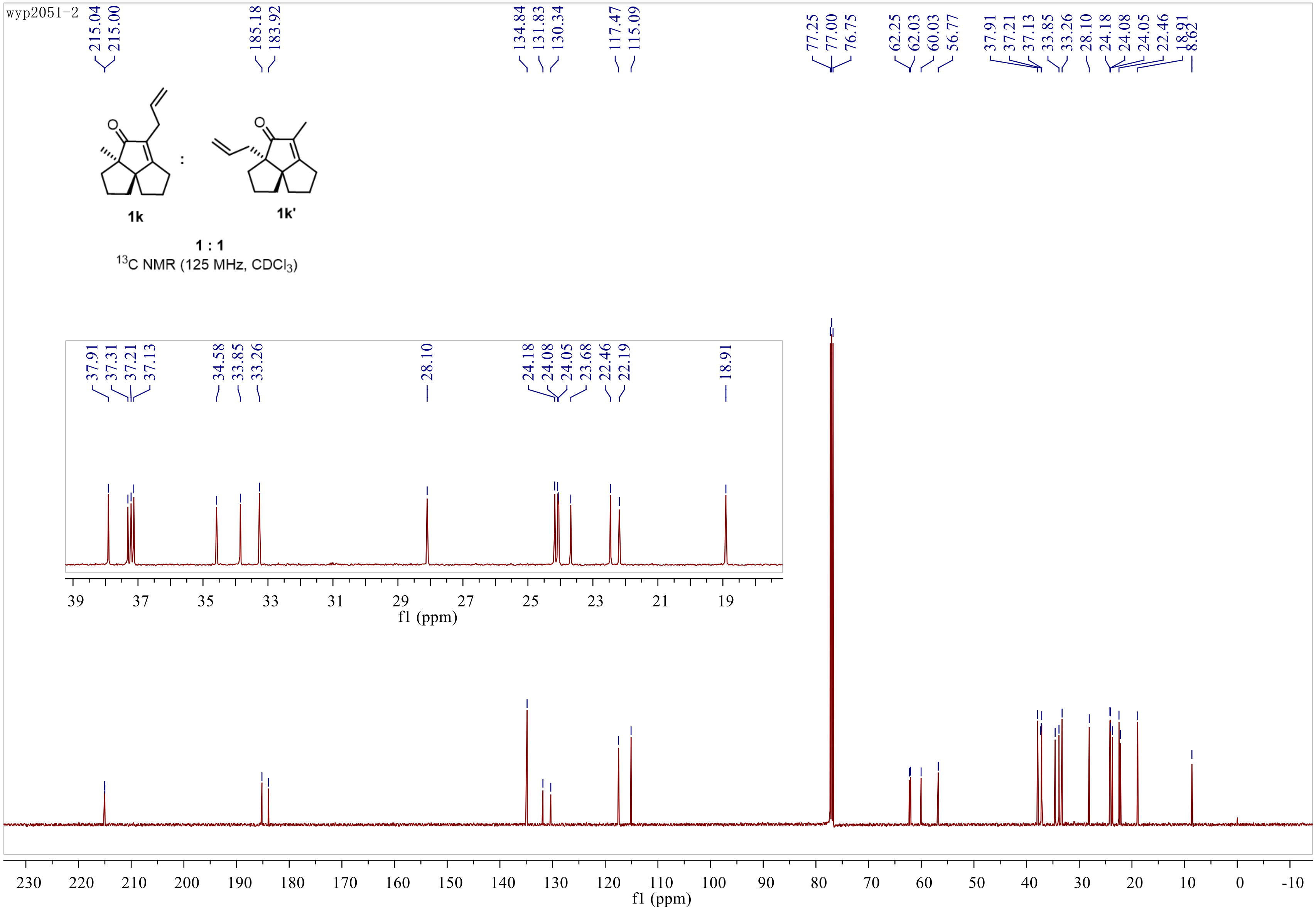

Supplementary Fig. 273. ^{13}C NMR spectra of compound **1k** and **1k'** in $CDCl_3$

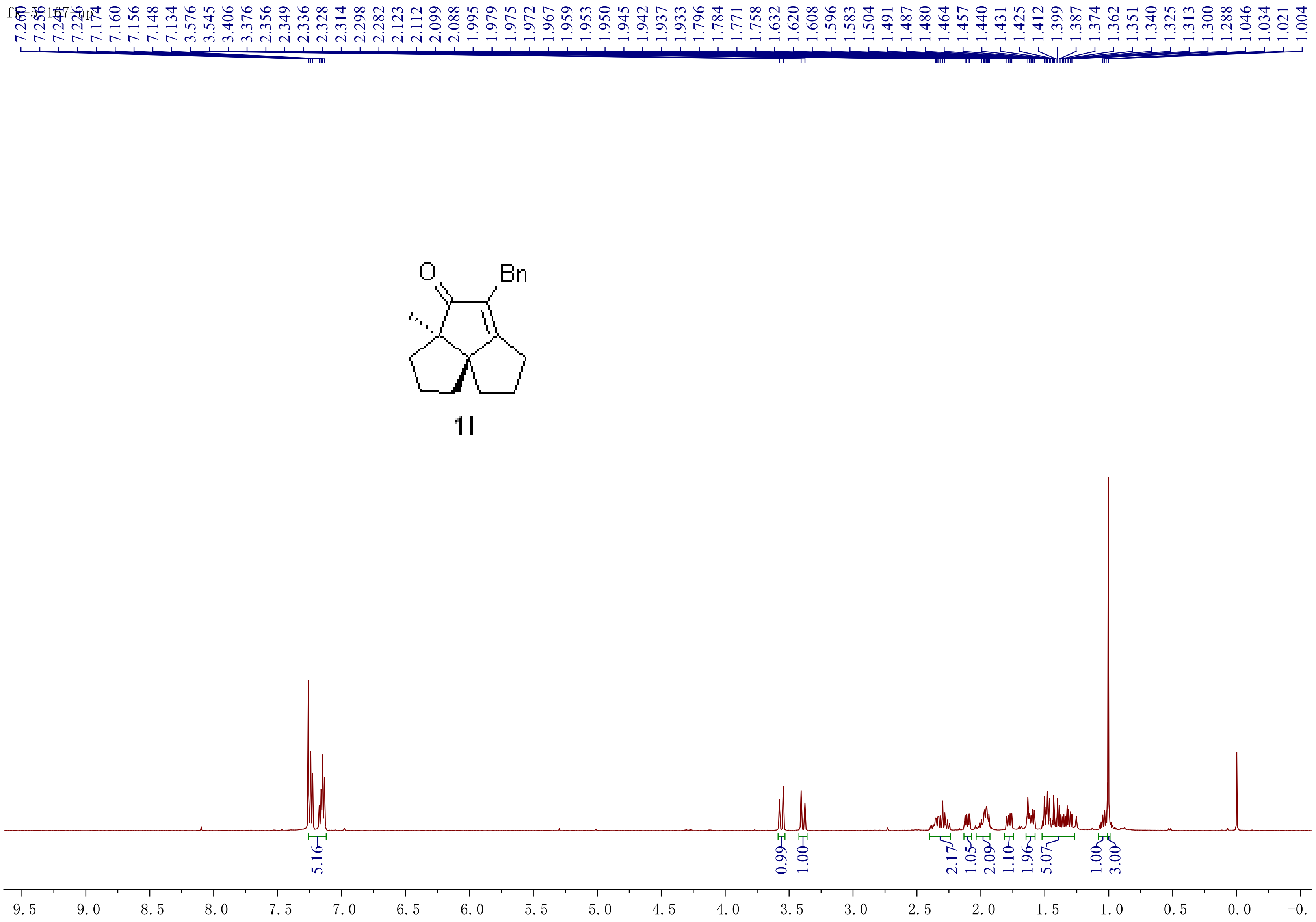

Supplementary Fig. 274. ^{1}H NMR spectra of compound **1l** in $CDCl_3$

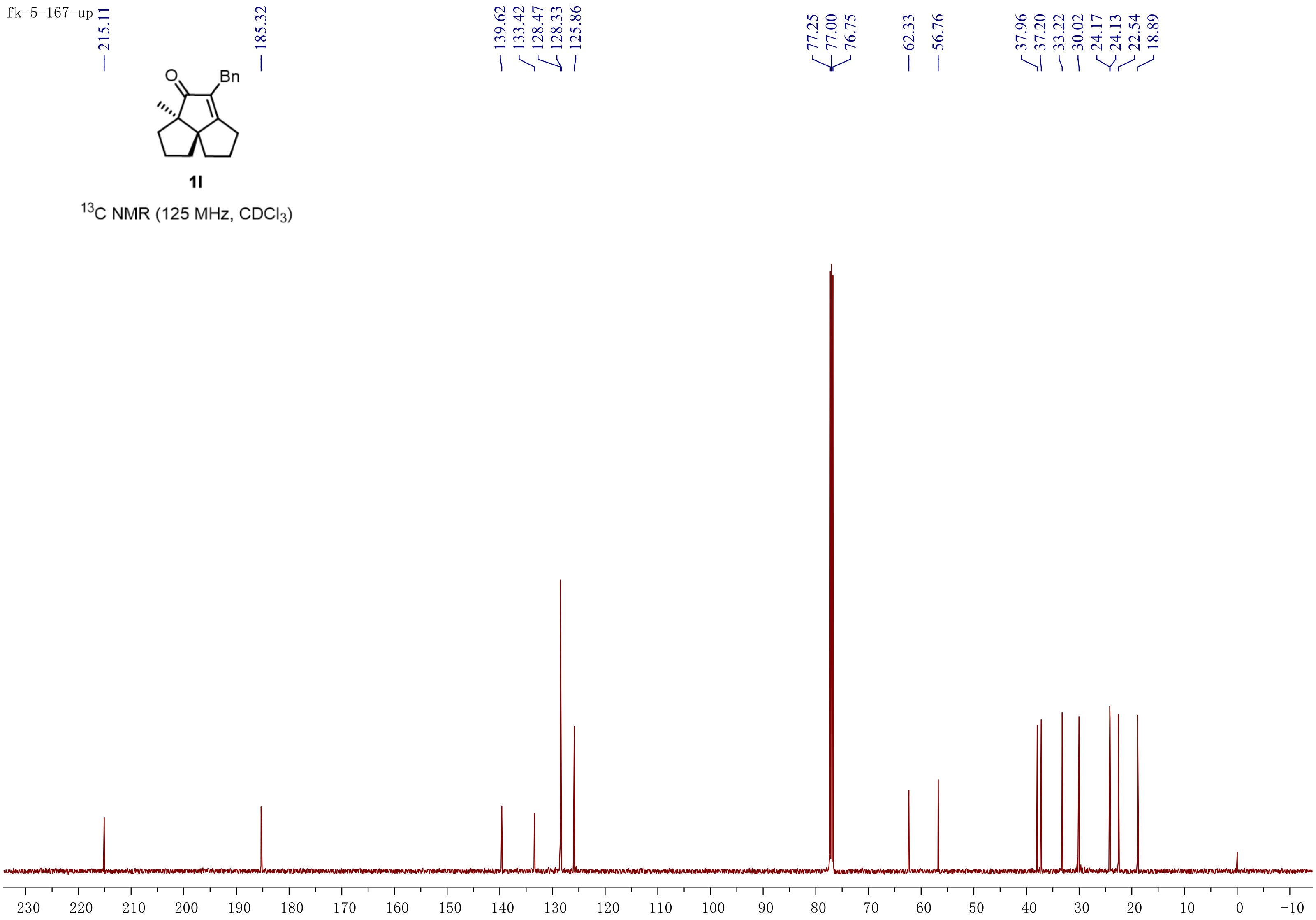

Supplementary Fig. 275. ^{13}C NMR spectra of compound **1l** in $CDCl_3$

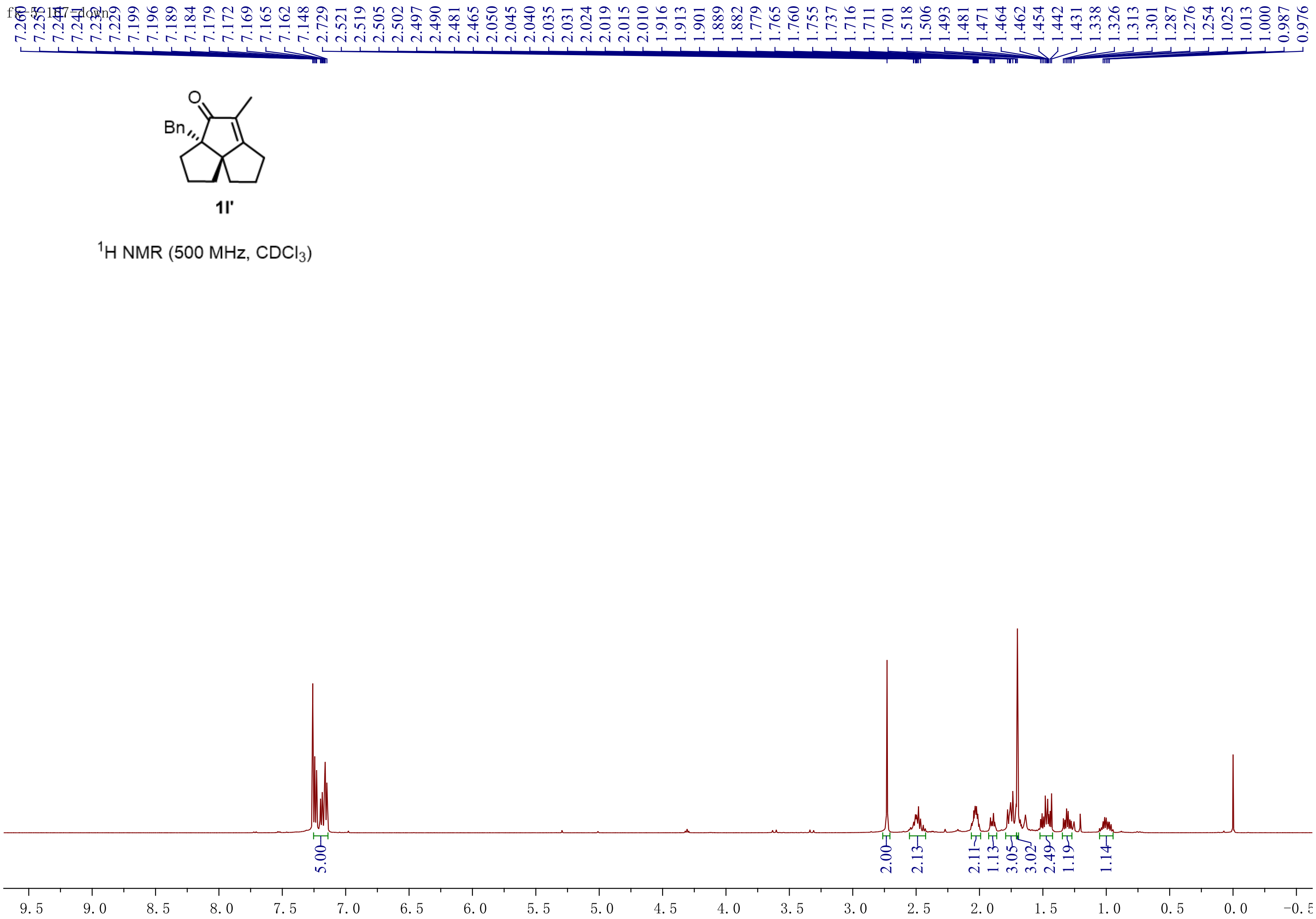

Supplementary Fig. 276. ^{1}H NMR spectra of compound **1l'** in $CDCl_3$

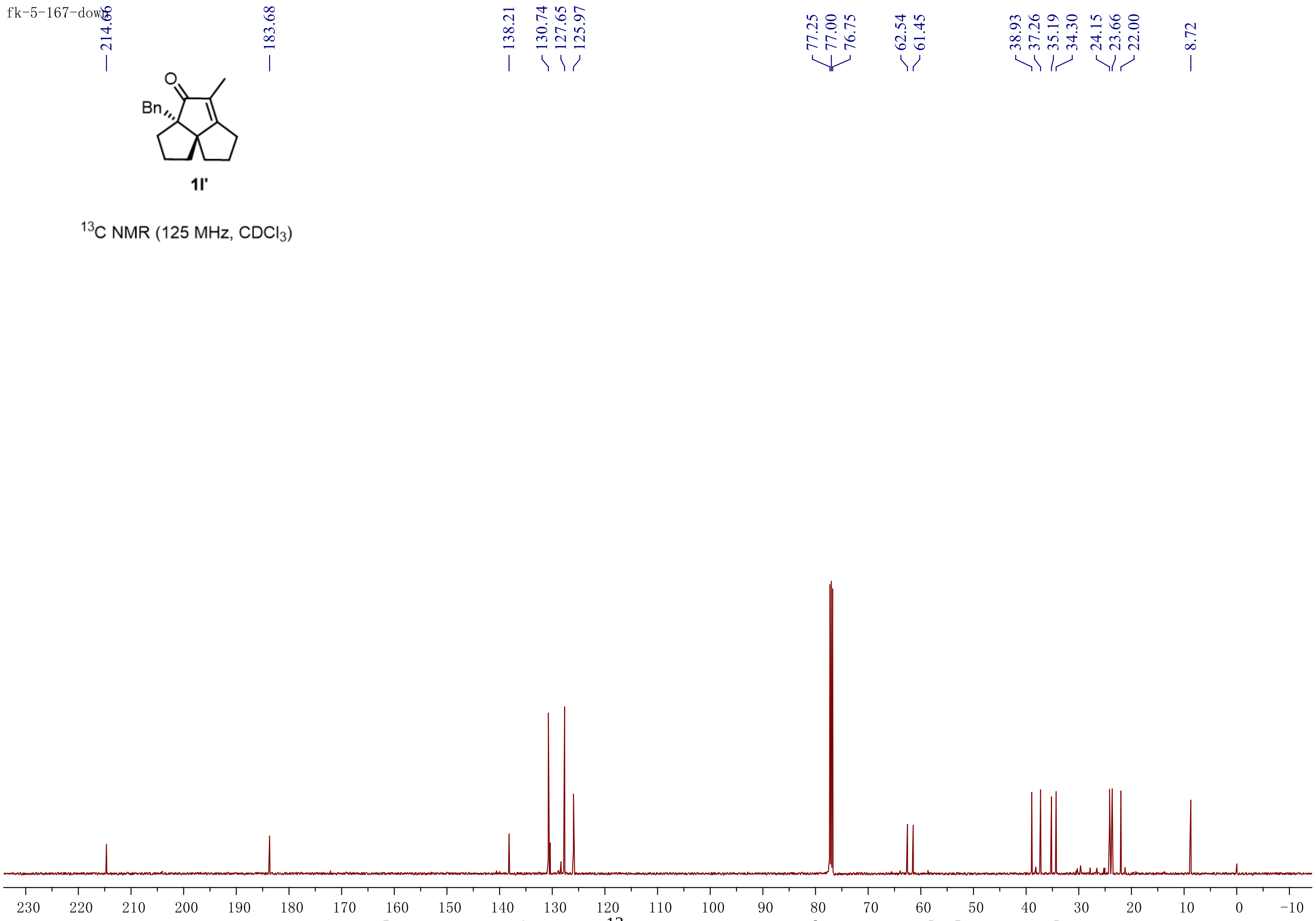

Supplementary Fig. 277. ^{13}C NMR spectra of compound **1l'** in $CDCl_3$

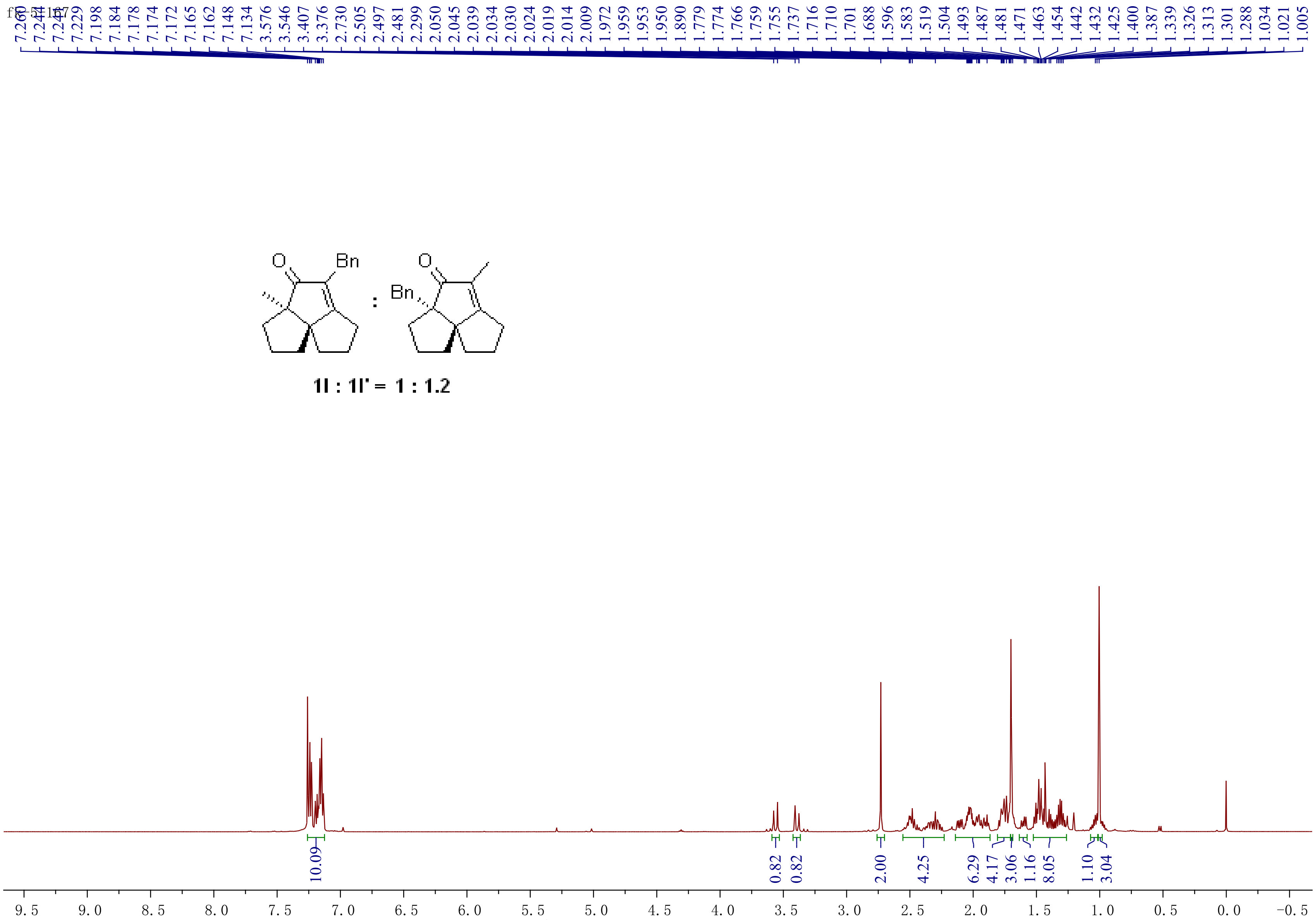

Supplementary Fig. 278. ^{1}H NMR spectra of compound **1l** and**1l'** in $CDCl_3$

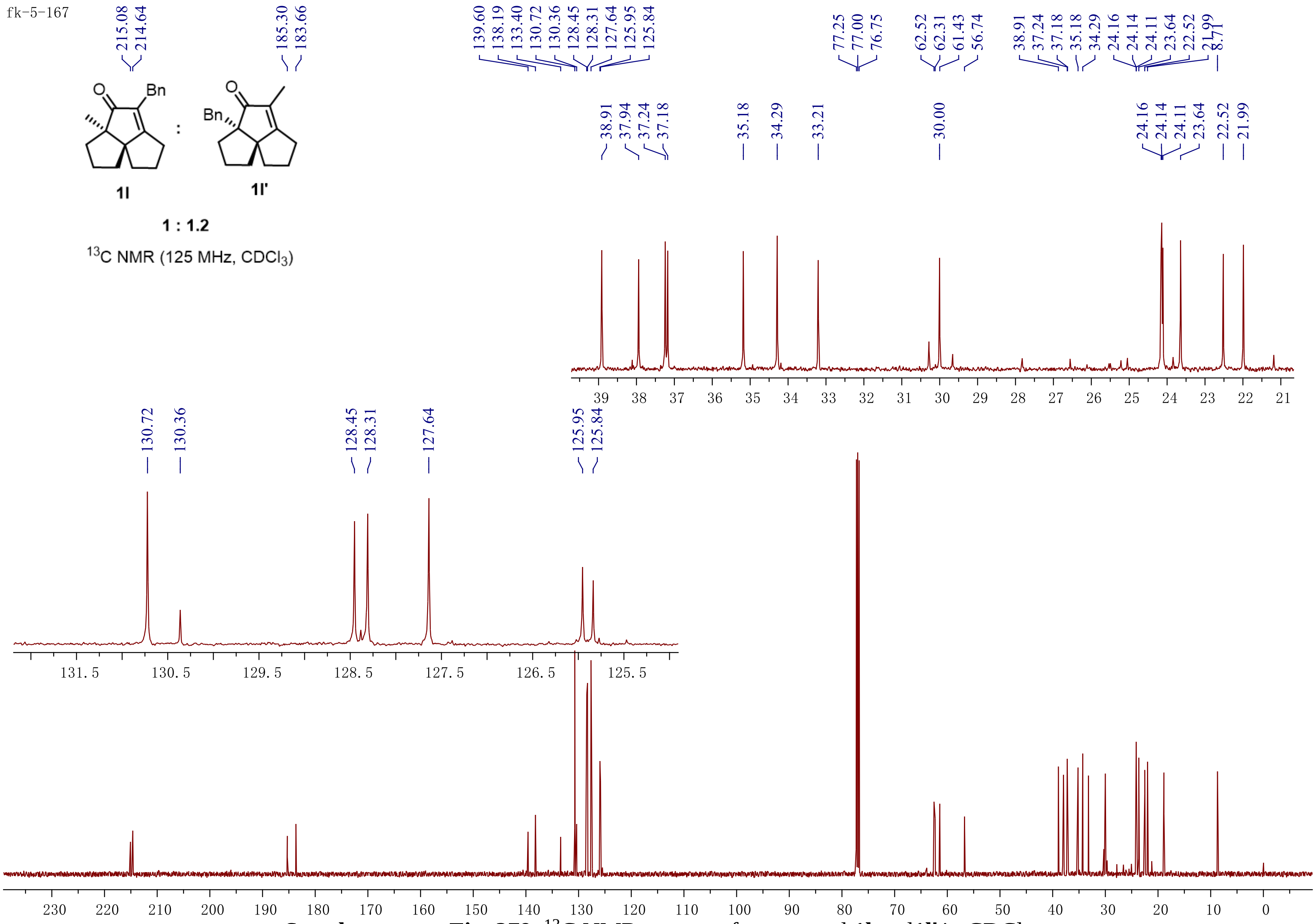

Supplementary Fig. 279. ^{13}C NMR spectra of compound **1l** and**1l'** in $CDCl_3$

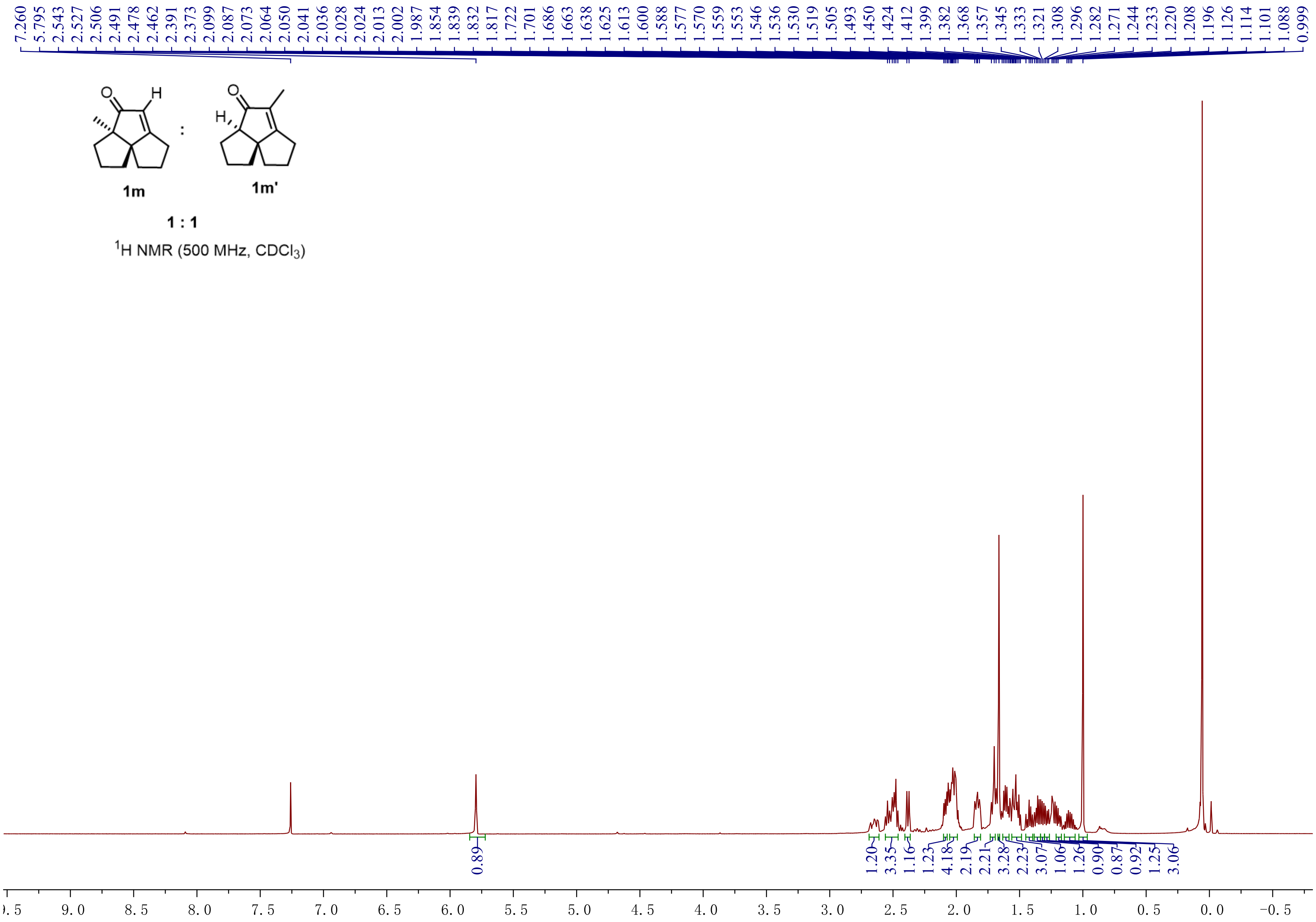

Supplementary Fig. 280. ^{1}H NMR spectra of compound **1m** and**1m'** in $CDCl_3$

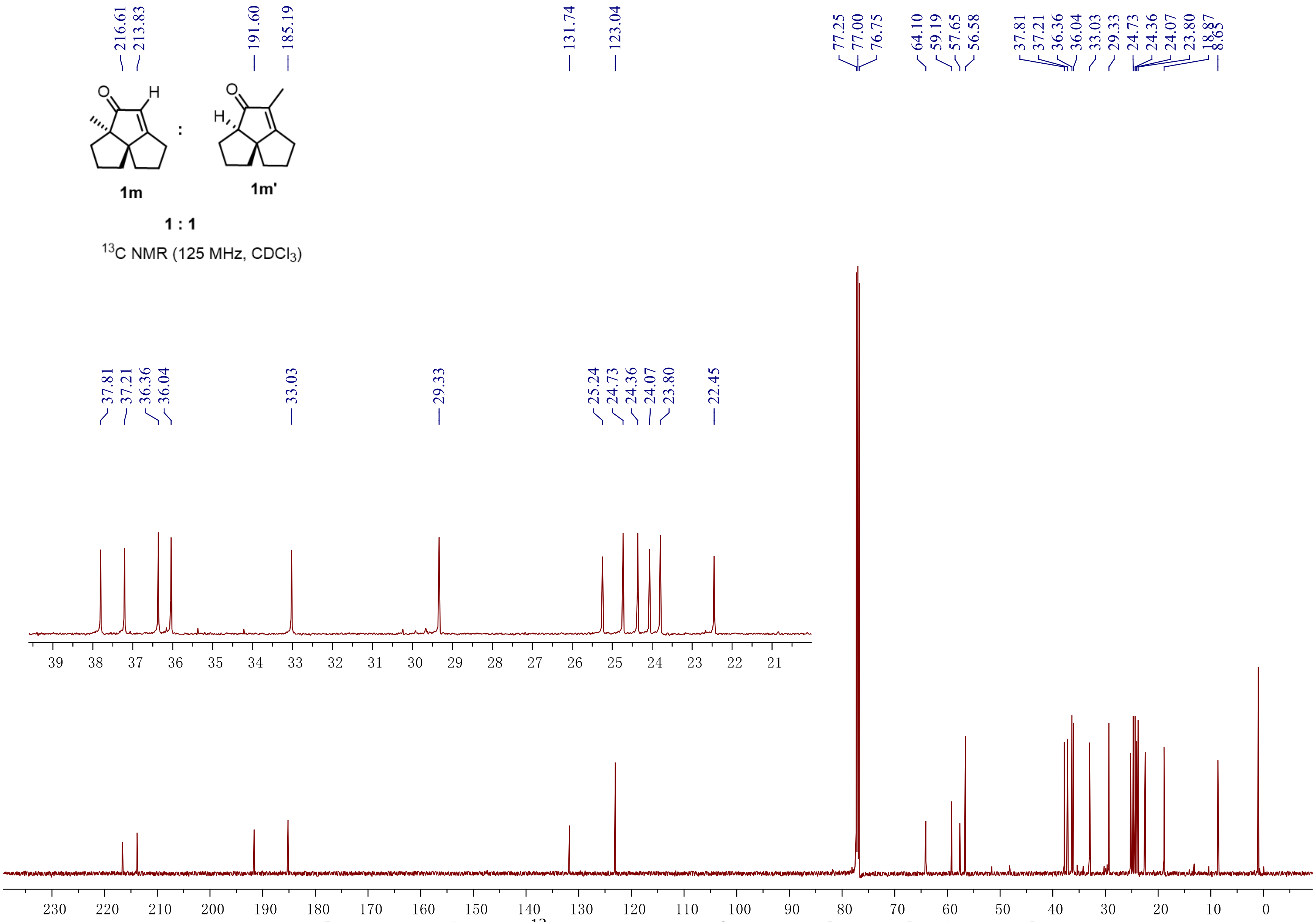

Supplementary Fig. 281. ^{13}C NMR spectra of compound **1m** and**1m'** in $CDCl_3$

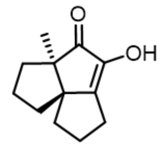

1n

^{1}H NMR (400 MHz, $CDCl_3$)

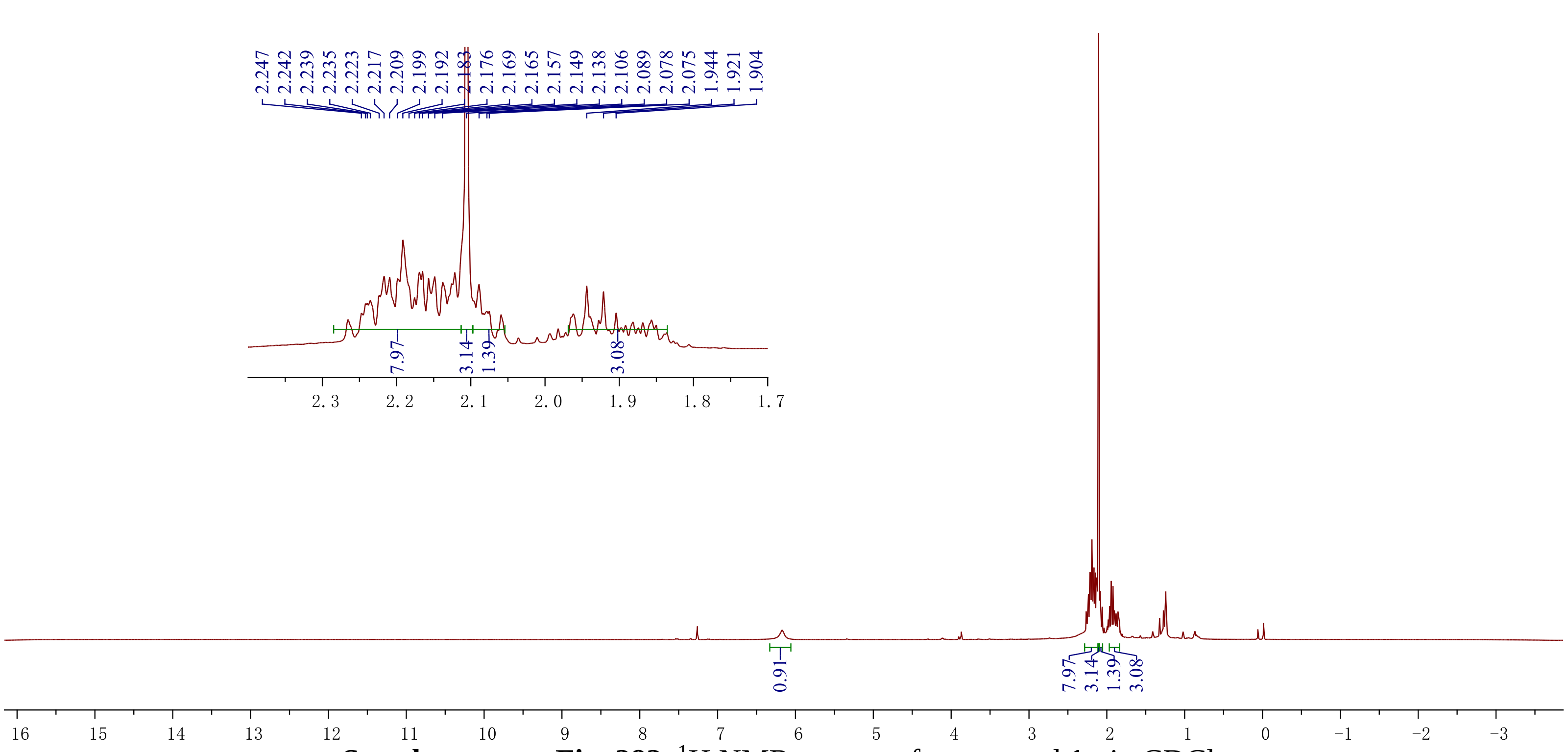

Supplementary Fig. 282. ^{1}H NMR spectra of compound **1n** in $CDCl_3$

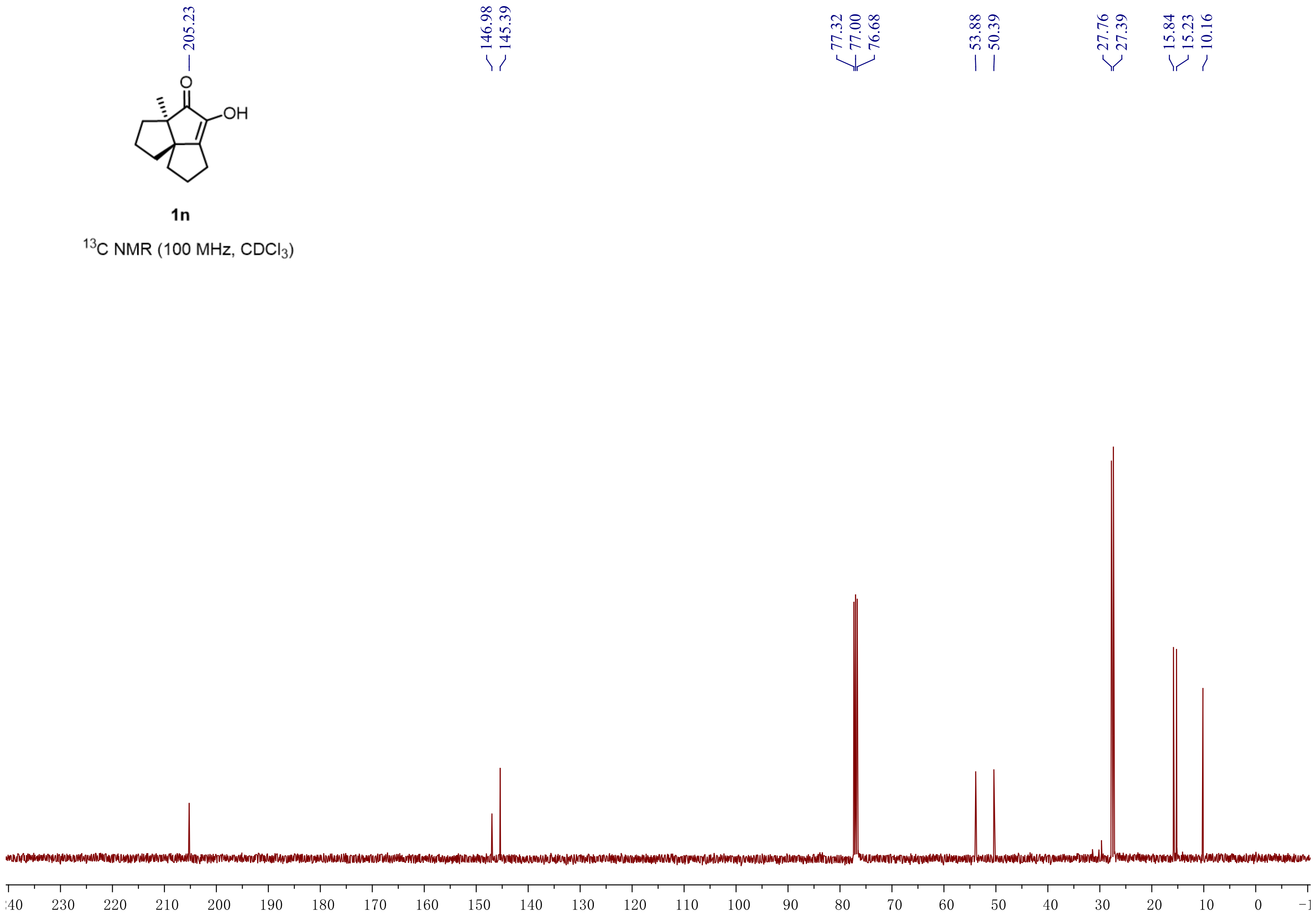

Supplementary Fig. 283. ^{13}C NMR spectra of compound **1n** in $CDCl_3$

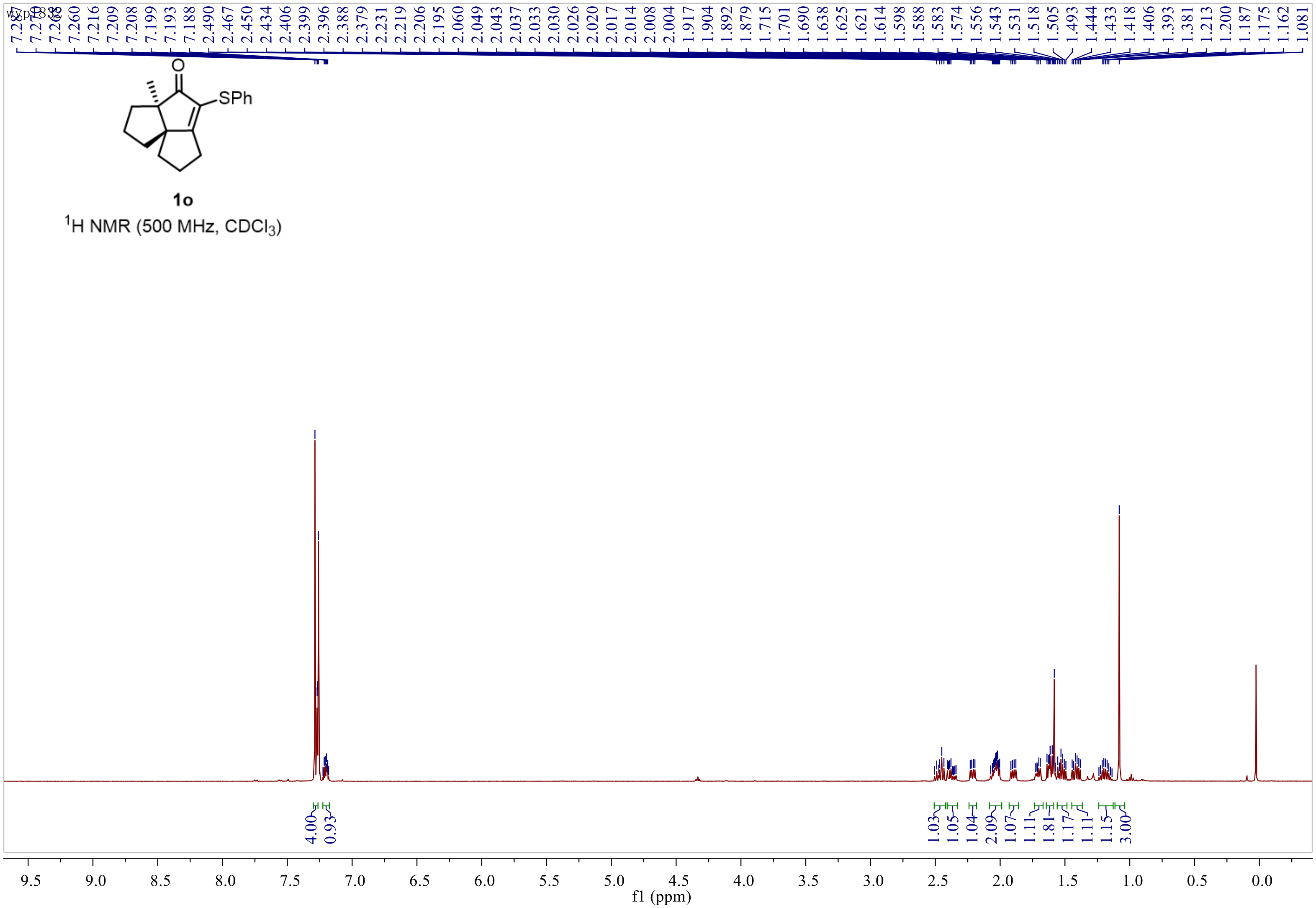

Supplementary Fig. 284. ^{1}H NMR spectra of compound **1o** in $CDCl_3$

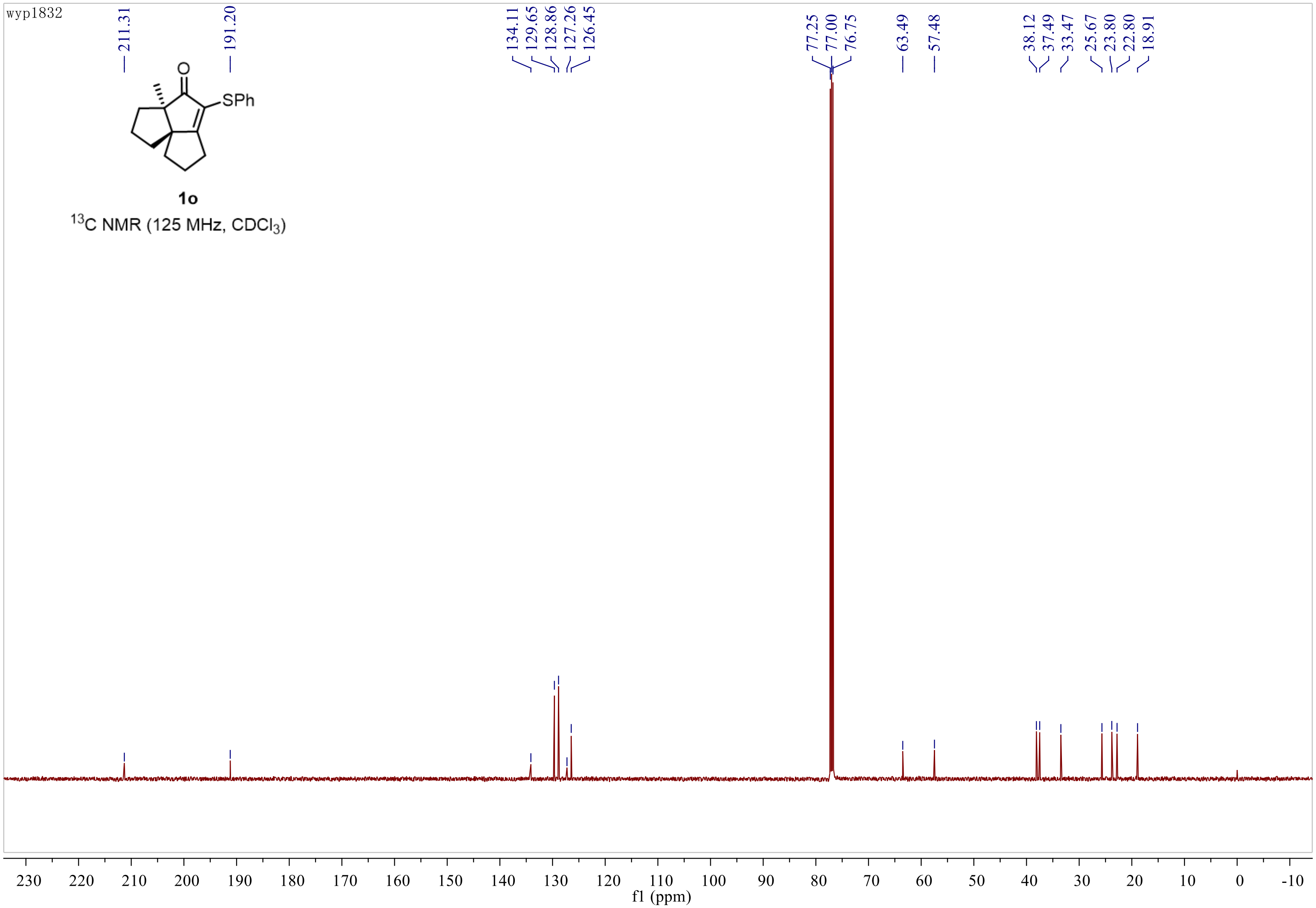

Supplementary Fig. 285. ^{13}C NMR spectra of compound **1o** in $CDCl_3$

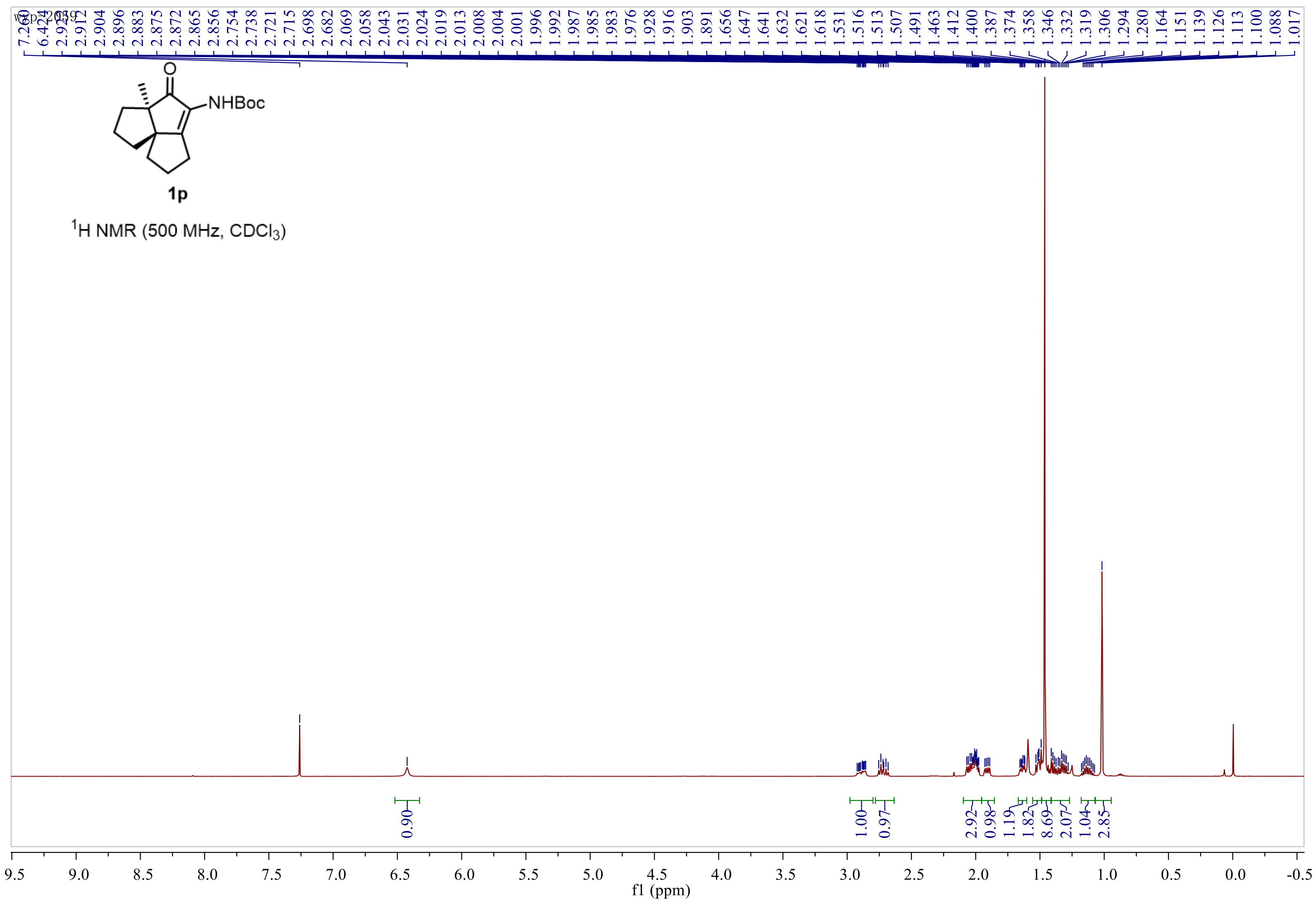

Supplementary Fig. 286. ^{1}H NMR spectra of compound **1p** in $CDCl_3$

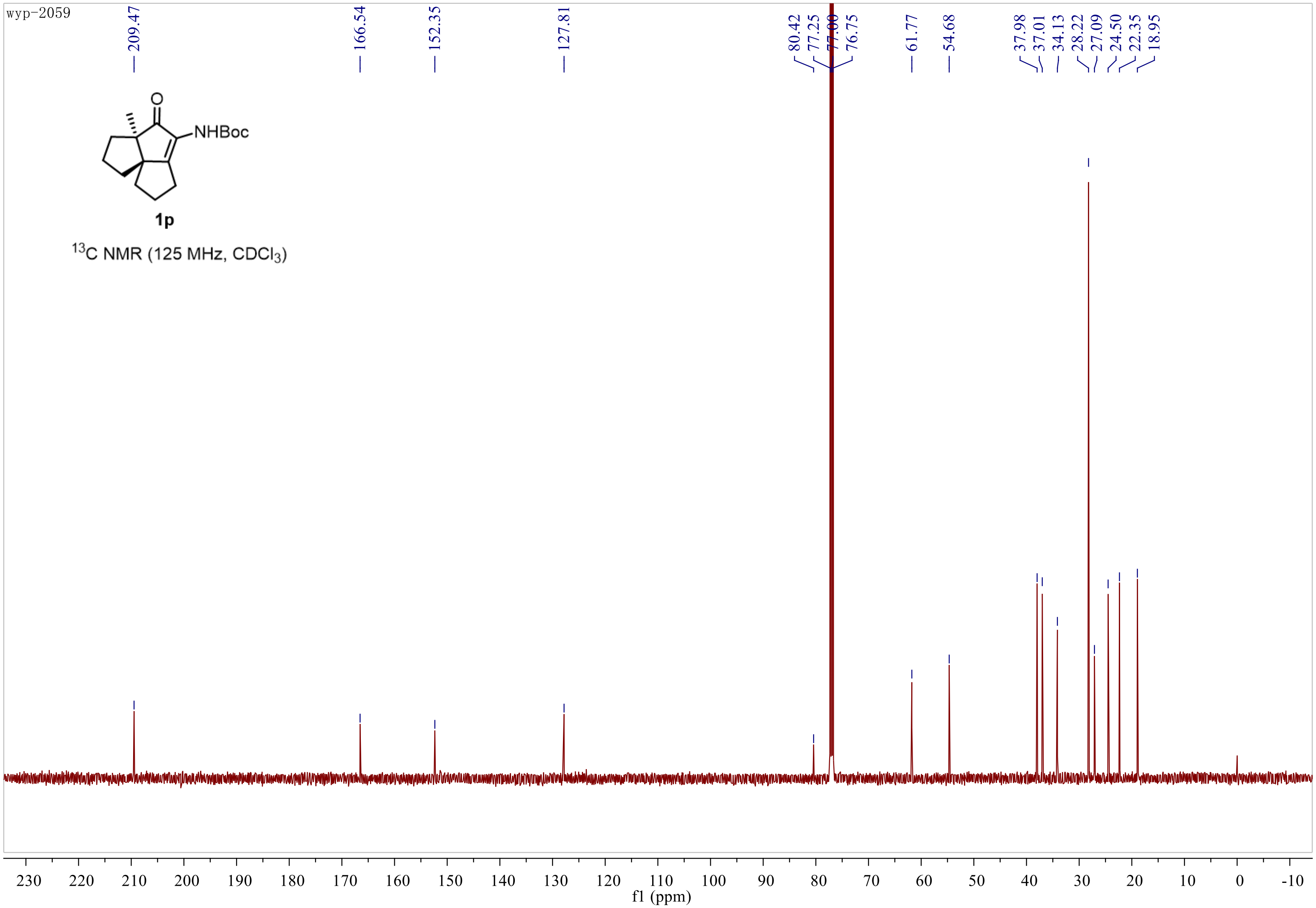

Supplementary Fig. 287. ^{13}C NMR spectra of compound **1p** in $CDCl_3$

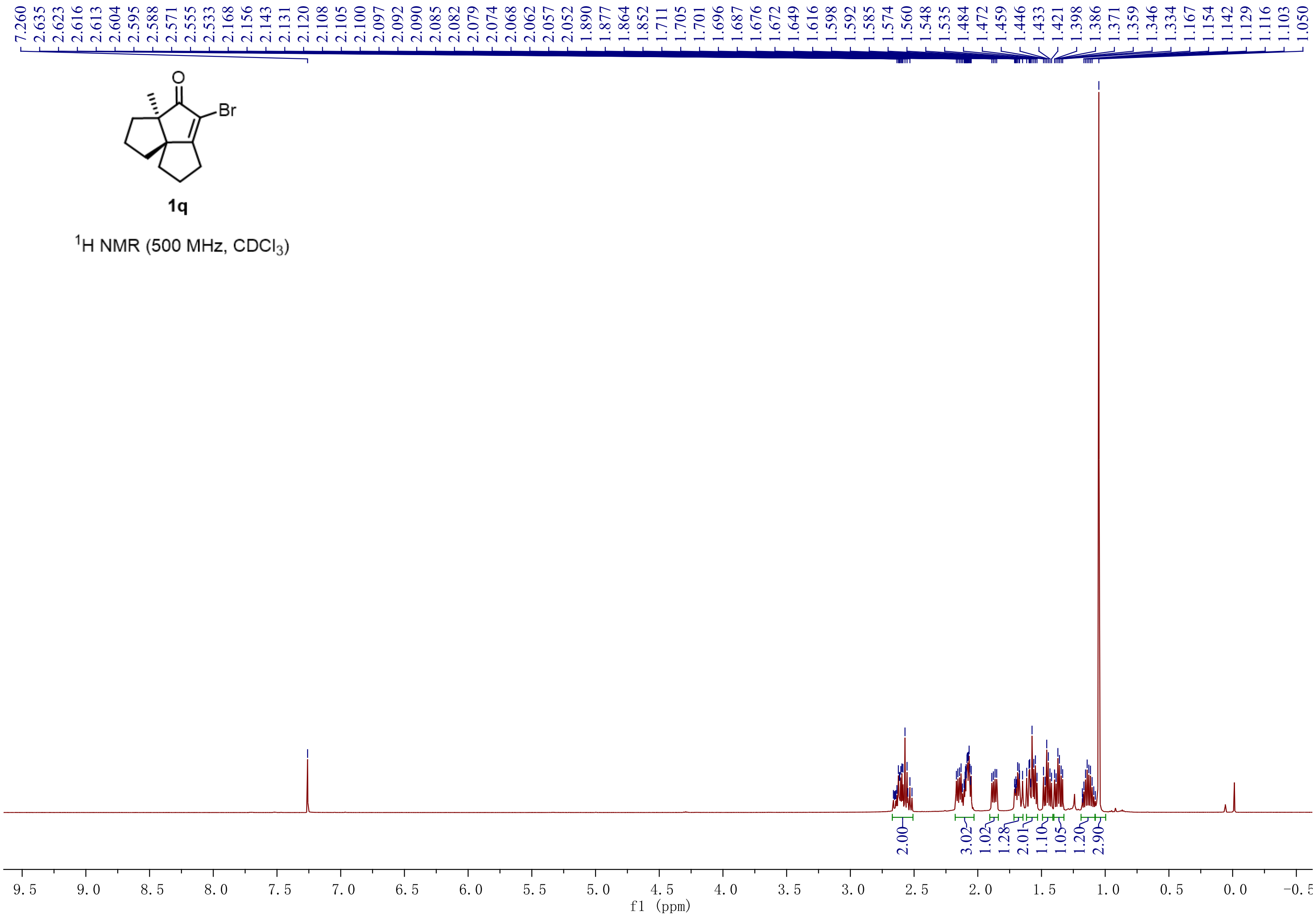

Supplementary Fig. 288. ^{1}H NMR spectra of compound **1q** in $CDCl_3$

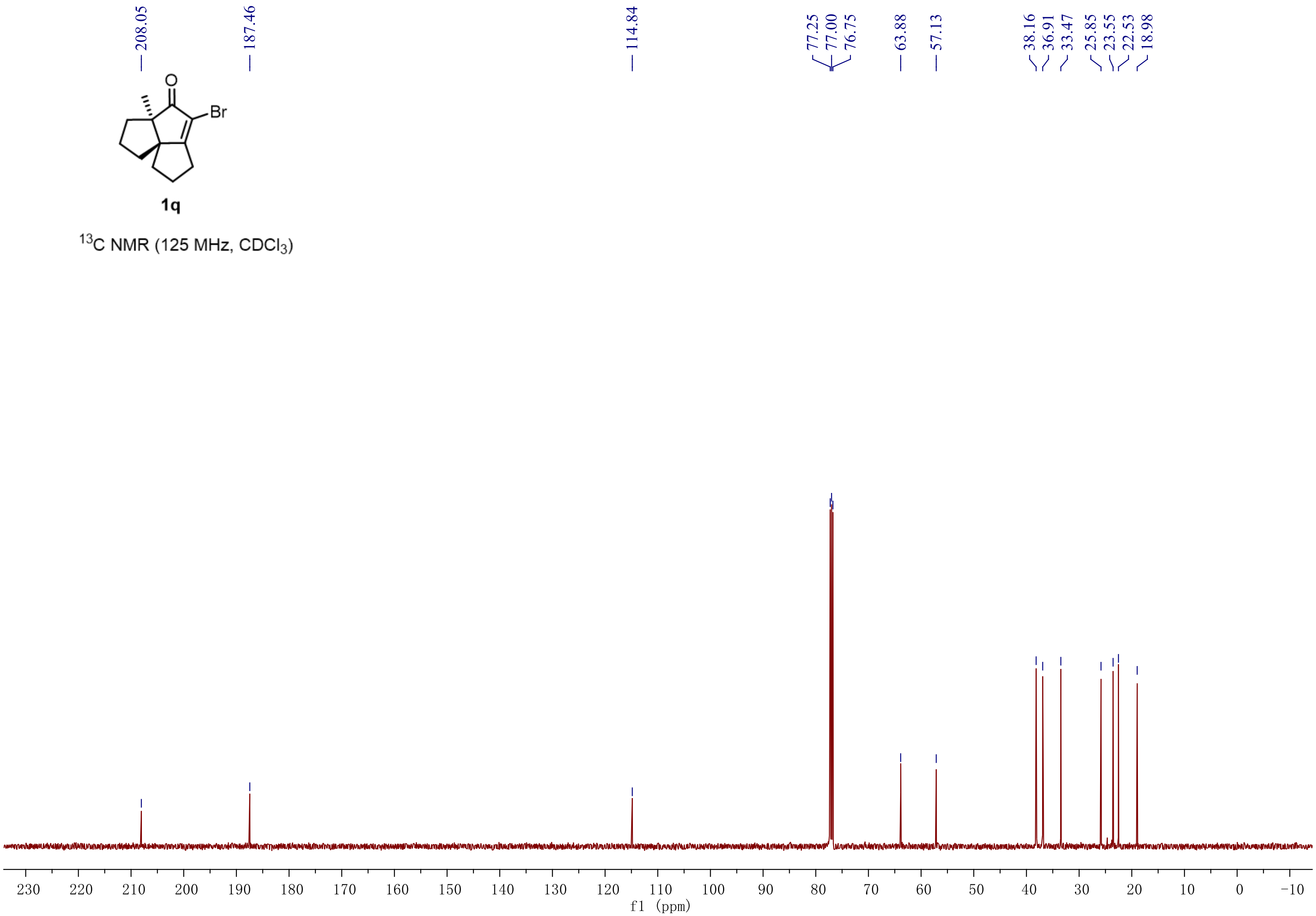

Supplementary Fig. 289. ^{13}C NMR spectra of compound **1q** in $CDCl_3$

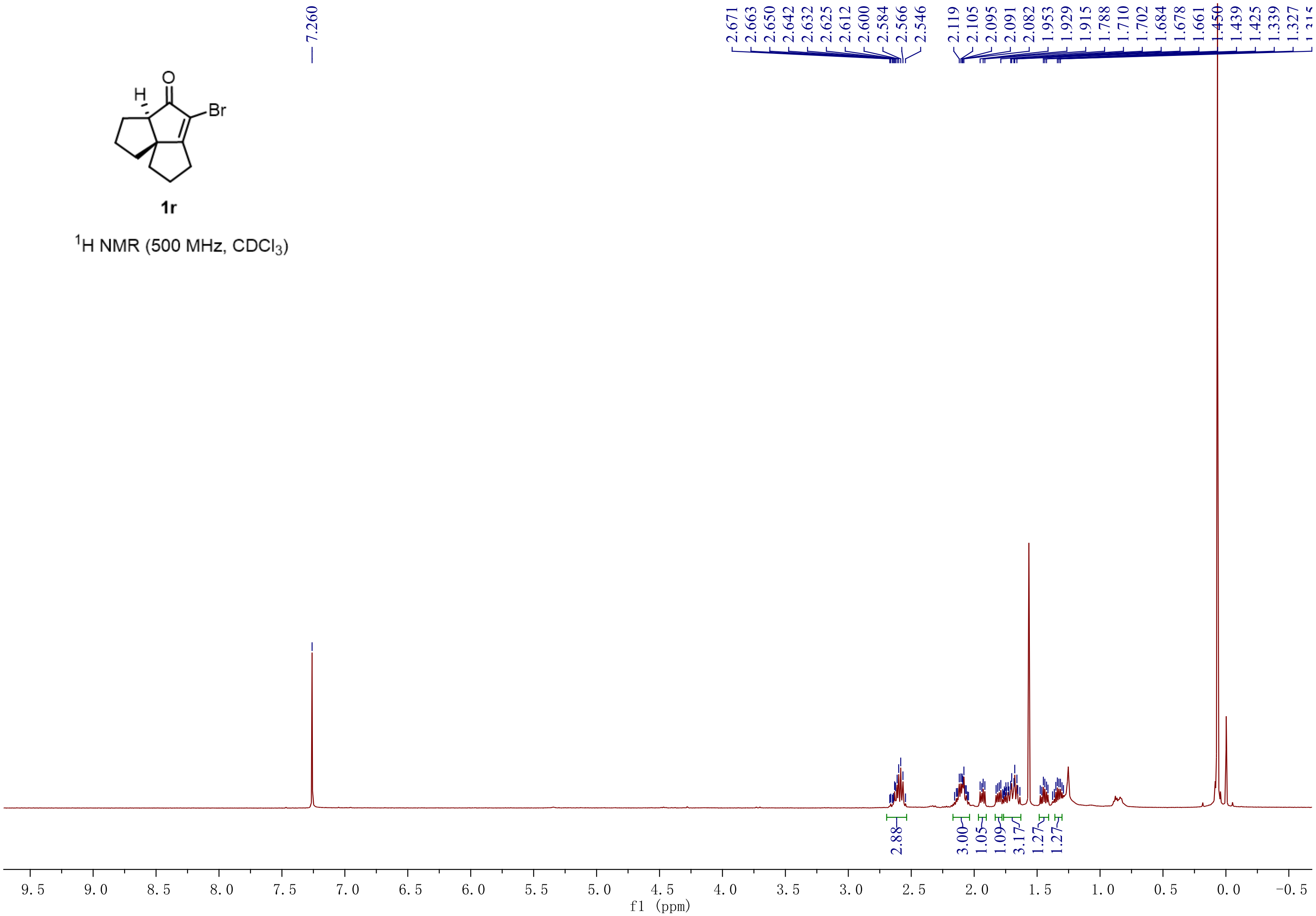

Supplementary Fig. 290. ^{1}H NMR spectra of compound **1r** in $CDCl_3$

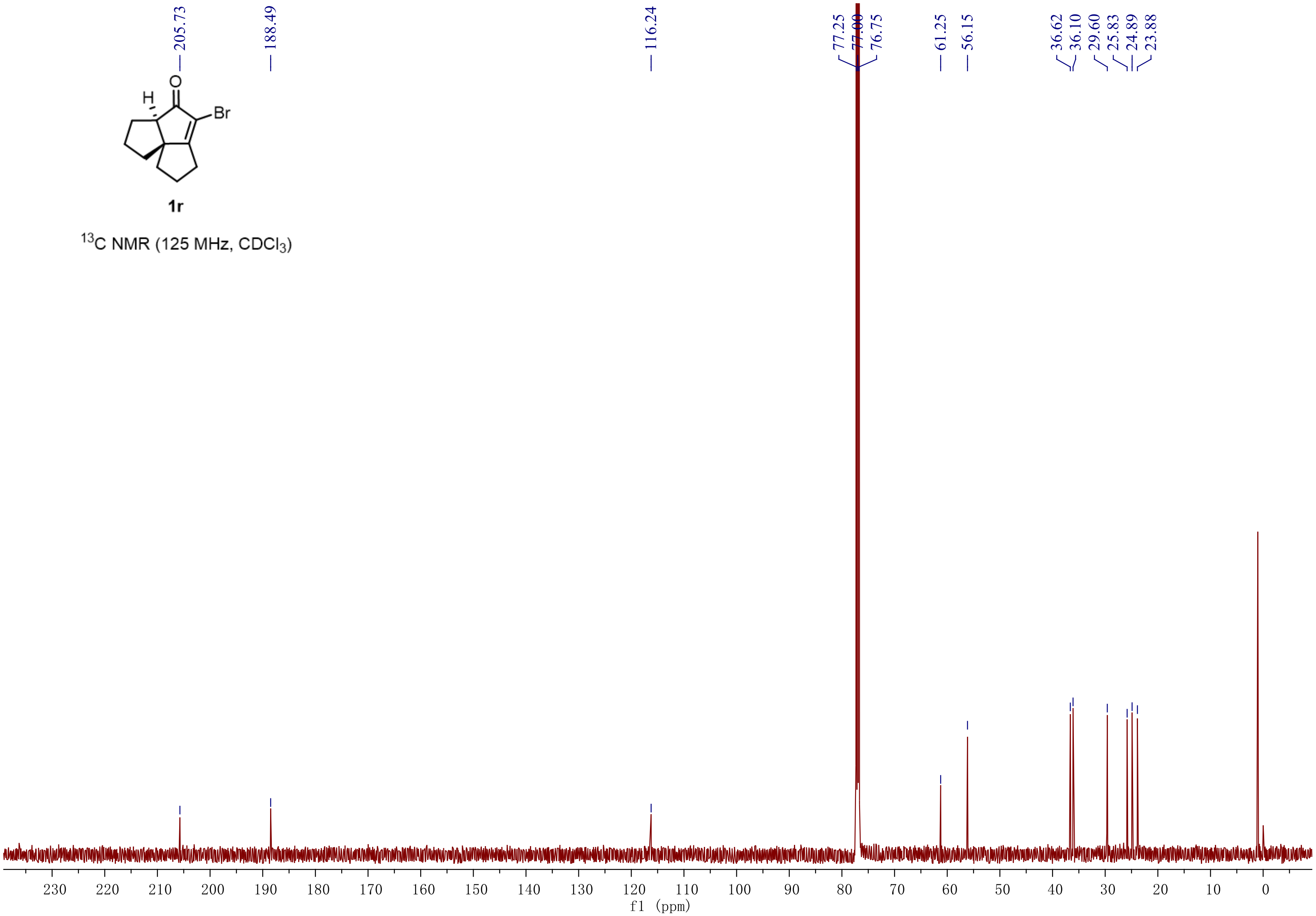

Supplementary Fig. 291. ^{13}C NMR spectra of compound **1r** in $CDCl_3$

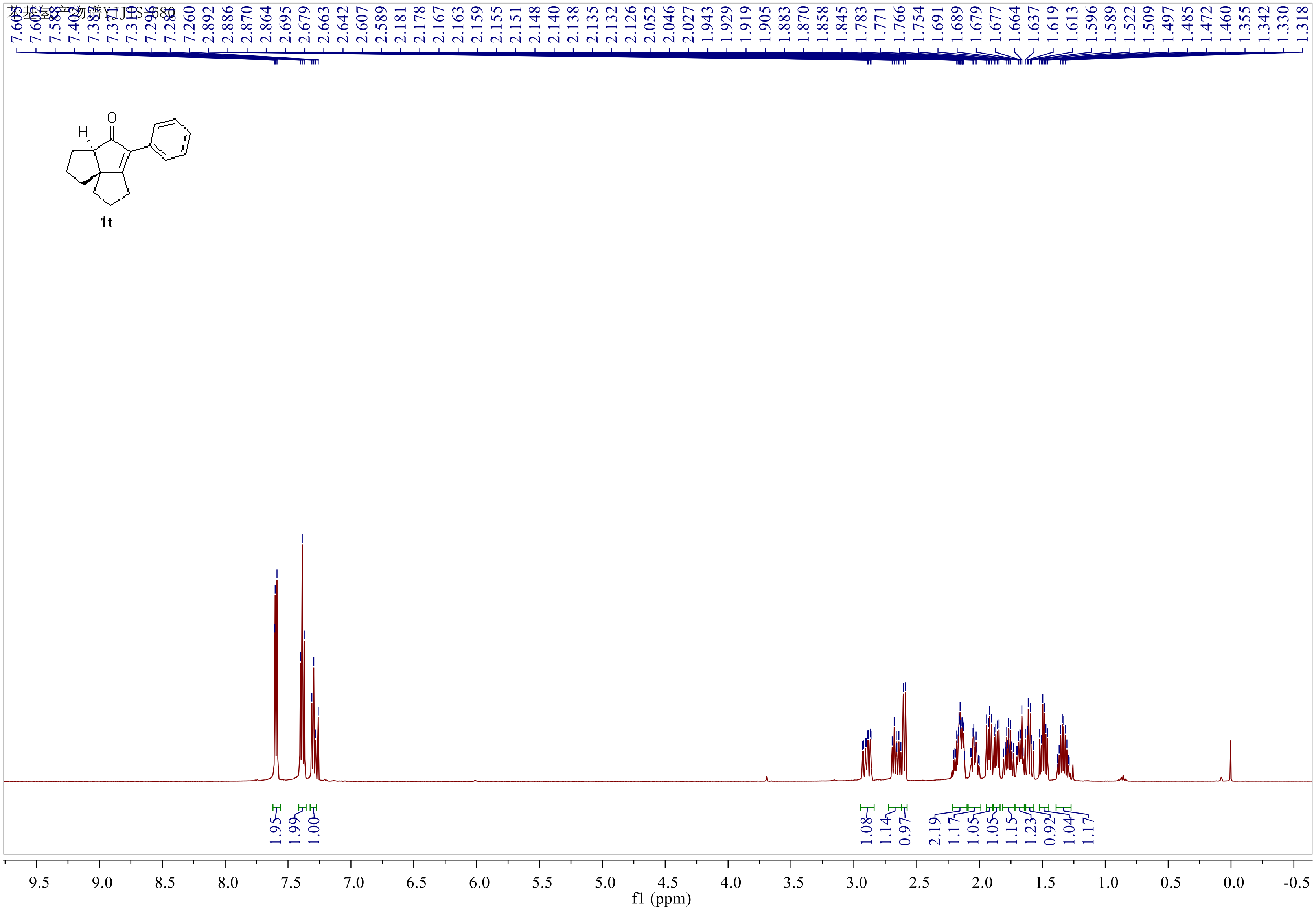

Supplementary Fig. 292. ^{1}H NMR spectra of compound **1t** in $CDCl_3$

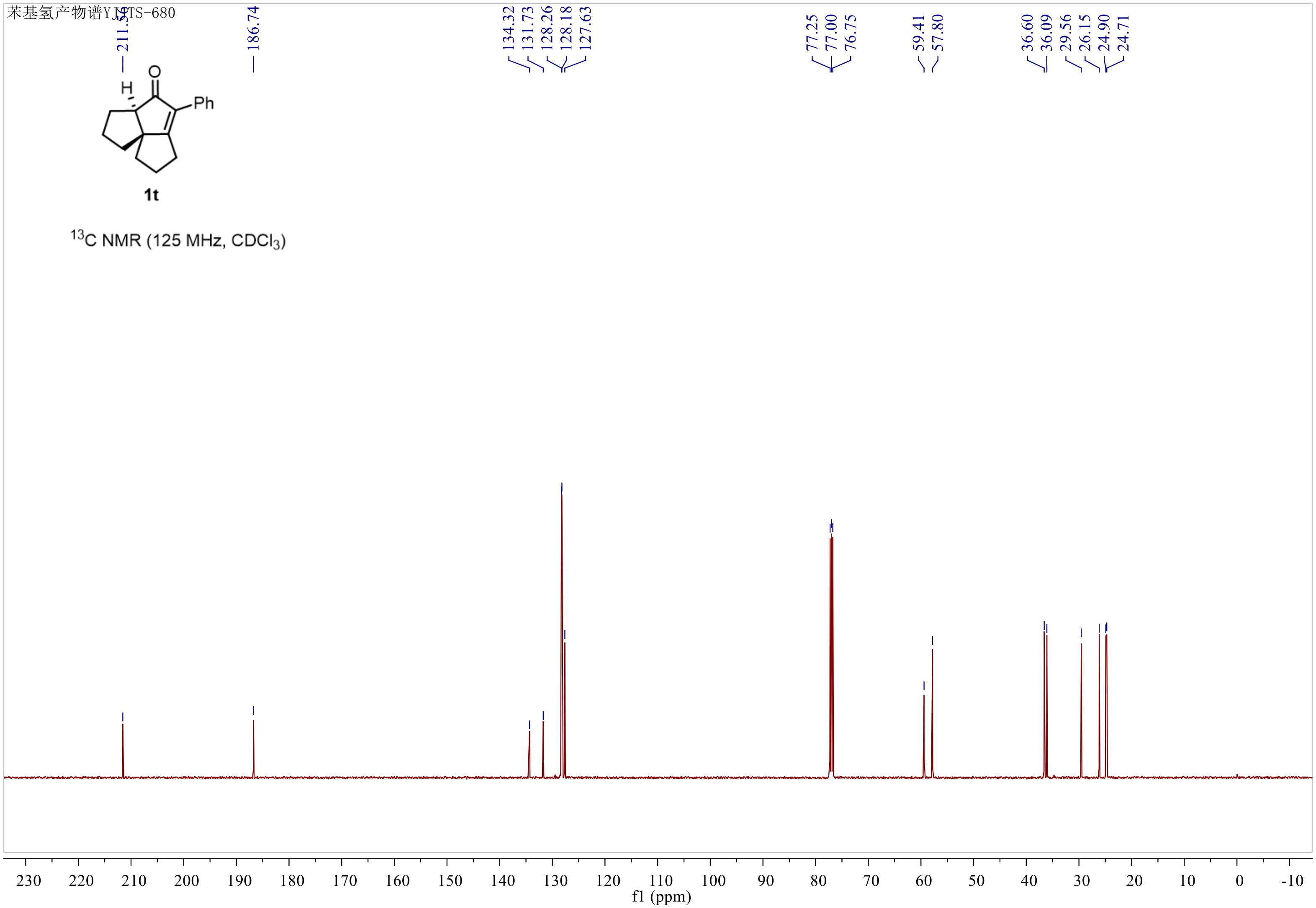

Supplementary Fig. 293. ^{13}C NMR spectra of compound **1t** in $CDCl_3$

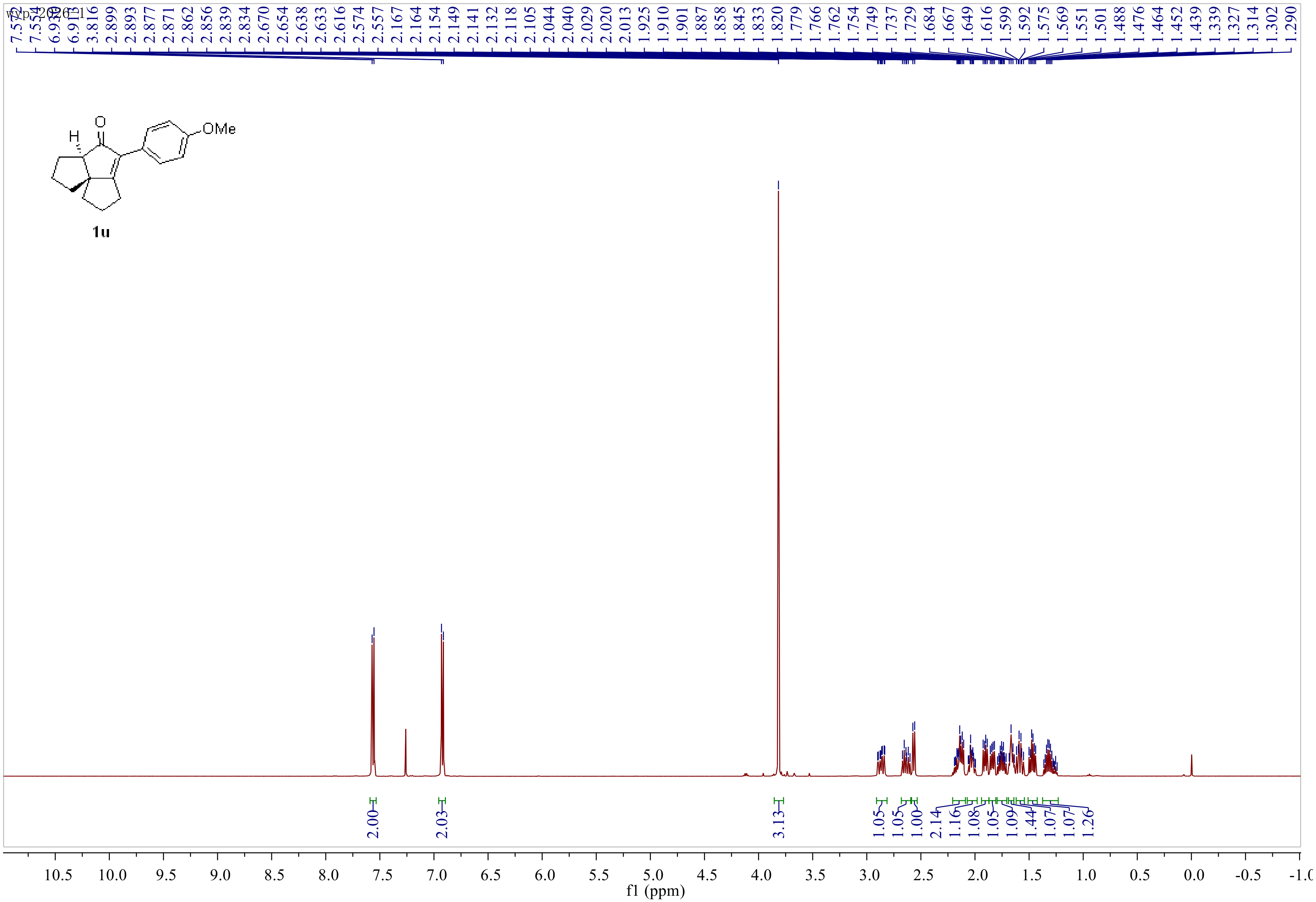

Supplementary Fig. 294. ^{1}H NMR spectra of compound **1u** in $CDCl_3$

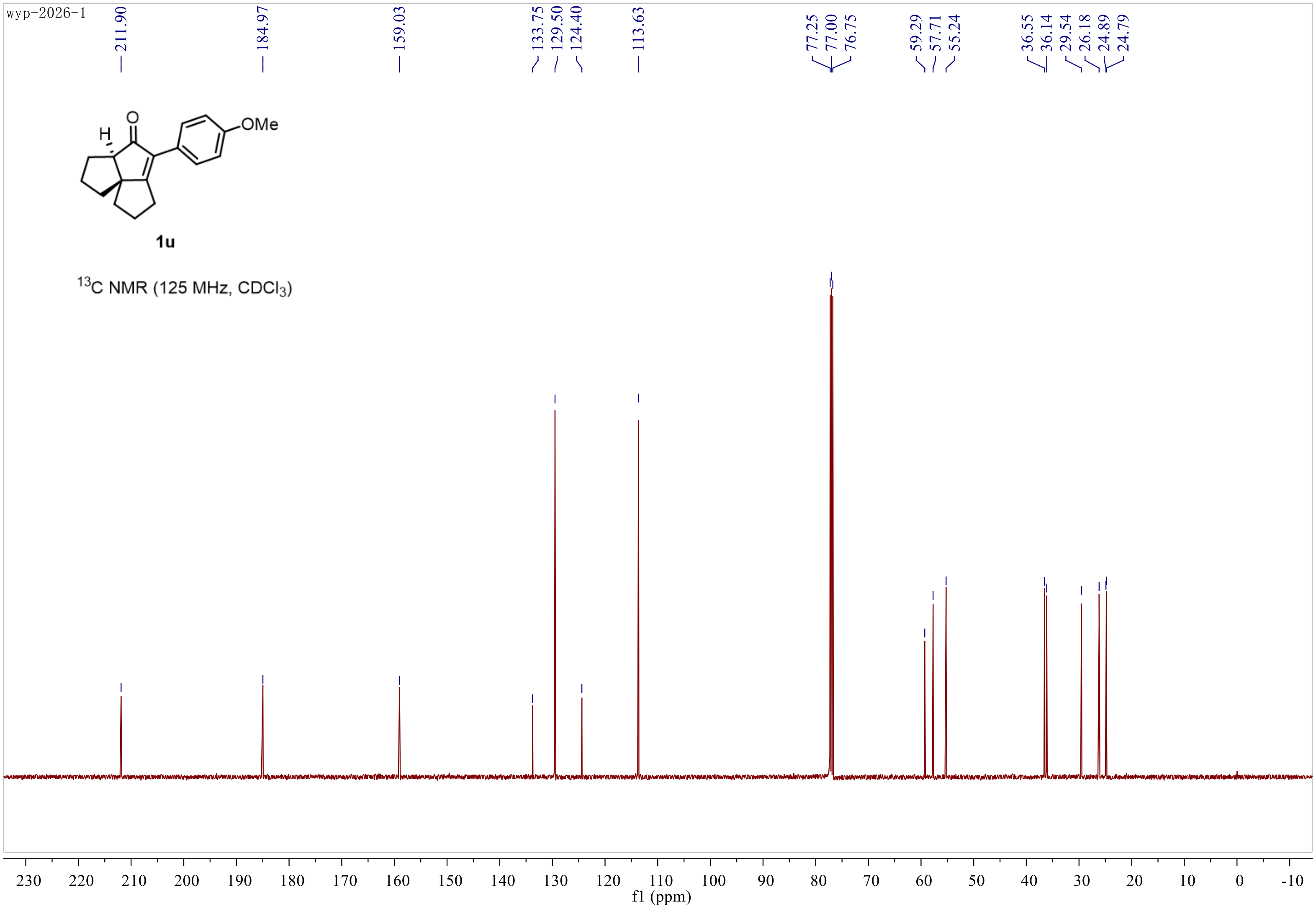

Supplementary Fig. 295. ^{13}C NMR spectra of compound **1u** in $CDCl_3$

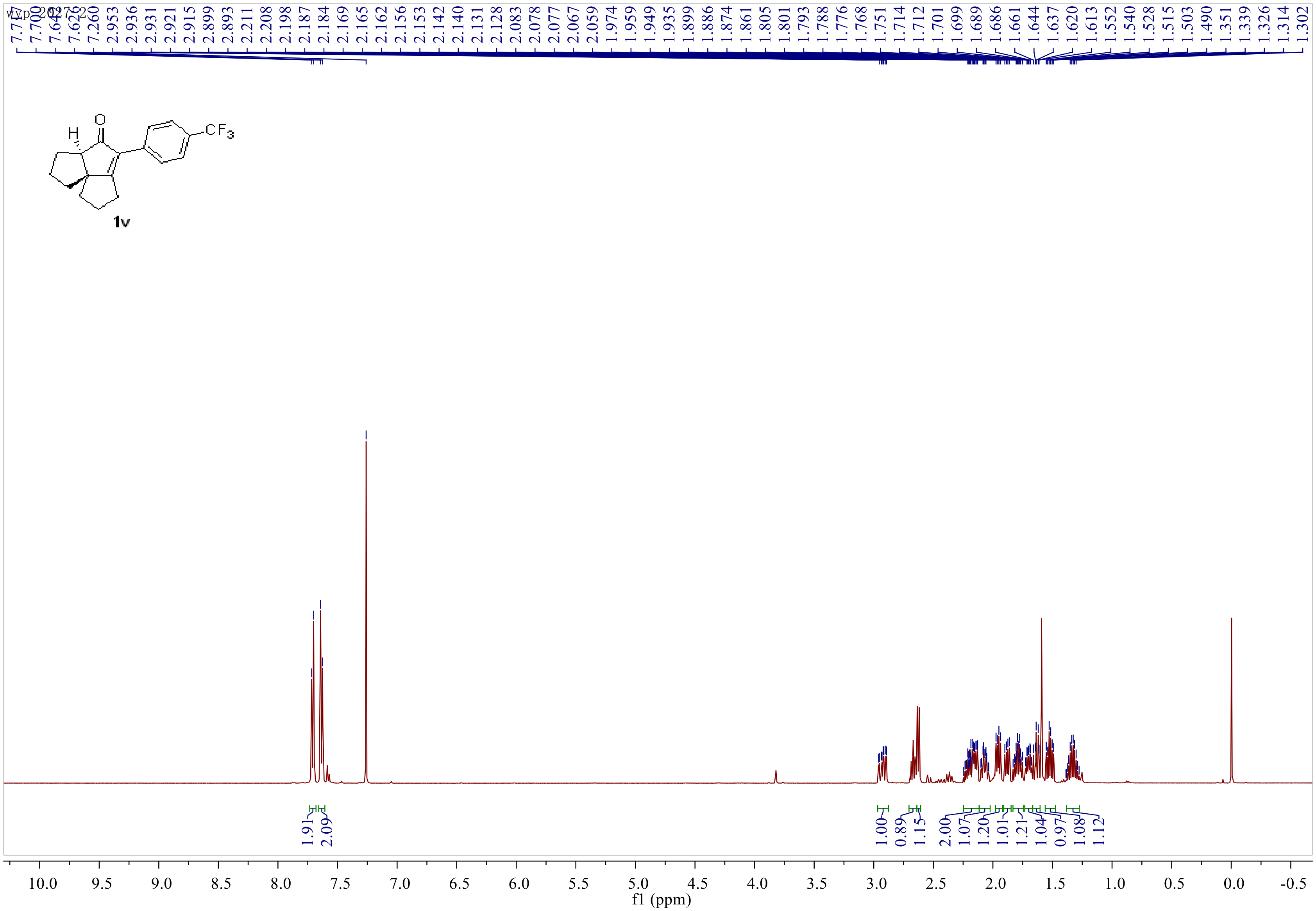

Supplementary Fig. 296. ^{1}H NMR spectra of compound **1v** in $CDCl_3$

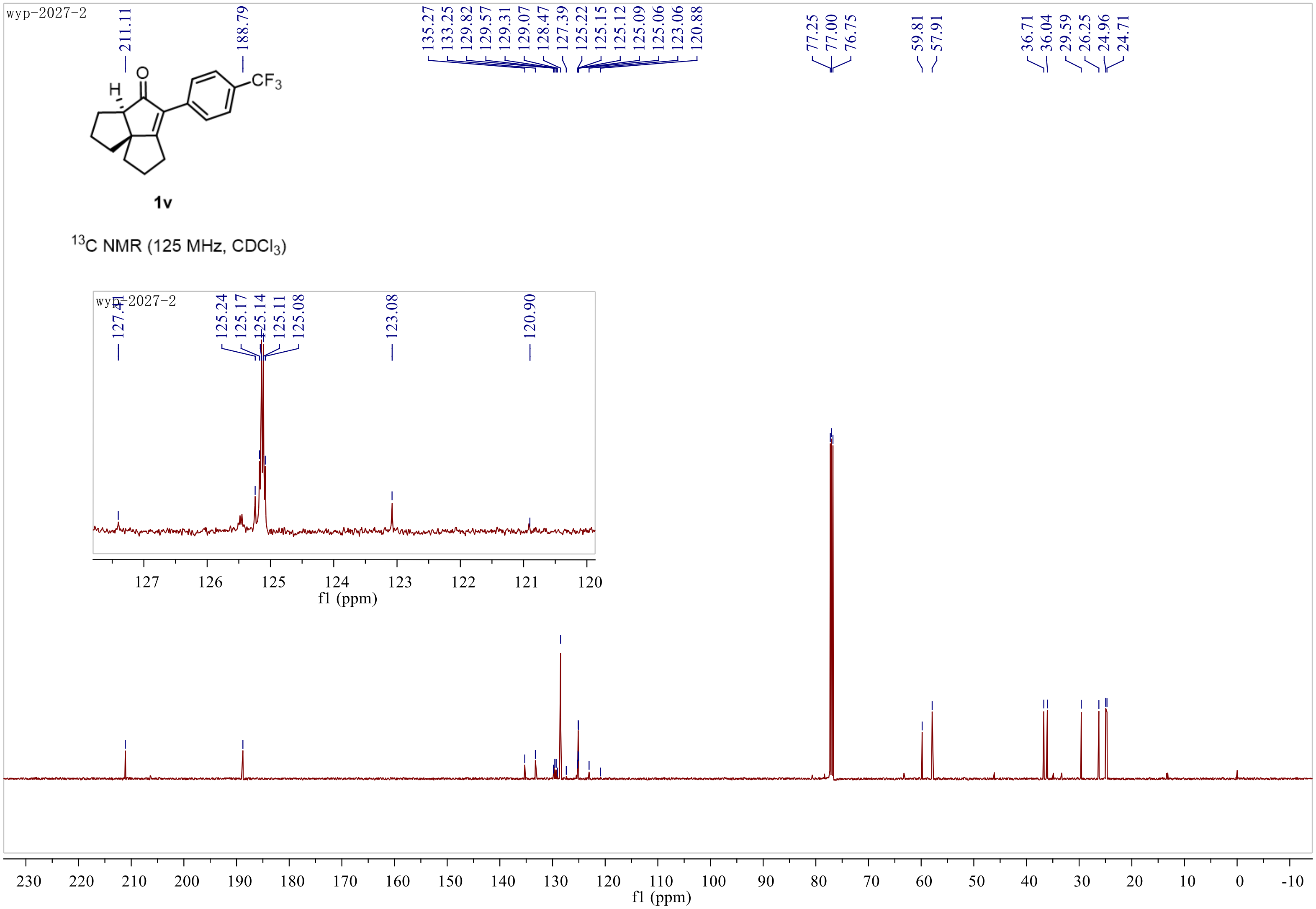

Supplementary Fig. 297. ^{13}C NMR spectra of compound **1v** in $CDCl_3$

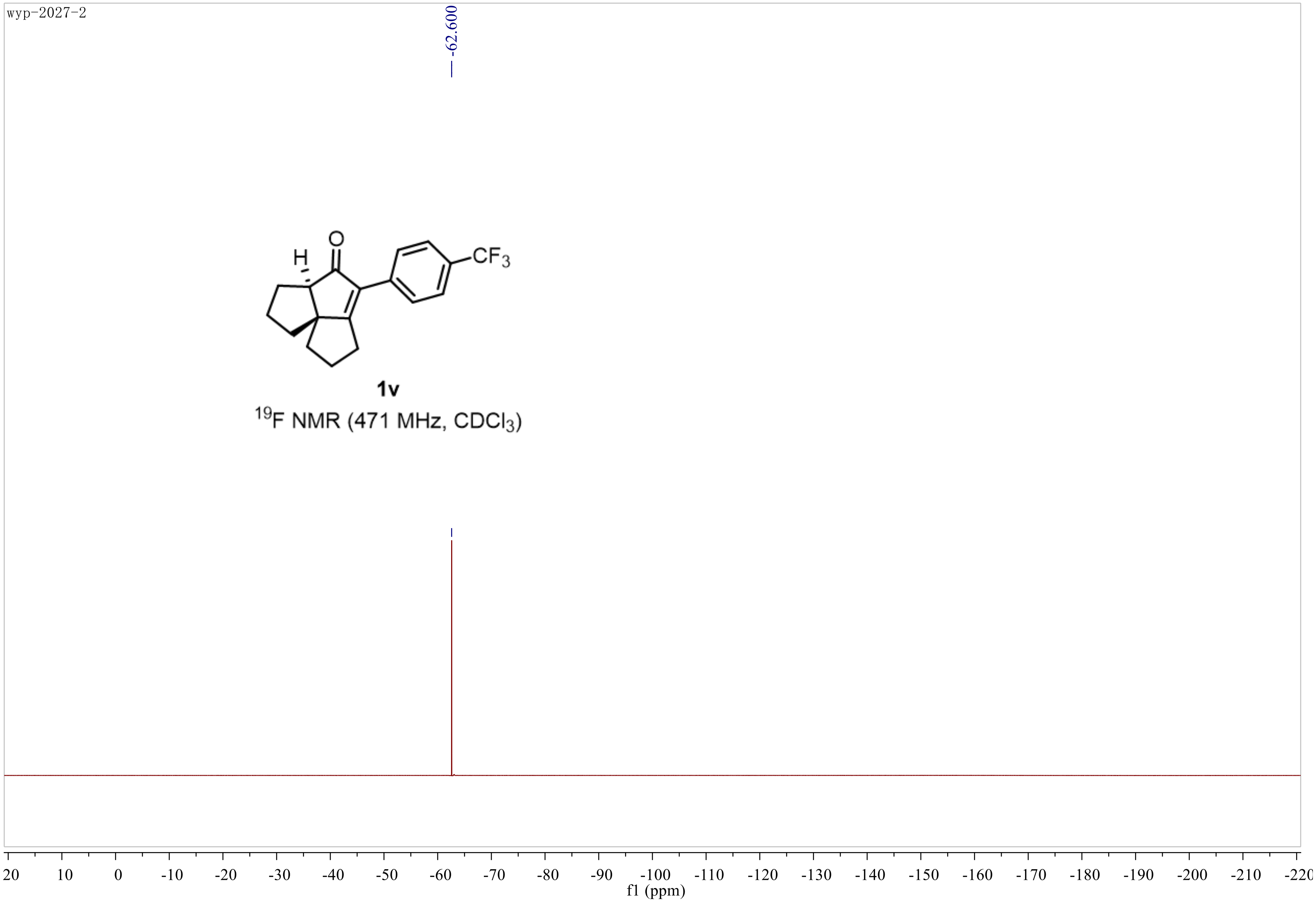

Supplementary Fig. 298. ^{19}F NMR spectra of compound **1v** in $CDCl_3$

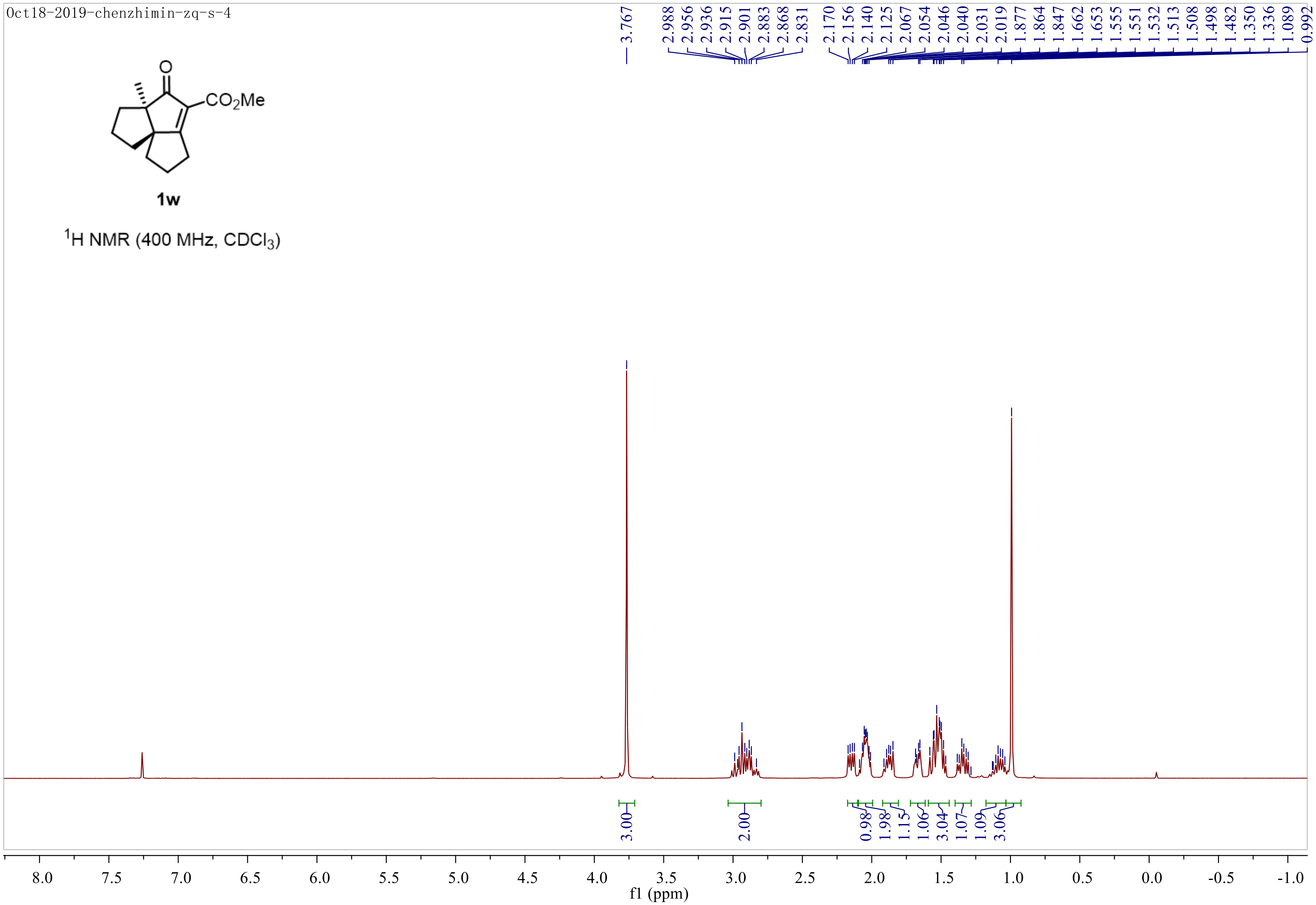

Supplementary Fig. 299. ^{1}H NMR spectra of compound **1w** in $CDCl_3$

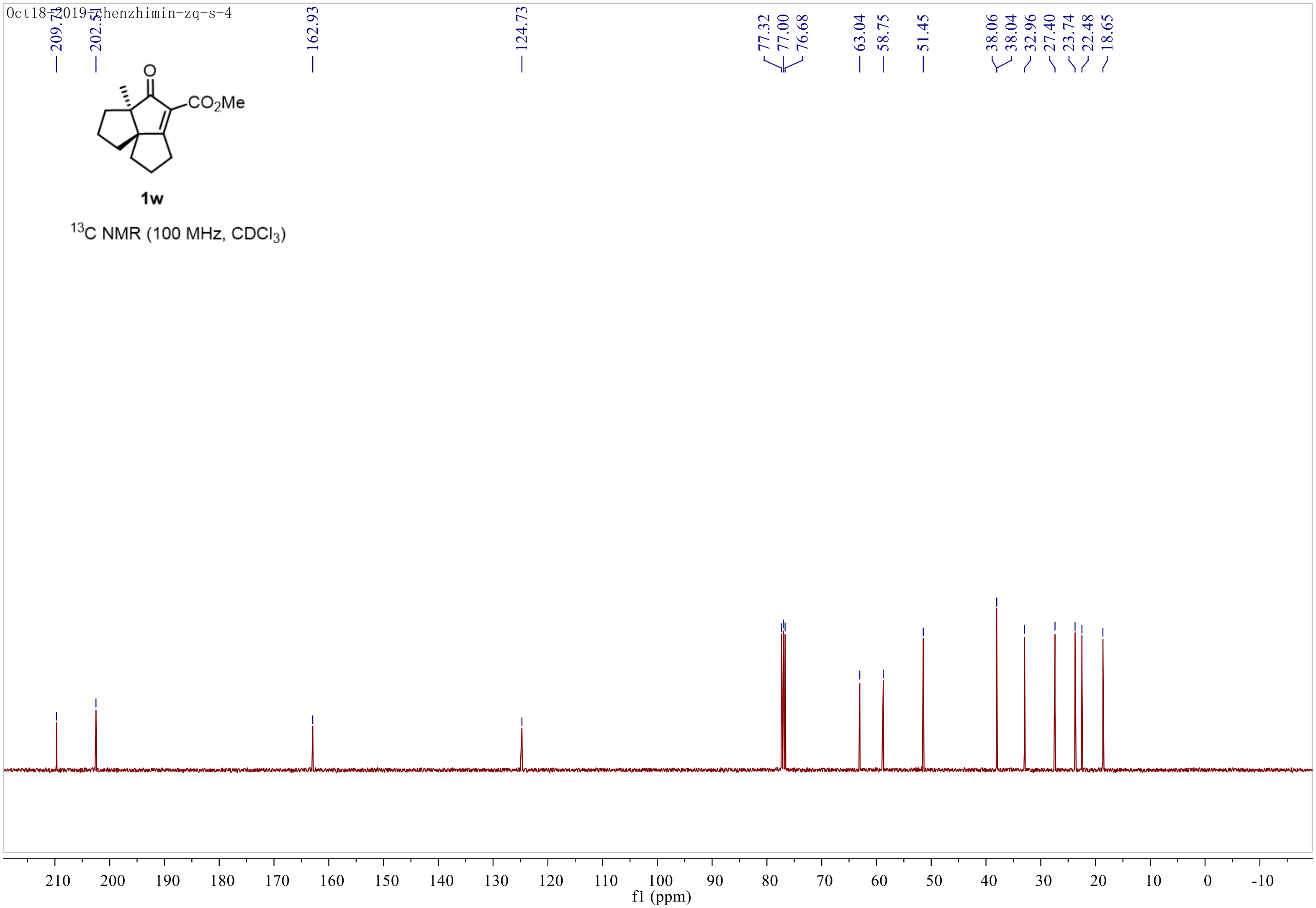

Supplementary Fig. 300. ^{13}C NMR spectra of compound **1w** in $CDCl_3$

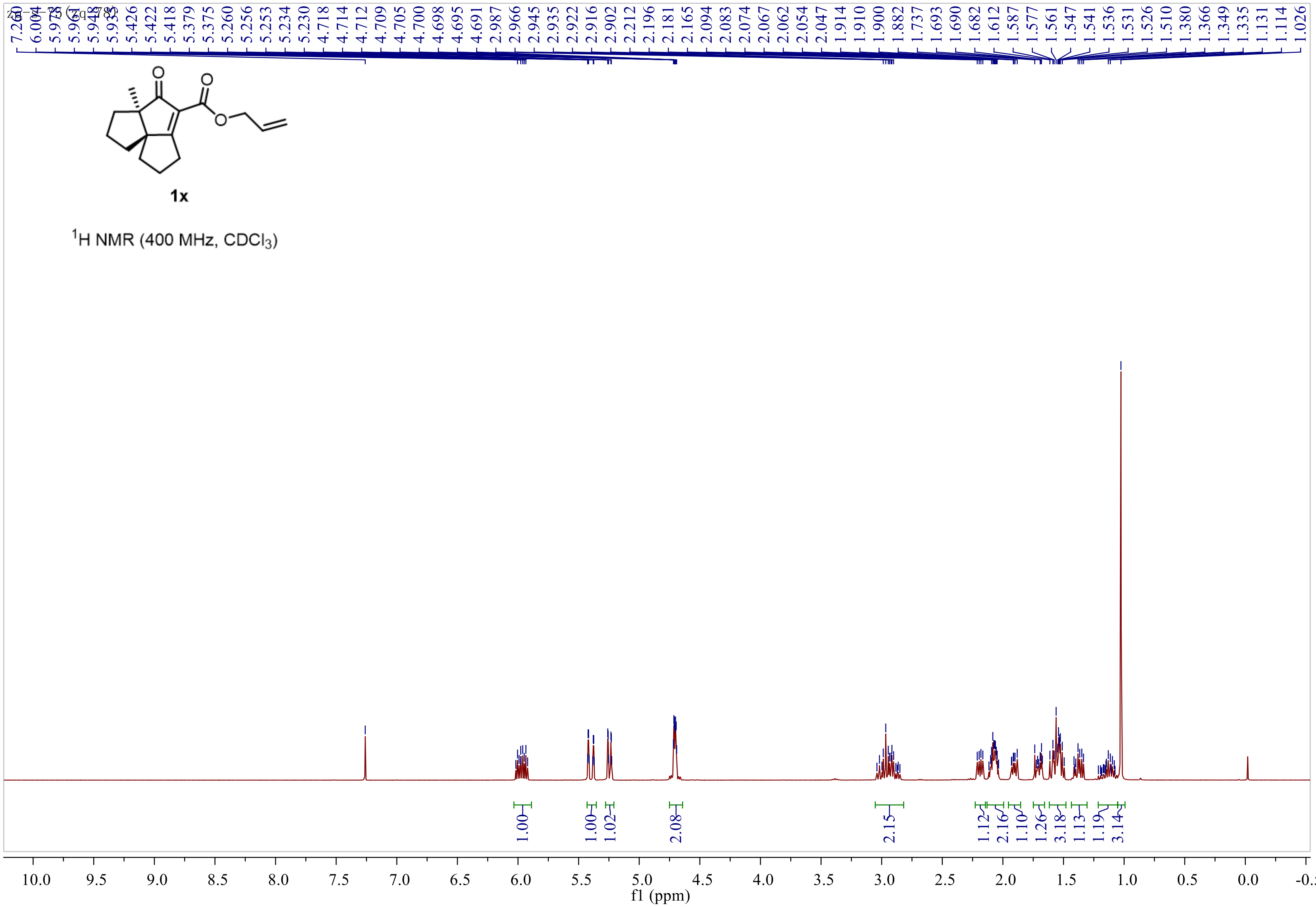

Supplementary Fig. 301. ^{1}H NMR spectra of compound **1x** in $CDCl_3$

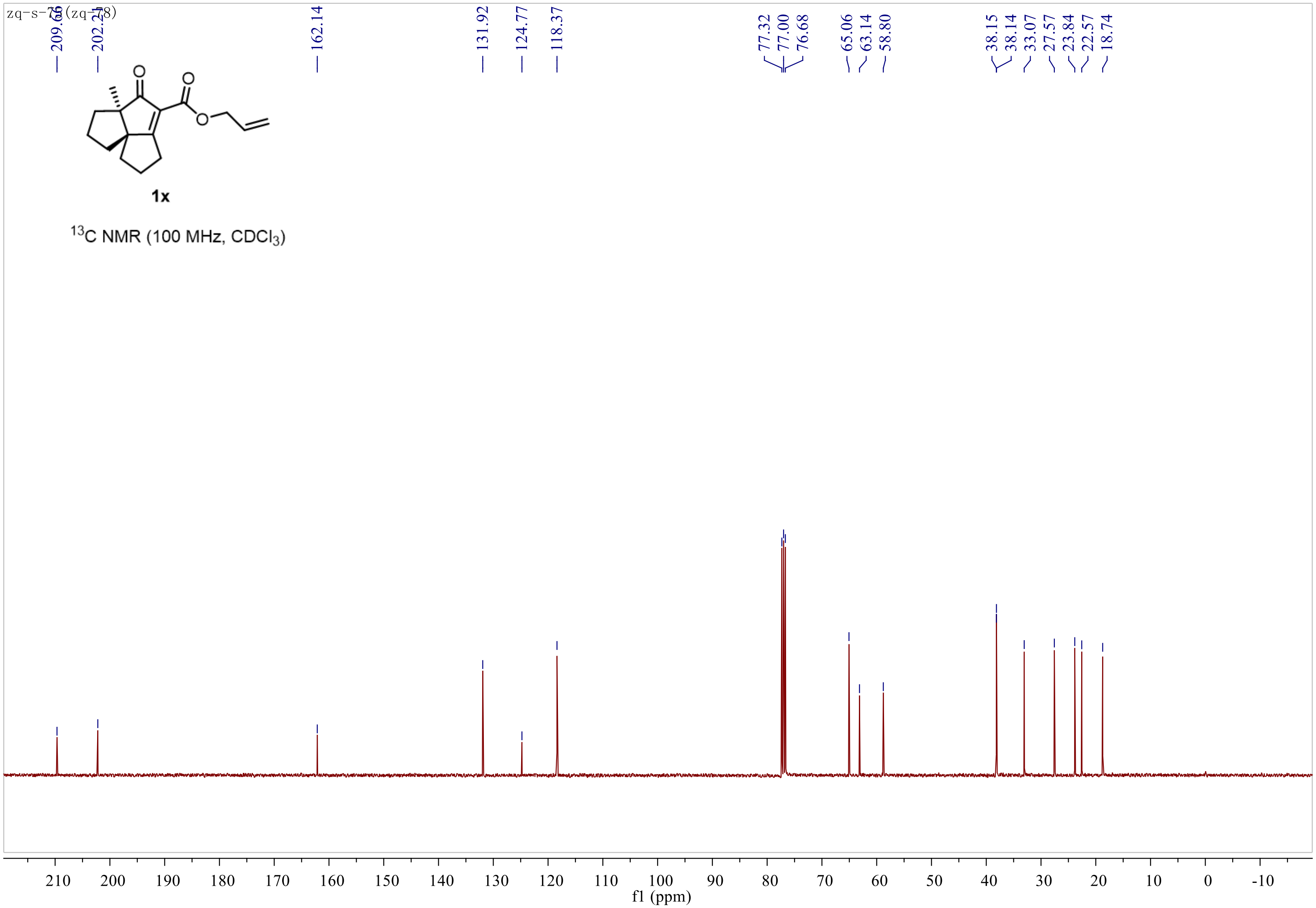

Supplementary Fig. 302. ^{13}C NMR spectra of compound **1x** in $CDCl_3$

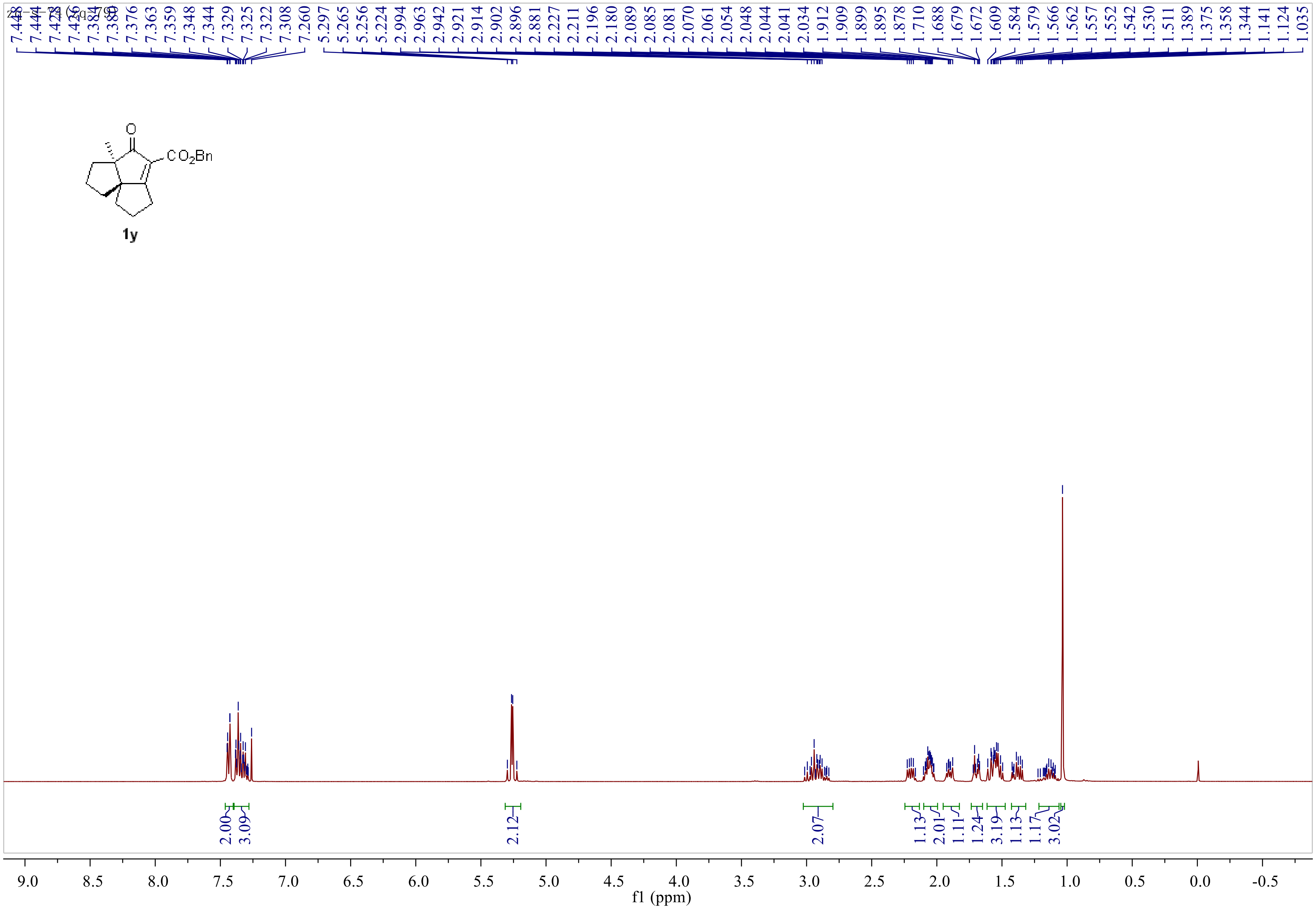

Supplementary Fig. 303. ^{1}H NMR spectra of compound **1y** in $CDCl_3$

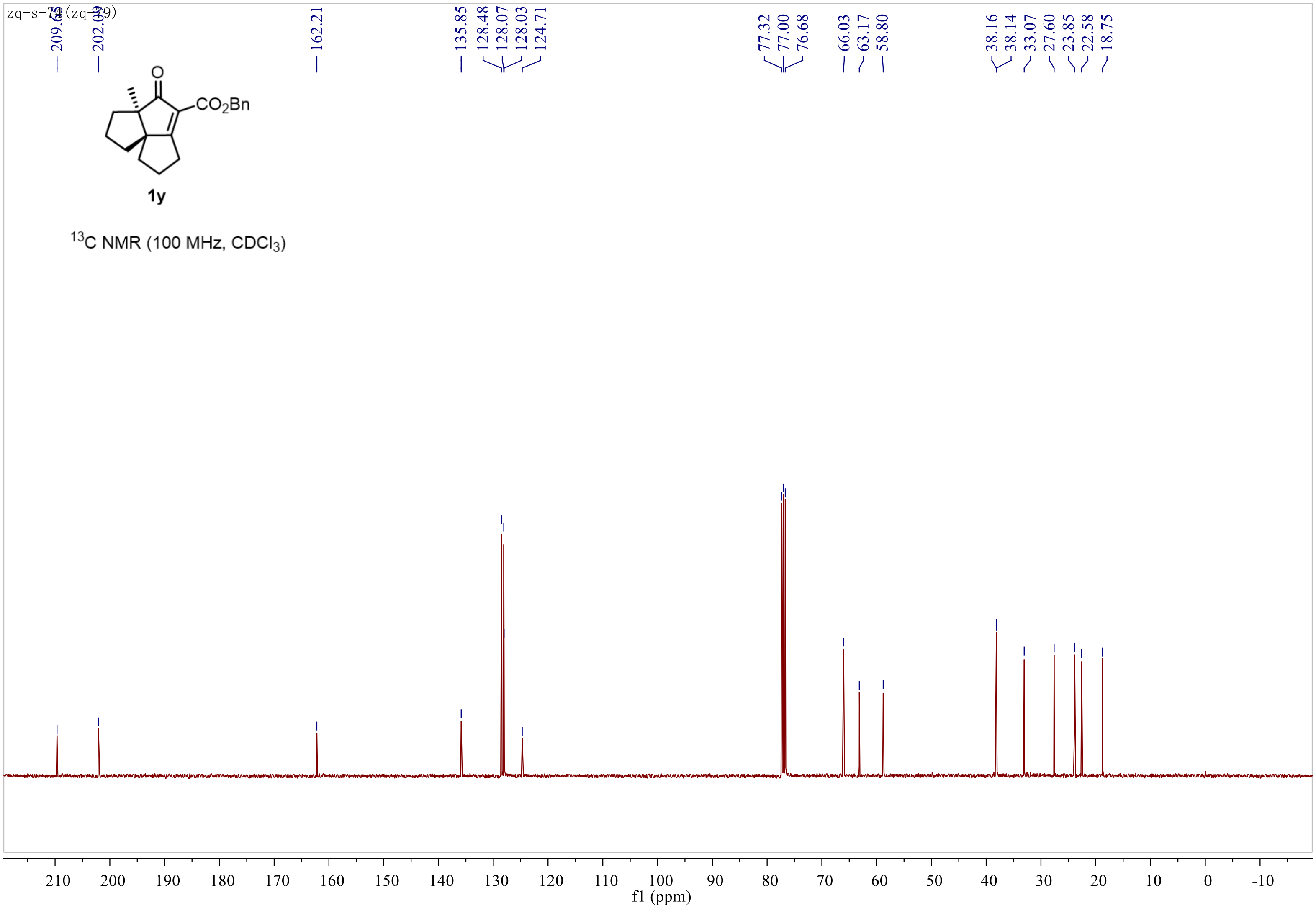

Supplementary Fig. 304. ^{13}C NMR spectra of compound **1y** in $CDCl_3$

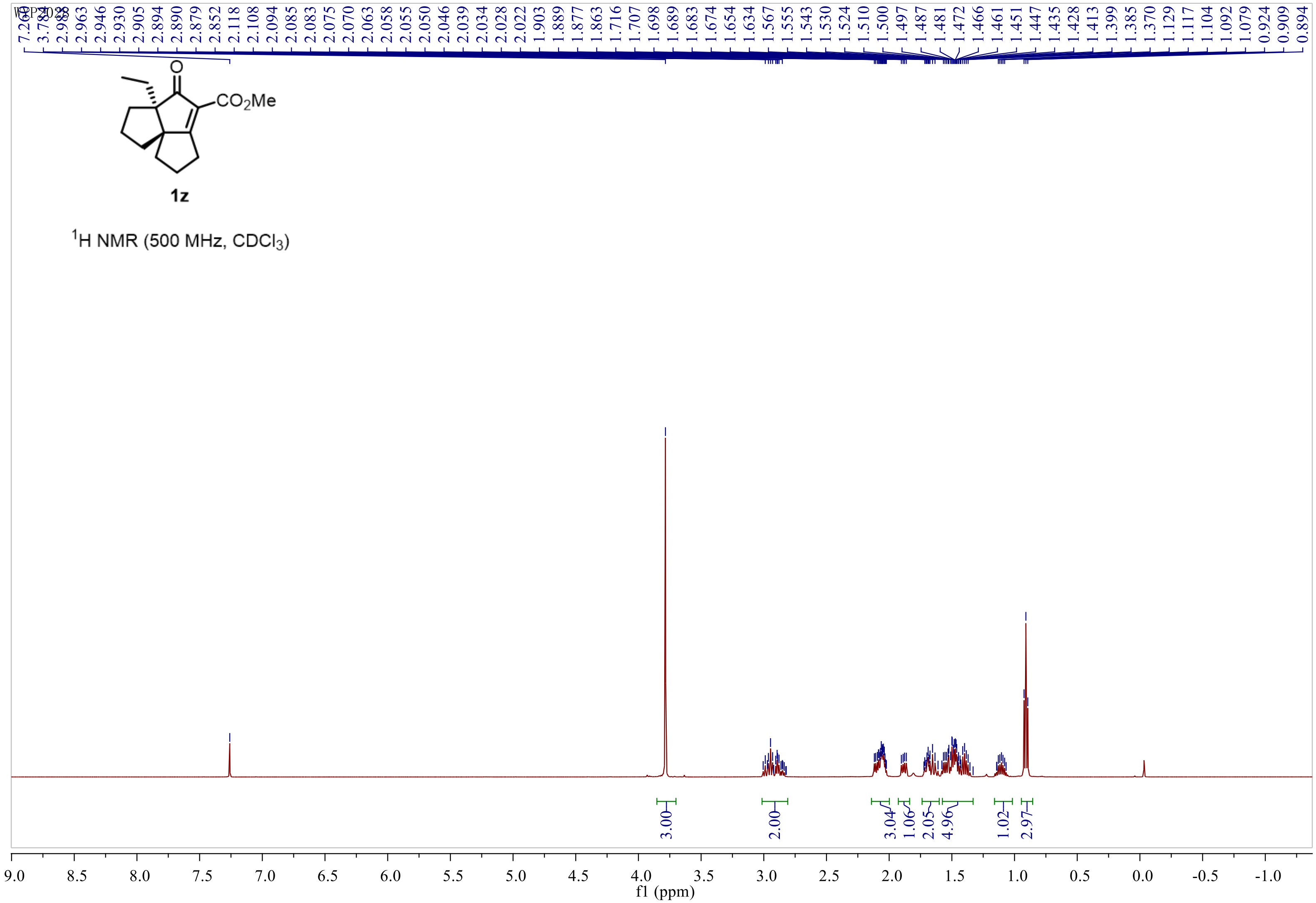

Supplementary Fig. 305. ^{1}H NMR spectra of compound **1z** in $CDCl_3$

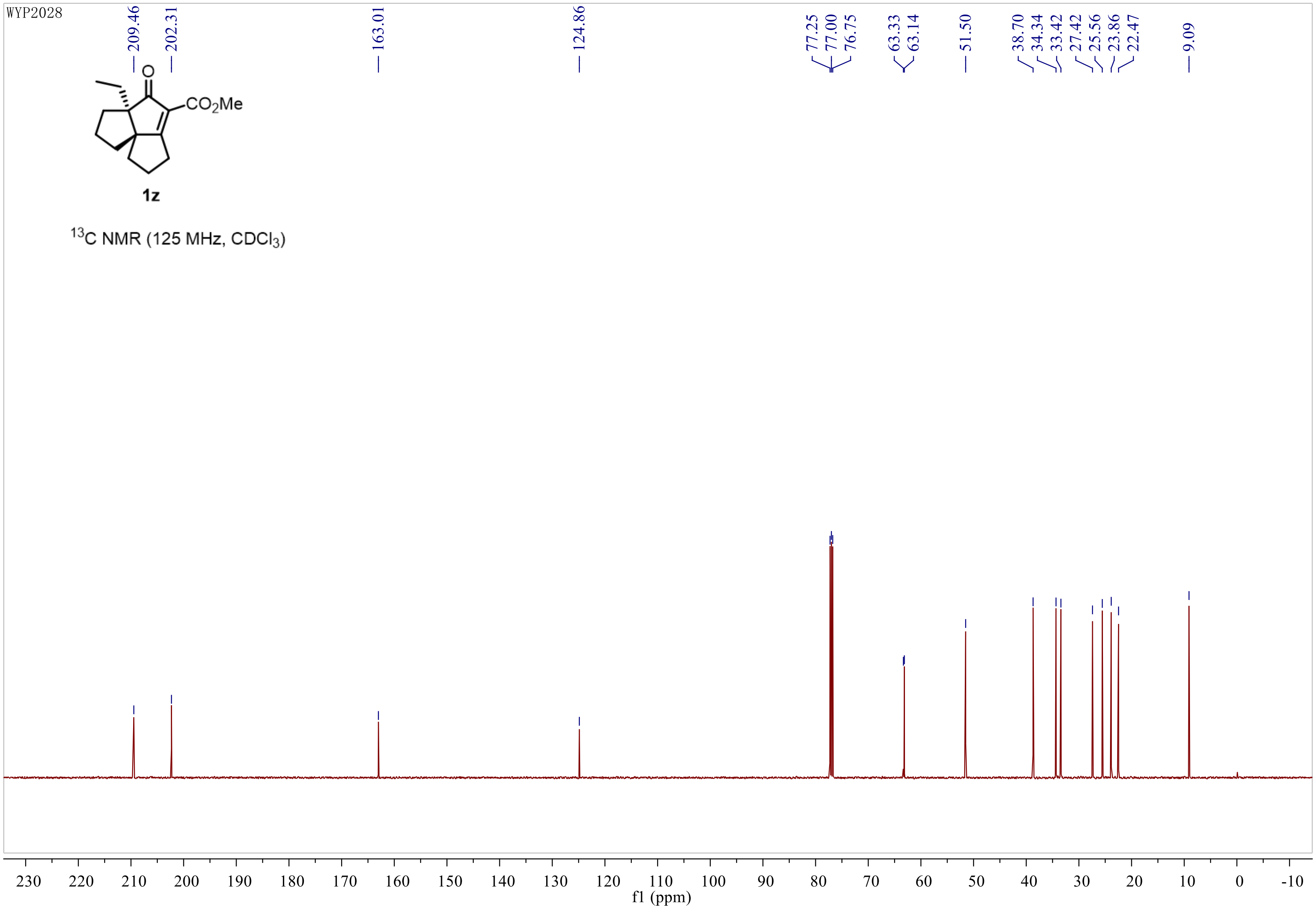

Supplementary Fig. 306. ^{13}C NMR spectra of compound **1z** in $CDCl_3$

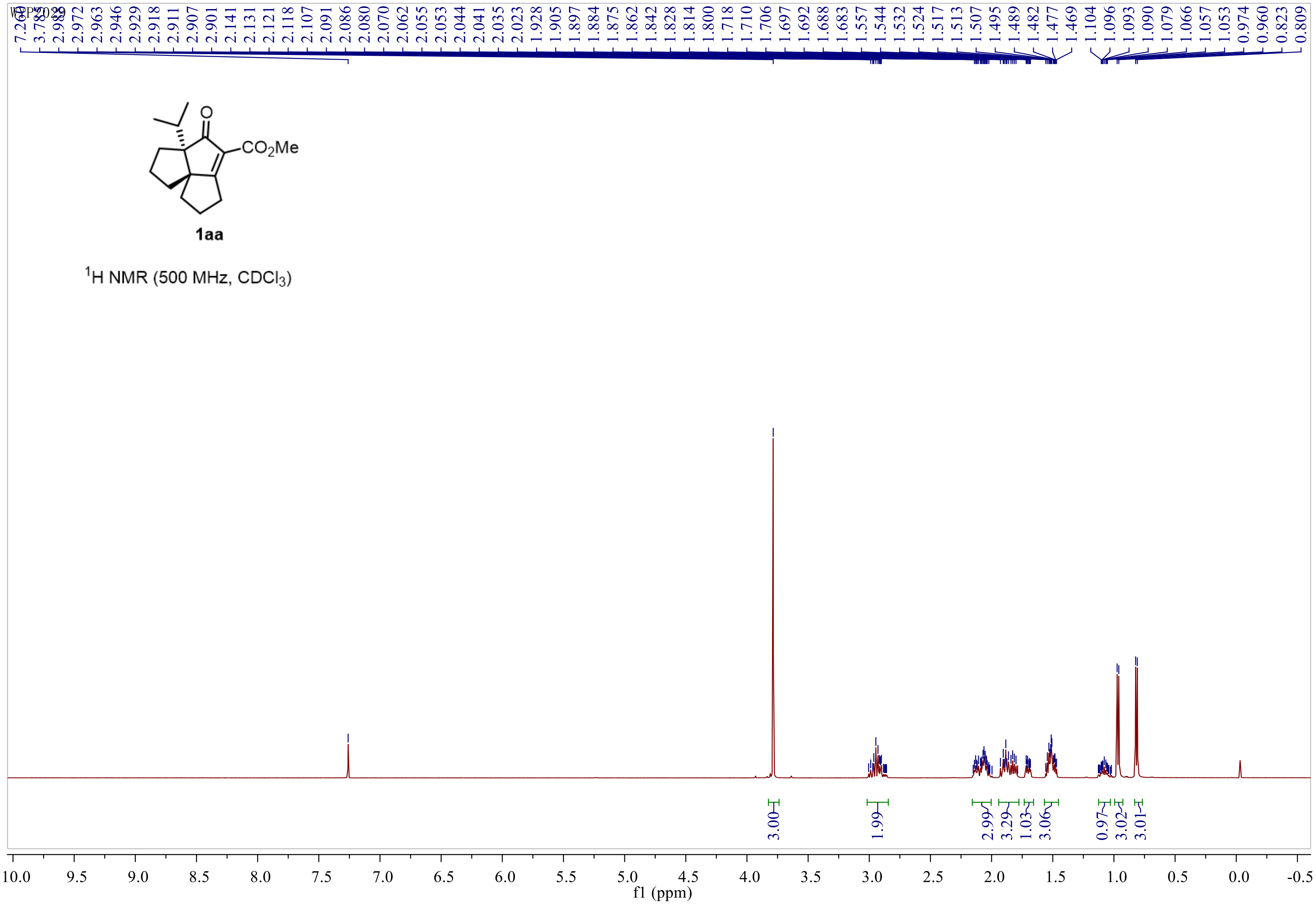

Supplementary Fig. 307. ^{1}H NMR spectra of compound **1aa** in $CDCl_3$

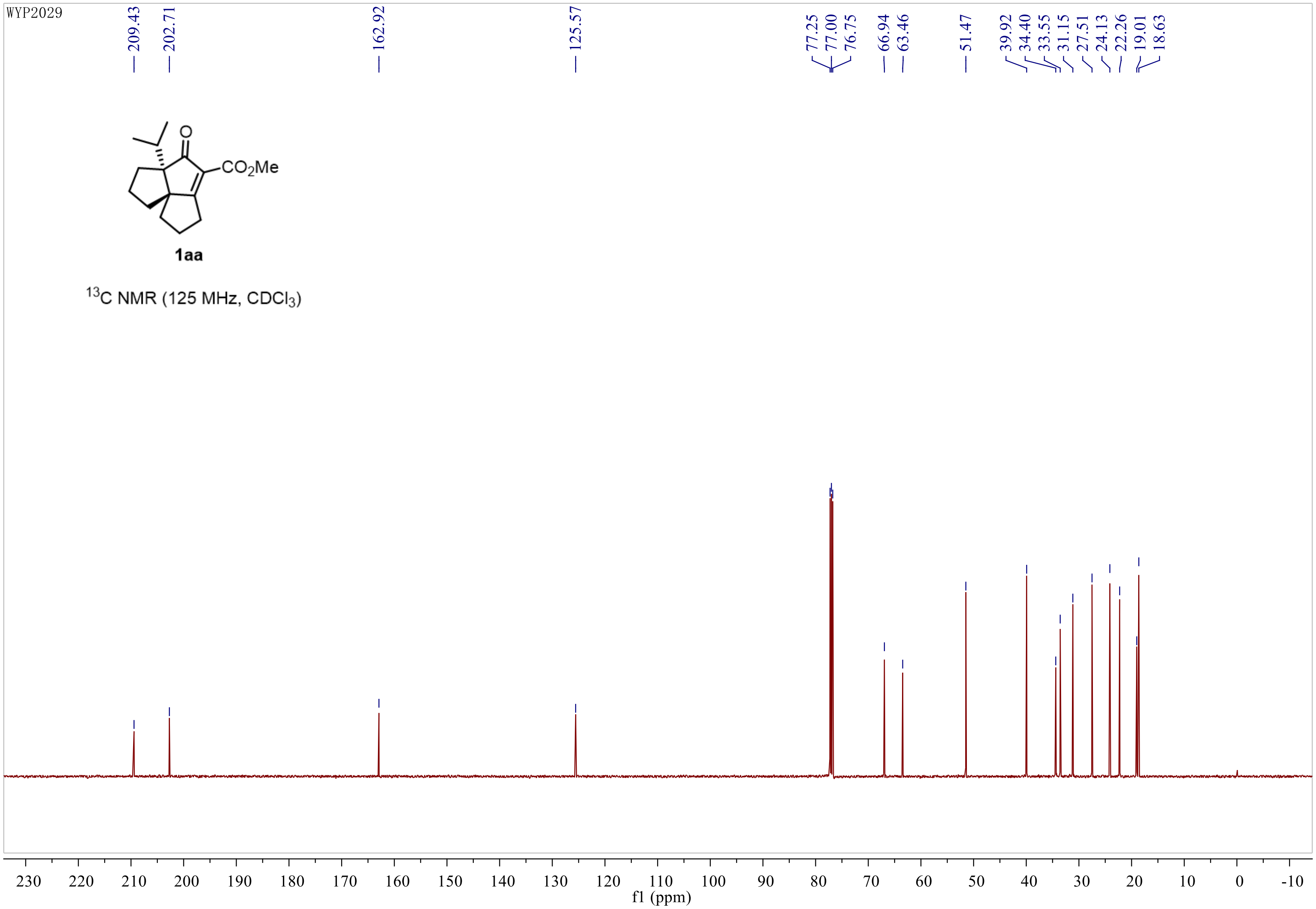

Supplementary Fig. 308. ^{13}C NMR spectra of compound **1aa** in $CDCl_3$

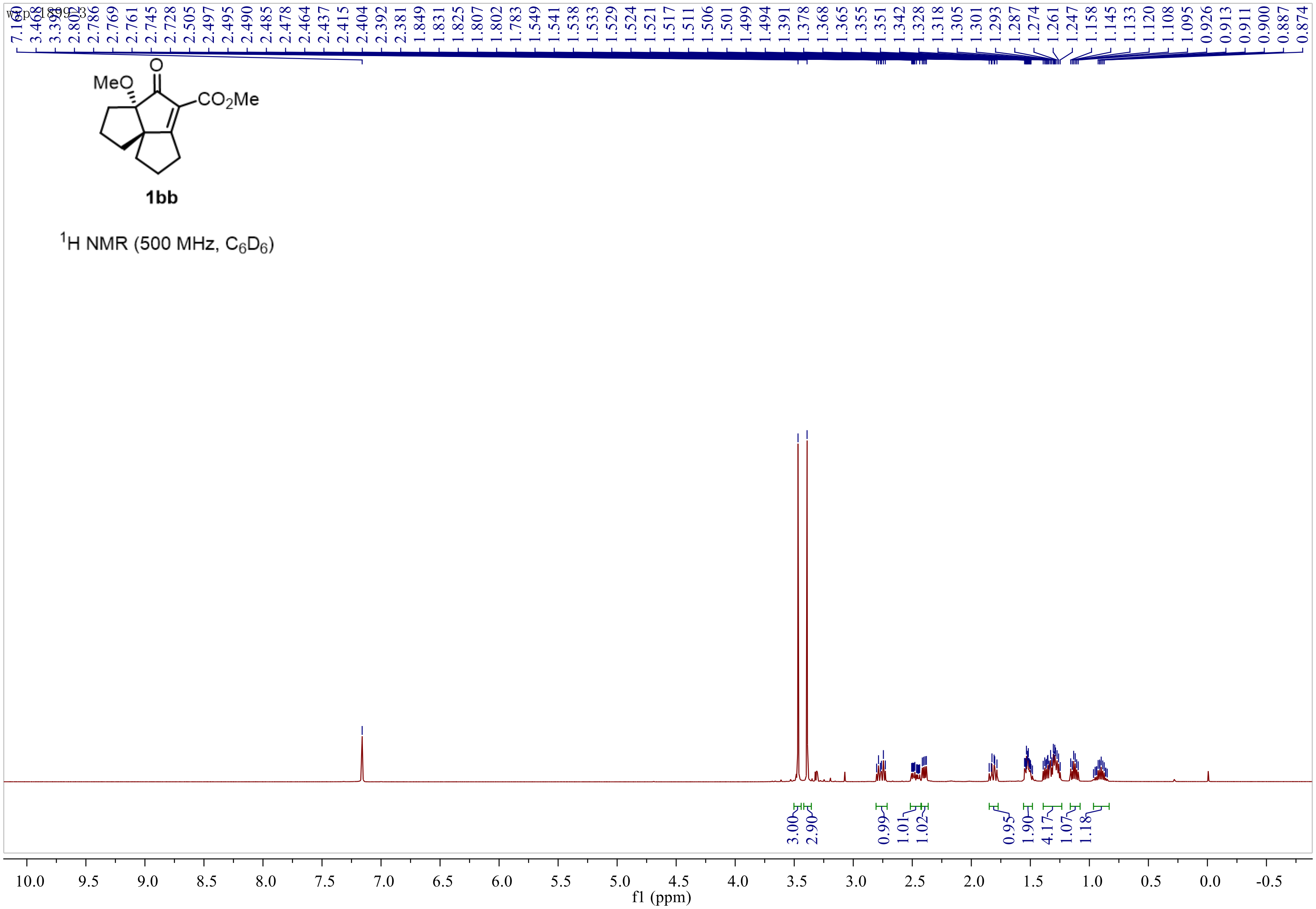

Supplementary Fig. 309. ^{1}H NMR spectra of compound **1bb** in C_6D_6

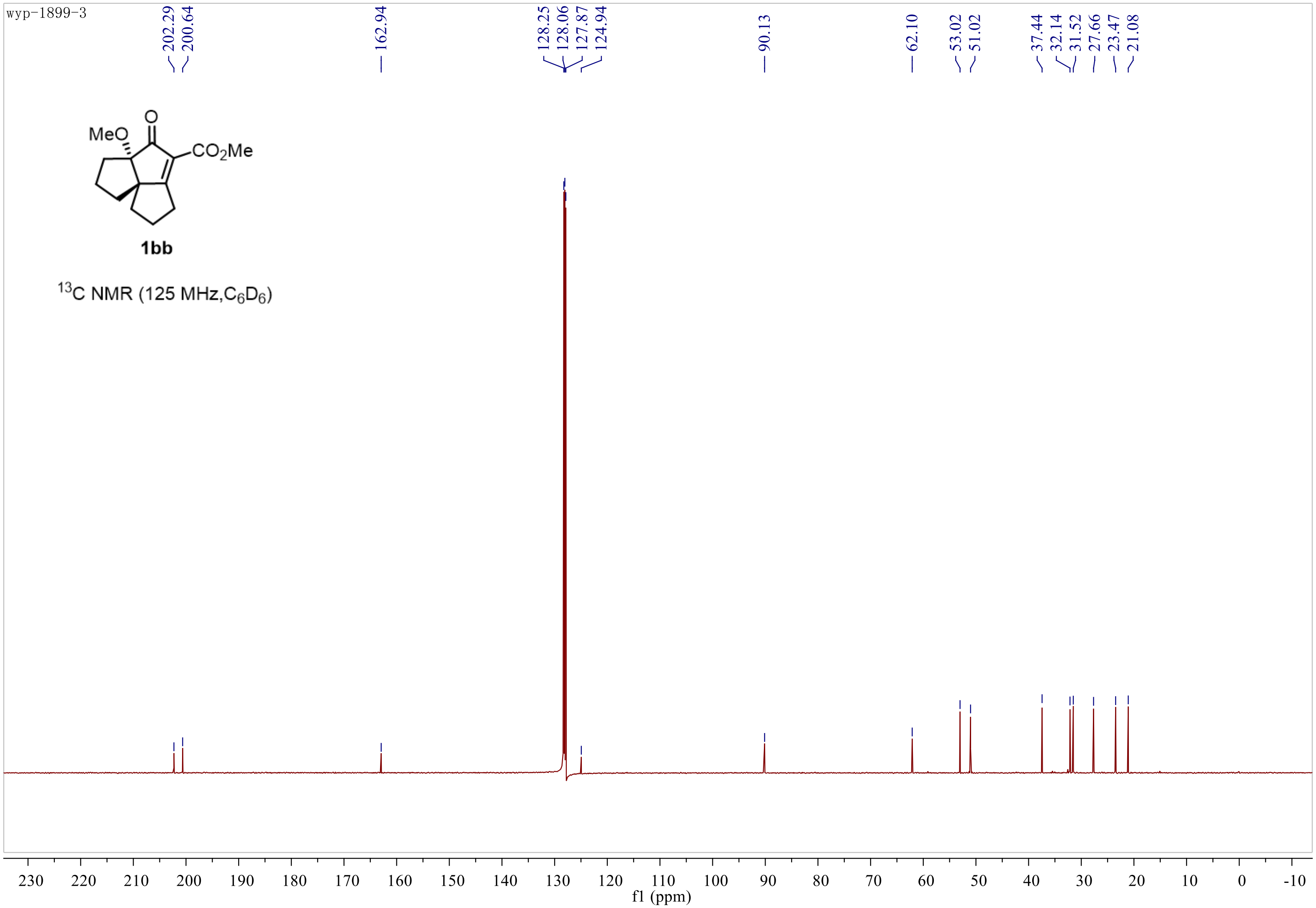

Supplementary Fig. 310. ^{13}C NMR spectra of compound **1bb** in C_6D_6

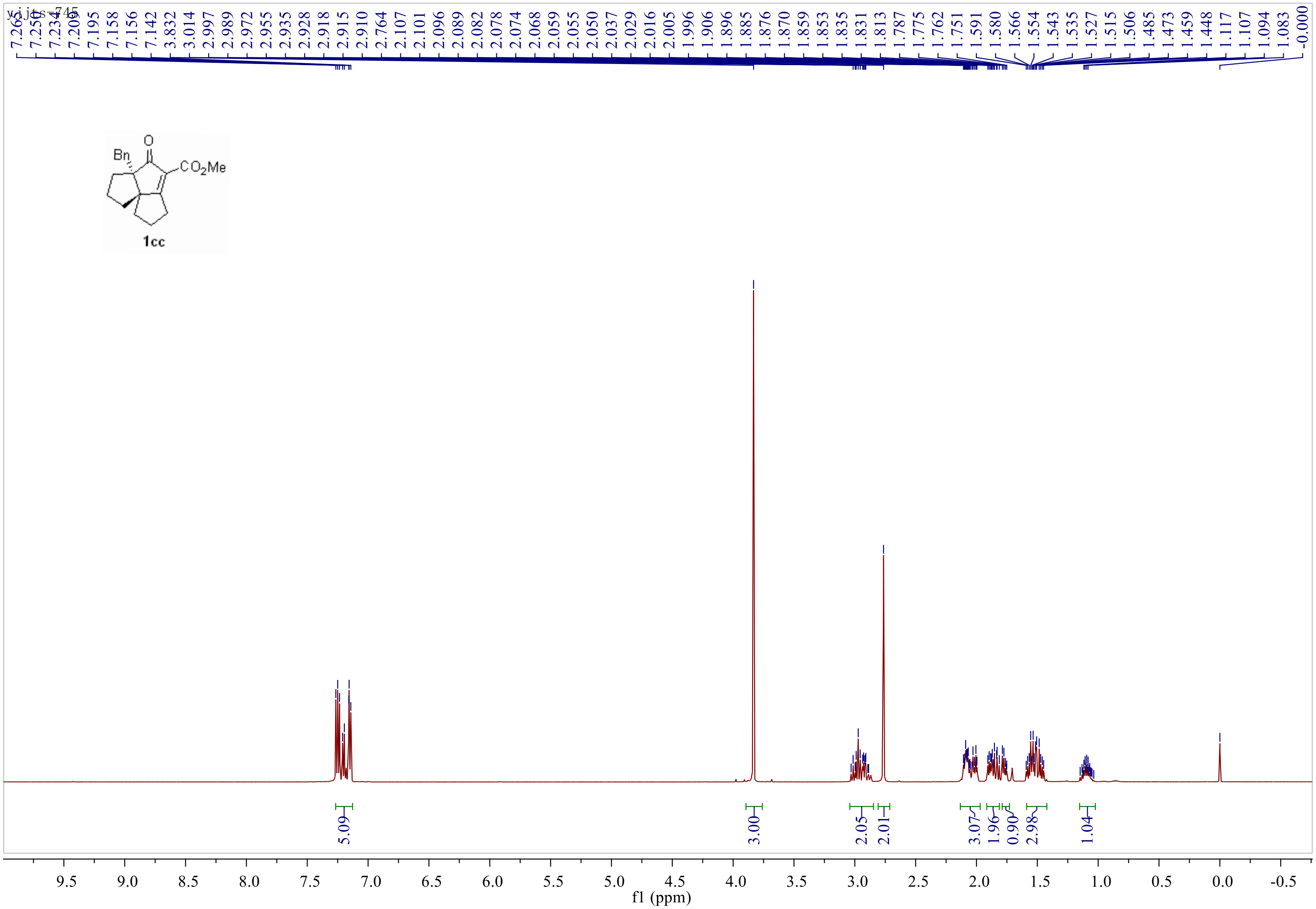

Supplementary Fig. 311. ^{1}H NMR spectra of compound **1cc** in $CDCl_3$

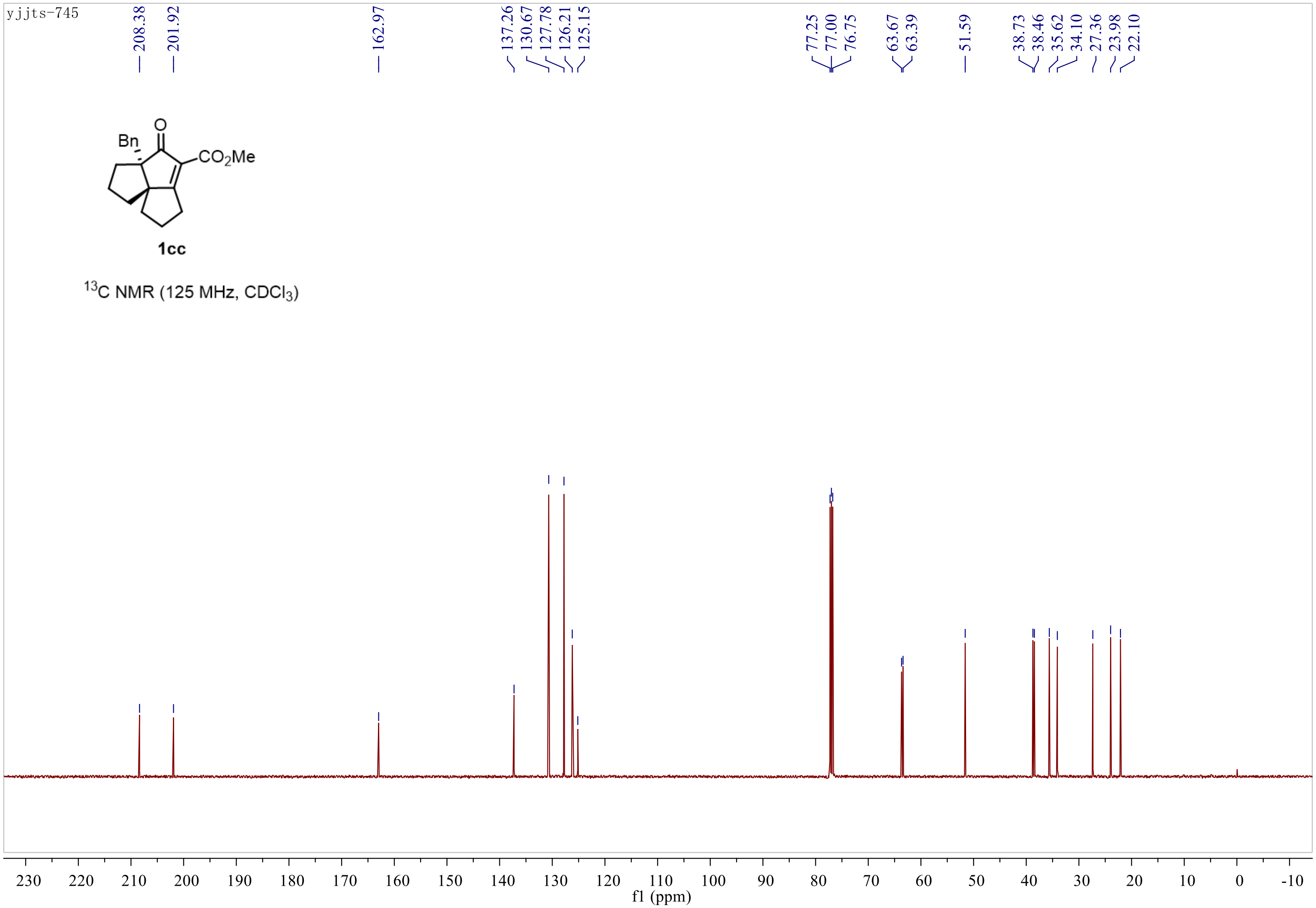

Supplementary Fig. 312. ^{13}C NMR spectra of compound **1cc** in $CDCl_3$

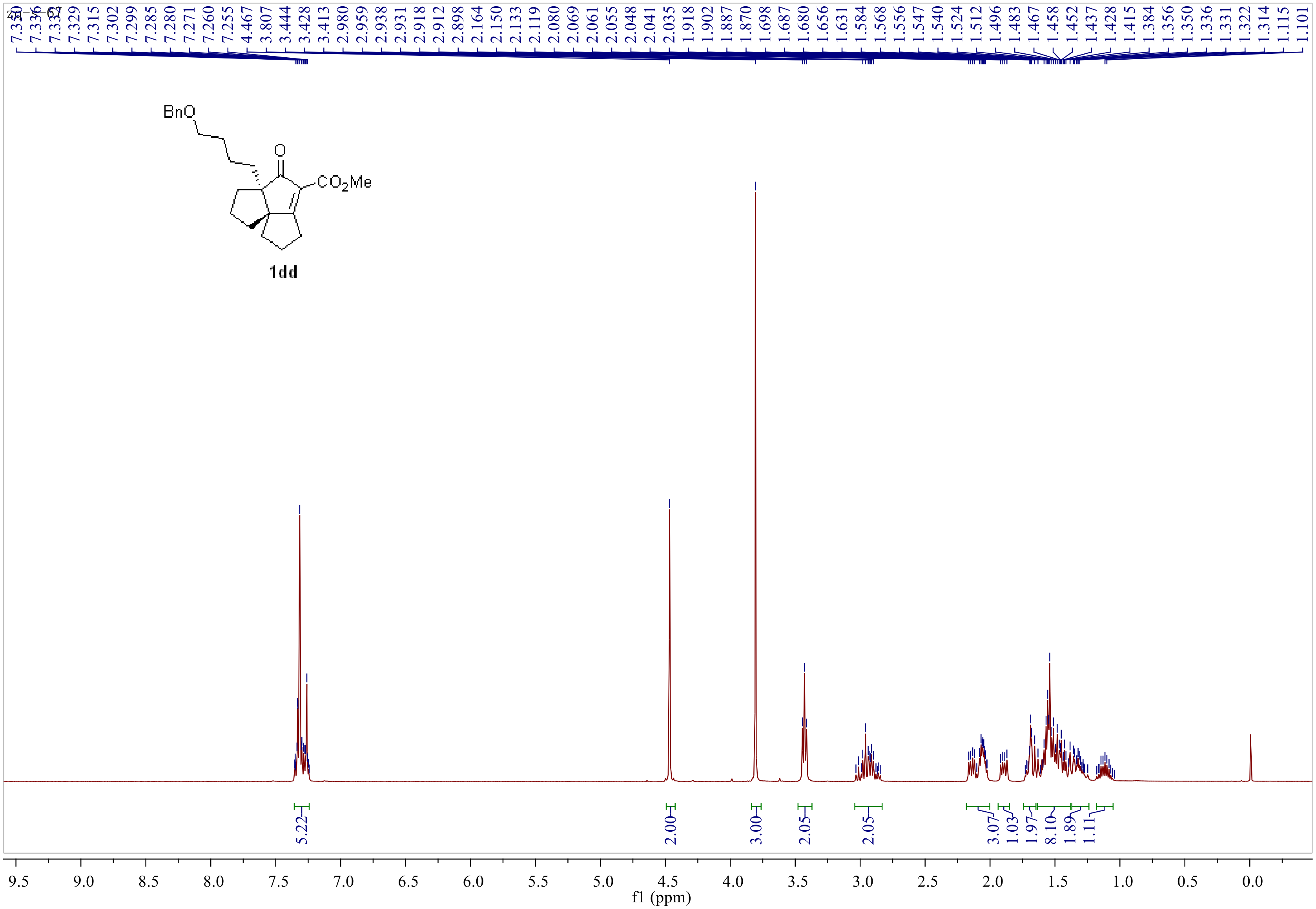

Supplementary Fig. 313. ^{1}H NMR spectra of compound **1dd** in $CDCl_3$

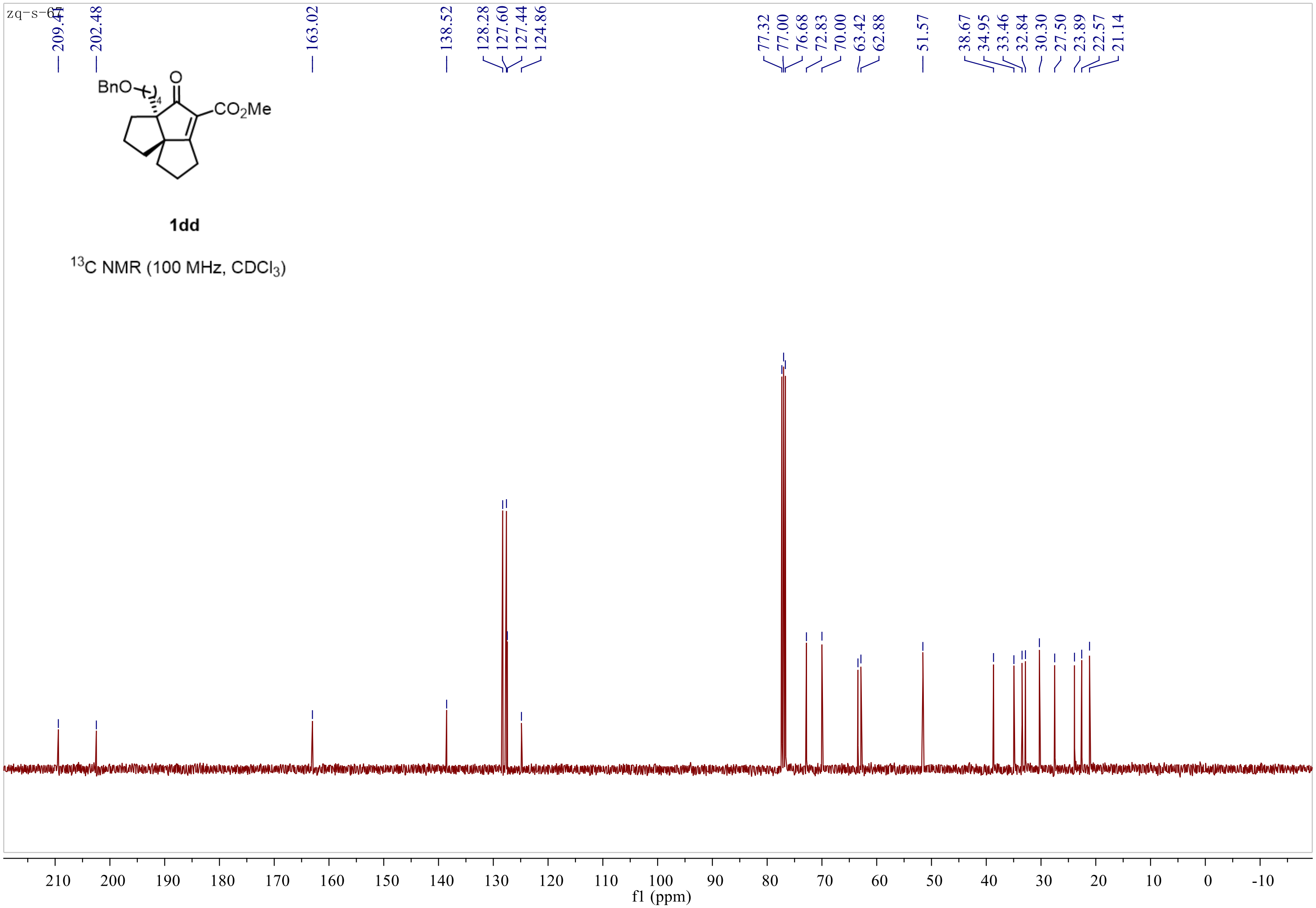

Supplementary Fig. 314. ^{13}C NMR spectra of compound **1dd** in $CDCl_3$

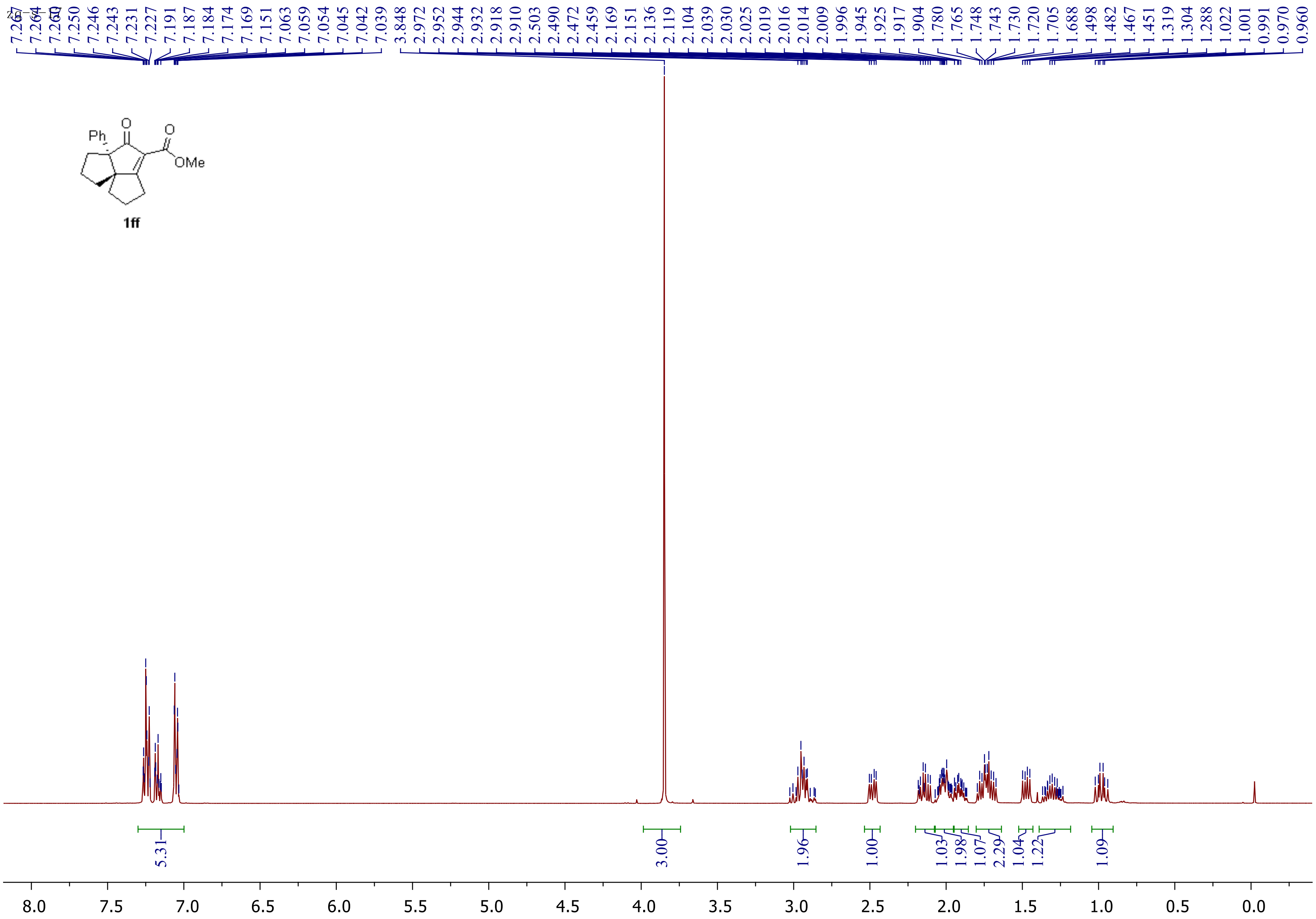

Supplementary Fig. 315. ^{1}H NMR spectra of compound **1ff** in $CDCl_3$

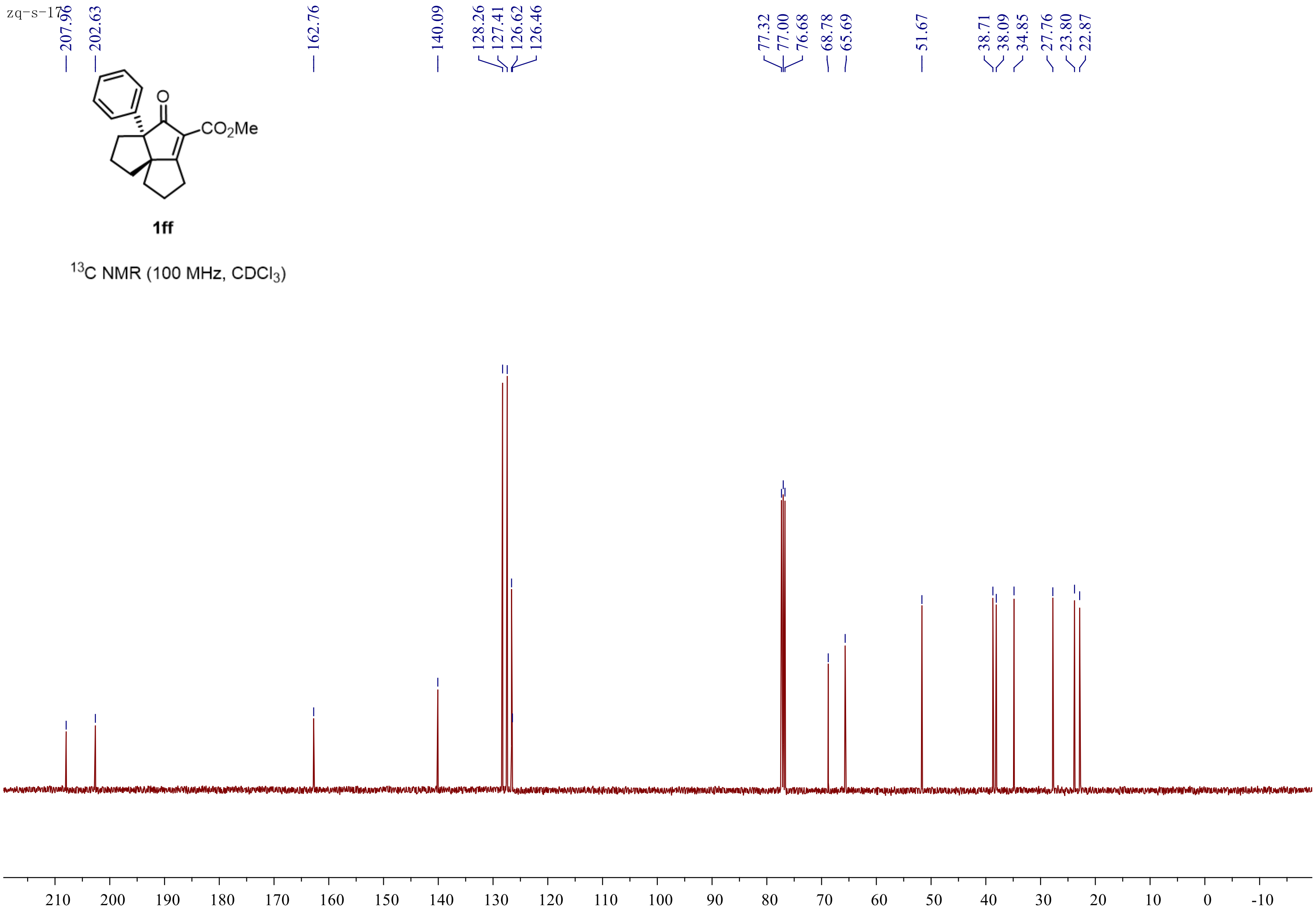

Supplementary Fig. 316. ^{13}C NMR spectra of compound **1ff** in $CDCl_3$

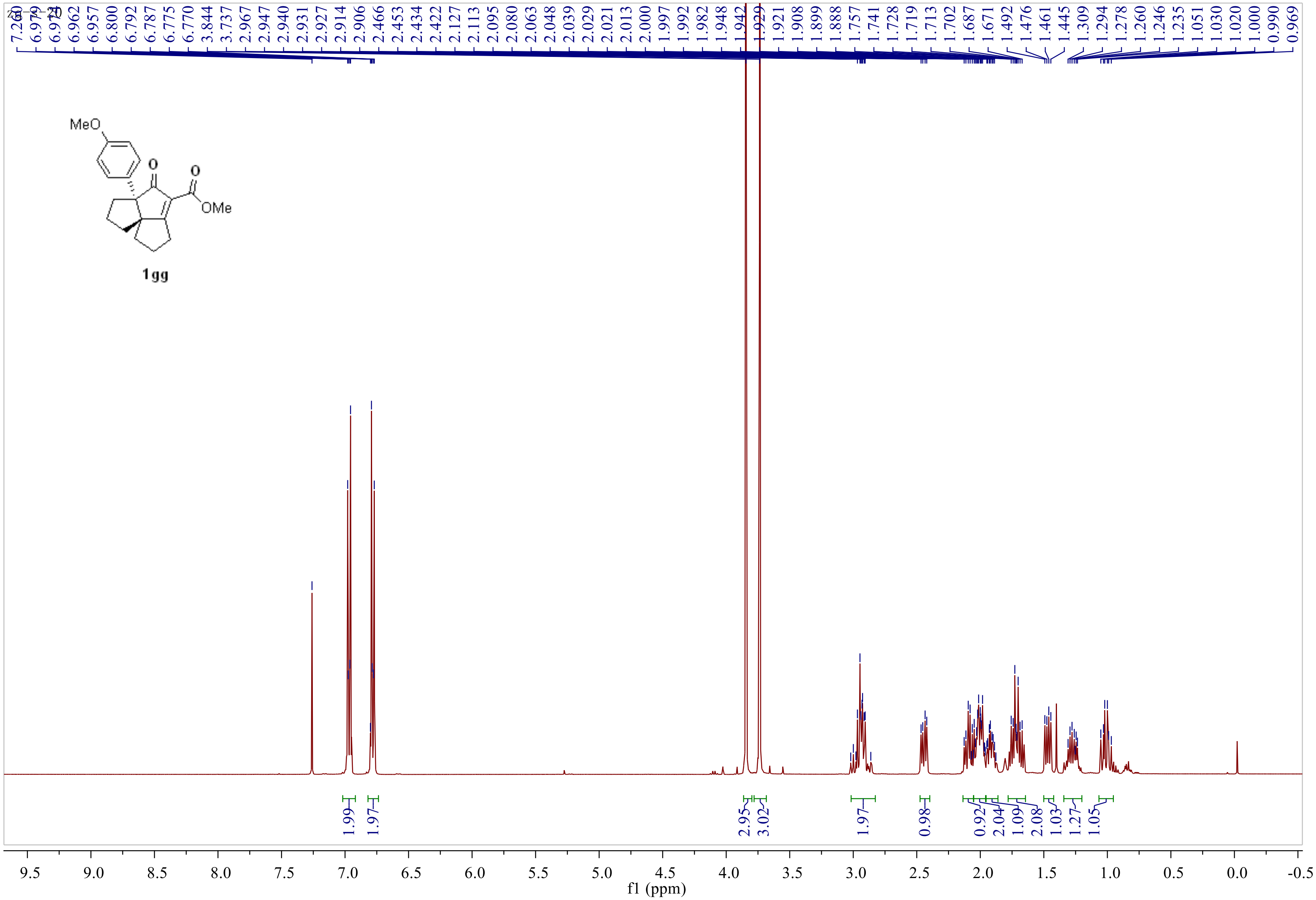

Supplementary Fig. 317. ^{1}H NMR spectra of compound **1gg** in $CDCl_3$

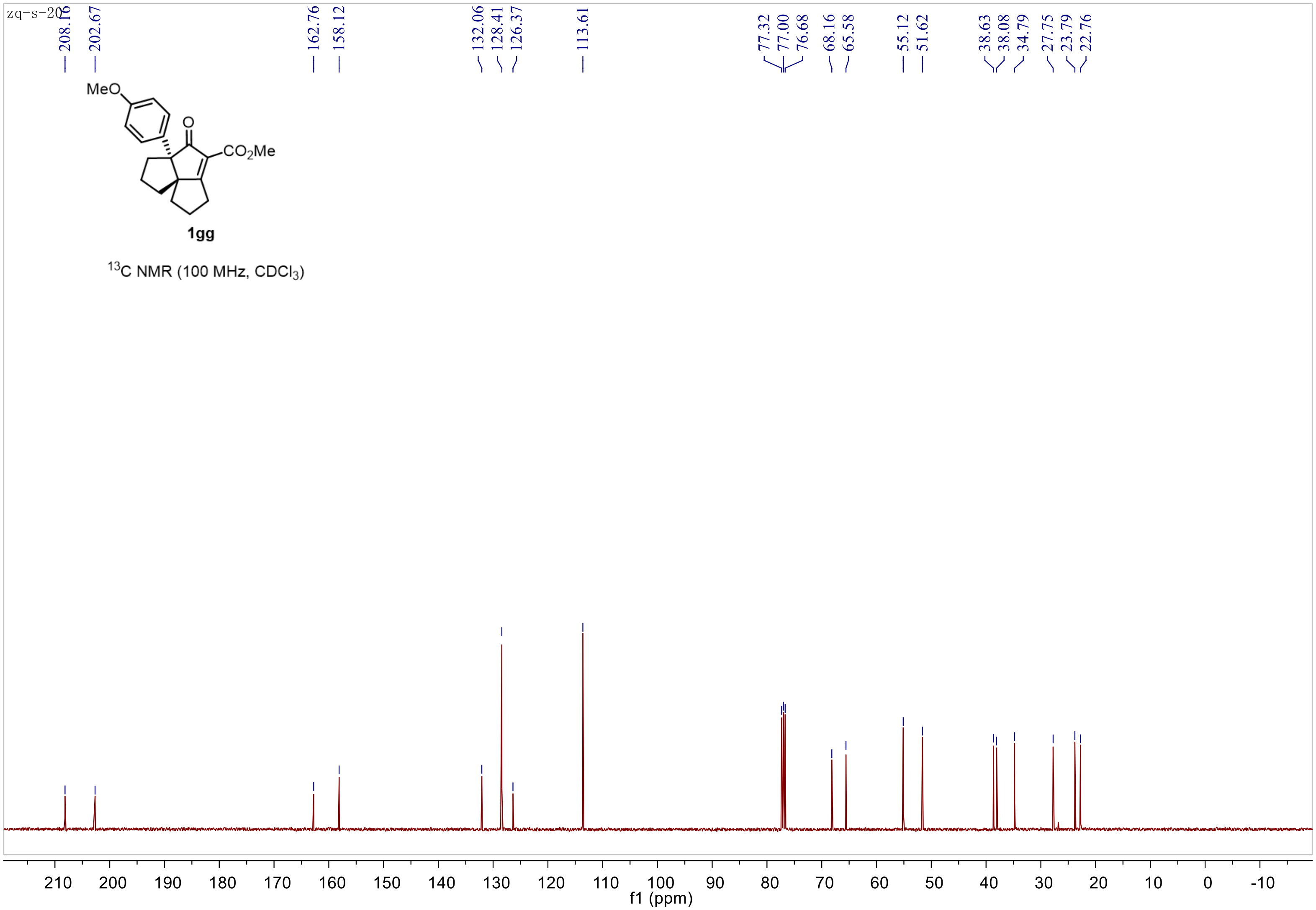

Supplementary Fig. 318. ^{13}C NMR spectra of compound **1gg** in $CDCl_3$

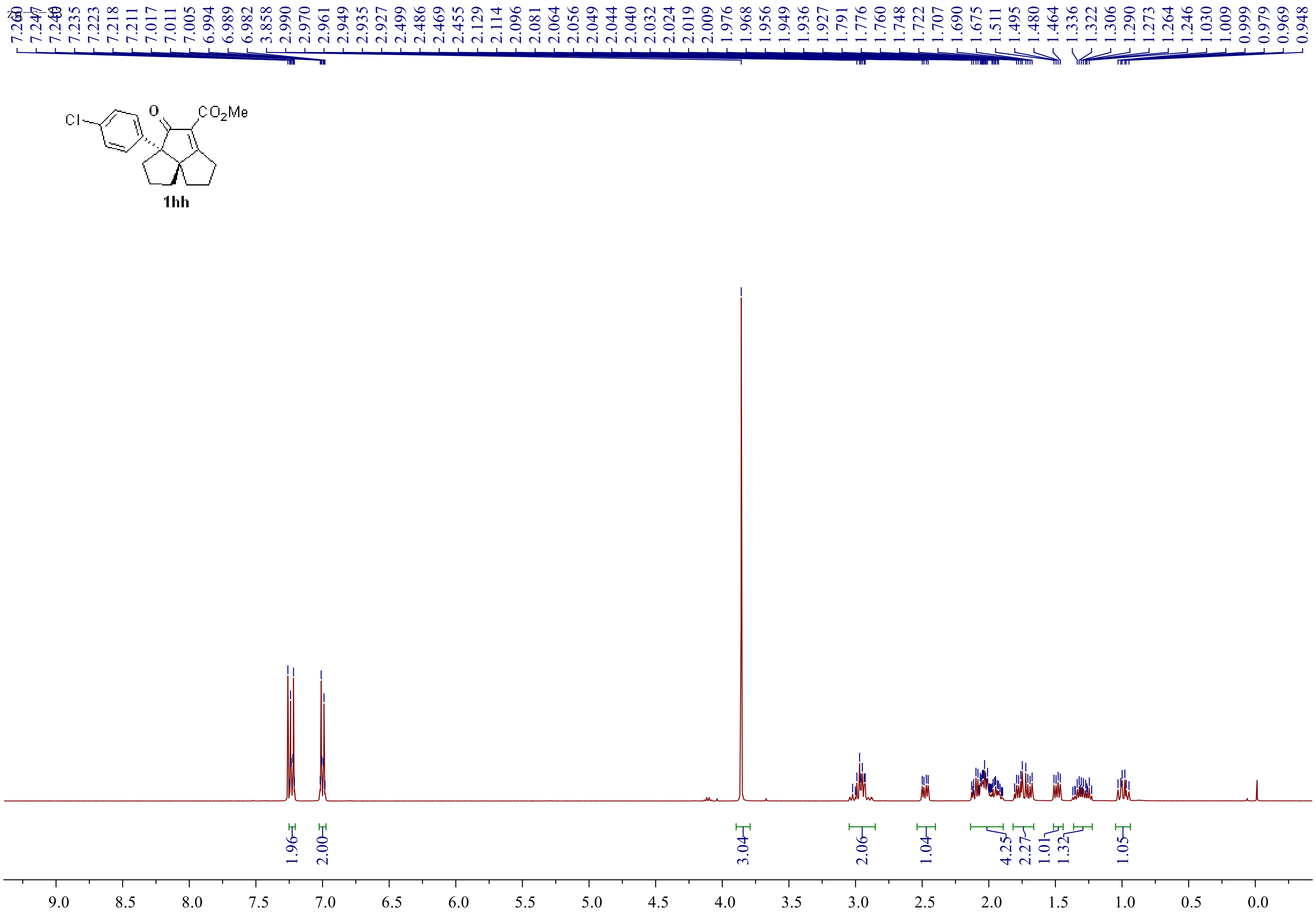

Supplementary Fig. 319. ^{1}H NMR spectra of compound **1hh** in $CDCl_3$

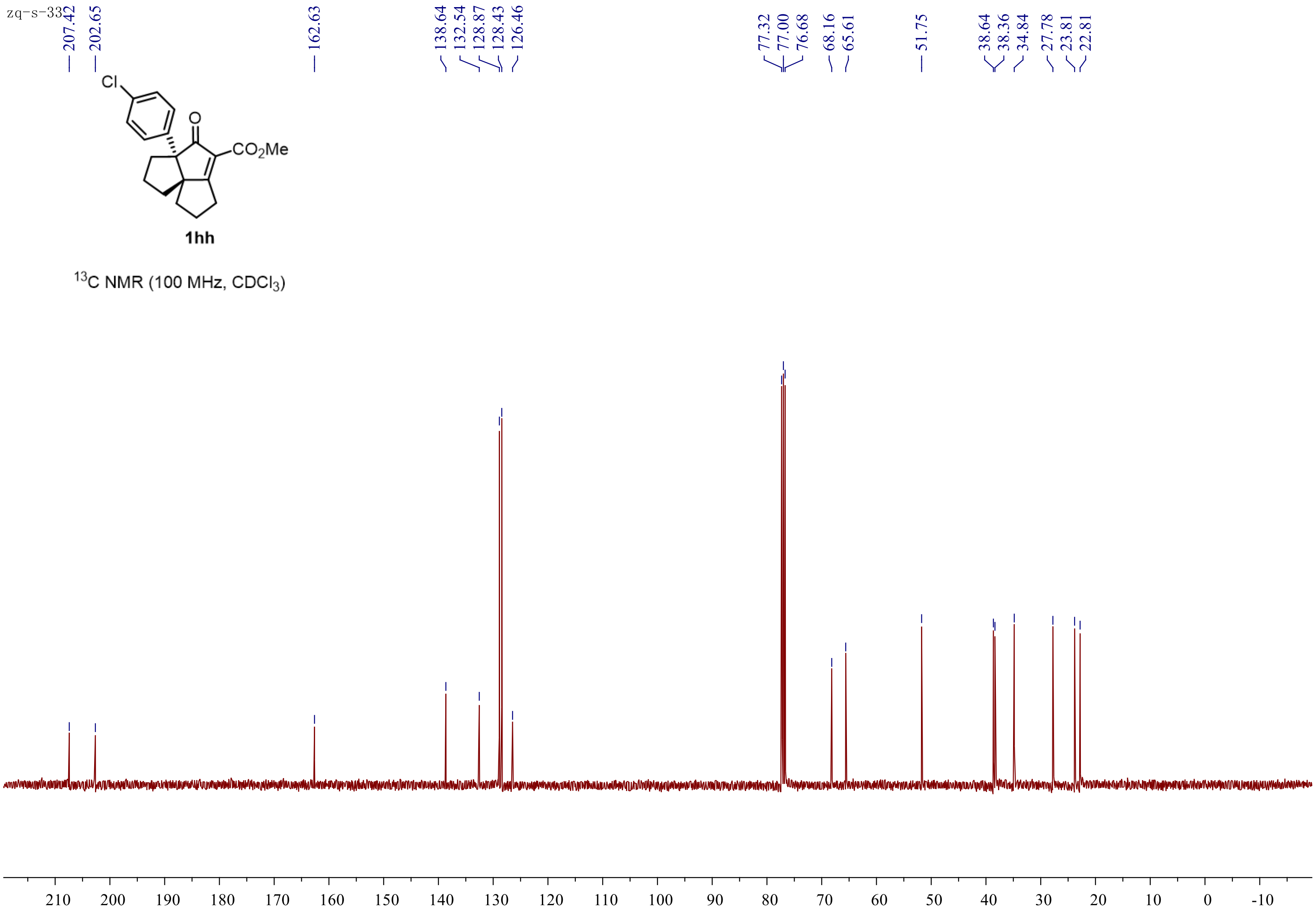

Supplementary Fig. 320. ^{13}C NMR spectra of compound **1hh** in $CDCl_3$

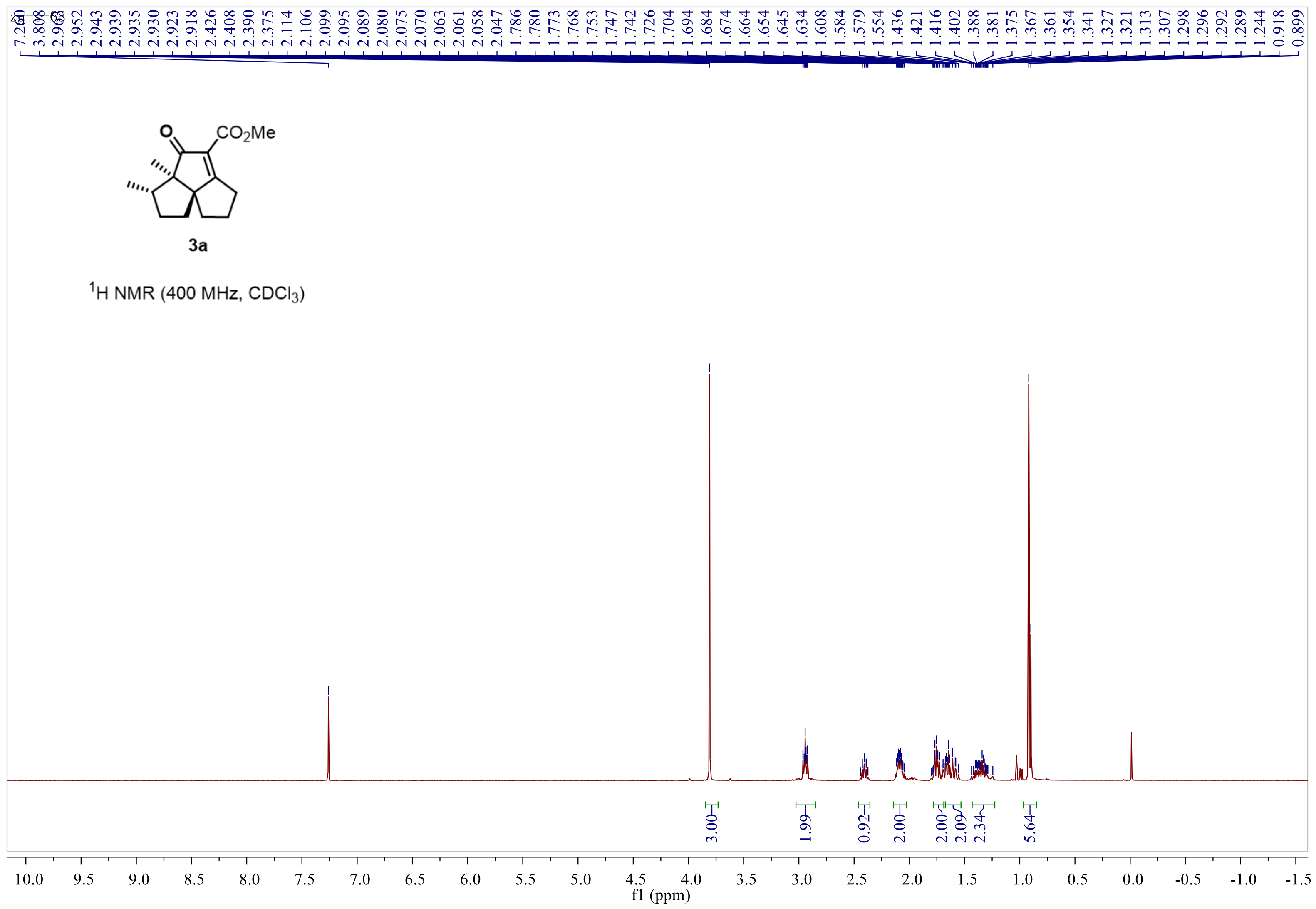

Supplementary Fig. 321. ^{1}H NMR spectra of compound **3a** in $CDCl_3$

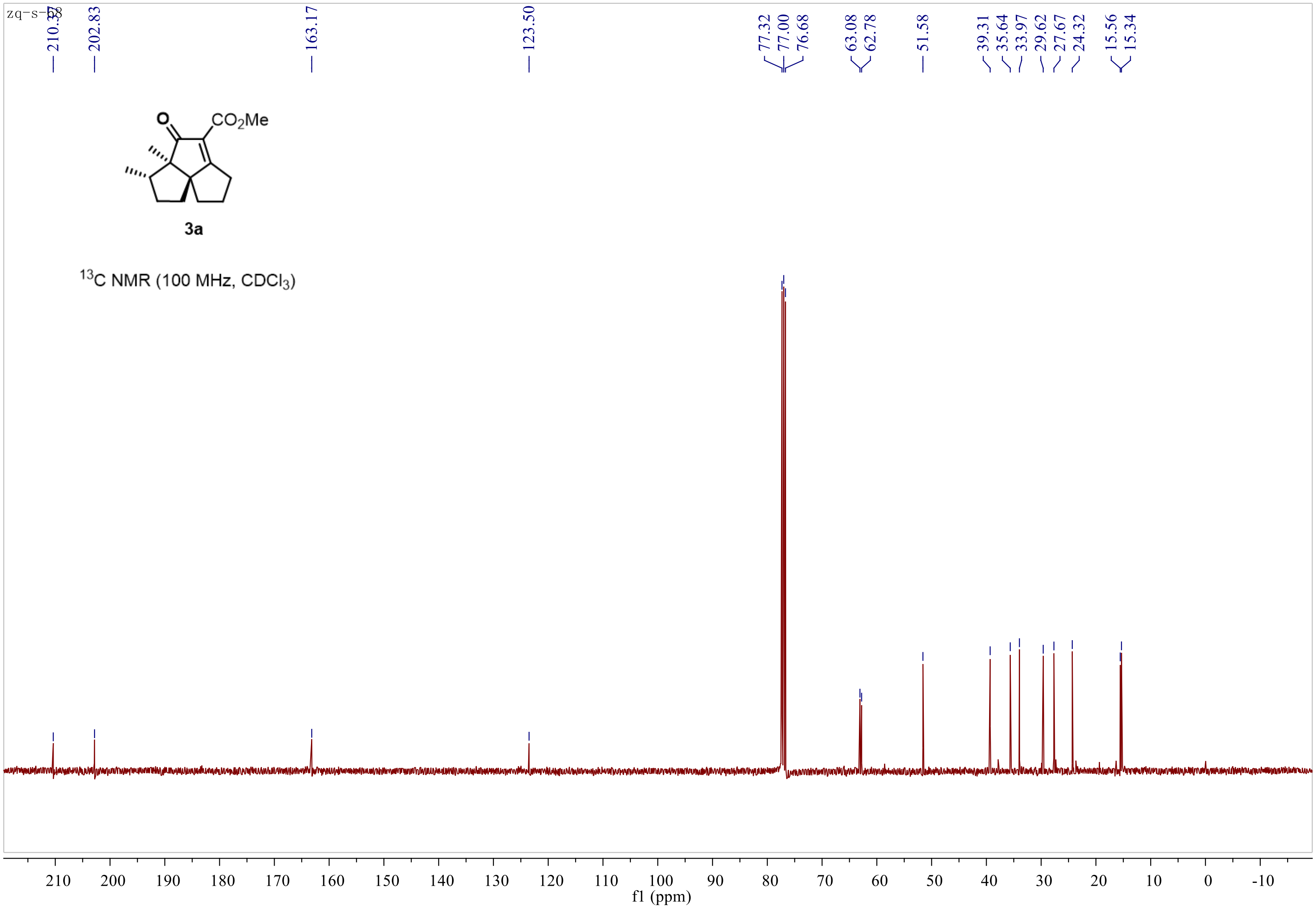

Supplementary Fig. 322. ^{13}C NMR spectra of compound **3a** in $CDCl_3$

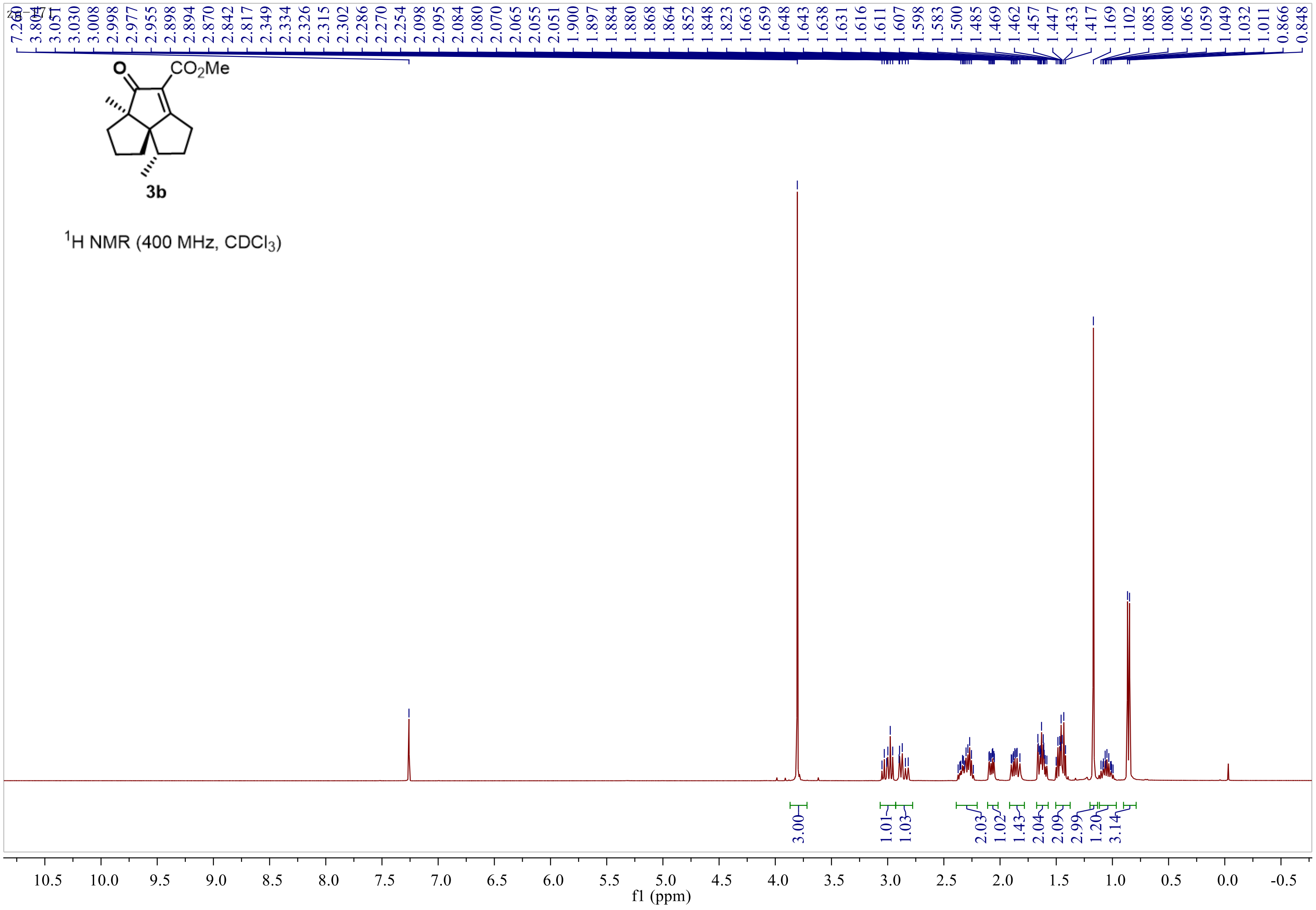

Supplementary Fig. 323. ^{1}H NMR spectra of compound **3b** in $CDCl_3$

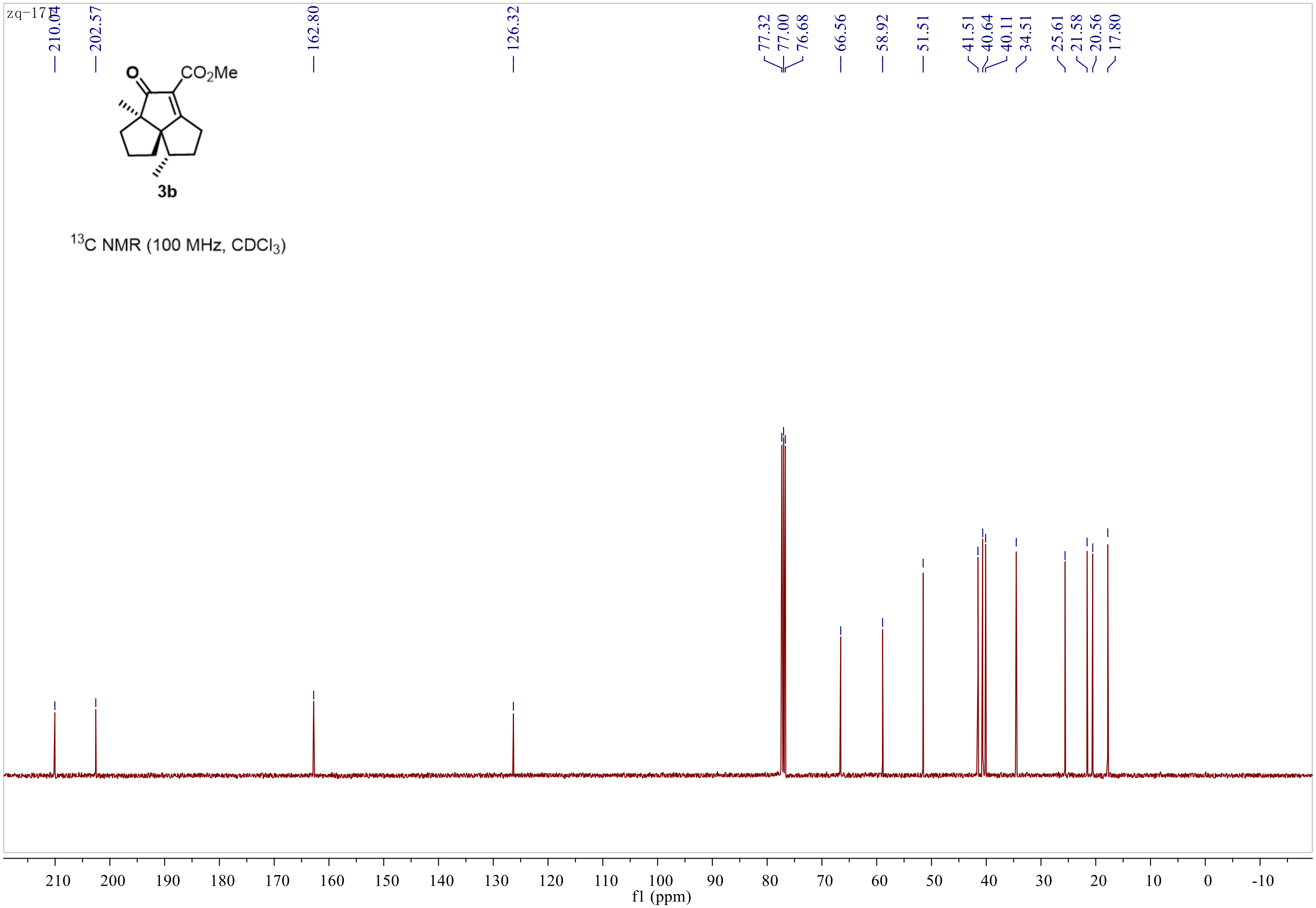

Supplementary Fig. 324. ^{13}C NMR spectra of compound **3b** in $CDCl_3$

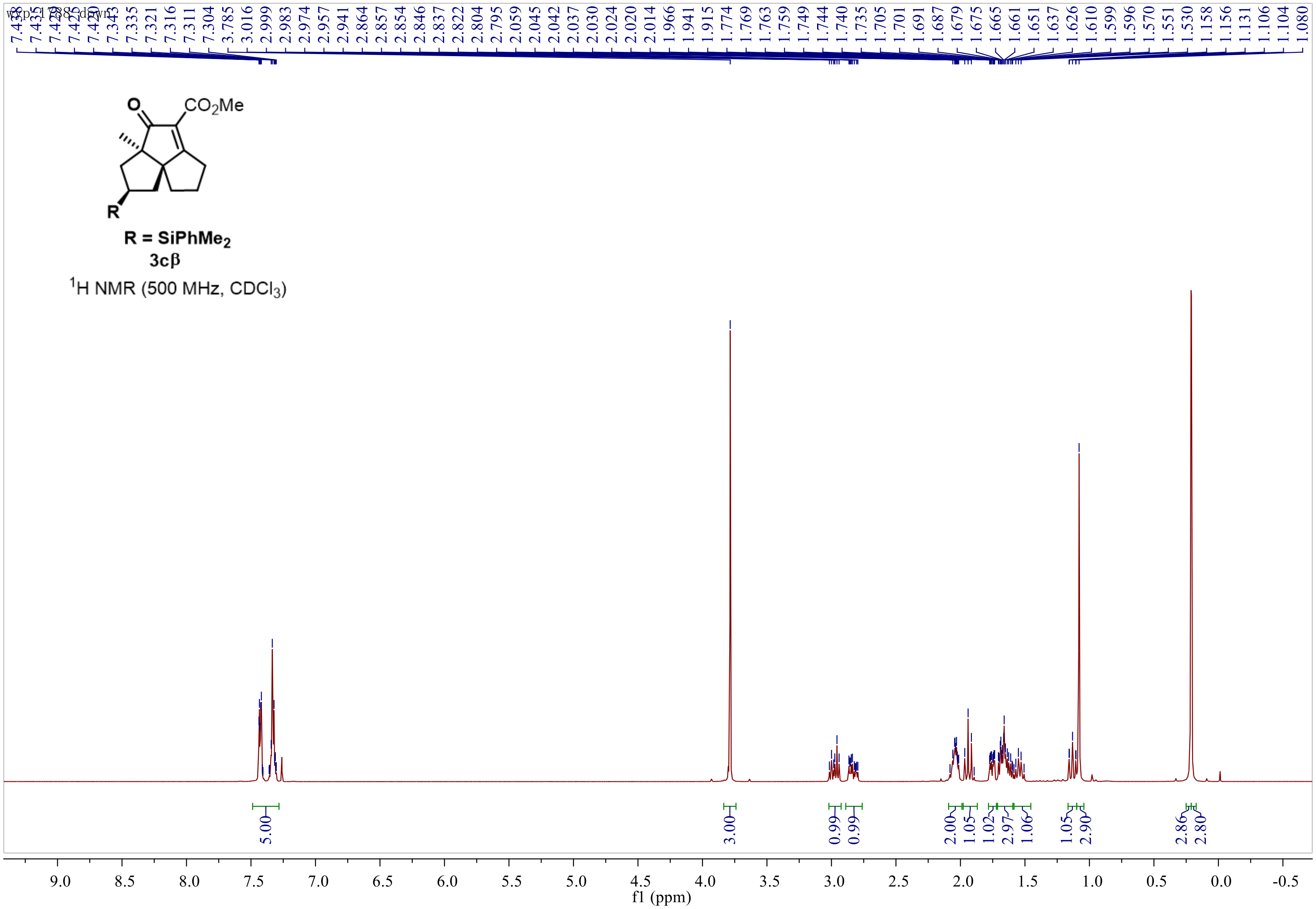

Supplementary Fig. 325. ^{1}H NMR spectra of compound **3cβ** in $CDCl_3$

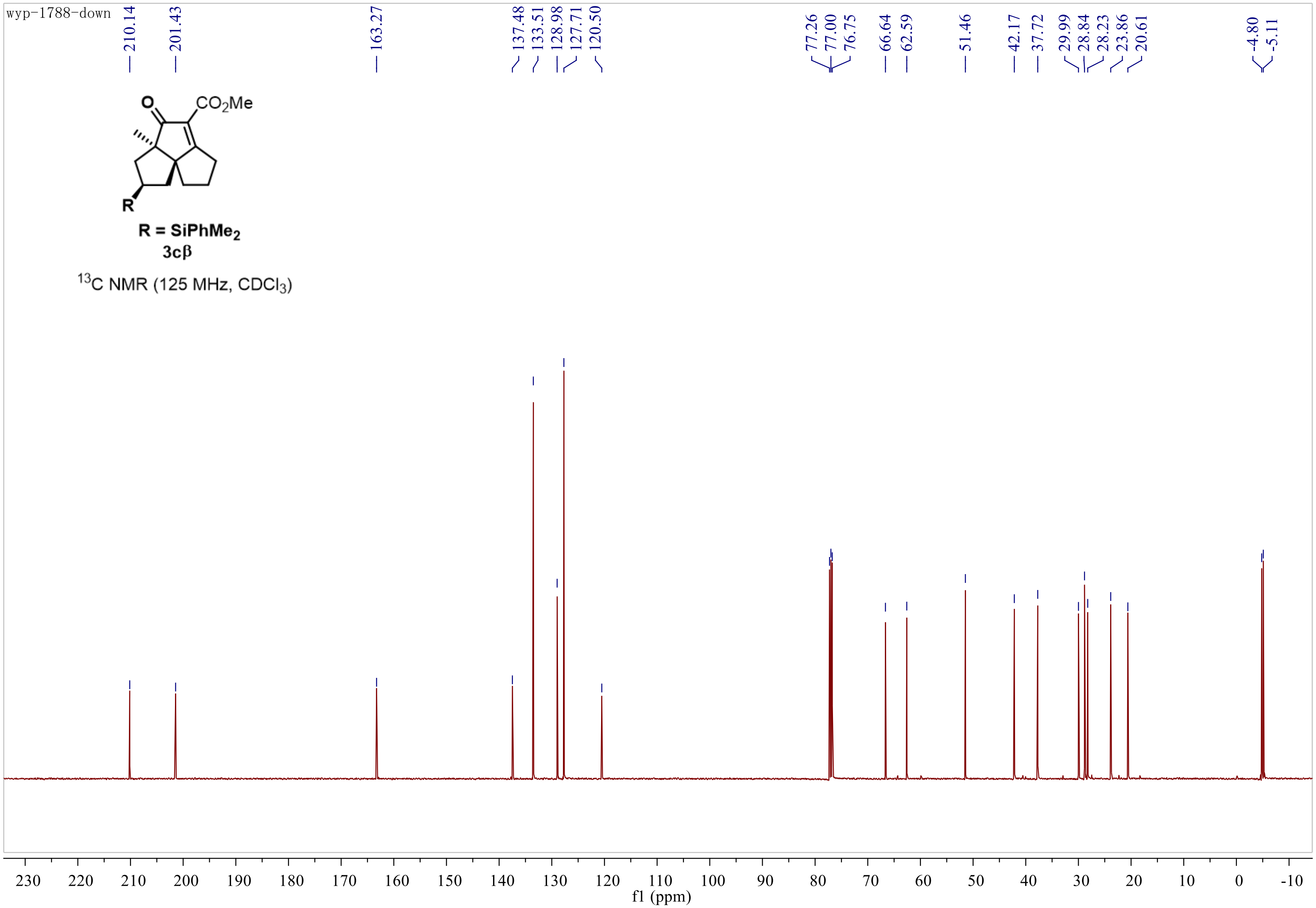

Supplementary Fig. 326. ^{13}C NMR spectra of compound **3cβ** in $CDCl_3$

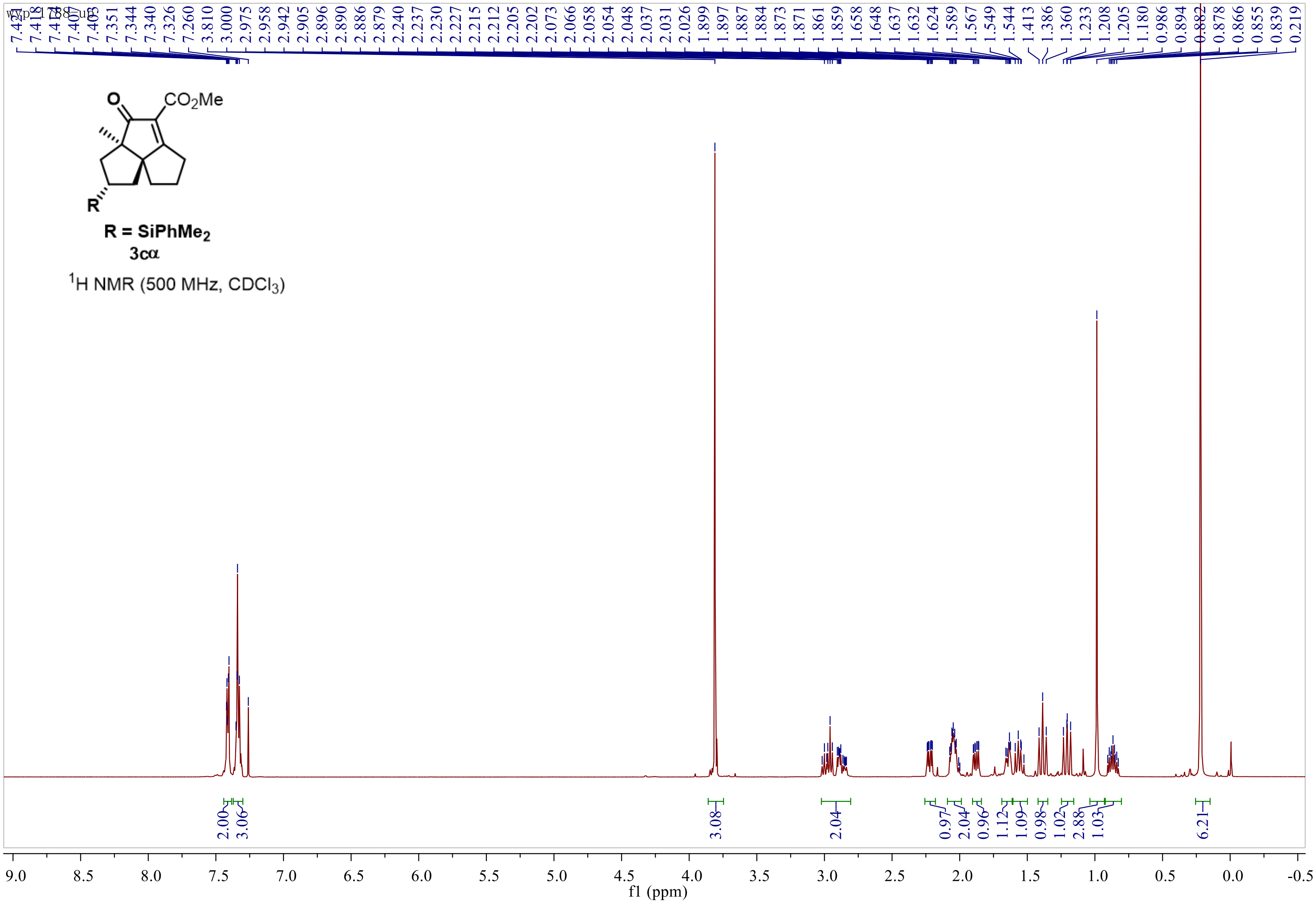

Supplementary Fig. 327. ^{1}H NMR spectra of compound **3cα** in $CDCl_3$

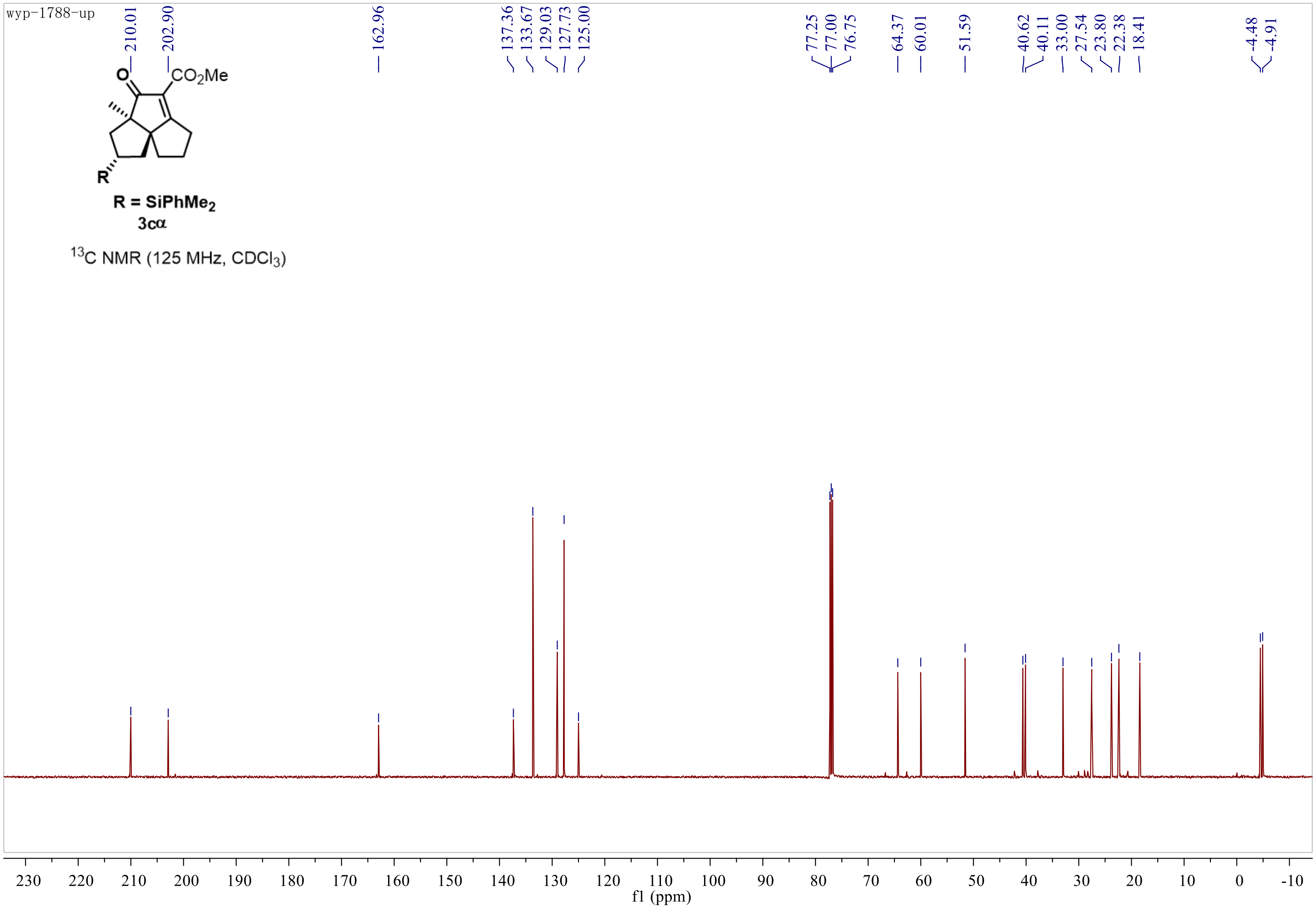

Supplementary Fig. 328. ^{13}C NMR spectra of compound **3cα** in $CDCl_3$

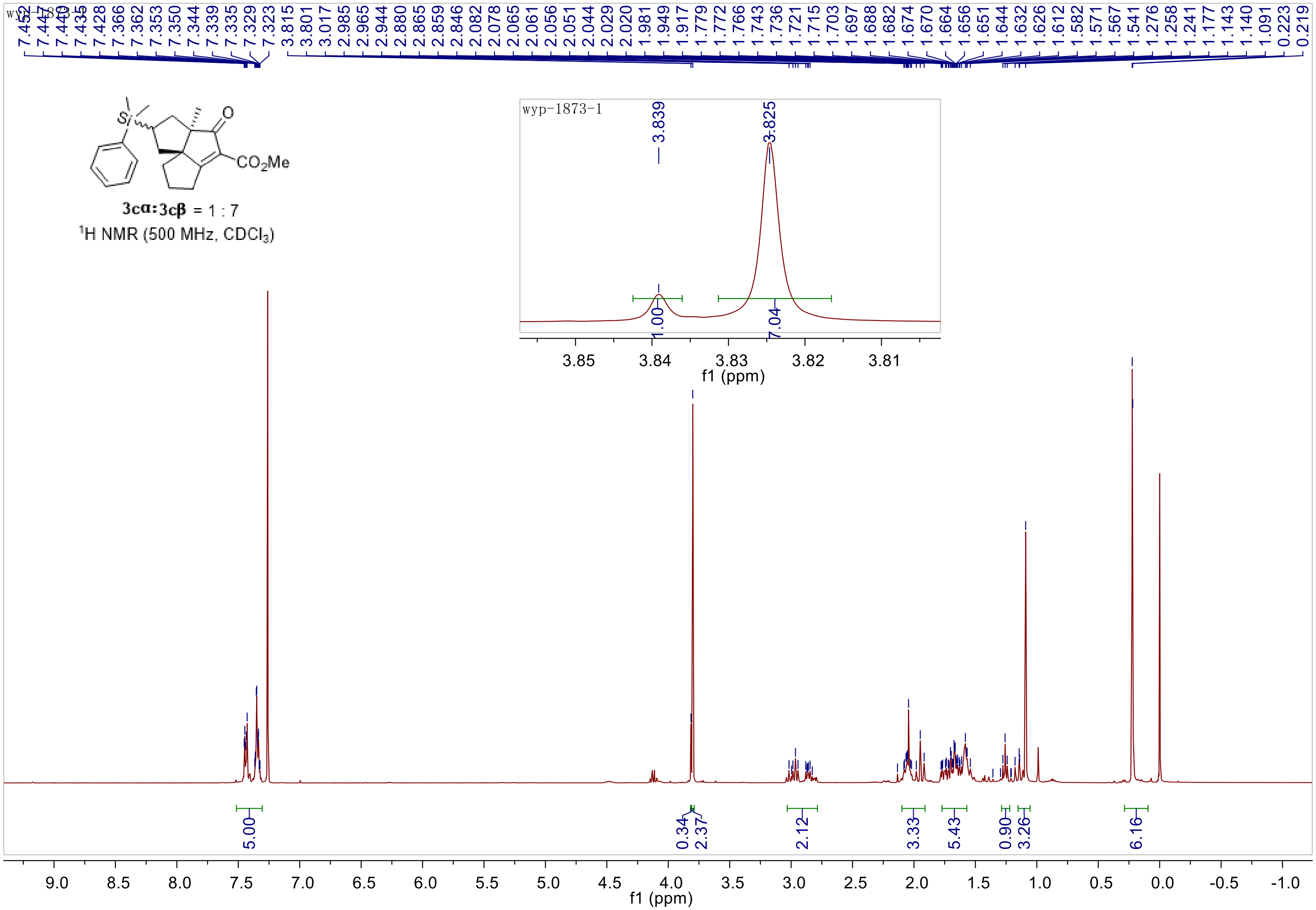

Supplementary Fig. 329. ^{1}H NMR spectra of compound **3cα** and **3cβ** in $CDCl_3$

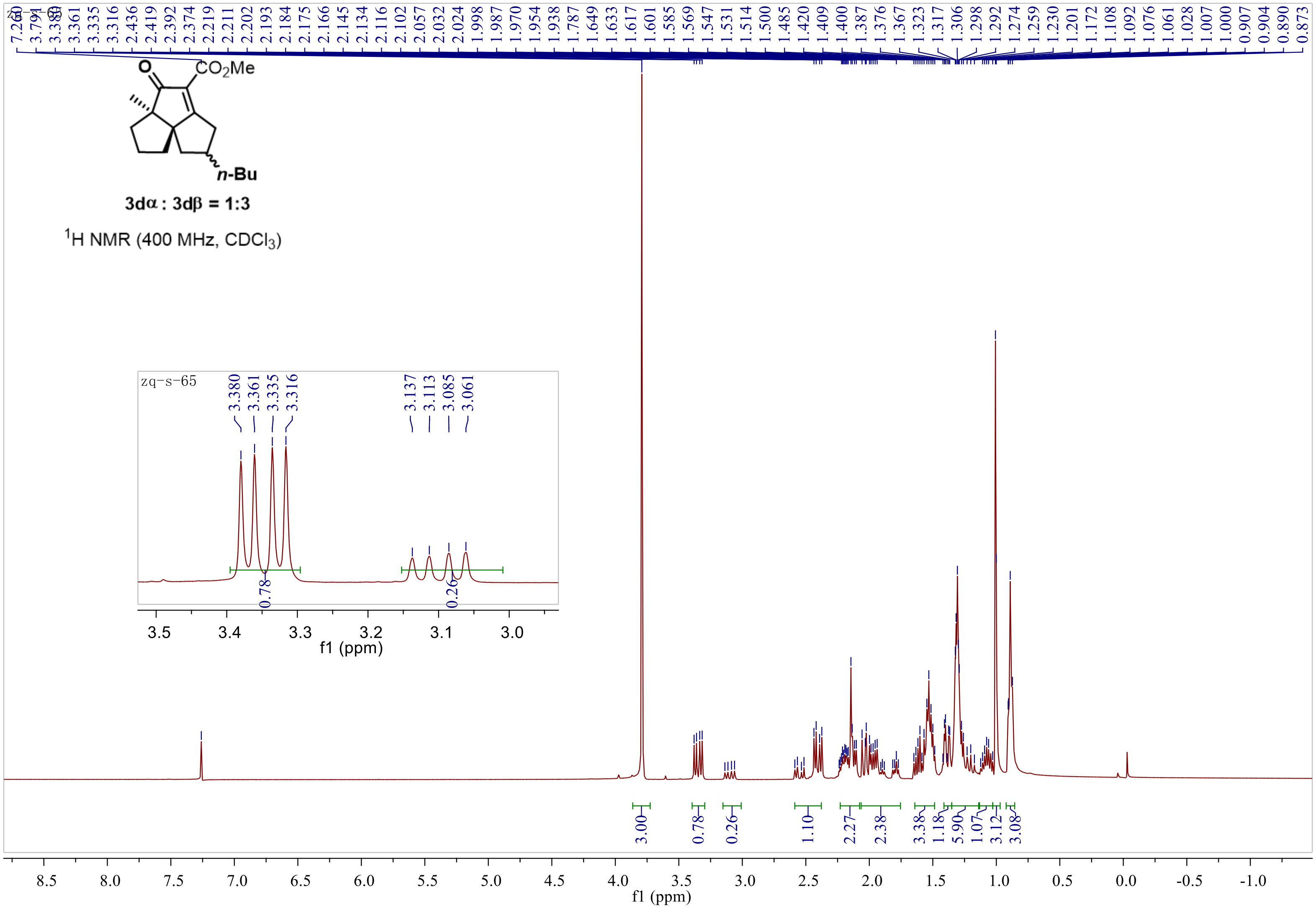

Supplementary Fig. 330. ^{1}H NMR spectra of compound **3dα** and **3dβ** in $CDCl_3$

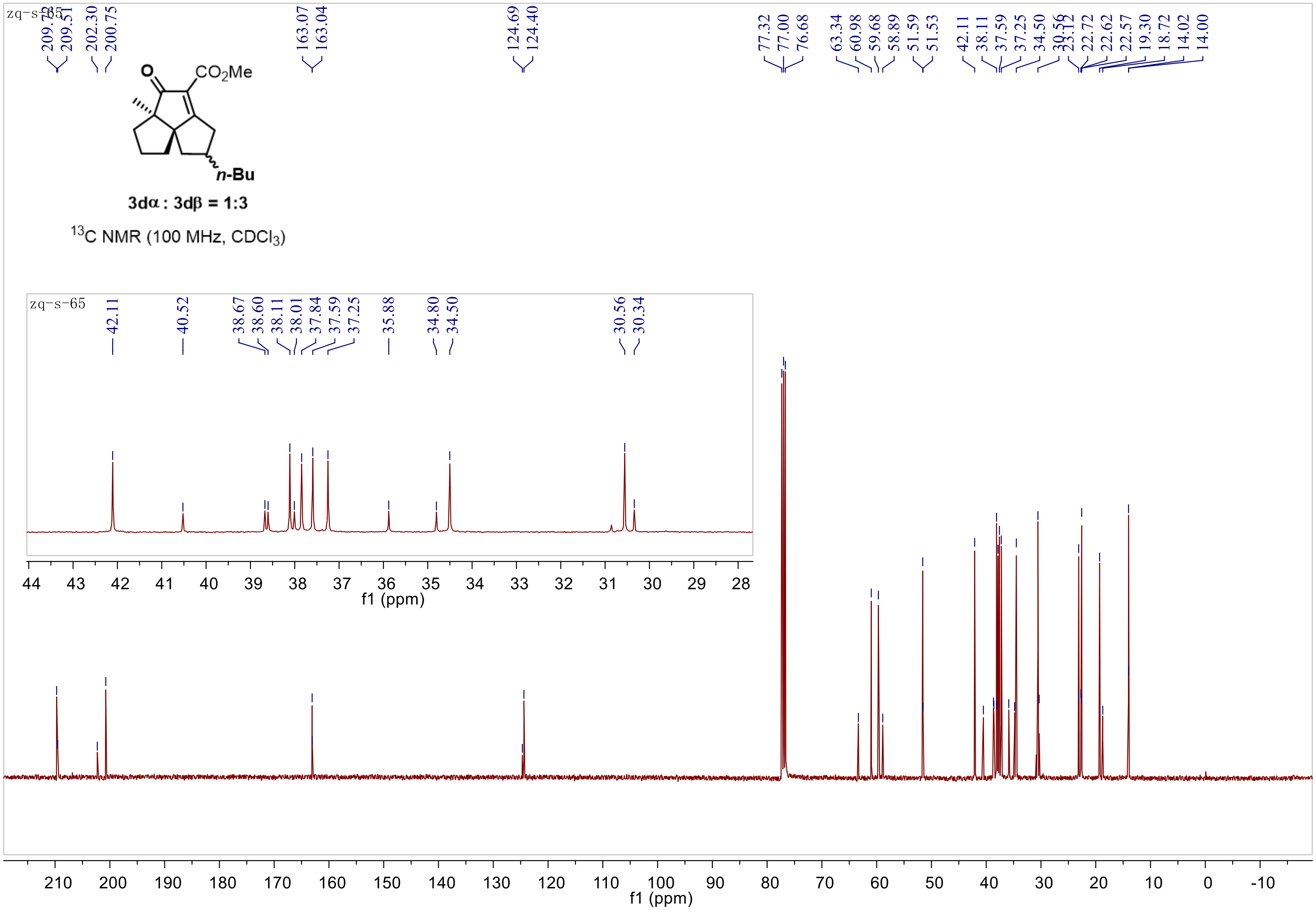

Supplementary Fig. 331. ^{13}C NMR spectra of compound **3dα** and **3dβ** in $CDCl_3$

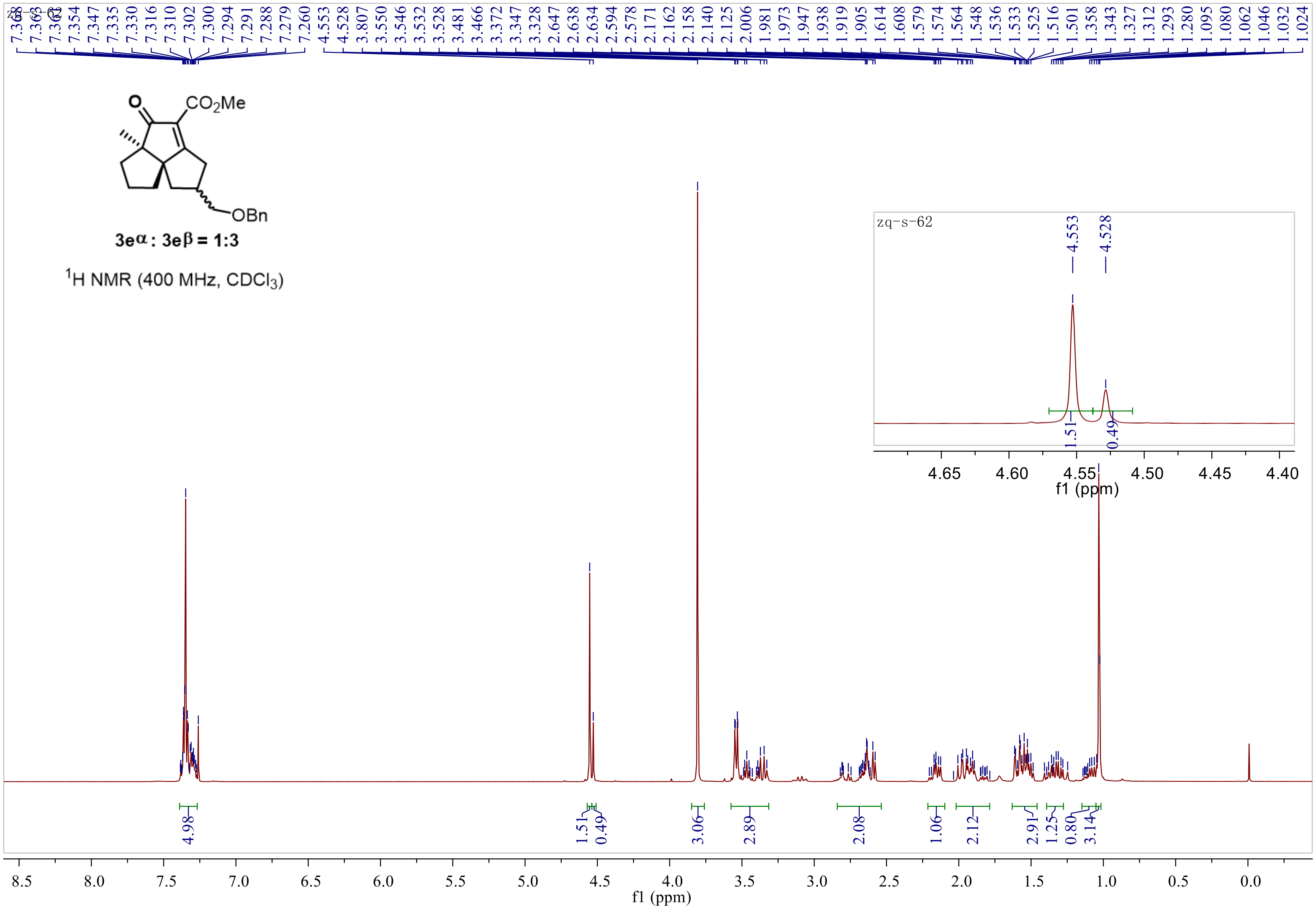

Supplementary Fig. 332. ^{1}H NMR spectra of compound **3eα** and **3eβ** in $CDCl_3$

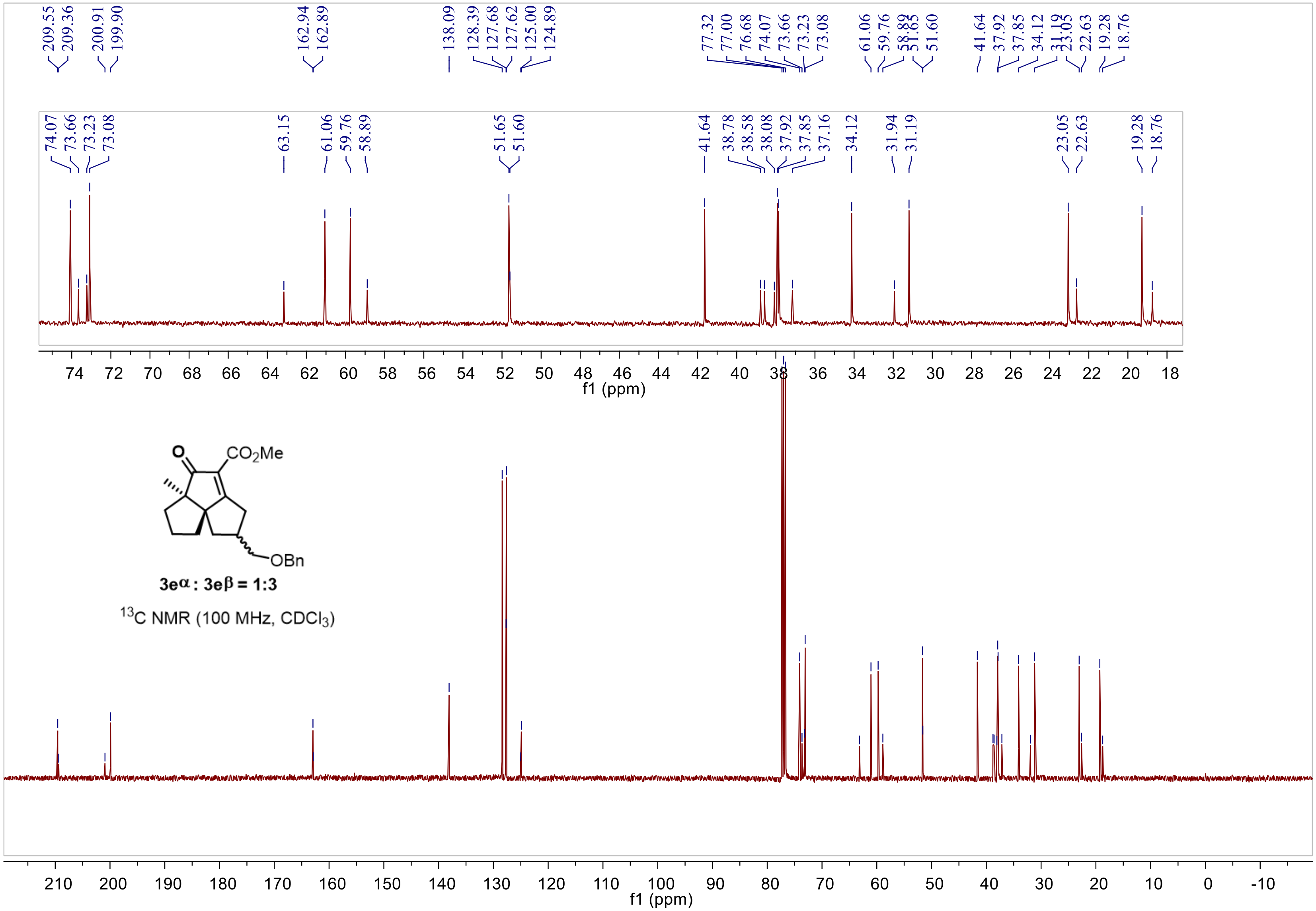

Supplementary Fig. 333. ^{13}C NMR spectra of compound **3eα** and **3eβ** in $CDCl_3$

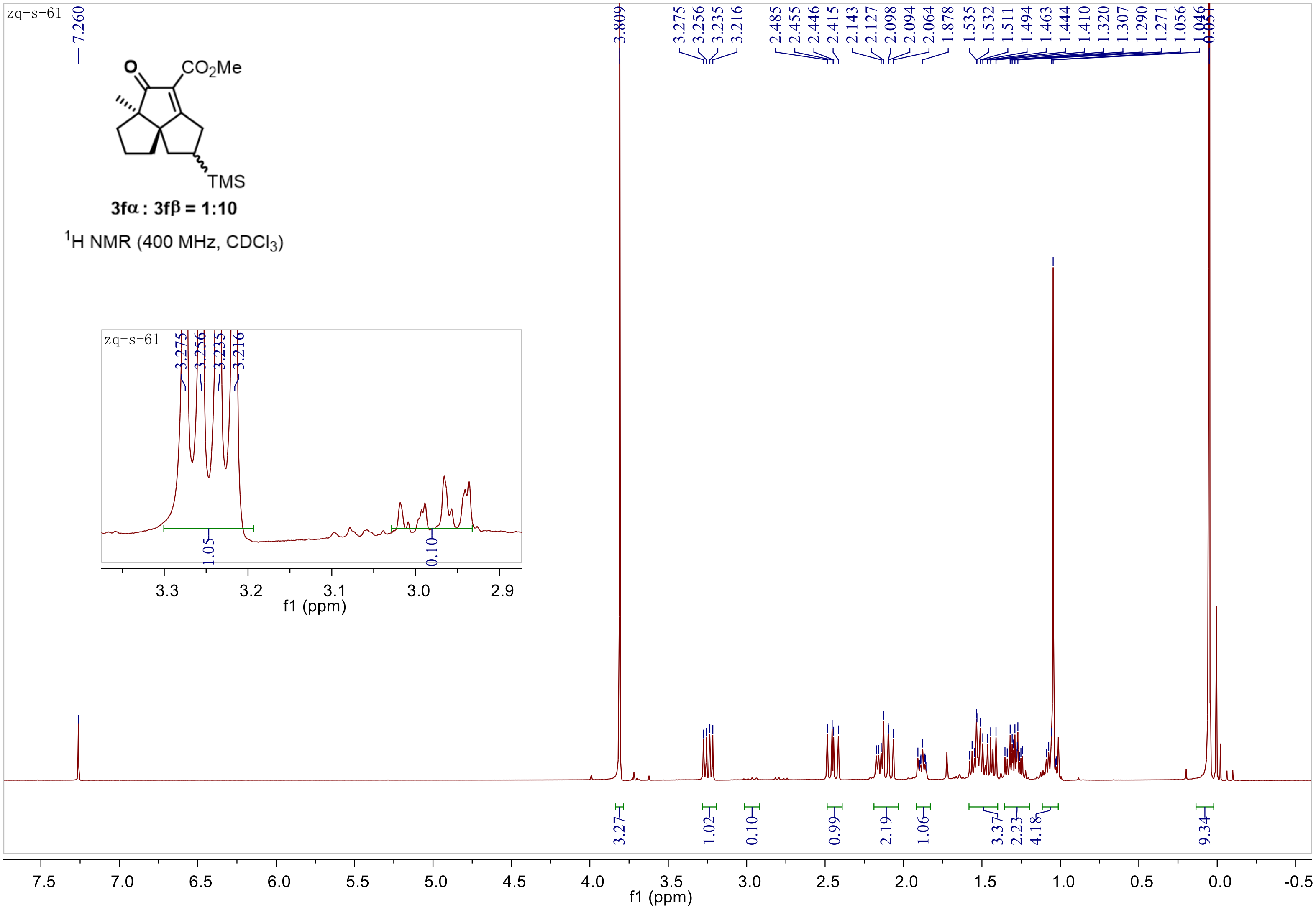

Supplementary Fig. 334. ^{1}H NMR spectra of compound **3fα** and **3fβ** in $CDCl_3$

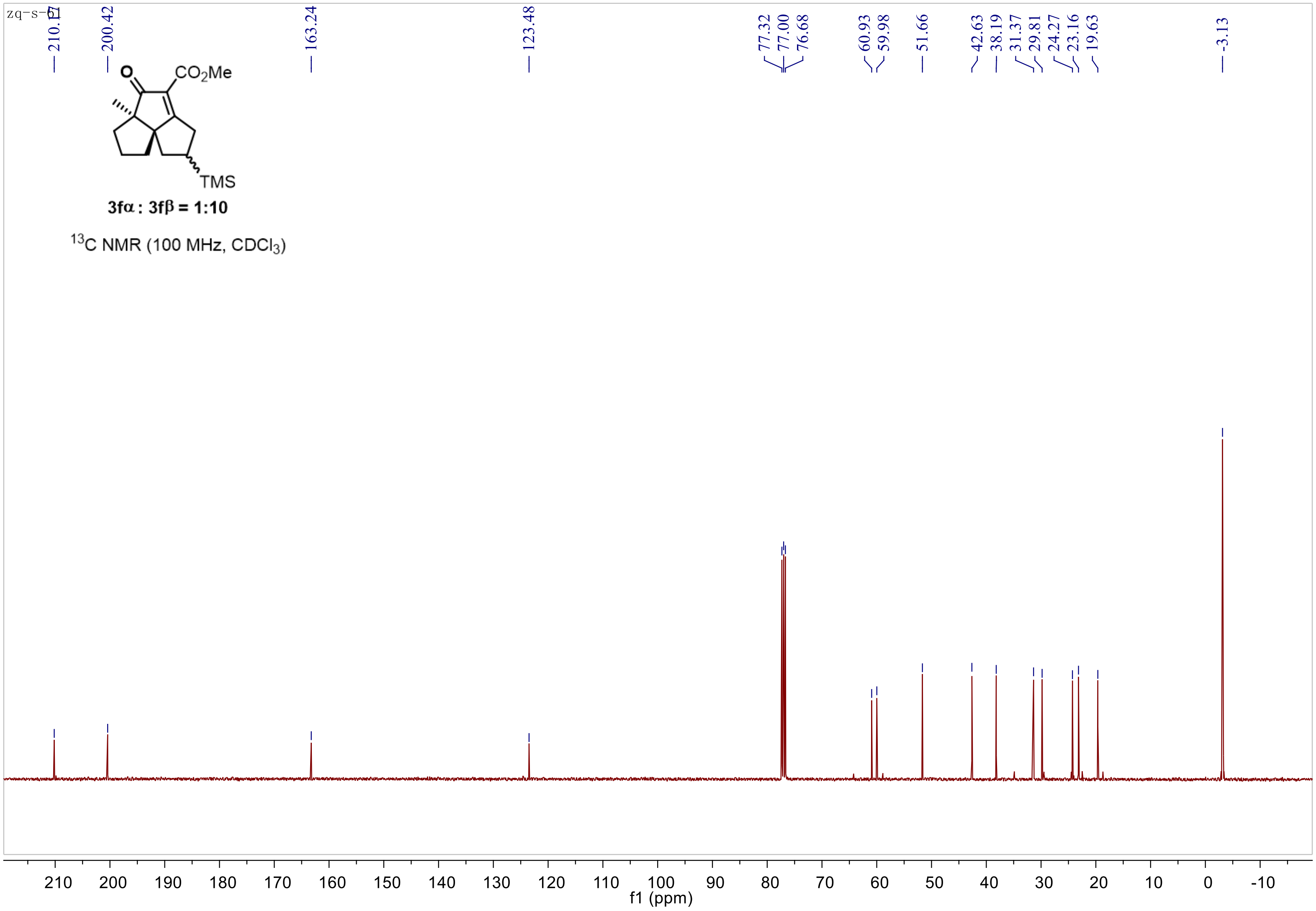

Supplementary Fig. 335. ^{13}C NMR spectra of compound **3fα** and **3fβ** in $CDCl_3$

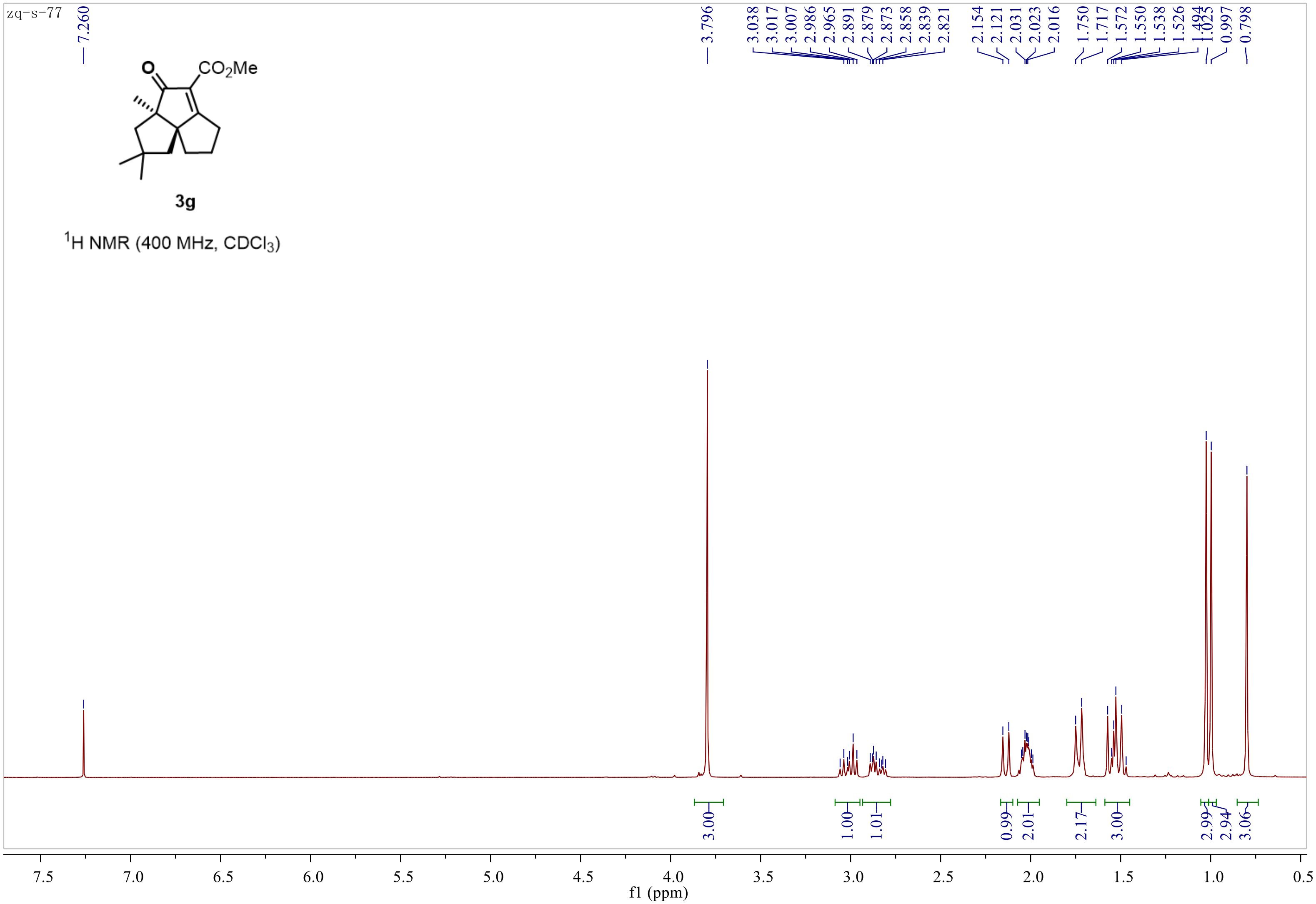

Supplementary Fig. 336. ^{1}H NMR spectra of compound **3g** in $CDCl_3$

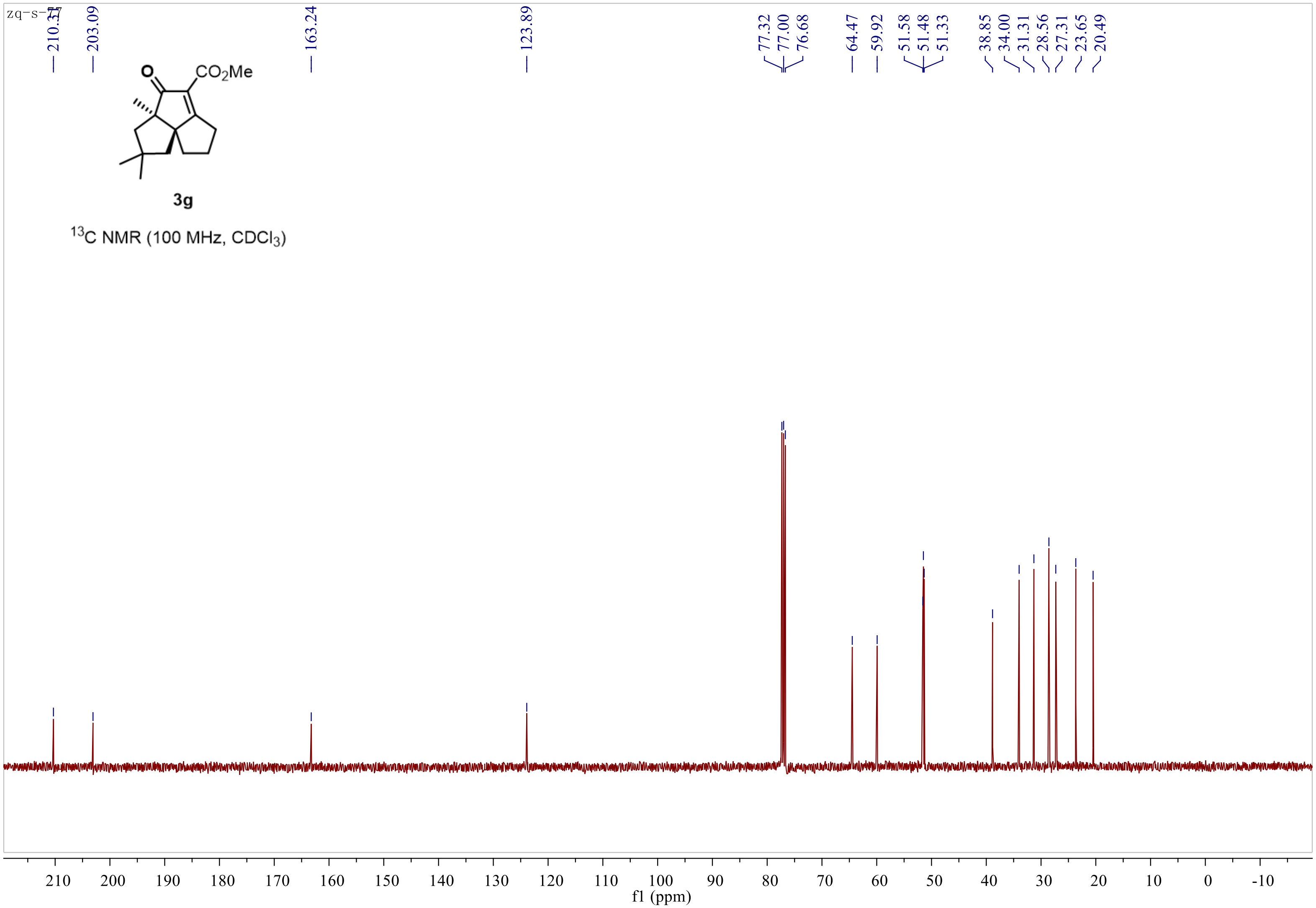

Supplementary Fig. 337. ^{13}C NMR spectra of compound **3g** in $CDCl_3$

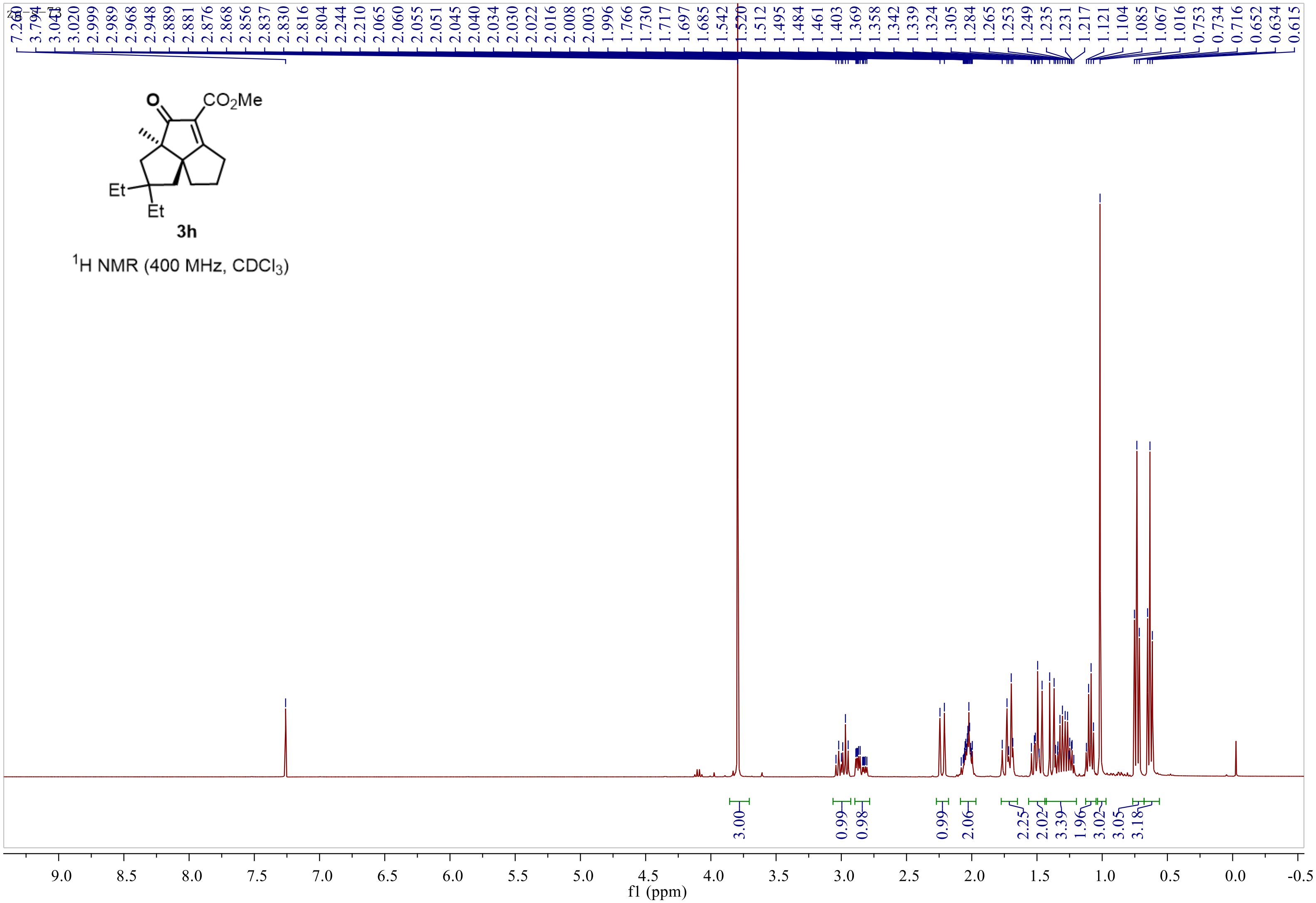

Supplementary Fig. 338. ^{1}H NMR spectra of compound **3h** in $CDCl_3$

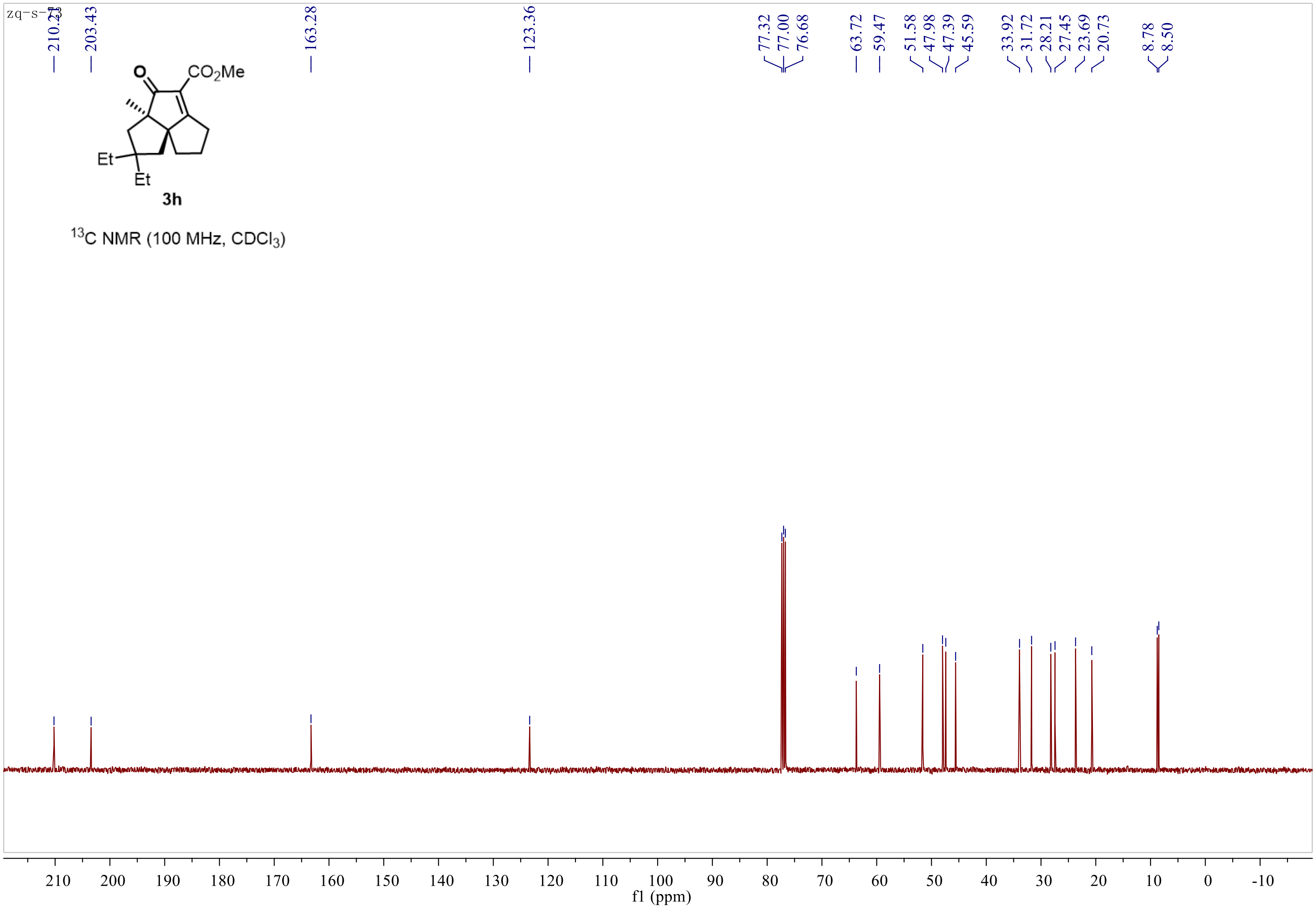

Supplementary Fig. 339. ^{13}C NMR spectra of compound **3h** in $CDCl_3$

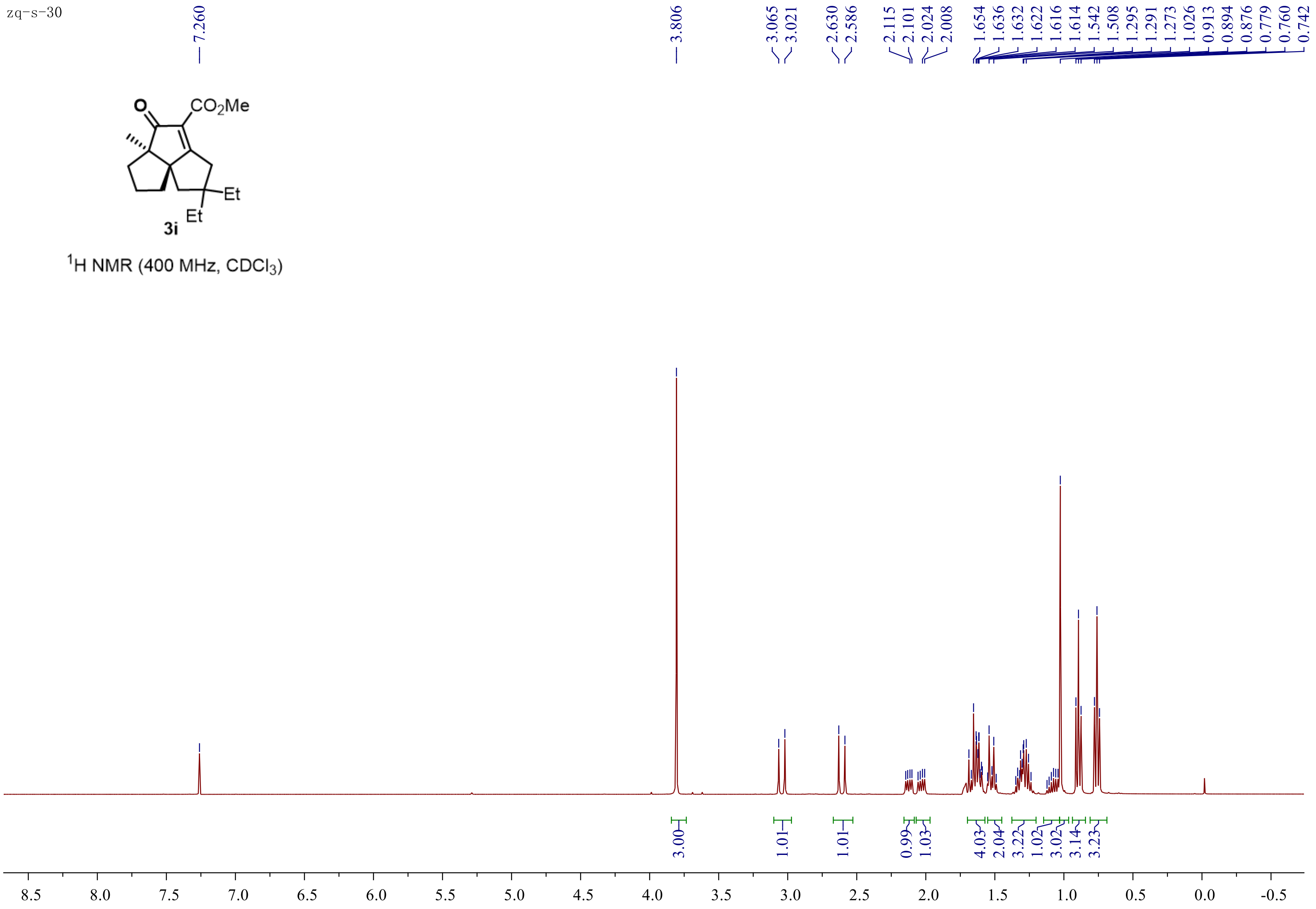

Supplementary Fig. 340. ^{1}H NMR spectra of compound **3i** in $CDCl_3$

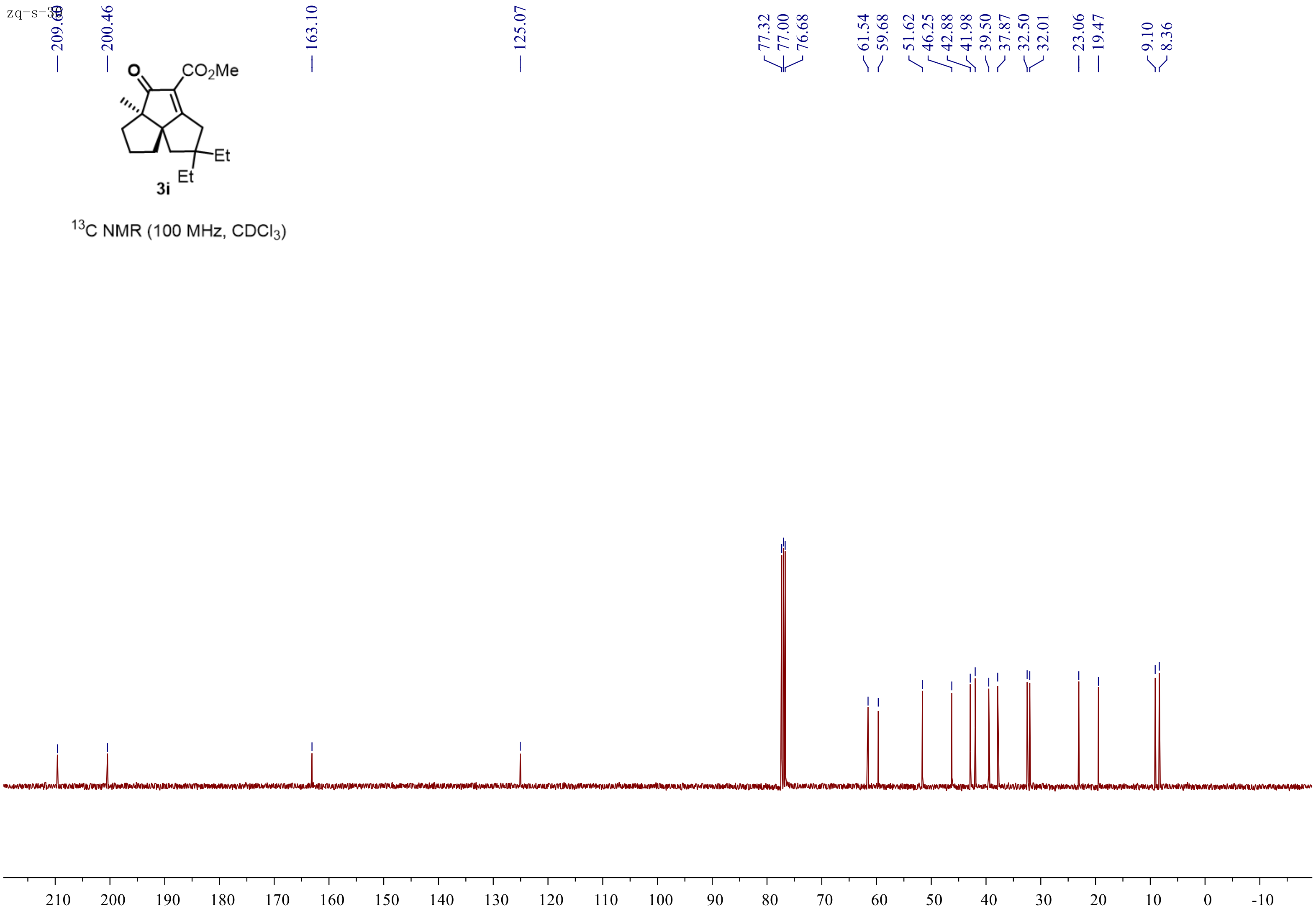

Supplementary Fig. 341. ^{13}C NMR spectra of compound **3i** in $CDCl_3$

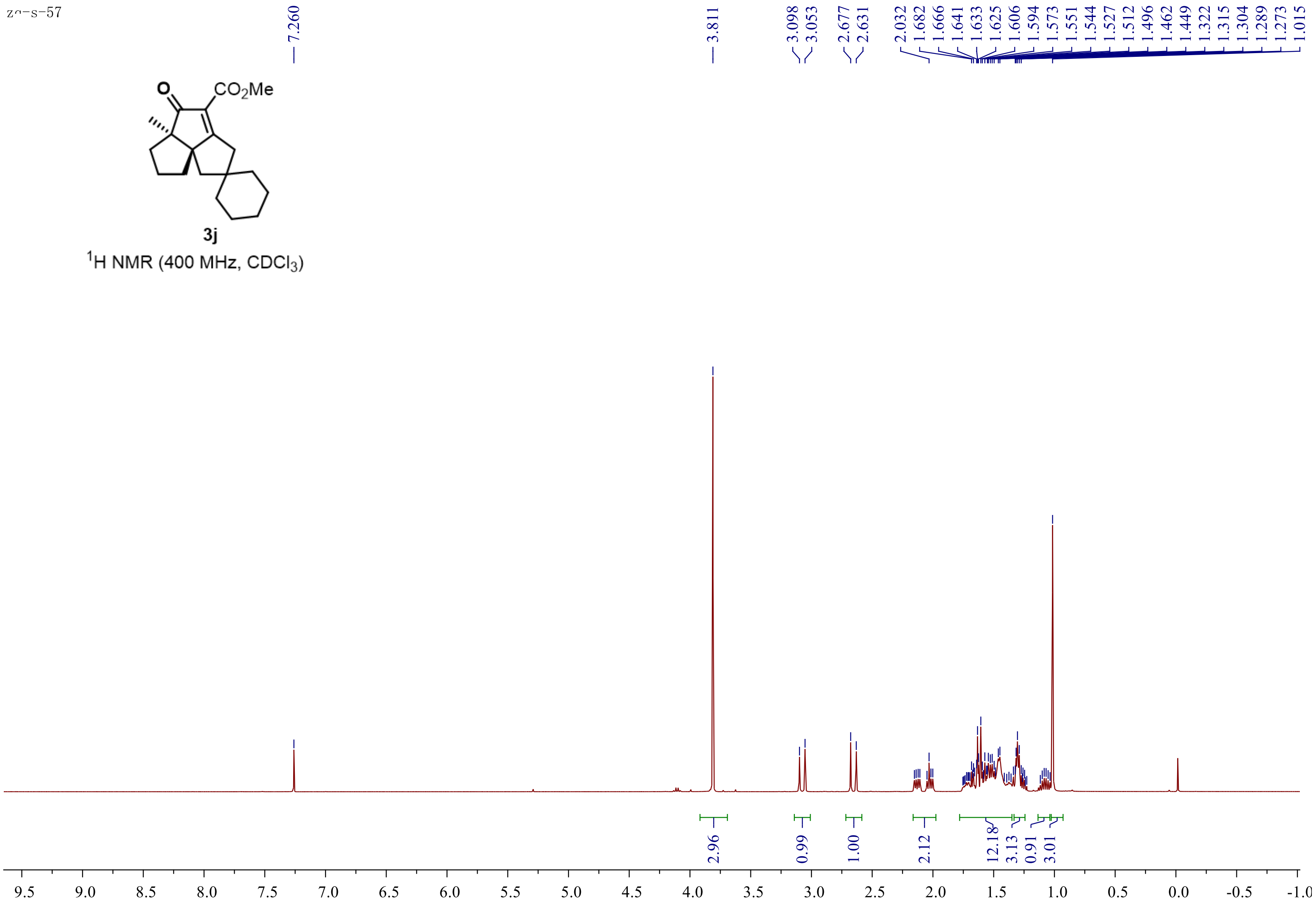

Supplementary Fig. 342. ^{1}H NMR spectra of compound **3j** in $CDCl_3$

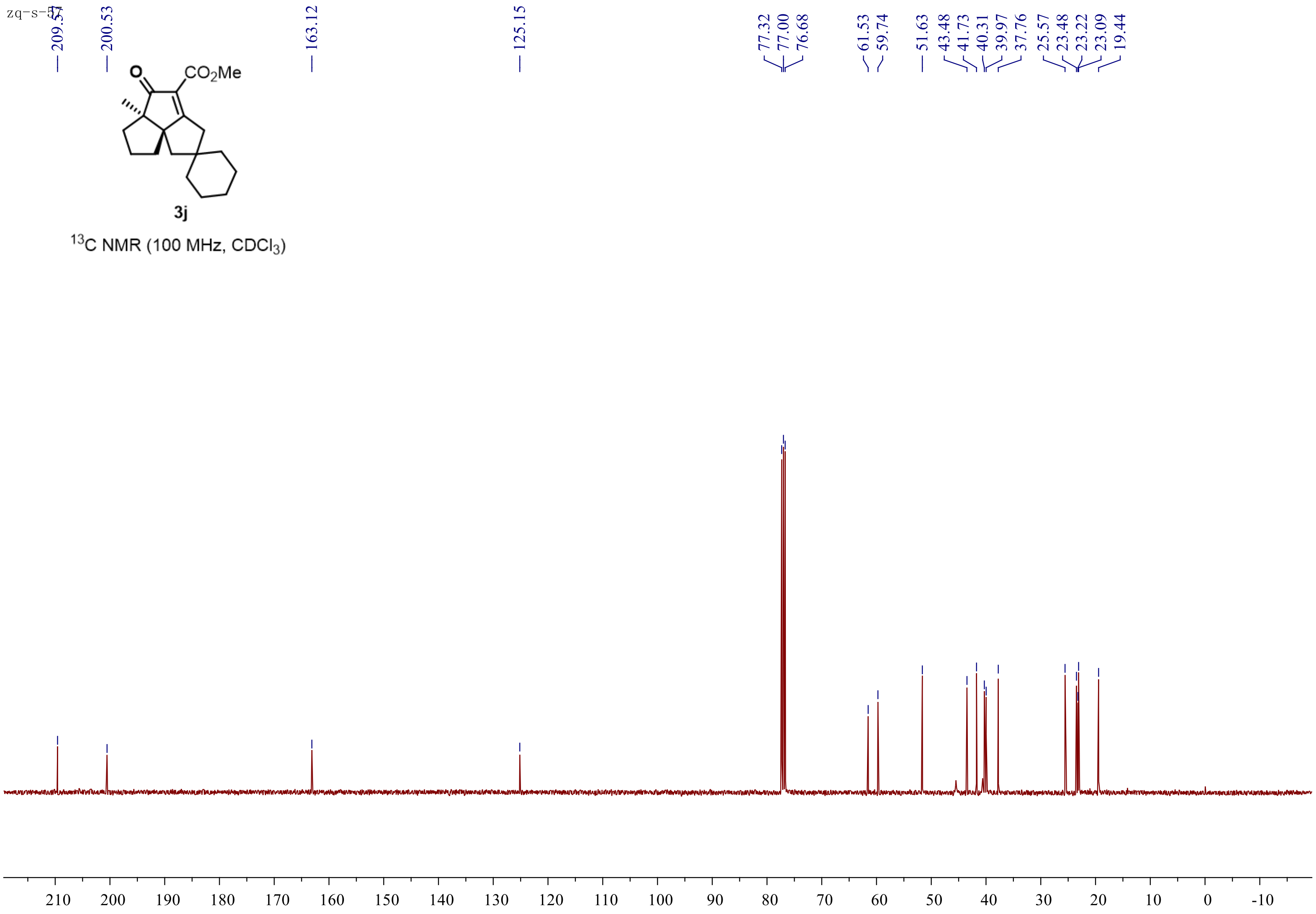

Supplementary Fig. 343. ^{13}C NMR spectra of compound **3j** in $CDCl_3$

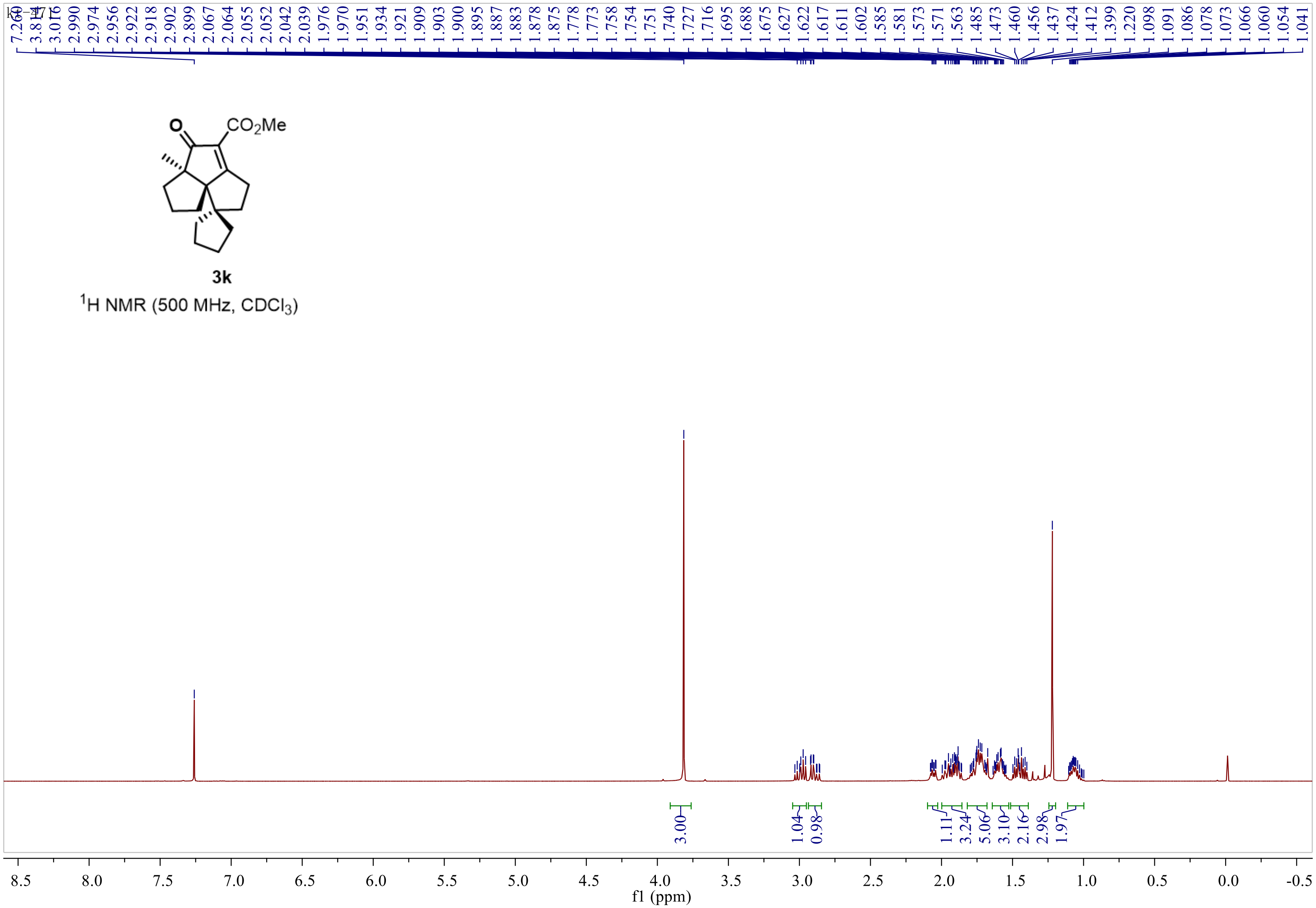

Supplementary Fig. 344. ^{1}H NMR spectra of compound **3k** in $CDCl_3$

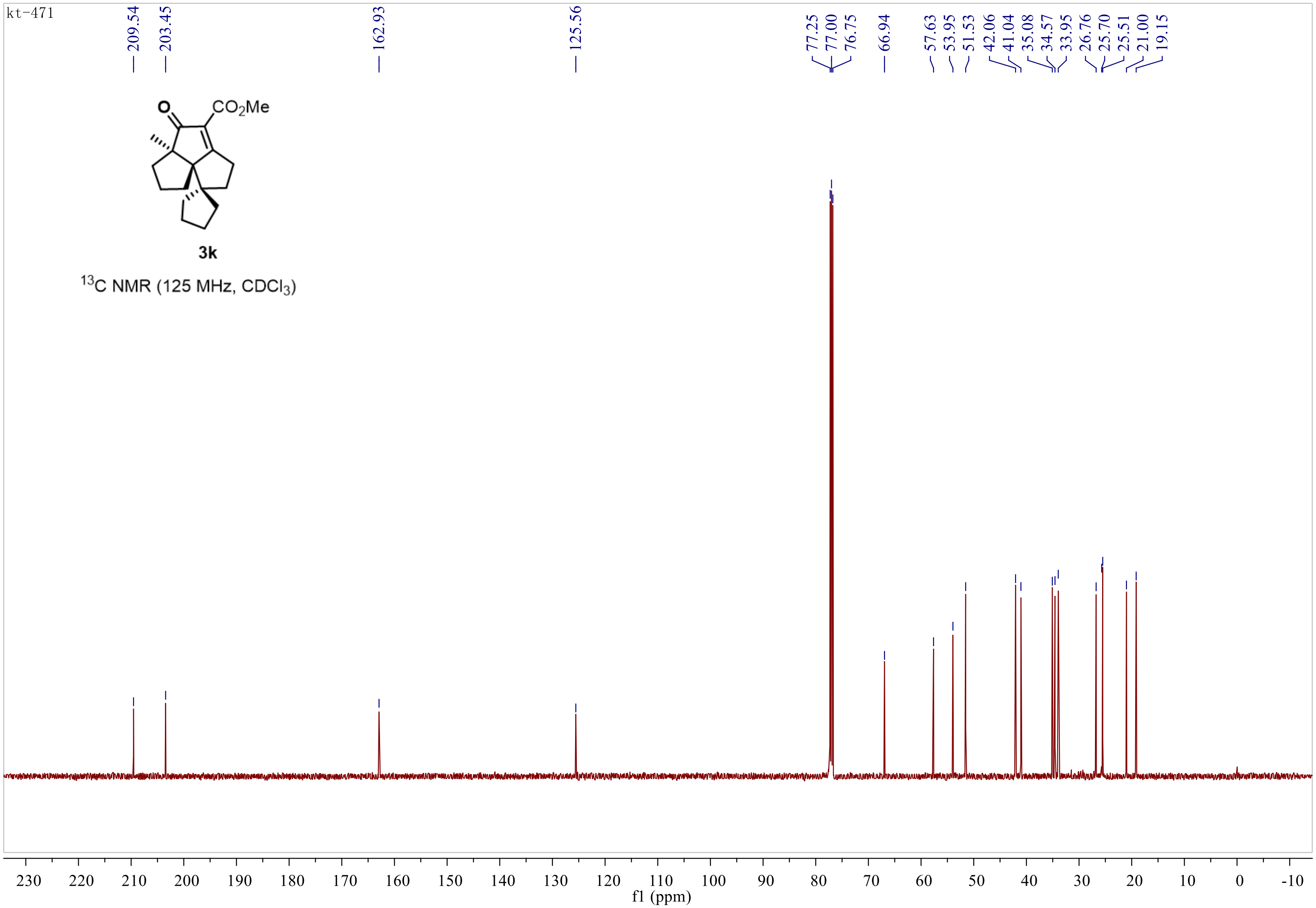

Supplementary Fig. 345. ^{13}C NMR spectra of compound **3k** in $CDCl_3$

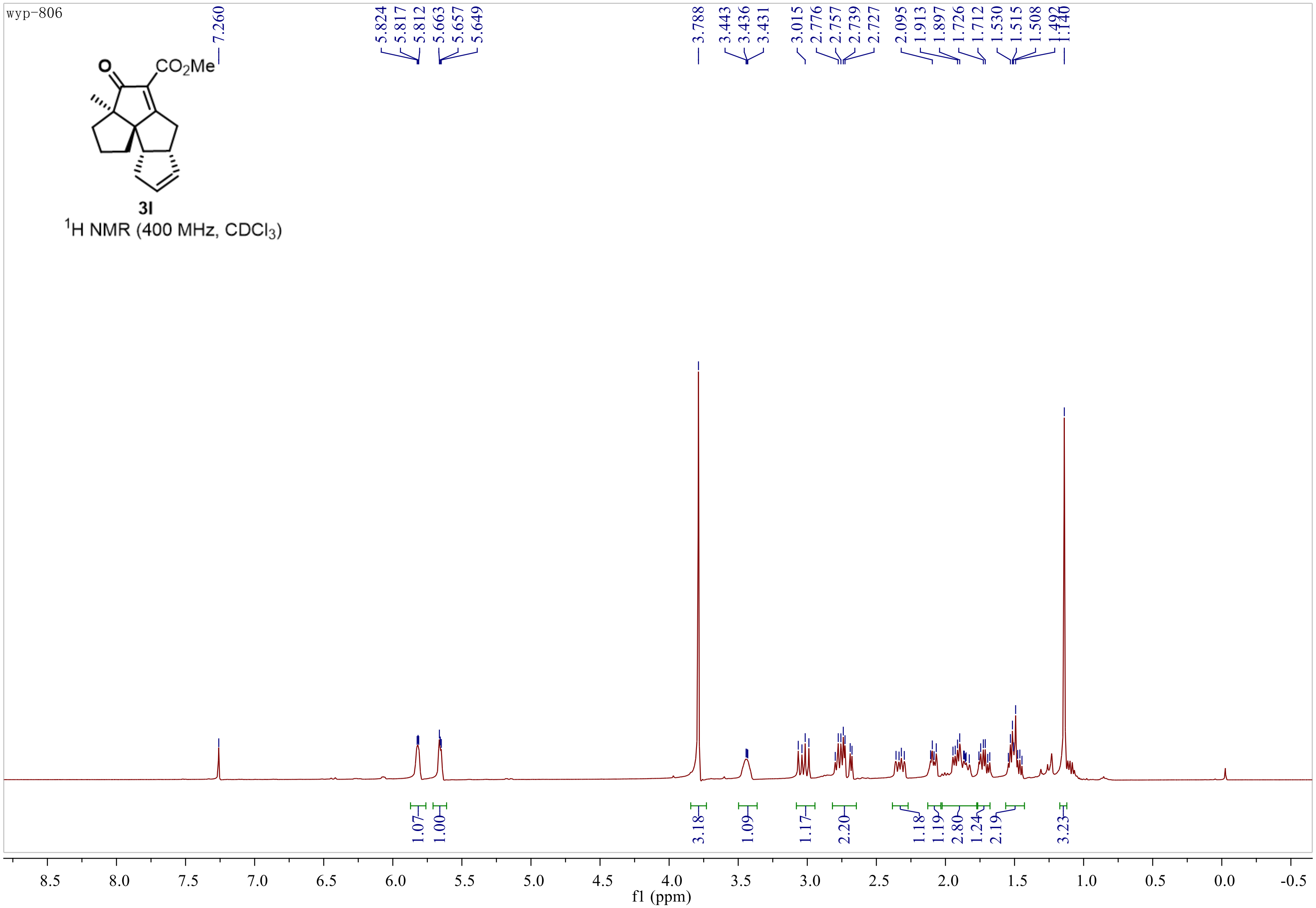

Supplementary Fig. 346. ^1H NMR spectra of compound **3l** in $CDCl_3$

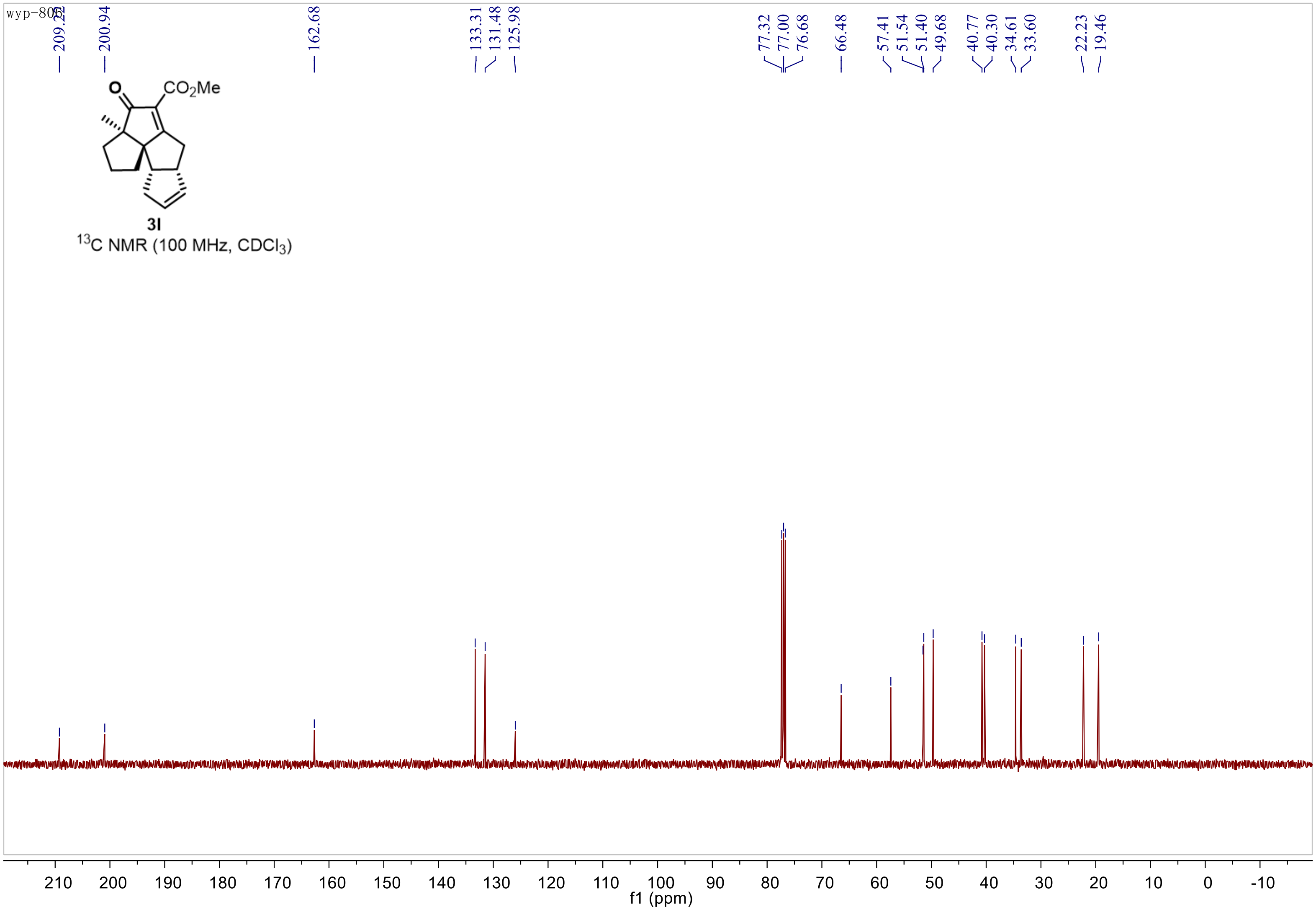

Supplementary Fig. 347. ^{13}C NMR spectra of compound **3l** in $CDCl_3$

=

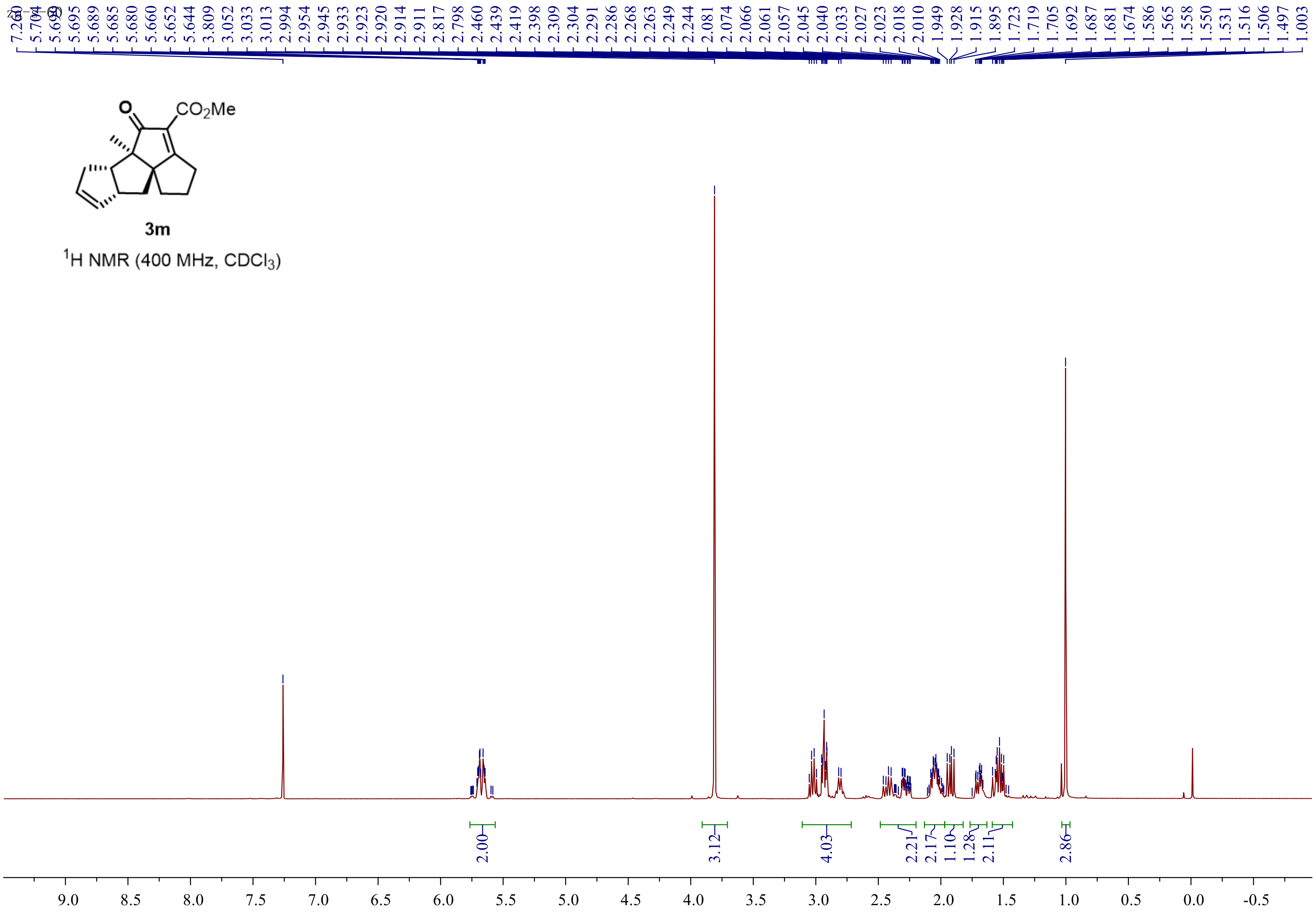

Supplementary Fig. 348. ^{1}H NMR spectra of compound **3m** in $CDCl_3$

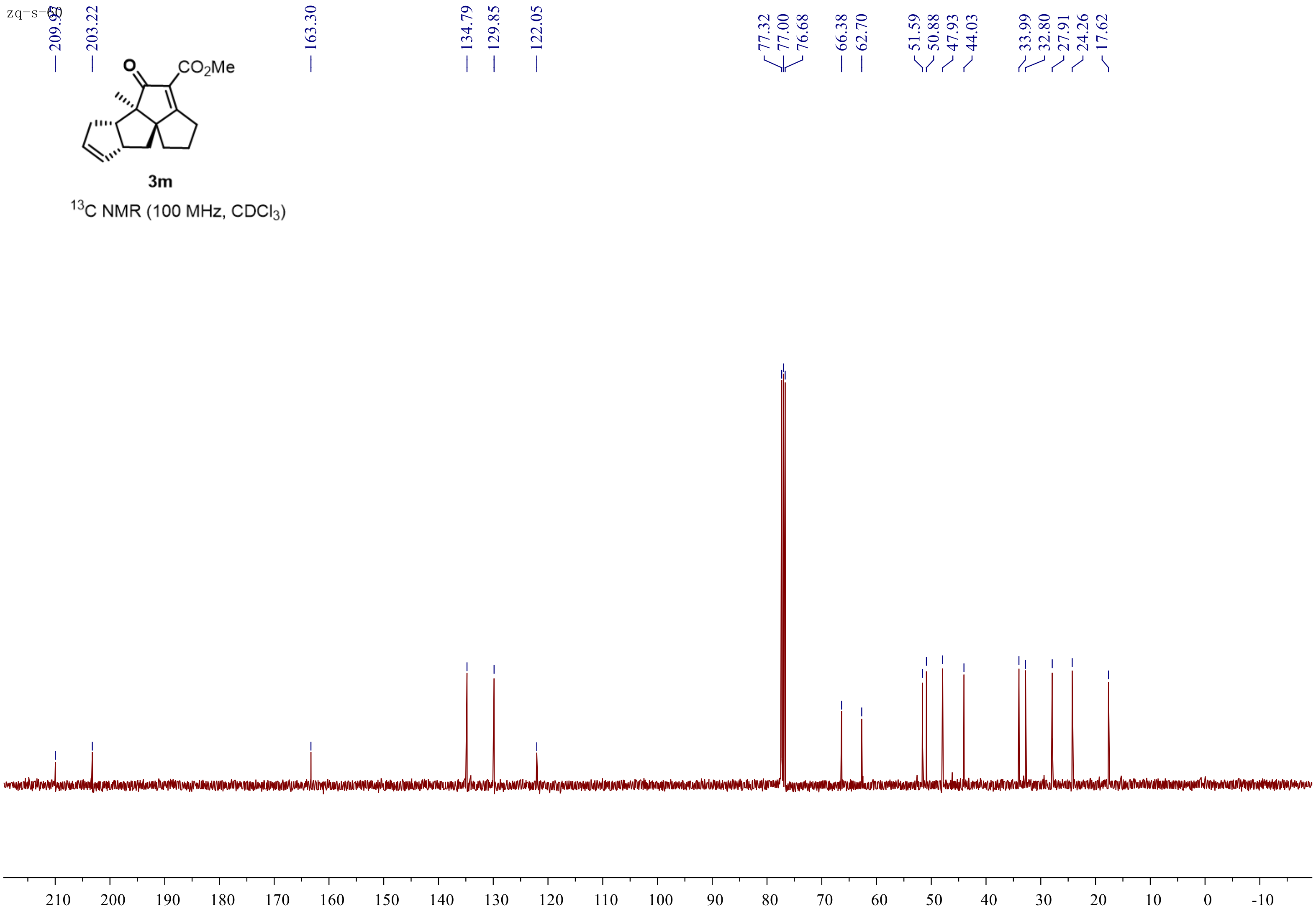

Supplementary Fig. 349. ^{13}C NMR spectra of compound **3m** in $CDCl_3$

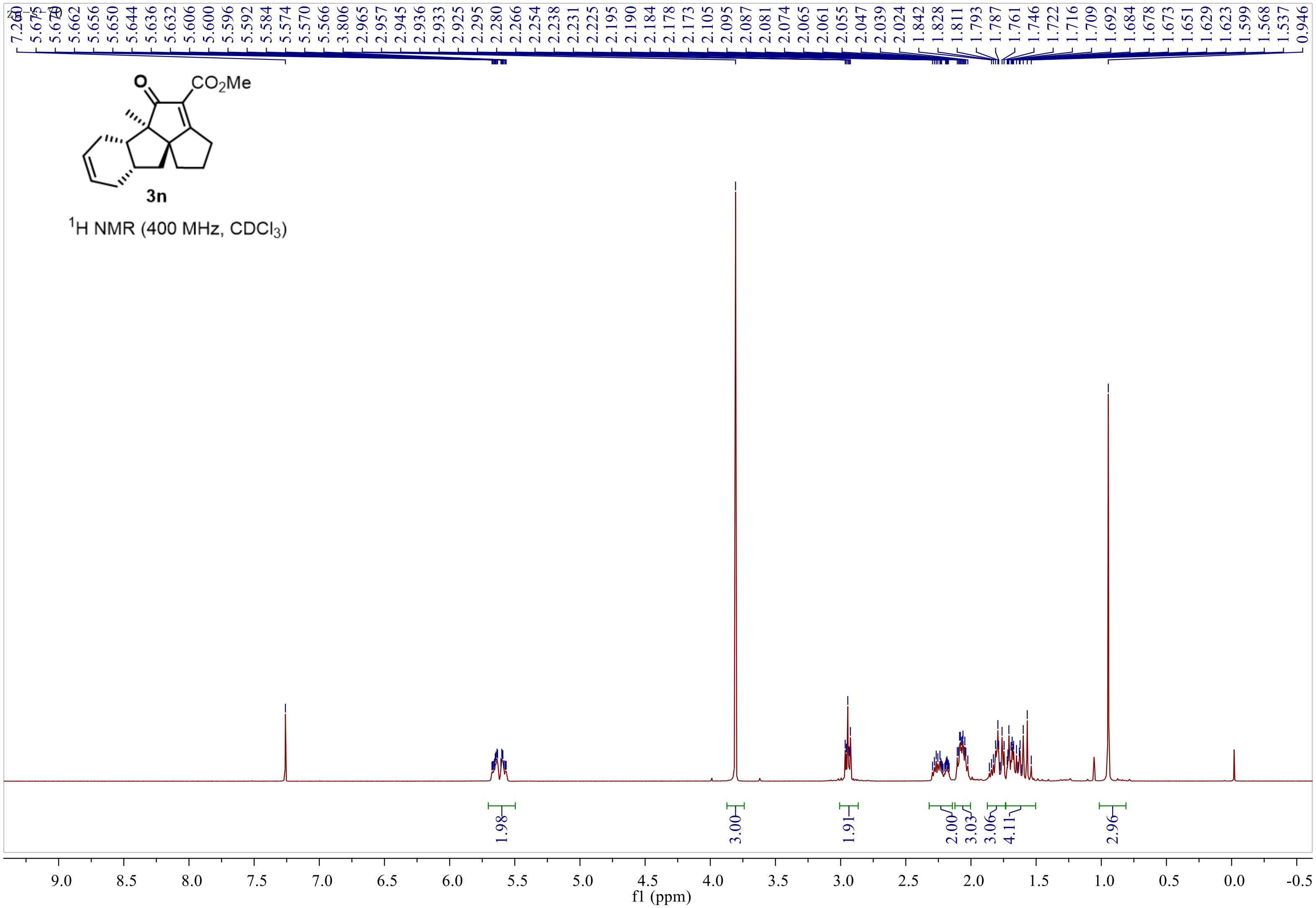

Supplementary Fig. 350. ^{1}H NMR spectra of compound **3n** in $CDCl_3$

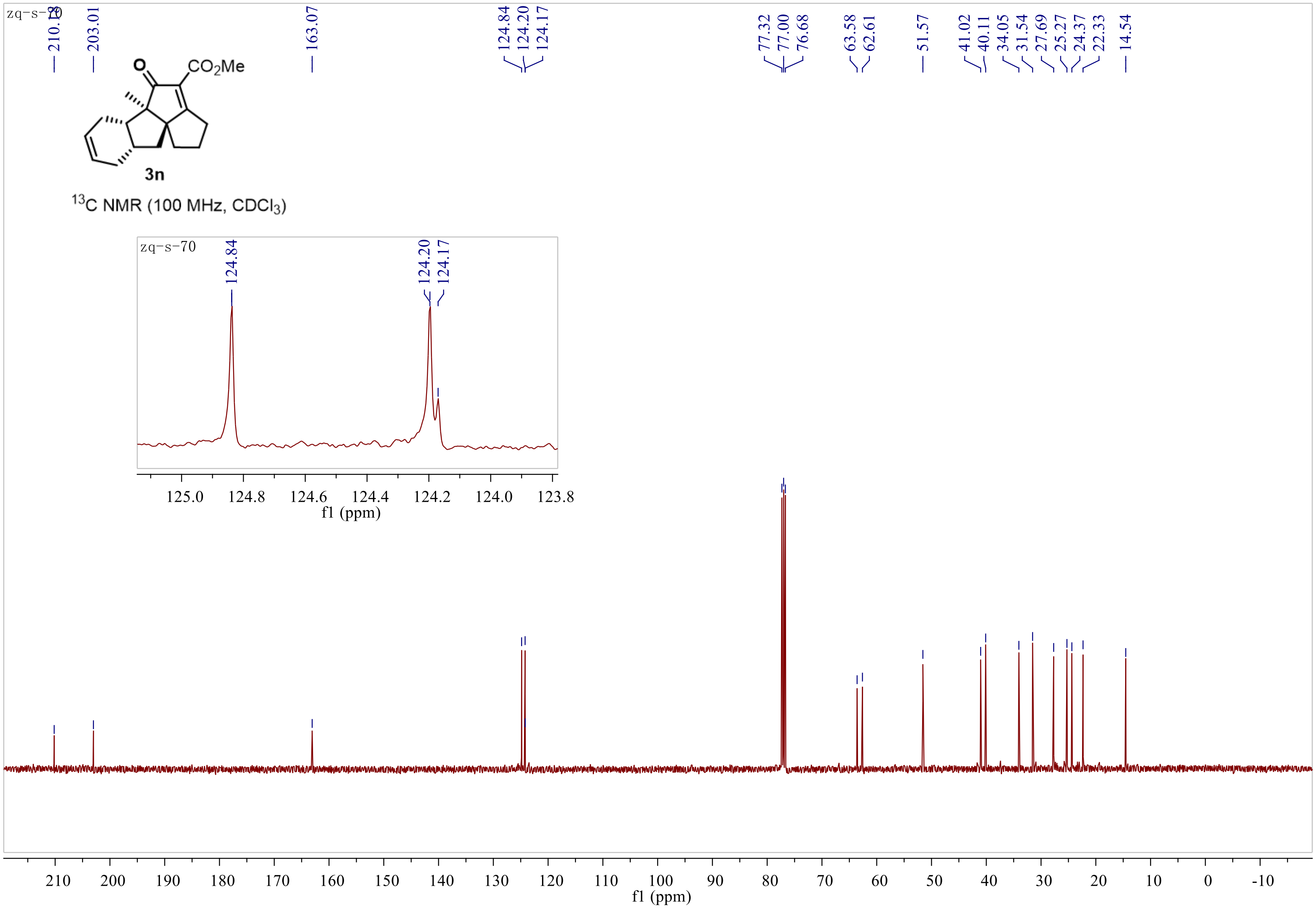

Supplementary Fig. 351. ^{13}C NMR spectra of compound **3n** in $CDCl_3$

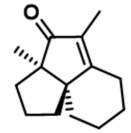

3o

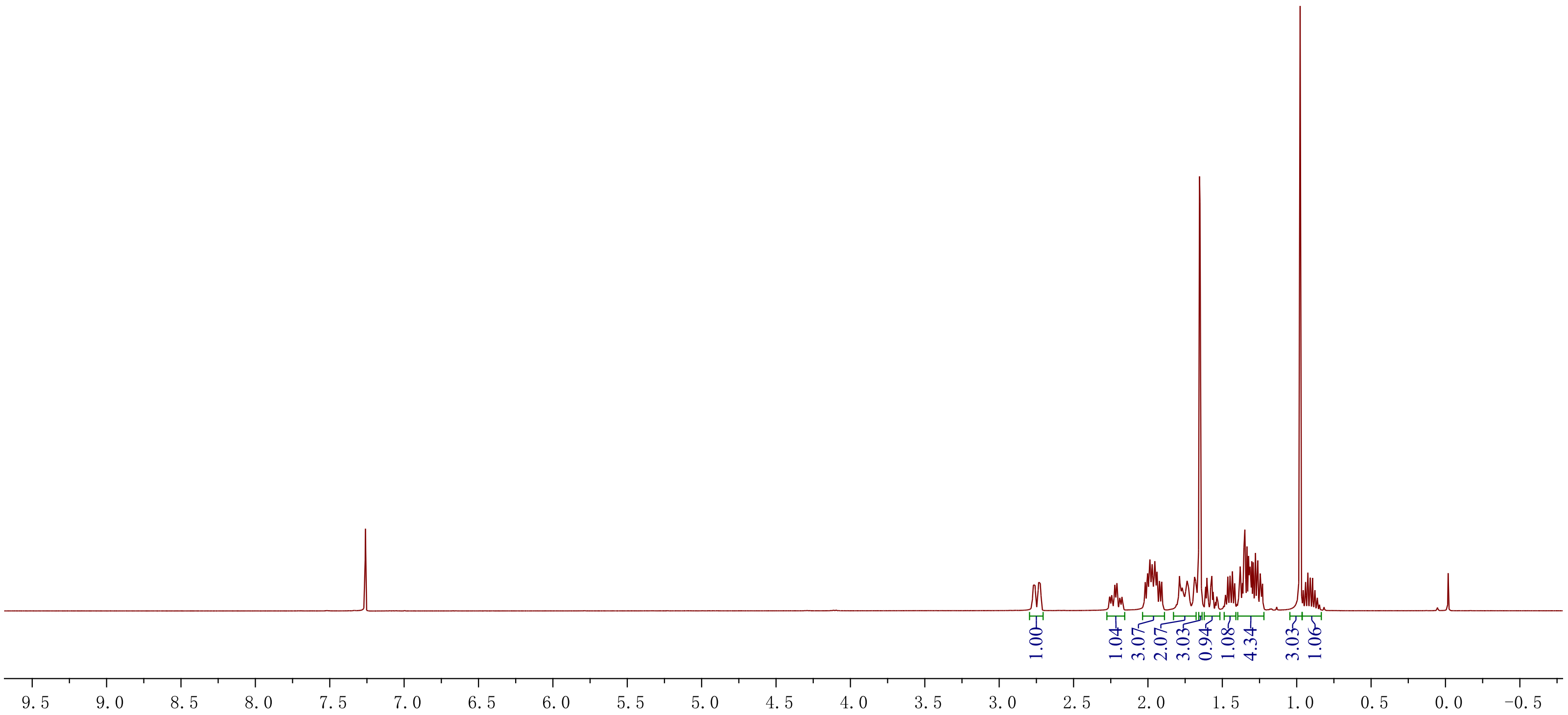

Supplementary Fig. 352. ^{1}H NMR spectra of compound **3o** in CDCl$_3$

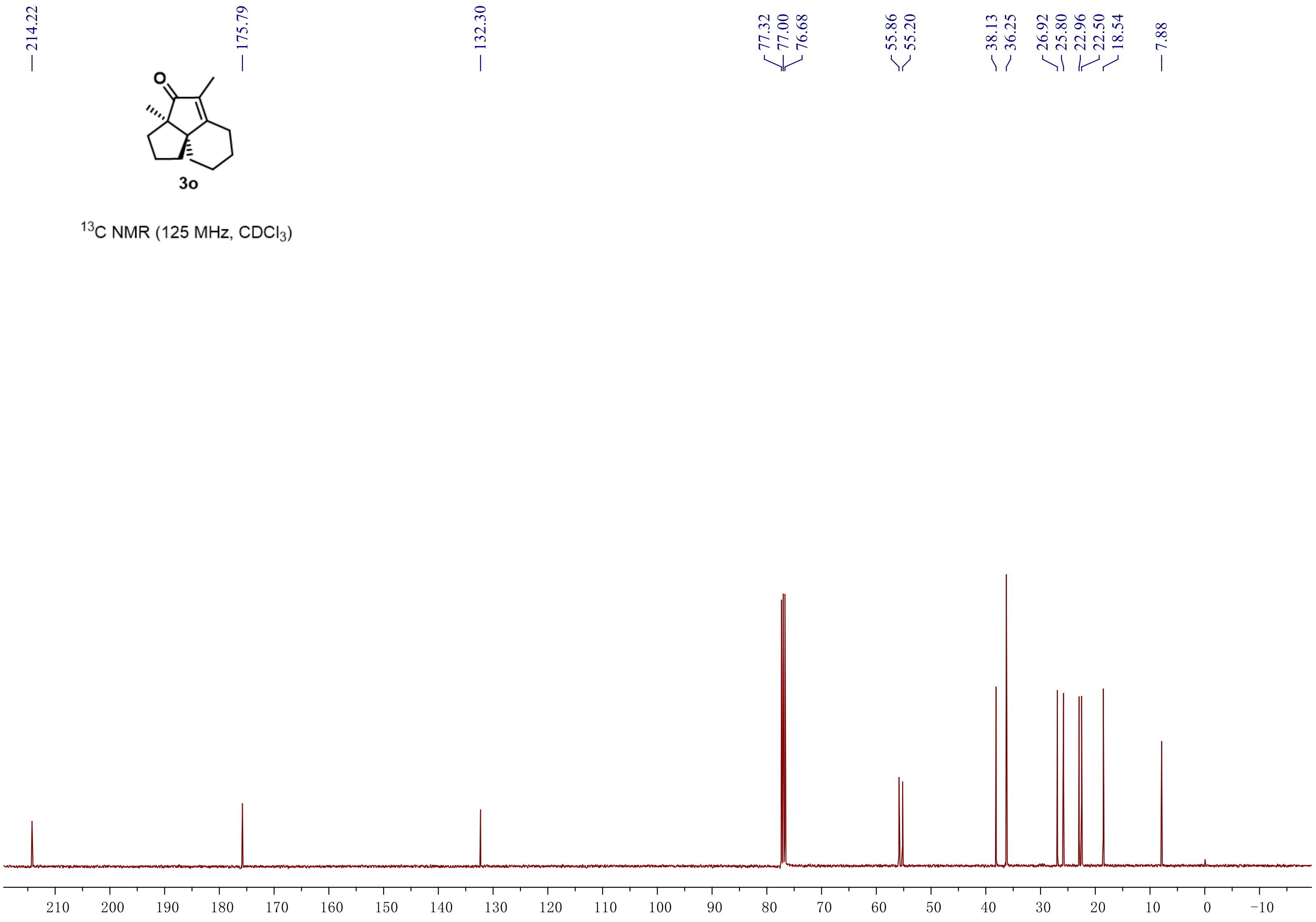

^{13}C NMR (125 MHz, $CDCl_3$)

Supplementary Fig. 353. ^{13}C NMR spectra of compound **3o** in $CDCl_3$

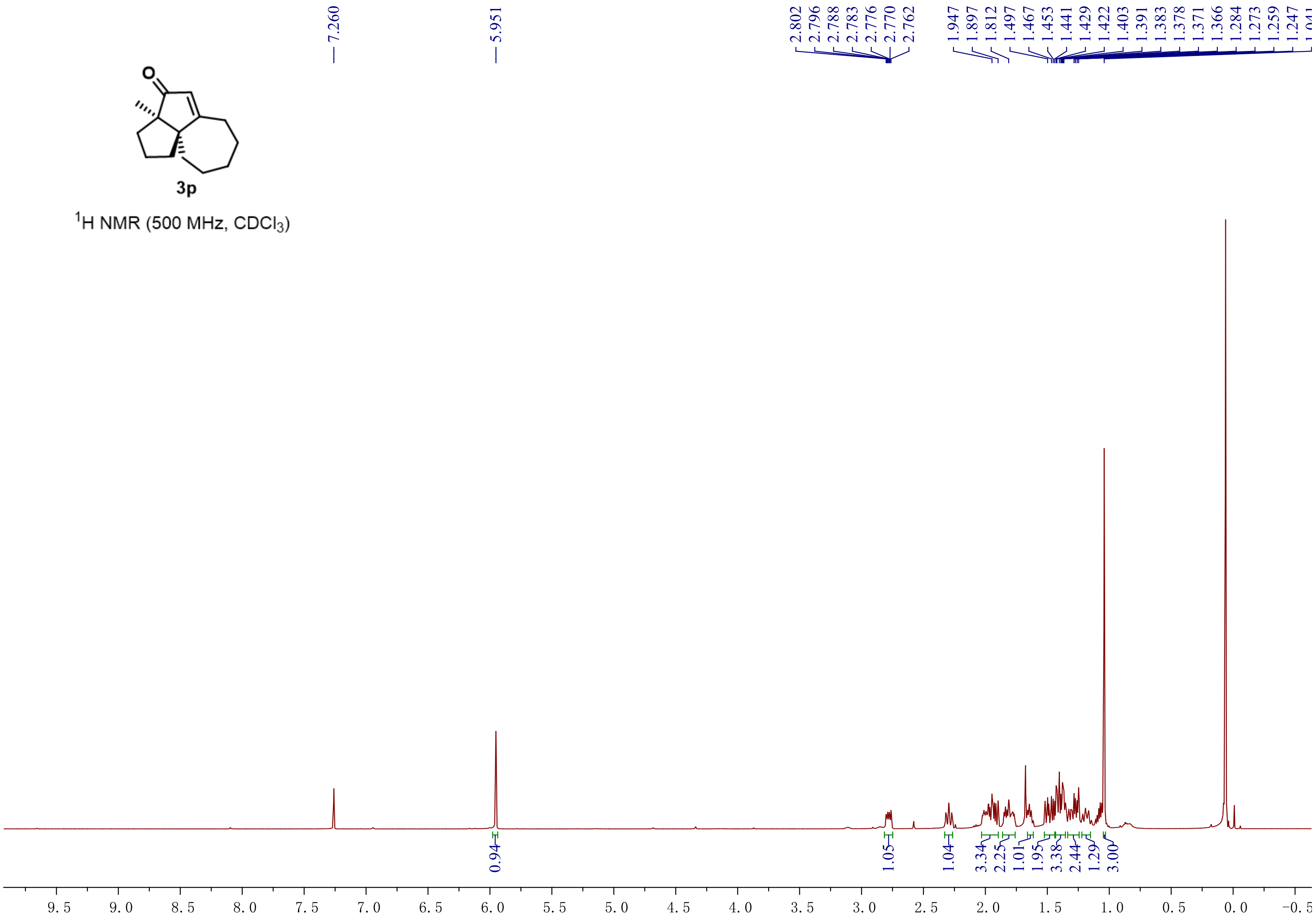

Supplementary Fig. 354. ^{1}H NMR spectra of compound **3p** in $CDCl_3$

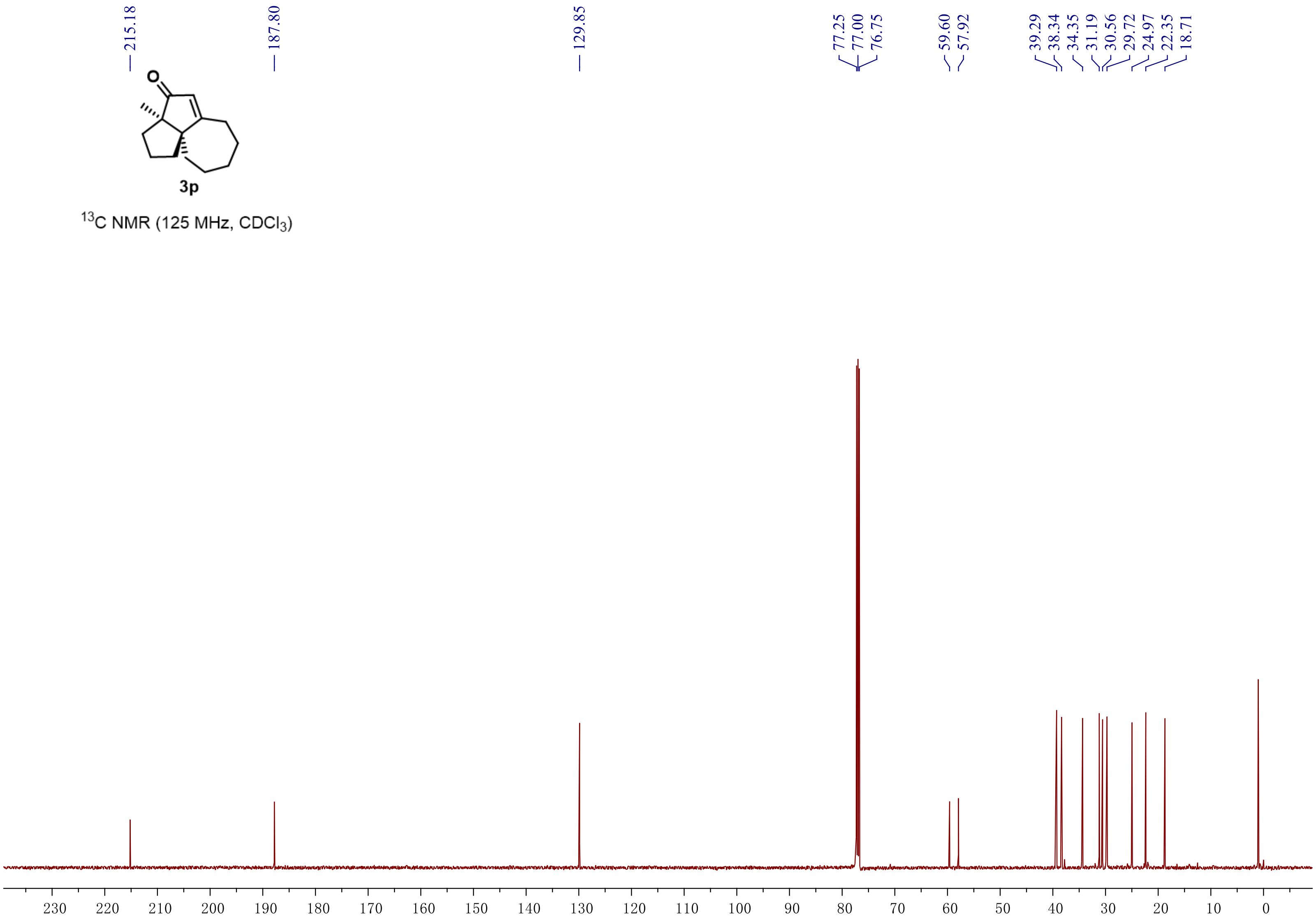

Supplementary Fig. 355. ^{13}C NMR spectra of compound **3p** in $CDCl_3$

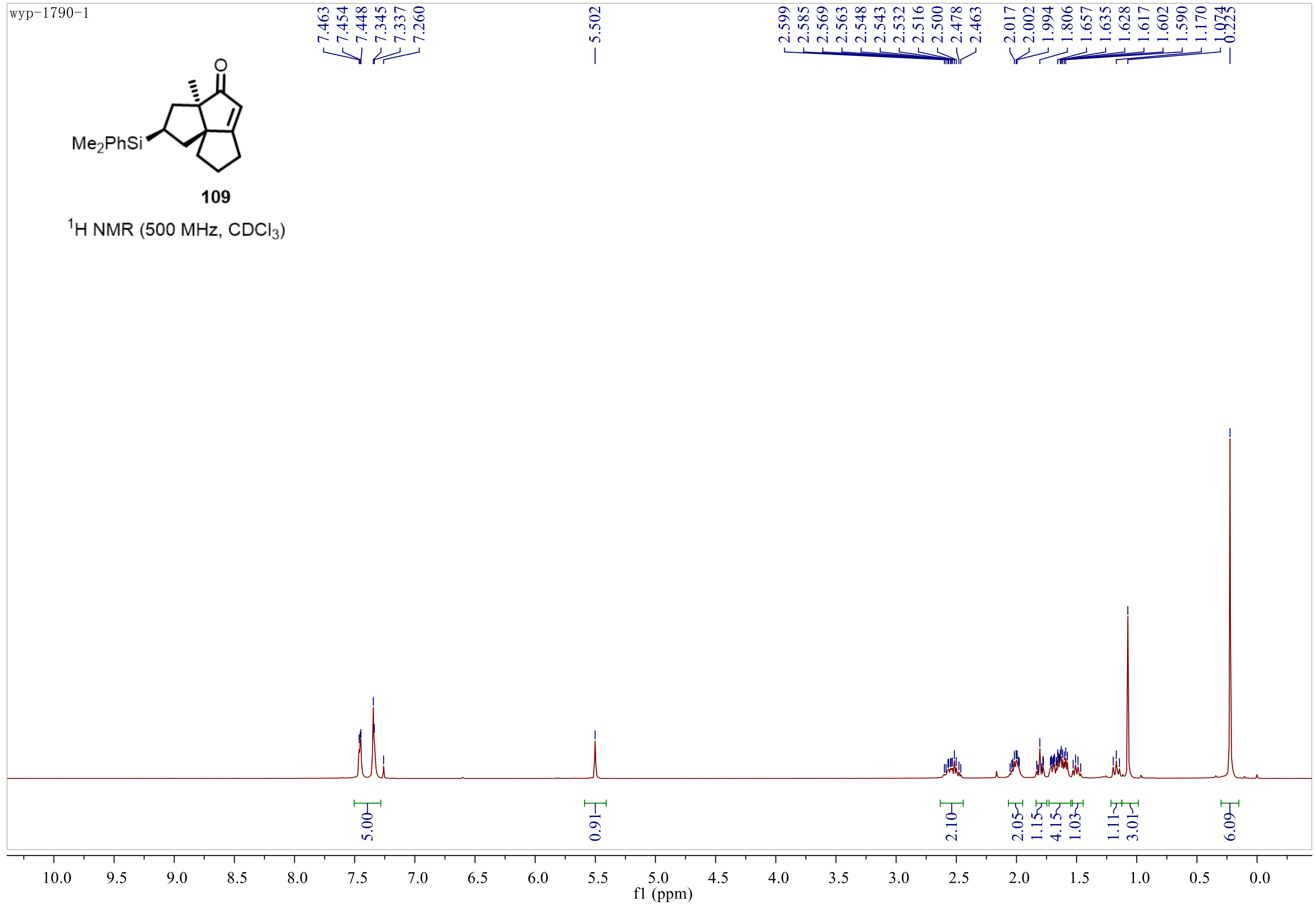

Supplementary Fig. 356. ^{1}H NMR spectra of compound **109** in $CDCl_3$

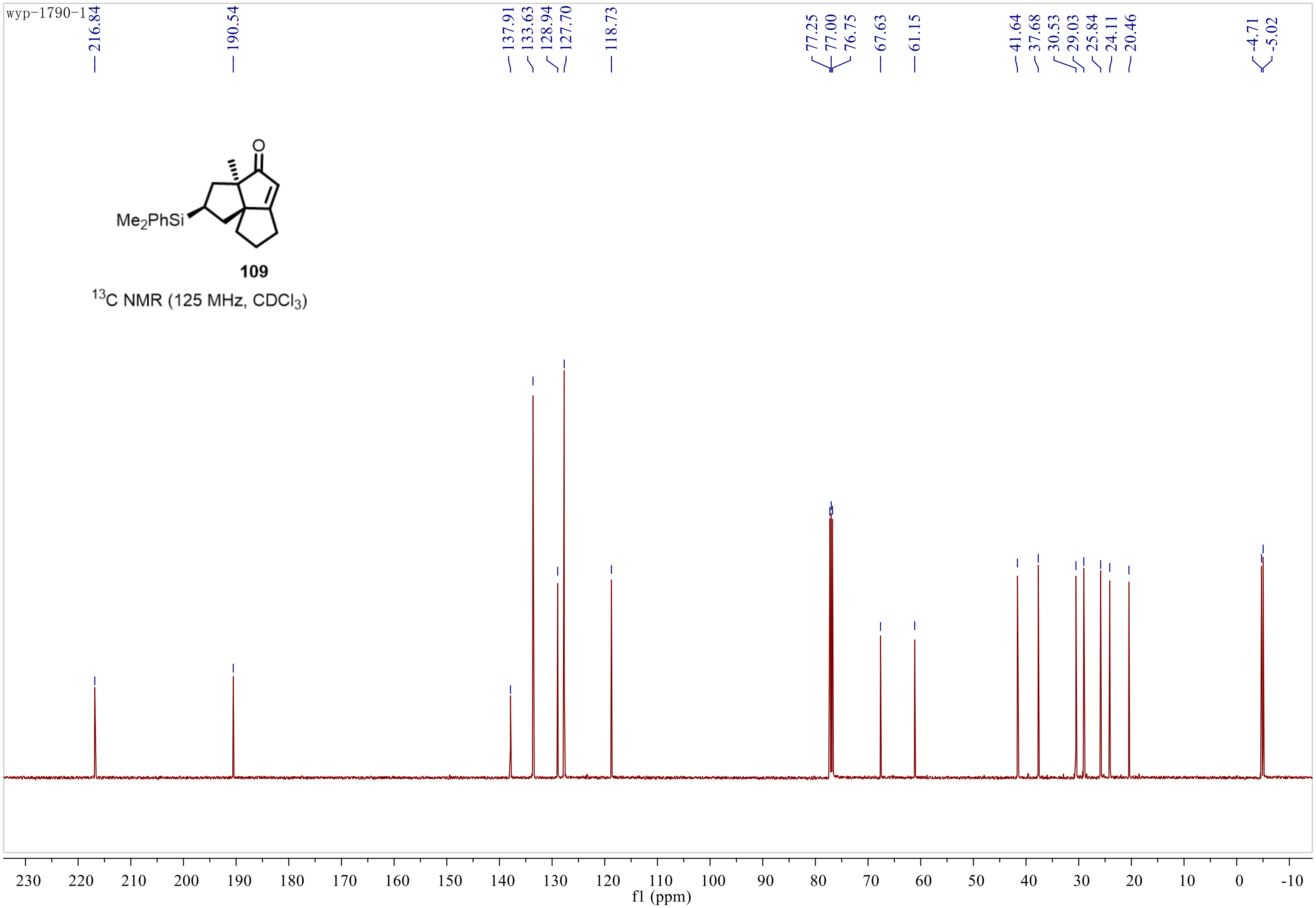

Supplementary Fig. 357. ^{13}C NMR spectra of compound **109** in $CDCl_3$

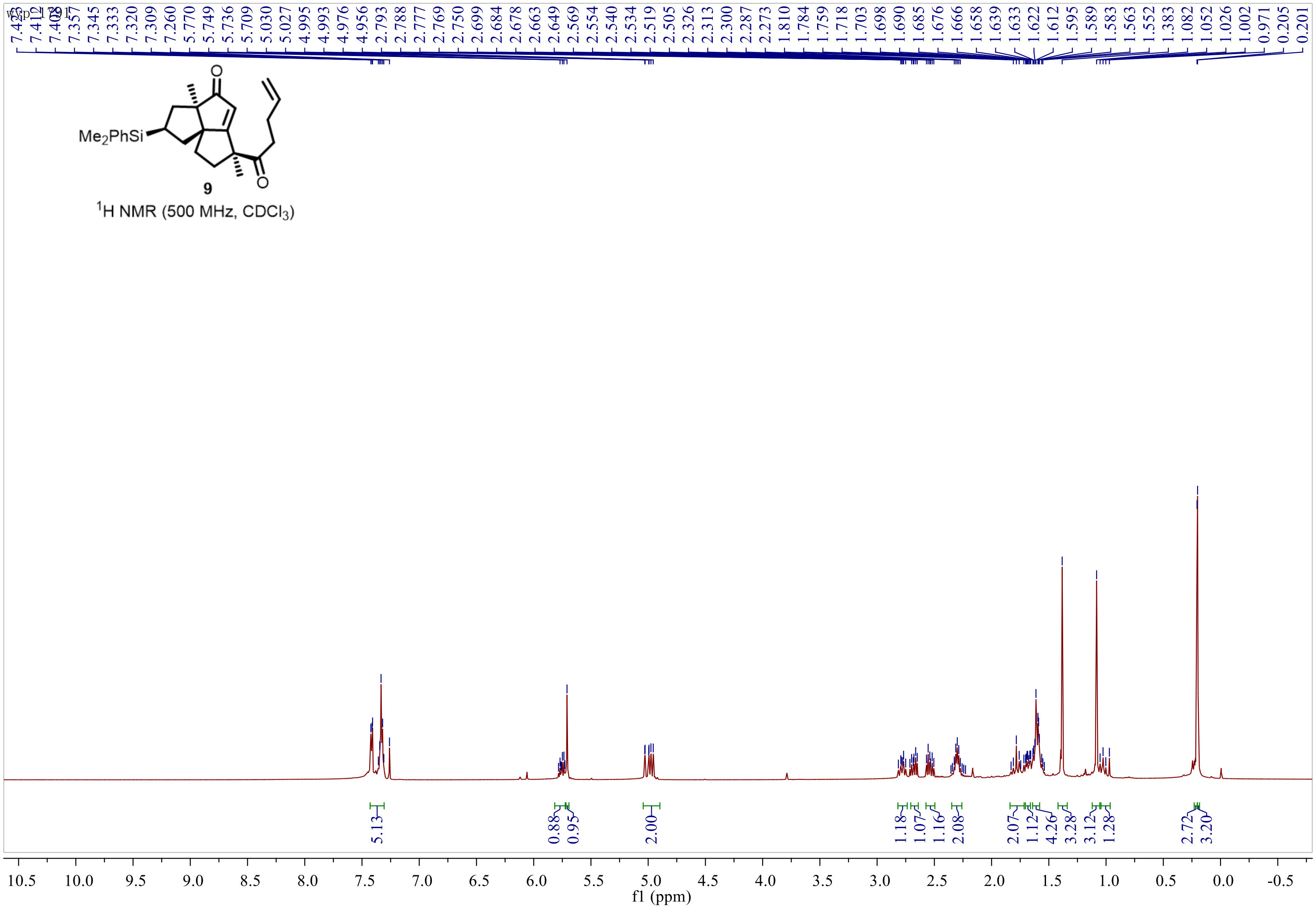

Supplementary Fig. 358. ^{1}H NMR spectra of compound **9** in $CDCl_3$

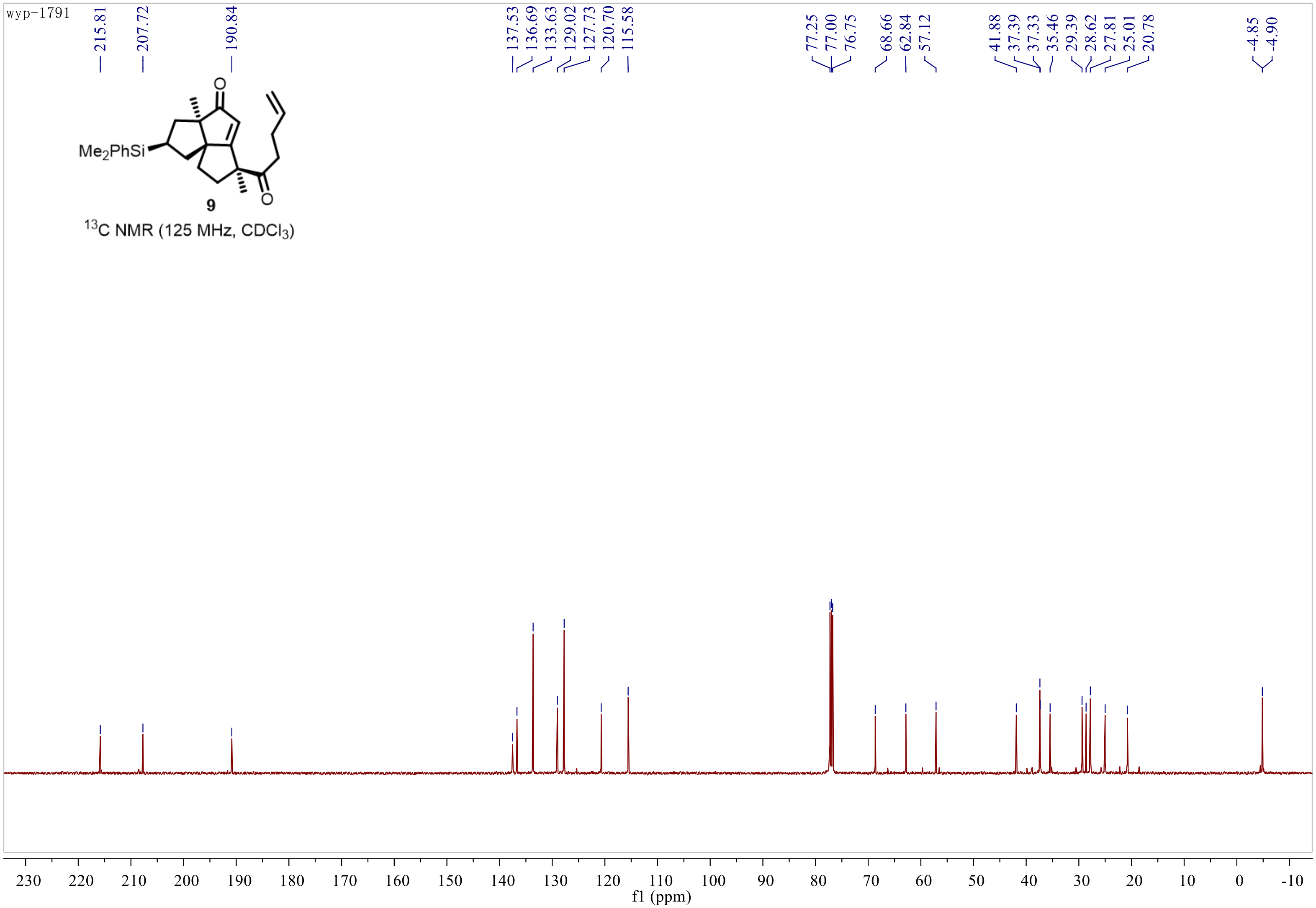

Supplementary Fig. 359. ^{13}C NMR spectra of compound **9** in $CDCl_3$

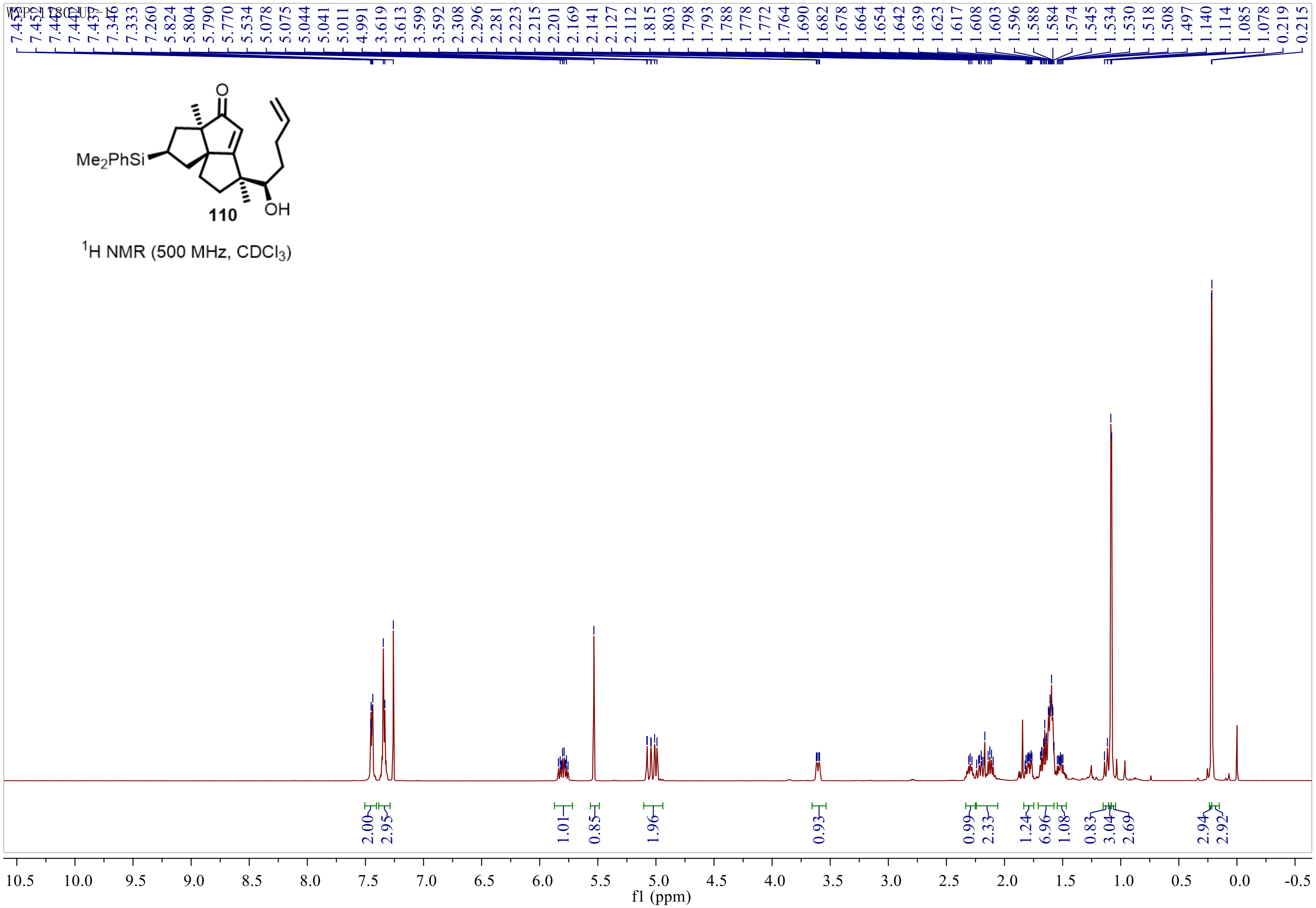

Supplementary Fig. 360. ^{1}H NMR spectra of compound **110** in $CDCl_3$

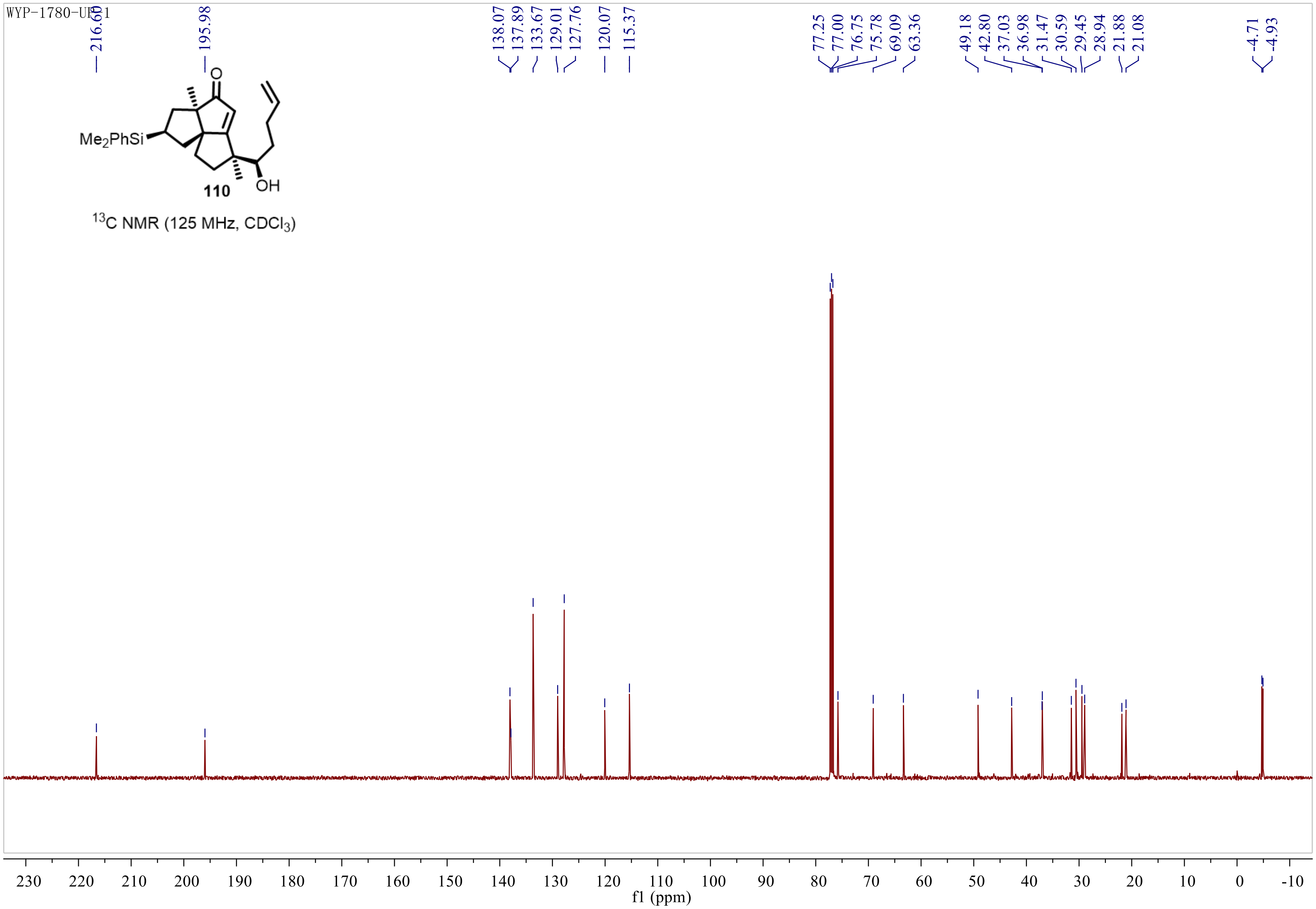

Supplementary Fig. 361. ^{13}C NMR spectra of compound **110** in $CDCl_3$

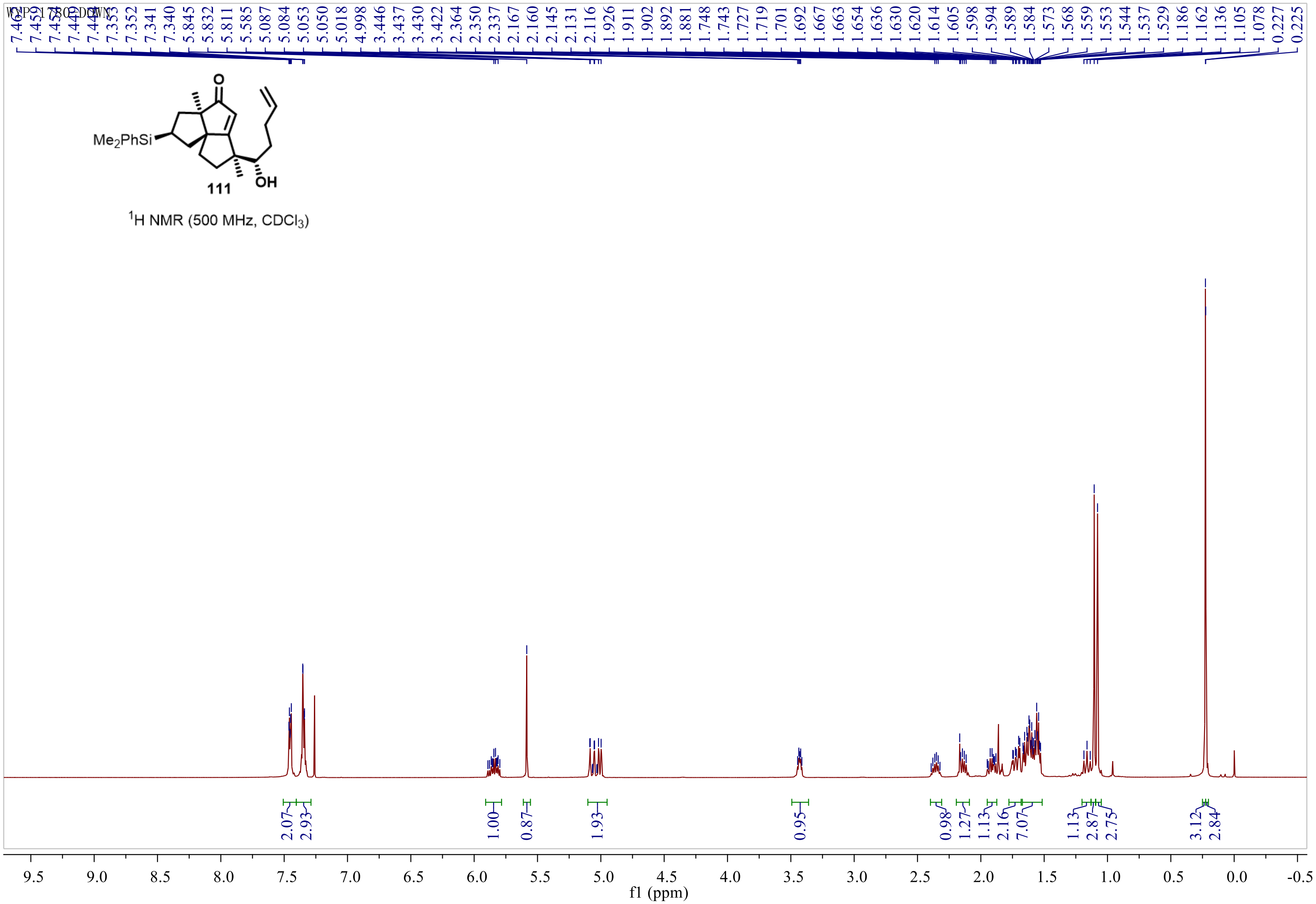

Supplementary Fig. 362. ^{1}H NMR spectra of compound **111** in $CDCl_3$

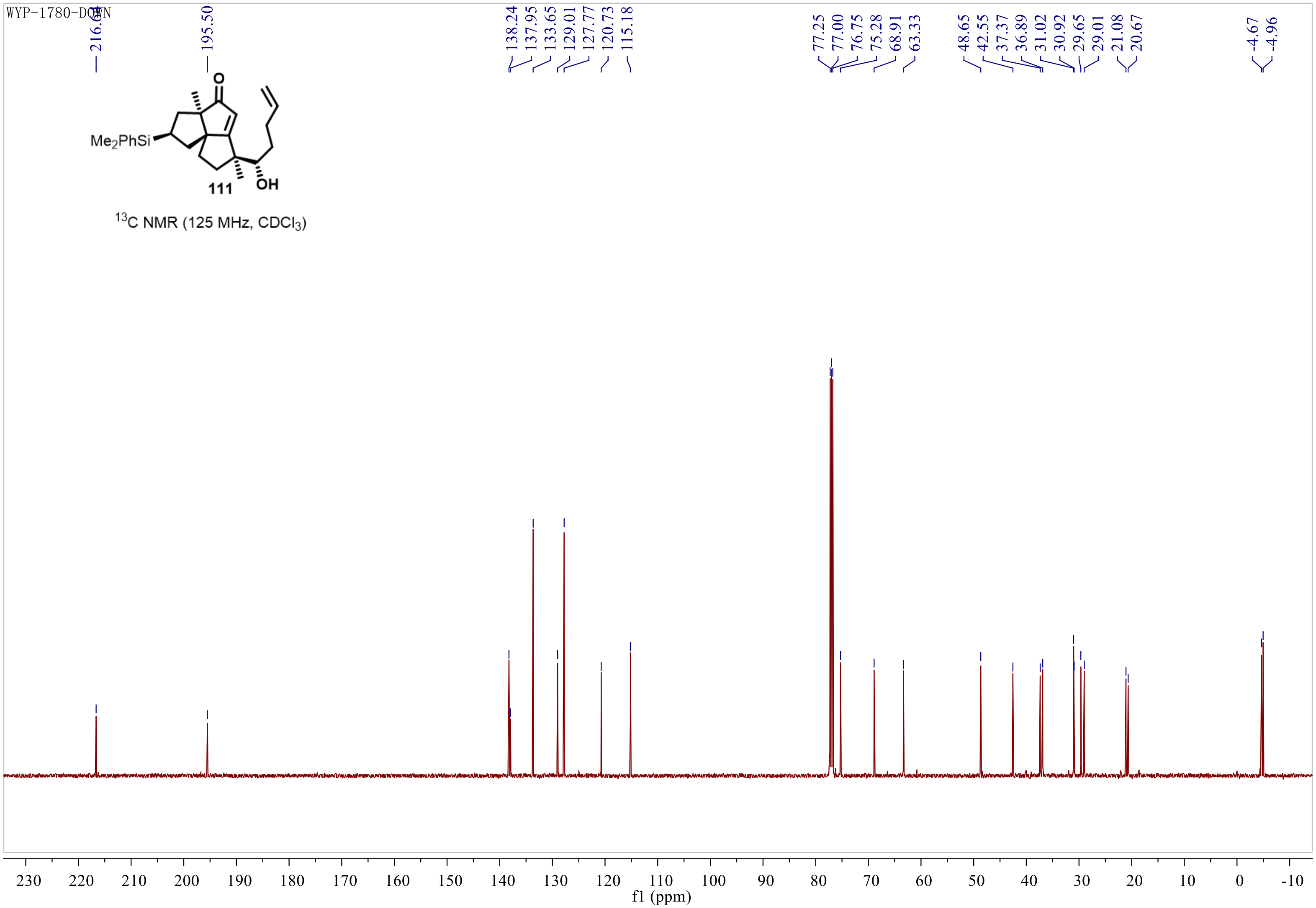

Supplementary Fig. 363. ^{13}C NMR spectra of compound **111** in $CDCl_3$

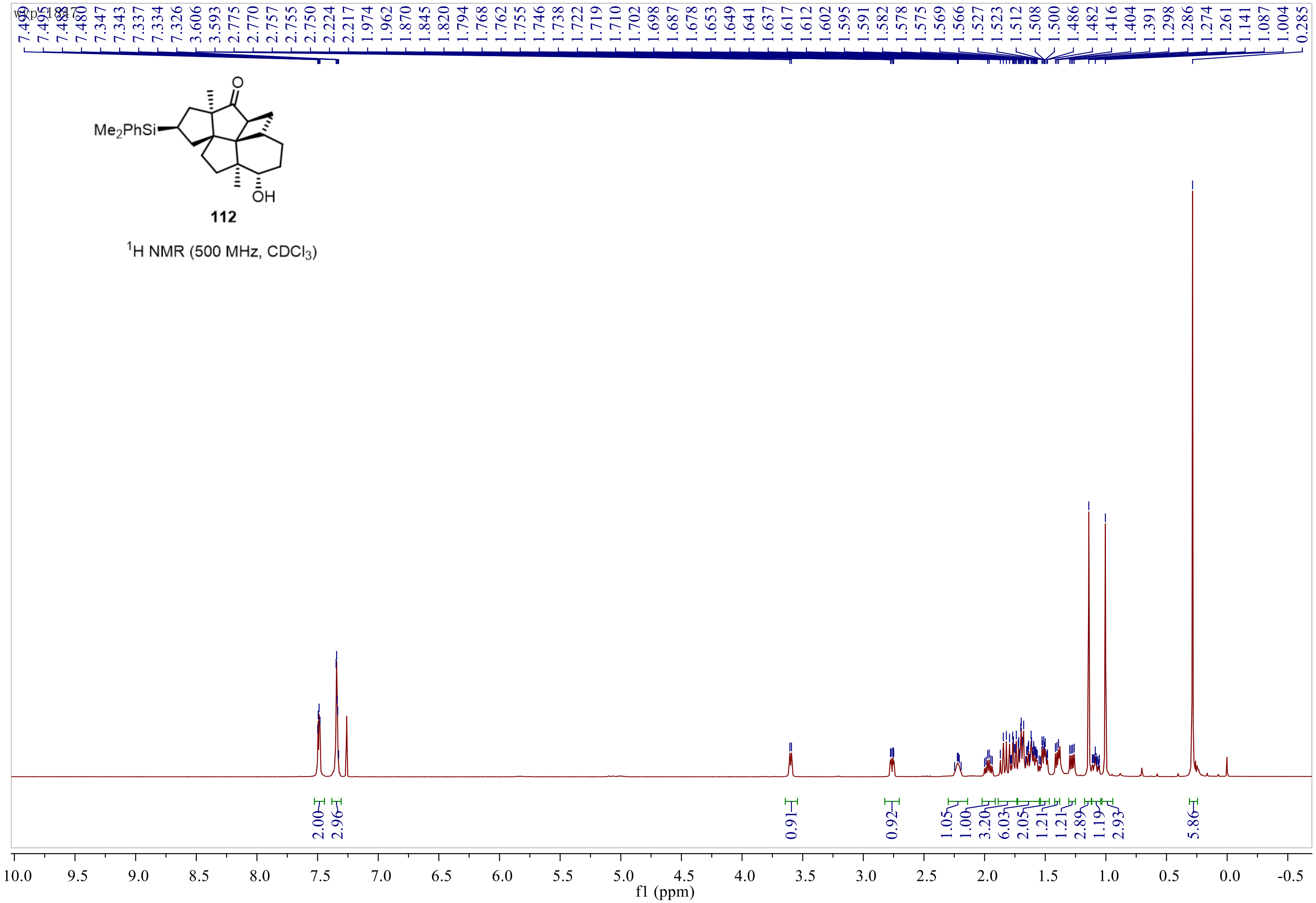

Supplementary Fig. 364. ^{1}H NMR spectra of compound **112** in $CDCl_3$

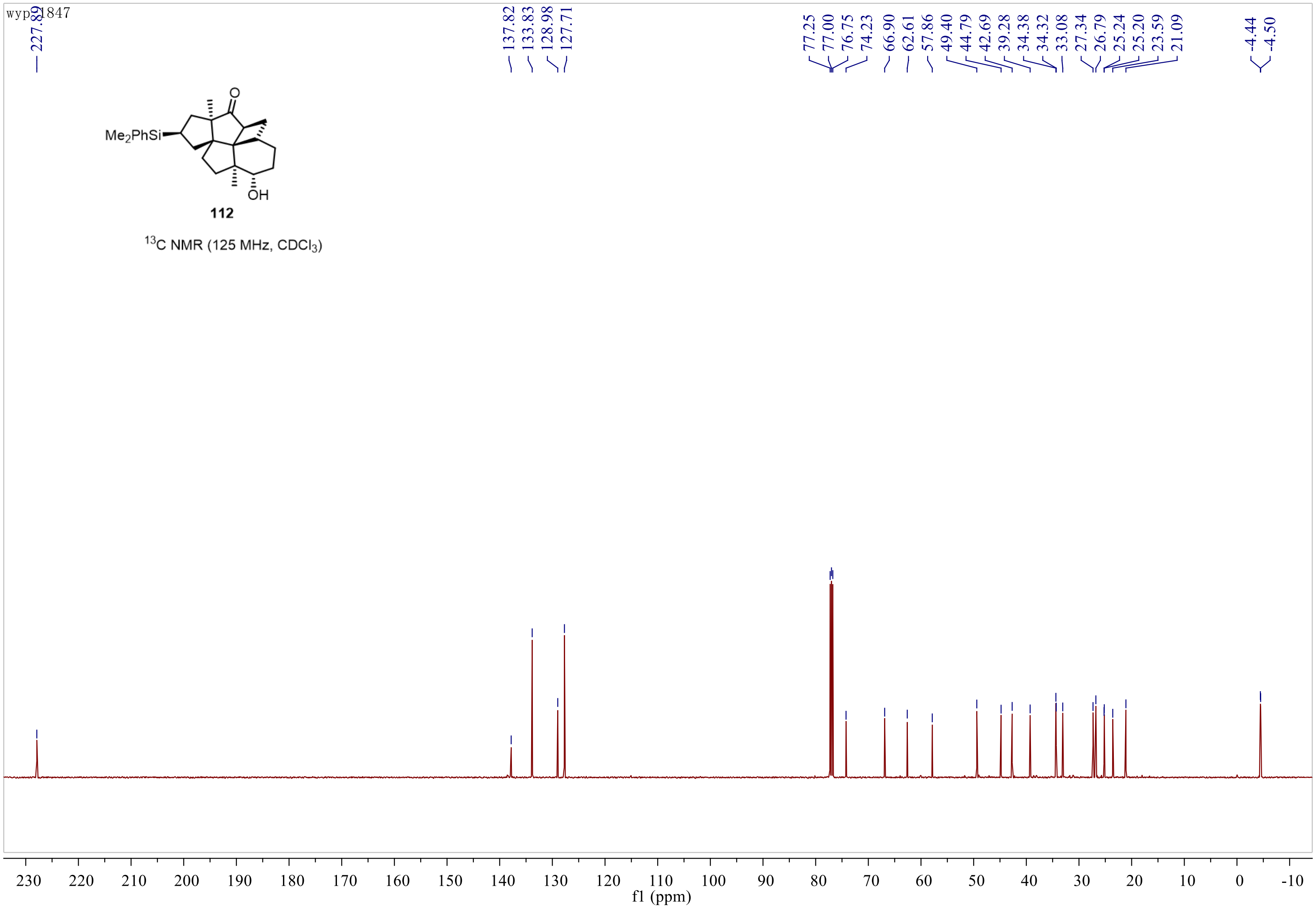

Supplementary Fig. 365. ^{13}C NMR spectra of compound **112** in $CDCl_3$

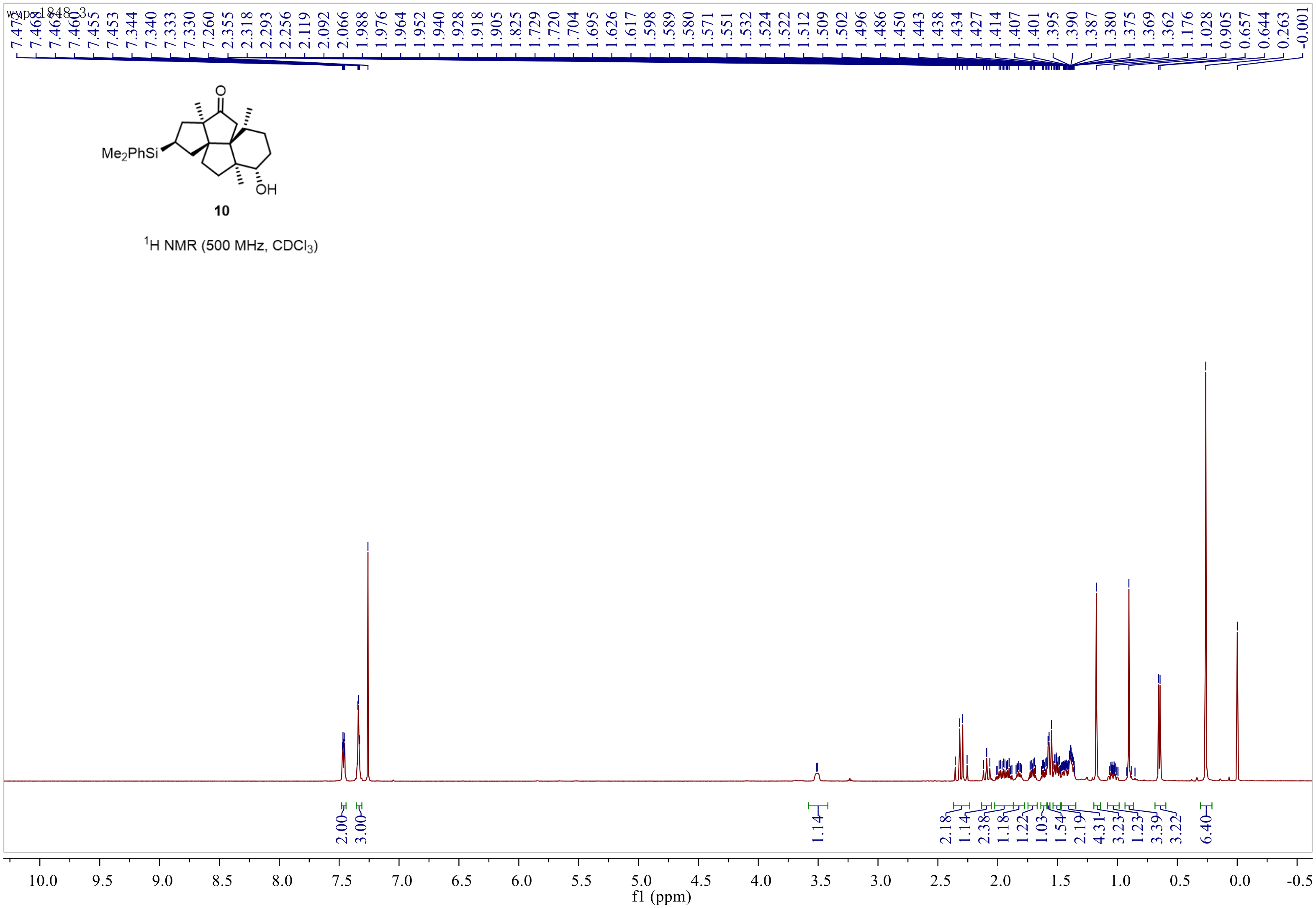

Supplementary Fig. 366. ^{1}H NMR spectra of compound **10** in $CDCl_3$

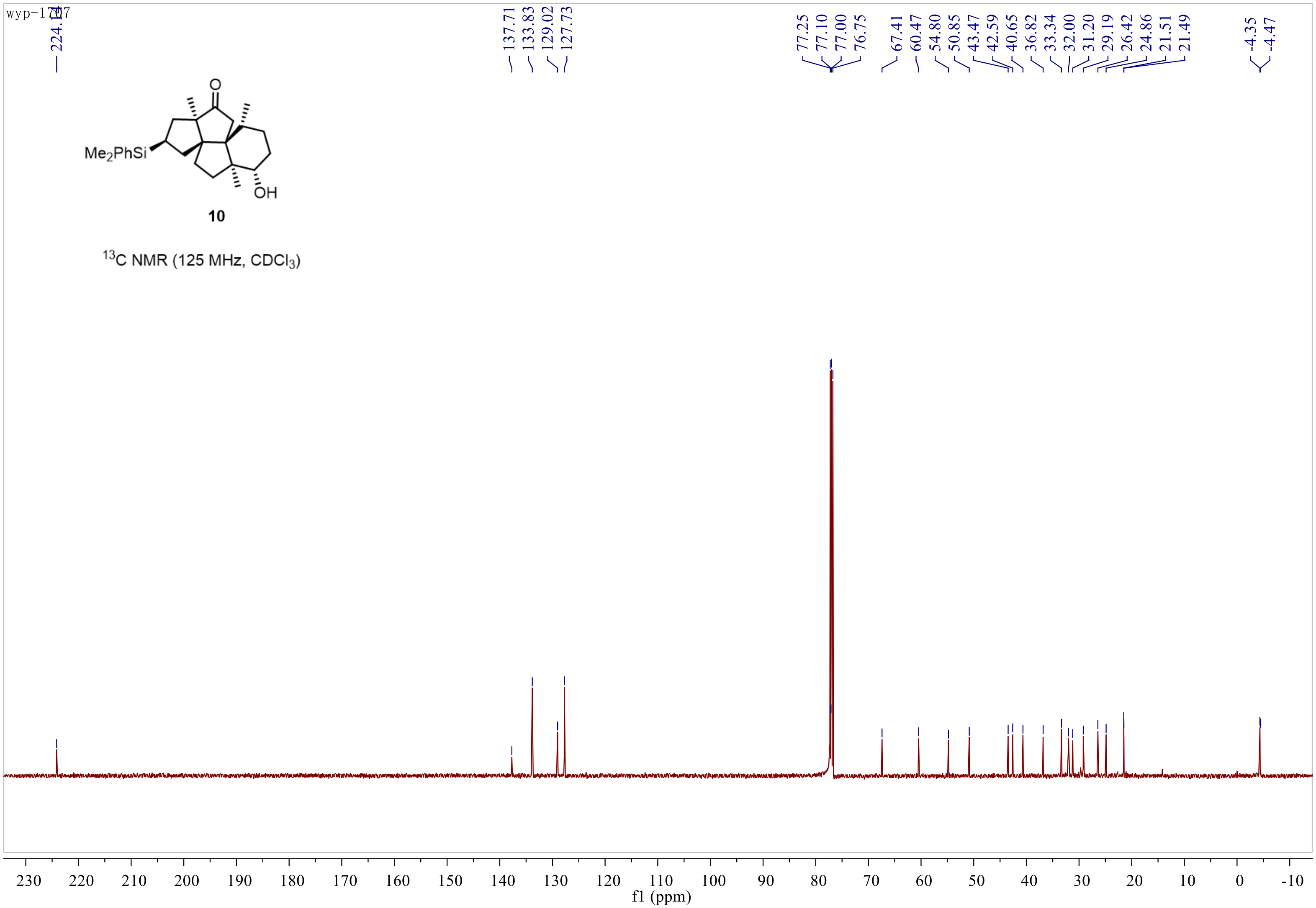

Supplementary Fig. 367. ^{13}C NMR spectra of compound **10** in $CDCl_3$

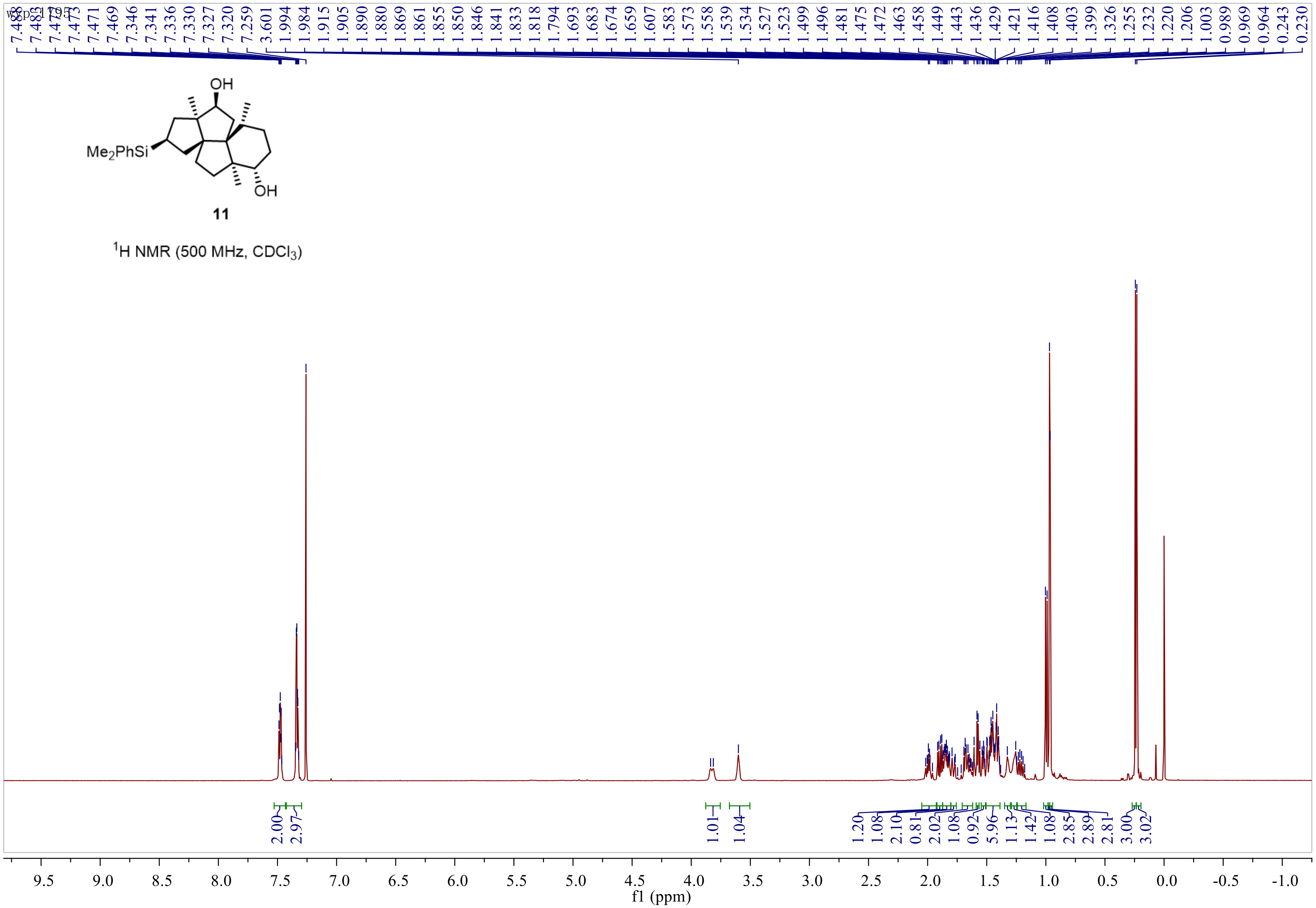

Supplementary Fig. 368. ^{1}H NMR spectra of compound **11** in $CDCl_3$

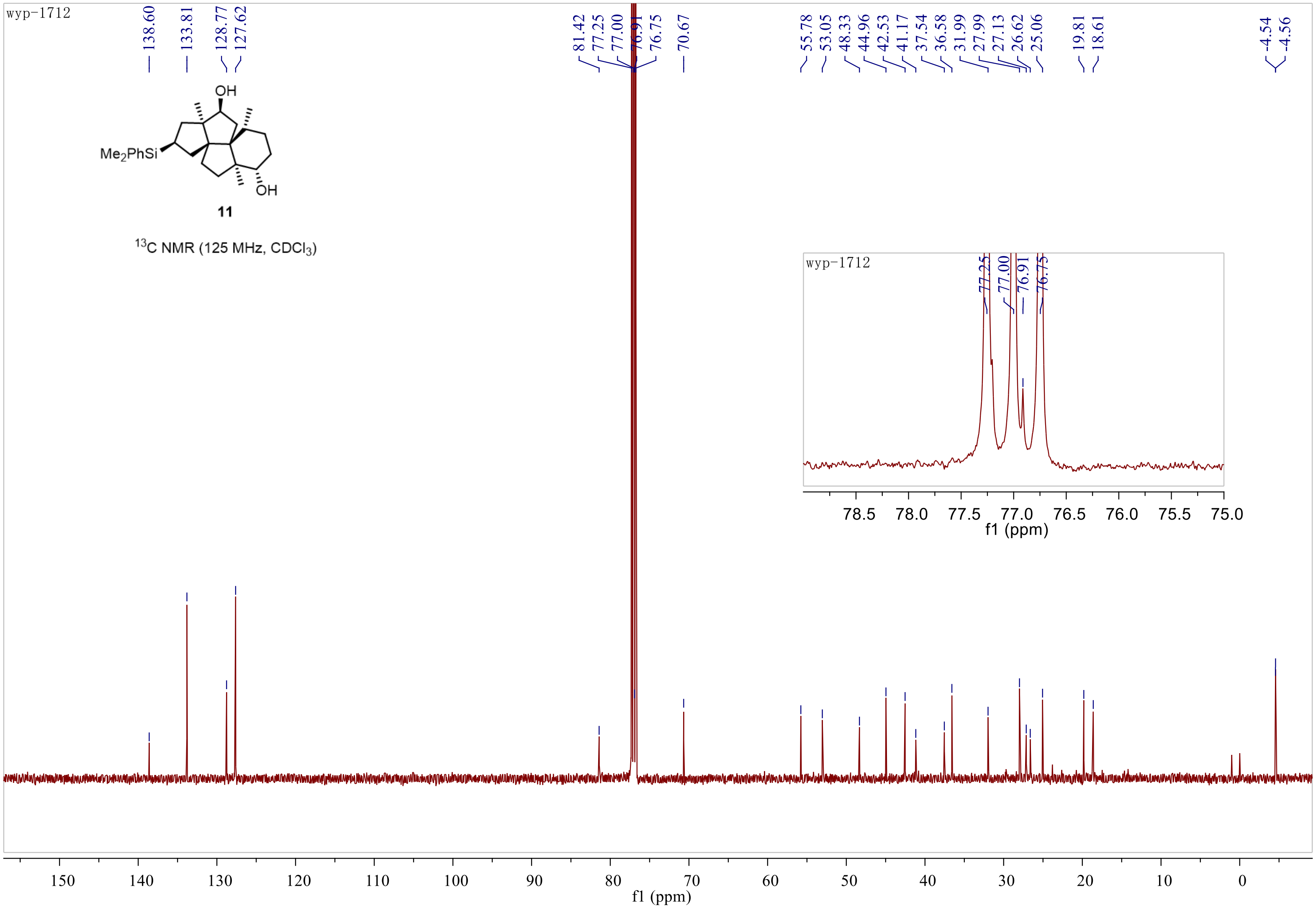

Supplementary Fig. 369. ^{13}C NMR spectra of compound **11** in $CDCl_3$

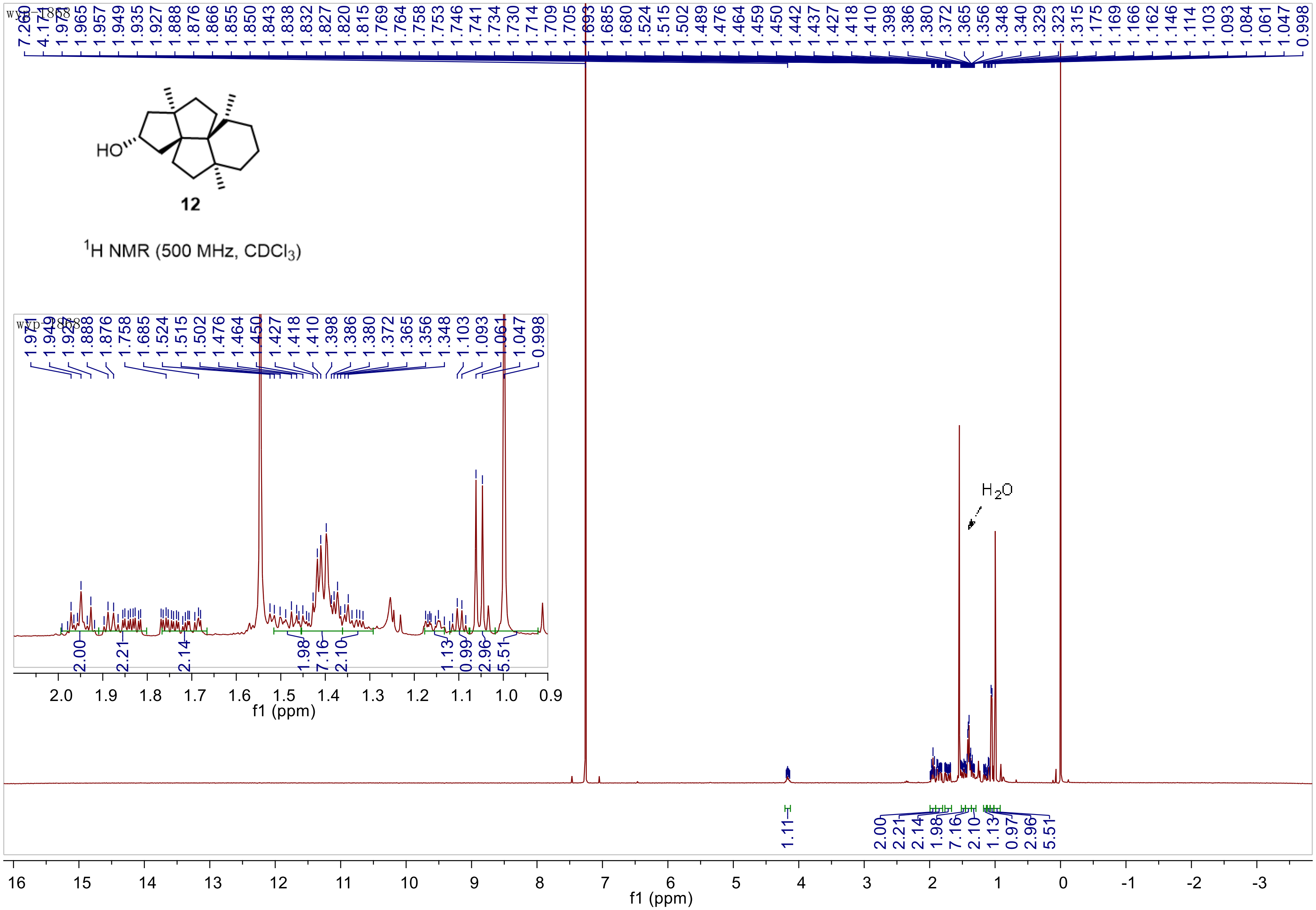

Supplementary Fig. 370. ^{1}H NMR spectra of compound **12** in $CDCl_3$

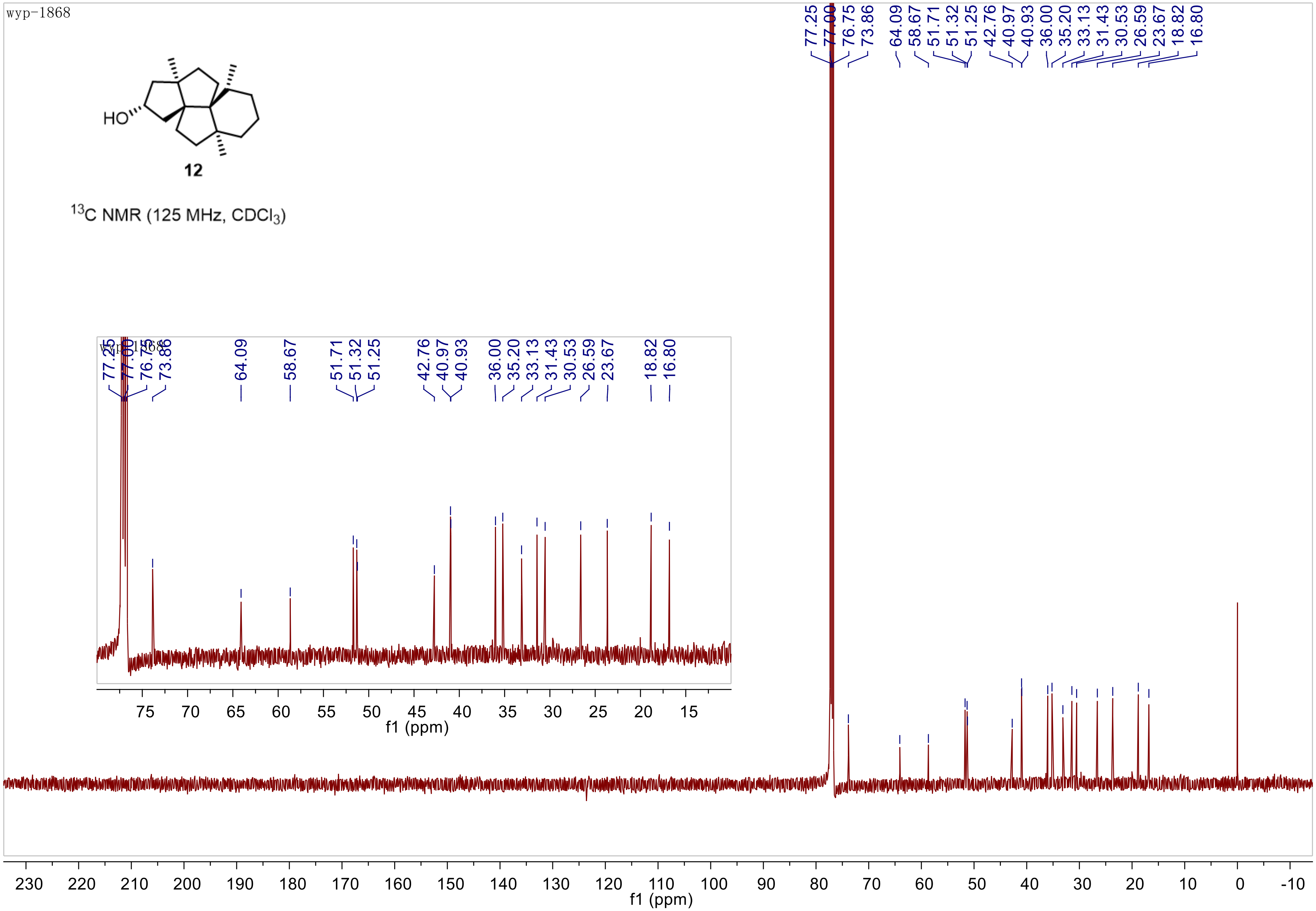

Supplementary Fig. 371. ^{13}C NMR spectra of compound **12** in $CDCl_3$

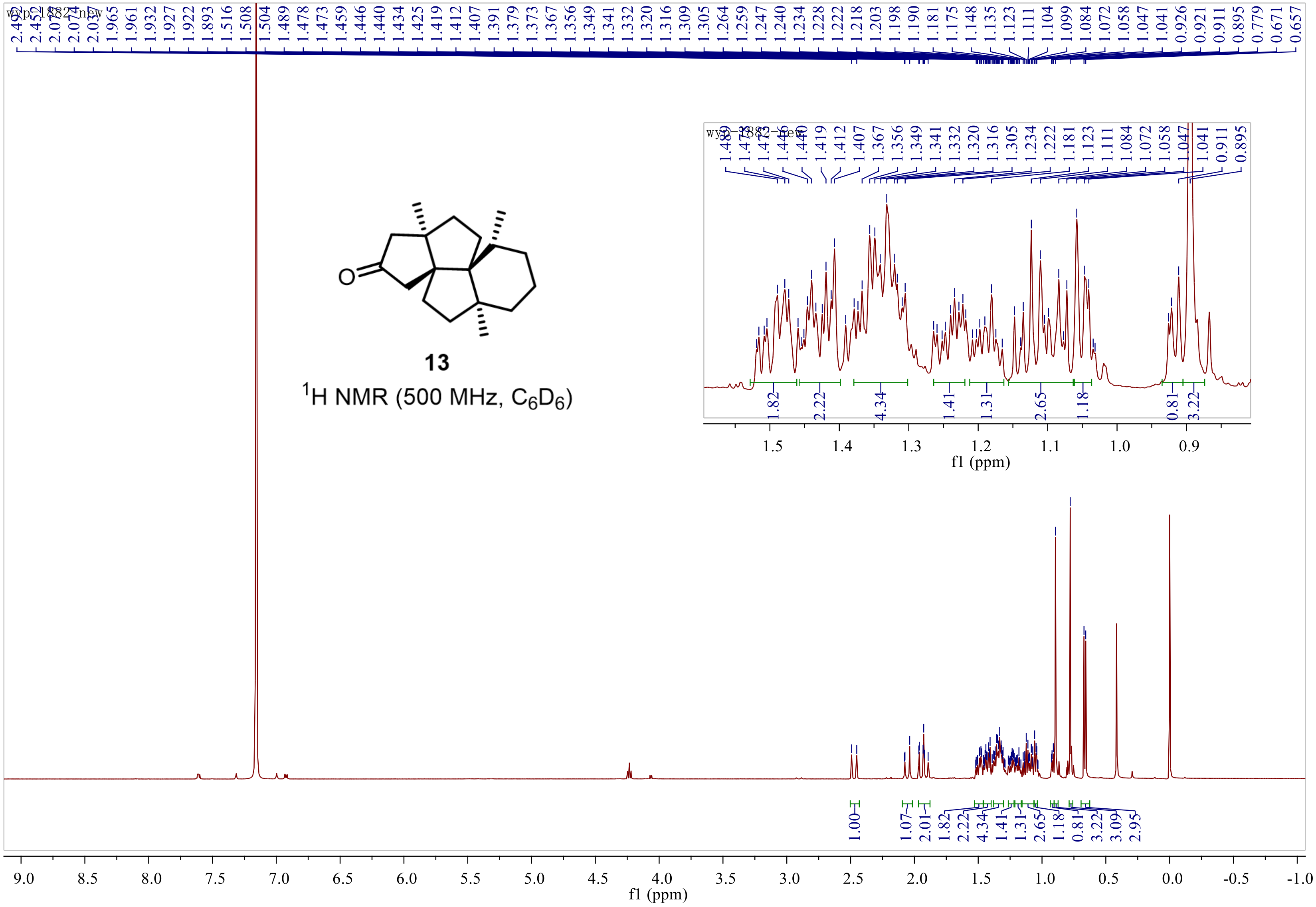

Supplementary Fig. 372. ^{1}H NMR spectra of compound **13** in C_6D_6

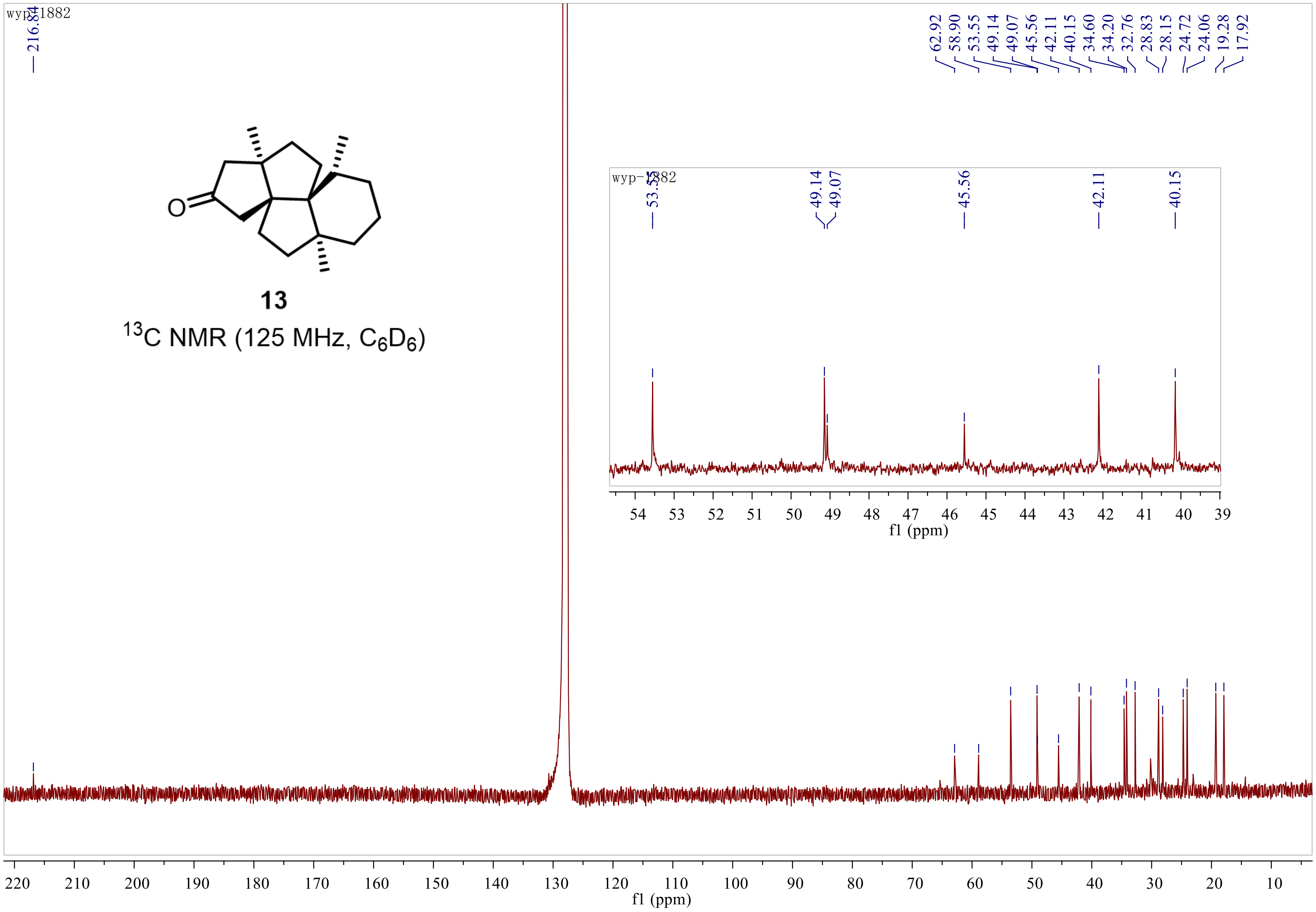

Supplementary Fig. 373. ^{13}C NMR spectra of compound **13** in C_6D_6

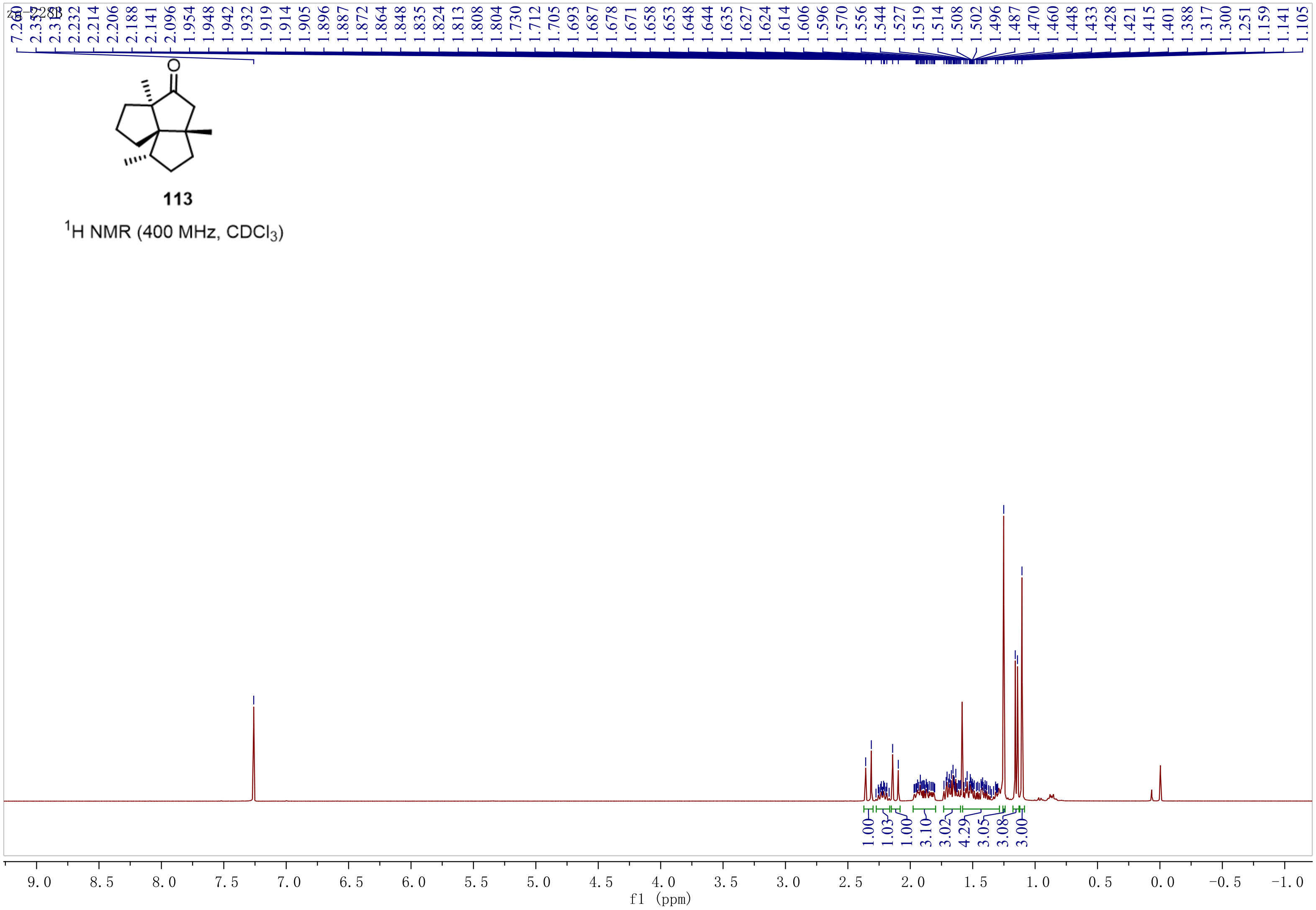

Supplementary Fig. 374. ^{1}H NMR spectra of compound **113** in $CDCl_3$

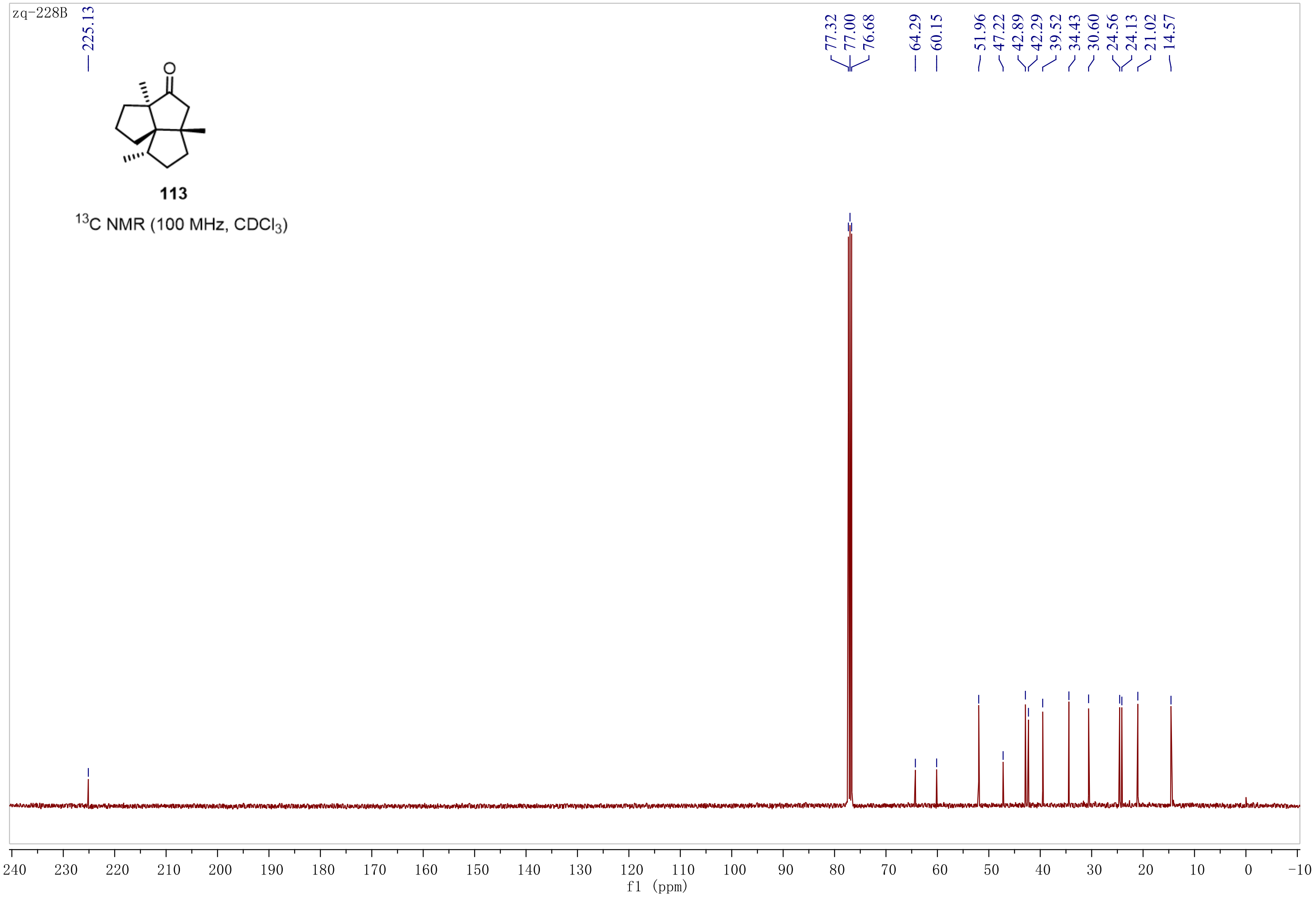

Supplementary Fig. 375. ^{13}C NMR spectra of compound **113** in $CDCl_3$

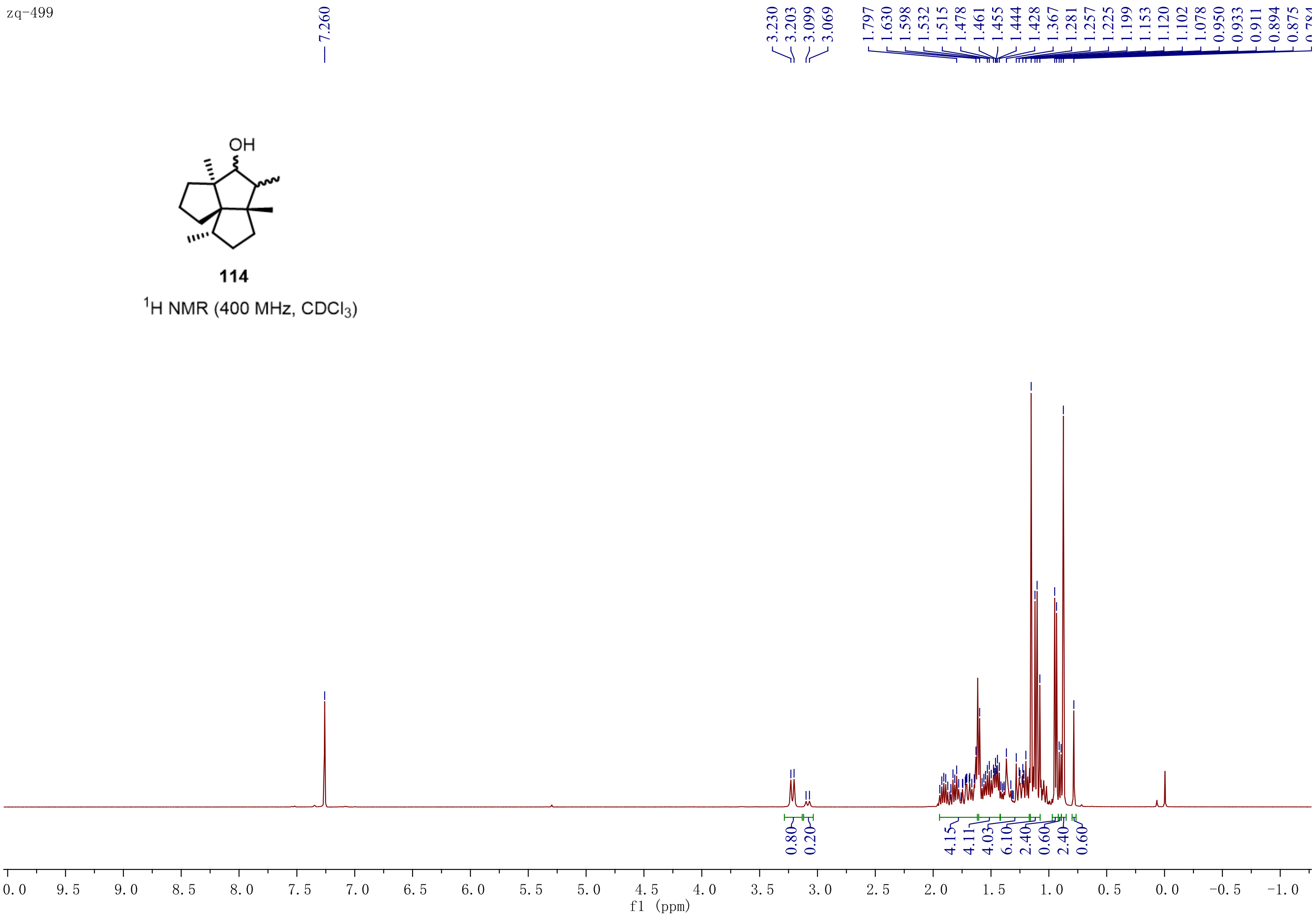

Supplementary Fig. 376. ^{1}H NMR spectra of compound **114** in $CDCl_3$

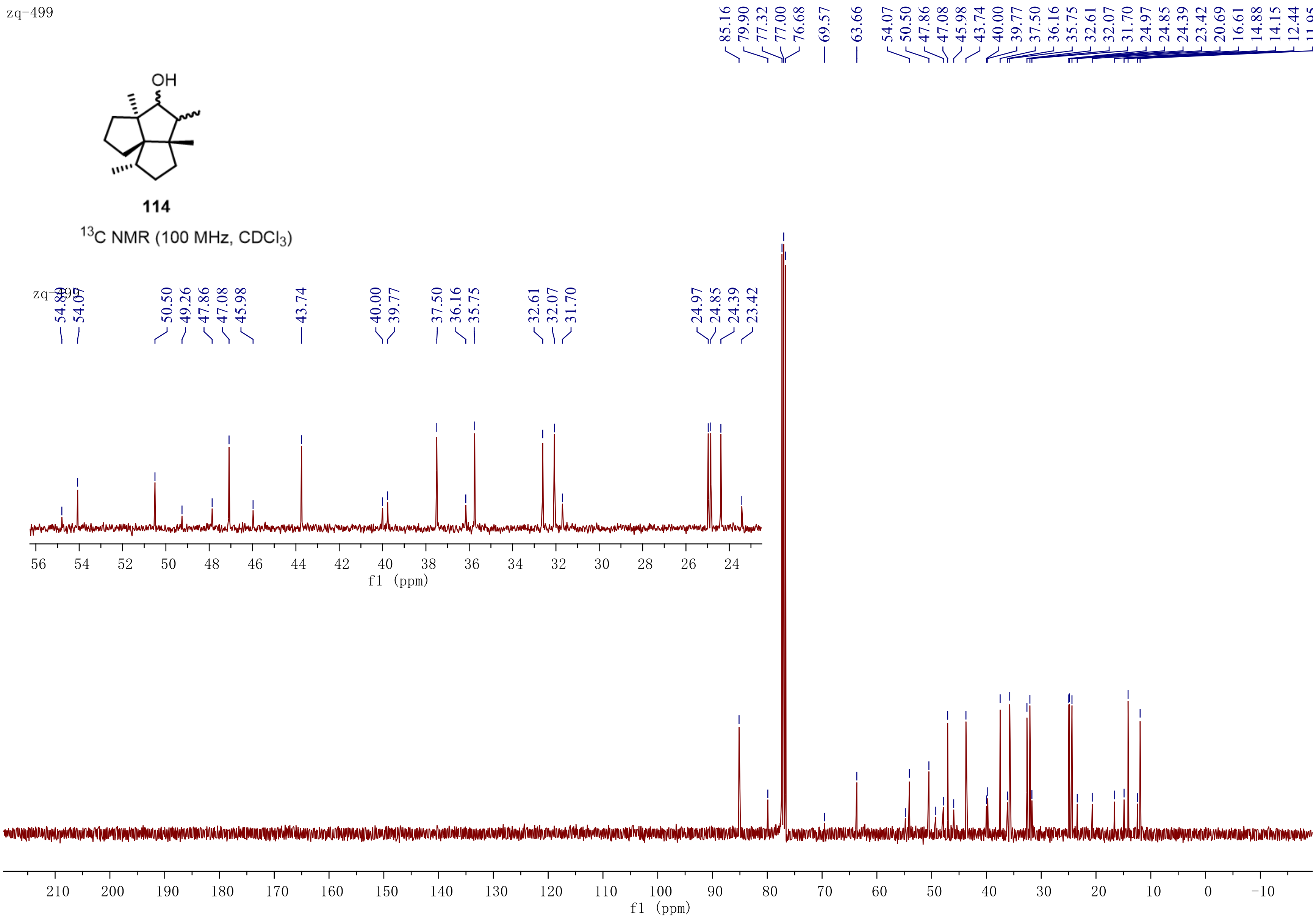

Supplementary Fig. 377. ^{13}C NMR spectra of compound **114** in $CDCl_3$

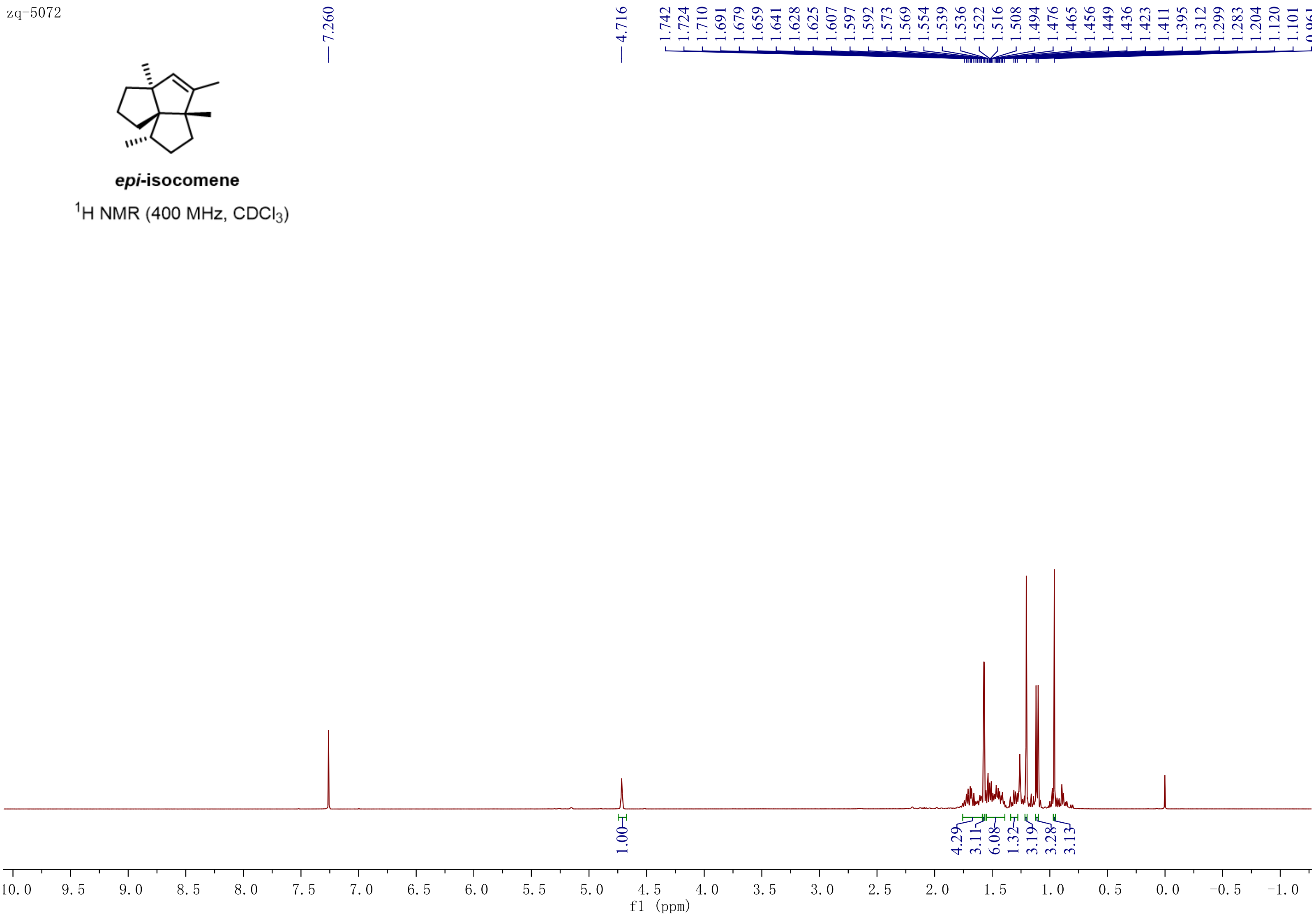

Supplementary Fig. 378. ^{1}H NMR spectra of compound ***epi*-isocomene** in $CDCl_3$

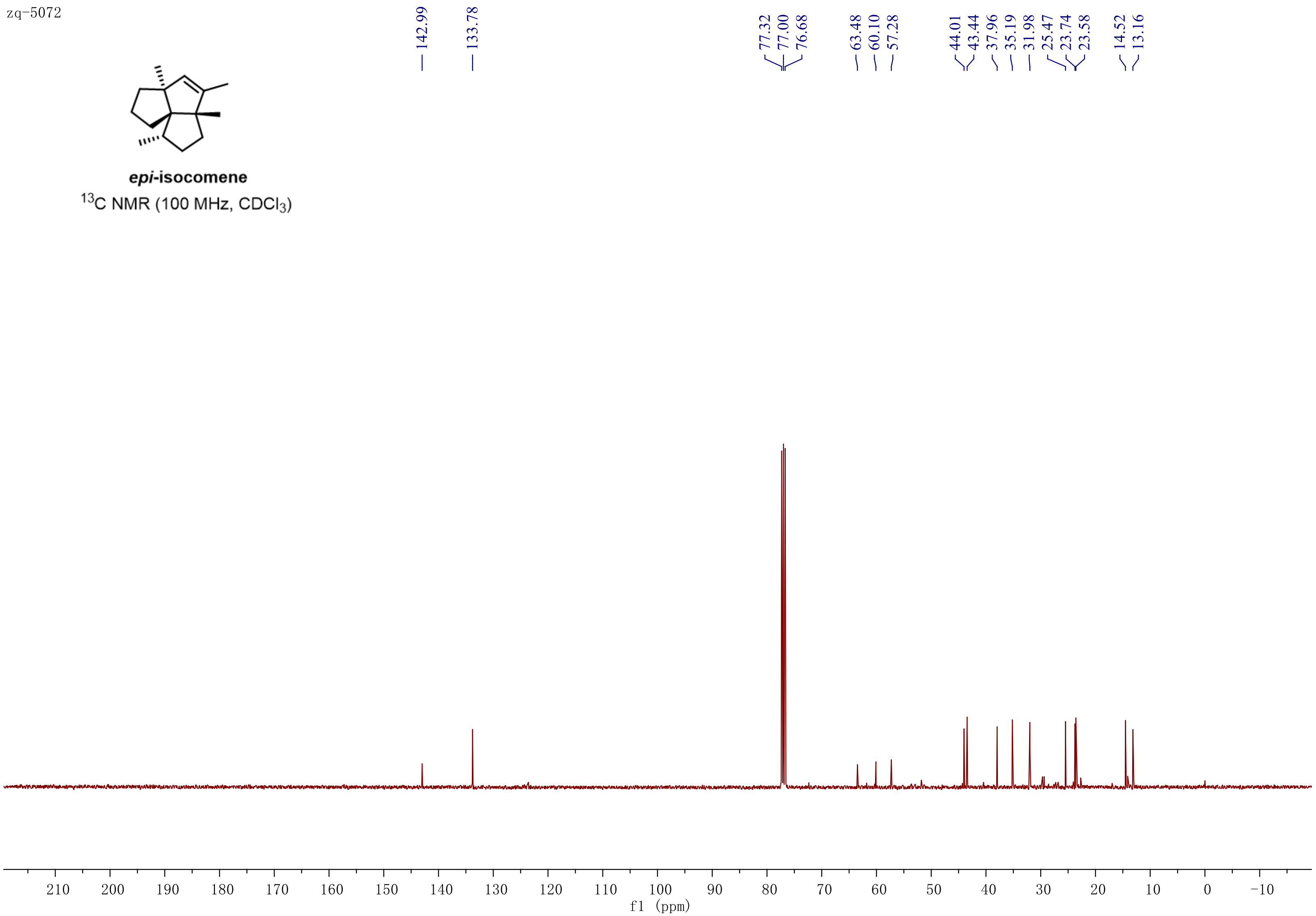

Supplementary Fig. 379. ^{13}C NMR spectra of compound ***epi*-isocomene** in $CDCl_3$

11.Supplementary References

[1] Chen, X. B.; Li, L.; Yang, W. C.; Song, K. L.; Wu, B.; Gan, W. E.; Cao, J.; Xu, L. W. Lewis Acid-Catalyzed Yne-Carbonyl Metathesis of Ynamides and Cyclobutanones: Facile Synthesis of Functionalized Alkylidenecyclobutanes. *Asian J. Org. Chem.* **7**, 374-377 (2018).

[2] Lou, J.; Wang, Q.; Zhou, Y.-G.; Yu, Z. Rhodium(III)-Catalyzed Annulative Coupling of Sulfoxonium Ylides and Allenoates: An Arene C–H Activation/Cyclopropanation Cascade. *Org. Lett.* **21**, 9217-9222 (2019).

[3] Trost, B. M.; Xie, J. Palladium-Catalyzed Diastereo- and Enantioselective Wagner−Meerwein Shift: Control of Absolute Stereochemistry in the C−C Bond Migration Event. *J. Am. Chem. Soc.* **130**, 6231-6242 (2008).

[4] Murakami, M.; Usui, I.; Hasegawa, M.; Matsuda, T. Contrasteric Stereochemical Dictation of the Cyclobutene Ring-Opening Reaction by a Vacant Boron p Orbital. *J. Am. Chem. Soc.* **127**, 1366-1367 (2005).

[5] Chang, S.; Holmes, M.; Mowat, J.; Meanwell, M.; Britton, R. α-Arylation and Ring Expansion of Annulated Cyclobutanones: Stereoselective Synthesis of Functionalized Tetralones. *Angew. Chem. Int. Ed.* **56**, 748-752 (2017).

[6] Volk, F.-J., Wagner, M. & Frahm, A. W. Cyclobutane amino acids (CBAAs): asymmetric Strecker synthesis of enantiopure cis- and trans-2,4-methanovalines. *Tetrahedron: Asymmetry* **14**, 497-502 (2003).

[7] Trost, B. M.; Bogdanowicz, M. J. New synthetic reactions. Cyclopentane annelation. *J. Am. Chem. Soc.* **95**, 289-290 (1973).

[8] Lee, H.; Kang, T.; Lee, H.-Y. Total Synthesis of (±)-Waihoensene. *Angew. Chem. Int. Ed.* **56**, 8254-8257 (2017).

[9] Qu, Y.; Wang, Z.; Zhang, Z.; Zhang, W.; Huang, J.; Yang, Z Asymmetric Total Synthesis of (+)-Waihoensene. *J. Am. Chem. Soc.* **142**, 6511-6515 (2020).

[10] Peng, C.; Arya, P.; Zhou, Z.; Snyder, S. A. A Concise Total Synthesis of (+)-Waihoensene Guided by Quaternary Center Analysis. *Angew. Chem. Int. Ed.* **59**, 13521-13525 (2020).

[11] Ranu, B. C.; Kavka, M.; Higgs, L. A.; Hudlickyl, T. Stereocontrolled total synthesis of isocomene sesquiterpenes. *Tetrahedron Letters* **25**, 2447-2450 (1984).

[12] Hudlicky, T.; Kwart, L. D.; Tiedje, M. H.; Ranu, B. C.; Short, R. P.; Frazier, J. O.; Rigby,

H. L. General Methodology for the Topologically Selective Preparation of Linear and Nonlinear Tricyclopentanoids of Hirsutane and Isocomene Type via a Claisen Rearrangement / Cyclopentene Annulation Sequence. Total Synthesis of (±)- Epiisocomene. *Synthesis* **9**, 716-727 (1986).
[13] Zhao, Y.; Truhlar, D. G. Density Functionals with Broad Applicability in Chemistry. *Acc. Chem. Res.* **41**, 157-167 (2008).
[14] Zhao, Y.; Truhlar, D. G. The M06 suite of density functionals for main group thermochemistry, thermochemical kinetics, noncovalent interactions, excited states, and transition elements: two new functionals and systematic testing of four M06-class functionals and 12 other functionals. *Theor. Chem. Acc.* **120**, 215-241 (2008).
[15] Hay, P. J.; Wadt, W. R. Ab initio effective core potentials for molecular calculations. Potentials for K to Au including the outermost core orbitals. *J. Chem. Phys.* **82**, 299-310 (1985).
[16] Roy, L. E.; Hay, P. J.; Martin, R. L. Revised Basis Sets for the LANL Effective Core Potentials. *J. Chem. Theory. C.* **4**, 1029-1031 (2008).
[17] Hariharan, P. C.; Pople, J. A. The influence of polarization functions on molecular orbital hydrogenation energies. *Theor. chim. acta* **28**, 213 (1973).
[18] Fukui, K. The path of chemical reactions - the IRC approach. *Acc. Chem. Res.* **14**, 363-368 (1981).
[19] Marenich, A. V.; Cramer, C. J.; Truhlar, D. G. Universal Solvation Model Based on Solute Electron Density and on a Continuum Model of the Solvent Defined by the Bulk Dielectric Constant and Atomic Surface Tensions. *J. Phys. Chem. B* **113**, 6378-6396 (2009).
[20] Andrae, D.; Häußermann, U.; Dolg, M.; Stoll, H.; Preuß, H. Energy-adjustedab initio pseudopotentials for the second and third row transition elements. *Theor; chim; acta* **77**, 123 (1990).
[21] Dolg, M.; Wedig, U.; Stoll, H.; Preuss, H. Energy‐adjusted ab initio pseudopotentials for the firstrow transition elements. *J. Chem. Phys.* **86**, 866-872 (1987).
[22] Krishnan, R.; Binkley, J. S.; Seeger, R.; Pople, J. A. Self‐consistent molecular orbital methods. XX. A basis set for correlated wave functions. *J. Chem. Phys.* **72**, 650-654 (1980).
[23] McLean, A. D.; Chandler, G. S. Contracted Gaussian basis sets for molecular calculations. I. Second row atoms, Z=11–18. *J. Chem. Phys.* **72**, 5639-5648 (1980).
[24] Frisch, M. J.; Trucks, G. W.; Schlegel, H. B.; Scuseria, G. E.; Robb, M. A.; Cheeseman, J. R.; Scalmani, G.; Barone, V.; Mennucci, B.; Petersson, G. A.; Nakatsuji, H.; Caricato, M.; Li, X.; Hratchian, H. P.; Izmaylov, A. F.; Bloino, J.; Zheng, G.; Sonnenberg, J. L.; Hada, M.; Ehara, M.; Toyota, K.; Fukuda, R.; Hasegawa, J.; Ishida, M.; Nakajima, T.; Honda, Y.; Kitao, O.; Nakai, H.; Vreven, T.; Montgomery, J. A.; Peralta, J. E.; Ogliaro, F.; Bearpark, M.; Heyd, J. J.; Brothers, E.; Kudin, K. N.; Staroverov, V. N.; Keith, T.; Kobayashi, R.; Normand, J.; Raghavachari, K.; Rendell, A.; Burant, J. C.; Iyengar, S. S.; Tomasi, J.; Cossi, M.; Rega, N.; Millam, N. J.; Klene, M.; Knox, J. E.; Cross, J. B.; Bakken, V.; Adamo, C.; Jaramillo, J.; Gomperts, R.; Stratmann, R. E.; Yazyev,

O.; Austin, A. J.; Cammi, R.; Pomelli, C.; Ochterski, J. W.; Martin, R. L.; Morokuma, K.; Zakrzewski, V. G.; Voth, G. A.; Salvador, P.; Dannenberg, J. J.; Dapprich, S.; Daniels, A. D.; Farkas, O.; Foresman, J. B.; Ortiz, J. V.; Cioslowski, J.; Fox, D. J.; Gaussian 09, Revision D.01, Gaussian, Inc., Wallingford CT, (2013).
